# Population FBA predicts metabolic phenotypes in yeast

**DOI:** 10.1371/journal.pcbi.1005728

**Published:** 2017-09-08

**Authors:** Piyush Labhsetwar, Marcelo C. R. Melo, John A. Cole, Zaida Luthey-Schulten

**Affiliations:** 1 Center for Biophysics and Quantitative Biology, University of Illinois at Urbana-Champaign, Urbana, Illinois, United States of America; 2 Department of Physics, University of Illinois at Urbana-Champaign, Urbana, Illinois, United States of America; 3 Department of Chemistry, University of Illinois at Urbana-Champaign, Urbana, Illinois, United States of America; University of Virginia, UNITED STATES

## Abstract

Using protein counts sampled from single cell proteomics distributions to constrain fluxes through a genome-scale model of metabolism, Population flux balance analysis (Population FBA) successfully described metabolic heterogeneity in a population of independent *Escherichia coli* cells growing in a defined medium. We extend the methodology to account for correlations in protein expression arising from the co-regulation of genes and apply it to study the growth of independent *Saccharomyces cerevisiae* cells in two different growth media. We find the partitioning of flux between fermentation and respiration predicted by our model agrees with recent ^13^C fluxomics experiments, and that our model largely recovers the Crabtree effect (the experimentally known bias among certain yeast species toward fermentation with the production of ethanol even in the presence of oxygen), while FBA without proteomics constraints predicts respirative metabolism almost exclusively. The comparisons to the ^13^C study showed improvement upon inclusion of the correlations and motivated a technique to systematically identify inconsistent kinetic parameters in the literature. The minor secretion fluxes for glycerol and acetate are underestimated by our method, which indicate a need for further refinements to the metabolic model. For yeast cells grown in synthetic defined (SD) medium, the calculated broad distribution of growth rates matches experimental observations from single cell studies, and we characterize several metabolic phenotypes within our modeled populations that make use of diverse pathways. Fast growing yeast cells are predicted to perform significant amount of respiration, use serine-glycine cycle and produce ethanol in mitochondria as opposed to slow growing cells. We use a genetic algorithm to determine the proteomics constraints necessary to reproduce the growth rate distributions seen experimentally. We find that a core set of 51 constraints are essential but that additional constraints are still necessary to recover the observed growth rate distribution in SD medium.

## Author summary

No two living cells are exactly the same. Even cells from a clonal population with identical genomes living in the same environment will express proteins in different numbers simply due to the random nature of the chemistry involved in gene expression. The consequences of this stochastic gene expression are complex and not well understood, especially at the level of large reaction networks like metabolism. Here we investigate how variability in the copy numbers of metabolic enzymes affects how individual cells extract nourishment from their environment and grow. We model 100,000 independent yeast cells, each with their own set of enzyme copy numbers sampled from experimental distributions, and use flux balance analysis (FBA) to compute the optimal way that each cell can use its metabolic pathways—an approach we dubbed Population FBA. We find that enzyme variability gives rise to a wide distribution of growth rates, and several metabolic phenotypes—subpopulations relying on diverse metabolic pathways. Most importantly, we compare the predicted fluxes through the different pathways to experimental values; we find that Population FBA is able to correctly predict Crabtree effect, while traditional FBA, which lacks the proteomics constraints our method imposes, differs both qualitatively and quantitatively from experiment.

## Introduction

A cell’s phenotype—its set of distinguishing observable traits—can be as much an emergent property of the cell’s environment and gene expression state as it is a result of the cell’s genotype. While some observables, like an organism’s response to Gram staining, can be immutable and tied to specific genes, others can be more fluid, varying from cell-to-cell with the random fluctuations in each cell’s molecular makeup [1–4]. A cell might by chance over- or under-express the enzymes involved in a given biosynthetic pathway, in which case the over- or underproduction of that pathway’s end product might signify a naturally occurring phenotype. Understanding this type of phenotypic variability requires models capable of connecting comprehensive gene expression profiles with cellular function.

Constraint-based methods like flux balance analysis (FBA) have proven to be among the more successful approaches to modeling complex enzyme-mediated biochemistry at the cell scale (for recent reviews and a primer, see [5–9]). In its simplest form, FBA seeks the flux distribution through a biochemical network that maximize the production of some specific objective, like biomass, while requiring that the concentrations of all other metabolites remain fixed (*i.e.* the flux into and out of each metabolite is *balanced*). Parsimonious FBA (pFBA) improves on the predicted flux distribution [10] by minimizing the total flux through all reactions while maintaining optimal objective function. Minimizing total flux reduces the number of feasible flux distributions and represents efficient enzyme usage by the cell. By imposing constraints on the flux allowable through certain reactions (such as substrate uptake reactions, or reactions catalyzed by mutated, knocked-out, or low-copy number enzymes), different environments, genetic perturbations, or gene expression states can be modeled. The use of FBA and related techniques has grown to include a large user-base that actively contributes to the development of both methods and models, and metabolic reconstructions now exist for a variety of model organisms ranging from bacteria and yeast up through humans [11–15]. A particularly vibrant area of research in the field has been the use of large -omics data sets to constrain models in ways that reflect the influence of the cell’s regulatory machinery. RNA microarray and RNA-Seq data can be used to impose reaction constraints according to the expression levels of the genes that encode their associated enzymes [16–20]. More recently the development of coupled metabolism and expression (ME) models has allowed for the direct prediction of the enzyme expression state that optimizes growth, yielding results that agree with experimental data sets [21]. While these methods yield insight into the average behavior of a population, they say little about cell-to-cell variability among sub-populations.

**Fig 1:**
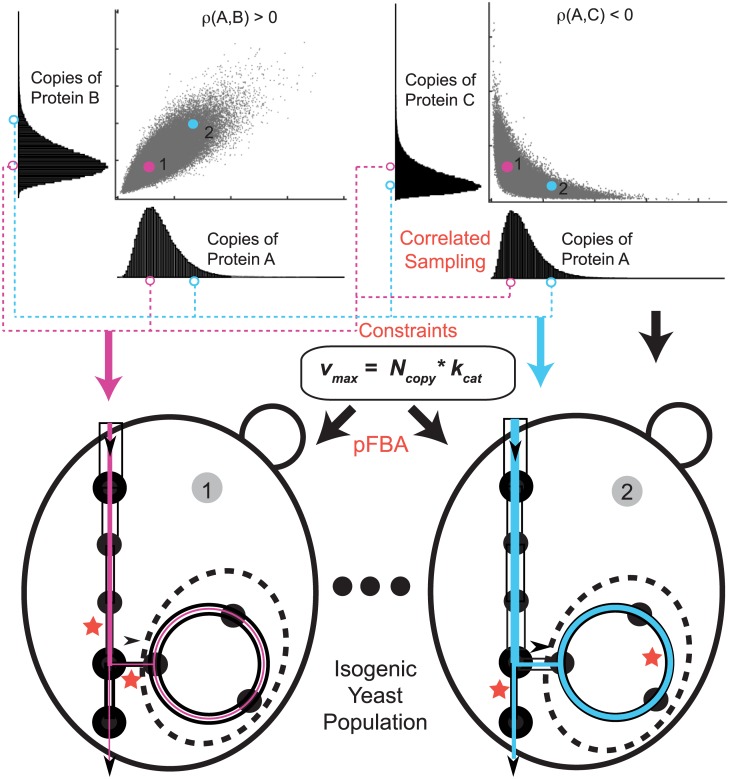
Population FBA methodology with correlated protein sampling. An isogenic population of yeast is created by correlated sampling of distributions of metabolic proteins obtained from fluorescence microscopy experiments. ρ(x,y) represents Pearson correlation coefficient between the proteins x and y. Assuming Michaelis-Menten kinetics whereby *v*_max_ = *N*_*copy*_ × *k*_cat_ (where *N*_*copy*_ is protein copy number and *k*_*cat*_ is the turnover number of the protein) the sampled enzyme copy numbers are used to impose upper bounds (*v*_max_) for the possible flux through their associated reactions. The correlations are obtained from microarray expression data. For two representative cells, the black hollow bars represent *v*_max_ values imposed on the reactions of an idealized network, while the solid color lines represent the actual flux that would be predicted to flow through the network. In each case, certain reactions, marked with red stars, constrain the flux through the network. Parsimonious Flux Balance Analysis (pFBA) [10] is used to calculate growth rate and flux distributions for each of the member of the population.

**Fig 2:**
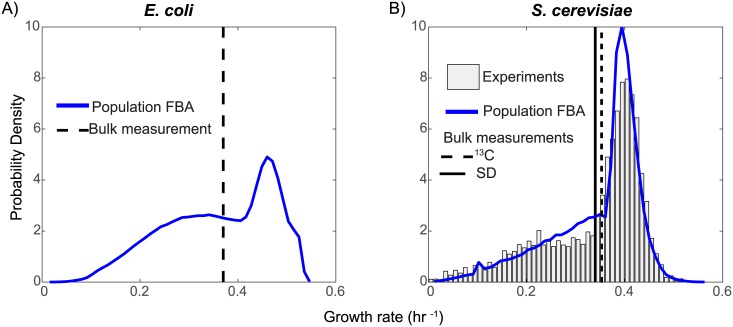
Growth rate distributions. (A) Predicted growth rate distribution from Population FBA analysis of *E. coli* with correlated sampling of protein distributions. Experimental bulk growth rate marked by black dashed line [34]. (B) Predicted growth rate distributions for 100,000 cells in glucose synthetic defined medium (SD) (blue). Also shown is the experimentally measured growth rate distribution in SD medium and ^13^C medium [38, 40]. Simulated growth curves have correlated sampling from protein distributions and mRNA microarrays. Growth rate distributions are made by binning growth rates into 50 bins.

Heterogeneity in gene expression has been the subject of intense experimental and theoretical research over the past several years [22–32], but relatively few studies have attempted to understand its effects on cellular function [33, 34]. Gene expression is known to correlate with growth rate [35]; Labhsetwar *et al.* [34] developed the Population FBA methodology (Fig 1) in order to show that by sampling experimentally determined enzyme copy number distributions in a correlated fashion and using them as constraints on a genome-scale model of *Escherichia coli* metabolism, independently simulated cells exhibit a broad distribution of growth rates and several behavioral phenotypes (*e.g.* some cells secrete acetate while others do not, or some cells make heavy use of the Entner-Doudoroff pathway while others predominantly use the Embden-Meyerhof-Parnas pathway). This study was made possible by the results of Taniguchi *et. al.*’s groundbreaking fluorescence microscopy investigation of protein expression in single cells with single molecule sensitivity [36]. With recent developments in microscopy and microfluidics, a number of research teams have begun to report direct observations of single-cell growth rates [37–39]. Intriguingly, growth rate distributions reported in yeast [38] bear a striking resemblance to that predicted in the Labhsetwar *et. al.*, article; in particular both show a broad “shoulder” of slow-growing cells and a distinctive peak of fast-growers (see Fig 2) [34, 38].

Here we extend the Population FBA approach developed in [34] in order to predict and characterize emergent metabolic phenotypes within yeast populations. Employing the yeast 7.6 metabolic reconstruction—the latest and most complete and predictive genome-scale metabolic reconstruction of *Saccharomyces cerevisiae* to date [14, 41]—along with comprehensive proteomics [42] and microarray [43] data sets, we construct highly realistic populations of independent *in silico S. cerevisiae* cells in two different growth media (the first, denoted SD represents the same synthetic defined medium used in [42], while the second, denoted ^13^*C*, represents the minimal medium used in a recent experimental fluxomics study [40], See Table A1 in S1 Text). By ensuring these populations realize experimentally observed growth-rates, levels of gene expression, and correlations among co-regulated genes, we are able to create detailed models of the intracellular metabolic fluxes of every individual cell (∼ 100,000). We show that the Population FBA methodology using scaled protein counts and the yeast 7.6 metabolic reconstruction give quantitative and qualitative agreement with experimentally observed intracellular fluxes (as determined by a ^13^C study [40]). The use of transcriptomics data in order to impose correlations among co-regulated genes marginally improves the fidelity of our predicted intracellular fluxes. We then characterize the dominant metabolic phenotypes within our modeled populations. Specifically we find a shift in the balance between fermentation and respiration among fast-growing cells, we find cells in amino acid-rich media that make use of a complex set of reactions involving the glycine cleavage system, we find cells in minimal media that leverage the pentose phosphate pathway in order to conserve NADH, and we find slow-growing cells whose uptake of certain amino acids from the media exhibits a distinctive bimodality. And finally, we characterize the degeneracy of the possible sets of enzyme-related constraints that can give rise to the experimentally observed growth rate distributions.

## Methods

### Model, software, and Population FBA methodology

The consensus yeast metabolic model version 7.6 [14] was chosen to describe the metabolic pathways in our simulations. This model (available from yeast.sourceforge.net) represents the most comprehensive yeast metabolic reconstruction to date [41]. All FBA calculations were performed using the COBRA toolbox version 2.0 [44] or COBRApy [45]. Gurobi 6 was used to perform all linear programming optimizations. Flux variability analysis (FVA) was performed using COBRA function fluxVariability() to determine robustness (minimum and maximum) in flux values given a percentage optimality. FVA was also used to identify proteins with significant copy numbers but zero predicted flux in their associated reactions.

For every cell in our modeled populations we sampled fluorescence values out of 535 experimentally determined distributions and converted them to enzyme copy number using Eq 1 (see Methods Section *Conversion of fluorescence to protein copy numbers and scaling*). Each sampled enzyme copy number was paired with a turnover rate corresponding to that enzyme’s function (*k*_*cat*_), and the product of these and a conversion factor yielded the upper bounds for the fluxes through the reactions catalyzed by each enzyme in each cell (See Methods Section *Constraint relaxation for realistic growth*). The conversion factor used was 3.0 × 10^−7^ s cell^−1^ mmol gDwt^−1^ hr^−1^, given by the number of seconds in an hour (3,600) divided by the average dry mass of a haploid yeast cell (2.0 × 10^−11^ g [46]) and the number of particles in a mmol (6.02 × 10^20^). In cases where multiple enzymes catalyze a given reaction, Gene-Protein-Reaction (GPR, part of the metabolic model) rules were used to determine the effective upper bound for the reaction from the upper bounds calculated for the individual enzymes. In cases involving “AND” relationships (*i.e.* an enzyme is made up of two subunits and both need to be present), the minimum of the individual upper bounds was used, whereas in cases involving “OR” relationships (*i.e.* different proteins can catalyze the same reaction), the sum of the individual upper bounds was used. If a count was missing for one of the enzymes involved in an “OR” relationship, the upper bound was left at the default value of 1,000 mmol gDwt^−1^ hr^−1^. After setting all protein-associated constraints, parsimonious FBA [10] was performed in order to predict the internal fluxes of each modeled cell.

### *In silico* growth conditions and strains

Upper bounds for the uptake substrates were applied depending on the growth medium being modeled. The SD medium included glucose, 19 amino acids, uracil, citrate, vitamins, and minerals; the upper bounds for the amino acids, uracil, citrate and the vitamins were estimated based on experimental data [47] (when no data was available the maximum experimental uptake was set, See Table A1 in S1 Text), those for oxygen and the minerals were unconstrained, and glucose upper bound was scaled to match experiment [40]. The strain, BY4741, used in the growth rate distribution [38] and proteomics [42] studies—both grown in SD medium—contained several gene deletions, including *his3*Δ*1*, *leu2*Δ*0*, *met15*Δ*0*, and *ura3*Δ*0*. To account for this, the genes YCL018W, YLR303W, YEL021W were inactivated, leading to zero flux being allowed through five reactions: 3-isopropylmalate dehydrogenase (r_0061), cysteine synthase (r_0312), O-acetylhomoserine (thiol)-lyase (r_0812, and r_0813) and orotidine-5-phosphate decarboxylase (r_0821). The histidine biosynthesis knockout is recovered when GFP is tagged to any protein, so the gene YOR202W was kept active.

The ^13^C medium included only glucose, some vitamins, and minerals (See Table A1 in S1 Text). As in the SD medium, vitamin uptake upper bounds were set based on experimental data [47], the oxygen and minerals were unconstrained and glucose upper bound was scaled to match experiment [40]. Glucose uptake upper bound of 20 mmol gDwt^−1^ hr^−1^ is also supported by Diderich *et al.* [48]. The strain used in the ^13^C medium, FY4 Mat a, is a wild-type strain, so no modifications were done to original yeast 7.6 model to simulate this.

### Conversion of fluorescence to protein copy numbers and scaling

**Fig 3:**
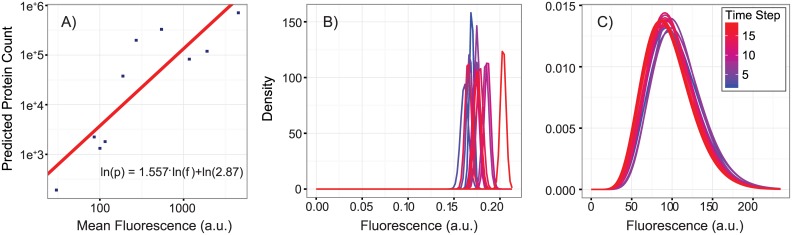
Fluorescence abundance distributions and conversion to protein counts. (A)Linear fit between ln() values of mean protein counts and mean observed fluorescence abundance. See Methods for the exact values and names of proteins used in the calibration. (B and C) Two examples of fluorescence abundance distributions for different proteins [42], the first set exhibit severe deconvolution problems and were removed from the data set (B), the second set exhibit fluorescence abundances with smooth distributions (C).

Protein abundances were obtained from single cell fluorescence measurements on yeast strain BY4741 grown on glucose SD medium [42]. The authors reported fluorescence distributions that were calculated from average pixel intensities over entire cells; we therefore considered all protein abundances to be size-normalized. For each GFP-labeled protein, Dénervaud *et al.* [42] deconvoluted the single cell fluorescence signal from the autofluorescence signal, and fitted the results to gamma distributions, providing shape and scale parameters for 4,159 proteins measured at 40 time points, taken 20 minutes apart (totaling 166,360 fluorescence distributions). Since the study aimed at observing changes in the proteome in response to stress, only 18 of the 40 time steps could be used for each protein (the ones before the induction of stress factors). Of the 74,862 remaining distributions, several displayed significant abnormalities, most likely resultant from the automated deconvolution procedure used to separate weak GFP fluorescence signals from the cell’s autofluorescence. The abnormality consisted of fluorescence distributions that were extremely narrow and usually had low mean fluorescence, hereafter referred to as “spikes”. Examples can be seen in Fig 3.

A multi-step procedure was developed to automate the processing of the almost 75 thousand fluorescence distributions, and when appropriate, censoring of spikey distributions. Only proteins that had data for all 18 time points were subjected to this process, which led to the removal of 59 proteins. First, a conservative lower bound of 0.1 was placed on standard deviations to remove the most obvious spikes, leading to the removal of all 18 time points for 7 proteins and a total of 2948 total fluorescence distributions being discarded across all proteins. Then, for each protein, the remaining distributions were used to determine a central reliable region for means and standard deviations, which were defined as the range from 1.5 times their IQR (inter quartile range) below the 25% quantile to 1.5 times the IQR above the 75% quantile. If a fluorescence distribution had either its mean or standard deviation outside this range, the distribution was discarded, leading to the removal of another 4175 distributions. After this step, only proteins that had 3 or more fluorescence distributions out of the original 18 were kept, which lead to the removal of another 3 proteins. Finally, the third step calculated the coefficient of variation (CV, defied as standard deviation over the mean) of the means and the CV of the standard deviation of the remaining distributions for all proteins. Only proteins whose distributions had both means and standard deviations with CVs lower than an upper bound of 0.5 were kept, removing 201 additional proteins. Proteins with mean fluorescence less than 7.98 A.U. were also removed because Dénervaud *et al.* [42] considered them unreliable. We found that these proteins had significantly less noise than proteins with means higher than 7.98 A.U. The final set of reliable fluorescence distributions represented a total of 3,647 proteins, which covered diverse cellular processes and compartments. The full dataset acquired after this process is reported in the S2 File, including parameters for fluorescence distributions in individual timesteps, and full plots for all fluorescence distributions used in our simulations.

The fluorescence distributions which were found to be reliable were then converted to absolute protein copy distributions. We used single cell quantification of 10 proteins (Table 1) from mass spectrometry (MS) [49] in order to relate fluorescence values to single cell copy numbers (Fig 3). The quantitative protein abundance from the MS study were determined using the same yeast strain as used in Dénervaud *et al.*, but were grown on complex media. In order to estimate protein counts for synthetic defined (SD) media, we used expression ratios observed in a single cell proteomics study [50], where protein abundances were measured in both complex and synthetic defined media. Finally, a linear fit between log values for protein counts and fluorescence was used to obtain the Eq (1) for converting fluorescence into protein counts:

$$\begin{array}{c} p=2.87*f^{1.5577} \end{array}$$ (1)

where *p* represents the single cell protein copy number, and *f* represents the fluorescence value. During sampling, we ensured a lower bound of 2.87 for all enzymes (if a copy number was sampled lower, it was replaced with 2.87), This was because we expect fluorescence values less than 1 to be unreliable.

**Table 1:** Proteins used in conversion from fluorescence abundance to protein counts.

Gene	Mean Protein [49]	Mean Fluorescence (a.u.) [42]	Ratio (SD/YEPD) [50]
YIL084C	162	26.7249	1.18
YKL145W	2114	100.3195	0.62
YHR107C	2317	113.8195	0.78
YEL031W	3125	85.5069	0.72
YBR249C	19459	185.6222	1.94
YHR183W	48926	1128.4	1.69
YLR058C	98940	267.899	2.01
YLR249W	189235	1904.1	0.63
YJL136C	370314	543.9343	0.89
YKL060C	996503	4462.6	0.71

See Methods Section *Conversion of Fluorescence to Protein Copy Numbers* and Fig 3 for details on the conversion of fluorescence abundances to proteins count distributions.

Protein counts were scaled in case of simulation for ^13^C medium because the protein distributions measured by Dénervaud *et al.* are in SD medium. Ratios to scale the protein counts were found from microarrays comparing gene expression of cells grown in SD medium to cells grown in SD medium without amino acids for 6 hours after being transferred from SD medium [51]. Top 10 proteins downregulated and top 10 proteins upregulated in minimal medium are shown in Table A2 in S1 Text.

### Correlated sampling of protein counts

Microarray datasets from Kemmeren *et. al.* [43] (available from the GEO database, Accession No. GSE42528), were used to calculate correlation coefficients among the 532 out of 535 metabolic proteins we sampled. Rest of the three proteins were not measured in these microarray experiments. This microarray data is well-suited for our study because it was produced using an almost identical strain of yeast, BY4742 which has same deletions but different mating type, and under similar growth conditions as that used in the proteomics study we rely on [42]. Absolute fluorescence values for the sample channel of the two-channel microarrays were used; because almost all of the genes evaluated had two probes on the microarray chip (Accession no. GPL11232), the mean value of the two probes was computed. Fluorescence values were then quantile normalized across the entire set of microarray data [52]. Correlation coefficients were calculated from these normalized fluorescence values. These correlation coefficients were then used to create correlated samples of protein counts using the usual Cholesky decomposition methodology [34].

The correlations observed show clear biological relevance. For example, the Crabtree effect, which is well known in *S. cerevisiae*, can be seen in the positive correlations among the Glucose transporter HXT1 and genes in the fermentative pathway as well as the negative correlations between HXT1 and genes involved in the TCA cycle and oxidative phosphorylation (see Fig A11 in S1 Text). Moreover, the correlations we see recover many experimentally known regulatory links in yeast (see Fig A12 and Section *Reliability of mRNA Microarray Correlation Data* in S1 Text).

### Constraint relaxation for realistic growth

Without internal constraints, the metabolic model *iJO1366* for *E. coli* and the yeast version 7.6 model both return higher growth rates for a given glucose uptake rate than is experimentally observed. However, as previously reported in the population studies on *E. coli* [34], imposing all of the possible constraints arising from the measured protein distributions and turnover rates does not allow the population to grow. The problem lies with either some of the protein counts or some of the turnover rates. In converting the fluorescence data to protein distributions, we already removed spurious data and low counts, so we were confident in the remaining distributions. Moreover, because a third of the *k*_*cat*_ values obtained from BRENDA have changed in the span of a year, we chose to keep the protein counts and raise the appropriate turnover rates in order to allow for growth. To deterministically find problematic turnover rates, we iteratively simulated populations of 400 cells and identified the reaction whose flux most often reached its imposed upper bound. *k*_*cat*_ for the enzyme associated with that reaction was doubled. If that reaction was catalyzed by multiple proteins, we doubled the *k*_*cat*_ value for the protein with highest protein mean count in case of isozymes (‘OR’ relationship) and all the subunits in case of an protein enzyme complex (‘AND’ relationship). We continued this procedure iteratively until the mean growth rate of the sampled population reached 0.35 hr^−1^, the bulk growth rate measured in both the proteomics [42] and single cell (microcolony) experiments [38]. First round of doublings helped us to focus on proteins which needed excessive doublings and hence manual search for those *k*_*cat*_ values was performed and any higher *k*_*cat*_ reported in literature was accepted. Manually found *k*_*cat*_ values can be found in Table A3 in S1 Text. Before going through the doubling procedure, we also raised *k*_*cat*_ values of all subunits in a protein complex to the highest *k*_*cat*_ among the subunits.

### Analysis of sub-populations

Each yeast cell in our modeled populations had a unique protein copy number for 535 genes, and a unique flux distribution throughout the metabolic network of over 3,400 reactions. Different fluxes in this metabolic network are linearly dependent on each other and constitute metabolic pathways. To find pathways that were differentially used by different segments of our modeled populations, we used principal component analysis (PCA) as implemented in MATLAB’s pca() function to elucidate orthogonal directions (in the 3,400-dimensional flux-space) in which the cells in our populations varied most. We chose 1,000 cells at random from the population for this analysis. Since the members of this population grew at different growth rates, we normalized all fluxes by the cell’s growth rate, allowing us to identify growth-independent differences in pathway usage. This methodology is similar to that used previously [34], but we didn’t need to rotate the components coming out of PCA as they aligned with canonical metabolic pathways.

### Genetic algorithm for constraint selection

A new procedure for filtering overly-constraining turnover rates based on the Micro Genetic Algorithm (GA) formalism was developed [53]. This method utilizes an entire growth distribution as a target for optimizing the selection of experimental constraints. Micro Genetic Algorithm was chosen instead of a “regular” Genetic Algorithm solely for computational cost concerns. In a “regular” GA algorithm in dozens to hundreds of genomes would have to be simulated at each generation, and several hundred generations could need to be evaluated to reach the same results. The computational cost would be extremely higher as compare to our GA implementation. In our attempt to reduce the size of search space we have restricted GA variables to binary values representing weather to use a particular *k*_*cat*_ or 38,000 *s*^−1^ rather than more flexible values *k*_*cat*_ can take in the doubling procedure. Briefly, a population of 10 “genomes” was simulated, each one composed of a list of “genes” that indicated if a protein’s *k*_*cat*_ would be kept at its BRENDA value, or if it would be raised to 38,000 s^−1^. The genomes were allowed to evolve by exchanging information, and each new generation was created by a random selection of solutions biased by their fitness, while always taking the best solution to the next generation (see SI Section *Extended Methodology: Genetic Algorithm for Constraint Selection for details*). The fitness of each genome was determined by simulating a cell population based in its *k*_*cat*_ selection, and then calculating the goodness-of-fit between the resulting growth rate distribution and the observed distribution [38].

## Results and discussion

### Modeling *S. cerevisiae* populations in SD and ^13^C media

The basic Population FBA methodology has been described previously [34]. Briefly, enzyme copy numbers are sampled from experimentally-determined distributions [42] from a single cell proteomics study; each sampled set of enzyme copy numbers represents a unique cell in its own gene expression state. Assuming Michaelis-Menten kinetics, each copy number—paired with an appropriate enzyme turnover rate (*k*_*cat*_) —represents the maximum reaction flux that the cell can maintain through the reaction(s) mediated by that enzyme. Many genes are known to exhibit some correlation in their expression levels. For bacteria, [34], this effect was handled fairly simply; proteins in the “extrinsic noise limit”, noise floor observed in proteins with high means, were assumed to exhibit a correlation coefficient of 0.66 suggested by the single cell proteomics study [36]. Due to the availability of large transcriptomics datasets, we are now able to take a more refined approach in which we systematically impose the types of correlations that should naturally arise among the copy numbers of co-regulated proteins. This was accomplished by extracting correlation coefficients for ∼ 4,000 *S. cerevisiae* gene products from an expansive collection of microarray gene expression datasets [43] and using them to draw correlated sets of protein copy numbers Constraints of this type were then imposed throughout a genome scale flux balance model of metabolism, and parsimonious flux balance analysis (pFBA) [10] was used to predict each cell’s metabolic behavior.

The copy number distributions that were used were adapted from a recent article by Dénervaud *et al.* [42] for yeast grown in SD medium. The authors used a GFP fusion library spanning 4,159 *S. cerevisiae* proteins and a unique parallel microchemostat microfluidic device to measure single cell fluorescence intensity distributions—representative of protein expression distributions—for approximately 2/3 of the yeast proteome. Intensities sampled from these distributions were transformed to copy numbers using a calibration curve (see Fig 3A). Several of the measured fluorescence distributions were abnormally “spikey,” likely as a result of poor deconvolution of the GFP signal and the cell’s own autofluorescence (see Fig 3B and 3C). We removed these spikey distributions by determining which distributions had abnormal means and standard deviations using a simple outlier-detection protocol (see Methods Section *Conversion of fluorescence to protein copy numbers and scaling*). Among the remaining 3,885 distributions, those with mean fluorescence lower than 7.98 A.U. (as measured by Dénervaud *et al.* [42]) had significantly lower noise than the proteins with similar means hence they were also removed. The 3,647 distributions that remained after this censoring procedure showed noise characteristics that agreed qualitatively with previously published results in *E. coli* (see Fig A1 in S1 Text). Only 535 of these remaining distributions were associated with enzymes involved in the yeast 7.6 metabolic reconstruction [14] (see S1 File), and thus only these were used in our study. We would like to note that GFP is extremely stable protein which might affect stability of tagged protein and hence bias the protein counts towards higher number than their numbers in untagged cells. Metabolic reconstruction of yeast accounts for 13 compartments which represent various organelles and their membranes. All metabolites are assigned to one of these compartments and reactions are either localized in a compartment if all the reactants and products are present in the compartment or facilitate transport of metabolites across compartments. When we associate an enzyme with a reaction using the gene-protein-reaction associations of the reconstructions, we assume all the copies of the enzyme are available to the reaction it is associated with. So even though the copies of the enzyme might be spread out over multiple compartments in real cells, in lack of that information we make all the copies available to all the reaction the enzyme is associated with.

Two sets of simulations were performed, corresponding to the two different environmental conditions. The first was intended to replicate the cell growth media used in a study ([40]) of ^13^C-labeled glucose utilization by several strains of yeast. This was done in order to accurately compare our predicted intracellular metabolic fluxes with those determined experimentally (see Results Section *Population FBA yields intracellular fluxes that agree with* ^13^*C fluxomics data*). The synthetic defined (SD) medium replicates the conditions used in single cell proteomics [42] and growth rate distribution studies [38]. This SD media included approximately the same concentrations of salts, double the glucose, and several metabolites not present in the ^13^C media. These included 19 amino acids (including the histidine, leucine, and methionine necessary for the growth of the *his3*Δ*1*, *leu2*Δ*0*, and *met15*Δ*0* experimental strain), as well as citrate, and uracil (necessary for the *ura3*Δ*0* also present in the experimental strain). Our modeled cells contained the same knockouts as the cells used in the experiments. Modeling of ^13^C media involved modifying relevant uptake rates in the metabolic model and scaling protein copy numbers measured in SD media to ^13^C media. Details of both modeled media and rescaling can be found in Section *Extended Methods: Metabolic Model and Experimental Data* and Table A1 and Table A2 in S1 Text. We assume that the relative composition of biomass is the same in all members of the population, although some experiments indicate the composition may change as a function of the growth rate [54, 55].

### Relaxation of imposed constraints

FBA models in general are underdetermined. By adding constraints in the form of reaction upper- and lower-bounds, modelers are able to whittle down the solution space (the right null space of the stoichiometry matrix) to the flux distributions that most accurately describe real cells ([7]). Metabolic reconstructions already include topological and thermodynamic constraints in terms of stoichiometric matrix and reaction reversibilities. Additional constraints are also routinely added to reflect the genetics of the strain (for example by fixing the flux through a reaction mediated by a “knocked-out” gene to zero) as well as the growth medium used (for example, by limiting the uptake of substrates absent from the media to zero). In Population FBA we add additional constraints based on protein copy numbers and their kinetic capacity. After censoring proteomics data to remove unreliable distributions, we believe we have good quality of protein copy number distributions. As for the kinetic capacity, we rely primarily on the BRENDA database [56, 57]. The BRENDA database often contains several sets of kinetic parameters for a given reaction. These can include values for enzymes from different organisms, strains, and most often *in vitro* conditions. A recent study concluded that *k*_*cat*_ measured *in vitro* generally agree with max *k*_*cat*_ *in vivo* estimated using omics data [58]. Whenever possible the largest *k*_*cat*_ value available for a wild-type *S. cerevisiae* strain was taken, otherwise the largest value reported for any mutant or other species was used. If no *k*_*cat*_ was available for an enzyme-mediated reaction, a value of 38,000*s*^−1^ (corresponding to the largest *k*_*cat*_ reported for a wild-type yeast enzyme in BRENDA) was set. These criteria were adopted in order to minimally constrain the model.

Importantly, the 535 sampled enzymes and *k*_*cat*_ values could in principle be used to impose constraints on 1,128 of the model’s 3,493 reactions (each enzyme catalyzes two reactions on average), but it was found that imposing all of these constraints impedes the growth of the modeled cells to levels well below that seen experimentally. Several enzyme turnover rates were found to have published values well below those necessary to allow realistic growth. For example, phosphofructokinase (PFK), which is made up of two subunits, had mean copy numbers measured to be 103,880 (*α* subunit) and 47,919 (*β* subunit) and a reported turnover rate of 62 s^−1^ (See Table 2). This led to a maximum reaction flux of 1.16 mmol gDwt^−1^ hr^−1^—approximately ten-fold smaller than the experimentally measured ^13^C glycolytic flux [40]. In cases like this, the *k*_*cat*_ values, which are relatively uncertain (reported values for phosphofructokinase, for example, range over four orders of magnitude [57]), were “doubled” until the mean population growth rate of 0.35 hr^−1^ was achieved (see Methods Section *Constraint relaxation for realistic growth* for details). Our doubling methodology involves iteratively generating small populations of modeled cells (400) and then determining which reaction most constrains cellular growth, and doubling its *k*_*cat*_. This strategy revealed that certain enzymes required excessive numbers of doublings (for example, the *k*_*cat*_ for Glycogen Synthase was doubled 19 times in our ^13^C simulations). These rates were investigated further, and in many cases we were able to find significantly higher *k*_*cat*_ values in the literature than were reported in BRENDA (See Table A3 in S1 Text). Even after including *k*_*cat*_ values from literature, some protein’s *k*_*cat*_ needed significant doubling e.g Acetylornithine aminotransferase needed 13 doublings. We also found *k*_*cat*_ for NAD dependent methylenetetrahydrafolate dehydrogenase (YKR080W), was wrongly listed in BRENDA as 1.63 s^−1^ and needed 12 doublings in simulation for SD medium. The correct *k*_*cat*_ is 1,643 s^−1^ [59] which is much closer to the value required to sustain flux in SD medium (6,676 s^−1^, Table A3 in S1 Text) and would be obtained after only 2 doublings. We note that approximately 1/3 of the *k*_*cat*_ values taken from the BRENDA database have changed within just the past year, further casting doubt on the reliability of these parameters and suggesting the need for further consistency checks among the metabolic fluxes.

**Table 2:** Examples of *k*_*cat*_ values from BRENDA and the minimal values required to produce the reaction fluxes measured in ^13^C fluxomics experiments. The minimal *k*_*cat*_ is obtained by dividing ^13^C fluxes by mean protein counts using the Gene-Protein-Reaction (GPR) rules. The final *k*_*cat*_ is obtained by the doubling procedure to reproduce a mean growth rate of 0.35 hr^−1^, and the final values of the associated mean *v*_*max*_ values obtained by taking mean *v*_*max*_ over 1000 cells from a population. Factor of 3.0 × 10^−7^ converts the units from cell^−1^ s^−1^ to mmol gDwt^−1^ hr^−1^. When more than one enzyme is responsible for a reaction, the GPR rules are used to determine how to process protein counts (sum of subunits in case of ‘or’ and minimum count among the subunits for ‘and’).

Reaction	Proteins	Mean Protein (×1,000)	Measured Flux (mmol.gDwt^−1^.hr^−1^)	minimal *k*_*cat*_ (s^−1^)	BRENDA *k*_*cat*_ (s^−1^)	final $k_{cat}^{*}$ (s^−1^)	Final *v*_*max*_ (mmol.gDwt^−1^.hr^−1^)
Glycolysis							
HX	HXK1 or HXK2 or GLK1	190, 156, 2.8	15.2	144.5	200, 200, 1490	200, 800, 1490	50.4
PGI	PGI1	98	13.8	466.9	3330	3330	98.3
PFK	PFK1 and PFK2	104, 48	13.8	959.1	62, 62	3968, 3968	56.4
FBA	FBA1	634	13.1	68.6	16.7	267	49.6
TIM	TPI1	9.6	11.84	4093	16700	33400	99
GAPDH	TDH1 or TDH2 or TDH3	9.9, 238, 1300	24.94	53.80	16.7, 16.7, 16.7	16.7, 16.7, 267	103
PGK	PGK1	1850	24.94	44.88	963	963	535
PGM	PGM1	170	24.94	488.06	490	1960	96.6
ENO	ENO1 or ENO2	180, 2350	24.94	32.83	78, 78	78, 156	113
PK	PYK2 or CDC19	.90, 125	24.94	657.8	232, 232	232, 3712	141
Citric Acid Cycle							
CS	CIT1 or CIT3	5.2, .36	0.4	237.8	2029, 167	2029, 167	3.22
MDH	MDH1	.68	0	0	8570	8570	1.72
Fermentation							
PYRDC	PDC1 or PDC5 or PDC6	167, .63, .35	23.2	459.7	12.4, 10.3, 9.21	6359, 10.3, 9.21	326
ALCD	ADH1 or ADH5	-, .09	23.2	-	895, 895	-	-

* The proteins with the most *k*_*cat*_ doublings are involved in arginine (ARG8), fatty acid biosynthesis (OLE1) and serine (SER1) biosynthesis respectively and shown in Table A3 in S1 Text. The full list of counts and *k*_*cat*_ values for the metabolic model can be found in S1 File.

### *S. cerevisiae* exhibit a broad distribution of growth rates

In both modeled environments, overly-constraining enzyme turnover rates were doubled until the mean population growth rate matched that reported in the respective experimental studies. In total, 391 doublings were performed which affected 121 *k*_*cat*_ values in order to match the SD media growth rate of 0.34 hr^−1^ reported in [42], while 506 doublings were performed which affected 146 *k*_*cat*_ values to match the ^13^C media growth rate of 0.35 hr^−1^, reported in [40]). In each case, the resulting population exhibited a spectrum of specific growth rates that ranged from nearly 0.0 to over ∼ 0.57 hr^−1^. *Importantly*, *although only the mean growth rate was fit*, *the distribution for the SD medium was nearly identical to the experimentally measured distribution [38]* *shown in* Fig 2b. While a mean growth rate was reported, the corresponding growth distribution curve is not available for cells grown in ^13^C medium. Sampling from protein distributions that have been rescaled for ^13^C medium allows us to predict such a curve (See Fig A3 in S1 Text). Both modeled populations exhibited the same broad shoulder of slow-growers, and prominent peak of fast-growing cells seen in experiments.

The fast-growing peak was predominantly the result of limitation in glucose uptake (although limitation in certain amino acids also contributed in the SD media). While slow-growing cells utilized glucose at rates below the maximum allowed rate; see Fig A5 and Fig A8 in S1 Text), the fast-growers did tend to reach their limit. This common glucose limitation resulted in most of these cells having very similar growth rates, which in turn resulted in the pronounced peak we see. The broader tail of slow-growing cells was the result of important enzymes being sampled at low copy numbers. For example, sampled upper bounds on the ATP synthase and ubiquinol-ferricytochrome c reductase reactions, which participate in respiration, limited the growth of approximately 50% of the slow-growing subpopulation (those growing at rates less than 0.34 hr^−1^, see Fig A9 and Fig A6 in S1 Text).

A similar glucose-associated peak arose in work modeling the growth rate distribution of *E. coli* [34]. In light of this and the agreement we see between our simulated distributions and the experimental one, we wonder if substrate (e.g. glucose, amino acids) limitation could in general lead to similar peaked growth rate distribution across a range of organisms. It should be straightforward to investigate this experimentally. In the simplest case, growth rate distribution experiments similar to that of [42] could be carried out using media with varying concentrations of substrates. In a similar vein, the contribution of individual substrates to the peak could be investigated in strains in which the transporters of a substrate are expressed under the control of an inducible regulatory element. In *E. coli*, for example, the glucose transporter *ptsG* might be expressed under the control of the *lac* system—the shape and location of any peak in the growth rate distributions of cells grown with and without IPTG (a lactose analogue used in this case to induce *ptsG* expression) could then indicate whether glucose uptake limitation is responsible.

In order to identify the features of protein distribution which play important role in obtaining the shape of the distribution, we performed some control simulations. We randomly omitted 50% (267) and 66% (356) of the 535 available protein distributions and used the rest of the protein distributions to perform Population FBA. The resulting growth rate distributions are very similar to experimental growth rate distributions with a broad tail at slow growth rates and a peak at fast growth rates (See Fig A14 in S1 Text). In our population of 100,000 cells, only 165 out of the 3,493 reactions are ever constrained which is indicative of few proteins giving the shape to the growth rate distribution. We also tried to change all the protein distributions from gamma distribution to either normal or uniform distributions while keeping the mean intact. Resulting growth rate distribution had no resemblance to the experimental growth rate distribution (Fig A15 in S1 Text) emphasizing the importance of the shape of protein distributions. We have seen this before with *E. coli* [34] that very few proteins constraint the growth of majority of cells in the population.

### Population FBA yields intracellular fluxes that agree with ^13^C fluxomics data

Flux balance models can be evaluated across several different measures of predictiveness. Among the more common is the model’s ability to discern lethal and non-lethal gene knockouts. While this type of information can have important implications in synthetic biology and bioengineering [60–62], it remains a somewhat blunt measure of a model’s overall utility. The yeast 7 model was recently shown to accurately predict gene essentiality [41], but this does not necessarily translate into accurate network fluxes or realistic growth rates. Because our current work deals with the ability of our Population FBA methodology to predict pathway usage and growth rates, the most convincing test of our predictions is to compare them directly to experimental network flux measurements.

**Fig 4:**
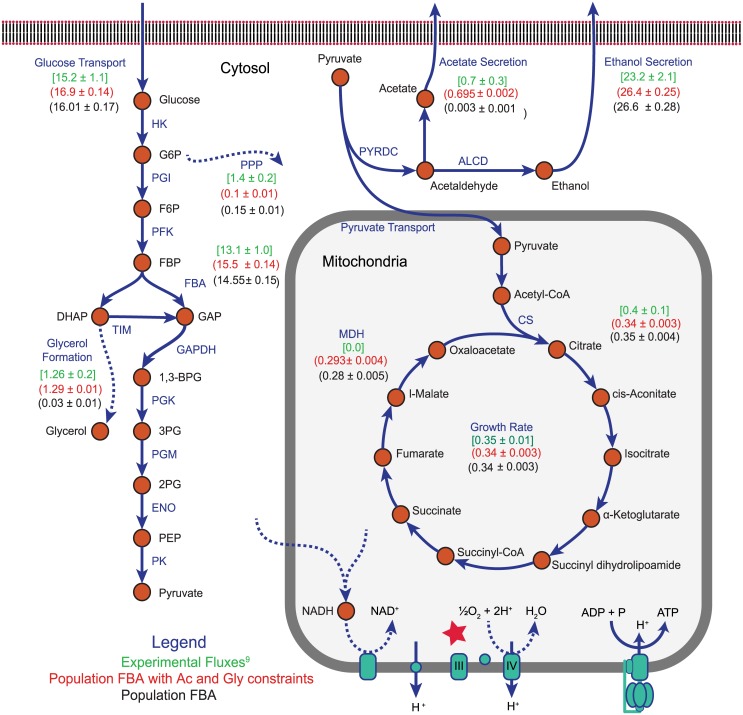
Depiction of yeast central metabolism covering glycolysis, TCA cycle and electron transport chain. Values next to reactions in the plot represent fluxes through those reactions. Values between parenthesis are derived from our simulations using yeast 7.6 model with (red) and without (black) constraints on acetate (Ac) and glycerol (Gly) secretion, whereas values between brackets (green) were taken from experimental ^13^C measurements [40]. A mapping between abbreviated reaction names and full names can be found in Table A4 in S1 Text. Constraint on ubiquinole-ferricytochrome c reductase reaction causes the Crabtree effect and is marked by red star.

Detailed measurements of intracellular metabolic fluxes are relatively scarce in the literature, owing in part to the cost and difficulty of such experiments. Nevertheless, a recent study used ^13^C-labeled glucose and some modeling to characterize eight fluxes involved in the central-metabolism of seven species of yeast [40]. These fluxes included glucose uptake, phosphogluconate dehydrogenase (in the pentose phosphate pathway), fructose bisphosphate aldolase (FBA), citrate synthase (CS), malate dehydrogenase (MDH) and the production of glycerol, ethanol, and acetate given in Table 2 and Fig 4 (for a full list of reaction names and abbreviations, see Table A4 in S1 Text). The authors found that *S. cerevisiae* (a “Crabtree-positive” yeast) respired little even under highly aerobic conditions. Using the wild-type yeast 7.6 model in ^13^C medium calculations were performed using our Population FBA methodology (see Methods Sections *Model, software, and Population FBA methodology* and *Constraint relaxation for realistic growth*, and Fig A3, A4 and A5 in S1 Text). The central metabolic fluxes of 1,000 simulated cells were compared to the experimental values. Comparison of these results with those predicted using FBA without proteomics constraints (Fig A2 in S1 Text) and our Population FBA methodology showed two important findings: 1) our Population FBA method imposes internal flux constraints in a manner that recovers the experimentally observed Crabtree effect, and yields mean network fluxes that by and large agree with experiment (with the exception of some underestimation of flux into the pentose phosphate pathway, and glycerol and acetate formation pathways, Fig 4); and 2) FBA without proteomics constraints fails to predict the Crabtree effect in *S. cerevisiae*.

#### Essential reactions for the Crabtree effect

Identical calculations (using the wild-type yeast 7.6 model in ^13^C medium) were performed using our Population FBA methodology. The central metabolic fluxes of 1,000 simulated cells were compared to experimental values. The mean predicted flux through each of the experimentally characterized reactions showed strong qualitative agreement (see Fig 4); not only was the Crabtree effect recovered, but the greatest absolute deviation between the any experimental flux and its corresponding simulated value was less than 3.4 mmol gDwt^−1^ hr^−1^ (corresponding to ethanol efflux, approximately a 14% error). One reaction in particular proved absolutely essential for prediction of significant fermentation in presence of oxygen and high glucose concentration or Crabtree effect. *The mean upper bound imposed on the ferricytochrome c reductase reaction, based on proteomics and kinetics data, was 1.20 mmol gDwt^−1^ hr^−1^. This is a key reaction in the electron transport chain within the mitochondria, and as a result of the low expression level of one of the proteins involved in catalyzing it (Subunit 8 of Coenzyme Q—cytochrome c reductase, QCR8, approximately 97 per cell) the simulations predicted a predominantly fermentative metabolism*. Several other mitochondrial proteins were expressed in small numbers and had similarly limiting effects on oxidative phosphorylation, including PDX1 (expressed at approximately 232 per cell) which catalyzes the pyruvate dehydrogenase (PDH) reaction—an important step linking glycolysis and the TCA cycle, and CIT3 (approximately 356 per cell) which catalyzes the citrate synthase (CS) reaction which is part of the TCA cycle. In general the protein copy numbers involved in the fermentation pathway are considerably higher. Had these flux constraints not been imposed, the model would have predicted greater amounts of respirative metabolism (see *FBA without Proteomics Constraints Spuriously Predicts Respiration in S. cerevisiae*), and would have been unable to recover the experimentally observed behavior. Importantly, we note that the agreement we found between experimental and simulated fluxes could not have been realized without raising several dubious turnover rates that had initially been collected from the BRENDA database (see Methods Section *Constraint relaxation for realistic growth*).

Previous attempts to predict Crabtree effect in *S. cerevisiae* using simple FBA approach included imposing experimentally determined oxygen uptake rate as constraint on the model [63]. With Population FBA we don’t need to explicitly restrict oxygen uptake rate but it emerges from the protein associated constraints on the network (See Fig A4A in S1 Text). Other approaches include using an alternative objective function for FBA optimization like maximizing the pathway flux [64] or game theoretic approach which account for protein cost of utilizing a pathway in addition to biomass yield [65]. We found that our method of imposing correlations among co-regulated genes (see Methods Section *Correlated sampling of protein counts*) was important but not essential to the agreement we see between our simulated fluxes and the experimental ones. As examples, the glucose uptake and ethanol secretion reactions were overestimated without imposed correlations by about 1.6 and 4.5 mmol gDwt^−1^ hr^−1^, respectively. With correlated sampling, these errors fell to only about 0.8 and 3.1 mmol gDwt^−1^ hr^−1^ (see Fig A7 in S1 Text). Overall, we found that the root-mean-squared-difference between the predicted and experimental mean fluxes was significantly lower with correlations (approximately 1.41, compared to 1.52 mmol gDwt^−1^ hr^−1^ without correlated sampling, and 7.98 mmol gDwt^−1^ hr^−1^ for FBA without proteomics constraints (Fig A2 in S1 Text)).

#### Underestimation of secretion products and partitioning to the pentose phosphate pathway

There are a few points of quantitative disagreement we observe between our predicted fluxes and those seen experimentally, most importantly in acetate and glycerol secretion, both of which were directly measured [40]. The experimental production rates for these byproducts were small (0.7 and 1.26 mmol gDwt^−1^ hr^−1^ for acetate and and glycerol, respectively); while our simulations do qualitatively predict small fluxes, we underestimate both production rates by a significant fraction. Acetate secretion, for example, was predicted to occur at a rate approximately 230-fold slower than was measured (see Fig 4). In order to understand the cause, we fixed the lower bound on acetate secretion to the experimentally observed value and investigated the reactions where fluxes changed significantly (relative to our simulations in which no bound on acetate secretion was imposed). This led to a very small decrease in mean growth rate and an increase in flux through the glycolysis pathway (which provides the carbon necessary for the increase in acetate production). This increase in glycolytic flux produced extra NADH (through the lower glycolytic glyceraldehyde-3-phosphate dehydrogenase (GAPDH) and acetaldehyde dehydrogenase (ALD) reaction steps) which was in turn oxidized via the glutamate synthase and cytosolic malate dehydrogenase reactions. Similar calculations were performed by imposing a lower bound on the glycerol secretion flux. As before, this resulted in a very slight decrease in mean growth rate and increased upper glycolysis flux (again providing the necessary carbon). Interestingly though, our simulations showed that because the glycerol production pathway branches off prior to the lower glycolysis steps that form NADH, the NADH necessary to reduce dihydroxyacetone phosphate to glycerol-3-phosphate *en route* to glycerol had to be produced elsewhere in the the metabolic model. This extra NADH it turned out was largely obtained by increased flux through glutamate synthase—but in the opposite direction observed when requiring acetate production. A final set of calculations requiring both acetate and glycerol production resulted again in increased glucose uptake, but this time because acetate and glycerol production have opposite NADH requirements, the impact on other NADH oxidizing or reducing reactions (like glutamate synthase and malate dehydrogenase) was largely canceled. NADH and NADPH metabolism remains an active area of investigation [66].

#### FBA without Proteomics Constraints Spuriously Predicts Respiration in *S. cerevisiae*

We performed FBA using the yeast 7.6 reconstruction without deletions (wild-type) and the ^13^C modeled media (Table A1 in S1 Text [40]). These calculations have no internal proteomics-based flux constraints imposed. The predicted central metabolic flux distribution differed markedly from experimental results (Fig A2 in S1 Text). No ethanol was produced by the modeled cells, but rather they were predicted to use the TCA-cycle to catabolize most of their glycolytic pyruvate. The reason for this is straightforward; respiration affords the microbes significantly greater ATP yield than does fermentation. Given the same glucose uptake rate, a respiring microbe can in principle grow faster than a fermenting one, and because FBA seeks the fastest-growing flux distribution, respiration is the strategy that is predicted. This also led to a predicted growth rate of approximately 1.9 hr^−1^—almost six times faster than the experimental growth rate of 0.35 hr^−1^.

### Analysis of metabolic phenotypes

Our modeled cells exhibited a range of different metabolic behaviors. In order to characterize the ways in which they tended to differentiate, we employed principal component analysis (PCA) in a manner similar to that described in [34] (see also Methods Section *Analysis of sub-populations*). The first two PCA components combined accounted for almost 85–95% of the total variability in pathway usage (Table A5 in S1 Text). Analysis of the loadings of the first component showed that it was associated with similar metabolic behaviors, namely a shift toward respirative metabolism by fast growing cells, regardless of environment. In SD growth medium the second PCA component was associated with the cell’s NADH/NADPH economy where fast growing cells employed Serine-Glycine cycle to generate NADH and NADPH. Finally, although not elucidated by PCA, a particularly compelling form of metabolic variability characterized by a bimodality in the utilization of certain amino acids was found to emerge among the slow-growing cells modeled in SD medium. Threonine, aspartate, asparagine, glutamate, and glutamine all showed little correlation with growth rate, but each was found to be either not taken up at all, or taken up at its maximal allowable rate (see Fig A8 in S1 Text). Variability in population behavior predicted by Population FBA in ^13^C growth medium is mostly one-dimensional (Table A5 in S1 Text), similar to the dimension identified in first component of SD medium population, where movement towards higher growth rate implies restricted fermentation and increased respiration (See Fig A4 in S1 Text).

#### Fermentation vs. respiration

**Fig 5:**
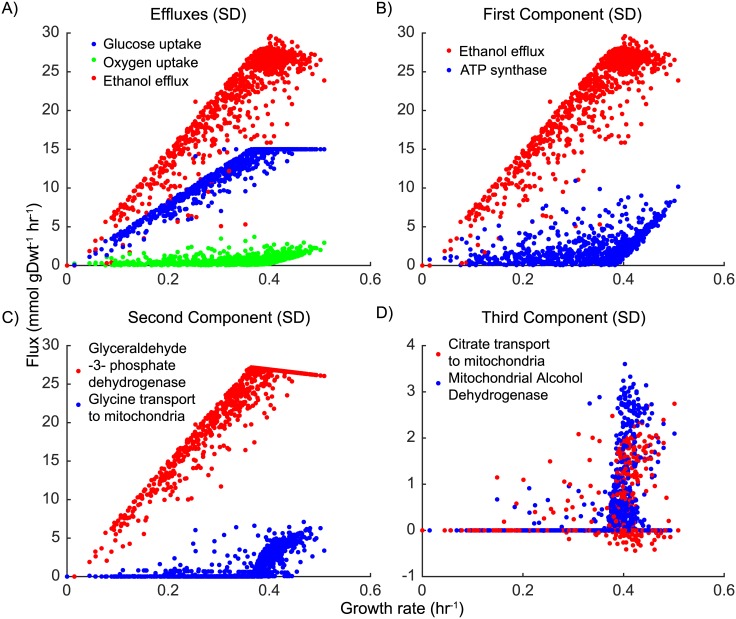
Analysis of metabolic fluxes from yeast 7.6 simulations in SD and ^13^C media. (A) Glucose uptake, oxygen uptake and ethanol efflux from simulations in SD medium. (B) First PCA component showing fast growing cells performing respiration along with fermentation. (C) Second PCA component showing transport of glycine to mitochondria as part of Serine-Glycine cycle to produce NADH or NADPH which compensates for reduced NADH production due to limited fermentation due to glucose limitation (D) Third PCA component from SD medium simulations shows burning of NADH by mitochondrial alcohol dehydrogenase produced in Serine-Glycine cycle and transport of citric acid to mitochondria, all by fast growing cells.

**Fig 6:**
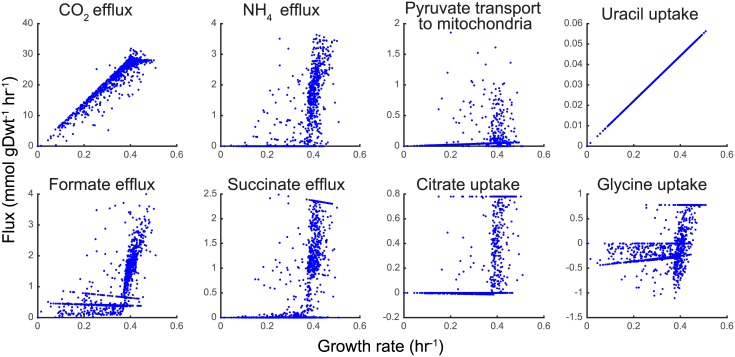
Variability in metabolite secretion and uptake among yeast cells. The plots show a representative selection of reaction fluxes, depicting different types of behavior. All cells depicted here were simulated using the Yeast 7.6 model in SD medium conditions. Glycine efflux plot has negative values which indicate net uptake of glycine.

Being a Crabtree-positive strain, our modeled *S. cerevisiae* generally fermented, even when oxygen was available. The overwhelming flow of carbon was through glycolysis and then out of the cells in the form of ethanol. Nevertheless, essentially all cells in our modeled populations engaged in some respirative behavior, although it tended to be at relatively low levels (see Fig 5A). Surprisingly, the degree of respiration was actually the main source of the cell-to-cell flux variability we observed. The first PCA component in both modeled growth mediums accounted for approximately 70–92% of the total metabolic variance (Table A5). The largest loadings in this PCA component were associated with oxidative phosphorylation (specifically ATP synthase) and respiration more generally (including an enhanced loading in oxygen uptake, see Fig 5B and Fig A4B in S1 Text). We found that the population diverged between slow-growing cells engaged in a basal amount of oxidative phosphorylation, and faster-growing cells that increasingly used the electron transport chain at higher growth rates. This increase began at growth-rates around 0.38 hr^−1^, and coincided with cells reaching their maximum allowable glucose uptake rate. Being effectively glucose-limited, these fast-growing cells funneled relatively more of their available carbon to the biosynthesis of metabolic building blocks (*e.g*. amino acids, nucleotides, lipids) and relied more heavily on the efficiency of their mitochondrial oxidative phosphorylation machinery. This resulted is a slight drop-off in ethanol and CO_2_ production (decreased fermentation) and marked increase in oxygen uptake, flux through ATP synthase (increased respiration; see Figs 5A and 5B and 6). It is worth noting that a direct analogue of this effect for the fermentation product acetate was described previously in *E. coli* [34].

#### SD media: Substrate level NAD^+^ Reduction, ADP phosphorylation, and organic acid efflux

**Fig 7:**
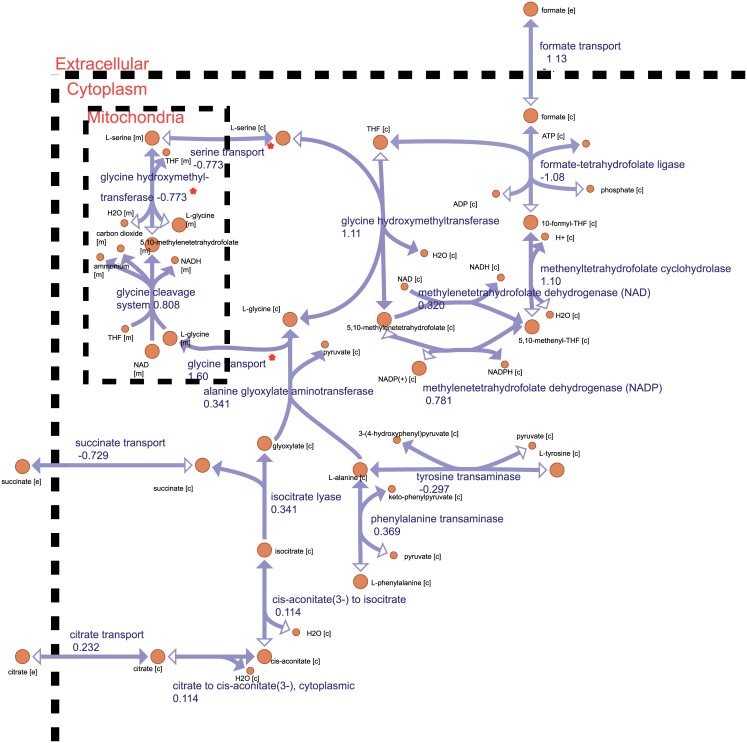
Map showing metabolic pathways for cycling of serine and glycine in cytoplasm and mitochondria. The Serine and Glycine cycle generates NADH, ATP, Succinate and Formate. Average fluxes over the 1,000 cells are shown besides each reaction. Reactions marked with red star were irreversible in previous versions of yeast metabolic model [13]. Letters in parenthesis after metabolite names indicate their location in the cell (c-cytoplasm, m- mitochondria, e-extracellular).

The second and third PCA component in SD media (accounting for ∼ 14% and 4% of the total flux variance respectively) was associated with increased mitochondrial NADH production and subsequent oxidative phosphorylation. Interestingly, this behavior was also associated with increased succinate and formate production—metabolic byproducts usually made during fermentation [67] (see Fig 6). The main contributor to the increased NADH production was found to be the so-called glycine cleavage system, which forms serine and NADH from two glycine molecules. Some of the resultant serine was found to then be transported out of the mitochondria where it was utilized to drive a cyclical series of reactions that 1) used tetrahydrofolate (THF) to recover glycine via the glycine hydroxymethyltransferase reaction (for subsequent transport back to the mitochondria), 2) reduced NAD^+^ (or NADP^+^) (via methylenetetrahydrofolate dehydrogenase), eventually forming 10formylTHF, and 3) phosphorylated ADP producing formate and the THF necessary to complete the cycle. The net effect of these coupled mitochondrial and cytoplasmic pathways, as summarized in Fig 7, was the consumption of glycine, the reduction of two NAD^+^ molecules, the production of ATP, and the formation and subsequent excretion of formate and some ammonium (see Fig 6). While there seems to be no direct experimental evidence of the glycine cleavage system and methylenetetrahydrofolate reductase (NAD or NADP dependent) being used predominantly for generating energy when amino acids are abundantly available in the growth medium, there is, however, indirect evidence [68] to suggest this might be true. In agreement with our predictions from Population FBA, experiments have shown glycine hydroxymethyltransferase (serine hydroxymethyltransferase in [68]) found in mitochondria and cytoplasm carry flux in opposite directions (Fig 7). Additionally the results of genetic algorithm (GA) simulations (see Section *Degeneracy of constraint selection* reveal that all replicates have serine glycine cycle usage among fast growing cells consistent with Fig 6. These are predictions which need further experimental validation. There is also evidence of cancer cells using this cycle to generate ATP, NADH and NADPH to support their fast growth [69–71]. It is worth noting that the serine and glycine transport reactions and the serine hydroxymethyl transferase reaction were only recently made reversible in the yeast 7.5 model [13] based on ^13^C labeling biochemical study [68, 72]. Therefore prior versions of the yeast consensus reconstruction would not have allowed for this form of energy metabolism (Fig A16 in S1 Text). Flux variability analysis was performed with 100% and 90% growth optimality for fixed total flux from pFBA to confirm that such a flux distribution is robust (Fig A17 and A18 respectively in S1 Text). In both cases the minimum and maximum fluxes through reactions in the serine glycine cycle show little variation except in the reactions involving methylenetetrahydrofolate dehydrogenase which are alternate pathways for NADH and NADPH production. At lower growth optimality the flux through the cycle is reduced due to decreased energy requirement.

In addition to some taken up from the modeled SD media, a significant portion of the glycine necessary to drive this system was found to originate from environmental citrate (also available in the media, see Fig 6). This citrate formed isocitrate via the reversible isocitrate dehydrogenase reaction, and this isocitrate in turn formed (via the cytoplasmic isocitrate lyase reaction) glyoxylate and the succinate we observed being excreted. Reacting with cytoplasmic alanine, the glyoxylate was then used to form much of the glycine necessary to maintain the glycine cleavage system (see Fig 7).

#### SD media: Bimodality in amino acid utilization

**Fig 8:**
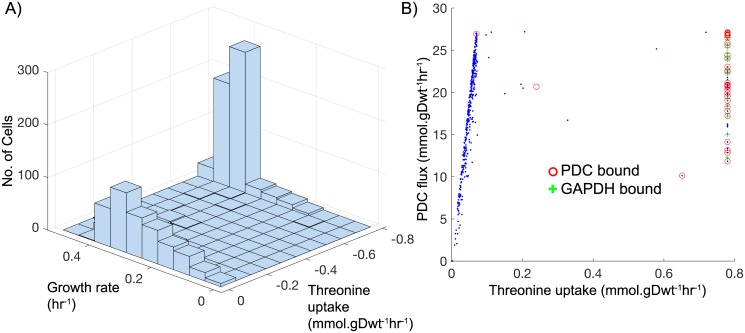
Bimodality in amino acid utilization. (A) 2D Histogram of growth rate and threonine uptake rate shows bimodality in threonine uptake among cells growing slower than 0.4 hr^−1^. (B) Scatter plot showing PDC flux and threonine uptake for slow growing cells (<0.35 hr^−1^). Whenever GAPDH or PDC flux is bound because of protein constraint (green + and red o respectively), cells take up threonine to fuel the glycine-serine cycle and generate NADH/NADPH and ATP which otherwise would have been generated from glycolysis.

A distinctive bimodality in the uptake of several amino acids emerged among the cells growing in SD media. This was especially prominent in the utilization of threonine by the slower-growing cells (those with growth rates less than 0.4 hr^−1^, see Fig 8). These cells were found to take up threonine at either its maximum allowable rate of 0.78 mmol gDwt^−1^ hr^−1^, or at a basal rate of about 0.04 mmol gDwt^−1^ hr^−1^, but very rarely (1% or less of slow growing cells) would threonine be taken up at rates in between. We found that whether or not threonine was being heavily utilized correlated strongly with whether or not the protein-associated glyceraldehyde-3-phosphate dehydrogenase (GAPDH) or pyruvate decarboxylase (PDC) reaction bound was constraining glycolytic flux. When it was not constraining, the cells engaged in as much glycolysis as was needed to serve their energy requirements (NAD^+^ reduction and ADP phosphorylation) and threonine was utilized at its basal rate in order to cover protein synthesis. Conversely, when the GAPDH or PDC reaction bound was constraining glycolysis threonine uptake was high. We found that much of this threonine was used by the cells to form glycine (via the threonine aldolase reaction) which in turn was used to fuel the glycine cleavage system (see Results Section *SD media: Substrate level NAD*^+^ *Reduction, ADP phosphorylation, and organic acid efflux*). This enabled the cells to make up some of the capacity for NAD^+^ reduction and ADP phosphorylation that was lost due to their limited glycolytic flux. Because of the relatively small amount of threonine uptake allowed, its use was essentially always at its maximum when the cells engaged in this behavior. Flux variability analysis was performed on over 400 cells growing slower than than 0.4 hr^−1^. It confirmed that predicted threonine uptake is robust and not a randomly chosen flux distribution among many possible degenerate solutions (Fig A19 in S1 Text). Replicates from GA as shown in Fig A10 in S1 Text also show bistability in threonine uptake. While in general amino acids can be used for generating energy [73–75], we do not have any experimental evidence for the predicted threonine bimodality except for FVA and GA analysis. Similar behaviors were observed in the utilization of aspartate, asparagine, glutamate, and glutamine (see Fig A8 in S1 Text); all of which can be converted to glycine through relatively few reaction steps and with no NADH or ATP investment.

### Degeneracy of constraint selection

The main doubling algorithm (see Methods Section *Constraint relaxation for realistic growth*) we chose for raising overly-constraining *k*_*cat*_ values focused on identifying constraints that most strongly limited cellular growth rate. This has the benefit of keeping as many of the experimental parameters available intact, but it also raises some questions, the most important of which is whether the set of turnover rates that are doubled is the only set that would lead to a mean growth rate similar to the measured one. Similarly, are all of the turnover rates that are kept intact after the doubling procedure actually necessary? In order to address these questions, we implemented an Evolutionary Algorithm based approach for finding constraint sets that yield growth rate distributions that approximately recover the one seen experimentally. This method made use of the Micro Genetic Algorithm formalism, a type of genetic algorithm (GA) that uses relatively small numbers of “genomes” and dispenses entirely with “mutations” [53] (see Methods Section *Genetic algorithm for constraint selection*).

**Fig 9:**
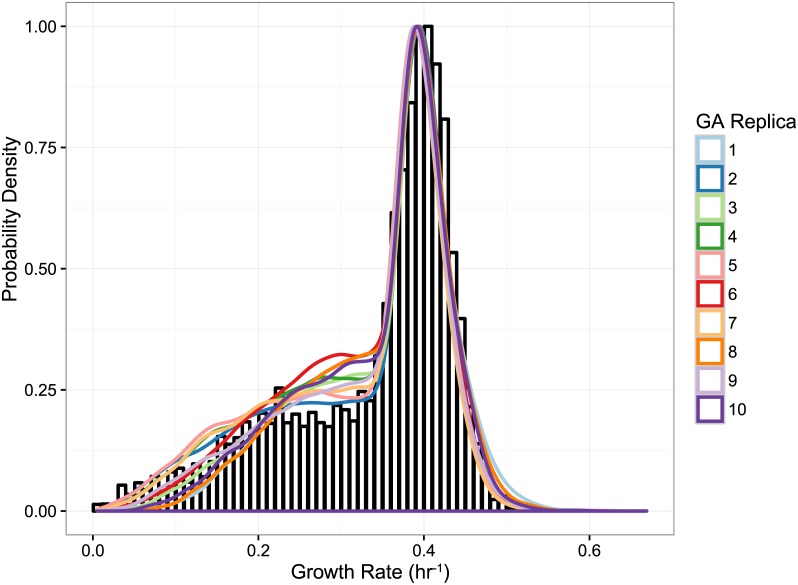
Growth rate distributions after GA optimizations. The plots show a comparison between the observed growth rate distribution [38] (black bars) and the distributions obtained after 10 GA optimizations (colored lines) using the Yeast 7.6 model in SD media conditions.

**Fig 10:**
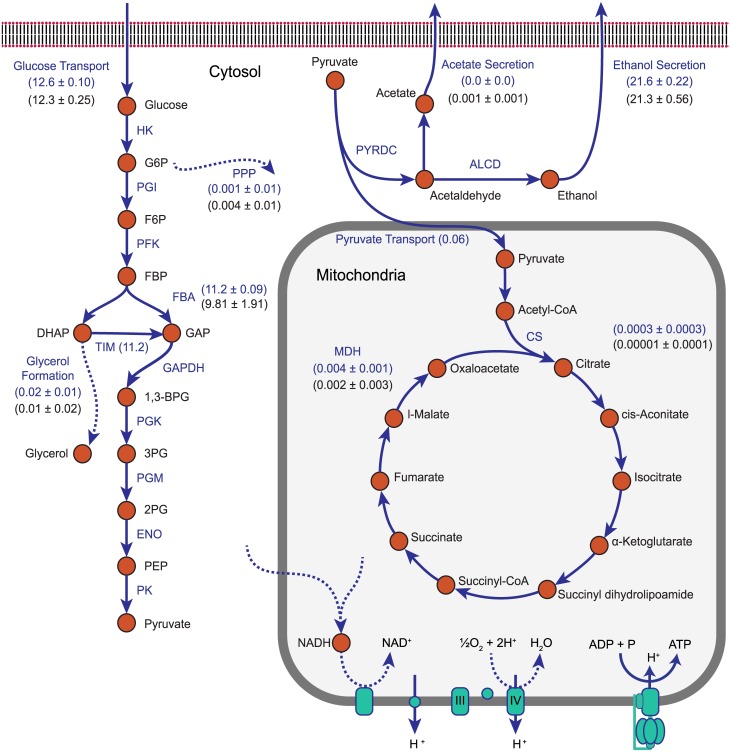
Analysis of GA replicates for serine glycine cycle usage. Upon sampling ten thousand cells from each of the 10 GA replicates shown in Fig 9, the fluxes through reactions in the serine glycine cycle occur only in fast growing members of the population. Legend containing color code for the GA replicas can be found in the top left panel.

**Fig 11:**
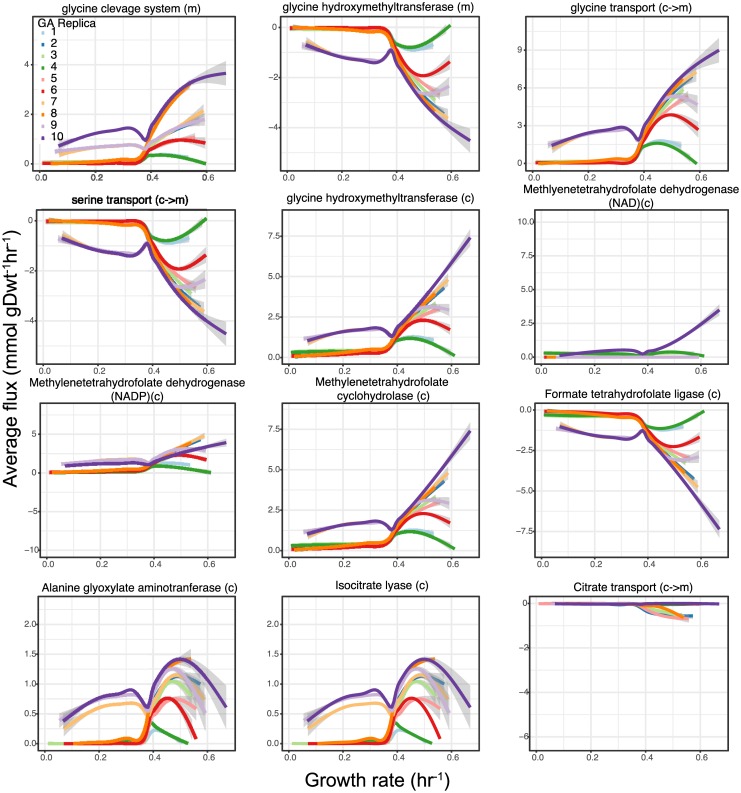
Comparison of metabolic fluxes from doubling procedure and independent GA optimizations. The figure shows a comparison between mean simulated flux through central metabolism after doubling procedure (blue), and the mean flux obtained across 10 GA optimizations (black). All simulations were done using Yeast 7.6 model in SD media conditions.

We performed 10 independent GA runs using the modeled SD media, and each resulted in a different set of turnover rates being lifted. In each case the resulting growth rate distribution closely matched the experimental one (see Fig 9). In general roughly twice as many turnover rates were lifted in each GA set as were affected by our main doubling method. This was because the GA associated no cost with filtering a value that did not impact the growth of the modeled cells. If, for example, a *k*_*cat*_ constrained a reaction involved in metabolizing a sugar like galactose that was not available in the media, the main doubling method would never lift it because it would not constrain the growth rate, but the GA, being fundamentally a random search method, might lift it simply by chance. Across all GA runs, only 51 turnover rates were consistently raised (see Table A6 in S1 Text and S1 File). Out of these 51, 49 were also affected by doublings during our main doubling method, meaning that they represent a core set of problematic *k*_*cat*_ values whose removal was necessary to achieve realistic growth. This core set was not by itself sufficient, however. In every GA run, between 150 and 181 additional turnover rates were also lifted. These extra *k*_*cat*_ values showed little overlap among the 10 sets, which indicates that beyond the core 51, the choice of which turnover rates to lift became highly degenerate. Despite this degeneracy, every set of turnover rate parameters found by the GA showed the same Crabtree effect and shift between fermentation and respiration yielded by our main doubling methodology (see Results Sections *Population FBA yields intracellular fluxes that agree with* ^13^*C fluxomics data, Fermentation vs. respiration* and Fig 10). As shown in Fig 11, all 10 populations show usage of the serine-glycine cycle by fast growing cells similar to that shown in Figs 5 and 6 in the population obtained by doubling methodology *SD media: Substrate level NAD*^+^ *Reduction, ADP phosphorylation, and organic acid efflux*.

Interestingly, the GAPDH-associated turnover rate that drove the bimodal amino acid utilization noted previously (see Results Section *SD media: Bimodality in amino acid utilization*) was lifted in some but not all of our GA runs. By comparing threonine usage across GA runs we found that when the GAPDH-associated *k*_*cat*_ was raised the bimodality among slow-growing cells essentially disappeared; instead, at growth rates lower than about 0.3 hr^−1^, the modeled cells all took up threonine at its basal rate and none were found to utilize the glycine cleavage system (see Fig A10 in S1 Text). This finding further supports the notion that it is the GAPDH constraint that gives rise to bimodal amino acid utilization we observed. Among faster-growing cells, the glycine cleavage system, and the related uptake and catabolization of amino acids occurred regardless of which constraints were raised by the GAs (Fig A10 in S1 Text). This is because glycolysis in the fast-growing cells is not constrained by enzyme copy-numbers, it is constrained by the glucose uptake rate itself; almost every cell growing faster that approximately 0.3 hr^−1^ experiences this limitation, and they engage in amino acid catabolism as a response.

### Conclusion

With the development of single cell and micro-colony imaging experiments [36–38, 42], instead of measuring a single growth rate (via optical density, for example) for an entire population, we can now observe a distribution of the growth rates of individual cells. To understand or interpret the general form of the growth rate distribution, we have to dig into the metabolic behavior of the underlying subpopulations. Recent systematic genome-wide fluorescence labeling studies have provided libraries of approximately 1,000 “strains” of labeled E. coli and 4,000 “strains” of labeled yeast. Examination of these strains has shown that proteins are not expressed at a specific number across a population. Due to the well-established innate stochasticity in essentially every cellular processes (transcription, translation, DNA replication, cell division, *etc*.), these studies have shown that proteins are expressed in varying numbers from cell to cell. In order to understand how any given cell’s protein expression state effects its behavior, and how that behavior relates to the overall behavior at the population level, these protein distributions must be sampled and realistic subpopulations of individual cells must be modeled. Our Population FBA approach provides such a method; allowing us to carry out the generation of realistic populations of cells and subsequent analysis of their intracellular fluxes and exchanges with the environment.

Simulations of the steady-state growth rates attainable by the cells in our modeled populations gives rise to a distribution that is in excellent agreement with the experimentally observed growth rate distribution [38]. In particular both show the same broad shoulder of slow growing cells (ranging in growth rates from nearly 0.0 to approximately 0.3 hr^−1^), and a dominant peak of fast-growing cells (ranging between approximately 0.3 and 0.7 hr^−1^, see Fig 2). We show that substrate availability is the main cause of this peak, and we believe this can be verified experimentally; in particular we suggest micro-colony experiments similar to that of [42] under growth conditions with varying levels of substrate availability or with substrate transporters placed under the control of inducible regulatory elements (see Results Section *S. cerevisiae Exhibit a Broad Distribution of Growth Rates*). Moreover, we find excellent agreement between experimental fluxomics data [40] and the computed intracellular fluxes predicted by our methodology, both within and between the cytosol and the mitochondria. These results underscore both the rigor of the Population FBA methodology as well as the high quality of the Yeast 7.6 metabolic model [14, 41].

The simulations presented here also allowed us to make quantitative predictions about the effects of growth in media where the main difference was the presence or absence of amino acids. The ^13^C experiments on wild-type yeast contained no amino acids in the media; our simulations showed that cells under these conditions depended on ammonium, sulfate, and phosphate salts taken up from the media. The gene knockouts that differentiated the the strains used in the ^13^C and SD experiments required the addition of uracil, leucine and histidine to the SD media. The SD media also contained an additional 17 amino acids, several of which were taken up and catabolized as a energy source. Despite the differences, both simulated populations displayed very similar growth rate distributions.

We have employed our Population FBA methodology to study metabolic heterogeneity in *S. cerevisiae*. One of the most important result of this study is that it underscores the need for imposing biologically realistic internal constraints in flux balance models. Without the types of constraints Population FBA imposes, the yeast 7.6 model gave fluxes, growth rates, and metabolic byproducts that were qualitatively and quantitatively inconsistent with the results of a ^13^C fluxomics study. Our study has shown that yeast populations exhibit the same types of cell-to-cell diversity in behavior that is coming to be recognized across the microbial world, and that although the particular sets of constraints that are necessary to recover the experimental growth rate distribution are not unique, any set that does recover the growth rate distribution also recovers the main metabolic behaviors we observed, including the Crabtree effect and the noted shift toward respiration seen among our fast-growing cells.

## Supporting information

S1 Text Extended results and methods. Extended figures and tables for further analysis and validation of experimental data, as well as further analysis of our results. The text also covers a more detailed explanation of our methods.
(PDF)

S1 File Metabolic models and parameters for protein sampling and constraint calculation. Model files contain all modifications to reflect gene deletions and media conditions. Parameter files with all shape and scale parameters for fluorescence distributions of proteins used in our simulations, along with their original *k*_*cat*_ values, final *k*_*cat*_ values after doubling procedure in both SD and ^13^C media as well as scaling ratios to convert sampled SD protein count to ^13^C count. There is also listing of final *k*_*cat*_ values obtained after 10 independent GA optimizations in SD media. Correlation matrix imposed while sampling distributions as well as its Cholesky decomposed factor is also provided.
(ZIP)

S2 File Parameters for fluorescence abundance distributions. Full parameter set acquired after removing all unreliable fluorescence distributions for protein abundances observed in Dénervaud *et*. *al*. [42], including parameters for fluorescence distributions in individual time steps, full plots for fluorescence distributions of proteins used in our simulations and the R code developed to systematically analyze and filter “spikey” distributions. Also provided is list of shape and scale parameters for fluorescence distribution of all 3647 proteins considered reliable along with means and variances for protein count distributions.
(ZIP)

S3 File Metabolic Map and Model files for ESCHER. JSON file for the metabolic model yeast 7.6 (y76Model.json) and corresponding metabolic map (y76Map.json) to be used with ESCHER (www.escher.github.io, [76]). This map contains glycolysis, TCA cycle, Oxidative phosphorylation, Uracil Biosynthesis and Glycine-Serine cycle.
(ZIP)
