# Supplementary material for: Population FBA predicts metabolic phenotypes in yeast
Source: PLoS Comput Biol. 2017 Sep 8;13(9):e1005728. doi: 10.1371/journal.pcbi.1005728 (PMC5626512; doi:10.1371/journal.pcbi.1005728)
Supplement: S1 Text — Extended figures and tables for further analysis and validation of experimental data, as well as further analysis of our results. The text also covers a more detailed explanation of our methods. (PDF) [file pcbi.1005728.s001.pdf]

Population FBA Predicts Metabolic Phenotypes in Yeast

Piyush Labhsetwar[1,◑], Marcelo C. R. Melo[1,◑], John A. Cole[2,◑], Zaida Luthey-Schulten[1,2,3,*],

1 Center for Biophysics and Quantitative Biology, University of Illinois at Urbana-Champaign, Urbana, Illinois, USA
2 Department of Physics, University of Illinois at Urbana-Champaign, Urbana, Illinois, USA
3 Department of Chemistry, University of Illinois at Urbana-Champaign, Urbana, Illinois, USA

◑These authors contributed equally to this work.
*** zan@illinois.edu**

A1 Supplementary information

A1.1 Extended Methods: Metabolic Model and Experimental Data

The Yeast Metabolic Model 7.6 [1] was chosen for this study because it is the most complete and up-to-date model available, and because it was shown to have equal or better prediction power when compared to other yeast models [2] (for a comparison between biomass pseudo-reactions from recent models, see SI Table A8). Due to the presence of knockouts in the strain used in the SD medium experiments (namely the proteomics and growth rate distribution experiments on which this work relies [3, 4]), when simulated under these conditions the model was changed accordingly. The strain in question is BY4741 (or ATCC 201388) with the following genotype: MATa, *his3*Δ*1*, *leu2*Δ*0*, *met15*Δ*0*, *ura3*Δ*0*. In our model, the genes YCL018W, YLR303W, YEL021W were inactivated, leading to zero flux being allowed through five reactions: 3-isopropylmalate dehydrogenase (r_0061), cysteine synthase (r_0312), O-acetylhomoserine (thiol)-lyase (r_0812, and r_0813) and orotidine-5-phosphate decarboxylase (r_0821). The histidine biosynthesis knockout is recovered when GFP is tagged to any protein, so the gene YOR202W was kept active.

The SD medium used contained 20 g/L glucose. The medium composition was taken from Sigma-Aldrich, Ref. #Y0626 and #Y1751. Uptake rates were obtained from the literature, searching conditions that closely matched the 30°C temperature and histidine dropout SD medium of the experiments [5]. Special attention was given to the glucose uptake rate, since there is extensive regulation of this cellular process which leads to different transporters being expressed and, consequently, different uptake rates depending on extra cellular glucose concentration. We set the uptake bound for glucose at 15 mmol $gDwt^{-1}$ hr^{-1}. For all other components of the medium, the uptake rate was set based on experimental values when available, or it was set to the highest experimental amino acid uptake rate. Table A1 shows the maximum uptake rate for all components available in the medium.

The turnover rates (k_{cat}), for enzymes which have predicted copy numbers and that are found in the metabolic model yeast 7.6 were gathered from BRENDA database [6, 7] using the SOAP-Python web interface or from literature (See Table A3). The highest

k_{cat} was always selected, preferably from experiments expressing wild type *S. cerevisiae* proteins, but when one was not available, mutants and other species were allowed. Finally, for proteins which did not have a turnover rate in BRENDA, the highest available for a wild-type yeast enzyme, $38,000$, was chosen so as to avoid over-constraining the model.

A1.2 Noise Properties of Proteins in *S. cerevisiae*

Variability in protein copy number has been investigated previously in single cell fluorescence studies of yeast and *E. coli* [8, 9]. Both studies observed two distinct regimes of noise behavior based on the mean copy number. Noise in low protein copy number proteins dropped hyperbolically with mean copy number; this noise has been described as intrinsic noise arising from the inherent stochastic nature of gene expression. Higher copy number proteins exhibit an approximately constant level of noise; this plateau is labeled associated with extrinsic sources arising from variability in common factors involved in gene expression like RNA polymerase or ribosomes. We observe similar behavior in noise measured by Dénervaud *et. al.,* (Fig A1). Proteins with mean copy numbers below approximately 1,000 show a decrease in their noise with mean copy number while proteins expressed at levels higher than approximately 1,000 have fairly constant noise.

A1.3 Reliability of mRNA Microarray Correlation Data

We considered two ways of determining the reliability of the mRNA microarray-based correlation data we used. The first was to determine if the correlations exhibit discernible behavioral traits, such as anti-correlation between genes associated with fermentation and respiration. To see if this was reflected in the correlation data, we considered the glucose transporter HXT1. As seen in Fig A11, we found negative correlations between HXT1 and several genes associated with the TCA cycle and oxidative phosphorylation. We also see positive correlation between HXT1 and ethanol fermentation genes, as would be expected in a Crabtree-positive yeast strain. The second way we evaluated the reliability of our correlation data was by comparing it to experimentally established regulatory links in yeast [10]. To perform this comparison, we devised a distance metric (Equation A1):

$$Distance = \sum_{i,j} Regs_{i,j}(1 - |\rho_{i,j}|) + \delta(Regs_{i,j})|\rho_{i,j}| \tag{A1}$$

where $Regs_{i,j}$ represents the number of common transcription factors regulating genes i and j, and $\delta(x)$ represents the Kronecker delta function (1 if the argument is 0, and 0 otherwise)

This metric is based on the idea that if two genes share a common transcription factor, their expression should be correlated, either positively or negatively depending on the regulatory relationship between transcription factor and the genes. The first term penalizes gene pairs with large numbers of shared transcription factors but weak correlation, while the second penalizes gene pairs with large correlation but no shared transcription factors. If the correlations generally reflect the known regulatory links, the distance metric will be smaller. To see if this is true we compared the distance metric obtained for correlations from actual expression data [11] with the distance metric obtained for correlations from randomized expression data. As seen in Fig A12 top, the distance metric for the actual expression data is significantly smaller than the distribution of distance metrics (Fig A12 bottom) obtained by randomizing expression data.

A1.4 Extended Methodology: Genetic Algorithm for Constraint Selection

Main Methods: A new procedure for filtering overly-constraining turnover rates based on the Micro Genetic Algorithm (GA) formalism was developed [12]. This method utilizes an entire growth distribution as a target for optimizing the selection of experimental constraints.Micro Genetic Algorithm was chosen instead of a "regular" Genetic Algorithm solely for computational cost concerns. In a "regular" GA algorithm in dozens to hundreds of genomes would have to be simulated at each generation, and several hundred generations could need to be evaluated to reach the same results. The computational cost would be extremely higher as compare to our GA implementation. In our attempt to reduce the size of search space we have restricted GA variables to binary values representing weather to use a particular k_{cat} or 38,000 s^{-1} rather than more flexible values k_{cat} can take in the doubling procedure. Briefly, a population of 10 "genomes" was simulated, each one composed of a list of "genes" that indicated if a protein's k_{cat} would be kept at its original value, or if it would be raised to $38,000\ \mathrm{s}^{-1}$. The original k_{cat} values are either obtained from BRENDA or from literature (See Table A3 and SI File S1). The genomes were allowed to evolve by exchanging information, and each new generation was created by a random selection of solutions biased by their fitness, while always taking the best solution to the next generation (see SI Section *Extended Methodology: Genetic Algorithm for Constraint Selection for details*). The fitness of each genome was determined by simulating a cell population based in its k_{cat} selection, and then calculating the goodness-of-fit between the resulting growth rate distribution and the observed distribution [4].

Extended Methods: Each genome was composed of 368 "Boolean genes", one for each protein that had a k_{cat} available. The value of the binary gene indicated whether a original k_{cat} would be used with its respective protein count to calculate a v_{max}, or if the maximum k_{cat} of $38,000\ \mathrm{s}^{-1}$ would be used for the v_{max} calculation. Since the micro GA applies a uniform cross-over operator, where each gene in an offspring is randomly selected from one of two parent genomes, no mutation operator was used. Moreover, a tournament selection strategy was applied with a sample size of 4, while also applying elitism for the fittest genome. This guarantees high variability and a fast convergence, while preserving optimal results. Population convergence was determined by comparing each genome to the fittest genome in a generation, and counting the number of different gene states between them. When all genomes had less than 5 different gene values when compared with the fittest genome, we determined the population had converged, in which case the fittest genome was kept and all other 9 genomes were re-set to random states. The fitness was calculated by simulating a 4800 cell population from each genome, and then calculating the Watson variation of the Cramér-von-Misses goodness of fit test, comparing the simulated and the experimentally observed growth rate distribution [4] for the entire population. The fitness function was defined as the inverse of the test statistic. The genetic algorithm took anywhere from 2.5 to 15.5 hours to achieve a good goodness-of-fit between simulated and observed growth distribution using 320 CPUs.

A1.5 Proteins with Significant Mean Copy Number But Zero Flux Predicted

Metabolic models are mappings between the genotype of an organism and the reactions that can be catalyzed by their gene products. Given a growth medium and knowledge of the strain (specifically the existence of gene knock-outs, *etc.*), these models can predict which reactions can carry flux and which cannot. Flux variability analysis with

zero optimal growth requirement was used to determine the minimum and maximum possible flux through each reaction. So called "dead ends"—reactions that can never carry flux due to incompleteness of the model—were not considered. We observed several proteins (Table A7) that were measured in significant copy number despite the reactions they catalyze being unable to carry any flux. This inconsistency may be due to two reasons. The first is that the function of the protein might be out of scope of the metabolic model. Some proteins could be involved in both metabolic and non-metabolic functions within the cell; the expression of such a protein might be predominantly associated with its "moonlighting" function. Another possibility is that certain proteins are highly expressed due to transcriptional regulation which is not accounted for by the metabolic model. For example *URA1* is expressed at a copy number of around 18,000 even though there is a deletion in *URA3*, a gene downstream of *URA1* in the strain being used. Other proteins in uracil biosynthesis like *URA4* and *URA5* (Fig A13) are also expressed in high copy number. This might be due to the inducing activity of dihydroorotic acid [13] which is a metabolic intermediate in uracil biosynthesis and is known to up-regulate expression of *URA1* and *URA4*. The deletion of *URA3* could result the in accumulation of dihydroorotic acid as cells might not have adapted to the deletion of this key biosynthetic enzyme. Similarly in the case of leucine biosynthesis, the *LEU2* deletion might be causing buildup of α-Isopropylmalate, also a metabolic intermediate, which regulates expression of *LEU1* [14].

A1.6 Metabolic Map

Metabolic maps are extensively applied by the systems biology community as a tool to both explore and understand metabolic activity in a cell. They allow an easy way of visualizing the flux distribution throughout the simulated metabolic pathways. Despite their importance, they suffer from a problem which afflicts most efforts to combine large scale biological information: conflicting naming conventions. Maps built for previously developed metabolic models can rarely be reused because metabolites and reaction identifiers constantly change from version to version, and between organisms and data sources (such as fluxomics, transcriptomics and proteomics). The latest versions of the yeast metabolic models tried to unify nomenclature and create a consistent pattern for metabolites and reaction names, but a new map was not created to make use of such developments. In this work, a comprehensive map representation for Yeast 7.6 was built using Escher [15], and it is presented and made available to the community.

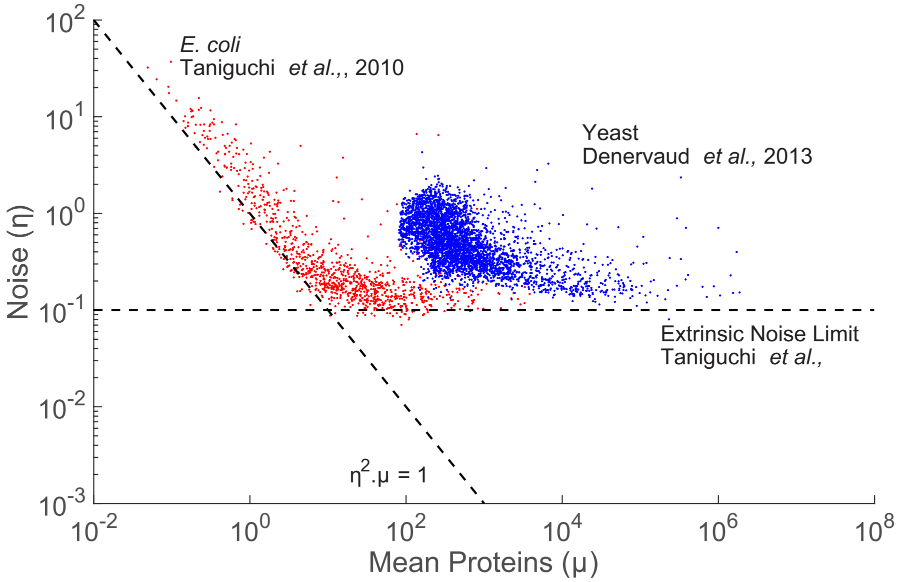

Fig. A1. Behavior of noise in protein copy number. Noise as function of protein mean copy number for *E. coli* data from Taniguchi *et. al.,* [9] and for *S. cerevisiae* from Denervaud *et. al.,* [3]

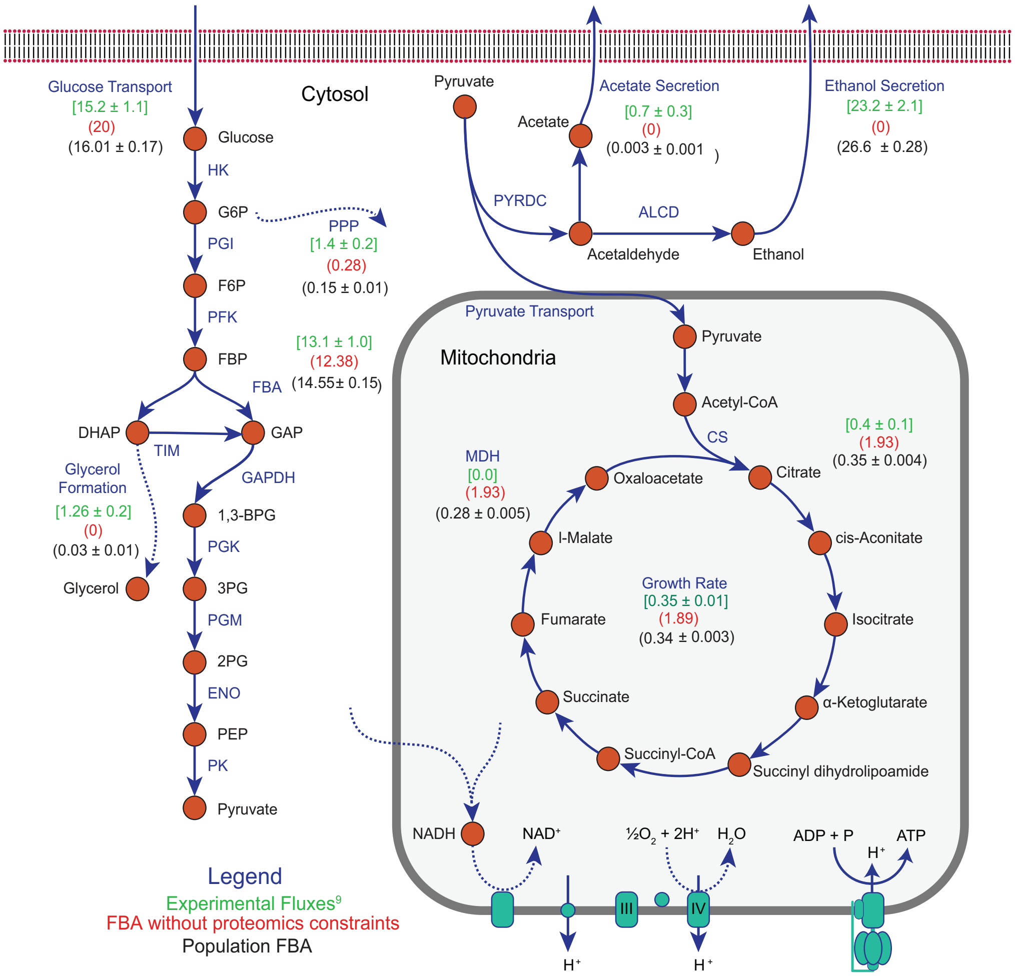

Fig. A2. Flux distribution using FBA without proteomics constraints Compared to the flux distribution in ^{13}C growth medium calculated without using protein constraints (red), the Flux distribution calculated using population FBA with protein constraints (black) agrees very well with experimentally determined fluxes (green)

Table A1. Growth medium composition for glucose minimal medium used for ^{13}C fluxomics experiments [16] and glucose synthetic defined medium (SD) for proteomics experiments [3]

	^{13}C Minimal Medium		SD Medium	
Chemical	Concentration mM	Uptake Bound mmol $gDwt^{-1}hr^{-1}$	Concentration mM	Uptake Bound mmol $gDwt^{-1}hr^{-1}$
<u>Carbon source</u>				
D-glucose	55.56	20	111.11	15
<u>Salts</u>				
Ammonium	37.84	1000	37.84	1000
Iron(3+)	0	1000	0.01	1000
Iron(2+)	0.01	1000	0	1000
Phosphate	22.04	1000	7.34	1000
Potassium	22.04	1000	7.34	1000
Sodium	0	1000	1.71	1000
Sulphate	39.87	1000	39.84	1000
Magensium	2.03	1000	2.03	1000
Calcium	0.031	1000	0.68	1000
Chloride	0.061	1000	3.07	1000
<u>Vitamins</u>				
(R)-pantothenate	0.0042	0.78	0.0016	0.78
4-aminobenzoate	0.0015	0.78	0.047	0.78
biotin	0.00020	0.78	0.0000082	0.78
Folic acid	0	0	0.0000045	0.78
myo-Inositol	0.14	0.78	0.0111	0.78
Nicotinate	0.0081	0.78	0.0033	0.78
Pyridoxine	0.0059	0.78	0.0019	0.78
Riboflavin	0	0	0.00053	0.78
Thiamine(1+)	0.0030	0.78	0.0012	0.78
<u>Other nutrients</u>				
Adenine	0	0	0.098	0.78
Citrate(3-)	0	0	1.67	0.78
Uracil	0	0	0.678	0.78
<u>Amino acids</u>				
Glycine	0	0	1.01	0.78
L-alanine	0	0	0.85	0.1
L-arginine	0	0	0.36	0.31
L-asparagine	0	0	0.51	0.36
L-aspartate	0	0	0.57	0.72
L-cysteine	0	0	0.43	0.78
L-glutamate	0	0	0.41	0.6
L-glutamine	0	0	0.52	0.23
L-isoleucine	0	0	0.58	0.78
L-leucine	0	0	2.90	0.78
L-lysine	0	0	0.42	0.78
L-methionine	0	0	0.51	0.78
L-phenylalanine	0	0	0.46	0.78
L-proline	0	0	0.66	0.78
L-serine	0	0	0.72	0.47
L-threonine	0	0	0.64	0.78
L-tryptophan	0	0	0.37	0.78
L-tyrosine	0	0	0.34	0.13
L-Valine	0	0	0.65	0.78

Table A2. Ratios of mRNA expressed by cells growing in ^{13}C and SD media. The ten most down- and up-regulated values are shown. Complete list of ratios is in SI File S2

Genes	Names	Ratios (^{13}C/SD)
	Downregulated in ^{13}C medium *vs.* SD medium	
YMR169C	Cytoplasmic aldehyde dehydrogenase	0.06
YLR038C	Subunit VIb of cytochrome c oxidase	0.08
YLR044C	Major of three pyruvate decarboxylase isozymes	0.10
YDR400W	Uridine nucleosidase (uridine-cytidine N-ribohydrolase)	0.10
YMR278W	Phosphoribomutase	0.12
YER178W	E1 alpha subunit of the pyruvate dehydrogenase (PDH) complex	0.13
YDL185W	Subunit A of the V1 peripheral membrane domain of V-ATPase	0.13
YGR260W	High affinity nicotinic acid plasma membrane permease	0.16
YDR380W	Phenylpyruvate decarboxylase	0.19
YOR128C	Phosphoribosylaminoimidazole carboxylase	0.30
	Upregulated in ^{13}C medium *vs.* SD medium	
YJR105W	Adenosine kinase	13.18
YLR231C	Kynureninase	9.99
YNL220W	Adenylosuccinate synthase	8.28
YNL241C	Glucose-6-phosphate dehydrogenase (G6PD)	7.26
YMR208W	Mevalonate kinase	6.36
YLR028C	Enzyme of de novo purine biosynthesis	5.74
YEL024W	Ubiquinol-cytochrome-c reductase	5.66
YMR062C	Mitochondrial ornithine acetyltransferase	5.62
YMR300C	Phosphoribosylpyrophosphate amidotransferase (PRPPAT)	5.31
YHR163W	6-phosphogluconolactonase	5.17

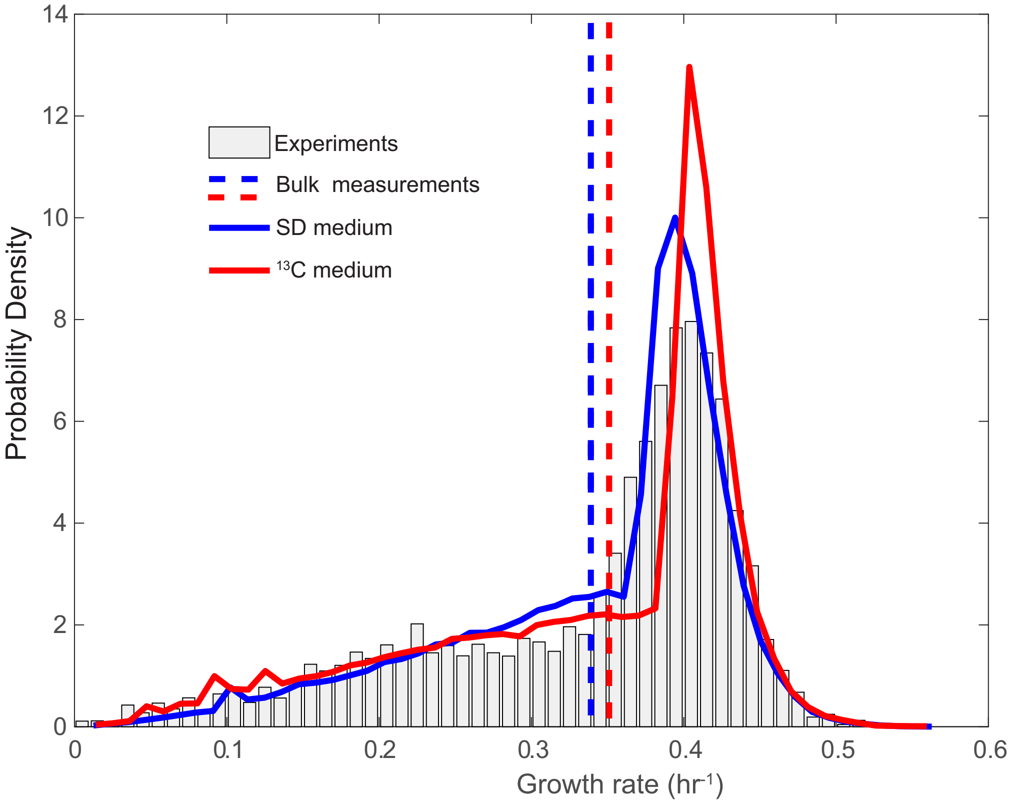

Fig. A3. Growth rate distribution predicted in ^{13}C growth medium. Growth rate distribution predicted in ^{13}C growth medium using Populations FBA with rescaled protein distributions

Table A3. List of k_{cat} values found manually from Literature. Corresponding doublings both for ^{13}C and SD medium are also listed. Final v_{max} was calculated for each protein using its mean copy, corresponding k_{cat}, doublings and unit conversion factor of 3.0×10^{-7} to convert units from s^{-1} to $mmol.gDwt^{-1}.hr^{-1}$.

ID	Name		k_{cat} (s^{-1})		Doublings				Final v_{max} ($mmol.gDwt^{-1}.hr^{-1}$)				
	Short	Full	BRENDA	Literature	^{13}C BRENDA	^{13}C Literature	SD BRENDA	SD Literature	^{13}C BRENDA	^{13}C Literature	SD BRENDA	SD Literature	Mean Protein (SD)
YLR258W	GSY2	Glycogen synthase	0.0183	694 [17]	19	3	19	2	4.91	2.84	4.91	1.42	1704.98
YOL140W	ARG8	Acetylornithine aminotransferase	1.55	2.45 [18]	14	13	0	10	2.11	1.67	0.00	0.21	276.87
YGL055W	OLE1	Delta(9) fatty acid desaturase	0.5	0.705 [19]	11	11	10	10	0.10	0.14	0.05	0.07	321.49
YGL148W	ARO2	Bifunctional chorismate synthase and flavin reductase	0.87	33 [20]	11	6	0	0	0.65	0.77	0.00	0.01	1209.06
YOR184W	SER1	3-phosphoserine aminotransferase	1.75	2.49 [21]	9	9	7	5	1.49	2.12	0.37	0.13	5538.01
YGR094W	VAS1	Mitochondrial and cytoplasmic valyl-tRNA synthetase	0.2	3.2 [22]	9	5	10	6	0.21	0.21	0.43	0.43	6976.33
YGL253W	HXK2	Hexokinase isoenzyme 2	1.67	200 [23]	9	2	8	2	27.03	25.3	13.5	25.3	105287.26
YNL104C	LEU4	Alpha-isopropylmalate synthase (2-isopropylmalate synthase)	2.717	13.79 [24]	9	6	0	0	1.21	0.76	0.00	0.01	2897.71
YDR354W	TRP4	Anthranilate phosphoribosyl transferase	2.9	69 [25]	9	4	0	0	0.20	0.15	0.00	0.01	446.59
YGL062W	PYC1	Pyruvate carboxylase isoform	60	89.79 [26]	7	7	0	0	4.97	7.43	0.04	0.06	2153.01
YCL040W	GLK1	Glucokinase	166	1492 [27]	7	0	7	0	19.44	1.37	19.42	1.37	3046.59
YML120C	NDI1	NADH:ubiquinone oxidoreductase	0.031	550 [28]	5	3	20	5	0.00	1.58	11.64	6.31	1193.41
YFL018C	LPD1	Dihydrolipoamide dehydrogenase	649	899 [29]	5	5	5	5	24.64	34.14	24.61	34.14	3950.66
YIL078W	THS1	Threonyl-tRNA synthetase	0.05	3.32 [30]	5	6	11	5	0.00	0.44	0.21	0.22	6862.9
YNL277W	MET2	L-homoserine-O-acetyltransferase	0.98	122 [31]	5	5	0	0	0.00	0.44	0.00	0.22	448.34
YGL245W	GUS1	Glutamyl-tRNA synthetase (GluRS)	0.016	4.4 [32]	4	5	12	4	0.00	0.53	0.25	0.27	12617.66
YGR061C	ADE6	Formylglycinamidine-ribonucleotide (FGAM)-synthetase	0.05	5 [33]	4	4	0	0	0.00	0.24	0.00	0.01	9802.89
YNL037C	IDH1	Subunit of mitochondrial NAD(+)-dependent isocitrate dehydrogenase	30	124 [34]	3	2	9	8	0.19	0.38	11.87	24.55	2574.91
YOR136W	IDH2	Subunit of mitochondrial NAD(+)-dependent isocitrate dehydrogenase	30	124 [34]	3	2	9	8	0.12	0.24	7.56	15.65	1641.49
YLL041C	SDH2	Iron-sulfur protein subunit of succinate dehydrogenase	60	658 [35]	2	3	6	2	0.04	0.85	0.62	0.42	536.45
YNR001C	CIT1	Citrate synthase	167	2100 [36]	2	0	0	0	0.53	1.65	0.13	1.65	2623.29
YLR044C	PDC1	Major of three pyruvate decarboxylase isozymes	–	12.42 [37]	NA	9	NA	4	NA	1603.33	NA	50.10	839441.26
YFR053C	HXK1	Hexokinase isoenzyme 1	1.67	200 [23]	0	0	0	0	0.04	4.75	0.04	4.75	79143.12
YLR450W	HMG2	HMG-CoA reductase	0.023	38000 *	0	0	16	0	0.00	6.60	0.26	6.60	578.88
YOR108W	LEU9	Alpha-isopropylmalate synthase II (2-isopropylmalate synthase)	2.717	7.28 [24]	0	0	0	0	0.00	0.00	0.00	0.00	1482.8
YDR226W	ADK1	Adenylate kinase, required for purine metabolism	0.0063	879.5 [38]	0	0	17	0	0.00	5.46	5.12	5.46	20657
YGR087C	PDC6	Minor isoform of pyruvate decarboxylase	–	9.21 [37]	NA	0	NA	0	NA	0.00	NA	0.00	940.76
YLR134W	PDC5	Minor isoform of pyruvate decarboxylase	–	10.32 [37]	NA	0	NA	0	NA	0.00	NA	0.00	1510.85
YPR081C	GRS2	Glycine-tRNA synthetase	0.73	0.15 [39]	0	0	0	0	0.00	0.00	0.00	0.00	471.03
YKR080W	MTD1	5,10-methylenetetrahydrafolate dehydrogenase (NAD)	1.63	1643 [40]	0	0	12	2	1.63	1.63	6676	6572	2707

'–' means not found in BRENDA, '*' means wrongly listed in BRENDA hence set to highest value known for yeast.

Table A4. Mapping Between Reaction Code, Abbreviation and Full Name.

Code	Abbreviation	Full Name
		Glycolysis
r_0534	HK	hexokinase (D-glucose:ATP)
r_0467	PGI	glucose-6-phosphate isomerase
r_0886	PFK	phosphofructokinase
r_0450	FBA	fructose-bisphosphate aldolase
r_1054	TIM	triose-phosphate isomerase
r_0486	GAPDH	glyceraldehyde-3-phosphate dehydrogenase
r_0892	PGK	phosphoglycerate kinase
r_0893	PGM	phosphoglycerate mutase
r_0366	ENO	enolase
r_0962	PK	pyruvate kinase
		TCA Cycle
r_2034	PYRt	pyruvate transport
r_0961	PDH	pyruvate dehydrogenase
r_0300	CS	citrate synthase
r_0302	ACONTa	citrate to cis-aconitate
r_0280	ACONTb	cis-aconitate to isocitrate
r_0658	ICDH	isocitrate dehydrogenase (NAD+)
r_0832	AKGDa	oxoglutarate dehydrogenase (lipoamide)
r_0831	AKGDb	oxoglutarate dehydrogenase (dihydrolipoamide S-succinyltransferase)
r_1022	SUCOAS	succinate-CoA ligase (ADP-forming)
r_1021	SUCD	succinate dehydrogenase (ubiquinone-6)
r_0451	FUM	fumarase
r_0713	MDH	malate dehydrogenase
		Electron Transport Chain
r_0770	NADH2c	NADH dehydrogenase cytosolic/mitochondrial
r_0773	NADHD	NADH:ubiquinone oxidoreductase mitochondrial
r_0439	CYOR	ferrocytochrome-c:oxygen oxidoreductase
r_0438	CYOO	ferrocytochrome-c:oxygen oxidoreductase (O2)
r_0226	ATPS	ATP synthase
		Ethanol Production
r_0959	PYRDC	pyruvate decarboxylase
r_2115	ALCD	alcohol dehydrogenase (acetaldehyde to ethanol)

All reaction codes reflect the yeast model 7 naming convention.

Table A5. Percentage variance accounted by first three PCA components accounting for maximum variance in the data for population simulated in ^{13}C fluxomics experiment and proteomics experiment

SD		^{13}C	
Pathway	% Var	Pathway	% Var
Fermentation *vs.* Respiration	71.07	Fermentation *vs.* Respiration	92.10
Glycine-Serine Cycle	14.40	Glucose uptake *vs.* ATP synthase	2.98
Mitochondrial Ethanol production	4.67	Glycerol production *vs.* pyruvate efflux	1.37

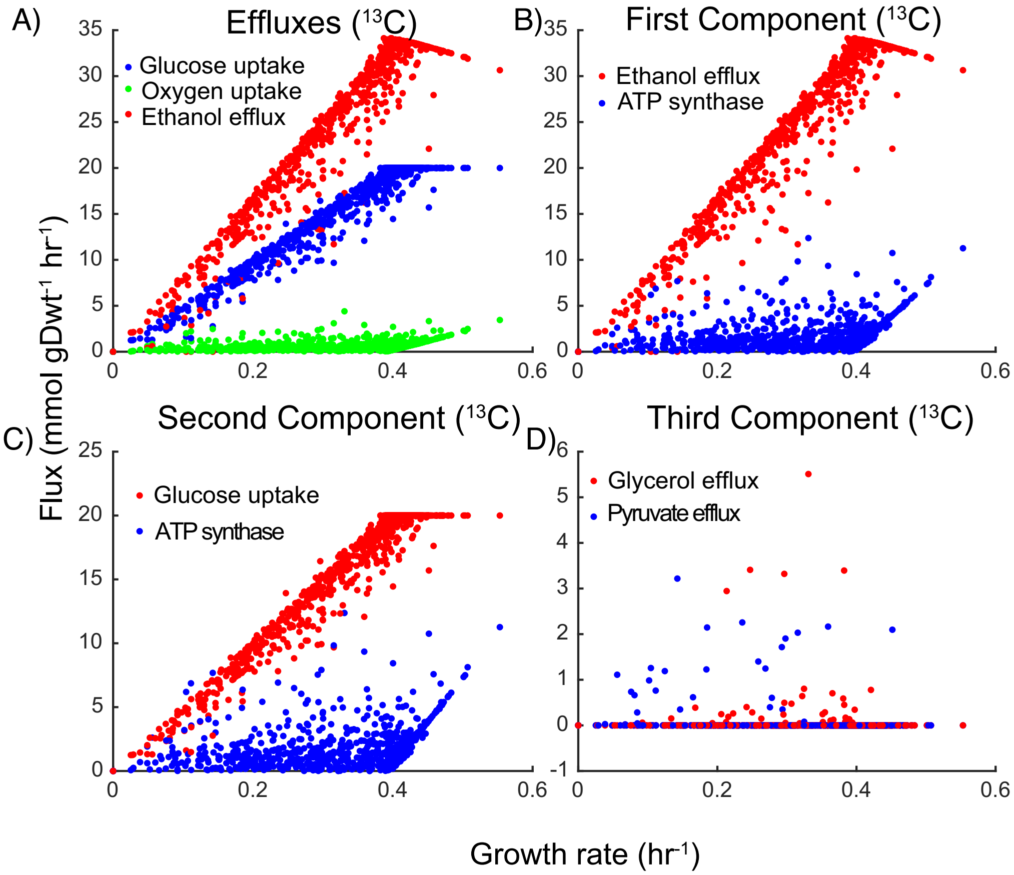

Fig. A4. Analysis of metabolic fluxes from yeast 7.6 simulations in ^{13}C medium. (A) Glucose uptake, oxygen uptake and ethanol efflux in glucose minimal medium. (B) First PCA component showing fast growing cells performing respiration along with fermentation. (C) Second PCA component showing rise in ATP synthase usage as glucose uptake gets limited in fast growing cells (D) Third PCA component showing few slower growing cells secreting pyruvate while few cells with intermediate growth rate secreting glycerol.

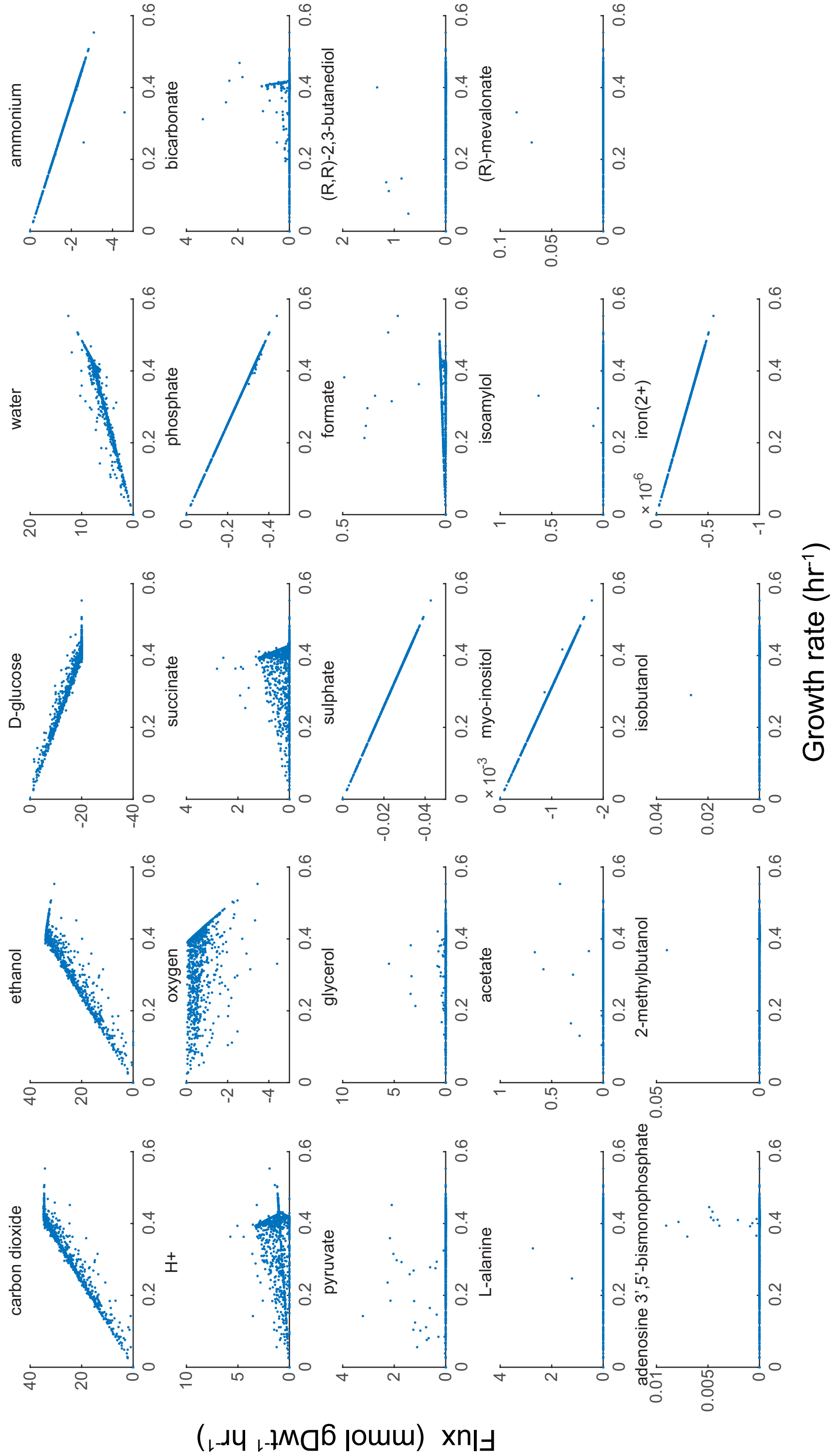

Fig. A5. All non-zero transport reactions in(negative) and out(positive) of the cell for population growing on glucose minimal medium used for ^{13}C fluxomics experiments

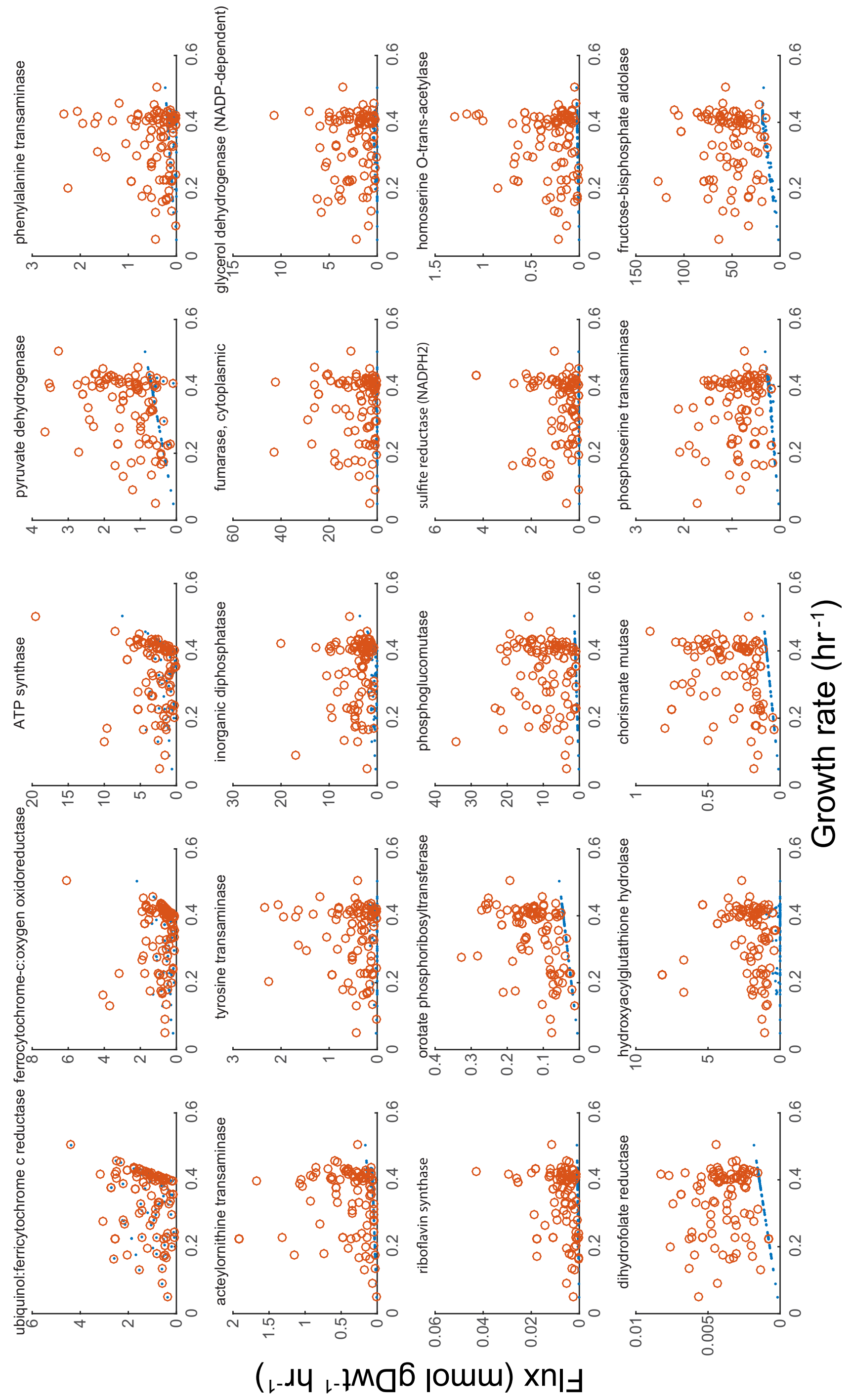

Fig. A6. Reactions constrained most often in ^{13}C growth medium. Blue dots are actual fluxes and Orange circles are upper bounds on the those reactions

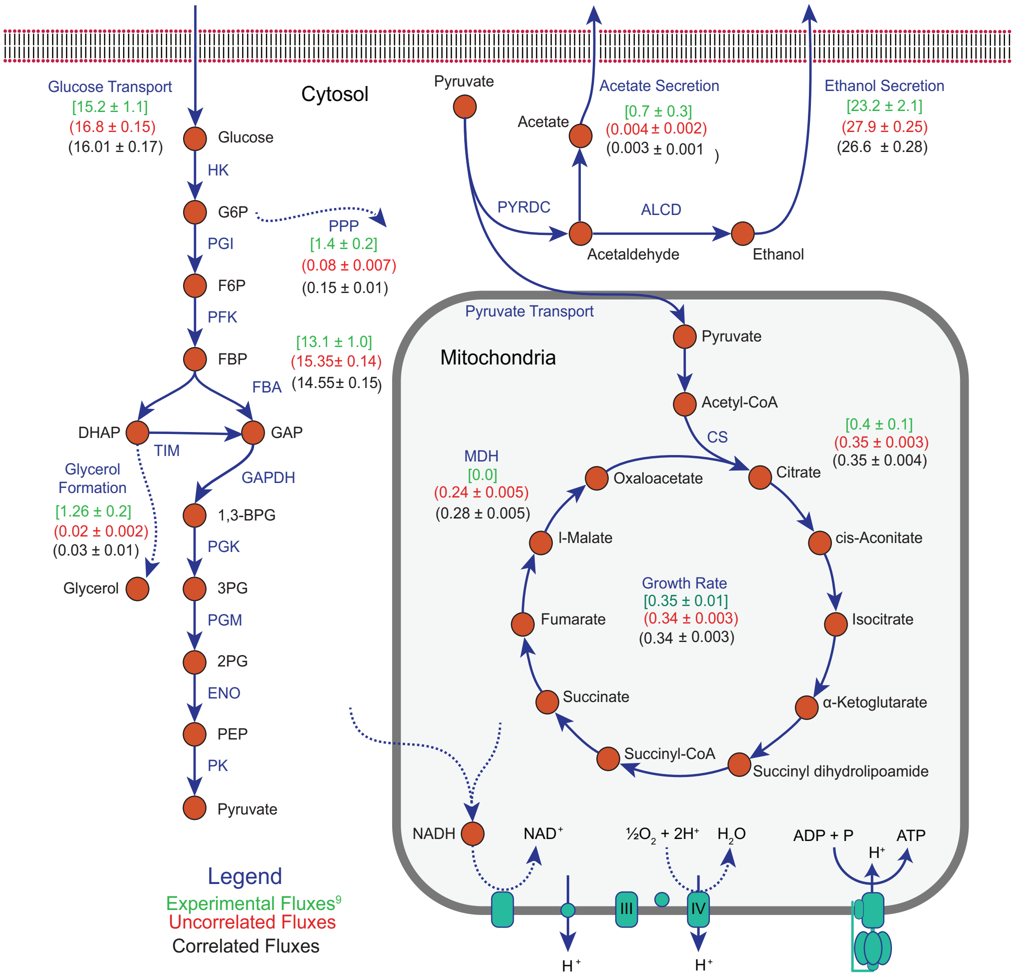

Fig. A7. Impact of correlated sampling of protein counts on simulated metabolic fluxes. Mean flux values through central metabolism for simulations that either imposed (blue) or did not impose (black) correlations in protein sampling. Values between brackets were taken from experimental measurements [16]. All simulations used yeast 7.6 and ^{13}C media conditions.

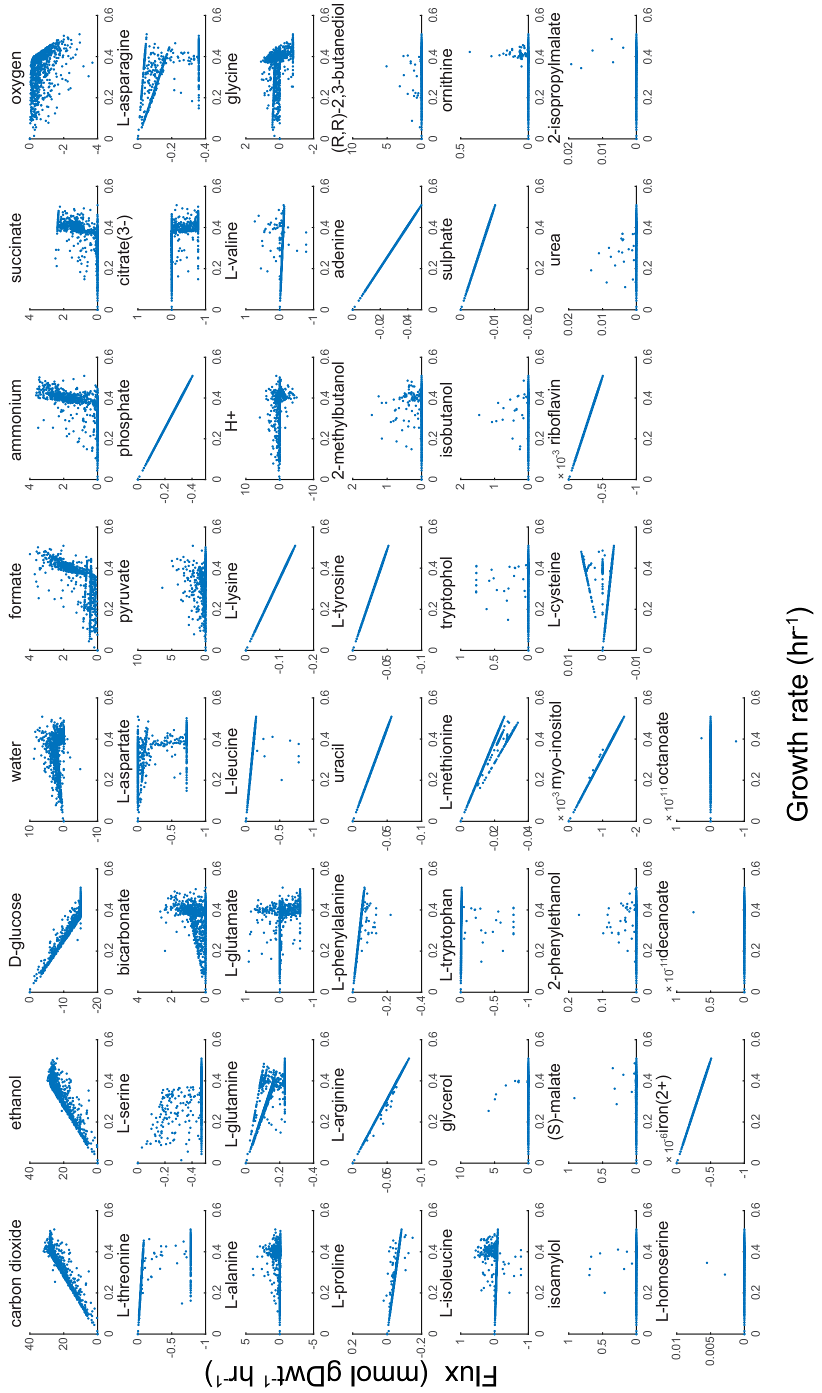

Fig. A8. Active transport reactions for yeast 7.6 model in SD medium. Negative and positive fluxes indicate secretion and uptake respectively of metabolites from the cell.

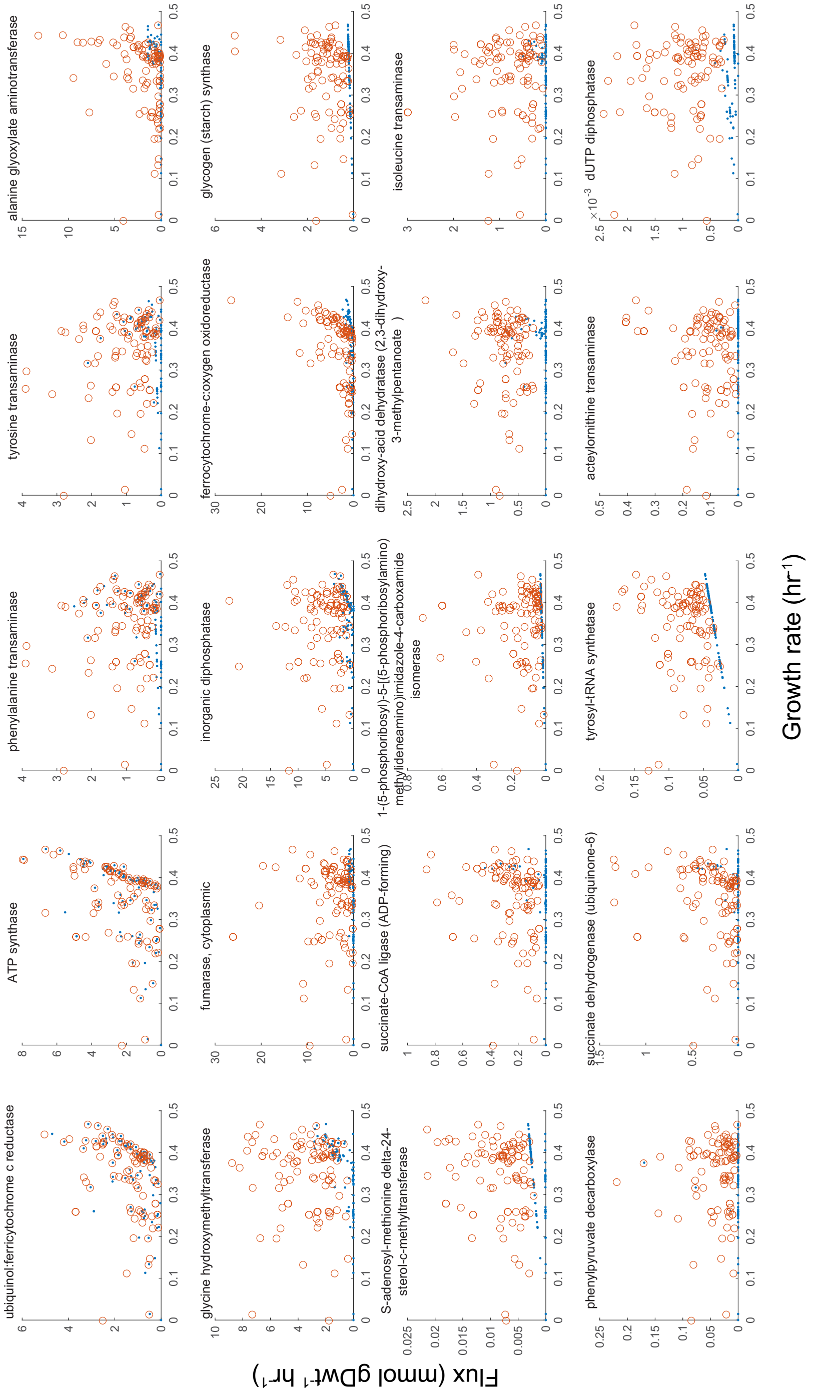

Fig. A9. Reactions constrained most often in SD growth medium. Blue dots are actual fluxes and Orange circles are upper bounds on the those reactions

Table A6. 51 genes consistently filtered by all 10 GA optimization runs in SD medium

Gene	Starting Kcat	Doublings	Mean Copy
YAL038W	232	5	52704.83
YBR011C	260	5	3465.71
YBR265W	1.07	8	264.36
YDL067C	1500	4	141.60
YEL024W	1500	4	162.02
YGL055W	0.705	10	171.07
YGL245W	4.4	4	12375.27
YGR094W	3.2	6	6209.55
YGR175C	0.076	7	3071.99
YGR185C	6.08	3	5268.56
YGR240C	62	5	76573.72
YHR042W	6.47	3	593.74
YHR190W	3.3	8	123.54
YIL020C	32	6	226.16
YIL078W	3.32	5	6074.67
YIL116W	4.1	5	2231.67
YJR121W	539	6	1974.70
YKL067W	13.3	9	1819.59
YKL152C	490	2	136288.75
YLL018C	16.9	4	4267.74
YLR100W	1.2	5	846.81
YML008C	0.01083	9	4823.98
YMR205C	62	5	62358.96
YMR220W	10.2	5	945.02
YNR043W	4.9	5	1920.91
YOR074C	1.2	4	1081.38
YPL160W	5	3	23765.41
YPR033C	40	2	2190.87
YPR183W	70.9	6	1030.60
YKL094W	2.66	0	760.17
YOR236W	11.5	1	564.86
YPR081C	0.15	0	277.52
YFL030W	45	9	261.77
YDL141W	30.1	6	691.07
YER099C	60.68	4	626.48
YMR267W	260	7	614.65
YBL099W	539	6	4342.78
YBR039W	539	6	1884.31
YDR377W	539	6	163.70
YKL016C	539	6	263.25
YLR295C	539	6	6067.36
YML081C-A	539	6	260.84
YPL271W	539	6	6758.82
YBL045C	1500	4	242.32
YFR033C	1500	4	753.44
YGR183C	1500	4	354.91
YHR001W-A	1500	4	311.99
YJL166W	1500	4	116.73
YJR048W	1500	4	263.19
YOR065W	1500	4	1030.83
YLR044C	12.42	4	1713243.78

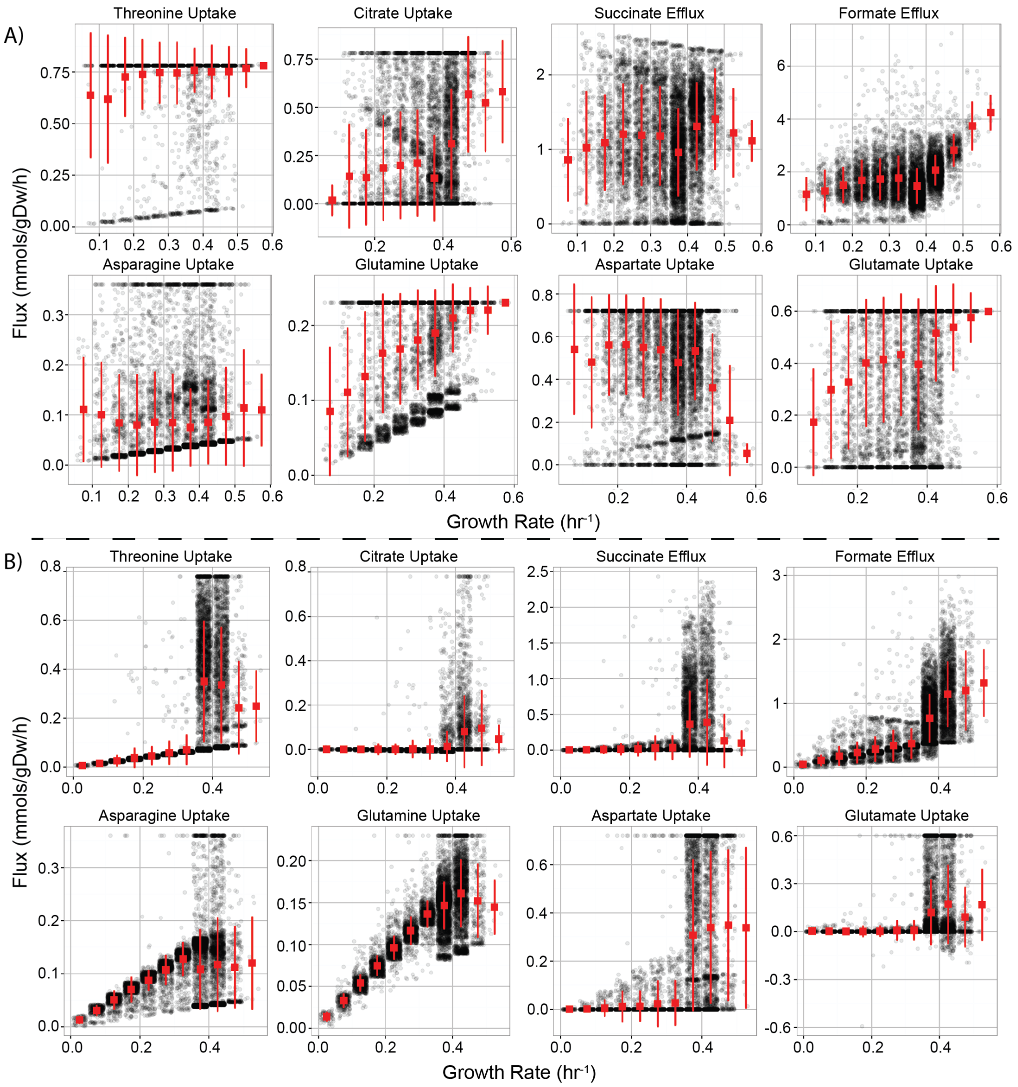

Fig. A10. Comparison of flux distributions between two independent GA optimizations. Bimodality in amino acid uptake can be observed in either slow growing cells (A) or fast growing cells (B) and was linked to the degree with which glycolysis is constrained (see Results Sections *SD media: Bimodality in Amino Acid Utilization and Degeneracy of Constraint Selection*). Black dots indicate individual cells, red squares and bars indicate mean and square deviation.

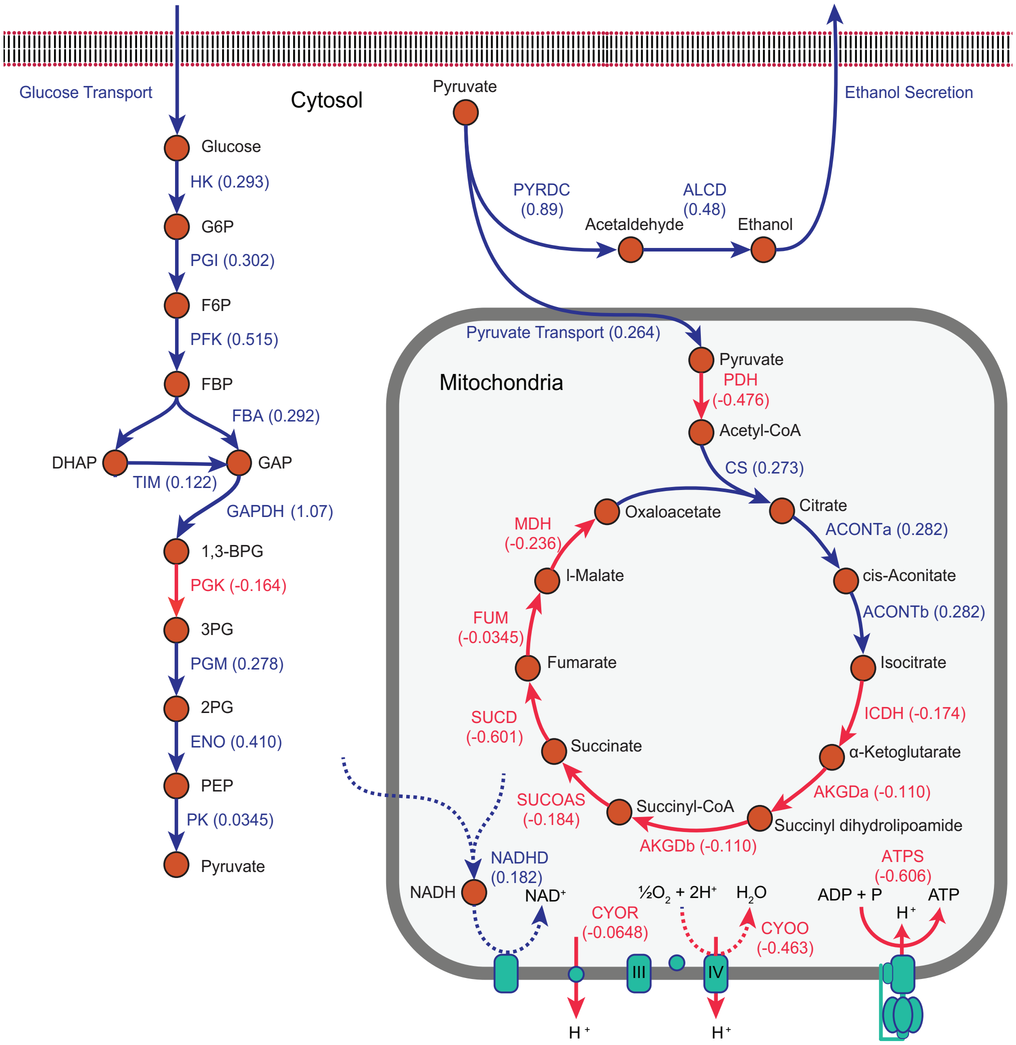

Fig. A11. Correlations between reactions in Central metabolism and glucose transporter HXT1. Correlations of reactions were calculated by processing the correlations of individual genes involved through Gene-Protein-Reaction (GPR) relationships from the metabolic model. Minimum correlation coefficient was taken in case of AND relationships and sum of correlation coefficients in case of OR relationship. Reactions with positive correlations were marked in blue and the ones with negative correlations in red.

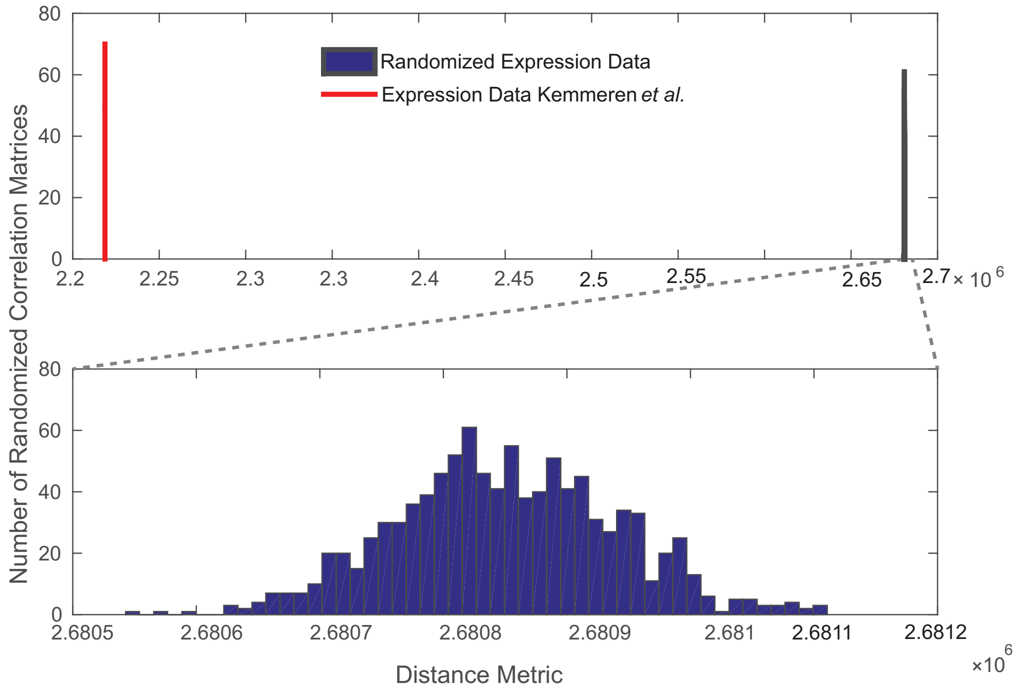

Fig. A12. Analysis of correlations in protein expression. Top: Distance metric of correlations from actual expression data as compared to randomized correlation data. Bottom: Distribution of distance metric obtained from randomizing expression data

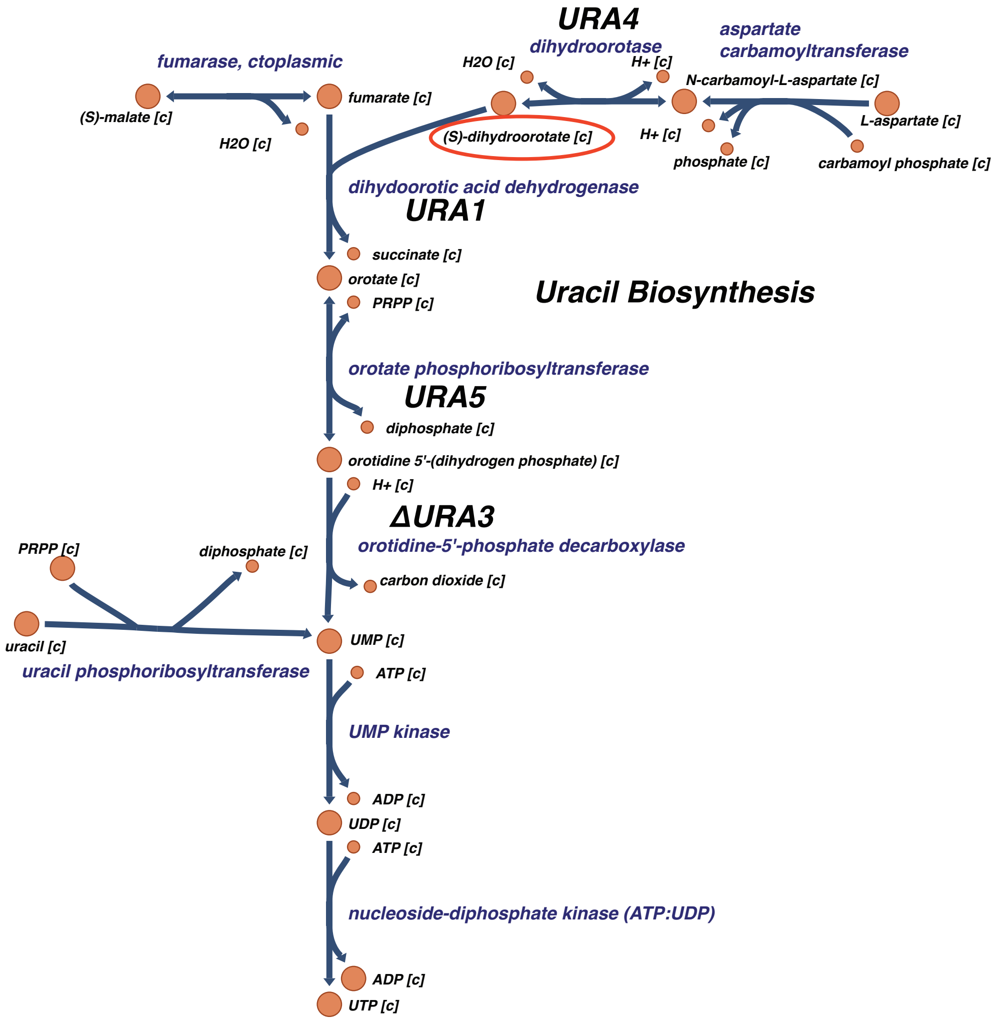

Fig. A13. Uracil biosynthesis pathway in yeast. *URA3* was deleted in this strain. Copy number of *URA1*, *URA4* and *URA5* were observed to be significant in spite of the deletion possibly due to upregulation by dihydroorotate (encircled)

Table A7. List of proteins with significant mean protein count but catalyze reactions which can't carry any flux in glucose SD medium growth conditions according to metabolic model for auxotrophic strain used in proteomics study based on Yeast 7.6 model

Systematic Name	Protein Name	Mean Copy	Name	Subsystem
YKL216W	URA1	18179.54	Dihydroorotate dehydrogenase	Pyrimidine biosynthesis
YGL009C	LEU1	16243.73	Isopropylmalate isomerase	leucine biosynthesis
YLR420W	URA4	2345.43	Dihydroorotase	Pyrimidine biosynthesis
YGR260W	TNA1	2052.47	Nicotinate permease	Permeases
YML106W	URA5	1945.02	orotate phosphoribosyltransferase isozyme	Pyrimidine biosynthesis
YMR113W	FOL3	1543.35	Dihydrofolate synthetase	folic acid biosynthesis
YDL100C	GET3	1085.58	Guanine nucleotide exchange factor	Cofactor biosynthesis
YGR255C	COQ6	1090.97	Flavin-dependent monooxygenase	ubiquinone biosynthesis
YPL059W	GRX5	830.09	Glutathione-dependent oxidoreductase	Iron sulfur center assembly
YGR010W	NMA2	800.00	Nicotinic acid mononucleotide adenylyl-transferase	NAD+ biosynthesis

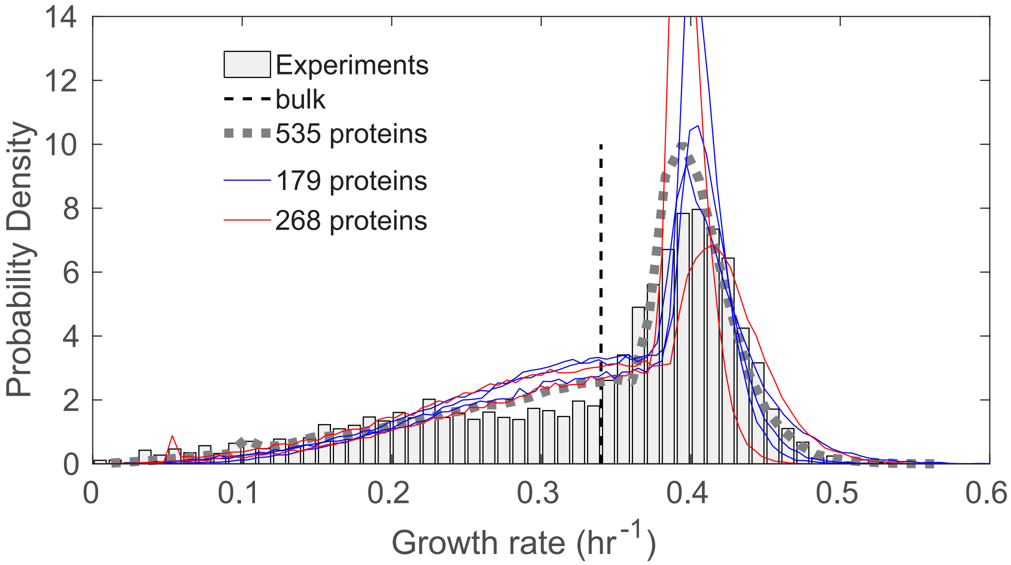

Fig. A14. Growth rate distributions predicted using 50 % of the available protein distributions in red (268 out of 535) and 33% of the available protein distributions in blue (179 out of 535) in SD medium. Also shown is experimental distribution in bars and growth rate distribution obtained using all 535 protein distributions in dashed line.

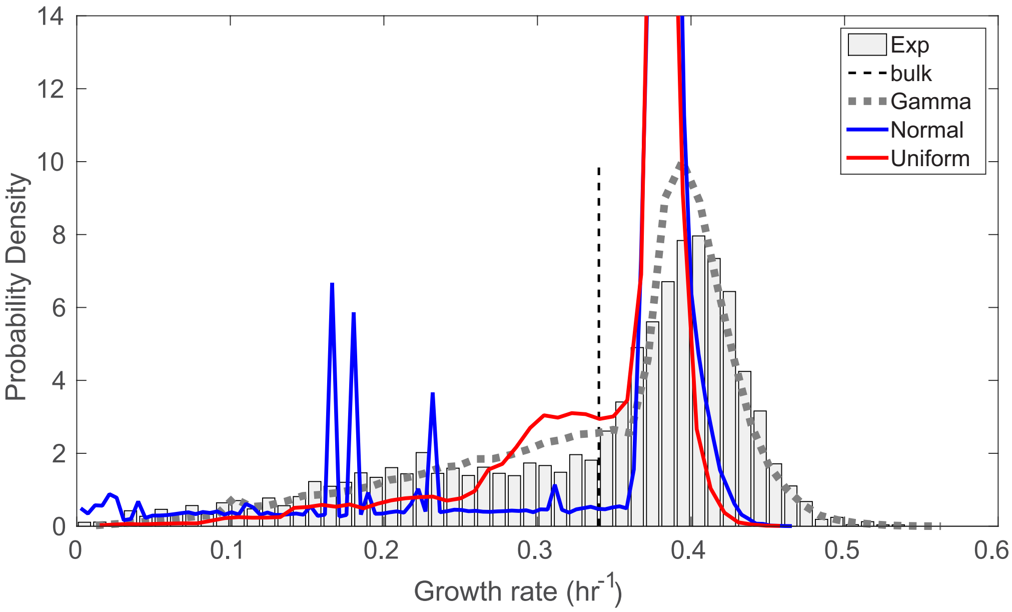

Fig. A15. Growth rate distributions predicted by replacing gamma distributions for all 535 proteins with uniform (red) or normal (blue) distributions keeping the mean protein copy number intact. Also shown is experimental growth rate distribution in bars and growth rate distribution obtained using gamma distributions in gray dashed line. Several artifacts emerge when using normally-distributed protein counts. Because normal distributions can take negative values, all sampled protein counts from the negative tails were changed to 2.87 (see Methods section "Conversion of Fluorescence to Protein Copy Numbers" in the main manuscript). This led to several "spikes" at low growth rates in our normally-distributed protein count population.

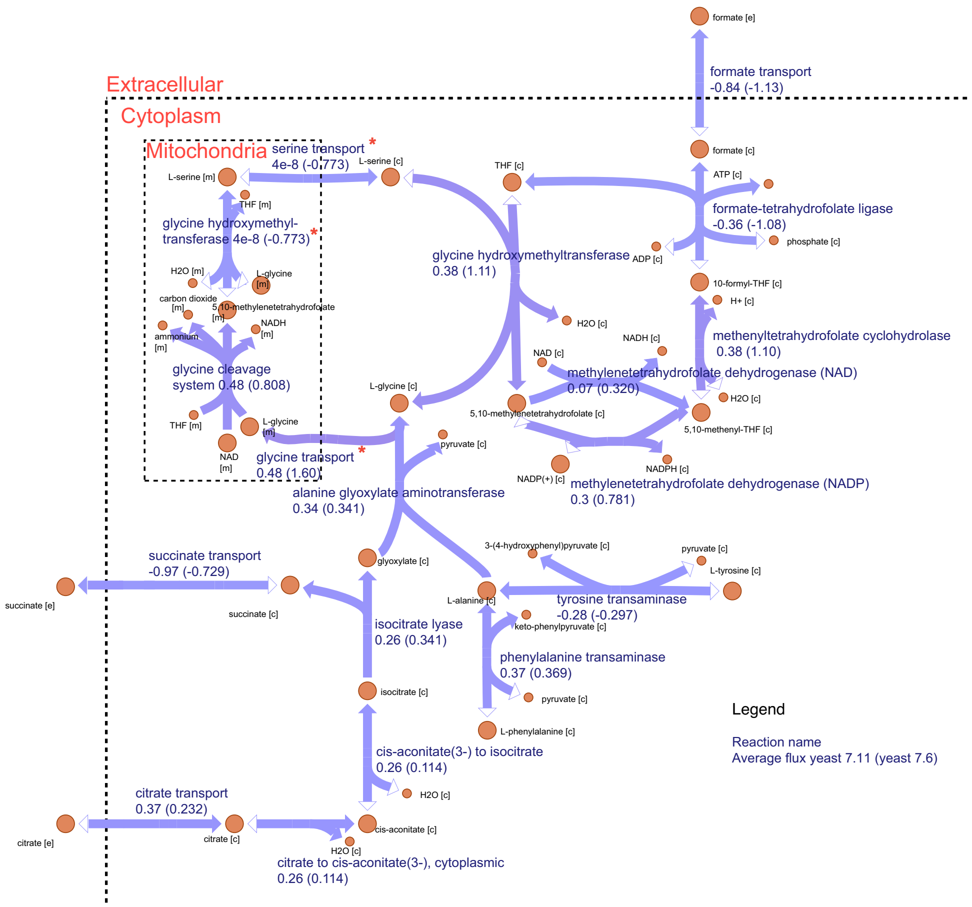

Fig. A16. Decrease in flux through serine glycine cycle because of irreversibility of three reactions involved Average flux from 1,000 cells generated using population FBA in SD medium and metabolic model with reversibilities for three reactions (marked with red star) from older version of the metabolic model yeast 7.11. Average flux obtained using latest version of the metabolic model yeast 7.6 are in parentheses. Directionality and thickness of reaction arrows are based on the flux distribution as predicted using yeast 7.6.

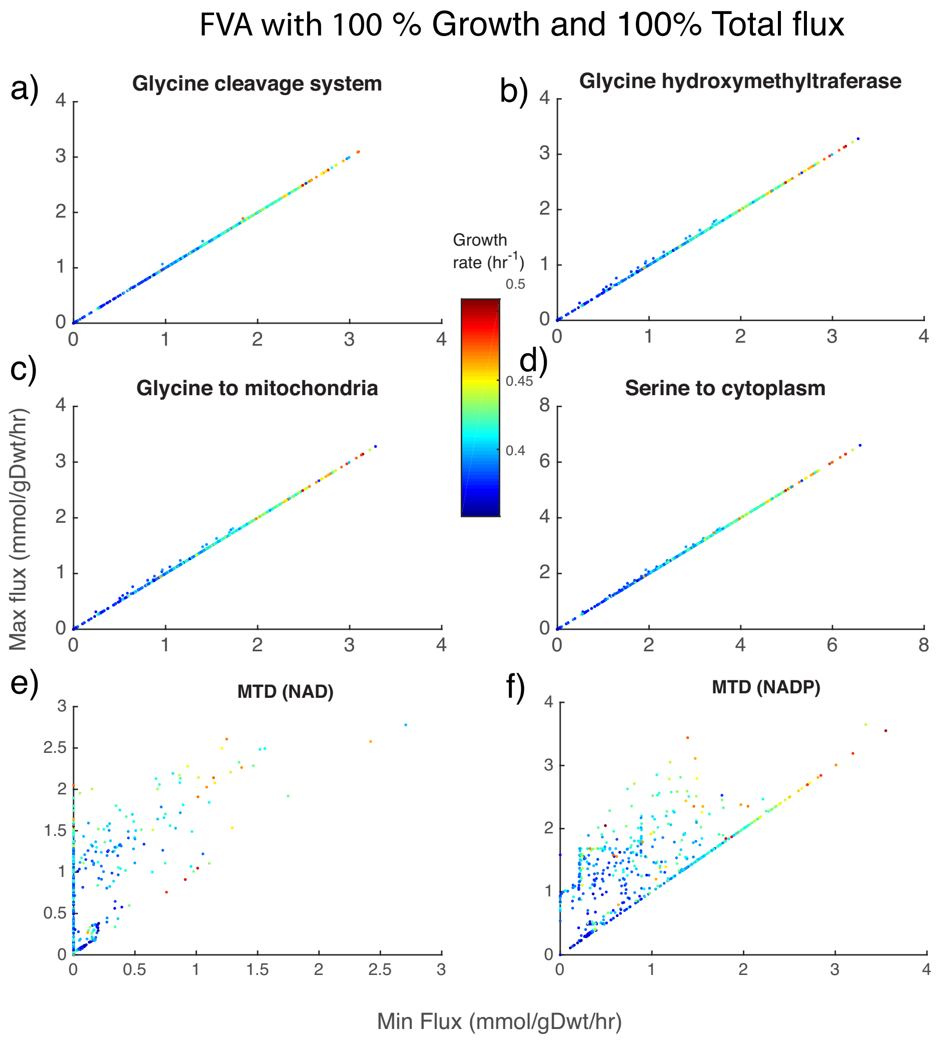

Fig. A17. Flux variability of reactions in serine glycine cycle among fast growing cells with 100 % optimality for growth and total flux Minimum and maximum fluxes through reactions in the serine glycine cycle according to flux variability analysis while maintaining same growth rate and total flux as calculated using pFBA. Fluxes are calculated for 605 fast growing cells with growth rate > 0.35 hr^{-1}. Little variation is exhibited except in the reactions involving methylenetetrahydrofolate dehydrogenase (MTD) which are alternate pathways for NADH and NADPH production.

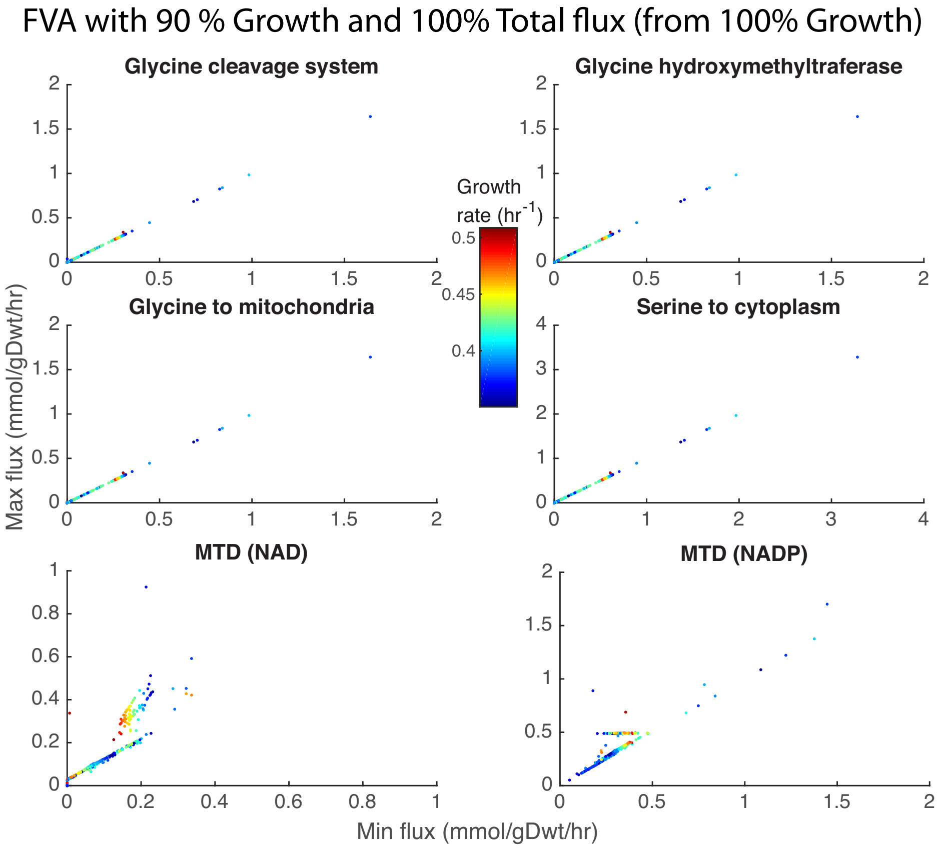

Fig. A18. Flux variability of reactions in serine glycine cycle among fast growing cells with 90 % optimality for growth and 100 % for total flux Minimum and maximum fluxes through reactions in the serine glycine cycle according to flux variability analysis while maintaining 90% of the optimal growth rate and 100 % of optimal total flux as calculated using pFBA. Optimal total flux is calculated at 100 % optimal growth. Fluxes are calculated for 605 fast growing cells with growth rate > 0.35 hr^{-1}. Little variation is exhibited except in the reactions involving methylenetetrahydrofolate dehydrogenase (MTD) which are alternate pathways for NADH and NADPH production. Lower fluxes are observed than the case when 100 % optimality is enforced both for growth rate and total flux because lower growth requirement reduces the energy requirement and hence the flux through the cycle.

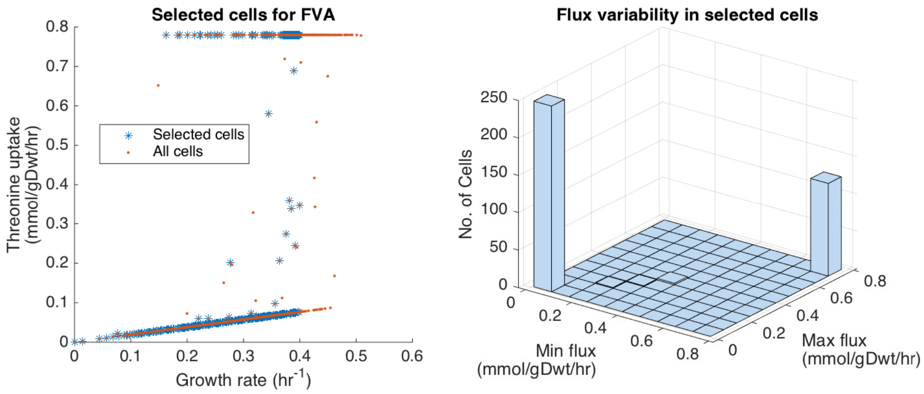

Fig. A19. Flux variability in threonine uptake among selected cells Flux variability in threonine uptake of over 400 cells selected randomly from cells growing slower than 0.4 hr^{-1} as shown in the left panel. Right panel shows histogram with no variability in threonine uptake while growth rate and total flux is fixed at its optimal value as determined using pFBA.

Table A8. Comparison of Biomass Pseudo-Reactions Among the Latest Yeast Metabolic Models.

Metabolite	Coefficients		Metabolite	Coefficients	
	iMM904 [41]	Yeast 5 [42]		Yeast 6 [43]	Yeast 7.6 [1]
(1-¿3)-beta-D-glucan	1.1348	1.14		1.1348	1.1348
(1-¿6)-beta-D-glucan				1.1348	1.1348
chitin	0.000001				0.000001
glycogen	0.5185	0.519		0.5185	0.5185
mannan	0.8079	0.821		0.8079	0.8079
trehalose	0.0234	0.0234		0.0234	0.0234
lipids		1		1	1
AMP	0.046	0.051		0.046	0.046
ATP	59.276	59.3		59.276	59.276
CMP	0.0447	0.05		0.0447	0.0447
GMP	0.046	0.051		0.046	0.046
UMP	0.0599	0.067		0.0599	0.0599
dAMP	0.0036	0.00359		0.0036	0.0036
dCMP	0.0024	0.00243		0.0024	0.0024
dGMP	0.0024	0.00243		0.0024	0.0024
dTMP	0.0036	0.00359		0.0036	0.0036
H2O	59.276	59.3		59.276	59.276
sulphate	0.02	0.02		0.02	0.02
riboflavin	0.00099	0.0009		0.00099	0.00099
heme a					0.000001
L-alanine	0.4588	0.357	Ala-tRNA(Ala)	0.4588	0.4588
L-arginine	0.1607	0.136	Arg-tRNA(Arg)	0.1607	0.1607
L-asparagine	0.1017	0.172	Asn-tRNA(Asn)	0.1017	0.1017
L-aspartate	0.2975	0.172	Asp-tRNA(Asp)	0.2975	0.2975
L-cysteine	0.0066	0.0429	Cys-tRNA(Cys)	0.0066	0.0066
L-glutamate	0.1054	0.268	Gln-tRNA(Gln)	0.1054	0.1054
L-glutamine	0.3018	0.268	Glu-tRNA(Glu)	0.3018	0.3018
L-glycine	0.2904	0.325	Gly-tRNA(Gly)	0.2904	0.2904
L-histidine	0.0663	0.075	His-tRNA(His)	0.0663	0.0663
L-isoleucine	0.1927	0.172	Ile-tRNA(Ile)	0.1927	0.1927
L-leucine	0.2964	0.25	Leu-tRNA(Leu)	0.2964	0.2964
L-lysine	0.2862	0.239	Lys-tRNA(Lys)	0.2862	0.2862
L-methionine	0.0507	0.05	Met-tRNA(Met)	0.0507	0.0507
L-phenylalanine	0.1339	0.114	Phe-tRNA(Phe)	0.1339	0.1339
L-proline	0.1647	0.129	Pro-tRNA(Pro)	0.1647	0.1647
L-serine	0.1854	0.254	Ser-tRNA(Ser)	0.1854	0.1854
L-threonine	0.1914	0.197	Thr-tRNA(Thr)	0.1914	0.1914
L-tryptophan	0.0284	0.028	Trp-tRNA(Trp)	0.0284	0.0284
L-tyrosine	0.102	0.0965	Tyr-tRNA(Tyr)	0.102	0.102
L-valine	0.2646	0.257	Val-tRNA(Val)	0.2646	0.2646
cAMP	0.000001				
CoA	0.000001				
Ergst	0.0007				
Glutathione reduced	0.000001				
FAD	0.000001				
Phosphatidate	0.000006				
Phosphatidylcholine	0.00006				
phosphatidylethanolamine	0.000045				
phosphatidyl-1D-myo-inositol	0.000053				
phosphatidylserine	0.000017				
zymosterol	0.0015				
triglyceride	0.000066				
protoheme	0.000001				
nad	0.000001				

Aminoacylation reactions are not present in all models, therefore their biomass pseudo-reaction uses either amino acids (iMM904 and Yeast 5), or charged tRNAs (Yeast 6 and Yeast 7). All yeast consensus models were taken from *yeast.sourceforge.net.*

References

1. Aung HW, Henry SA, Walker LP. Revising the representation of fatty acid, glycerolipid, and glycerophospholipid metabolism in the consensus model of yeast metabolism. 2013;9(4):215–228.

2. Heavner BD, Price ND. Comparative Analysis of Yeast Metabolic Network Models Highlights Progress, Opportunities for Metabolic Reconstruction. 2015;11(11):e1004530.

3. Dénervaud N, Becker J, Ricard D, Damay P, Rajkumar AS, Unser M, et al. A chemostat array enables the spatio-temporal analysis of the yeast proteome. 2013;110(39):15842–15847.

4. Levy SF, Ziv N, Siegal ML. Bet hedging in yeast by heterogeneous, age-correlated expression of a stress protectant. 2012;10(5):e1001325.

5. Stewart BJ, Navid A, Turteltaub KW, Bench G. Yeast Dynamic Metabolic Flux Measurement In Nutrient-Rich Media By HPLC And Accelerator Mass Spectrometer. 2010;82(23):9812–9817.

6. Schomburg I, Hofmann O, Baensch C, Chang A, Schomburg D. Enzyme data and metabolic information: BRENDA, a resource for research in biology, biochemistry, and medicine. 2000 oct;1(3-4):109–118.

7. Chang A, Schomburg I, Placzek S, Jeske L, Ulbrich M, Xiao M, et al. BRENDA in 2015: exciting developments in its 25th year of existence. 2015 jan;43(Database issue):D439–46.

8. Newman JRS, Ghaemmaghami S, Ihmels J, Breslow DK, Noble M, DeRisi JL, et al. Single-cell proteomic analysis of *S. cerevisiae* reveals the architecture of biological noise. Nature. 2006;441(7095):840–846.

9. Taniguchi Y, Choi PJ, Li GW, Chen H, Babu M, Hearn J, et al. Quantifying *E. coli* proteome and transcriptome with single-molecule sensitivity in single cells. Science. 2010;329(5991):533–538.

10. Chandrasekaran S, Price ND. Metabolic constraint-based refinement of transcriptional regulatory networks. 2013;9(12):e1003370.

11. Kemmeren P, Sameith K, Van De Pasch LaL, Benschop JJ, Lenstra TL, Margaritis T, et al. Large-scale genetic perturbations reveal regulatory networks and an abundance of gene-specific repressors. Cell. 2014 apr;157(3):740–752.

12. Krishnakumar K. Micro-genetic algorithms for stationary and non-stationary function optimization. In: 1989 Advances in Intelligent Robotics Systems Conference. International Society for Optics and Photonics; 1990. p. 289–296.

13. Roy A, Exinger F, Losson R. cis- and trans-acting regulatory elements of the yeast URA3 promoter. 1990;10(10):5257–5270.

14. Baichwal VR, Cunningham TS, Gatzek PR, Kohlhaw GB. Leucine biosynthesis in yeast. Current Genetics. 1983;7(5):369–377.

15. King ZA, Dräger A, Ebrahim A, Sonnenschein N, Lewis NE, Palsson BO. Escher: A Web Application for Building, Sharing, and Embedding Data-Rich Visualizations of Biological Pathways. PLoS Comput Biol. 2015 aug;11(8):e1004321.

16. Christen S, Sauer U. Intracellular characterization of aerobic glucose metabolism in seven yeast species by 13C flux analysis and metabolomics. 2011;11(3):263–272.

17. Furukawa K, Tagaya M, Tanizawa K, Fukui T. Identification of Lys277 at the active site of Escherichia coli glycogen synthase. Application of affinity labeling combined with site-directed mutagenesis. Journal of Biological Chemistry. 1994;269(2):868–871.

18. Villegas-Torres MF, Martinez-Torres RJ, Cazeres A, Hailes H, Baganz F, Ward J. Multi-step biocatalytic strategies for chiral amino alcohol synthesis. Enzyme and microbial technology. 2015;81:23–30.

19. Whittle E, Shanklin J. Engineering Δ9-16: 0-acyl carrier protein (ACP) desaturase specificity based on combinatorial saturation mutagenesis and logical redesign of the castor Δ9-18: 0-ACP desaturase. Journal of Biological Chemistry. 2001;276(24):21500–21505.

20. Bornemann S, Lowe DJ, Thorneley RN. The transient kinetics of Escherichia coli chorismate synthase: substrate consumption, product formation, phosphate dissociation, and characterization of a flavin intermediate. Biochemistry. 1996;35(30):9907–9916.

21. Basurko MJ, Marche M, Darriet M, Cassaigne A. Phosphoserine Aminotransferase, the Second Step-Catalyzing Enzyme for Serine Biosynthesis. IUBMB life. 1999;48(5):525–529.

22. Freist W, Cramer F. Valyl-tRNA synthetase from yeast. European Journal of Biochemistry. 1990;191(1):123–129.

23. Rose IA, O'Connell EL, Litwin S, Tana JB. Determination of the rate of hexokinase-glucose dissociation by the isotope-trapping method. Journal of Biological Chemistry. 1974;249(16):5163–5168.

24. López G, Quezada H, Duhne M, González J, Lezama M, El-Hafidi M, et al. Diversification of Paralogous α-Isopropylmalate Synthases by Modulation of Feedback Control and Hetero-Oligomerization in Saccharomyces cerevisiae. Eukaryotic cell. 2015;14(6):564–577.

25. Hommel U, Eberhard M, Kirschner K. Phosphoribosyl anthranilate isomerase catalyzes a reversible amadori reaction. Biochemistry. 1995;34(16):5429–5439.

26. Branson JP, Nezic M, Wallace JC, Attwood PV. Kinetic characterization of yeast pyruvate carboxylase isozyme pyc1. Biochemistry. 2002;41(13):4459–4466.

27. Cáceres AJ, Quiñones W, Gualdrón M, Cordeiro A, Avilán L, Michels PA, et al. Molecular and biochemical characterization of novel glucokinases from Trypanosoma cruzi and Leishmania spp. Molecular and biochemical parasitology. 2007;156(2):235–245.

28. Vries S, Grivell LA. Purification and characterization of a rotenone-insensitive NADH: Q6 oxidoreductase from mitochondria of Saccharomyces cerevisiae. European journal of biochemistry. 1988;176(2):377–384.

29. Kim H. Characterization of Two Site-specific Mutations in Human Dihydrolipoamide Dehydrogenase. Bulletin of the Korean Chemical Society. 2013;34(6):1621–1622.

30. Ling J, Peterson KM, Simonović I, Söll D, Simonović M. The mechanism of pre-transfer editing in yeast mitochondrial threonyl-tRNA synthetase. Journal of Biological Chemistry. 2012;287(34):28518–28525.

31. Nazi I, Scott A, Sham A, Rossi L, Williamson PR, Kronstad JW, et al. Role of homoserine transacetylase as a new target for antifungal agents. Antimicrobial agents and chemotherapy. 2007;51(5):1731–1736.

32. Graindorge JS, Senger B, Tritch D, Simos G, Fasiolo F. Role of Arc1p in the modulation of yeast glutamyl-tRNA synthetase activity. Biochemistry. 2005;44(4):1344–1352.

33. Hoskins AA, Anand R, Ealick SE, Stubbe J. The formylglycinamide ribonucleotide amidotransferase complex from Bacillus subtilis: metabolite-mediated complex formation. Biochemistry. 2004;43(32):10314–10327.

34. Wang P, Song P, Jin M, Zhu G. Isocitrate dehydrogenase from Streptococcus mutans: biochemical properties and evaluation of a putative phosphorylation site at Ser102. PloS one. 2013;8(3):e58918.

35. Turner K, Doherty M, Heering H, Armstrong F, Reid G, Chapman S. Redox properties of flavocytochrome c 3 from Shewanella frigidimarina NCIMB400. Biochemistry. 1999;38(11):3302–3309.

36. Johnson DE, Hanson RS. Bacterial citrate synthases: purification, molecular weight and kinetic mechanism. Biochimica et Biophysica Acta (BBA)-Enzymology. 1974;350(2):336–353.

37. Jameson D, Verma M, Westerhoff HV. Methods in Systems Biology. Manchester, UK: Academic Press; 2011.

38. Abrusci P, Chiarelli LR, Galizzi A, Fermo E, Bianchi P, Zanella A, et al. Erythrocyte adenylate kinase deficiency: characterization of recombinant mutant forms and relationship with nonspherocytic hemolytic anemia. Experimental hematology. 2007;35(8):1182–1189.

39. Wu YH, Chang CP, Chien CI, Tseng YK, Wang CC. An insertion peptide in yeast glycyl-tRNA synthetase facilitates both productive docking and catalysis of cognate tRNAs. Molecular and cellular biology. 2013;33(17):3515–3523.

40. Ragsdale S, Ljungdahl L. Purification and properties of NAD-dependent 5, 10-methylenetetrahydrofolate dehydrogenase from Acetobacterium woodii. Journal of Biological Chemistry. 1984;259(6):3499–3503.

41. Mo ML, Palsson BO, Herrgå rd MJ. Connecting extracellular metabolomic measurements to intracellular flux states in yeast. 2009 Jan;3(1):37.

42. Heavner BD, Smallbone K, Barker B, Mendes P, Walker LP. Yeast 5 - an expanded reconstruction of the Saccharomyces cerevisiae metabolic network. 2012 Jan;6(1):55.

43. Heavner BD, Smallbone K, Price ND, Walker LP. Version 6 of the consensus yeast metabolic network refines biochemical coverage and improves model performance. Database. 2013 Aug;2013(0):bat059.
