# Supplementary material for: Population FBA predicts metabolic phenotypes in yeast
Source: PLoS Comput Biol. 2017 Sep 8;13(9):e1005728. doi: 10.1371/journal.pcbi.1005728 (PMC5626512; doi:10.1371/journal.pcbi.1005728)
Supplement: S1 File — Model files contain all modifications to reflect gene deletions and media conditions. Parameter files with all shape and scale parameters for fluorescence distributions of proteins used in our simulations, along with their original kcat values, final kcat values after doubling procedure in both SD and 13C media as well as scaling ratios to convert sampled SD protein count to 13C count. There is also listing of final kcat values obtained after 10 independent GA optimizations in SD media. Correlation matrix imposed while sampling distributions as well as its Cholesky decomposed factor is also provided. (ZIP) [file pcbi.1005728.s002.zip › FluorescenceData/CombinedPlots_Distributions_MeanSD_CVs_Clusters_Calibration.pdf]

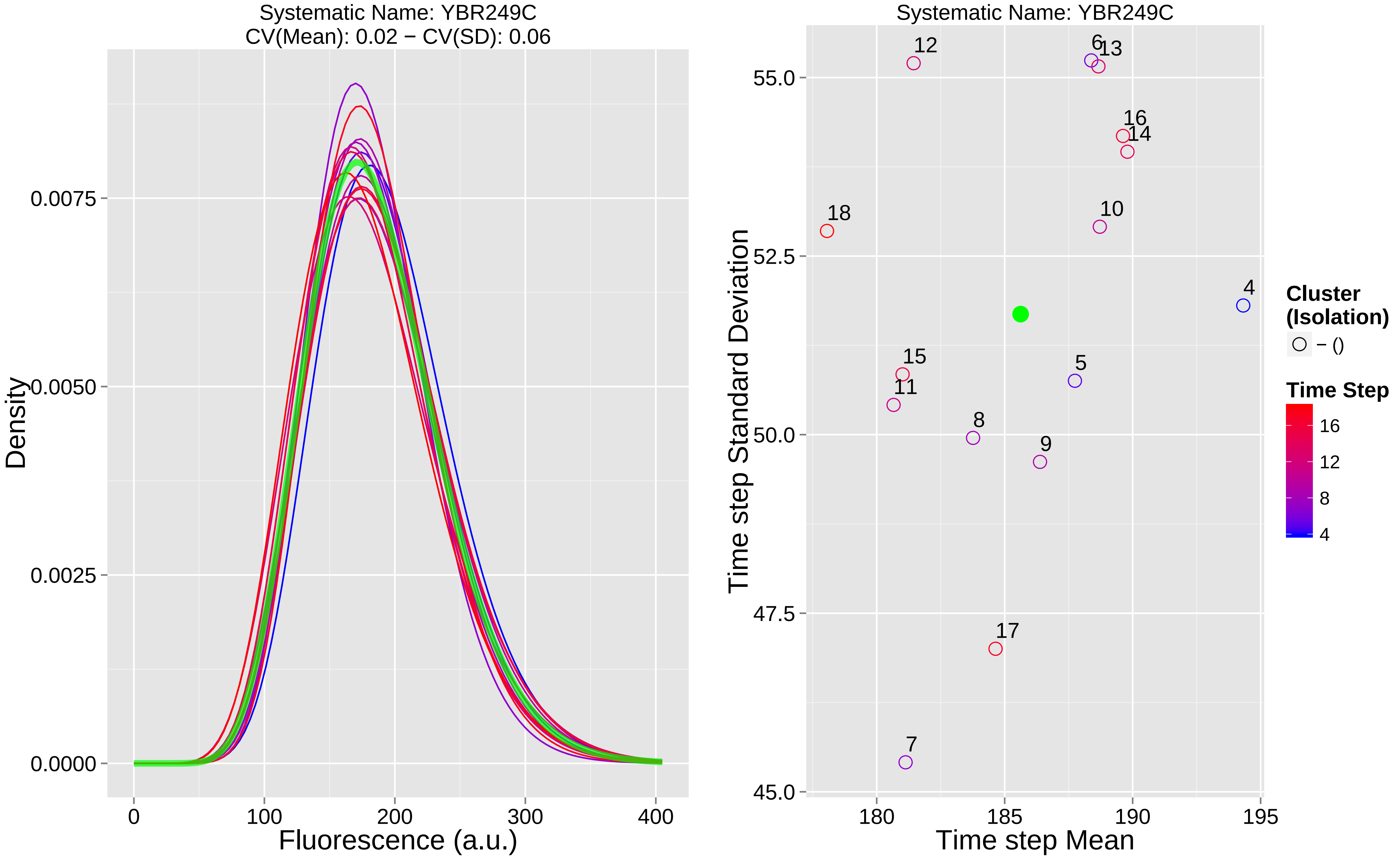
Systematic Name: YBR249C
CV(Mean): 0.02 − CV(SD): 0.06
Density
0.0075
0.0050
0.0025
0.0000
0
100
200
300
400
Fluorescence (a.u.)
Systematic Name: YBR249C
Time step Standard Deviation
55.0
52.5
50.0
47.5
45.0
180
185
190
195
Time step Mean
12
6
13
16
14
18
10
4
15
5
11
8
9
17
7
Cluster
(Isolation)
− ()
Time Step
16
12
8
4

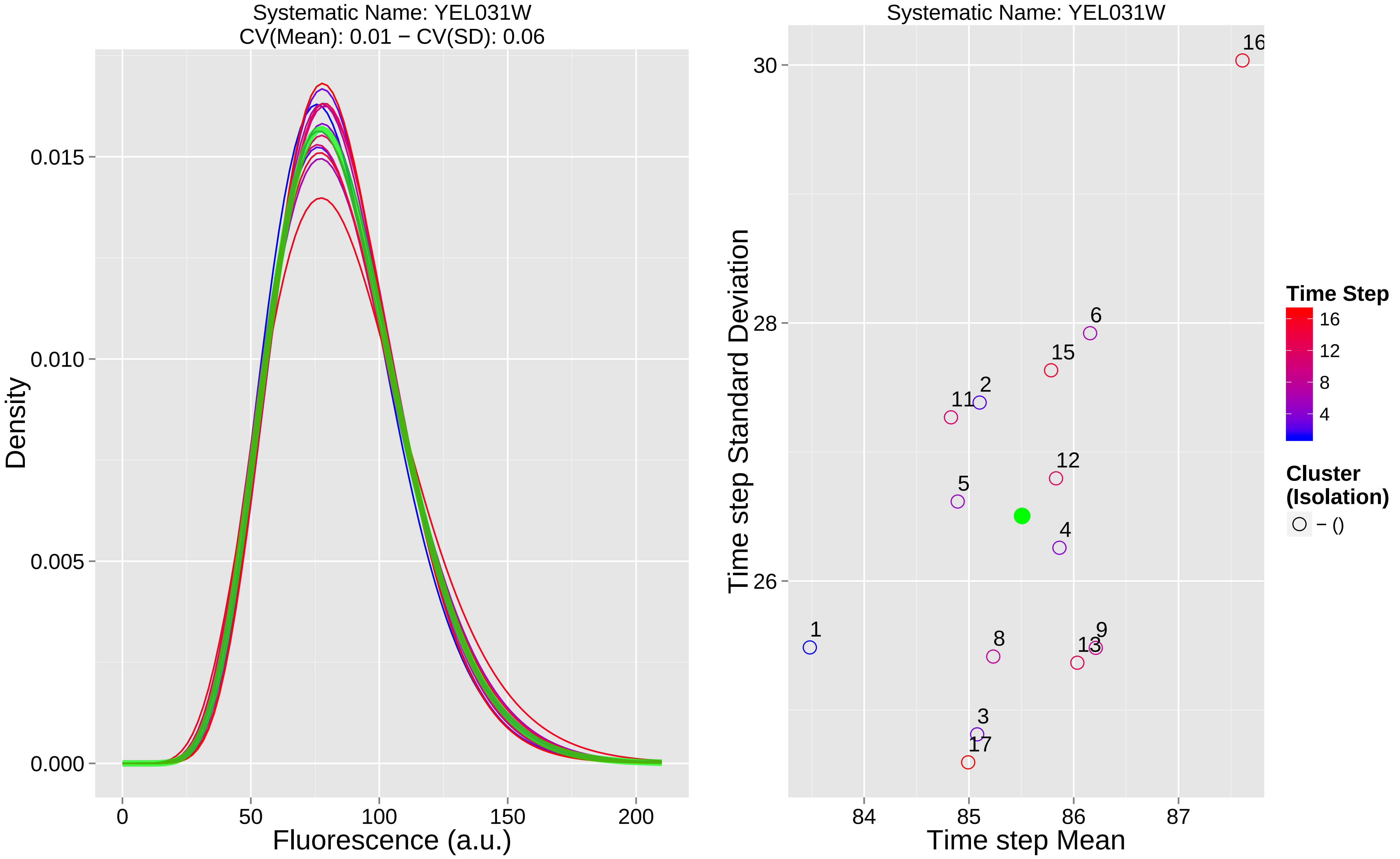

Systematic Name: YEL031W
CV(Mean): 0.01 − CV(SD): 0.06
Density
0.000
0.005
0.010
0.015
0
50
100
150
200
Fluorescence (a.u.)
Systematic Name: YEL031W
Time step Standard Deviation
30
28
26
84
85
86
87
Time step Mean
1
2
3
4
5
6
8
9
11
12
13
15
16
17
Time Step
16
12
8
4
Cluster
(Isolation)
− ()

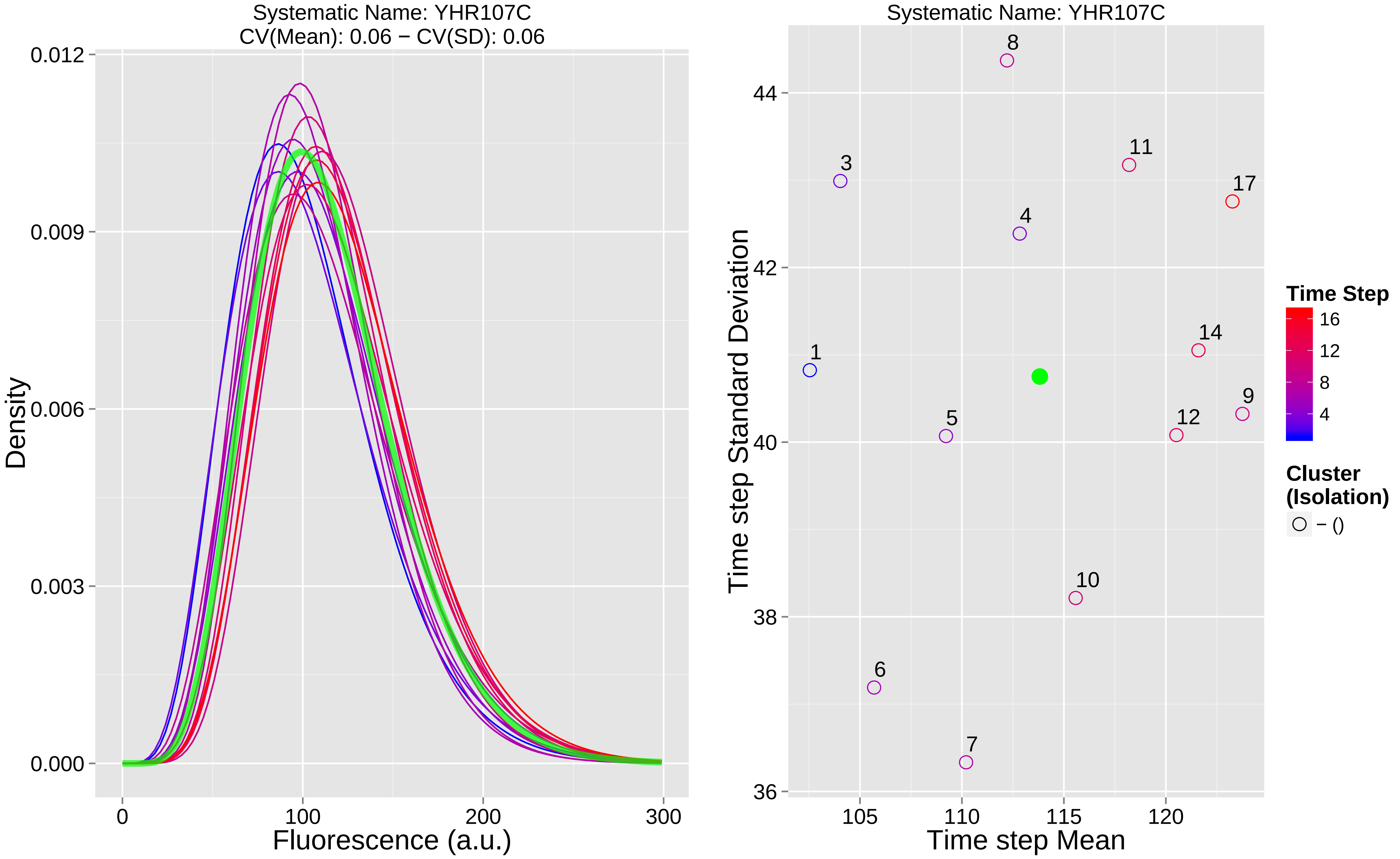
Systematic Name: YHR107C
CV(Mean): 0.06 − CV(SD): 0.06
Density
0.012
0.009
0.006
0.003
0.000
0
100
200
300
Fluorescence (a.u.)
Systematic Name: YHR107C
Time step Standard Deviation
44
42
40
38
36
105
110
115
120
Time step Mean
1
3
4
5
6
7
8
9
10
11
12
14
17
Time Step
16
12
8
4
Cluster
(Isolation)
− ()

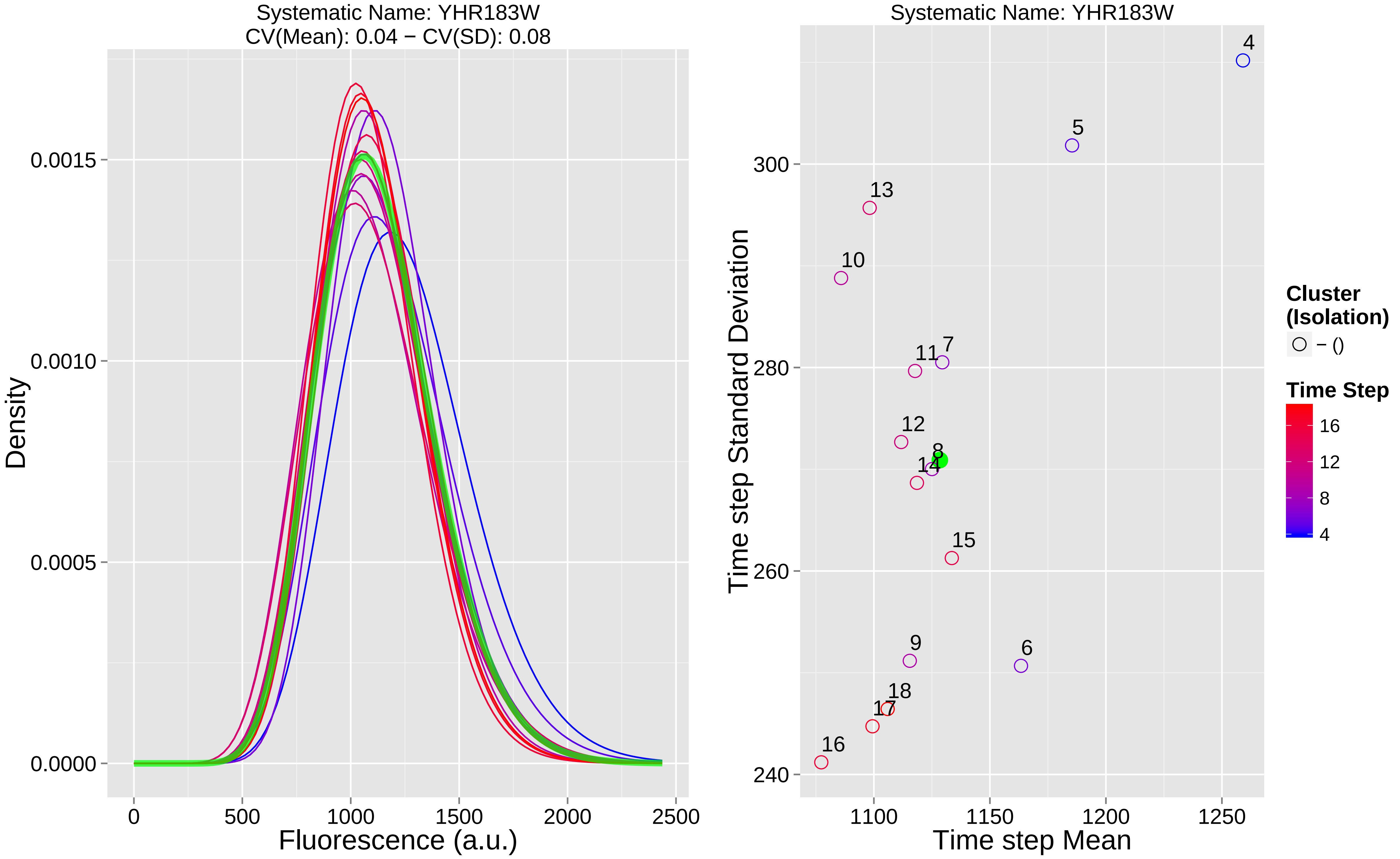
Systematic Name: YHR183W
CV(Mean): 0.04 − CV(SD): 0.08
Density
0.0015
0.0010
0.0005
0.0000
0
500
1000
1500
2000
2500
Fluorescence (a.u.)
Systematic Name: YHR183W
Time step Standard Deviation
300
280
260
240
1100
1150
1200
1250
Time step Mean
4
5
13
10
11
7
12
8
14
15
9
6
18
17
16
Cluster (Isolation)
− ()
Time Step
16
12
8
4

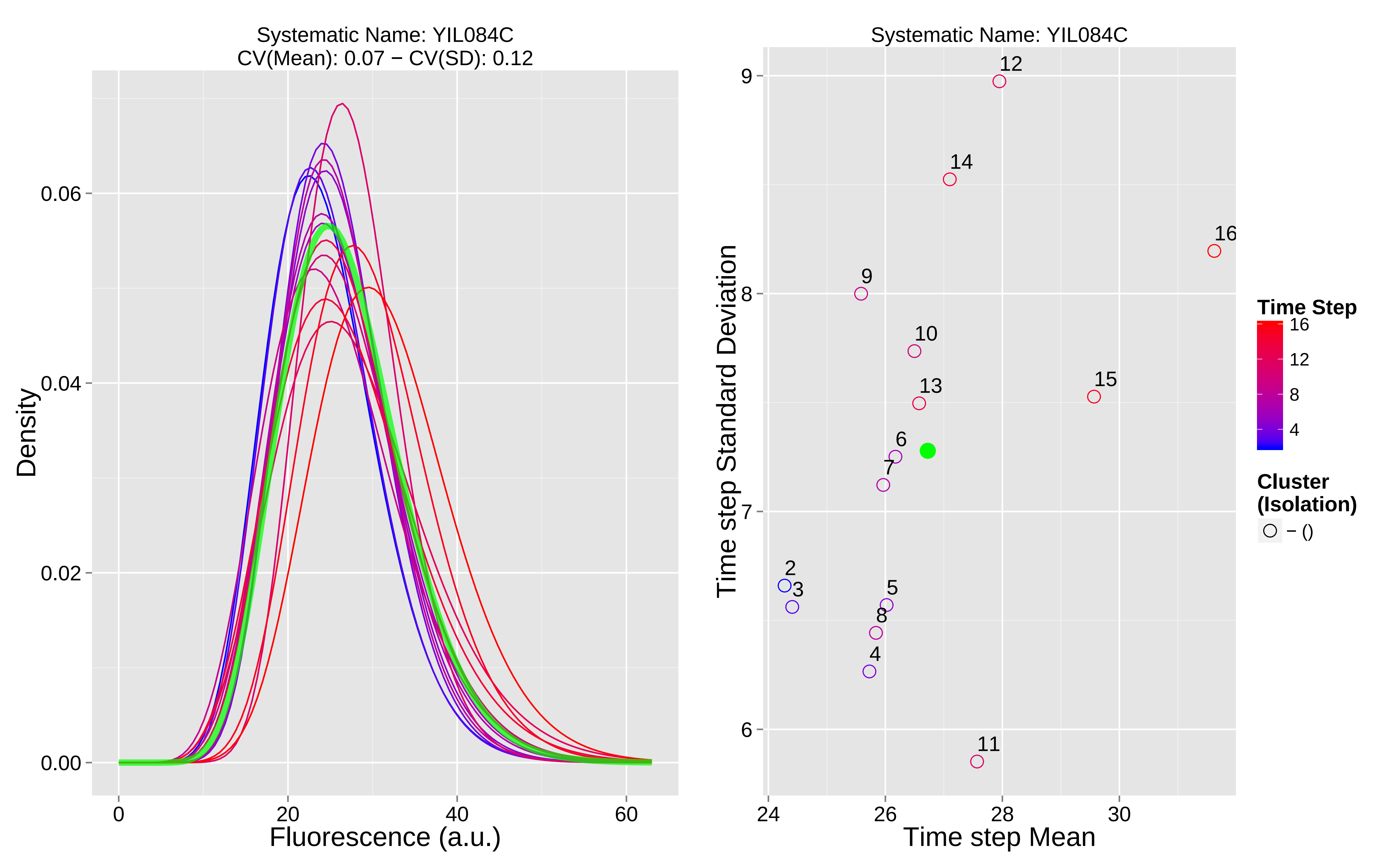
Systematic Name: YIL084C
CV(Mean): 0.07 − CV(SD): 0.12
Density
0.00
0.02
0.04
0.06
0
20
40
60
Fluorescence (a.u.)
Systematic Name: YIL084C
Time step Standard Deviation
6
7
8
9
24
26
28
30
Time step Mean
12
14
16
9
10
13
15
6
7
2
3
5
8
4
11
Time Step
16
12
8
4
Cluster
(Isolation)
− ()

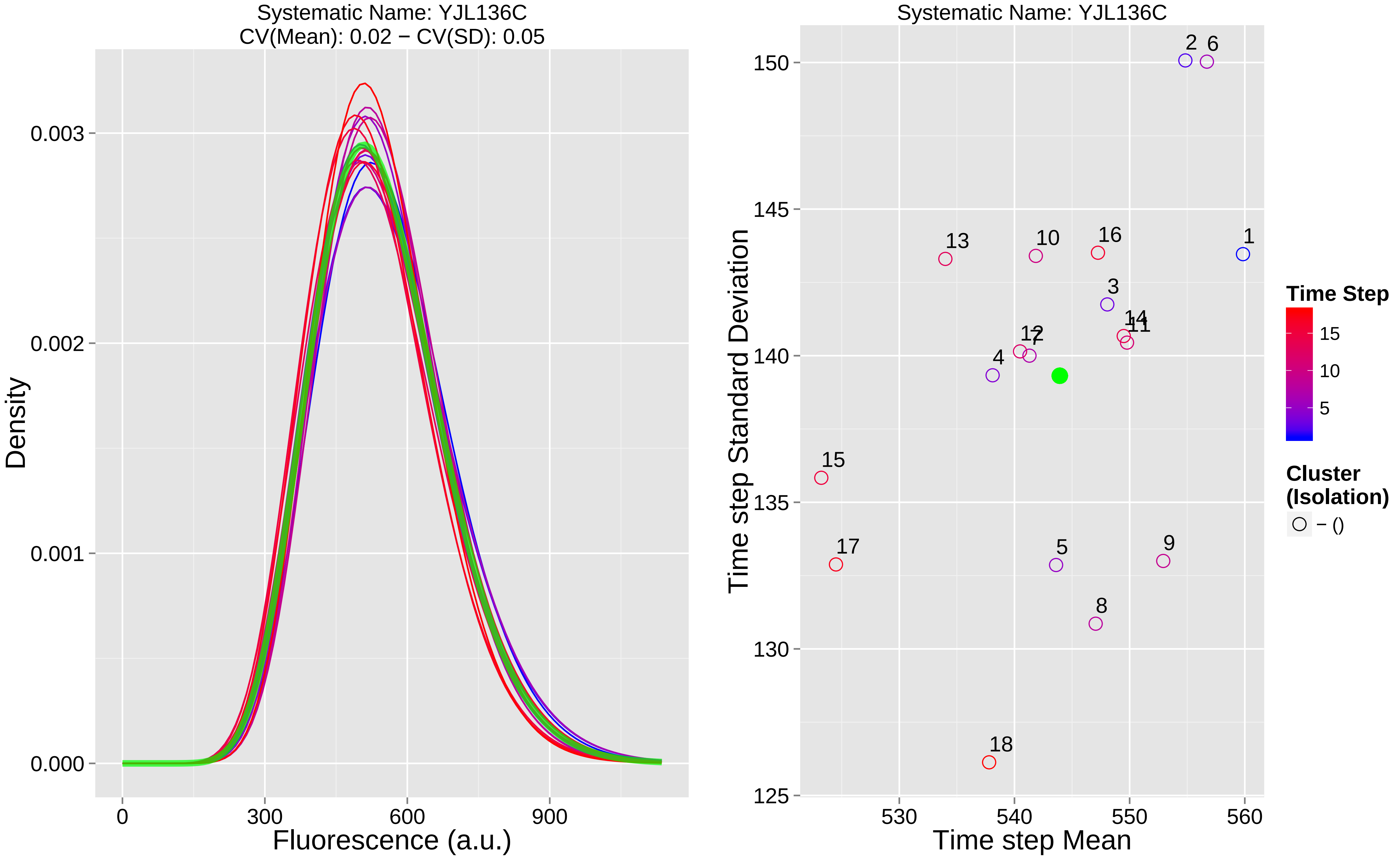

Systematic Name: YJL136C
CV(Mean): 0.02 − CV(SD): 0.05
Density
0.003
0.002
0.001
0.000
0
300
600
900
Fluorescence (a.u.)
Systematic Name: YJL136C
Time step Standard Deviation
150
145
140
135
130
125
530
540
550
560
Time step Mean
2
6
13
10
16
1
3
14
11
12
7
4
15
17
5
9
8
18
Time Step
15
10
5
Cluster
(Isolation)
− ()

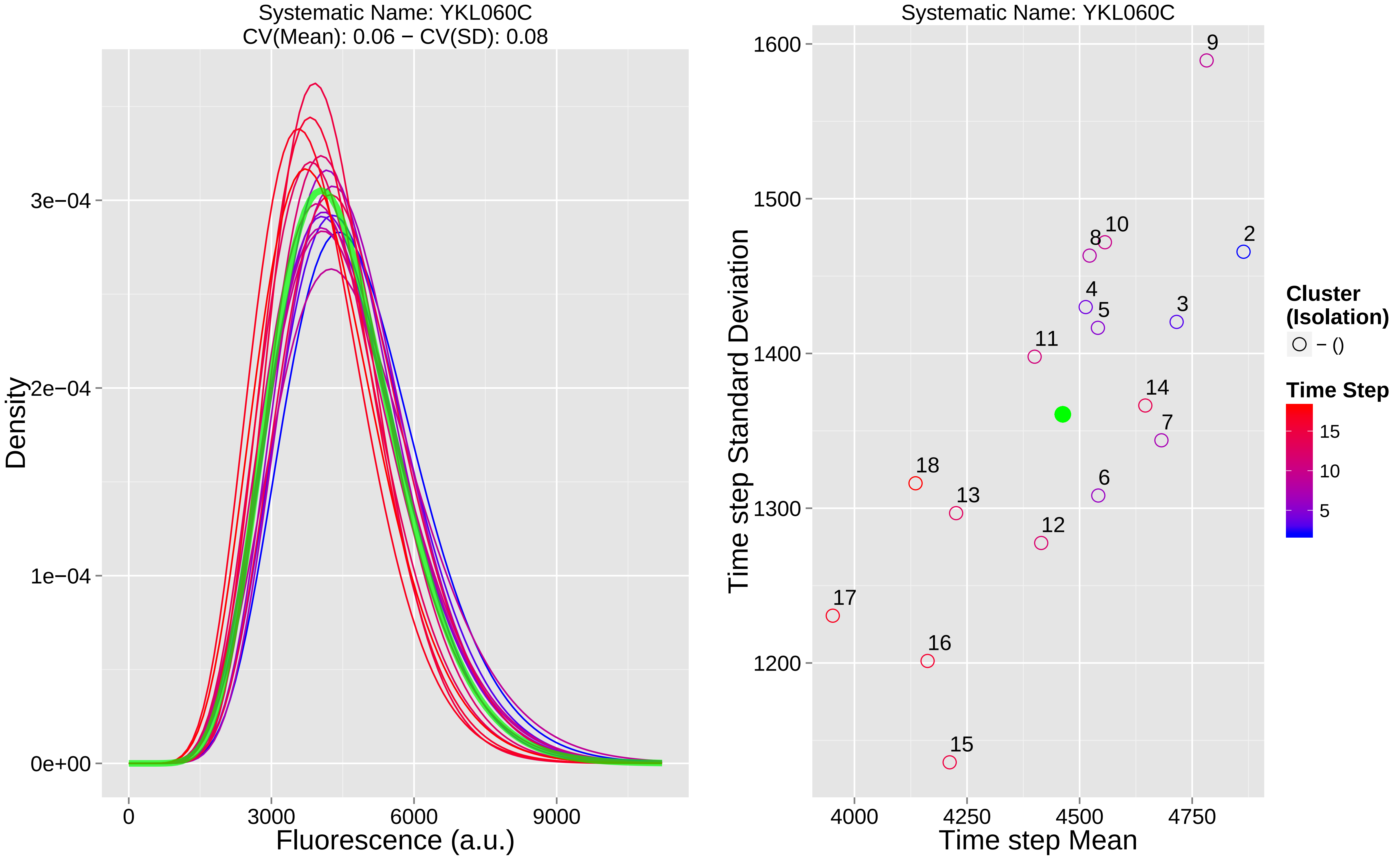
Systematic Name: YKL060C
CV(Mean): 0.06 − CV(SD): 0.08
Density
3e−04
2e−04
1e−04
0e+00
0
3000
6000
9000
Fluorescence (a.u.)
Systematic Name: YKL060C
Time step Standard Deviation
1600
1500
1400
1300
1200
4000
4250
4500
4750
Time step Mean
9
10
8
2
4
5
3
11
14
7
18
13
6
12
17
16
15
Cluster
(Isolation)
− ()
Time Step
15
10
5

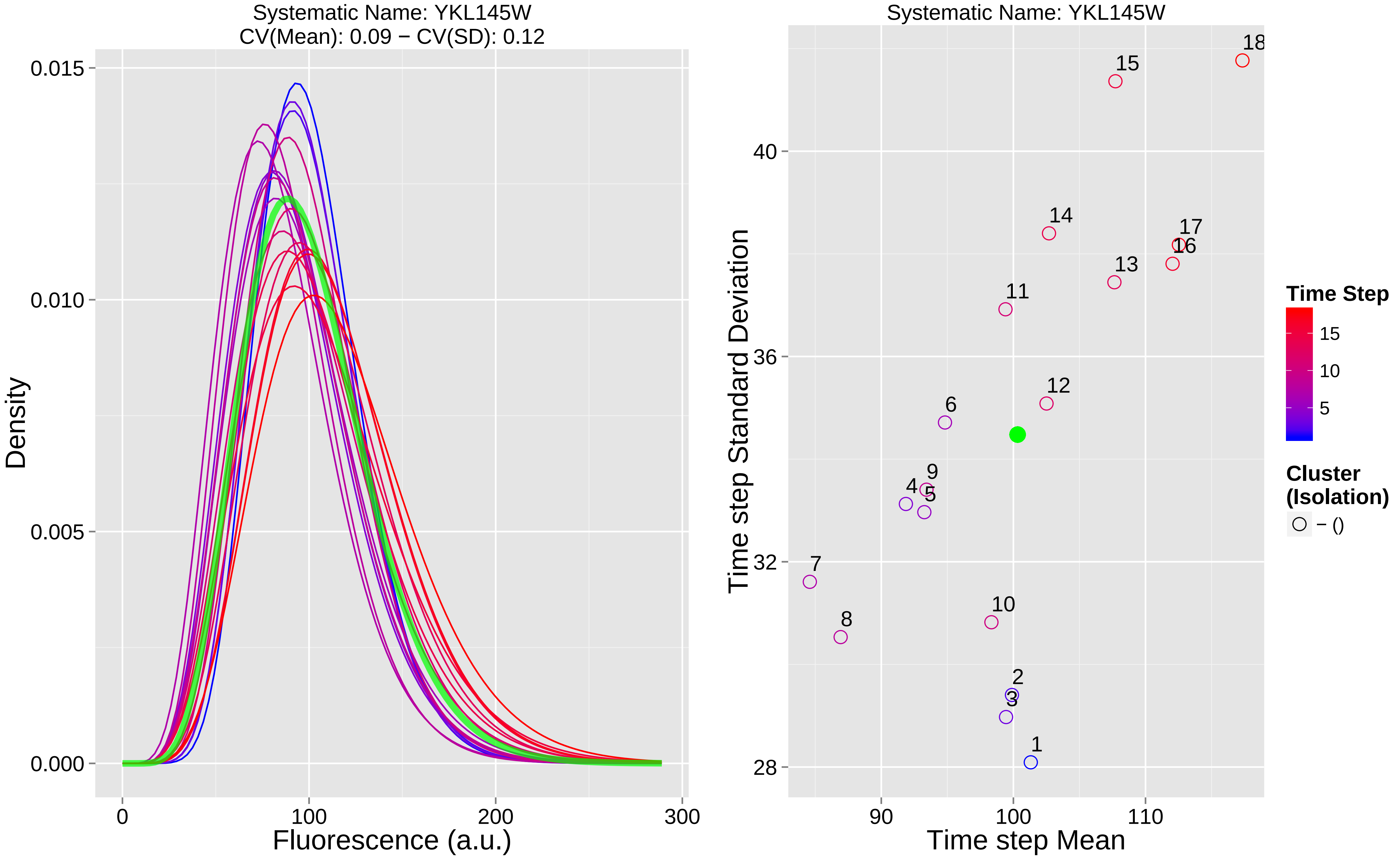

Systematic Name: YKL145W
CV(Mean): 0.09 − CV(SD): 0.12
Density
0.015
0.010
0.005
0.000
0
100
200
300
Fluorescence (a.u.)
Systematic Name: YKL145W
Time step Standard Deviation
40
36
32
28
90
100
110
Time step Mean
1
2
3
4
5
6
7
8
9
10
11
12
13
14
15
16
17
18
Time Step
15
10
5
Cluster
(Isolation)
− ()

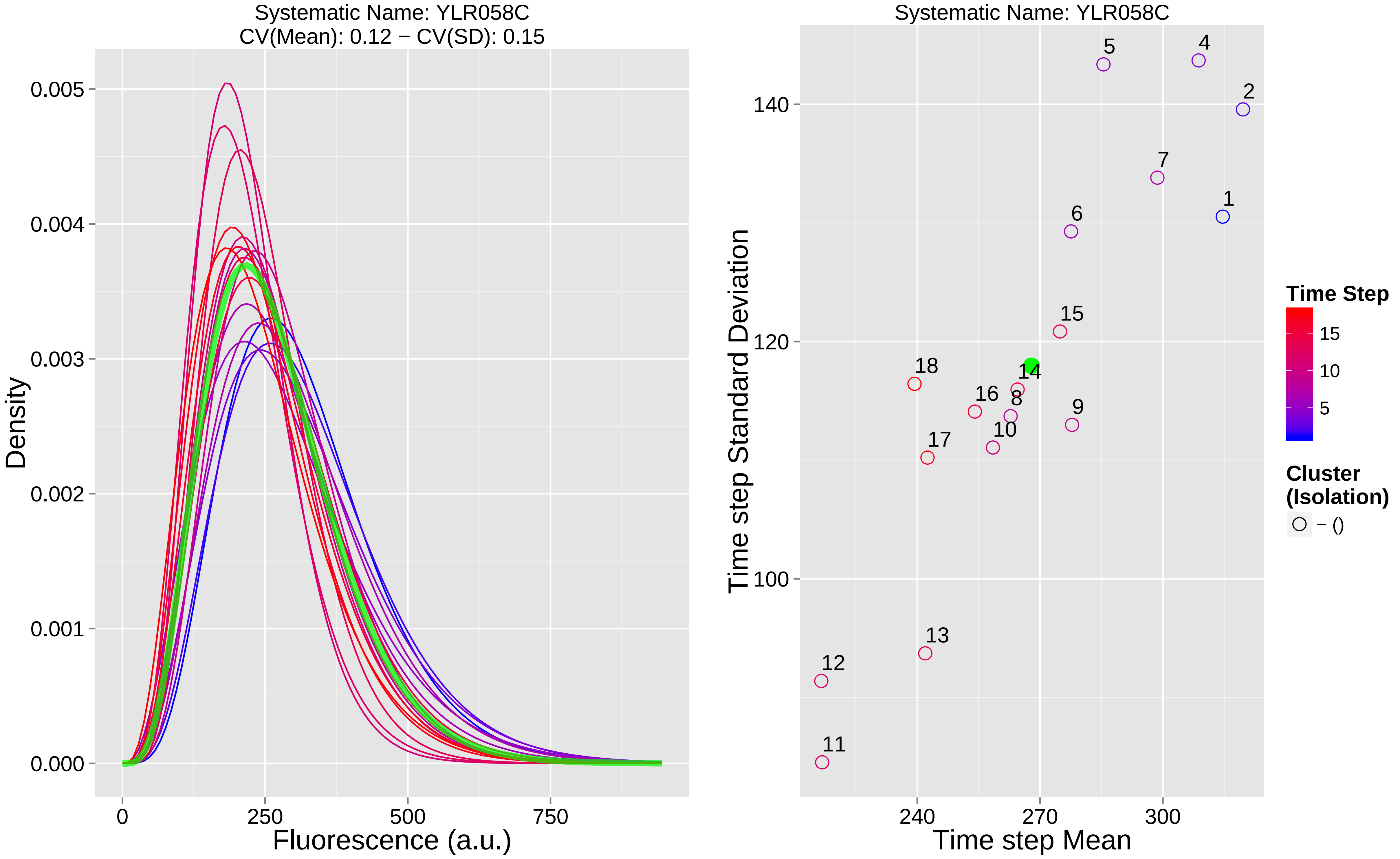

Systematic Name: YLR058C
CV(Mean): 0.12 − CV(SD): 0.15
Density
0.005
0.004
0.003
0.002
0.001
0.000
0
250
500
750
Fluorescence (a.u.)
Systematic Name: YLR058C
Time step Standard Deviation
140
120
100
240
270
300
Time step Mean
1
2
4
5
6
7
8
9
10
11
12
13
14
15
16
17
18
Time Step
15
10
5
Cluster
(Isolation)
− ()

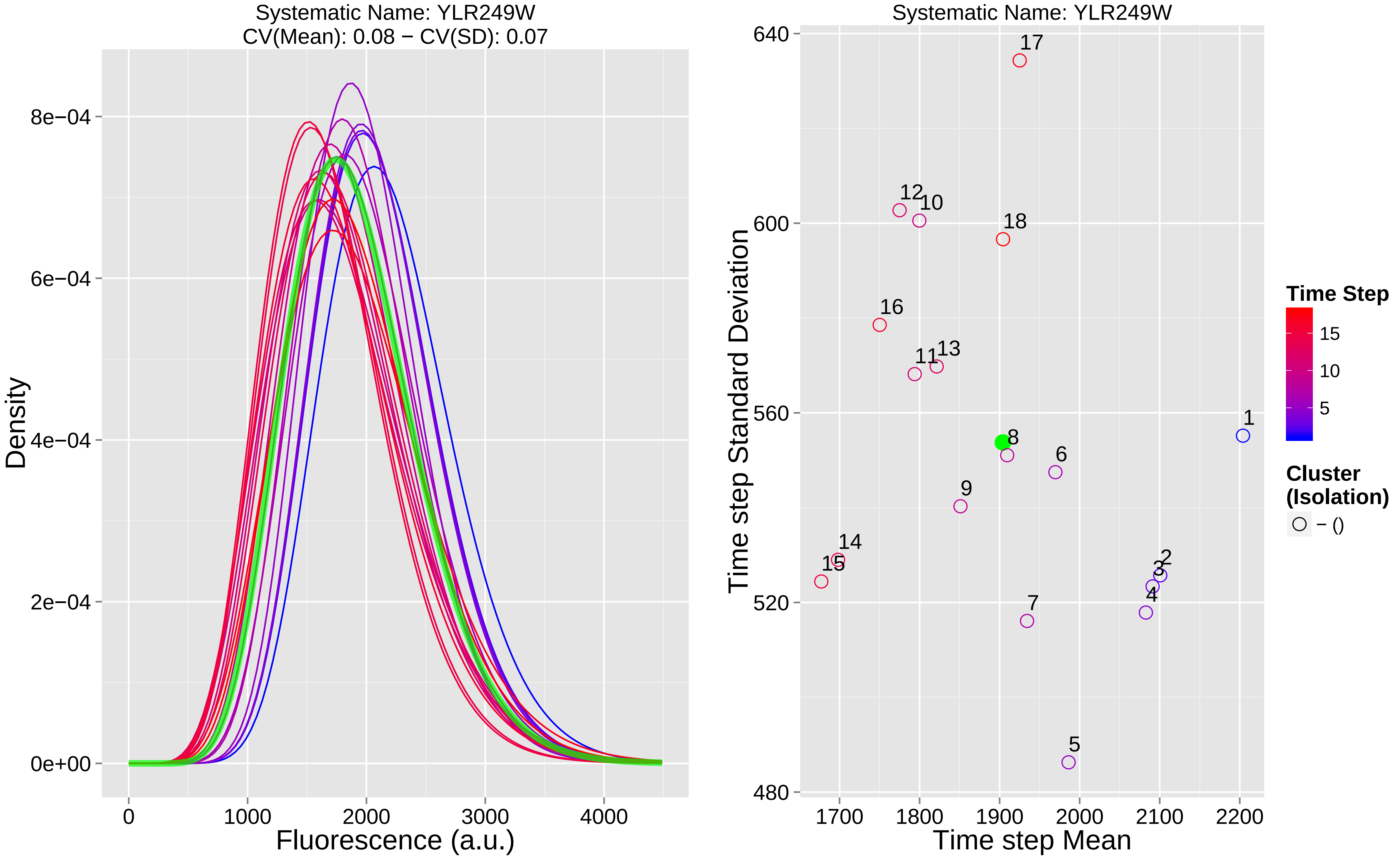

Systematic Name: YLR249W
CV(Mean): 0.08 − CV(SD): 0.07
Density
Fluorescence (a.u.)
8e−04
6e−04
4e−04
2e−04
0e+00
0
1000
2000
3000
4000
Systematic Name: YLR249W
Time step Standard Deviation
Time step Mean
640
600
560
520
480
1700
1800
1900
2000
2100
2200
Time Step
15
10
5
Cluster
(Isolation)
− ()
