# Supplementary figures and images for: Population FBA predicts metabolic phenotypes in yeast
Source: PLoS Comput Biol. 2017 Sep 8;13(9):e1005728. doi: 10.1371/journal.pcbi.1005728 (PMC5626512; doi:10.1371/journal.pcbi.1005728)

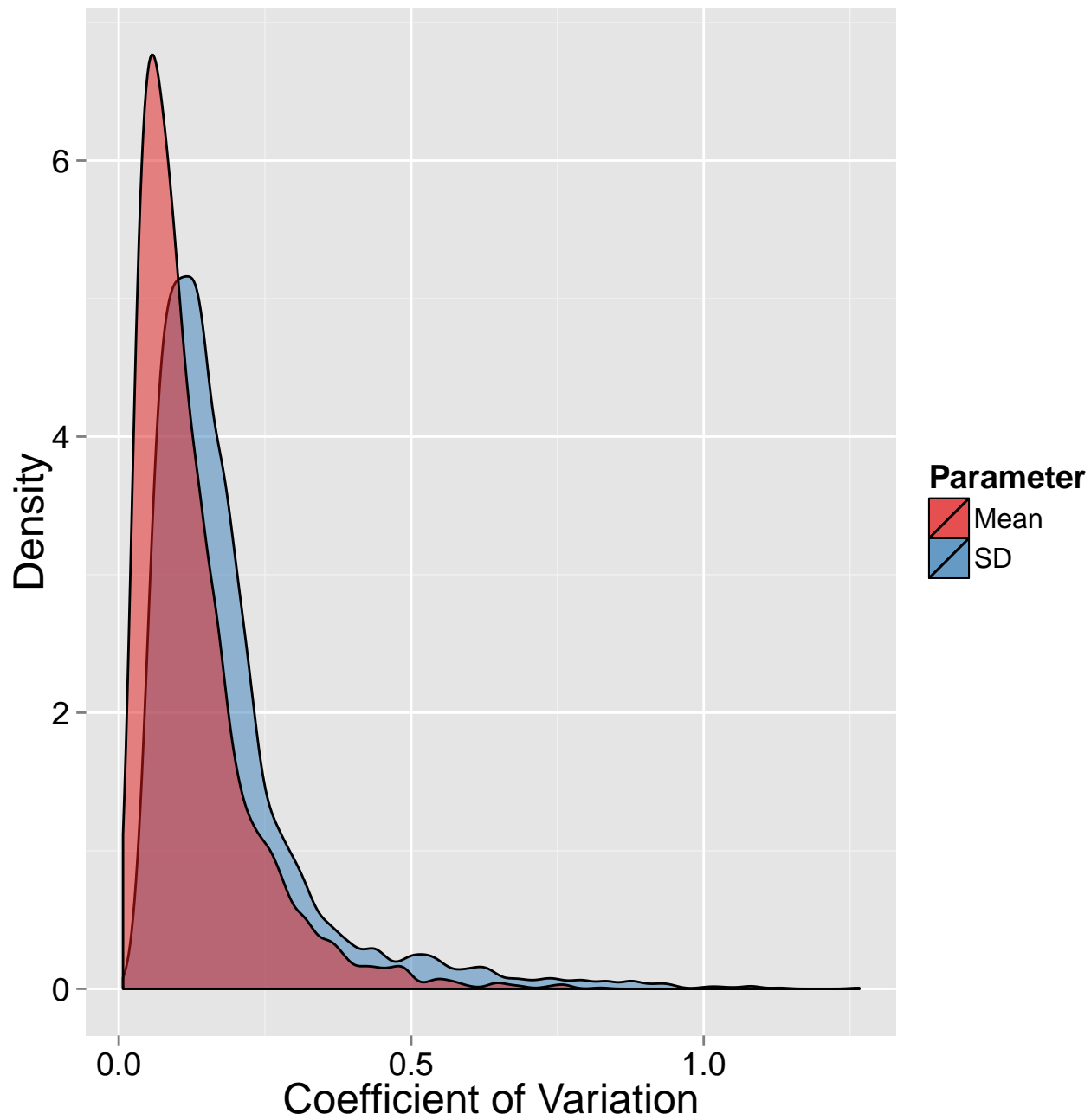

Supplement: S1 File — Model files contain all modifications to reflect gene deletions and media conditions. Parameter files with all shape and scale parameters for fluorescence distributions of proteins used in our simulations, along with their original kcat values, final kcat values after doubling procedure in both SD and 13C media as well as scaling ratios to convert sampled SD protein count to 13C count. There is also listing of final kcat values obtained after 10 independent GA optimizations in SD media. Correlation matrix imposed while sampling distributions as well as its Cholesky decomposed factor is also provided. (ZIP) [file pcbi.1005728.s002.zip › FluorescenceData/CV_MeanSD_AllProteins.pdf]
