# Supplementary material for: Population FBA predicts metabolic phenotypes in yeast
Source: PLoS Comput Biol. 2017 Sep 8;13(9):e1005728. doi: 10.1371/journal.pcbi.1005728 (PMC5626512; doi:10.1371/journal.pcbi.1005728)
Supplement: S1 File — Model files contain all modifications to reflect gene deletions and media conditions. Parameter files with all shape and scale parameters for fluorescence distributions of proteins used in our simulations, along with their original kcat values, final kcat values after doubling procedure in both SD and 13C media as well as scaling ratios to convert sampled SD protein count to 13C count. There is also listing of final kcat values obtained after 10 independent GA optimizations in SD media. Correlation matrix imposed while sampling distributions as well as its Cholesky decomposed factor is also provided. (ZIP) [file CombinedPlots_Distributions_MeanSD_CVs_Clusters_ActiveGenes.pdf › FluorescenceData/CombinedPlots_Distributions_MeanSD_CVs_Clusters_ActiveGenes.pdf]

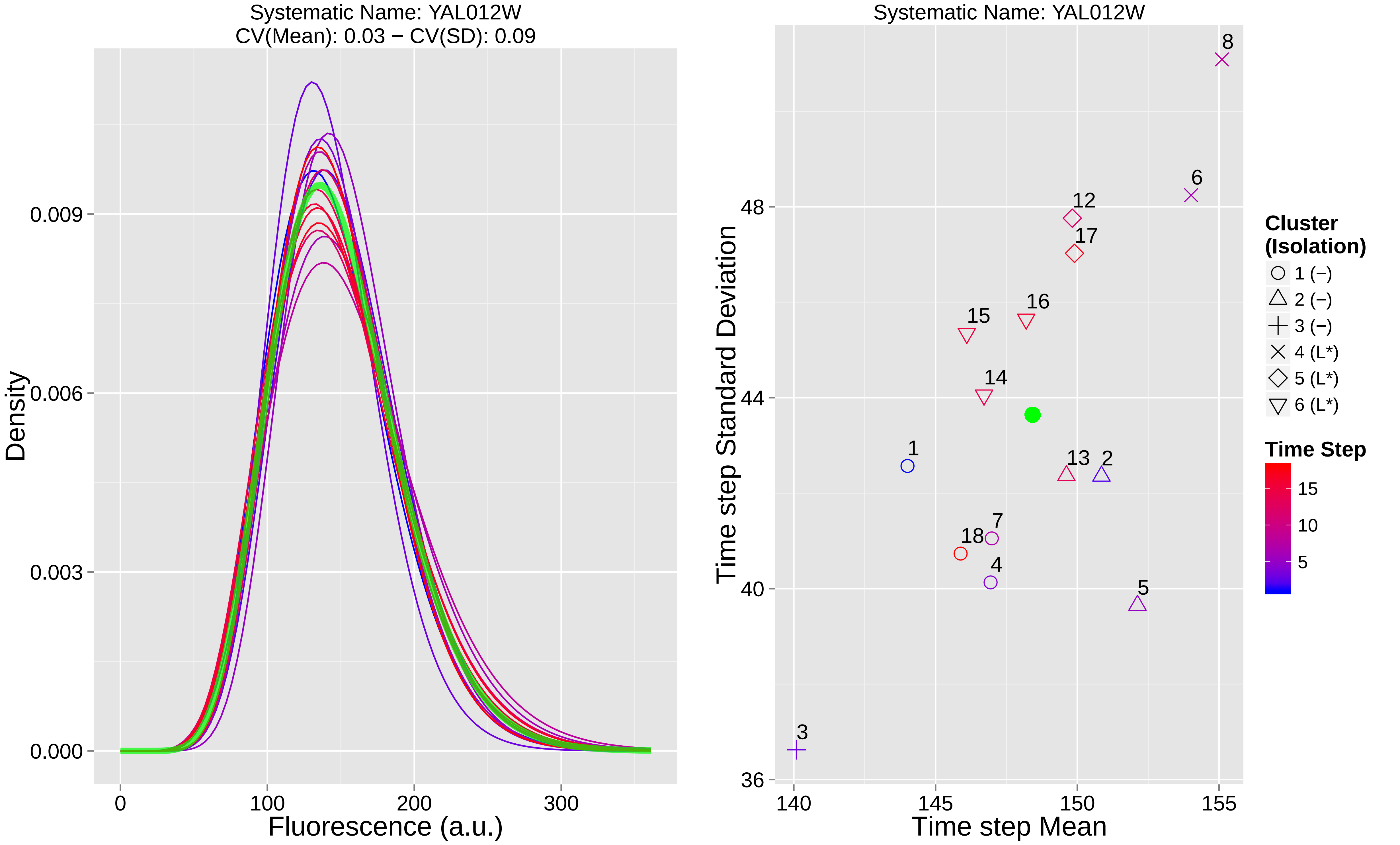

Systematic Name: YAL012W
CV(Mean): 0.03 − CV(SD): 0.09
Density
0.009
0.006
0.003
0.000
0
100
200
300
Fluorescence (a.u.)
Systematic Name: YAL012W
Time step Standard Deviation
48
44
40
36
140
145
150
155
Time step Mean
8
6
12
17
16
15
14
1
13
2
7
18
4
5
3
Cluster
(Isolation)
1 (−)
2 (−)
3 (−)
4 (L*)
5 (L*)
6 (L*)
Time Step
15
10
5

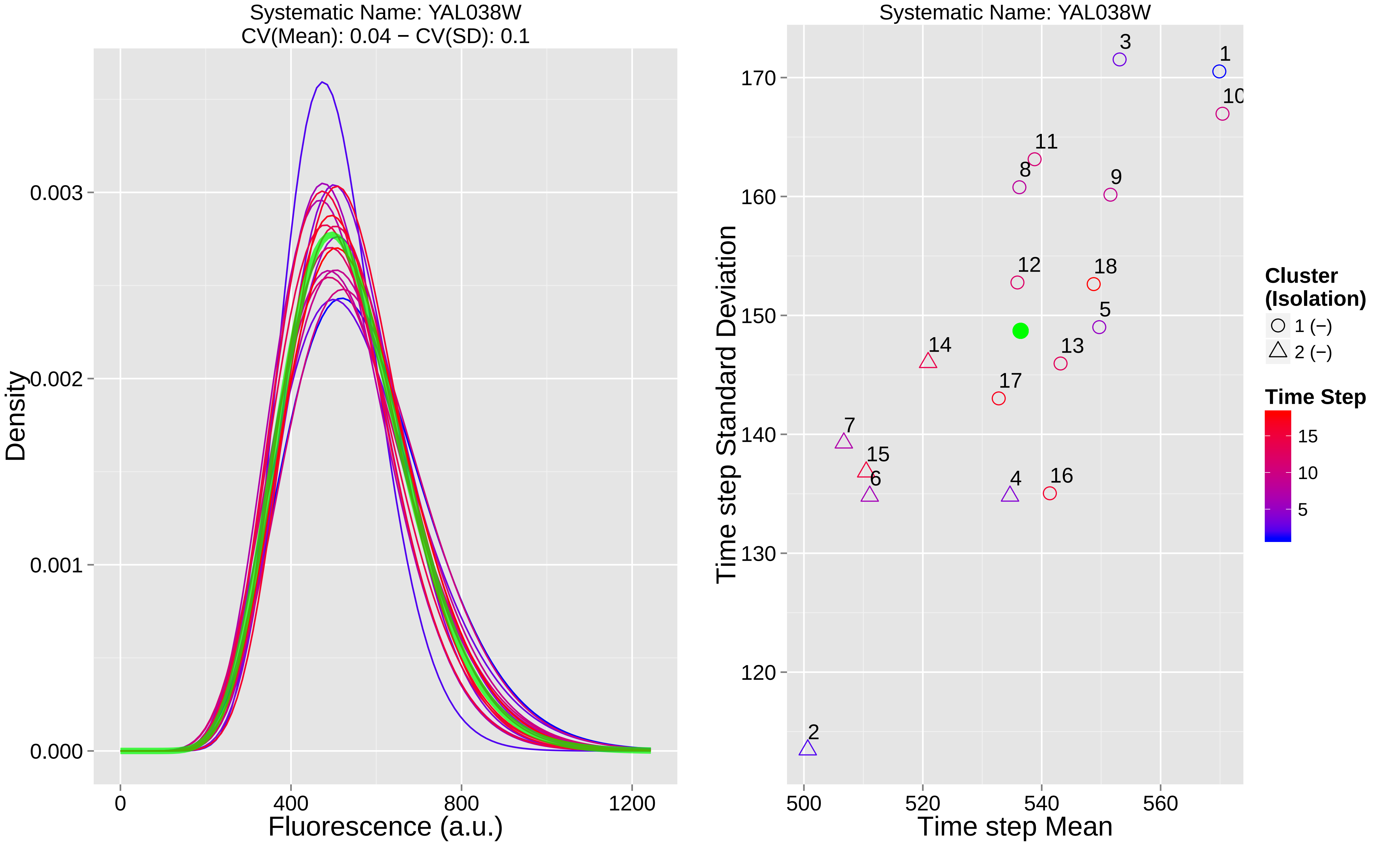
Systematic Name: YAL038W
CV(Mean): 0.04 − CV(SD): 0.1
Density
0.000
0.001
0.002
0.003
0
400
800
1200
Fluorescence (a.u.)
Systematic Name: YAL038W
Time step Standard Deviation
120
130
140
150
160
170
500
520
540
560
Time step Mean
1
2
3
4
5
6
7
8
9
10
11
12
13
14
15
16
17
18
Cluster
(Isolation)
1 (−)
2 (−)
Time Step
15
10
5

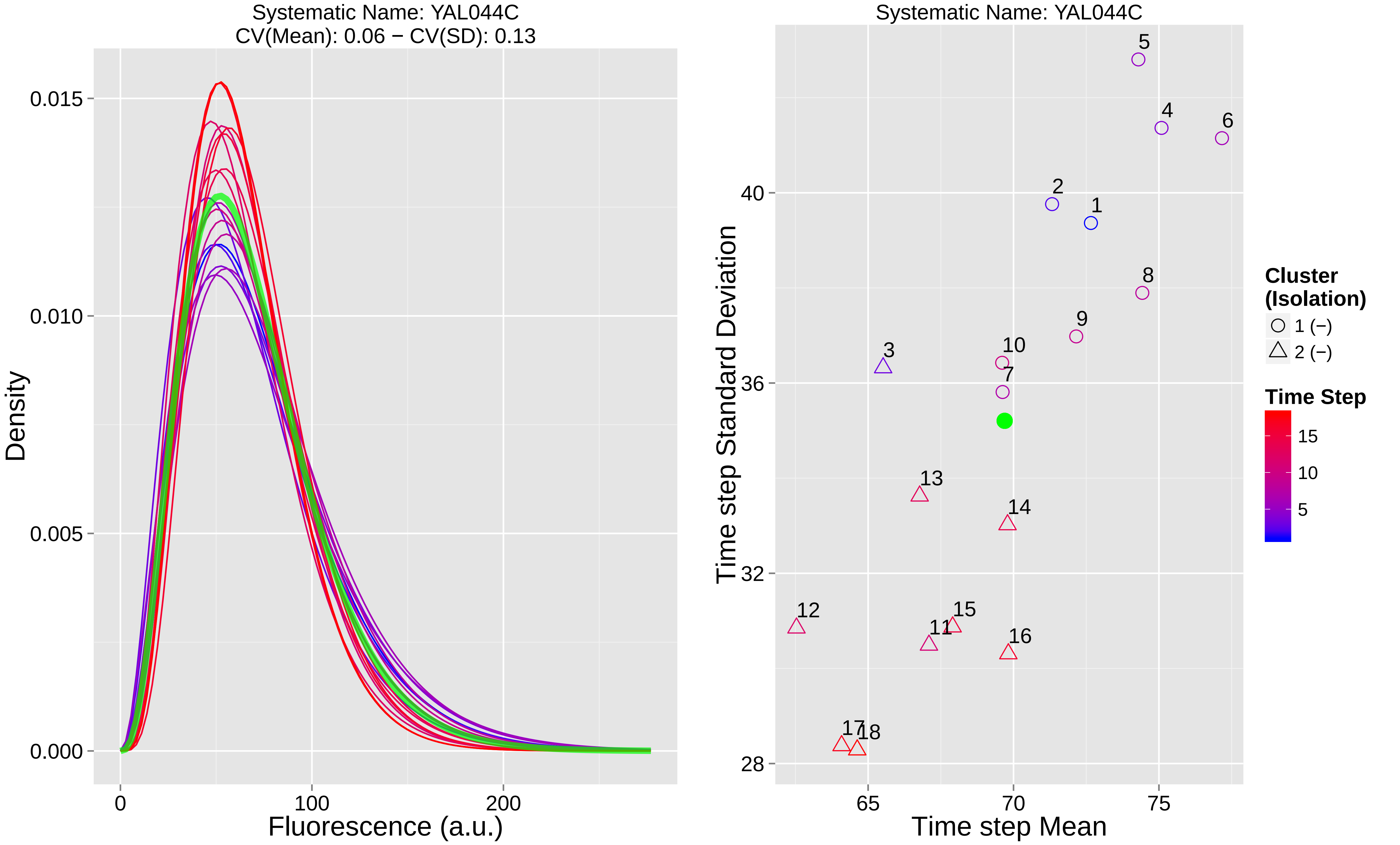

Systematic Name: YAL044C
CV(Mean): 0.06 − CV(SD): 0.13
Density
0.015
0.010
0.005
0.000
0
100
200
Fluorescence (a.u.)
Systematic Name: YAL044C
Time step Standard Deviation
40
36
32
28
65
70
75
Time step Mean
1
2
3
4
5
6
7
8
9
10
11
12
13
14
15
16
17
18
Cluster (Isolation)
1 (−)
2 (−)
Time Step
15
10
5

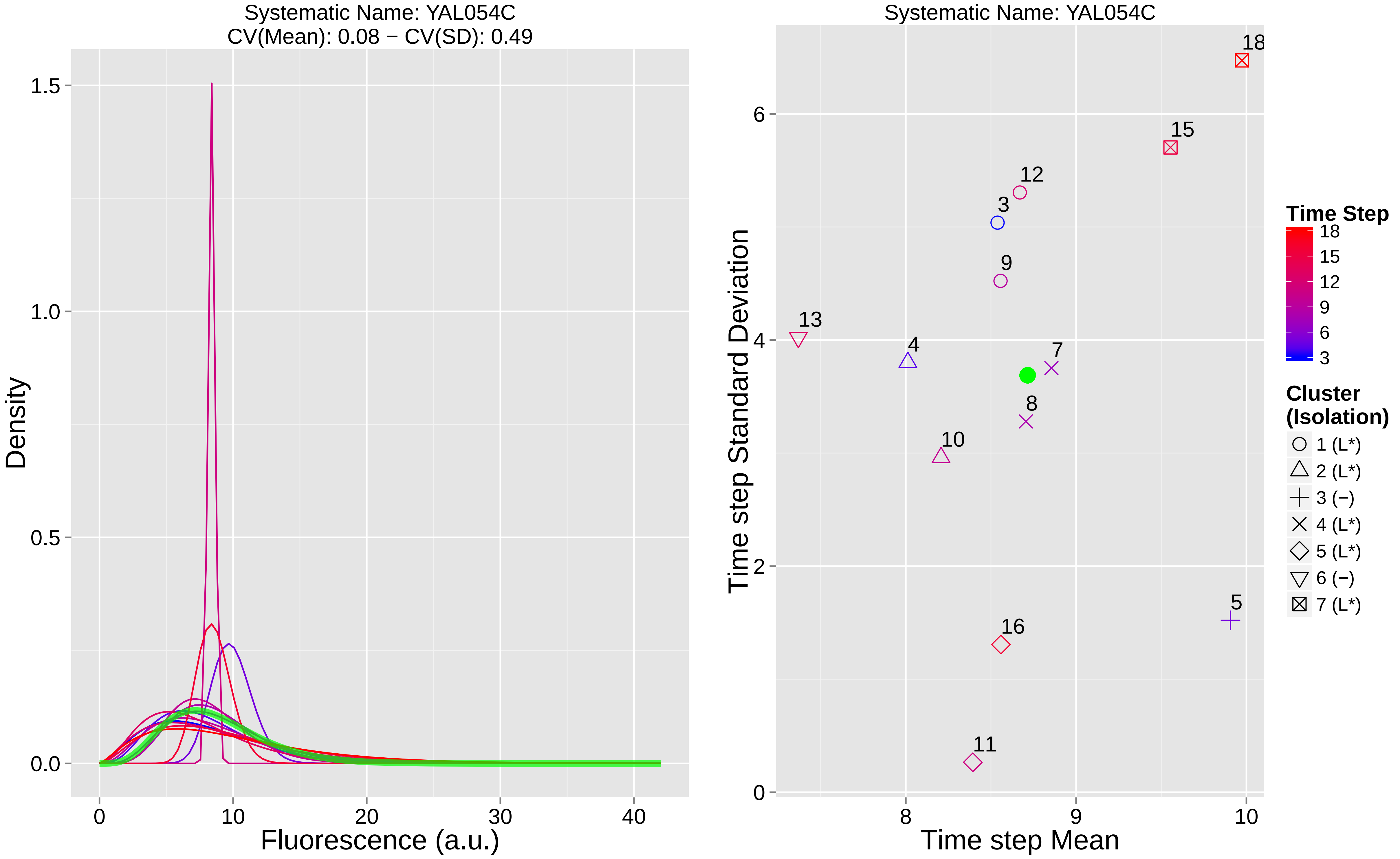

Systematic Name: YAL054C
CV(Mean): 0.08 − CV(SD): 0.49
Density
Fluorescence (a.u.)
0.0
0.5
1.0
1.5
0
10
20
30
40
Systematic Name: YAL054C
Time step Standard Deviation
Time step Mean
0
2
4
6
8
9
10
18
15
12
3
9
13
4
7
8
10
5
16
11
Time Step
18
15
12
9
6
3
Cluster (Isolation)
1 (L*)
2 (L*)
3 (−)
4 (L*)
5 (L*)
6 (−)
7 (L*)

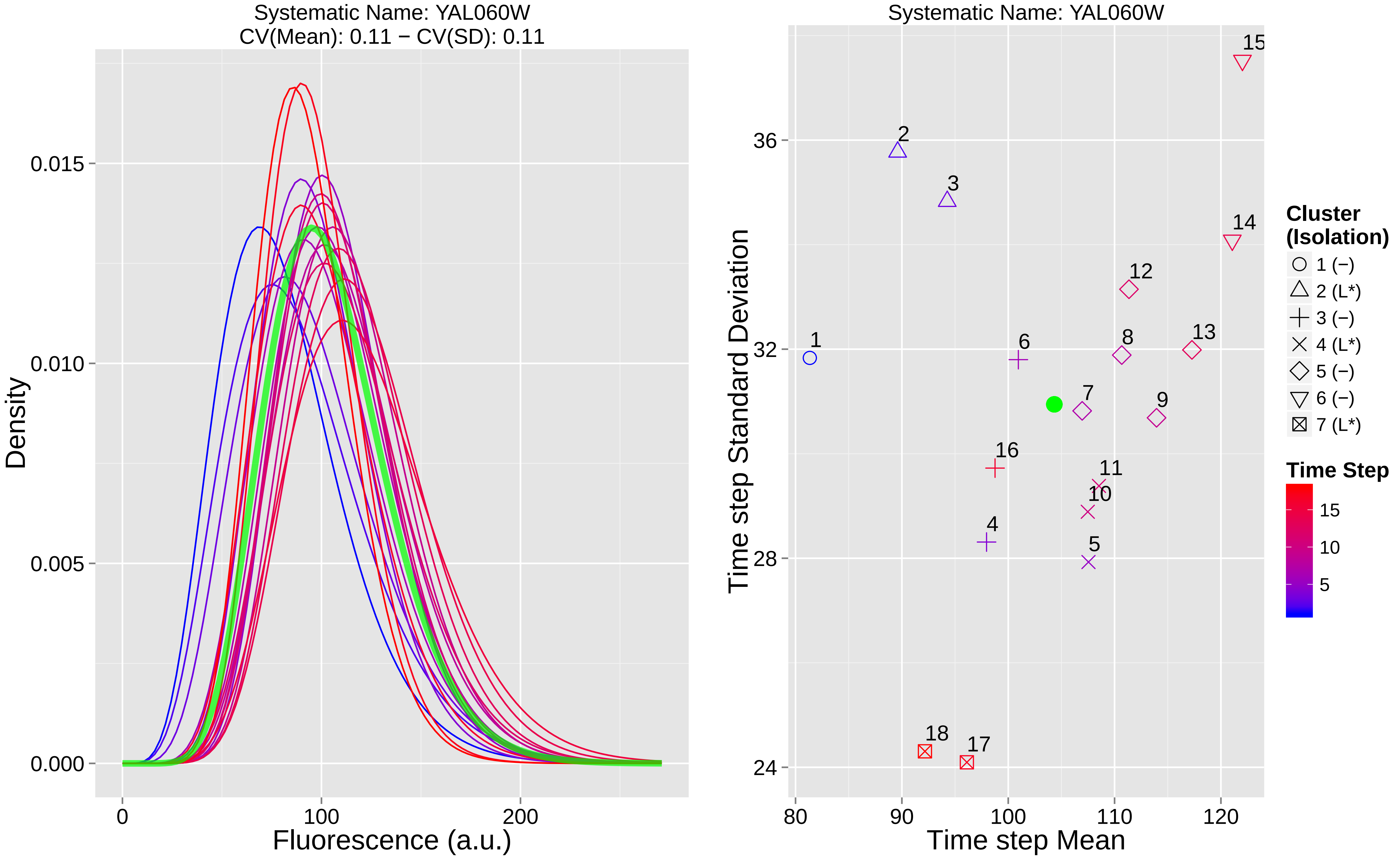

Systematic Name: YAL060W
CV(Mean): 0.11 − CV(SD): 0.11
Density
Fluorescence (a.u.)
0.000
0.005
0.010
0.015
0
100
200
Systematic Name: YAL060W
Time step Standard Deviation
Time step Mean
24
28
32
36
80
90
100
110
120
Cluster (Isolation)
1 (−)
2 (L*)
3 (−)
4 (L*)
5 (−)
6 (−)
7 (L*)
Time Step
5
10
15

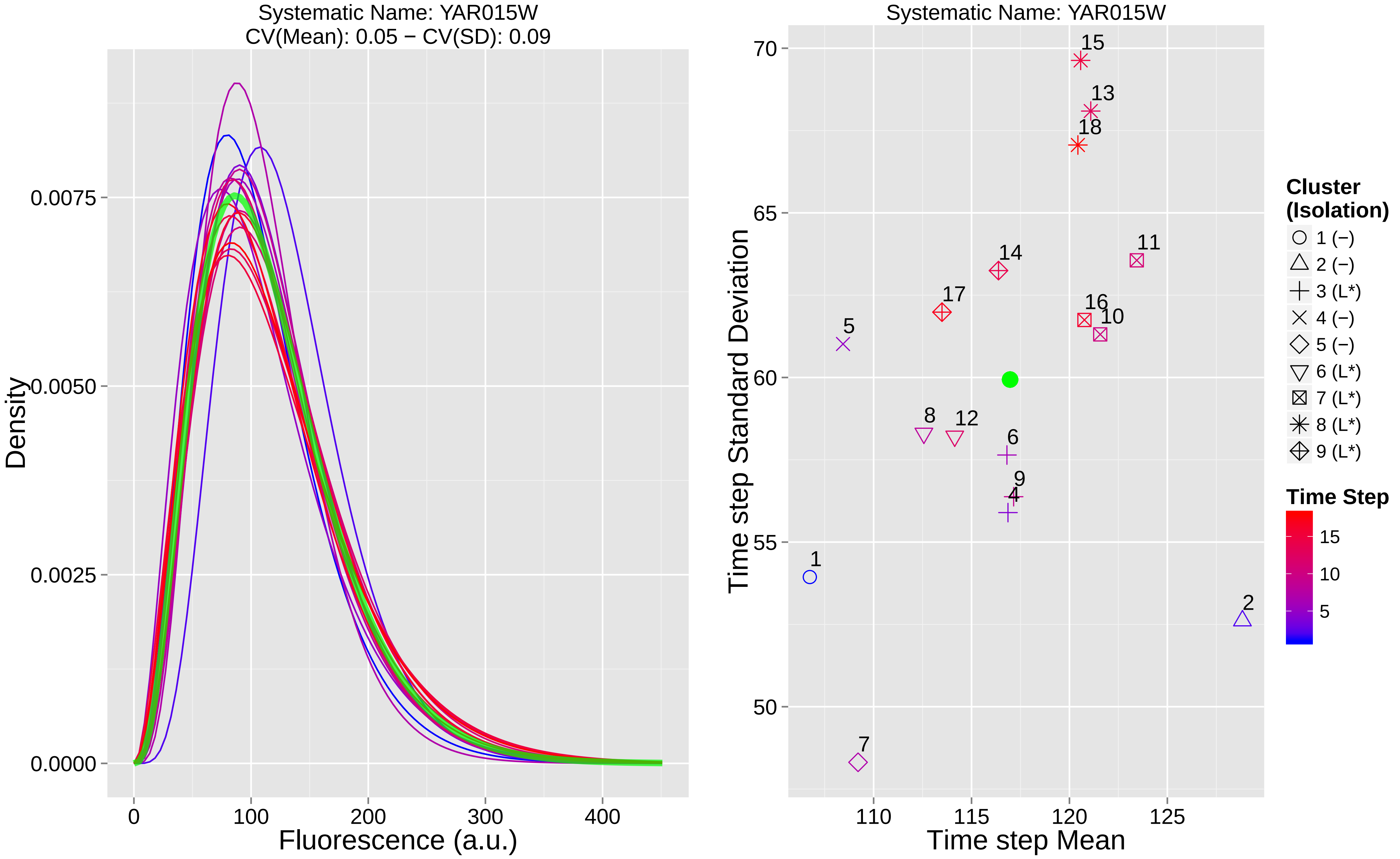

Systematic Name: YAR015W
CV(Mean): 0.05 − CV(SD): 0.09
Density
0.0075
0.0050
0.0025
0.0000
0
100
200
300
400
Fluorescence (a.u.)
Systematic Name: YAR015W
Time step Standard Deviation
70
65
60
55
50
110
115
120
125
Time step Mean
15
13
18
14
11
17
16
10
5
8
12
6
9
4
1
2
7
Cluster (Isolation)
1 (−)
2 (−)
3 (L*)
4 (−)
5 (−)
6 (L*)
7 (L*)
8 (L*)
9 (L*)
Time Step
15
10
5

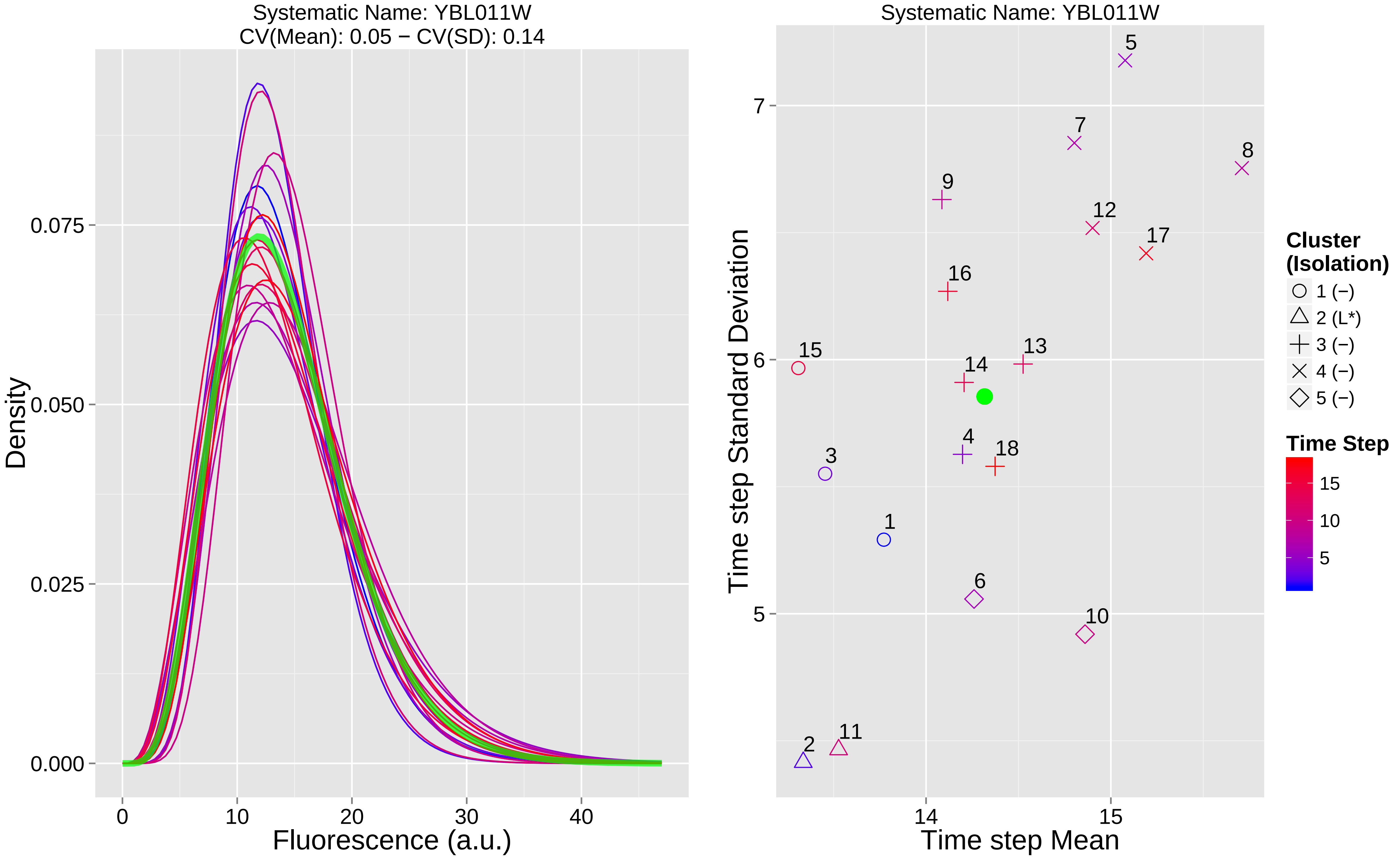

Systematic Name: YBL011W
CV(Mean): 0.05 − CV(SD): 0.14
Density
Fluorescence (a.u.)
0.000
0.025
0.050
0.075
0
10
20
30
40
Systematic Name: YBL011W
Time step Standard Deviation
Time step Mean
5
6
7
14
15
1
2
3
4
5
6
7
8
9
10
11
12
13
14
15
16
17
18
Cluster
(Isolation)
1 (−)
2 (L*)
3 (−)
4 (−)
5 (−)
Time Step
15
10
5

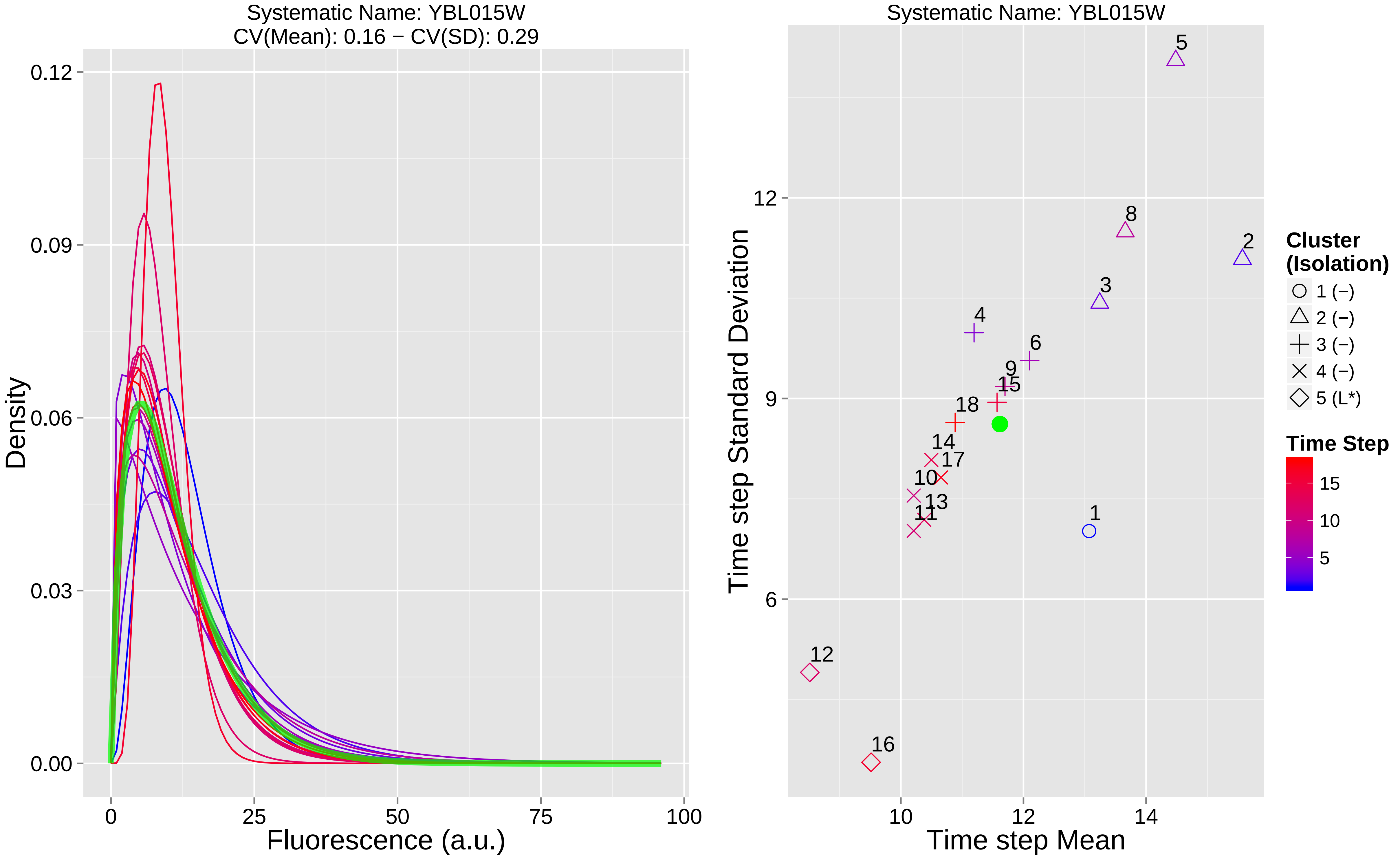

Systematic Name: YBL015W
CV(Mean): 0.16 − CV(SD): 0.29
Density
0.12
0.09
0.06
0.03
0.00
0
25
50
75
100
Fluorescence (a.u.)
Systematic Name: YBL015W
Time step Standard Deviation
12
9
6
10
12
14
Time step Mean
5
8
2
3
4
6
9
15
18
14
17
10
13
11
1
12
16
Cluster
(Isolation)
1 (−)
2 (−)
3 (−)
4 (−)
5 (L*)
Time Step
15
10
5

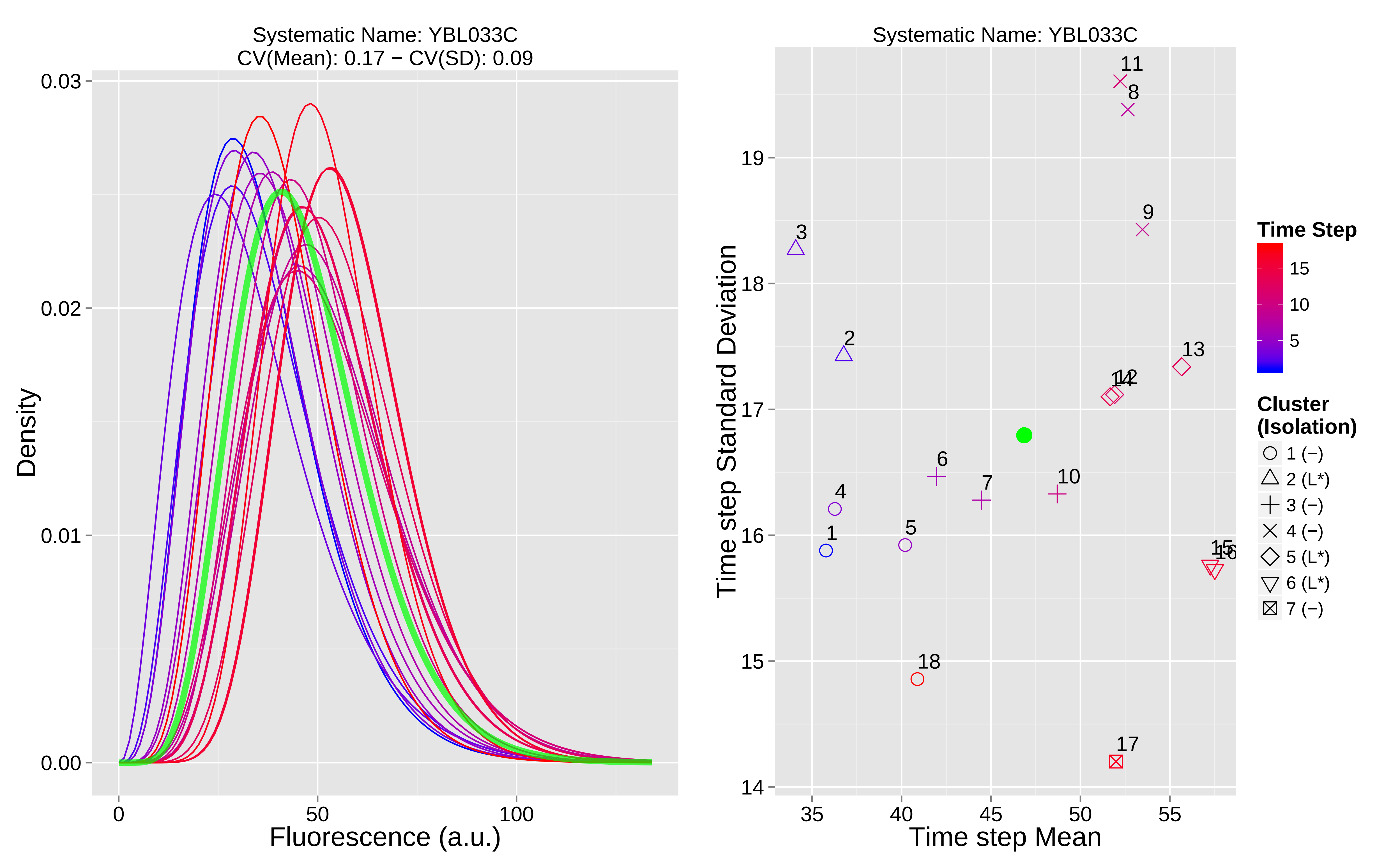

Systematic Name: YBL033C
CV(Mean): 0.17 − CV(SD): 0.09
Density
Fluorescence (a.u.)
0.03
0.02
0.01
0.00
0
50
100
Systematic Name: YBL033C
Time step Standard Deviation
Time step Mean
19
18
17
16
15
14
35
40
45
50
55
1
2
3
4
5
6
7
8
9
10
11
12
13
14
15
16
17
18
Time Step
15
10
5
Cluster (Isolation)
1 (−)
2 (L*)
3 (−)
4 (−)
5 (L*)
6 (L*)
7 (−)

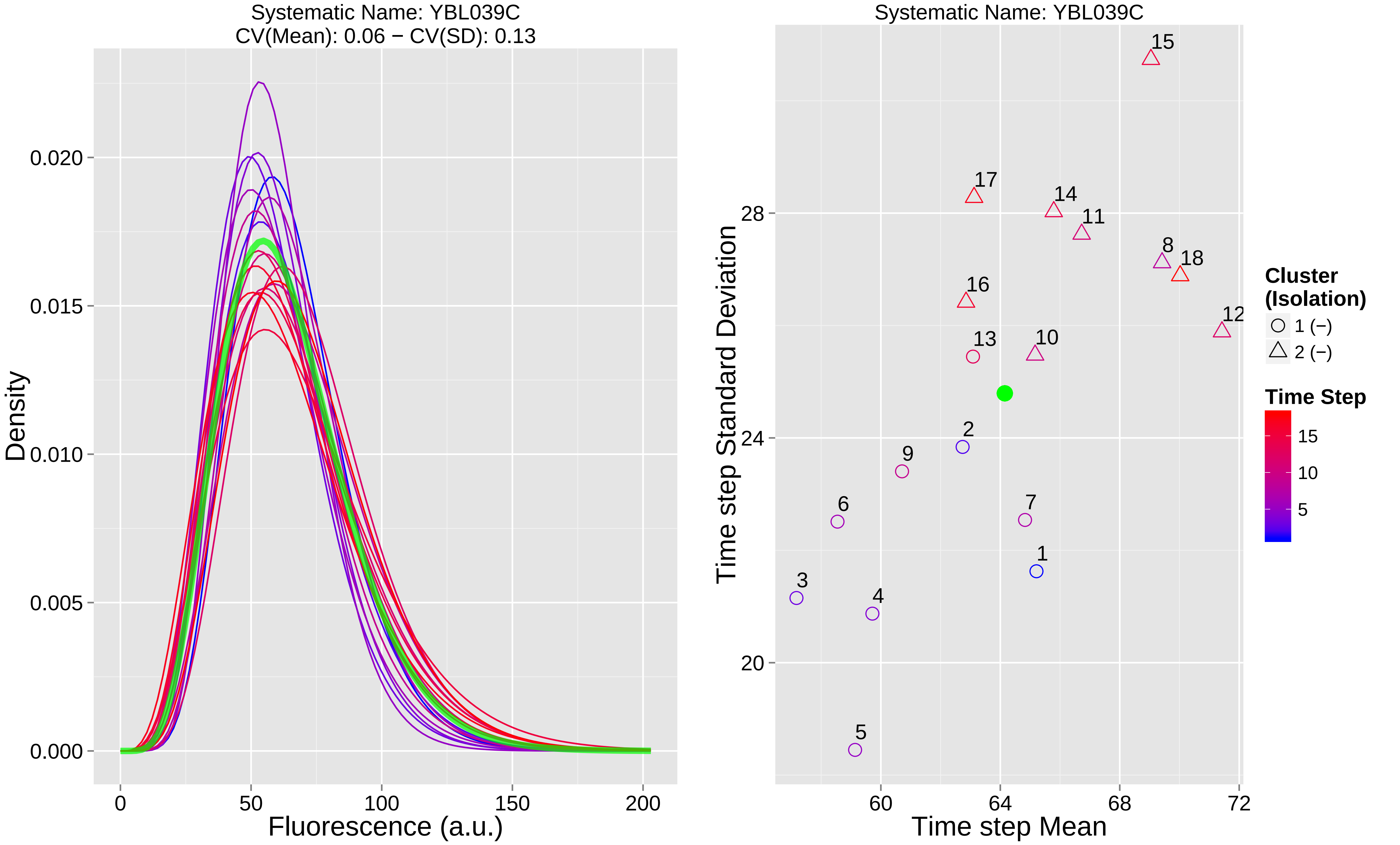
Systematic Name: YBL039C
CV(Mean): 0.06 − CV(SD): 0.13
Density
Fluorescence (a.u.)
0.000
0.005
0.010
0.015
0.020
0
50
100
150
200
Systematic Name: YBL039C
Time step Standard Deviation
Time step Mean
20
24
28
60
64
68
72
1
2
3
4
5
6
7
8
9
10
11
12
13
14
15
16
17
18
Cluster
(Isolation)
1 (−)
2 (−)
Time Step
5
10
15

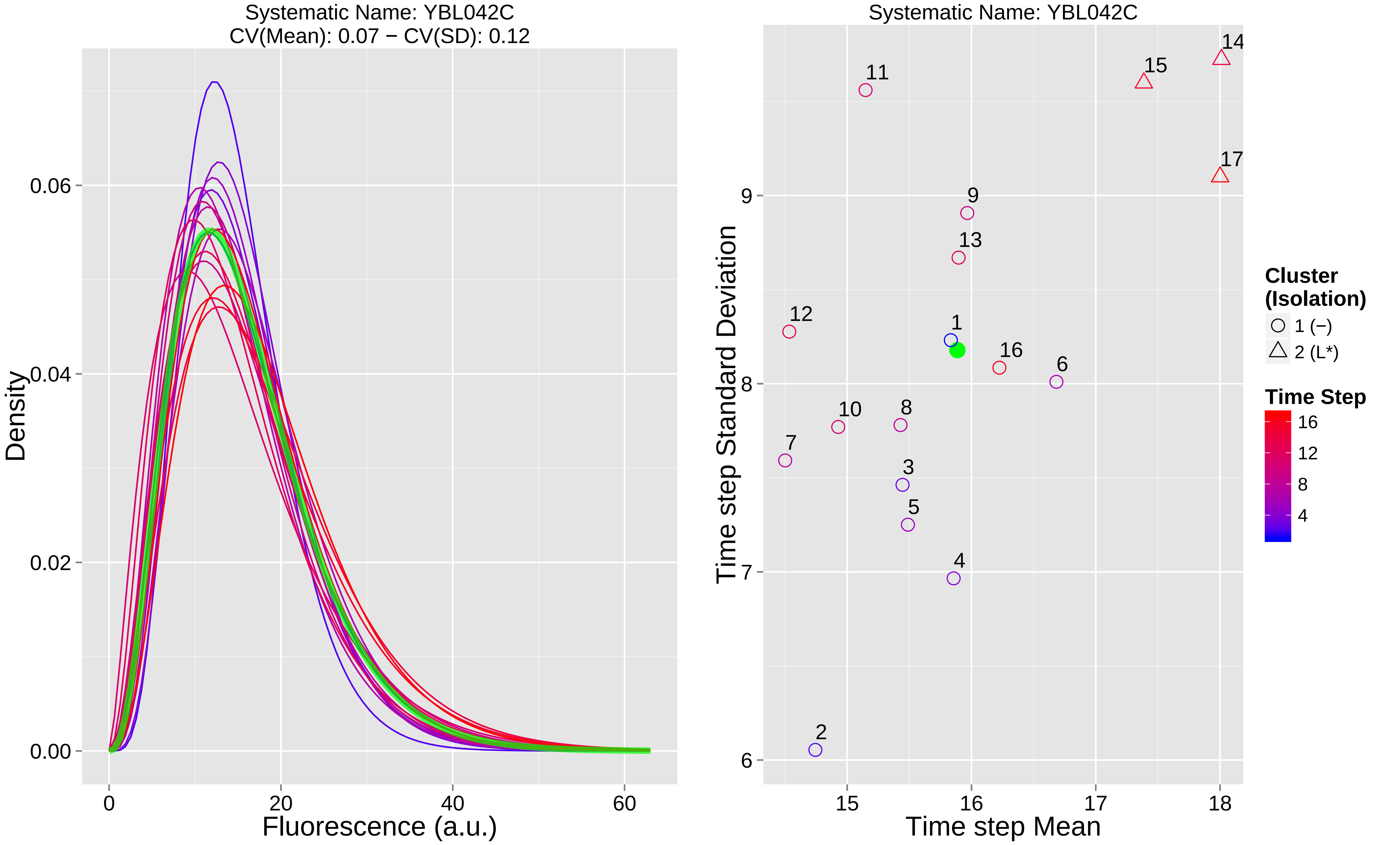

Systematic Name: YBL042C
CV(Mean): 0.07 − CV(SD): 0.12
Density
Fluorescence (a.u.)
0.00
0.02
0.04
0.06
0
20
40
60
Systematic Name: YBL042C
Time step Standard Deviation
Time step Mean
6
7
8
9
15
16
17
18
1
2
3
4
5
6
7
8
9
10
11
12
13
14
15
16
17
Cluster (Isolation)
1 (−)
2 (L*)
Time Step
4
8
12
16

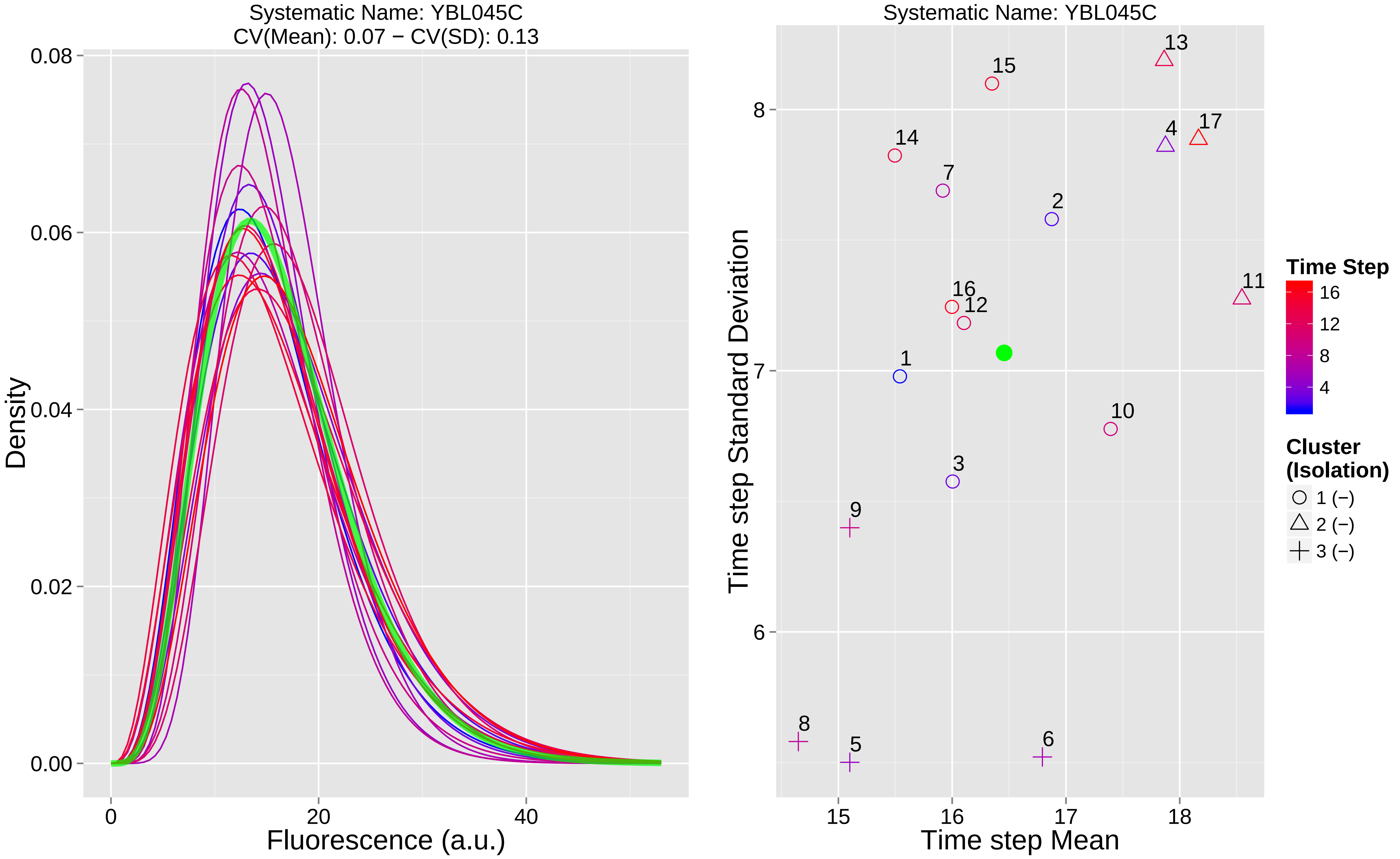

Systematic Name: YBL045C
CV(Mean): 0.07 − CV(SD): 0.13
Density
Fluorescence (a.u.)
0.00
0.02
0.04
0.06
0.08
0
20
40
Systematic Name: YBL045C
Time step Standard Deviation
Time step Mean
15
16
17
18
6
7
8
1
2
3
4
5
6
7
8
9
10
11
12
13
14
15
16
17
Time Step
16
12
8
4
Cluster (Isolation)
1 (−)
2 (−)
3 (−)

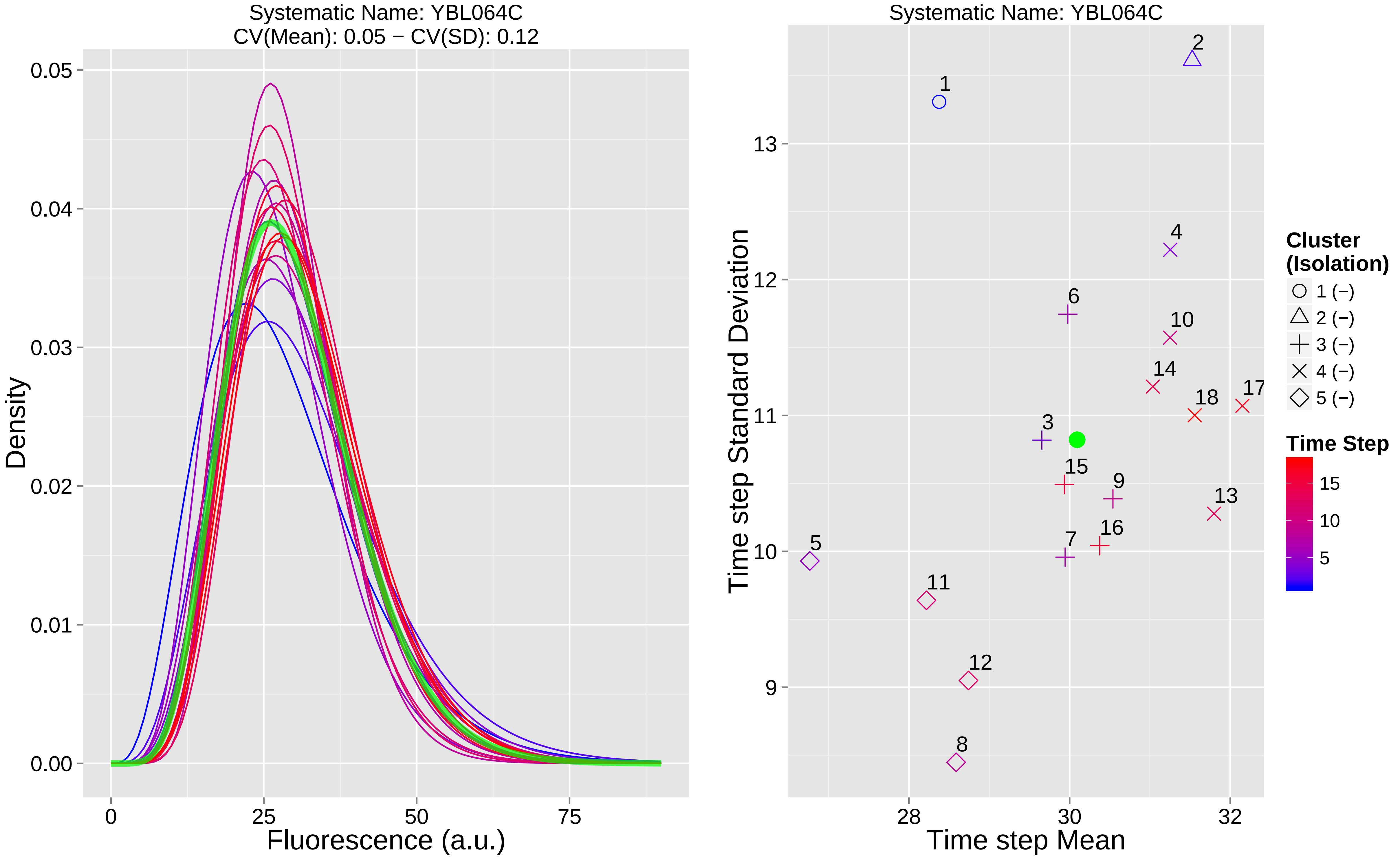

Systematic Name: YBL064C
CV(Mean): 0.05 − CV(SD): 0.12
Density
Fluorescence (a.u.)
0.00
0.01
0.02
0.03
0.04
0.05
0
25
50
75
Systematic Name: YBL064C
Time step Standard Deviation
Time step Mean
9
10
11
12
13
28
30
32
1
2
3
4
5
6
7
8
9
10
11
12
13
14
15
16
17
18
Cluster (Isolation)
1 (−)
2 (−)
3 (−)
4 (−)
5 (−)
Time Step
5
10
15

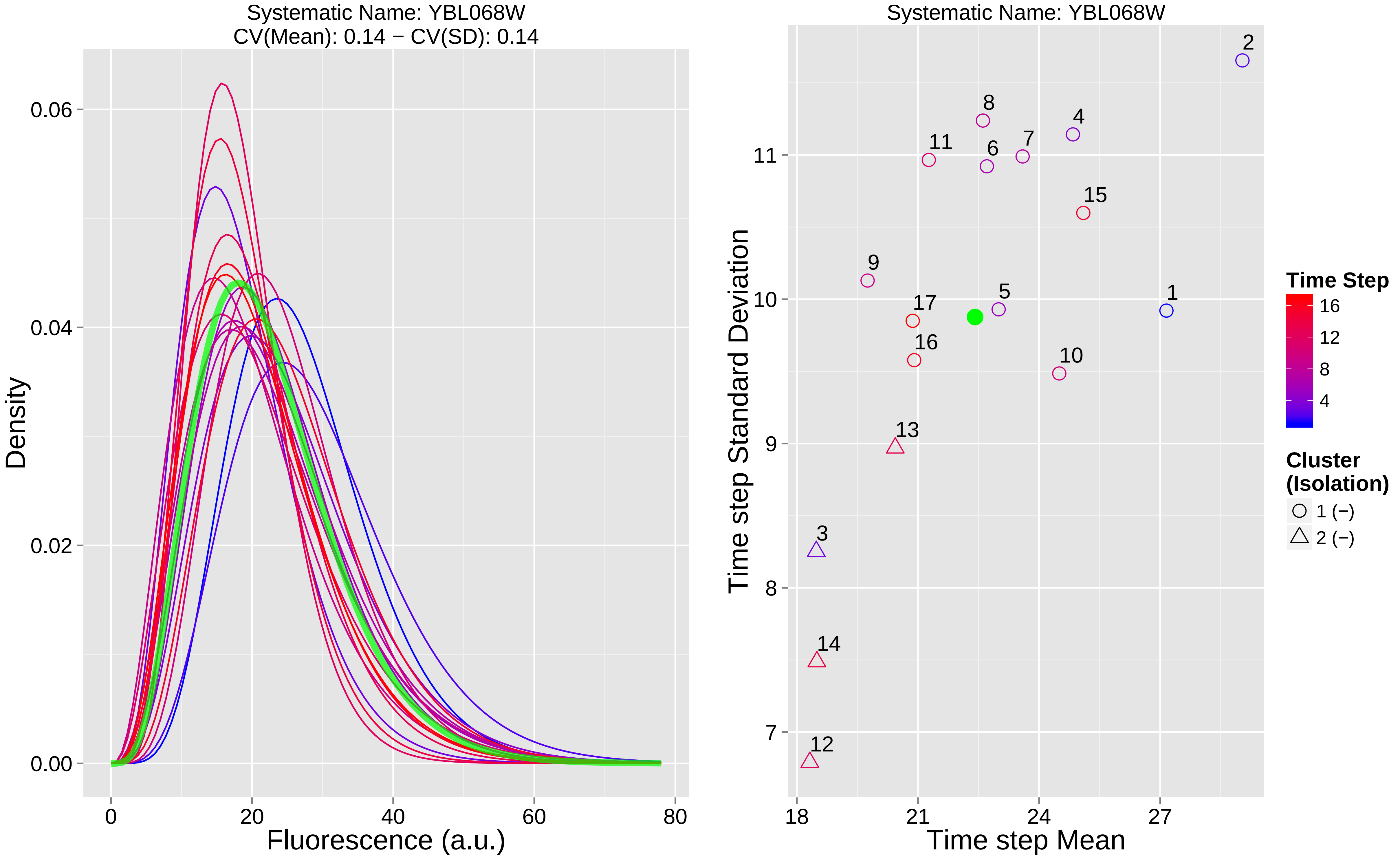

Systematic Name: YBL068W
CV(Mean): 0.14 − CV(SD): 0.14
Density
0.06
0.04
0.02
0.00
0
20
40
60
80
Fluorescence (a.u.)
Systematic Name: YBL068W
Time step Standard Deviation
11
10
9
8
7
18
21
24
27
Time step Mean
Time Step
16
12
8
4
Cluster (Isolation)
1 (−)
2 (−)

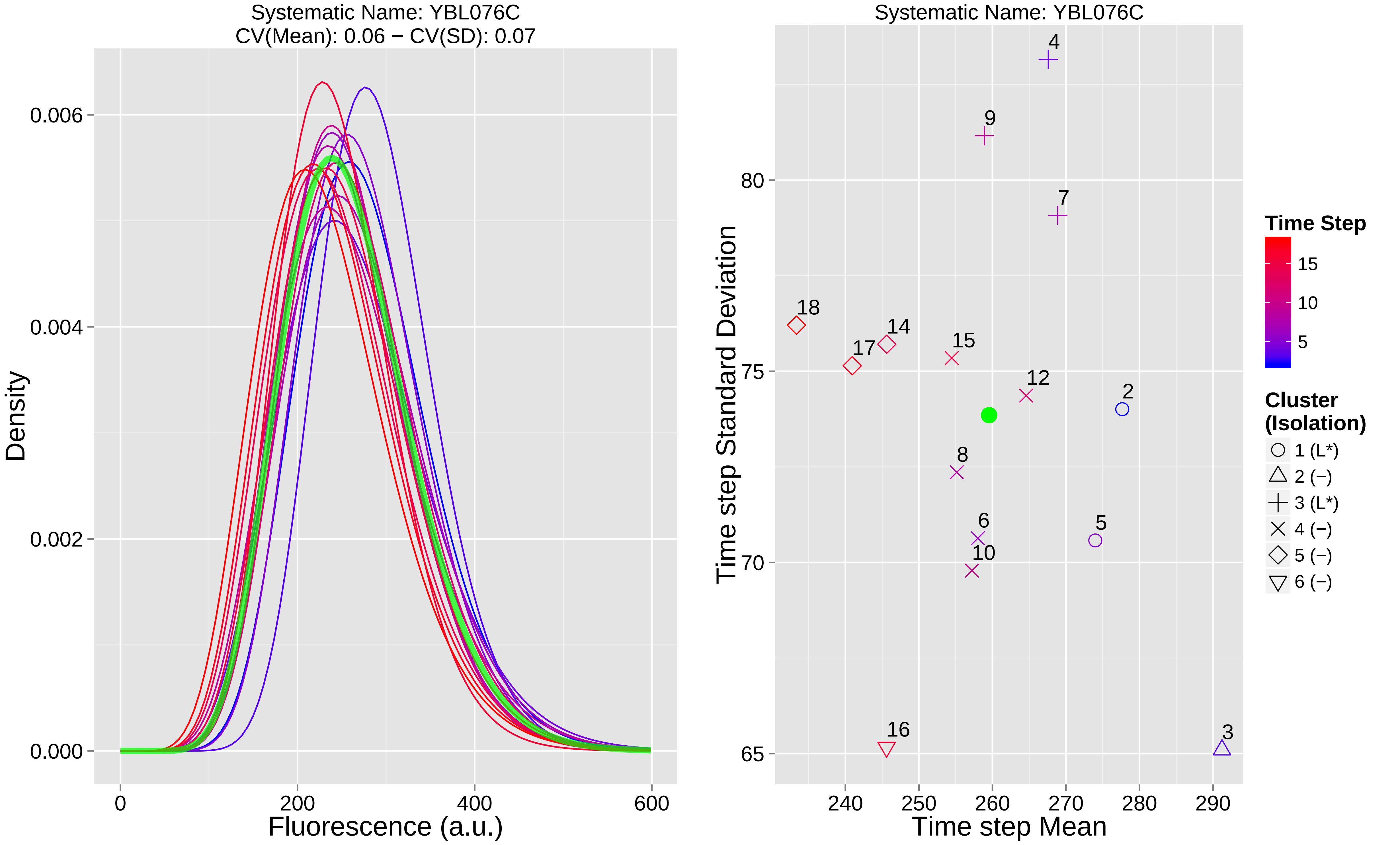

Systematic Name: YBL076C
CV(Mean): 0.06 − CV(SD): 0.07
Density
0.000
0.002
0.004
0.006
0
200
400
600
Fluorescence (a.u.)
Systematic Name: YBL076C
Time step Standard Deviation
65
70
75
80
240
250
260
270
280
290
Time step Mean
4
9
7
18
14
17
15
12
2
8
6
10
5
16
3
Time Step
15
10
5
Cluster
(Isolation)
1 (L*)
2 (−)
3 (L*)
4 (−)
5 (−)
6 (−)

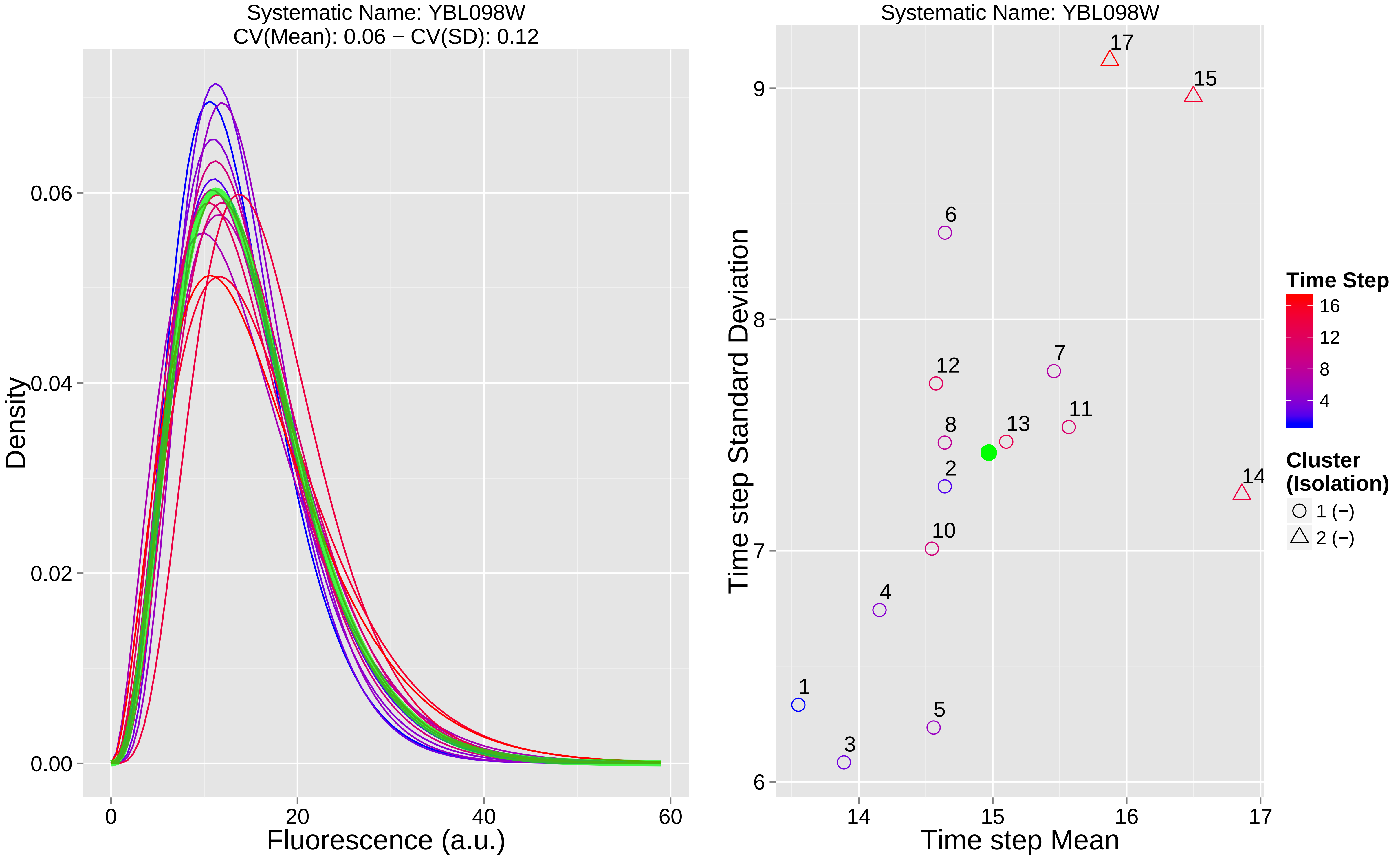

Systematic Name: YBL098W
CV(Mean): 0.06 − CV(SD): 0.12
Density
0.00
0.02
0.04
0.06
0
20
40
60
Fluorescence (a.u.)
Systematic Name: YBL098W
Time step Standard Deviation
Time step Mean
6
7
8
9
14
15
16
17
1
2
3
4
5
6
7
8
10
11
12
13
14
15
17
Time Step
16
12
8
4
Cluster (Isolation)
1 (−)
2 (−)

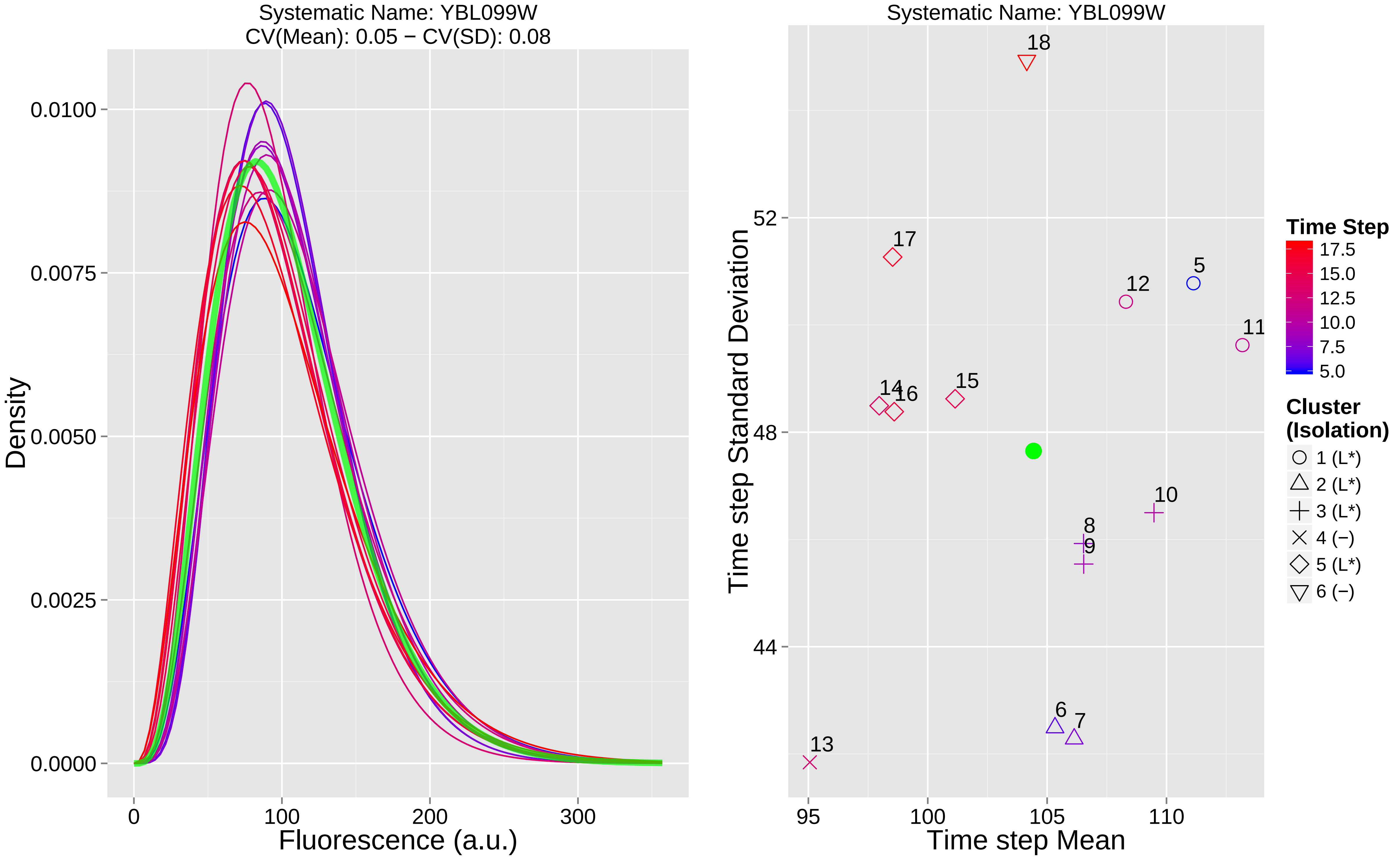

Systematic Name: YBL099W
CV(Mean): 0.05 − CV(SD): 0.08
Density
Fluorescence (a.u.)
0.0000
0.0025
0.0050
0.0075
0.0100
0
100
200
300
Systematic Name: YBL099W
Time step Standard Deviation
Time step Mean
44
48
52
95
100
105
110
18
17
5
12
11
14
16
15
10
8
9
6
7
13
Time Step
17.5
15.0
12.5
10.0
7.5
5.0
Cluster
(Isolation)
1 (L*)
2 (L*)
3 (L*)
4 (−)
5 (L*)
6 (−)

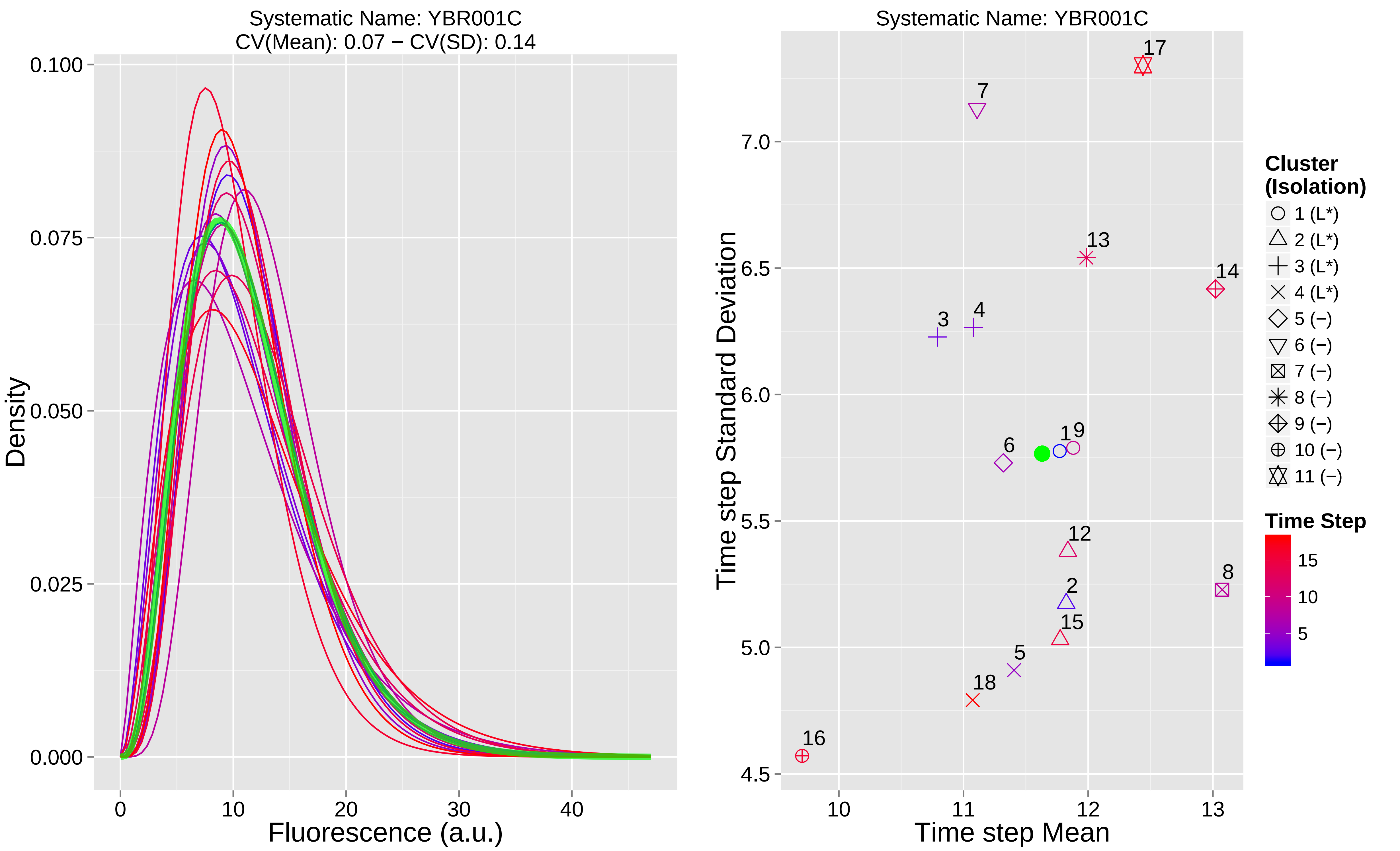

Systematic Name: YBR001C
CV(Mean): 0.07 − CV(SD): 0.14
Density
Fluorescence (a.u.)
0.100
0.075
0.050
0.025
0.000
0
10
20
30
40
Systematic Name: YBR001C
Time step Standard Deviation
Time step Mean
Cluster (Isolation)
1 (L*)
2 (L*)
3 (L*)
4 (L*)
5 (−)
6 (−)
7 (−)
8 (−)
9 (−)
10 (−)
11 (−)
Time Step
15
10
5

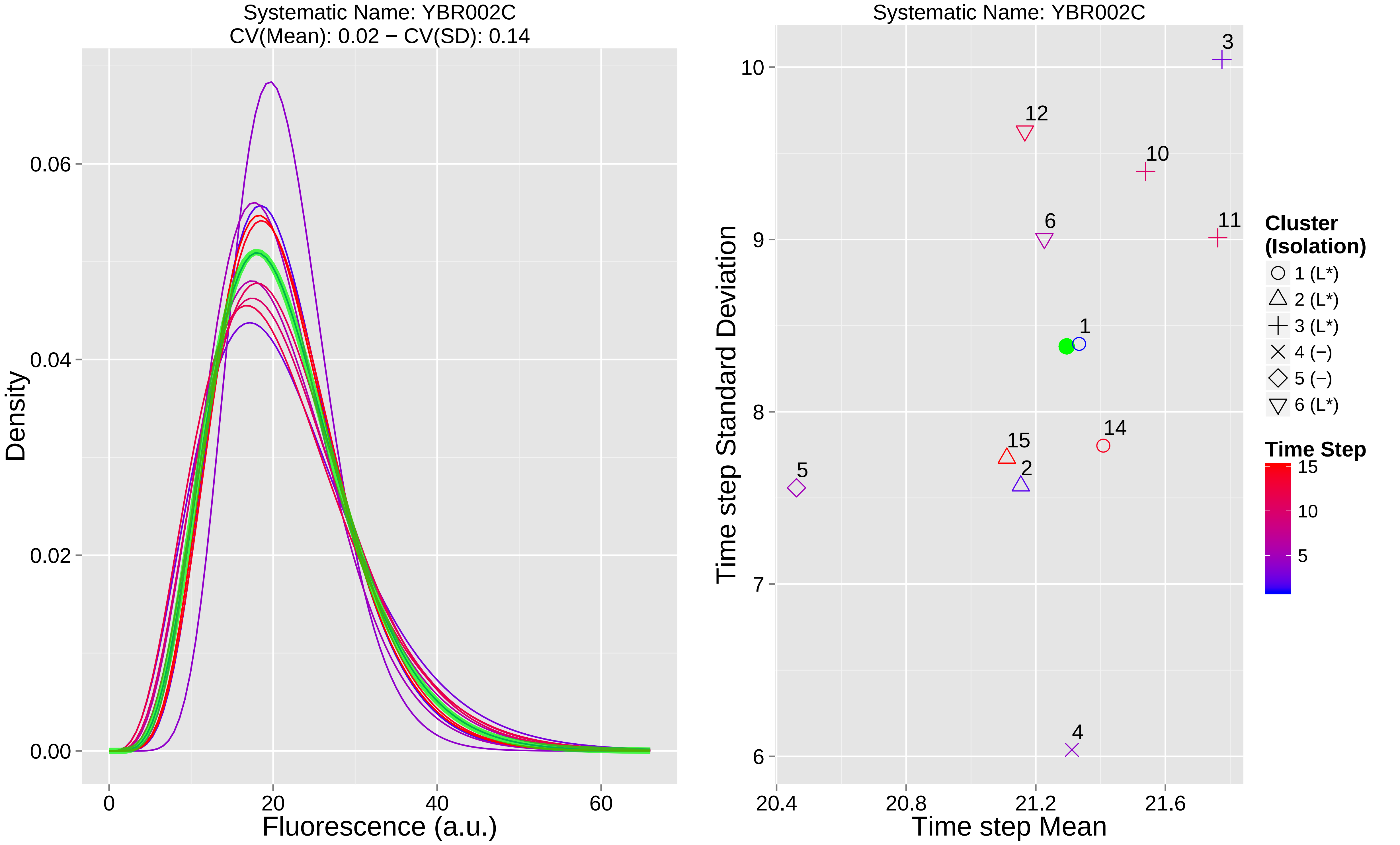

Systematic Name: YBR002C
CV(Mean): 0.02 − CV(SD): 0.14
Density
0.06
0.04
0.02
0.00
0
20
40
60
Fluorescence (a.u.)
Systematic Name: YBR002C
Time step Standard Deviation
10
9
8
7
6
20.4
20.8
21.2
21.6
Time step Mean
3
12
10
6
11
1
14
15
2
5
4
Cluster (Isolation)
1 (L*)
2 (L*)
3 (L*)
4 (−)
5 (−)
6 (L*)
Time Step
15
10
5

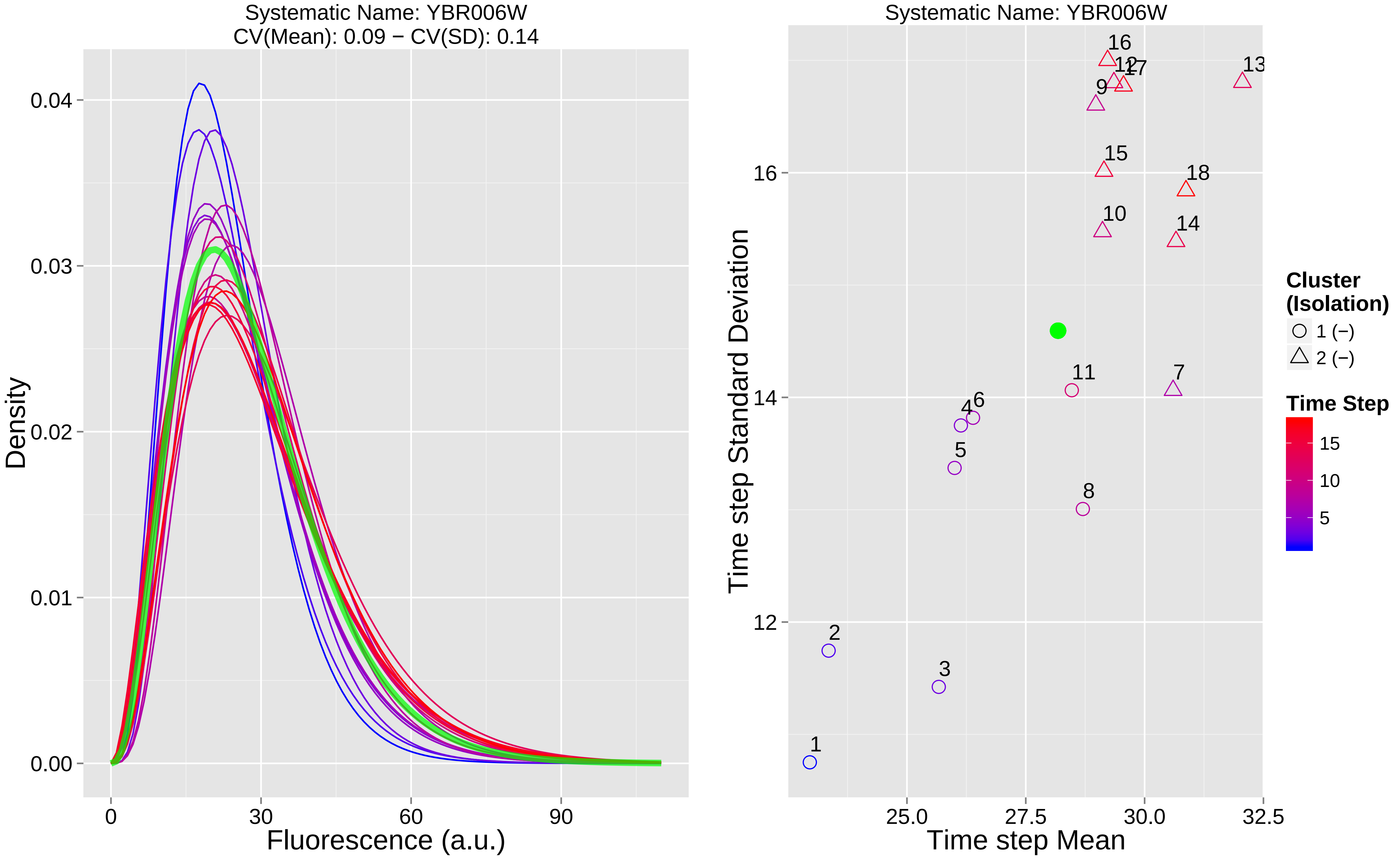

Systematic Name: YBR006W
CV(Mean): 0.09 − CV(SD): 0.14
Density
Fluorescence (a.u.)
0.00
0.01
0.02
0.03
0.04
0
30
60
90
Systematic Name: YBR006W
Time step Standard Deviation
Time step Mean
12
14
16
25.0
27.5
30.0
32.5
Cluster
(Isolation)
1 (−)
2 (−)
Time Step
15
10
5

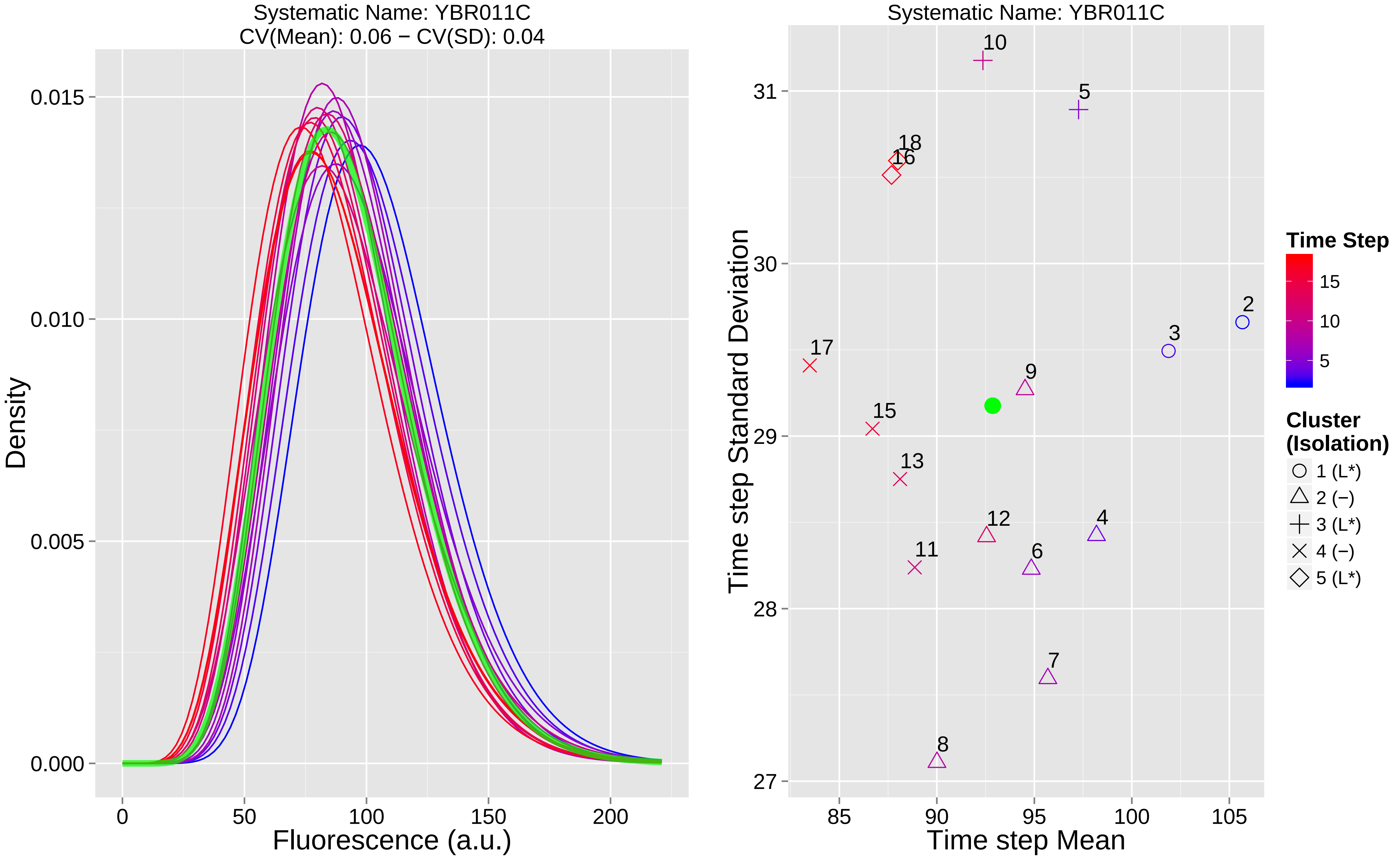

Systematic Name: YBR011C
CV(Mean): 0.06 − CV(SD): 0.04
Density
0.015
0.010
0.005
0.000
0
50
100
150
200
Fluorescence (a.u.)
Systematic Name: YBR011C
Time step Standard Deviation
31
30
29
28
27
85
90
95
100
105
Time step Mean
10
5
18
16
2
3
17
9
15
13
12
4
11
6
7
8
Time Step
15
10
5
Cluster
(Isolation)
1 (L*)
2 (−)
3 (L*)
4 (−)
5 (L*)

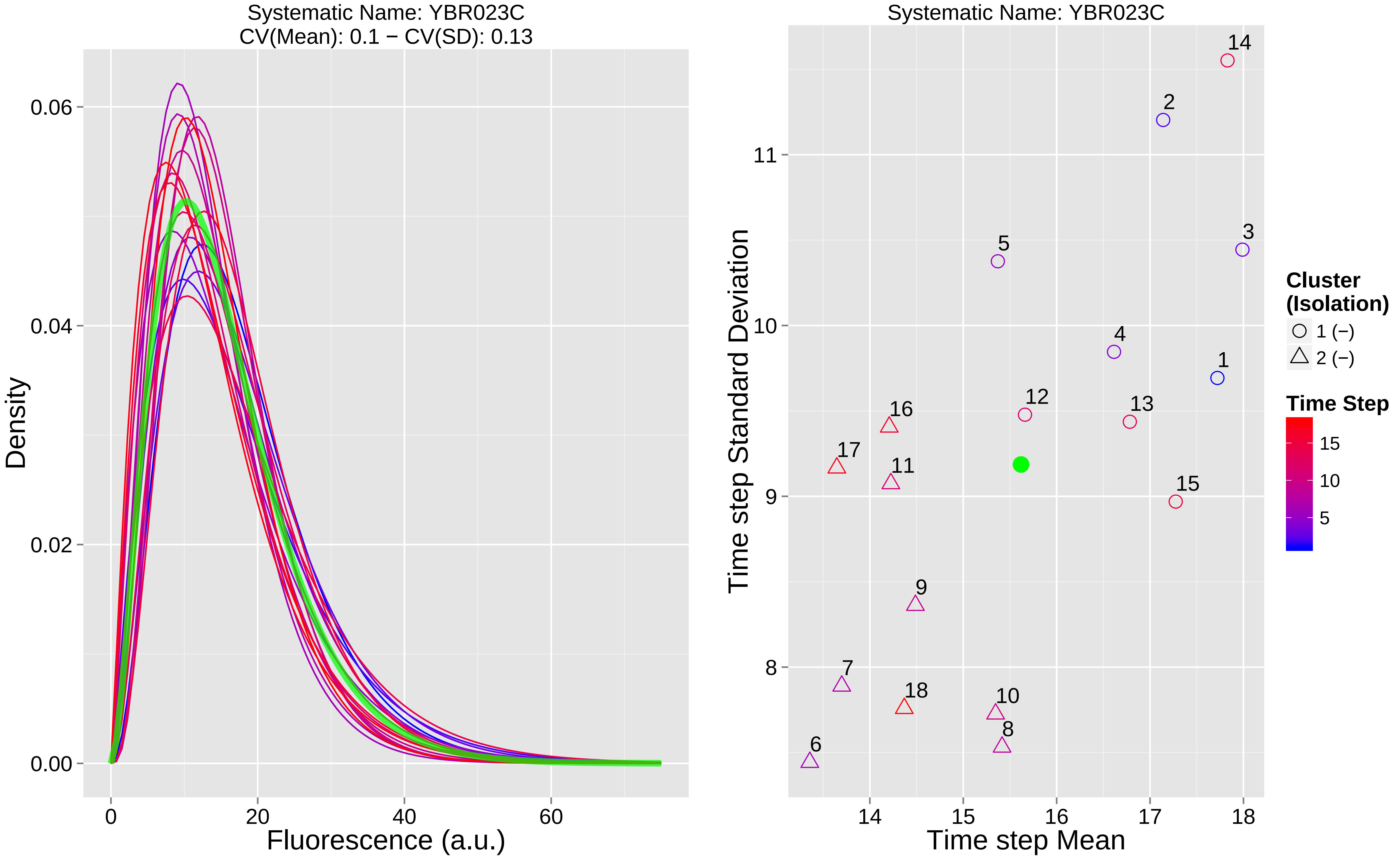

Systematic Name: YBR023C
CV(Mean): 0.1 − CV(SD): 0.13
Density
Fluorescence (a.u.)
0.00
0.02
0.04
0.06
0
20
40
60
Systematic Name: YBR023C
Time step Standard Deviation
Time step Mean
8
9
10
11
14
15
16
17
18
1
2
3
4
5
6
7
8
9
10
11
12
13
14
15
16
17
18
Cluster (Isolation)
1 (−)
2 (−)
Time Step
5
10
15

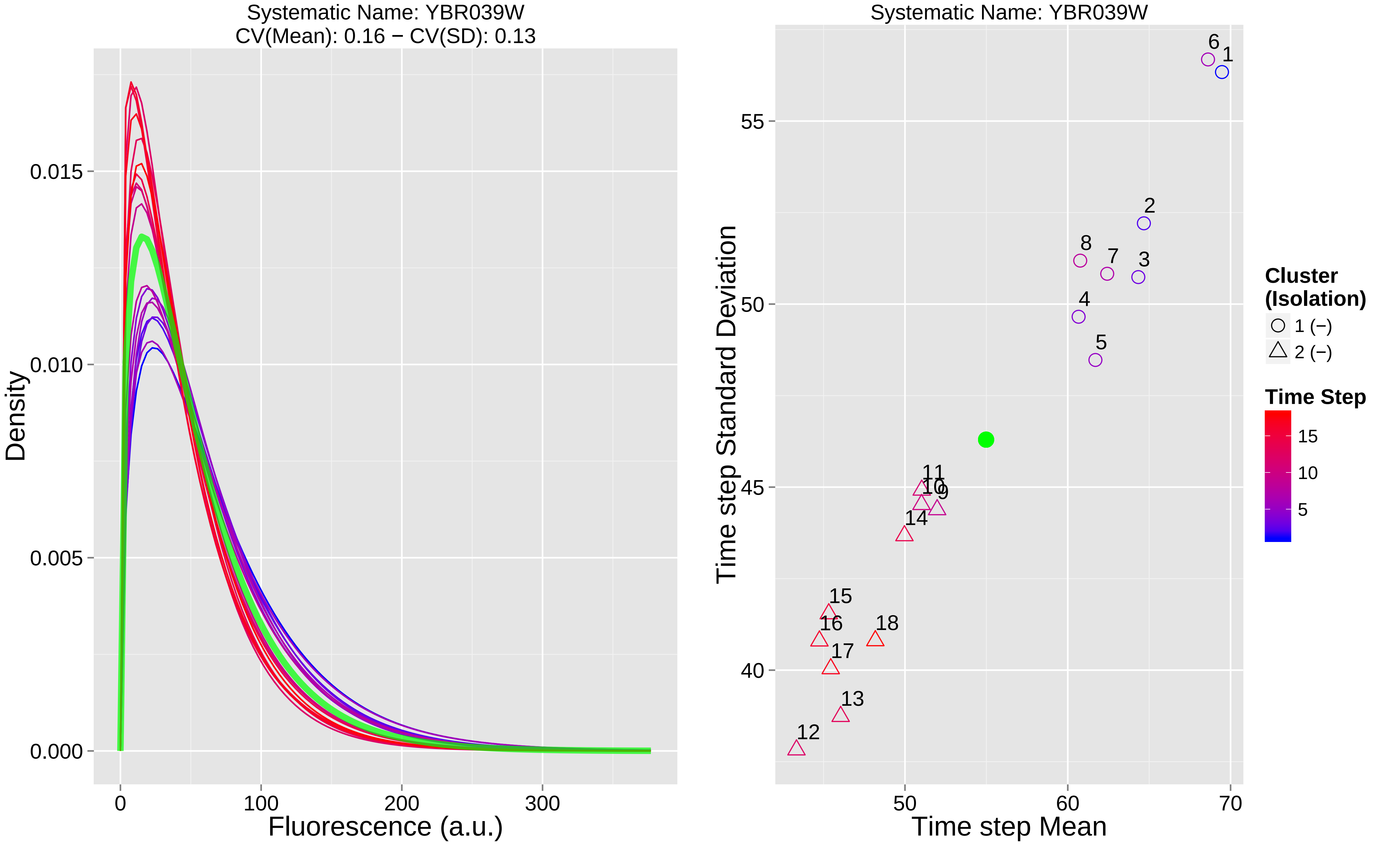

Systematic Name: YBR039W
CV(Mean): 0.16 − CV(SD): 0.13
Density
0.000
0.005
0.010
0.015
0
100
200
300
Fluorescence (a.u.)
Systematic Name: YBR039W
Time step Standard Deviation
40
45
50
55
50
60
70
Time step Mean
Cluster
(Isolation)
1 (−)
2 (−)
Time Step
15
10
5

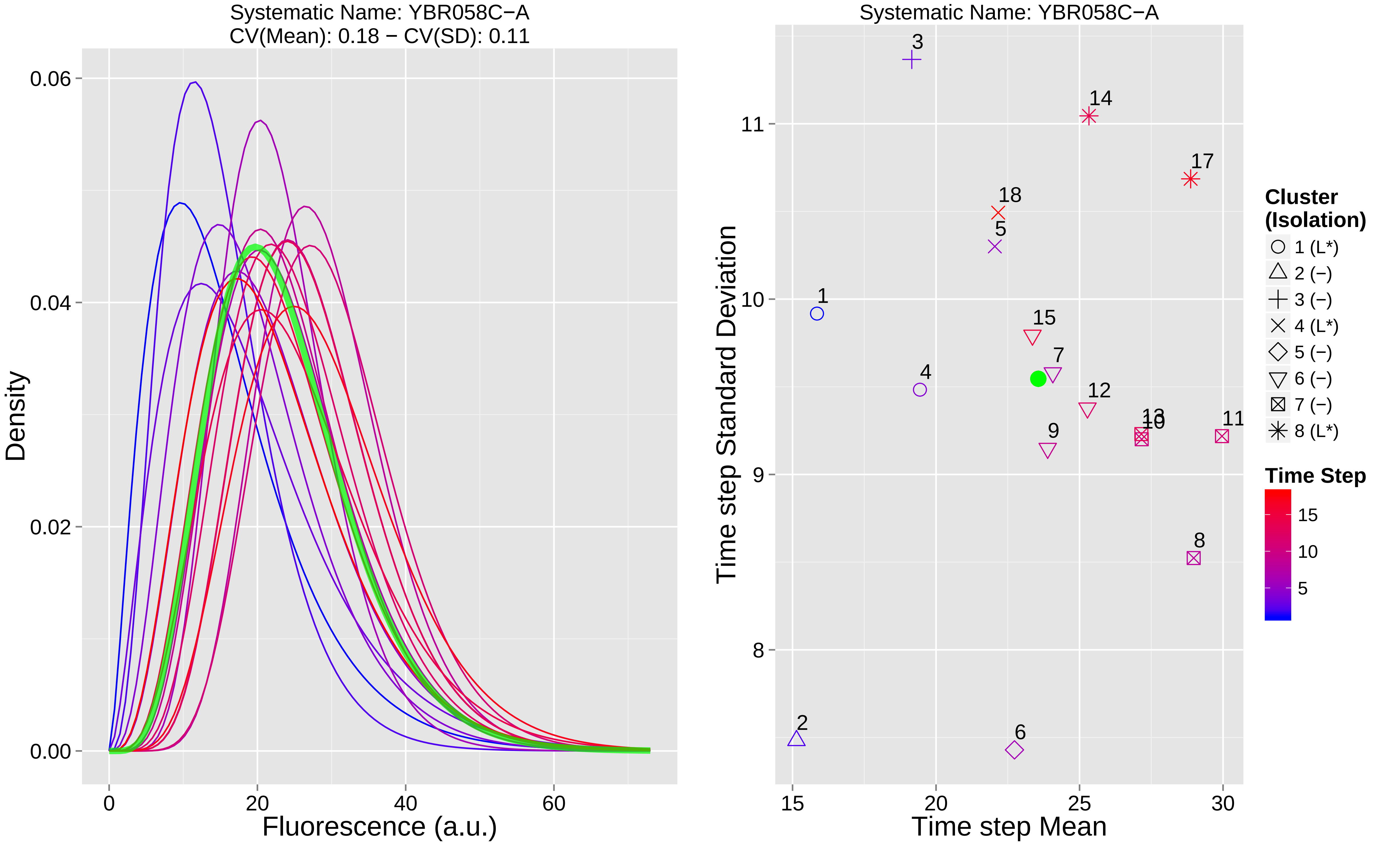
Systematic Name: YBR058C−A
CV(Mean): 0.18 − CV(SD): 0.11
Density
0.06
0.04
0.02
0.00
0
20
40
60
Fluorescence (a.u.)
Systematic Name: YBR058C−A
Time step Standard Deviation
11
10
9
8
15
20
25
30
Time step Mean
3
14
17
18
5
1
15
7
4
12
13
16
11
9
8
2
6
Cluster
(Isolation)
1 (L*)
2 (−)
3 (−)
4 (L*)
5 (−)
6 (−)
7 (−)
8 (L*)
Time Step
15
10
5

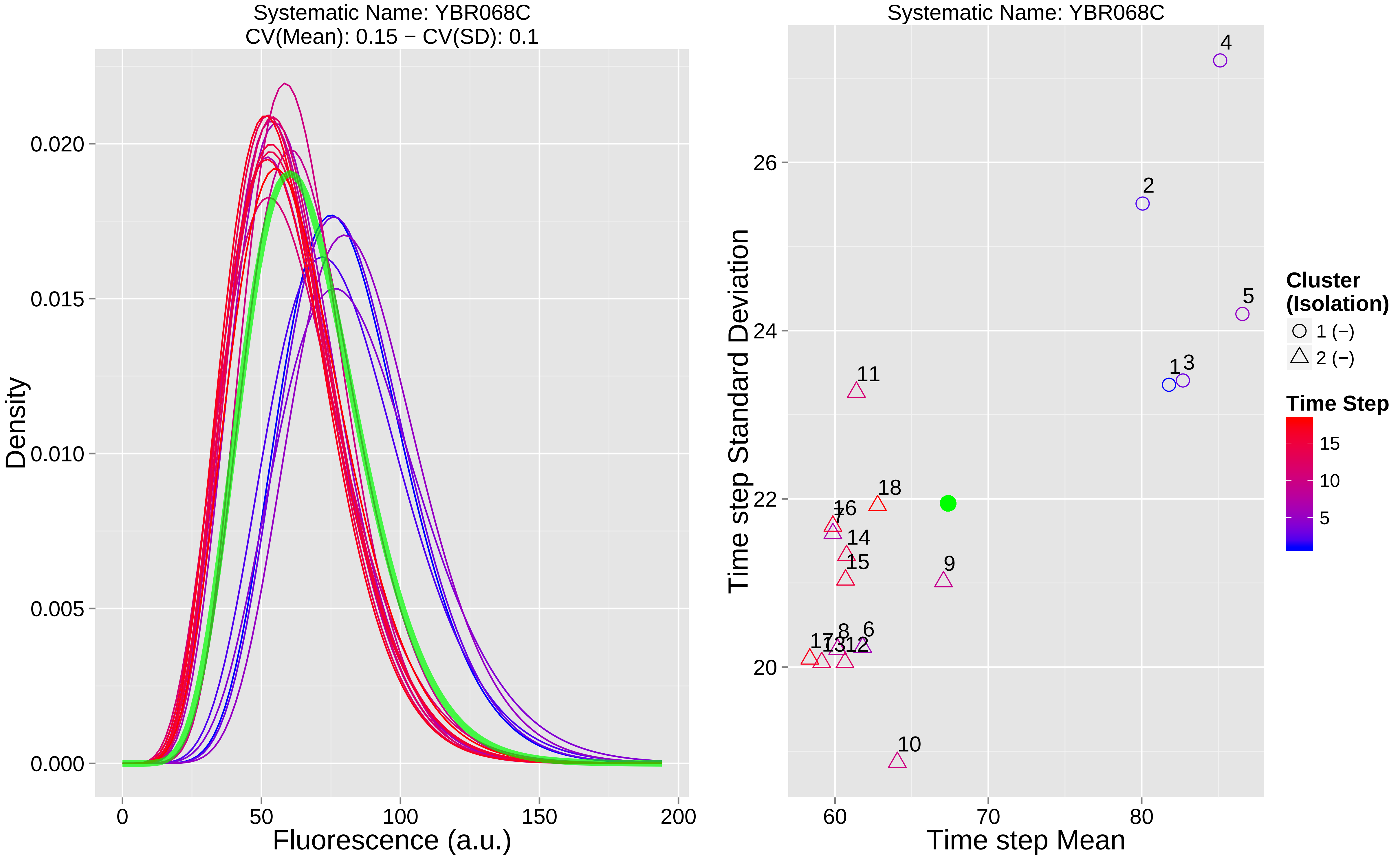

Systematic Name: YBR068C
CV(Mean): 0.15 − CV(SD): 0.1
Density
0.020
0.015
0.010
0.005
0.000
0
50
100
150
200
Fluorescence (a.u.)
Systematic Name: YBR068C
Time step Standard Deviation
26
24
22
20
60
70
80
Time step Mean
4
2
5
1
3
11
18
16
7
14
15
9
17
13
8
12
6
10
Cluster (Isolation)
1 (−)
2 (−)
Time Step
15
10
5

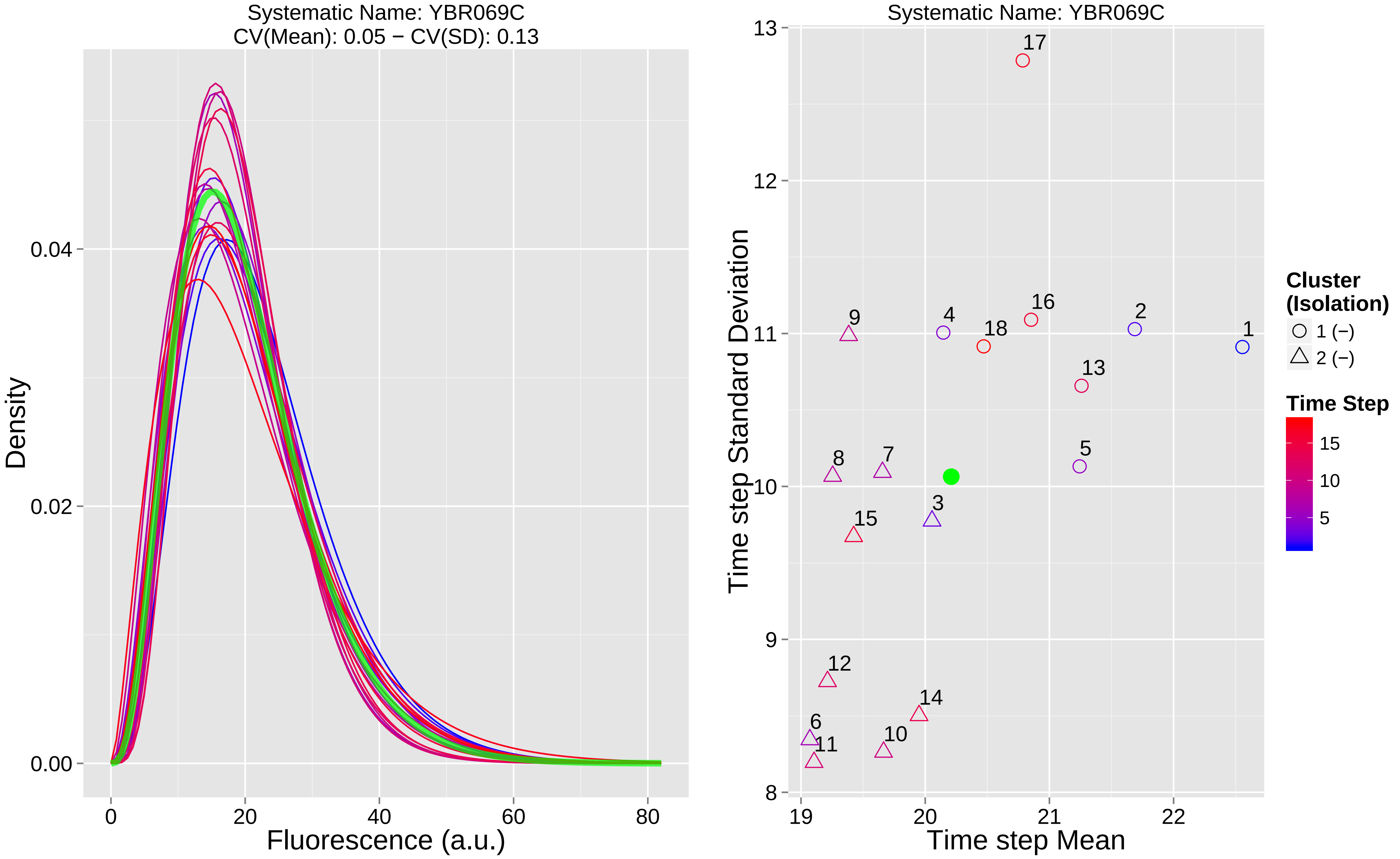

Systematic Name: YBR069C
CV(Mean): 0.05 − CV(SD): 0.13
Density
0.04
0.02
0.00
0
20
40
60
80
Fluorescence (a.u.)
Systematic Name: YBR069C
Time step Standard Deviation
13
12
11
10
9
8
19
20
21
22
Time step Mean
17
16
2
1
9
4
18
13
8
7
5
3
15
12
14
6
10
11
Cluster (Isolation)
1 (−)
2 (−)
Time Step
15
10
5

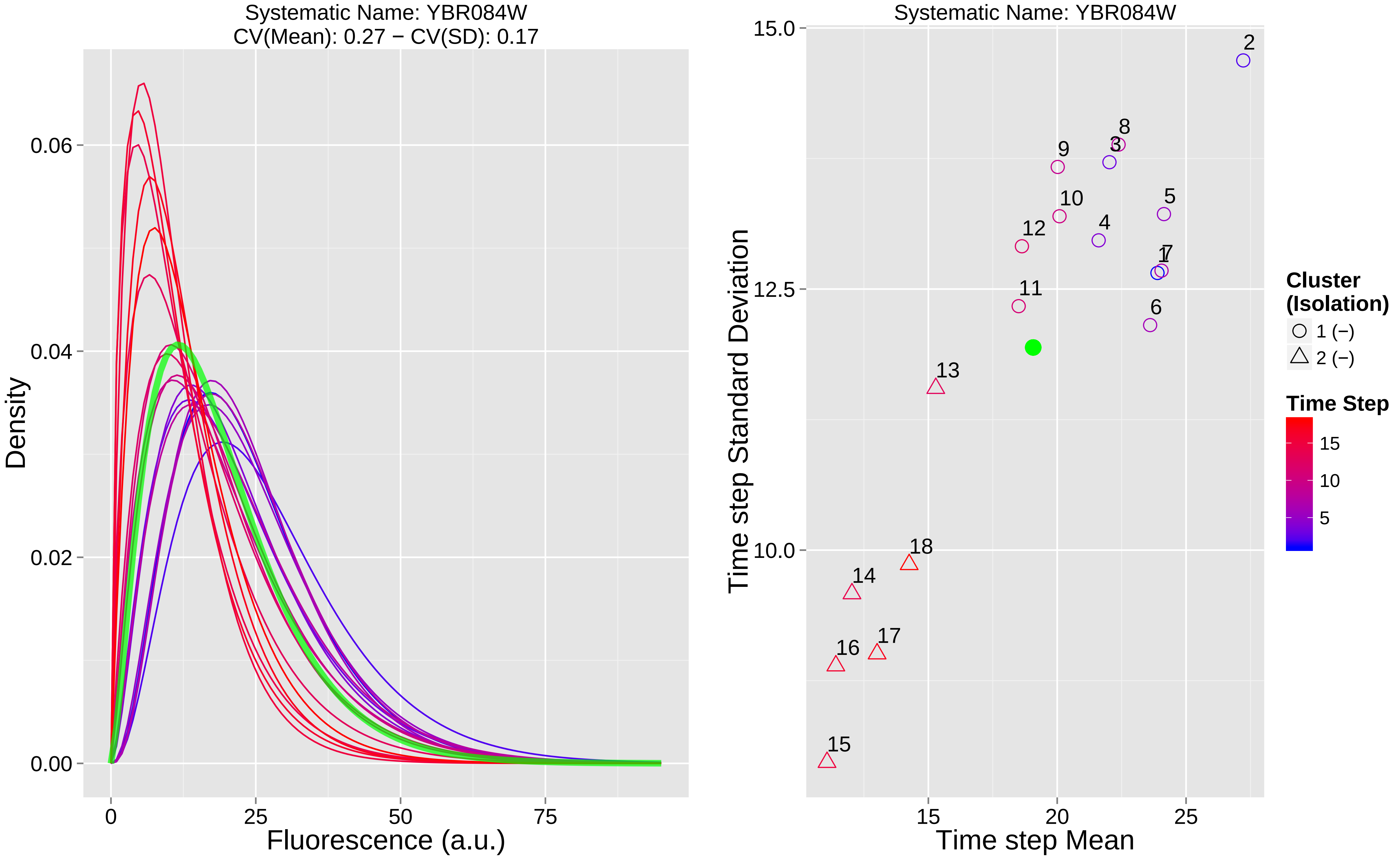

Systematic Name: YBR084W
CV(Mean): 0.27 − CV(SD): 0.17
Density
0.06
0.04
0.02
0.00
0
25
50
75
Fluorescence (a.u.)
Systematic Name: YBR084W
Time step Standard Deviation
15.0
12.5
10.0
15
20
25
Time step Mean
2
8
3
9
10
5
4
12
1
7
11
6
13
18
14
17
16
15
Cluster (Isolation)
1 (−)
2 (−)
Time Step
15
10
5

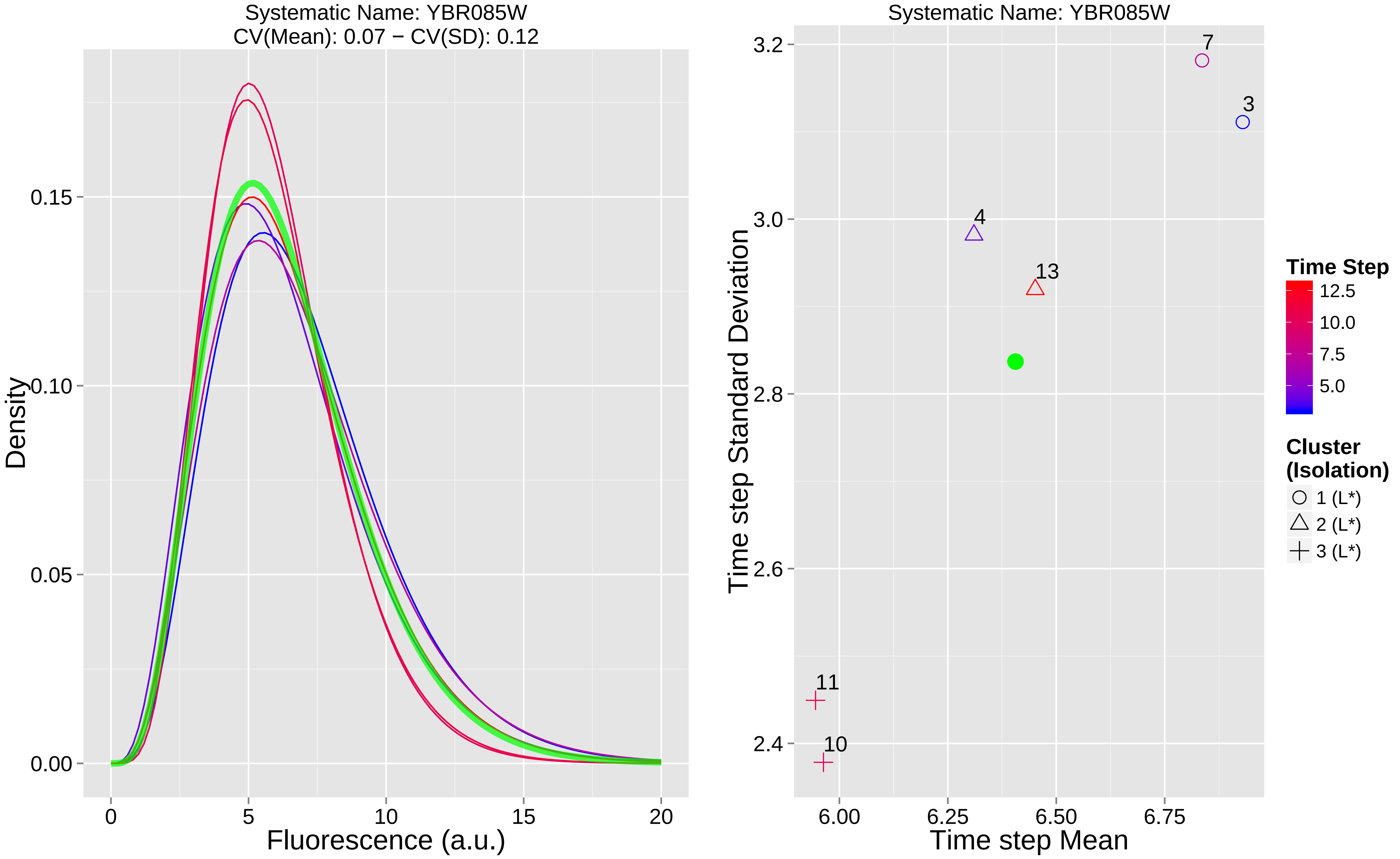
Systematic Name: YBR085W
CV(Mean): 0.07 − CV(SD): 0.12
Density
0.00
0.05
0.10
0.15
0
5
10
15
20
Fluorescence (a.u.)
Systematic Name: YBR085W
Time step Standard Deviation
2.4
2.6
2.8
3.0
3.2
6.00
6.25
6.50
6.75
Time step Mean
7
3
4
13
11
10
Time Step
12.5
10.0
7.5
5.0
Cluster
(Isolation)
1 (L*)
2 (L*)
3 (L*)

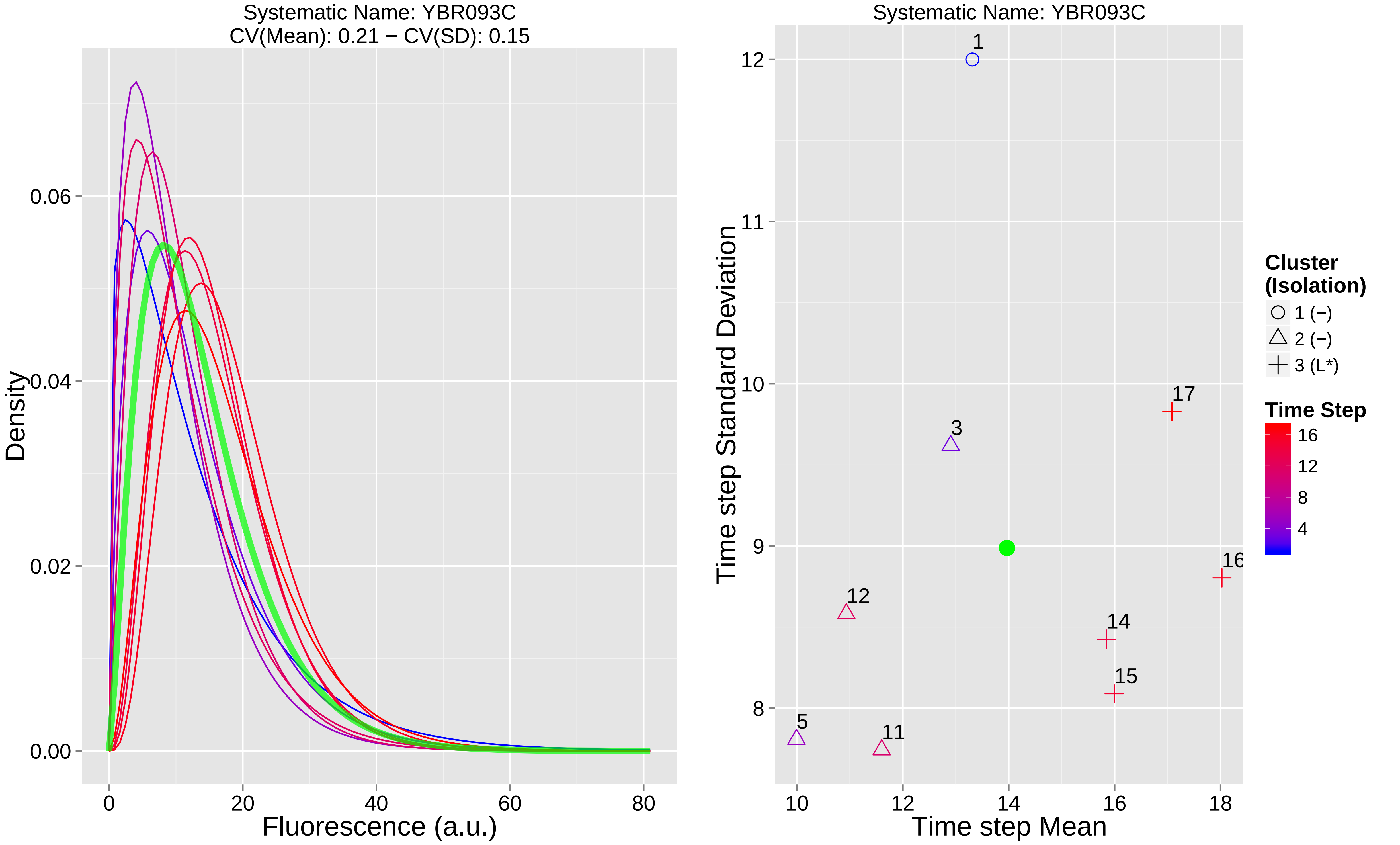

Systematic Name: YBR093C
CV(Mean): 0.21 − CV(SD): 0.15
Density
0.06
0.04
0.02
0.00
0
20
40
60
80
Fluorescence (a.u.)
Systematic Name: YBR093C
Time step Standard Deviation
12
11
10
9
8
10
12
14
16
18
Time step Mean
1
3
5
11
12
14
15
16
17
Cluster (Isolation)
1 (−)
2 (−)
3 (L*)
Time Step
16
12
8
4

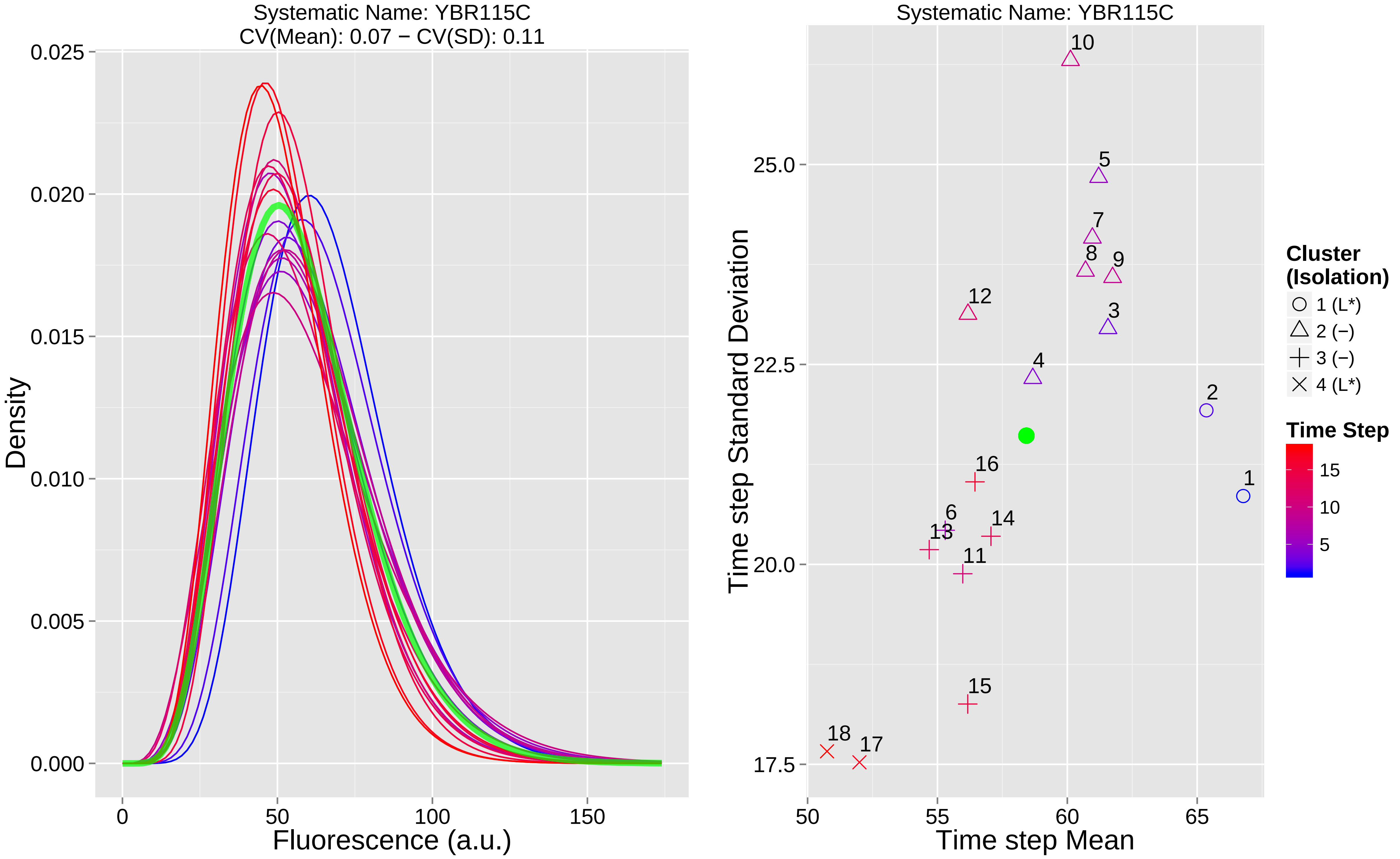

Systematic Name: YBR115C
CV(Mean): 0.07 − CV(SD): 0.11
Density
Fluorescence (a.u.)
0.025
0.020
0.015
0.010
0.005
0.000
0
50
100
150
Systematic Name: YBR115C
Time step Standard Deviation
Time step Mean
25.0
22.5
20.0
17.5
50
55
60
65
1
2
3
4
5
6
7
8
9
10
11
12
13
14
15
16
17
18
Cluster (Isolation)
1 (L*)
2 (−)
3 (−)
4 (L*)
Time Step
15
10
5

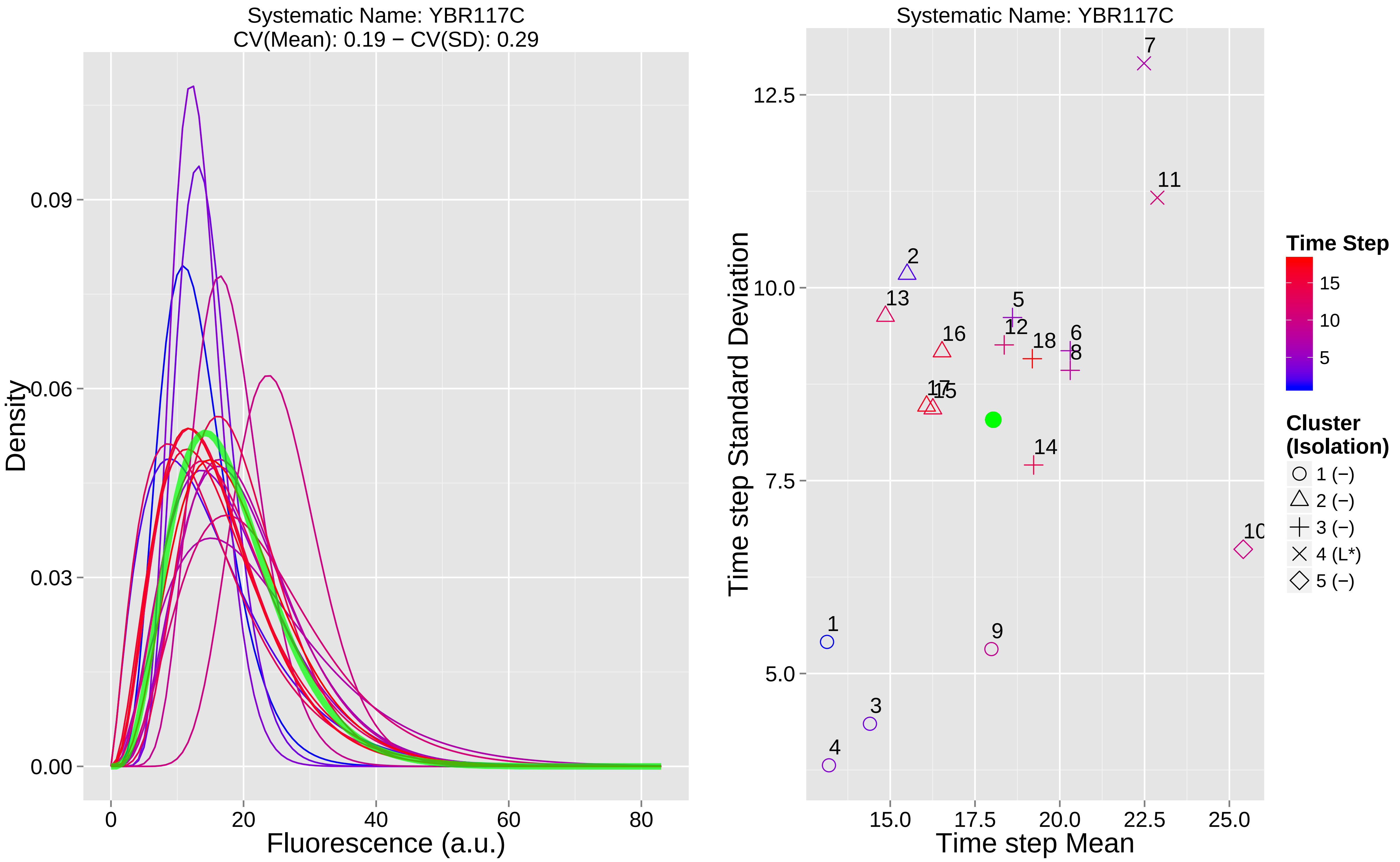

Systematic Name: YBR117C
CV(Mean): 0.19 − CV(SD): 0.29
Density
0.09
0.06
0.03
0.00
0
20
40
60
80
Fluorescence (a.u.)
Systematic Name: YBR117C
Time step Standard Deviation
12.5
10.0
7.5
5.0
15.0
17.5
20.0
22.5
25.0
Time step Mean
7
11
2
13
5
12
18
6
8
16
17
15
14
10
1
9
3
4
Time Step
15
10
5
Cluster (Isolation)
1 (−)
2 (−)
3 (−)
4 (L*)
5 (−)

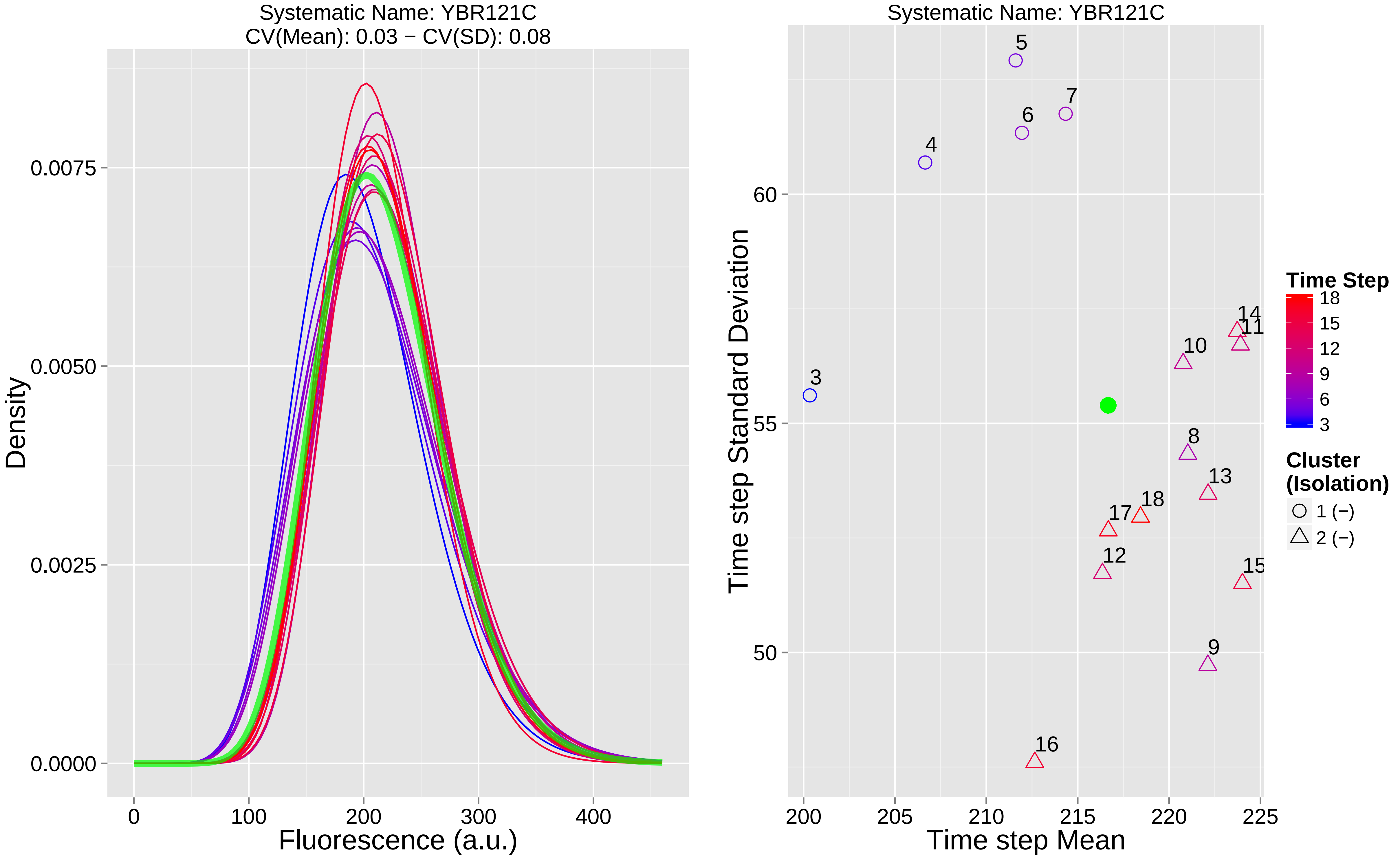
Systematic Name: YBR121C
CV(Mean): 0.03 − CV(SD): 0.08
Density
Fluorescence (a.u.)
0.0000
0.0025
0.0050
0.0075
0
100
200
300
400
Systematic Name: YBR121C
Time step Standard Deviation
Time step Mean
50
55
60
200
205
210
215
220
225
3
4
5
6
7
8
9
10
11
12
13
14
15
16
17
18
Time Step
18
15
12
9
6
3
Cluster
(Isolation)
1 (−)
2 (−)

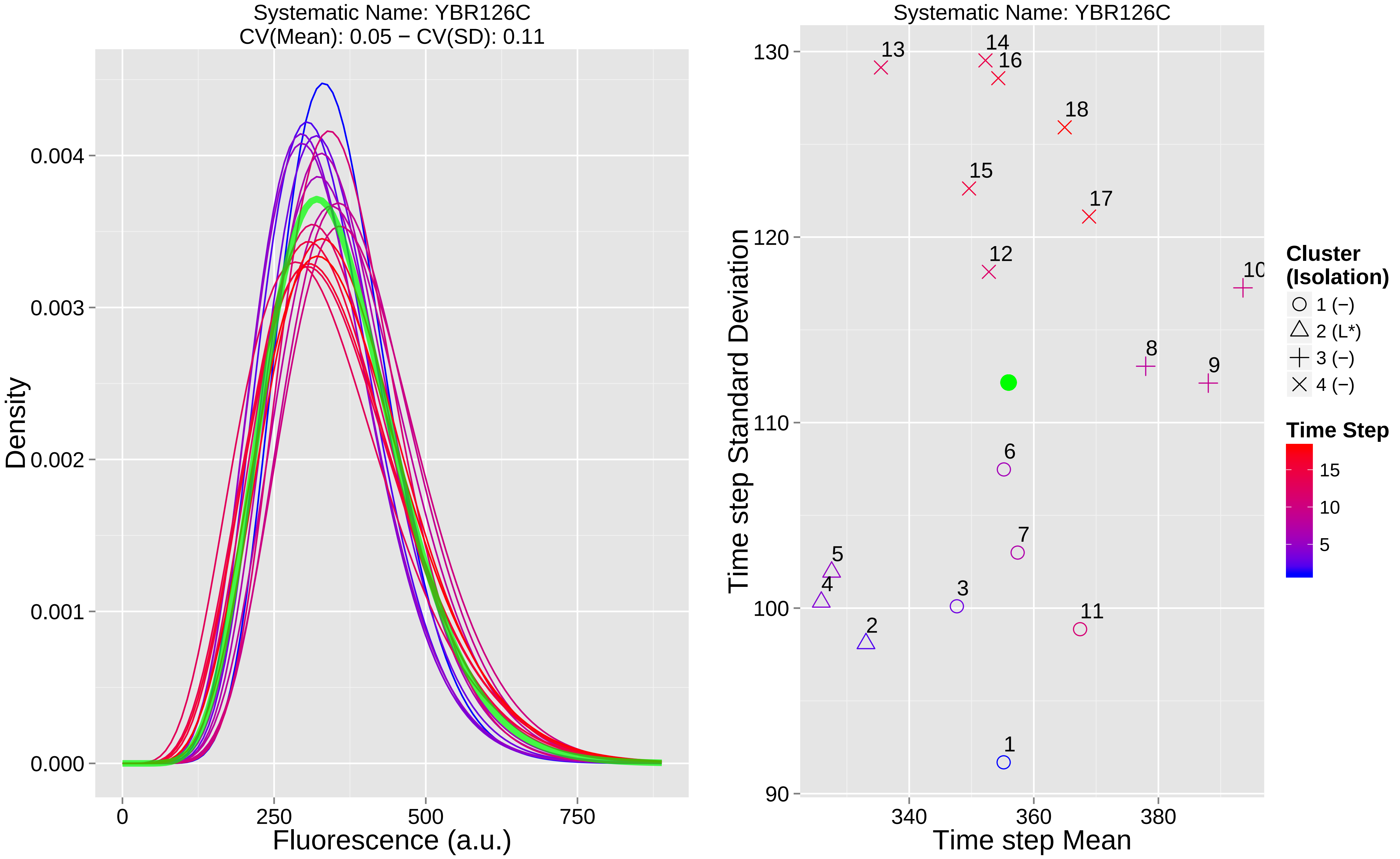
Systematic Name: YBR126C
CV(Mean): 0.05 − CV(SD): 0.11
Density
0.000
0.001
0.002
0.003
0.004
0
250
500
750
Fluorescence (a.u.)
Systematic Name: YBR126C
Time step Standard Deviation
90
100
110
120
130
340
360
380
Time step Mean
1
2
3
4
5
6
7
8
9
10
11
12
13
14
15
16
17
18
Cluster (Isolation)
1 (−)
2 (L*)
3 (−)
4 (−)
Time Step
5
10
15

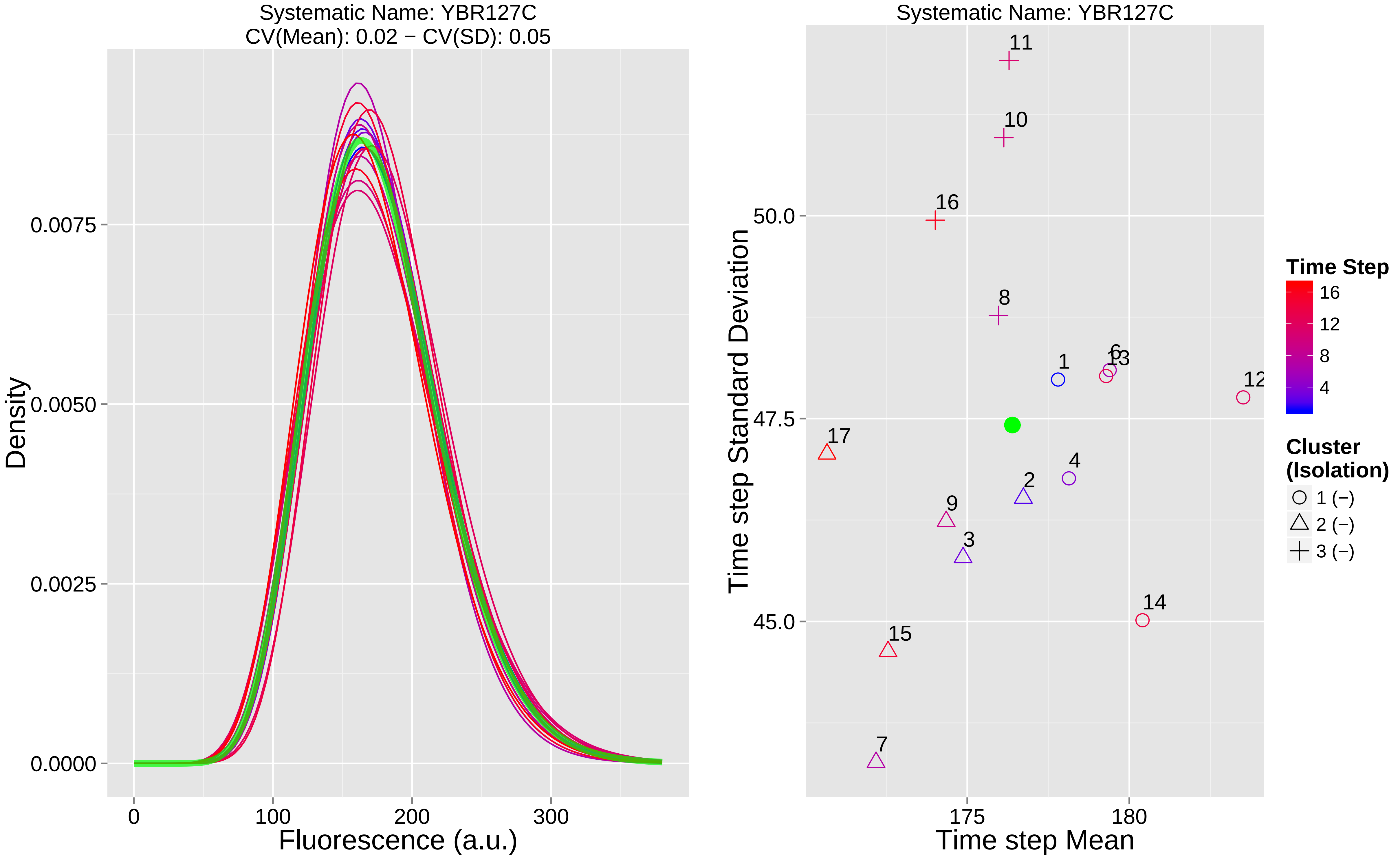
Systematic Name: YBR127C
CV(Mean): 0.02 − CV(SD): 0.05
Density
0.0075
0.0050
0.0025
0.0000
0
100
200
300
Fluorescence (a.u.)
Systematic Name: YBR127C
Time step Standard Deviation
50.0
47.5
45.0
175
180
Time step Mean
11
10
16
8
1
6
13
12
17
4
2
9
3
14
15
7
Time Step
16
12
8
4
Cluster (Isolation)
1 (−)
2 (−)
3 (−)

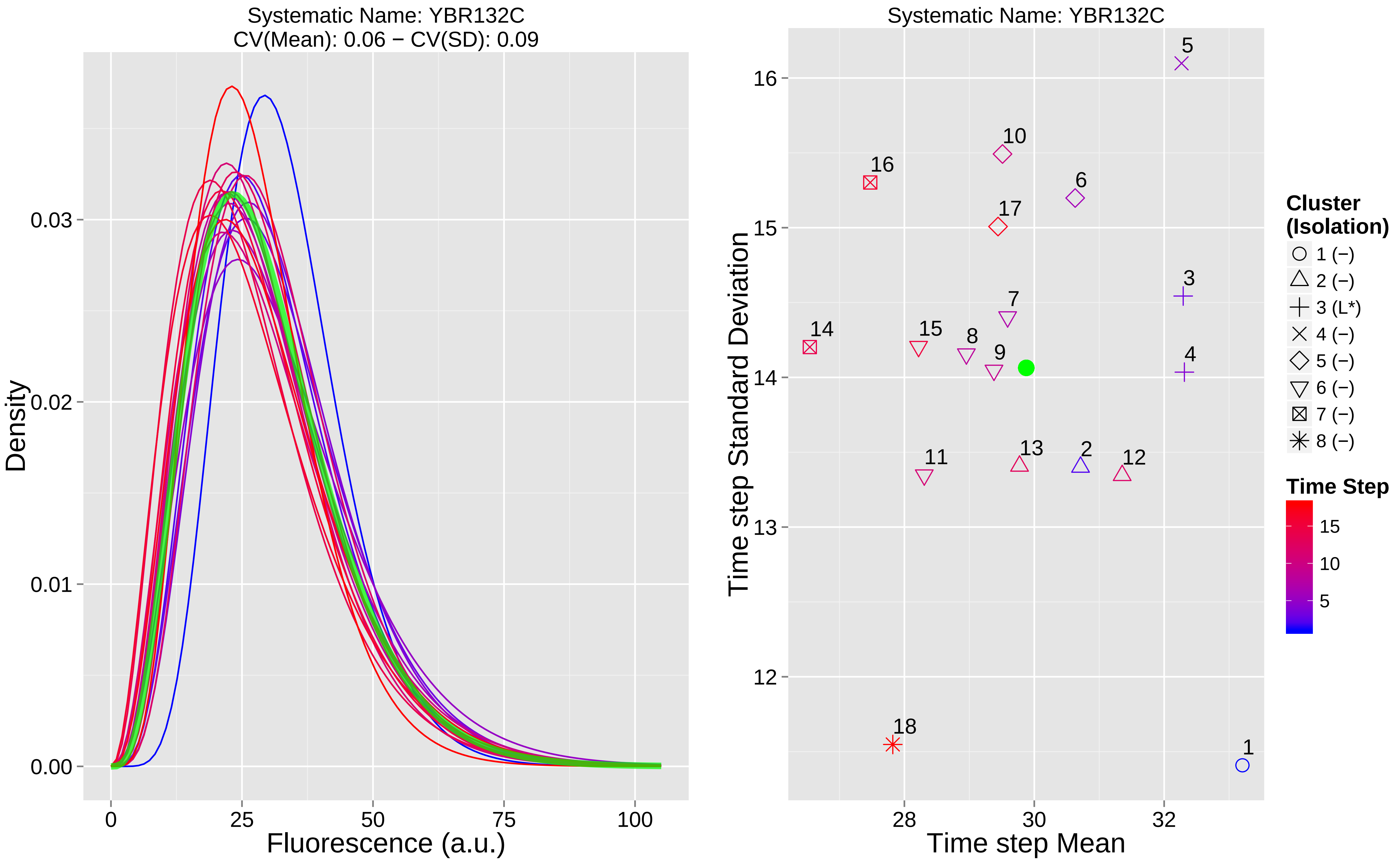

Systematic Name: YBR132C
CV(Mean): 0.06 − CV(SD): 0.09
Density
Fluorescence (a.u.)
0.00
0.01
0.02
0.03
0
25
50
75
100
Systematic Name: YBR132C
Time step Standard Deviation
Time step Mean
12
13
14
15
16
28
30
32
Cluster (Isolation)
1 (−)
2 (−)
3 (L*)
4 (−)
5 (−)
6 (−)
7 (−)
8 (−)
Time Step
15
10
5

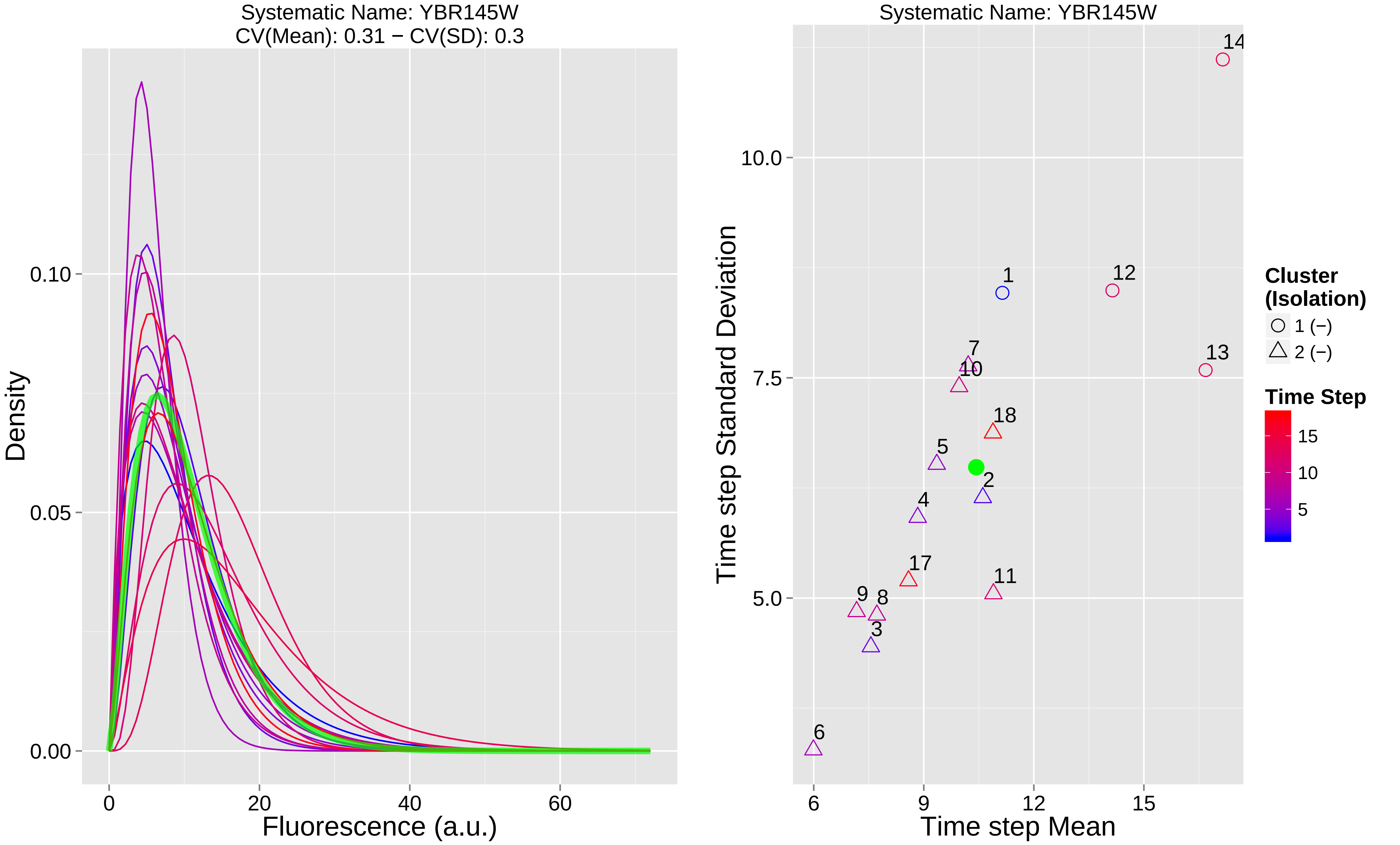

Systematic Name: YBR145W
CV(Mean): 0.31 − CV(SD): 0.3
Density
0.00
0.05
0.10
0
20
40
60
Fluorescence (a.u.)
Systematic Name: YBR145W
Time step Standard Deviation
5.0
7.5
10.0
6
9
12
15
Time step Mean
1
2
3
4
5
6
7
8
9
10
11
12
13
14
17
18
Cluster
(Isolation)
1 (−)
2 (−)
Time Step
15
10
5

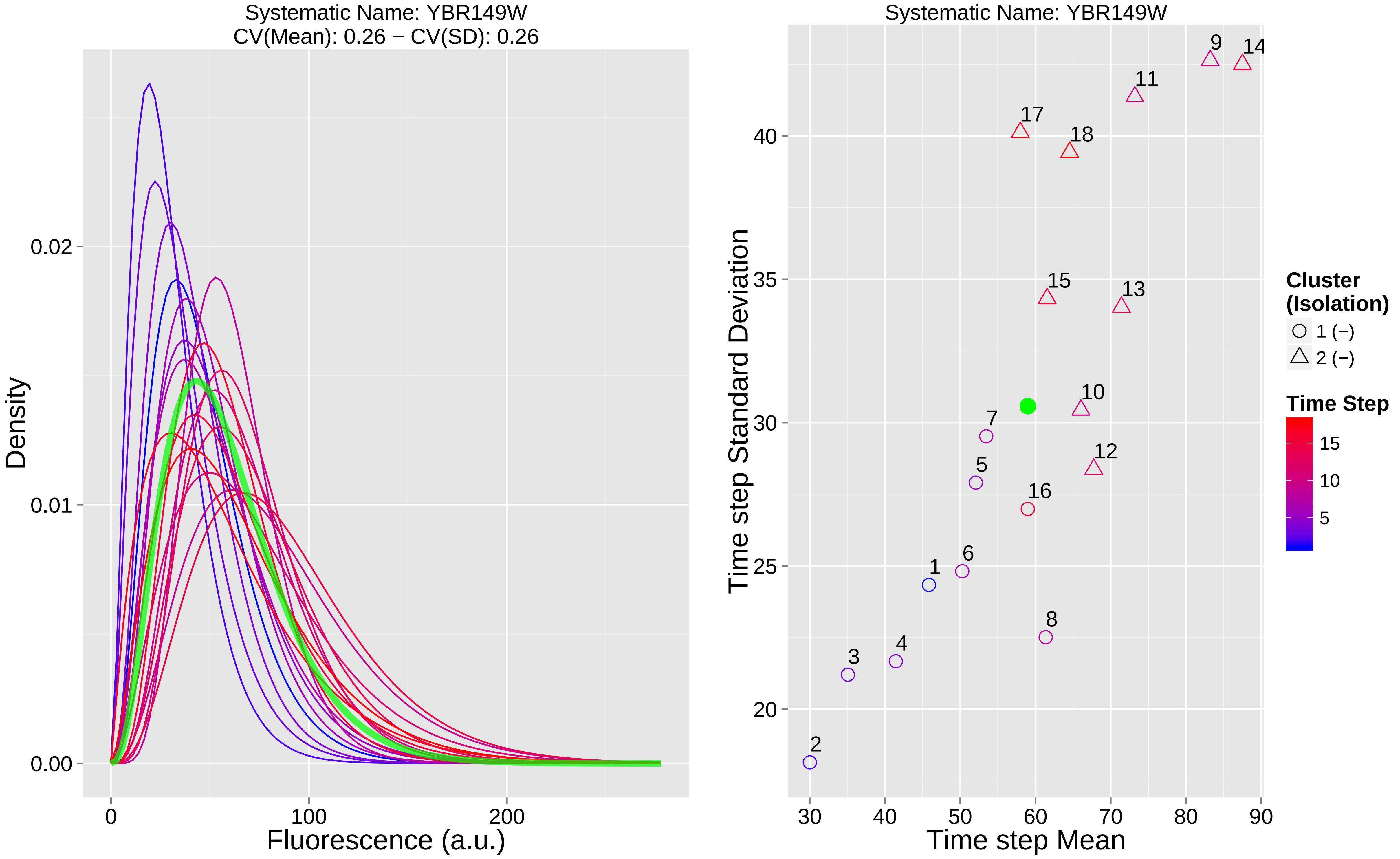

Systematic Name: YBR149W
CV(Mean): 0.26 − CV(SD): 0.26
Density
0.00
0.01
0.02
0
100
200
Fluorescence (a.u.)
Systematic Name: YBR149W
Time step Standard Deviation
20
25
30
35
40
30
40
50
60
70
80
90
Time step Mean
1
2
3
4
5
6
7
8
9
10
11
12
13
14
15
16
17
18
Cluster (Isolation)
1 (−)
2 (−)
Time Step
5
10
15

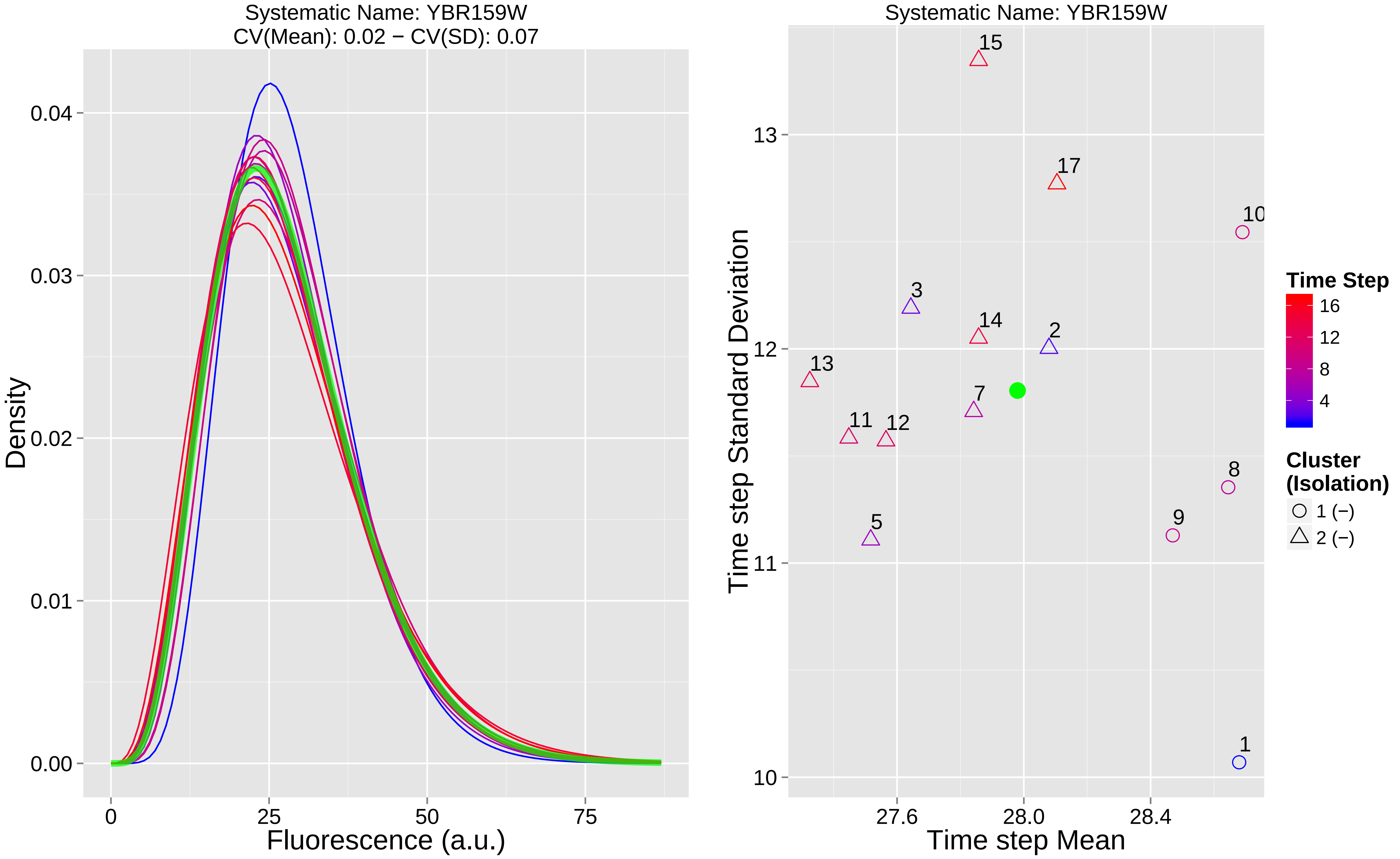
Systematic Name: YBR159W
CV(Mean): 0.02 − CV(SD): 0.07
Density
0.00
0.01
0.02
0.03
0.04
0
25
50
75
Fluorescence (a.u.)
Systematic Name: YBR159W
Time step Standard Deviation
10
11
12
13
27.6
28.0
28.4
Time step Mean
15
17
10
3
14
2
13
7
11
12
8
5
9
1
Time Step
16
12
8
4
Cluster
(Isolation)
1 (−)
2 (−)

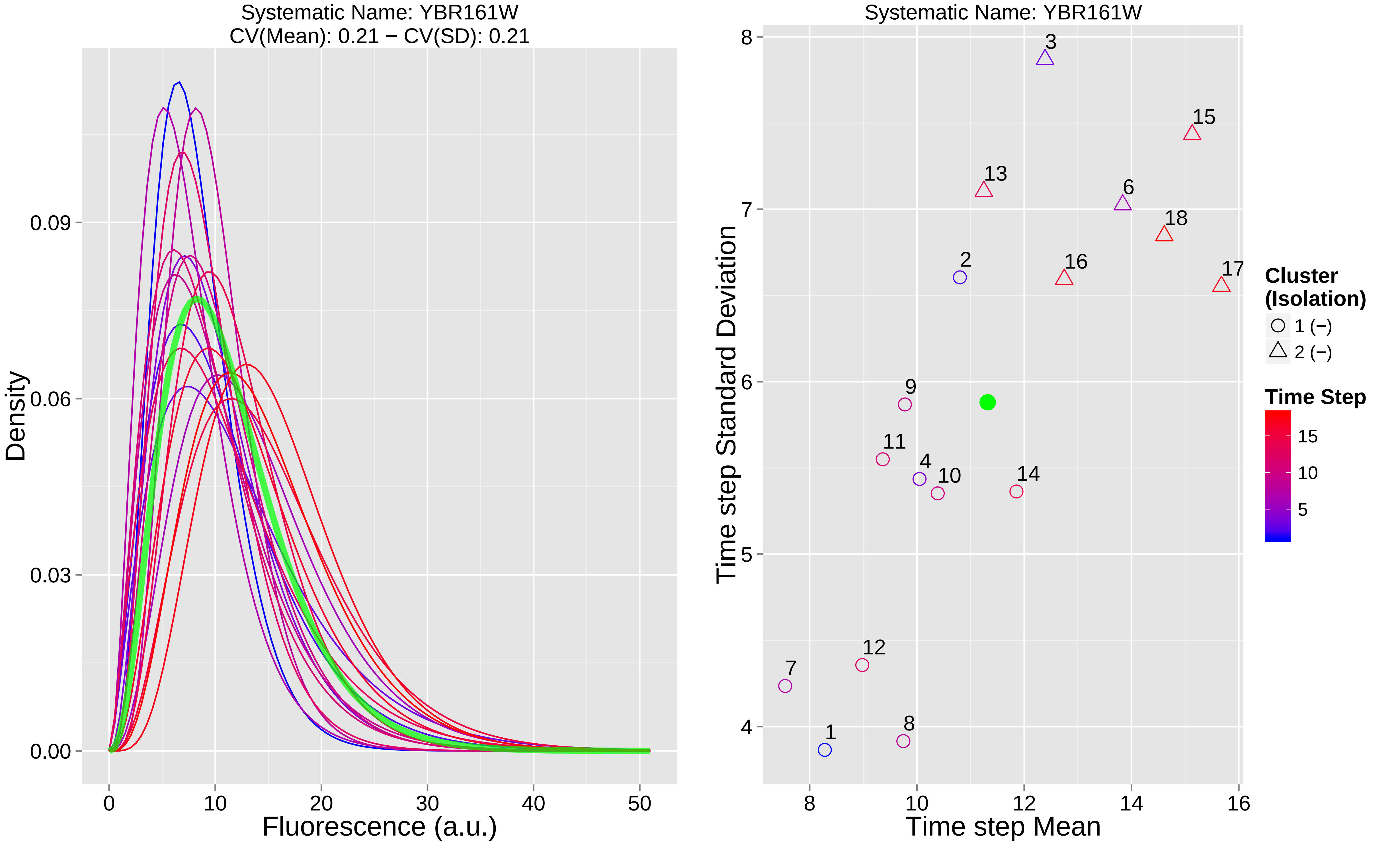
Systematic Name: YBR161W
CV(Mean): 0.21 − CV(SD): 0.21
Density
0.00
0.03
0.06
0.09
0
10
20
30
40
50
Fluorescence (a.u.)
Systematic Name: YBR161W
Time step Standard Deviation
Time step Mean
4
5
6
7
8
8
10
12
14
16
1
2
3
4
6
7
8
9
10
11
12
13
14
15
16
17
18
Cluster
(Isolation)
1 (−)
2 (−)
Time Step
15
10
5

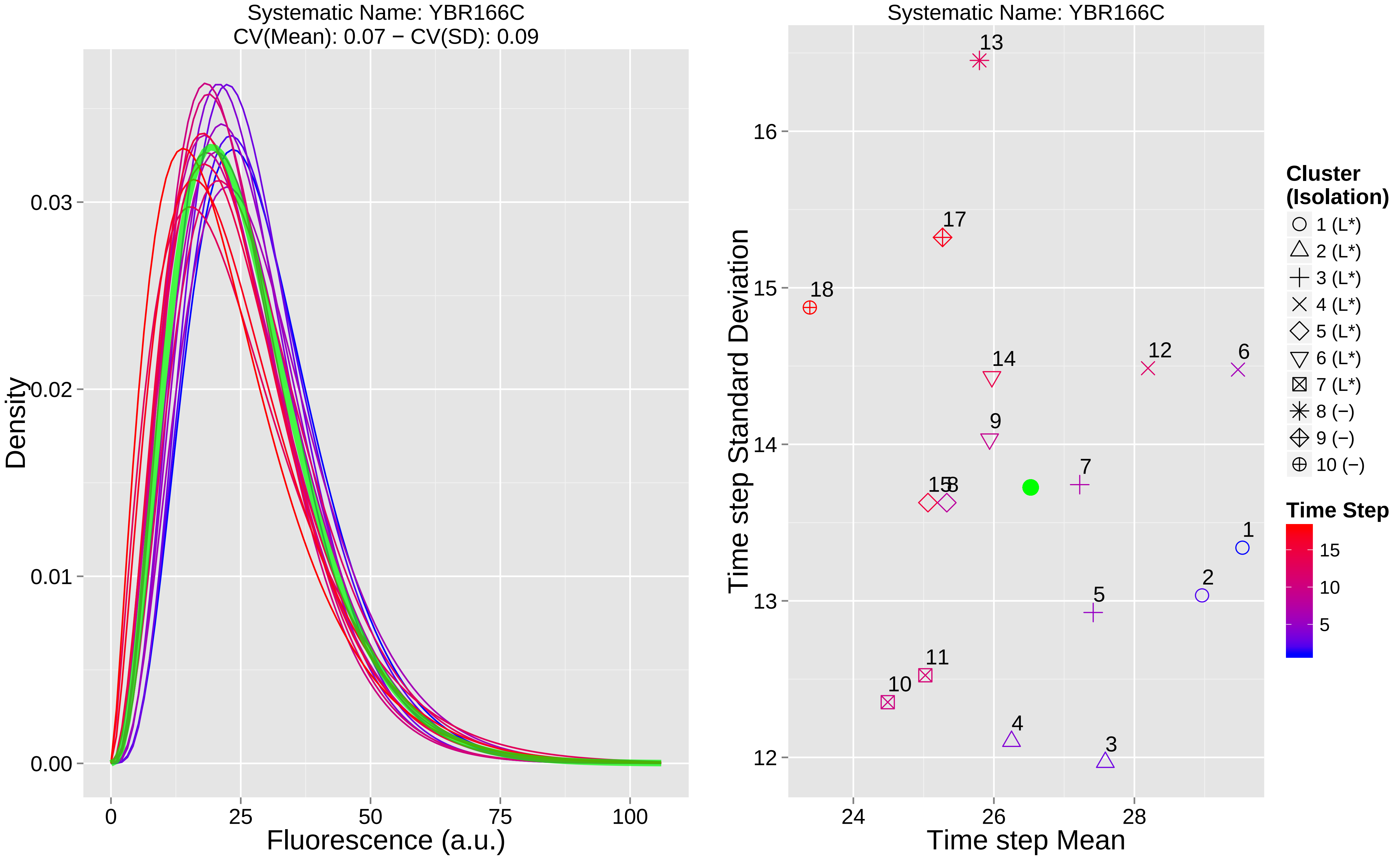
Systematic Name: YBR166C
CV(Mean): 0.07 − CV(SD): 0.09
Density
Fluorescence (a.u.)
Systematic Name: YBR166C
Time step Standard Deviation
Time step Mean
Cluster (Isolation)
1 (L*)
2 (L*)
3 (L*)
4 (L*)
5 (L*)
6 (L*)
7 (L*)
8 (−)
9 (−)
10 (−)
Time Step

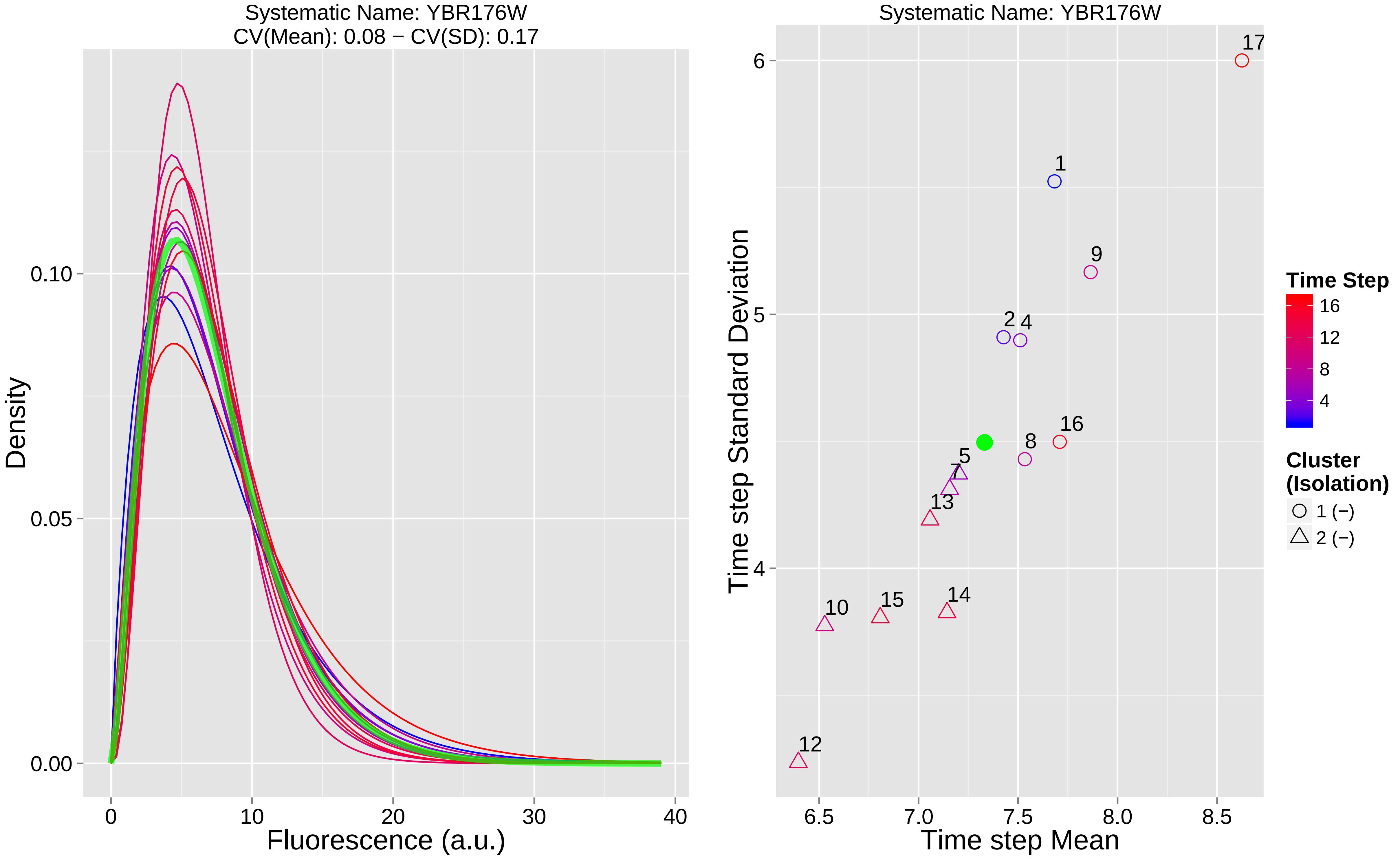

Systematic Name: YBR176W
CV(Mean): 0.08 − CV(SD): 0.17
Density
0.00
0.05
0.10
0
10
20
30
40
Fluorescence (a.u.)
Systematic Name: YBR176W
Time step Standard Deviation
4
5
6
6.5
7.0
7.5
8.0
8.5
Time step Mean
17
1
9
2
4
16
8
5
7
13
10
15
14
12
Time Step
16
12
8
4
Cluster
(Isolation)
1 (−)
2 (−)

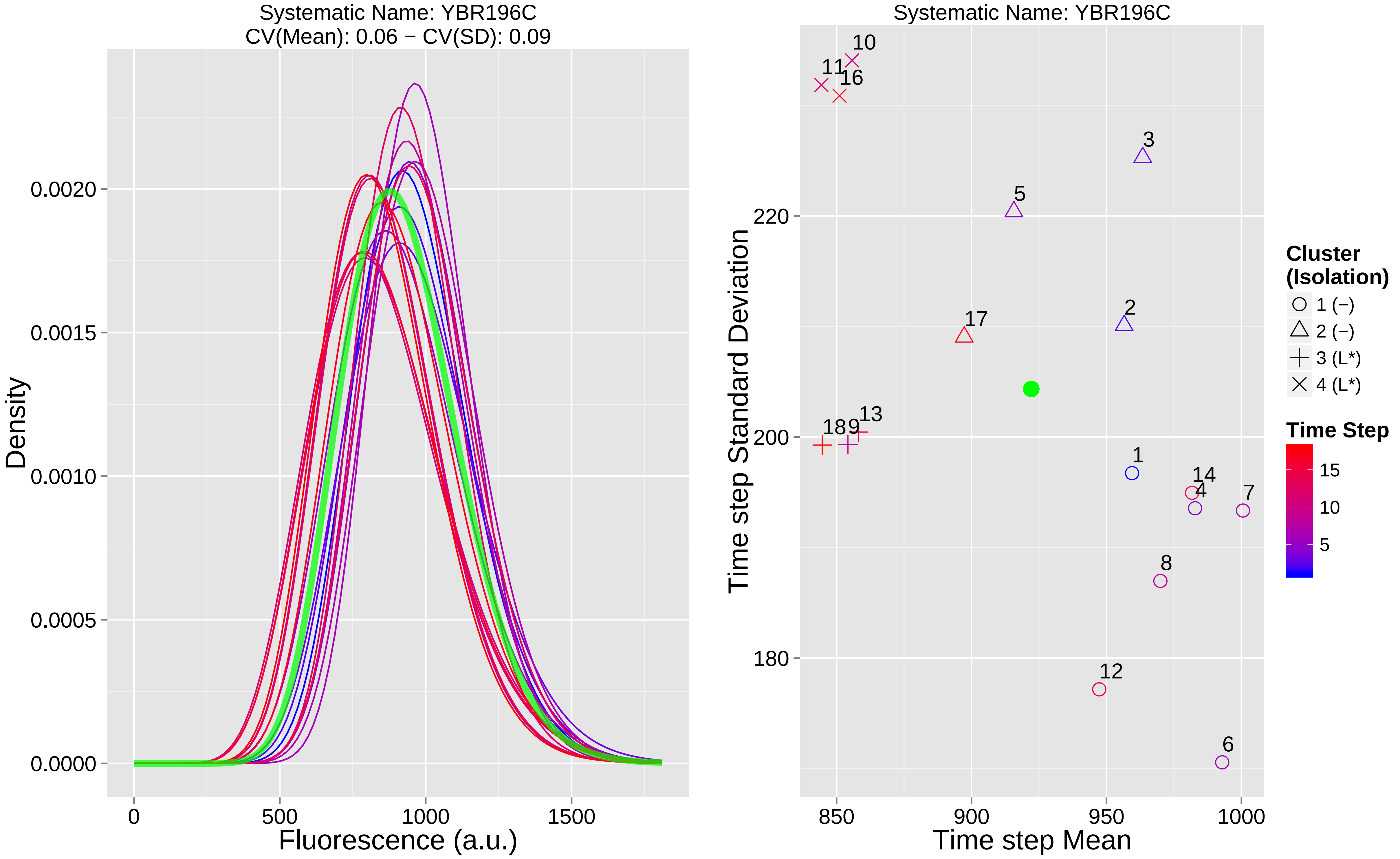
Systematic Name: YBR196C
CV(Mean): 0.06 − CV(SD): 0.09
Density
Fluorescence (a.u.)
Systematic Name: YBR196C
Time step Standard Deviation
Time step Mean
Cluster (Isolation)
1 (−)
2 (−)
3 (L*)
4 (L*)
Time Step

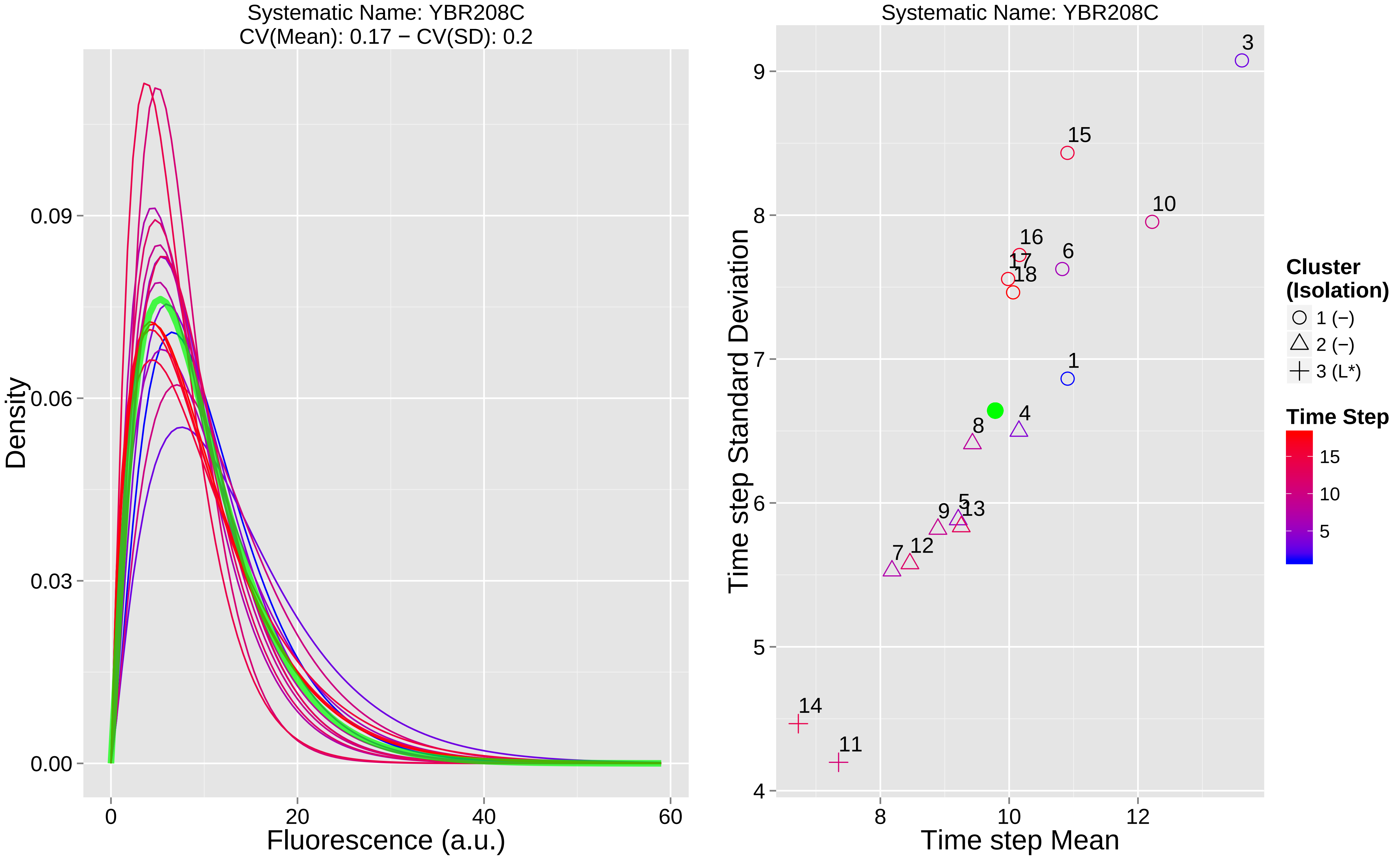

Systematic Name: YBR208C
CV(Mean): 0.17 − CV(SD): 0.2
Density
0.00
0.03
0.06
0.09
0
20
40
60
Fluorescence (a.u.)
Systematic Name: YBR208C
Time step Standard Deviation
4
5
6
7
8
9
8
10
12
Time step Mean
3
15
10
16
6
17
18
1
4
8
9
5
13
7
12
14
11
Cluster (Isolation)
1 (−)
2 (−)
3 (L*)
Time Step
15
10
5

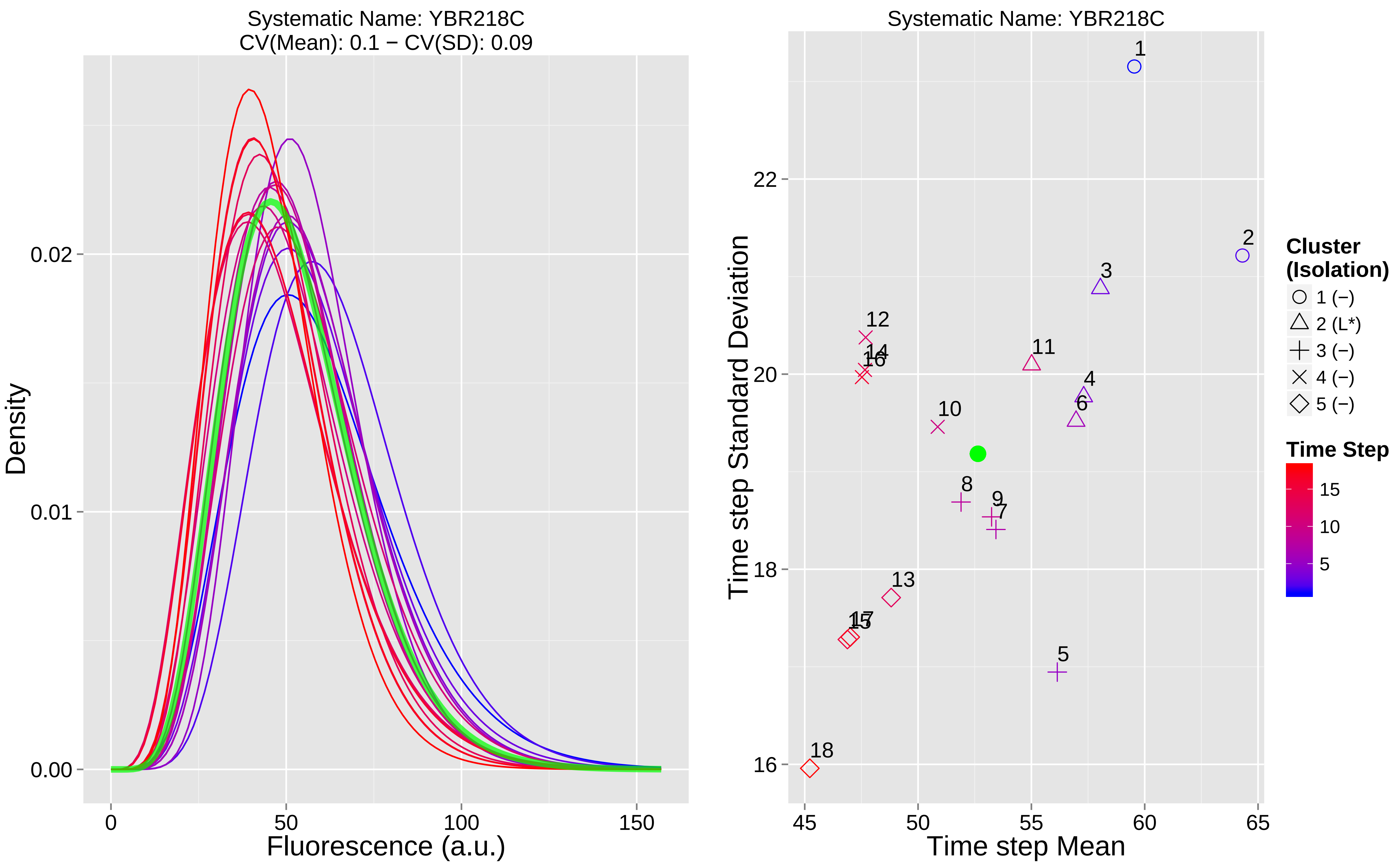

Systematic Name: YBR218C
CV(Mean): 0.1 − CV(SD): 0.09
Density
Fluorescence (a.u.)
0.00
0.01
0.02
0
50
100
150
Systematic Name: YBR218C
Time step Standard Deviation
Time step Mean
16
18
20
22
45
50
55
60
65
Cluster (Isolation)
1 (−)
2 (L*)
3 (−)
4 (−)
5 (−)
Time Step
15
10
5

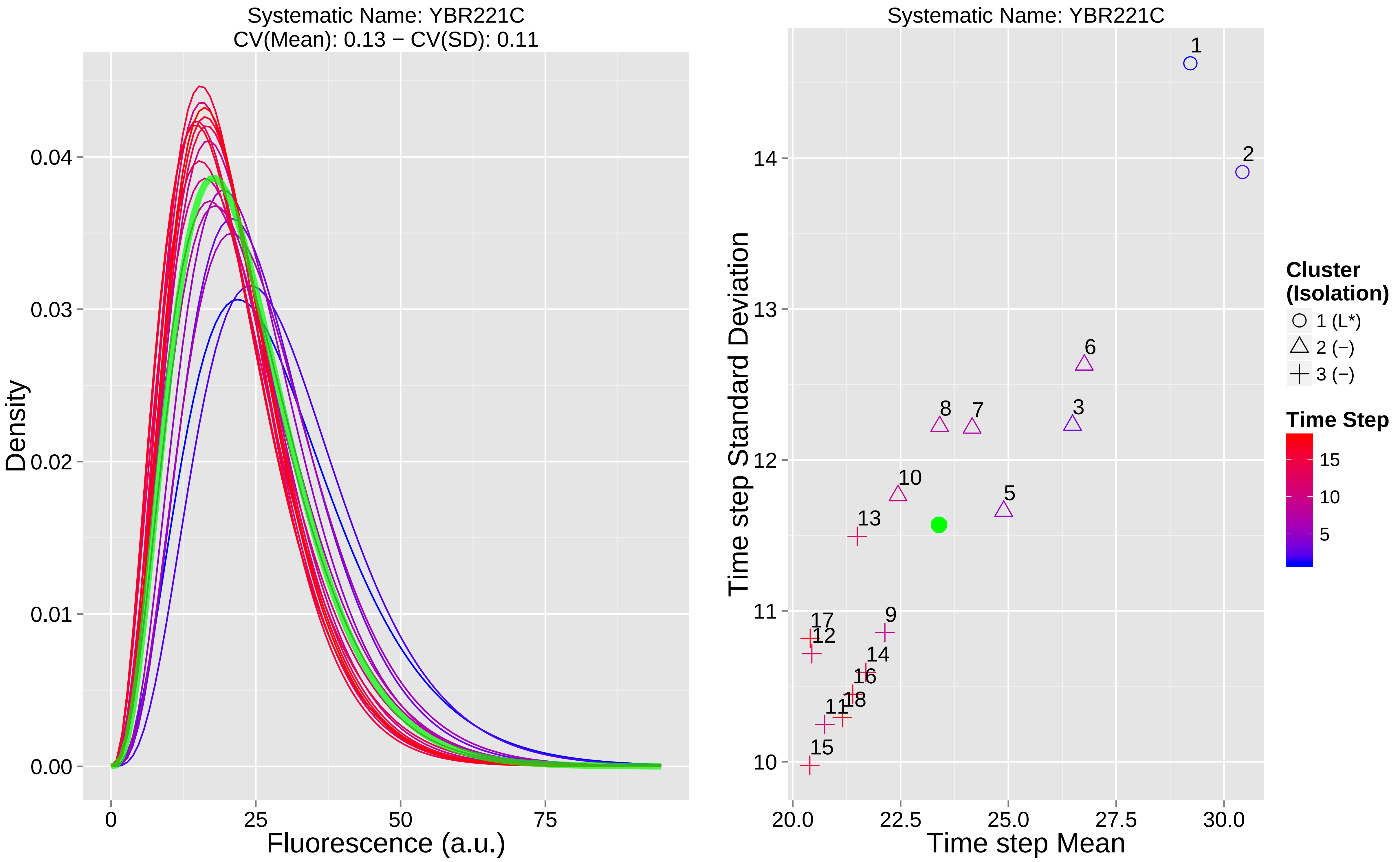

Systematic Name: YBR221C
CV(Mean): 0.13 − CV(SD): 0.11
Density
0.00
0.01
0.02
0.03
0.04
0
25
50
75
Fluorescence (a.u.)
Systematic Name: YBR221C
Time step Standard Deviation
10
11
12
13
14
20.0
22.5
25.0
27.5
30.0
Time step Mean
1
2
3
5
6
7
8
9
10
11
12
13
14
15
16
17
18
Cluster (Isolation)
1 (L*)
2 (−)
3 (−)
Time Step
15
10
5

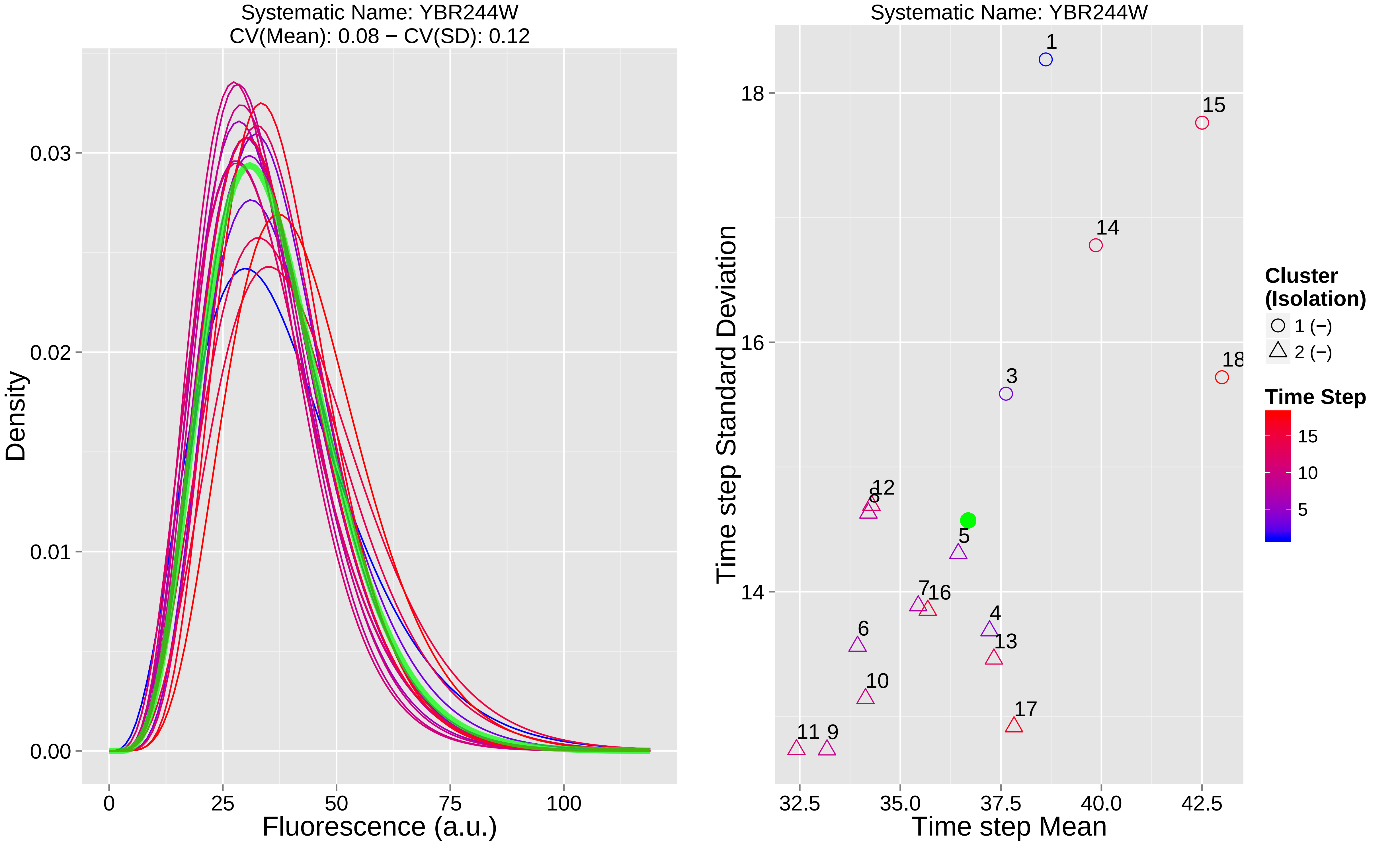

Systematic Name: YBR244W
CV(Mean): 0.08 − CV(SD): 0.12
Density
Fluorescence (a.u.)
0.00
0.01
0.02
0.03
0
25
50
75
100
Systematic Name: YBR244W
Time step Standard Deviation
Time step Mean
14
16
18
32.5
35.0
37.5
40.0
42.5
1
3
4
5
6
7
8
9
10
11
12
13
14
15
16
17
18
Cluster (Isolation)
1 (−)
2 (−)
Time Step
5
10
15

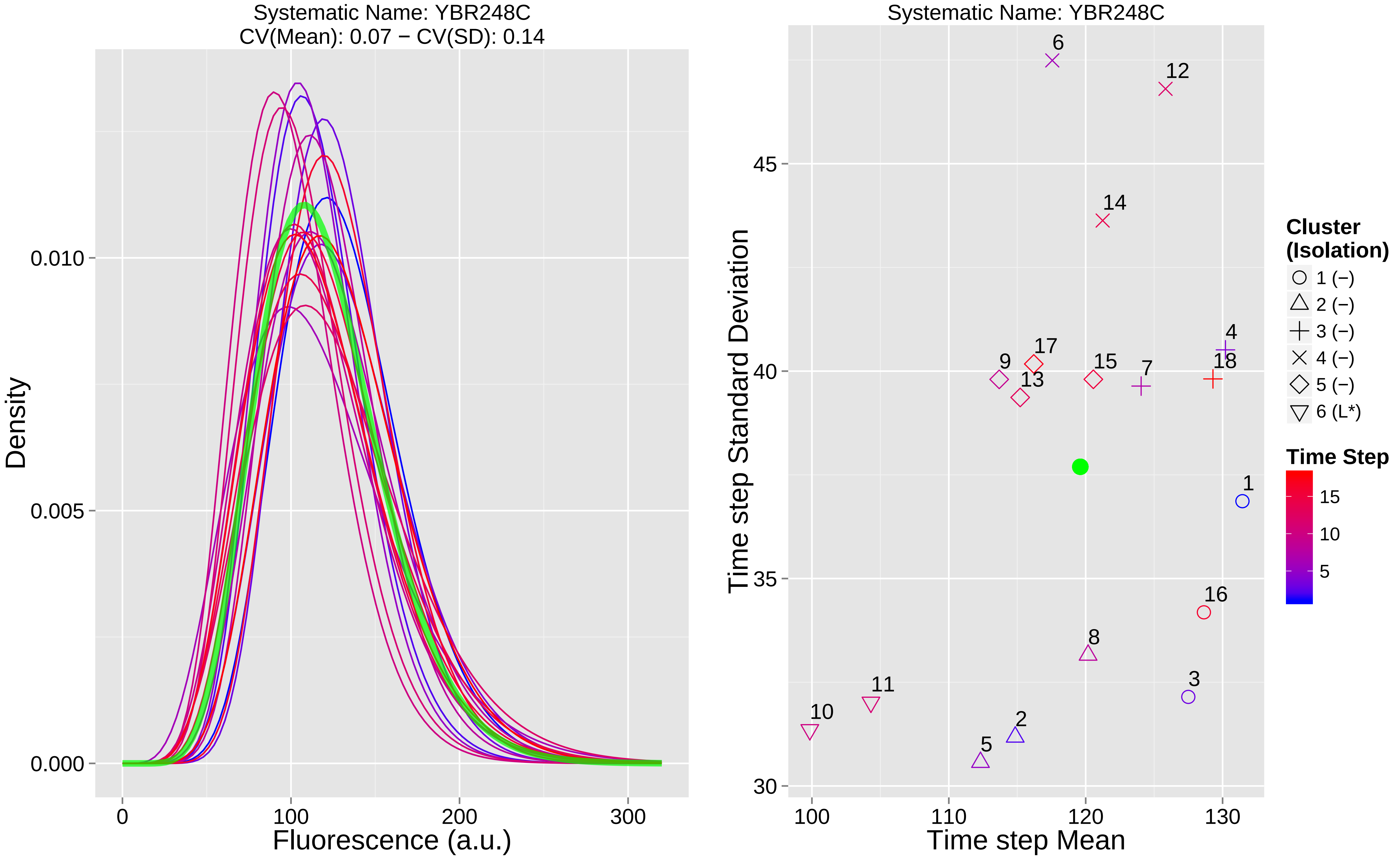
Systematic Name: YBR248C
CV(Mean): 0.07 − CV(SD): 0.14
Density
Fluorescence (a.u.)
0.000
0.005
0.010
0
100
200
300
Systematic Name: YBR248C
Time step Standard Deviation
Time step Mean
30
35
40
45
100
110
120
130
1
2
3
4
5
6
7
8
9
10
11
12
13
14
15
16
17
18
Cluster (Isolation)
1 (−)
2 (−)
3 (−)
4 (−)
5 (−)
6 (L*)
Time Step
5
10
15

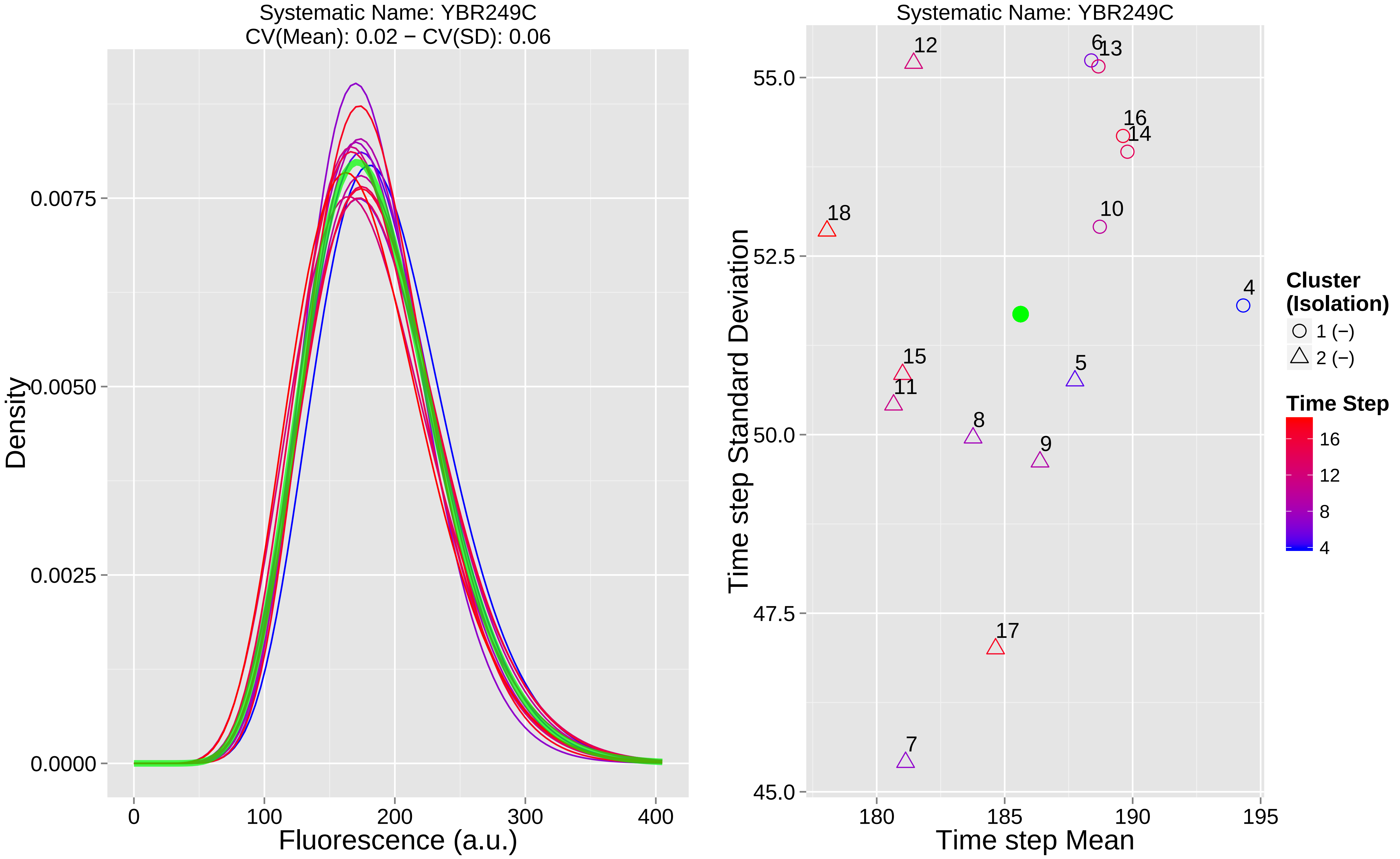

Systematic Name: YBR249C
CV(Mean): 0.02 − CV(SD): 0.06
Density
Fluorescence (a.u.)
0.0000
0.0025
0.0050
0.0075
0
100
200
300
400
Systematic Name: YBR249C
Time step Standard Deviation
Time step Mean
45.0
47.5
50.0
52.5
55.0
180
185
190
195
12
6
13
16
14
18
10
4
15
5
11
8
9
17
7
Cluster
(Isolation)
1 (−)
2 (−)
Time Step
16
12
8
4

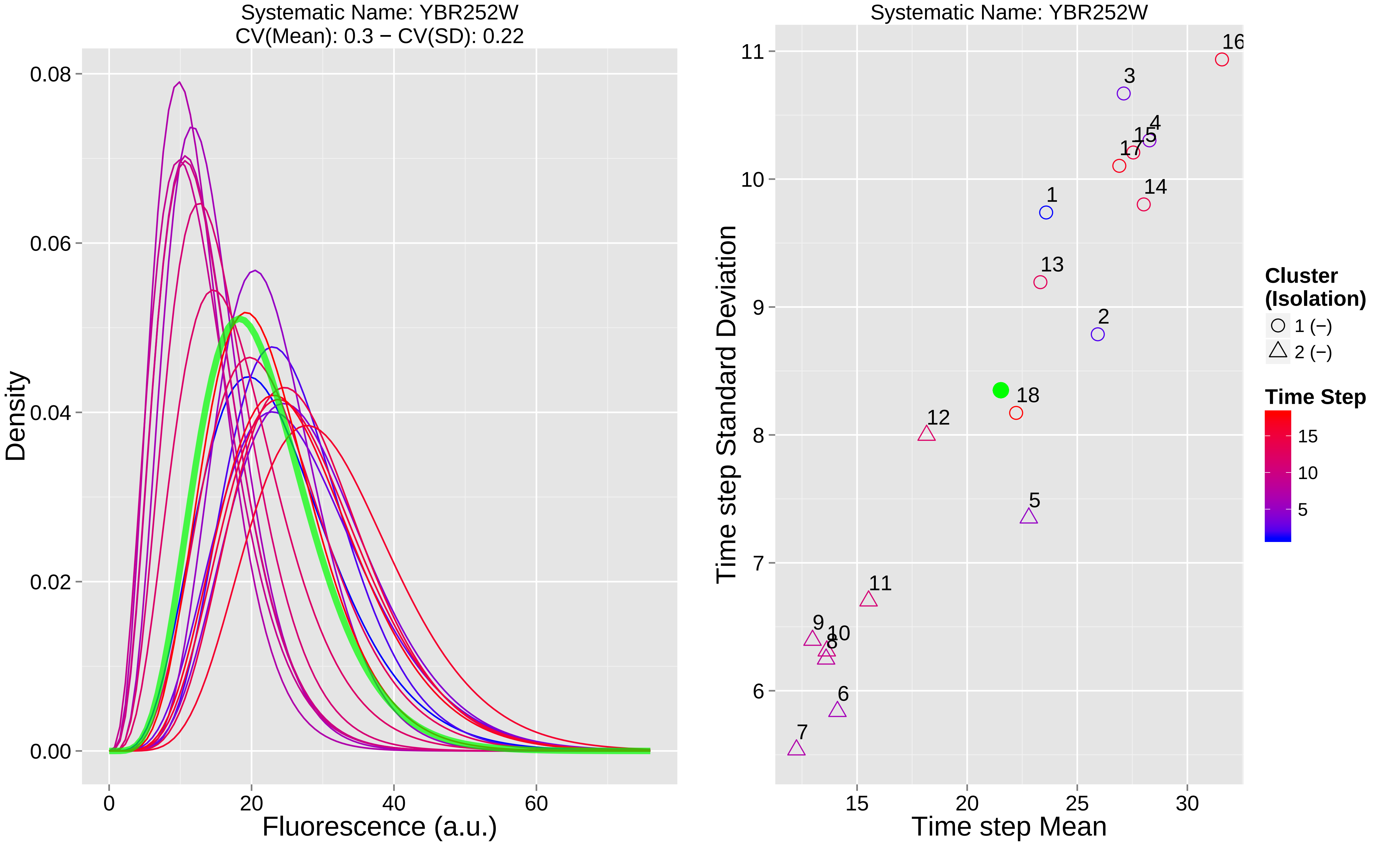

Systematic Name: YBR252W
CV(Mean): 0.3 − CV(SD): 0.22
Density
Fluorescence (a.u.)
0.00
0.02
0.04
0.06
0.08
0
20
40
60
Systematic Name: YBR252W
Time step Standard Deviation
Time step Mean
6
7
8
9
10
11
15
20
25
30
Cluster
(Isolation)
1 (−)
2 (−)
Time Step
5
10
15

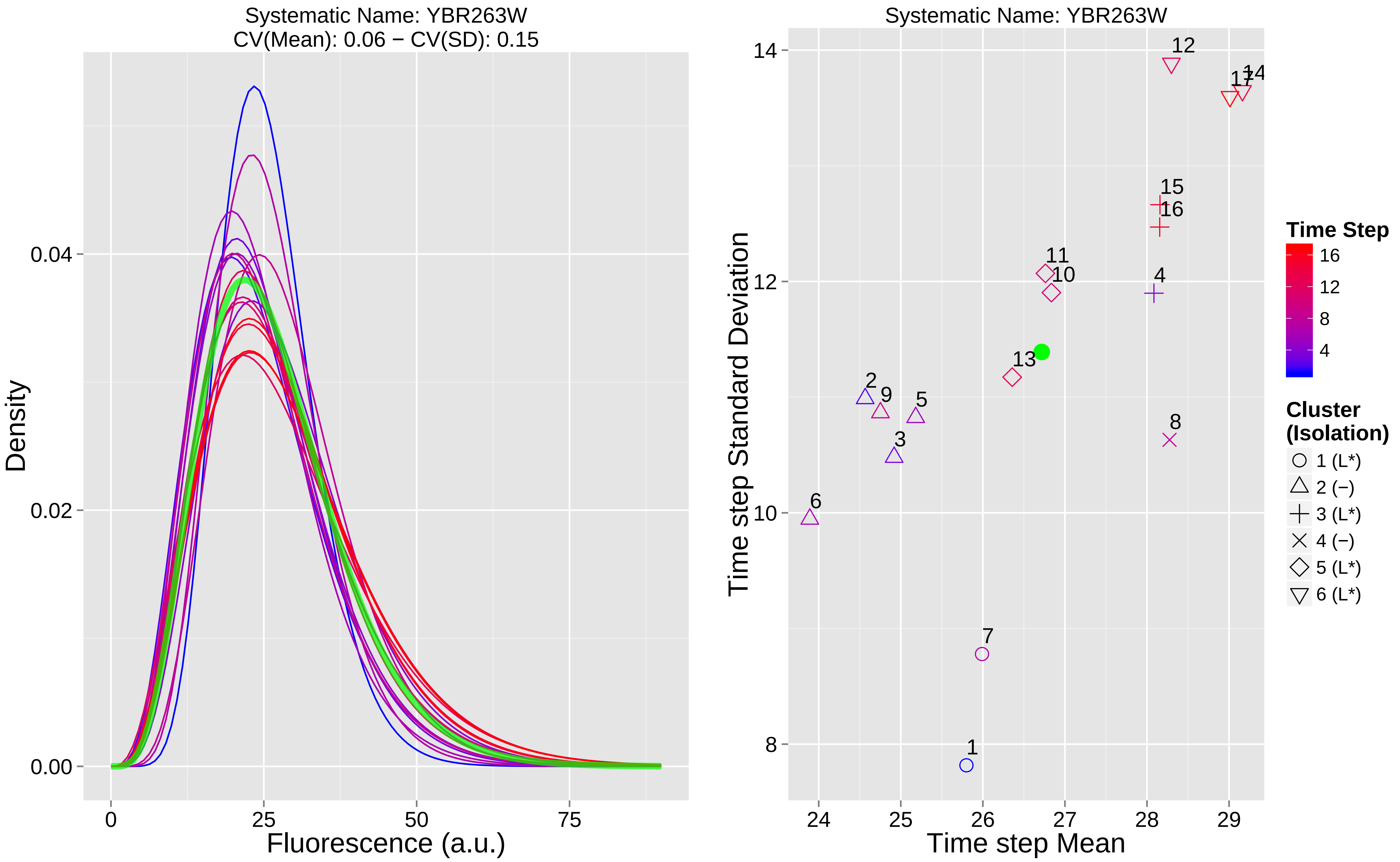
Systematic Name: YBR263W
CV(Mean): 0.06 − CV(SD): 0.15
Density
0.00
0.02
0.04
0
25
50
75
Fluorescence (a.u.)
Systematic Name: YBR263W
Time step Standard Deviation
Time step Mean
8
10
12
14
24
25
26
27
28
29
Time Step
16
12
8
4
Cluster
(Isolation)
1 (L*)
2 (−)
3 (L*)
4 (−)
5 (L*)
6 (L*)

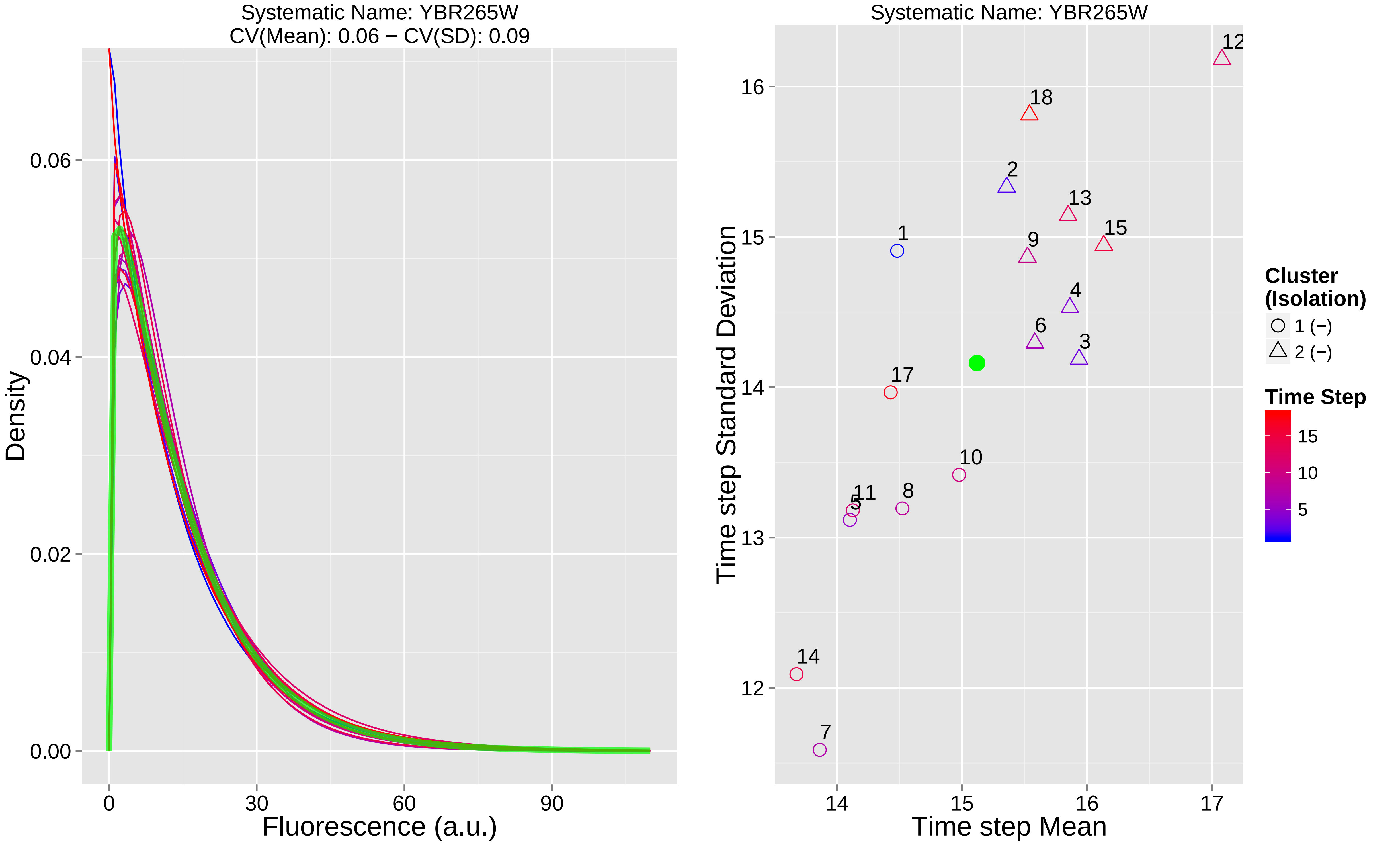

Systematic Name: YBR265W
CV(Mean): 0.06 − CV(SD): 0.09
Density
0.06
0.04
0.02
0.00
0
30
60
90
Fluorescence (a.u.)
Systematic Name: YBR265W
Time step Standard Deviation
16
15
14
13
12
14
15
16
17
Time step Mean
1
2
3
4
5
6
7
8
9
10
11
12
13
14
15
17
18
Cluster
(Isolation)
1 (−)
2 (−)
Time Step
15
10
5

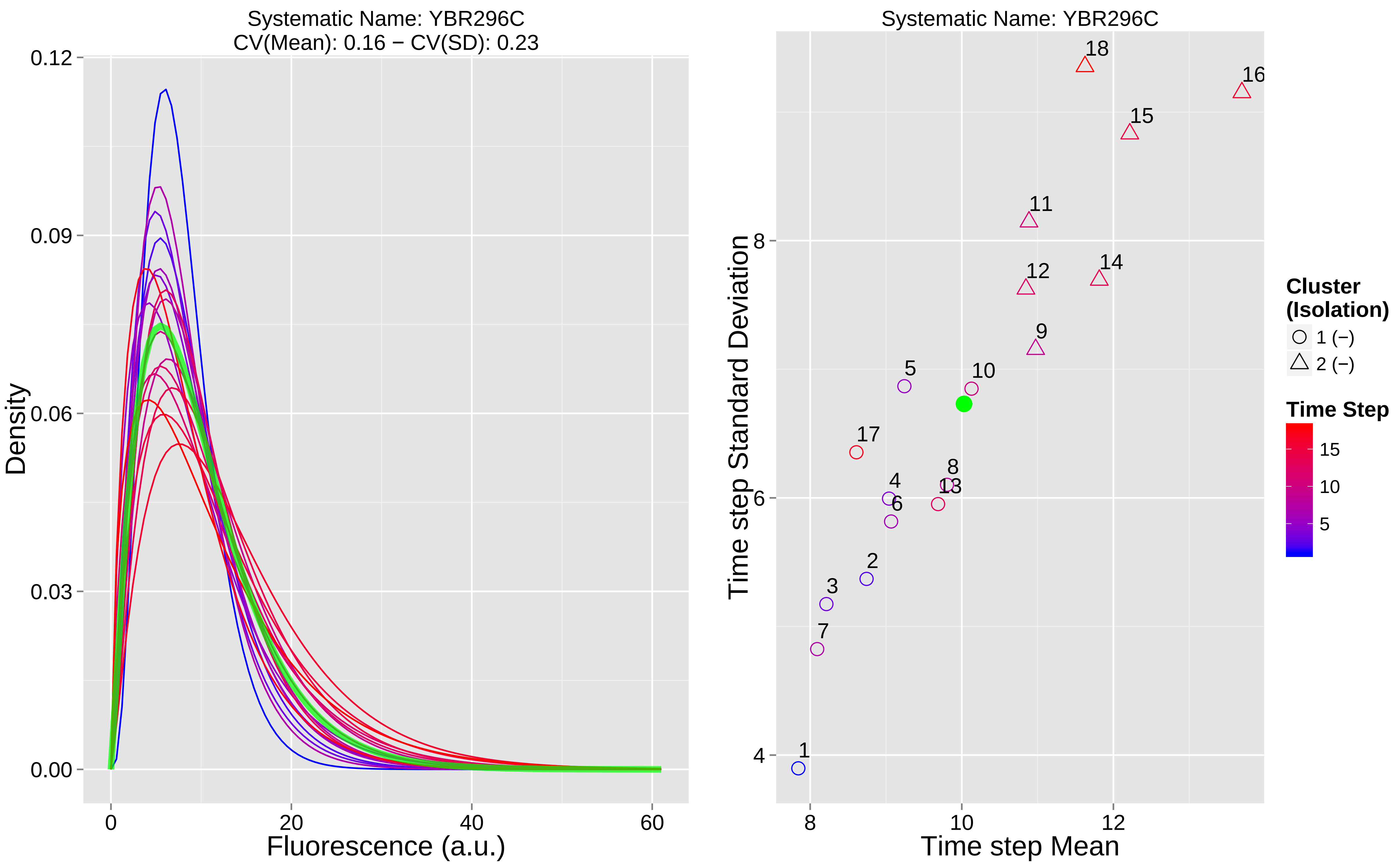

Systematic Name: YBR296C
CV(Mean): 0.16 − CV(SD): 0.23
Density
Fluorescence (a.u.)
0.12
0.09
0.06
0.03
0.00
0
20
40
60
Systematic Name: YBR296C
Time step Standard Deviation
Time step Mean
4
6
8
10
12
1
2
3
4
5
6
7
8
9
10
11
12
13
14
15
16
17
18
Cluster
(Isolation)
1 (−)
2 (−)
Time Step
15
10
5

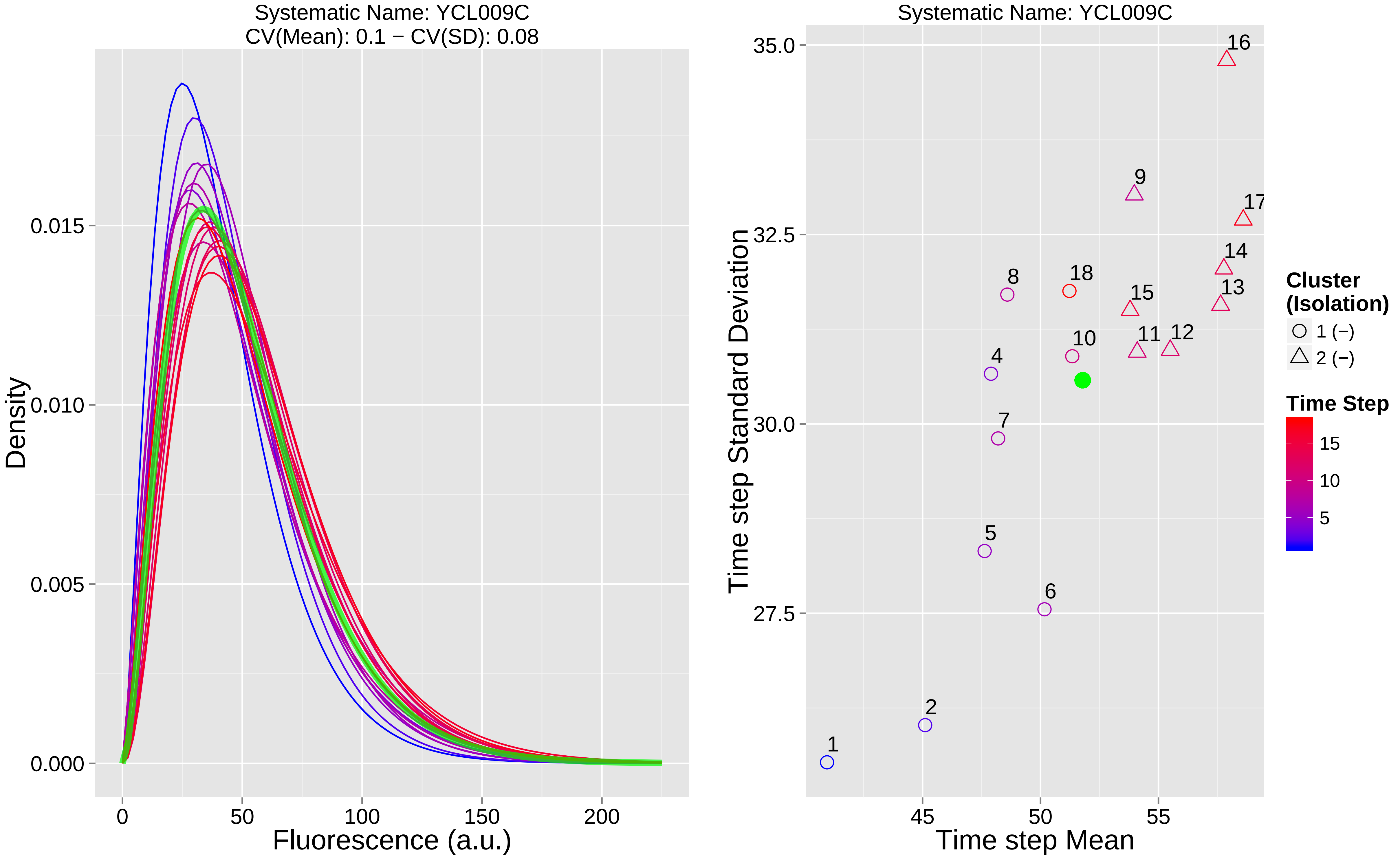
Systematic Name: YCL009C
CV(Mean): 0.1 − CV(SD): 0.08
Density
0.000
0.005
0.010
0.015
0
50
100
150
200
Fluorescence (a.u.)
Systematic Name: YCL009C
Time step Standard Deviation
27.5
30.0
32.5
35.0
45
50
55
Time step Mean
Cluster
(Isolation)
1 (−)
2 (−)
Time Step
5
10
15

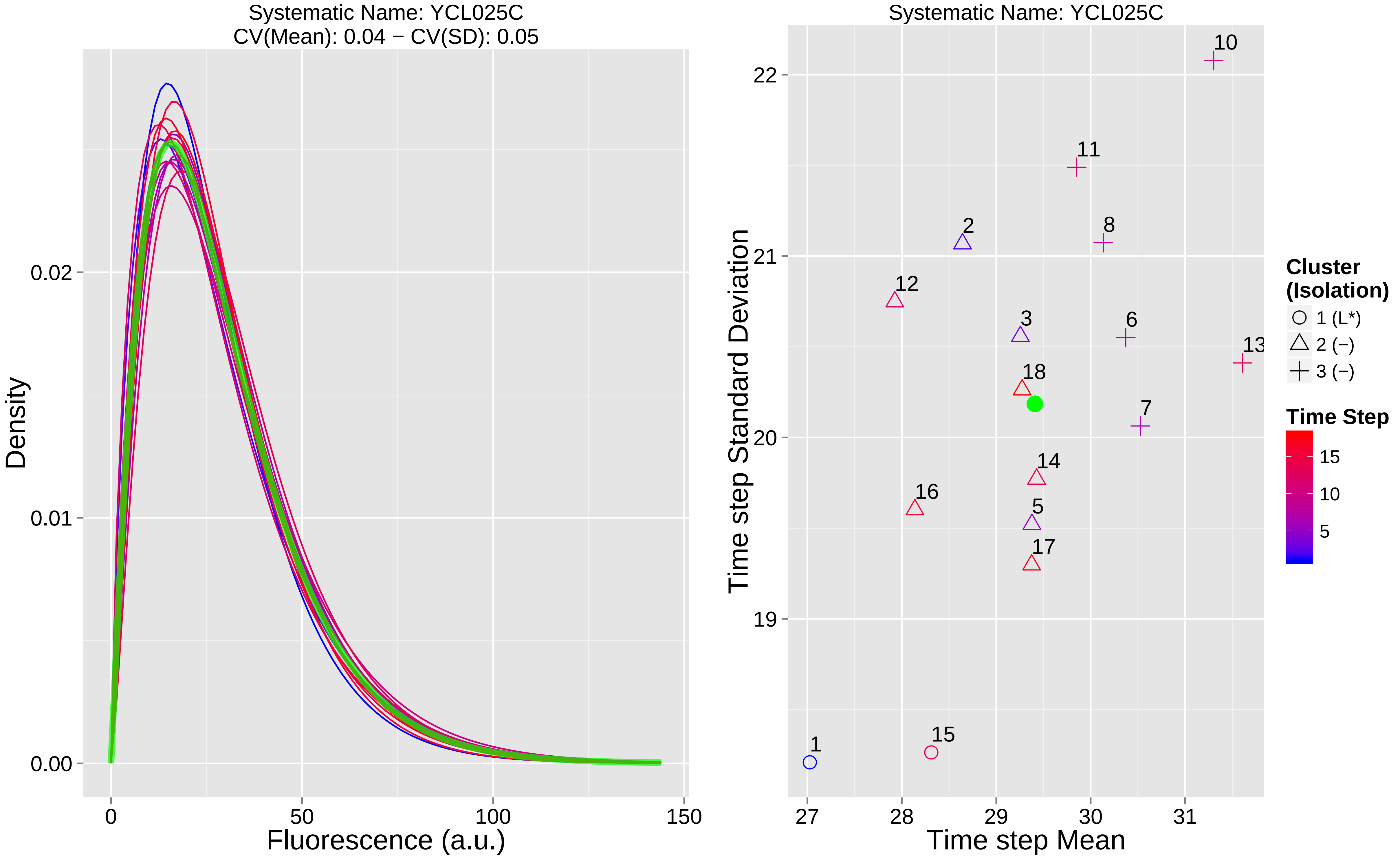

Systematic Name: YCL025C
CV(Mean): 0.04 − CV(SD): 0.05
Density
0.00
0.01
0.02
0
50
100
150
Fluorescence (a.u.)
Systematic Name: YCL025C
Time step Standard Deviation
19
20
21
22
27
28
29
30
31
Time step Mean
1
2
3
5
6
7
8
10
11
12
13
14
15
16
17
18
Cluster (Isolation)
1 (L*)
2 (−)
3 (−)
Time Step
5
10
15

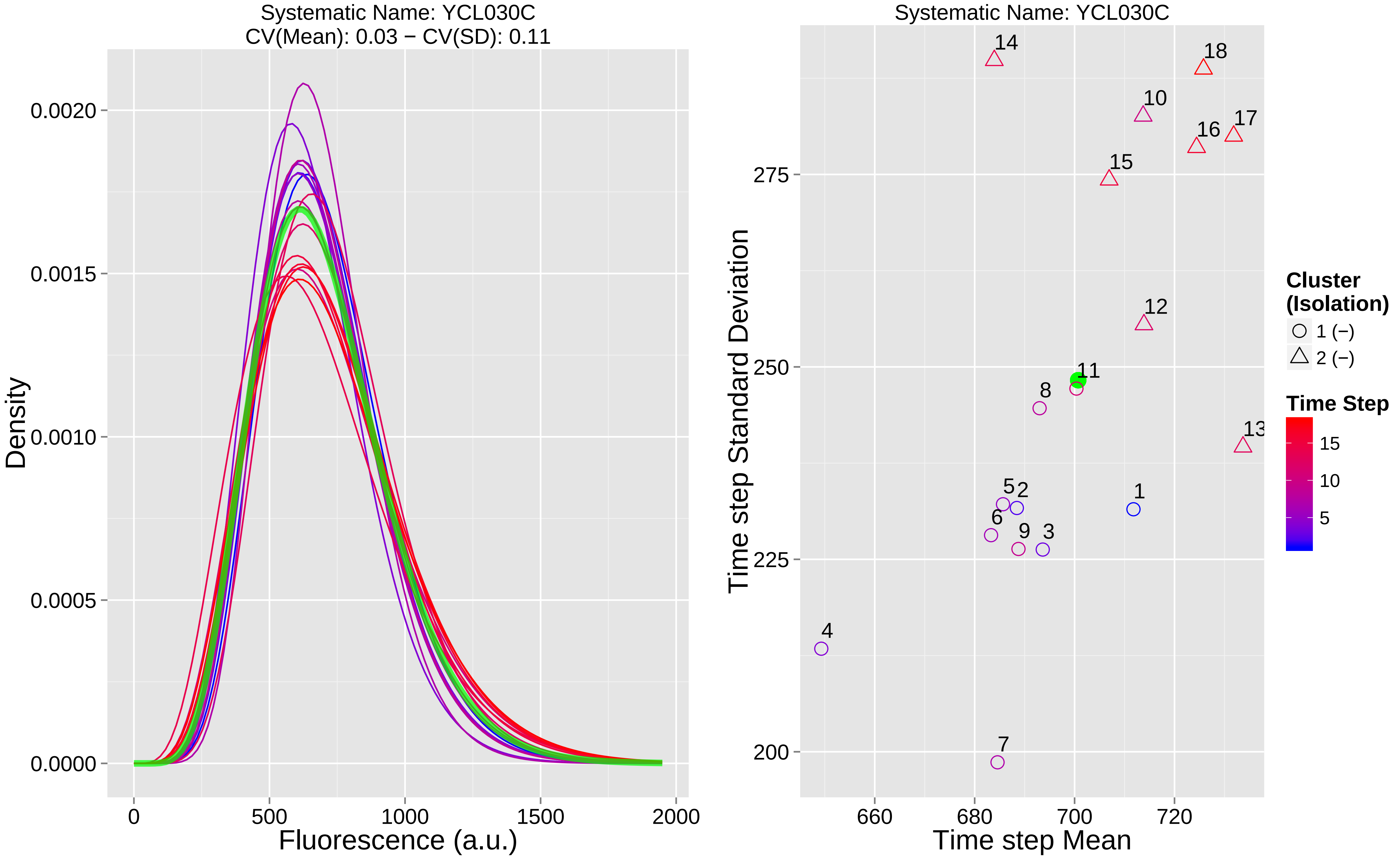

Systematic Name: YCL030C
CV(Mean): 0.03 − CV(SD): 0.11
Density
Fluorescence (a.u.)
0.0000
0.0005
0.0010
0.0015
0.0020
0
500
1000
1500
2000
Systematic Name: YCL030C
Time step Standard Deviation
Time step Mean
200
225
250
275
660
680
700
720
Cluster (Isolation)
1 (−)
2 (−)
Time Step
5
10
15

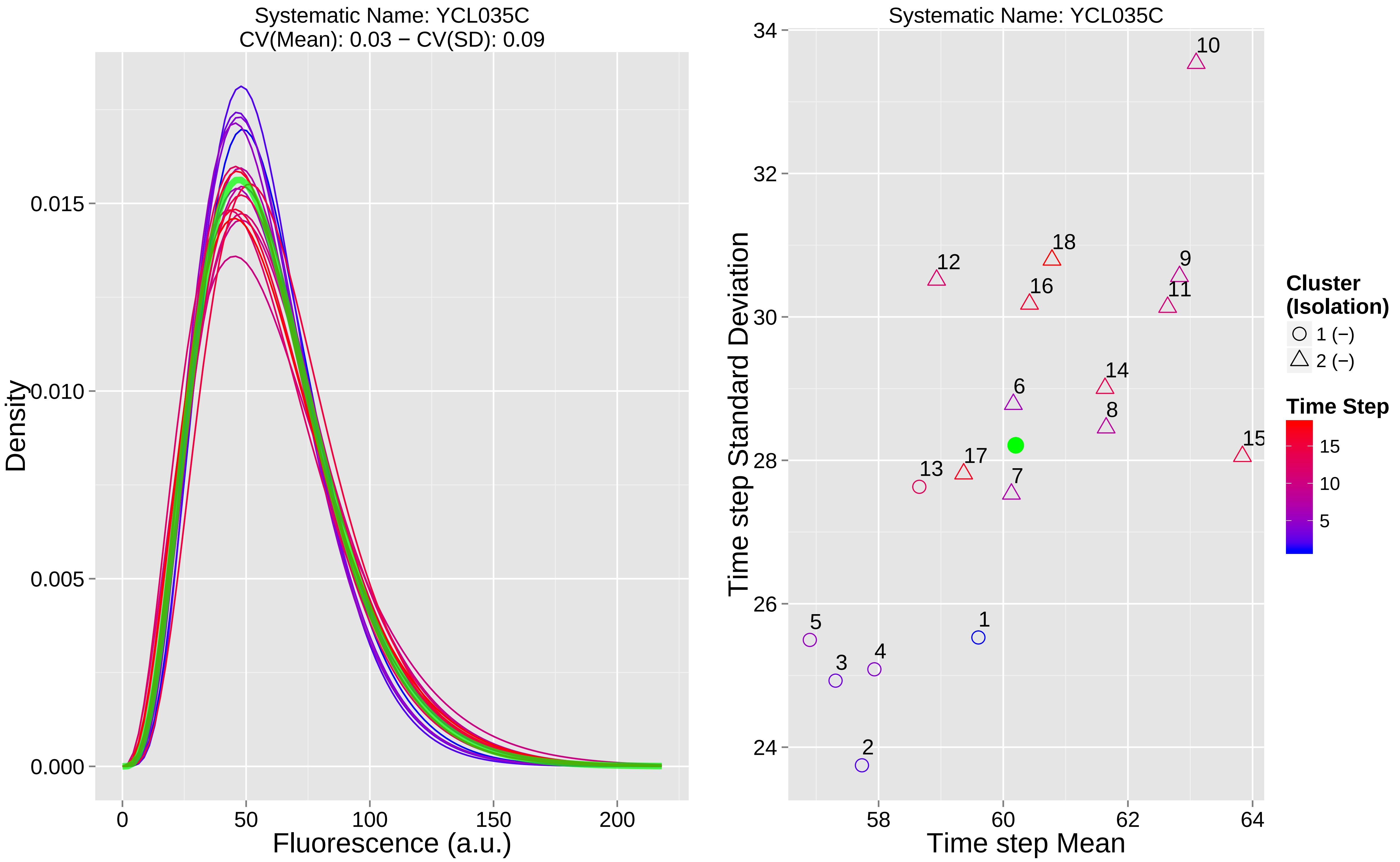

Systematic Name: YCL035C
CV(Mean): 0.03 − CV(SD): 0.09
Density
0.000
0.005
0.010
0.015
0
50
100
150
200
Fluorescence (a.u.)
Systematic Name: YCL035C
Time step Standard Deviation
24
26
28
30
32
34
58
60
62
64
Time step Mean
1
2
3
4
5
6
7
8
9
10
11
12
13
14
15
16
17
18
Cluster
(Isolation)
1 (−)
2 (−)
Time Step
15
10
5

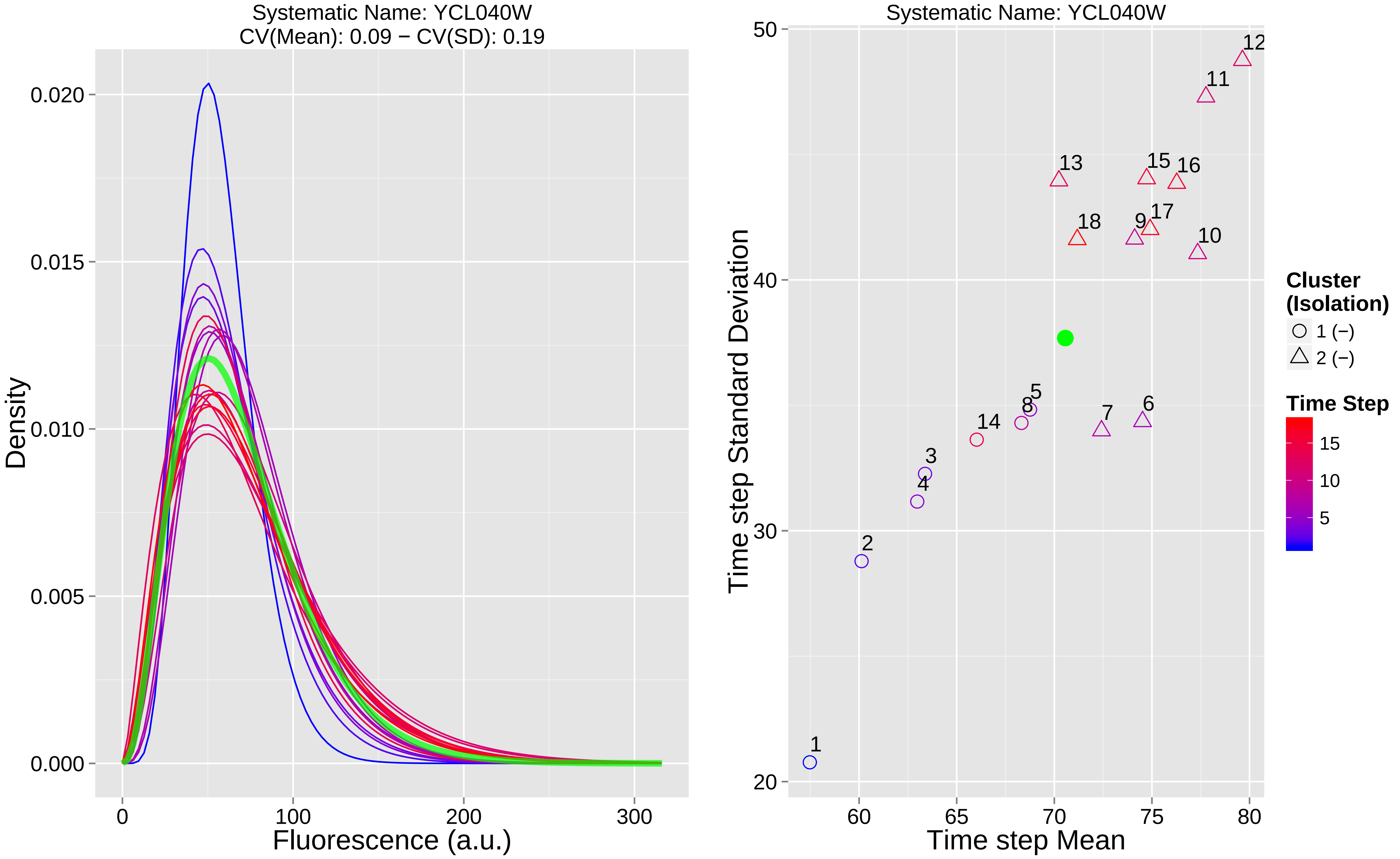
Systematic Name: YCL040W
CV(Mean): 0.09 − CV(SD): 0.19
Density
0.000
0.005
0.010
0.015
0.020
0
100
200
300
Fluorescence (a.u.)
Systematic Name: YCL040W
Time step Standard Deviation
20
30
40
50
60
65
70
75
80
Time step Mean
1
2
3
4
5
6
7
8
9
10
11
12
13
14
15
16
17
18
Cluster
(Isolation)
1 (−)
2 (−)
Time Step
15
10
5

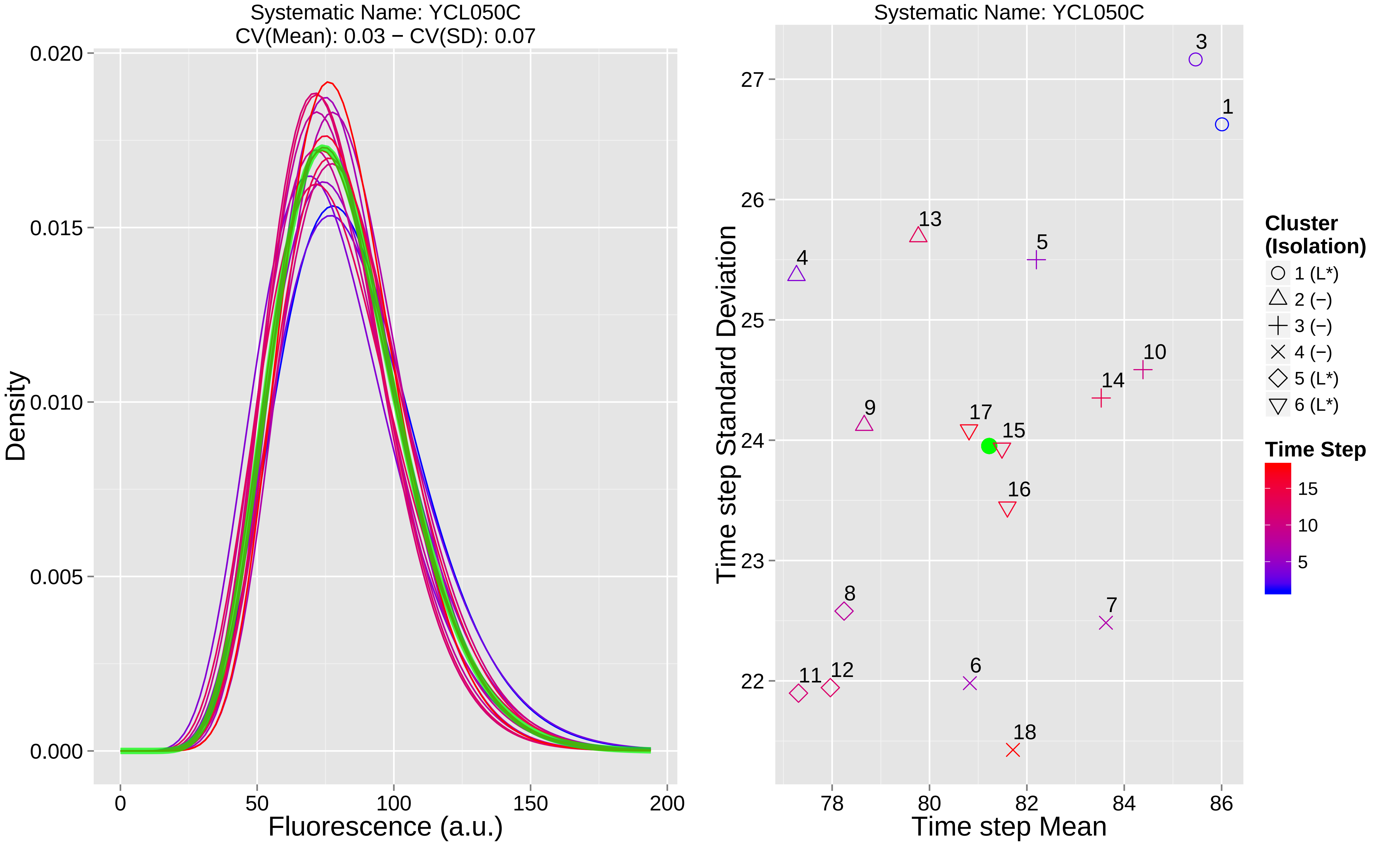
Systematic Name: YCL050C
CV(Mean): 0.03 − CV(SD): 0.07
Density
Fluorescence (a.u.)
0.000
0.005
0.010
0.015
0.020
0
50
100
150
200
Systematic Name: YCL050C
Time step Standard Deviation
Time step Mean
22
23
24
25
26
27
78
80
82
84
86
1
2
3
4
5
6
7
8
9
10
11
12
13
14
15
16
17
18
Cluster
(Isolation)
1 (L*)
2 (−)
3 (−)
4 (−)
5 (L*)
6 (L*)
Time Step
15
10
5

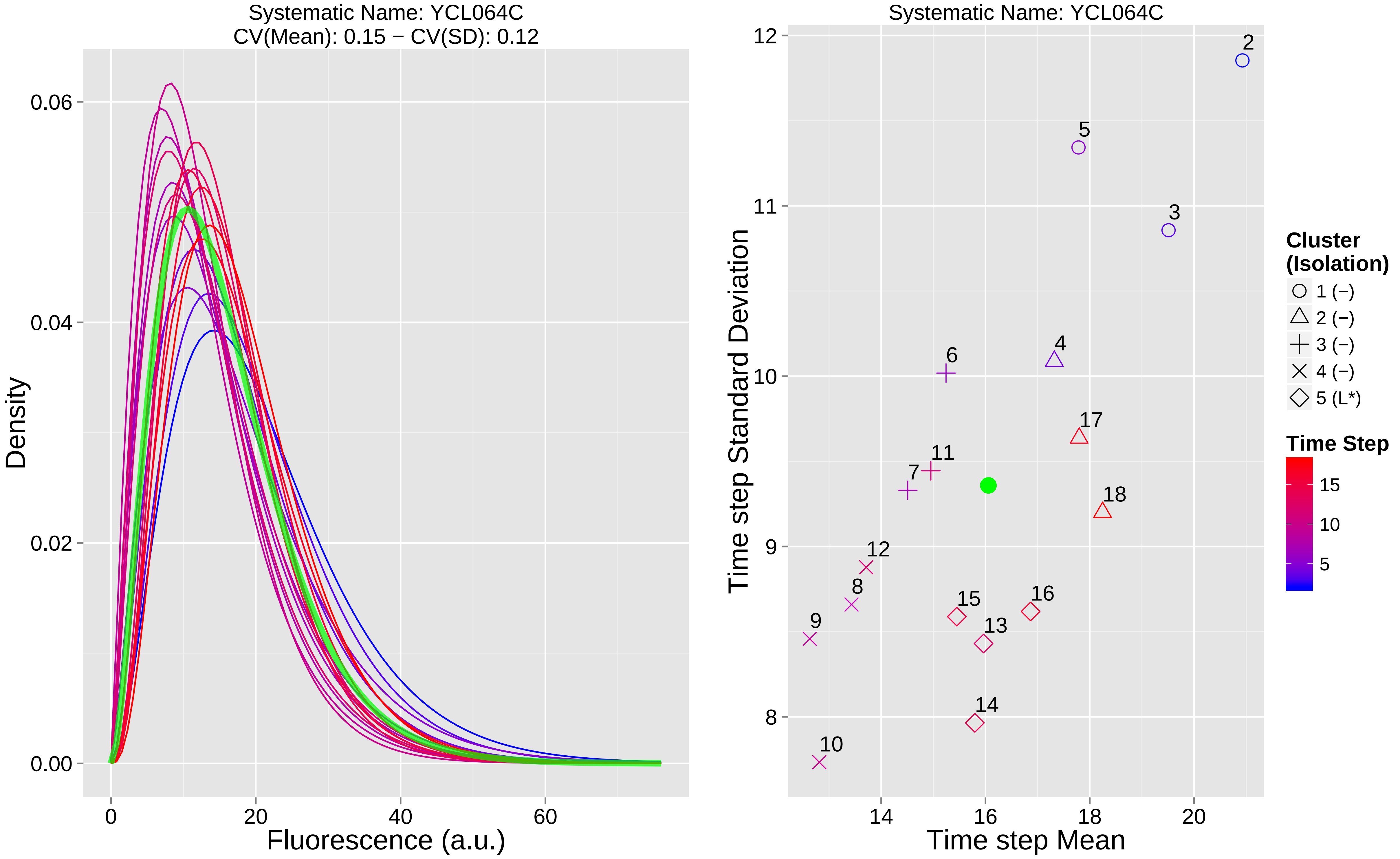

Systematic Name: YCL064C
CV(Mean): 0.15 − CV(SD): 0.12
Density
0.06
0.04
0.02
0.00
0
20
40
60
Fluorescence (a.u.)
Systematic Name: YCL064C
Time step Standard Deviation
12
11
10
9
8
14
16
18
20
Time step Mean
2
5
3
4
6
17
11
7
18
12
8
15
16
9
13
14
10
Cluster (Isolation)
1 (−)
2 (−)
3 (−)
4 (−)
5 (L*)
Time Step
15
10
5

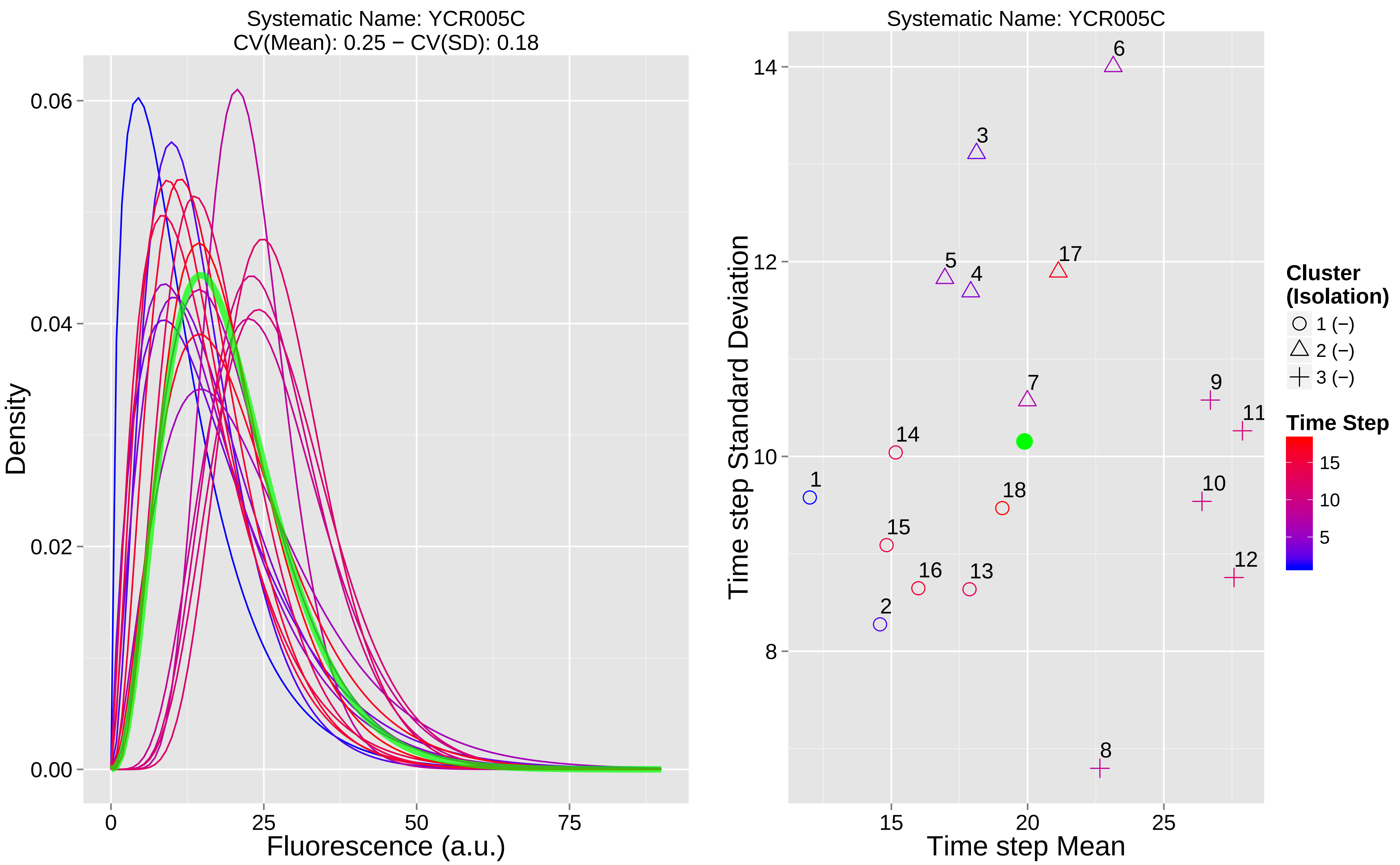

Systematic Name: YCR005C
CV(Mean): 0.25 − CV(SD): 0.18
Density
0.06
0.04
0.02
0.00
0
25
50
75
Fluorescence (a.u.)
Systematic Name: YCR005C
Time step Standard Deviation
14
12
10
8
15
20
25
Time step Mean
1
2
3
4
5
6
7
8
9
10
11
12
13
14
15
16
17
18
Cluster (Isolation)
1 (−)
2 (−)
3 (−)
Time Step
15
10
5

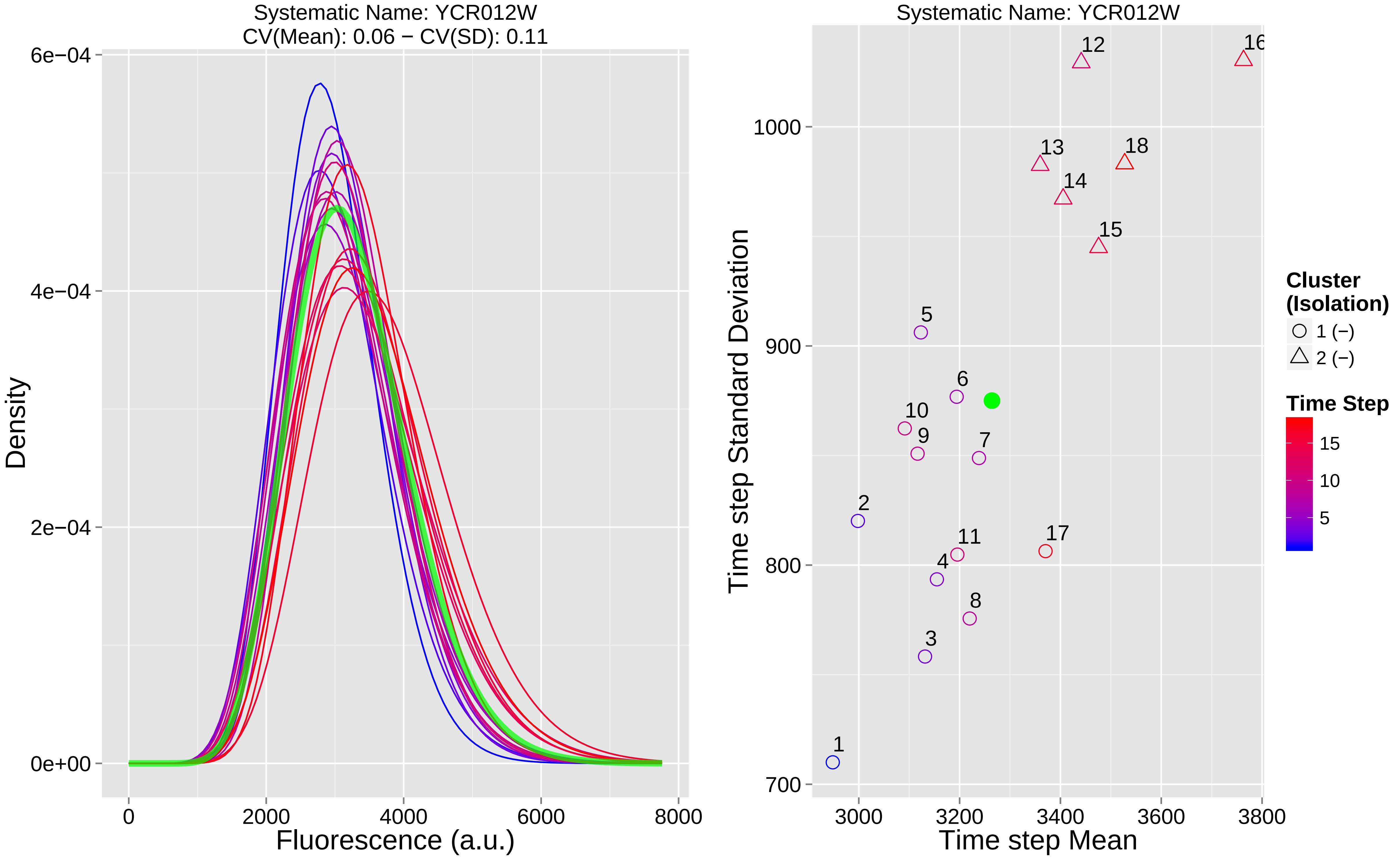
Systematic Name: YCR012W
CV(Mean): 0.06 − CV(SD): 0.11
Density
Fluorescence (a.u.)
6e−04
4e−04
2e−04
0e+00
0
2000
4000
6000
8000
Systematic Name: YCR012W
Time step Standard Deviation
Time step Mean
1000
900
800
700
3000
3200
3400
3600
3800
1
2
3
4
5
6
7
8
9
10
11
12
13
14
15
16
17
18
Cluster
(Isolation)
1 (−)
2 (−)
Time Step
15
10
5

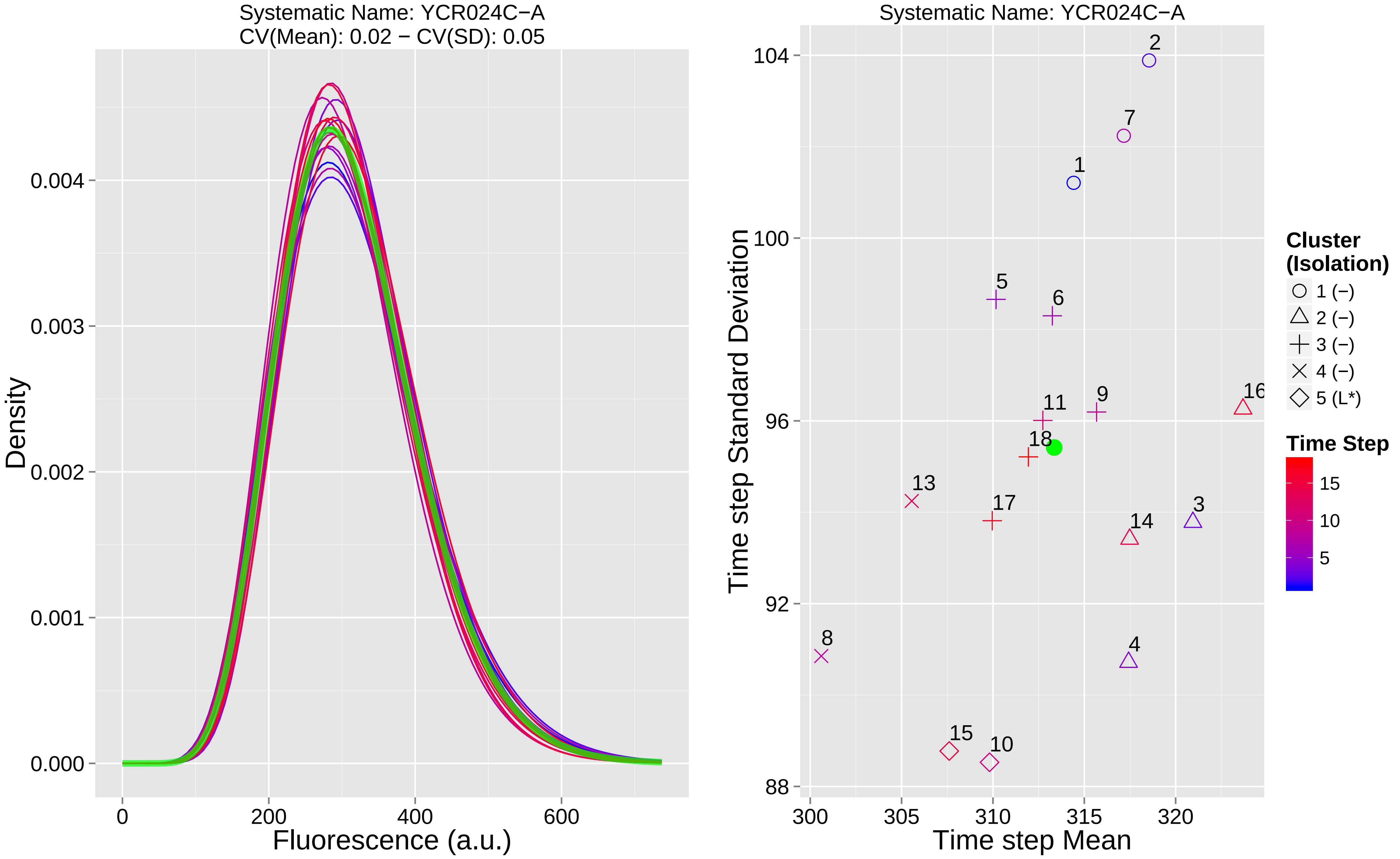
Systematic Name: YCR024C−A
CV(Mean): 0.02 − CV(SD): 0.05
Density
0.000
0.001
0.002
0.003
0.004
0
200
400
600
Fluorescence (a.u.)
Systematic Name: YCR024C−A
Time step Standard Deviation
88
92
96
100
104
300
305
310
315
320
Time step Mean
1
2
3
4
5
6
7
8
9
10
11
13
14
15
16
17
18
Cluster
(Isolation)
1 (−)
2 (−)
3 (−)
4 (−)
5 (L*)
Time Step
5
10
15

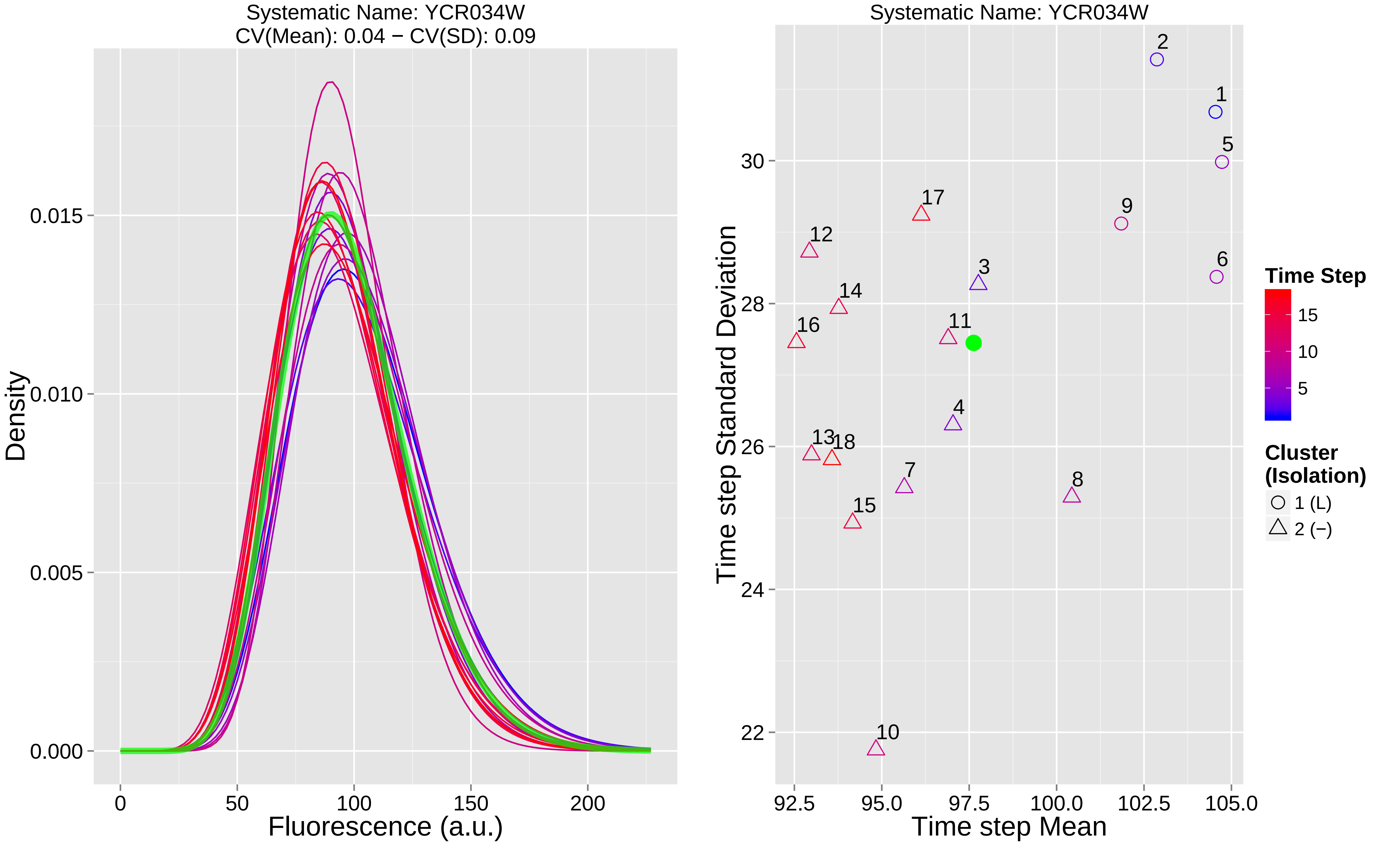

Systematic Name: YCR034W
CV(Mean): 0.04 − CV(SD): 0.09
Density
Fluorescence (a.u.)
0.000
0.005
0.010
0.015
0
50
100
150
200
Systematic Name: YCR034W
Time step Standard Deviation
Time step Mean
22
24
26
28
30
92.5
95.0
97.5
100.0
102.5
105.0
1
2
3
4
5
6
7
8
9
10
11
12
13
14
15
16
17
18
Time Step
15
10
5
Cluster
(Isolation)
1 (L)
2 (−)

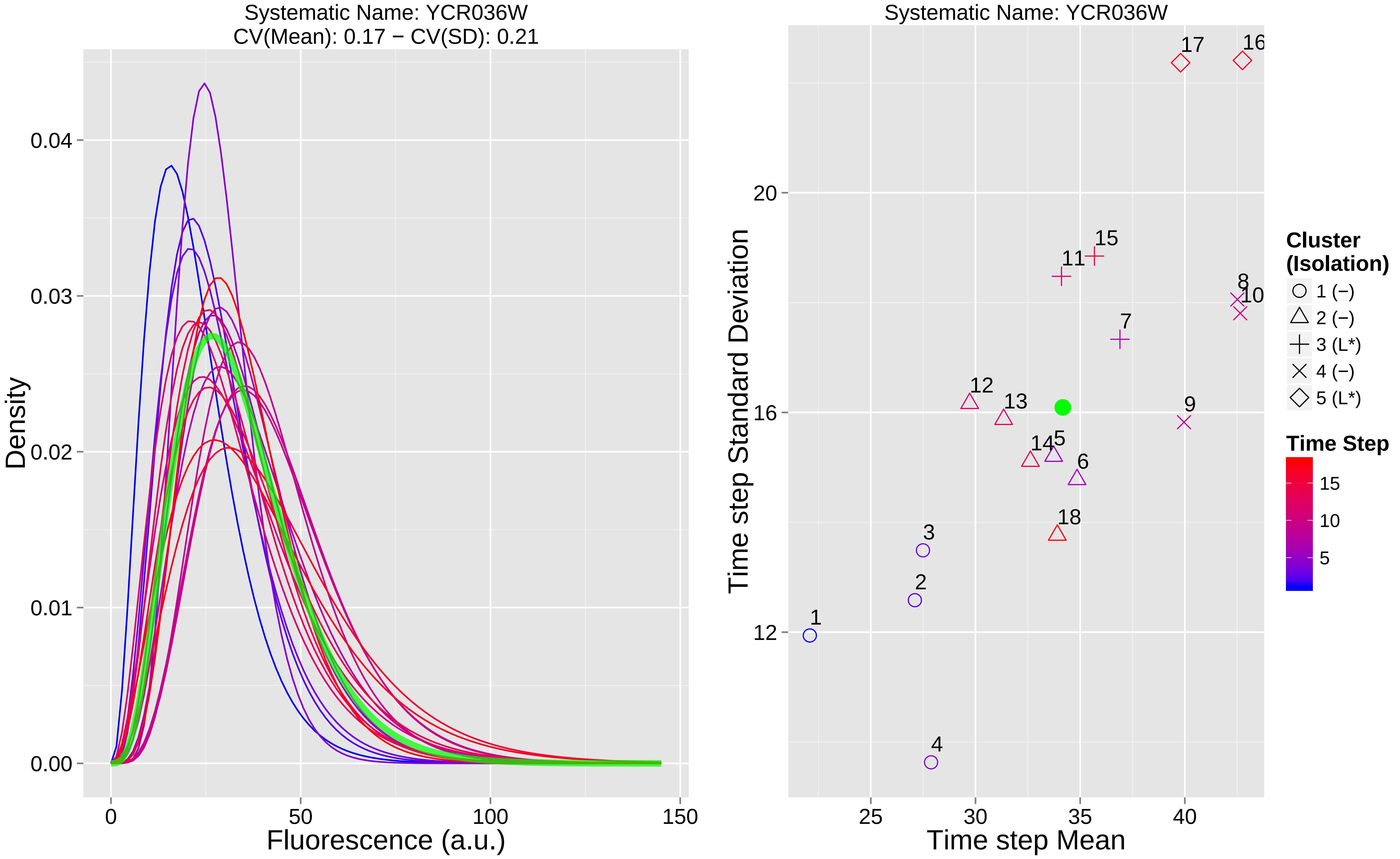

Systematic Name: YCR036W
CV(Mean): 0.17 − CV(SD): 0.21
Density
0.00
0.01
0.02
0.03
0.04
0
50
100
150
Fluorescence (a.u.)
Systematic Name: YCR036W
Time step Standard Deviation
12
16
20
25
30
35
40
Time step Mean
1
2
3
4
5
6
7
8
9
10
11
12
13
14
15
16
17
18
Cluster (Isolation)
1 (−)
2 (−)
3 (L*)
4 (−)
5 (L*)
Time Step
5
10
15

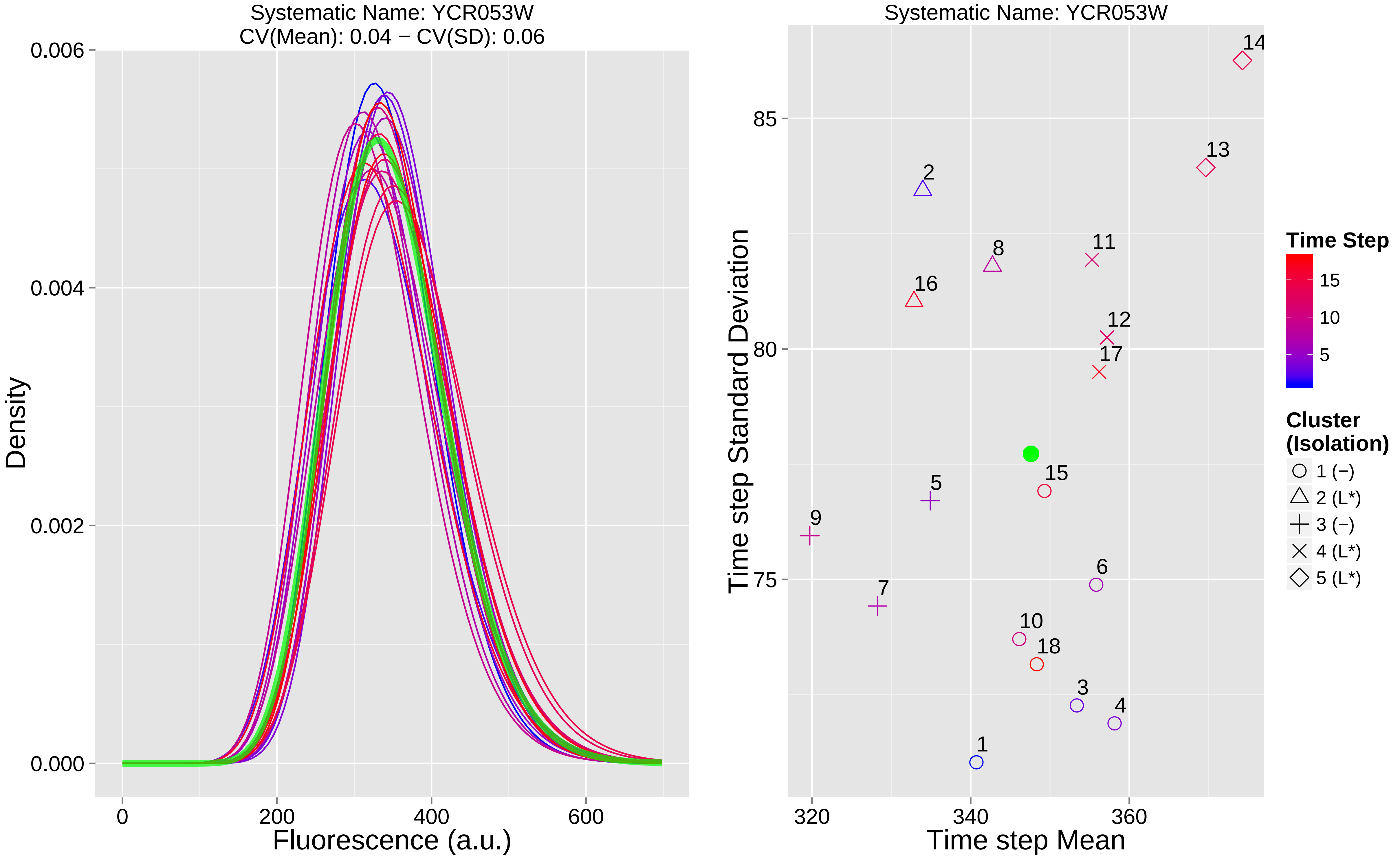
Systematic Name: YCR053W
CV(Mean): 0.04 − CV(SD): 0.06
Density
Fluorescence (a.u.)
0.006
0.004
0.002
0.000
0
200
400
600
Systematic Name: YCR053W
Time step Standard Deviation
Time step Mean
85
80
75
320
340
360
Time Step
15
10
5
Cluster (Isolation)
1 (−)
2 (L*)
3 (−)
4 (L*)
5 (L*)

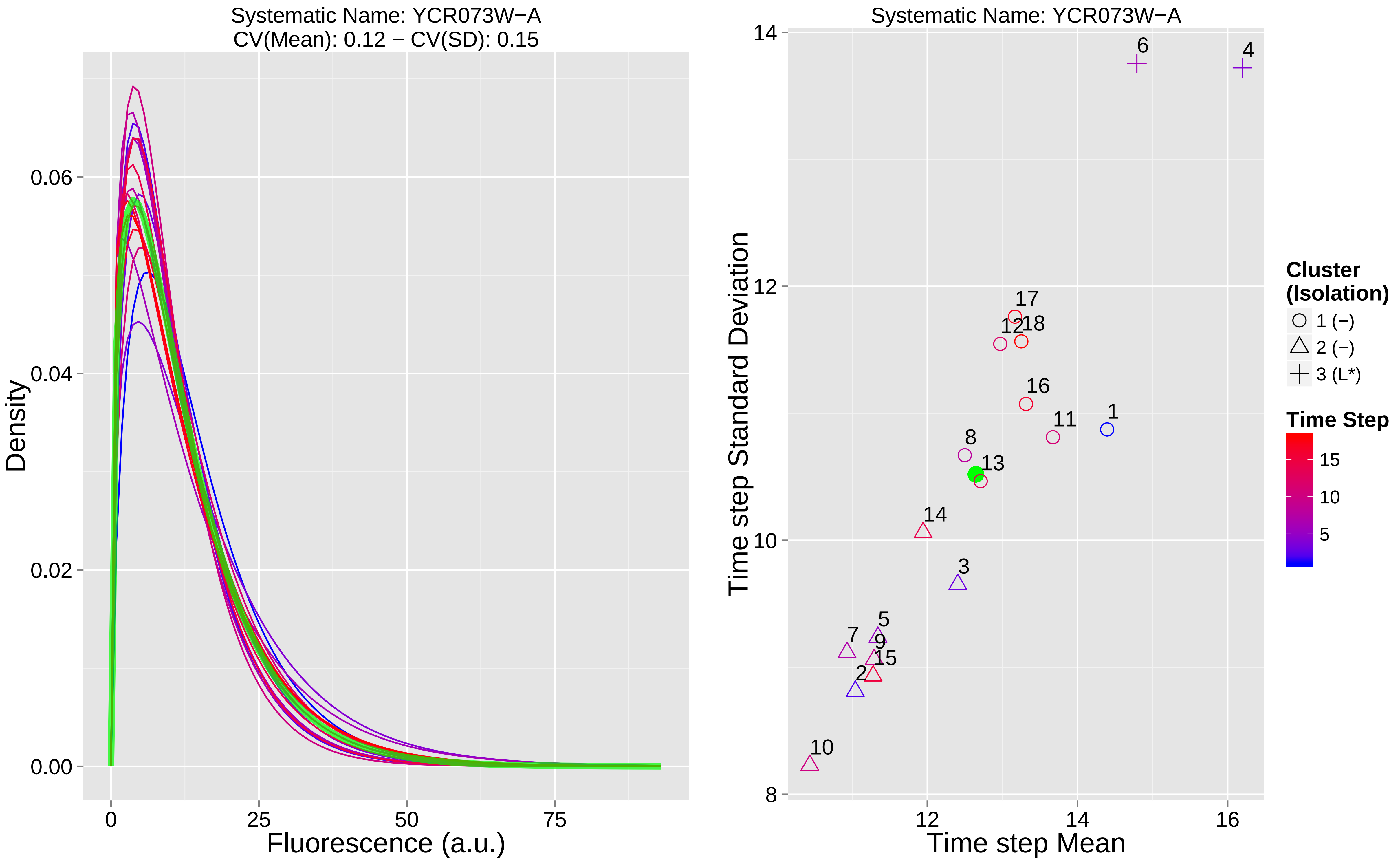

Systematic Name: YCR073W−A
CV(Mean): 0.12 − CV(SD): 0.15
Density
Fluorescence (a.u.)
0.00
0.02
0.04
0.06
0
25
50
75
Systematic Name: YCR073W−A
Time step Standard Deviation
Time step Mean
8
10
12
14
12
14
16
1
2
3
4
5
6
7
8
9
10
11
12
13
14
15
16
17
18
Cluster (Isolation)
1 (−)
2 (−)
3 (L*)
Time Step
5
10
15

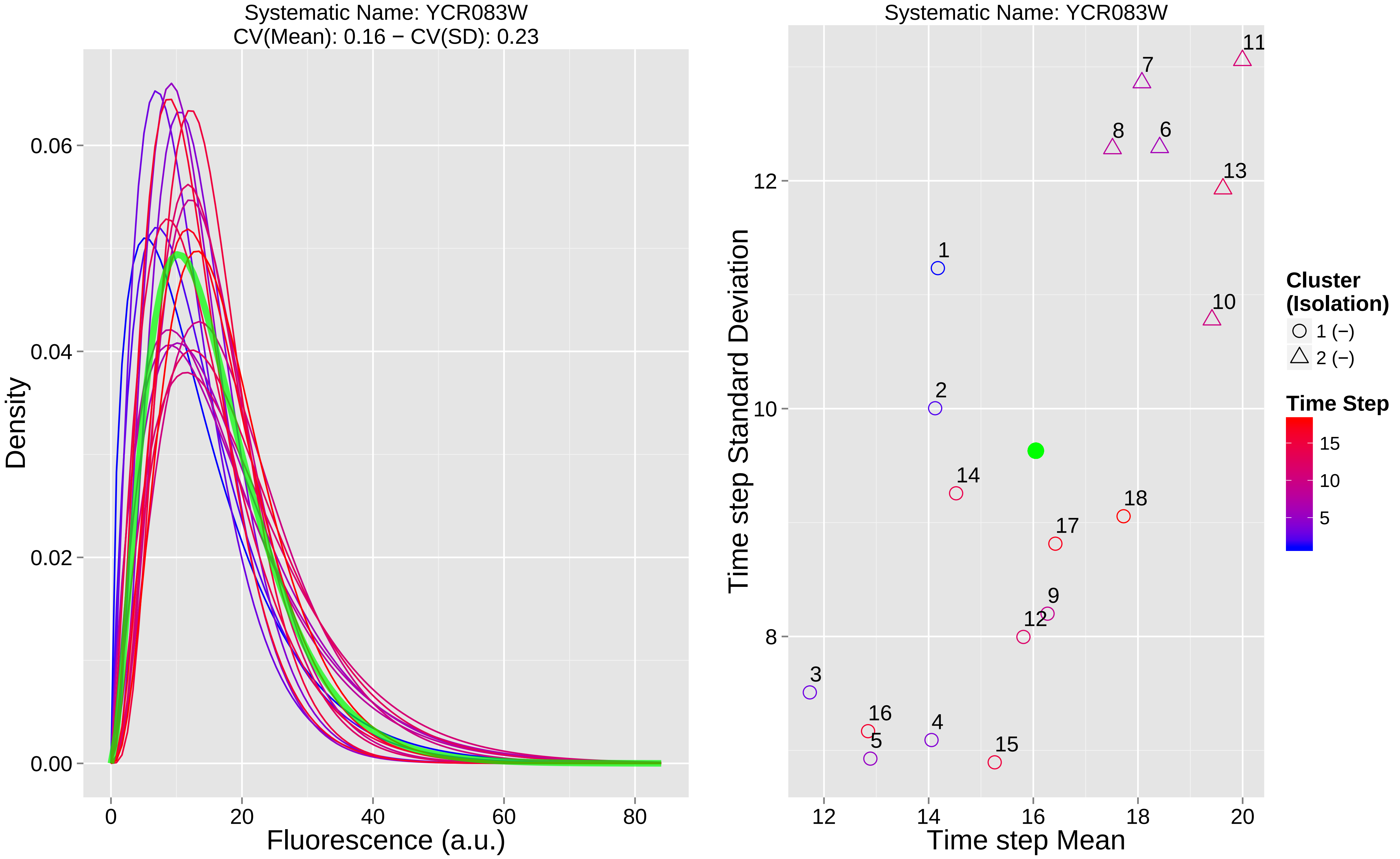
Systematic Name: YCR083W
CV(Mean): 0.16 − CV(SD): 0.23
Density
Fluorescence (a.u.)
0.00
0.02
0.04
0.06
0
20
40
60
80
Systematic Name: YCR083W
Time step Standard Deviation
Time step Mean
8
10
12
12
14
16
18
20
1
2
3
4
5
6
7
8
9
10
11
12
13
14
15
16
17
18
Cluster (Isolation)
1 (−)
2 (−)
Time Step
5
10
15

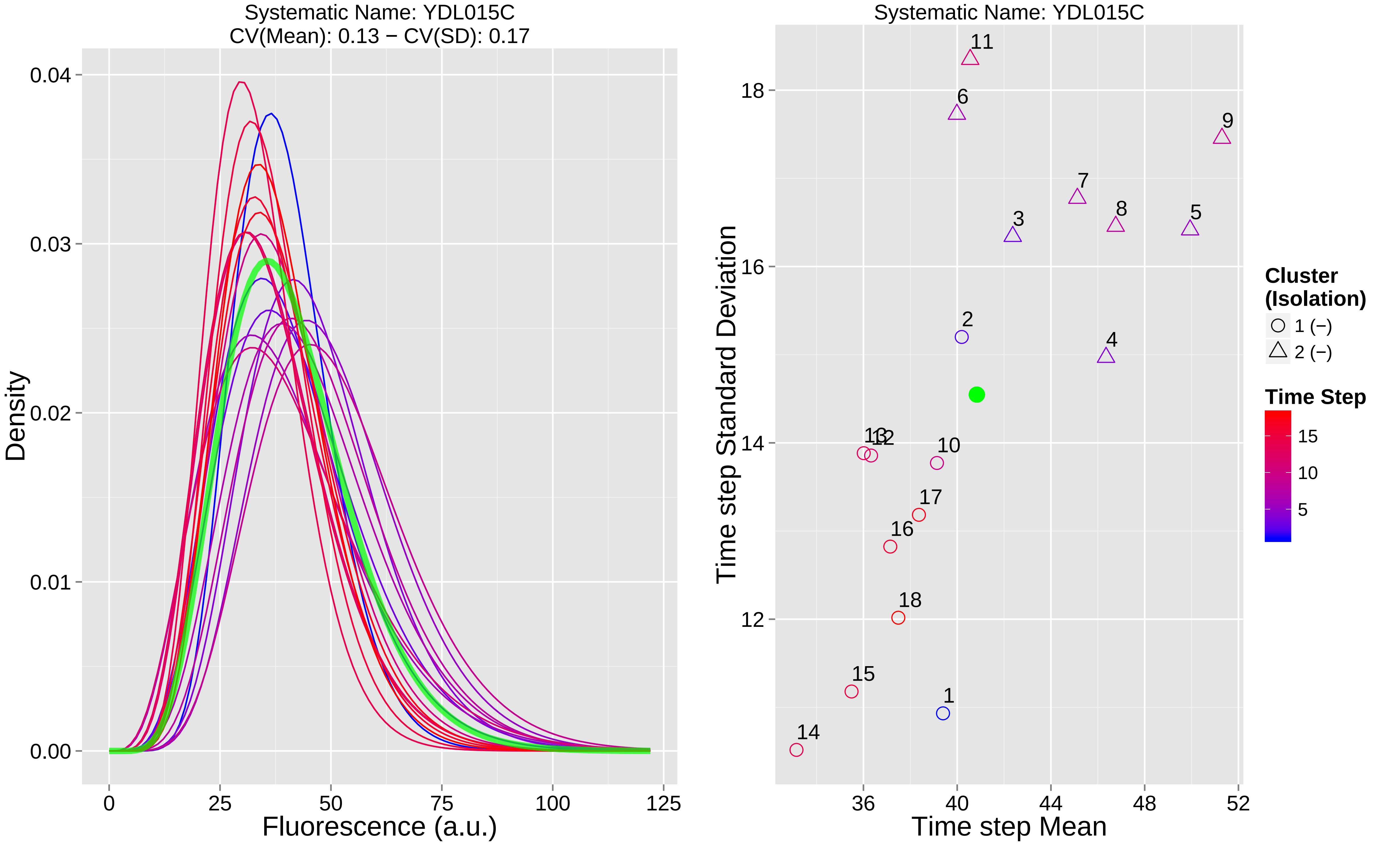
Systematic Name: YDL015C
CV(Mean): 0.13 − CV(SD): 0.17
Density
Fluorescence (a.u.)
0.00
0.01
0.02
0.03
0.04
0
25
50
75
100
125
Systematic Name: YDL015C
Time step Standard Deviation
Time step Mean
12
14
16
18
36
40
44
48
52
Cluster
(Isolation)
1 (−)
2 (−)
Time Step
5
10
15

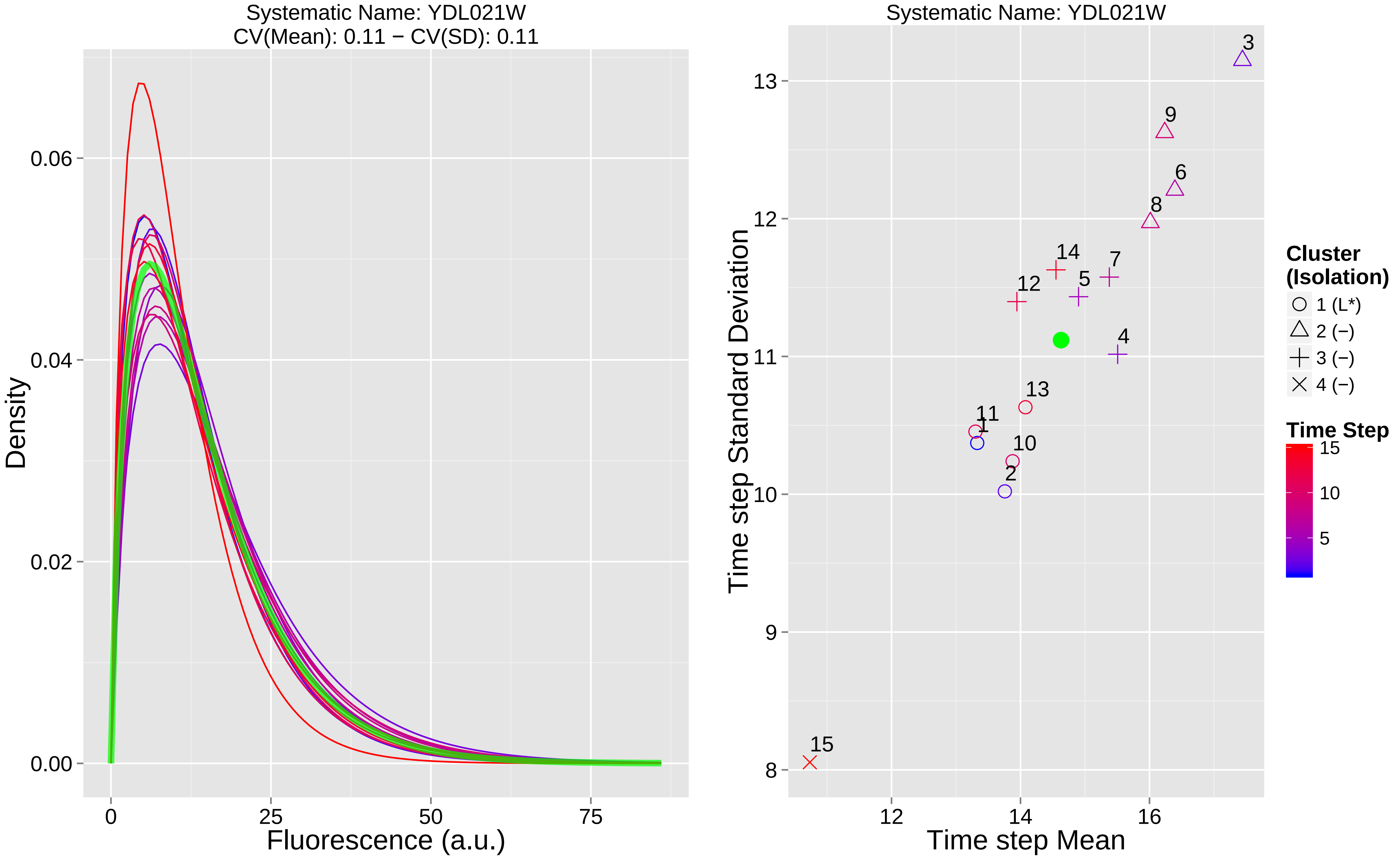

Systematic Name: YDL021W
CV(Mean): 0.11 − CV(SD): 0.11
Density
Fluorescence (a.u.)
0.00
0.02
0.04
0.06
0
25
50
75
Systematic Name: YDL021W
Time step Standard Deviation
Time step Mean
8
9
10
11
12
13
12
14
16
1
2
3
4
5
6
7
8
9
10
11
12
13
14
15
Cluster (Isolation)
1 (L*)
2 (−)
3 (−)
4 (−)
Time Step
15
10
5

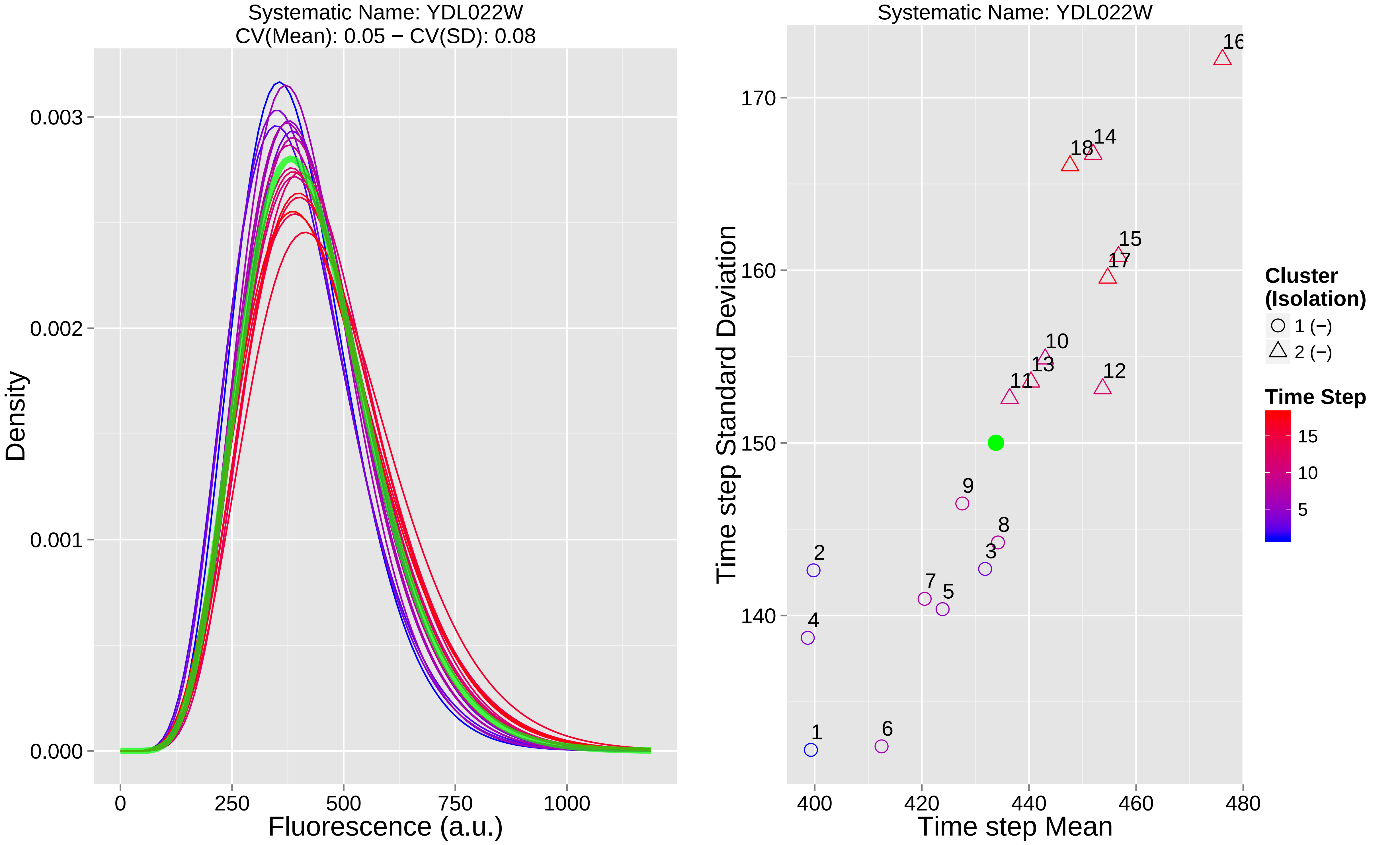

Systematic Name: YDL022W
CV(Mean): 0.05 − CV(SD): 0.08
Density
Fluorescence (a.u.)
0.000
0.001
0.002
0.003
0
250
500
750
1000
Systematic Name: YDL022W
Time step Standard Deviation
Time step Mean
140
150
160
170
400
420
440
460
480
1
2
3
4
5
6
7
8
9
10
11
12
13
14
15
16
17
18
Cluster (Isolation)
1 (−)
2 (−)
Time Step
5
10
15

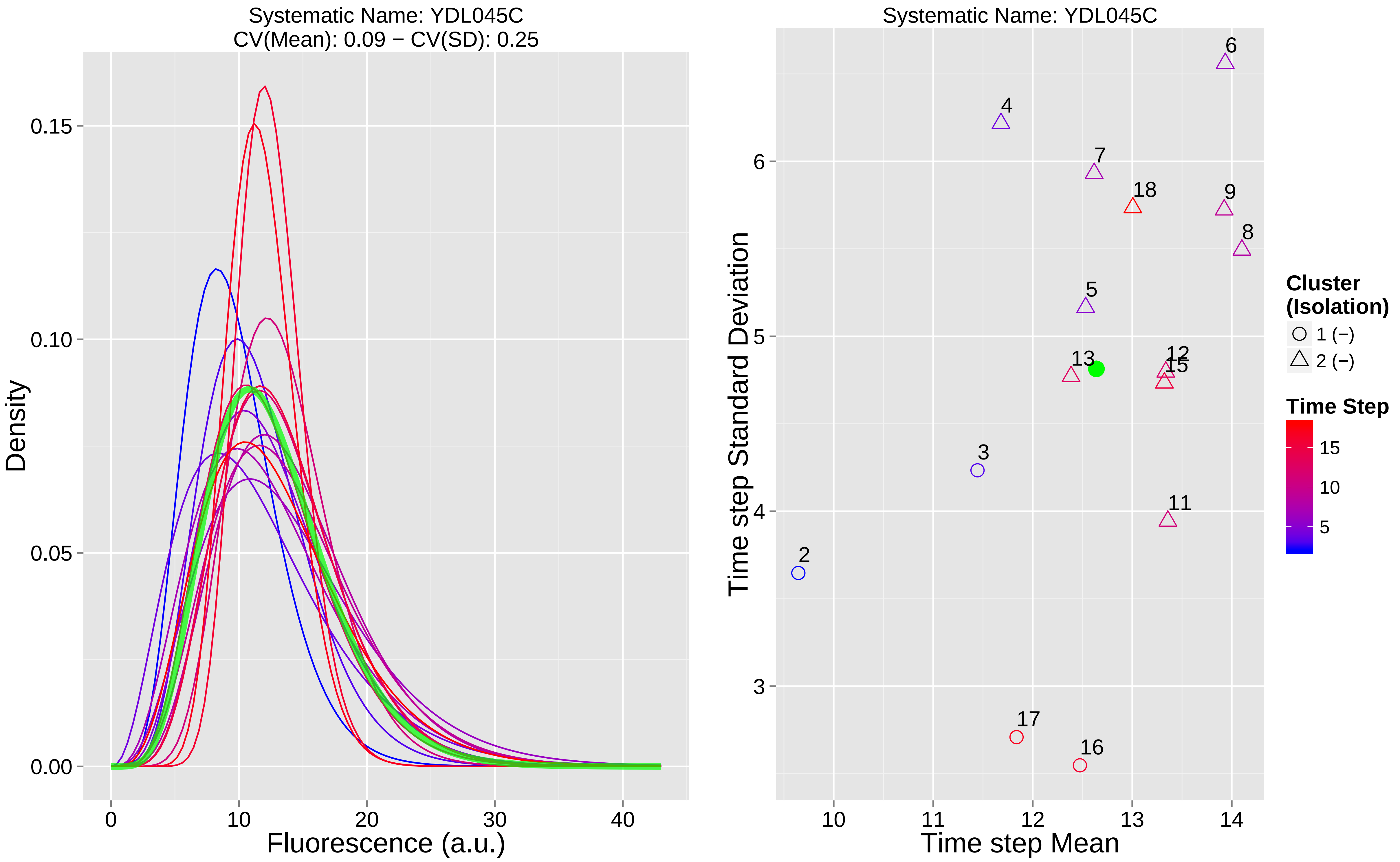

Systematic Name: YDL045C
CV(Mean): 0.09 − CV(SD): 0.25
Density
0.15
0.10
0.05
0.00
0
10
20
30
40
Fluorescence (a.u.)
Systematic Name: YDL045C
Time step Standard Deviation
6
5
4
3
10
11
12
13
14
Time step Mean
6
4
7
18
9
8
5
13
12
15
3
11
2
17
16
Cluster
(Isolation)
1 (−)
2 (−)
Time Step
15
10
5

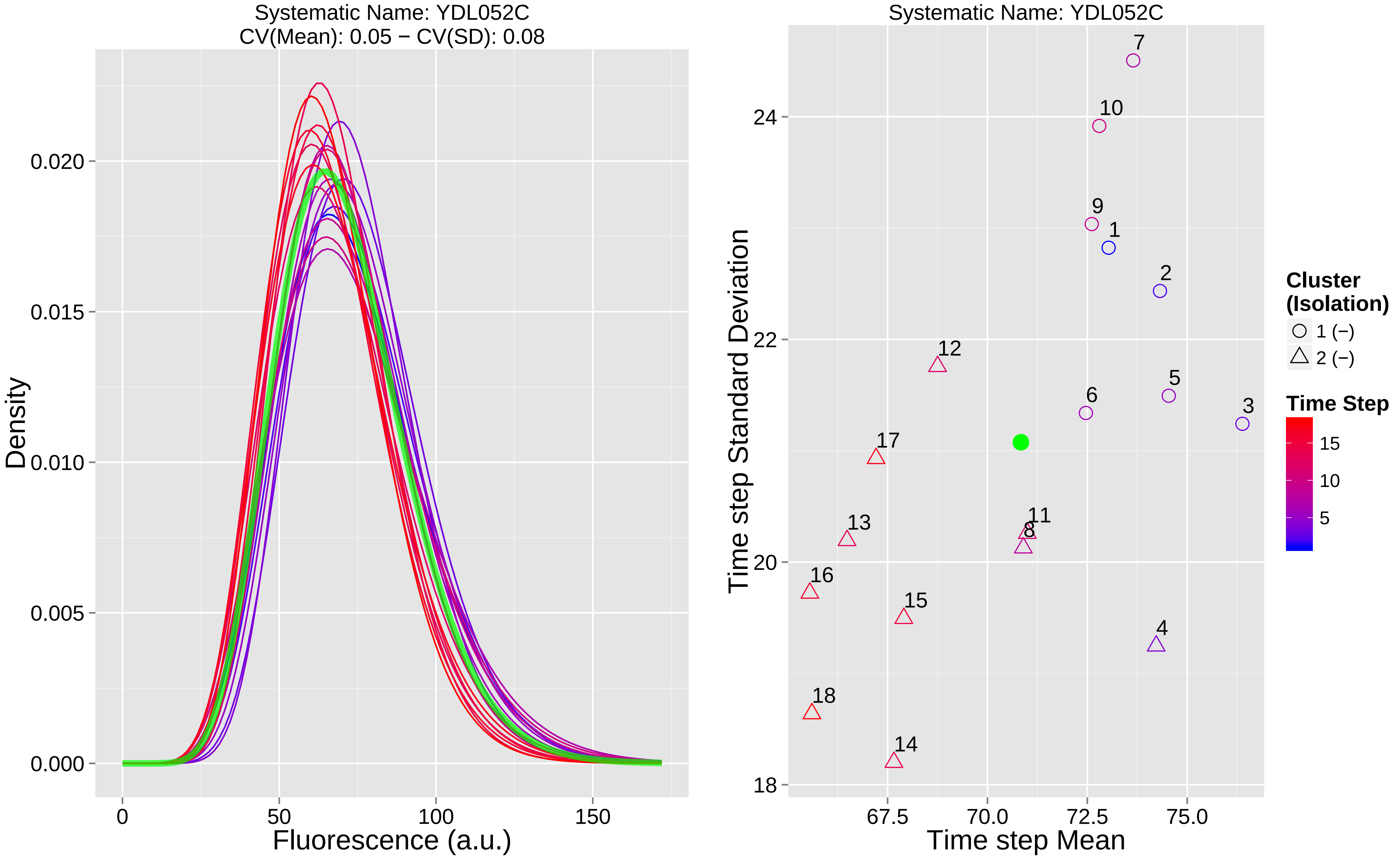

Systematic Name: YDL052C
CV(Mean): 0.05 − CV(SD): 0.08
Density
0.000
0.005
0.010
0.015
0.020
0
50
100
150
Fluorescence (a.u.)
Systematic Name: YDL052C
Time step Standard Deviation
18
20
22
24
67.5
70.0
72.5
75.0
Time step Mean
1
2
3
4
5
6
7
8
9
10
11
12
13
14
15
16
17
18
Cluster
(Isolation)
1 (−)
2 (−)
Time Step
15
10
5

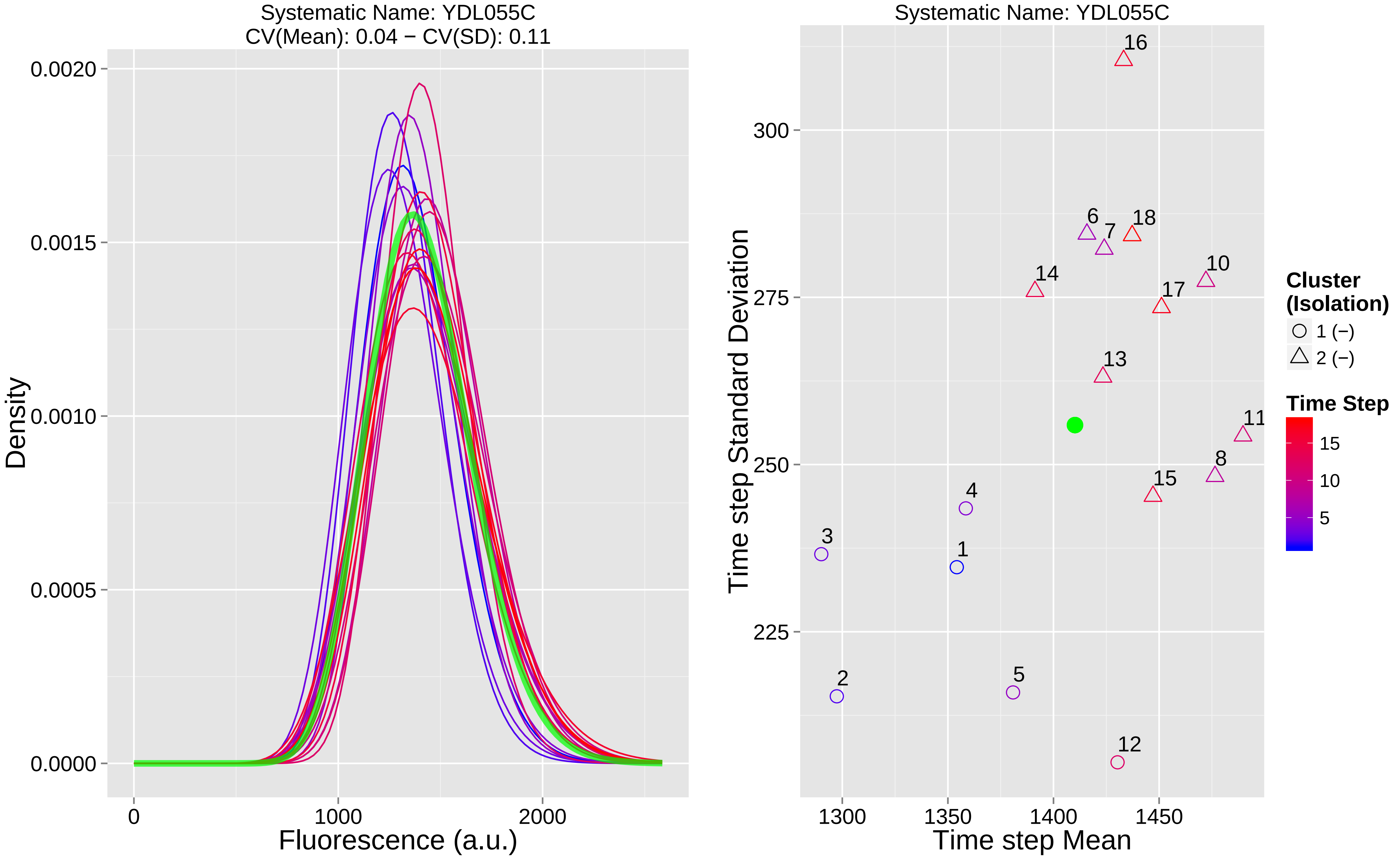

Systematic Name: YDL055C
CV(Mean): 0.04 − CV(SD): 0.11
Density
0.0020
0.0015
0.0010
0.0005
0.0000
0
1000
2000
Fluorescence (a.u.)
Systematic Name: YDL055C
Time step Standard Deviation
300
275
250
225
1300
1350
1400
1450
Time step Mean
16
6
7
18
10
14
17
13
11
8
15
4
3
1
2
5
12
Cluster (Isolation)
1 (−)
2 (−)
Time Step
15
10
5

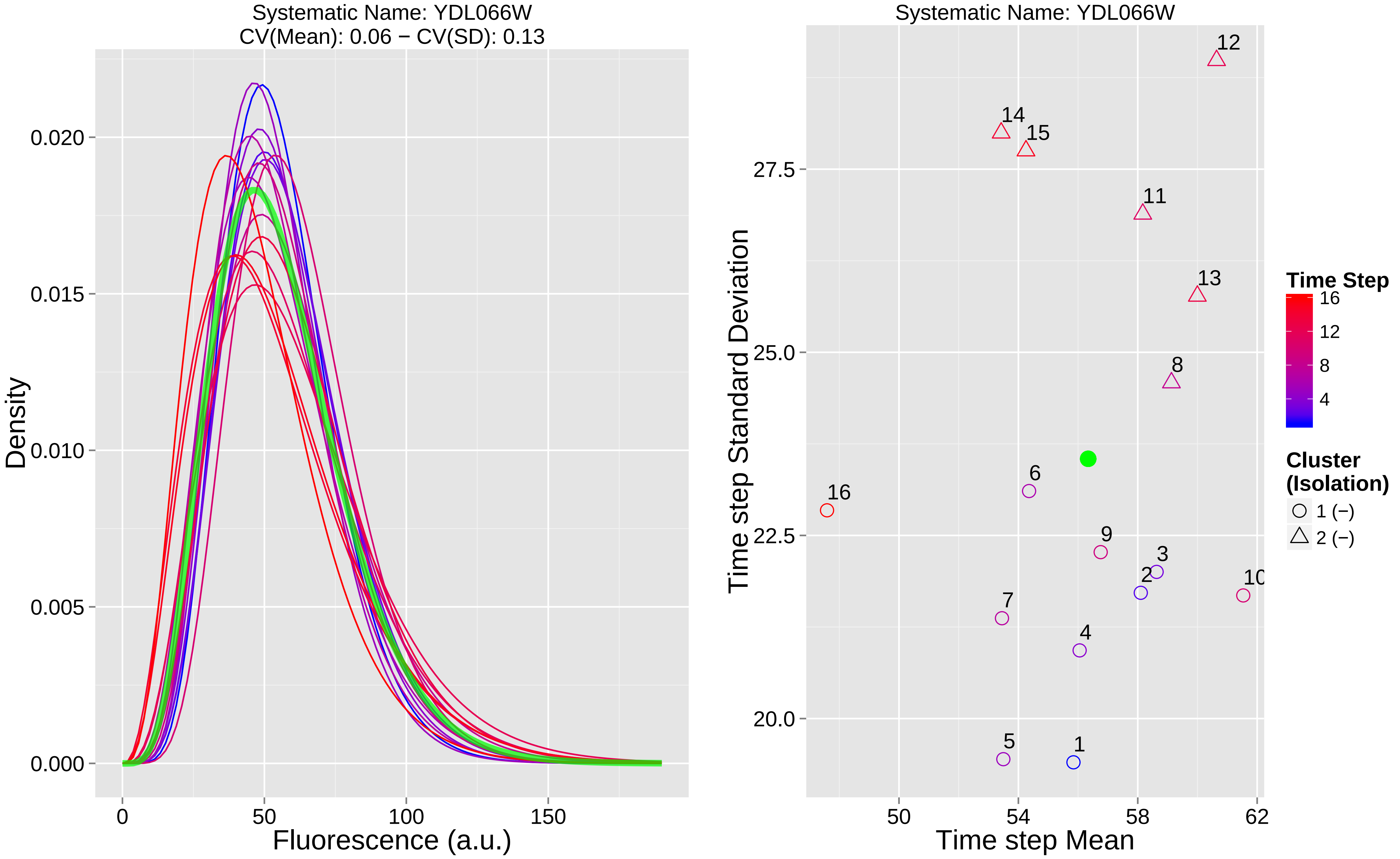

Systematic Name: YDL066W
CV(Mean): 0.06 − CV(SD): 0.13
Density
0.000
0.005
0.010
0.015
0.020
0
50
100
150
Fluorescence (a.u.)
Systematic Name: YDL066W
Time step Standard Deviation
20.0
22.5
25.0
27.5
50
54
58
62
Time step Mean
1
2
3
4
5
6
7
8
9
10
11
12
13
14
15
16
Time Step
16
12
8
4
Cluster
(Isolation)
1 (−)
2 (−)

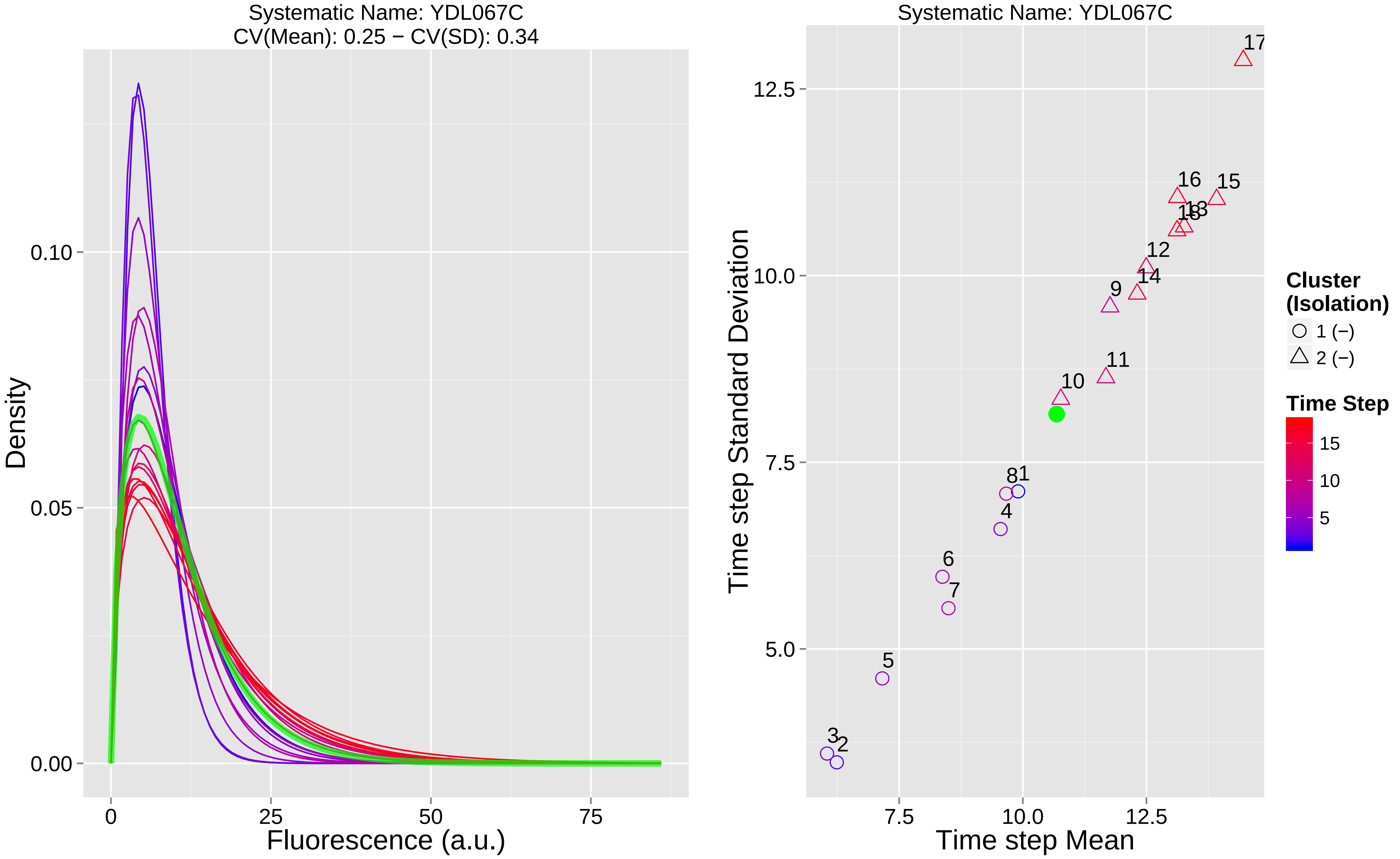

Systematic Name: YDL067C
CV(Mean): 0.25 − CV(SD): 0.34
Density
0.10
0.05
0.00
0
25
50
75
Fluorescence (a.u.)
Systematic Name: YDL067C
Time step Standard Deviation
12.5
10.0
7.5
5.0
7.5
10.0
12.5
Time step Mean
17
16
15
12
14
9
11
10
1
4
6
7
5
3
2
Cluster
(Isolation)
1 (−)
2 (−)
Time Step
15
10
5

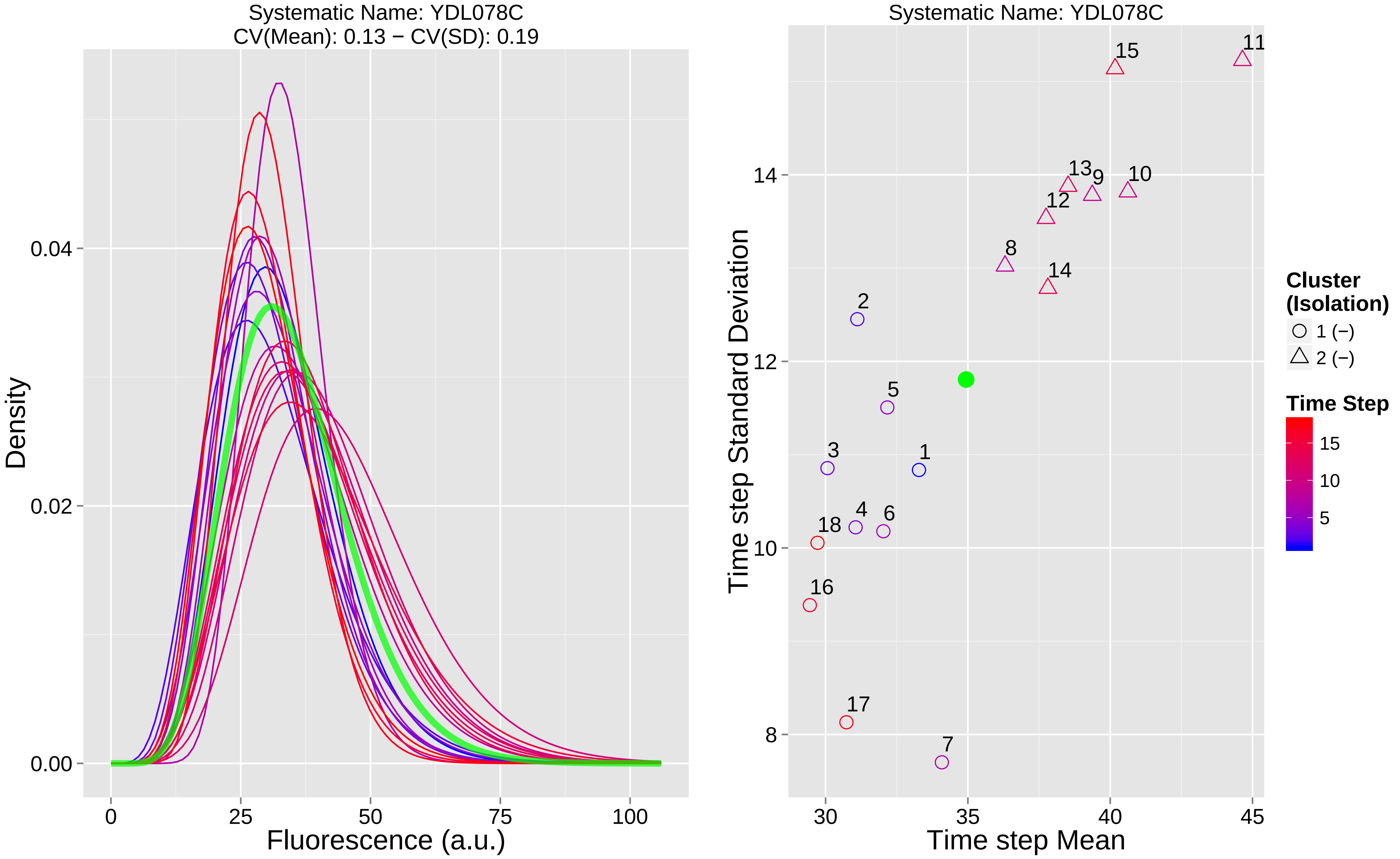

Systematic Name: YDL078C
CV(Mean): 0.13 − CV(SD): 0.19
Density
0.00
0.02
0.04
0
25
50
75
100
Fluorescence (a.u.)
Systematic Name: YDL078C
Time step Standard Deviation
Time step Mean
8
10
12
14
30
35
40
45
1
2
3
4
5
6
7
8
9
10
11
12
13
14
15
16
17
18
Cluster
(Isolation)
1 (−)
2 (−)
Time Step
5
10
15

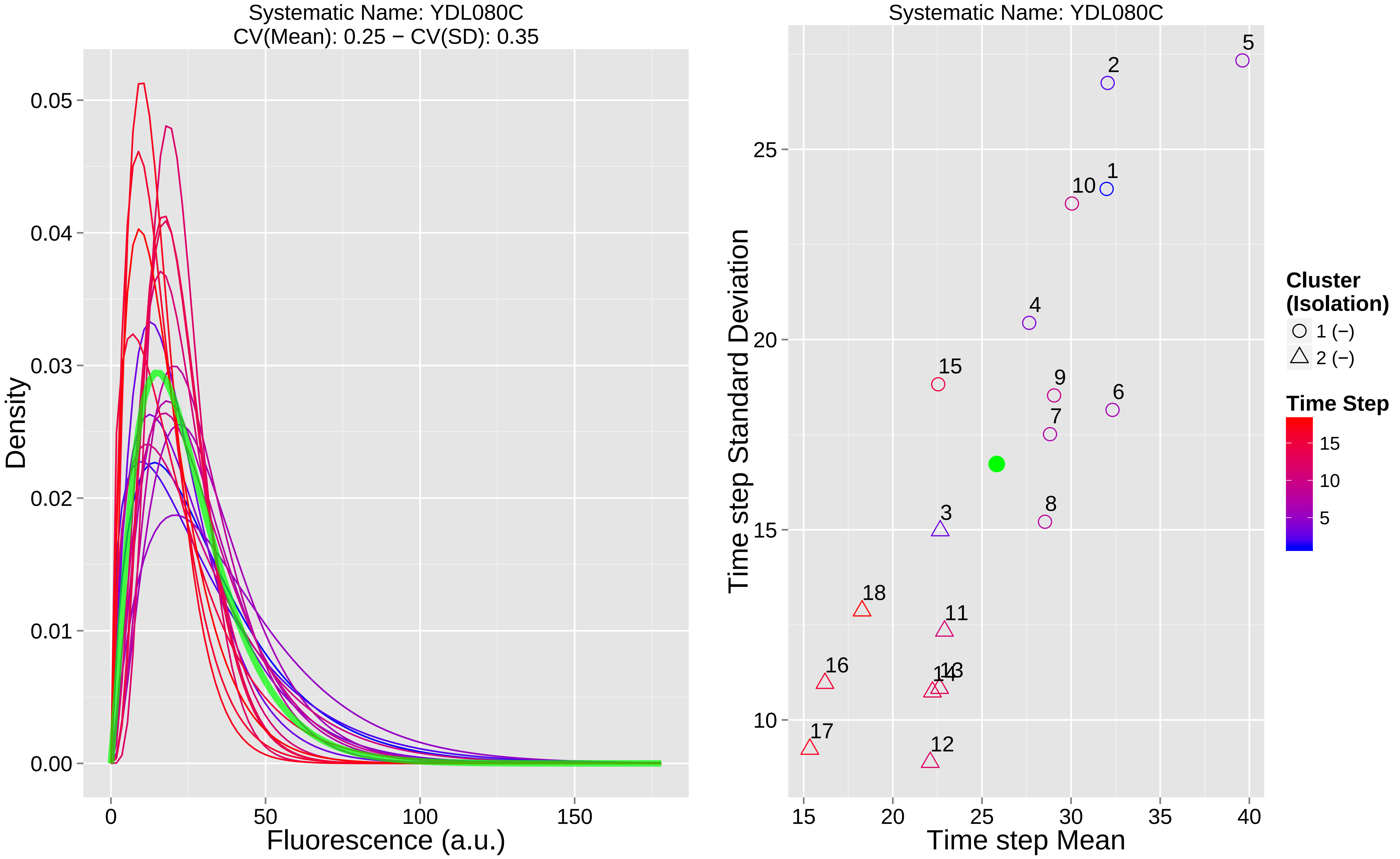

Systematic Name: YDL080C
CV(Mean): 0.25 − CV(SD): 0.35
Density
0.05
0.04
0.03
0.02
0.01
0.00
0
50
100
150
Fluorescence (a.u.)
Systematic Name: YDL080C
Time step Standard Deviation
25
20
15
10
Time step Mean
15
20
25
30
35
40
5
2
1
10
4
15
9
6
7
8
3
18
11
16
14
13
17
12
Cluster
(Isolation)
1 (−)
2 (−)
Time Step
15
10
5

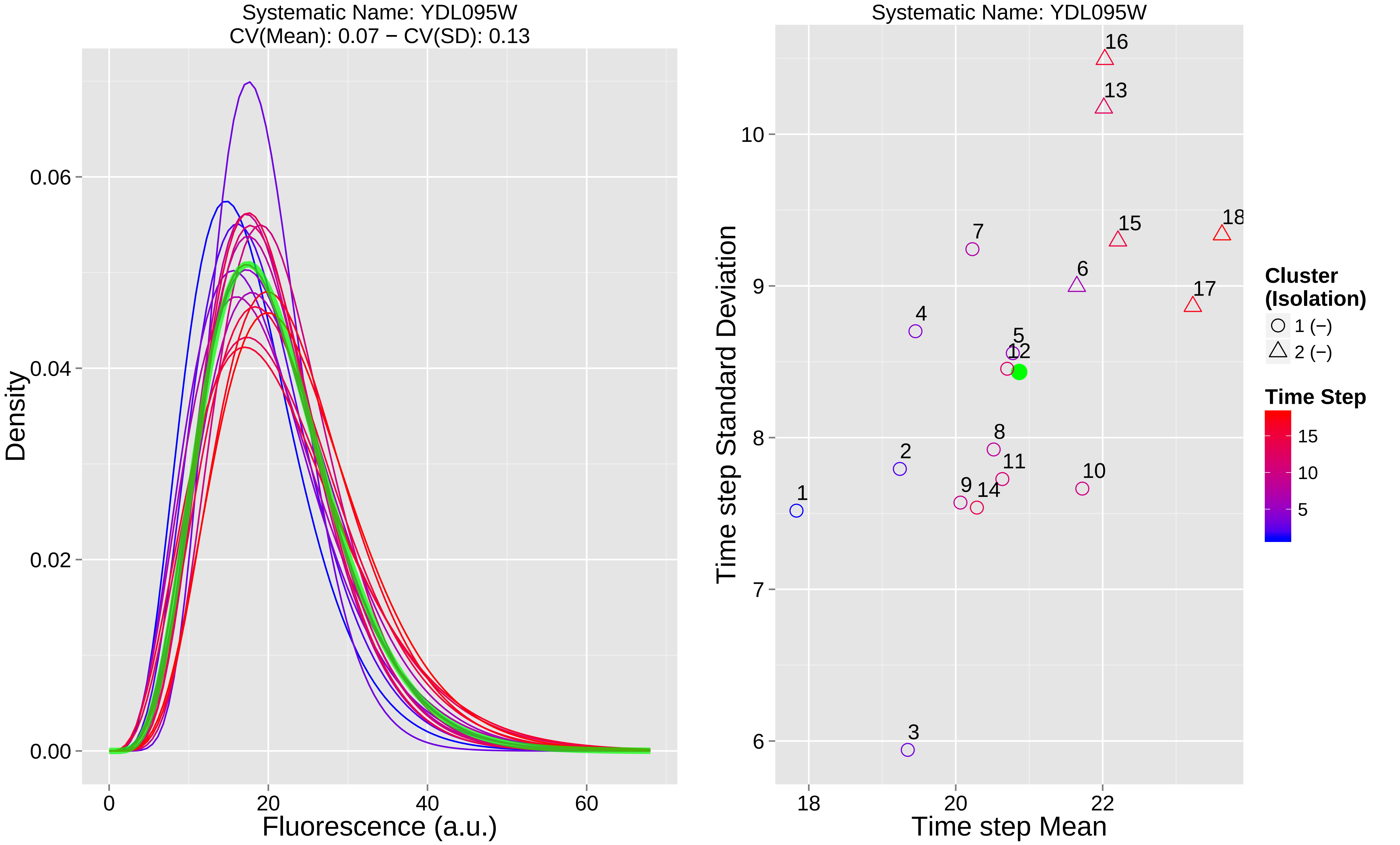

Systematic Name: YDL095W
CV(Mean): 0.07 − CV(SD): 0.13
Density
0.00
0.02
0.04
0.06
0
20
40
60
Fluorescence (a.u.)
Systematic Name: YDL095W
Time step Standard Deviation
6
7
8
9
10
18
20
22
Time step Mean
1
2
3
4
5
6
7
8
9
10
11
12
13
14
15
16
17
18
Cluster (Isolation)
1 (−)
2 (−)
Time Step
5
10
15

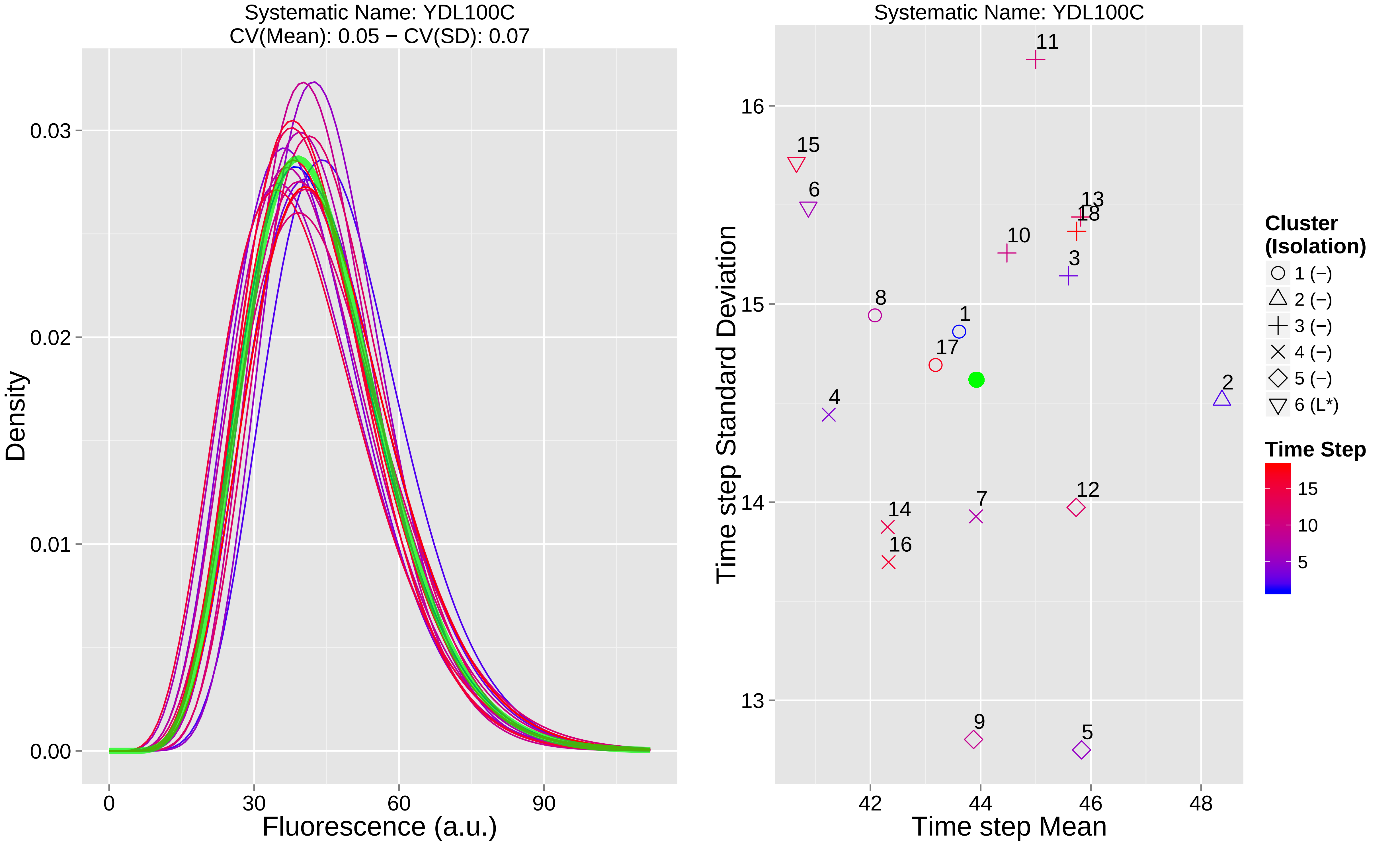

Systematic Name: YDL100C
CV(Mean): 0.05 − CV(SD): 0.07
Density
0.00
0.01
0.02
0.03
0
30
60
90
Fluorescence (a.u.)
Systematic Name: YDL100C
Time step Standard Deviation
13
14
15
16
42
44
46
48
Time step Mean
11
15
6
13
18
10
3
8
1
17
4
2
14
16
7
12
9
5
Cluster
(Isolation)
1 (−)
2 (−)
3 (−)
4 (−)
5 (−)
6 (L*)
Time Step
15
10
5

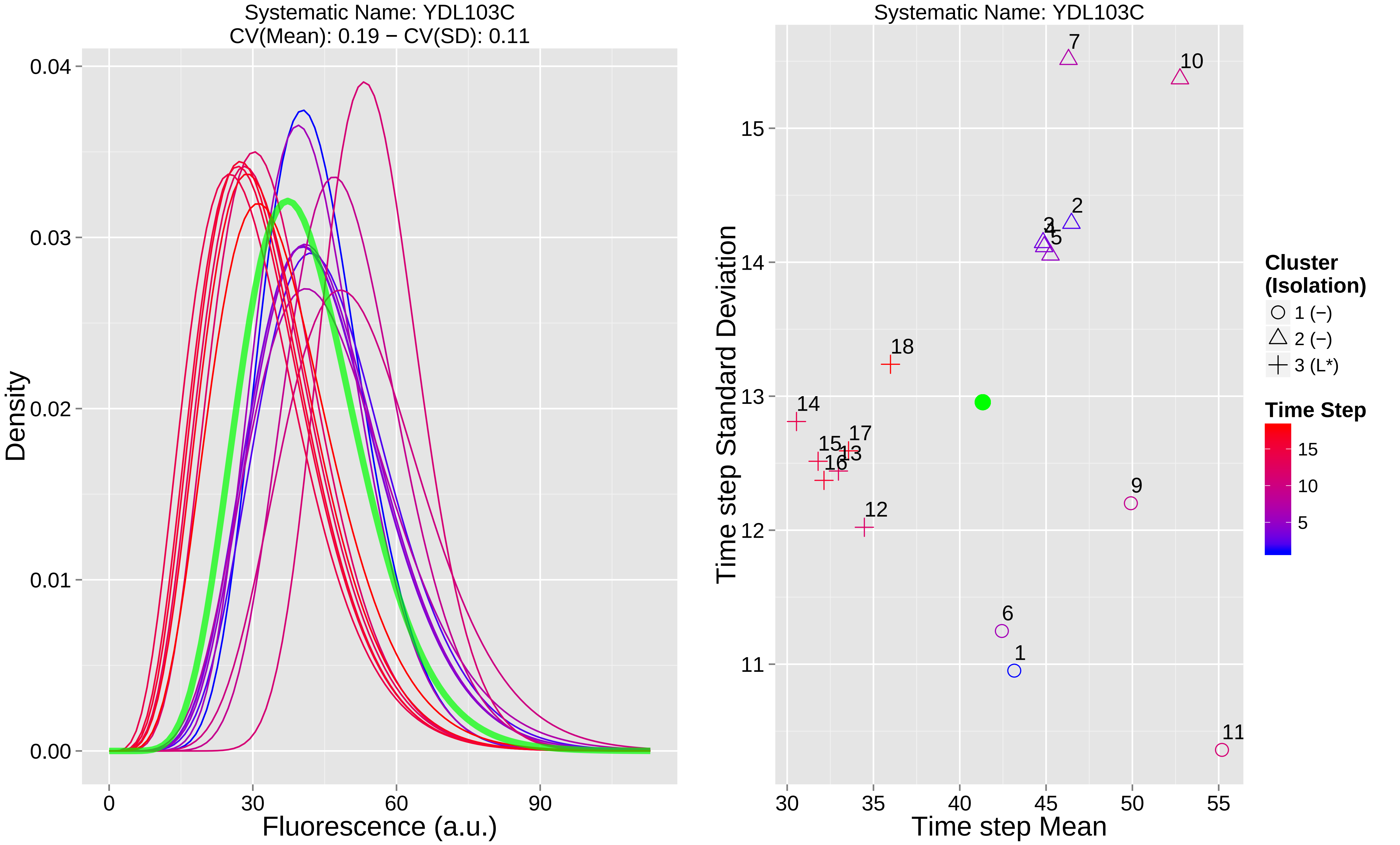

Systematic Name: YDL103C
CV(Mean): 0.19 − CV(SD): 0.11
Density
Fluorescence (a.u.)
0.00
0.01
0.02
0.03
0.04
0
30
60
90
Systematic Name: YDL103C
Time step Standard Deviation
Time step Mean
11
12
13
14
15
30
35
40
45
50
55
Cluster (Isolation)
1 (−)
2 (−)
3 (L*)
Time Step
15
10
5

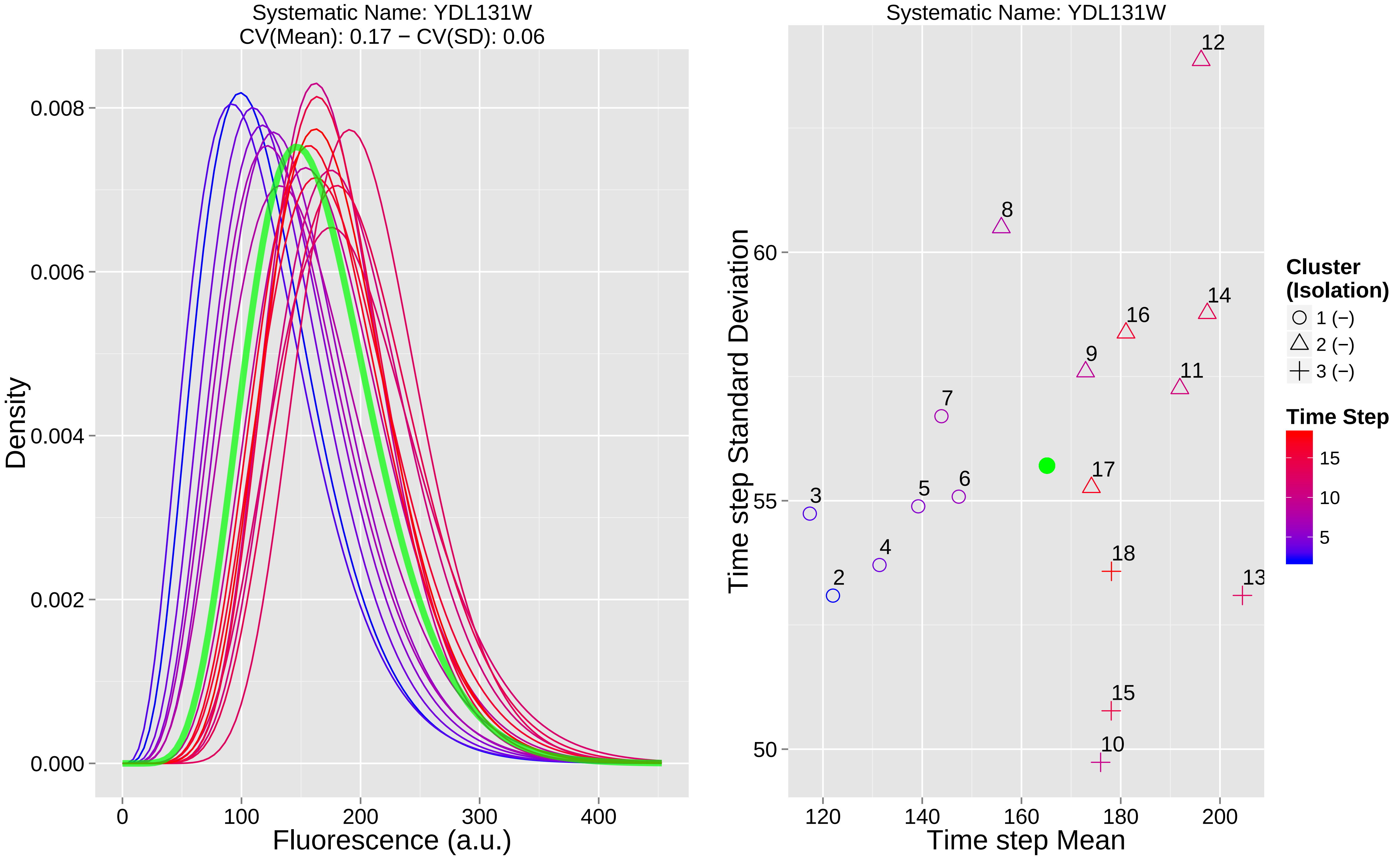

Systematic Name: YDL131W
CV(Mean): 0.17 − CV(SD): 0.06
Density
Fluorescence (a.u.)
0.000
0.002
0.004
0.006
0.008
0
100
200
300
400
Systematic Name: YDL131W
Time step Standard Deviation
Time step Mean
50
55
60
120
140
160
180
200
2
3
4
5
6
7
8
9
10
11
12
13
14
15
16
17
18
Cluster (Isolation)
1 (−)
2 (−)
3 (−)
Time Step
5
10
15

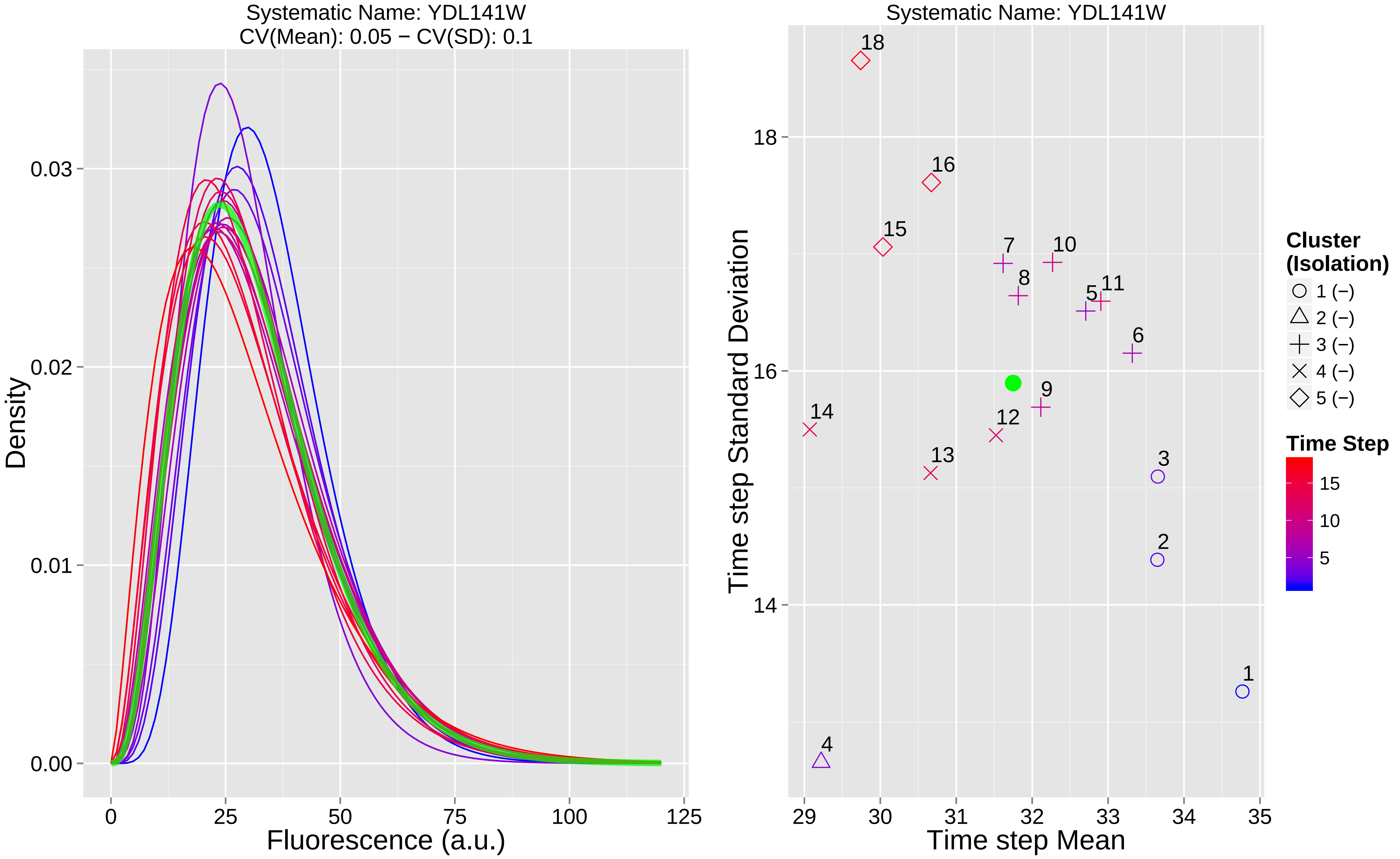
Systematic Name: YDL141W
CV(Mean): 0.05 − CV(SD): 0.1
Density
0.00
0.01
0.02
0.03
0
25
50
75
100
125
Fluorescence (a.u.)
Systematic Name: YDL141W
Time step Standard Deviation
Time step Mean
14
16
18
29
30
31
32
33
34
35
1
2
3
4
5
6
7
8
9
10
11
12
13
14
15
16
18
Cluster
(Isolation)
1 (−)
2 (−)
3 (−)
4 (−)
5 (−)
Time Step
15
10
5

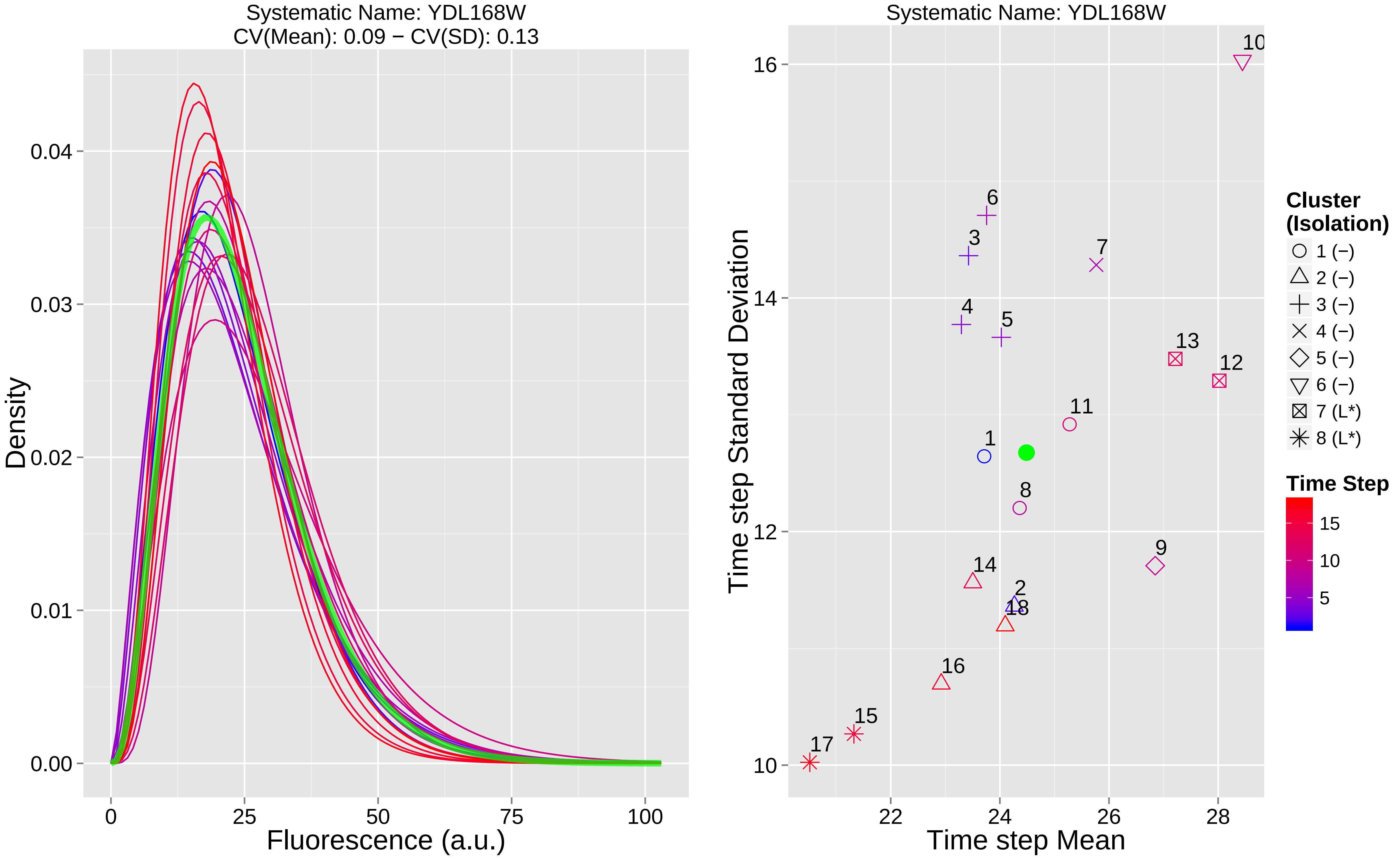

Systematic Name: YDL168W
CV(Mean): 0.09 − CV(SD): 0.13
Density
Fluorescence (a.u.)
0.00
0.01
0.02
0.03
0.04
0
25
50
75
100
Systematic Name: YDL168W
Time step Standard Deviation
Time step Mean
10
12
14
16
22
24
26
28
1
2
3
4
5
6
7
8
9
10
11
12
13
14
15
16
17
18
Cluster (Isolation)
1 (−)
2 (−)
3 (−)
4 (−)
5 (−)
6 (−)
7 (L*)
8 (L*)
Time Step
15
10
5

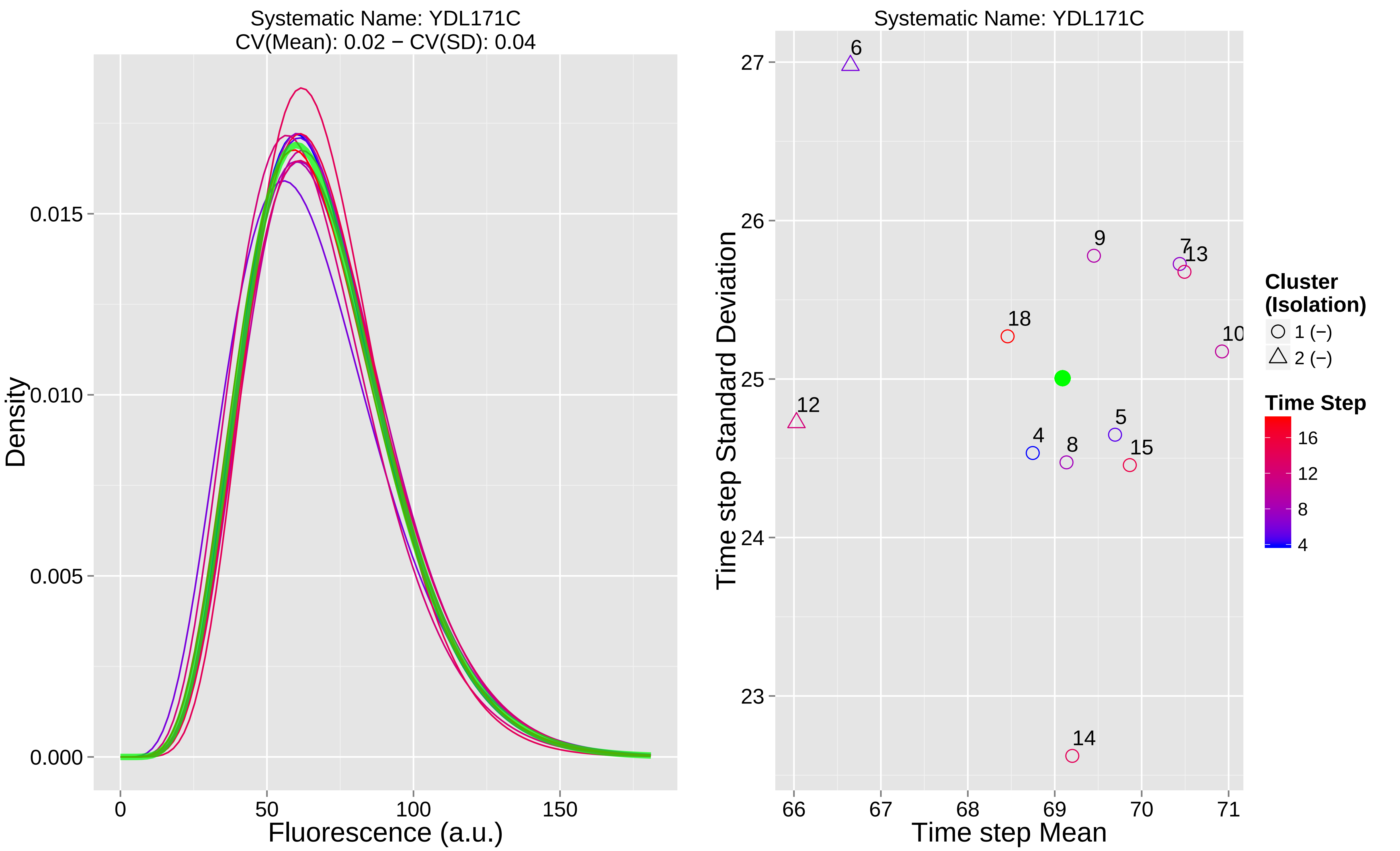
Systematic Name: YDL171C
CV(Mean): 0.02 − CV(SD): 0.04
Density
0.015
0.010
0.005
0.000
0
50
100
150
Fluorescence (a.u.)
Systematic Name: YDL171C
Time step Standard Deviation
27
26
25
24
23
66
67
68
69
70
71
Time step Mean
6
9
7
13
18
10
12
5
4
8
15
14
Cluster
(Isolation)
1 (−)
2 (−)
Time Step
16
12
8
4

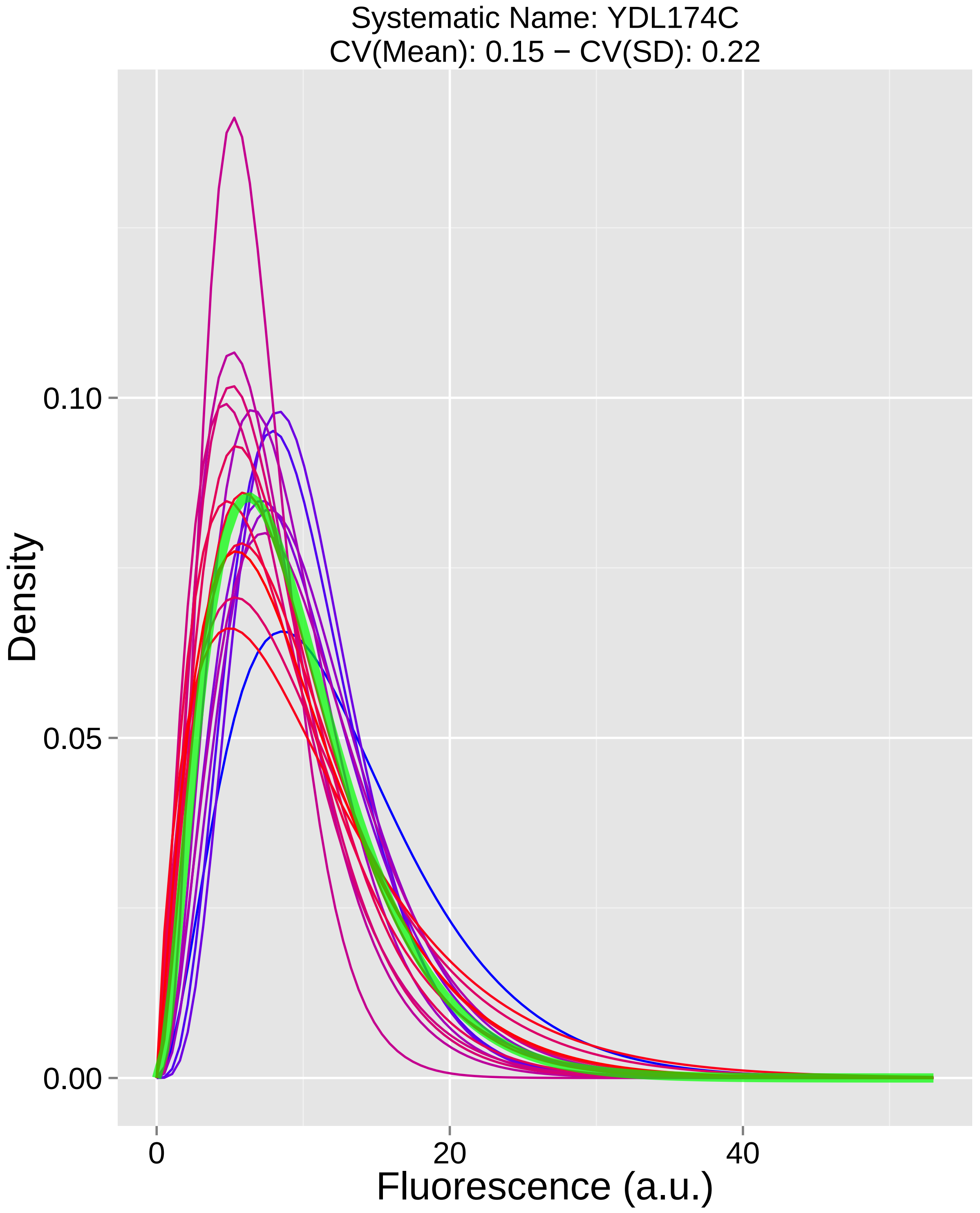

Systematic Name: YDL174C
CV(Mean): 0.15 − CV(SD): 0.22
Density
0.00
0.05
0.10
0
20
40
Fluorescence (a.u.)

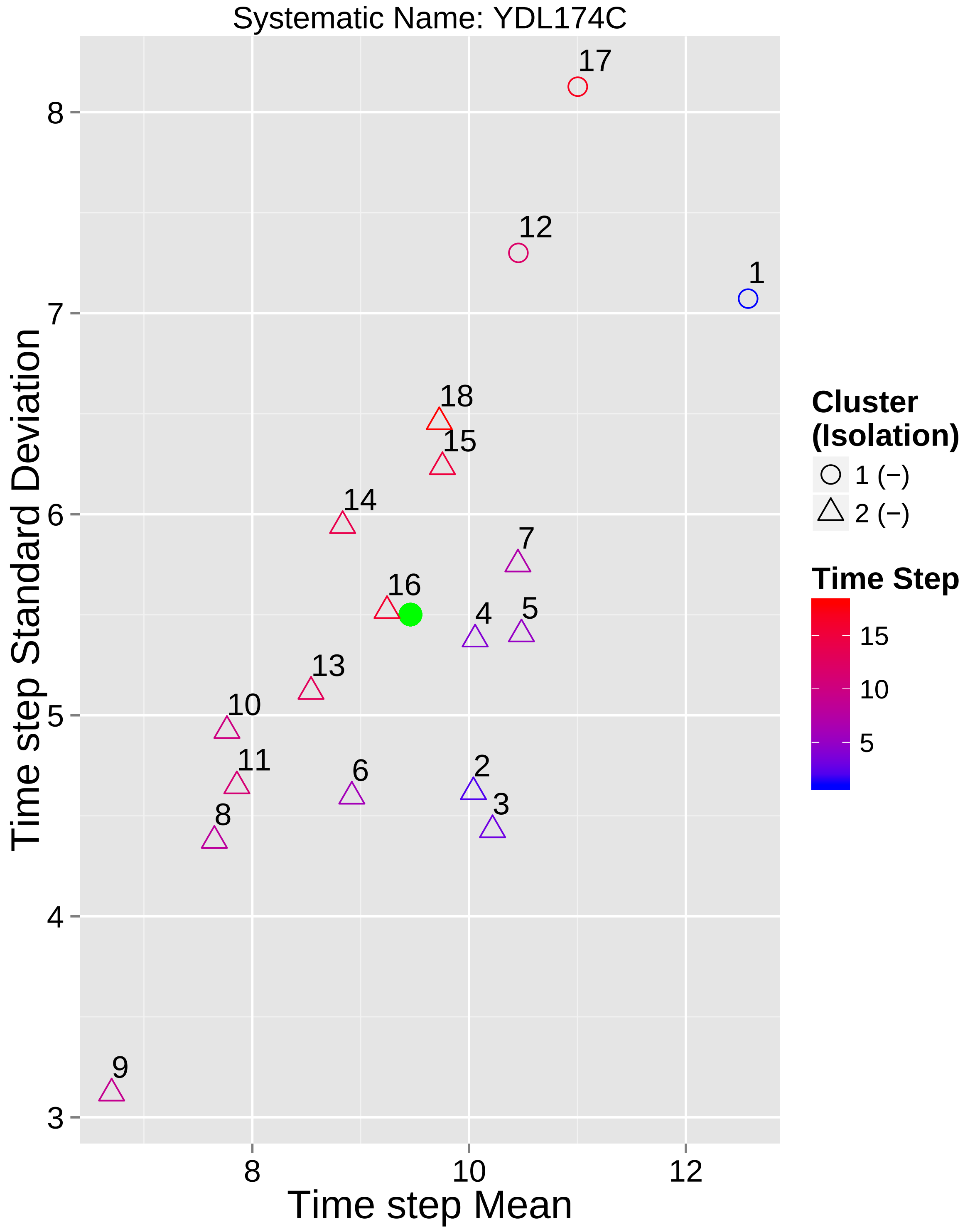

Systematic Name: YDL174C
Time step Standard Deviation
3
4
5
6
7
8
8
10
12
Time step Mean
1
2
3
4
5
6
7
8
9
10
11
12
13
14
15
16
17
18
Cluster (Isolation)
1 (−)
2 (−)
Time Step
5
10
15

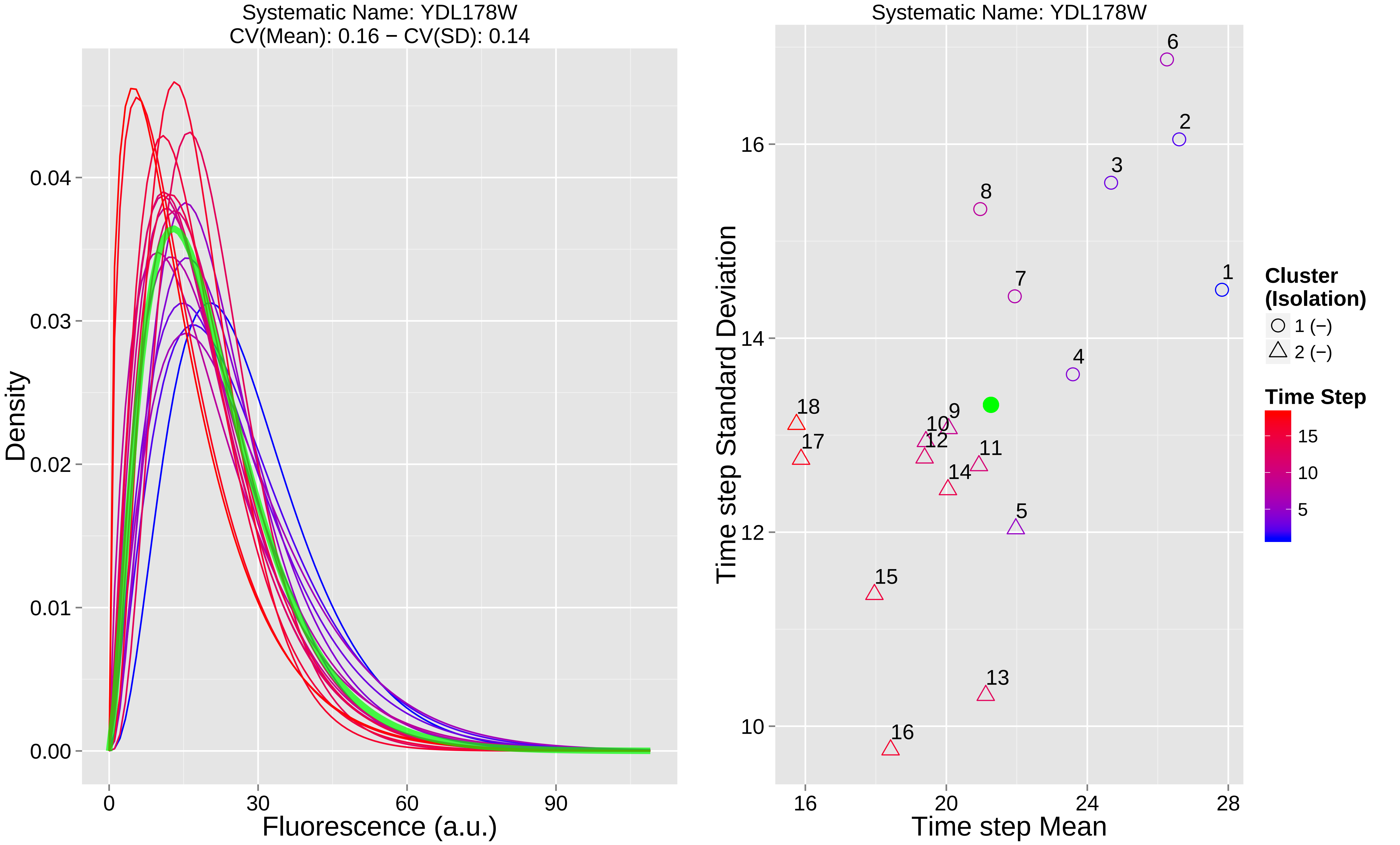
Systematic Name: YDL178W
CV(Mean): 0.16 − CV(SD): 0.14
Density
0.00
0.01
0.02
0.03
0.04
0
30
60
90
Fluorescence (a.u.)
Systematic Name: YDL178W
Time step Standard Deviation
10
12
14
16
16
20
24
28
Time step Mean
1
2
3
4
5
6
7
8
9
10
11
12
13
14
15
16
17
18
Cluster
(Isolation)
1 (−)
2 (−)
Time Step
15
10
5

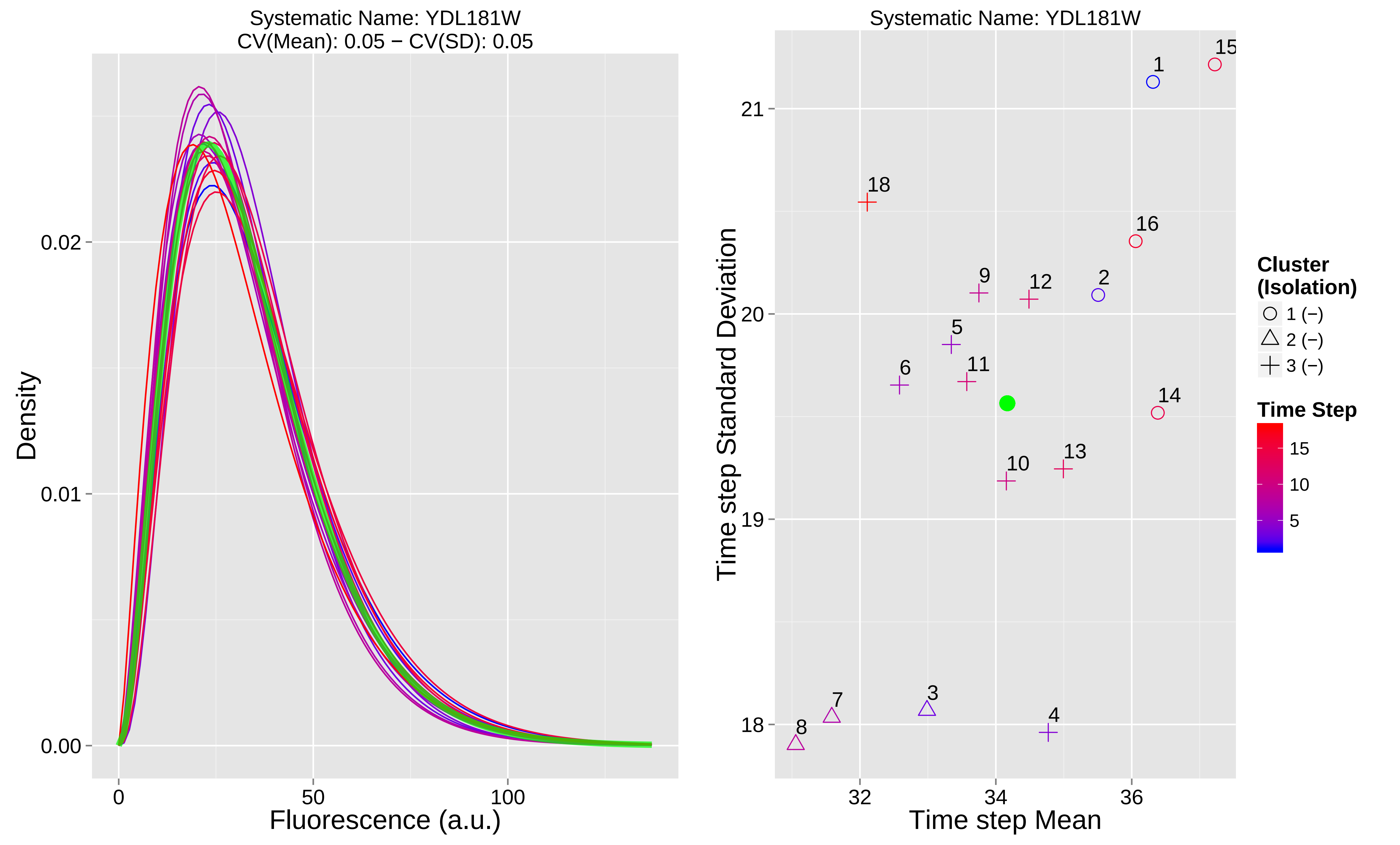
Systematic Name: YDL181W
CV(Mean): 0.05 − CV(SD): 0.05
Density
0.00
0.01
0.02
0
50
100
Fluorescence (a.u.)
Systematic Name: YDL181W
Time step Standard Deviation
Time step Mean
18
19
20
21
32
34
36
1
2
3
4
5
6
7
8
9
10
11
12
13
14
15
16
18
Cluster (Isolation)
1 (−)
2 (−)
3 (−)
Time Step
5
10
15

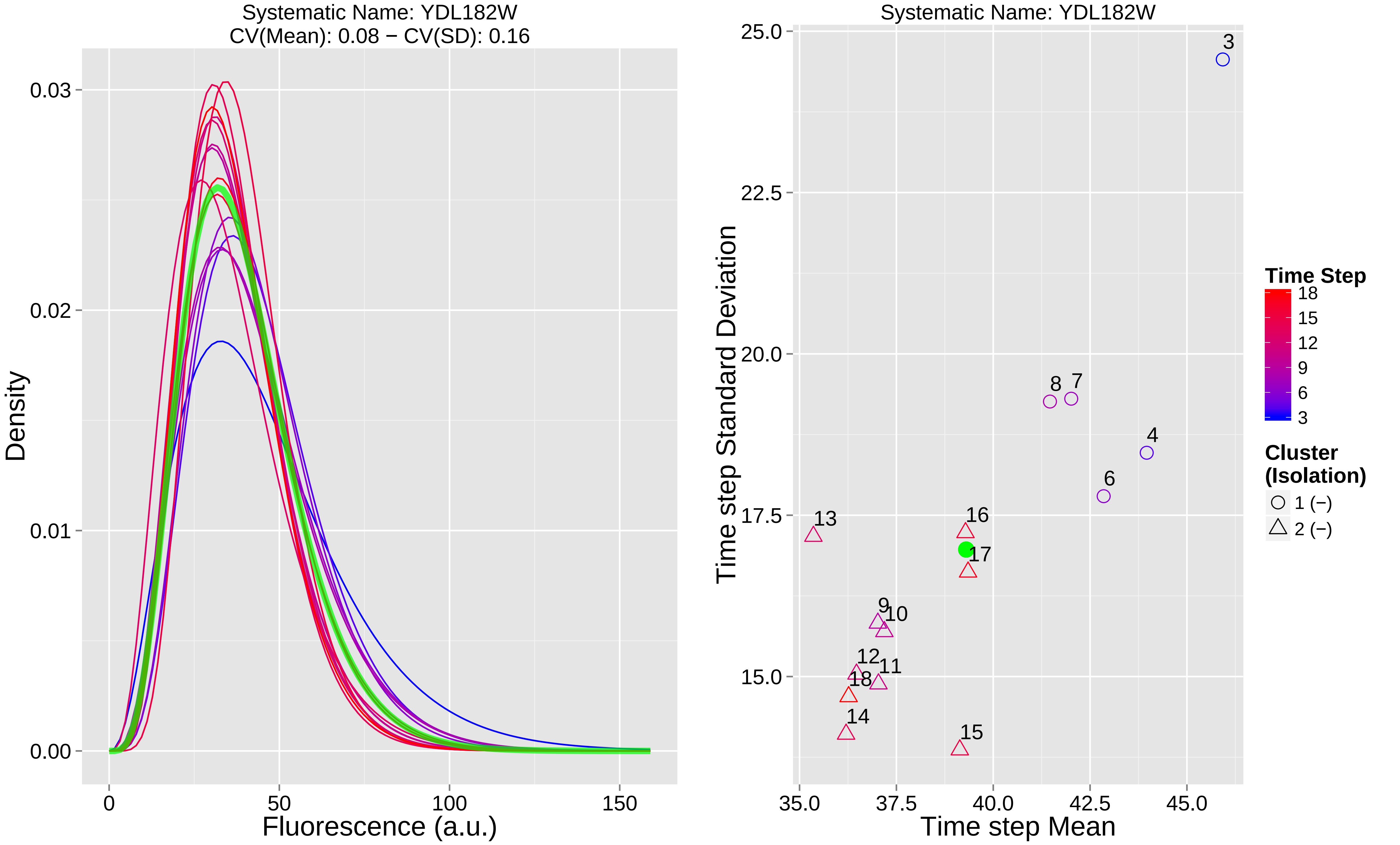
Systematic Name: YDL182W
CV(Mean): 0.08 − CV(SD): 0.16
Density
Fluorescence (a.u.)
0.00
0.01
0.02
0.03
0
50
100
150
Systematic Name: YDL182W
Time step Standard Deviation
Time step Mean
15.0
17.5
20.0
22.5
25.0
35.0
37.5
40.0
42.5
45.0
3
4
6
7
8
9
10
11
12
13
14
15
16
17
18
Time Step
18
15
12
9
6
3
Cluster
(Isolation)
1 (−)
2 (−)

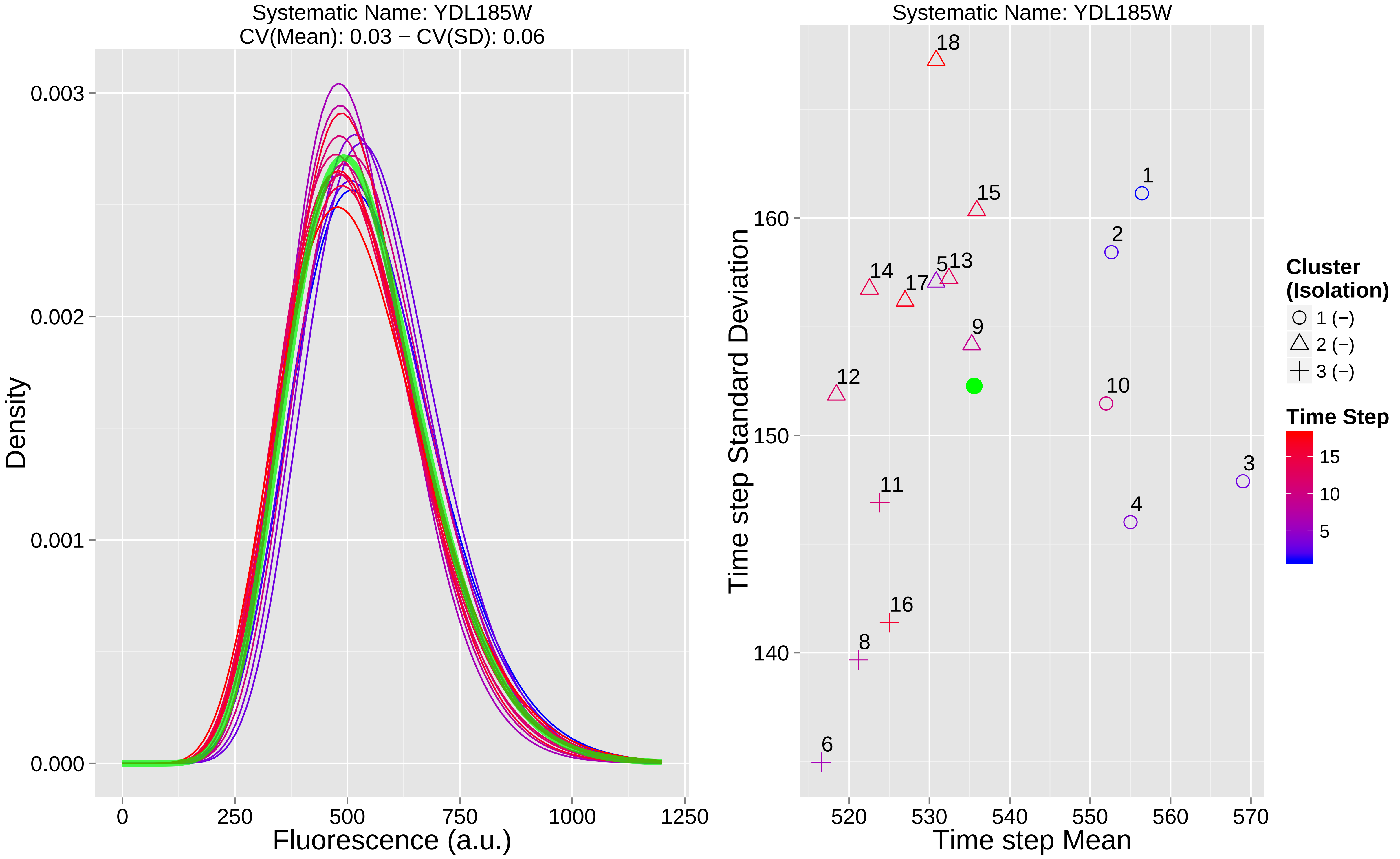
Systematic Name: YDL185W
CV(Mean): 0.03 − CV(SD): 0.06
Density
0.003
0.002
0.001
0.000
0
250
500
750
1000
1250
Fluorescence (a.u.)
Systematic Name: YDL185W
Time step Standard Deviation
160
150
140
520
530
540
550
560
570
Time step Mean
18
15
1
2
14
17
5
13
9
12
10
3
4
11
16
8
6
Cluster
(Isolation)
1 (−)
2 (−)
3 (−)
Time Step
15
10
5

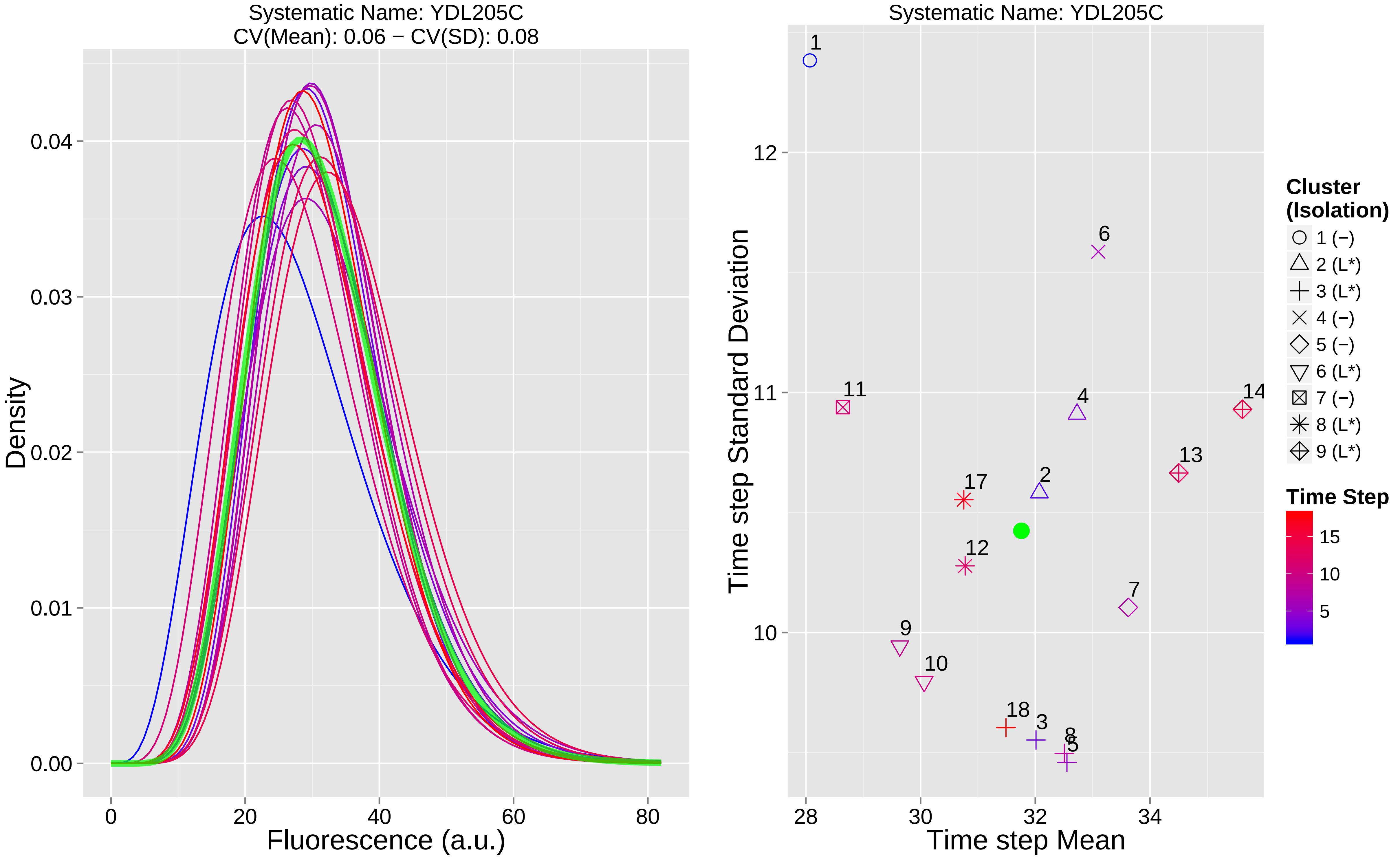

Systematic Name: YDL205C
CV(Mean): 0.06 − CV(SD): 0.08
Density
Fluorescence (a.u.)
0.00
0.01
0.02
0.03
0.04
0
20
40
60
80
Systematic Name: YDL205C
Time step Standard Deviation
Time step Mean
10
11
12
28
30
32
34
1
2
3
4
5
6
7
8
9
10
11
12
13
14
17
18
Cluster (Isolation)
1 (−)
2 (L*)
3 (L*)
4 (−)
5 (−)
6 (L*)
7 (−)
8 (L*)
9 (L*)
Time Step
15
10
5

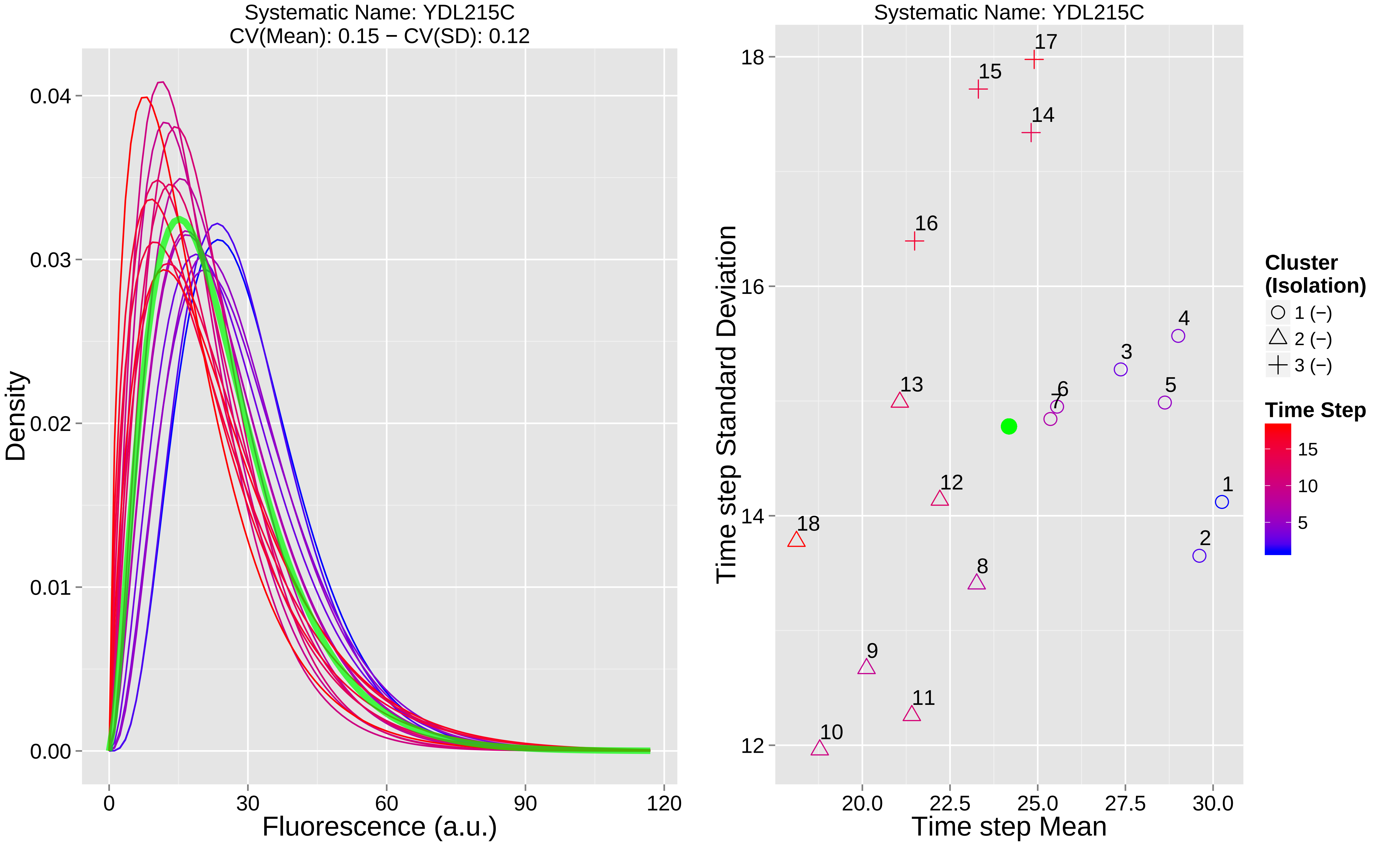

Systematic Name: YDL215C
CV(Mean): 0.15 − CV(SD): 0.12
Density
0.04
0.03
0.02
0.01
0.00
0
30
60
90
120
Fluorescence (a.u.)
Systematic Name: YDL215C
Time step Standard Deviation
18
16
14
12
20.0
22.5
25.0
27.5
30.0
Time step Mean
Cluster
(Isolation)
1 (−)
2 (−)
3 (−)
Time Step
15
10
5

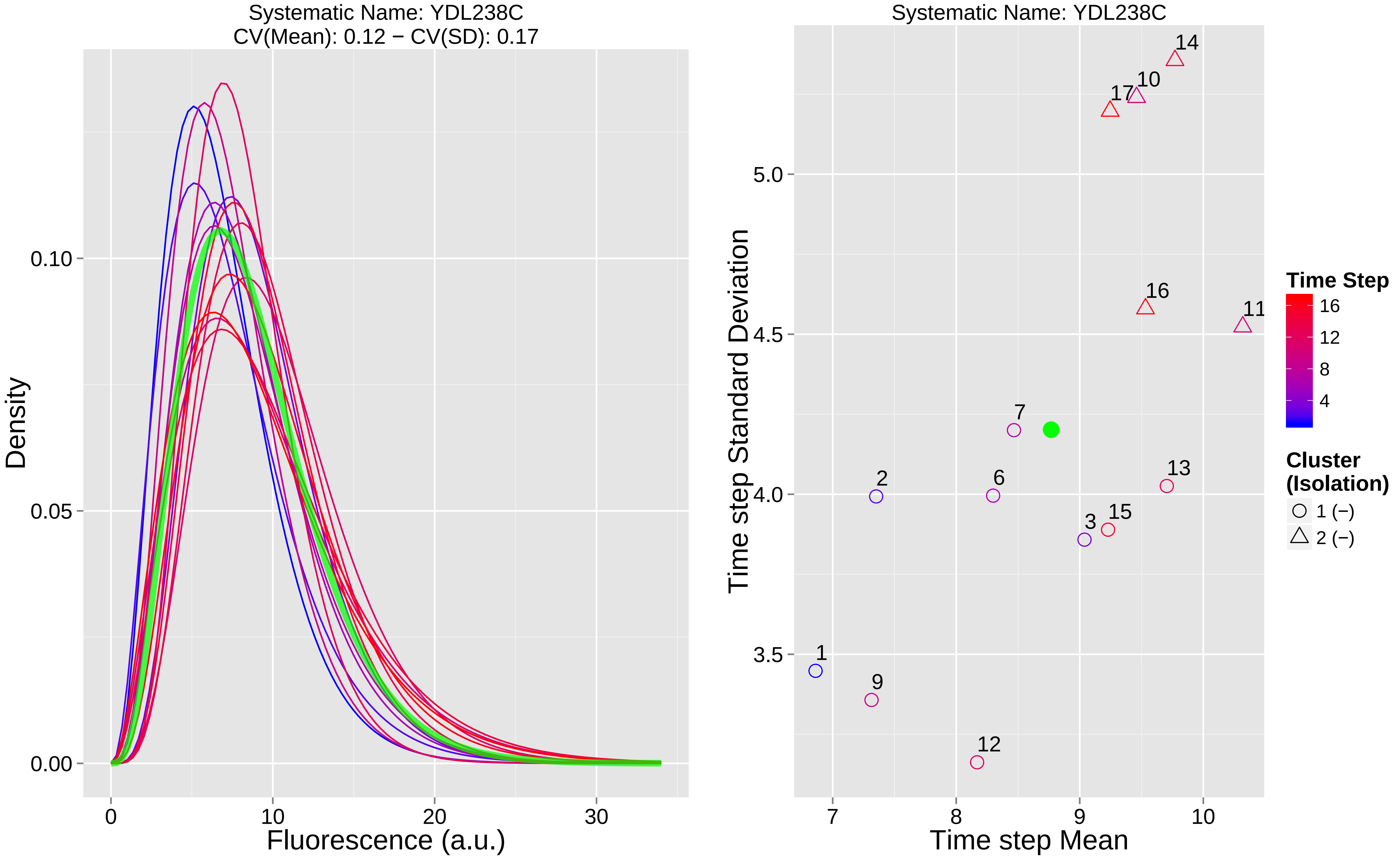

Systematic Name: YDL238C
CV(Mean): 0.12 − CV(SD): 0.17
Density
0.00
0.05
0.10
0
10
20
30
Fluorescence (a.u.)
Systematic Name: YDL238C
Time step Standard Deviation
3.5
4.0
4.5
5.0
7
8
9
10
Time step Mean
1
2
3
6
7
9
10
11
12
13
14
15
16
17
Time Step
16
12
8
4
Cluster
(Isolation)
1 (−)
2 (−)

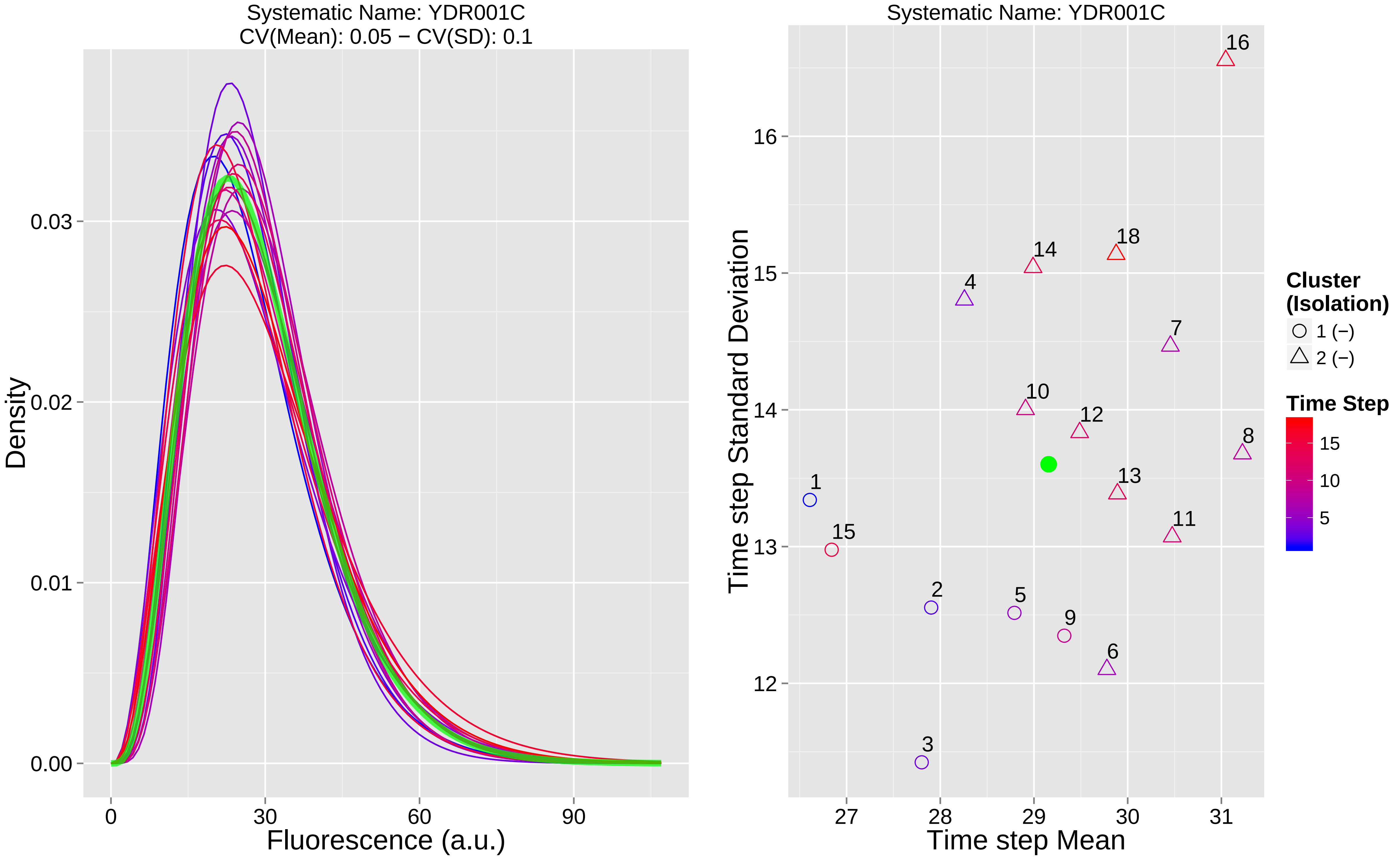

Systematic Name: YDR001C
CV(Mean): 0.05 − CV(SD): 0.1
Density
0.00
0.01
0.02
0.03
0
30
60
90
Fluorescence (a.u.)
Systematic Name: YDR001C
Time step Standard Deviation
12
13
14
15
16
27
28
29
30
31
Time step Mean
1
2
3
4
5
6
7
8
9
10
11
12
13
14
15
16
18
Cluster
(Isolation)
1 (−)
2 (−)
Time Step
15
10
5

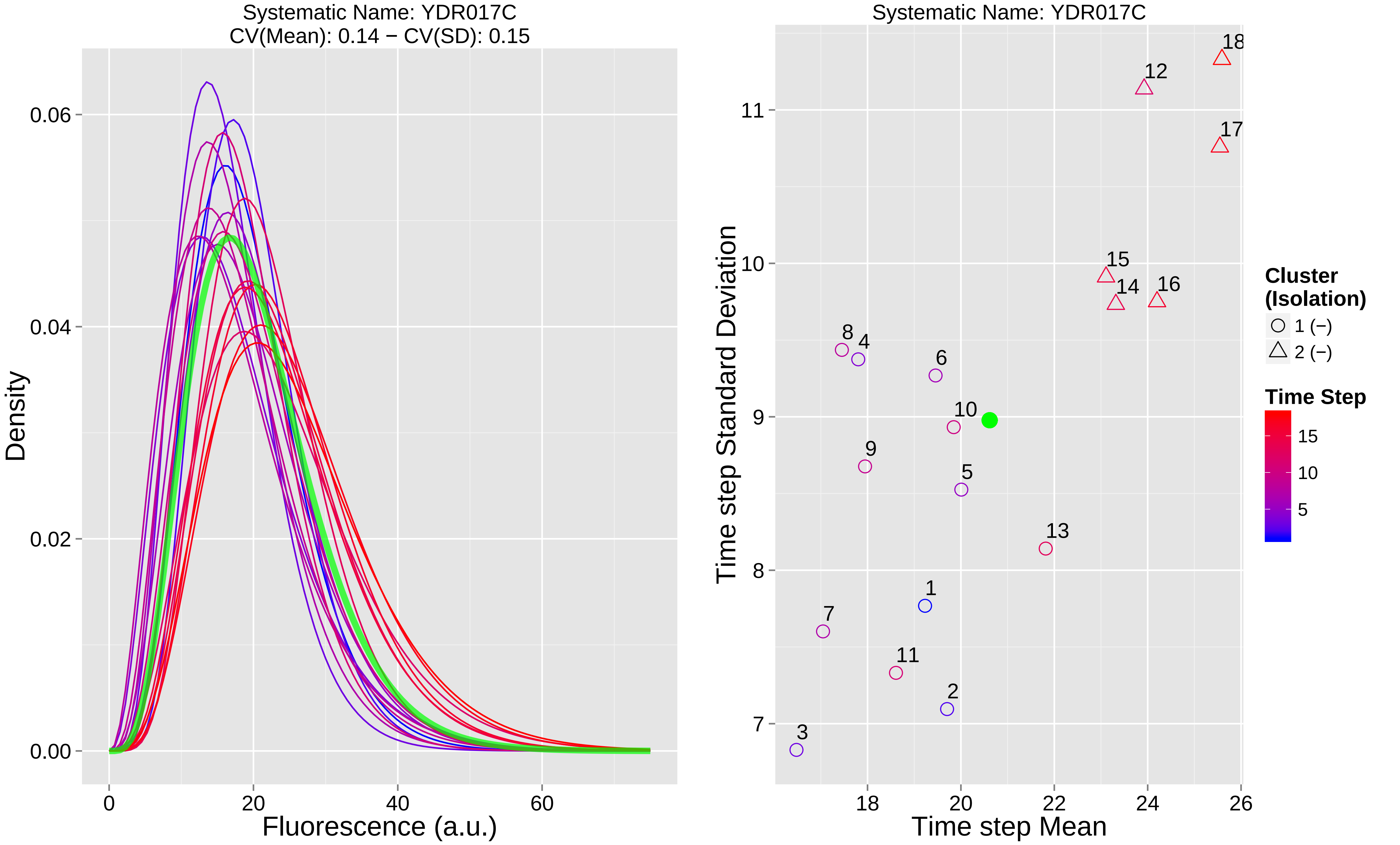

Systematic Name: YDR017C
CV(Mean): 0.14 − CV(SD): 0.15
Density
0.00
0.02
0.04
0.06
0
20
40
60
Fluorescence (a.u.)
Systematic Name: YDR017C
Time step Standard Deviation
7
8
9
10
11
18
20
22
24
26
Time step Mean
1
2
3
4
5
6
7
8
9
10
11
12
13
14
15
16
17
18
Cluster (Isolation)
1 (−)
2 (−)
Time Step
5
10
15

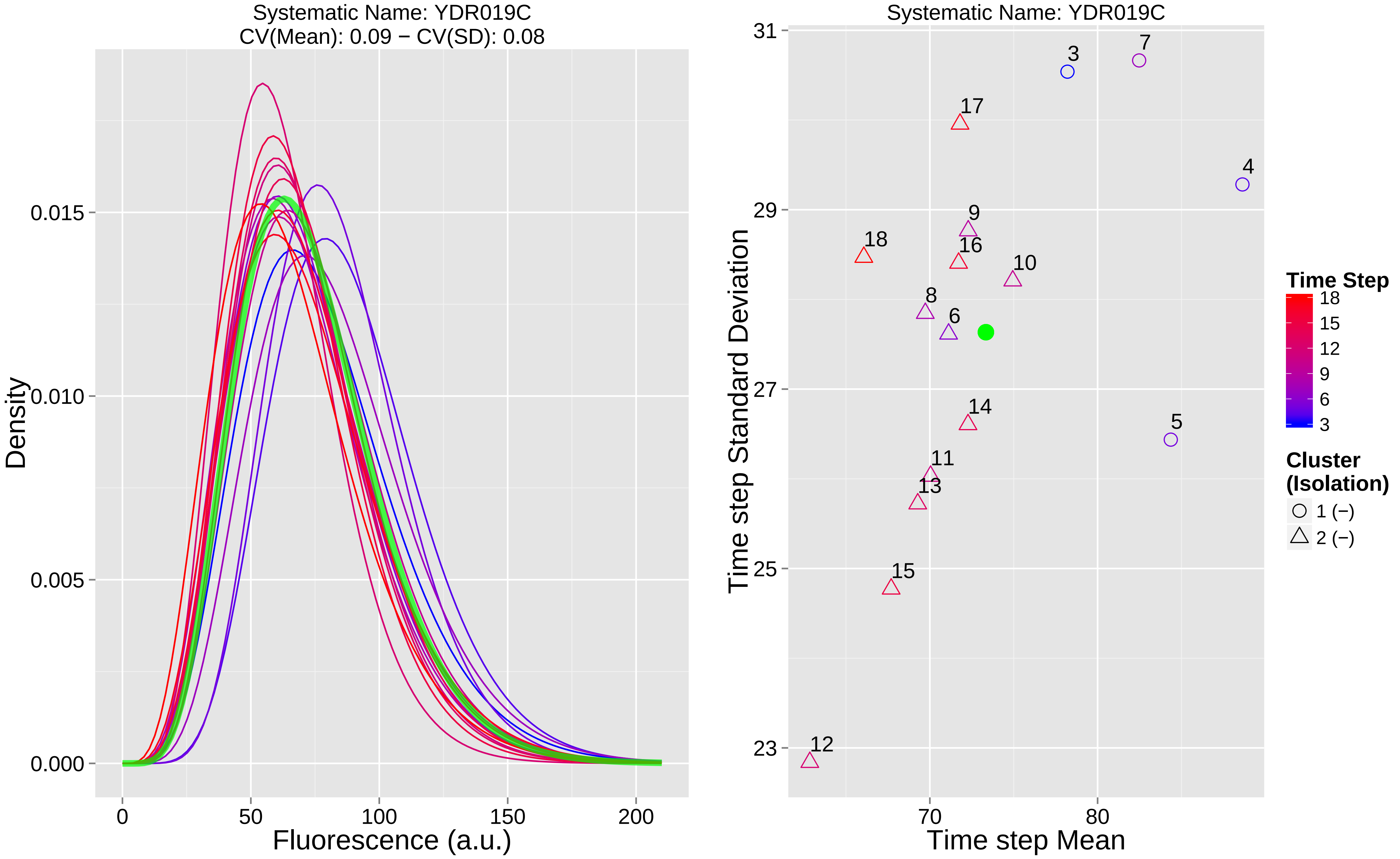

Systematic Name: YDR019C
CV(Mean): 0.09 − CV(SD): 0.08
Density
0.000
0.005
0.010
0.015
0
50
100
150
200
Fluorescence (a.u.)
Systematic Name: YDR019C
Time step Standard Deviation
23
25
27
29
31
70
80
Time step Mean
3
7
17
4
9
18
16
10
8
6
14
5
11
13
15
12
Time Step
18
15
12
9
6
3
Cluster (Isolation)
1 (−)
2 (−)

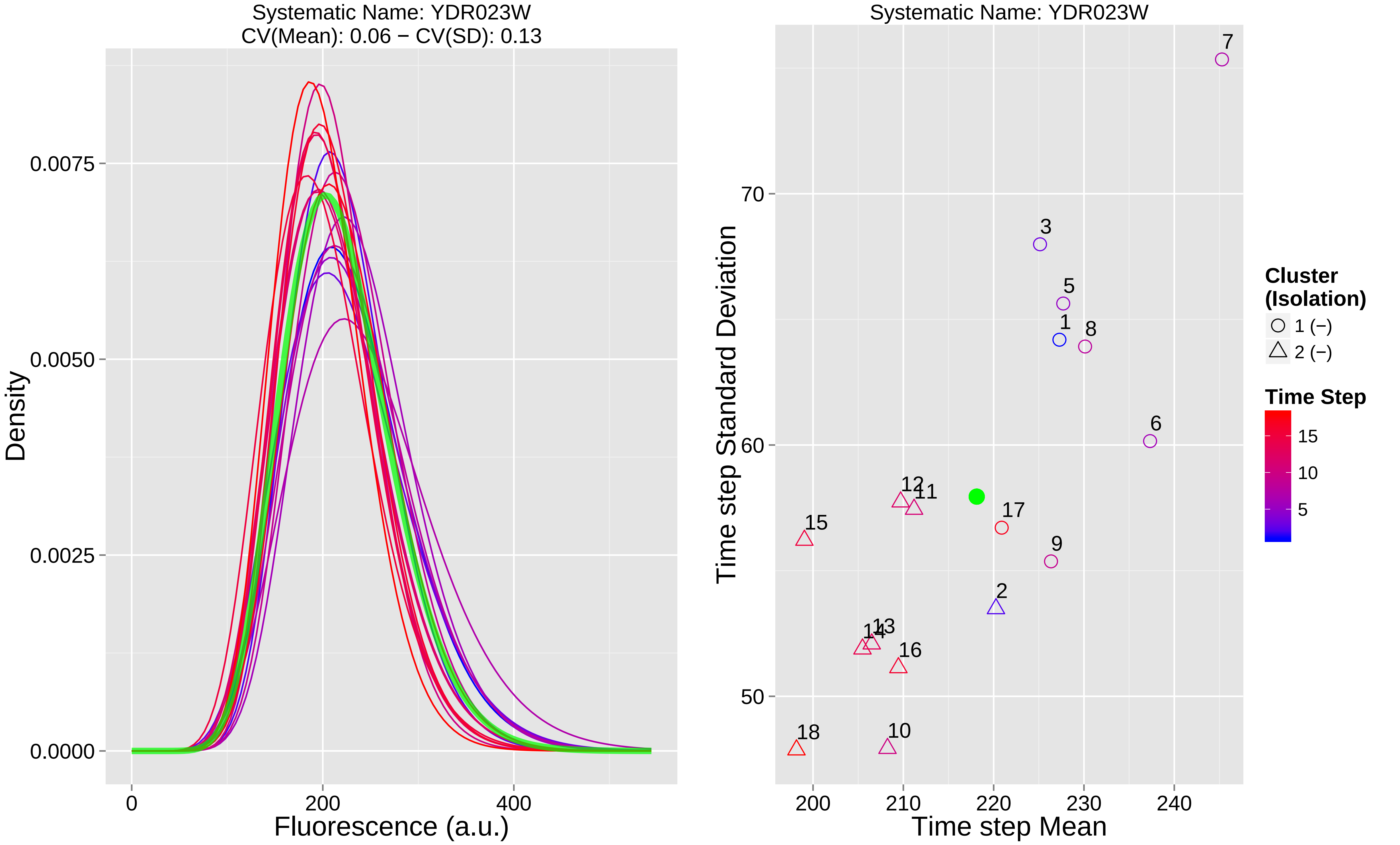
Systematic Name: YDR023W
CV(Mean): 0.06 − CV(SD): 0.13
Density
0.0075
0.0050
0.0025
0.0000
0
200
400
Fluorescence (a.u.)
Systematic Name: YDR023W
Time step Standard Deviation
70
60
50
200
210
220
230
240
Time step Mean
7
3
5
1
8
6
12
11
17
15
9
2
14
13
16
18
10
Cluster
(Isolation)
1 (−)
2 (−)
Time Step
15
10
5

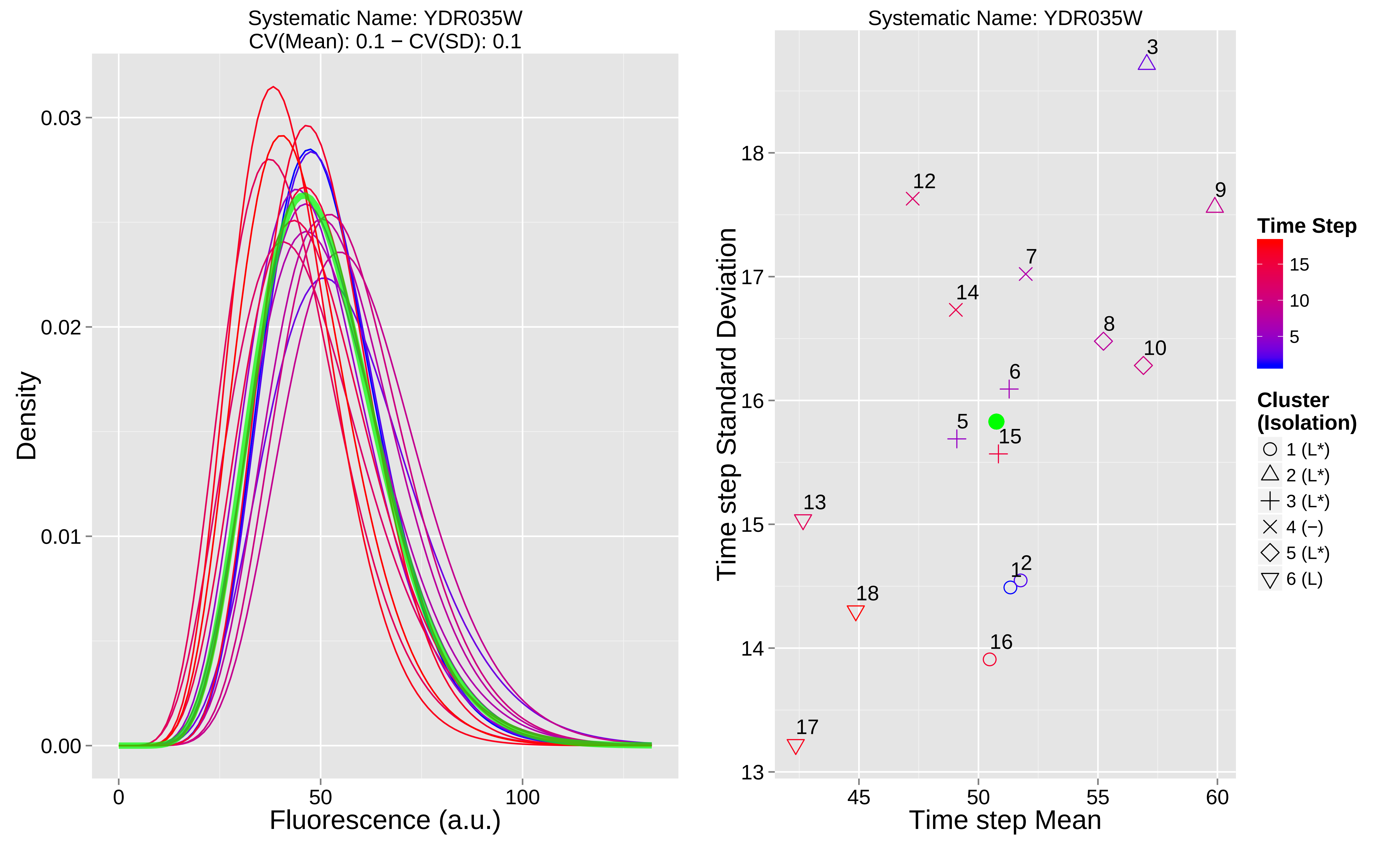

Systematic Name: YDR035W
CV(Mean): 0.1 − CV(SD): 0.1
Density
0.00
0.01
0.02
0.03
0
50
100
Fluorescence (a.u.)
Systematic Name: YDR035W
Time step Standard Deviation
Time step Mean
13
14
15
16
17
18
45
50
55
60
Time Step
15
10
5
Cluster
(Isolation)
1 (L*)
2 (L*)
3 (L*)
4 (−)
5 (L*)
6 (L)

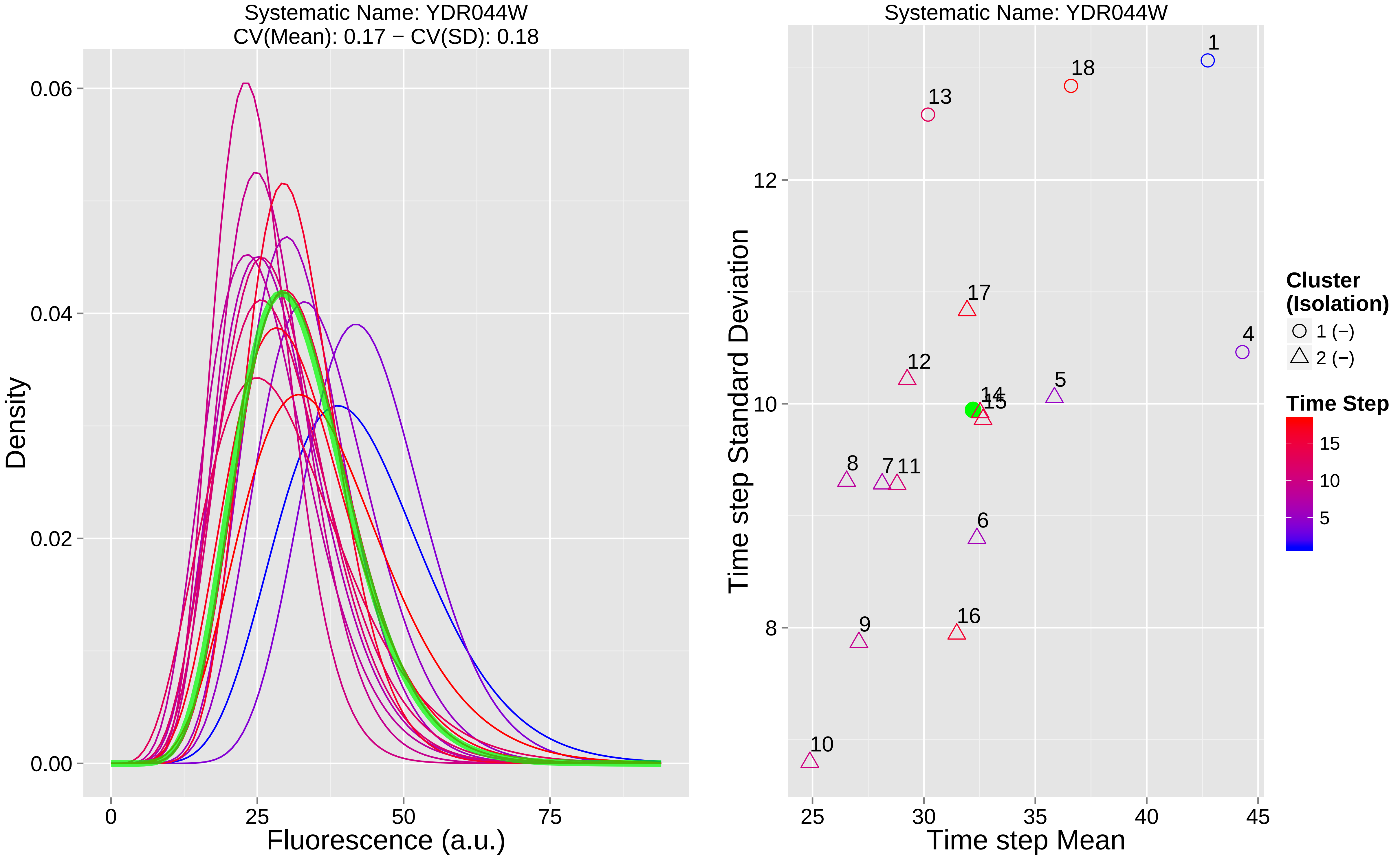

Systematic Name: YDR044W
CV(Mean): 0.17 − CV(SD): 0.18
Density
0.06
0.04
0.02
0.00
0
25
50
75
Fluorescence (a.u.)
Systematic Name: YDR044W
Time step Standard Deviation
12
10
8
25
30
35
40
45
Time step Mean
1
18
13
17
4
12
5
14
15
8
7
11
6
16
9
10
Cluster
(Isolation)
1 (−)
2 (−)
Time Step
15
10
5

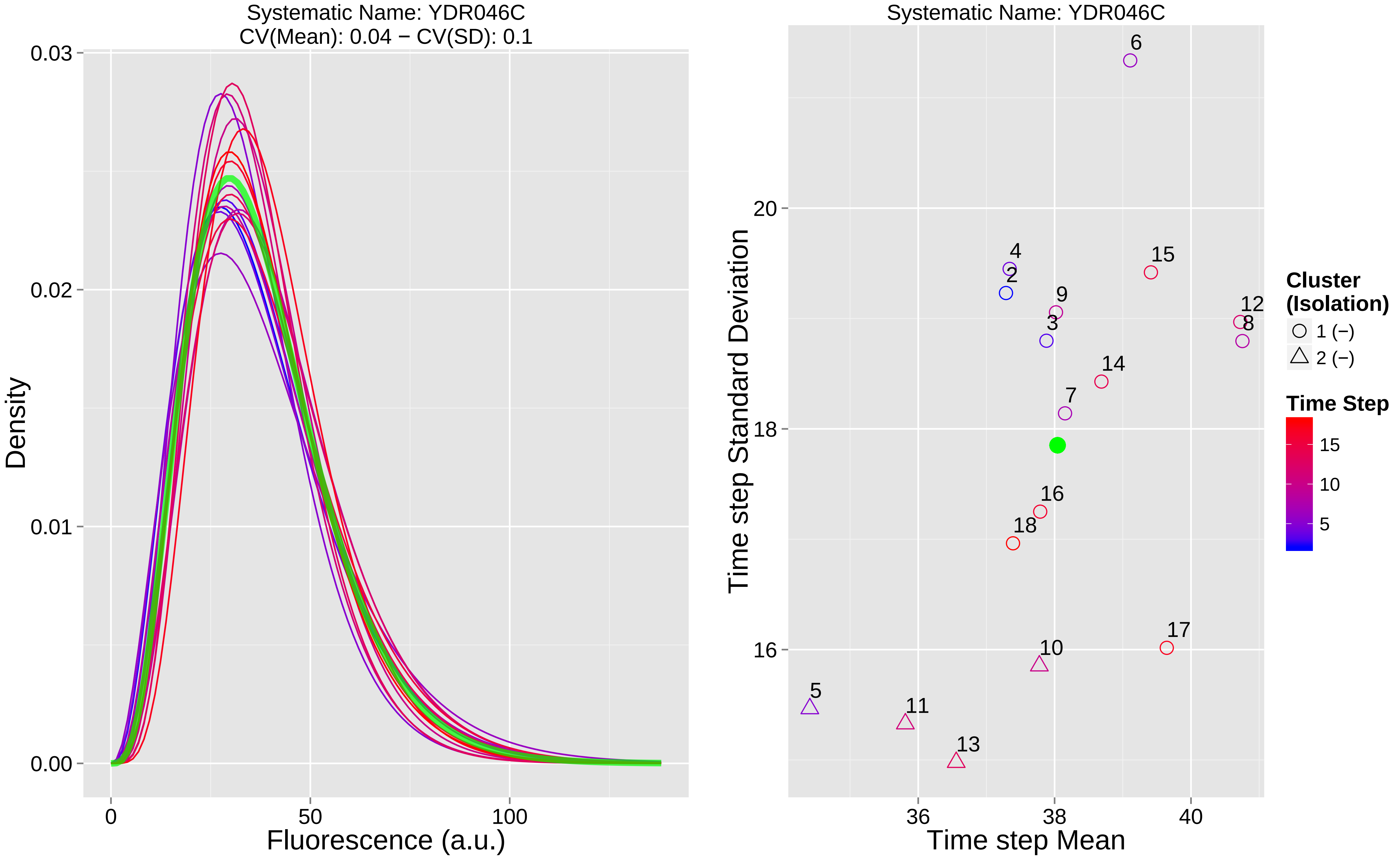
Systematic Name: YDR046C
CV(Mean): 0.04 − CV(SD): 0.1
Density
0.03
0.02
0.01
0.00
0
50
100
Fluorescence (a.u.)
Systematic Name: YDR046C
Time step Standard Deviation
20
18
16
36
38
40
Time step Mean
6
4
2
15
9
3
12
8
14
7
16
18
17
10
5
11
13
Cluster (Isolation)
1 (−)
2 (−)
Time Step
15
10
5

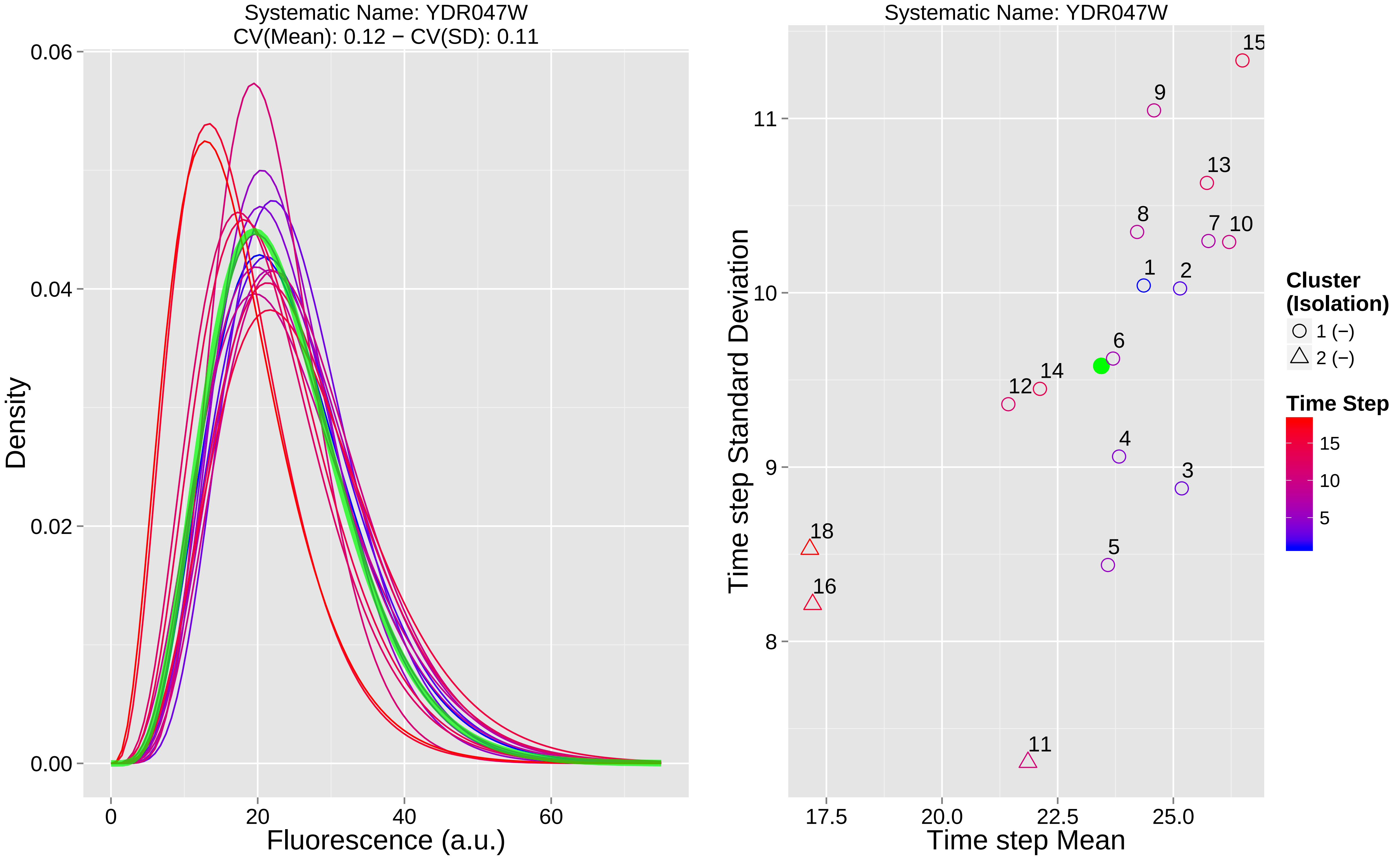

Systematic Name: YDR047W
CV(Mean): 0.12 − CV(SD): 0.11
Density
0.06
0.04
0.02
0.00
0
20
40
60
Fluorescence (a.u.)
Systematic Name: YDR047W
Time step Standard Deviation
11
10
9
8
17.5
20.0
22.5
25.0
Time step Mean
15
9
13
8
7
10
1
2
6
14
12
4
3
18
5
16
11
Cluster (Isolation)
1 (−)
2 (−)
Time Step
15
10
5

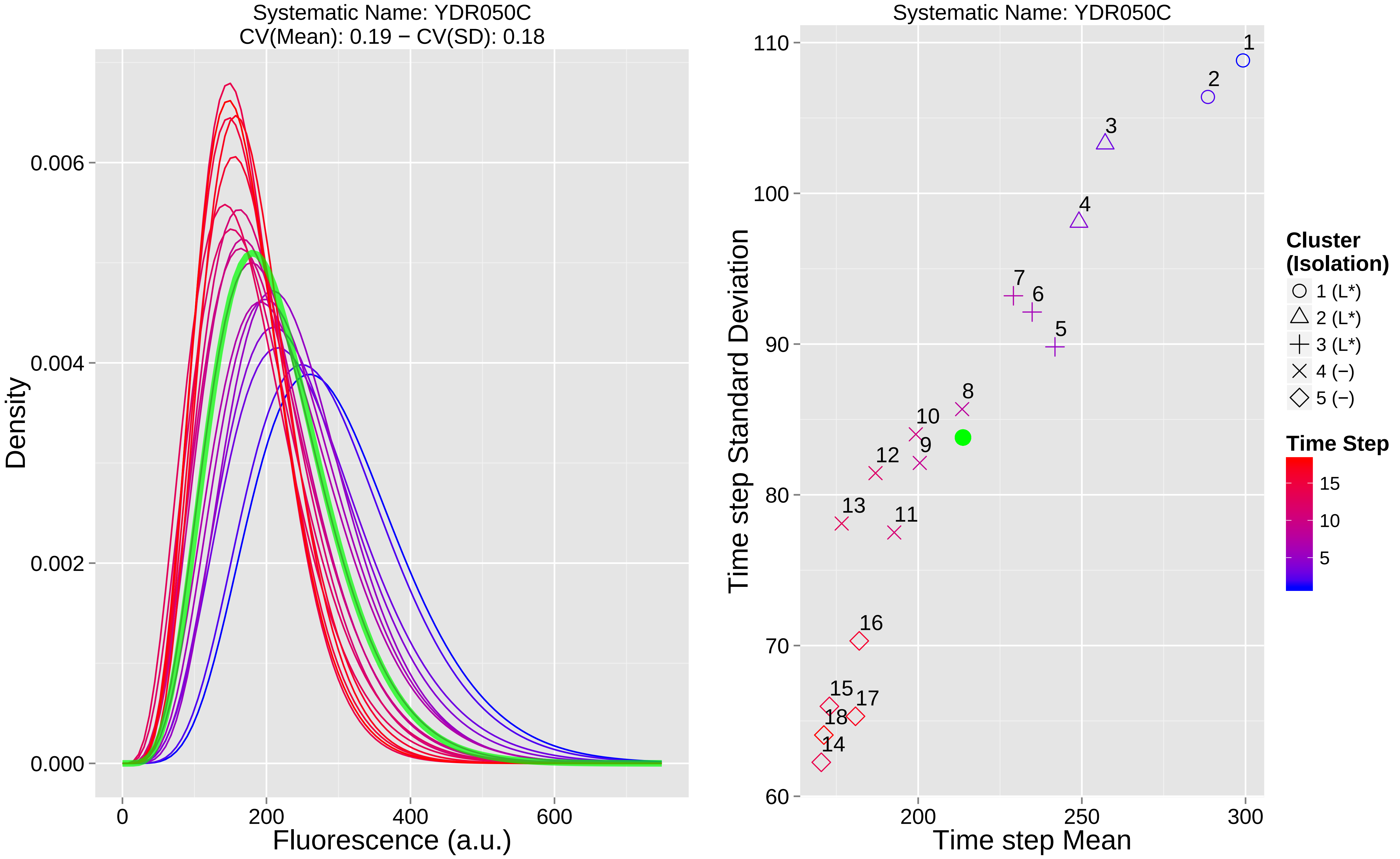

Systematic Name: YDR050C
CV(Mean): 0.19 − CV(SD): 0.18
Density
0.000
0.002
0.004
0.006
0
200
400
600
Fluorescence (a.u.)
Systematic Name: YDR050C
Time step Standard Deviation
60
70
80
90
100
110
200
250
300
Time step Mean
1
2
3
4
5
6
7
8
9
10
11
12
13
14
15
16
17
18
Cluster (Isolation)
1 (L*)
2 (L*)
3 (L*)
4 (−)
5 (−)
Time Step
15
10
5

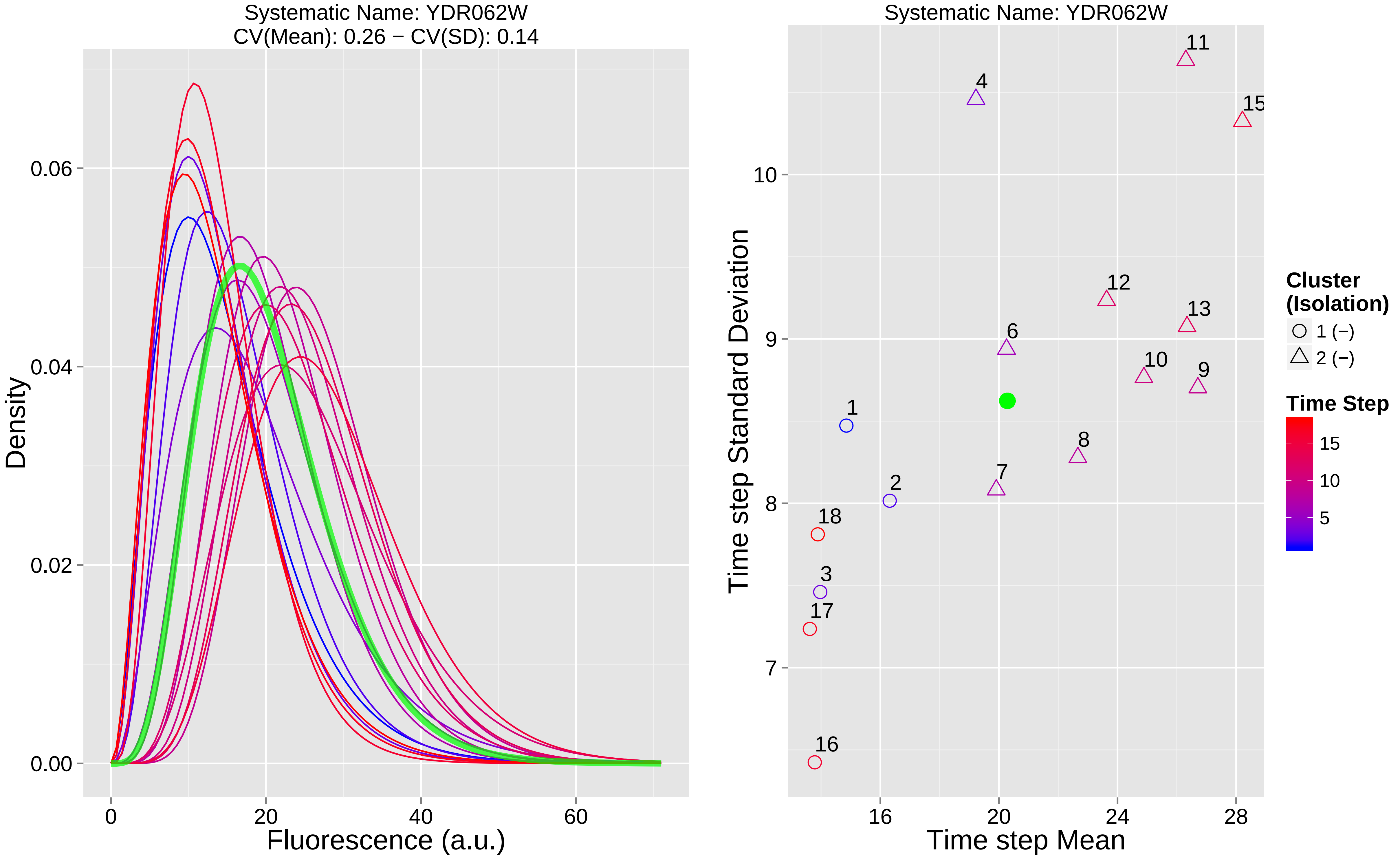

Systematic Name: YDR062W
CV(Mean): 0.26 − CV(SD): 0.14
Density
0.06
0.04
0.02
0.00
0
20
40
60
Fluorescence (a.u.)
Systematic Name: YDR062W
Time step Standard Deviation
10
9
8
7
16
20
24
28
Time step Mean
1
2
3
4
6
7
8
9
10
11
12
13
15
16
17
18
Cluster (Isolation)
1 (−)
2 (−)
Time Step
15
10
5

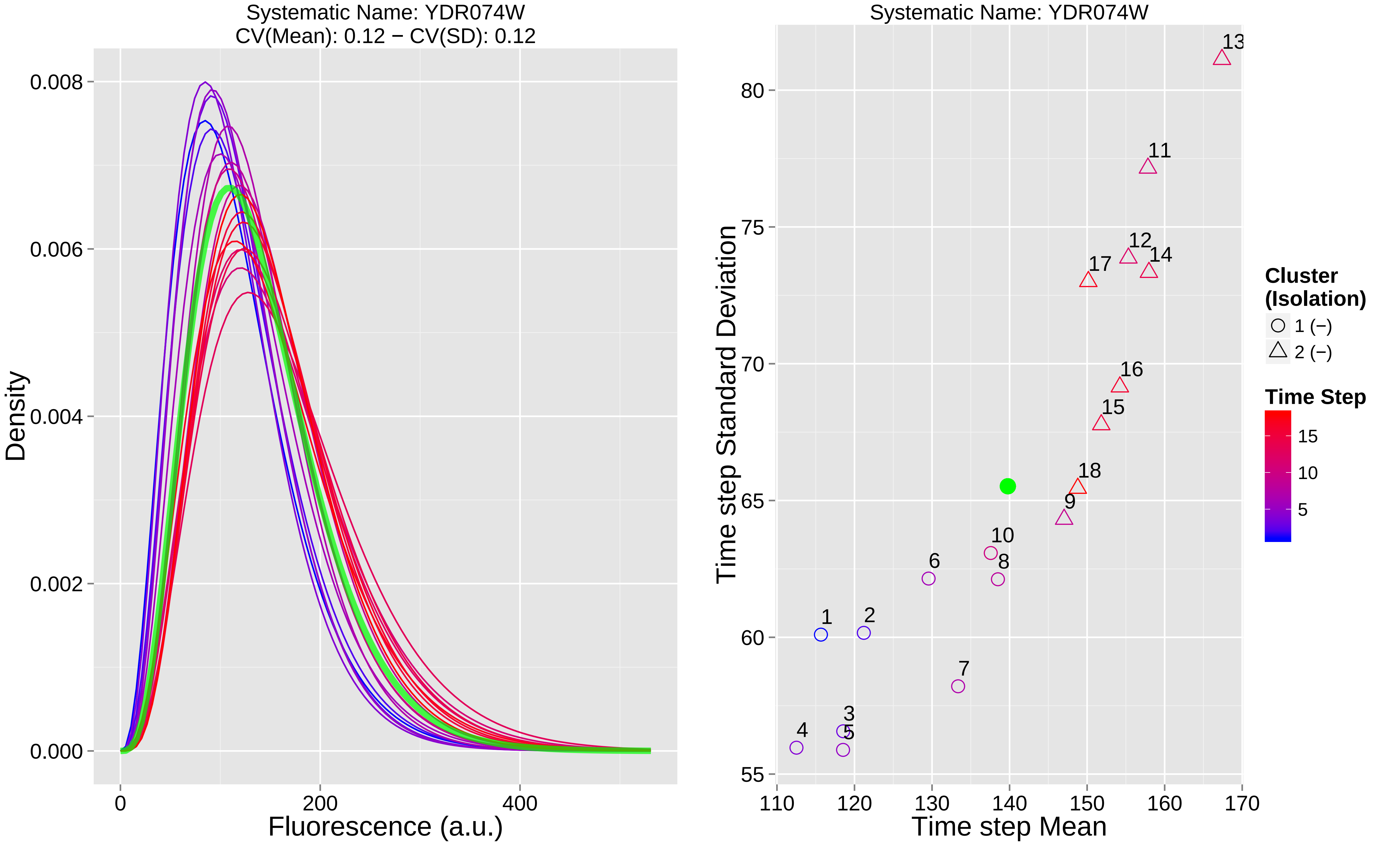

Systematic Name: YDR074W
CV(Mean): 0.12 − CV(SD): 0.12
Density
0.008
0.006
0.004
0.002
0.000
0
200
400
Fluorescence (a.u.)
Systematic Name: YDR074W
Time step Standard Deviation
80
75
70
65
60
55
110
120
130
140
150
160
170
Time step Mean
1
2
3
4
5
6
7
8
9
10
11
12
13
14
15
16
17
18
Cluster
(Isolation)
1 (−)
2 (−)
Time Step
15
10
5

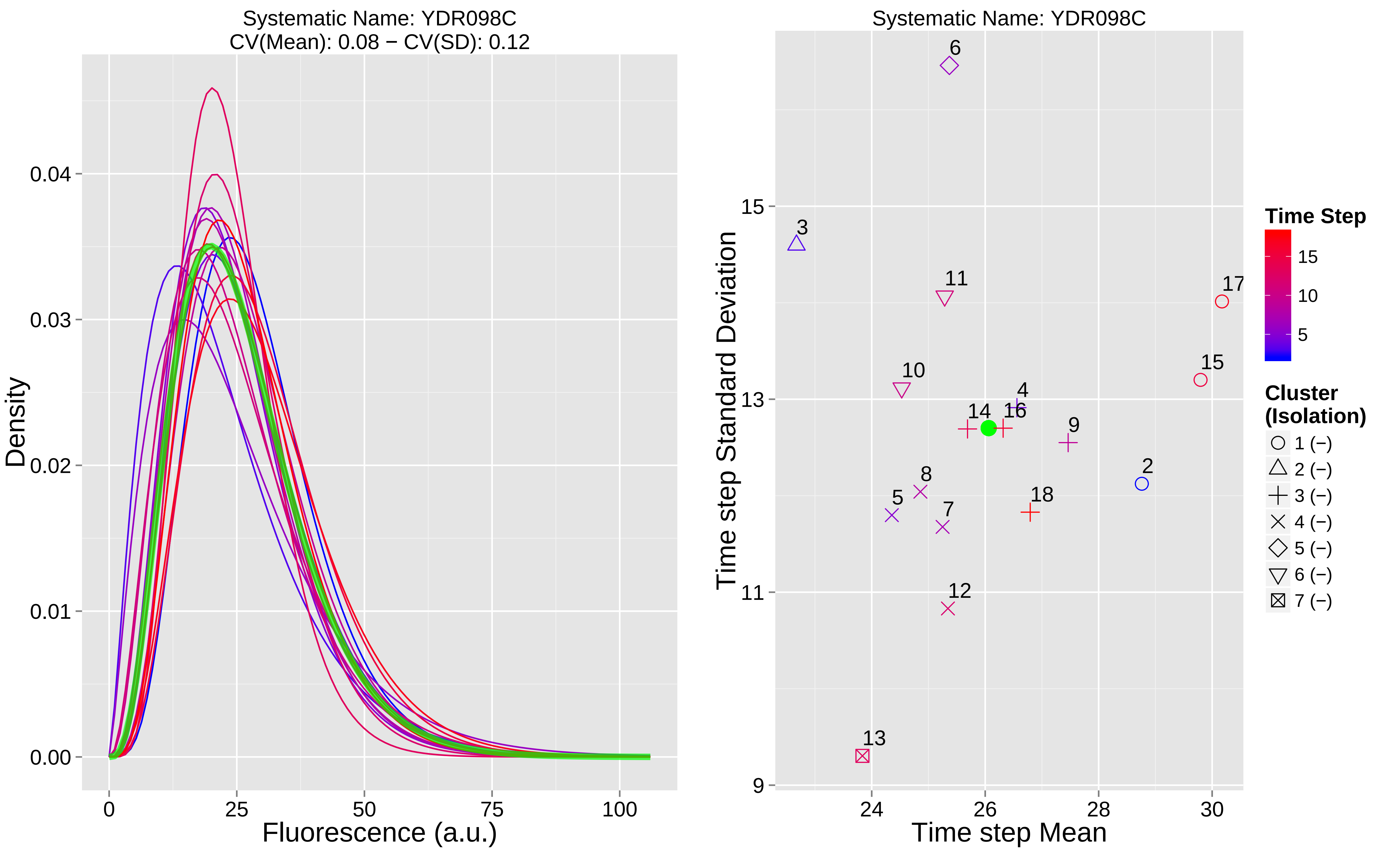

Systematic Name: YDR098C
CV(Mean): 0.08 − CV(SD): 0.12
Density
Fluorescence (a.u.)
0.00
0.01
0.02
0.03
0.04
0
25
50
75
100
Systematic Name: YDR098C
Time step Standard Deviation
Time step Mean
9
11
13
15
24
26
28
30
Time Step
15
10
5
Cluster (Isolation)
1 (−)
2 (−)
3 (−)
4 (−)
5 (−)
6 (−)
7 (−)

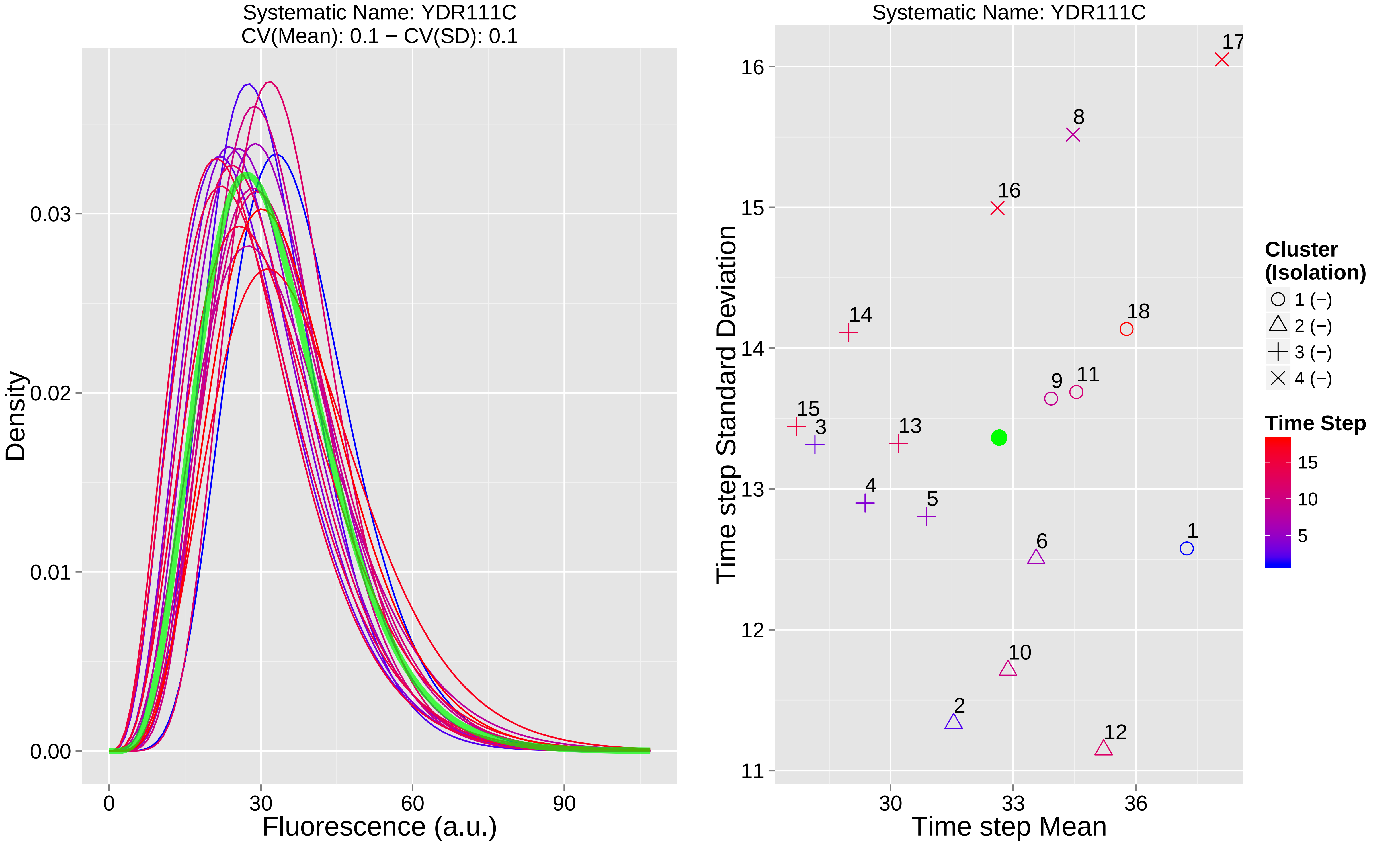

Systematic Name: YDR111C
CV(Mean): 0.1 − CV(SD): 0.1
Density
0.00
0.01
0.02
0.03
0
30
60
90
Fluorescence (a.u.)
Systematic Name: YDR111C
Time step Standard Deviation
11
12
13
14
15
16
30
33
36
Time step Mean
17
8
16
14
18
9
11
15
3
13
4
5
6
1
10
2
12
Cluster
(Isolation)
1 (−)
2 (−)
3 (−)
4 (−)
Time Step
15
10
5

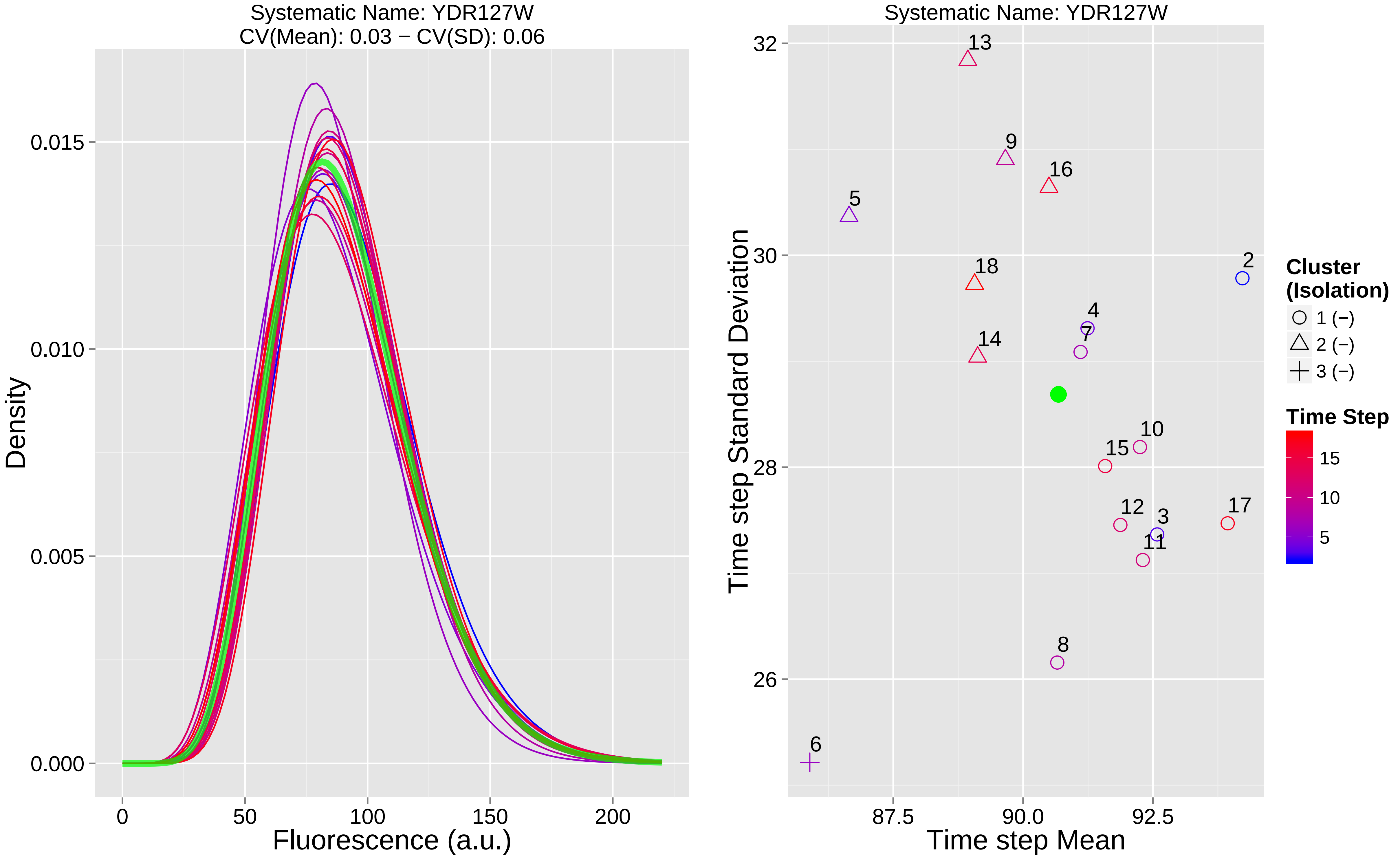

Systematic Name: YDR127W
CV(Mean): 0.03 − CV(SD): 0.06
Density
0.015
0.010
0.005
0.000
0
50
100
150
200
Fluorescence (a.u.)
Systematic Name: YDR127W
Time step Standard Deviation
32
30
28
26
87.5
90.0
92.5
Time step Mean
13
9
16
5
2
18
4
7
14
10
15
12
3
11
17
8
6
Cluster (Isolation)
1 (−)
2 (−)
3 (−)
Time Step
15
10
5

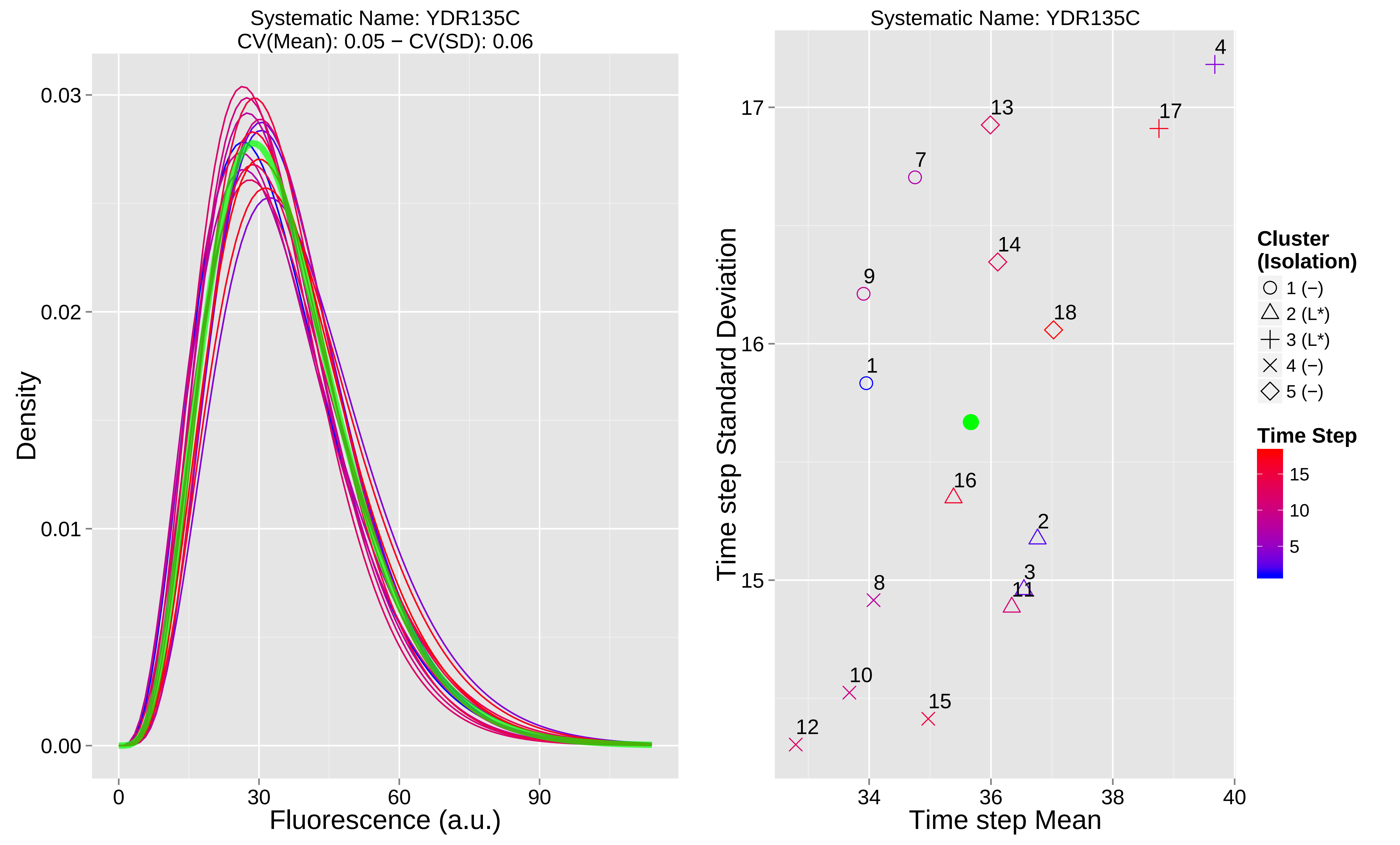
Systematic Name: YDR135C
CV(Mean): 0.05 − CV(SD): 0.06
Density
0.03
0.02
0.01
0.00
0
30
60
90
Fluorescence (a.u.)
Systematic Name: YDR135C
Time step Standard Deviation
17
16
15
34
36
38
40
Time step Mean
4
13
17
7
14
9
18
1
16
2
3
11
8
10
15
12
Cluster (Isolation)
1 (−)
2 (L*)
3 (L*)
4 (−)
5 (−)
Time Step
15
10
5

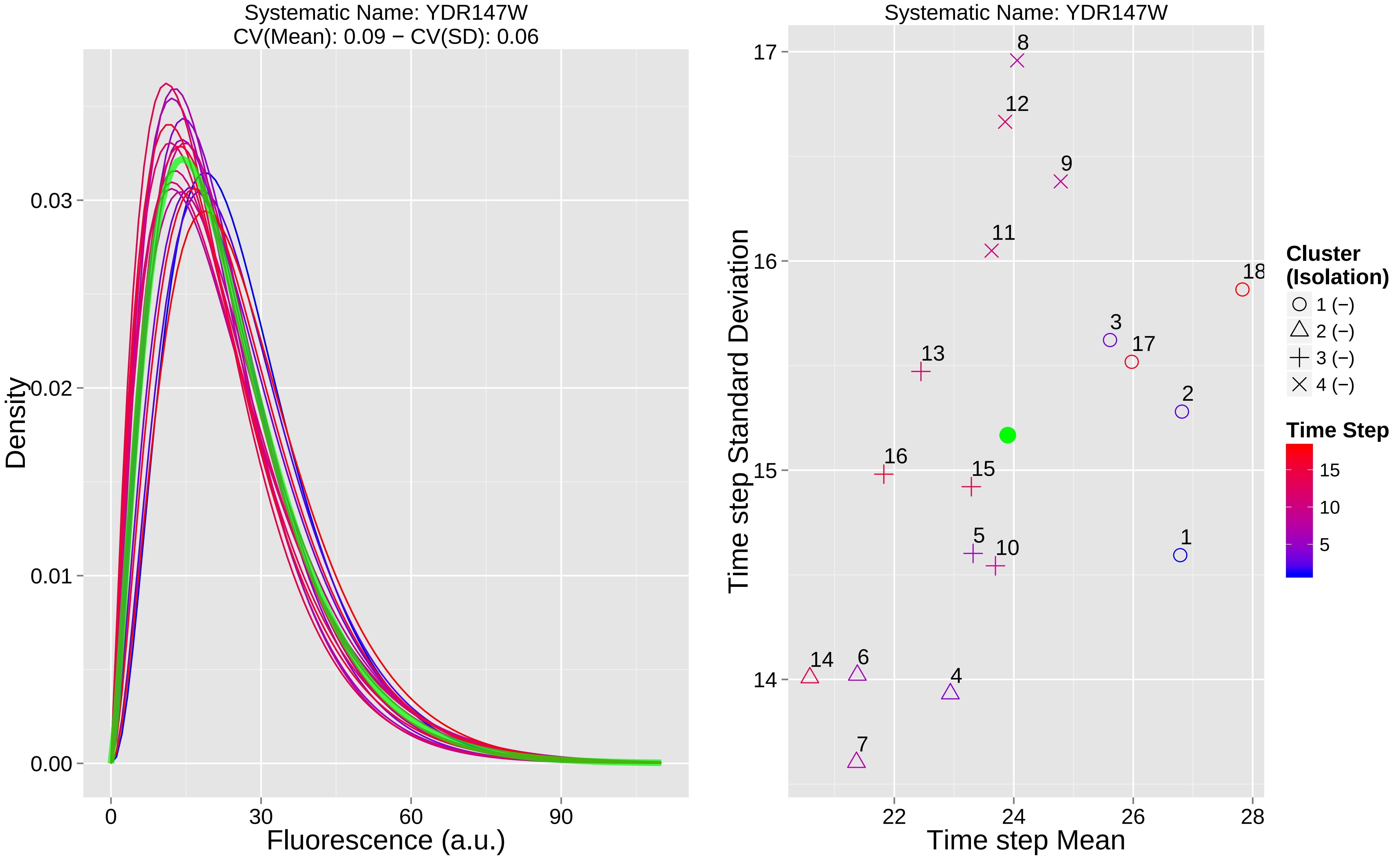
Systematic Name: YDR147W
CV(Mean): 0.09 − CV(SD): 0.06
Density
0.00
0.01
0.02
0.03
0
30
60
90
Fluorescence (a.u.)
Systematic Name: YDR147W
Time step Standard Deviation
14
15
16
17
22
24
26
28
Time step Mean
1
2
3
4
5
6
7
8
9
10
11
12
13
14
15
16
17
18
Cluster
(Isolation)
1 (−)
2 (−)
3 (−)
4 (−)
Time Step
15
10
5

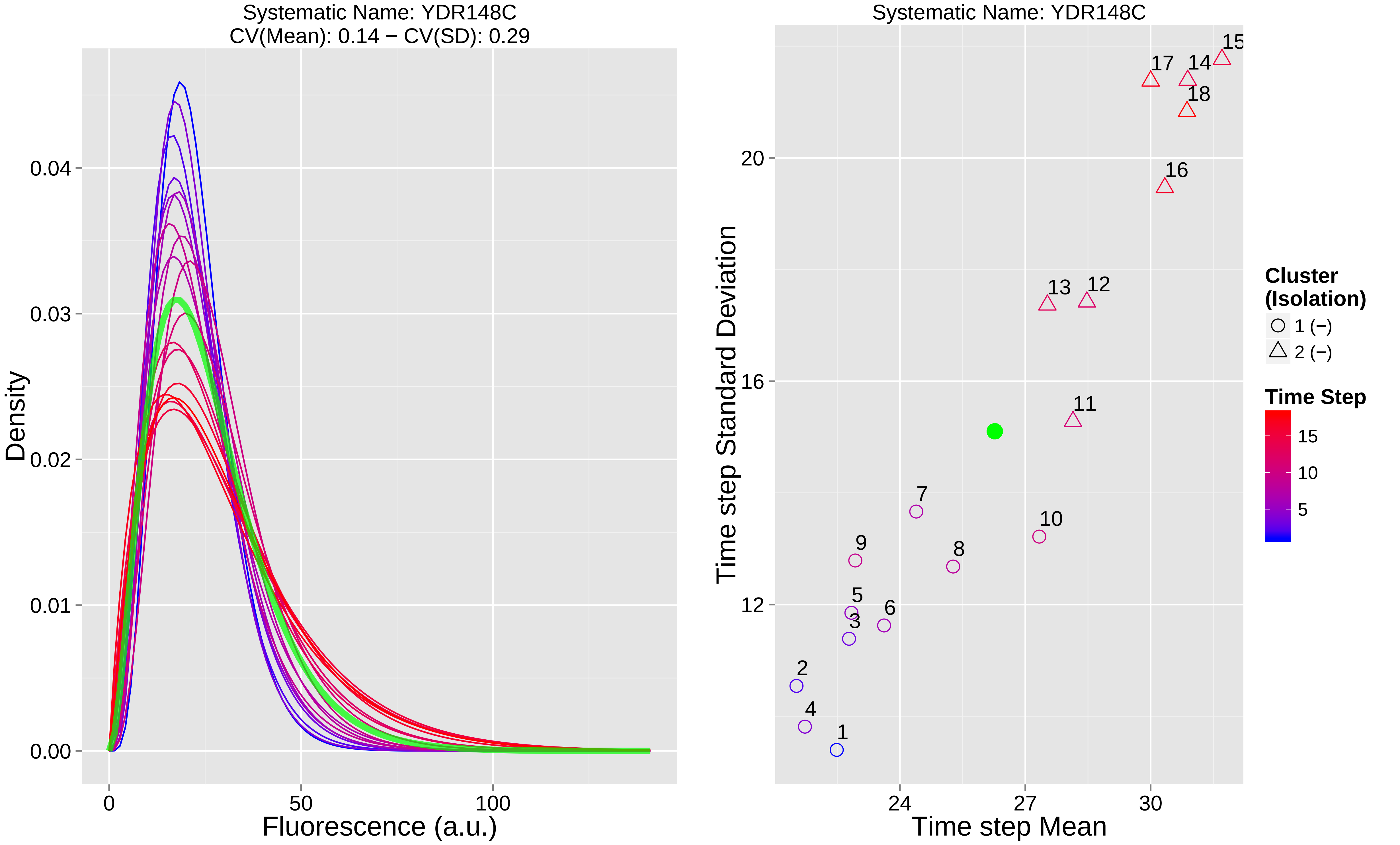

Systematic Name: YDR148C
CV(Mean): 0.14 − CV(SD): 0.29
Density
0.00
0.01
0.02
0.03
0.04
0
50
100
Fluorescence (a.u.)
Systematic Name: YDR148C
Time step Standard Deviation
12
16
20
24
27
30
Time step Mean
1
2
3
4
5
6
7
8
9
10
11
12
13
14
15
16
17
18
Cluster
(Isolation)
1 (−)
2 (−)
Time Step
5
10
15

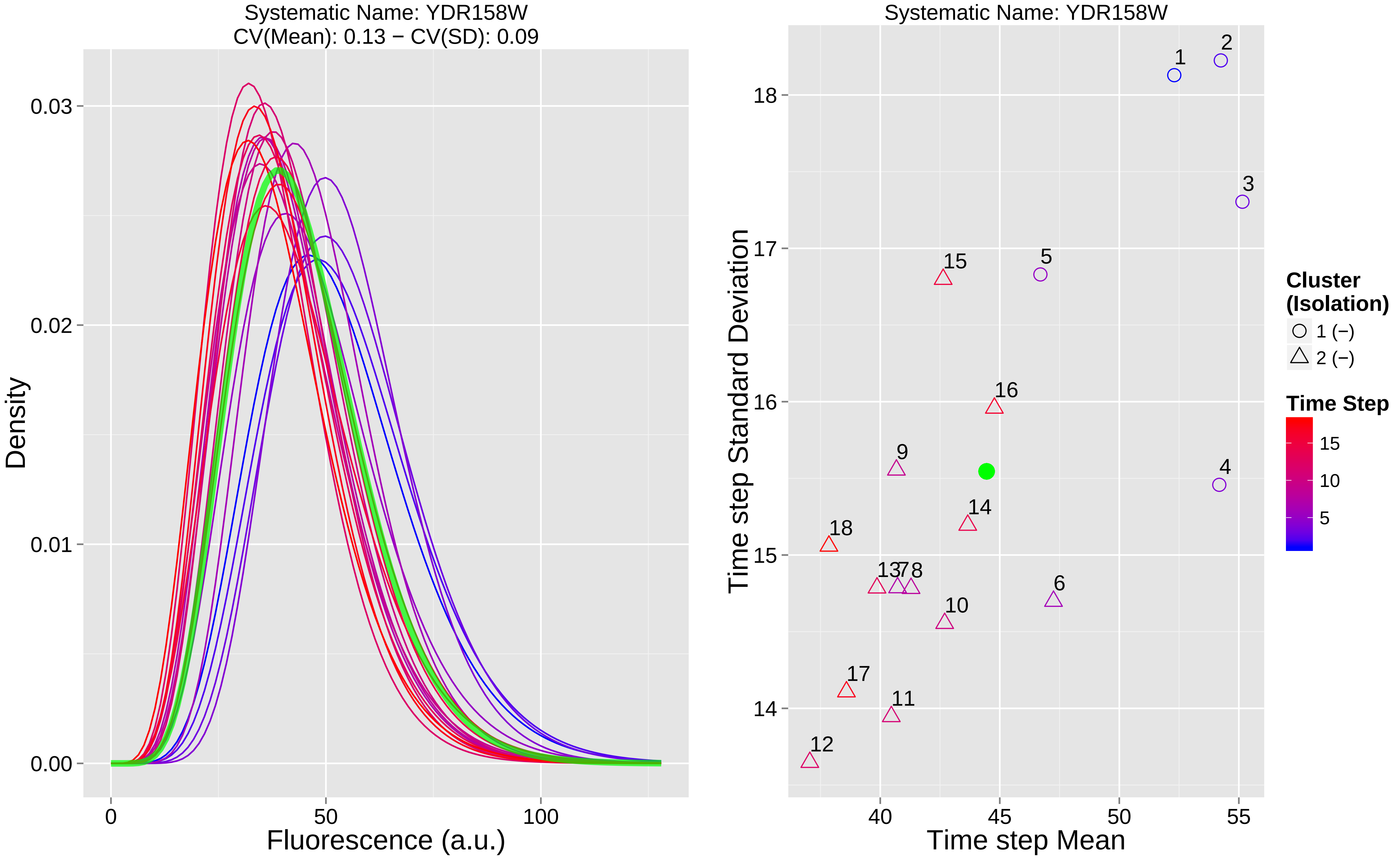

Systematic Name: YDR158W
CV(Mean): 0.13 − CV(SD): 0.09
Density
Fluorescence (a.u.)
0.00
0.01
0.02
0.03
0
50
100
Systematic Name: YDR158W
Time step Standard Deviation
Time step Mean
14
15
16
17
18
40
45
50
55
1
2
3
4
5
6
7
8
9
10
11
12
13
14
15
16
17
18
Cluster
(Isolation)
1 (−)
2 (−)
Time Step
15
10
5

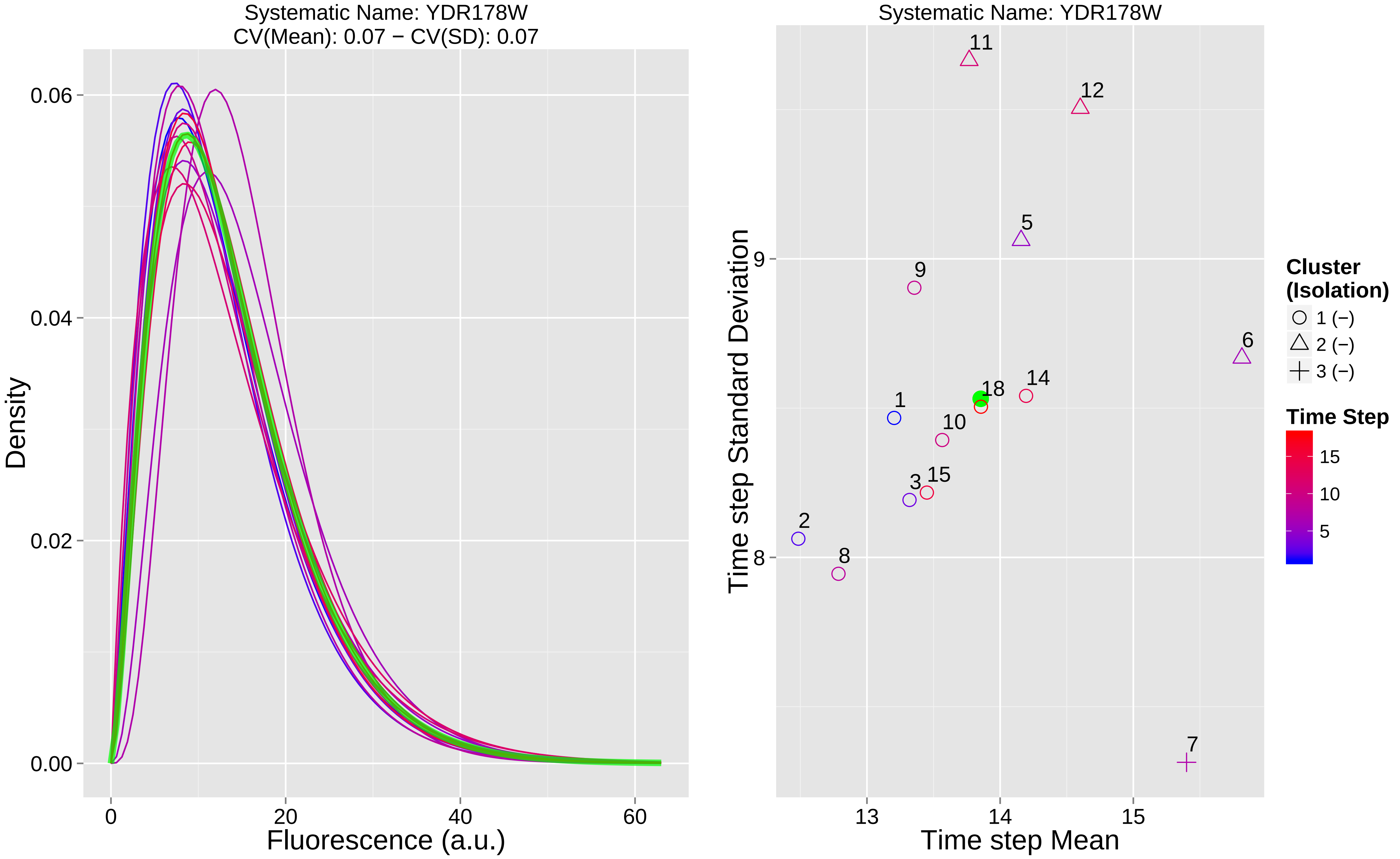
Systematic Name: YDR178W
CV(Mean): 0.07 − CV(SD): 0.07
Density
Fluorescence (a.u.)
0.00
0.02
0.04
0.06
0
20
40
60
Systematic Name: YDR178W
Time step Standard Deviation
Time step Mean
8
9
13
14
15
1
2
3
5
6
7
8
9
10
11
12
14
15
18
Cluster (Isolation)
1 (−)
2 (−)
3 (−)
Time Step
5
10
15

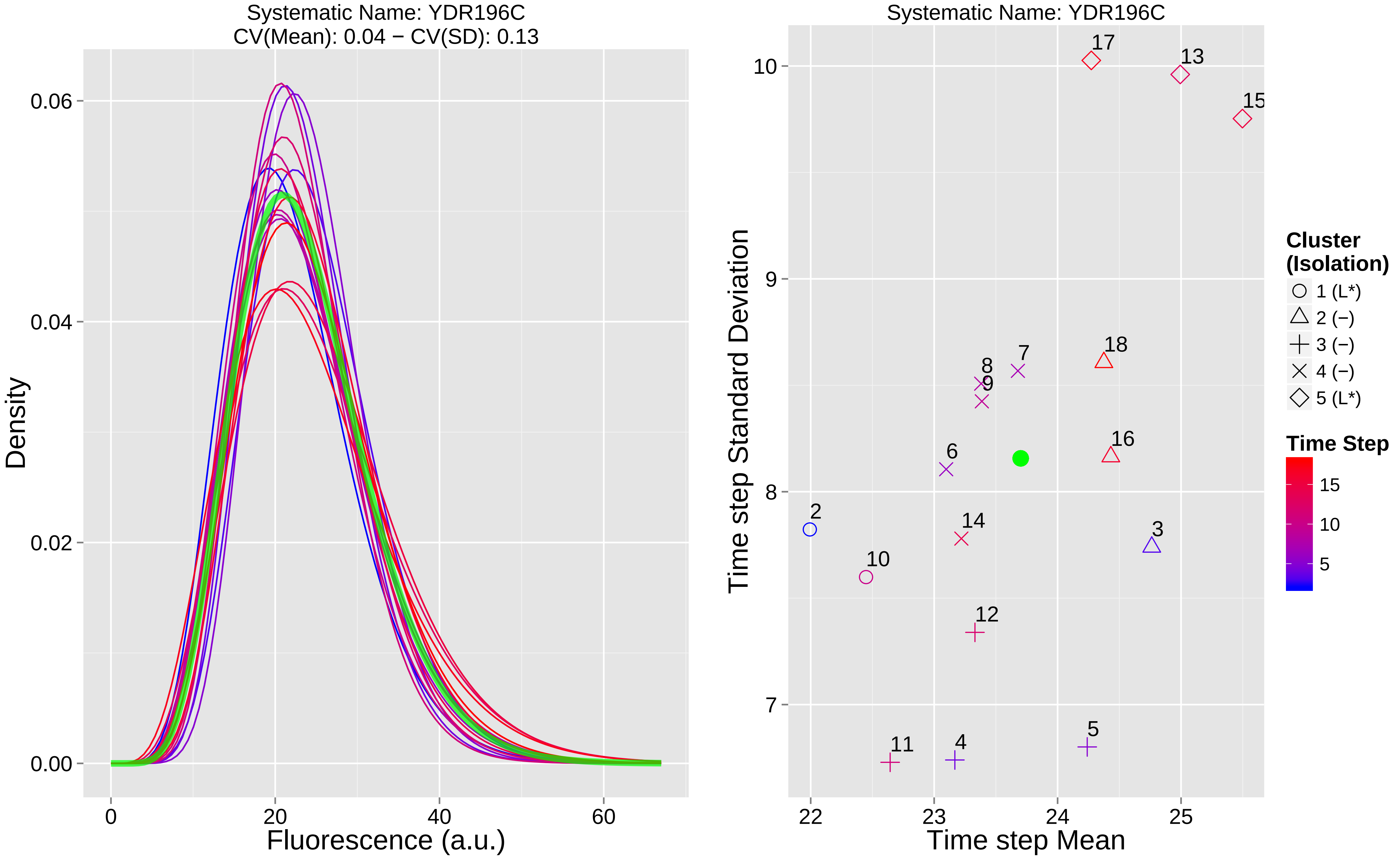
Systematic Name: YDR196C
CV(Mean): 0.04 − CV(SD): 0.13
Density
Fluorescence (a.u.)
0.00
0.02
0.04
0.06
0
20
40
60
Systematic Name: YDR196C
Time step Standard Deviation
Time step Mean
7
8
9
10
22
23
24
25
17
13
15
18
7
8
9
6
16
2
14
3
10
12
11
4
5
Cluster (Isolation)
1 (L*)
2 (−)
3 (−)
4 (−)
5 (L*)
Time Step
15
10
5

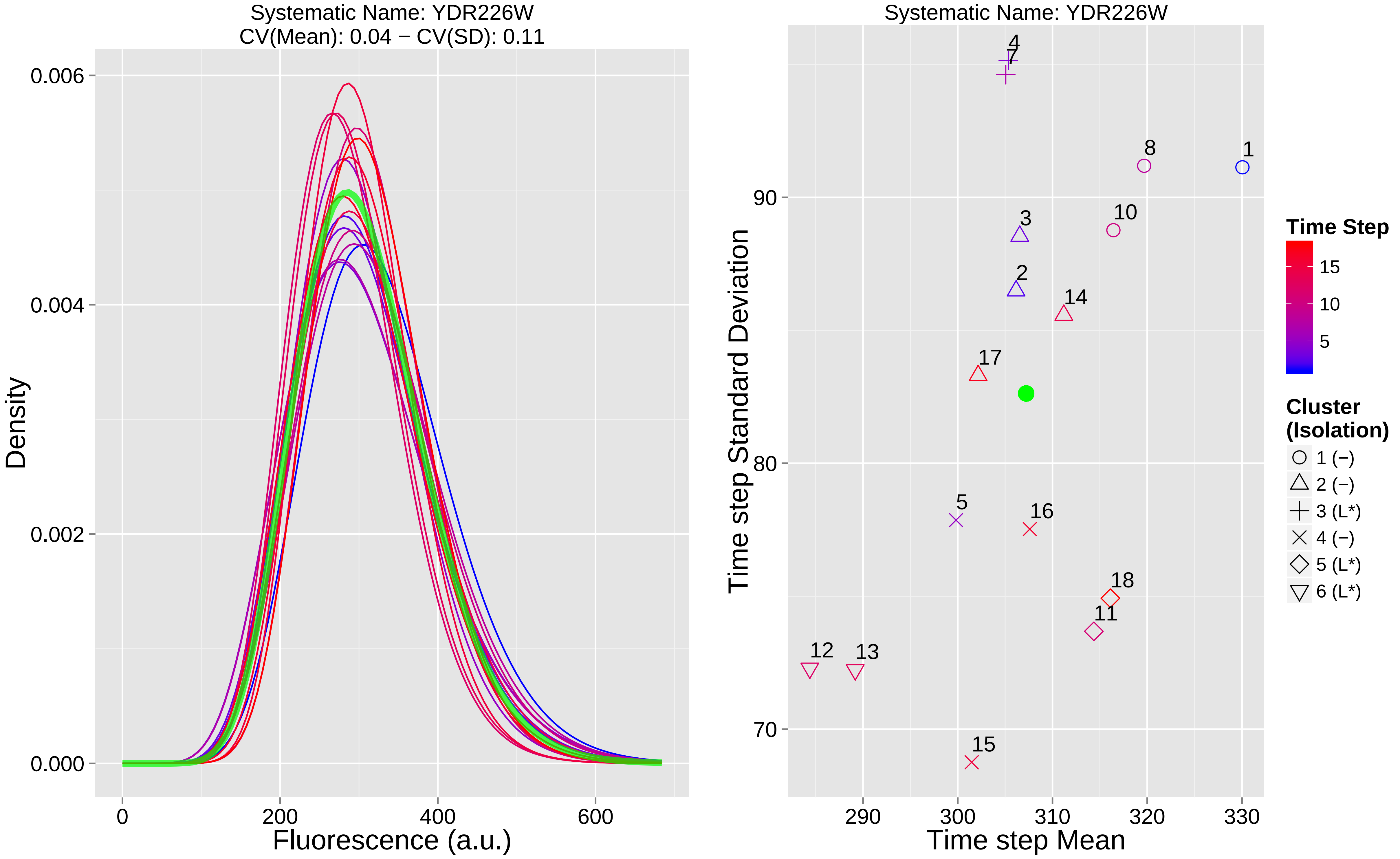
Systematic Name: YDR226W
CV(Mean): 0.04 − CV(SD): 0.11
Density
0.006
0.004
0.002
0.000
0
200
400
600
Fluorescence (a.u.)
Systematic Name: YDR226W
Time step Standard Deviation
90
80
70
290
300
310
320
330
Time step Mean
4
7
8
1
10
3
2
14
17
5
16
18
11
12
13
15
Time Step
15
10
5
Cluster
(Isolation)
1 (−)
2 (−)
3 (L*)
4 (−)
5 (L*)
6 (L*)

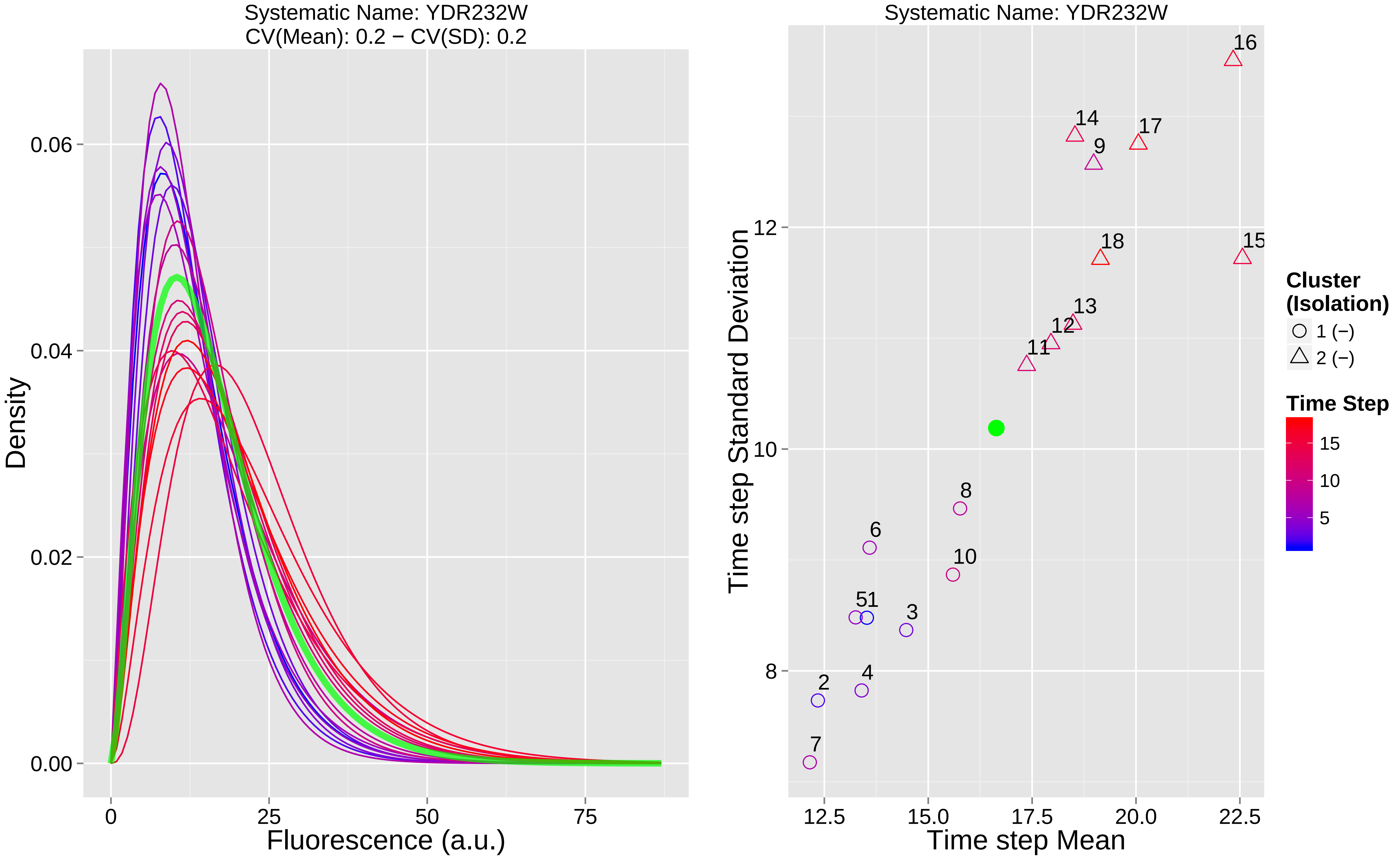

Systematic Name: YDR232W
CV(Mean): 0.2 − CV(SD): 0.2
Density
Fluorescence (a.u.)
0.00
0.02
0.04
0.06
0
25
50
75
Systematic Name: YDR232W
Time step Standard Deviation
Time step Mean
8
10
12
12.5
15.0
17.5
20.0
22.5
Cluster
(Isolation)
1 (−)
2 (−)
Time Step
5
10
15

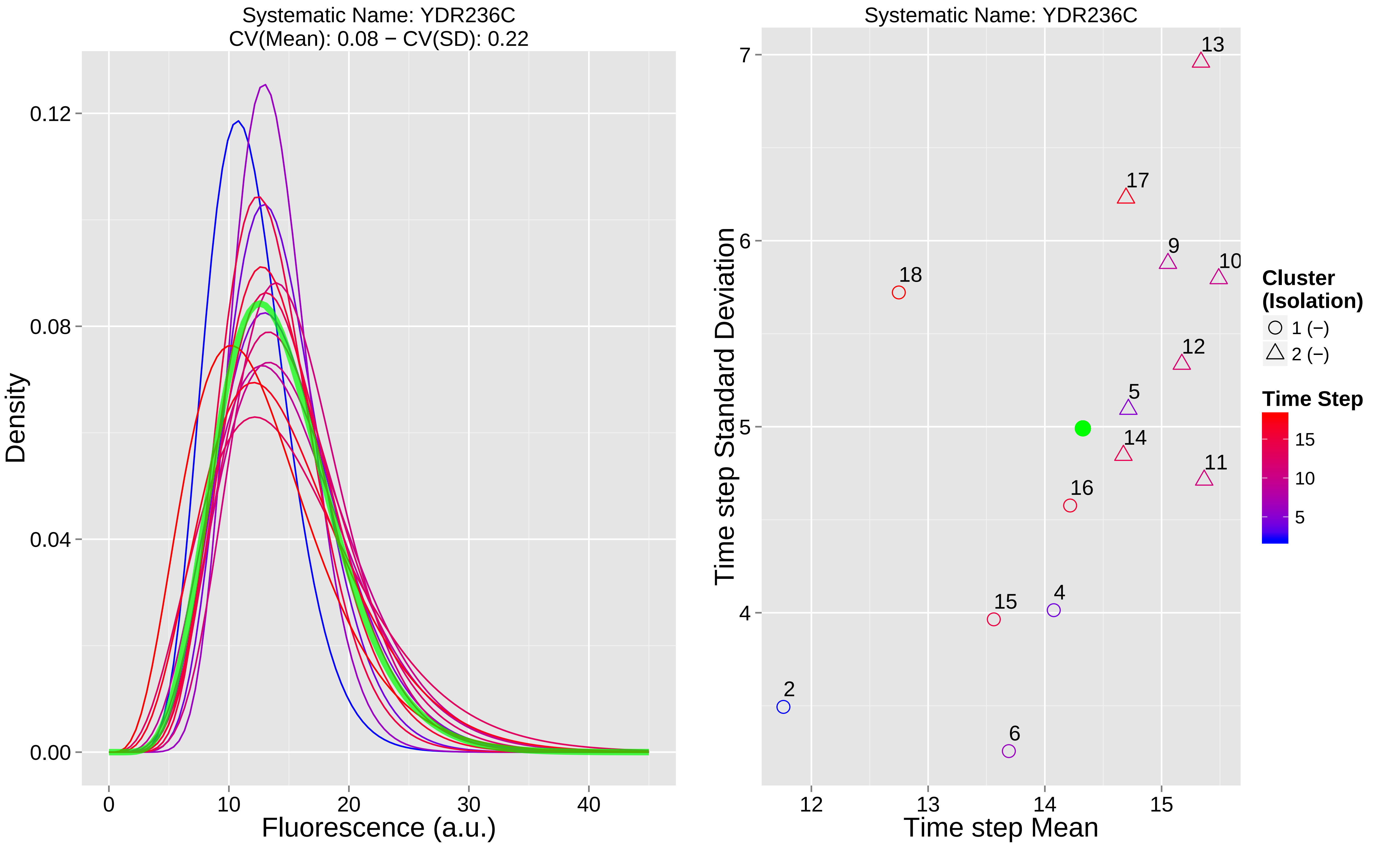

Systematic Name: YDR236C
CV(Mean): 0.08 − CV(SD): 0.22
Density
0.12
0.08
0.04
0.00
0
10
20
30
40
Fluorescence (a.u.)
Systematic Name: YDR236C
Time step Standard Deviation
7
6
5
4
12
13
14
15
Time step Mean
13
17
9
10
18
12
5
14
11
16
15
4
2
6
Cluster (Isolation)
1 (−)
2 (−)
Time Step
15
10
5

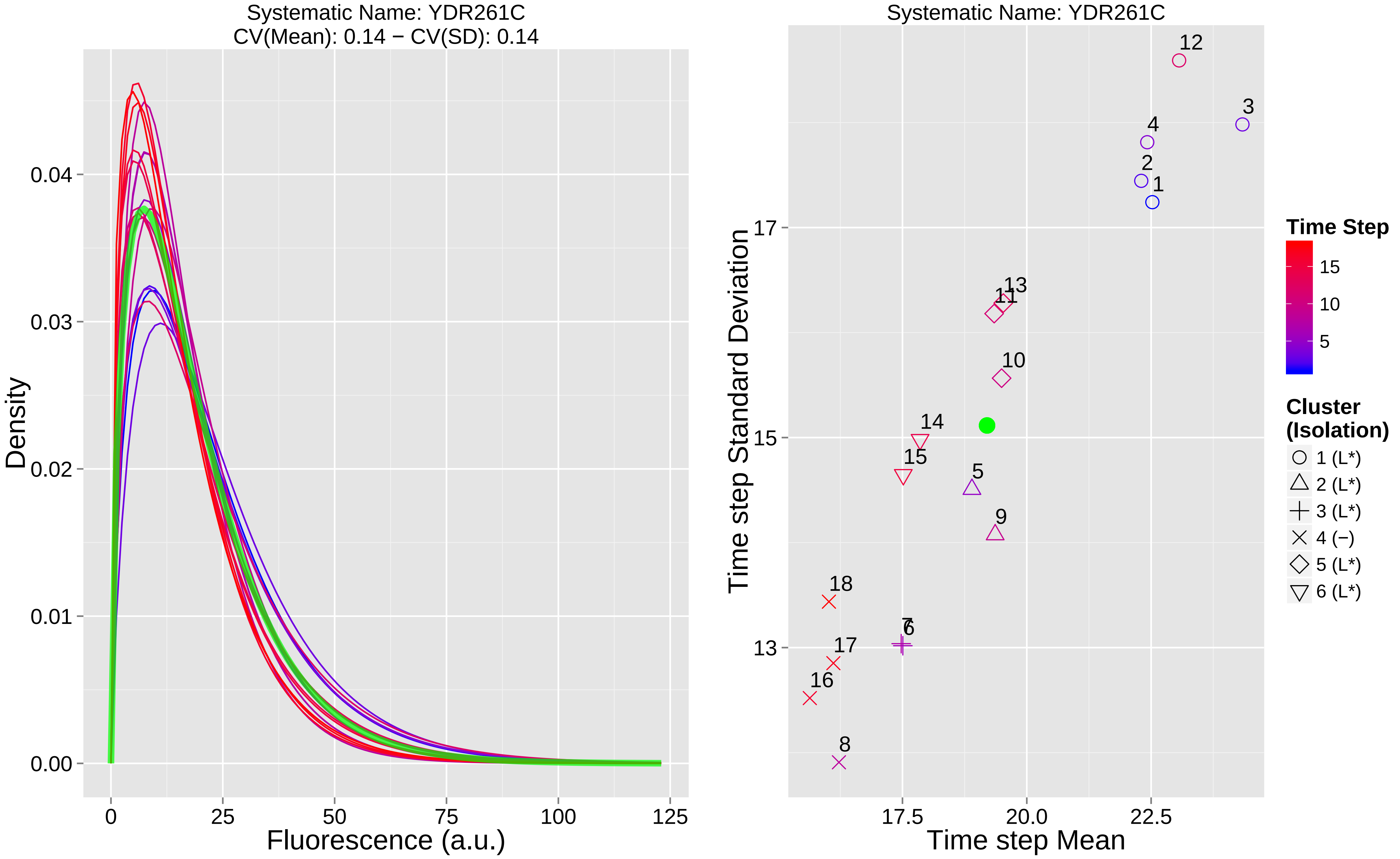

Systematic Name: YDR261C
CV(Mean): 0.14 − CV(SD): 0.14
Density
0.00
0.01
0.02
0.03
0.04
0
25
50
75
100
125
Fluorescence (a.u.)
Systematic Name: YDR261C
Time step Standard Deviation
13
15
17
17.5
20.0
22.5
Time step Mean
Time Step
15
10
5
Cluster (Isolation)
1 (L*)
2 (L*)
3 (L*)
4 (−)
5 (L*)
6 (L*)

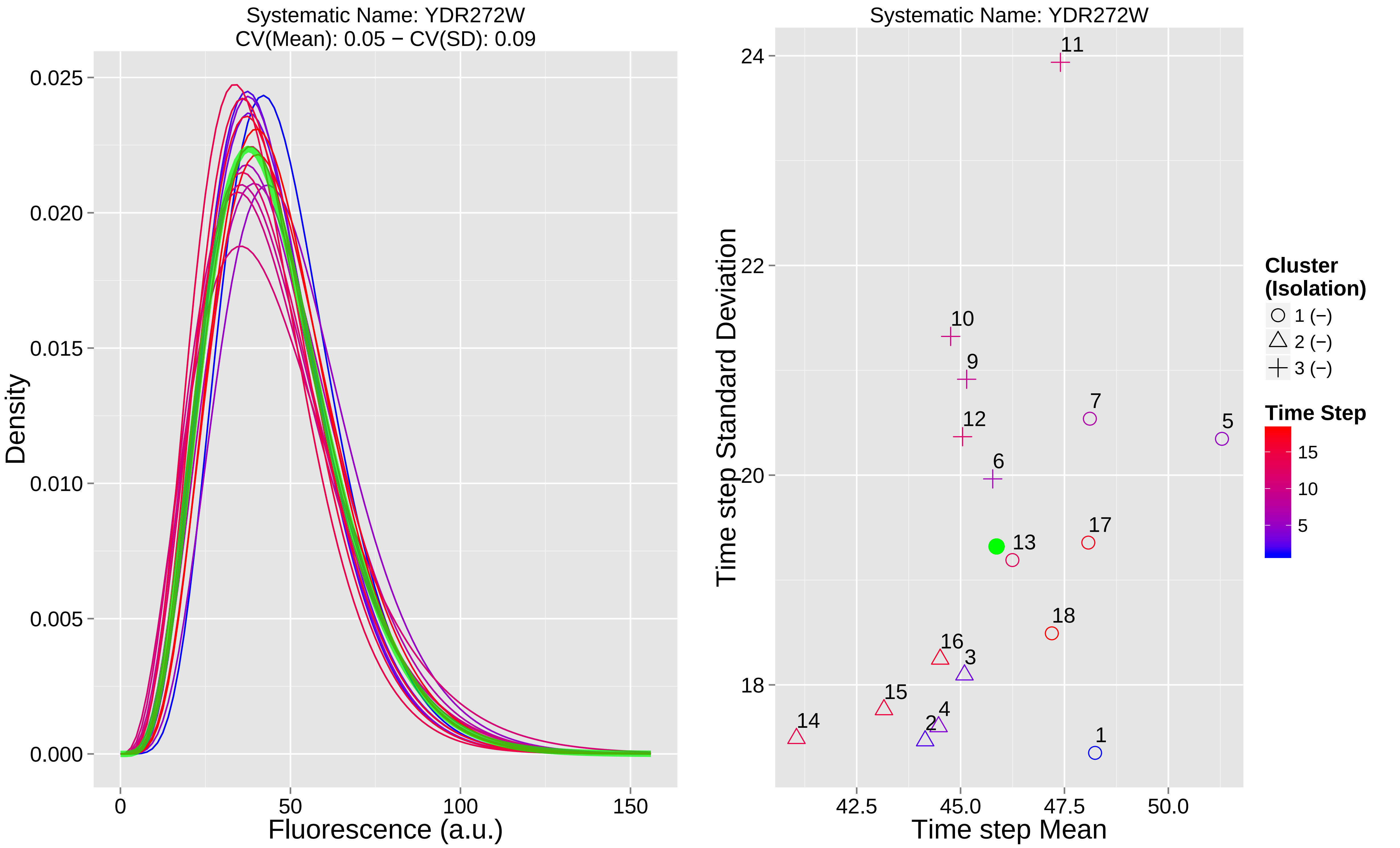

Systematic Name: YDR272W
CV(Mean): 0.05 − CV(SD): 0.09
Density
0.025
0.020
0.015
0.010
0.005
0.000
0
50
100
150
Fluorescence (a.u.)
Systematic Name: YDR272W
Time step Standard Deviation
24
22
20
18
42.5
45.0
47.5
50.0
Time step Mean
11
10
9
12
6
7
5
13
17
18
16
3
15
4
2
14
1
Cluster (Isolation)
1 (−)
2 (−)
3 (−)
Time Step
15
10
5

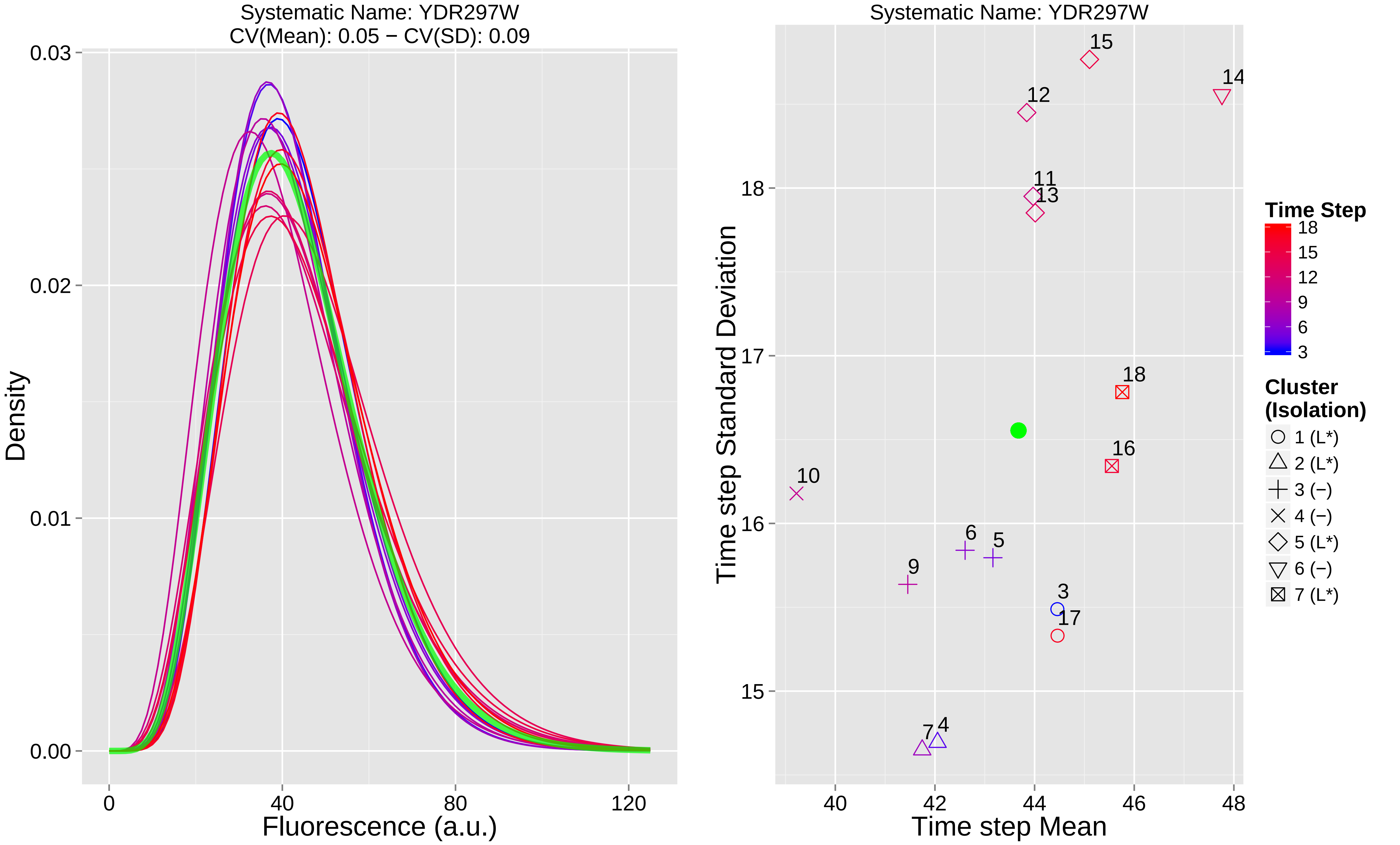

Systematic Name: YDR297W
CV(Mean): 0.05 − CV(SD): 0.09
Density
Fluorescence (a.u.)
0.03
0.02
0.01
0.00
0
40
80
120
Systematic Name: YDR297W
Time step Standard Deviation
Time step Mean
18
17
16
15
40
42
44
46
48
15
14
12
11
13
18
16
10
6
5
9
3
17
7
4
Time Step
18
15
12
9
6
3
Cluster (Isolation)
1 (L*)
2 (L*)
3 (−)
4 (−)
5 (L*)
6 (−)
7 (L*)

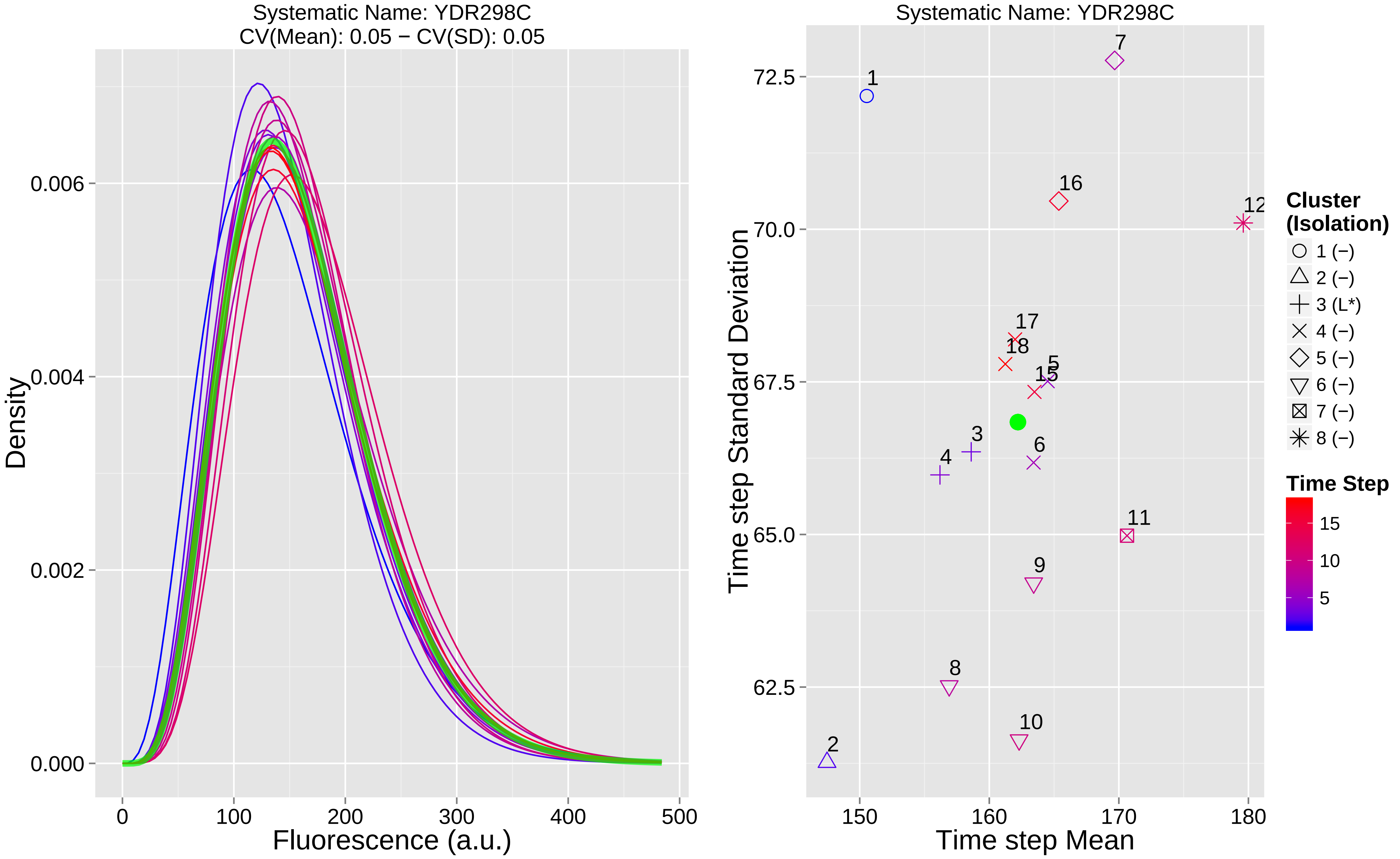
Systematic Name: YDR298C
CV(Mean): 0.05 − CV(SD): 0.05
Density
Fluorescence (a.u.)
0.000
0.002
0.004
0.006
0
100
200
300
400
500
Systematic Name: YDR298C
Time step Standard Deviation
Time step Mean
62.5
65.0
67.5
70.0
72.5
150
160
170
180
Cluster
(Isolation)
1 (−)
2 (−)
3 (L*)
4 (−)
5 (−)
6 (−)
7 (−)
8 (−)
Time Step
15
10
5

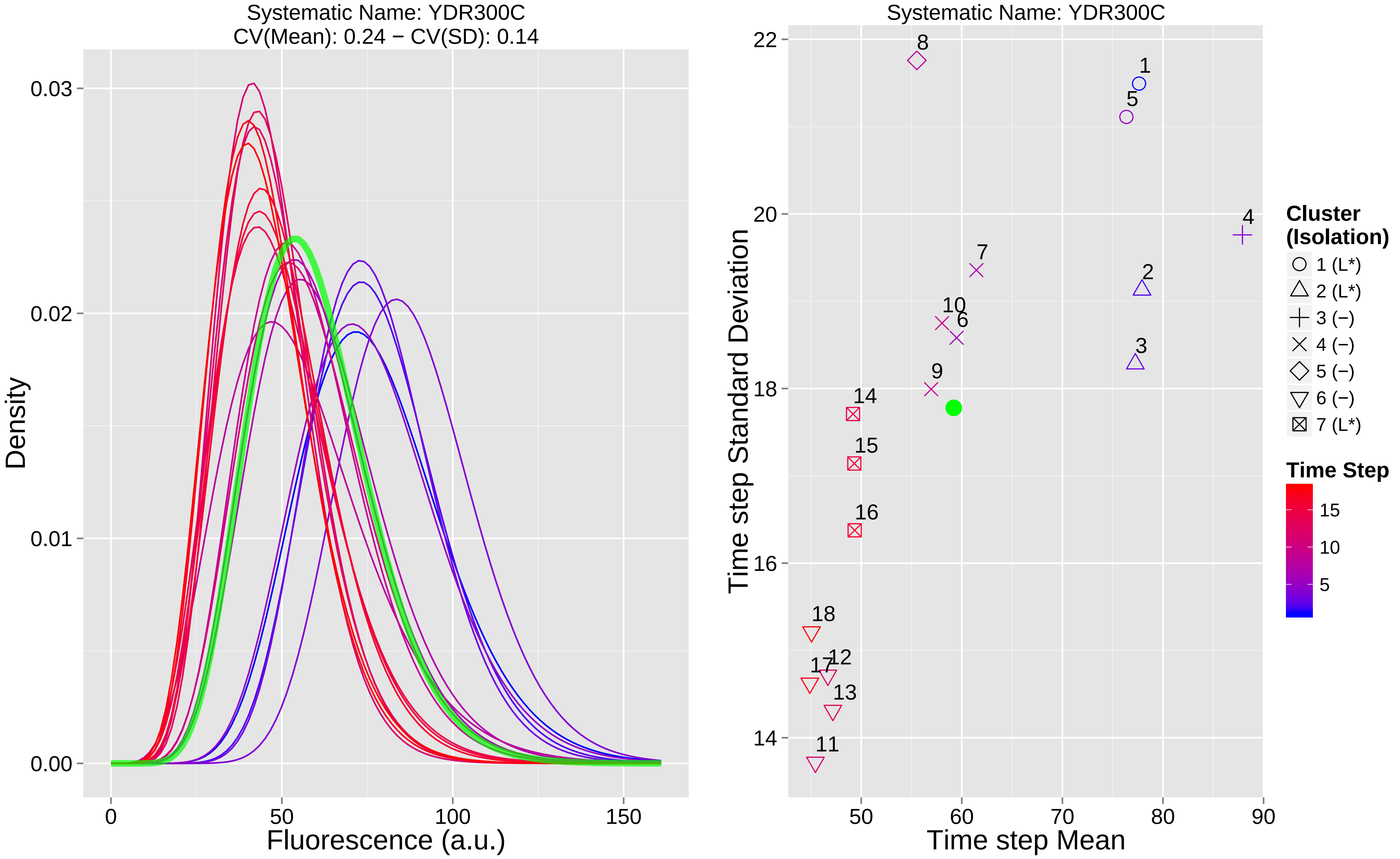

Systematic Name: YDR300C
CV(Mean): 0.24 − CV(SD): 0.14
Density
0.03
0.02
0.01
0.00
0
50
100
150
Fluorescence (a.u.)
Systematic Name: YDR300C
Time step Standard Deviation
22
20
18
16
14
50
60
70
80
90
Time step Mean
Cluster (Isolation)
1 (L*)
2 (L*)
3 (−)
4 (−)
5 (−)
6 (−)
7 (L*)
Time Step
15
10
5

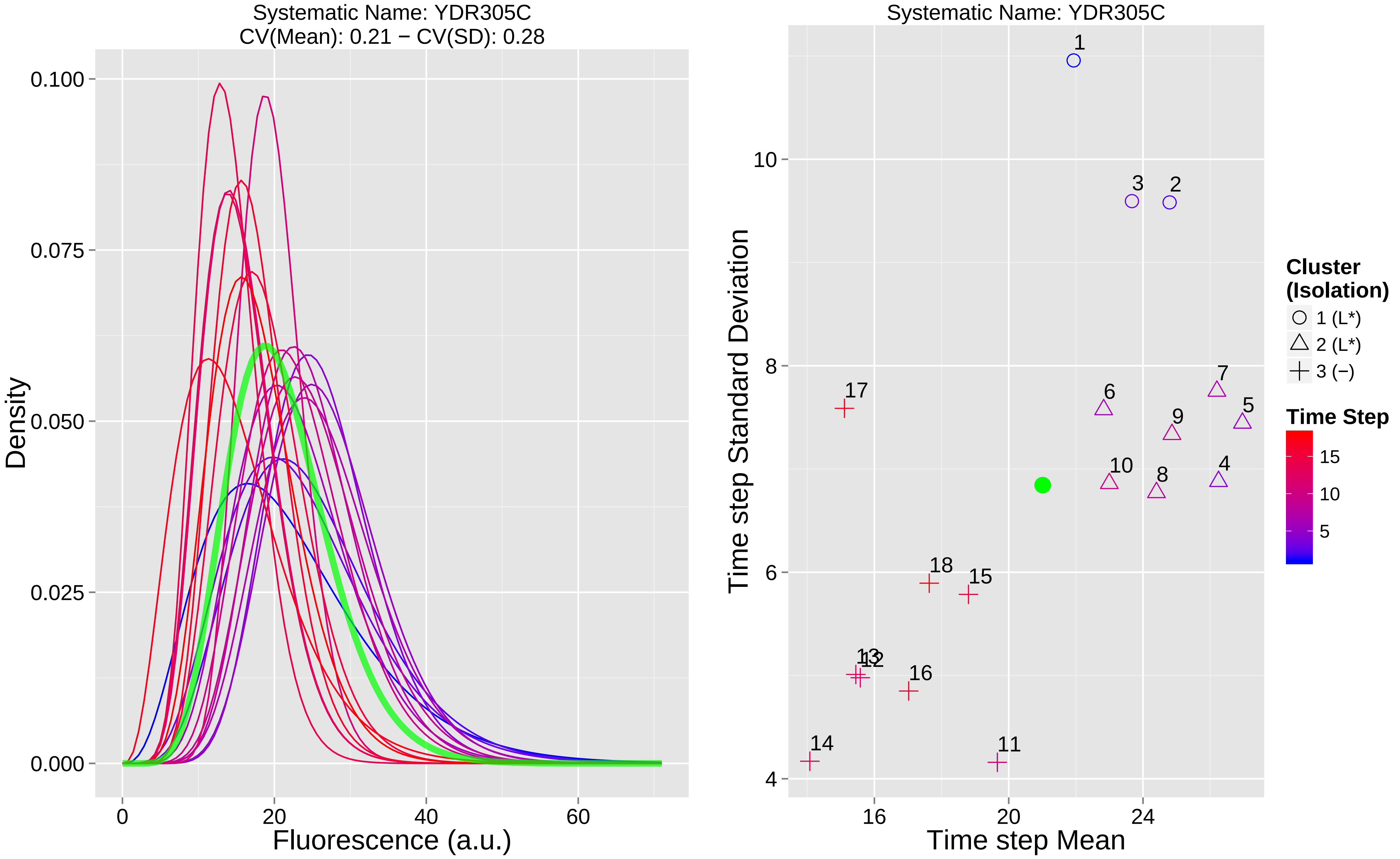

Systematic Name: YDR305C
CV(Mean): 0.21 − CV(SD): 0.28
Density
0.100
0.075
0.050
0.025
0.000
0
20
40
60
Fluorescence (a.u.)
Systematic Name: YDR305C
Time step Standard Deviation
10
8
6
4
16
20
24
Time step Mean
1
2
3
4
5
6
7
8
9
10
11
12
13
14
15
16
17
18
Cluster (Isolation)
1 (L*)
2 (L*)
3 (−)
Time Step
15
10
5

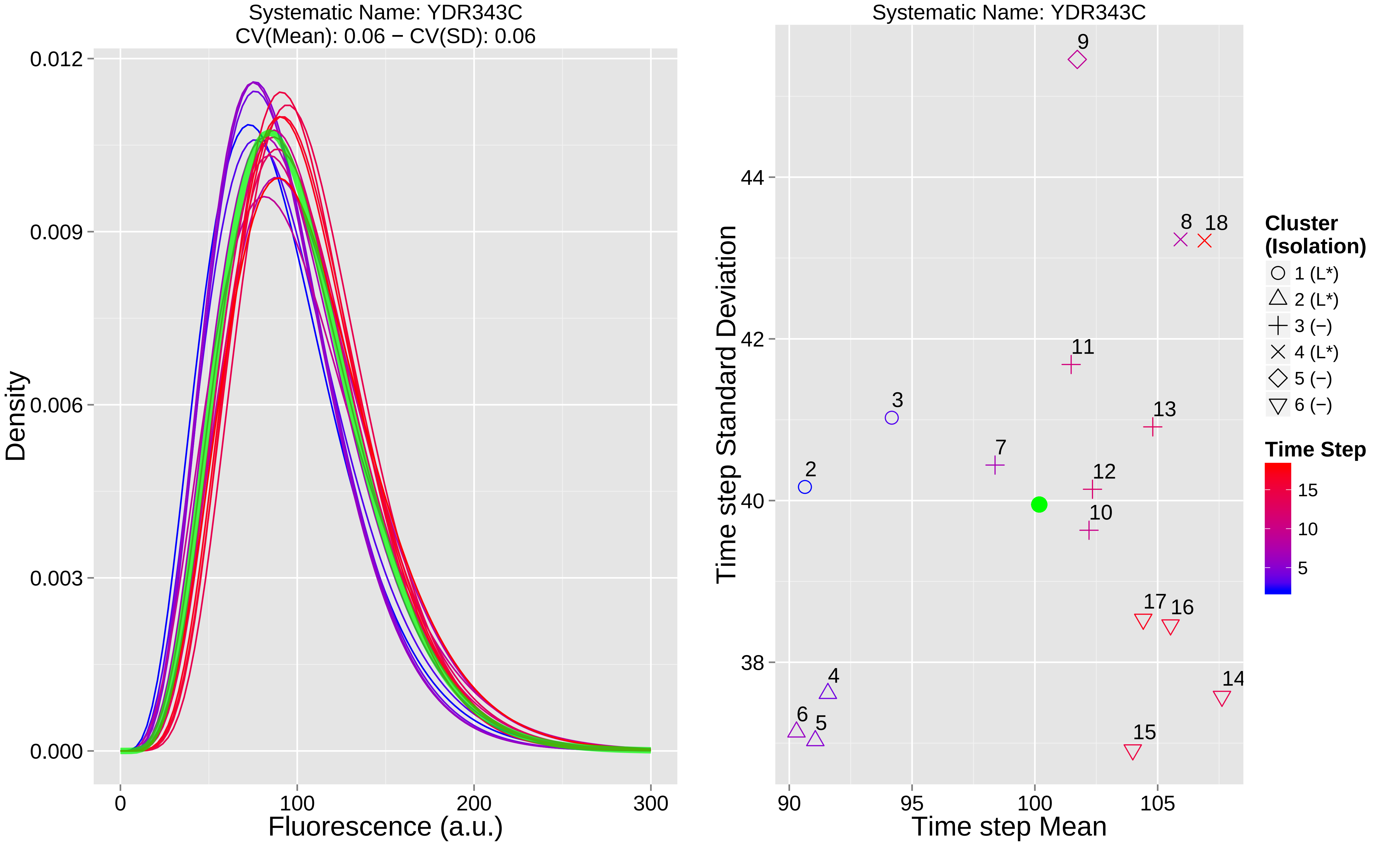
Systematic Name: YDR343C
CV(Mean): 0.06 − CV(SD): 0.06
Density
Fluorescence (a.u.)
0.012
0.009
0.006
0.003
0.000
0
100
200
300
Systematic Name: YDR343C
Time step Standard Deviation
Time step Mean
44
42
40
38
90
95
100
105
9
8
18
11
3
13
7
2
12
10
17
16
4
14
6
5
15
Cluster (Isolation)
1 (L*)
2 (L*)
3 (−)
4 (L*)
5 (−)
6 (−)
Time Step
15
10
5

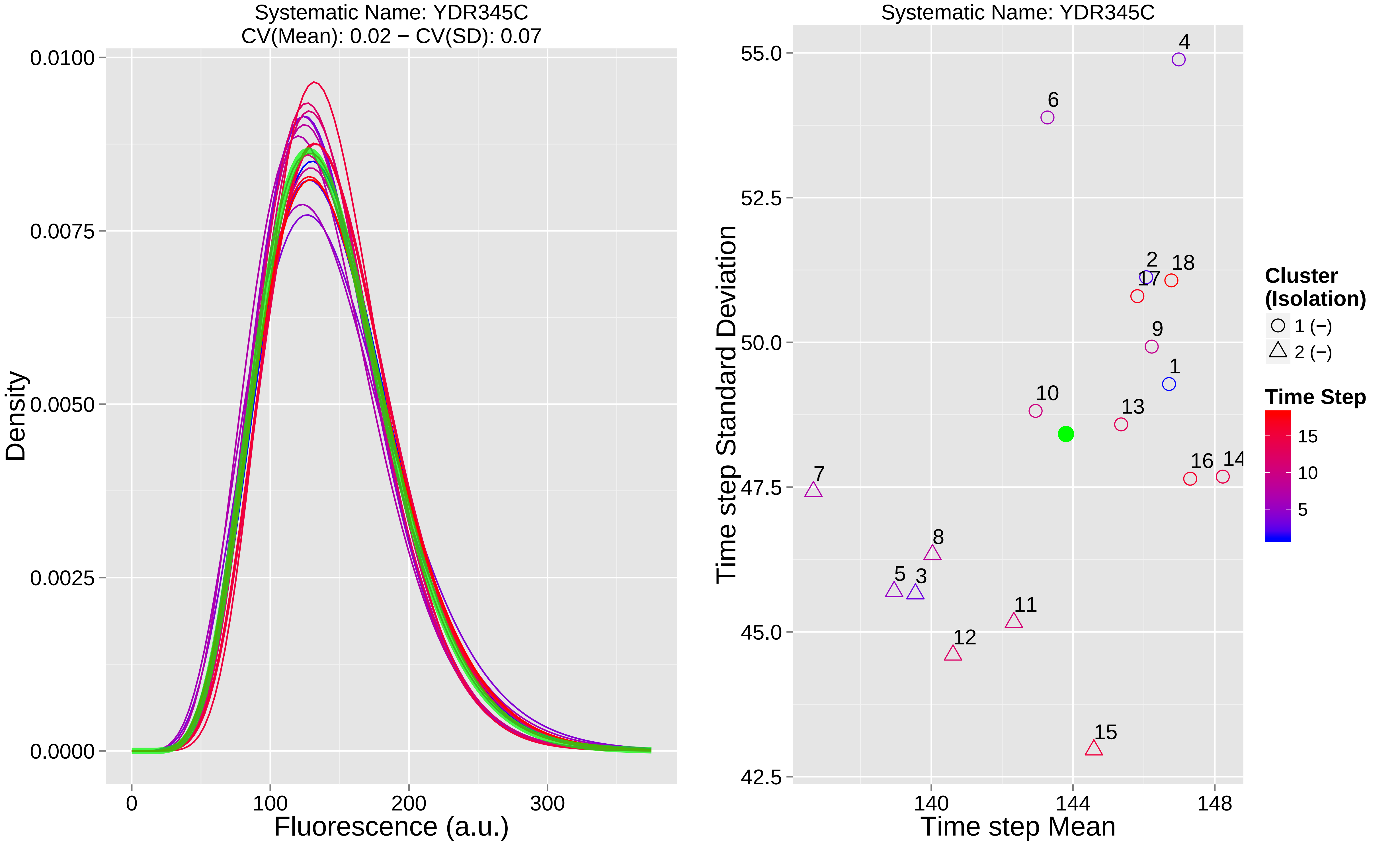
Systematic Name: YDR345C
CV(Mean): 0.02 − CV(SD): 0.07
0.0100
0.0075
0.0050
0.0025
0.0000
Density
0
100
200
300
Fluorescence (a.u.)
Systematic Name: YDR345C
55.0
52.5
50.0
47.5
45.0
42.5
Time step Standard Deviation
140
144
148
Time step Mean
4
6
2
18
17
9
1
10
13
16
14
7
8
5
3
11
12
15
Cluster (Isolation)
1 (−)
2 (−)
Time Step
15
10
5

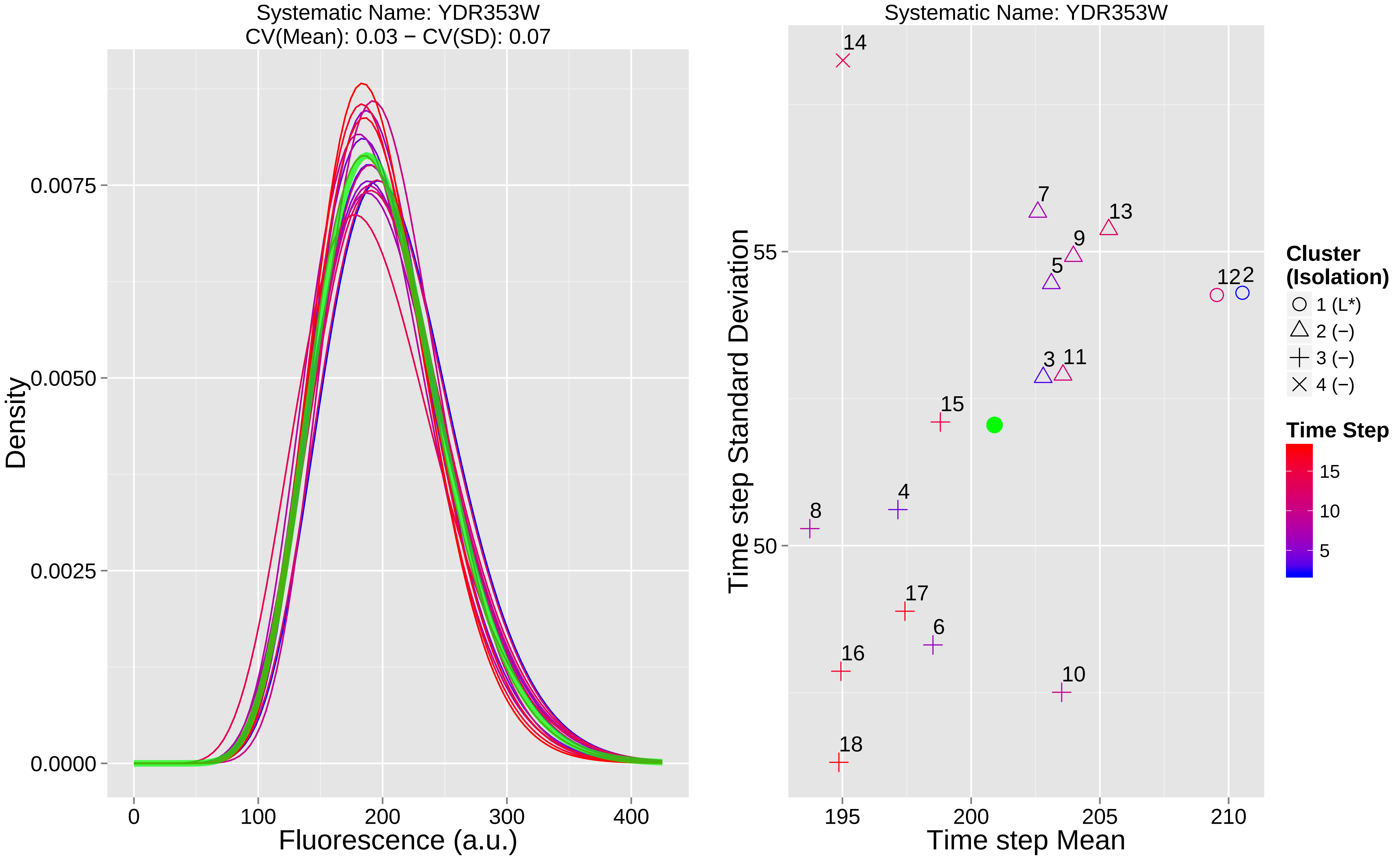

Systematic Name: YDR353W
CV(Mean): 0.03 − CV(SD): 0.07
Density
0.0075
0.0050
0.0025
0.0000
0
100
200
300
400
Fluorescence (a.u.)
Systematic Name: YDR353W
Time step Standard Deviation
55
50
195
200
205
210
Time step Mean
14
7
13
9
5
12
2
3
11
15
4
8
17
6
16
10
18
Cluster (Isolation)
1 (L*)
2 (−)
3 (−)
4 (−)
Time Step
15
10
5

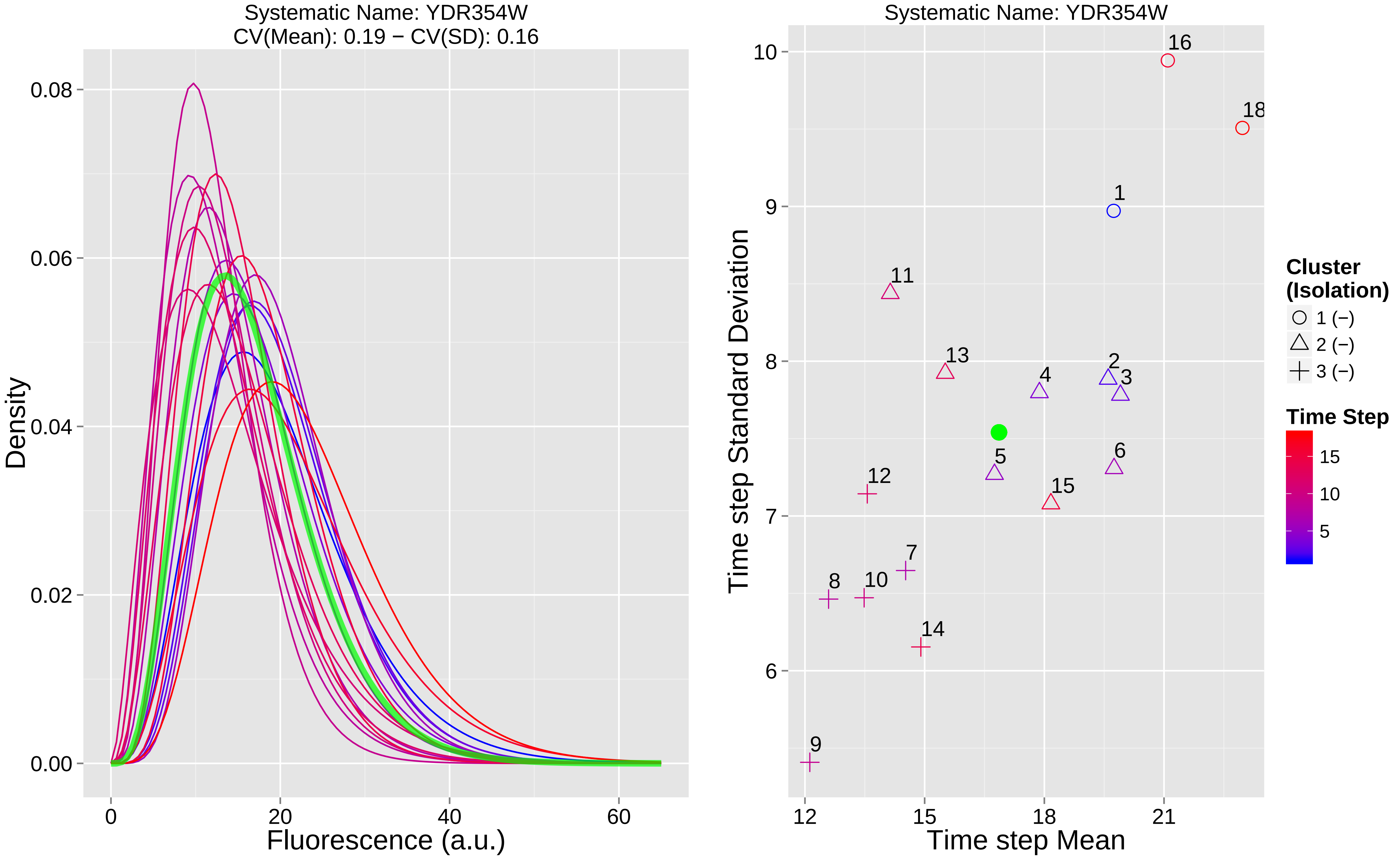

Systematic Name: YDR354W
CV(Mean): 0.19 − CV(SD): 0.16
Density
Fluorescence (a.u.)
0.00
0.02
0.04
0.06
0.08
0
20
40
60
Systematic Name: YDR354W
Time step Standard Deviation
Time step Mean
6
7
8
9
10
12
15
18
21
1
2
3
4
5
6
7
8
9
10
11
12
13
14
15
16
18
Cluster
(Isolation)
1 (−)
2 (−)
3 (−)
Time Step
15
10
5

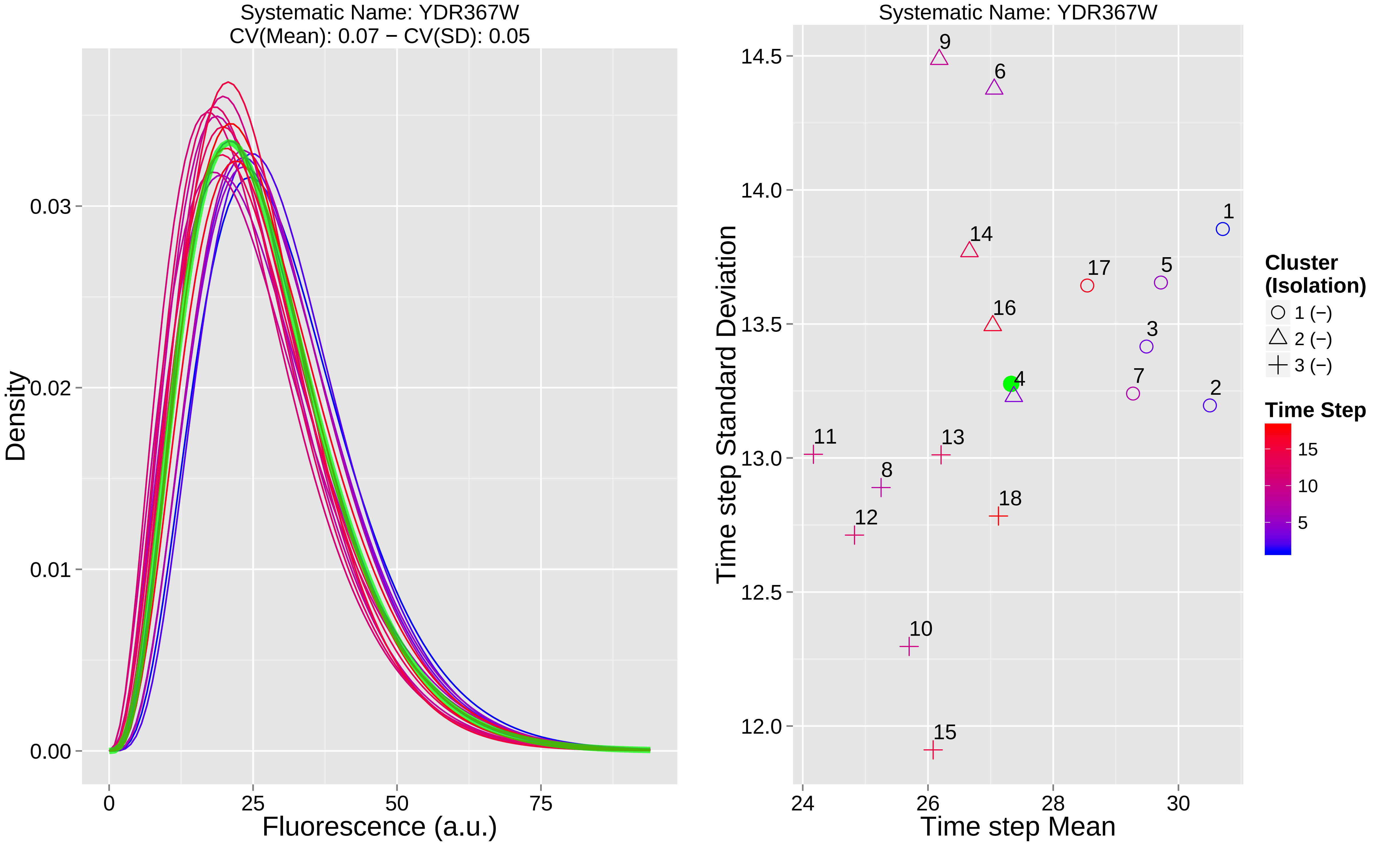
Systematic Name: YDR367W
CV(Mean): 0.07 − CV(SD): 0.05
Density
Fluorescence (a.u.)
0.00
0.01
0.02
0.03
0
25
50
75
Systematic Name: YDR367W
Time step Standard Deviation
Time step Mean
12.0
12.5
13.0
13.5
14.0
14.5
24
26
28
30
1
2
3
4
5
6
7
8
9
10
11
12
13
14
15
16
17
18
Cluster (Isolation)
1 (−)
2 (−)
3 (−)
Time Step
15
10
5

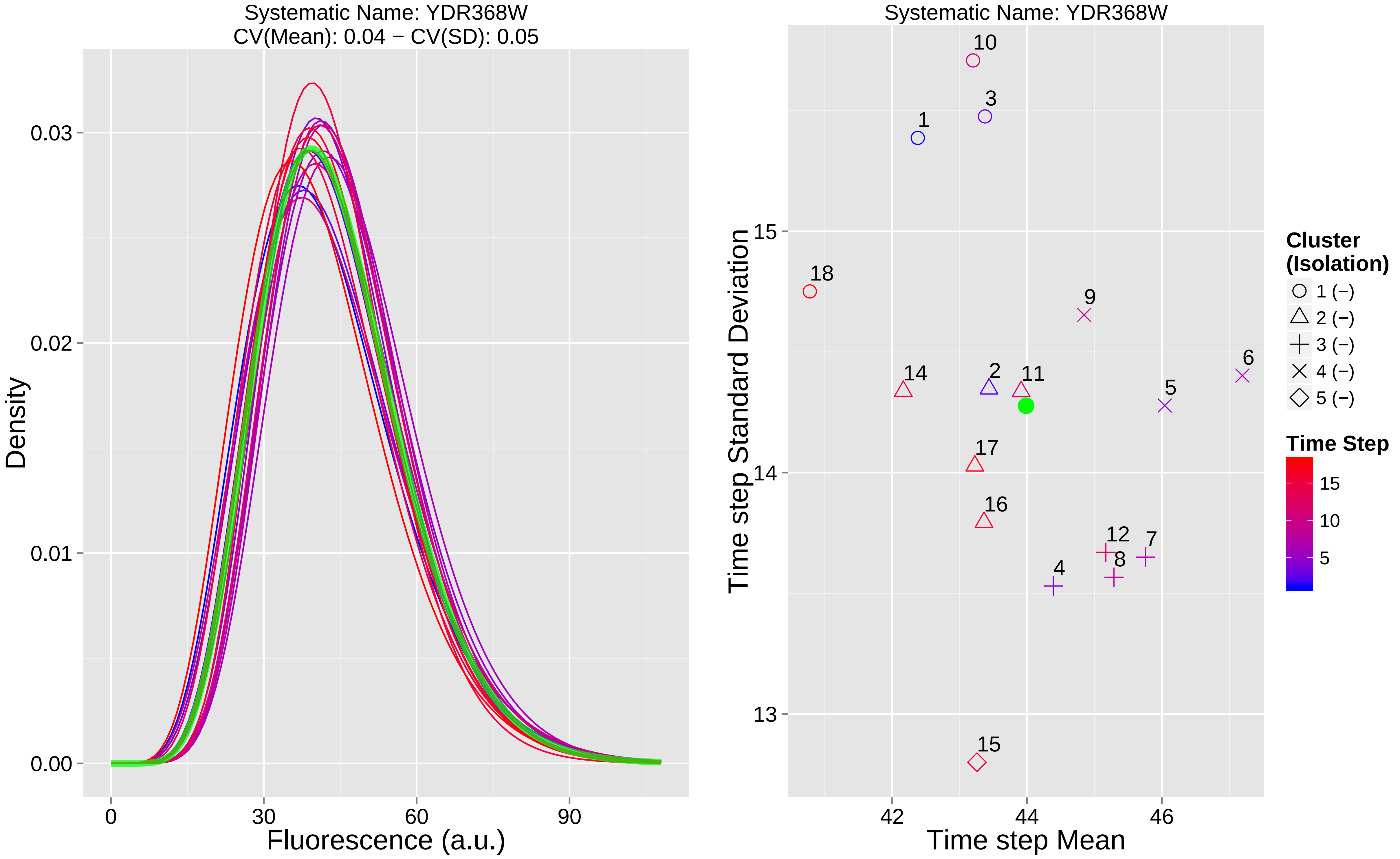

Systematic Name: YDR368W
CV(Mean): 0.04 − CV(SD): 0.05
Density
Fluorescence (a.u.)
0.00
0.01
0.02
0.03
0
30
60
90
Systematic Name: YDR368W
Time step Standard Deviation
Time step Mean
13
14
15
42
44
46
1
2
3
4
5
6
7
8
9
10
11
12
14
15
16
17
18
Cluster (Isolation)
1 (−)
2 (−)
3 (−)
4 (−)
5 (−)
Time Step
15
10
5

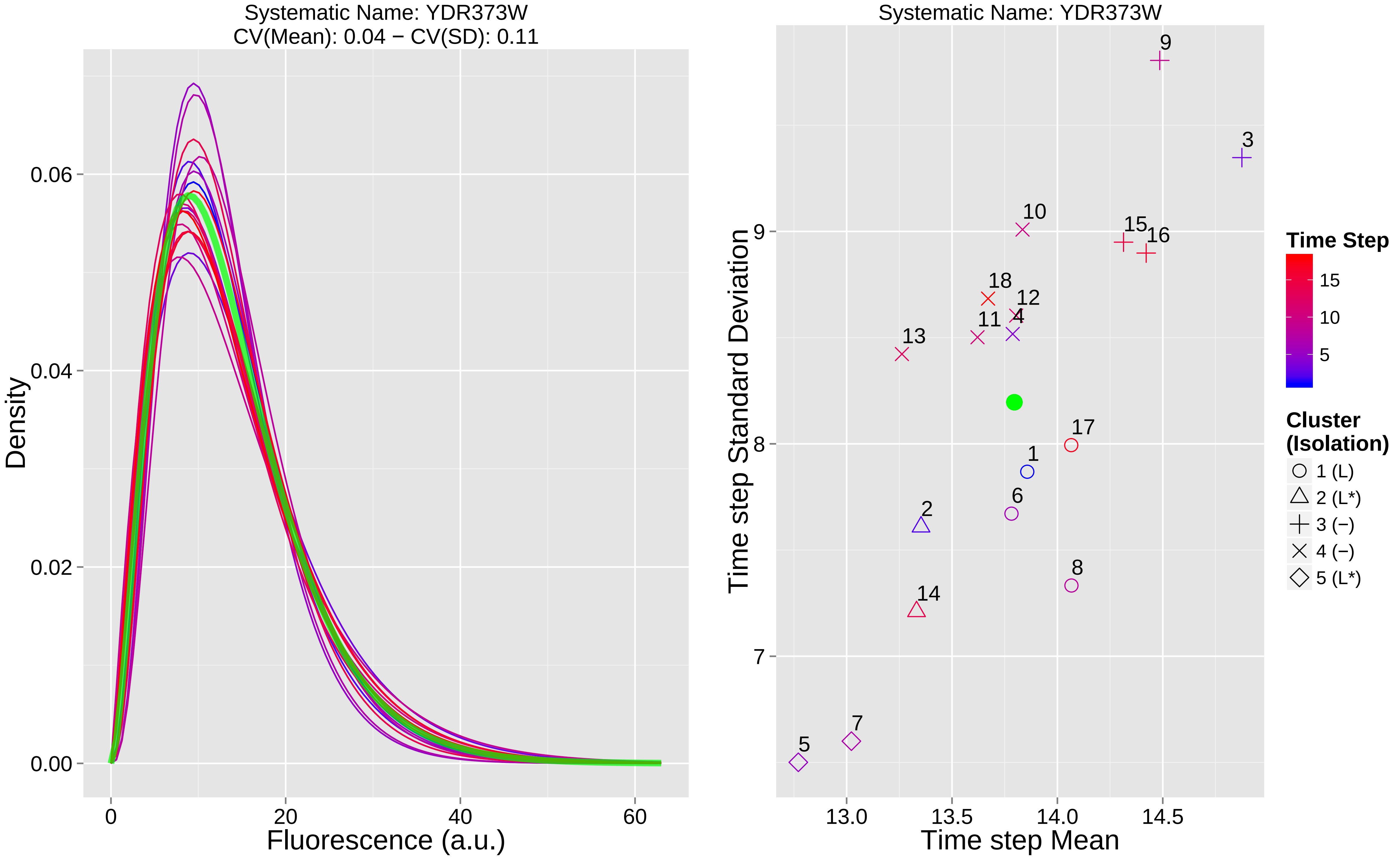
Systematic Name: YDR373W
CV(Mean): 0.04 − CV(SD): 0.11
Density
0.00
0.02
0.04
0.06
0
20
40
60
Fluorescence (a.u.)
Systematic Name: YDR373W
Time step Standard Deviation
7
8
9
13.0
13.5
14.0
14.5
Time step Mean
1
2
3
4
5
6
7
8
9
10
11
12
13
14
15
16
17
18
Time Step
15
10
5
Cluster
(Isolation)
1 (L)
2 (L*)
3 (−)
4 (−)
5 (L*)

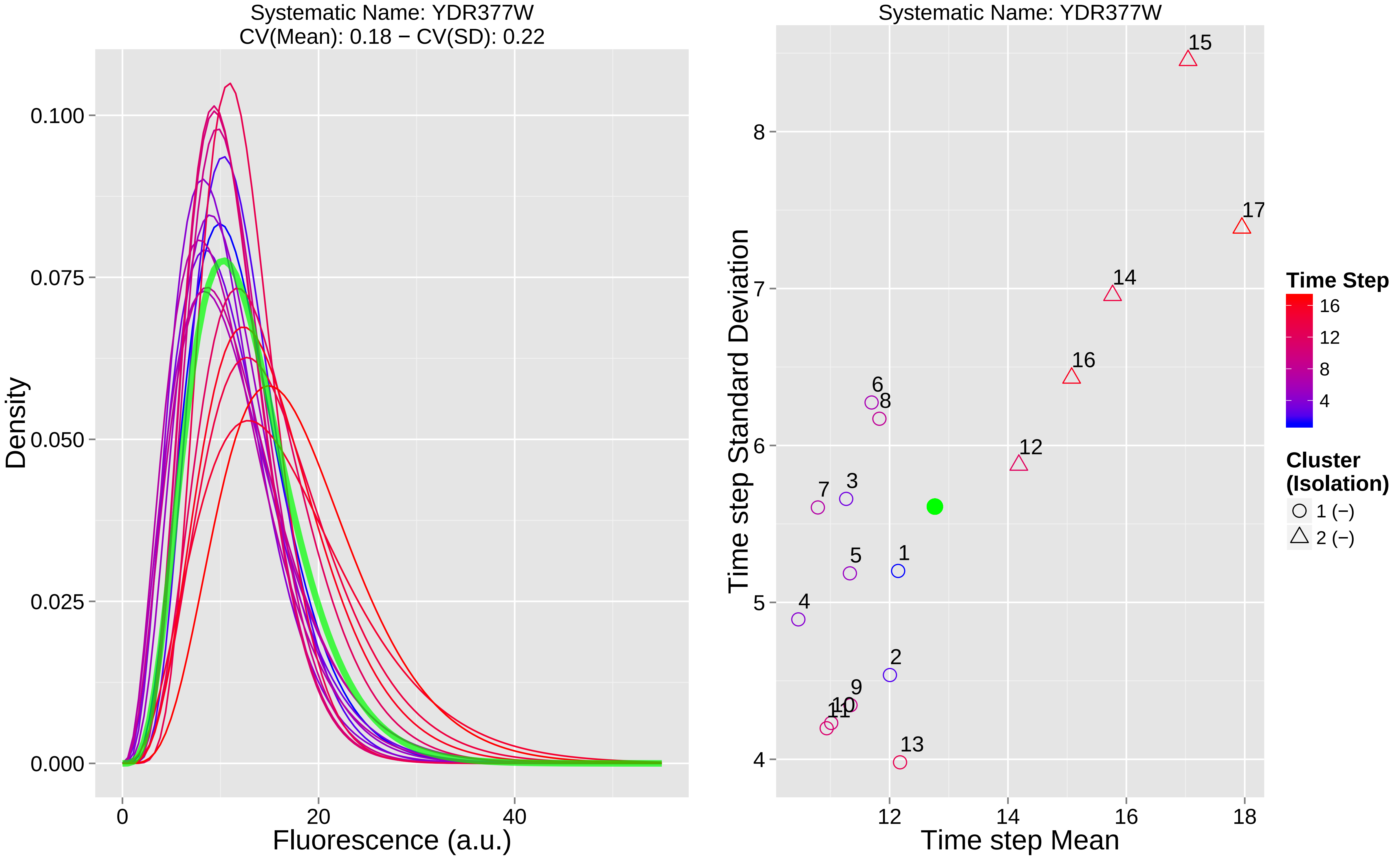

Systematic Name: YDR377W
CV(Mean): 0.18 − CV(SD): 0.22
Density
0.100
0.075
0.050
0.025
0.000
0
20
40
Fluorescence (a.u.)
Systematic Name: YDR377W
Time step Standard Deviation
8
7
6
5
4
12
14
16
18
Time step Mean
15
17
14
16
6
8
12
7
3
1
5
4
2
9
10
11
13
Time Step
16
12
8
4
Cluster
(Isolation)
1 (−)
2 (−)

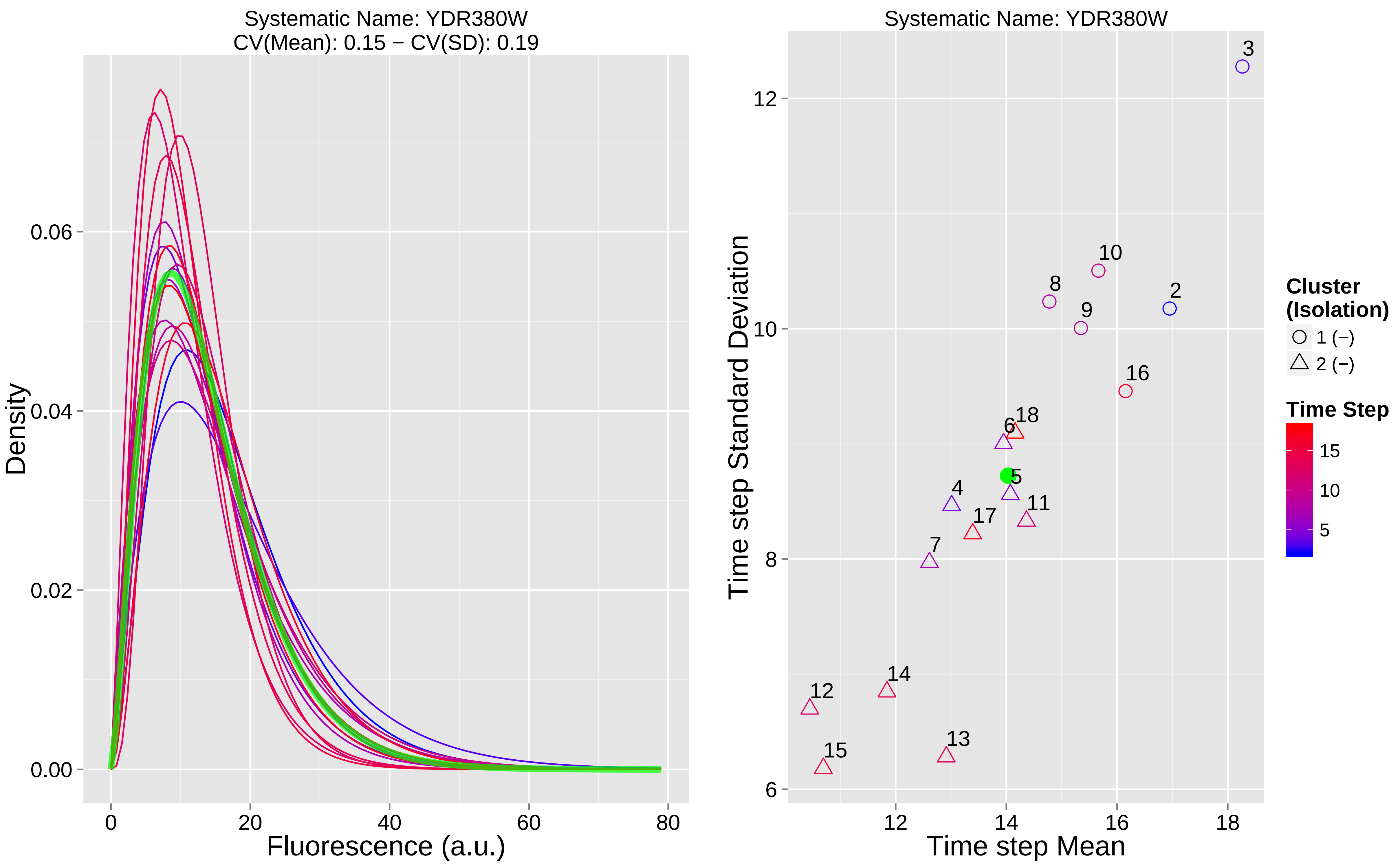

Systematic Name: YDR380W
CV(Mean): 0.15 − CV(SD): 0.19
Density
0.00
0.02
0.04
0.06
0
20
40
60
80
Fluorescence (a.u.)
Systematic Name: YDR380W
Time step Standard Deviation
6
8
10
12
12
14
16
18
Time step Mean
3
10
8
2
9
16
6
18
5
4
11
17
7
14
12
13
15
Cluster
(Isolation)
1 (−)
2 (−)
Time Step
15
10
5

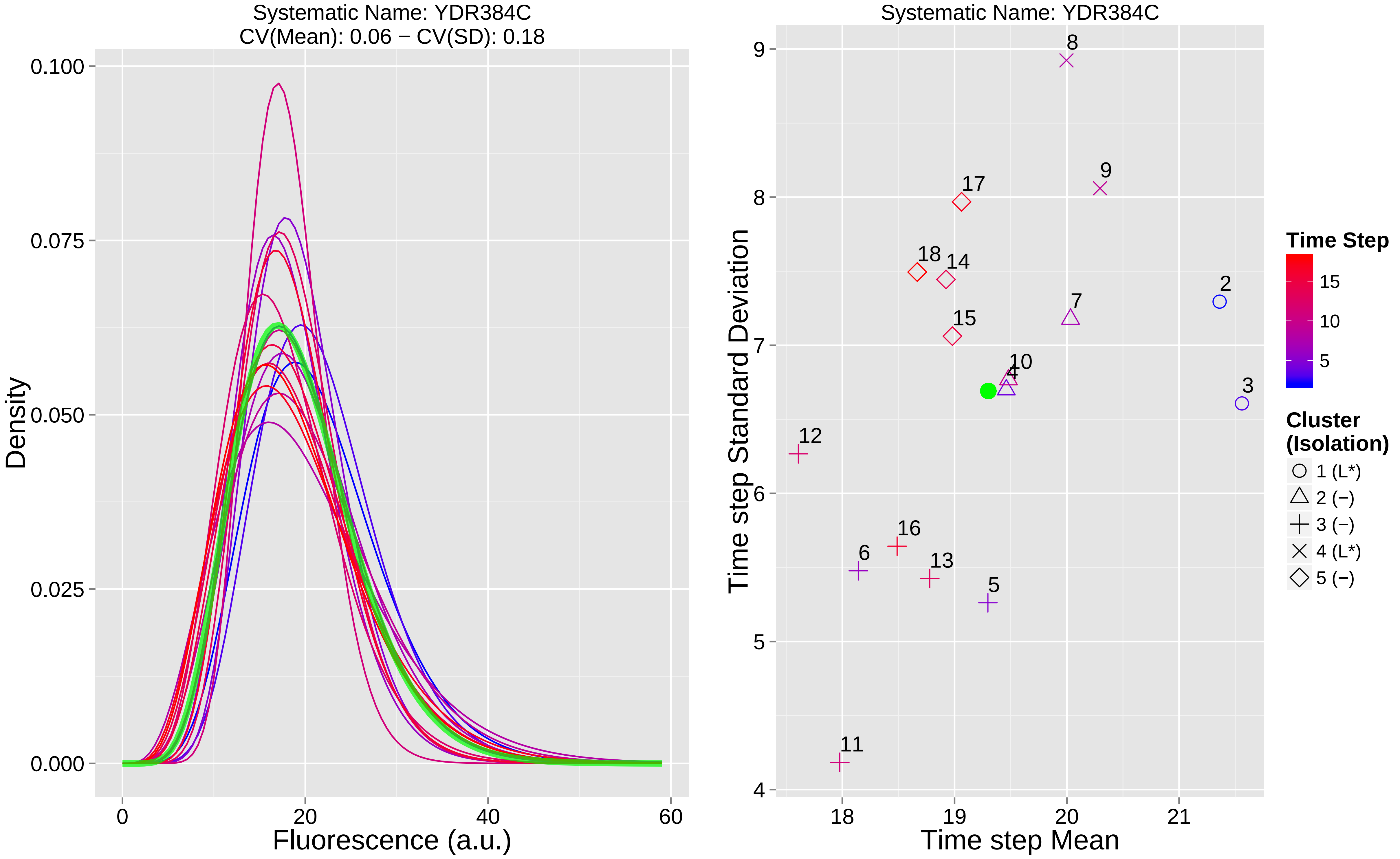

Systematic Name: YDR384C
CV(Mean): 0.06 − CV(SD): 0.18
Density
0.100
0.075
0.050
0.025
0.000
0
20
40
60
Fluorescence (a.u.)
Systematic Name: YDR384C
Time step Standard Deviation
9
8
7
6
5
4
18
19
20
21
Time step Mean
8
9
17
18
14
2
7
15
10
4
3
12
16
6
13
5
11
Time Step
15
10
5
Cluster
(Isolation)
1 (L*)
2 (−)
3 (−)
4 (L*)
5 (−)

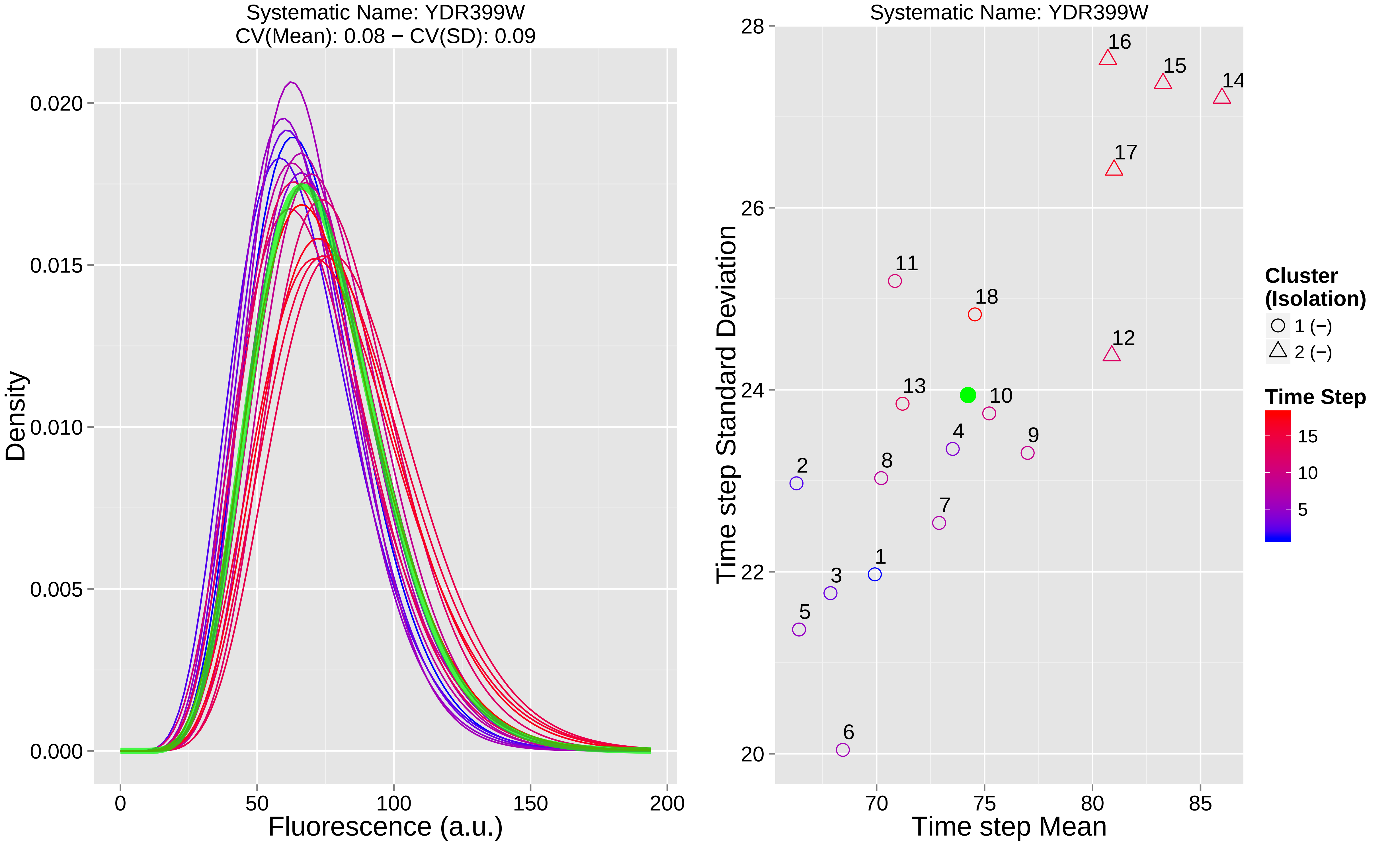

Systematic Name: YDR399W
CV(Mean): 0.08 − CV(SD): 0.09
Density
Fluorescence (a.u.)
0.000
0.005
0.010
0.015
0.020
0
50
100
150
200
Systematic Name: YDR399W
Time step Standard Deviation
Time step Mean
20
22
24
26
28
70
75
80
85
Cluster
(Isolation)
1 (−)
2 (−)
Time Step
5
10
15
1
2
3
4
5
6
7
8
9
10
11
12
13
14
15
16
17
18

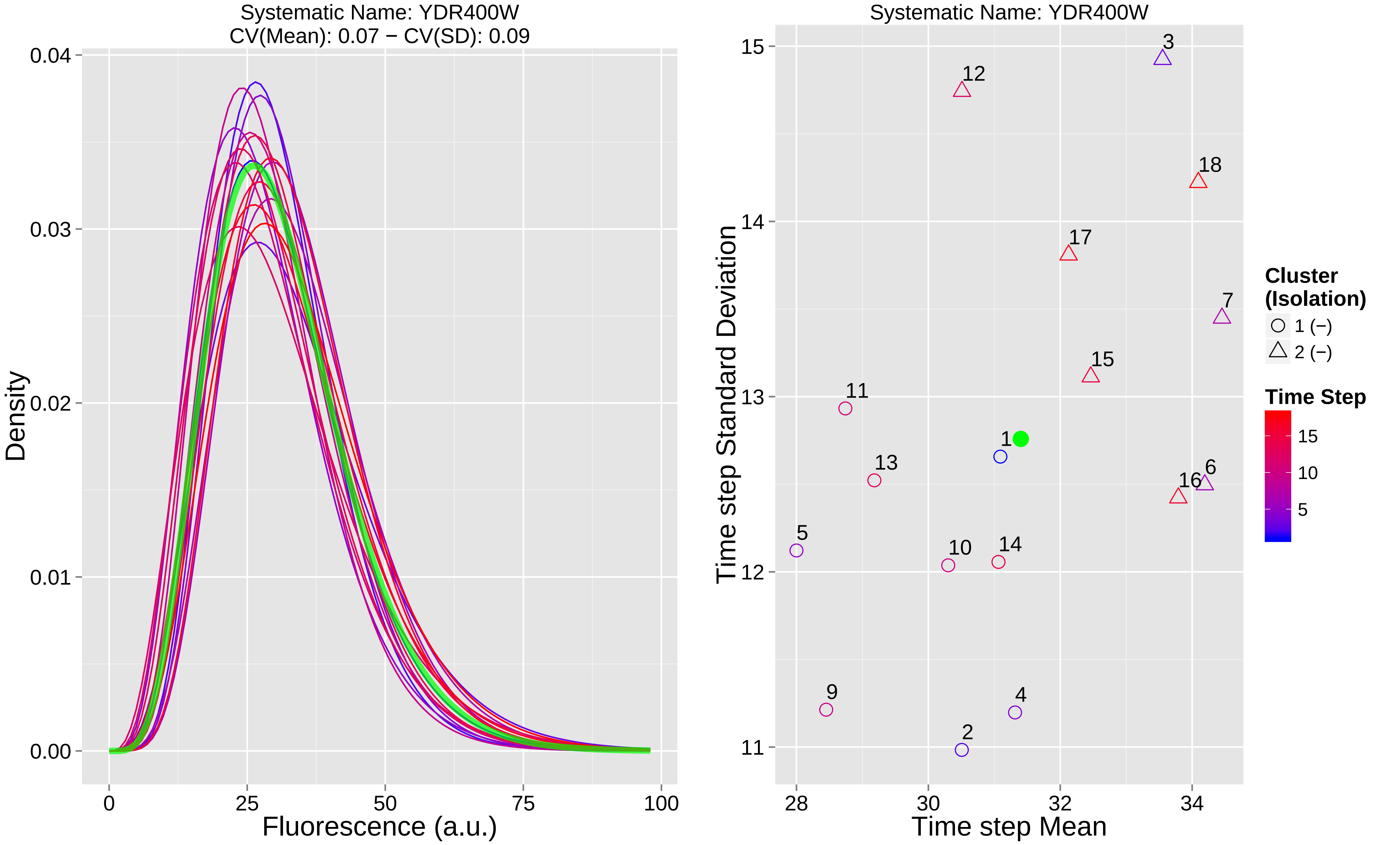

Systematic Name: YDR400W
CV(Mean): 0.07 − CV(SD): 0.09
Density
Fluorescence (a.u.)
0.00
0.01
0.02
0.03
0.04
0
25
50
75
100
Systematic Name: YDR400W
Time step Standard Deviation
Time step Mean
11
12
13
14
15
28
30
32
34
1
2
3
4
5
6
7
9
10
11
12
13
14
15
16
17
18
Cluster (Isolation)
1 (−)
2 (−)
Time Step
15
10
5

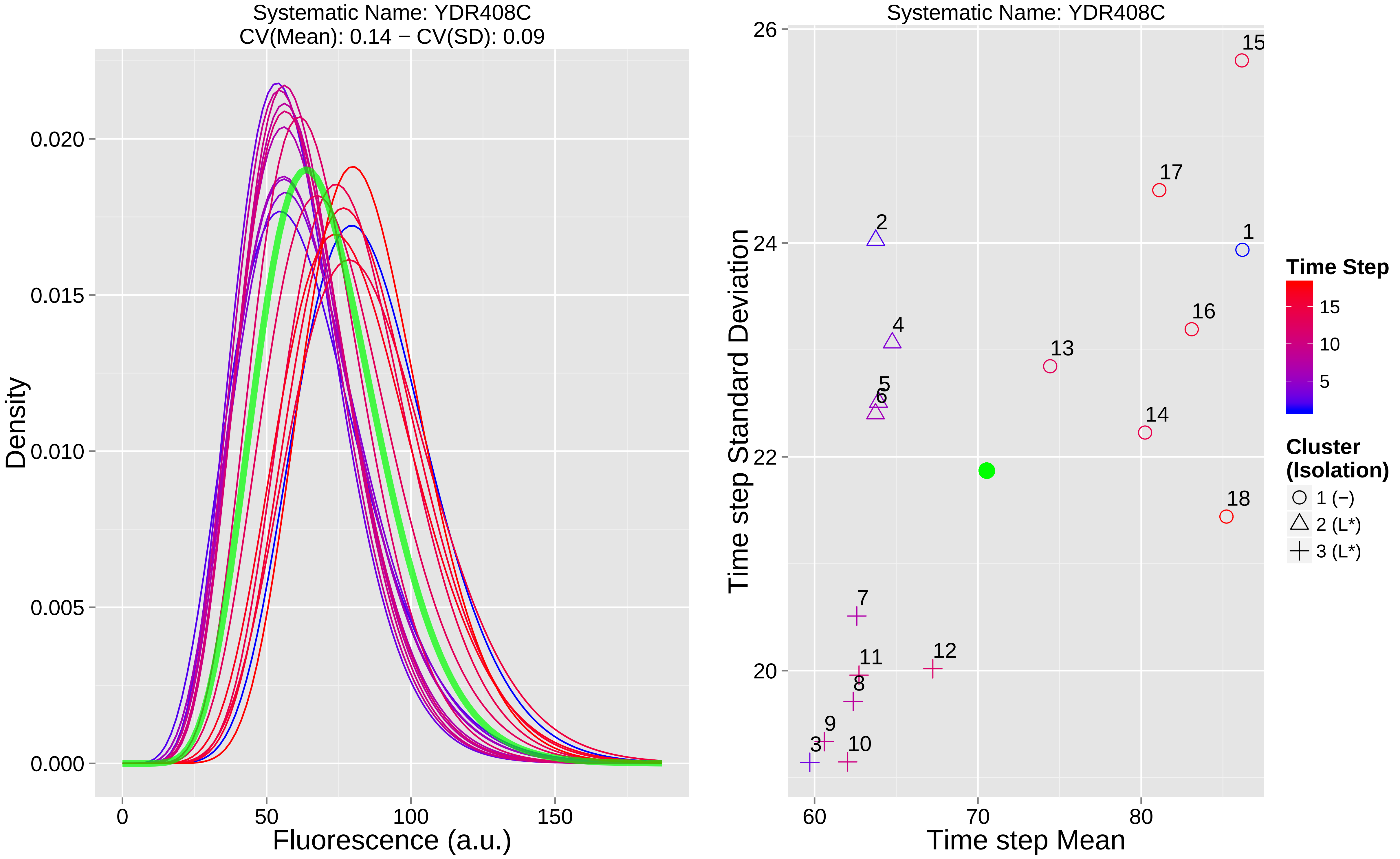

Systematic Name: YDR408C
CV(Mean): 0.14 − CV(SD): 0.09
Density
0.000
0.005
0.010
0.015
0.020
0
50
100
150
Fluorescence (a.u.)
Systematic Name: YDR408C
Time step Standard Deviation
20
22
24
26
60
70
80
Time step Mean
1
2
3
4
5
6
7
8
9
10
11
12
13
14
15
16
17
18
Time Step
5
10
15
Cluster
(Isolation)
1 (−)
2 (L*)
3 (L*)

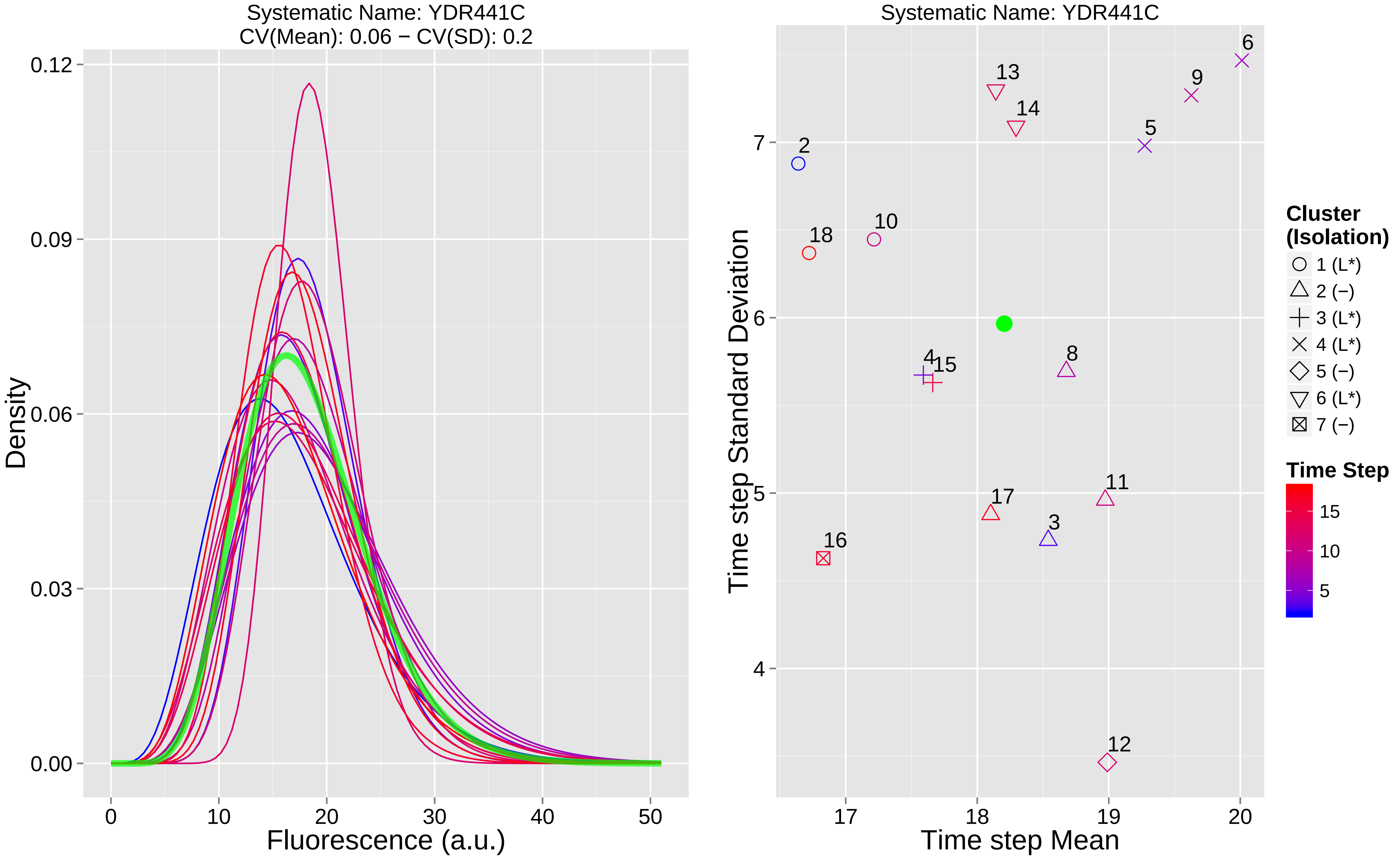
Systematic Name: YDR441C
CV(Mean): 0.06 − CV(SD): 0.2
Density
Fluorescence (a.u.)
0.00
0.03
0.06
0.09
0.12
0
10
20
30
40
50
Systematic Name: YDR441C
Time step Standard Deviation
Time step Mean
4
5
6
7
17
18
19
20
2
18
10
13
14
6
9
5
4
15
8
17
3
11
16
12
Cluster (Isolation)
1 (L*)
2 (−)
3 (L*)
4 (L*)
5 (−)
6 (L*)
7 (−)
Time Step
15
10
5

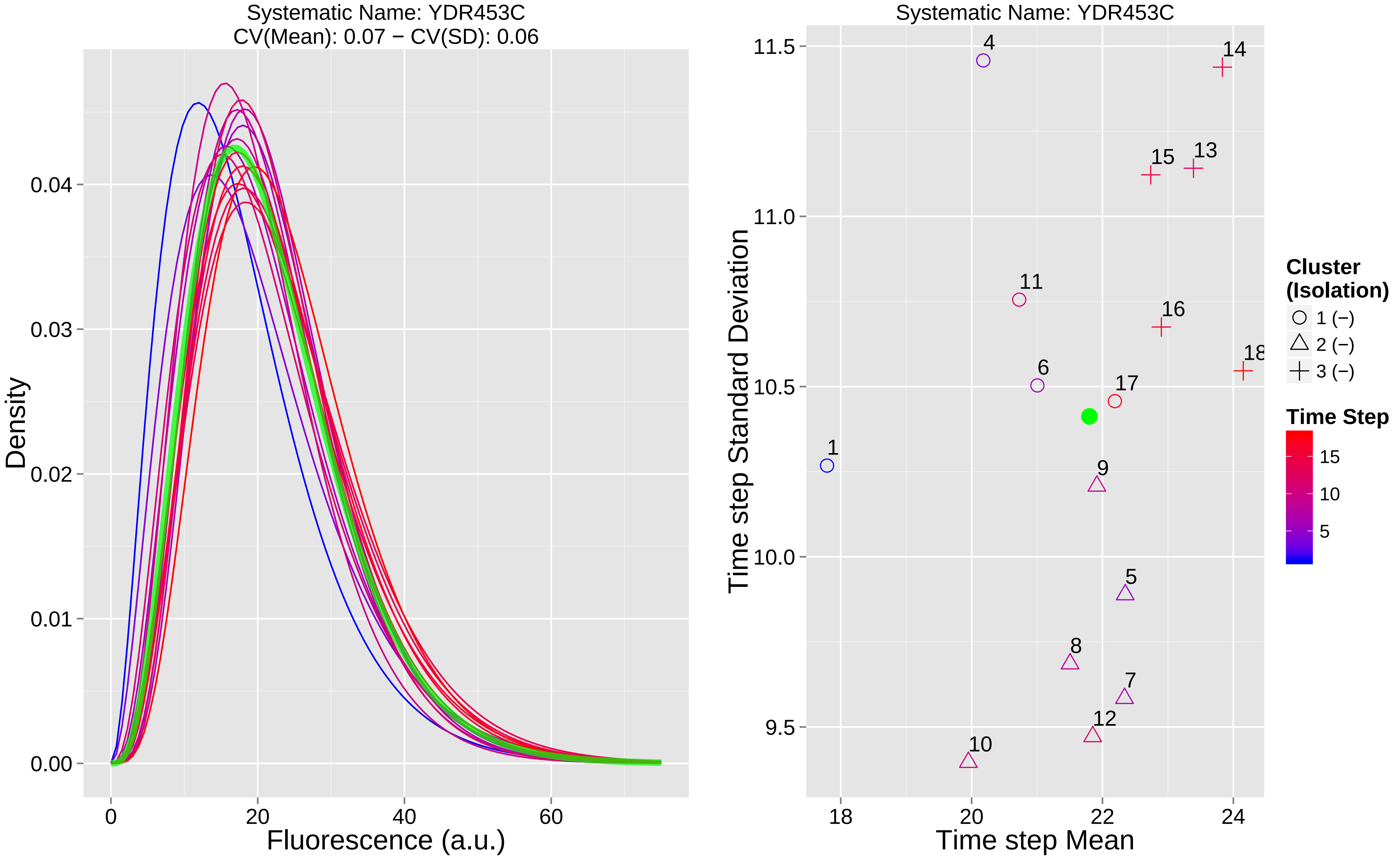
Systematic Name: YDR453C
CV(Mean): 0.07 − CV(SD): 0.06
Density
0.00
0.01
0.02
0.03
0.04
0
20
40
60
Fluorescence (a.u.)
Systematic Name: YDR453C
Time step Standard Deviation
9.5
10.0
10.5
11.0
11.5
18
20
22
24
Time step Mean
1
4
6
11
17
9
5
8
7
12
10
14
15
13
16
18
Cluster (Isolation)
1 (−)
2 (−)
3 (−)
Time Step
15
10
5

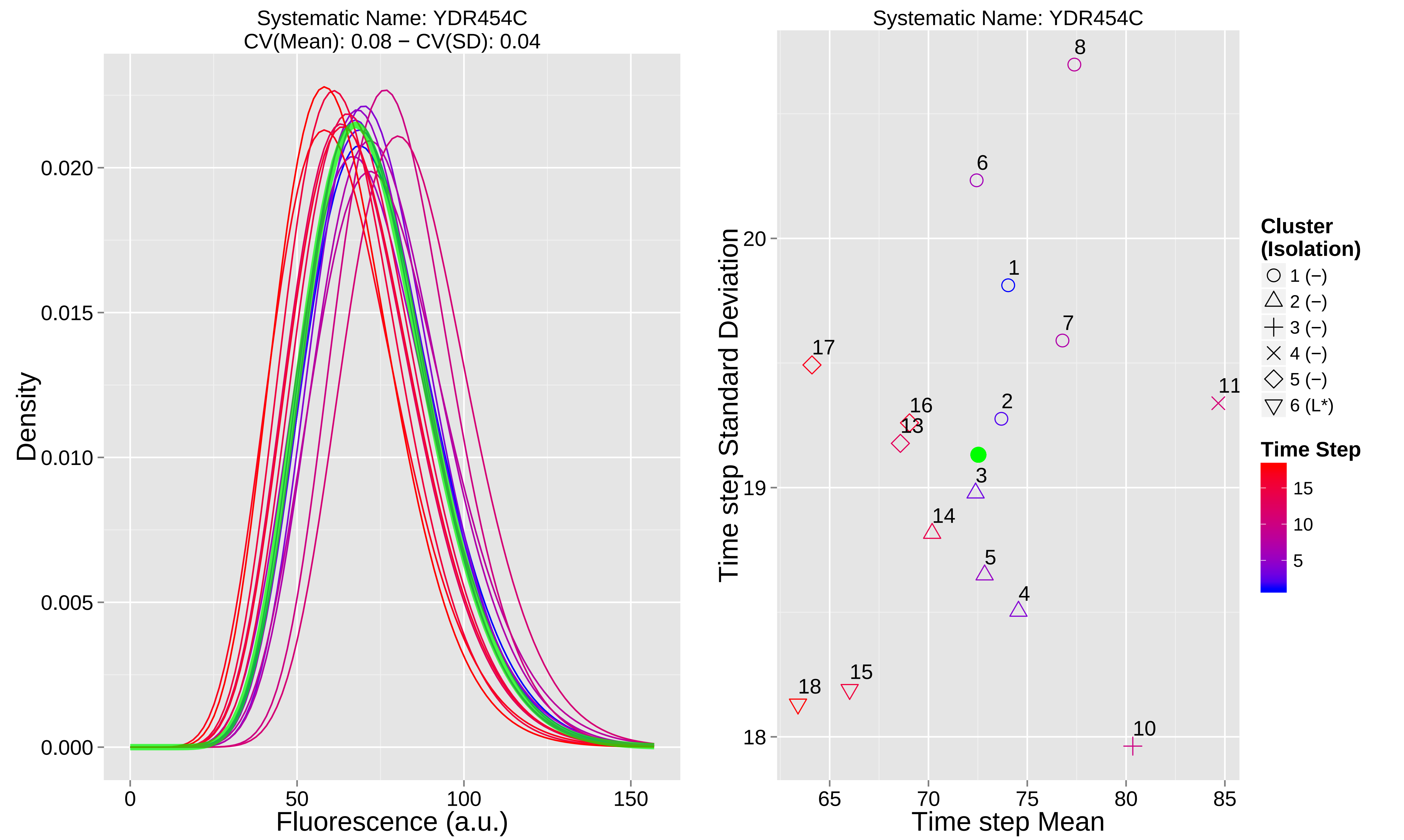

Systematic Name: YDR454C
CV(Mean): 0.08 − CV(SD): 0.04
Density
0.020
0.015
0.010
0.005
0.000
0
50
100
150
Fluorescence (a.u.)
Systematic Name: YDR454C
Time step Standard Deviation
20
19
18
65
70
75
80
85
Time step Mean
8
6
1
7
17
11
16
13
2
3
14
5
4
18
15
10
Cluster (Isolation)
1 (−)
2 (−)
3 (−)
4 (−)
5 (−)
6 (L*)
Time Step
15
10
5

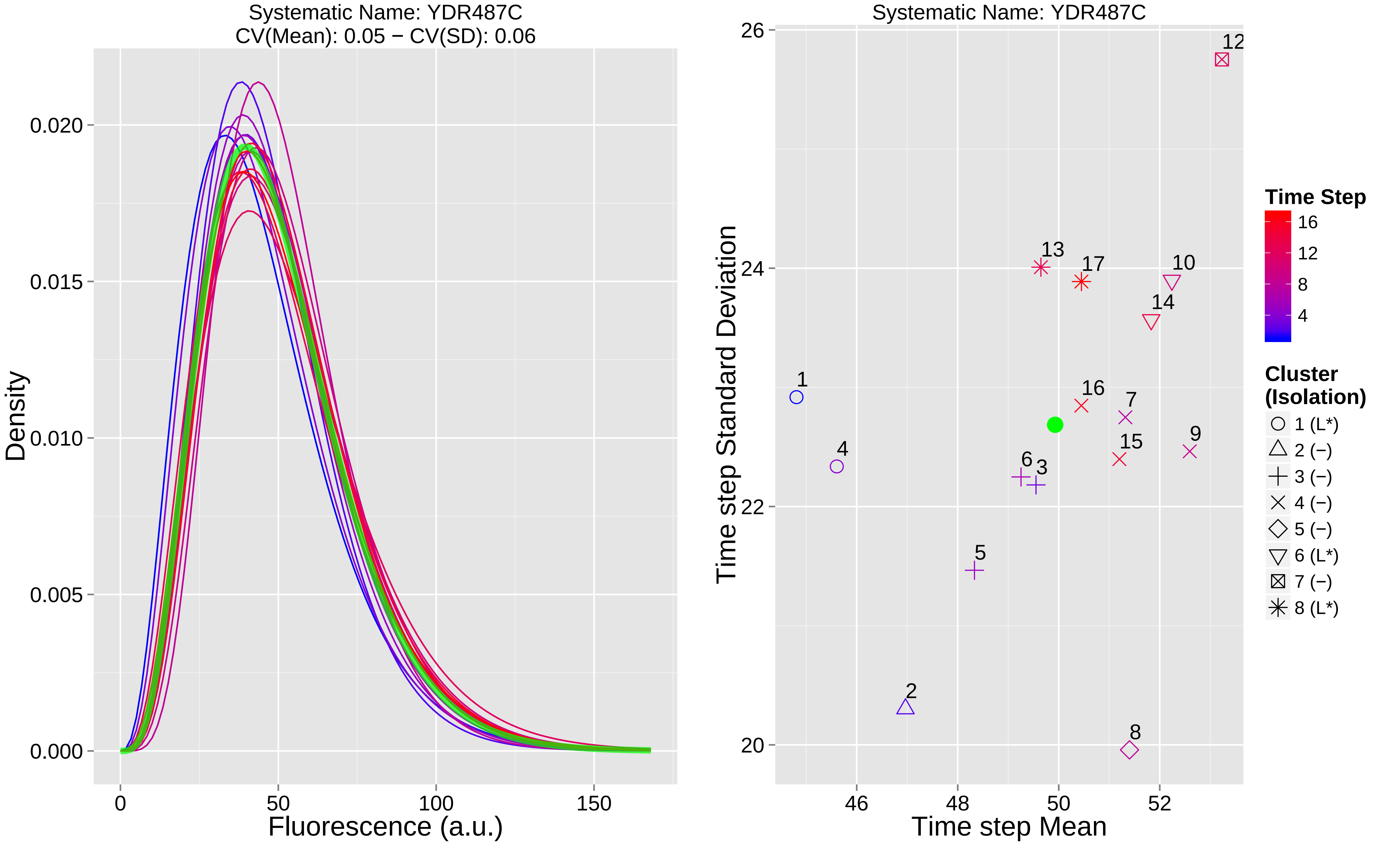
Systematic Name: YDR487C
CV(Mean): 0.05 − CV(SD): 0.06
Density
0.000
0.005
0.010
0.015
0.020
0
50
100
150
Fluorescence (a.u.)
Systematic Name: YDR487C
Time step Standard Deviation
20
22
24
26
46
48
50
52
Time step Mean
1
2
3
4
5
6
7
8
9
10
12
13
14
15
16
17
Time Step
16
12
8
4
Cluster (Isolation)
1 (L*)
2 (−)
3 (−)
4 (−)
5 (−)
6 (L*)
7 (−)
8 (L*)

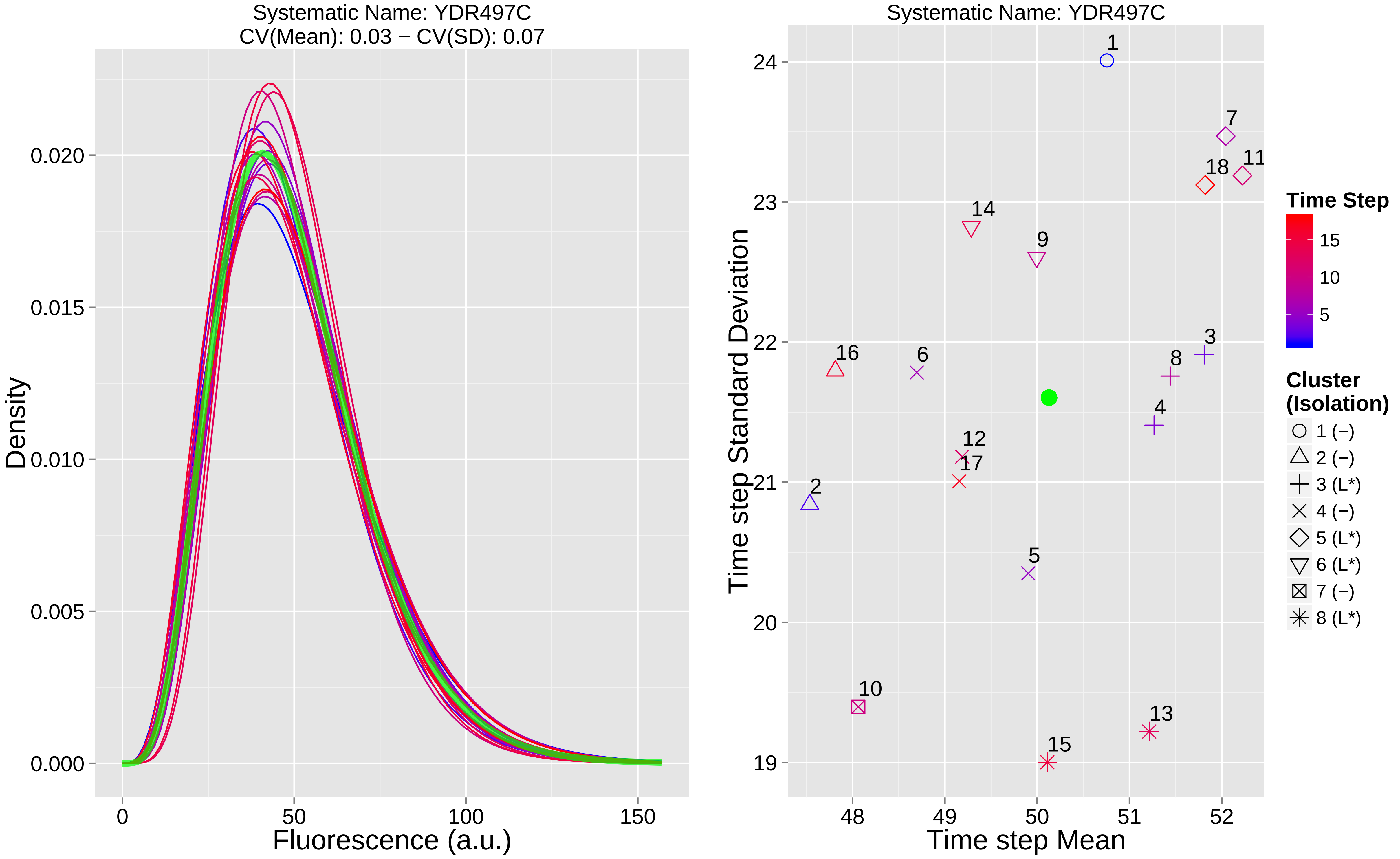
Systematic Name: YDR497C
CV(Mean): 0.03 − CV(SD): 0.07
Density
0.000
0.005
0.010
0.015
0.020
0
50
100
150
Fluorescence (a.u.)
Systematic Name: YDR497C
Time step Standard Deviation
19
20
21
22
23
24
48
49
50
51
52
Time step Mean
1
2
3
4
5
6
7
8
9
10
11
12
13
14
15
16
17
18
Time Step
5
10
15
Cluster (Isolation)
1 (−)
2 (−)
3 (L*)
4 (−)
5 (L*)
6 (L*)
7 (−)
8 (L*)

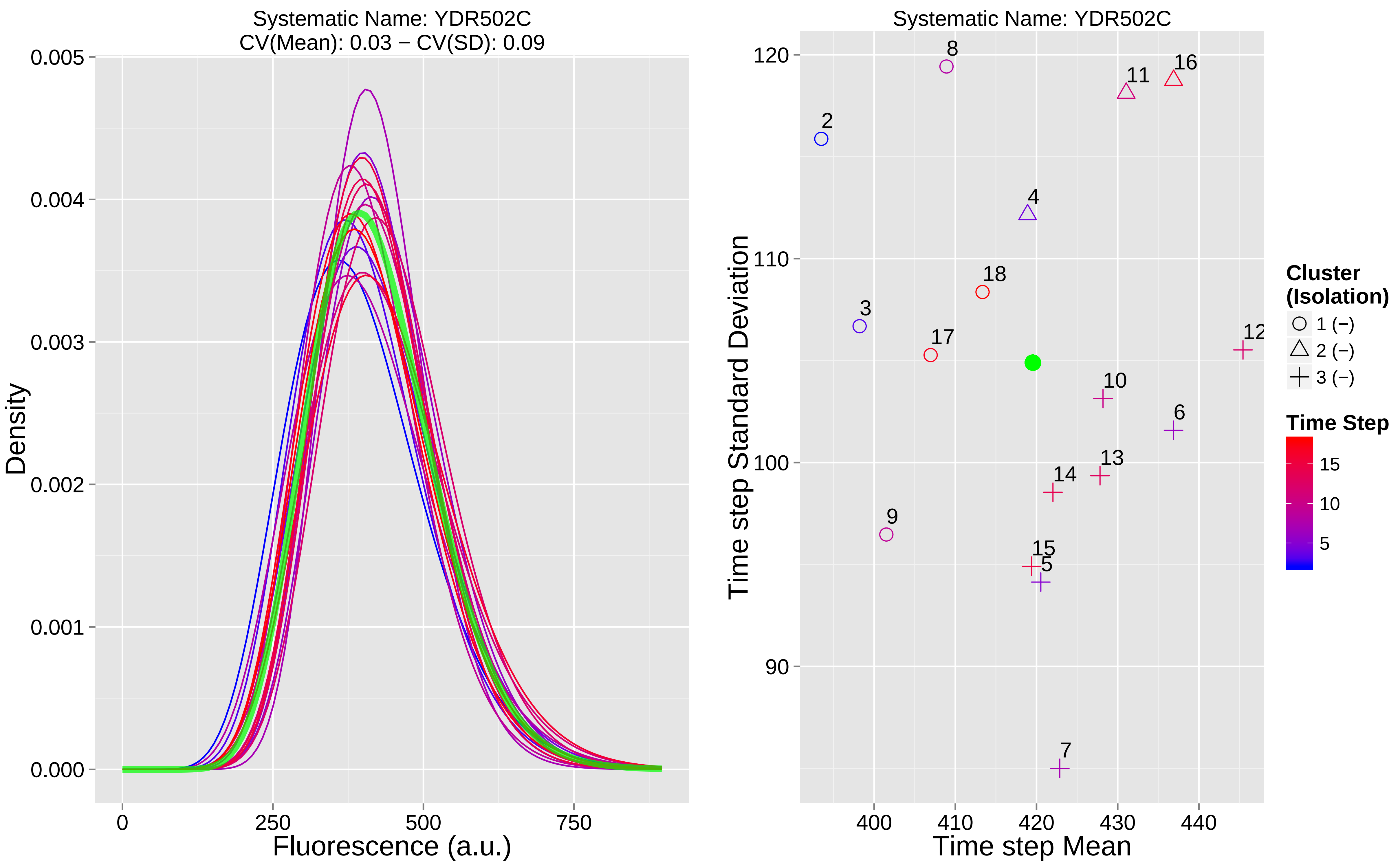

Systematic Name: YDR502C
CV(Mean): 0.03 − CV(SD): 0.09
Density
0.005
0.004
0.003
0.002
0.001
0.000
0
250
500
750
Fluorescence (a.u.)
Systematic Name: YDR502C
Time step Standard Deviation
120
110
100
90
400
410
420
430
440
Time step Mean
2
3
4
5
6
7
8
9
10
11
12
13
14
15
16
17
18
Cluster (Isolation)
1 (−)
2 (−)
3 (−)
Time Step
15
10
5

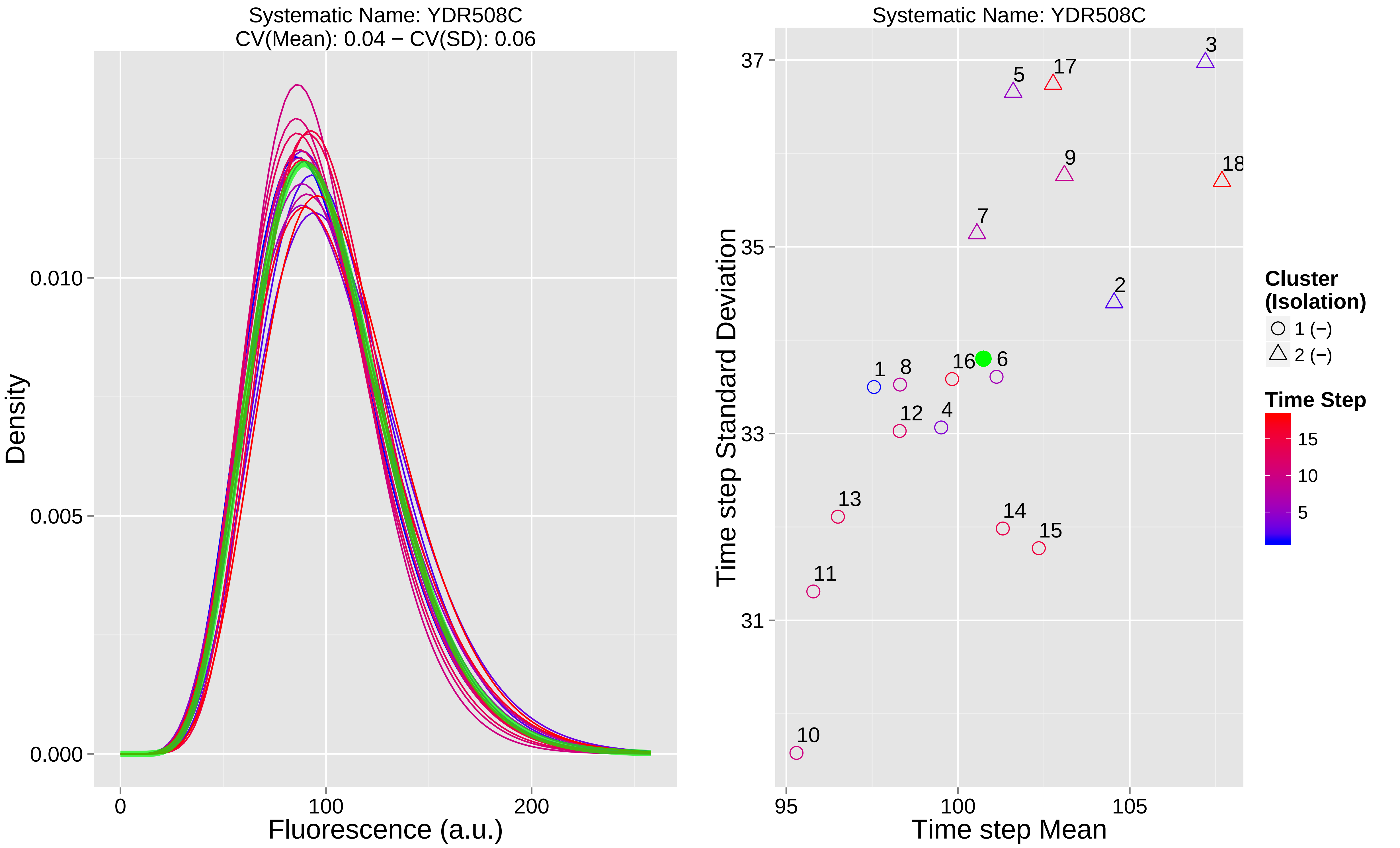
Systematic Name: YDR508C
CV(Mean): 0.04 − CV(SD): 0.06
Density
0.010
0.005
0.000
0
100
200
Fluorescence (a.u.)
Systematic Name: YDR508C
Time step Standard Deviation
37
35
33
31
95
100
105
Time step Mean
1
2
3
4
5
6
7
8
9
10
11
12
13
14
15
16
17
18
Cluster (Isolation)
1 (−)
2 (−)
Time Step
15
10
5

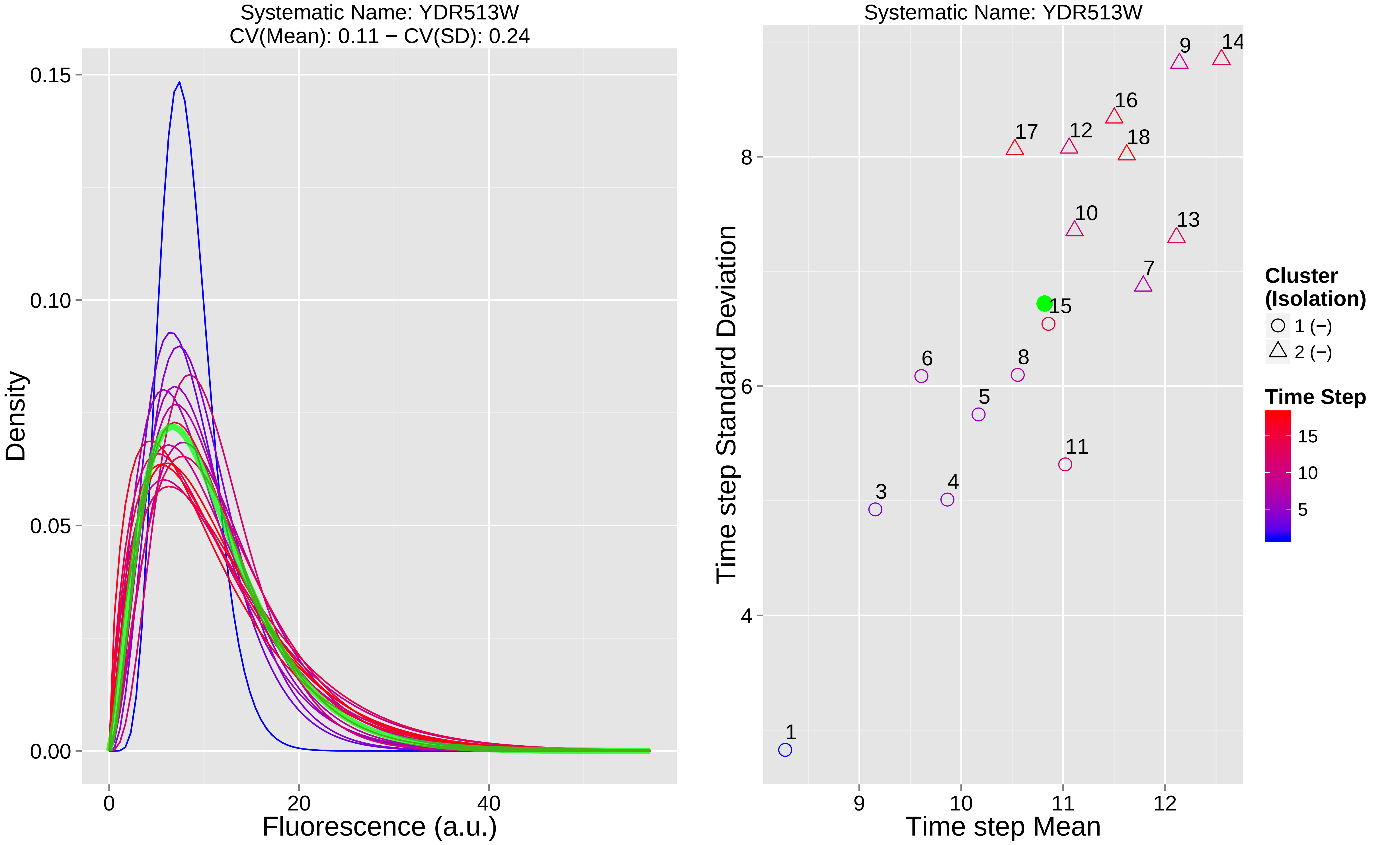

Systematic Name: YDR513W
CV(Mean): 0.11 − CV(SD): 0.24
0.15
0.10
0.05
0.00
Density
0
20
40
Fluorescence (a.u.)
Systematic Name: YDR513W
Time step Standard Deviation
8
6
4
9
10
11
12
Time step Mean
1
3
4
5
6
8
11
15
7
10
13
17
12
18
16
9
14
Cluster (Isolation)
1 (−)
2 (−)
Time Step
15
10
5

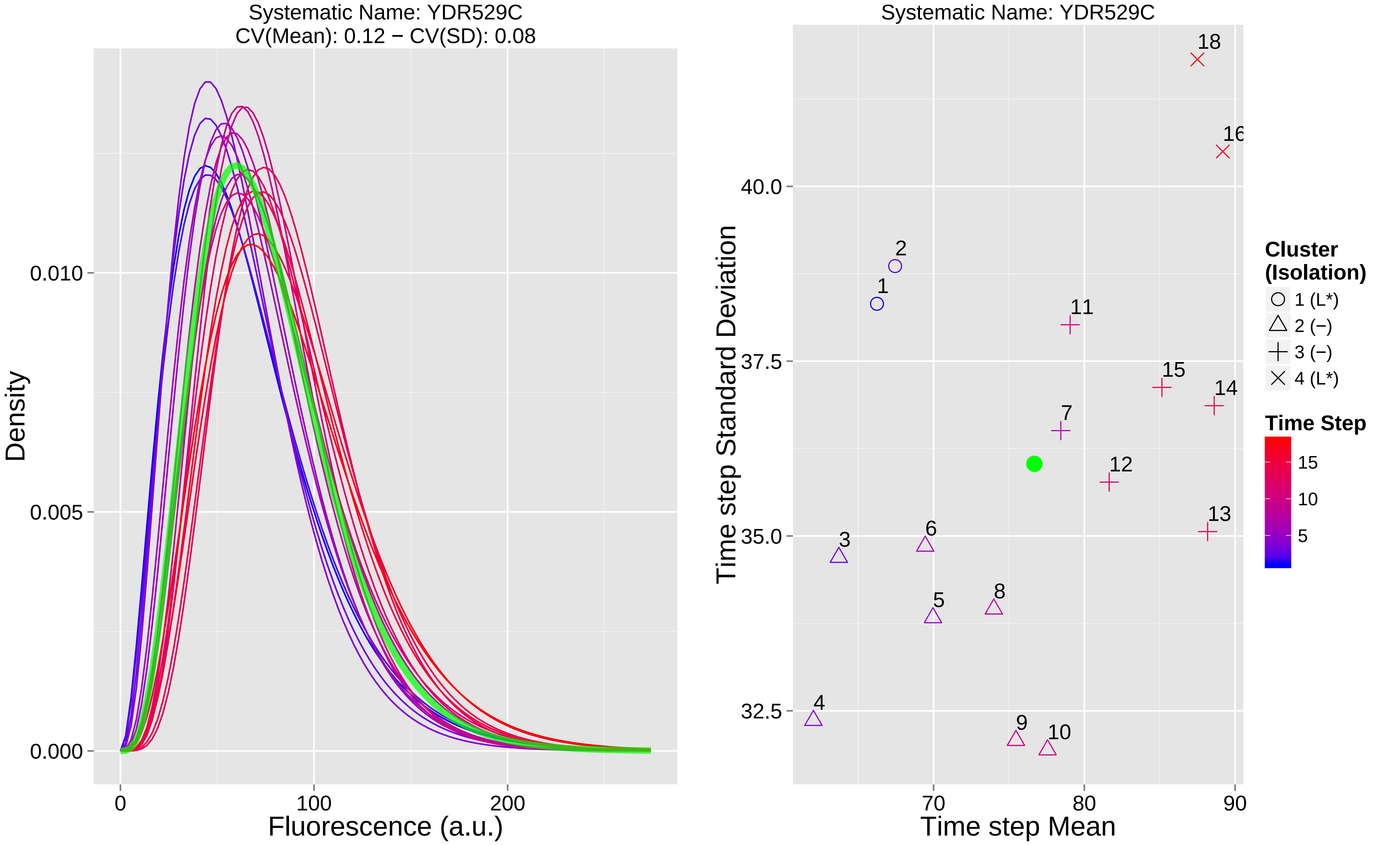
Systematic Name: YDR529C
CV(Mean): 0.12 − CV(SD): 0.08
Density
0.010
0.005
0.000
0
100
200
Fluorescence (a.u.)
Systematic Name: YDR529C
Time step Standard Deviation
40.0
37.5
35.0
32.5
70
80
90
Time step Mean
Cluster (Isolation)
1 (L*)
2 (−)
3 (−)
4 (L*)
Time Step
15
10
5
1
2
3
4
5
6
7
8
9
10
11
12
13
14
15
16
18

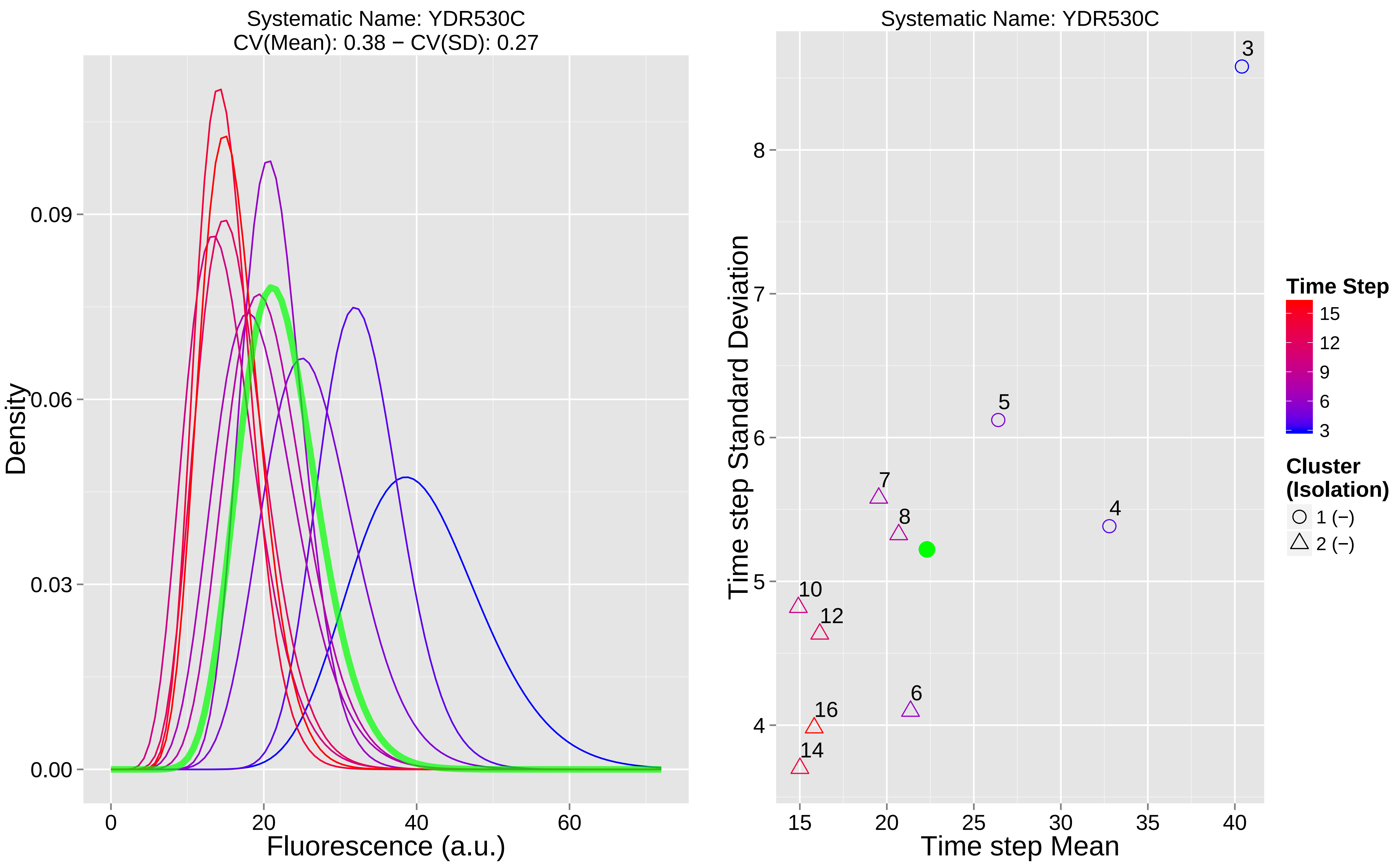

Systematic Name: YDR530C
CV(Mean): 0.38 − CV(SD): 0.27
Density
Fluorescence (a.u.)
0.00
0.03
0.06
0.09
0
20
40
60
Systematic Name: YDR530C
Time step Standard Deviation
Time step Mean
4
5
6
7
8
15
20
25
30
35
40
3
4
5
6
7
8
10
12
14
16
Time Step
15
12
9
6
3
Cluster
(Isolation)
1 (−)
2 (−)

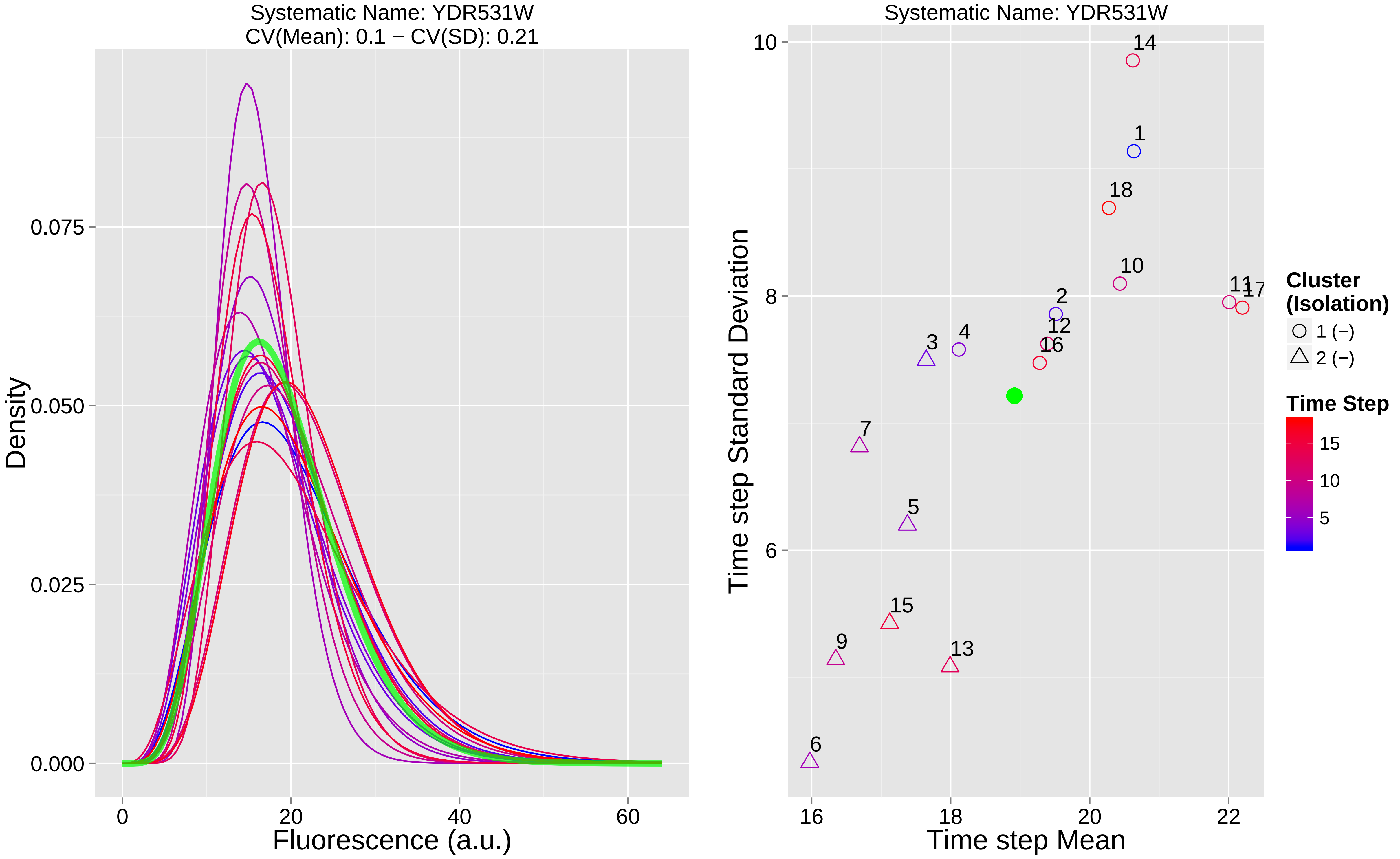
Systematic Name: YDR531W
CV(Mean): 0.1 − CV(SD): 0.21
Density
0.000
0.025
0.050
0.075
0
20
40
60
Fluorescence (a.u.)
Systematic Name: YDR531W
Time step Standard Deviation
6
8
10
16
18
20
22
Time step Mean
1
2
3
4
5
6
7
9
10
11
12
13
14
15
16
17
18
Cluster
(Isolation)
1 (−)
2 (−)
Time Step
5
10
15

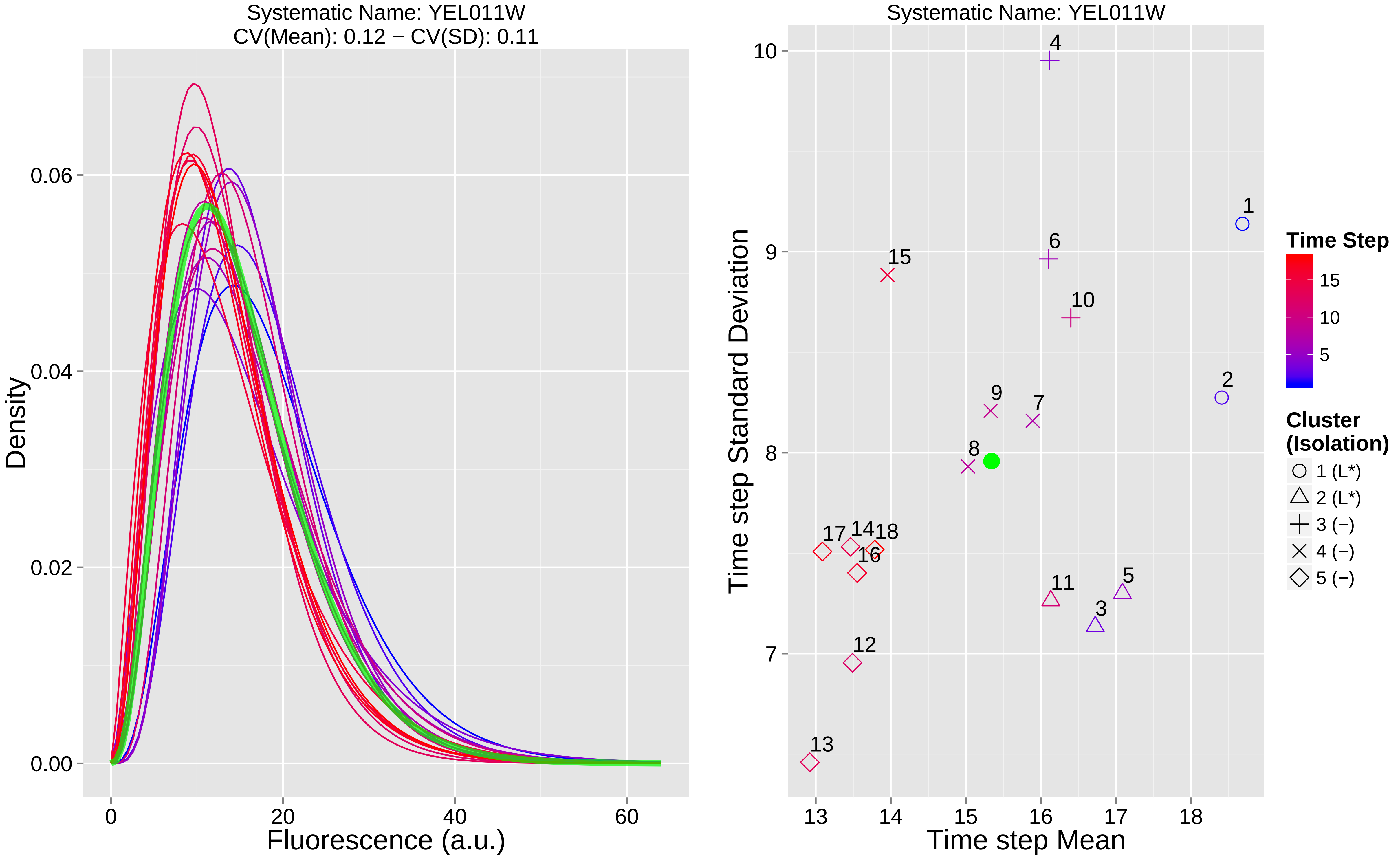

Systematic Name: YEL011W
CV(Mean): 0.12 − CV(SD): 0.11
Density
0.00
0.02
0.04
0.06
0
20
40
60
Fluorescence (a.u.)
Systematic Name: YEL011W
Time step Standard Deviation
7
8
9
10
13
14
15
16
17
18
Time step Mean
1
2
3
4
5
6
7
8
9
10
11
12
13
14
15
16
17
18
Time Step
15
10
5
Cluster (Isolation)
1 (L*)
2 (L*)
3 (−)
4 (−)
5 (−)

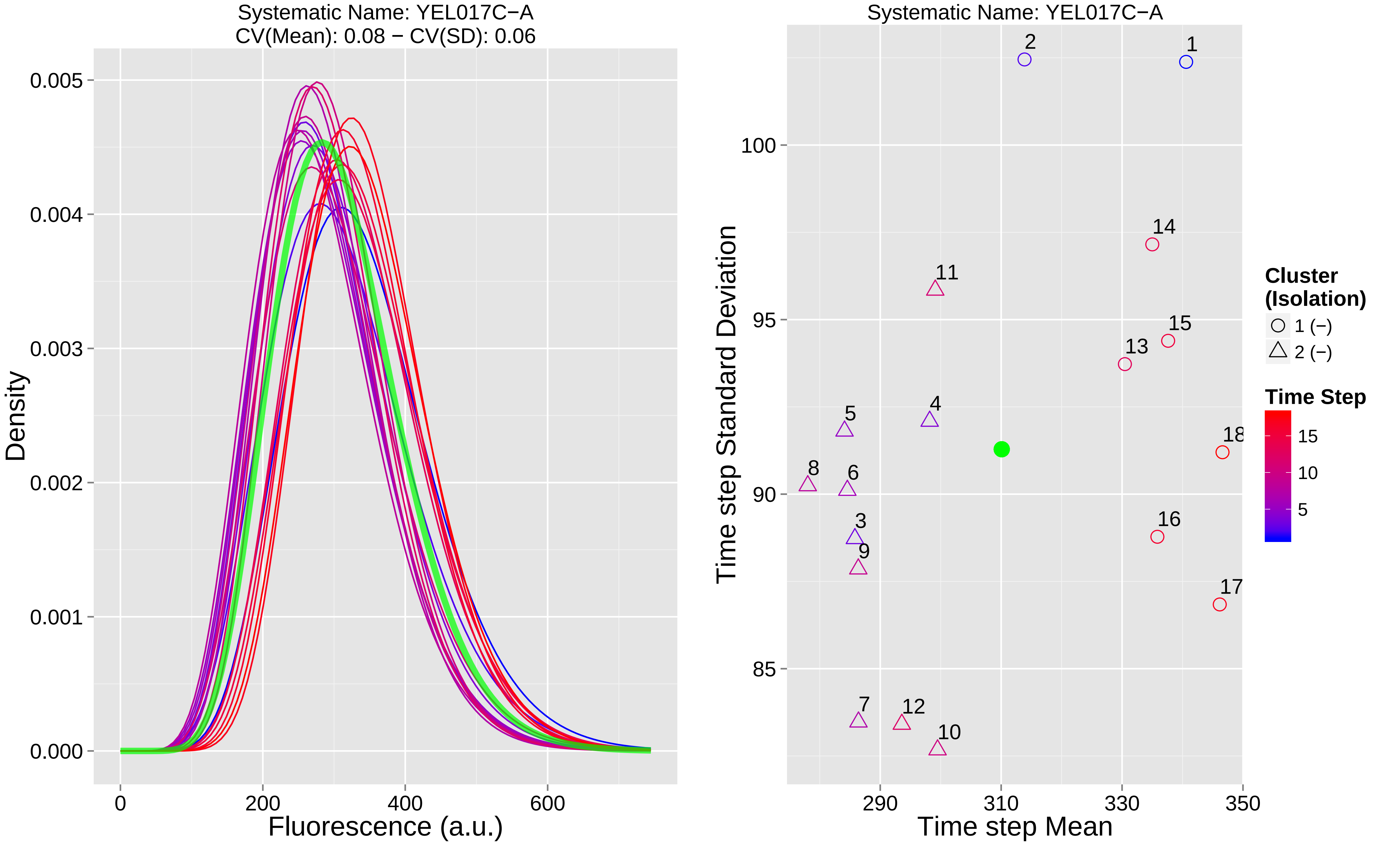
Systematic Name: YEL017C−A
CV(Mean): 0.08 − CV(SD): 0.06
Density
Fluorescence (a.u.)
0.005
0.004
0.003
0.002
0.001
0.000
0
200
400
600
Systematic Name: YEL017C−A
Time step Standard Deviation
Time step Mean
100
95
90
85
290
310
330
350
Cluster (Isolation)
1 (−)
2 (−)
Time Step
15
10
5

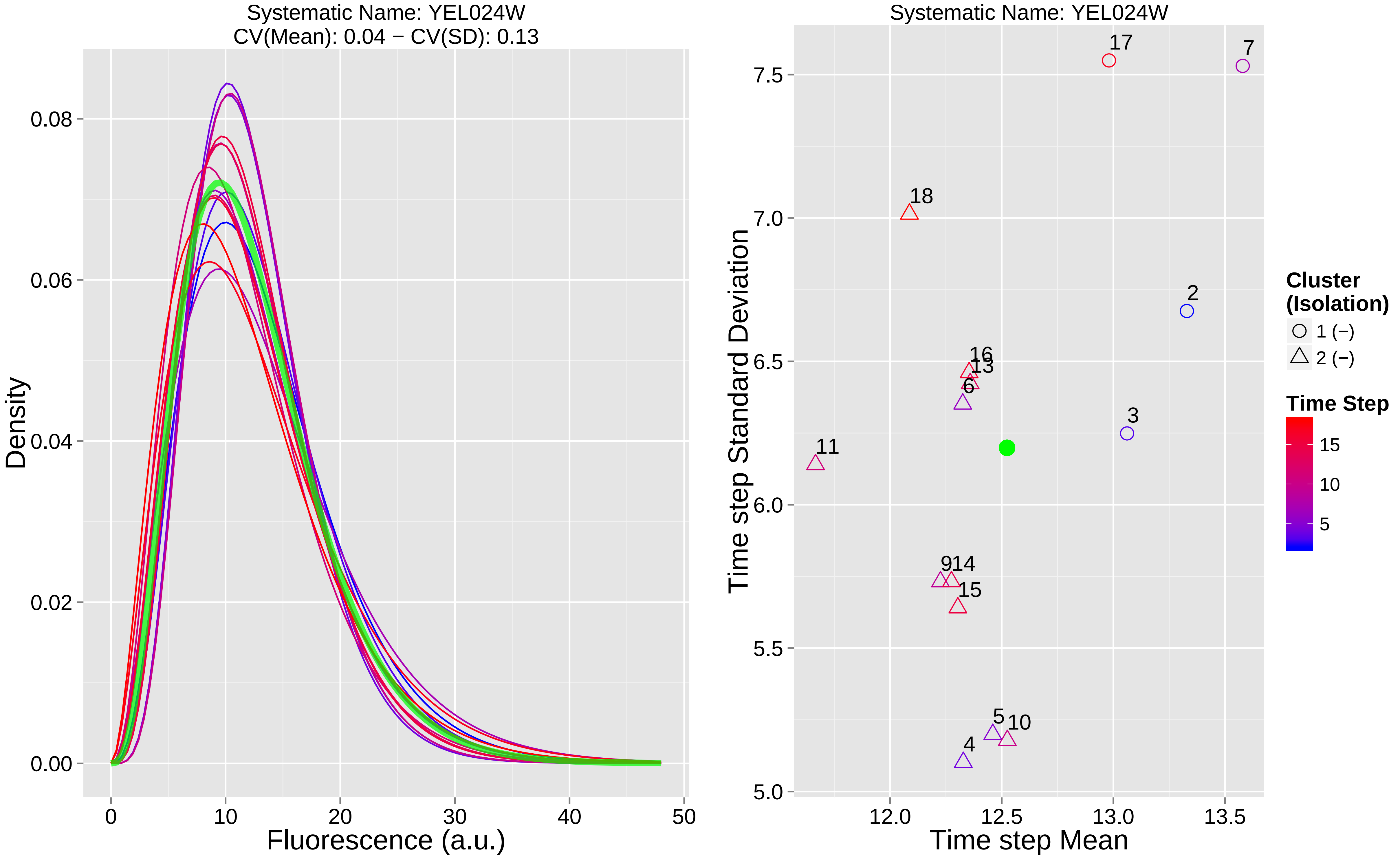

Systematic Name: YEL024W
CV(Mean): 0.04 − CV(SD): 0.13
Density
Fluorescence (a.u.)
0.00
0.02
0.04
0.06
0.08
0
10
20
30
40
50
Systematic Name: YEL024W
Time step Standard Deviation
Time step Mean
5.0
5.5
6.0
6.5
7.0
7.5
12.0
12.5
13.0
13.5
17
7
18
2
16
13
6
3
11
9
14
15
5
10
4
Cluster
(Isolation)
1 (−)
2 (−)
Time Step
15
10
5

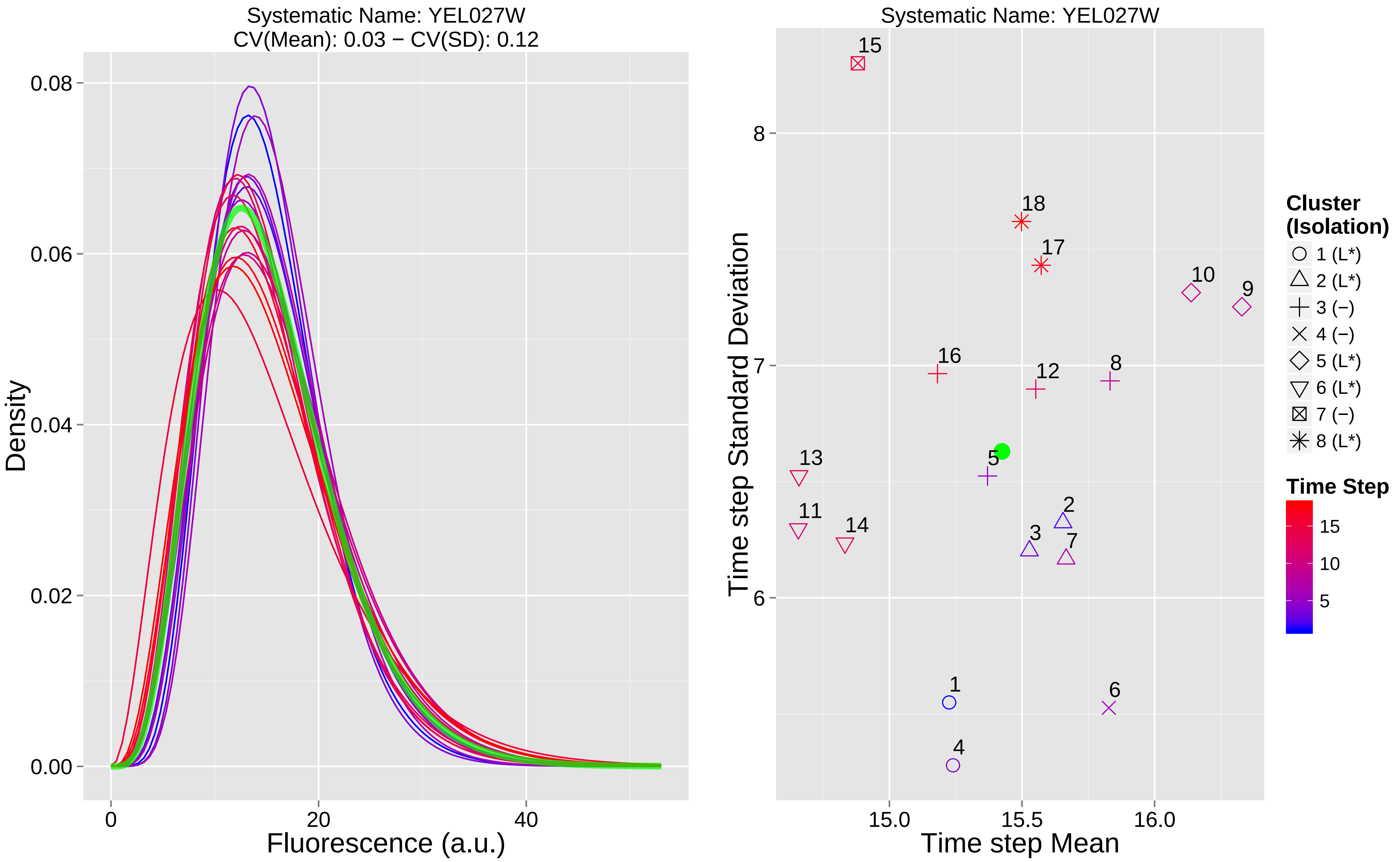

Systematic Name: YEL027W
CV(Mean): 0.03 − CV(SD): 0.12
Density
0.08
0.06
0.04
0.02
0.00
0
20
40
Fluorescence (a.u.)
Systematic Name: YEL027W
Time step Standard Deviation
8
7
6
15.0
15.5
16.0
Time step Mean
15
18
17
10
9
16
12
8
13
5
2
11
14
3
7
1
6
4
Cluster (Isolation)
1 (L*)
2 (L*)
3 (−)
4 (−)
5 (L*)
6 (L*)
7 (−)
8 (L*)
Time Step
15
10
5

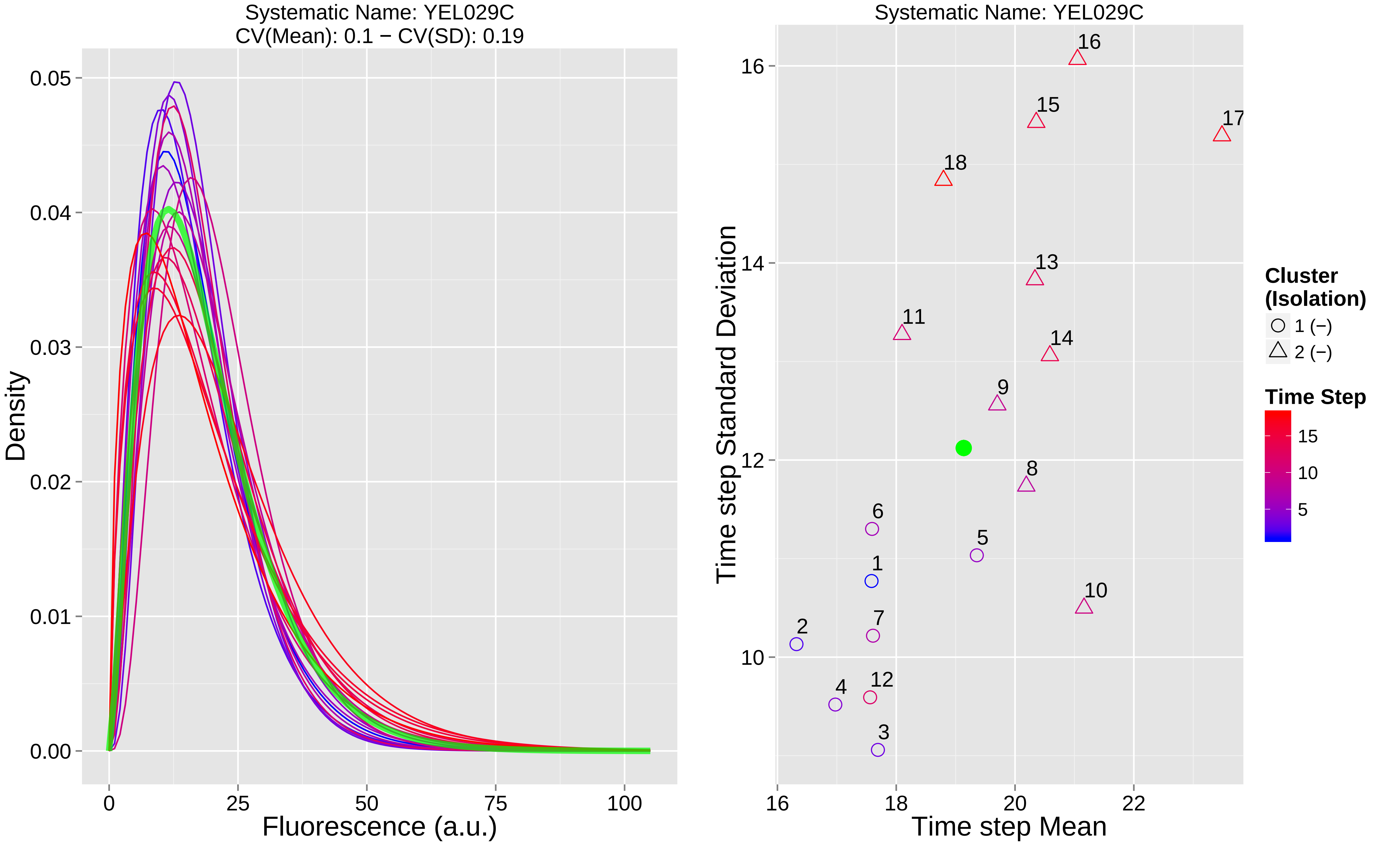

Systematic Name: YEL029C
CV(Mean): 0.1 − CV(SD): 0.19
Density
Fluorescence (a.u.)
0.00
0.01
0.02
0.03
0.04
0.05
0
25
50
75
100
Systematic Name: YEL029C
Time step Standard Deviation
Time step Mean
10
12
14
16
16
18
20
22
Cluster (Isolation)
1 (−)
2 (−)
Time Step
5
10
15

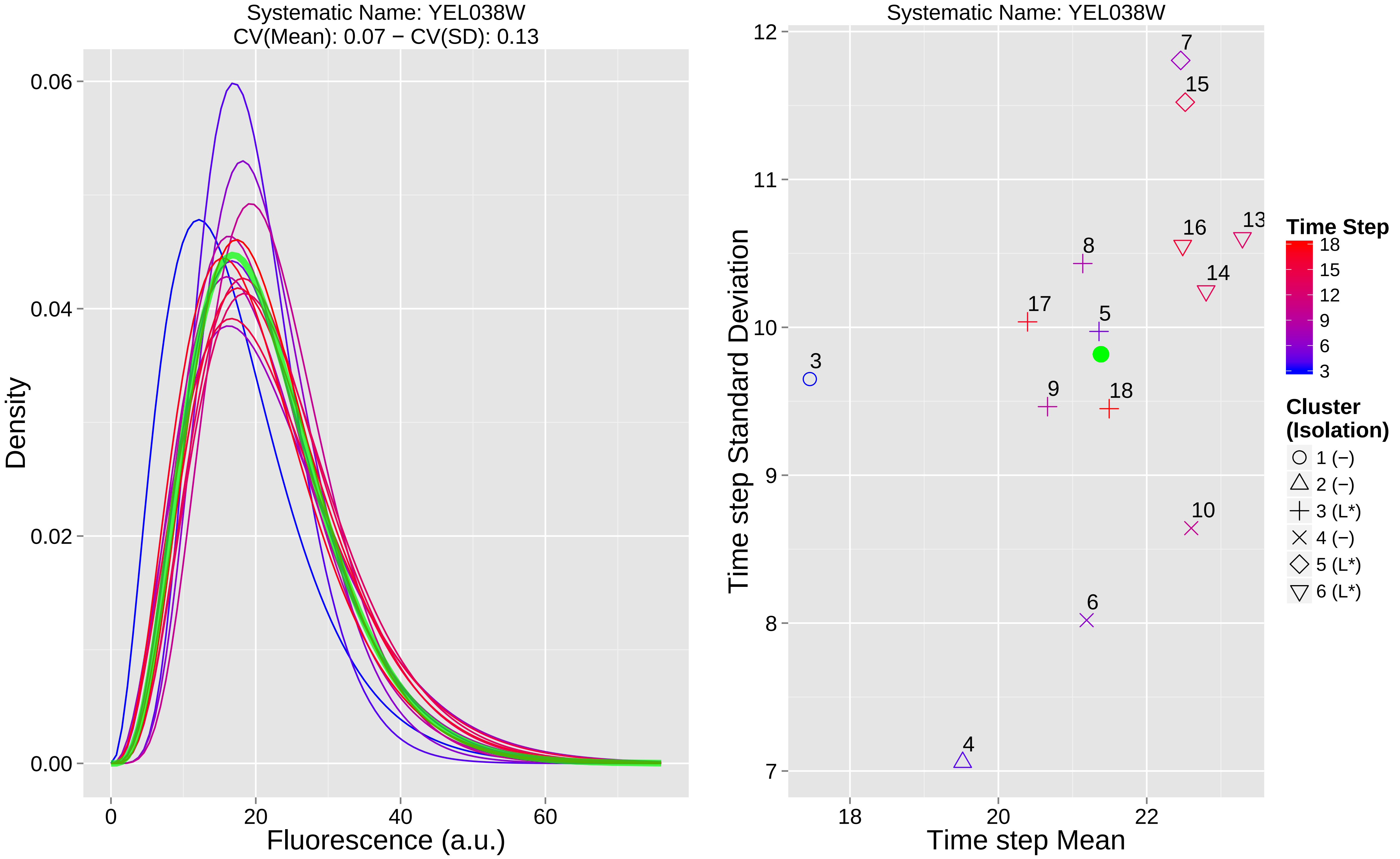
Systematic Name: YEL038W
CV(Mean): 0.07 − CV(SD): 0.13
Density
0.06
0.04
0.02
0.00
0
20
40
60
Fluorescence (a.u.)
Systematic Name: YEL038W
Time step Standard Deviation
12
11
10
9
8
7
18
20
22
Time step Mean
7
15
16
13
14
8
17
5
3
9
18
10
6
4
Time Step
18
15
12
9
6
3
Cluster
(Isolation)
1 (−)
2 (−)
3 (L*)
4 (−)
5 (L*)
6 (L*)

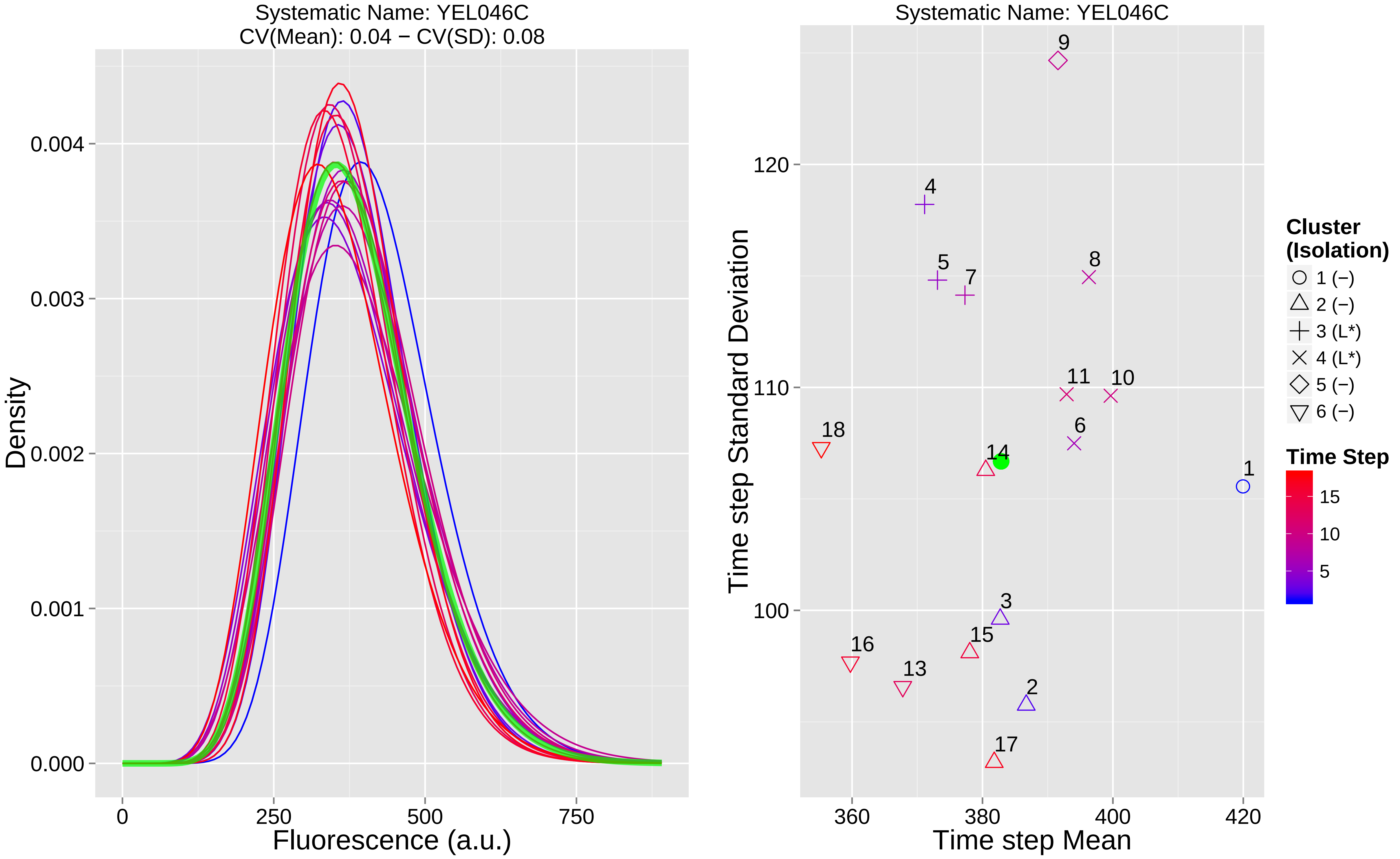

Systematic Name: YEL046C
CV(Mean): 0.04 − CV(SD): 0.08
Density
0.000
0.001
0.002
0.003
0.004
0
250
500
750
Fluorescence (a.u.)
Systematic Name: YEL046C
Time step Standard Deviation
100
110
120
360
380
400
420
Time step Mean
1
2
3
4
5
6
7
8
9
10
11
13
14
15
16
17
18
Cluster (Isolation)
1 (−)
2 (−)
3 (L*)
4 (L*)
5 (−)
6 (−)
Time Step
5
10
15

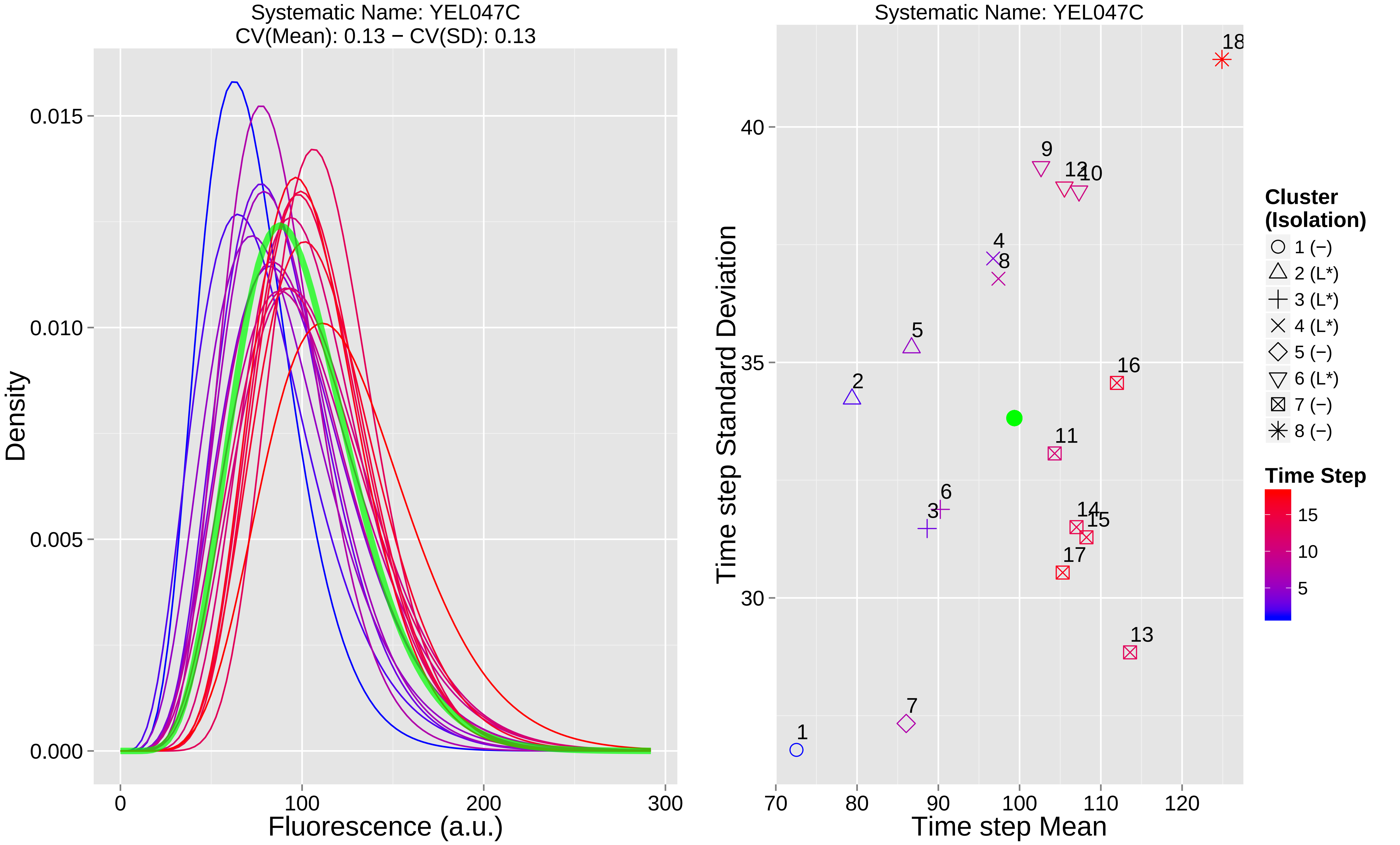

Systematic Name: YEL047C
CV(Mean): 0.13 − CV(SD): 0.13
Density
0.015
0.010
0.005
0.000
0
100
200
300
Fluorescence (a.u.)
Systematic Name: YEL047C
Time step Standard Deviation
40
35
30
70
80
90
100
110
120
Time step Mean
1
2
3
4
5
6
7
8
9
10
11
12
13
14
15
16
17
18
Cluster
(Isolation)
1 (−)
2 (L*)
3 (L*)
4 (L*)
5 (−)
6 (L*)
7 (−)
8 (−)
Time Step
15
10
5

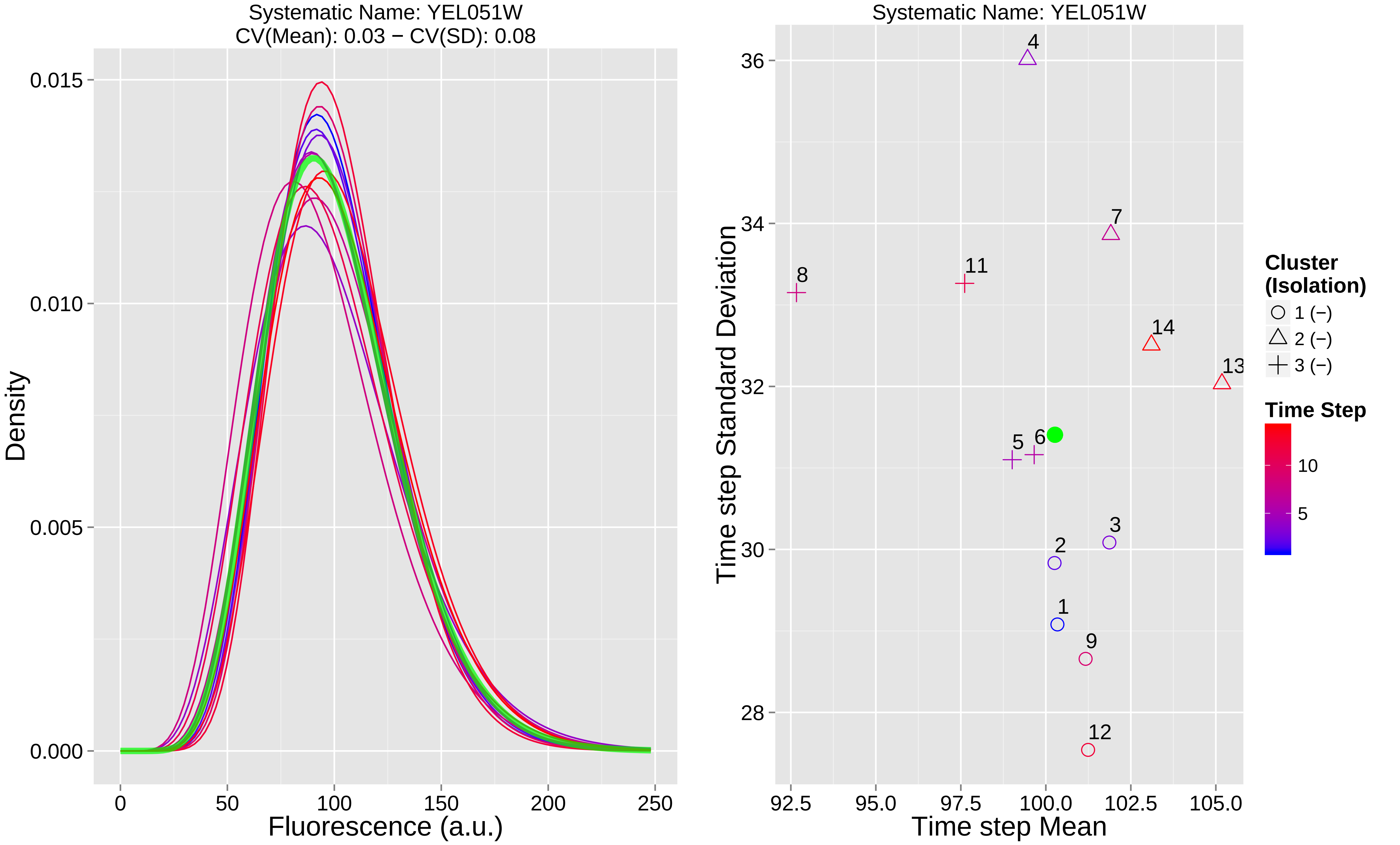
Systematic Name: YEL051W
CV(Mean): 0.03 − CV(SD): 0.08
Density
Fluorescence (a.u.)
0.015
0.010
0.005
0.000
0
50
100
150
200
250
Systematic Name: YEL051W
Time step Standard Deviation
Time step Mean
36
34
32
30
28
92.5
95.0
97.5
100.0
102.5
105.0
1
2
3
4
5
6
7
8
9
11
12
13
14
Cluster (Isolation)
1 (−)
2 (−)
3 (−)
Time Step
10
5

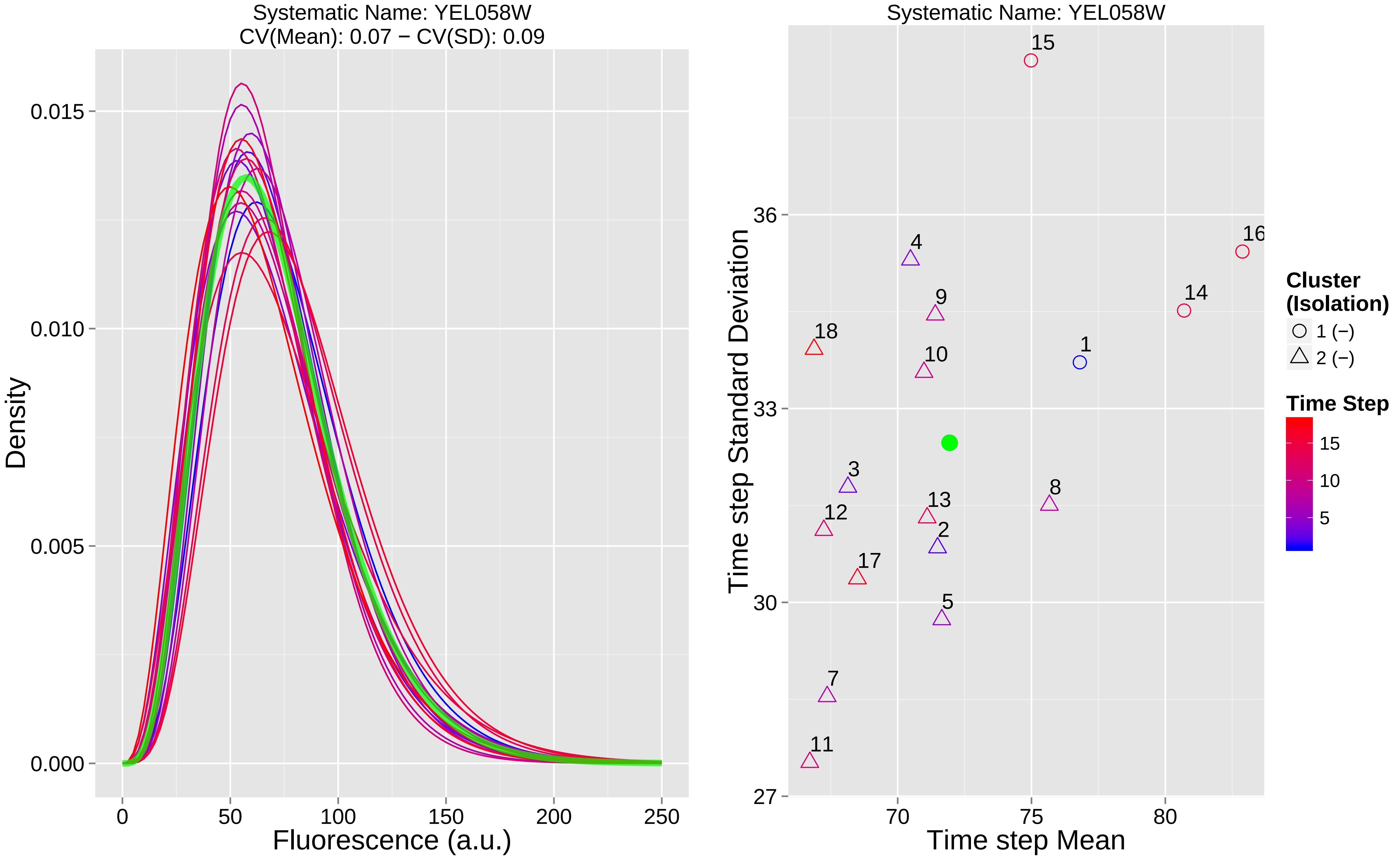

Systematic Name: YEL058W
CV(Mean): 0.07 − CV(SD): 0.09
Density
0.015
0.010
0.005
0.000
0
50
100
150
200
250
Fluorescence (a.u.)
Systematic Name: YEL058W
Time step Standard Deviation
36
33
30
27
70
75
80
Time step Mean
15
16
4
9
14
18
1
10
3
8
13
12
2
17
5
7
11
Cluster
(Isolation)
1 (−)
2 (−)
Time Step
15
10
5

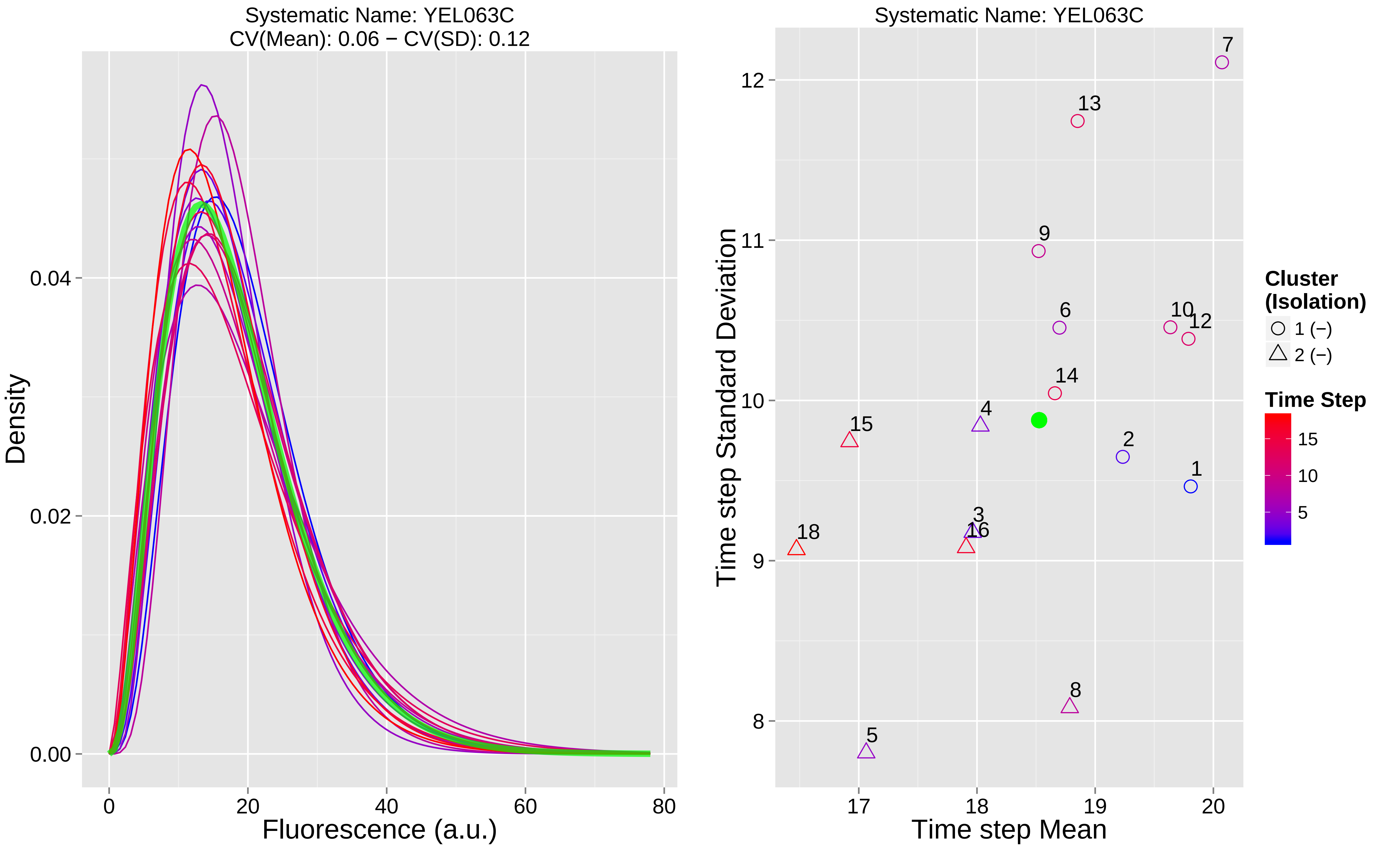

Systematic Name: YEL063C
CV(Mean): 0.06 − CV(SD): 0.12
Density
0.00
0.02
0.04
0
20
40
60
80
Fluorescence (a.u.)
Systematic Name: YEL063C
Time step Standard Deviation
8
9
10
11
12
17
18
19
20
Time step Mean
1
2
3
4
5
6
7
8
9
10
12
13
14
15
16
18
Cluster (Isolation)
1 (−)
2 (−)
Time Step
5
10
15

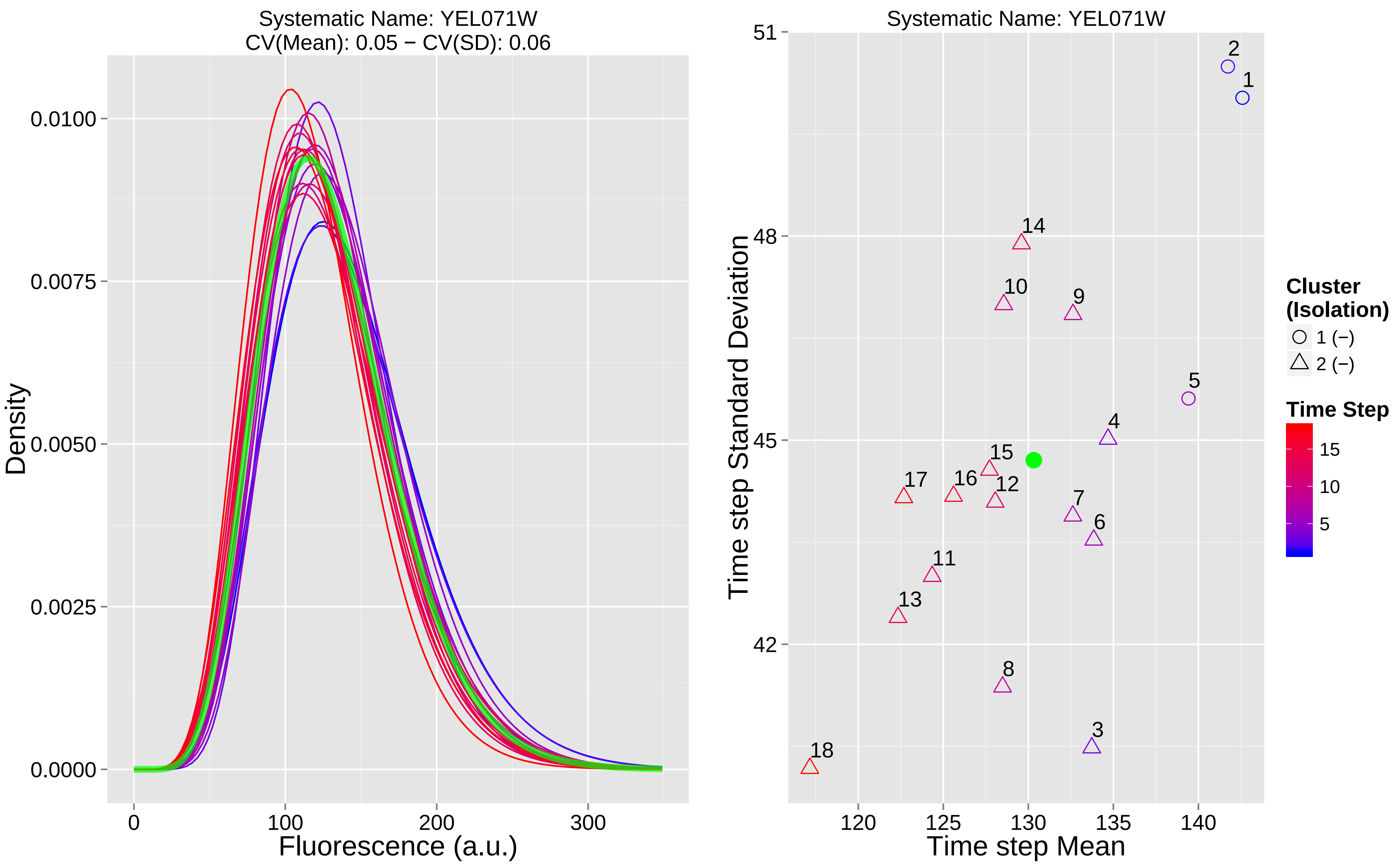
Systematic Name: YEL071W
CV(Mean): 0.05 − CV(SD): 0.06
Density
0.0100
0.0075
0.0050
0.0025
0.0000
0
100
200
300
Fluorescence (a.u.)
Systematic Name: YEL071W
Time step Standard Deviation
51
48
45
42
120
125
130
135
140
Time step Mean
Cluster
(Isolation)
1 (−)
2 (−)
Time Step
15
10
5

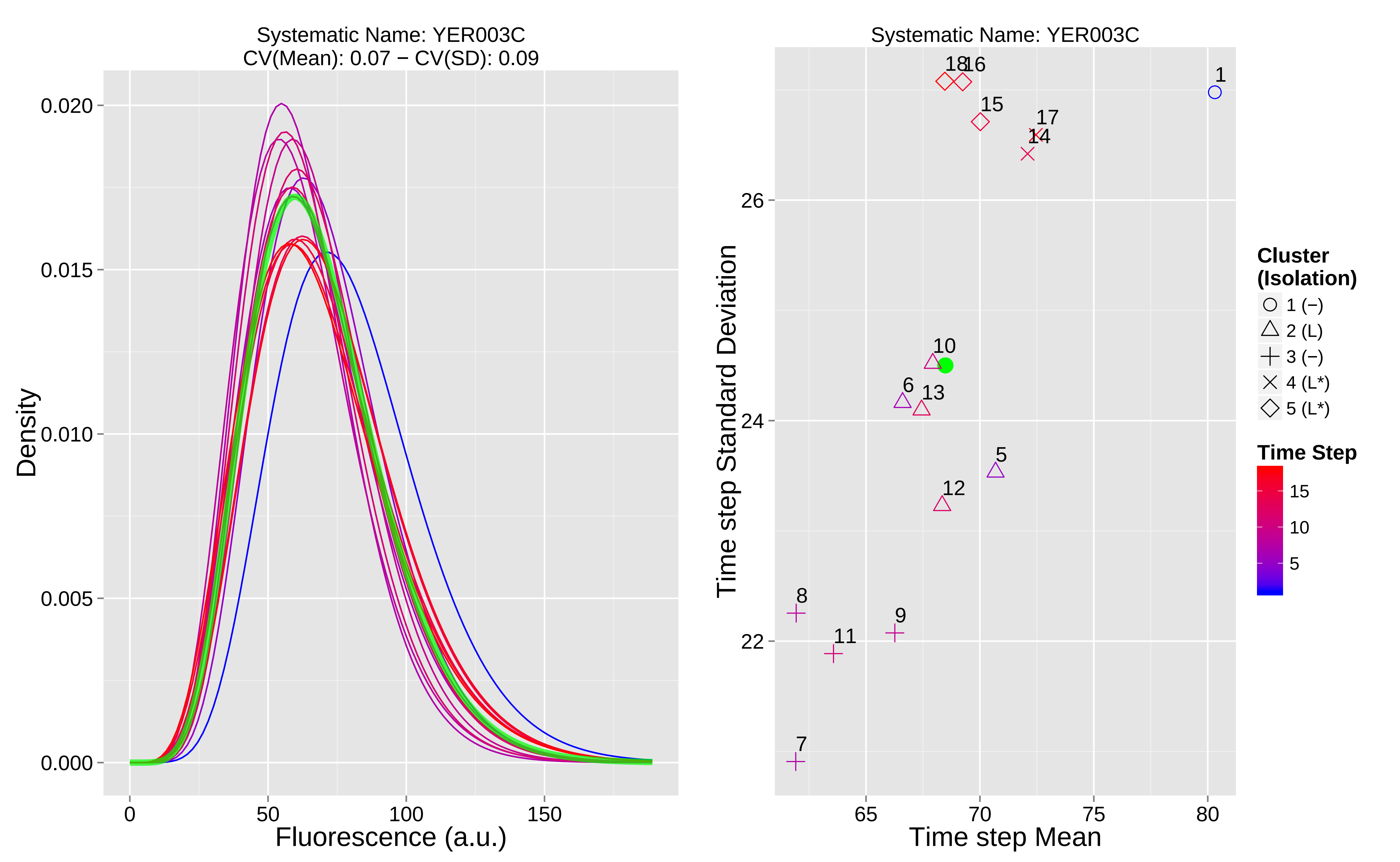
Systematic Name: YER003C
CV(Mean): 0.07 − CV(SD): 0.09
Density
0.020
0.015
0.010
0.005
0.000
0
50
100
150
Fluorescence (a.u.)
Systematic Name: YER003C
Time step Standard Deviation
26
24
22
65
70
75
80
Time step Mean
1
18
16
15
17
14
10
6
13
5
12
8
9
11
7
Cluster
(Isolation)
1 (−)
2 (L)
3 (−)
4 (L*)
5 (L*)
Time Step
15
10
5

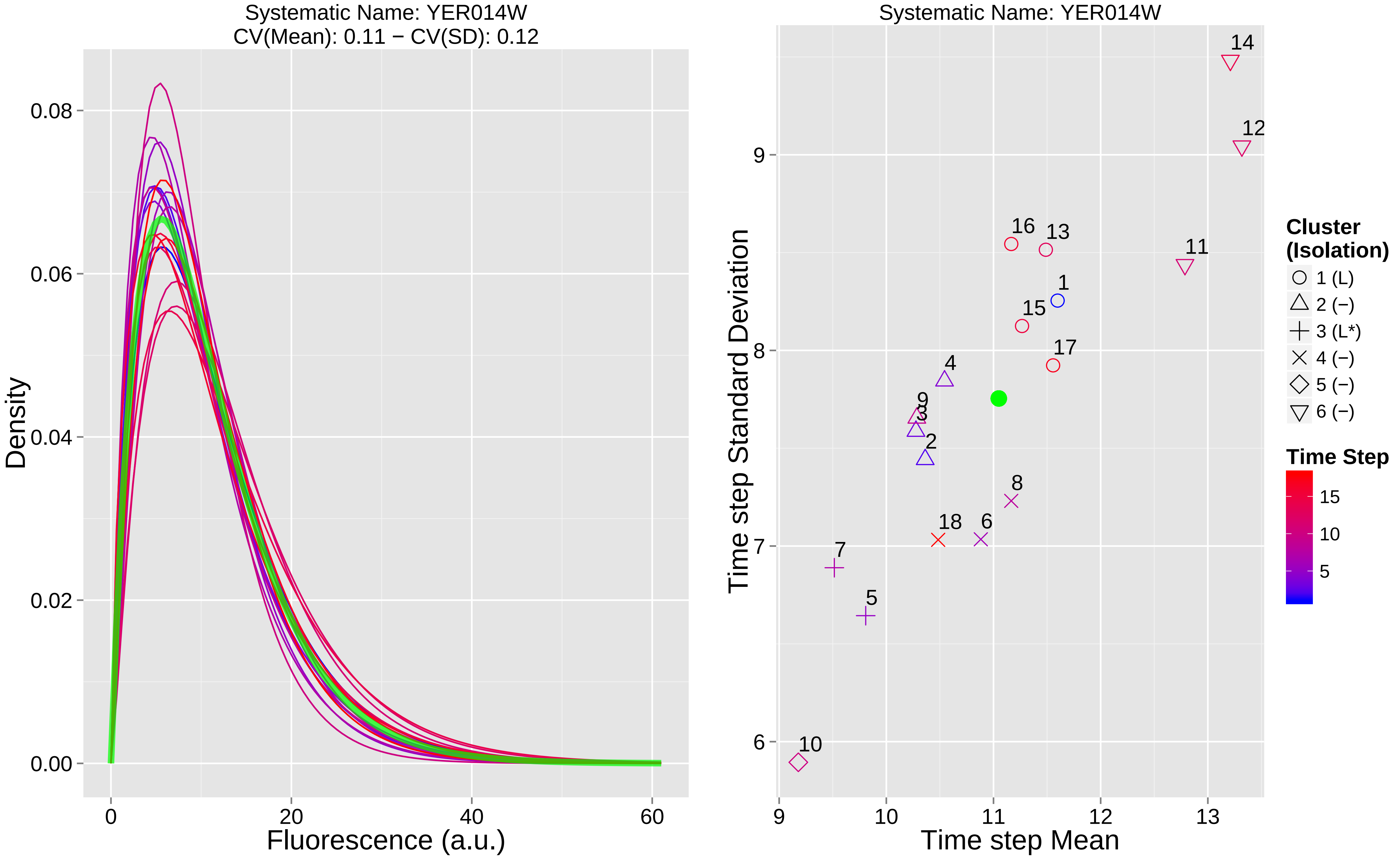
Systematic Name: YER014W
CV(Mean): 0.11 − CV(SD): 0.12
Density
Fluorescence (a.u.)
0.00
0.02
0.04
0.06
0.08
0
20
40
60
Systematic Name: YER014W
Time step Standard Deviation
Time step Mean
6
7
8
9
9
10
11
12
13
Cluster (Isolation)
1 (L)
2 (−)
3 (L*)
4 (−)
5 (−)
6 (−)
Time Step
15
10
5
14
12
16
13
11
1
15
17
4
9
3
2
8
18
6
7
5
10

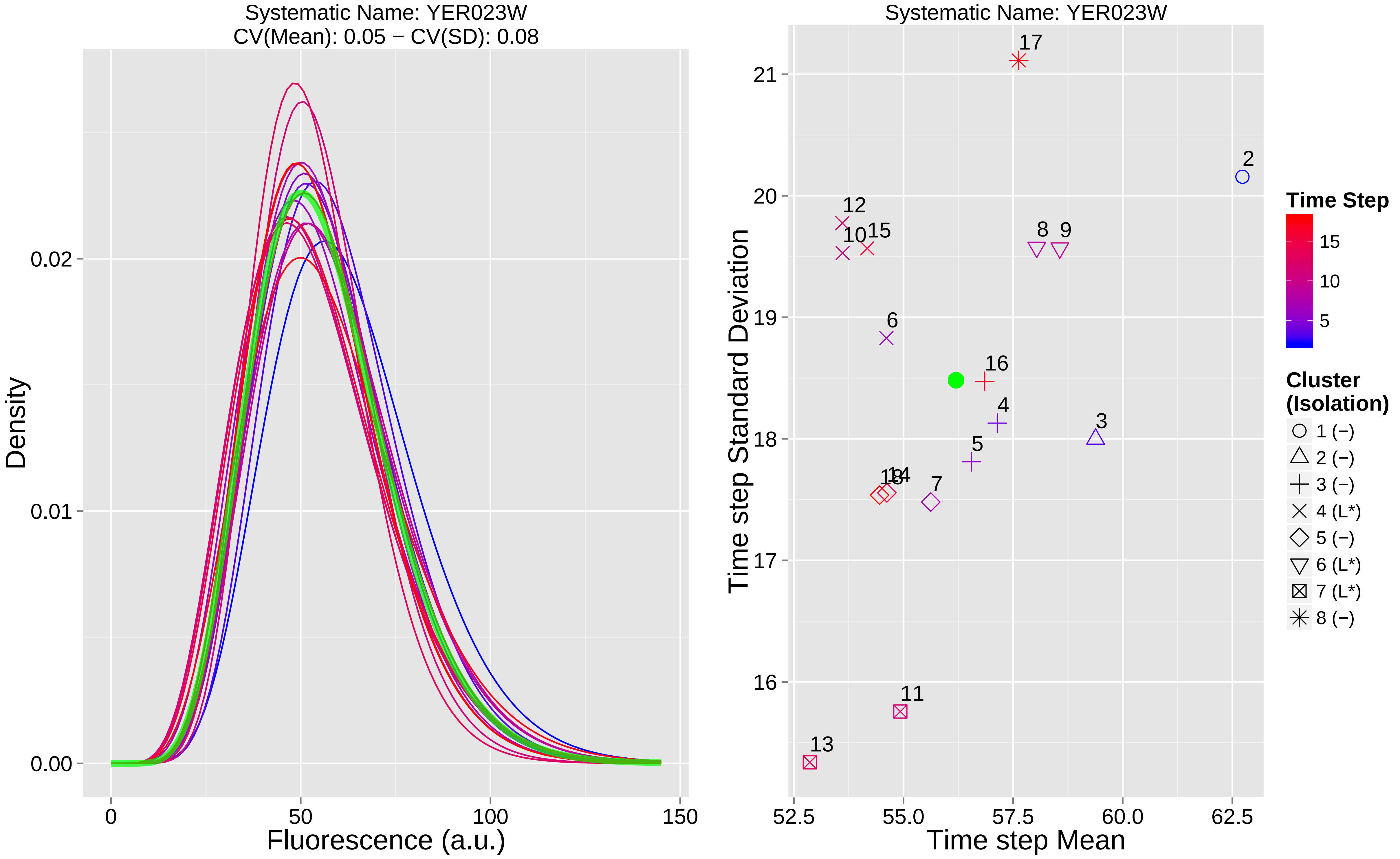

Systematic Name: YER023W
CV(Mean): 0.05 − CV(SD): 0.08
Density
Fluorescence (a.u.)
0.00
0.01
0.02
0
50
100
150
Systematic Name: YER023W
Time step Standard Deviation
Time step Mean
52.5
55.0
57.5
60.0
62.5
16
17
18
19
20
21
Time Step
15
10
5
Cluster
(Isolation)
1 (−)
2 (−)
3 (−)
4 (L*)
5 (−)
6 (L*)
7 (L*)
8 (−)

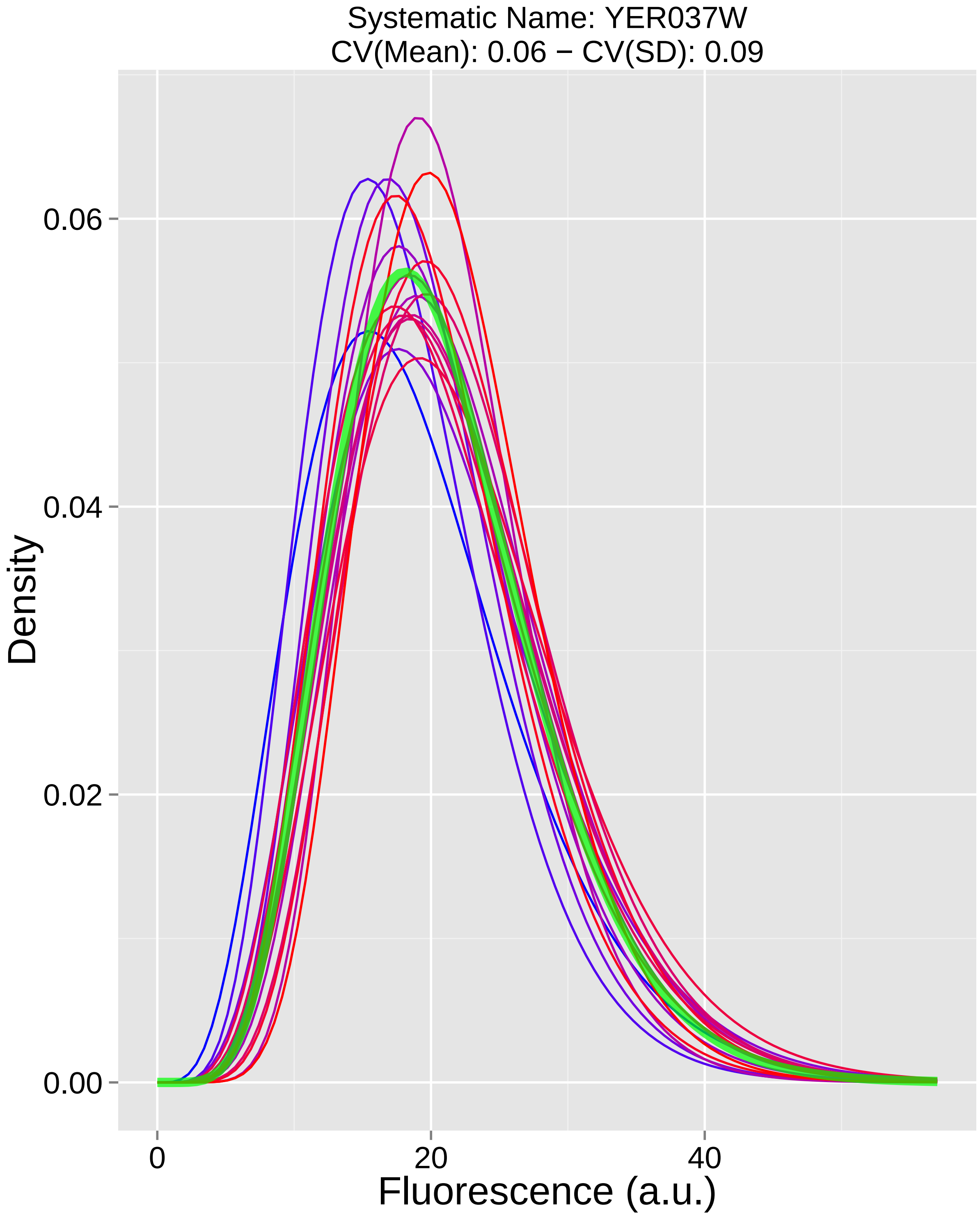

Systematic Name: YER037W
CV(Mean): 0.06 − CV(SD): 0.09
Density
0.06
0.04
0.02
0.00
0
20
40
Fluorescence (a.u.)

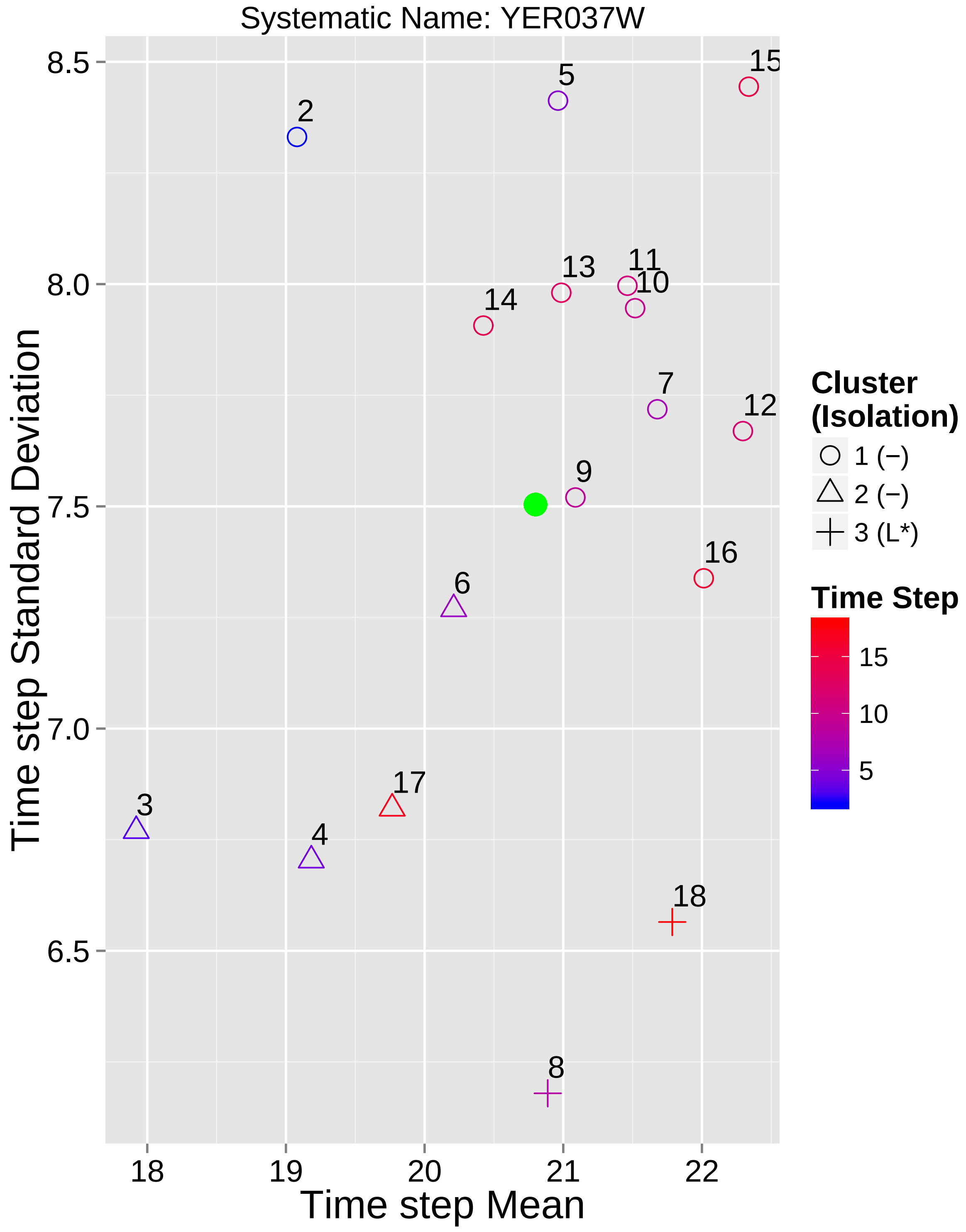

Systematic Name: YER037W
Time step Standard Deviation
8.5
8.0
7.5
7.0
6.5
18
19
20
21
22
Time step Mean
2
5
15
13
11
10
14
7
12
9
16
6
17
3
4
18
8
Cluster (Isolation)
1 (−)
2 (−)
3 (L*)
Time Step
15
10
5

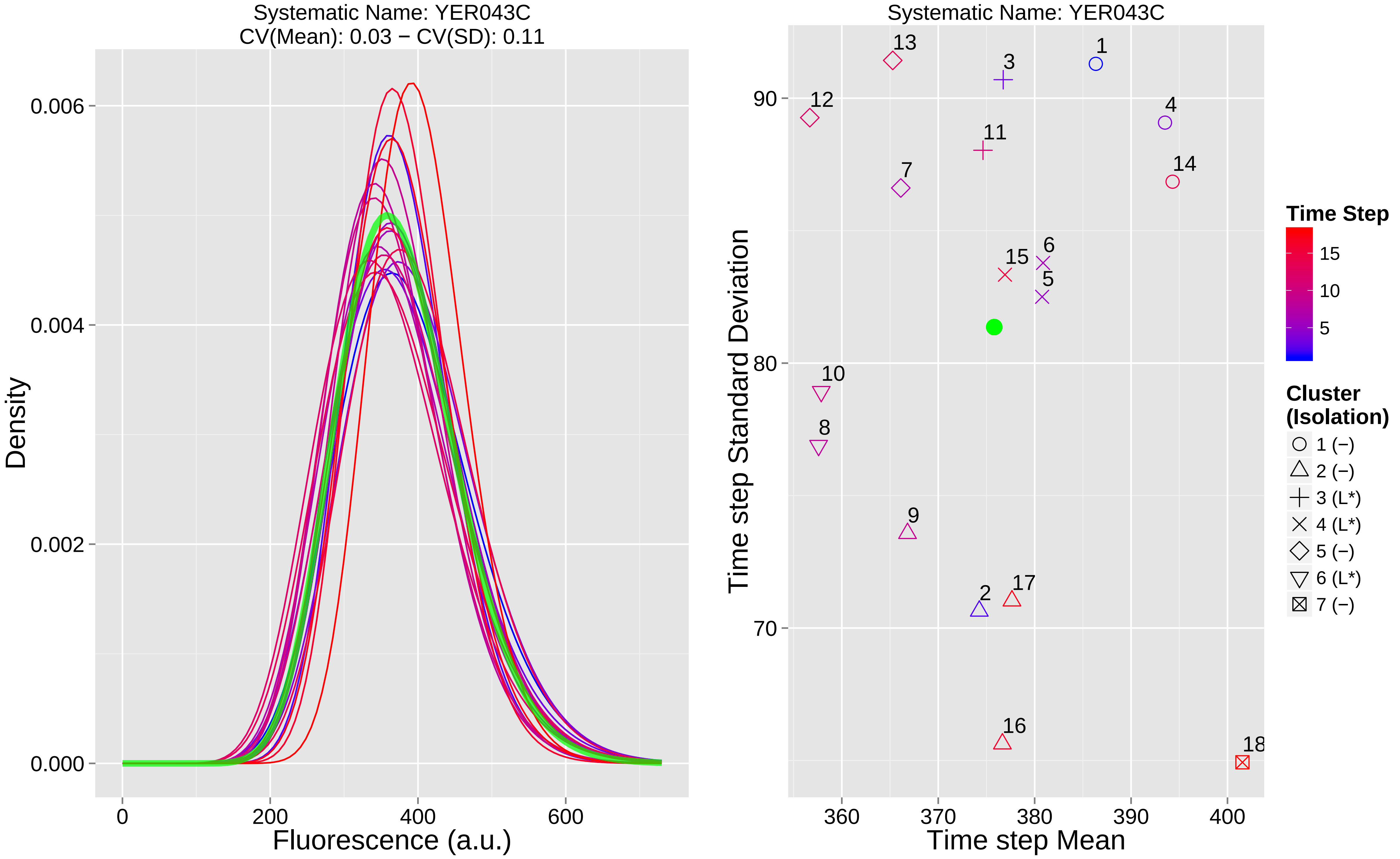

Systematic Name: YER043C
CV(Mean): 0.03 − CV(SD): 0.11
Density
Fluorescence (a.u.)
0.000
0.002
0.004
0.006
0
200
400
600
Systematic Name: YER043C
Time step Standard Deviation
Time step Mean
70
80
90
360
370
380
390
400
1
2
3
4
5
6
7
8
9
10
11
12
13
14
15
16
17
18
Time Step
15
10
5
Cluster
(Isolation)
1 (−)
2 (−)
3 (L*)
4 (L*)
5 (−)
6 (L*)
7 (−)

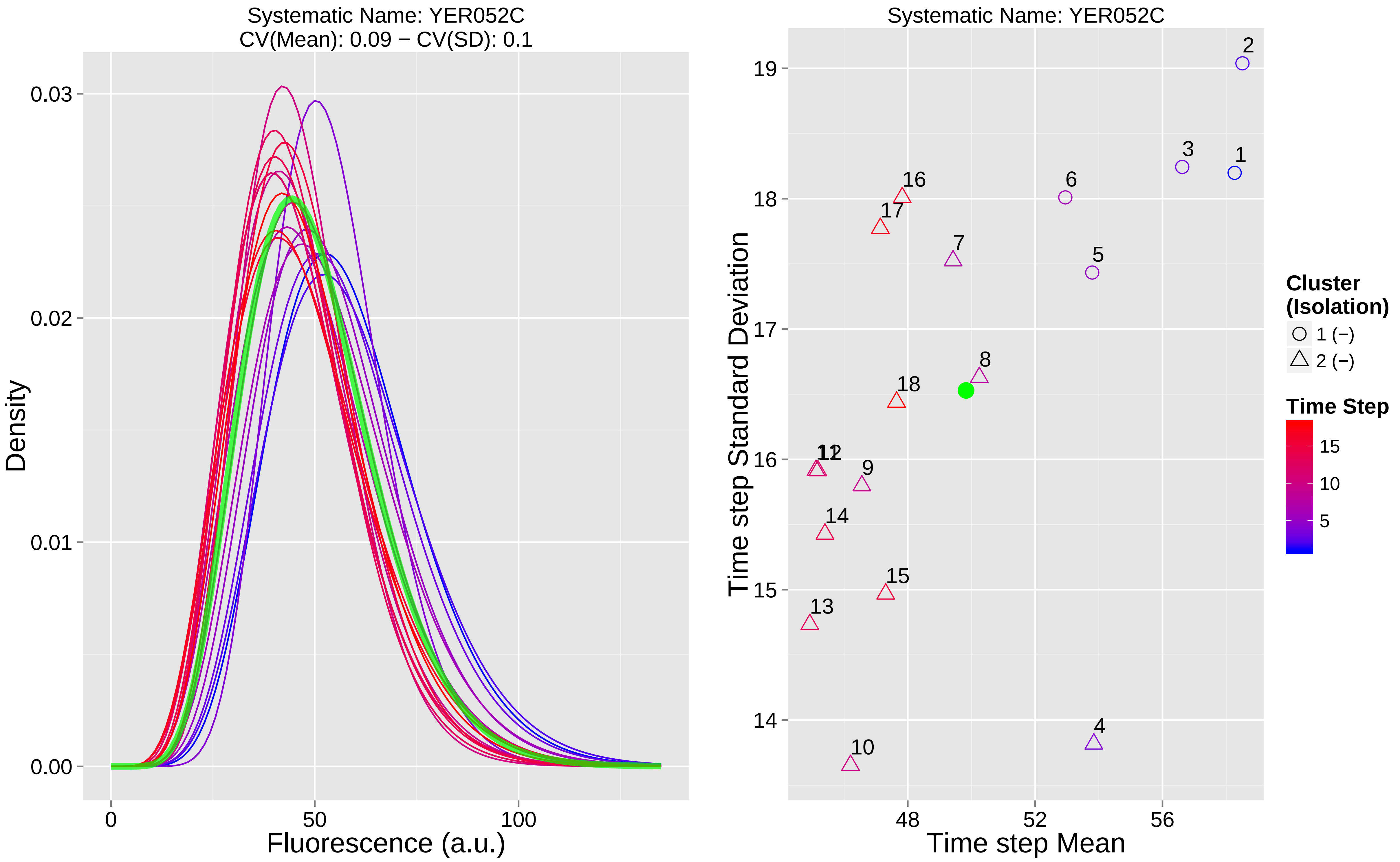

Systematic Name: YER052C
CV(Mean): 0.09 − CV(SD): 0.1
Density
0.03
0.02
0.01
0.00
0
50
100
Fluorescence (a.u.)
Systematic Name: YER052C
Time step Standard Deviation
19
18
17
16
15
14
48
52
56
Time step Mean
2
3
1
16
6
17
7
5
8
18
11
12
9
14
15
13
4
10
Cluster (Isolation)
1 (−)
2 (−)
Time Step
15
10
5

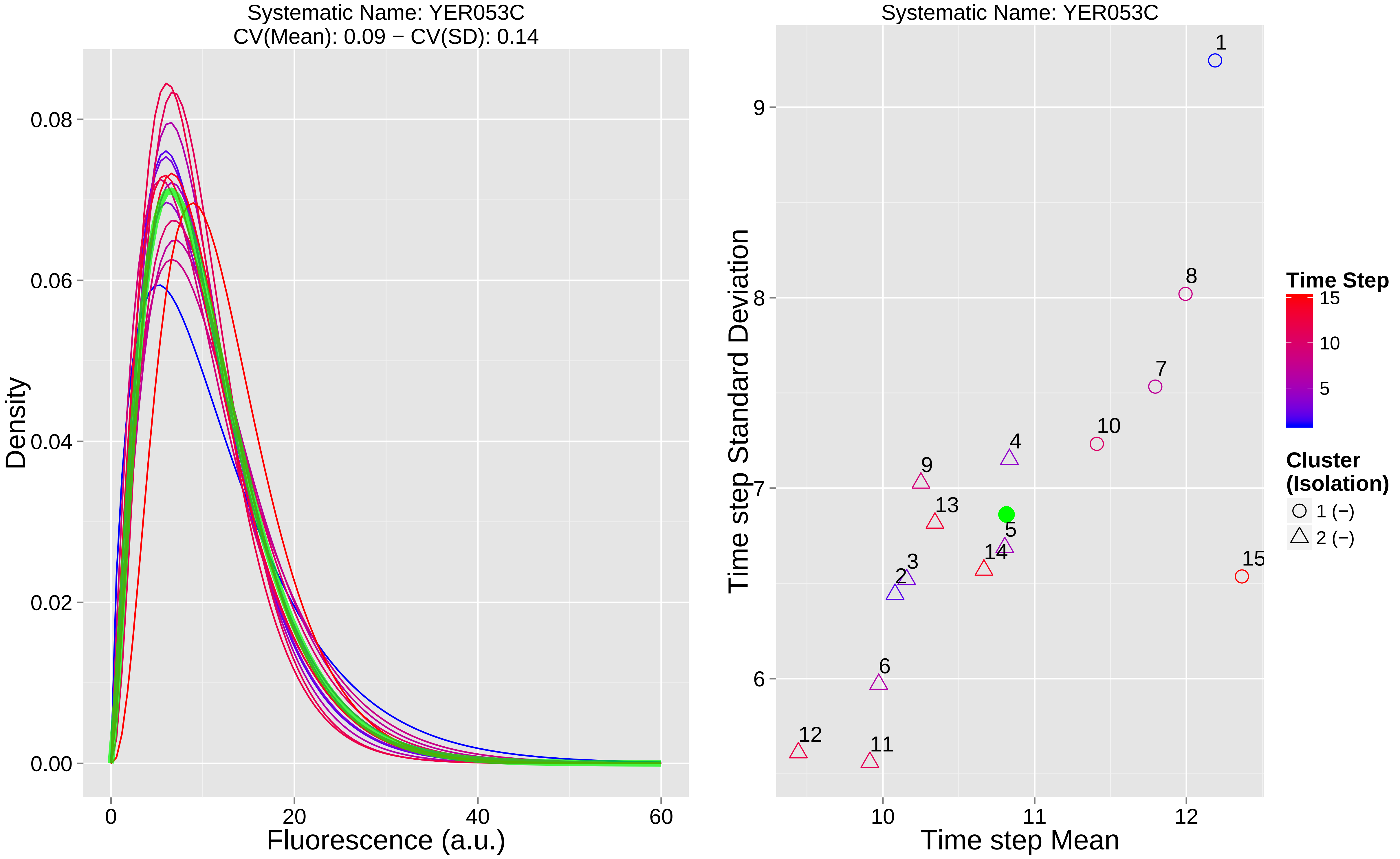

Systematic Name: YER053C
CV(Mean): 0.09 − CV(SD): 0.14
Density
Fluorescence (a.u.)
0.00
0.02
0.04
0.06
0.08
0
20
40
60
Systematic Name: YER053C
Time step Standard Deviation
Time step Mean
6
7
8
9
10
11
12
1
2
3
4
5
6
7
8
9
10
11
12
13
14
15
Time Step
15
10
5
Cluster (Isolation)
1 (−)
2 (−)

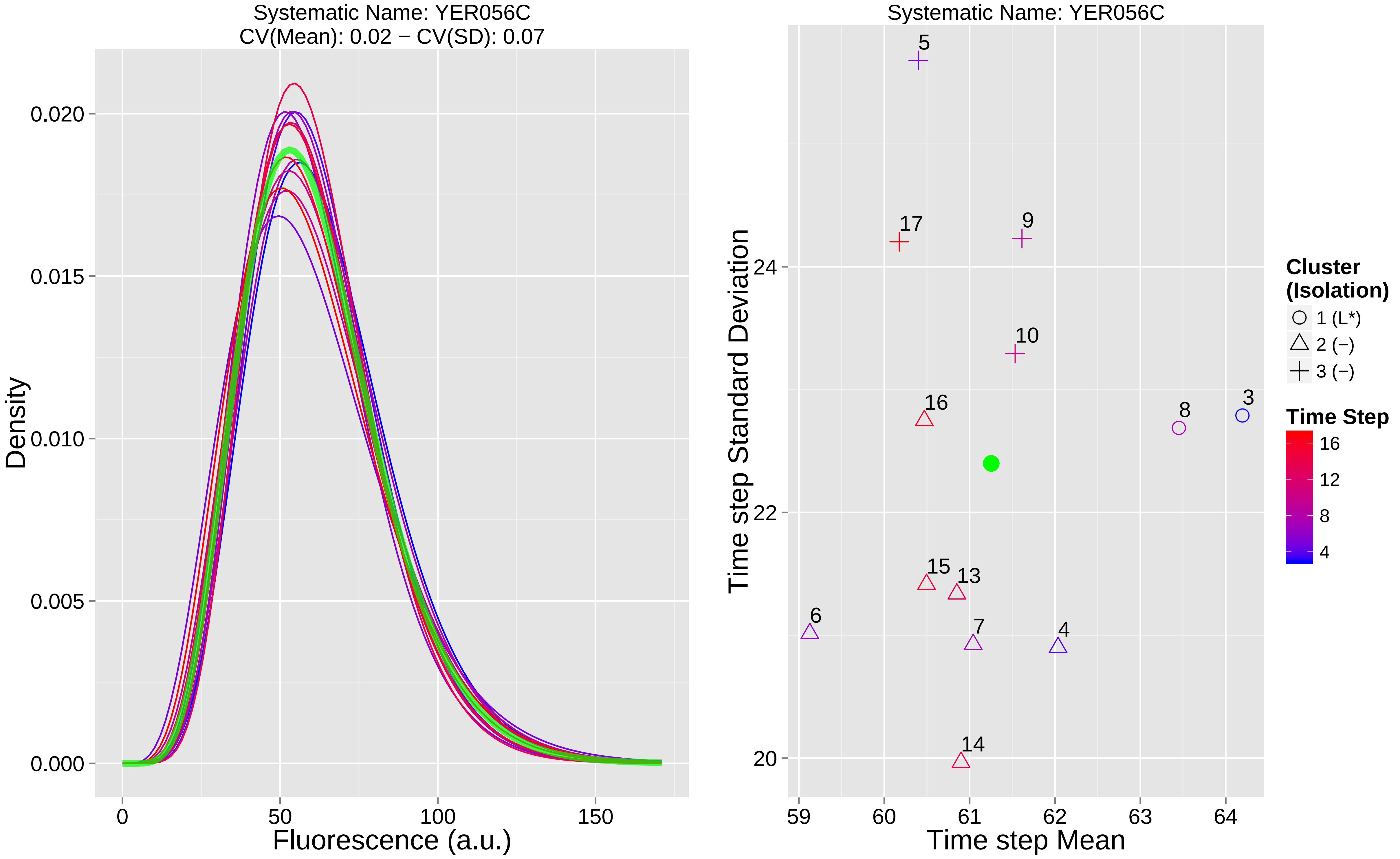
Systematic Name: YER056C
CV(Mean): 0.02 − CV(SD): 0.07
Density
0.020
0.015
0.010
0.005
0.000
0
50
100
150
Fluorescence (a.u.)
Systematic Name: YER056C
Time step Standard Deviation
24
22
20
59
60
61
62
63
64
Time step Mean
5
17
9
10
16
8
3
15
13
6
7
4
14
Cluster
(Isolation)
1 (L*)
2 (−)
3 (−)
Time Step
16
12
8
4

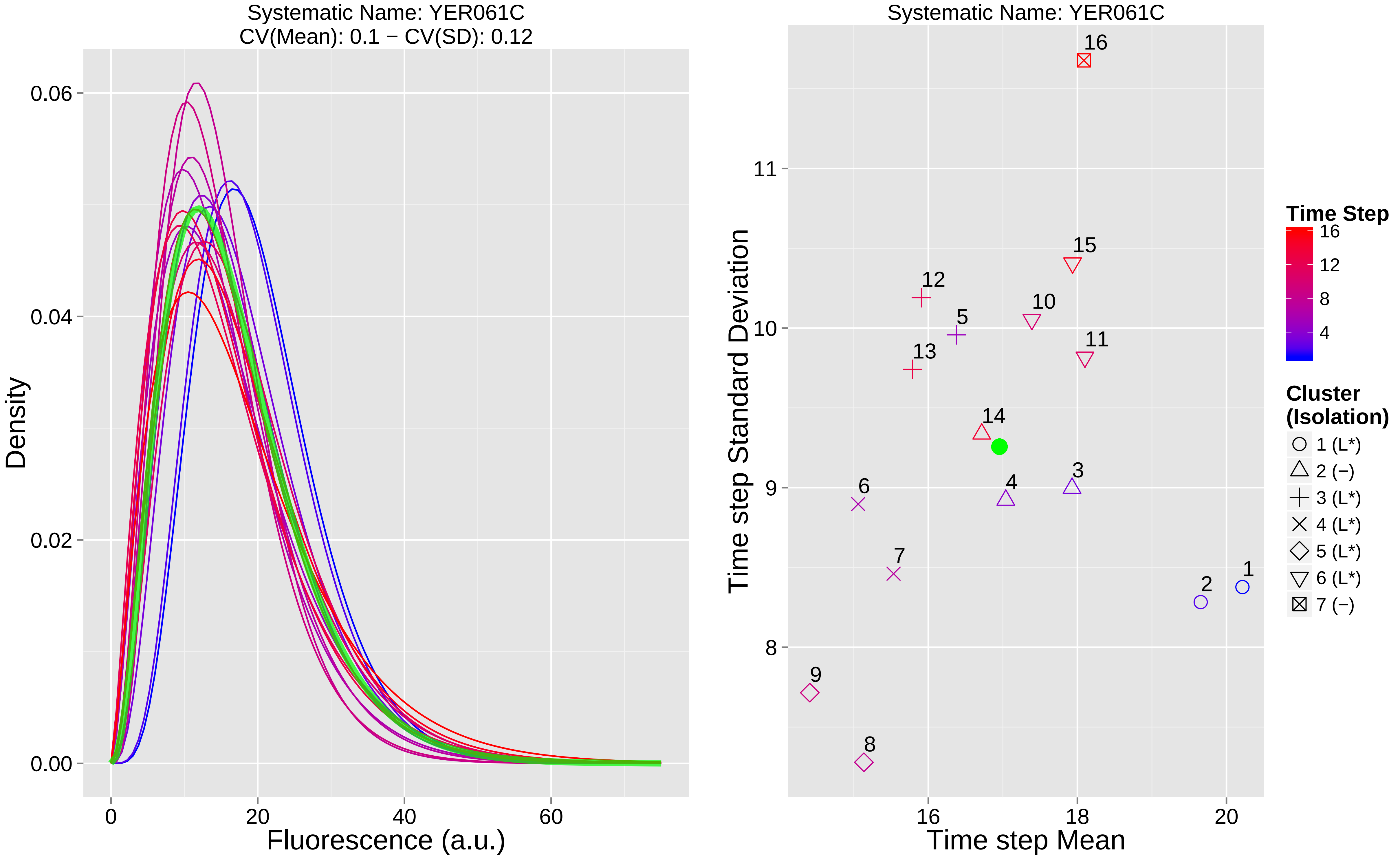

Systematic Name: YER061C
CV(Mean): 0.1 − CV(SD): 0.12
Density
0.06
0.04
0.02
0.00
0
20
40
60
Fluorescence (a.u.)
Systematic Name: YER061C
Time step Standard Deviation
11
10
9
8
16
18
20
Time step Mean
Time Step
16
12
8
4
Cluster (Isolation)
1 (L*)
2 (−)
3 (L*)
4 (L*)
5 (L*)
6 (L*)
7 (−)

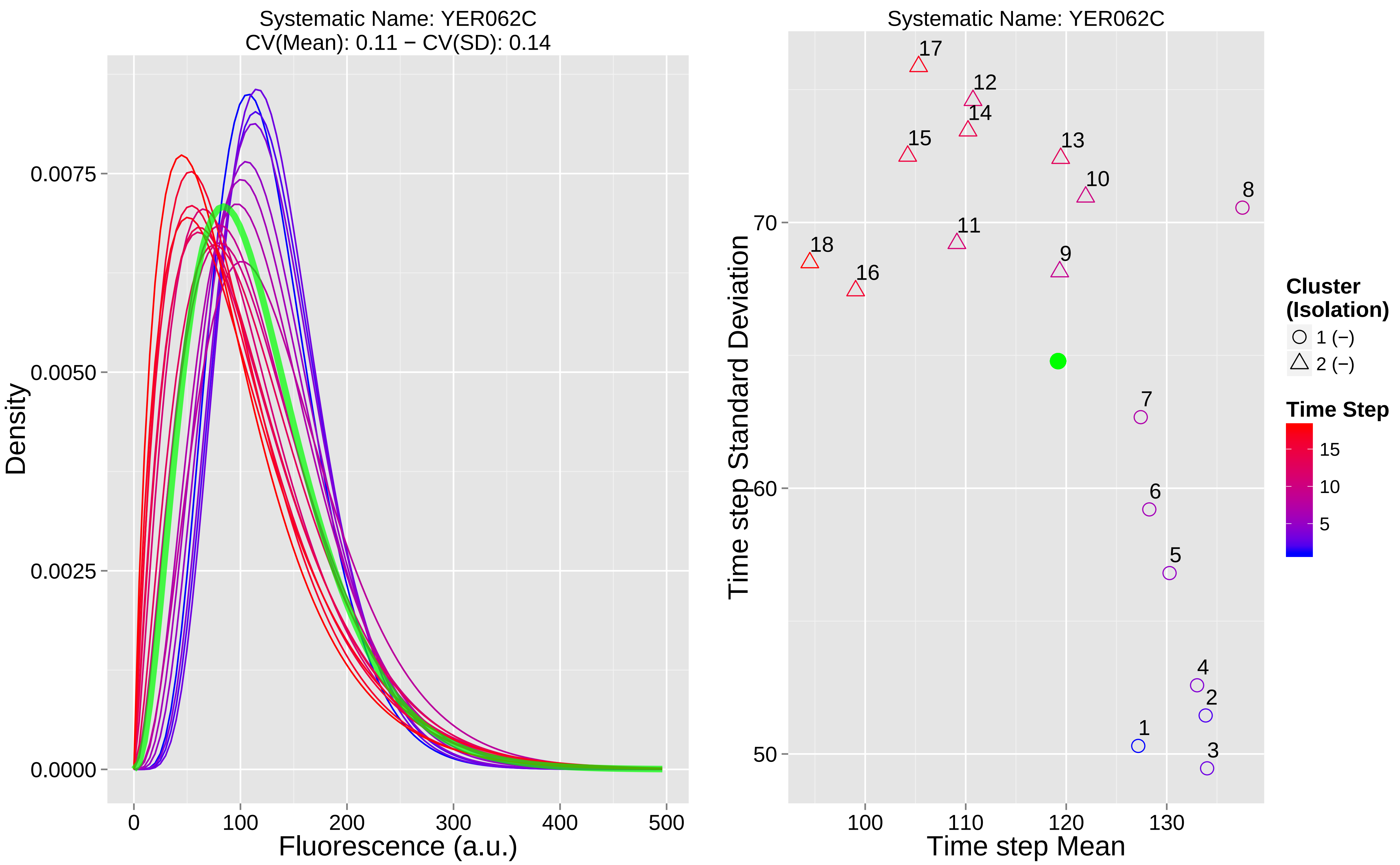

Systematic Name: YER062C
CV(Mean): 0.11 − CV(SD): 0.14
Density
0.0075
0.0050
0.0025
0.0000
0
100
200
300
400
500
Fluorescence (a.u.)
Systematic Name: YER062C
Time step Standard Deviation
70
60
50
100
110
120
130
Time step Mean
17
12
14
15
13
10
8
11
18
9
16
7
6
5
4
2
1
3
Cluster (Isolation)
1 (−)
2 (−)
Time Step
15
10
5

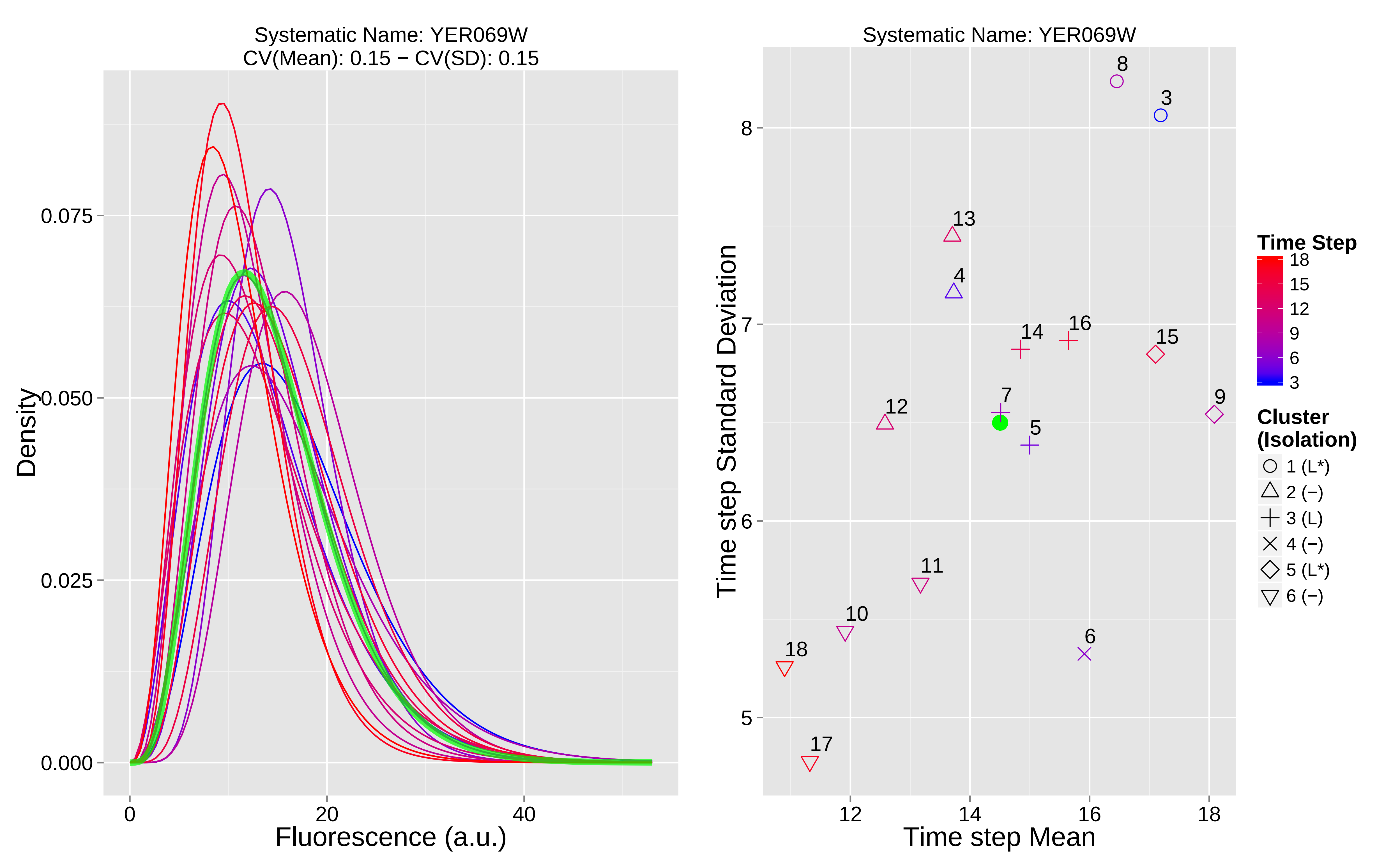

Systematic Name: YER069W
CV(Mean): 0.15 − CV(SD): 0.15
Density
0.000
0.025
0.050
0.075
0
20
40
Fluorescence (a.u.)
Systematic Name: YER069W
Time step Standard Deviation
5
6
7
8
12
14
16
18
Time step Mean
8
3
13
4
14
16
15
12
7
5
9
11
10
18
6
17
Time Step
18
15
12
9
6
3
Cluster
(Isolation)
1 (L*)
2 (−)
3 (L)
4 (−)
5 (L*)
6 (−)

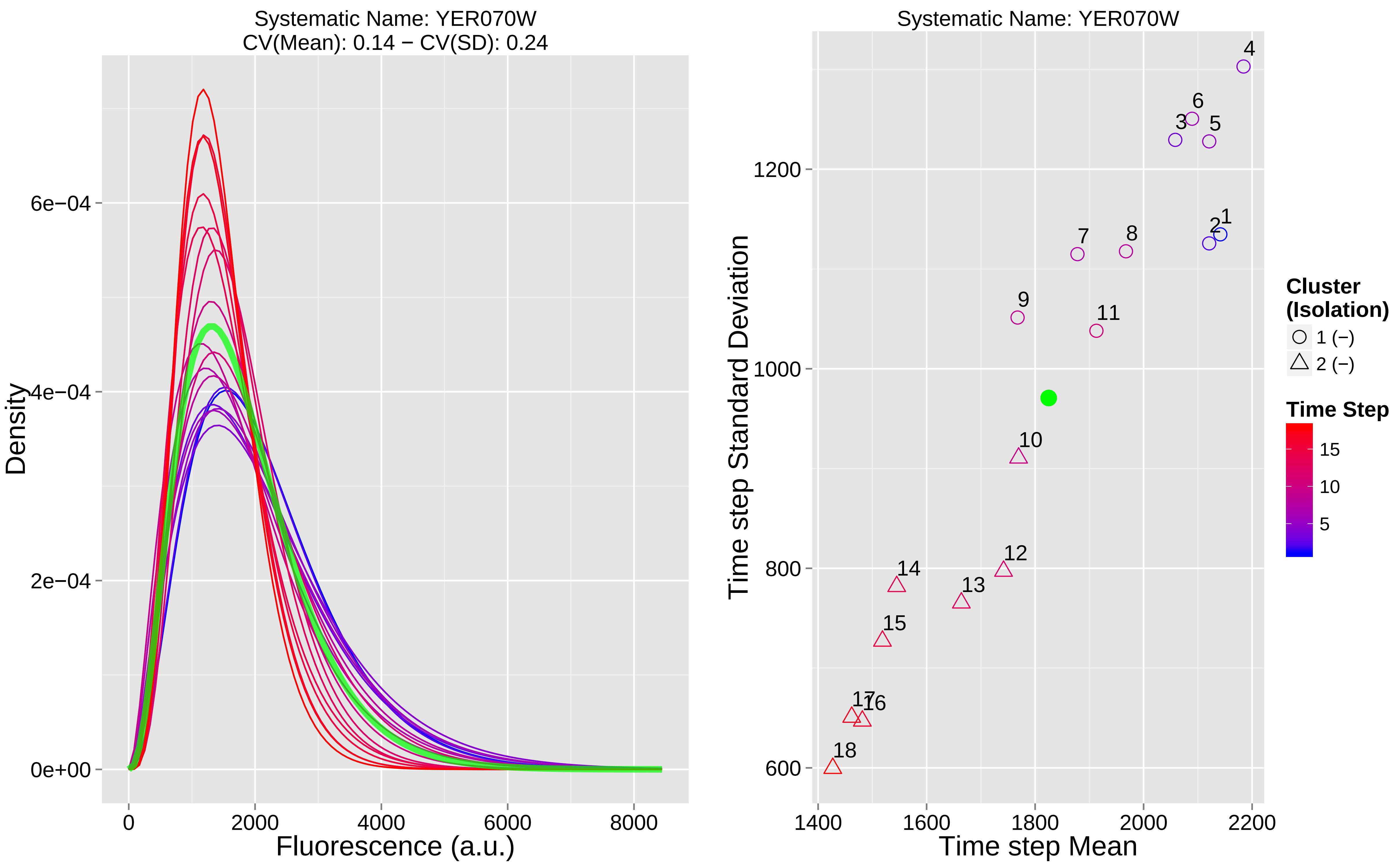
Systematic Name: YER070W
CV(Mean): 0.14 − CV(SD): 0.24
Density
6e−04
4e−04
2e−04
0e+00
0
2000
4000
6000
8000
Fluorescence (a.u.)
Systematic Name: YER070W
Time step Standard Deviation
1200
1000
800
600
1400
1600
1800
2000
2200
Time step Mean
Cluster
(Isolation)
1 (−)
2 (−)
Time Step
15
10
5

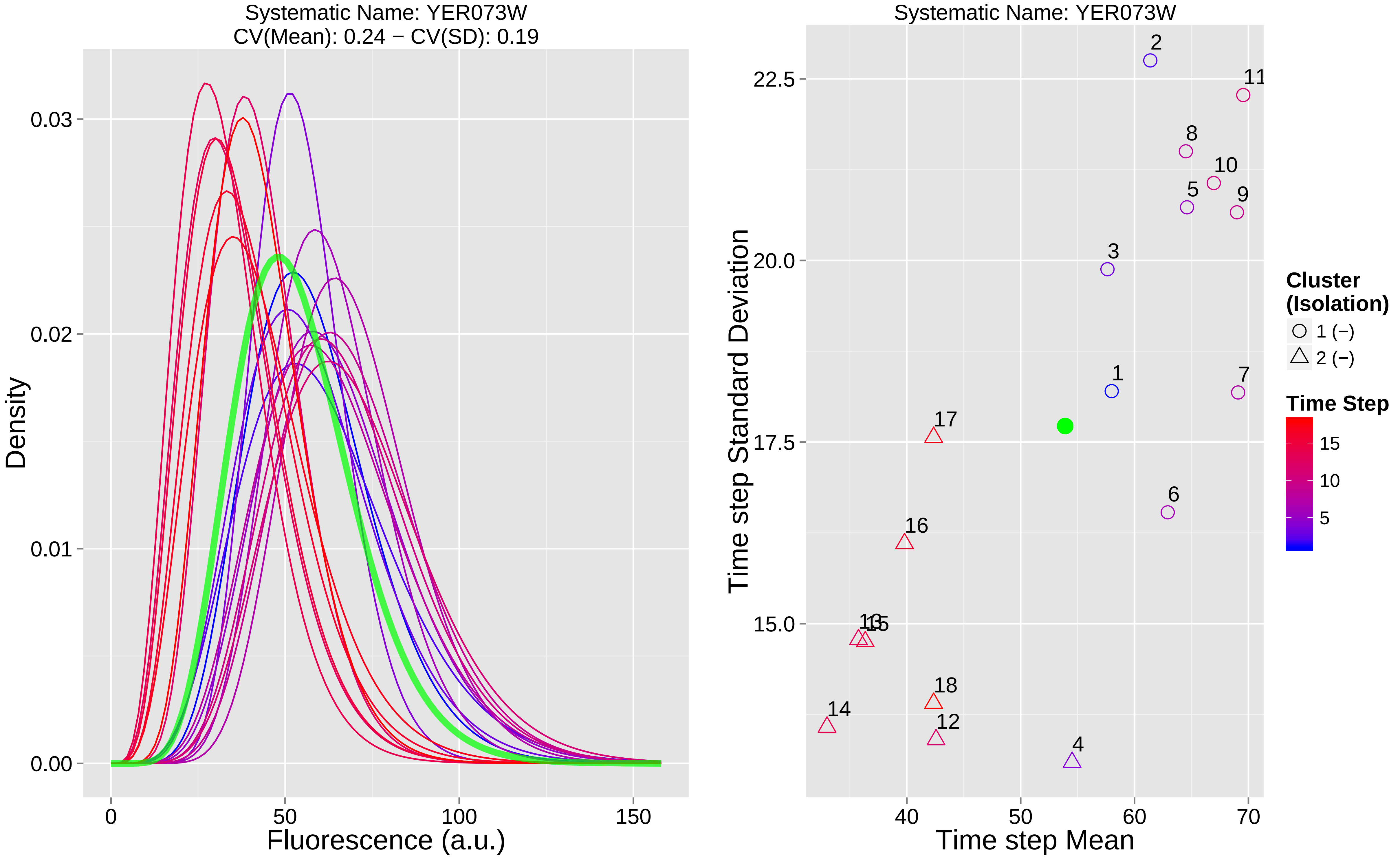
Systematic Name: YER073W
CV(Mean): 0.24 − CV(SD): 0.19
Density
0.03
0.02
0.01
0.00
0
50
100
150
Fluorescence (a.u.)
Systematic Name: YER073W
Time step Standard Deviation
22.5
20.0
17.5
15.0
40
50
60
70
Time step Mean
Cluster
(Isolation)
1 (−)
2 (−)
Time Step
15
10
5

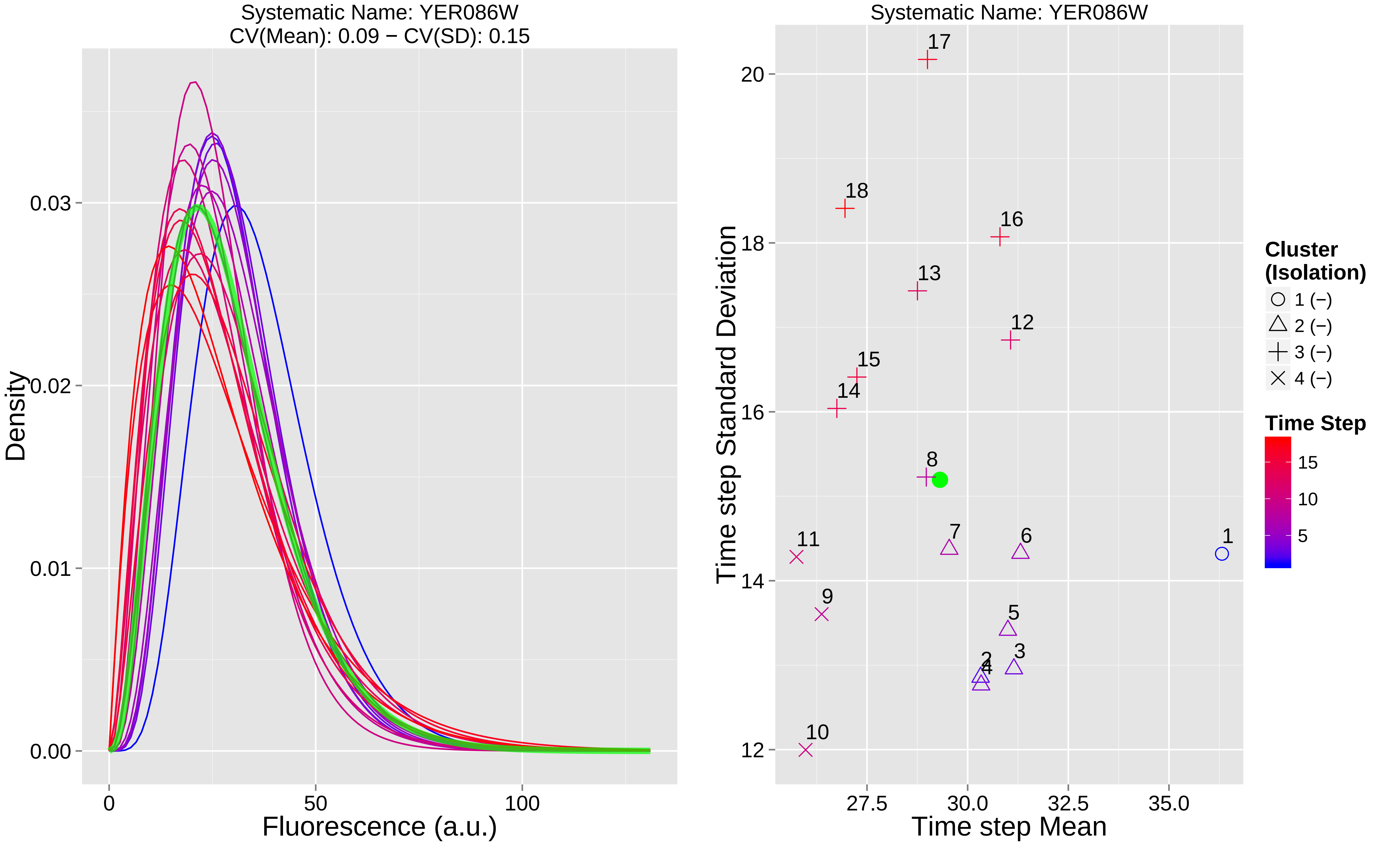

Systematic Name: YER086W
CV(Mean): 0.09 − CV(SD): 0.15
Density
Fluorescence (a.u.)
0.00
0.01
0.02
0.03
0
50
100
Systematic Name: YER086W
Time step Standard Deviation
Time step Mean
12
14
16
18
20
27.5
30.0
32.5
35.0
1
2
3
4
5
6
7
8
9
10
11
12
13
14
15
16
17
18
Cluster (Isolation)
1 (−)
2 (−)
3 (−)
4 (−)
Time Step
5
10
15

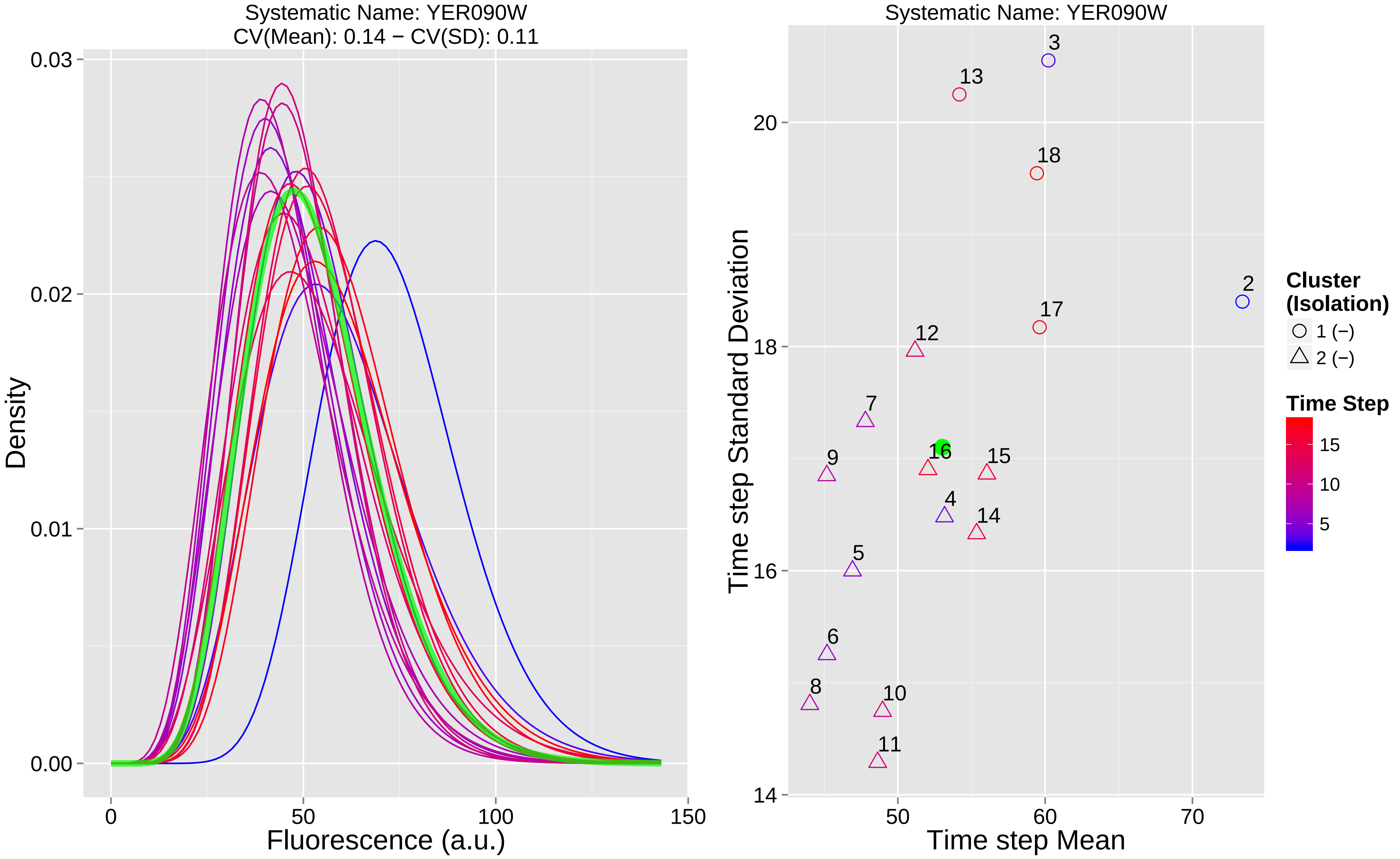

Systematic Name: YER090W
CV(Mean): 0.14 − CV(SD): 0.11
Density
Fluorescence (a.u.)
0.00
0.01
0.02
0.03
0
50
100
150
Systematic Name: YER090W
Time step Standard Deviation
Time step Mean
14
16
18
20
50
60
70
Cluster
(Isolation)
1 (−)
2 (−)
Time Step
5
10
15

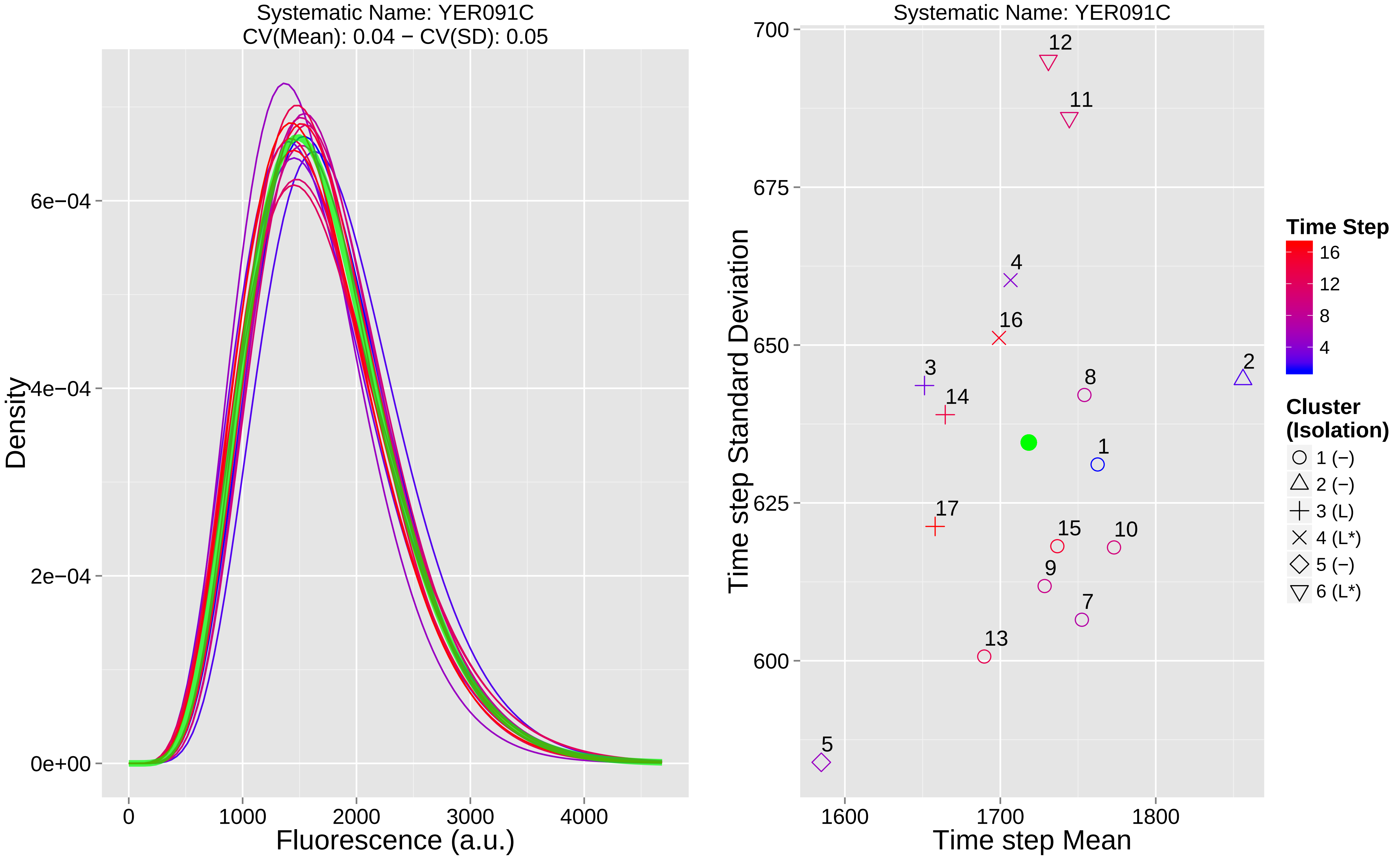

Systematic Name: YER091C
CV(Mean): 0.04 − CV(SD): 0.05
Density
6e−04
4e−04
2e−04
0e+00
0
1000
2000
3000
4000
Fluorescence (a.u.)
Systematic Name: YER091C
Time step Standard Deviation
700
675
650
625
600
1600
1700
1800
Time step Mean
12
11
4
16
3
14
8
2
1
17
15
10
9
7
13
5
Time Step
16
12
8
4
Cluster (Isolation)
1 (−)
2 (−)
3 (L)
4 (L*)
5 (−)
6 (L*)

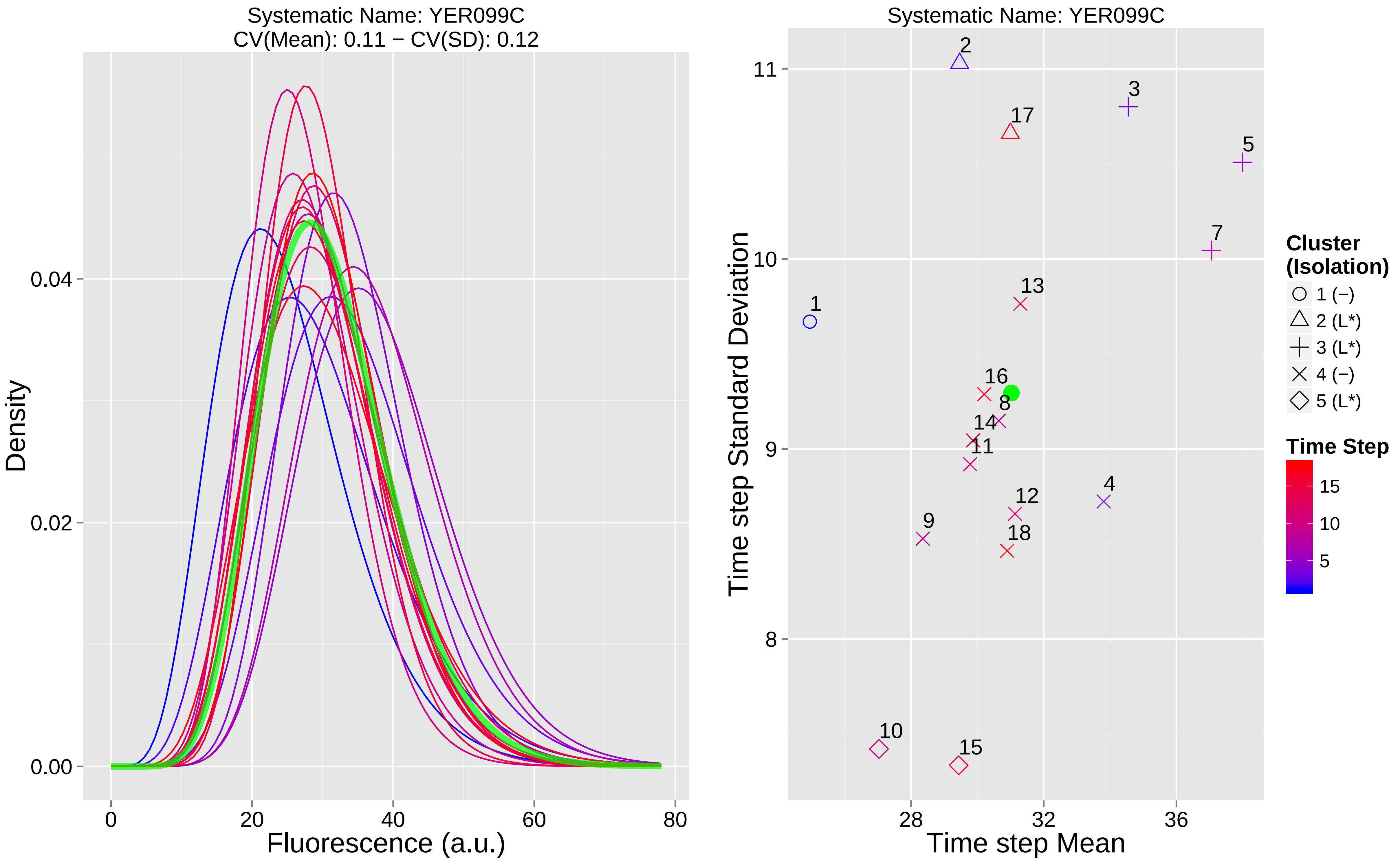

Systematic Name: YER099C
CV(Mean): 0.11 − CV(SD): 0.12
Density
0.04
0.02
0.00
0
20
40
60
80
Fluorescence (a.u.)
Systematic Name: YER099C
Time step Standard Deviation
11
10
9
8
28
32
36
Time step Mean
1
2
3
4
5
7
8
9
10
11
12
13
14
15
16
17
18
Cluster (Isolation)
1 (−)
2 (L*)
3 (L*)
4 (−)
5 (L*)
Time Step
15
10
5

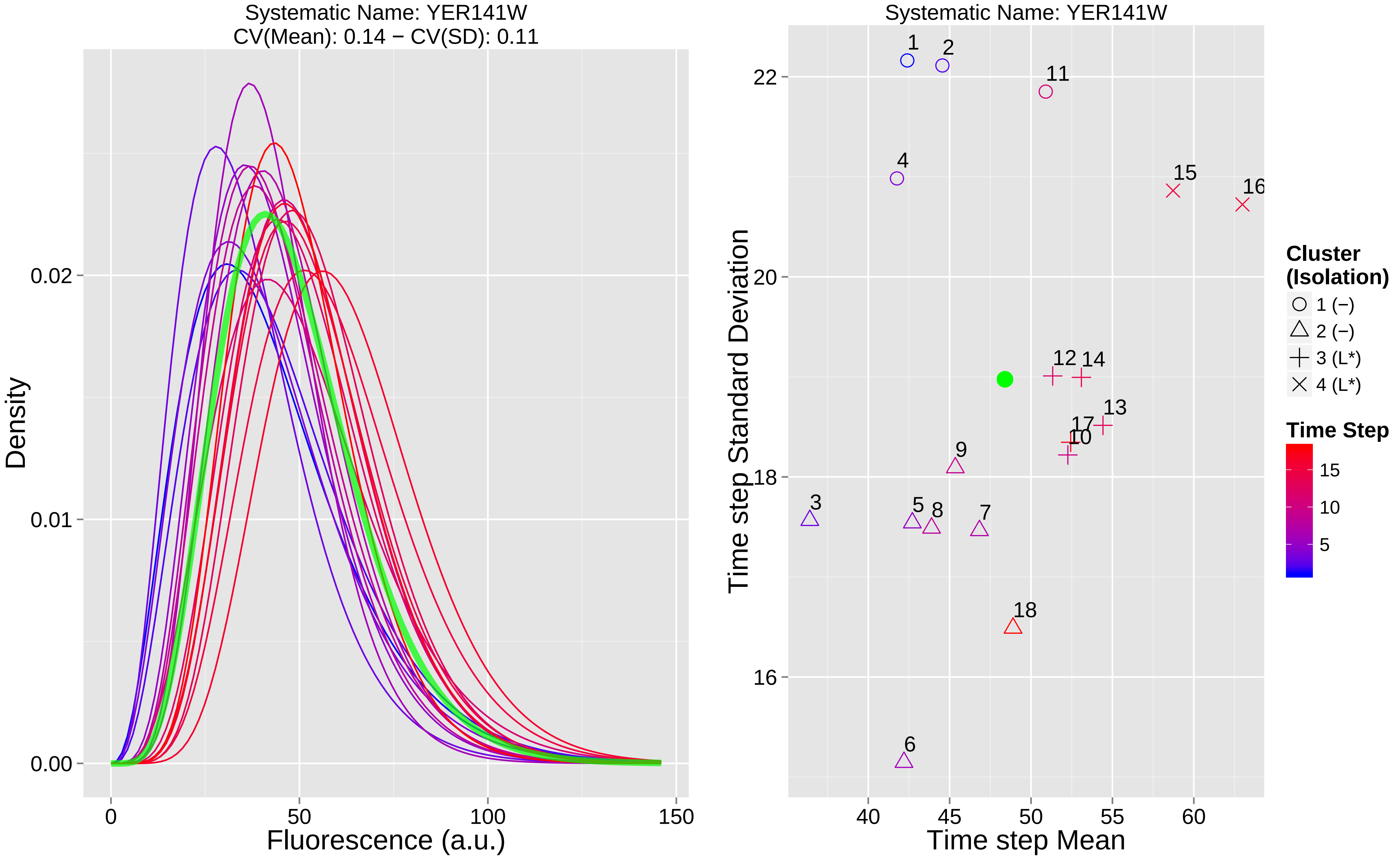

Systematic Name: YER141W
CV(Mean): 0.14 − CV(SD): 0.11
Density
0.00
0.01
0.02
0
50
100
150
Fluorescence (a.u.)
Systematic Name: YER141W
Time step Standard Deviation
16
18
20
22
40
45
50
55
60
Time step Mean
1
2
3
4
5
6
7
8
9
10
11
12
13
14
15
16
17
18
Cluster (Isolation)
1 (−)
2 (−)
3 (L*)
4 (L*)
Time Step
5
10
15

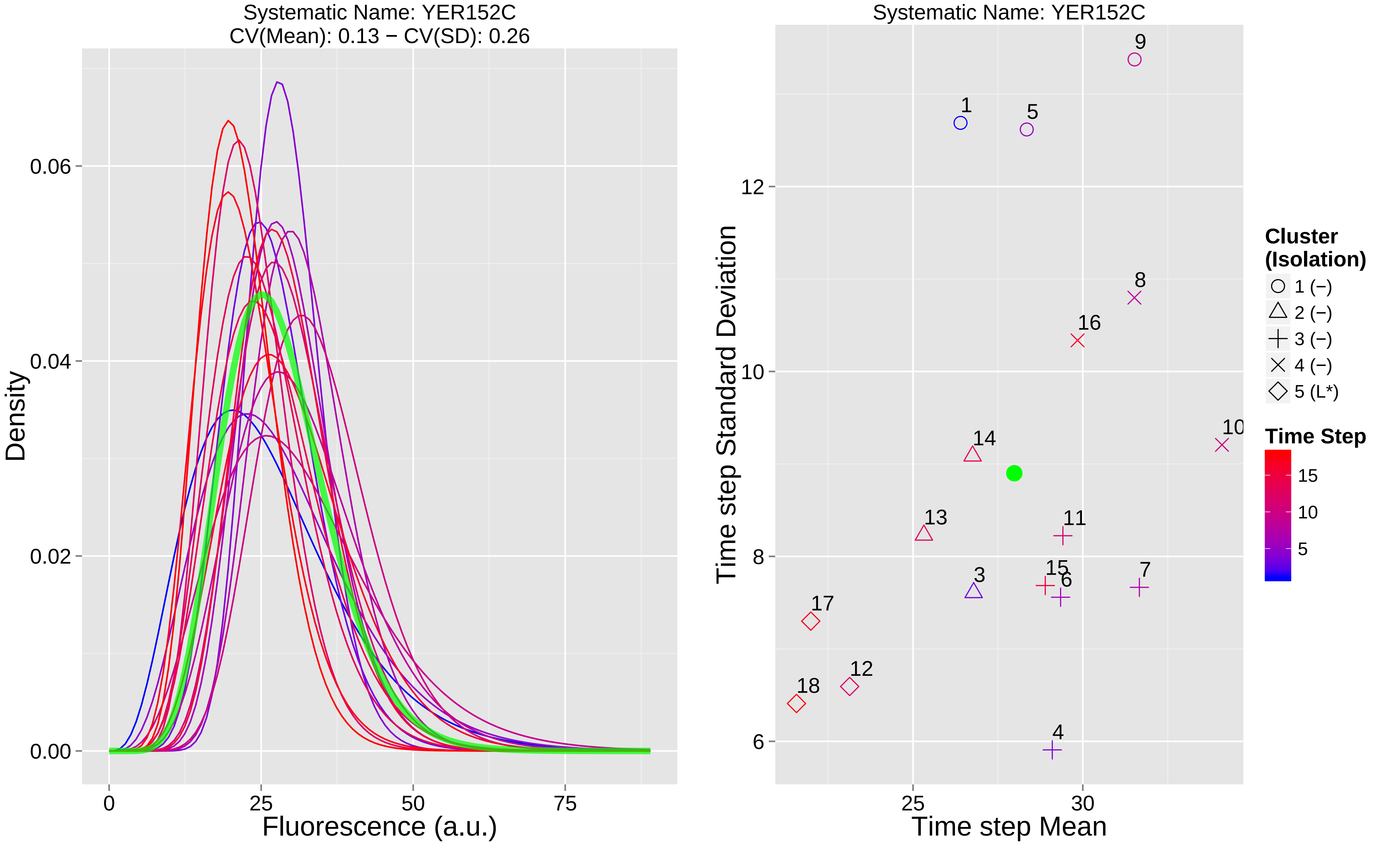
Systematic Name: YER152C
CV(Mean): 0.13 − CV(SD): 0.26
Density
0.06
0.04
0.02
0.00
0
25
50
75
Fluorescence (a.u.)
Systematic Name: YER152C
Time step Standard Deviation
12
10
8
6
25
30
Time step Mean
1
2
3
4
5
6
7
8
9
10
11
12
13
14
15
16
17
18
Cluster
(Isolation)
1 (−)
2 (−)
3 (−)
4 (−)
5 (L*)
Time Step
15
10
5

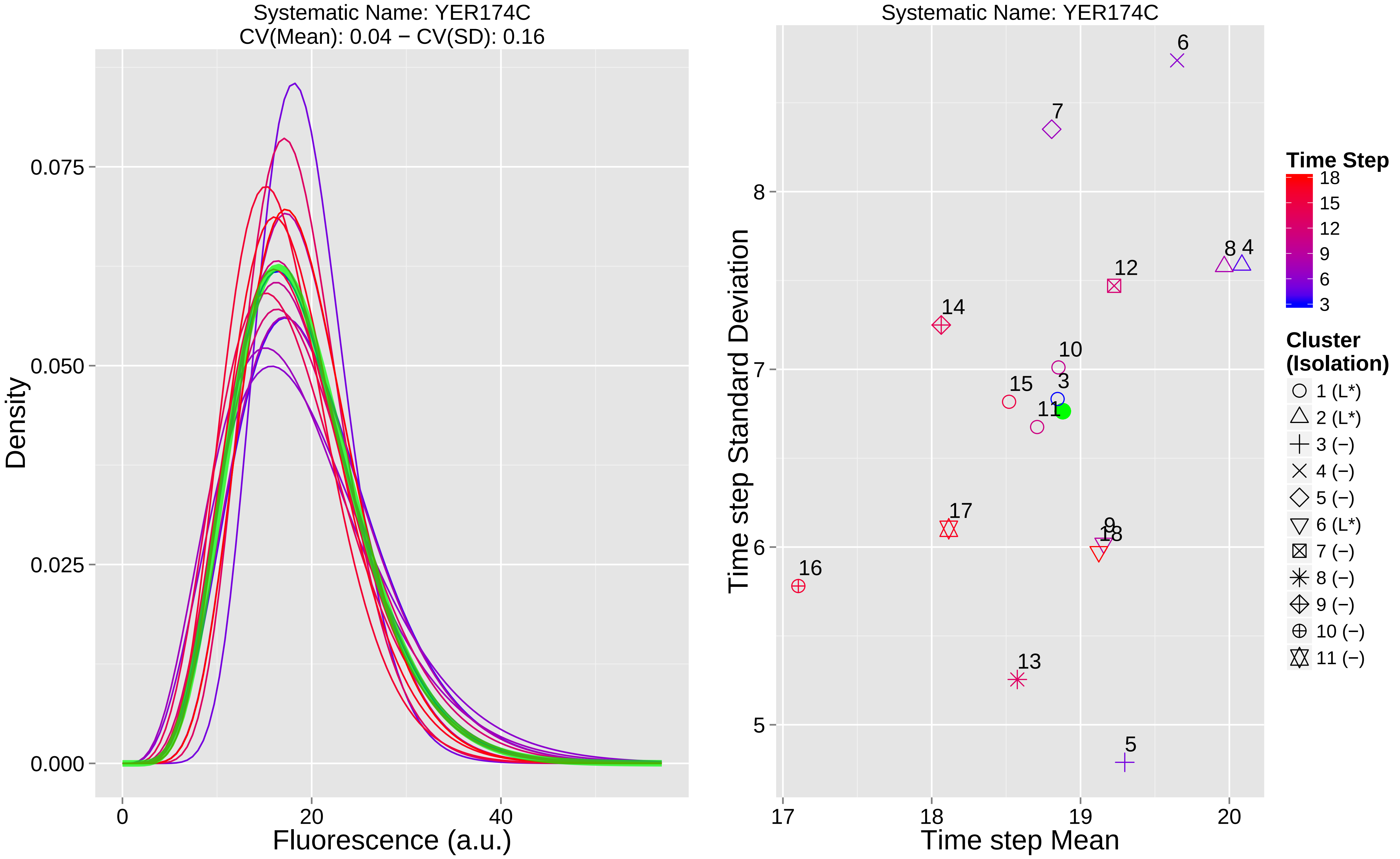

Systematic Name: YER174C
CV(Mean): 0.04 − CV(SD): 0.16
Density
0.075
0.050
0.025
0.000
0
20
40
Fluorescence (a.u.)
Systematic Name: YER174C
Time step Standard Deviation
8
7
6
5
17
18
19
20
Time step Mean
6
7
8
4
12
14
10
15
3
11
17
9
18
16
13
5
Time Step
18
15
12
9
6
3
Cluster (Isolation)
1 (L*)
2 (L*)
3 (−)
4 (−)
5 (−)
6 (L*)
7 (−)
8 (−)
9 (−)
10 (−)
11 (−)

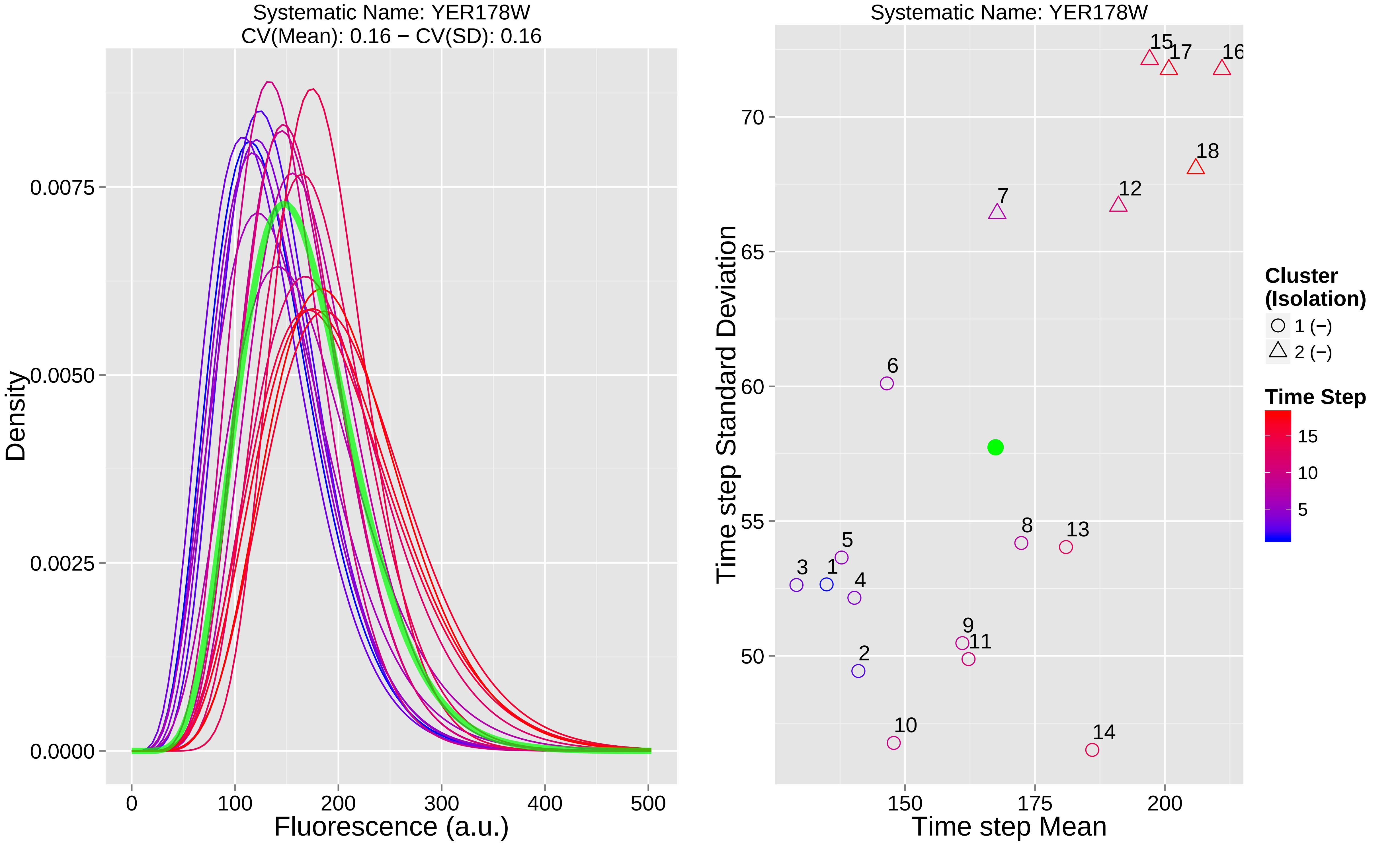

Systematic Name: YER178W
CV(Mean): 0.16 − CV(SD): 0.16
Density
0.0075
0.0050
0.0025
0.0000
0
100
200
300
400
500
Fluorescence (a.u.)
Systematic Name: YER178W
Time step Standard Deviation
70
65
60
55
50
150
175
200
Time step Mean
15
17
16
18
7
12
6
8
13
5
3
1
4
9
11
2
10
14
Cluster
(Isolation)
1 (−)
2 (−)
Time Step
15
10
5

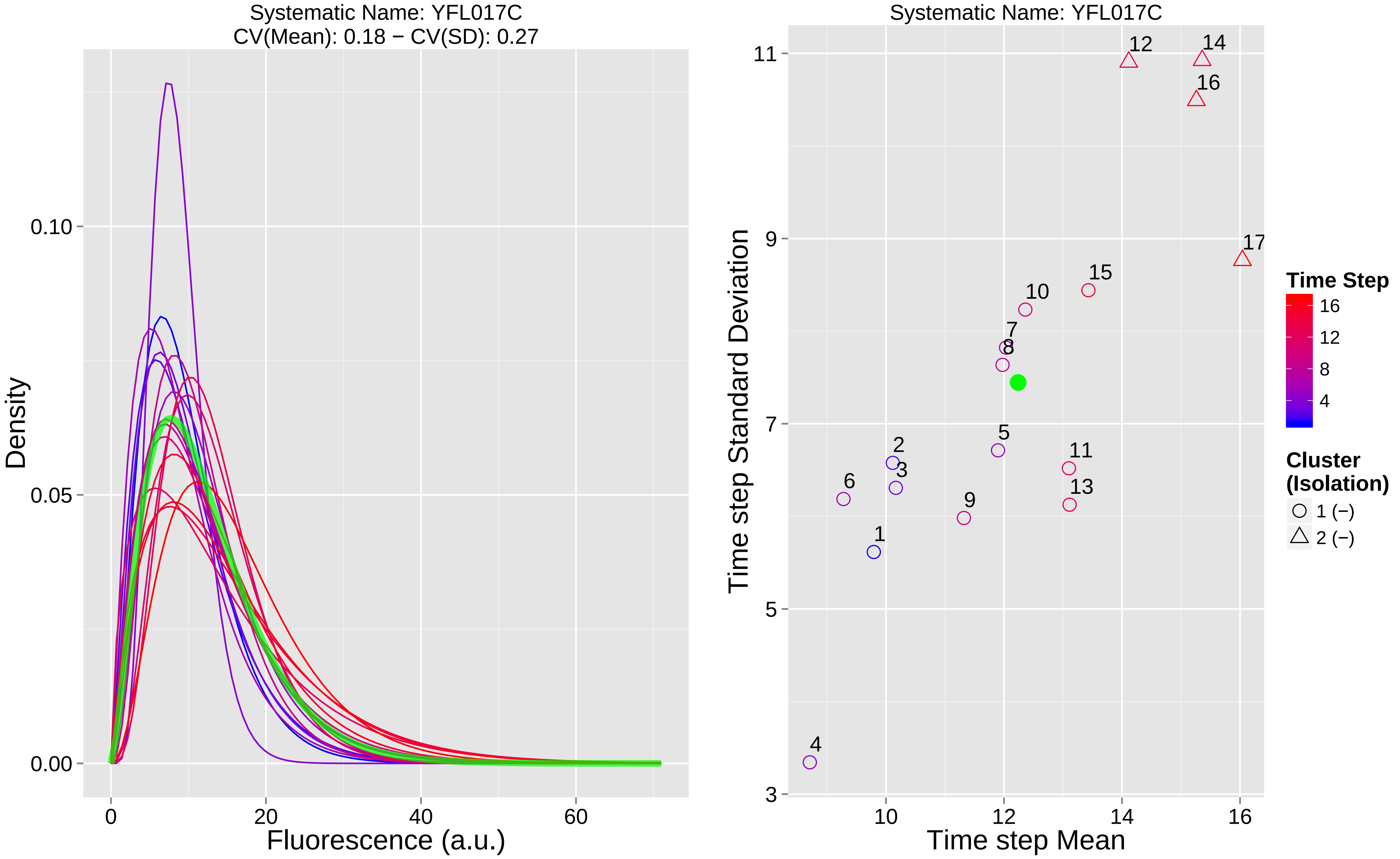

Systematic Name: YFL017C
CV(Mean): 0.18 − CV(SD): 0.27
Density
0.10
0.05
0.00
0
20
40
60
Fluorescence (a.u.)
Systematic Name: YFL017C
Time step Standard Deviation
11
9
7
5
3
10
12
14
16
Time step Mean
Time Step
16
12
8
4
Cluster
(Isolation)
1 (−)
2 (−)

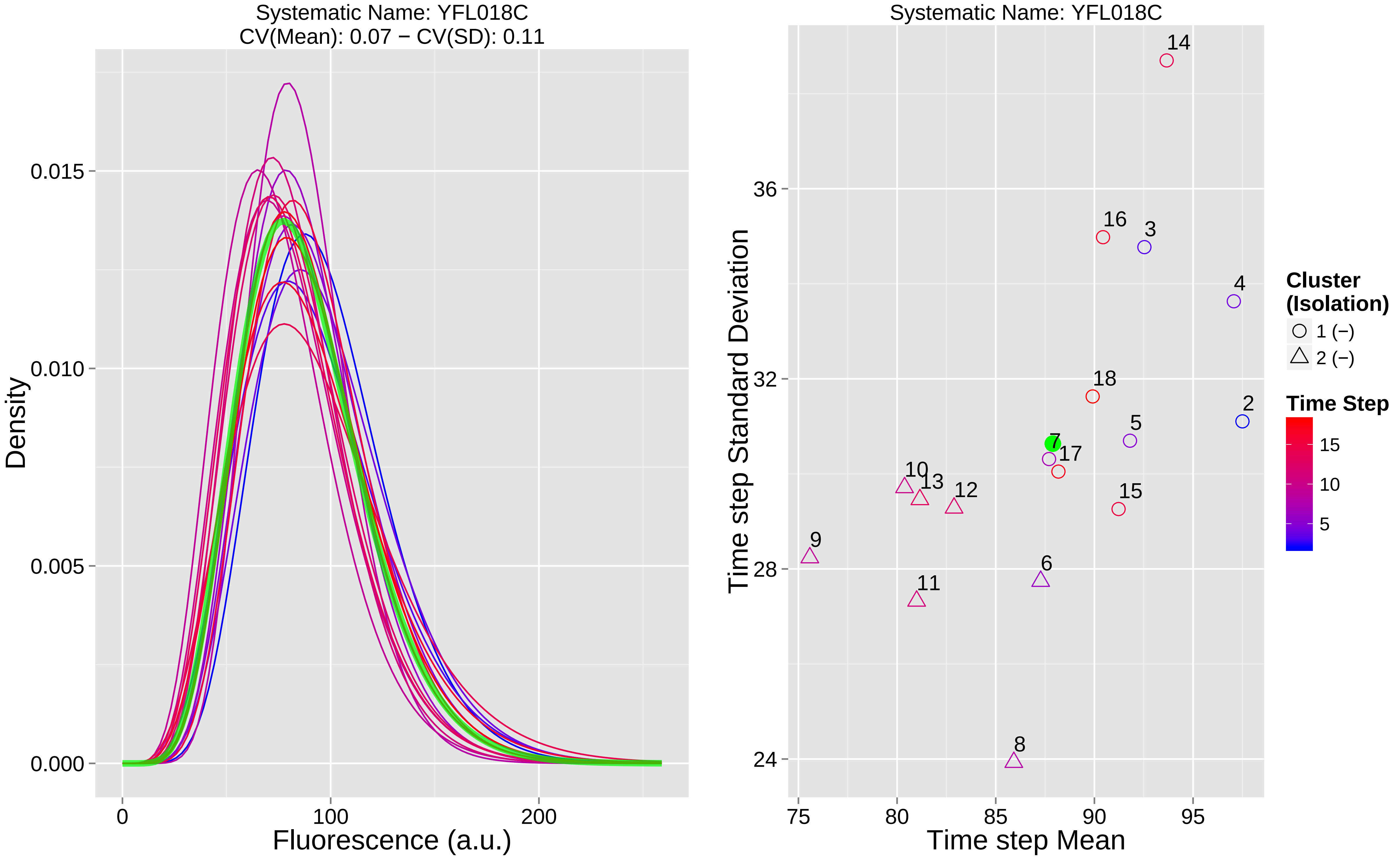

Systematic Name: YFL018C
CV(Mean): 0.07 − CV(SD): 0.11
Density
0.015
0.010
0.005
0.000
0
100
200
Fluorescence (a.u.)
Systematic Name: YFL018C
Time step Standard Deviation
36
32
28
24
75
80
85
90
95
Time step Mean
14
16
3
4
18
2
5
7
17
10
13
12
15
9
6
11
8
Cluster
(Isolation)
1 (−)
2 (−)
Time Step
15
10
5

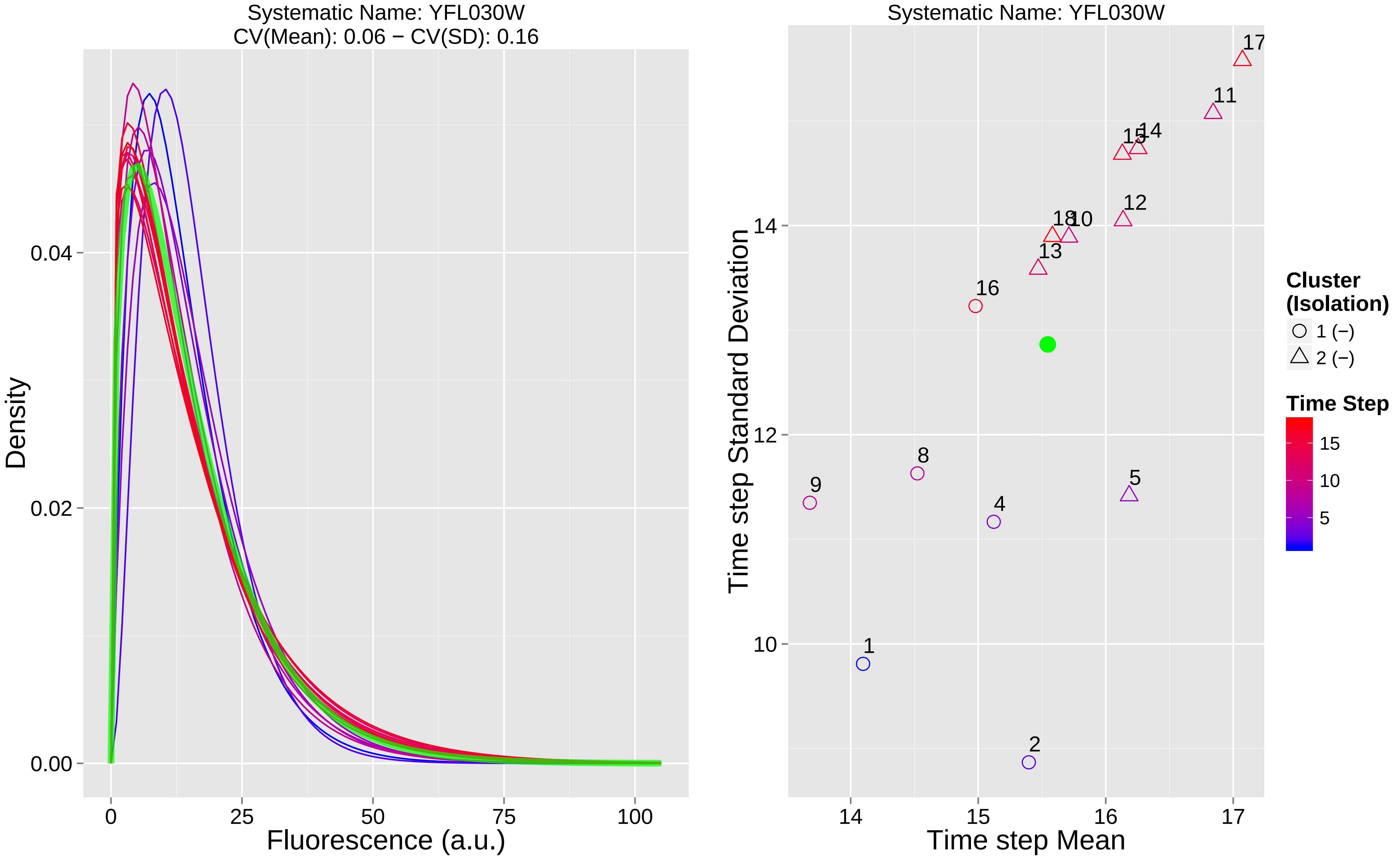

Systematic Name: YFL030W
CV(Mean): 0.06 − CV(SD): 0.16
Density
Fluorescence (a.u.)
0.00
0.02
0.04
0
25
50
75
100
Systematic Name: YFL030W
Time step Standard Deviation
Time step Mean
10
12
14
14
15
16
17
1
2
4
5
8
9
10
11
12
13
14
15
16
17
18
Cluster
(Isolation)
1 (−)
2 (−)
Time Step
5
10
15

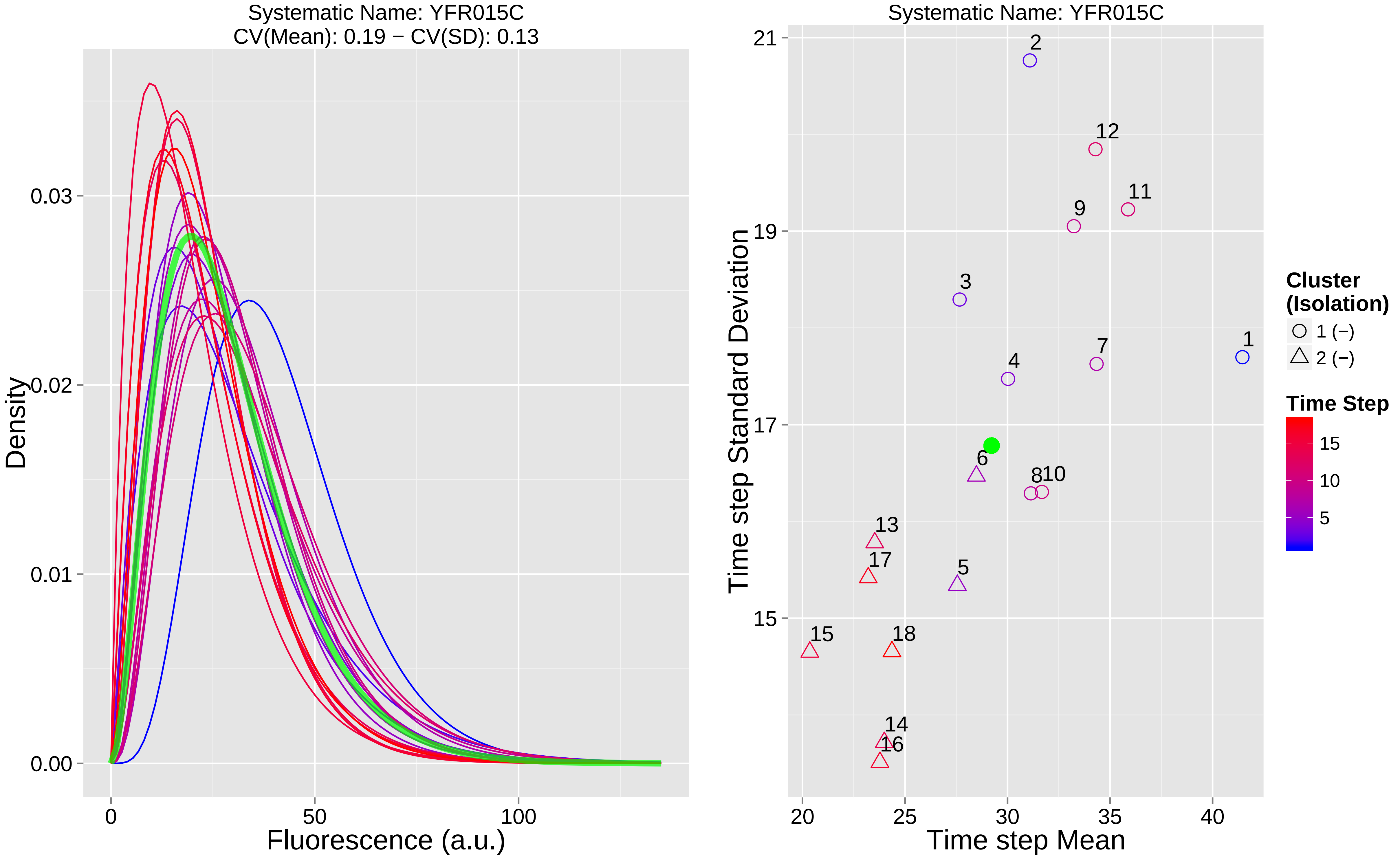

Systematic Name: YFR015C
CV(Mean): 0.19 − CV(SD): 0.13
Density
0.00
0.01
0.02
0.03
0
50
100
Fluorescence (a.u.)
Systematic Name: YFR015C
Time step Standard Deviation
15
17
19
21
20
25
30
35
40
Time step Mean
1
2
3
4
5
6
7
8
9
10
11
12
13
14
15
16
17
18
Cluster
(Isolation)
1 (−)
2 (−)
Time Step
5
10
15

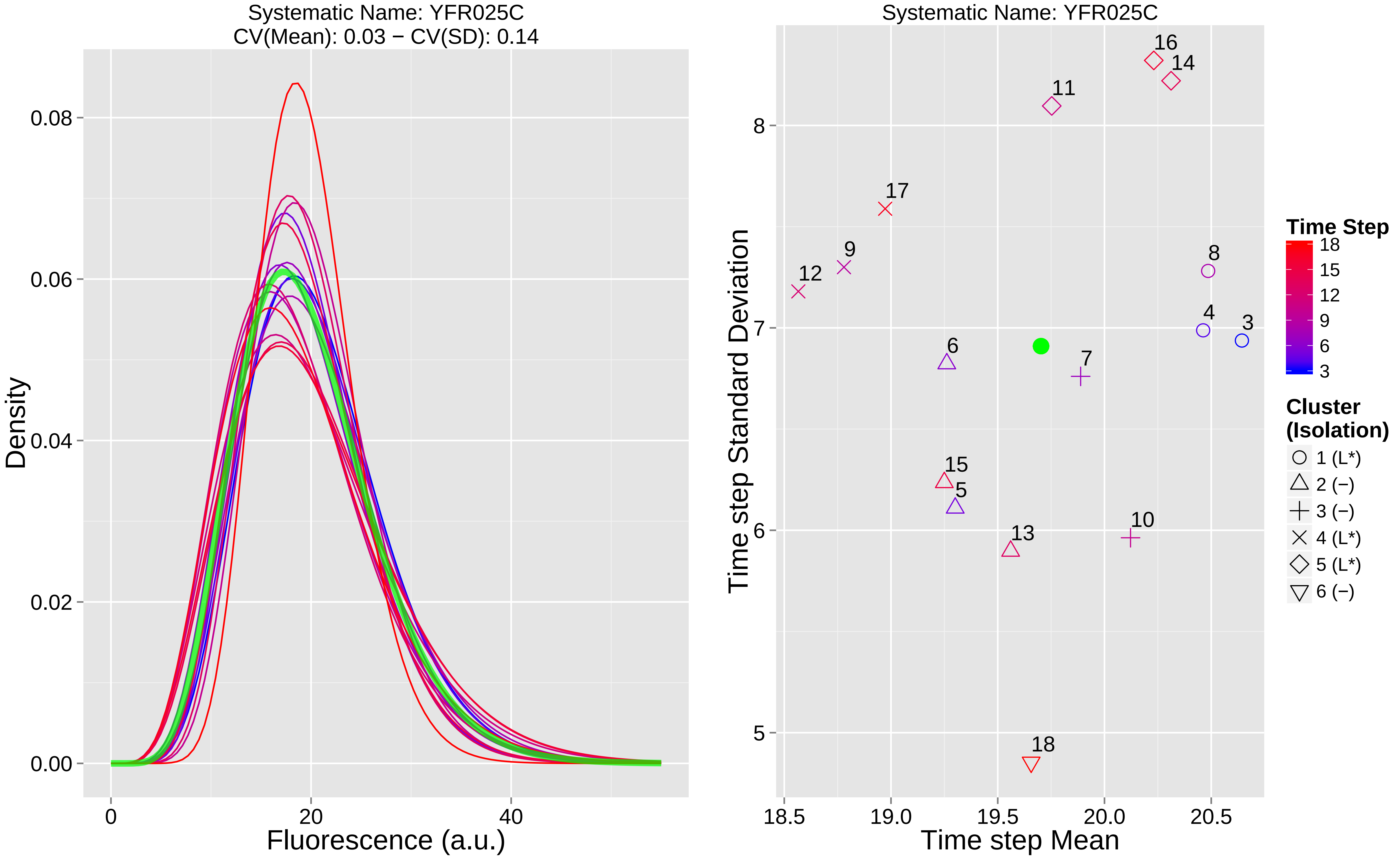
Systematic Name: YFR025C
CV(Mean): 0.03 − CV(SD): 0.14
Density
0.00
0.02
0.04
0.06
0.08
0
20
40
Fluorescence (a.u.)
Systematic Name: YFR025C
Time step Standard Deviation
5
6
7
8
18.5
19.0
19.5
20.0
20.5
Time step Mean
16
14
11
17
9
12
8
4
3
6
7
15
5
13
10
18
Time Step
18
15
12
9
6
3
Cluster
(Isolation)
1 (L*)
2 (−)
3 (−)
4 (L*)
5 (L*)
6 (−)

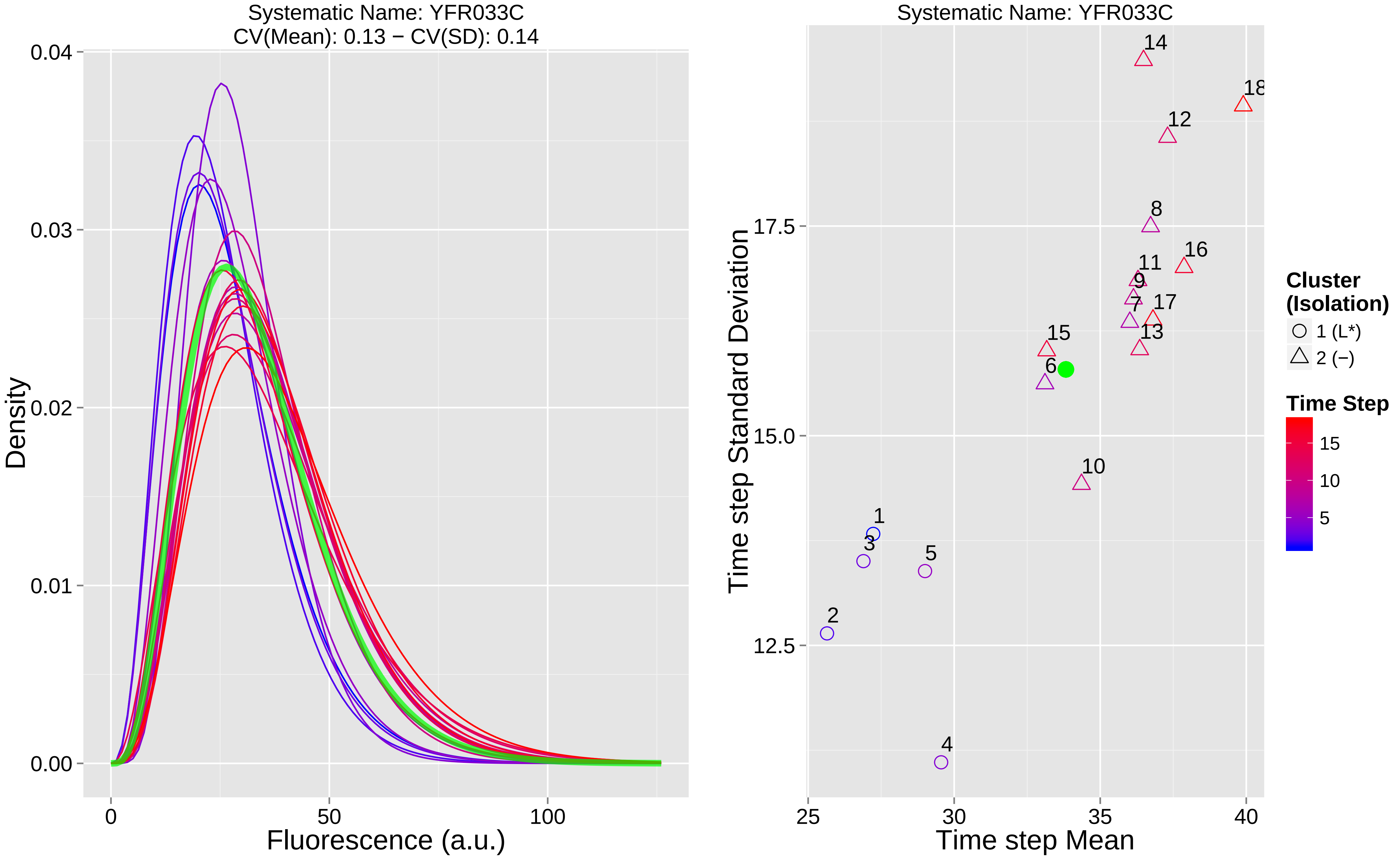

Systematic Name: YFR033C
CV(Mean): 0.13 − CV(SD): 0.14
Density
Fluorescence (a.u.)
0.00
0.01
0.02
0.03
0.04
0
50
100
Systematic Name: YFR033C
Time step Standard Deviation
Time step Mean
12.5
15.0
17.5
25
30
35
40
1
2
3
4
5
6
7
8
9
10
11
12
13
14
15
16
17
18
Cluster (Isolation)
1 (L*)
2 (−)
Time Step
15
10
5

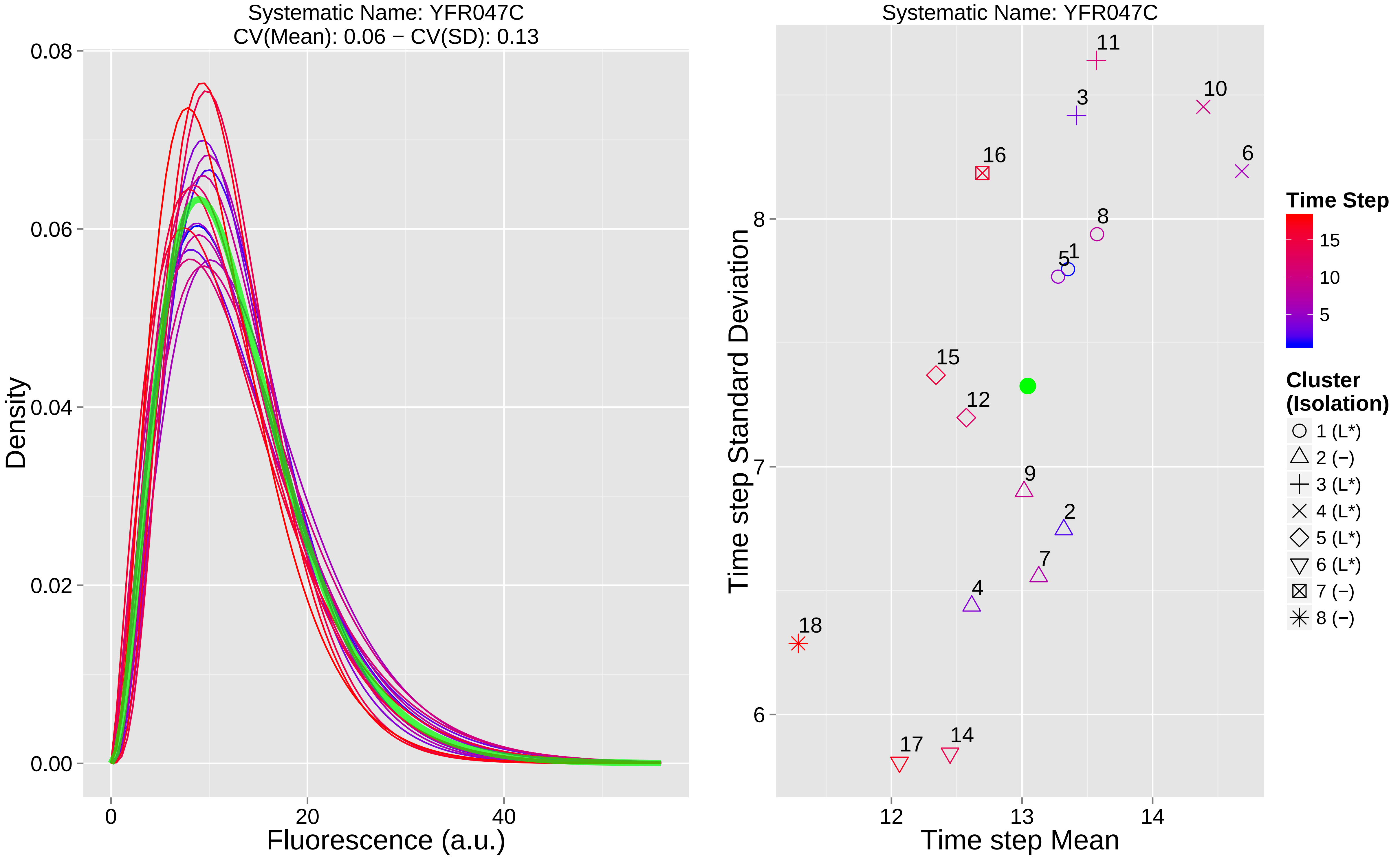

Systematic Name: YFR047C
CV(Mean): 0.06 − CV(SD): 0.13
Density
Fluorescence (a.u.)
0.00
0.02
0.04
0.06
0.08
0
20
40
Systematic Name: YFR047C
Time step Standard Deviation
Time step Mean
6
7
8
12
13
14
11
10
3
6
16
8
5
1
15
12
9
2
7
4
18
17
14
Time Step
15
10
5
Cluster (Isolation)
1 (L*)
2 (−)
3 (L*)
4 (L*)
5 (L*)
6 (L*)
7 (−)
8 (−)

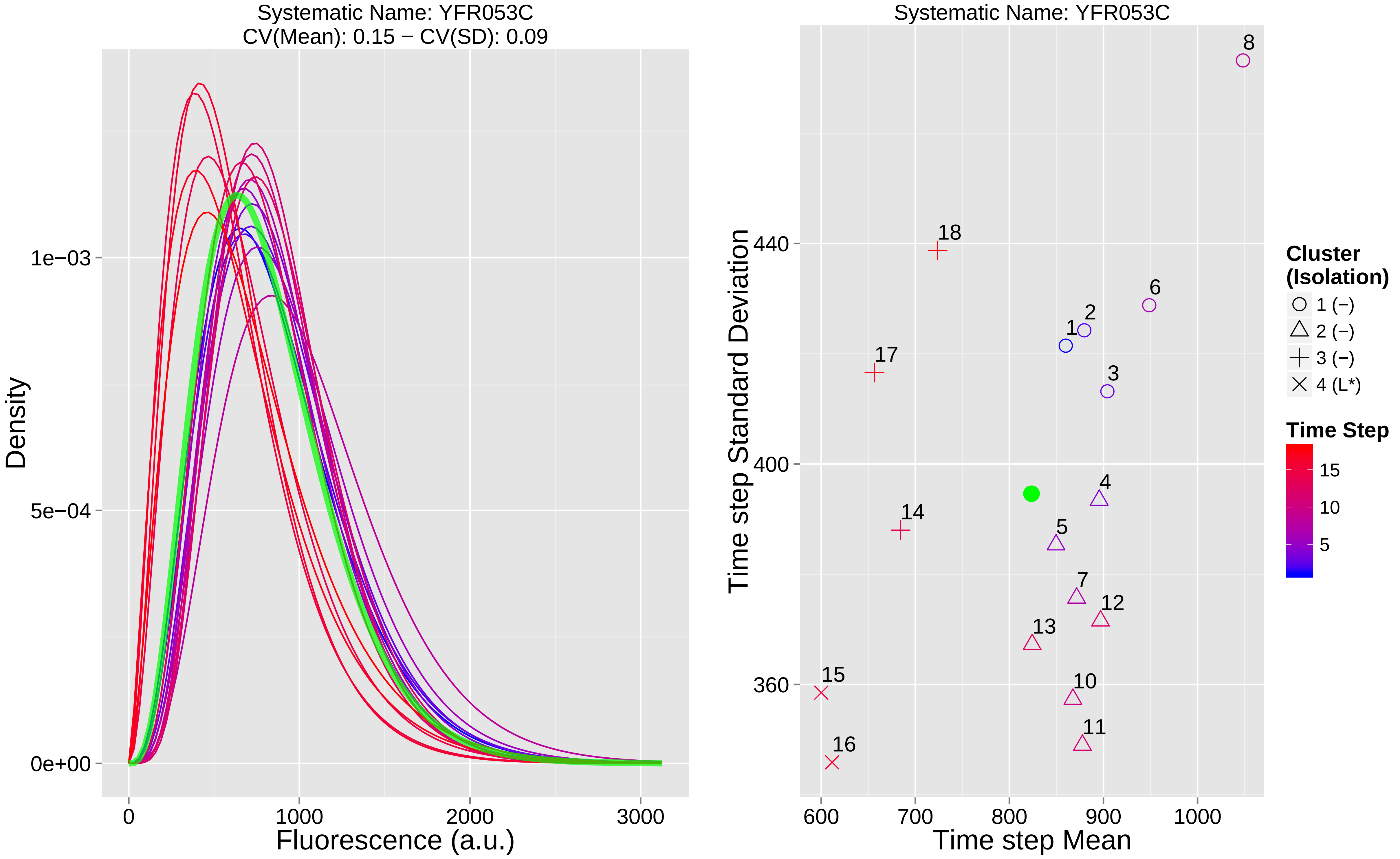

Systematic Name: YFR053C
CV(Mean): 0.15 − CV(SD): 0.09
Density
1e−03
5e−04
0e+00
0
1000
2000
3000
Fluorescence (a.u.)
Systematic Name: YFR053C
Time step Standard Deviation
440
400
360
600
700
800
900
1000
Time step Mean
1
2
3
4
5
6
7
8
10
11
12
13
14
15
16
17
18
Cluster (Isolation)
1 (−)
2 (−)
3 (−)
4 (L*)
Time Step
15
10
5

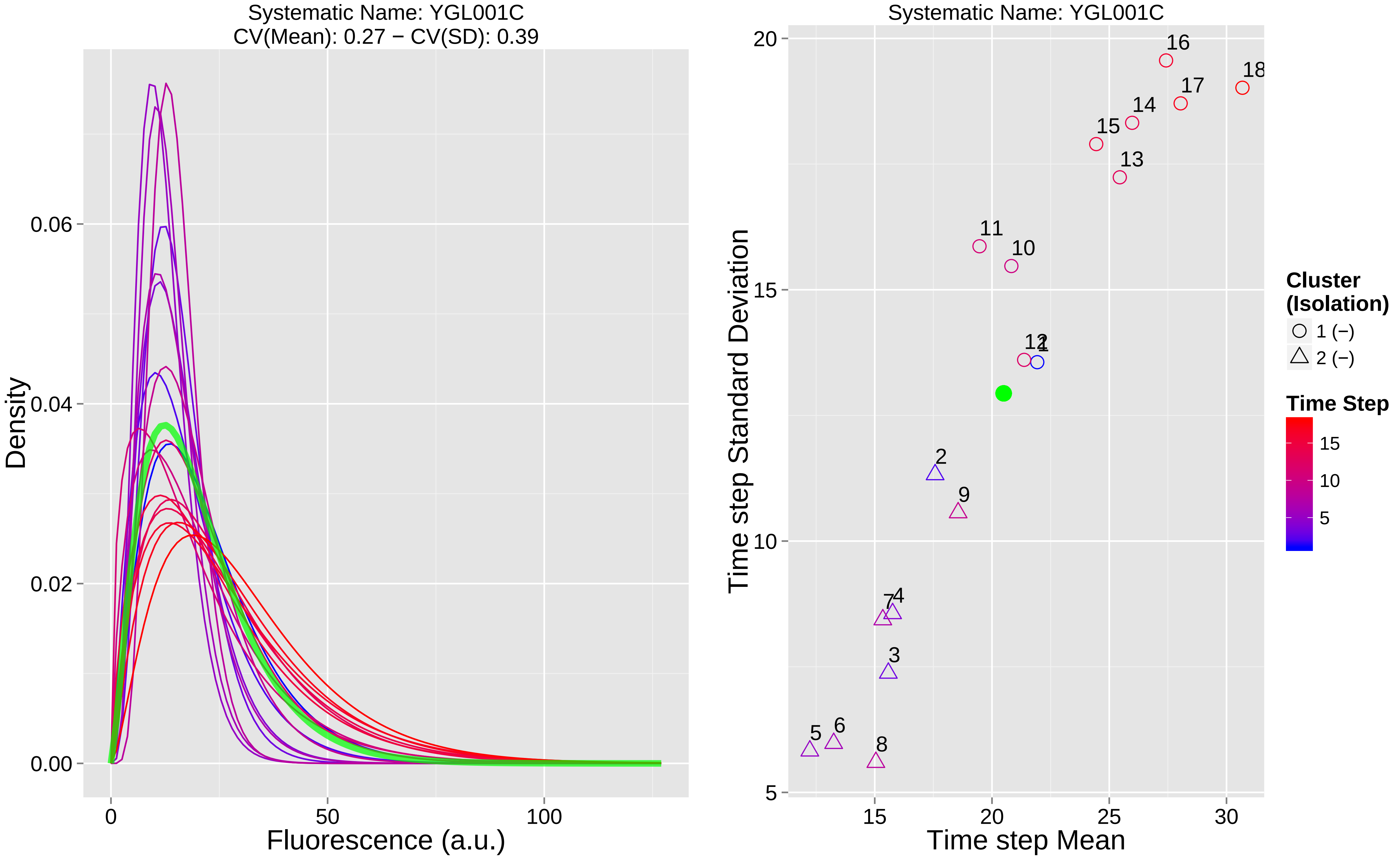
Systematic Name: YGL001C
CV(Mean): 0.27 − CV(SD): 0.39
Density
0.06
0.04
0.02
0.00
0
50
100
Fluorescence (a.u.)
Systematic Name: YGL001C
Time step Standard Deviation
20
15
10
5
15
20
25
30
Time step Mean
16
18
17
14
15
13
11
10
12
1
2
9
7
4
3
5
6
8
Cluster (Isolation)
1 (−)
2 (−)
Time Step
15
10
5

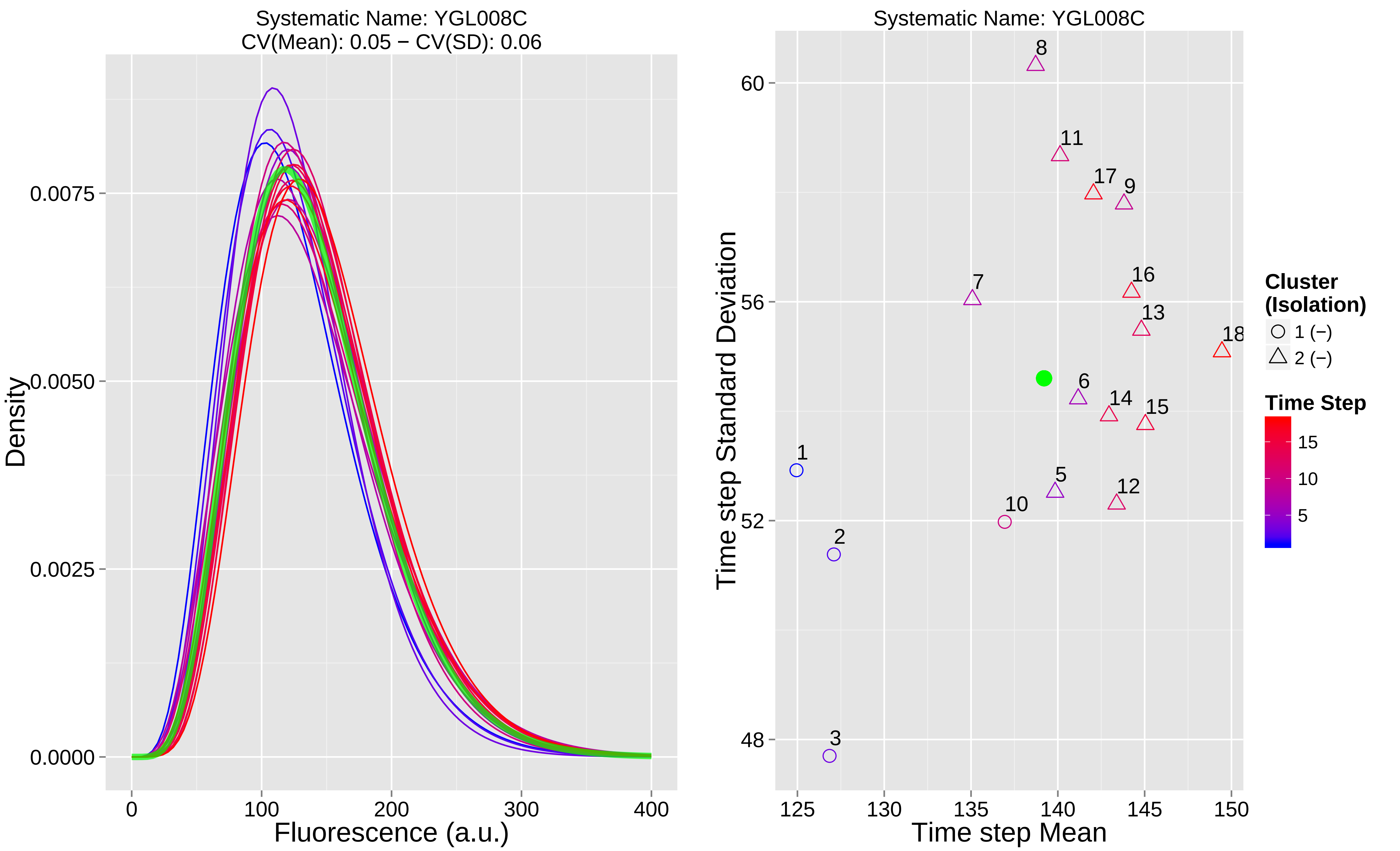
Systematic Name: YGL008C
CV(Mean): 0.05 − CV(SD): 0.06
Density
0.0075
0.0050
0.0025
0.0000
0
100
200
300
400
Fluorescence (a.u.)
Systematic Name: YGL008C
Time step Standard Deviation
60
56
52
48
125
130
135
140
145
150
Time step Mean
1
2
3
5
6
7
8
9
10
11
12
13
14
15
16
17
18
Cluster
(Isolation)
1 (−)
2 (−)
Time Step
15
10
5

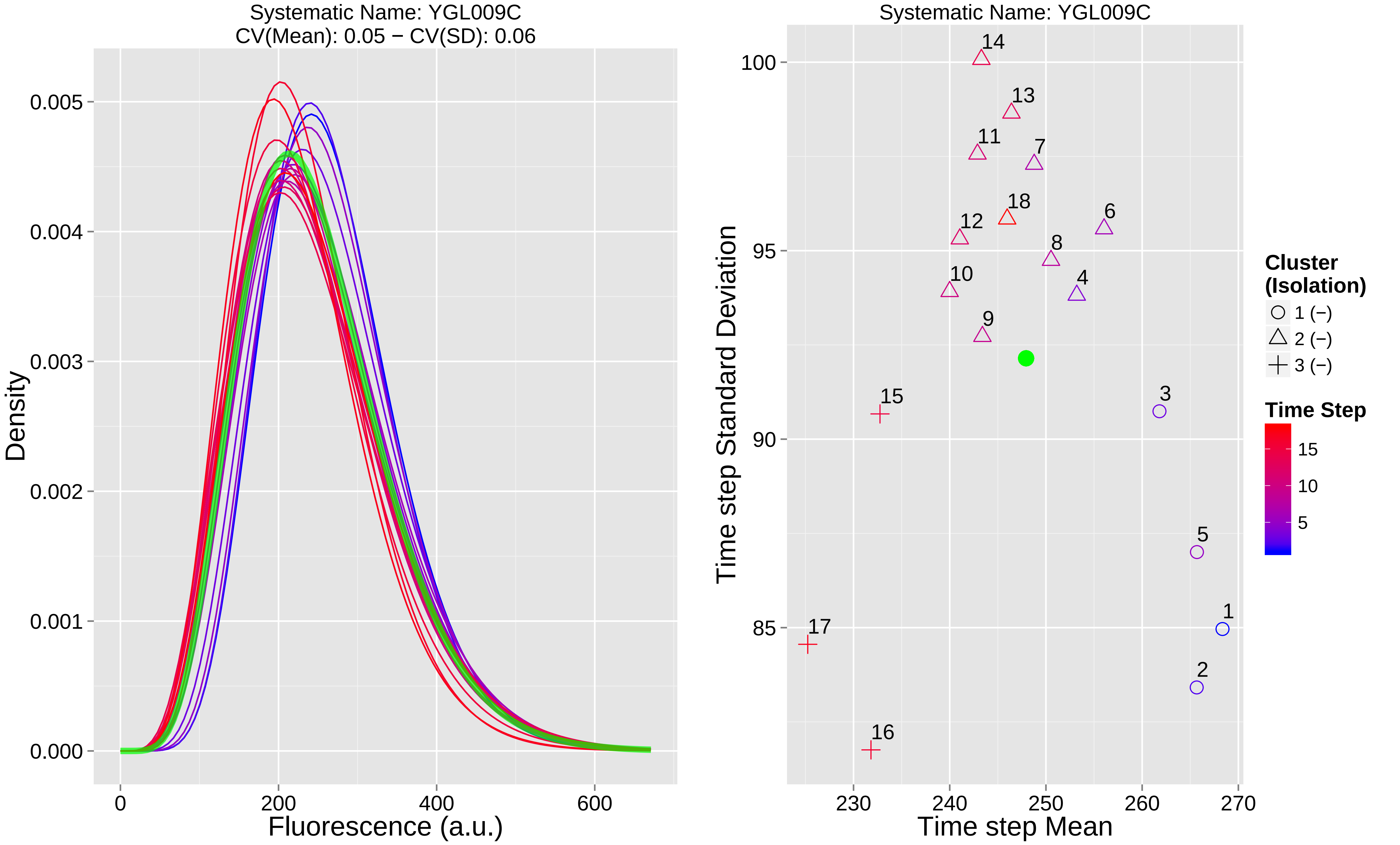

Systematic Name: YGL009C
CV(Mean): 0.05 − CV(SD): 0.06
Density
Fluorescence (a.u.)
0.000
0.001
0.002
0.003
0.004
0.005
0
200
400
600
Systematic Name: YGL009C
Time step Standard Deviation
Time step Mean
85
90
95
100
230
240
250
260
270
Cluster (Isolation)
1 (−)
2 (−)
3 (−)
Time Step
15
10
5

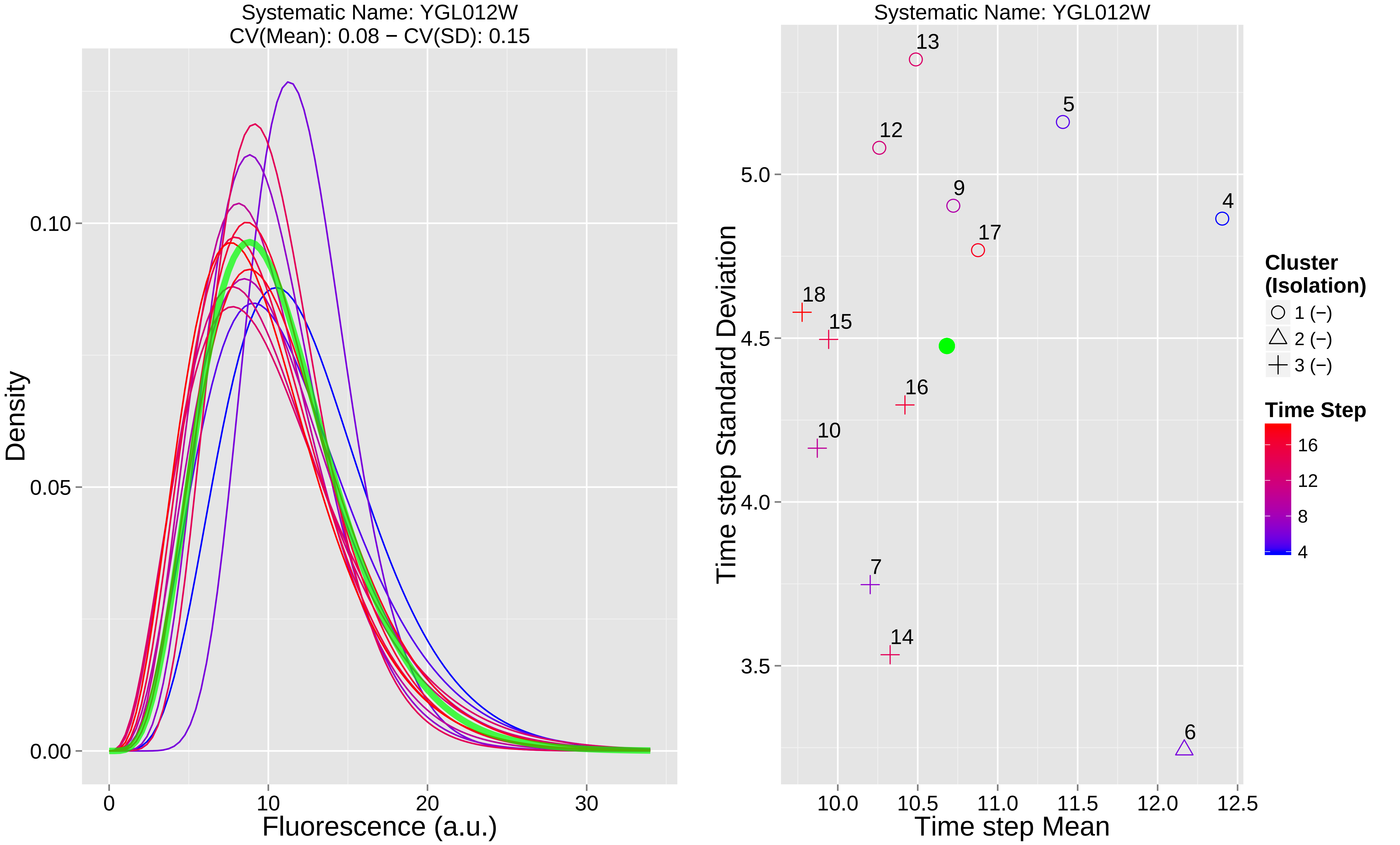

Systematic Name: YGL012W
CV(Mean): 0.08 − CV(SD): 0.15
Density
Fluorescence (a.u.)
0.00
0.05
0.10
0
10
20
30
Systematic Name: YGL012W
Time step Standard Deviation
Time step Mean
3.5
4.0
4.5
5.0
10.0
10.5
11.0
11.5
12.0
12.5
13
5
12
9
4
17
18
15
16
10
7
14
6
Cluster
(Isolation)
1 (−)
2 (−)
3 (−)
Time Step
16
12
8
4

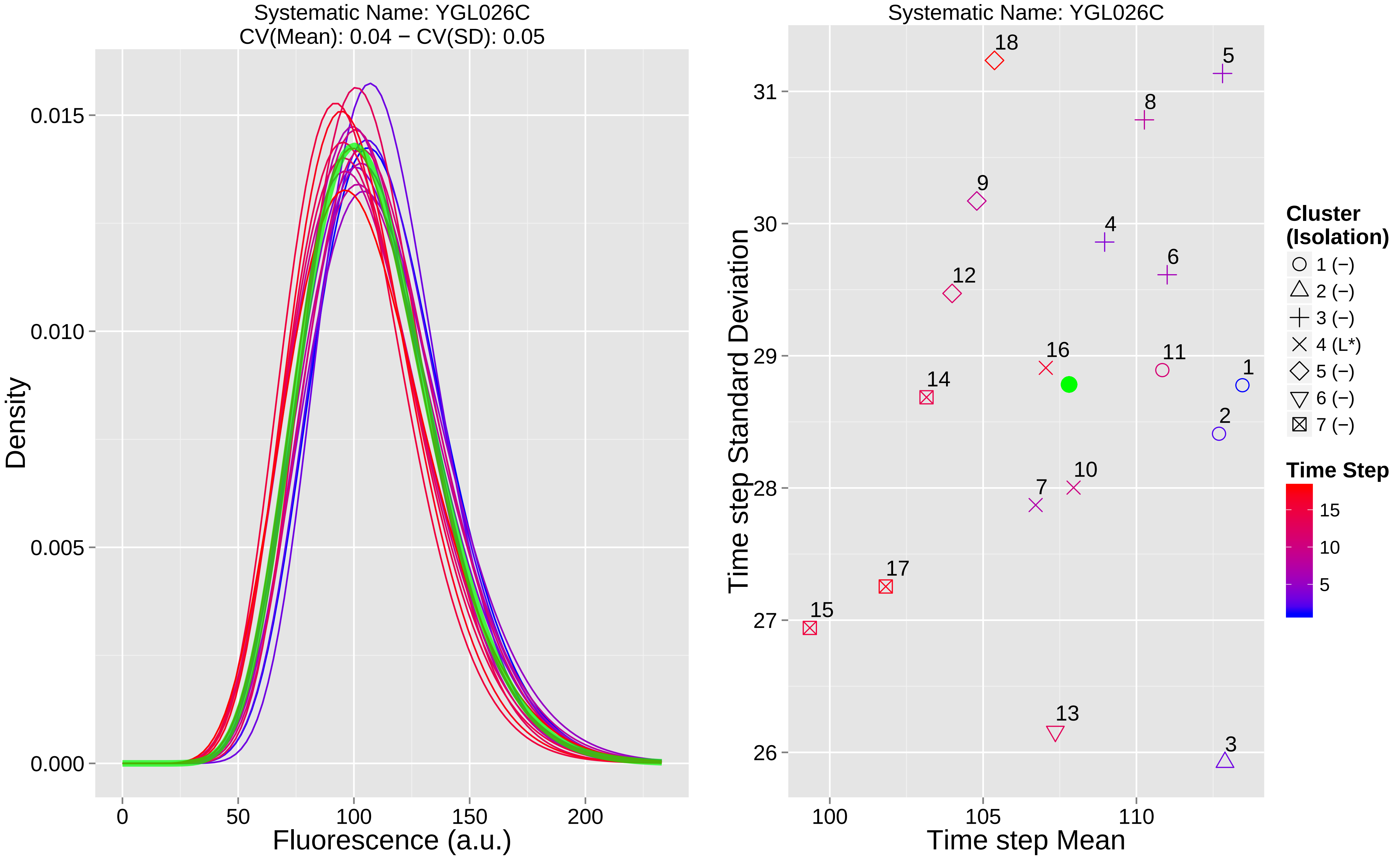
Systematic Name: YGL026C
CV(Mean): 0.04 − CV(SD): 0.05
Density
0.015
0.010
0.005
0.000
0
50
100
150
200
Fluorescence (a.u.)
Systematic Name: YGL026C
Time step Standard Deviation
31
30
29
28
27
26
100
105
110
Time step Mean
18
5
8
9
4
6
12
16
11
1
14
2
10
7
17
15
13
3
Cluster (Isolation)
1 (−)
2 (−)
3 (−)
4 (L*)
5 (−)
6 (−)
7 (−)
Time Step
15
10
5

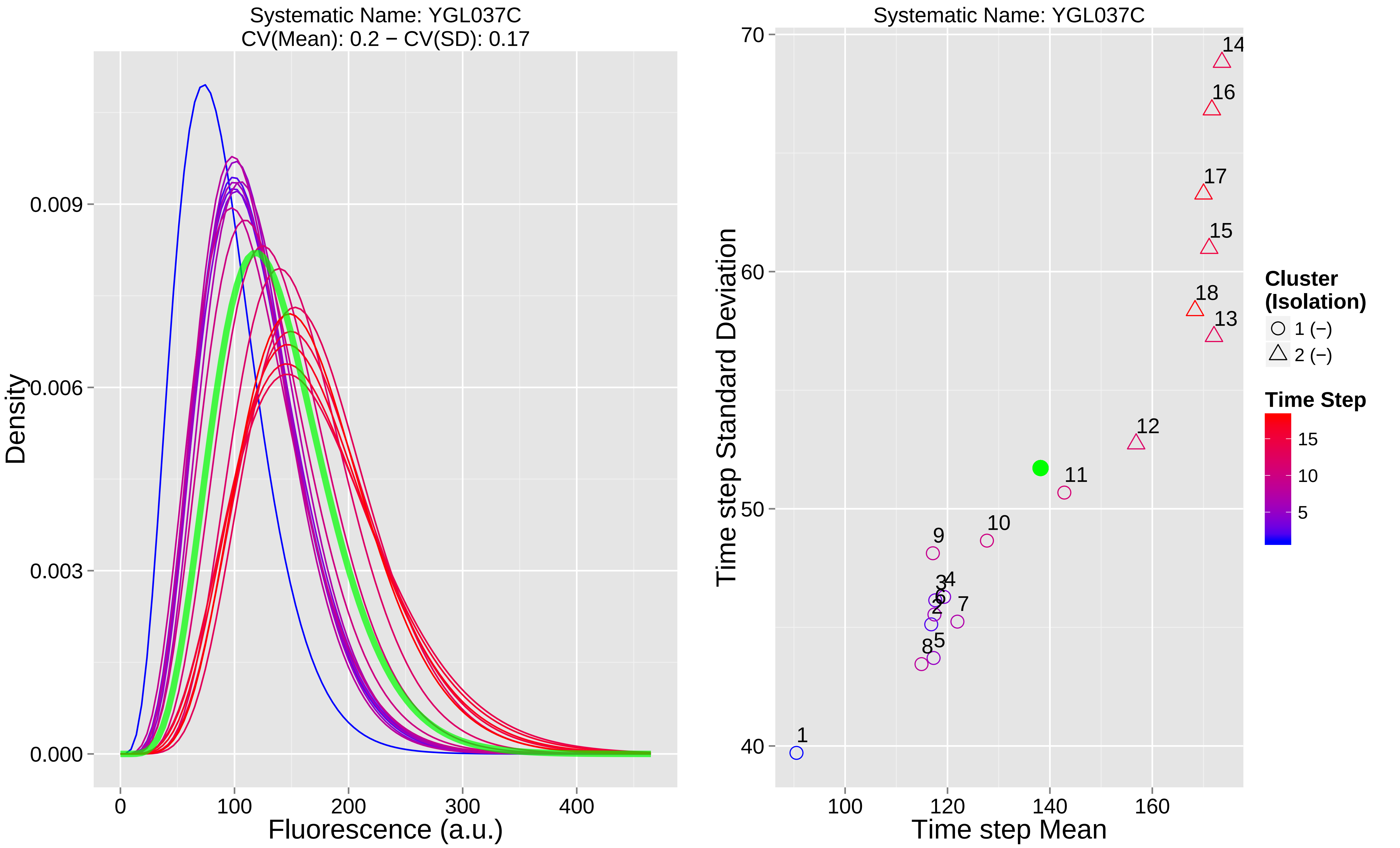

Systematic Name: YGL037C
CV(Mean): 0.2 − CV(SD): 0.17
Density
0.009
0.006
0.003
0.000
0
100
200
300
400
Fluorescence (a.u.)
Systematic Name: YGL037C
Time step Standard Deviation
70
60
50
40
100
120
140
160
Time step Mean
1
2
3
4
5
6
7
8
9
10
11
12
13
14
15
16
17
18
Cluster
(Isolation)
1 (−)
2 (−)
Time Step
15
10
5

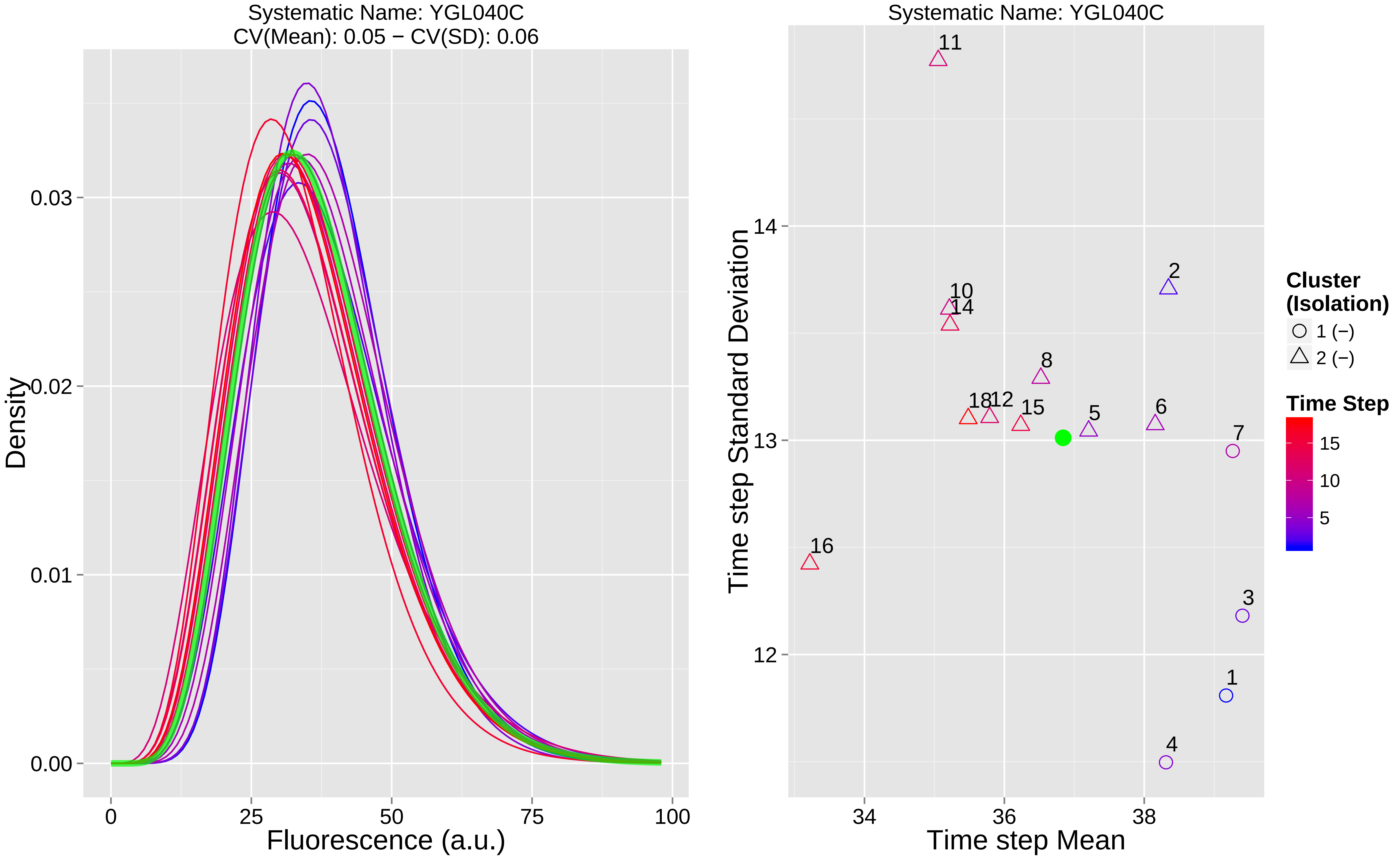

Systematic Name: YGL040C
CV(Mean): 0.05 − CV(SD): 0.06
Density
0.00
0.01
0.02
0.03
0
25
50
75
100
Fluorescence (a.u.)
Systematic Name: YGL040C
Time step Standard Deviation
12
13
14
34
36
38
Time step Mean
11
10
14
2
8
18
12
15
5
6
7
16
3
1
4
Cluster (Isolation)
1 (−)
2 (−)
Time Step
15
10
5

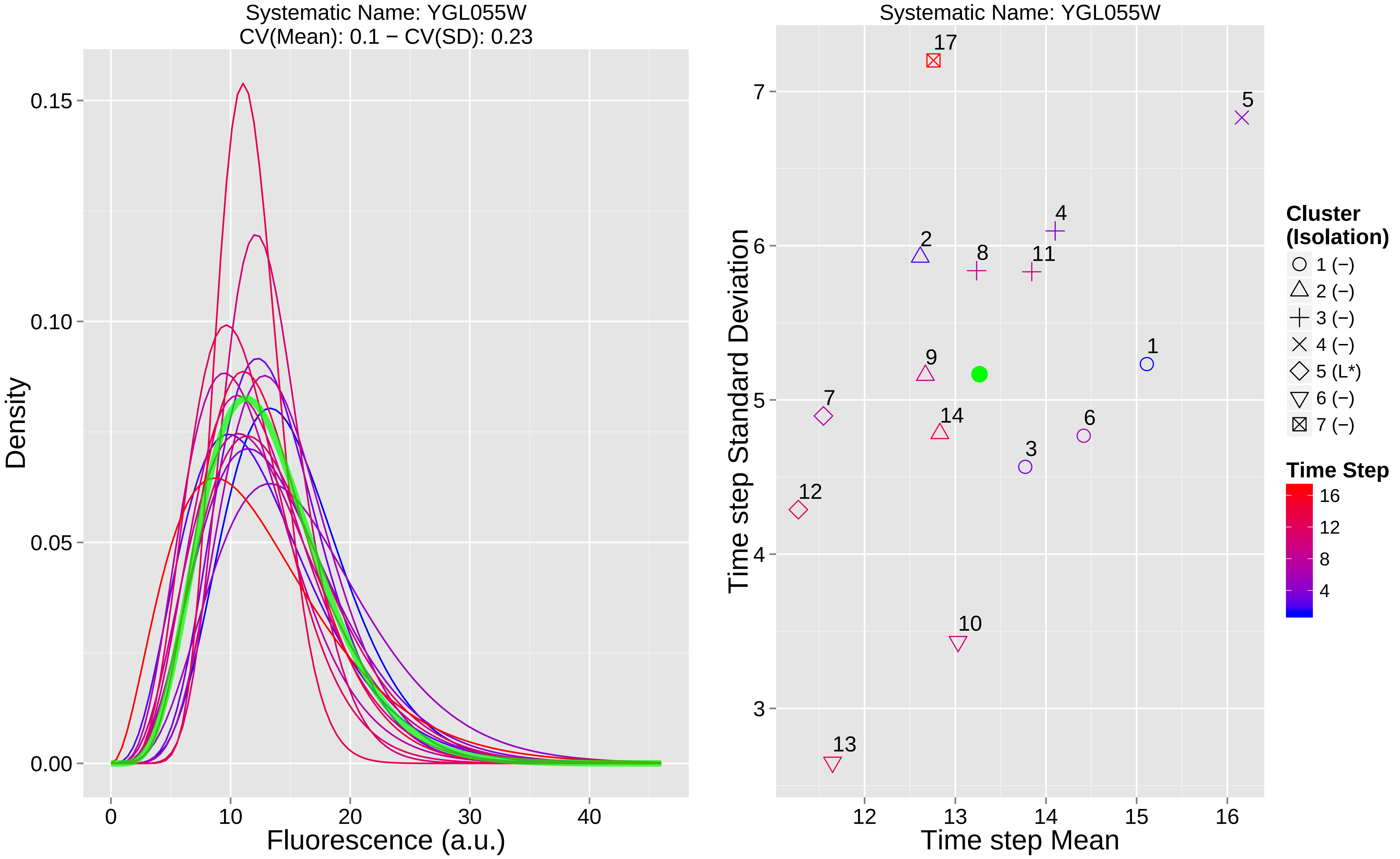

Systematic Name: YGL055W
CV(Mean): 0.1 − CV(SD): 0.23
Density
Fluorescence (a.u.)
0.15
0.10
0.05
0.00
0
10
20
30
40
Systematic Name: YGL055W
Time step Standard Deviation
Time step Mean
7
6
5
4
3
12
13
14
15
16
17
5
4
2
8
11
9
1
7
14
6
3
12
10
13
Cluster (Isolation)
1 (−)
2 (−)
3 (−)
4 (−)
5 (L*)
6 (−)
7 (−)
Time Step
16
12
8
4

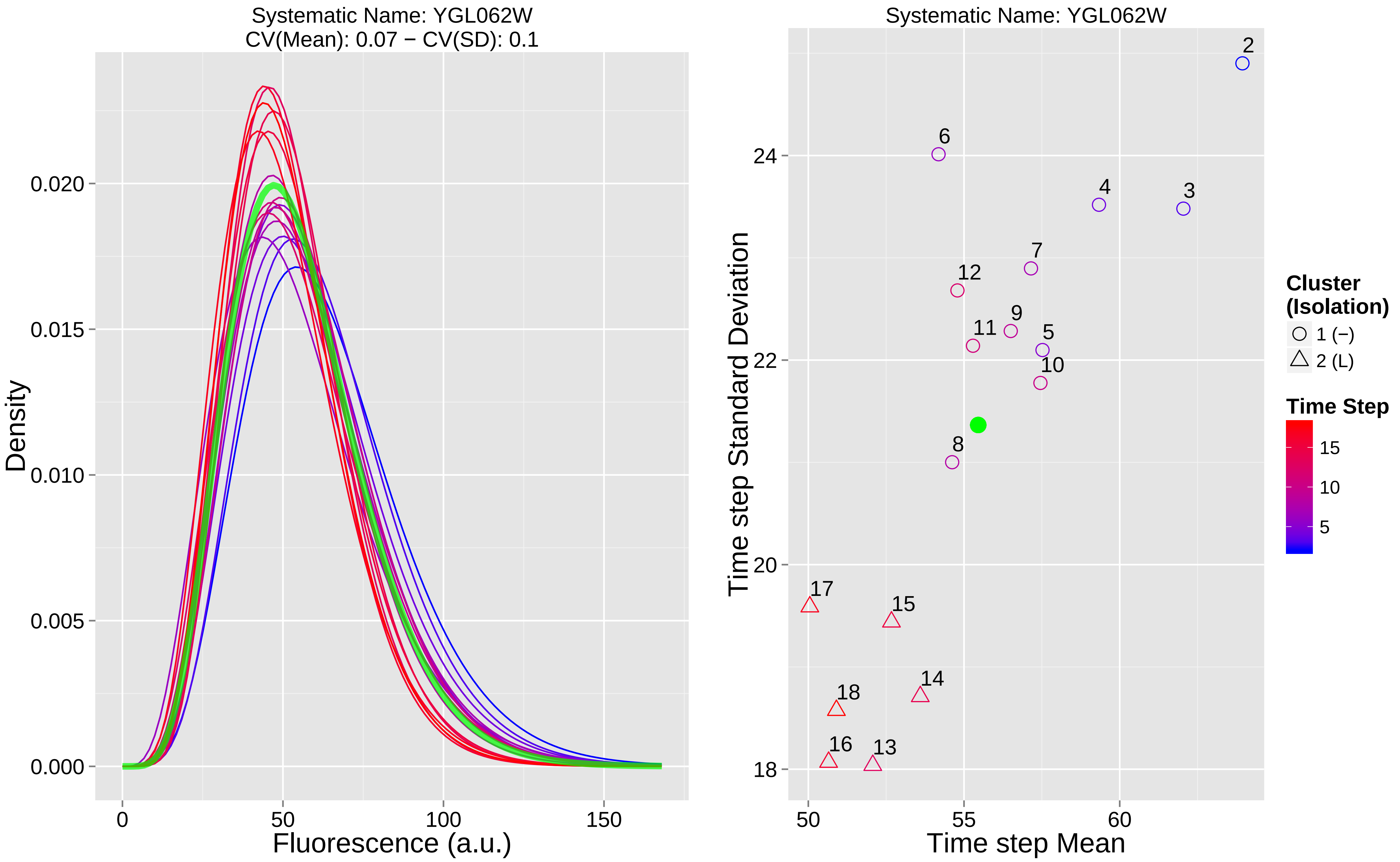

Systematic Name: YGL062W
CV(Mean): 0.07 − CV(SD): 0.1
Density
Fluorescence (a.u.)
0.000
0.005
0.010
0.015
0.020
0
50
100
150
Systematic Name: YGL062W
Time step Standard Deviation
Time step Mean
18
20
22
24
50
55
60
2
3
4
5
6
7
8
9
10
11
12
13
14
15
16
17
18
Cluster
(Isolation)
1 (−)
2 (L)
Time Step
15
10
5

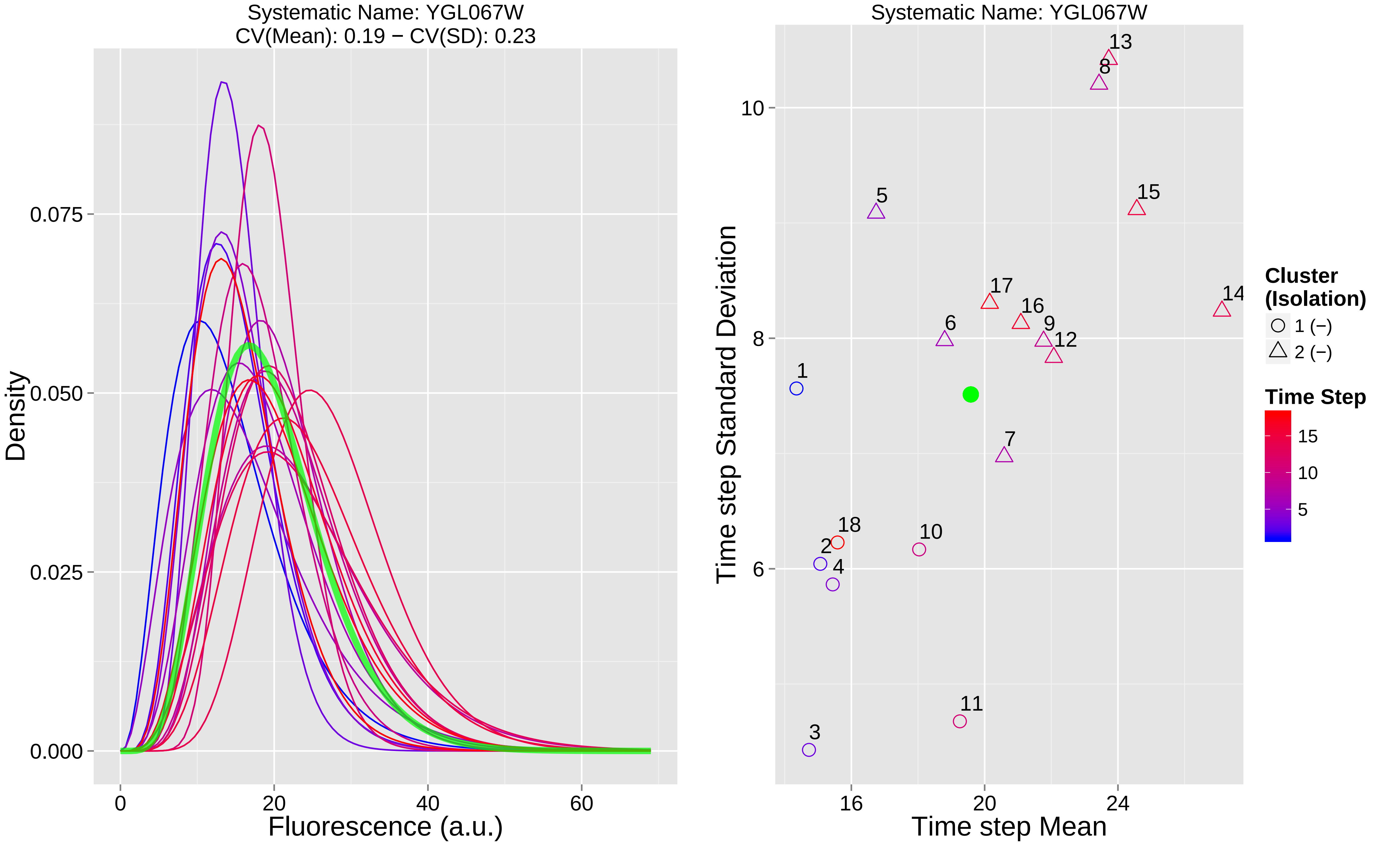

Systematic Name: YGL067W
CV(Mean): 0.19 − CV(SD): 0.23
Density
0.000
0.025
0.050
0.075
0
20
40
60
Fluorescence (a.u.)
Systematic Name: YGL067W
Time step Standard Deviation
6
8
10
16
20
24
Time step Mean
1
2
3
4
5
6
7
8
9
10
11
12
13
14
15
16
17
18
Cluster
(Isolation)
1 (−)
2 (−)
Time Step
15
10
5

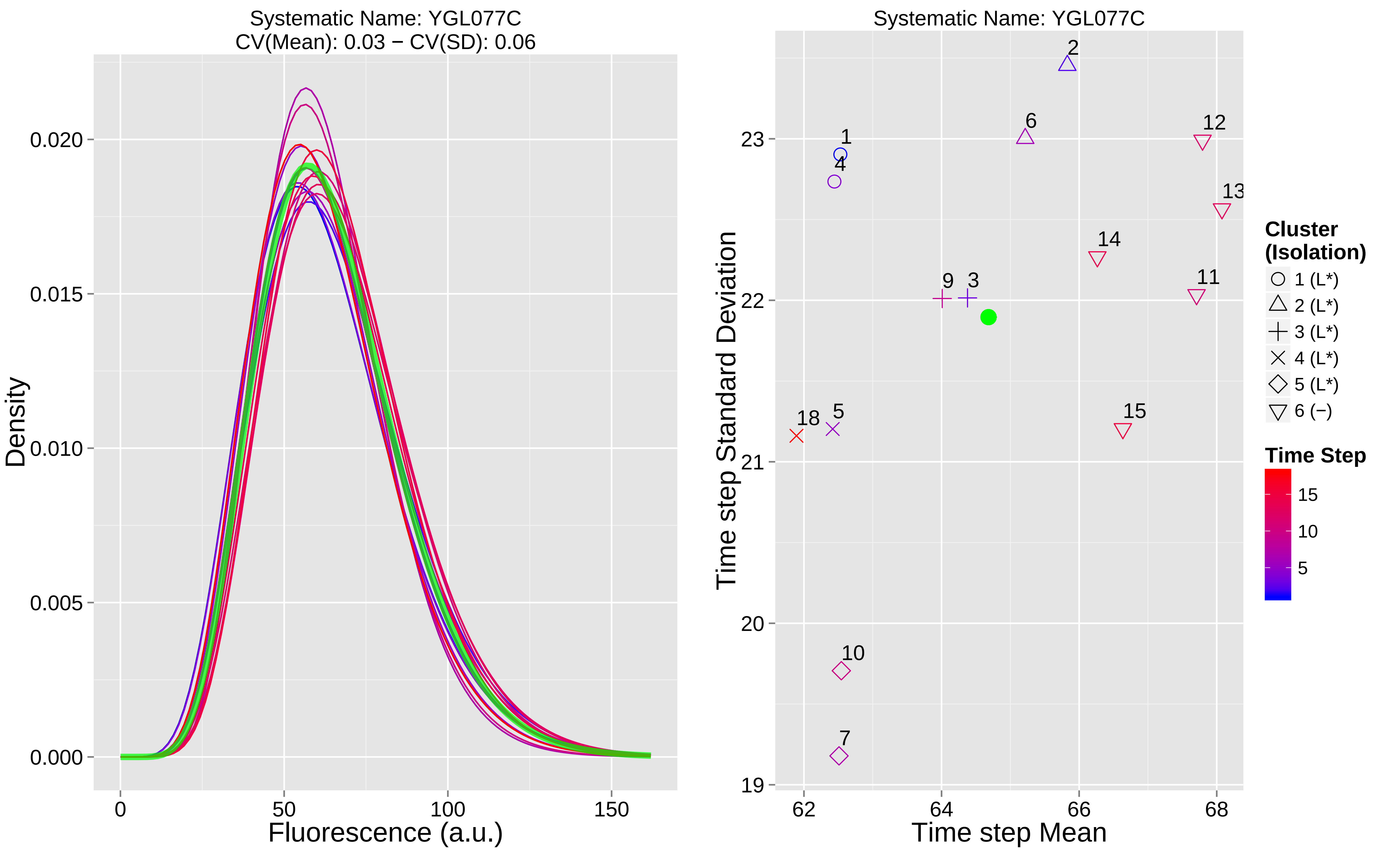

Systematic Name: YGL077C
CV(Mean): 0.03 − CV(SD): 0.06
Density
0.000
0.005
0.010
0.015
0.020
0
50
100
150
Fluorescence (a.u.)
Systematic Name: YGL077C
Time step Standard Deviation
19
20
21
22
23
62
64
66
68
Time step Mean
1
2
3
4
5
6
7
9
10
11
12
13
14
15
18
Cluster
(Isolation)
1 (L*)
2 (L*)
3 (L*)
4 (L*)
5 (L*)
6 (−)
Time Step
15
10
5

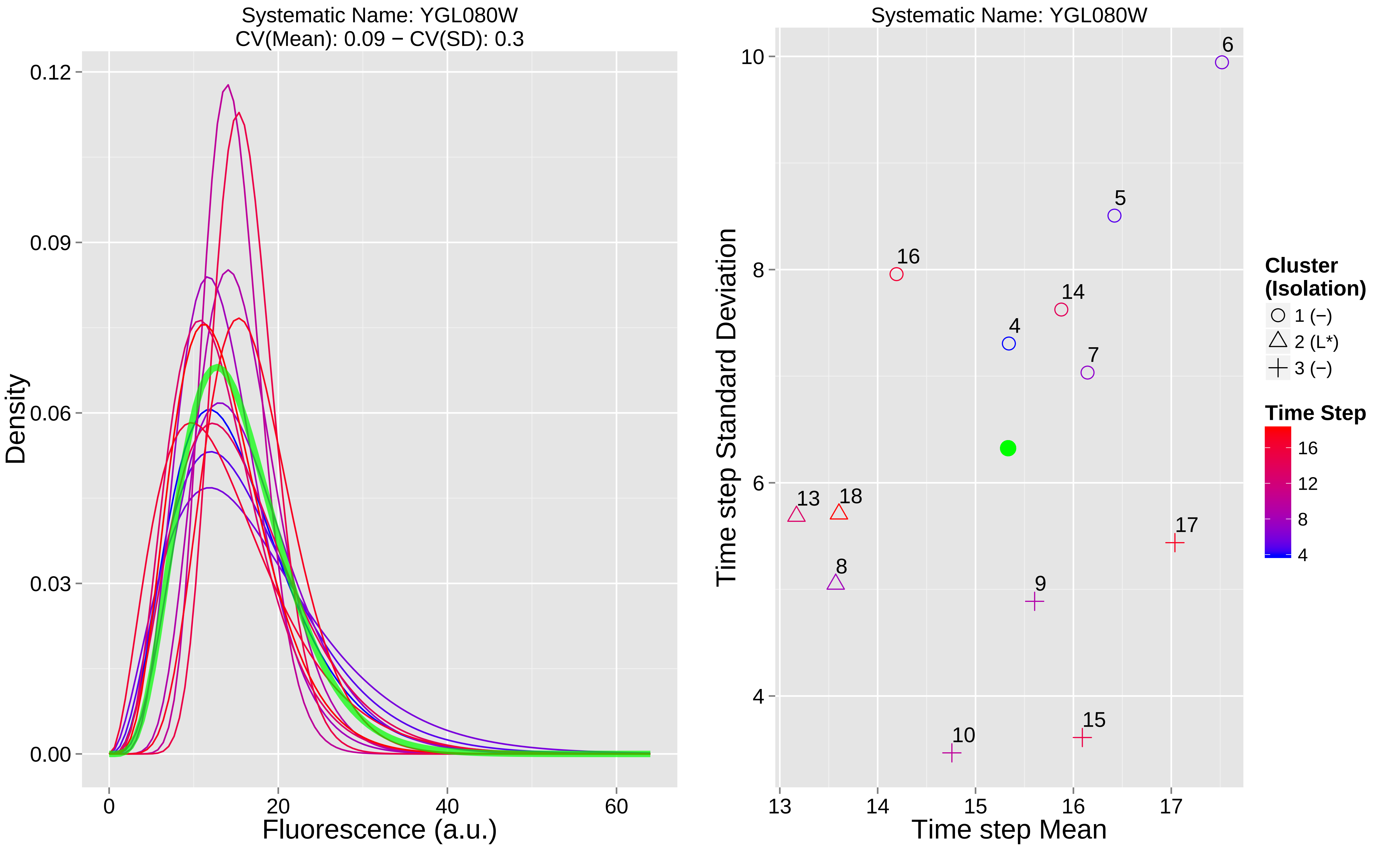

Systematic Name: YGL080W
CV(Mean): 0.09 − CV(SD): 0.3
Density
0.12
0.09
0.06
0.03
0.00
0
20
40
60
Fluorescence (a.u.)
Systematic Name: YGL080W
Time step Standard Deviation
10
8
6
4
13
14
15
16
17
Time step Mean
6
5
16
14
4
7
13
18
8
17
9
10
15
Cluster (Isolation)
1 (−)
2 (L*)
3 (−)
Time Step
16
12
8
4

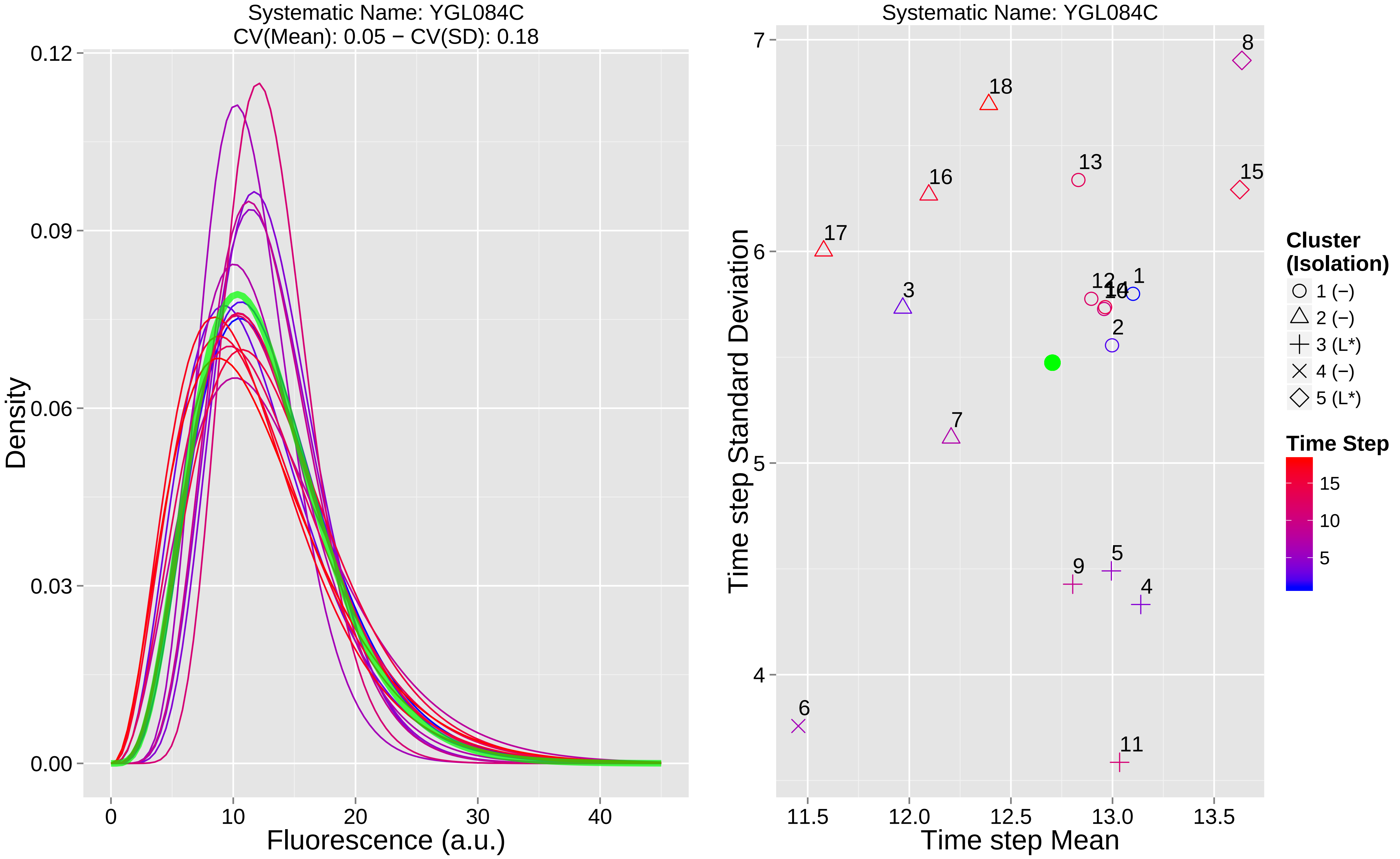

Systematic Name: YGL084C
CV(Mean): 0.05 − CV(SD): 0.18
Density
Fluorescence (a.u.)
0.00
0.03
0.06
0.09
0.12
0
10
20
30
40
Systematic Name: YGL084C
Time step Standard Deviation
Time step Mean
11.5
12.0
12.5
13.0
13.5
4
5
6
7
Cluster (Isolation)
1 (−)
2 (−)
3 (L*)
4 (−)
5 (L*)
Time Step
5
10
15

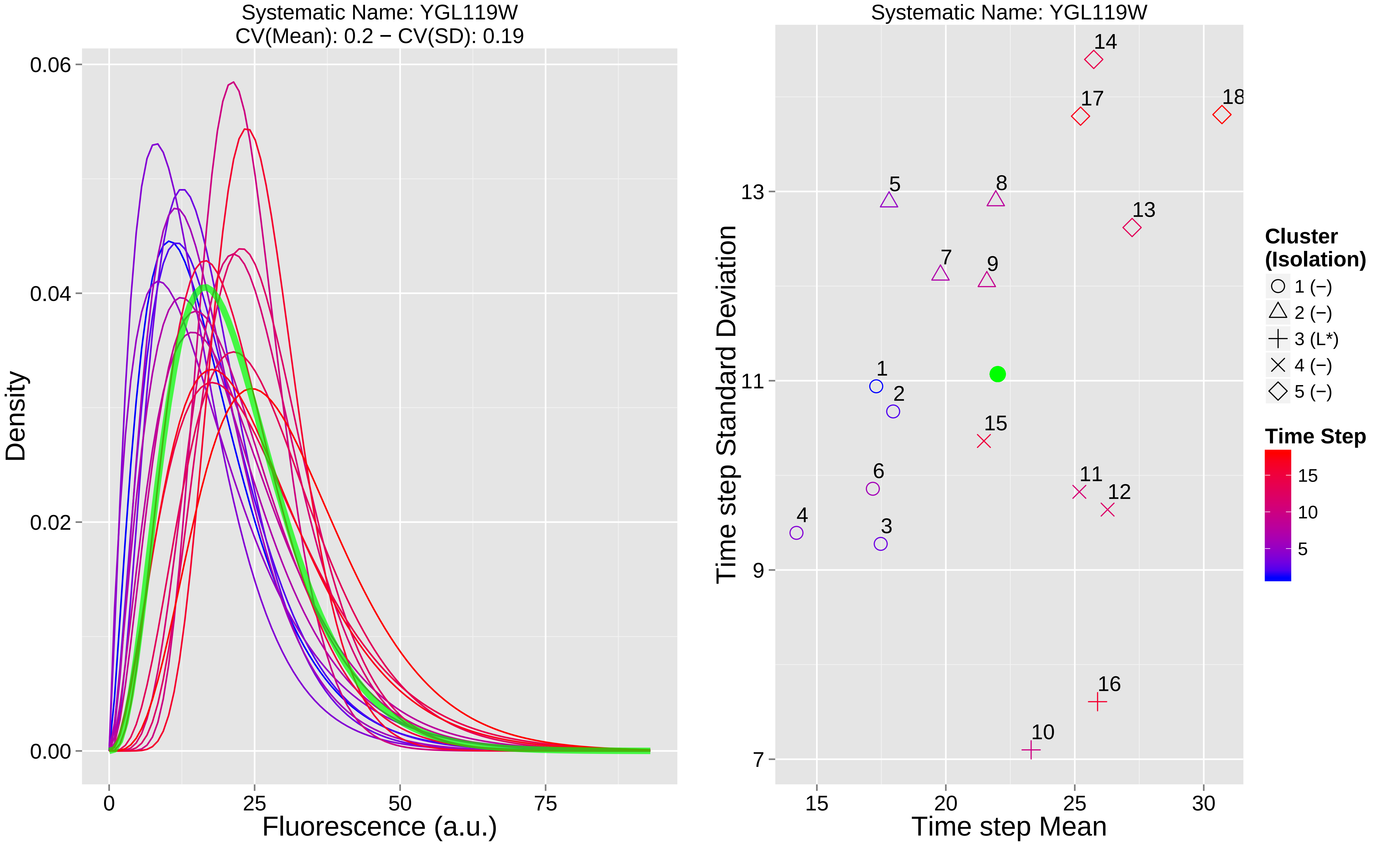

Systematic Name: YGL119W
CV(Mean): 0.2 − CV(SD): 0.19
Density
0.06
0.04
0.02
0.00
0
25
50
75
Fluorescence (a.u.)
Systematic Name: YGL119W
Time step Standard Deviation
13
11
9
7
15
20
25
30
Time step Mean
1
2
3
4
5
6
7
8
9
10
11
12
13
14
15
16
17
18
Cluster
(Isolation)
1 (−)
2 (−)
3 (L*)
4 (−)
5 (−)
Time Step
15
10
5

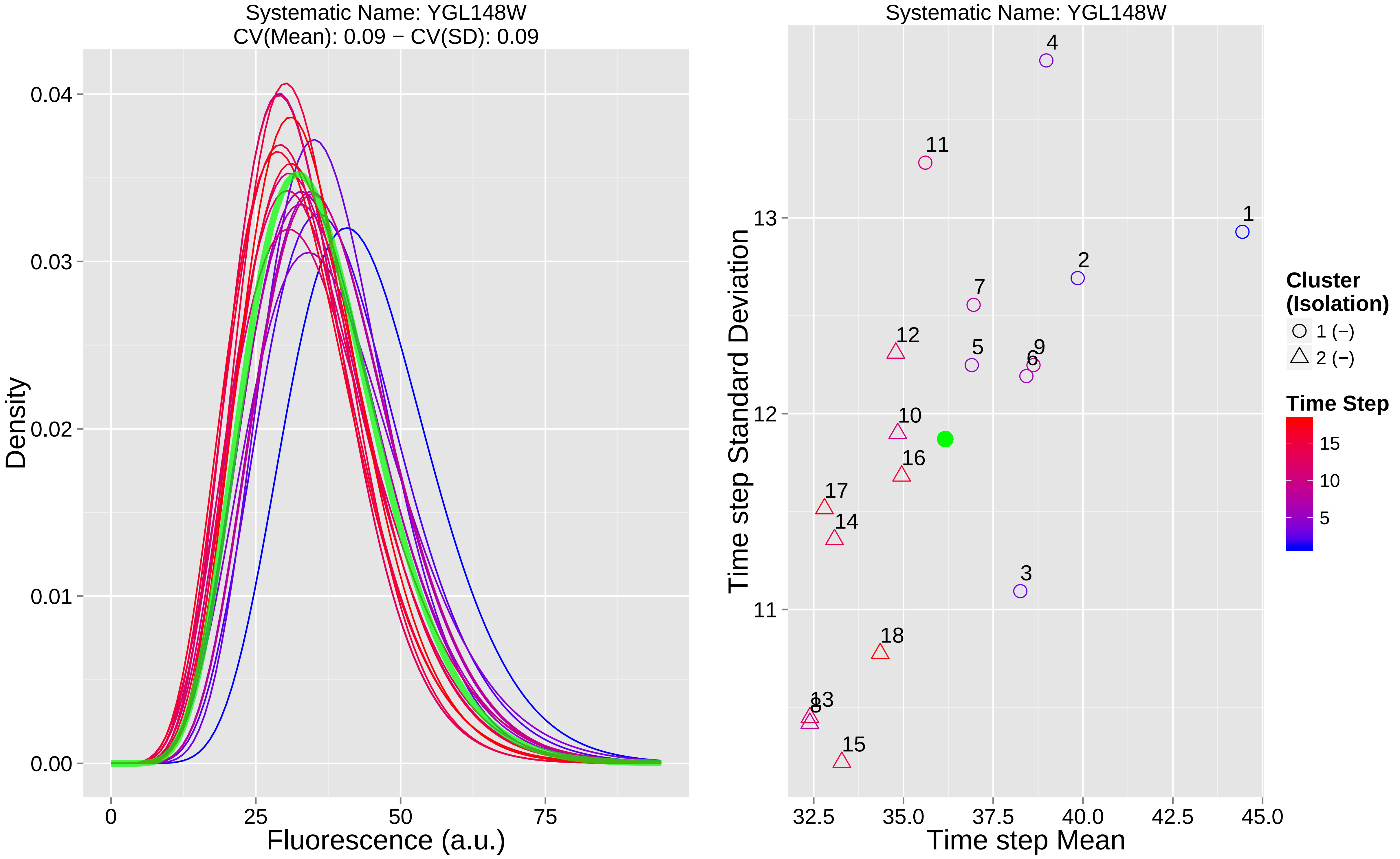

Systematic Name: YGL148W
CV(Mean): 0.09 − CV(SD): 0.09
Density
0.00
0.01
0.02
0.03
0.04
0
25
50
75
Fluorescence (a.u.)
Systematic Name: YGL148W
Time step Standard Deviation
11
12
13
32.5
35.0
37.5
40.0
42.5
45.0
Time step Mean
1
2
3
4
5
6
7
8
9
10
11
12
13
14
15
16
17
18
Cluster (Isolation)
1 (−)
2 (−)
Time Step
15
10
5

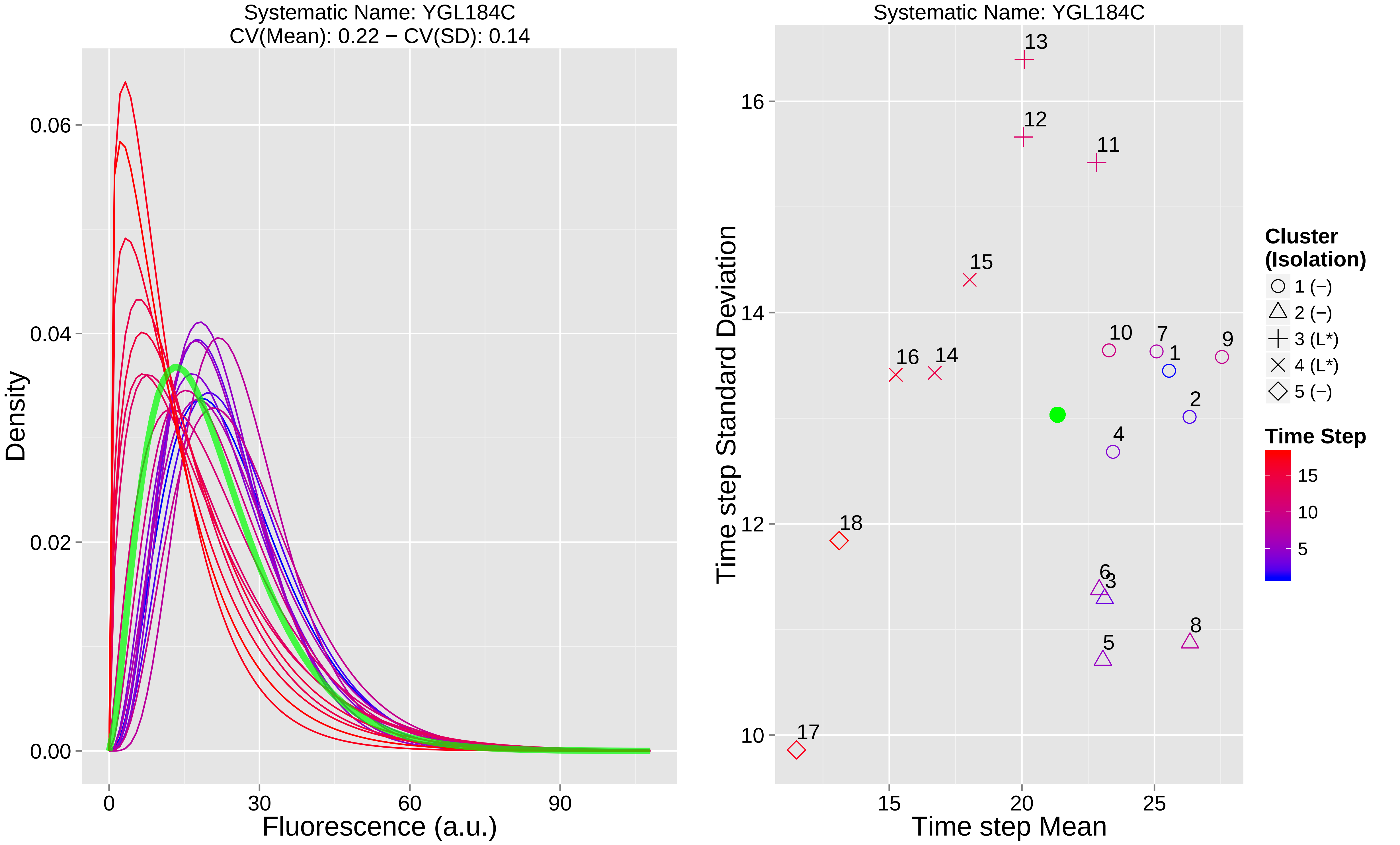
Systematic Name: YGL184C
CV(Mean): 0.22 − CV(SD): 0.14
Density
0.06
0.04
0.02
0.00
0
30
60
90
Fluorescence (a.u.)
Systematic Name: YGL184C
Time step Standard Deviation
16
14
12
10
15
20
25
Time step Mean
13
12
11
15
16
14
10
7
1
9
2
4
18
6
3
8
5
17
Cluster (Isolation)
1 (−)
2 (−)
3 (L*)
4 (L*)
5 (−)
Time Step
15
10
5

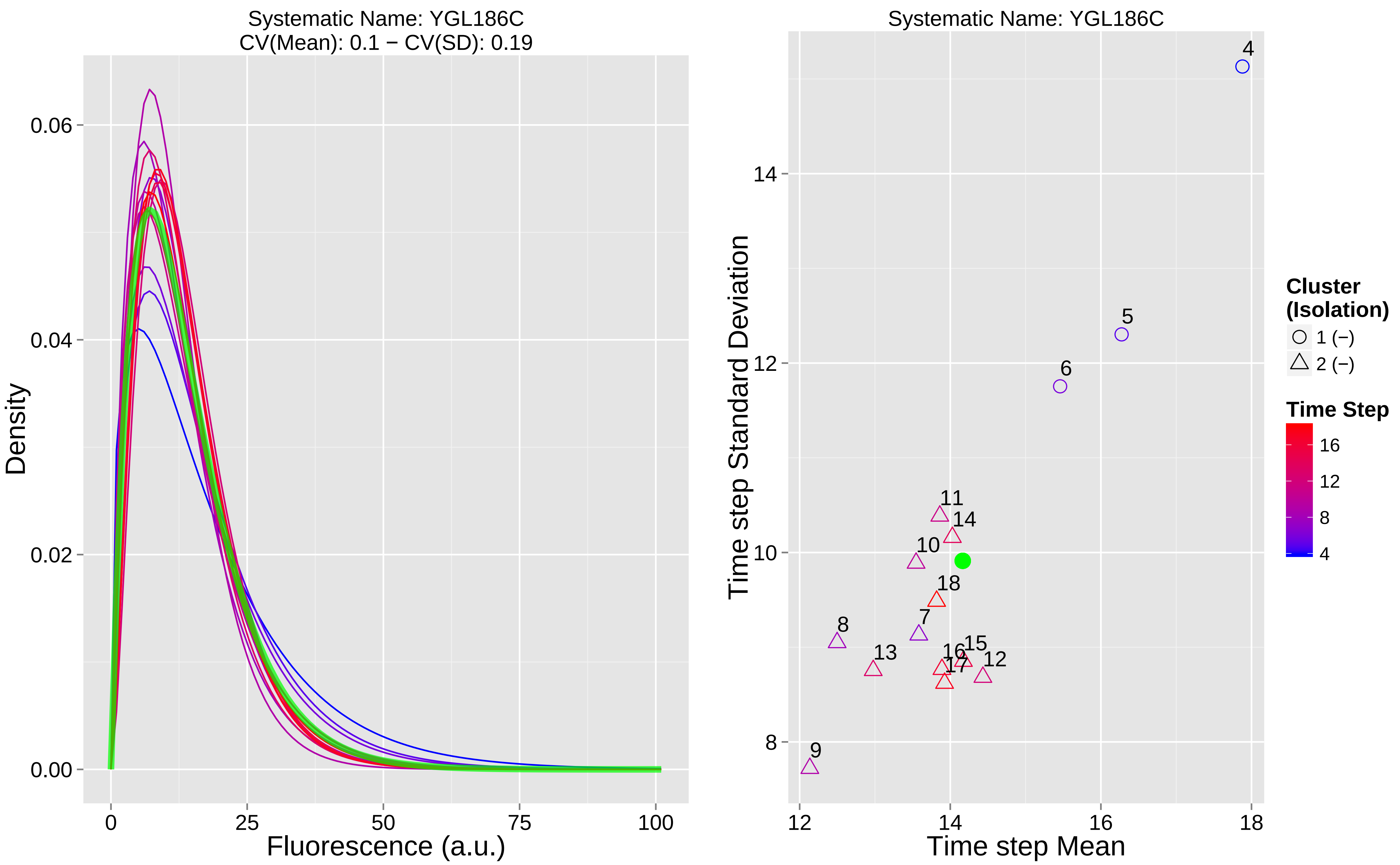
Systematic Name: YGL186C
CV(Mean): 0.1 − CV(SD): 0.19
Density
Fluorescence (a.u.)
0.00
0.02
0.04
0.06
0
25
50
75
100
Systematic Name: YGL186C
Time step Standard Deviation
Time step Mean
8
10
12
14
12
14
16
18
4
5
6
7
8
9
10
11
12
13
14
15
16
17
18
Cluster (Isolation)
1 (−)
2 (−)
Time Step
16
12
8
4

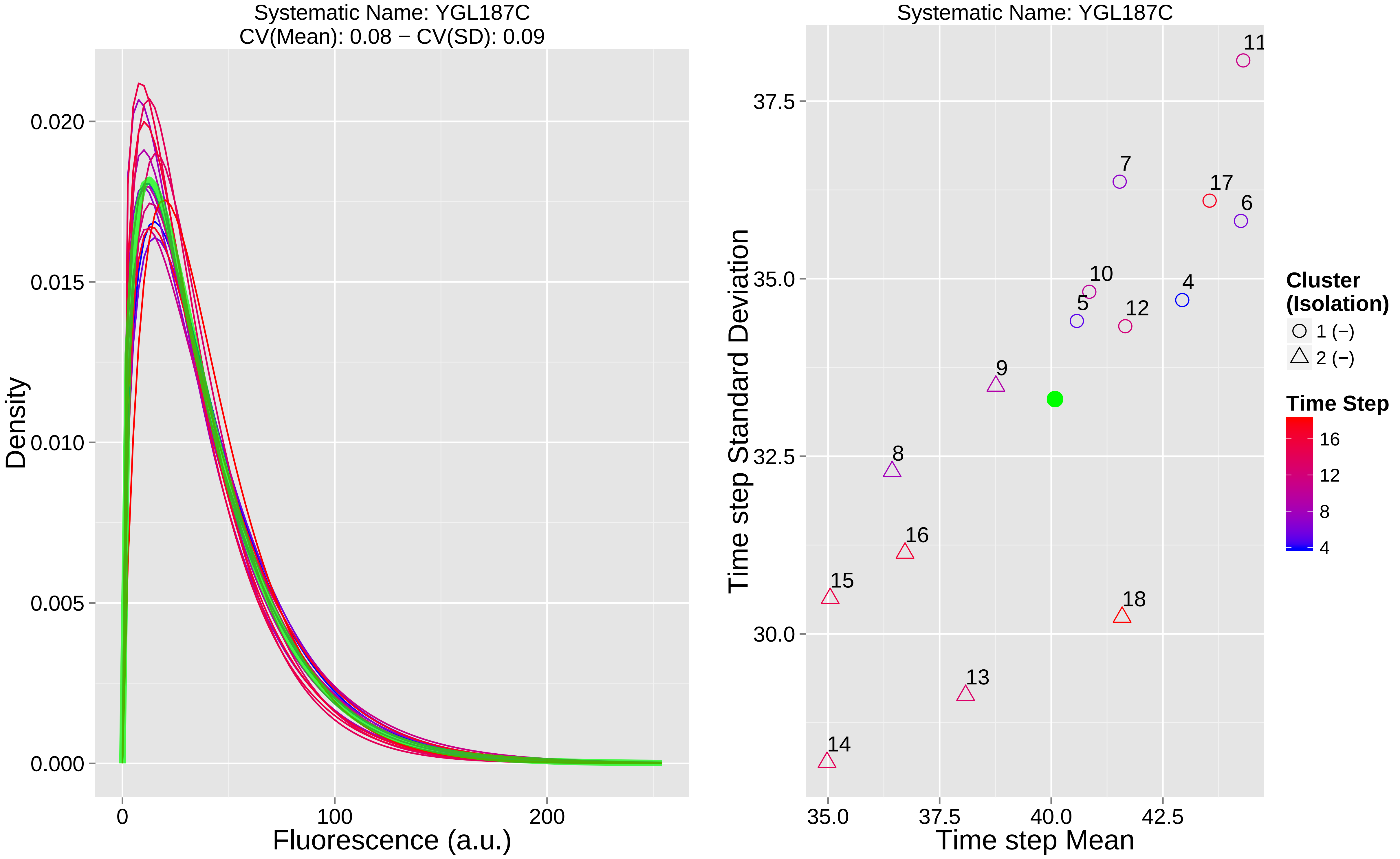

Systematic Name: YGL187C
CV(Mean): 0.08 − CV(SD): 0.09
Density
Fluorescence (a.u.)
0.000
0.005
0.010
0.015
0.020
0
100
200
Systematic Name: YGL187C
Time step Standard Deviation
Time step Mean
30.0
32.5
35.0
37.5
35.0
37.5
40.0
42.5
11
7
17
6
10
4
5
12
9
8
16
15
18
13
14
Cluster
(Isolation)
1 (−)
2 (−)
Time Step
16
12
8
4

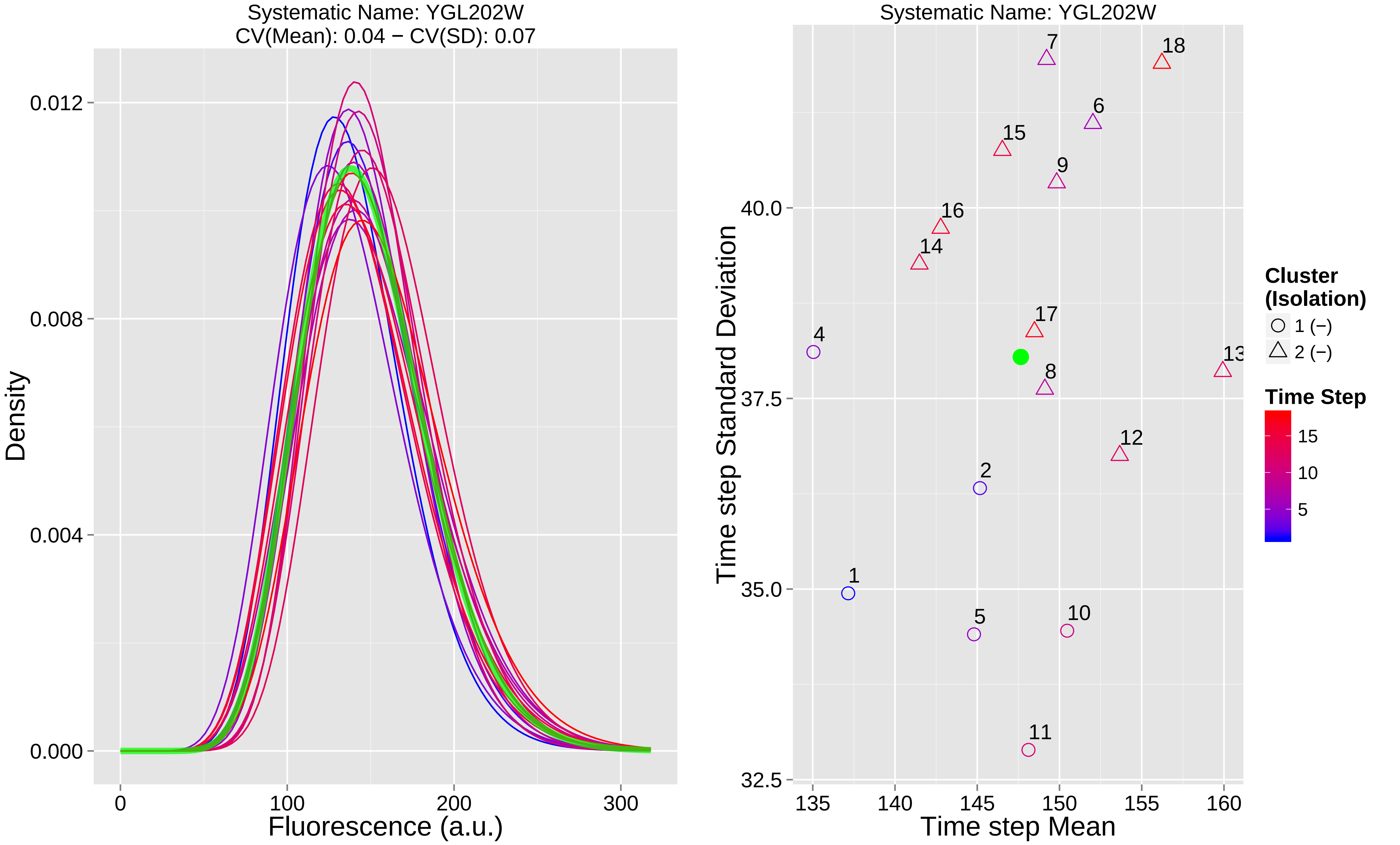

Systematic Name: YGL202W
CV(Mean): 0.04 − CV(SD): 0.07
Density
Fluorescence (a.u.)
0.000
0.004
0.008
0.012
0
100
200
300
Systematic Name: YGL202W
Time step Standard Deviation
Time step Mean
32.5
35.0
37.5
40.0
135
140
145
150
155
160
1
2
4
5
6
7
8
9
10
11
12
13
14
15
16
17
18
Cluster
(Isolation)
1 (−)
2 (−)
Time Step
5
10
15

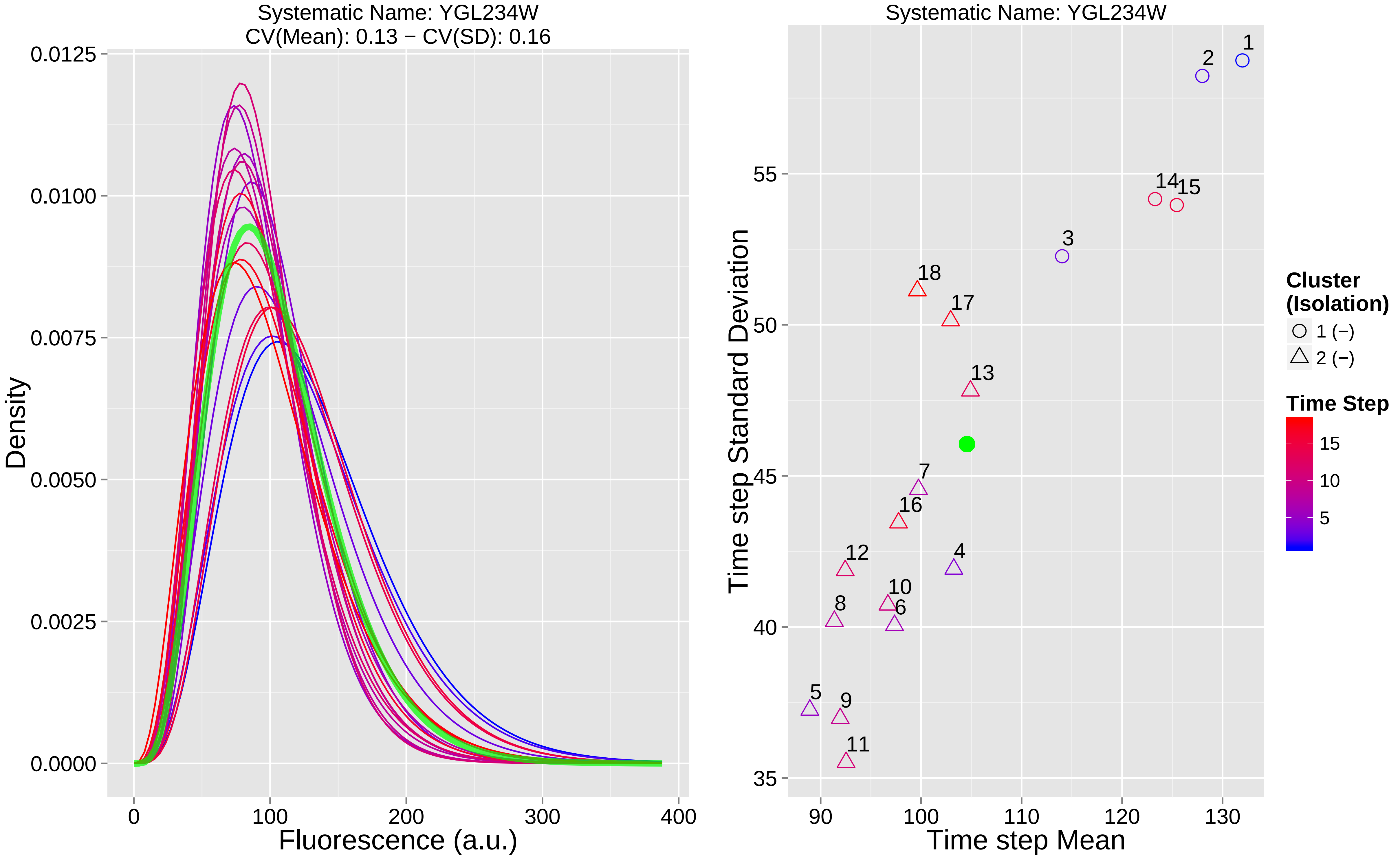

Systematic Name: YGL234W
CV(Mean): 0.13 − CV(SD): 0.16
Density
0.0125
0.0100
0.0075
0.0050
0.0025
0.0000
0
100
200
300
400
Fluorescence (a.u.)
Systematic Name: YGL234W
Time step Standard Deviation
55
50
45
40
35
90
100
110
120
130
Time step Mean
1
2
14
15
3
18
17
13
7
16
12
4
10
6
8
5
9
11
Cluster
(Isolation)
1 (−)
2 (−)
Time Step
15
10
5

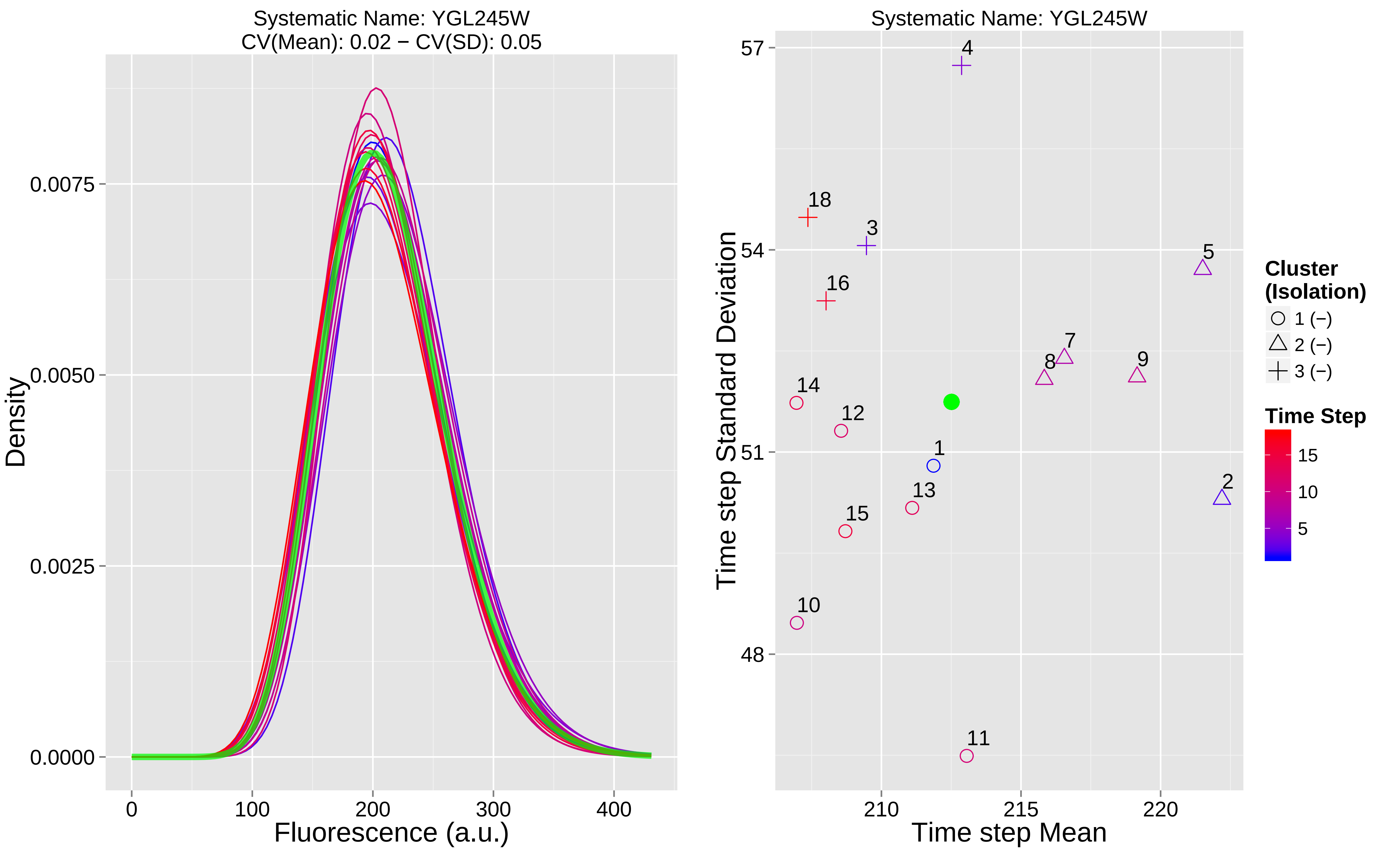

Systematic Name: YGL245W
CV(Mean): 0.02 − CV(SD): 0.05
Density
0.0075
0.0050
0.0025
0.0000
0
100
200
300
400
Fluorescence (a.u.)
Systematic Name: YGL245W
Time step Standard Deviation
57
54
51
48
210
215
220
Time step Mean
4
18
3
16
5
7
8
9
14
12
1
2
13
15
10
11
Cluster
(Isolation)
1 (−)
2 (−)
3 (−)
Time Step
15
10
5

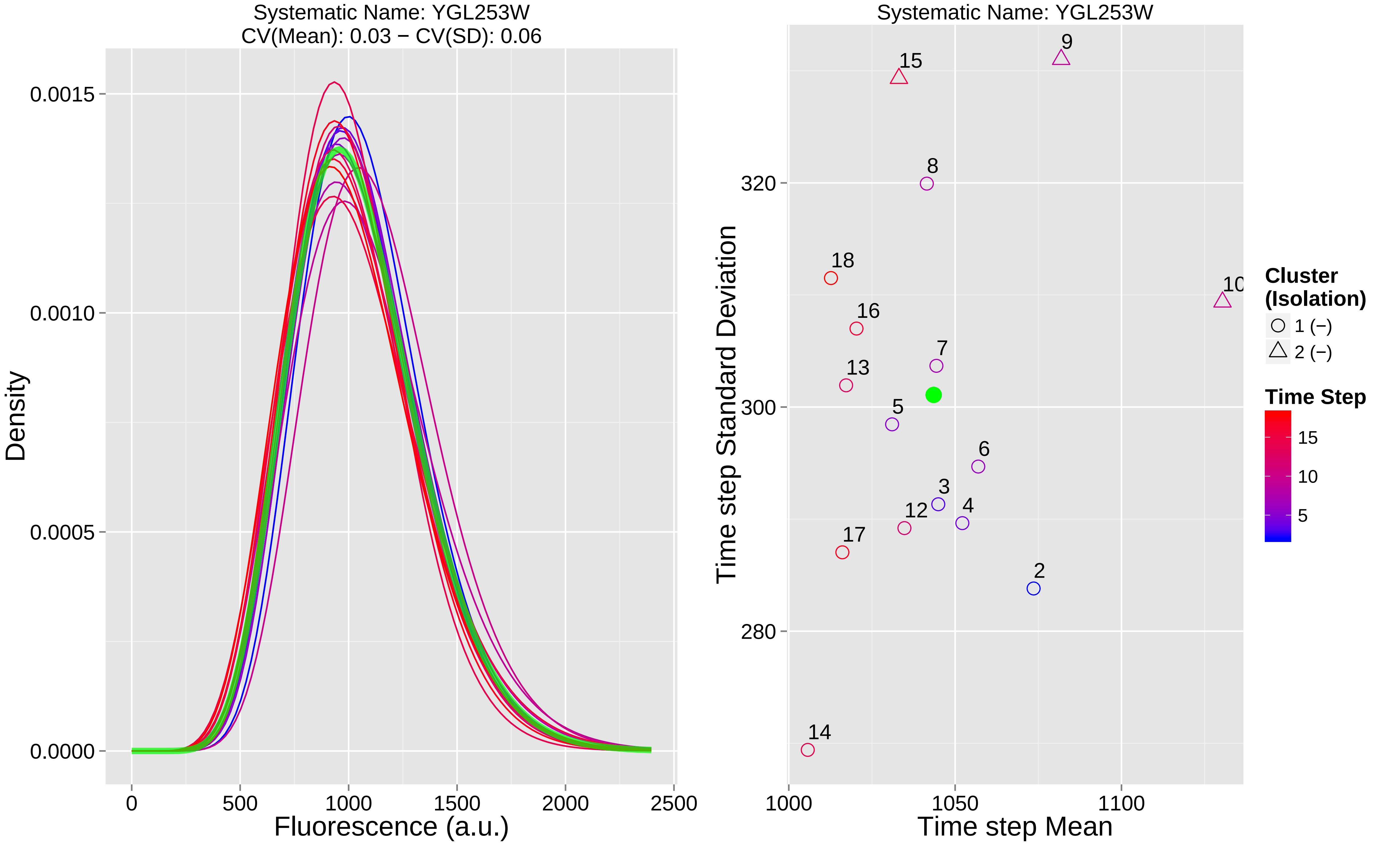

Systematic Name: YGL253W
CV(Mean): 0.03 − CV(SD): 0.06
Density
0.0015
0.0010
0.0005
0.0000
0
500
1000
1500
2000
2500
Fluorescence (a.u.)
Systematic Name: YGL253W
Time step Standard Deviation
320
300
280
1000
1050
1100
Time step Mean
Cluster (Isolation)
1 (−)
2 (−)
Time Step
15
10
5

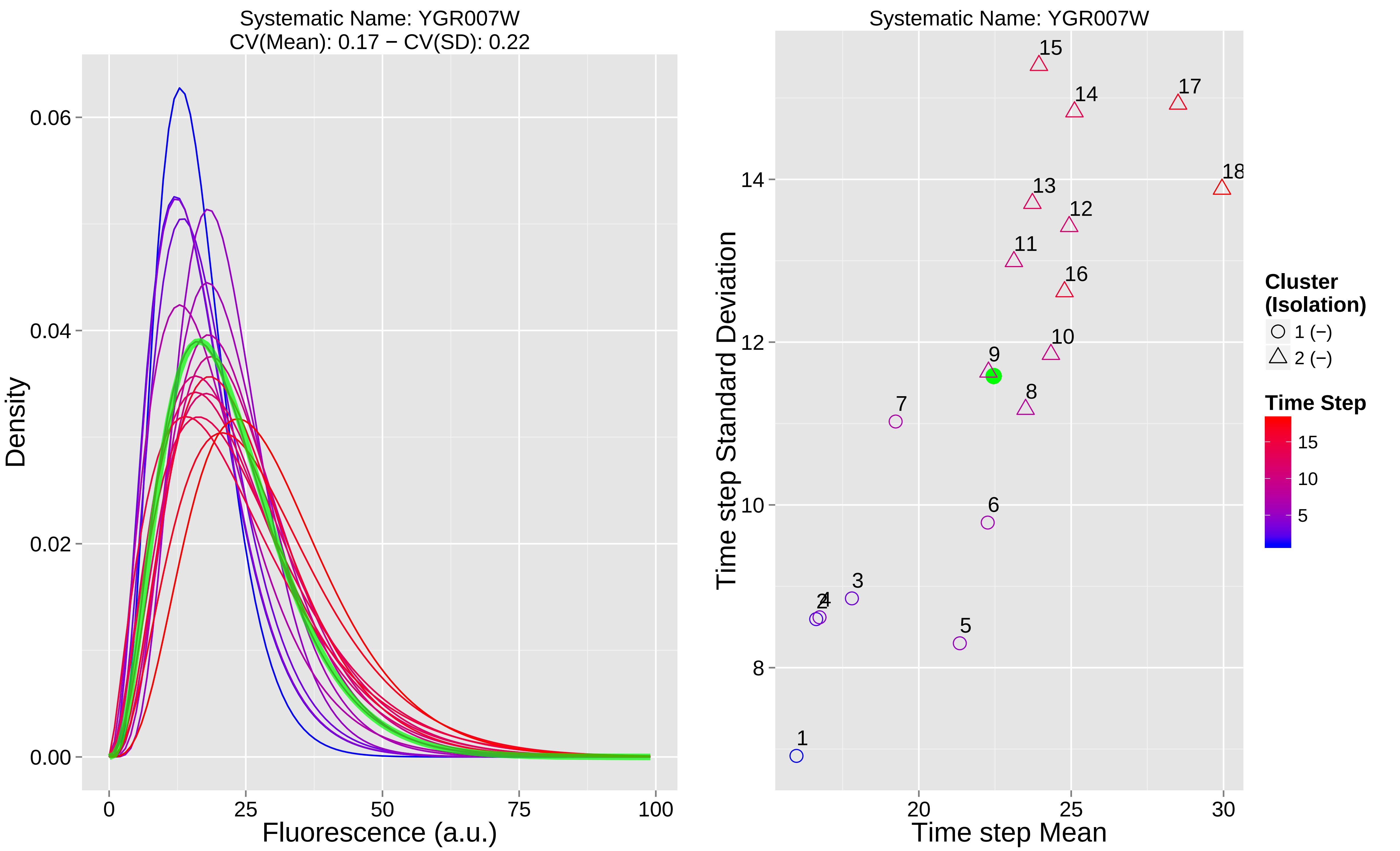

Systematic Name: YGR007W
CV(Mean): 0.17 − CV(SD): 0.22
Density
0.00
0.02
0.04
0.06
0
25
50
75
100
Fluorescence (a.u.)
Systematic Name: YGR007W
Time step Standard Deviation
8
10
12
14
20
25
30
Time step Mean
1
2
3
4
5
6
7
8
9
10
11
12
13
14
15
16
17
18
Cluster
(Isolation)
1 (−)
2 (−)
Time Step
5
10
15

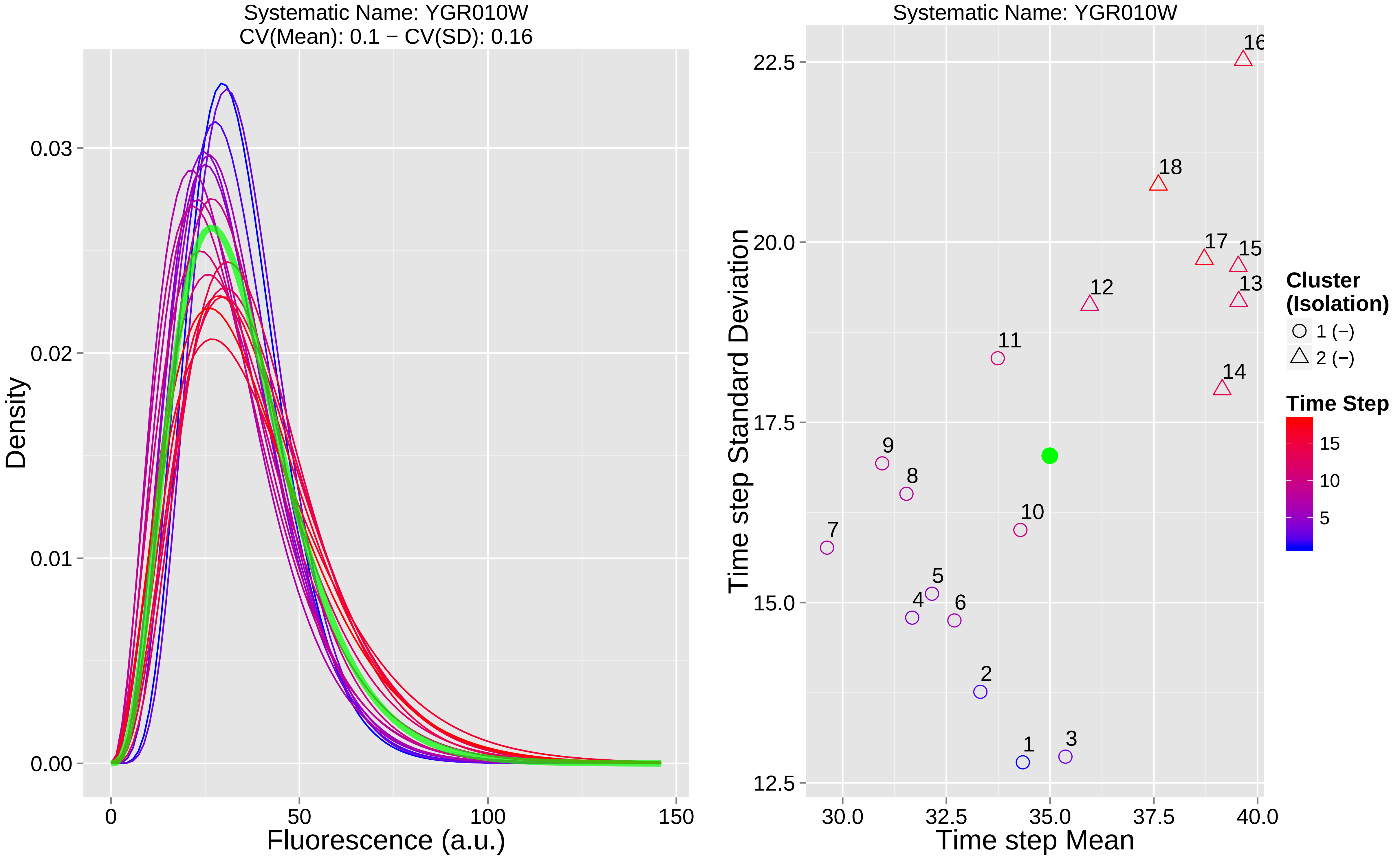

Systematic Name: YGR010W
CV(Mean): 0.1 − CV(SD): 0.16
Density
Fluorescence (a.u.)
0.00
0.01
0.02
0.03
0
50
100
150
Systematic Name: YGR010W
Time step Standard Deviation
Time step Mean
12.5
15.0
17.5
20.0
22.5
30.0
32.5
35.0
37.5
40.0
Cluster (Isolation)
1 (−)
2 (−)
Time Step
5
10
15

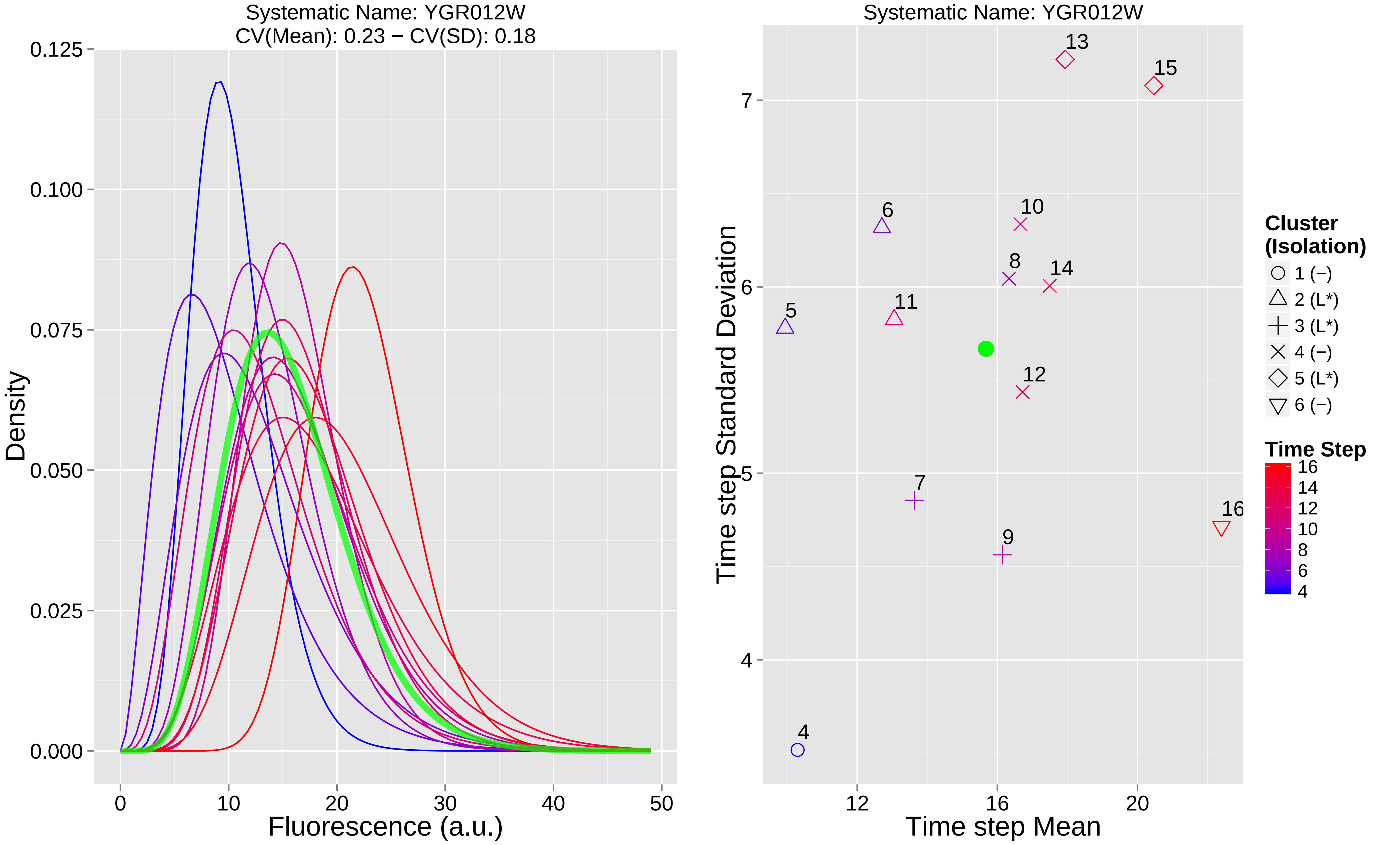

Systematic Name: YGR012W
CV(Mean): 0.23 − CV(SD): 0.18
Density
Fluorescence (a.u.)
0.125
0.100
0.075
0.050
0.025
0.000
0
10
20
30
40
50
Systematic Name: YGR012W
Time step Standard Deviation
Time step Mean
7
6
5
4
12
16
20
13
15
6
10
8
14
5
11
12
7
16
9
4
Cluster
(Isolation)
1 (−)
2 (L*)
3 (L*)
4 (−)
5 (L*)
6 (−)
Time Step
16
14
12
10
8
6
4

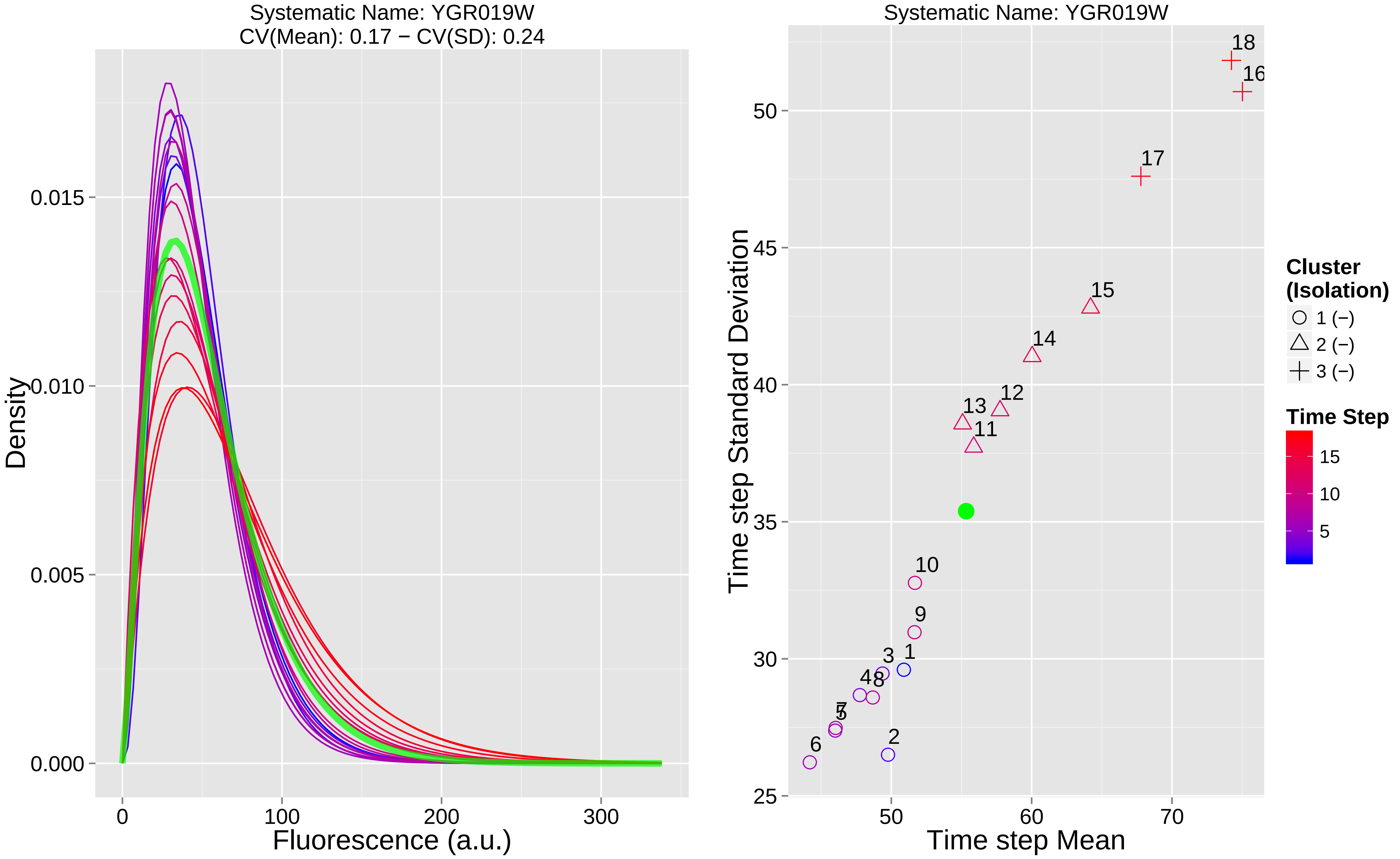

Systematic Name: YGR019W
CV(Mean): 0.17 − CV(SD): 0.24
Density
0.000
0.005
0.010
0.015
0
100
200
300
Fluorescence (a.u.)
Systematic Name: YGR019W
Time step Standard Deviation
25
30
35
40
45
50
50
60
70
Time step Mean
18
16
17
15
14
13
12
11
10
9
3
1
48
7
5
6
2
Cluster
(Isolation)
1 (−)
2 (−)
3 (−)
Time Step
15
10
5

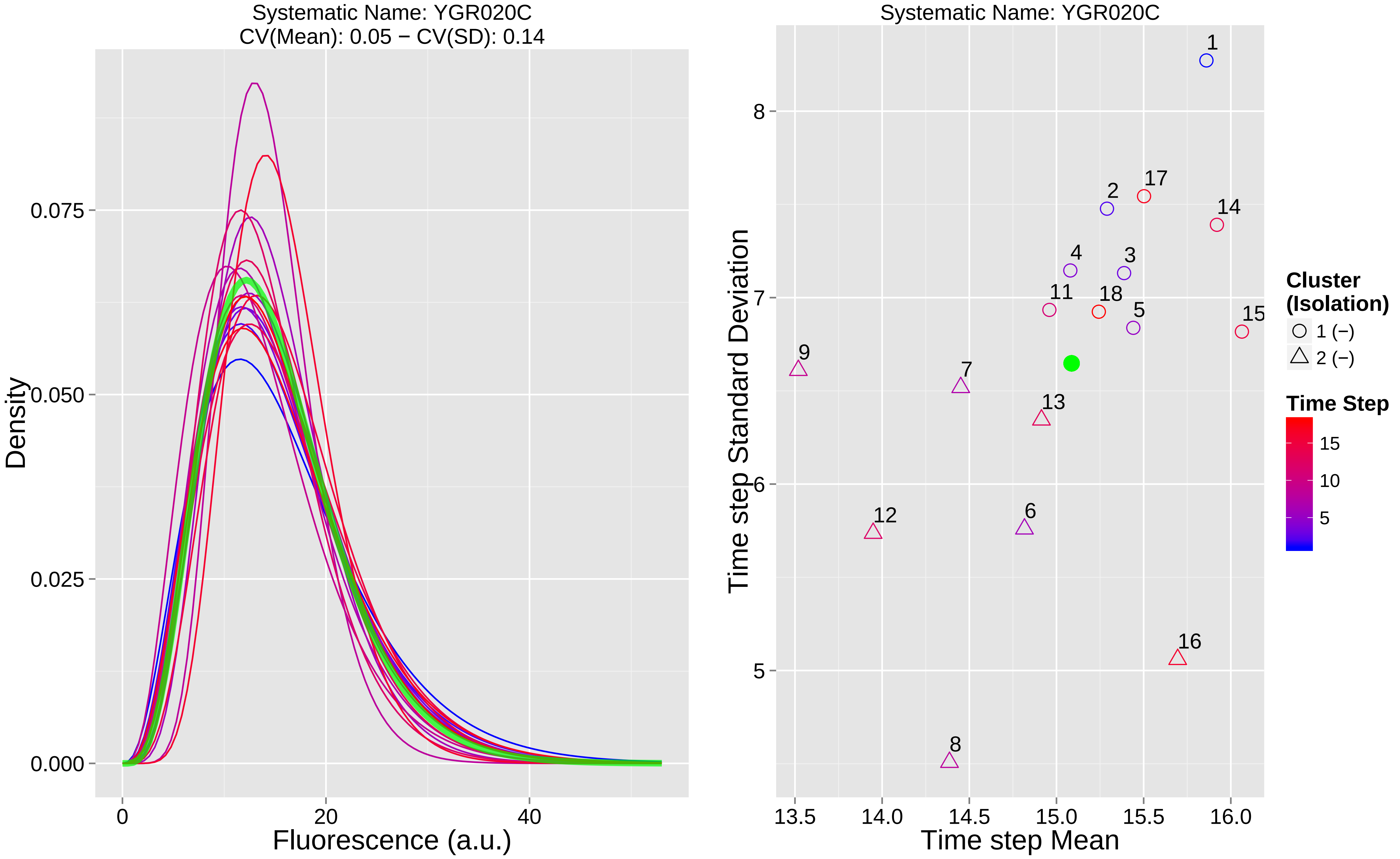
Systematic Name: YGR020C
CV(Mean): 0.05 − CV(SD): 0.14
Density
0.000
0.025
0.050
0.075
0
20
40
Fluorescence (a.u.)
Systematic Name: YGR020C
Time step Standard Deviation
5
6
7
8
13.5
14.0
14.5
15.0
15.5
16.0
Time step Mean
1
2
3
4
5
6
7
8
9
11
12
13
14
15
16
17
18
Cluster
(Isolation)
1 (−)
2 (−)
Time Step
5
10
15

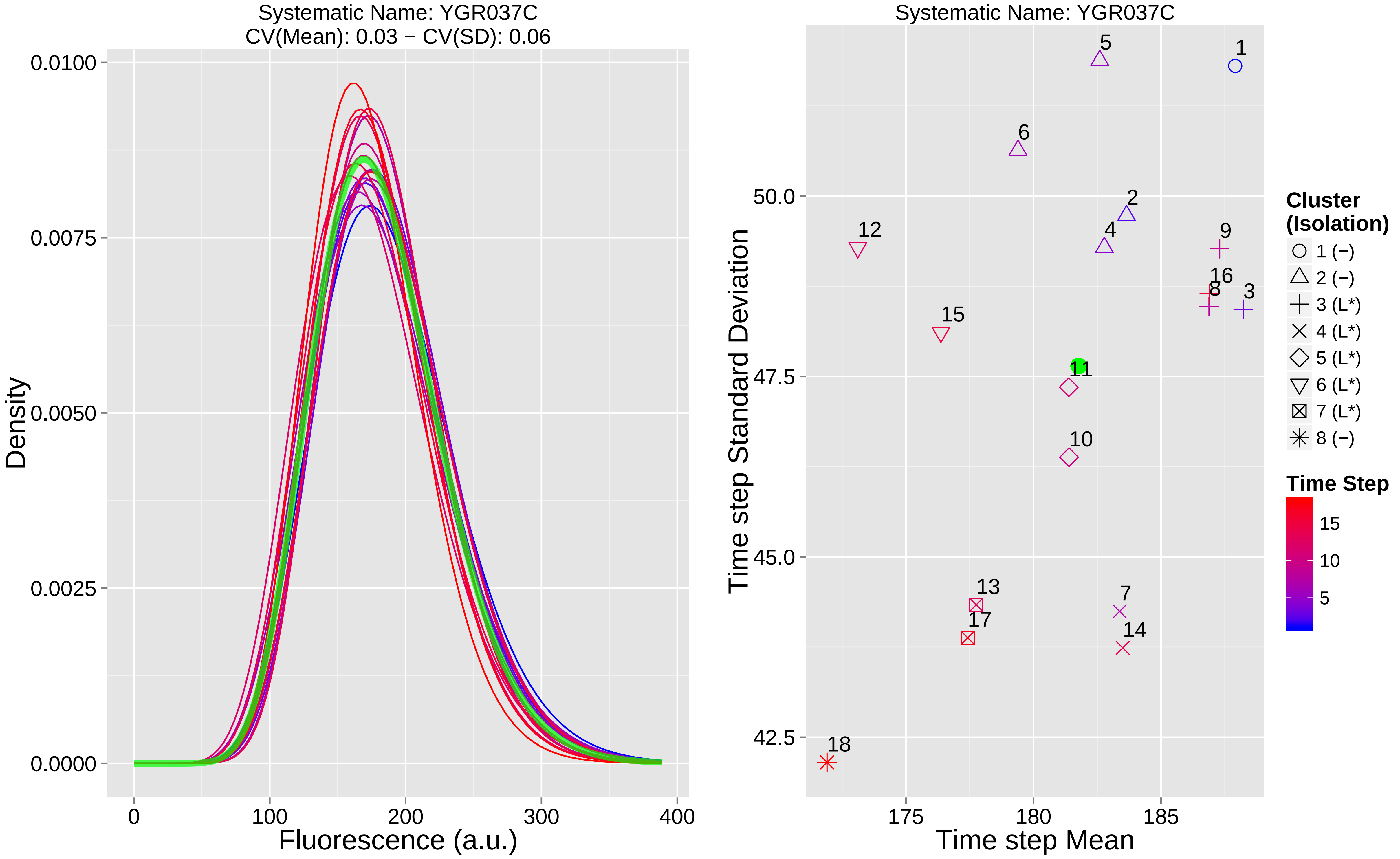
Systematic Name: YGR037C
CV(Mean): 0.03 − CV(SD): 0.06
Density
Fluorescence (a.u.)
0.0100
0.0075
0.0050
0.0025
0.0000
0
100
200
300
400
Systematic Name: YGR037C
Time step Standard Deviation
Time step Mean
50.0
47.5
45.0
42.5
175
180
185
Cluster (Isolation)
1 (−)
2 (−)
3 (L*)
4 (L*)
5 (L*)
6 (L*)
7 (L*)
8 (−)
Time Step
15
10
5

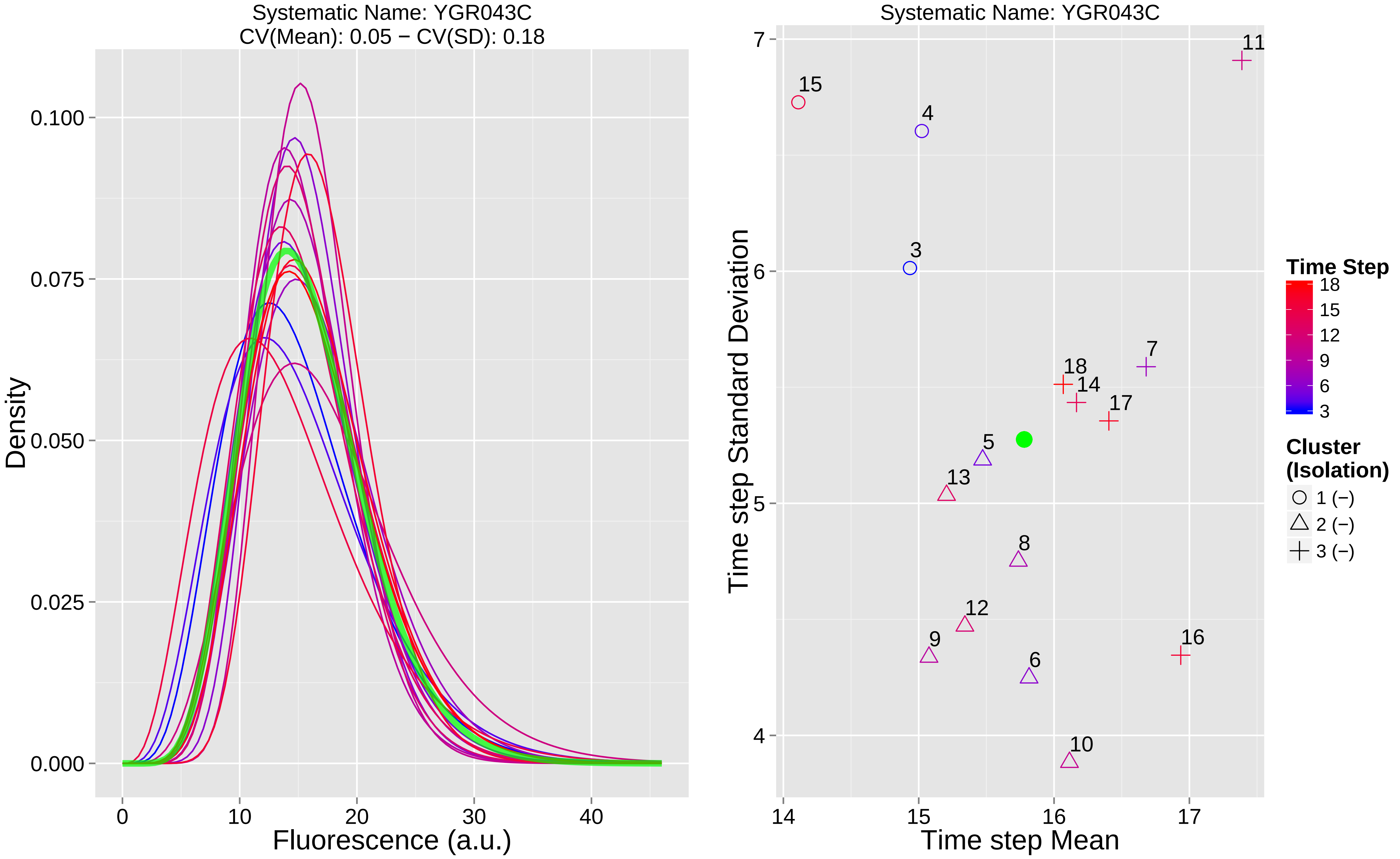

Systematic Name: YGR043C
CV(Mean): 0.05 − CV(SD): 0.18
Density
0.000
0.025
0.050
0.075
0.100
0
10
20
30
40
Fluorescence (a.u.)
Systematic Name: YGR043C
Time step Standard Deviation
4
5
6
7
14
15
16
17
Time step Mean
15
4
3
11
7
18
14
17
5
13
8
12
9
6
16
10
Time Step
18
15
12
9
6
3
Cluster
(Isolation)
1 (−)
2 (−)
3 (−)

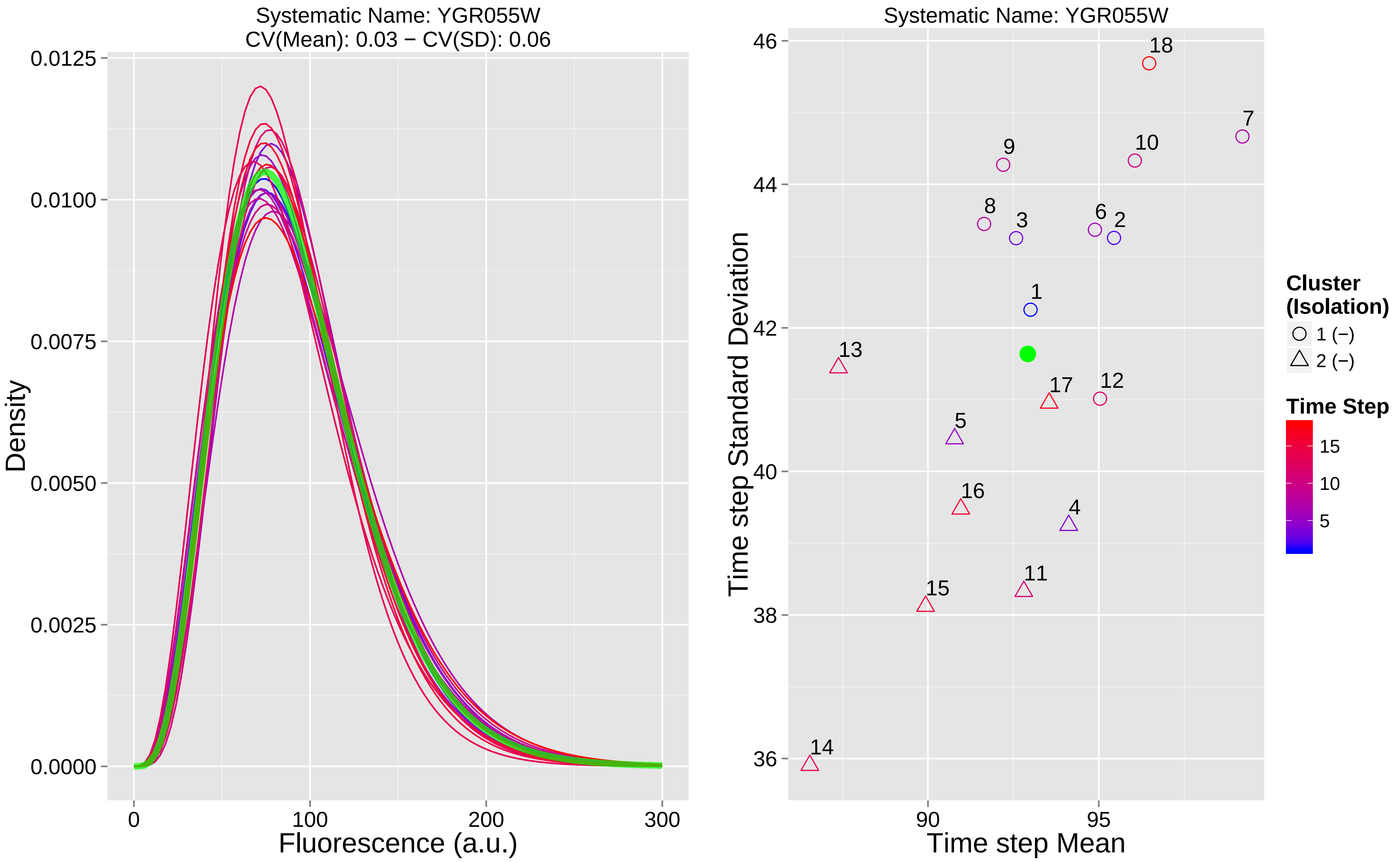

Systematic Name: YGR055W
CV(Mean): 0.03 − CV(SD): 0.06
Density
Fluorescence (a.u.)
0.0125
0.0100
0.0075
0.0050
0.0025
0.0000
0
100
200
300
Systematic Name: YGR055W
Time step Standard Deviation
Time step Mean
46
44
42
40
38
36
90
95
18
7
9
10
8
3
6
2
1
13
17
12
5
16
4
11
15
14
Cluster (Isolation)
1 (−)
2 (−)
Time Step
15
10
5

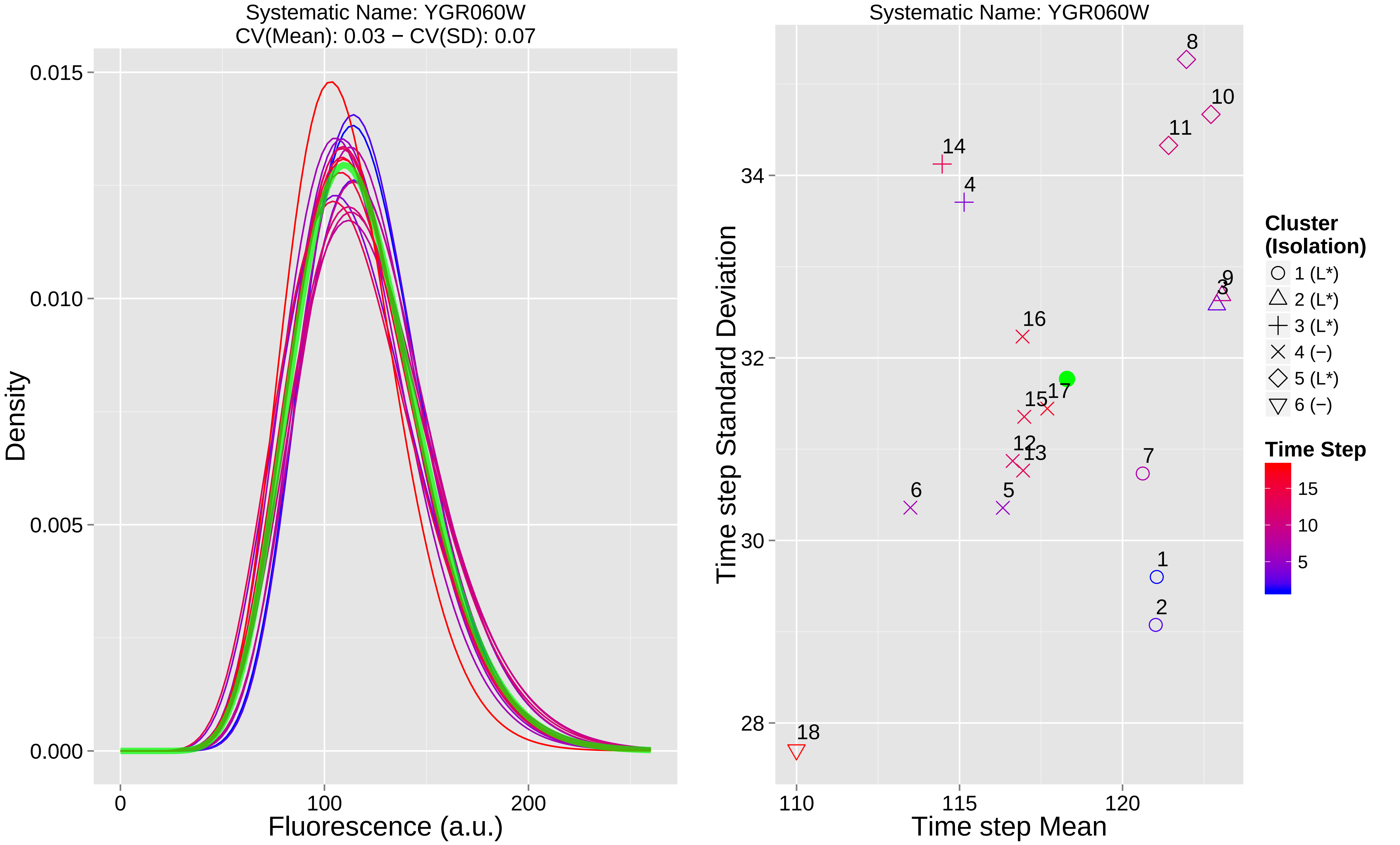
Systematic Name: YGR060W
CV(Mean): 0.03 − CV(SD): 0.07
Density
0.015
0.010
0.005
0.000
0
100
200
Fluorescence (a.u.)
Systematic Name: YGR060W
Time step Standard Deviation
Time step Mean
34
32
30
28
110
115
120
Cluster (Isolation)
1 (L*)
2 (L*)
3 (L*)
4 (−)
5 (L*)
6 (−)
Time Step
15
10
5

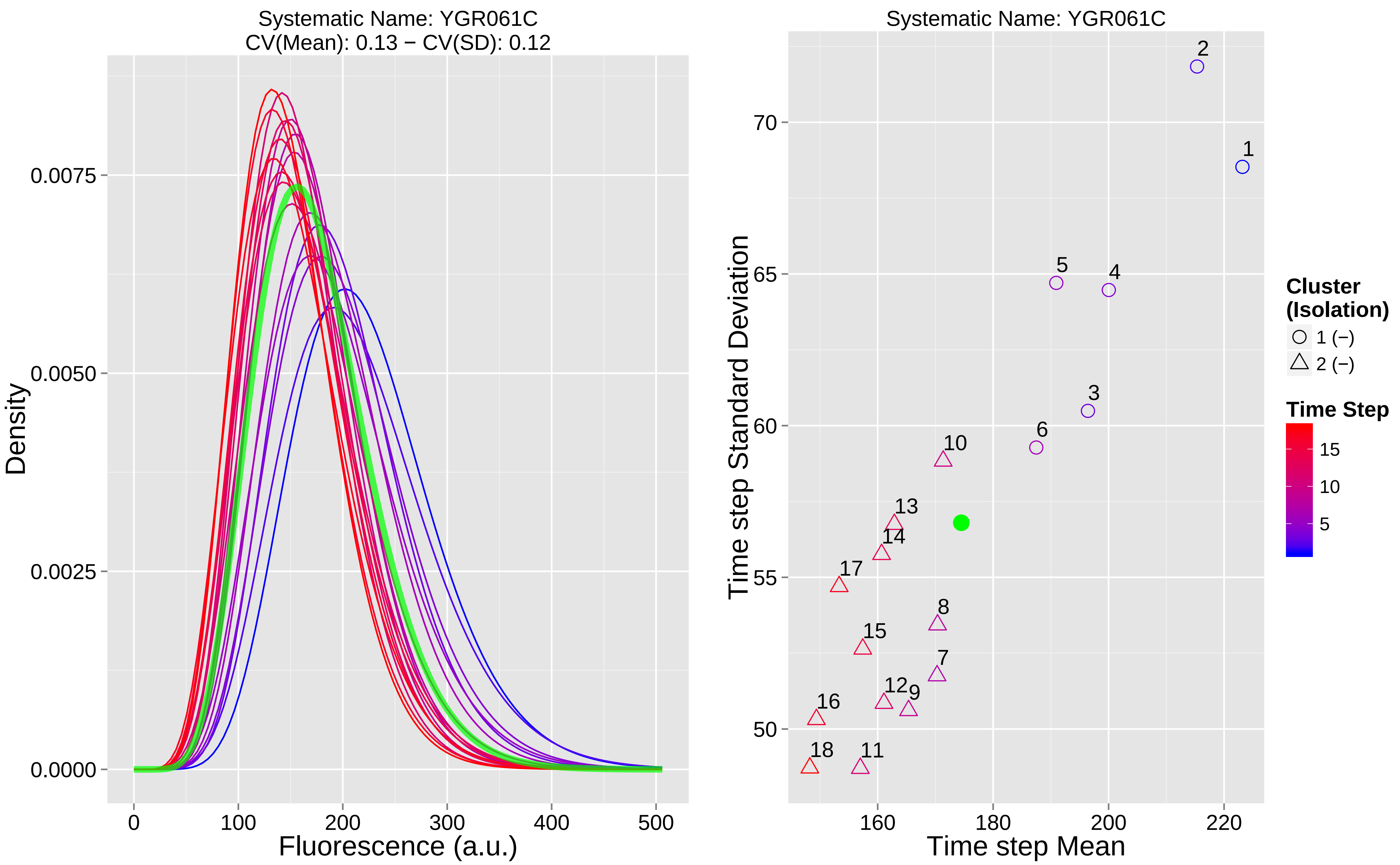

Systematic Name: YGR061C
CV(Mean): 0.13 − CV(SD): 0.12
Density
0.0075
0.0050
0.0025
0.0000
0
100
200
300
400
500
Fluorescence (a.u.)
Systematic Name: YGR061C
Time step Standard Deviation
70
65
60
55
50
160
180
200
220
Time step Mean
1
2
3
4
5
6
7
8
9
10
11
12
13
14
15
16
17
18
Cluster (Isolation)
1 (−)
2 (−)
Time Step
15
10
5

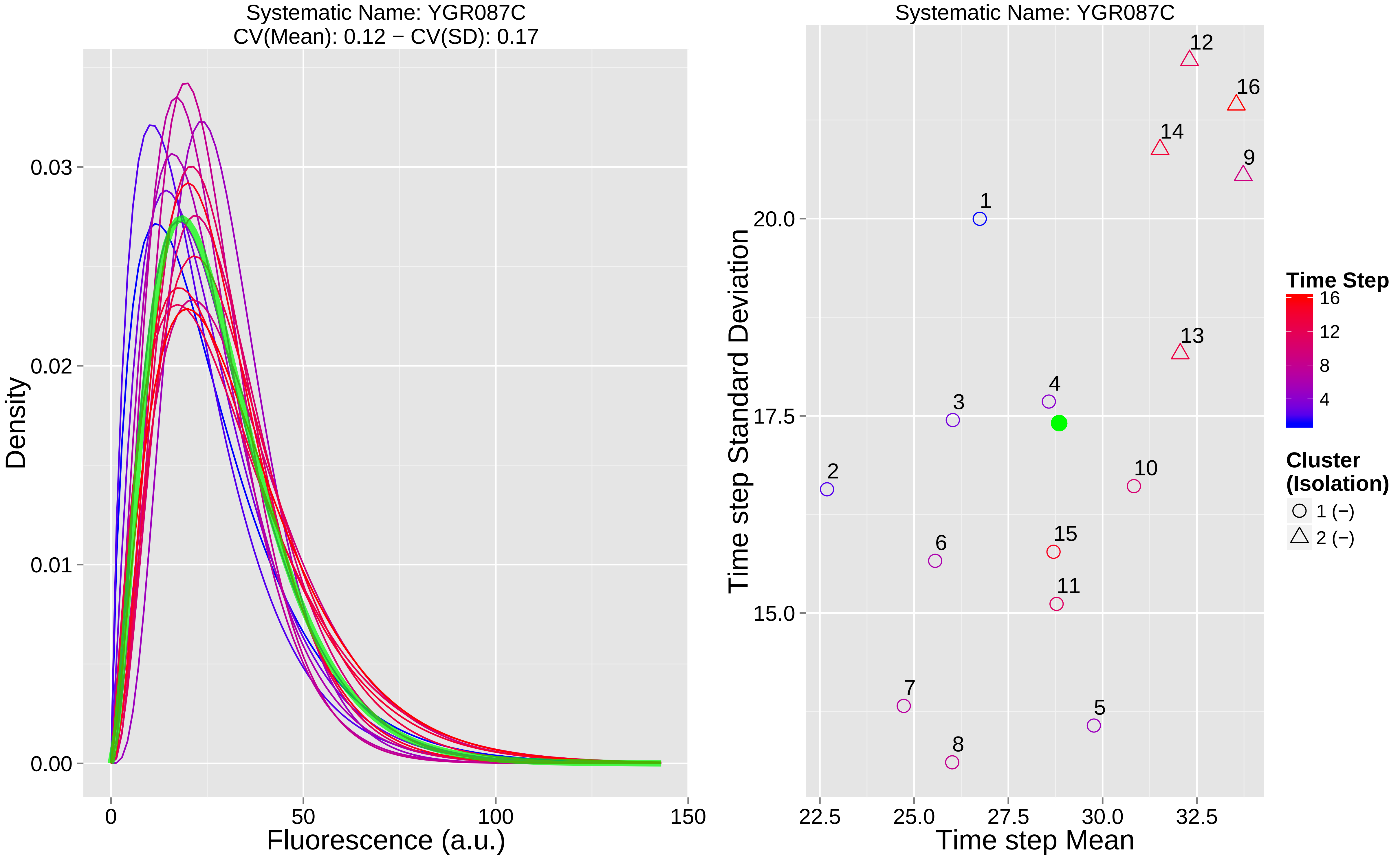

Systematic Name: YGR087C
CV(Mean): 0.12 − CV(SD): 0.17
Density
0.03
0.02
0.01
0.00
0
50
100
150
Fluorescence (a.u.)
Systematic Name: YGR087C
Time step Standard Deviation
20.0
17.5
15.0
22.5
25.0
27.5
30.0
32.5
Time step Mean
1
2
3
4
5
6
7
8
9
10
11
12
13
14
15
16
Time Step
16
12
8
4
Cluster (Isolation)
1 (−)
2 (−)

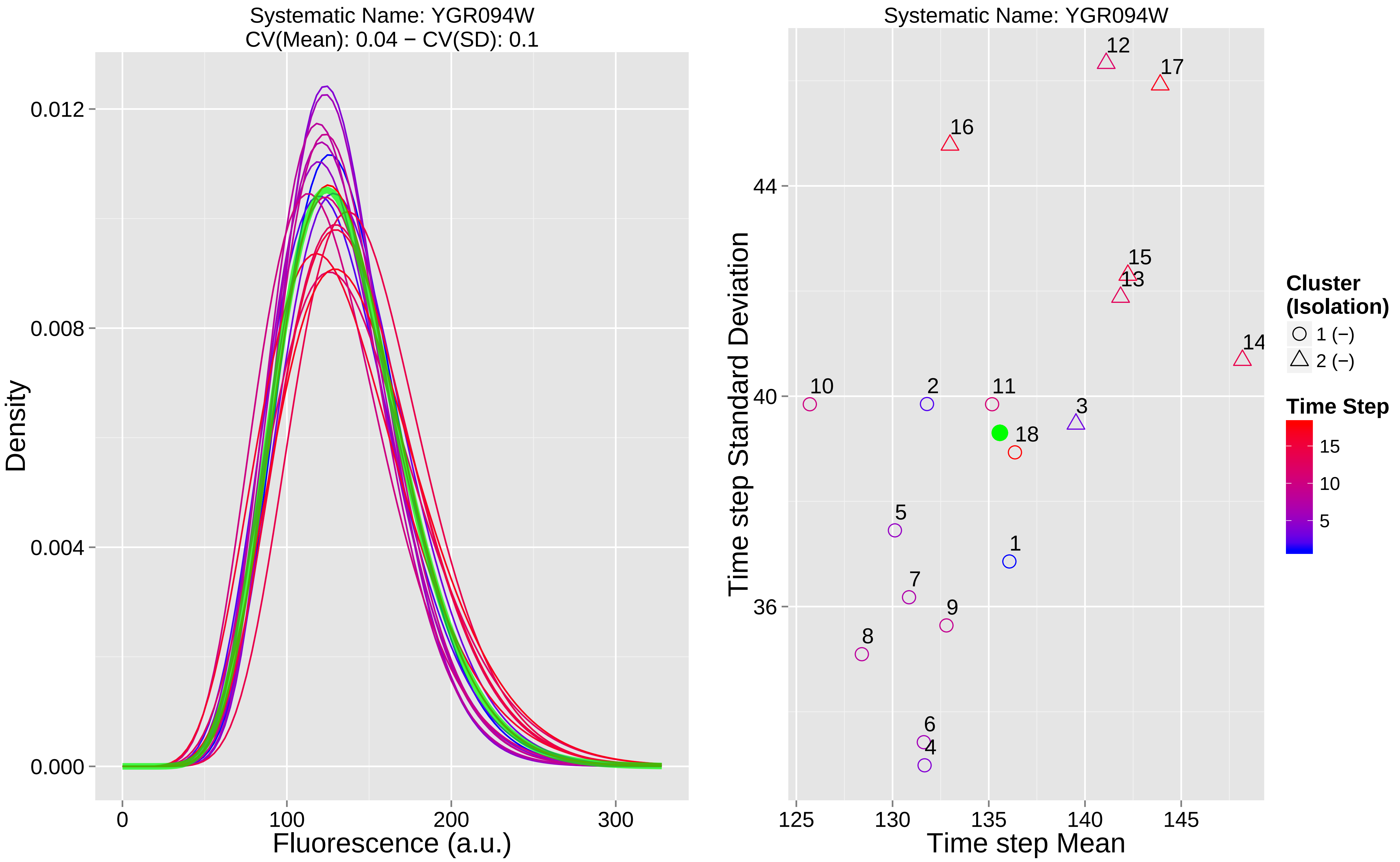

Systematic Name: YGR094W
CV(Mean): 0.04 − CV(SD): 0.1
Density
Fluorescence (a.u.)
0.000
0.004
0.008
0.012
0
100
200
300
Systematic Name: YGR094W
Time step Standard Deviation
Time step Mean
36
40
44
125
130
135
140
145
1
2
3
4
5
6
7
8
9
10
11
12
13
14
15
16
17
18
Cluster (Isolation)
1 (−)
2 (−)
Time Step
15
10
5

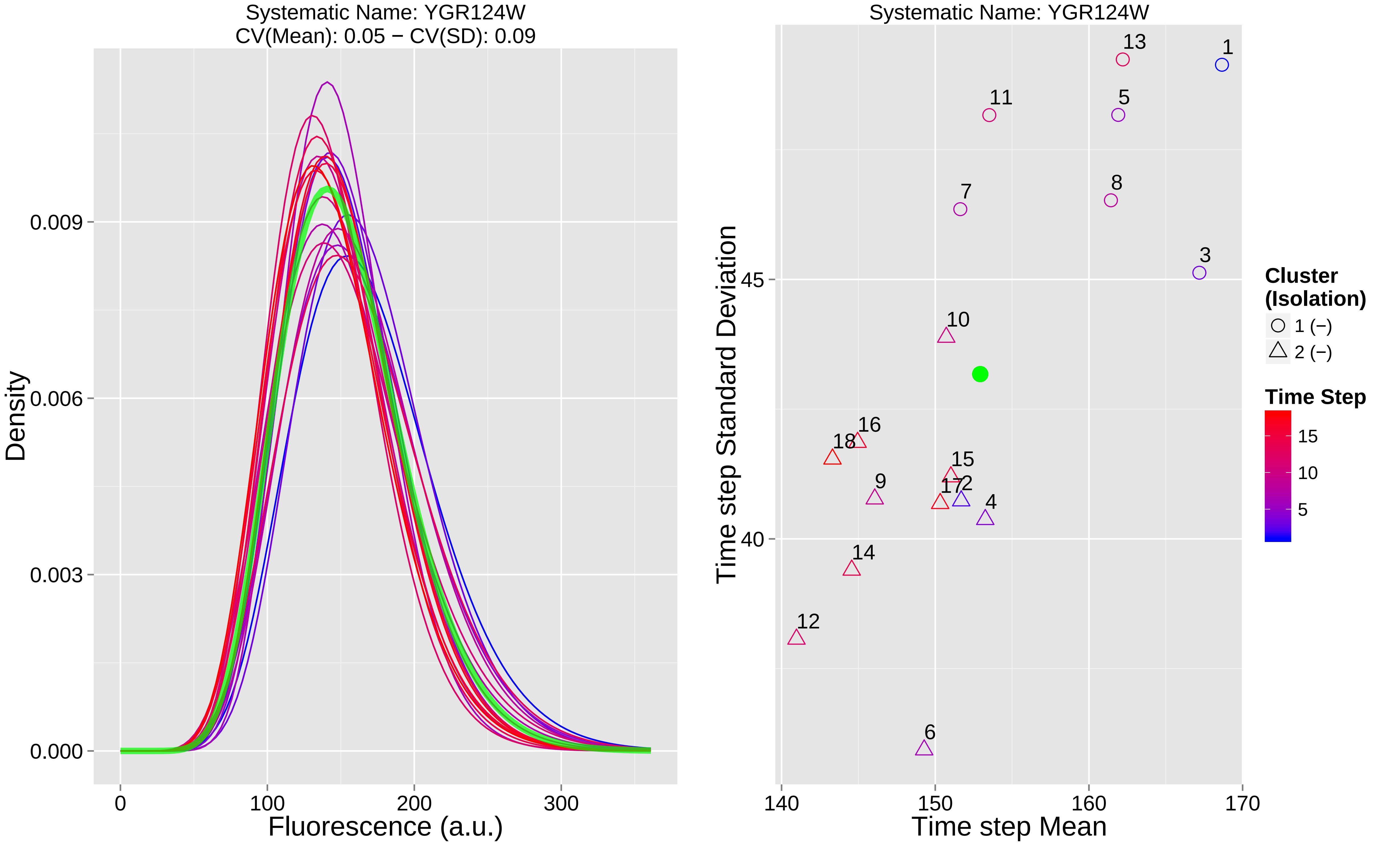

Systematic Name: YGR124W
CV(Mean): 0.05 − CV(SD): 0.09
Density
0.000
0.003
0.006
0.009
0
100
200
300
Fluorescence (a.u.)
Systematic Name: YGR124W
Time step Standard Deviation
40
45
140
150
160
170
Time step Mean
13
1
11
5
7
8
3
10
16
18
15
9
17
2
4
14
12
6
Cluster
(Isolation)
1 (−)
2 (−)
Time Step
15
10
5

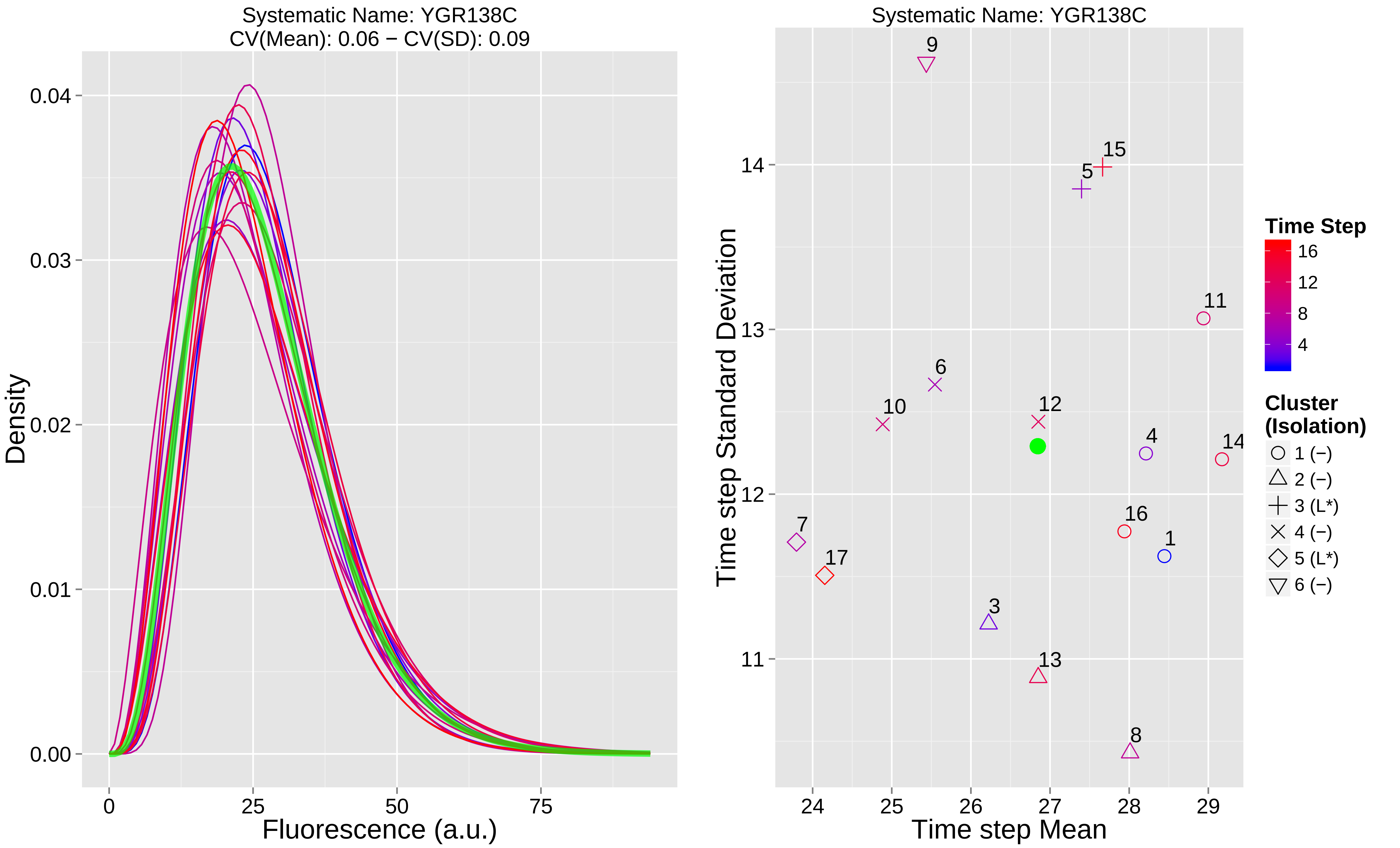

Systematic Name: YGR138C
CV(Mean): 0.06 − CV(SD): 0.09
Density
Fluorescence (a.u.)
0.04
0.03
0.02
0.01
0.00
0
25
50
75
Systematic Name: YGR138C
Time step Standard Deviation
Time step Mean
14
13
12
11
24
25
26
27
28
29
9
15
5
11
6
10
12
4
14
16
1
7
17
3
13
8
Time Step
16
12
8
4
Cluster
(Isolation)
1 (−)
2 (−)
3 (L*)
4 (−)
5 (L*)
6 (−)

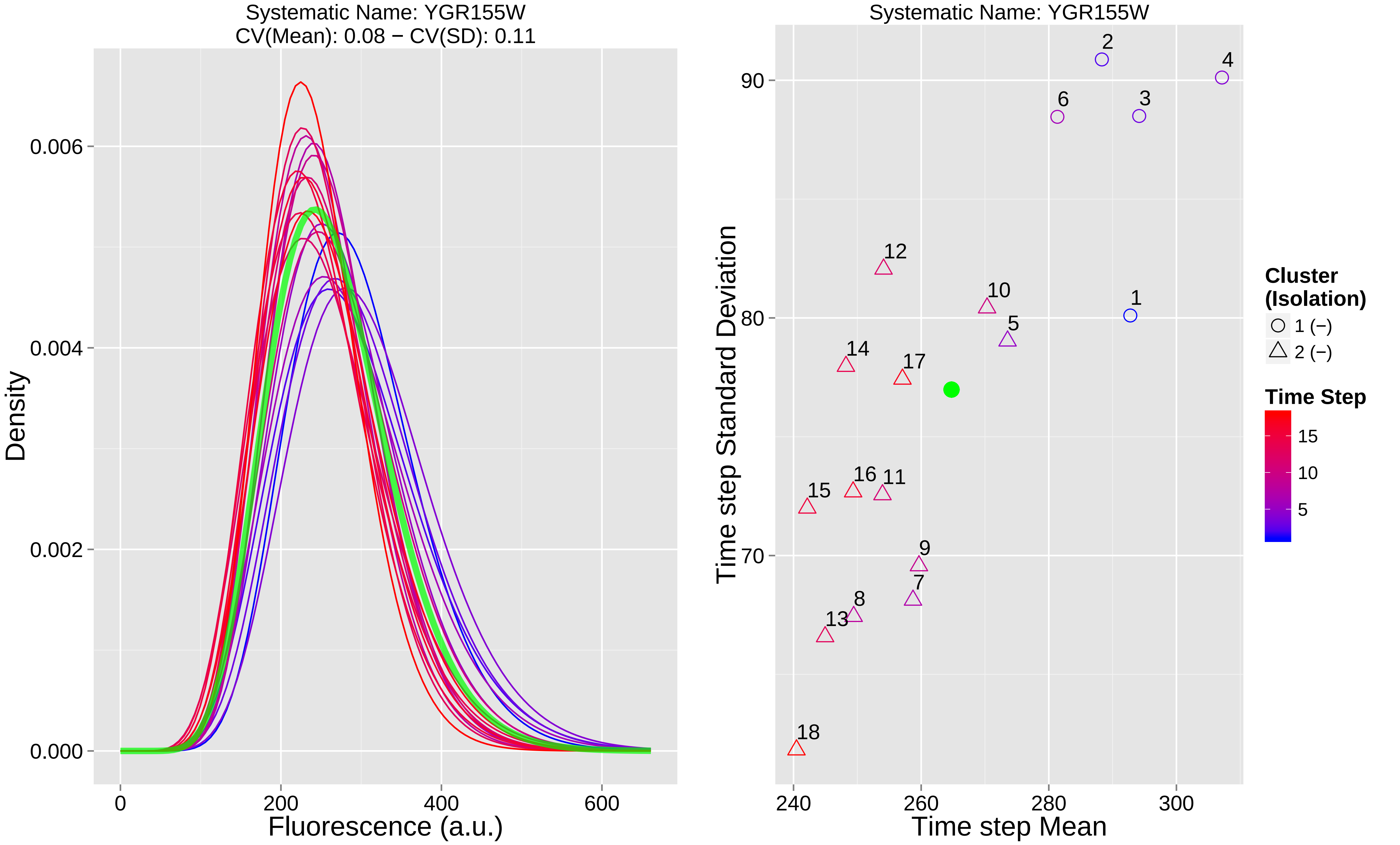
Systematic Name: YGR155W
CV(Mean): 0.08 − CV(SD): 0.11
Density
0.000
0.002
0.004
0.006
0
200
400
600
Fluorescence (a.u.)
Systematic Name: YGR155W
Time step Standard Deviation
70
80
90
240
260
280
300
Time step Mean
1
2
3
4
5
6
7
8
9
10
11
12
13
14
15
16
17
18
Cluster
(Isolation)
1 (−)
2 (−)
Time Step
15
10
5

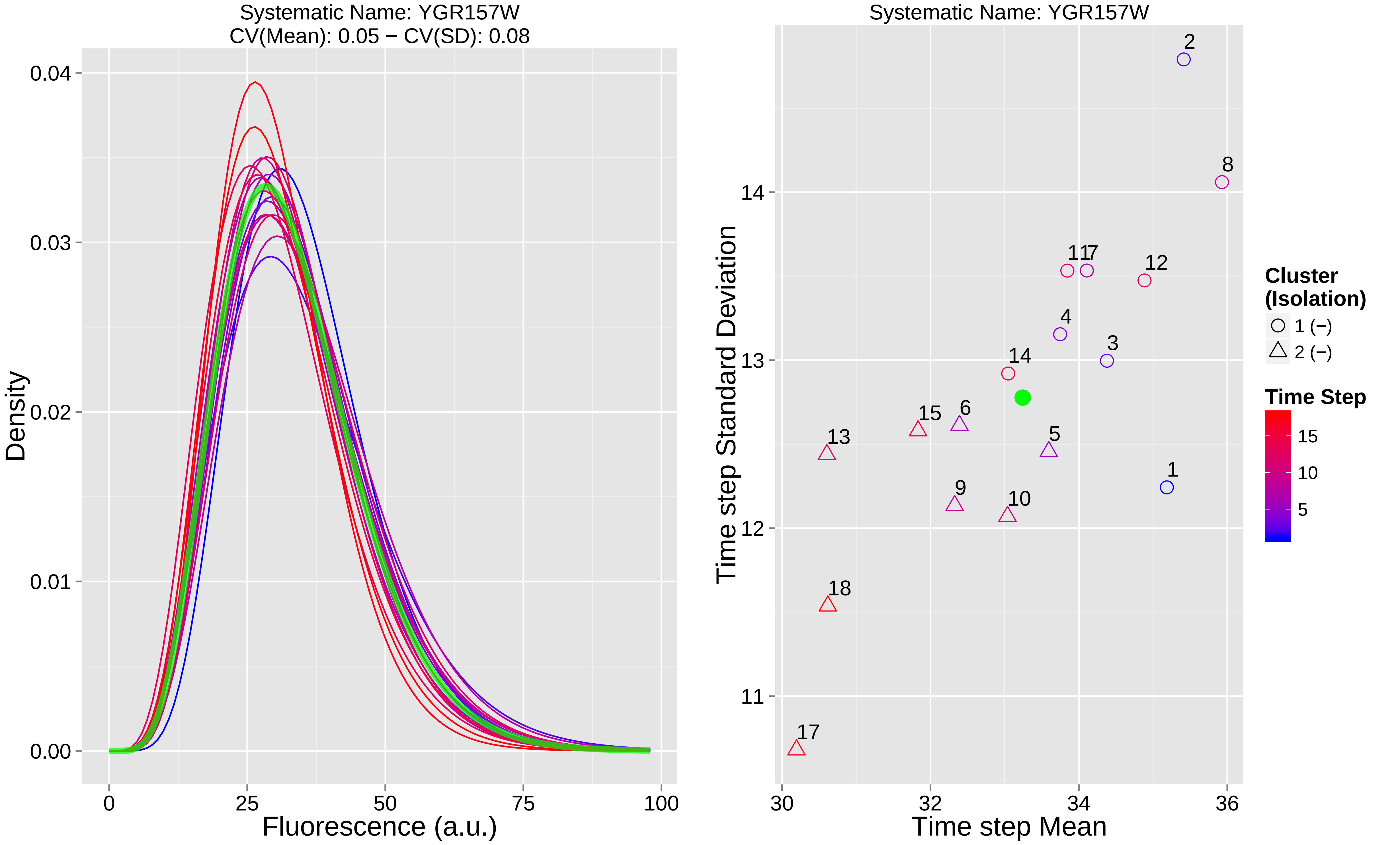

Systematic Name: YGR157W
CV(Mean): 0.05 − CV(SD): 0.08
Density
Fluorescence (a.u.)
0.00
0.01
0.02
0.03
0.04
0
25
50
75
100
Systematic Name: YGR157W
Time step Standard Deviation
Time step Mean
11
12
13
14
30
32
34
36
1
2
3
4
5
6
7
8
9
10
11
12
13
14
15
17
18
Cluster
(Isolation)
1 (−)
2 (−)
Time Step
15
10
5

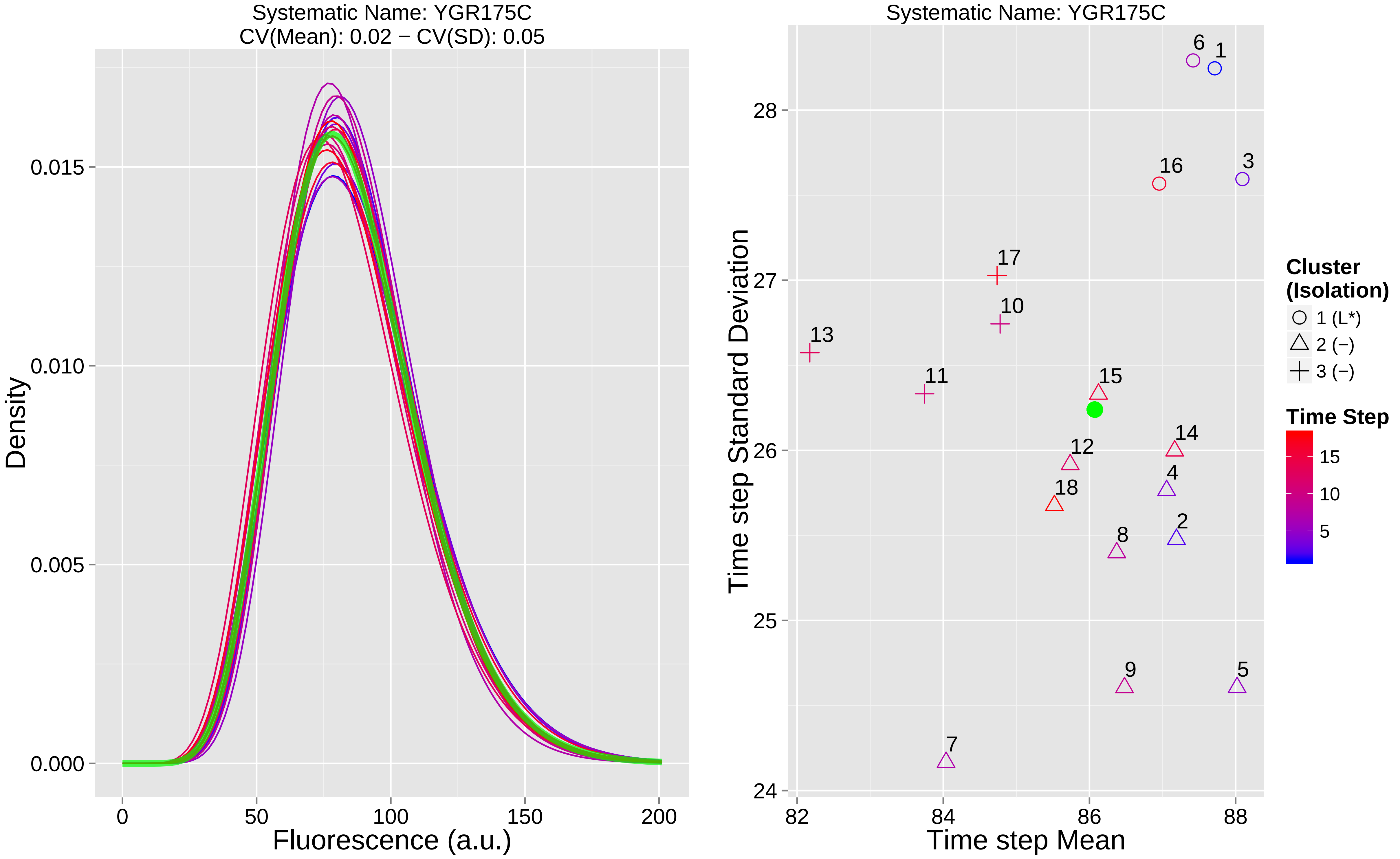
Systematic Name: YGR175C
CV(Mean): 0.02 − CV(SD): 0.05
Density
0.015
0.010
0.005
0.000
0
50
100
150
200
Fluorescence (a.u.)
Systematic Name: YGR175C
Time step Standard Deviation
28
27
26
25
24
82
84
86
88
Time step Mean
6
1
16
3
17
10
13
11
15
14
12
4
18
2
8
9
5
7
Cluster (Isolation)
1 (L*)
2 (−)
3 (−)
Time Step
15
10
5

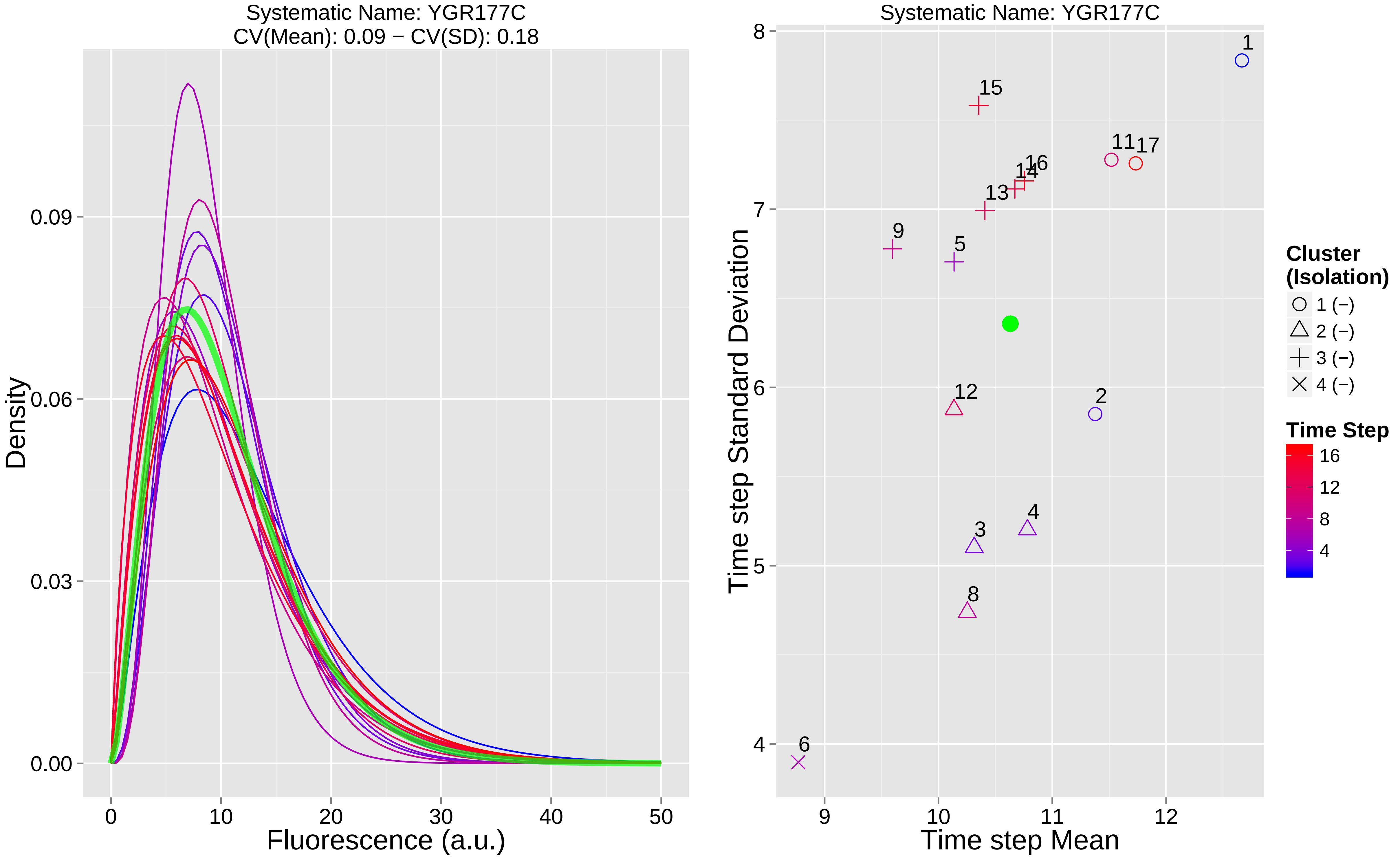

Systematic Name: YGR177C
CV(Mean): 0.09 − CV(SD): 0.18
Density
Fluorescence (a.u.)
0.00
0.03
0.06
0.09
0
10
20
30
40
50
Systematic Name: YGR177C
Time step Standard Deviation
Time step Mean
4
5
6
7
8
9
10
11
12
1
2
3
4
5
6
8
9
11
12
13
14
15
16
17
Cluster (Isolation)
1 (−)
2 (−)
3 (−)
4 (−)
Time Step
16
12
8
4

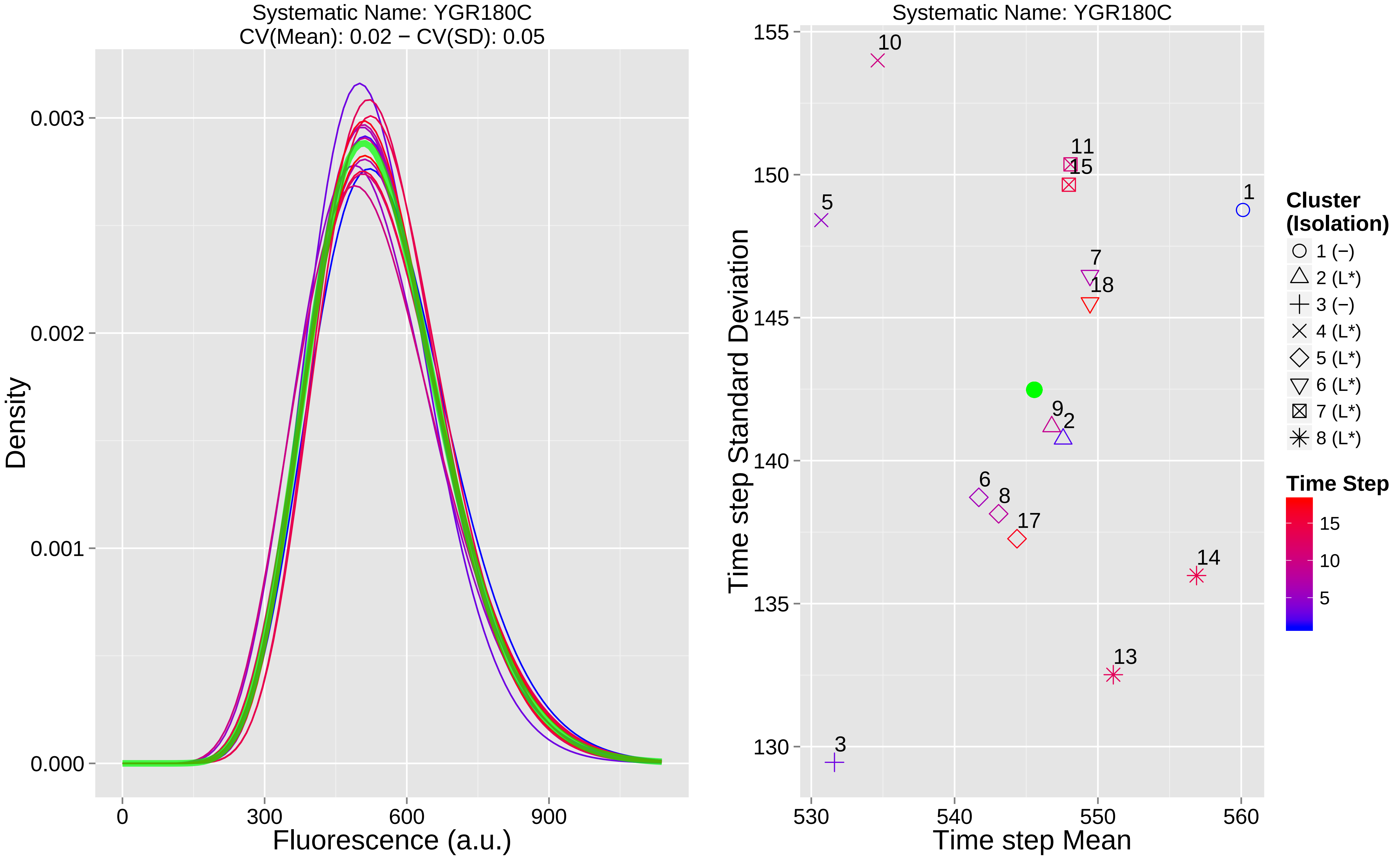

Systematic Name: YGR180C
CV(Mean): 0.02 − CV(SD): 0.05
Density
0.003
0.002
0.001
0.000
0
300
600
900
Fluorescence (a.u.)
Systematic Name: YGR180C
Time step Standard Deviation
155
150
145
140
135
130
530
540
550
560
Time step Mean
10
11
15
5
1
7
18
9
2
6
8
17
14
13
3
Cluster
(Isolation)
1 (−)
2 (L*)
3 (−)
4 (L*)
5 (L*)
6 (L*)
7 (L*)
8 (L*)
Time Step
15
10
5

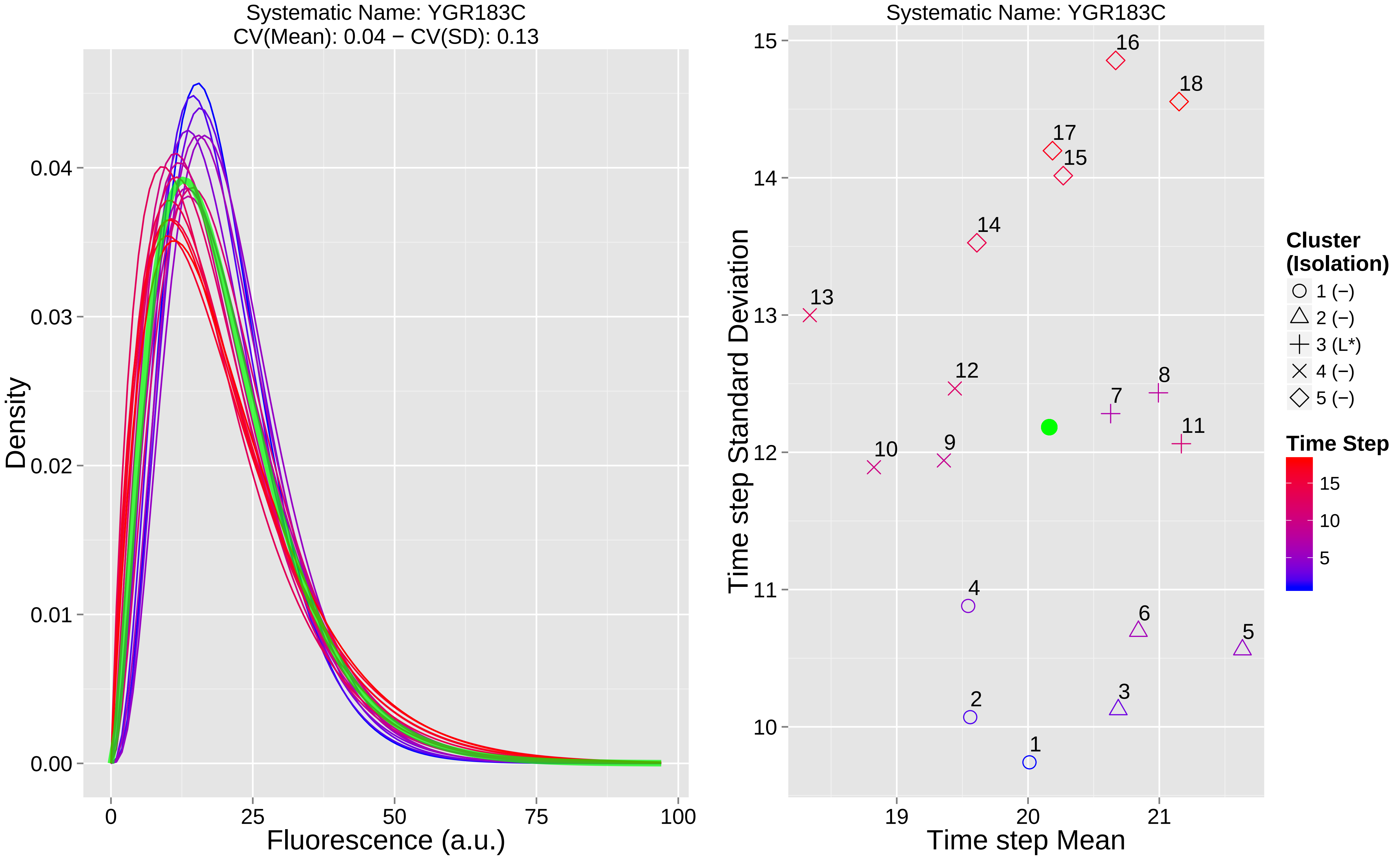

Systematic Name: YGR183C
CV(Mean): 0.04 − CV(SD): 0.13
Density
0.00
0.01
0.02
0.03
0.04
0
25
50
75
100
Fluorescence (a.u.)
Systematic Name: YGR183C
Time step Standard Deviation
10
11
12
13
14
15
19
20
21
Time step Mean
1
2
3
4
5
6
7
8
9
10
11
12
13
14
15
16
17
18
Cluster
(Isolation)
1 (−)
2 (−)
3 (L*)
4 (−)
5 (−)
Time Step
15
10
5

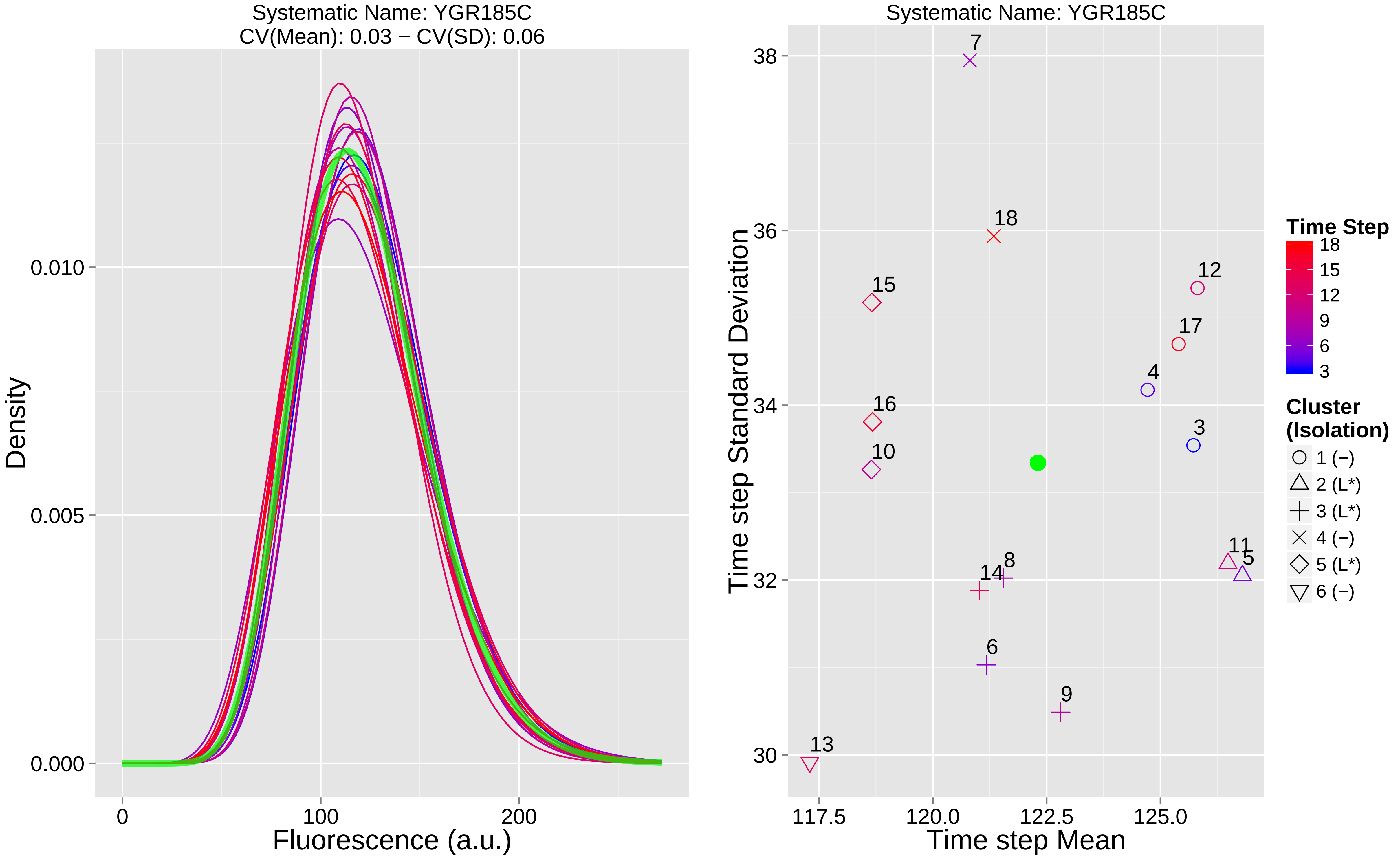

Systematic Name: YGR185C
CV(Mean): 0.03 − CV(SD): 0.06
Density
0.010
0.005
0.000
0
100
200
Fluorescence (a.u.)
Systematic Name: YGR185C
Time step Standard Deviation
38
36
34
32
30
117.5
120.0
122.5
125.0
Time step Mean
7
18
15
12
17
4
16
3
10
11
5
14
8
6
9
13
Time Step
18
15
12
9
6
3
Cluster
(Isolation)
1 (−)
2 (L*)
3 (L*)
4 (−)
5 (L*)
6 (−)

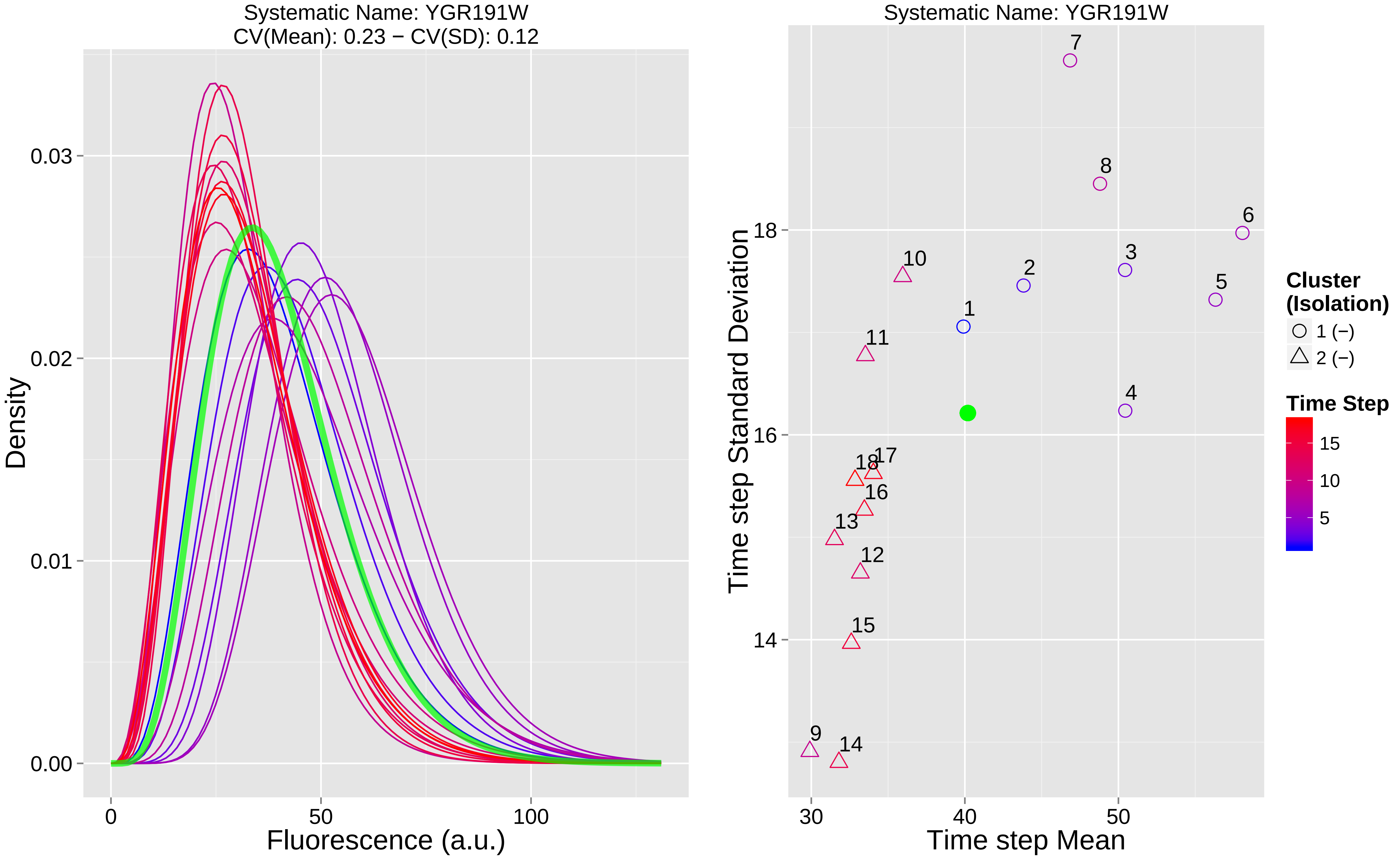
Systematic Name: YGR191W
CV(Mean): 0.23 − CV(SD): 0.12
Density
Fluorescence (a.u.)
0.00
0.01
0.02
0.03
0
50
100
Systematic Name: YGR191W
Time step Standard Deviation
Time step Mean
14
16
18
30
40
50
Cluster (Isolation)
1 (−)
2 (−)
Time Step
5
10
15

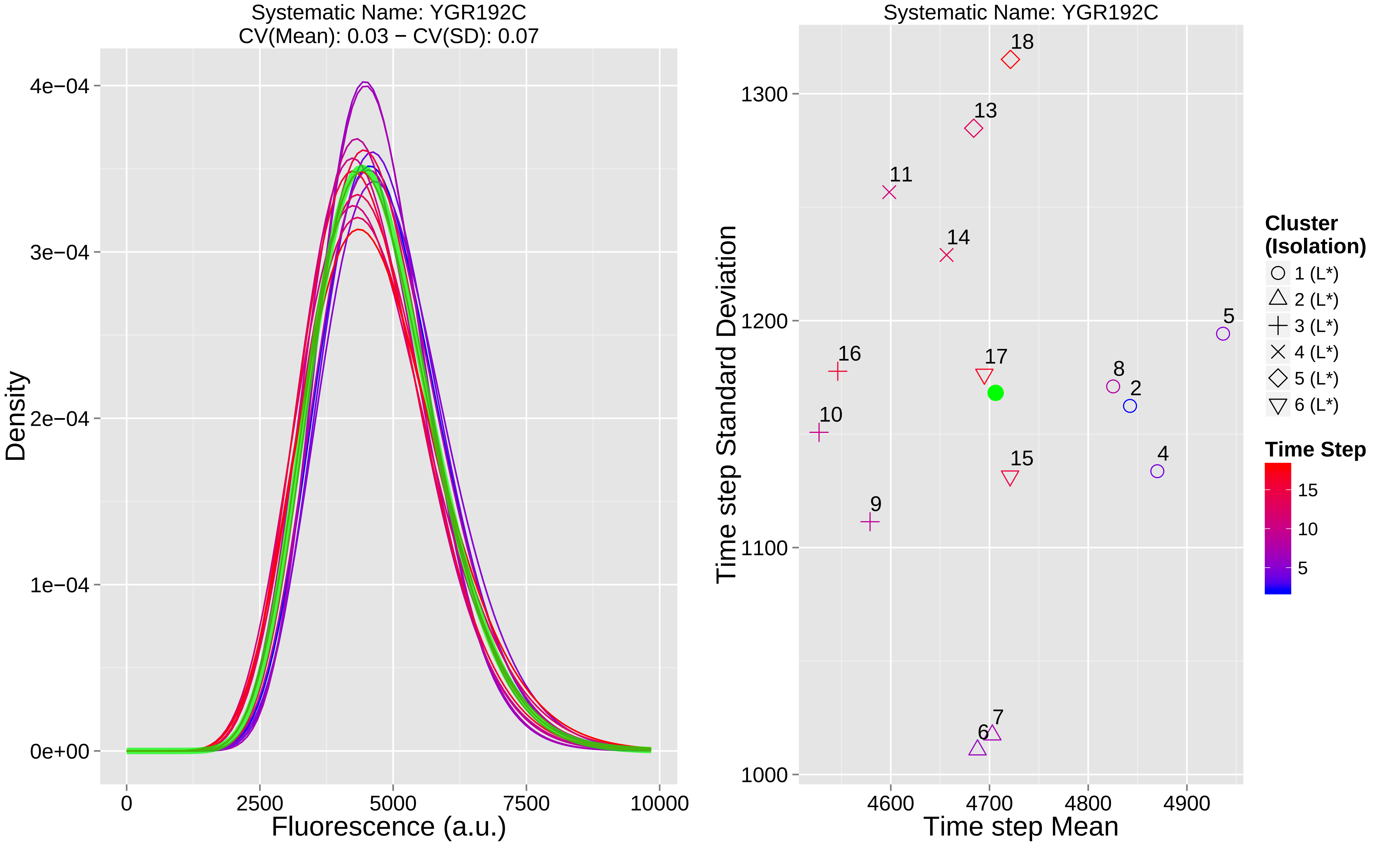
Systematic Name: YGR192C
CV(Mean): 0.03 − CV(SD): 0.07
Density
Fluorescence (a.u.)
4e−04
3e−04
2e−04
1e−04
0e+00
0
2500
5000
7500
10000
Systematic Name: YGR192C
Time step Standard Deviation
Time step Mean
1300
1200
1100
1000
4600
4700
4800
4900
18
13
11
14
5
16
17
8
2
10
4
15
9
7
6
Cluster
(Isolation)
1 (L*)
2 (L*)
3 (L*)
4 (L*)
5 (L*)
6 (L*)
Time Step
15
10
5

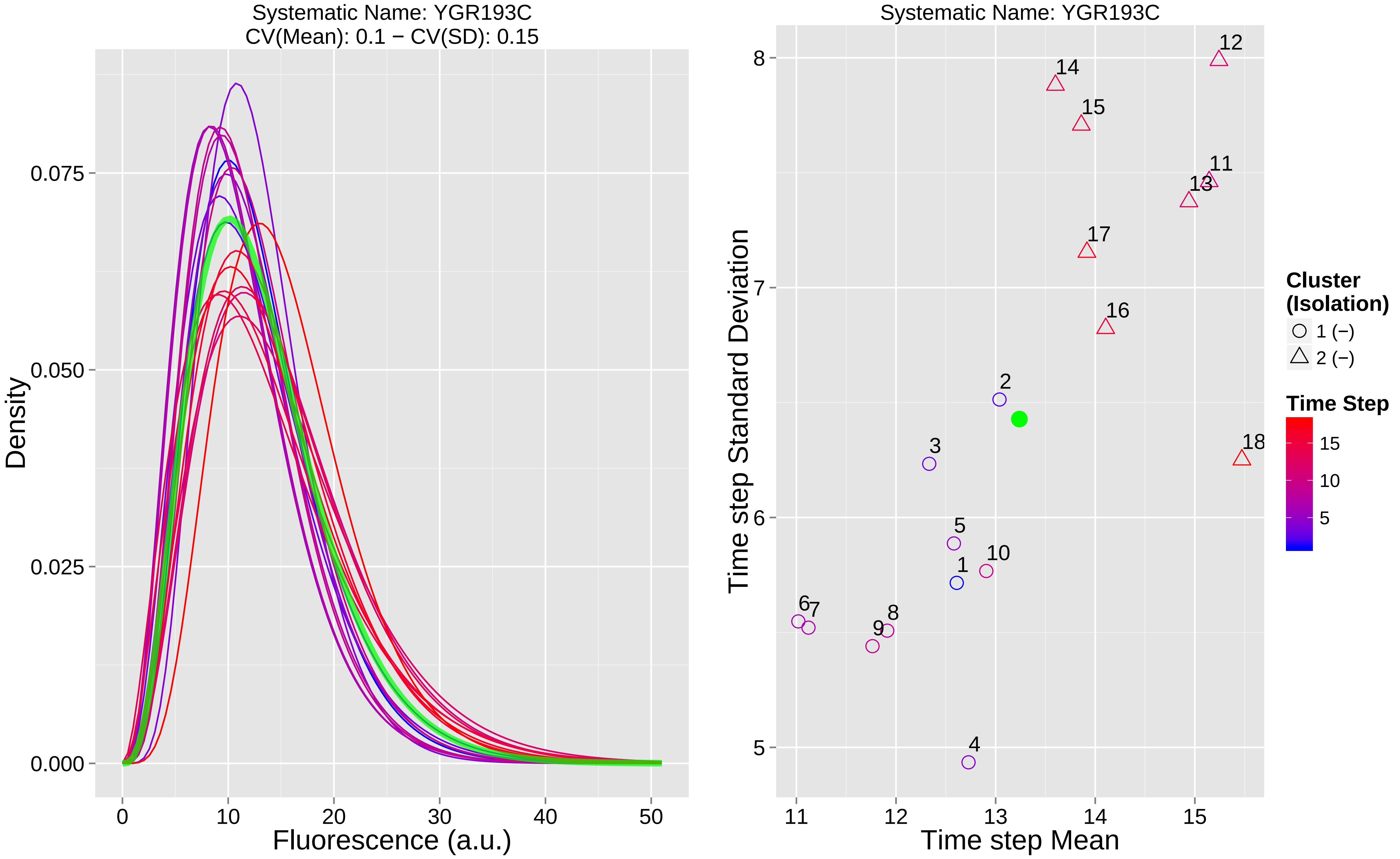

Systematic Name: YGR193C
CV(Mean): 0.1 − CV(SD): 0.15
Density
0.000
0.025
0.050
0.075
0
10
20
30
40
50
Fluorescence (a.u.)
Systematic Name: YGR193C
Time step Standard Deviation
5
6
7
8
11
12
13
14
15
Time step Mean
1
2
3
4
5
6
7
8
9
10
11
12
13
14
15
16
17
18
Cluster
(Isolation)
1 (−)
2 (−)
Time Step
15
10
5

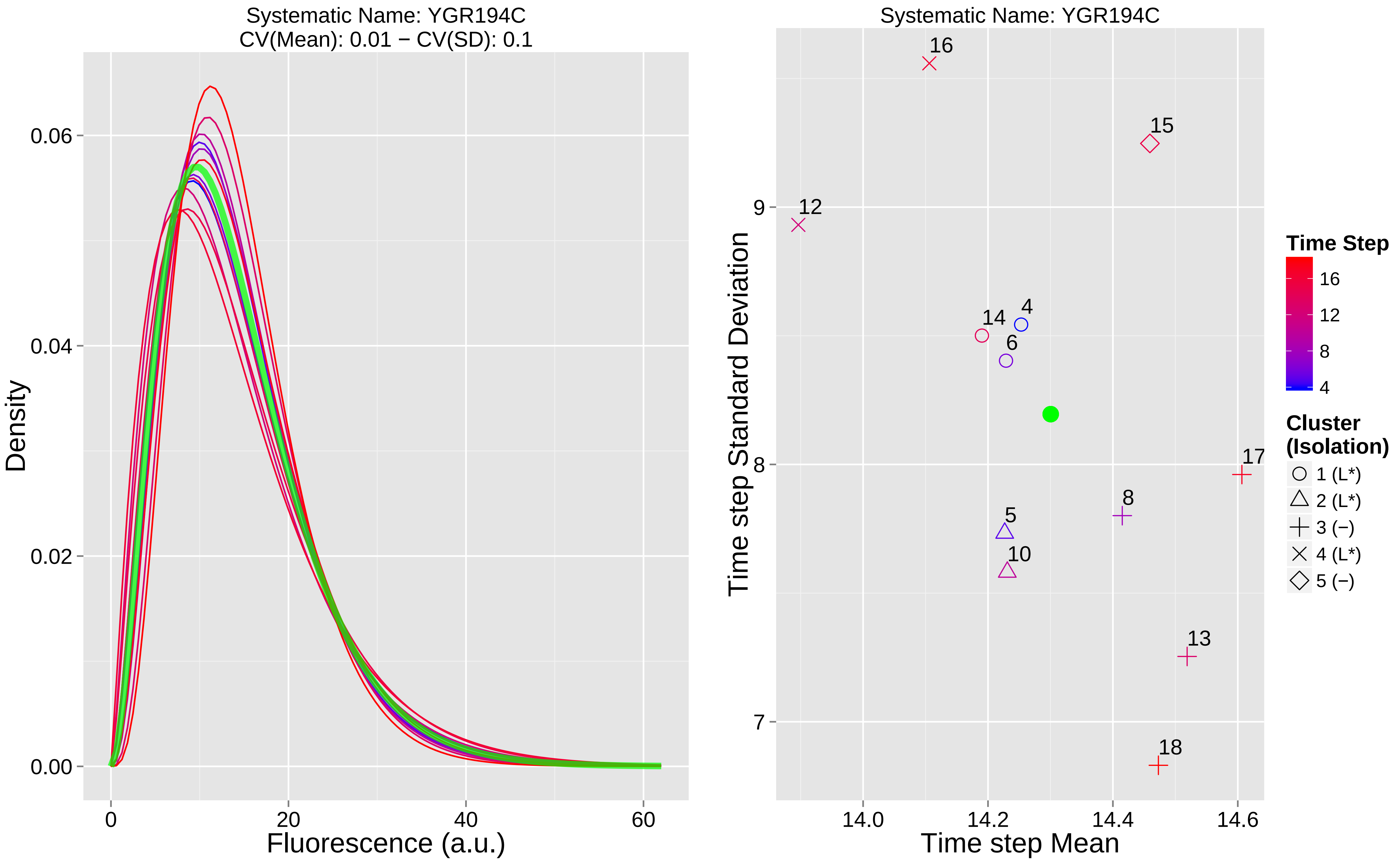
Systematic Name: YGR194C
CV(Mean): 0.01 − CV(SD): 0.1
Density
0.06
0.04
0.02
0.00
0
20
40
60
Fluorescence (a.u.)
Systematic Name: YGR194C
Time step Standard Deviation
9
8
7
14.0
14.2
14.4
14.6
Time step Mean
16
15
12
14
4
6
17
8
5
10
13
18
Time Step
16
12
8
4
Cluster
(Isolation)
1 (L*)
2 (L*)
3 (−)
4 (L*)
5 (−)

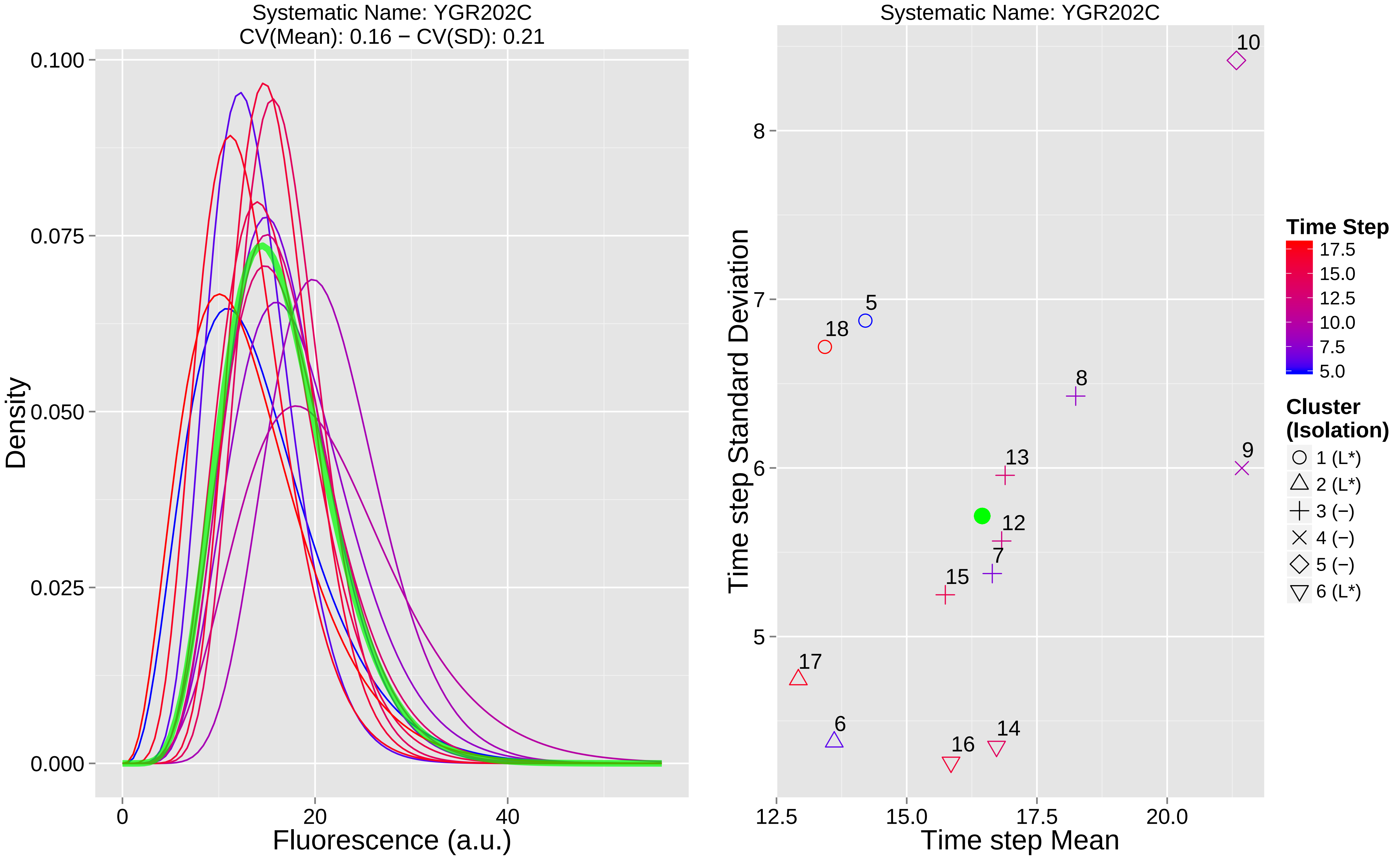
Systematic Name: YGR202C
CV(Mean): 0.16 − CV(SD): 0.21
Density
Fluorescence (a.u.)
0.100
0.075
0.050
0.025
0.000
0
20
40
Systematic Name: YGR202C
Time step Standard Deviation
Time step Mean
12.5
15.0
17.5
20.0
8
7
6
5
10
5
18
8
13
9
12
7
15
17
6
16
14
Time Step
17.5
15.0
12.5
10.0
7.5
5.0
Cluster
(Isolation)
1 (L*)
2 (L*)
3 (−)
4 (−)
5 (−)
6 (L*)

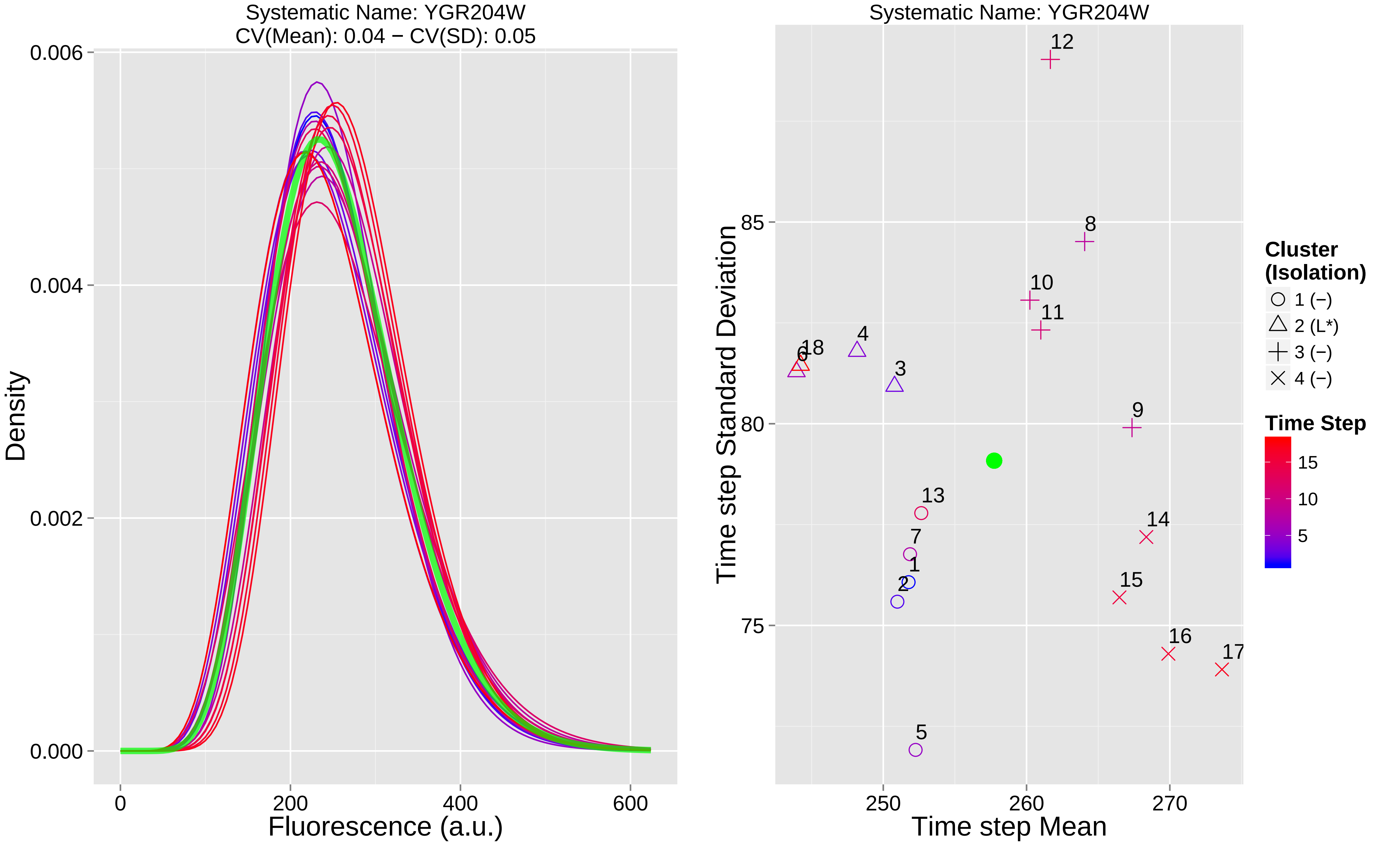

Systematic Name: YGR204W
CV(Mean): 0.04 − CV(SD): 0.05
Density
0.006
0.004
0.002
0.000
0
200
400
600
Fluorescence (a.u.)
Systematic Name: YGR204W
Time step Standard Deviation
85
80
75
250
260
270
Time step Mean
12
8
10
11
4
6
18
3
9
13
14
7
1
2
15
16
17
5
Cluster (Isolation)
1 (−)
2 (L*)
3 (−)
4 (−)
Time Step
15
10
5

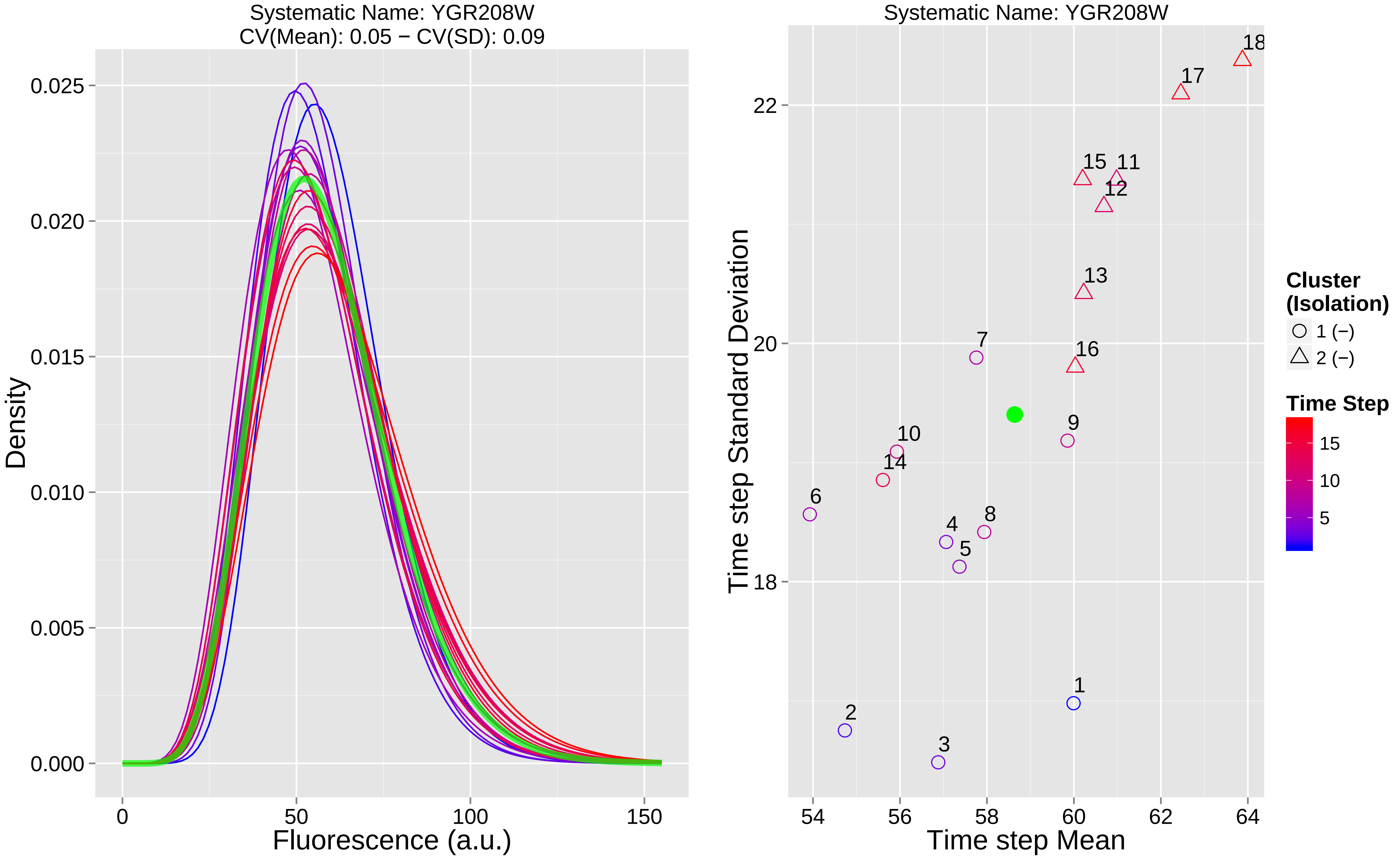

Systematic Name: YGR208W
CV(Mean): 0.05 − CV(SD): 0.09
Density
0.025
0.020
0.015
0.010
0.005
0.000
0
50
100
150
Fluorescence (a.u.)
Systematic Name: YGR208W
Time step Standard Deviation
22
20
18
54
56
58
60
62
64
Time step Mean
1
2
3
4
5
6
7
8
9
10
11
12
13
14
15
16
17
18
Cluster (Isolation)
1 (−)
2 (−)
Time Step
15
10
5

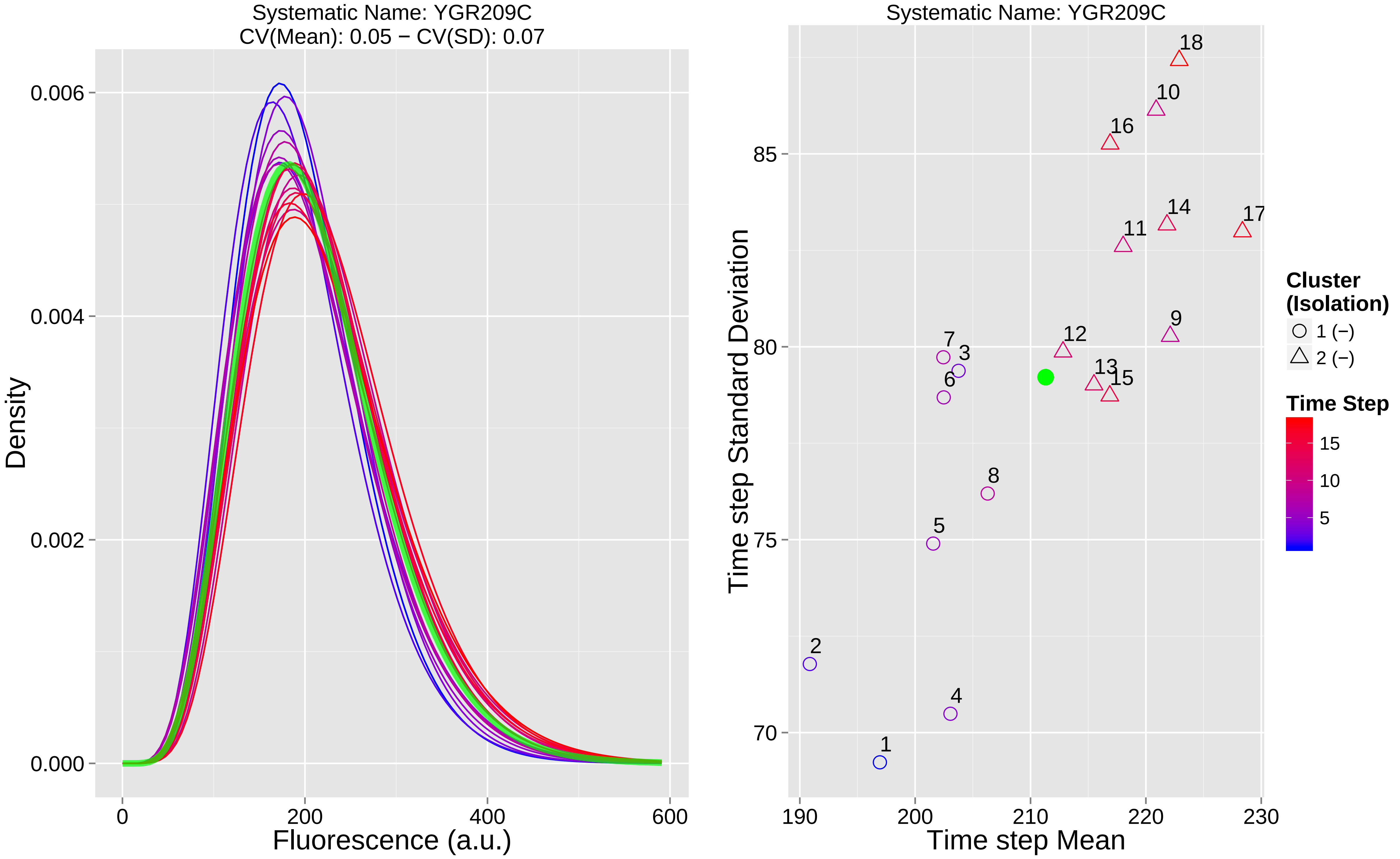
Systematic Name: YGR209C
CV(Mean): 0.05 − CV(SD): 0.07
Density
Fluorescence (a.u.)
0.000
0.002
0.004
0.006
0
200
400
600
Systematic Name: YGR209C
Time step Standard Deviation
Time step Mean
70
75
80
85
190
200
210
220
230
Cluster
(Isolation)
1 (−)
2 (−)
Time Step
5
10
15

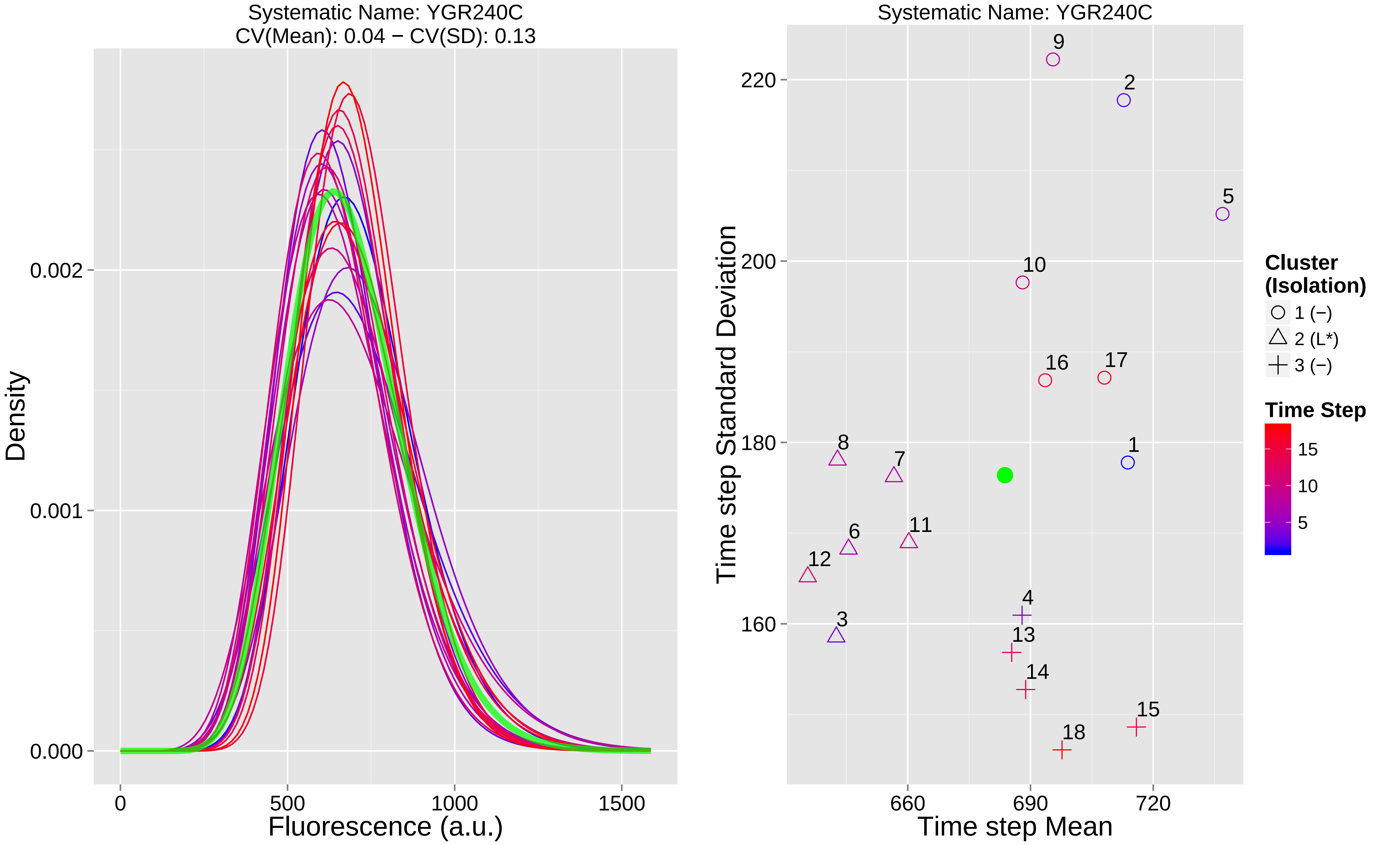

Systematic Name: YGR240C
CV(Mean): 0.04 − CV(SD): 0.13
Density
0.000
0.001
0.002
0
500
1000
1500
Fluorescence (a.u.)
Systematic Name: YGR240C
Time step Standard Deviation
160
180
200
220
660
690
720
Time step Mean
1
2
3
4
5
6
7
8
9
10
11
12
13
14
15
16
17
18
Cluster (Isolation)
1 (−)
2 (L*)
3 (−)
Time Step
5
10
15

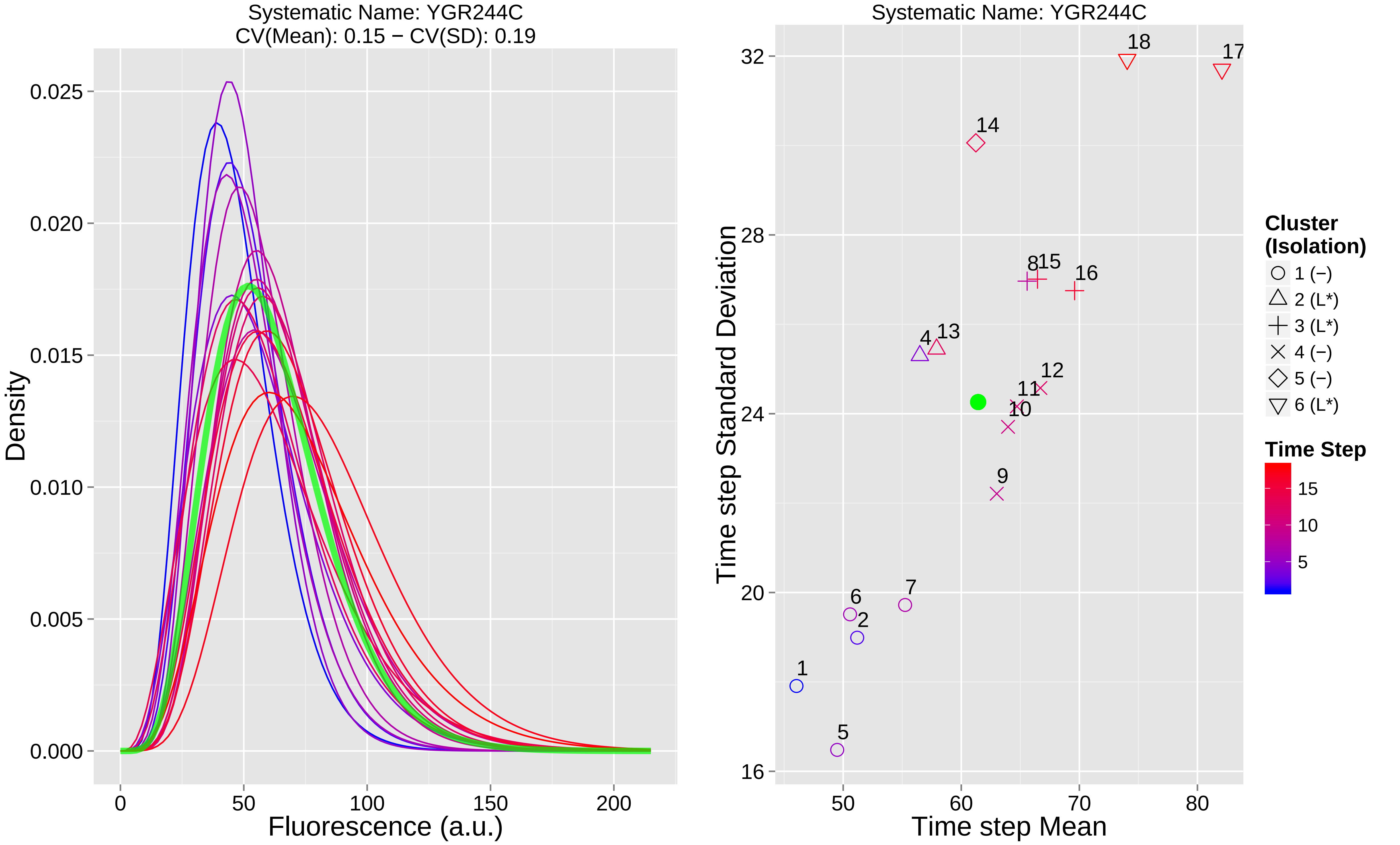
Systematic Name: YGR244C
CV(Mean): 0.15 − CV(SD): 0.19
Density
Fluorescence (a.u.)
0.000
0.005
0.010
0.015
0.020
0.025
0
50
100
150
200
Systematic Name: YGR244C
Time step Standard Deviation
Time step Mean
16
20
24
28
32
50
60
70
80
1
2
4
5
6
7
8
9
10
11
12
13
14
15
16
17
18
Cluster (Isolation)
1 (−)
2 (L*)
3 (L*)
4 (−)
5 (−)
6 (L*)
Time Step
5
10
15

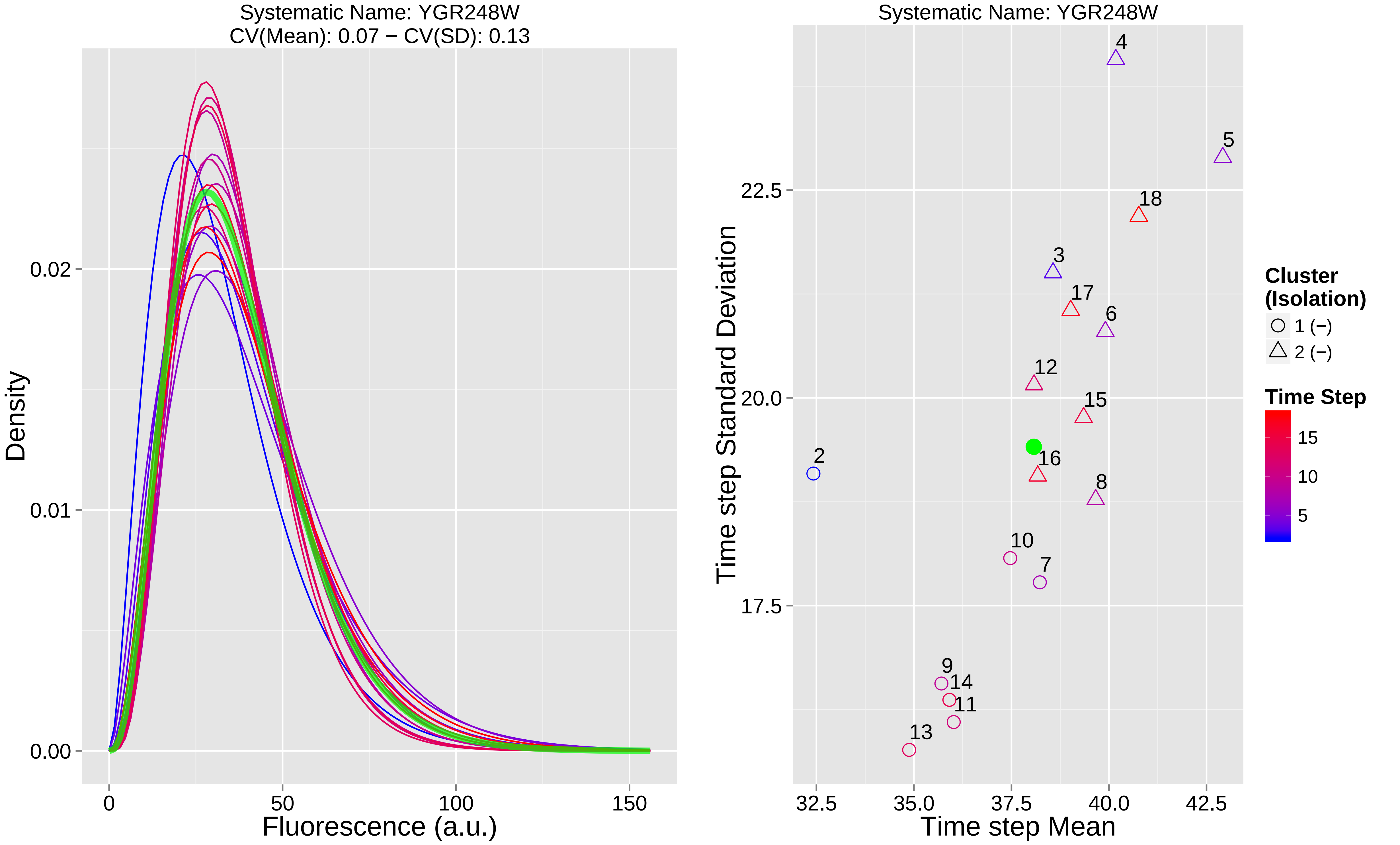

Systematic Name: YGR248W
CV(Mean): 0.07 − CV(SD): 0.13
Density
Fluorescence (a.u.)
0.00
0.01
0.02
0
50
100
150
Systematic Name: YGR248W
Time step Standard Deviation
Time step Mean
17.5
20.0
22.5
32.5
35.0
37.5
40.0
42.5
2
3
4
5
6
7
8
9
10
11
12
13
14
15
16
17
18
Cluster (Isolation)
1 (−)
2 (−)
Time Step
5
10
15

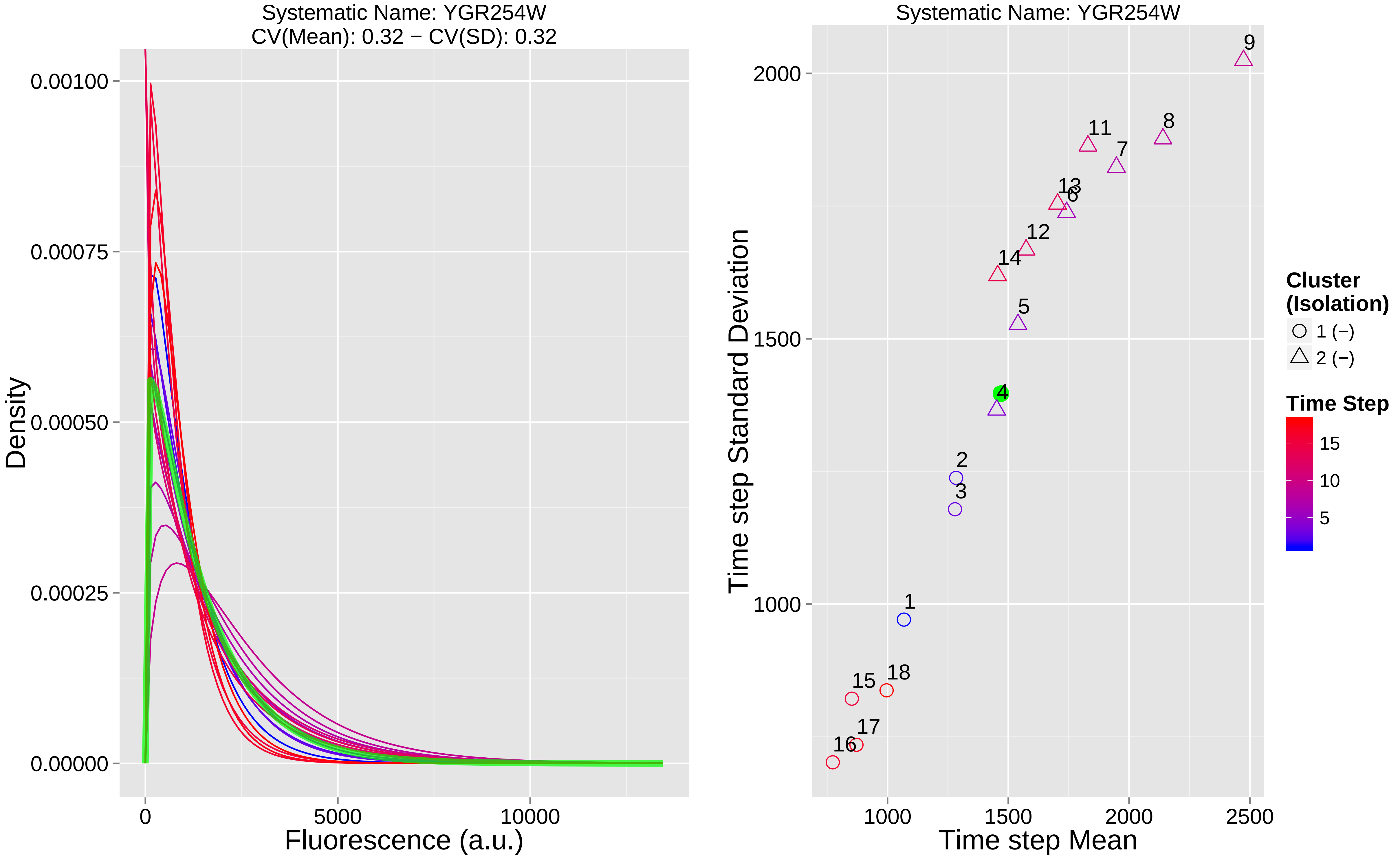

Systematic Name: YGR254W
CV(Mean): 0.32 − CV(SD): 0.32
Density
0.00100
0.00075
0.00050
0.00025
0.00000
0
5000
10000
Fluorescence (a.u.)
Systematic Name: YGR254W
Time step Standard Deviation
2000
1500
1000
1000
1500
2000
2500
Time step Mean
1 2 3 4 5 6 7 8 9 11 12 13 14 15 16 17 18
Cluster (Isolation)
1 (−)
2 (−)
Time Step
15
10
5

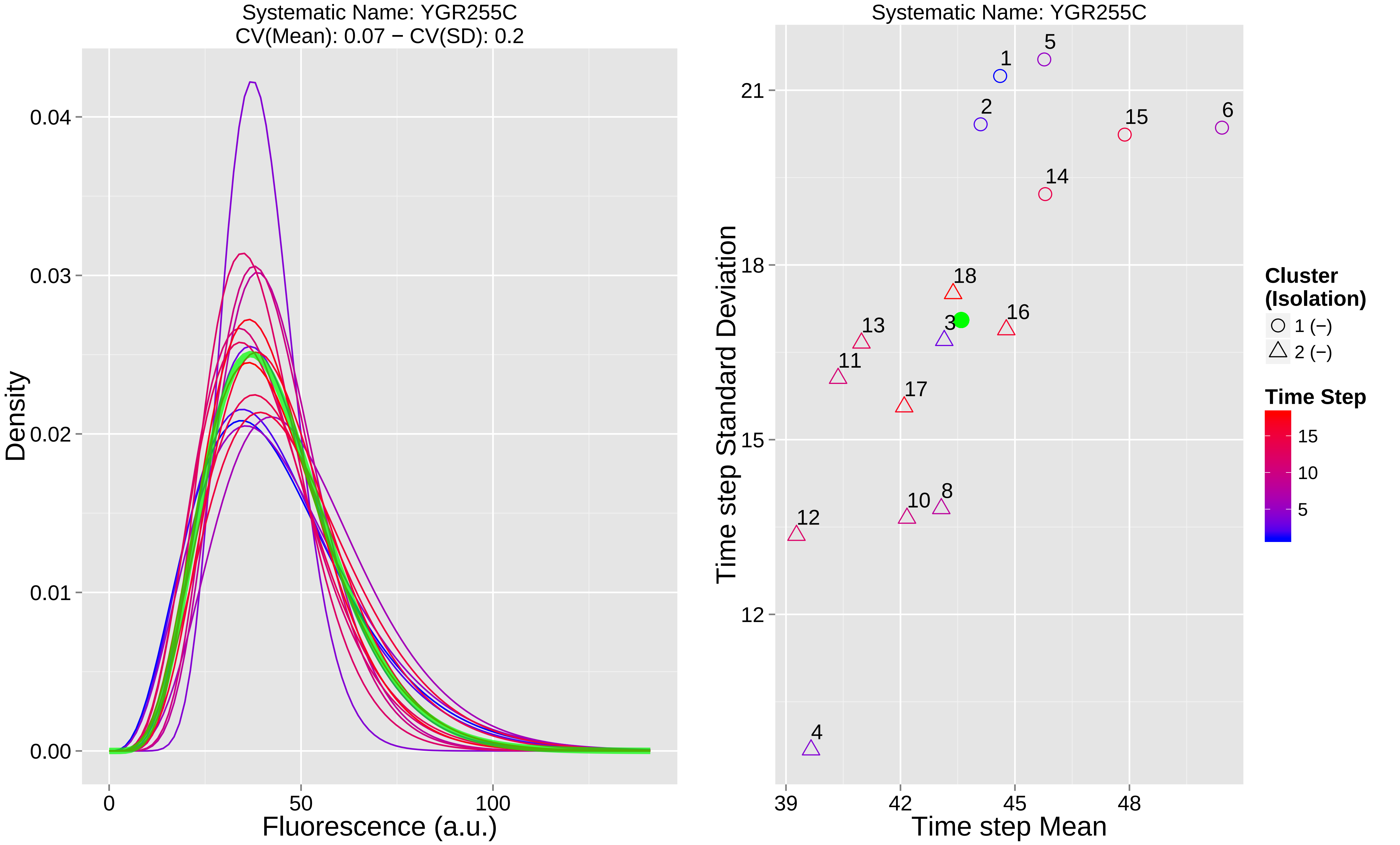
Systematic Name: YGR255C
CV(Mean): 0.07 − CV(SD): 0.2
Density
0.00
0.01
0.02
0.03
0.04
0
50
100
Fluorescence (a.u.)
Systematic Name: YGR255C
Time step Standard Deviation
12
15
18
21
39
42
45
48
Time step Mean
1
2
3
4
5
6
8
10
11
12
13
14
15
16
17
18
Cluster (Isolation)
1 (−)
2 (−)
Time Step
5
10
15

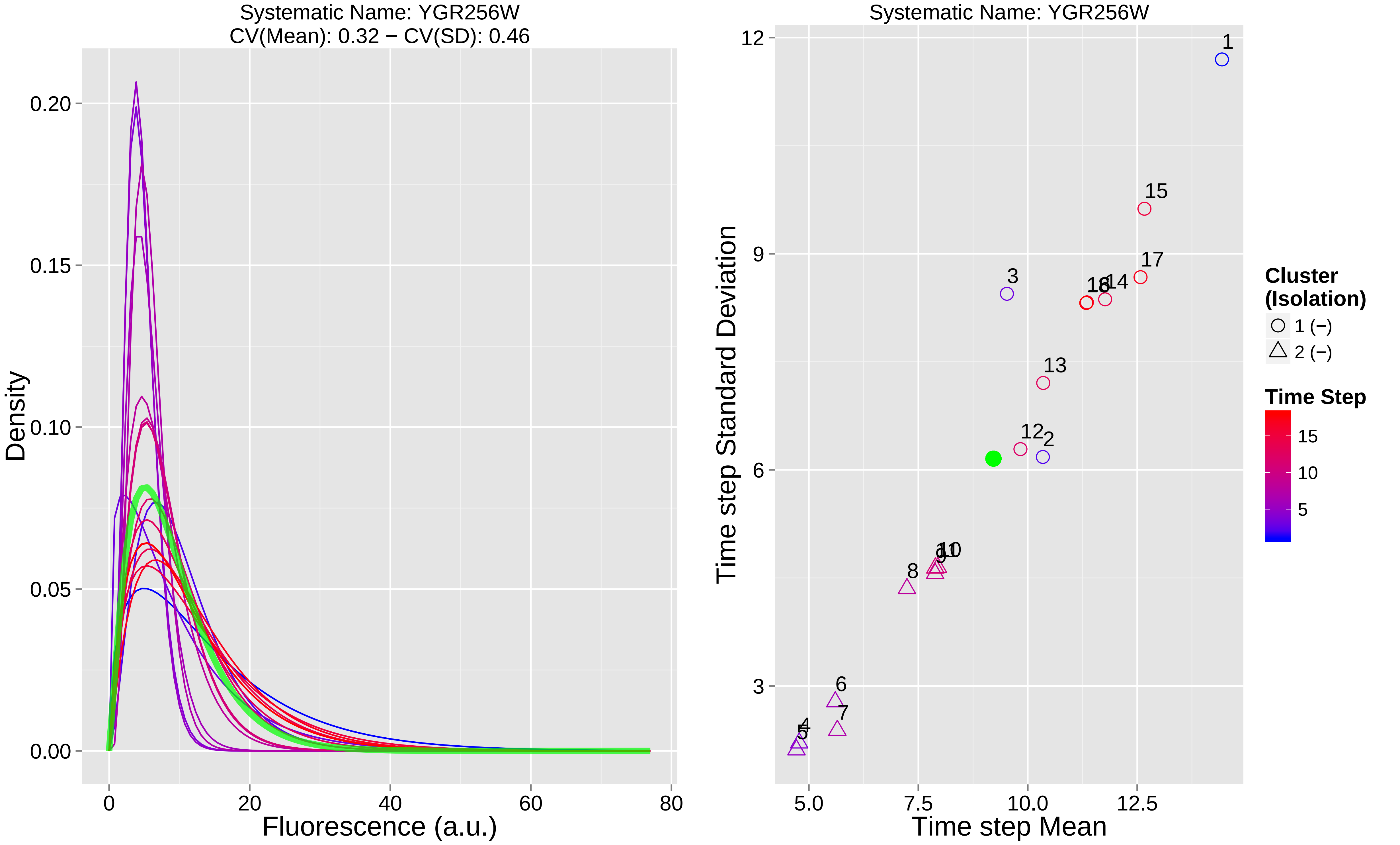
Systematic Name: YGR256W
CV(Mean): 0.32 − CV(SD): 0.46
Density
Fluorescence (a.u.)
Systematic Name: YGR256W
Time step Standard Deviation
Time step Mean
Cluster (Isolation)
1 (−)
2 (−)
Time Step

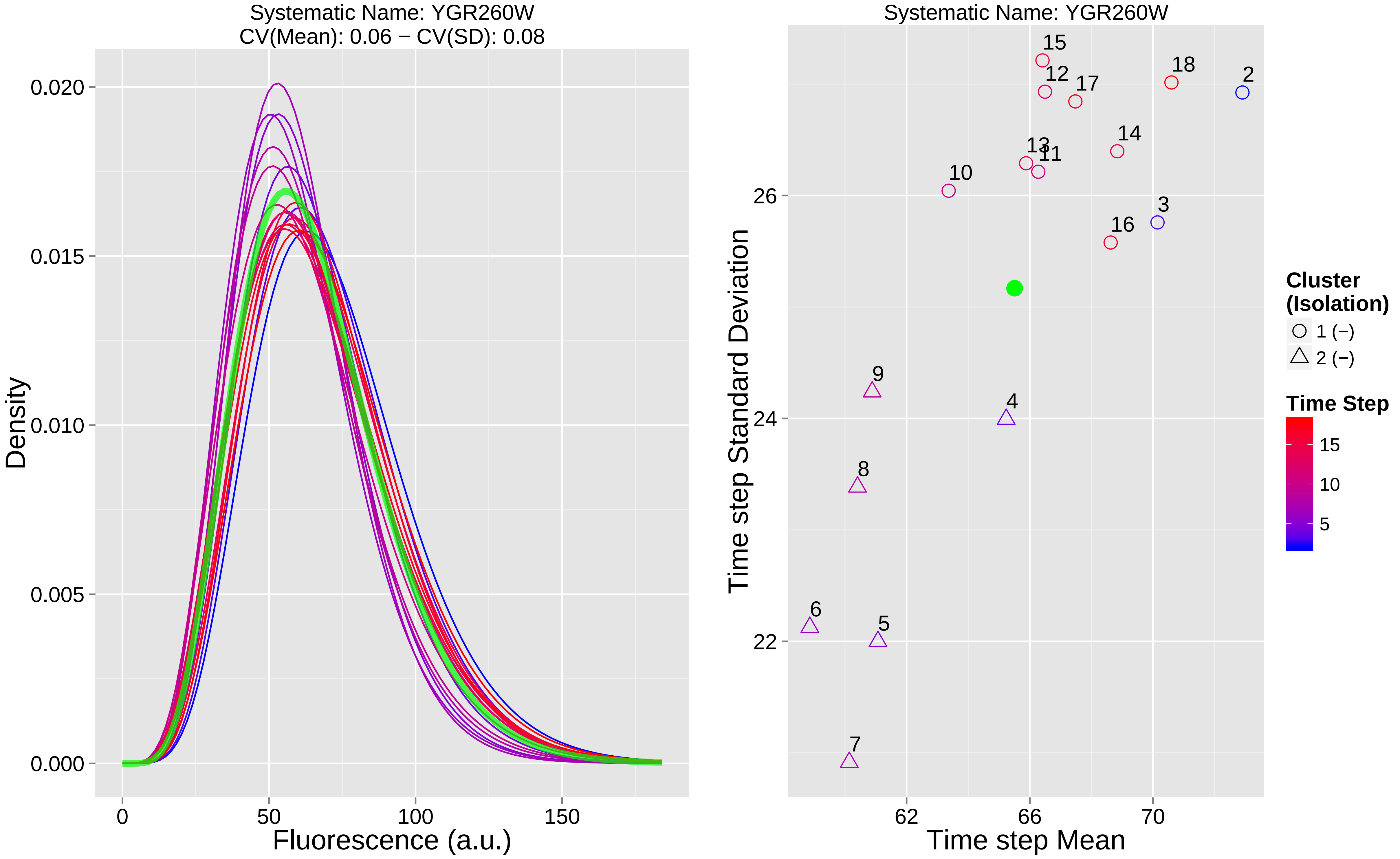

Systematic Name: YGR260W
CV(Mean): 0.06 − CV(SD): 0.08
Density
0.020
0.015
0.010
0.005
0.000
0
50
100
150
Fluorescence (a.u.)
Systematic Name: YGR260W
Time step Standard Deviation
26
24
22
62
66
70
Time step Mean
15
12
17
18
2
14
13
11
10
3
16
9
4
8
6
5
7
Cluster
(Isolation)
1 (−)
2 (−)
Time Step
15
10
5

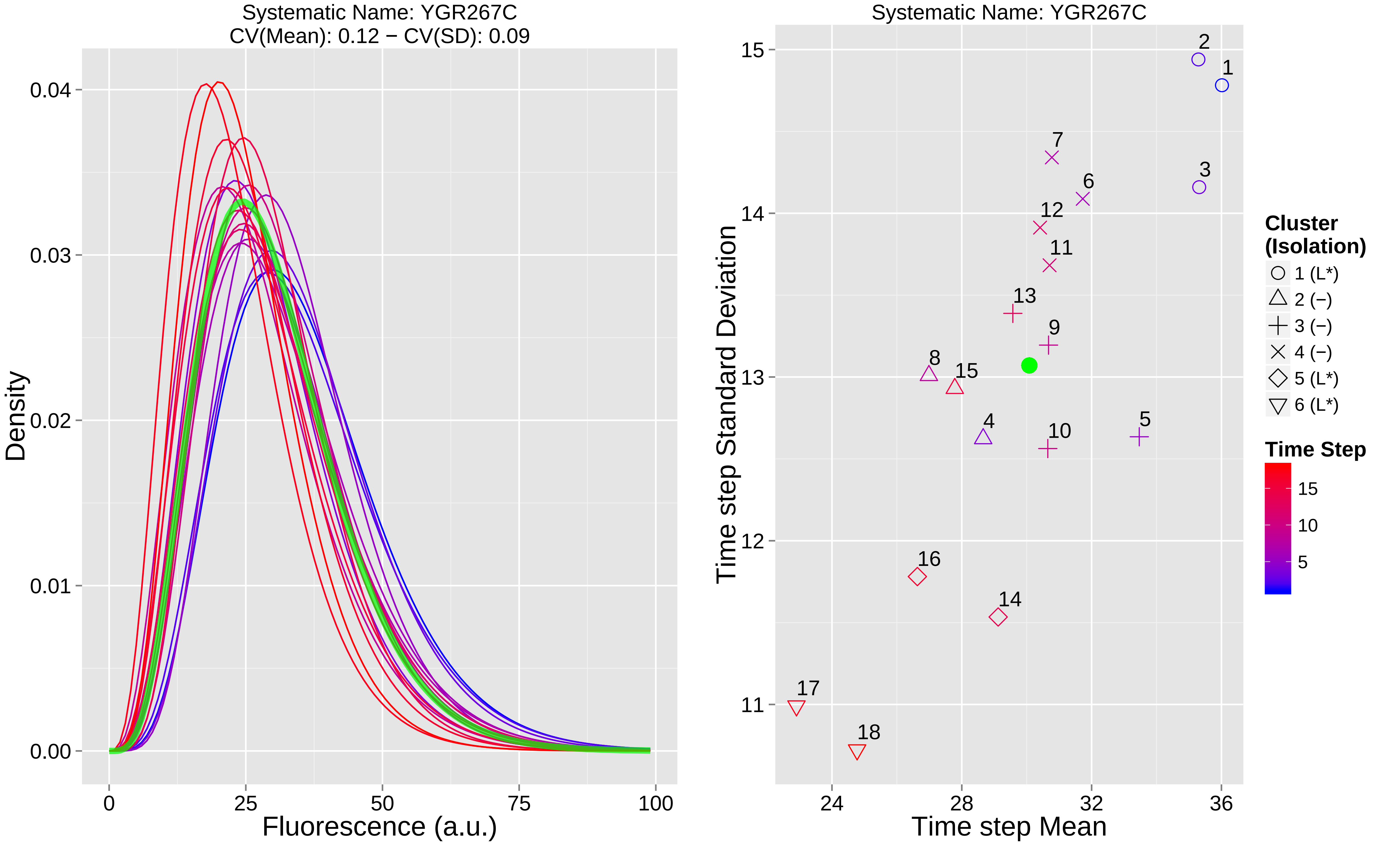
Systematic Name: YGR267C
CV(Mean): 0.12 − CV(SD): 0.09
Density
Fluorescence (a.u.)
0.00
0.01
0.02
0.03
0.04
0
25
50
75
100
Systematic Name: YGR267C
Time step Standard Deviation
Time step Mean
11
12
13
14
15
24
28
32
36
1
2
3
4
5
6
7
8
9
10
11
12
13
14
15
16
17
18
Cluster
(Isolation)
1 (L*)
2 (−)
3 (−)
4 (−)
5 (L*)
6 (L*)
Time Step
15
10
5

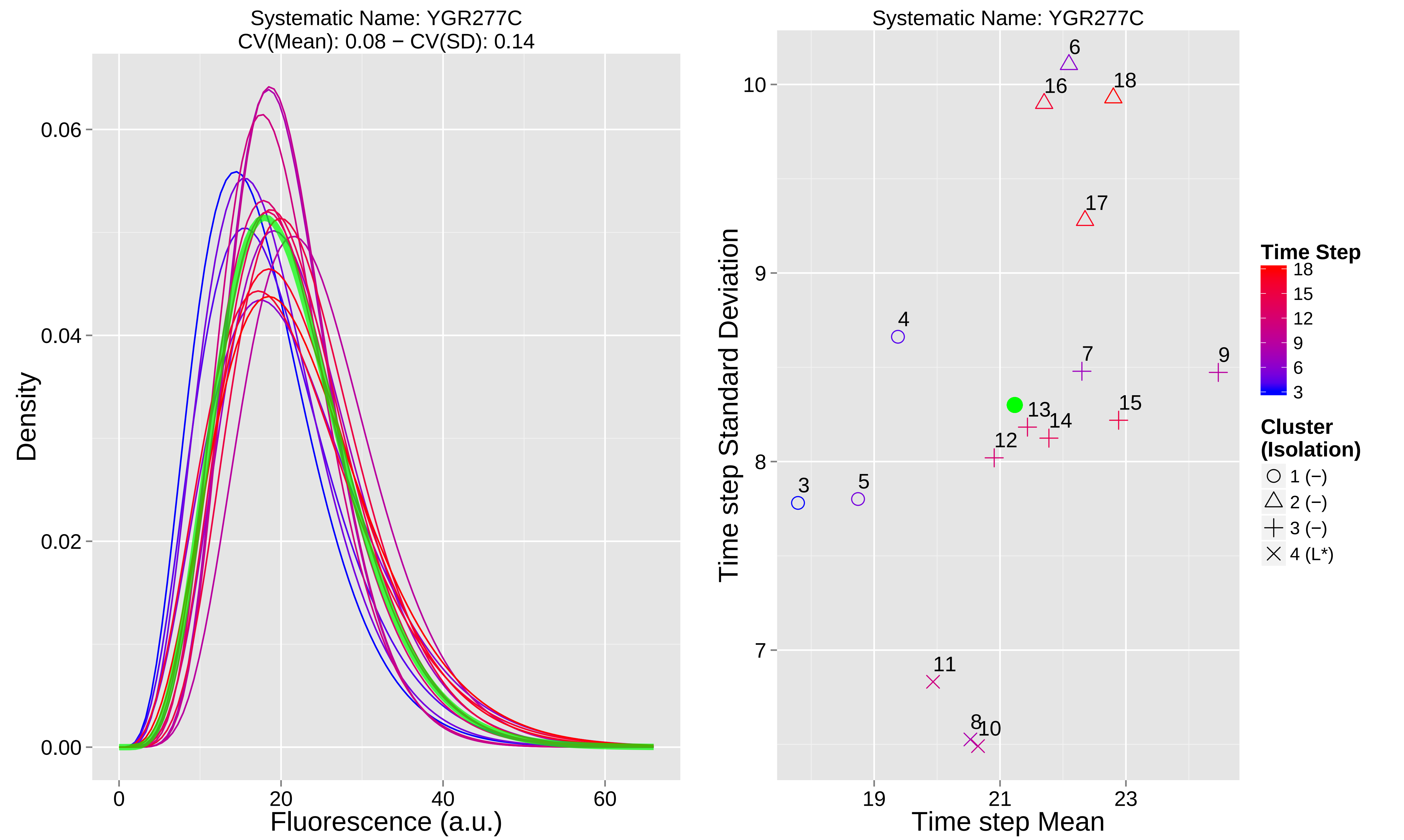

Systematic Name: YGR277C
CV(Mean): 0.08 − CV(SD): 0.14
Density
0.06
0.04
0.02
0.00
0
20
40
60
Fluorescence (a.u.)
Systematic Name: YGR277C
Time step Standard Deviation
10
9
8
7
19
21
23
Time step Mean
3
4
5
6
7
8
9
10
11
12
13
14
15
16
17
18
Time Step
18
15
12
9
6
3
Cluster (Isolation)
1 (−)
2 (−)
3 (−)
4 (L*)

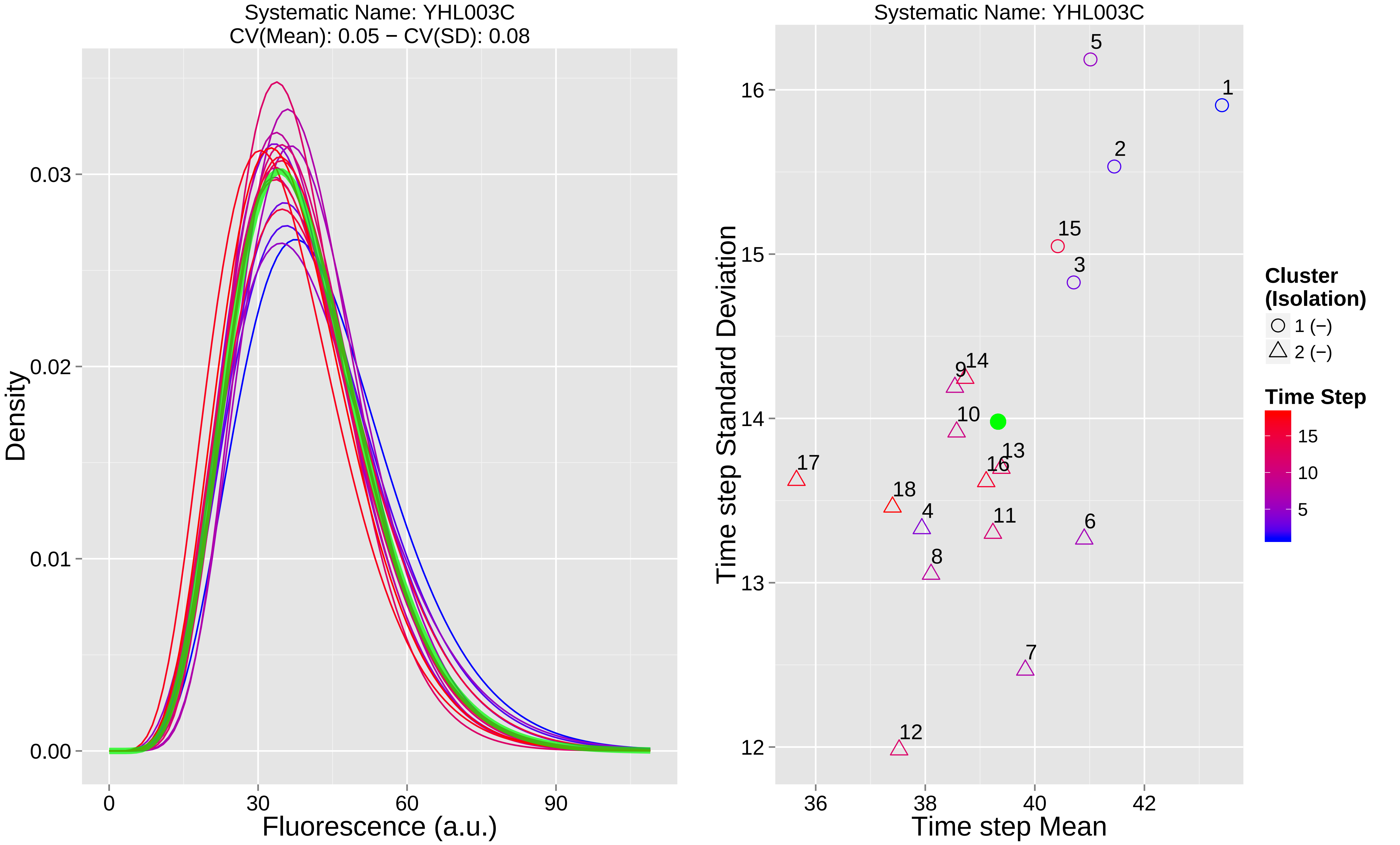

Systematic Name: YHL003C
CV(Mean): 0.05 − CV(SD): 0.08
Density
0.00
0.01
0.02
0.03
0
30
60
90
Fluorescence (a.u.)
Systematic Name: YHL003C
Time step Standard Deviation
12
13
14
15
16
36
38
40
42
Time step Mean
1
2
3
4
5
6
7
8
9
10
11
12
13
14
15
16
17
18
Cluster (Isolation)
1 (−)
2 (−)
Time Step
5
10
15

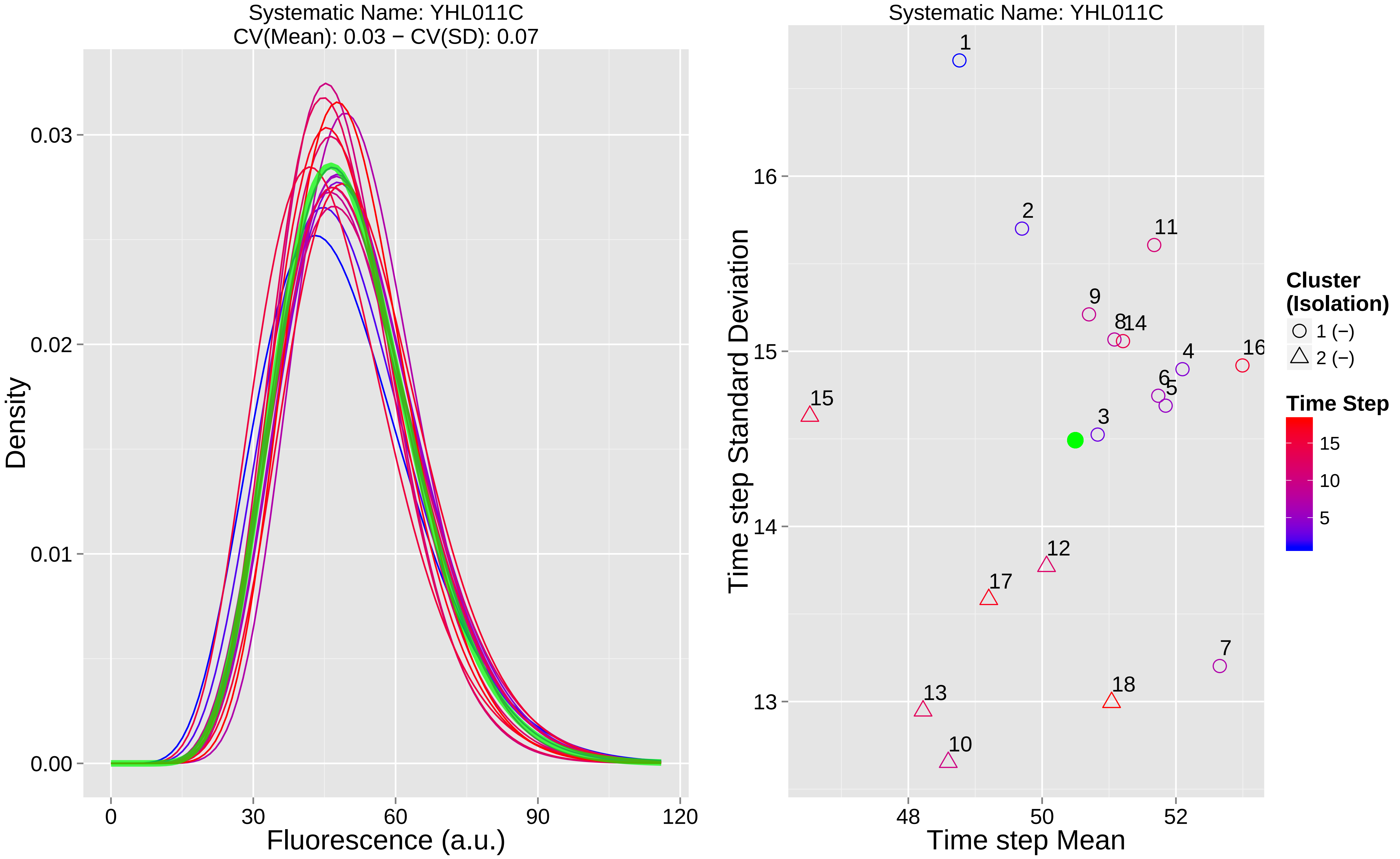

Systematic Name: YHL011C
CV(Mean): 0.03 − CV(SD): 0.07
Density
0.03
0.02
0.01
0.00
0
30
60
90
120
Fluorescence (a.u.)
Systematic Name: YHL011C
Time step Standard Deviation
16
15
14
13
48
50
52
Time step Mean
1
2
3
4
5
6
7
8
9
10
11
12
13
14
15
16
17
18
Cluster (Isolation)
1 (−)
2 (−)
Time Step
15
10
5

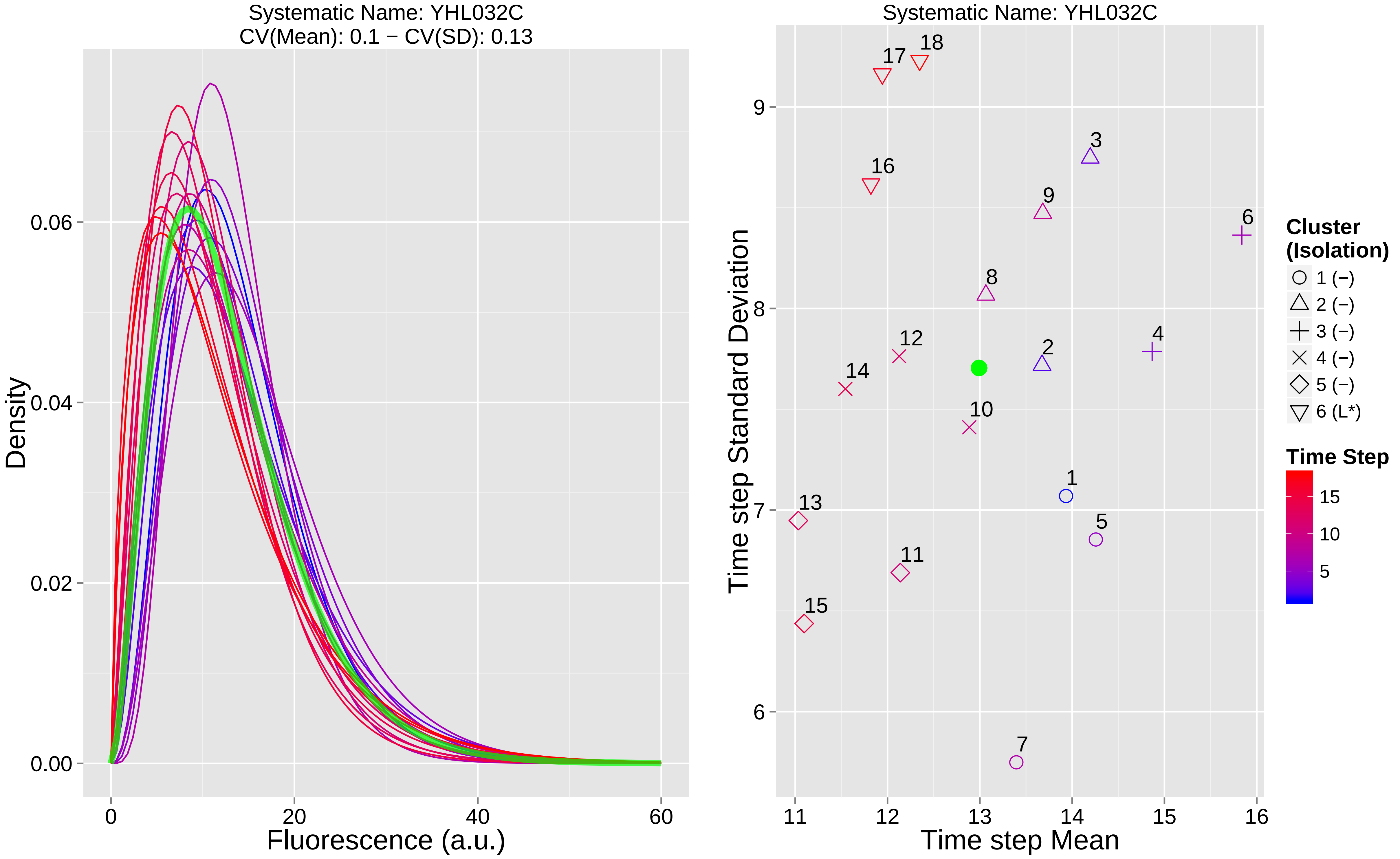

Systematic Name: YHL032C
CV(Mean): 0.1 − CV(SD): 0.13
Density
0.00
0.02
0.04
0.06
0
20
40
60
Fluorescence (a.u.)
Systematic Name: YHL032C
Time step Standard Deviation
6
7
8
9
11
12
13
14
15
16
Time step Mean
1
2
3
4
5
6
7
8
9
10
11
12
13
14
15
16
17
18
Cluster (Isolation)
1 (−)
2 (−)
3 (−)
4 (−)
5 (−)
6 (L*)
Time Step
5
10
15

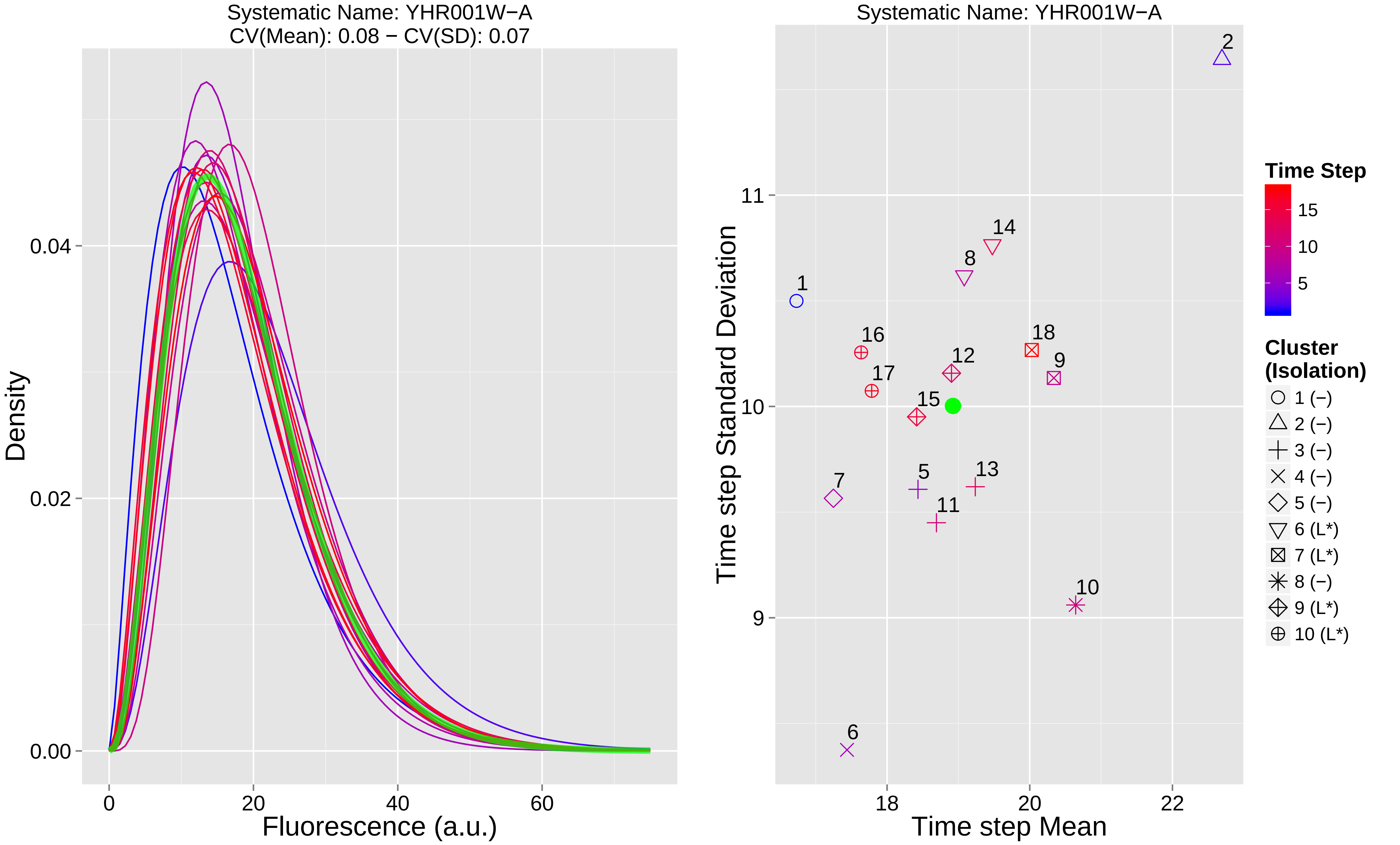
Systematic Name: YHR001W−A
CV(Mean): 0.08 − CV(SD): 0.07
Density
0.04
0.02
0.00
0
20
40
60
Fluorescence (a.u.)
Systematic Name: YHR001W−A
Time step Standard Deviation
11
10
9
18
20
22
Time step Mean
1
2
5
6
7
8
9
10
11
12
13
14
15
16
17
18
Time Step
15
10
5
Cluster
(Isolation)
1 (−)
2 (−)
3 (−)
4 (−)
5 (−)
6 (L*)
7 (L*)
8 (−)
9 (L*)
10 (L*)

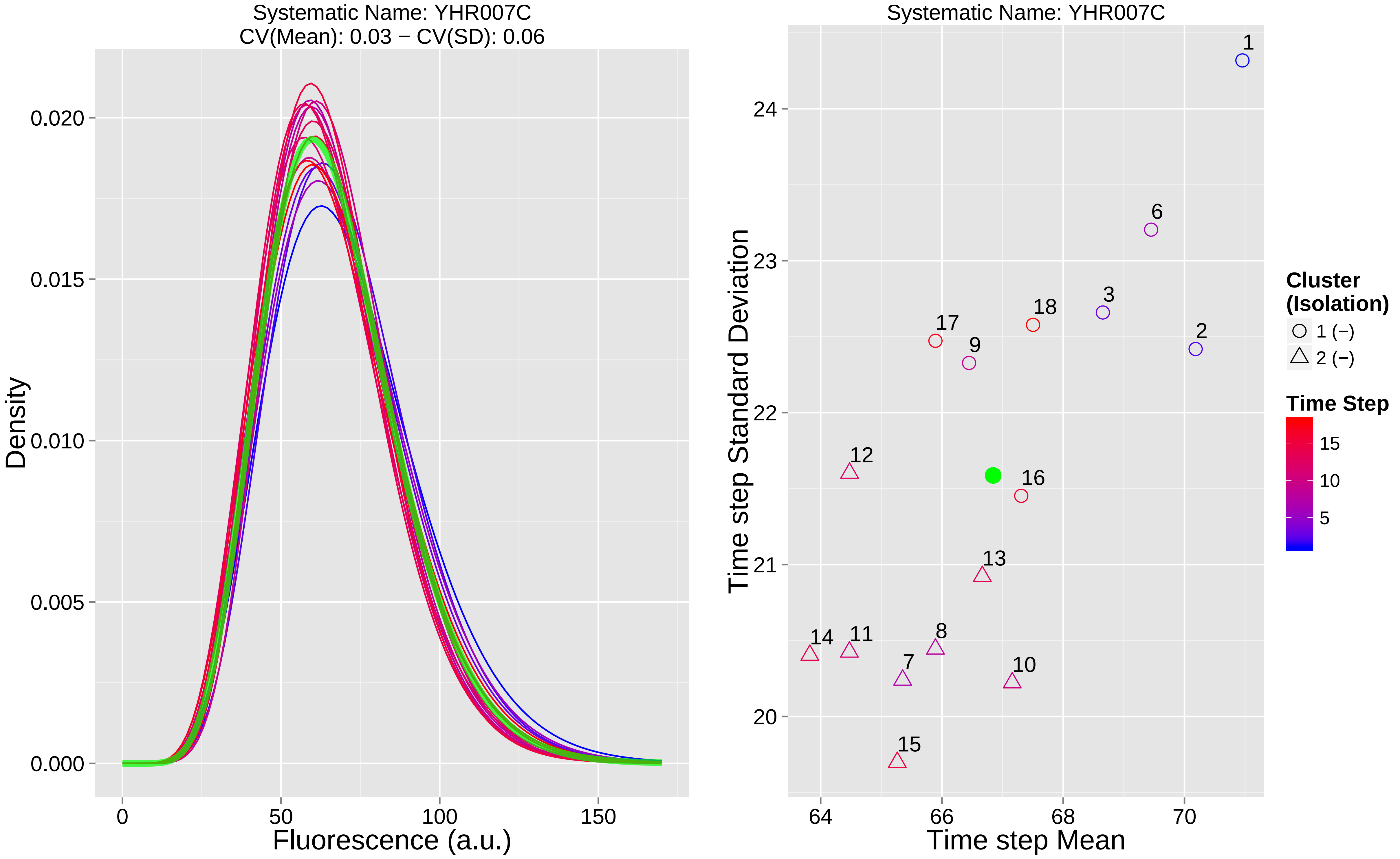
Systematic Name: YHR007C
CV(Mean): 0.03 − CV(SD): 0.06
Density
0.020
0.015
0.010
0.005
0.000
0
50
100
150
Fluorescence (a.u.)
Systematic Name: YHR007C
Time step Standard Deviation
24
23
22
21
20
64
66
68
70
Time step Mean
1
2
3
6
9
17
18
12
16
13
14
11
8
7
10
15
Cluster
(Isolation)
1 (−)
2 (−)
Time Step
15
10
5

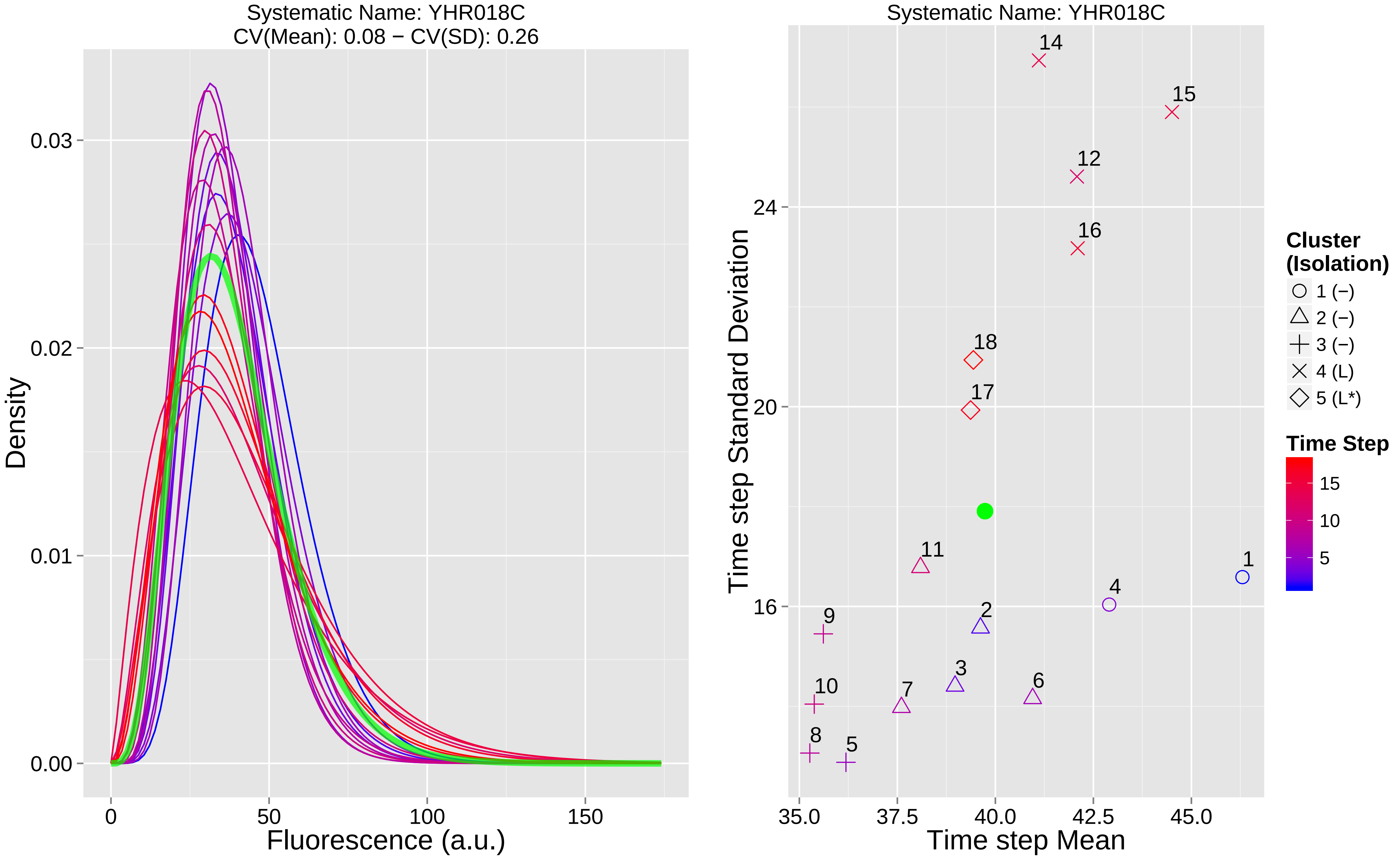

Systematic Name: YHR018C
CV(Mean): 0.08 − CV(SD): 0.26
Density
Fluorescence (a.u.)
0.00
0.01
0.02
0.03
0
50
100
150
Systematic Name: YHR018C
Time step Standard Deviation
Time step Mean
16
20
24
35.0
37.5
40.0
42.5
45.0
Cluster (Isolation)
1 (−)
2 (−)
3 (−)
4 (L)
5 (L*)
Time Step
5
10
15

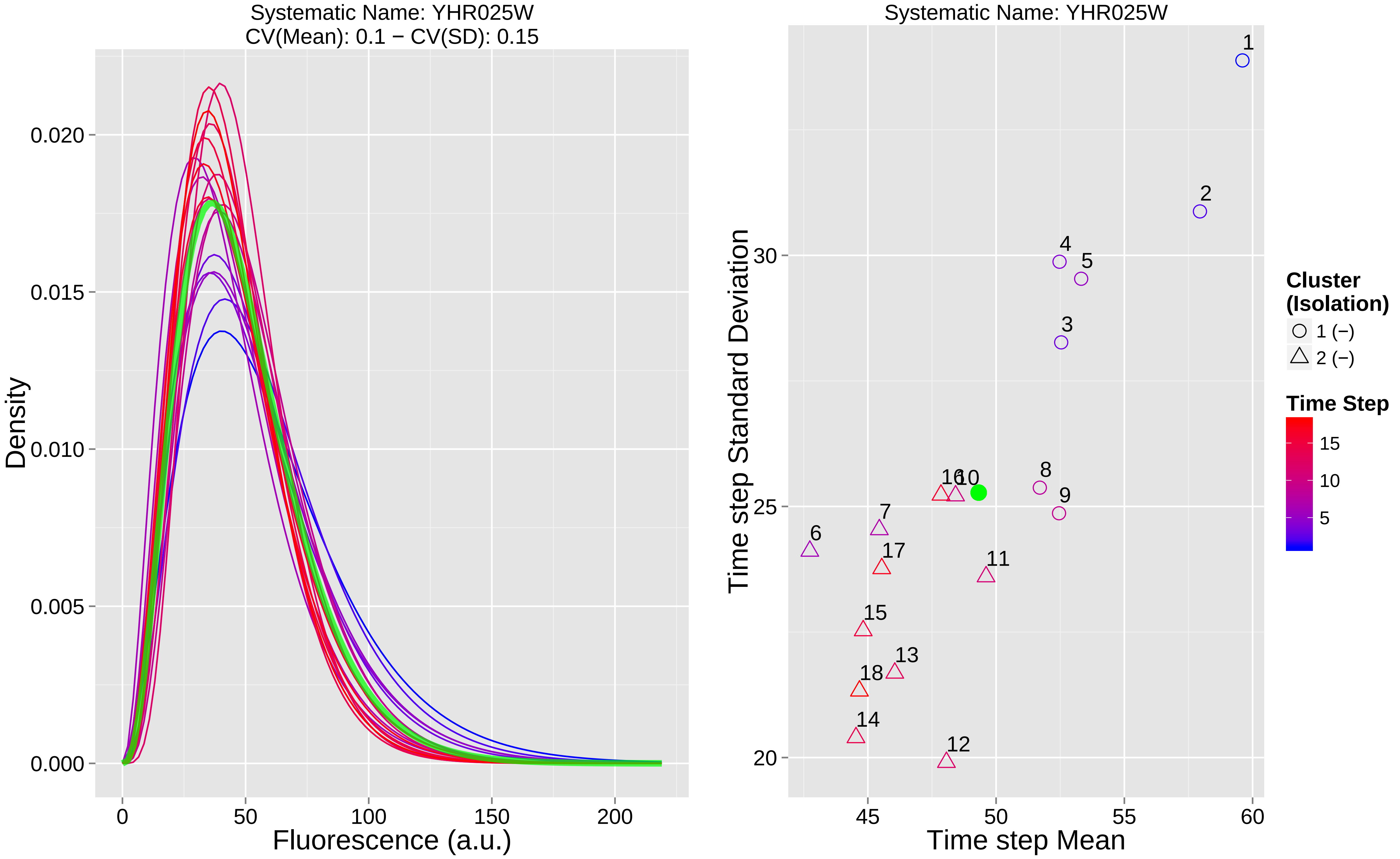

Systematic Name: YHR025W
CV(Mean): 0.1 − CV(SD): 0.15
Density
0.020
0.015
0.010
0.005
0.000
0
50
100
150
200
Fluorescence (a.u.)
Systematic Name: YHR025W
Time step Standard Deviation
30
25
20
45
50
55
60
Time step Mean
1
2
3
4
5
6
7
8
9
10
11
12
13
14
15
16
17
18
Cluster (Isolation)
1 (−)
2 (−)
Time Step
15
10
5

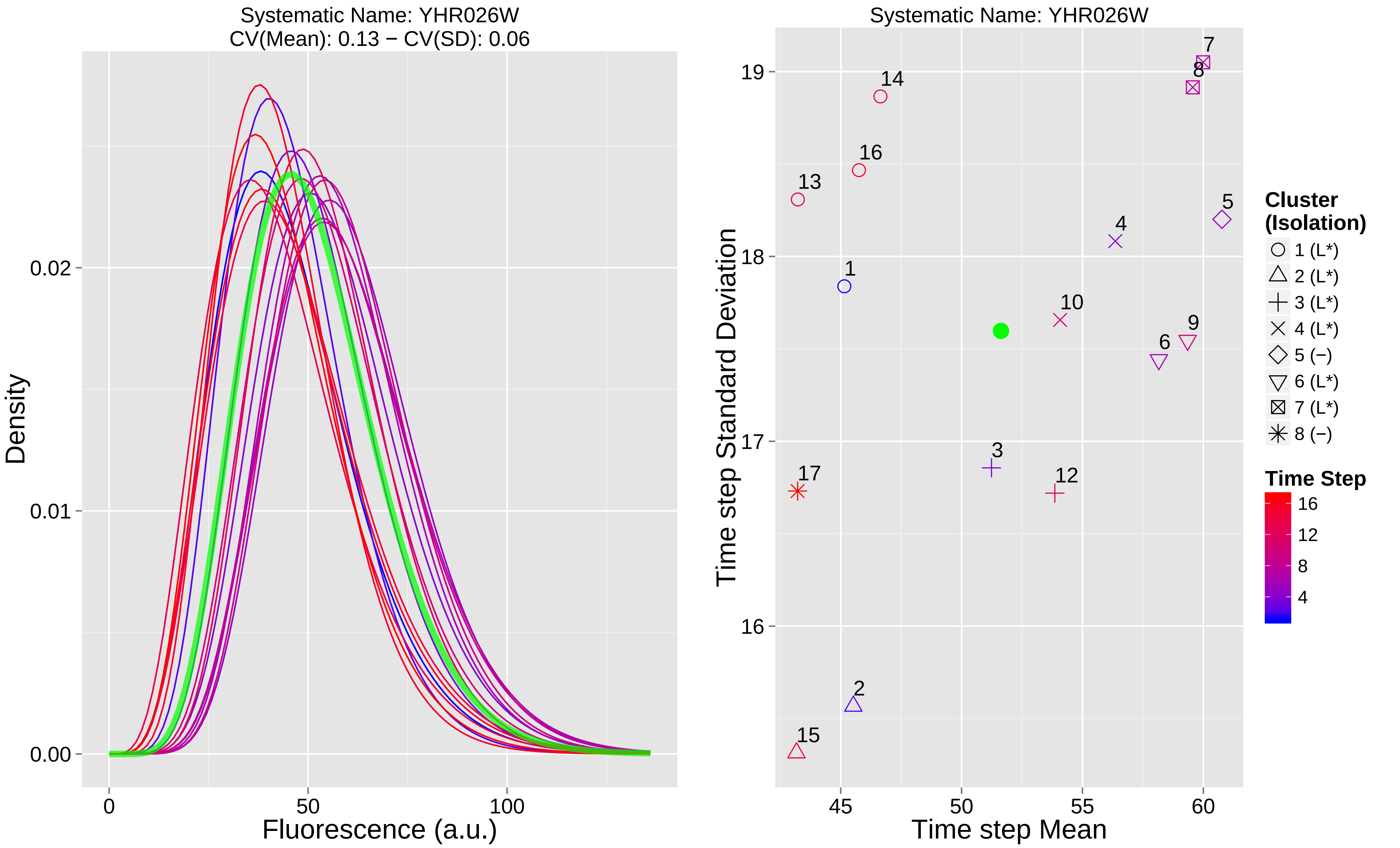

Systematic Name: YHR026W
CV(Mean): 0.13 − CV(SD): 0.06
Density
0.00
0.01
0.02
0
50
100
Fluorescence (a.u.)
Systematic Name: YHR026W
Time step Standard Deviation
16
17
18
19
45
50
55
60
Time step Mean
1
2
3
4
5
6
7
8
9
10
12
13
14
15
16
17
Cluster (Isolation)
1 (L*)
2 (L*)
3 (L*)
4 (L*)
5 (−)
6 (L*)
7 (L*)
8 (−)
Time Step
16
12
8
4

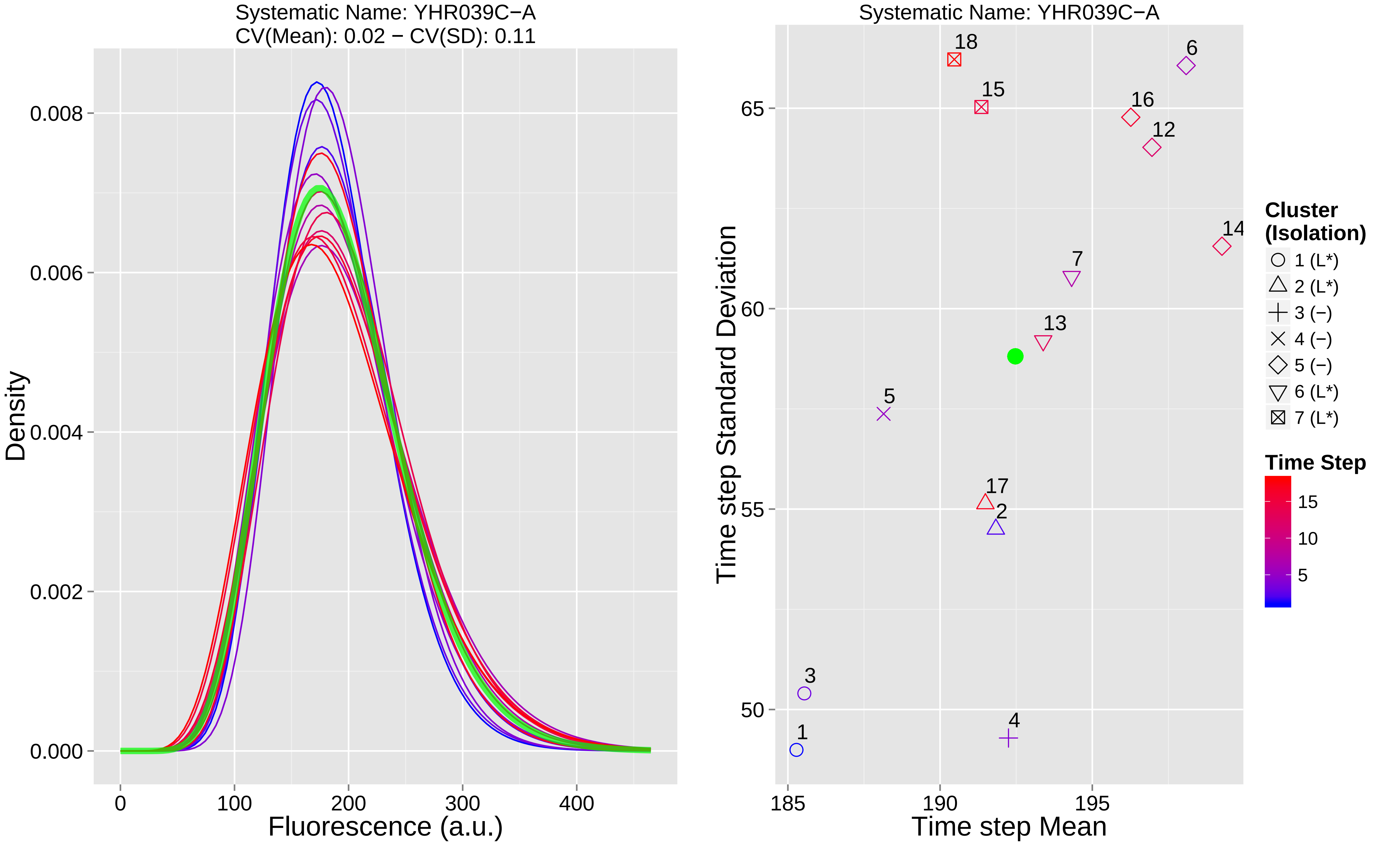
Systematic Name: YHR039C−A
CV(Mean): 0.02 − CV(SD): 0.11
Density
0.008
0.006
0.004
0.002
0.000
0
100
200
300
400
Fluorescence (a.u.)
Systematic Name: YHR039C−A
Time step Standard Deviation
65
60
55
50
185
190
195
Time step Mean
18
15
6
16
12
14
7
13
5
17
2
3
1
4
Cluster
(Isolation)
1 (L*)
2 (L*)
3 (−)
4 (−)
5 (−)
6 (L*)
7 (L*)
Time Step
15
10
5

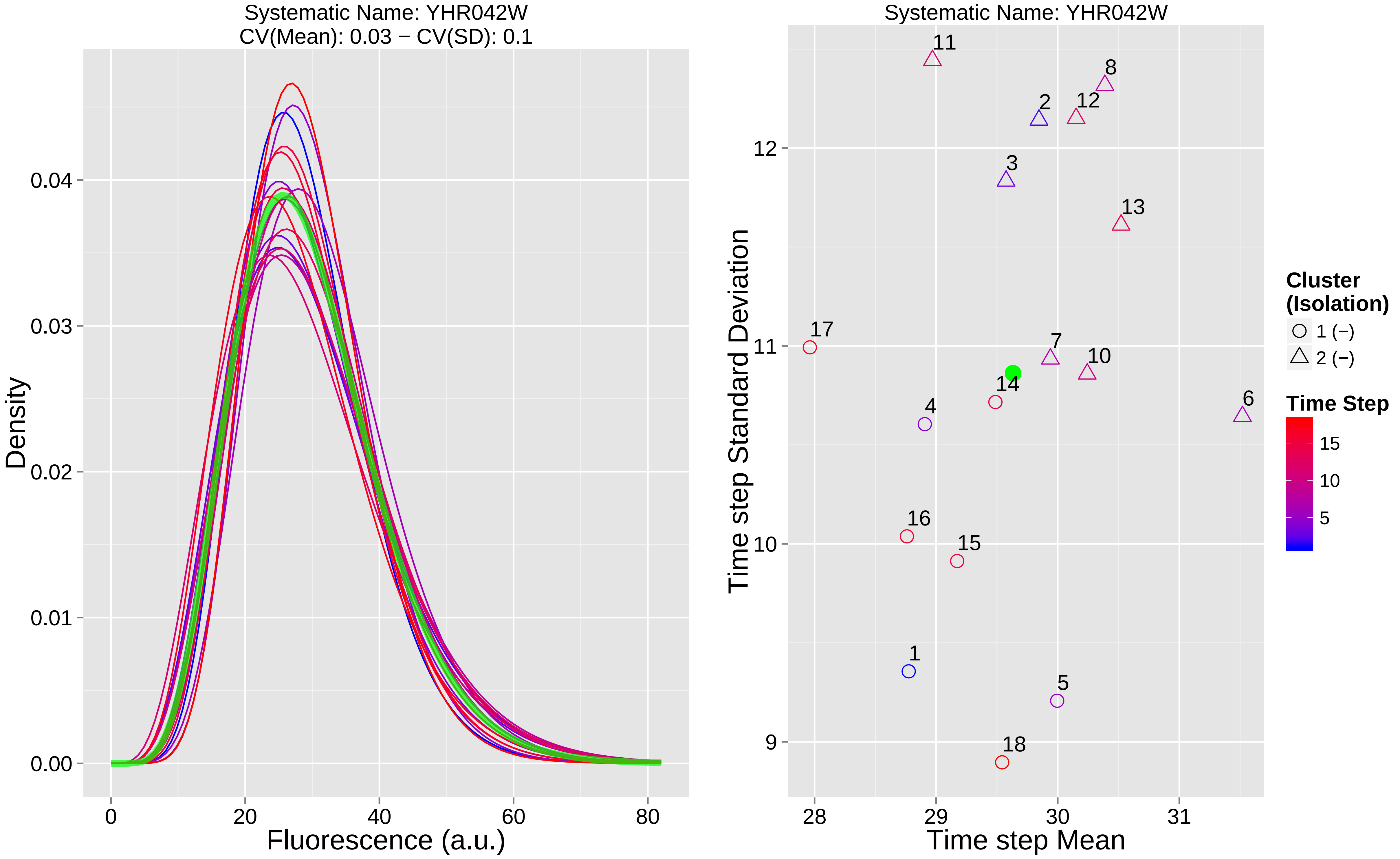
Systematic Name: YHR042W
CV(Mean): 0.03 − CV(SD): 0.1
Density
0.00
0.01
0.02
0.03
0.04
0
20
40
60
80
Fluorescence (a.u.)
Systematic Name: YHR042W
Time step Standard Deviation
9
10
11
12
28
29
30
31
Time step Mean
1
2
3
4
5
6
7
8
10
11
12
13
14
15
16
17
18
Cluster
(Isolation)
1 (−)
2 (−)
Time Step
15
10
5

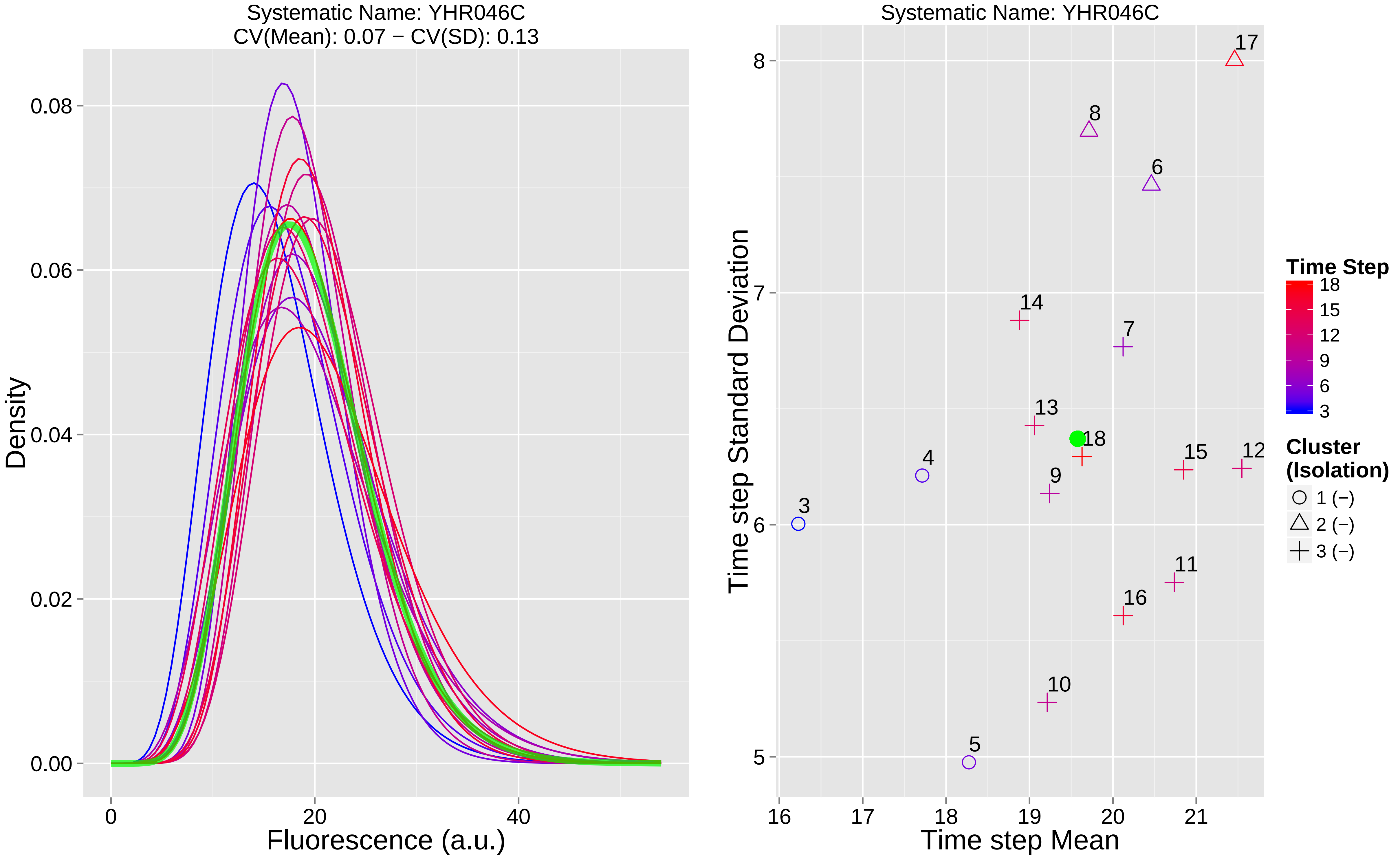

Systematic Name: YHR046C
CV(Mean): 0.07 − CV(SD): 0.13
Density
Fluorescence (a.u.)
0.00
0.02
0.04
0.06
0.08
0
20
40
Systematic Name: YHR046C
Time step Standard Deviation
Time step Mean
5
6
7
8
16
17
18
19
20
21
3
4
5
6
7
8
9
10
11
12
13
14
15
16
17
18
Time Step
18
15
12
9
6
3
Cluster
(Isolation)
1 (−)
2 (−)
3 (−)

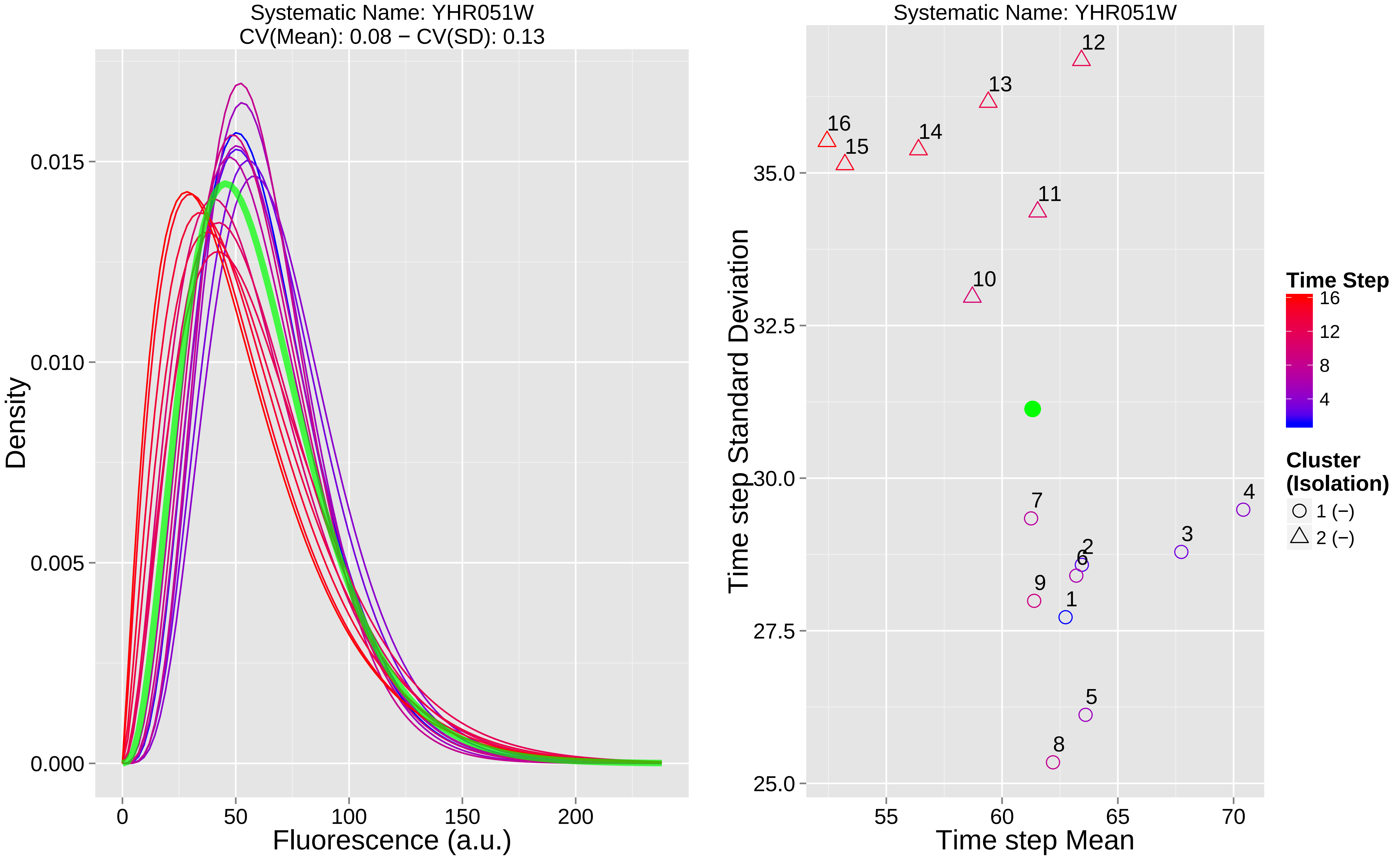
Systematic Name: YHR051W
CV(Mean): 0.08 − CV(SD): 0.13
Density
Fluorescence (a.u.)
0.000
0.005
0.010
0.015
0
50
100
150
200
Systematic Name: YHR051W
Time step Standard Deviation
Time step Mean
25.0
27.5
30.0
32.5
35.0
55
60
65
70
Time Step
16
12
8
4
Cluster
(Isolation)
1 (−)
2 (−)

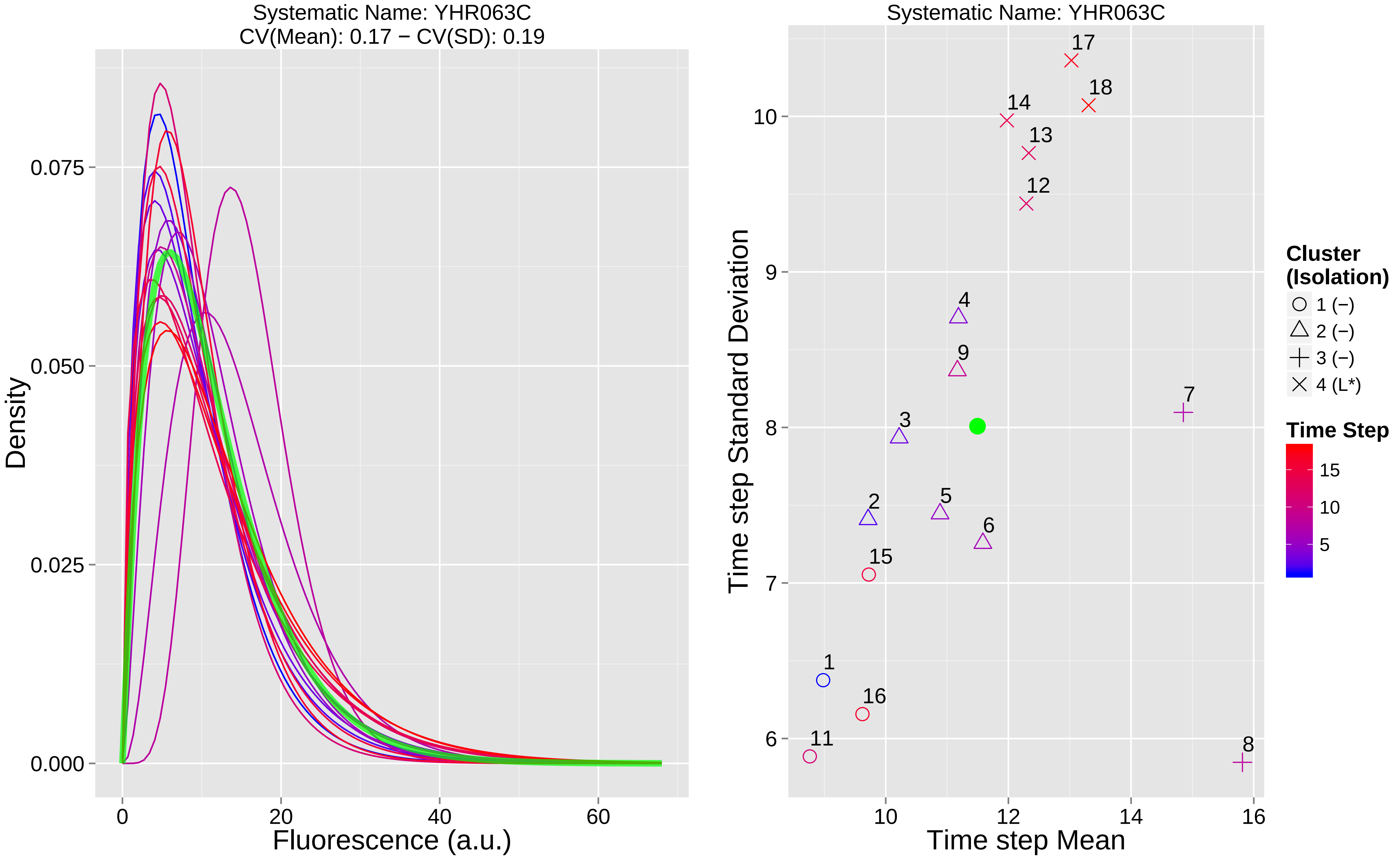

Systematic Name: YHR063C
CV(Mean): 0.17 − CV(SD): 0.19
Density
0.075
0.050
0.025
0.000
0
20
40
60
Fluorescence (a.u.)
Systematic Name: YHR063C
Time step Standard Deviation
10
9
8
7
6
Time step Mean
10
12
14
16
17
18
14
13
12
4
9
3
7
2
5
6
15
1
16
11
8
Cluster
(Isolation)
1 (−)
2 (−)
3 (−)
4 (L*)
Time Step
15
10
5

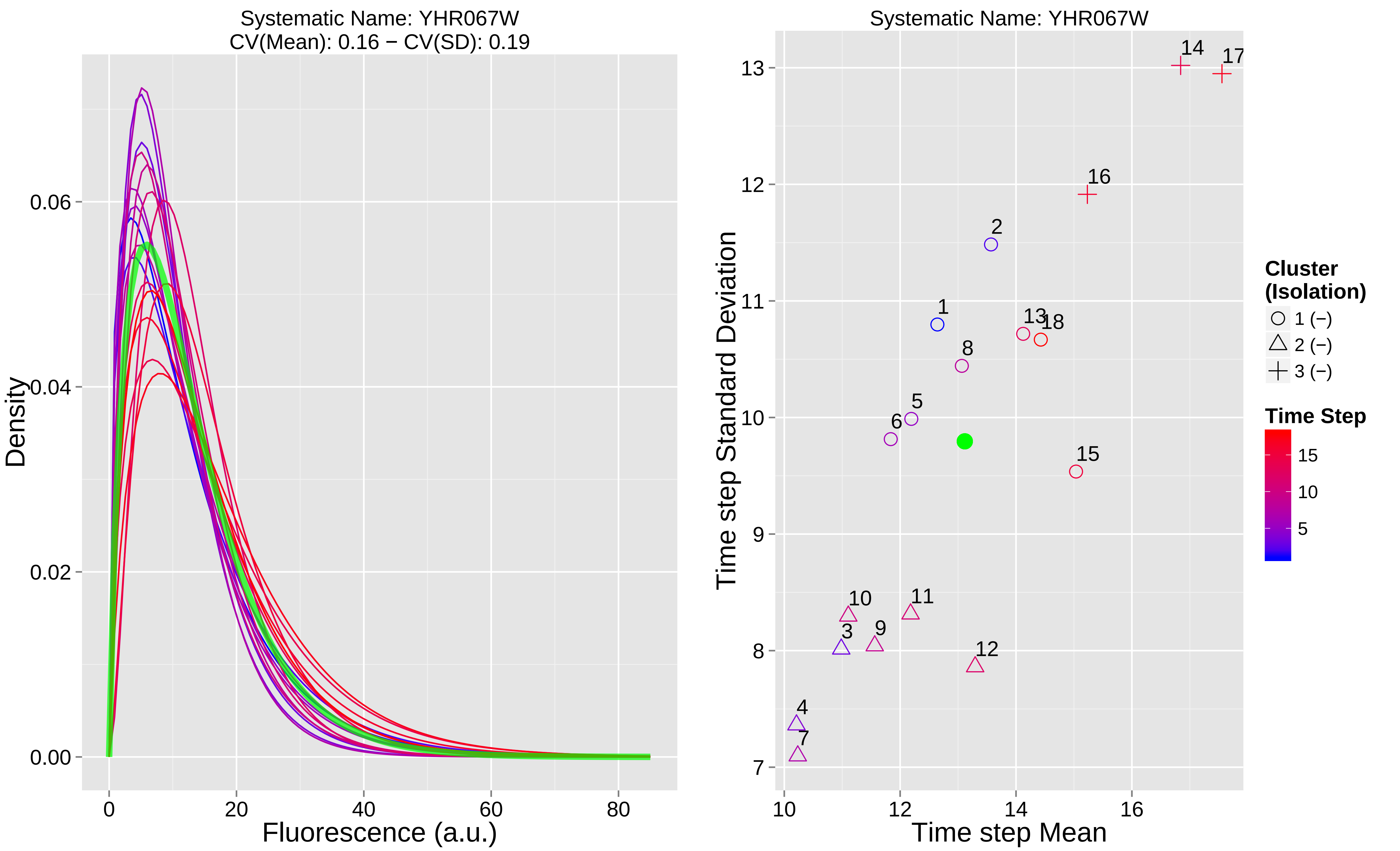
Systematic Name: YHR067W
CV(Mean): 0.16 − CV(SD): 0.19
Density
0.00
0.02
0.04
0.06
0
20
40
60
80
Fluorescence (a.u.)
Systematic Name: YHR067W
Time step Standard Deviation
7
8
9
10
11
12
13
10
12
14
16
Time step Mean
1
2
3
4
5
6
7
8
9
10
11
12
13
14
15
16
17
18
Cluster
(Isolation)
1 (−)
2 (−)
3 (−)
Time Step
15
10
5

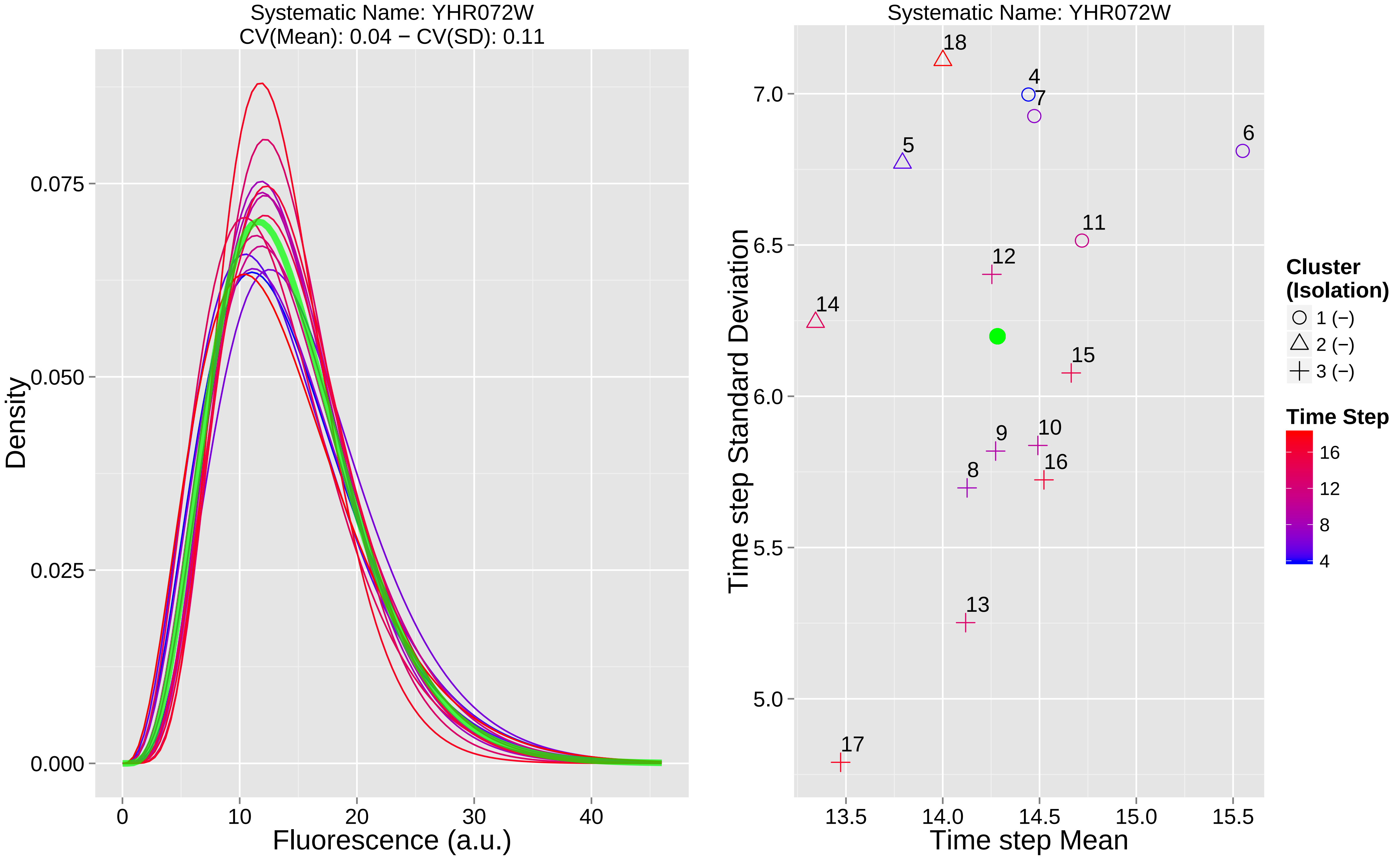
Systematic Name: YHR072W
CV(Mean): 0.04 − CV(SD): 0.11
Density
0.075
0.050
0.025
0.000
0
10
20
30
40
Fluorescence (a.u.)
Systematic Name: YHR072W
Time step Standard Deviation
7.0
6.5
6.0
5.5
5.0
13.5
14.0
14.5
15.0
15.5
Time step Mean
18
4
7
6
5
11
12
14
15
9
10
8
16
13
17
Cluster
(Isolation)
1 (−)
2 (−)
3 (−)
Time Step
16
12
8
4

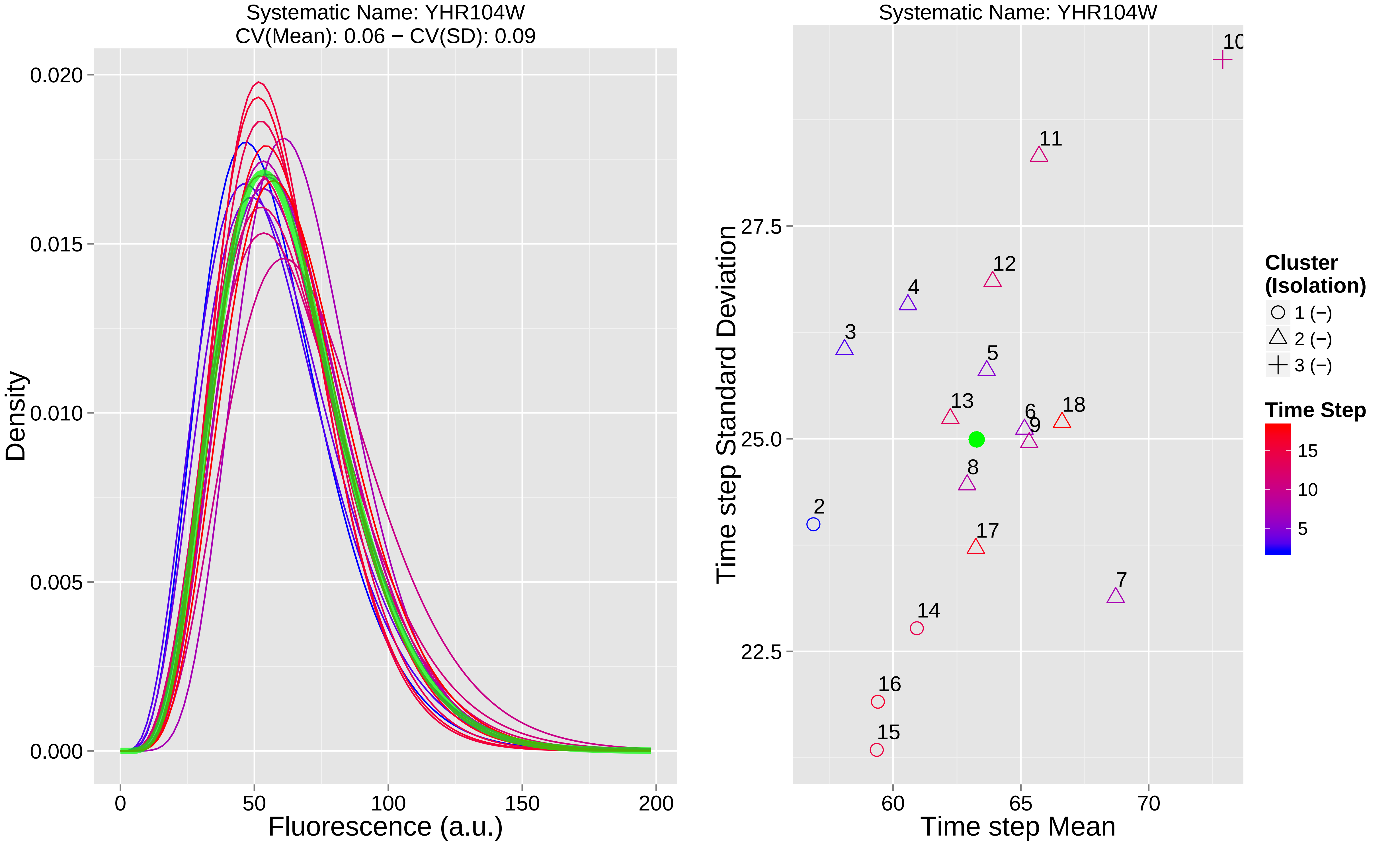

Systematic Name: YHR104W
CV(Mean): 0.06 − CV(SD): 0.09
Density
0.000
0.005
0.010
0.015
0.020
0
50
100
150
200
Fluorescence (a.u.)
Systematic Name: YHR104W
Time step Standard Deviation
22.5
25.0
27.5
60
65
70
Time step Mean
2
3
4
5
6
7
8
9
11
12
13
14
15
16
17
18
Cluster (Isolation)
1 (−)
2 (−)
3 (−)
Time Step
5
10
15

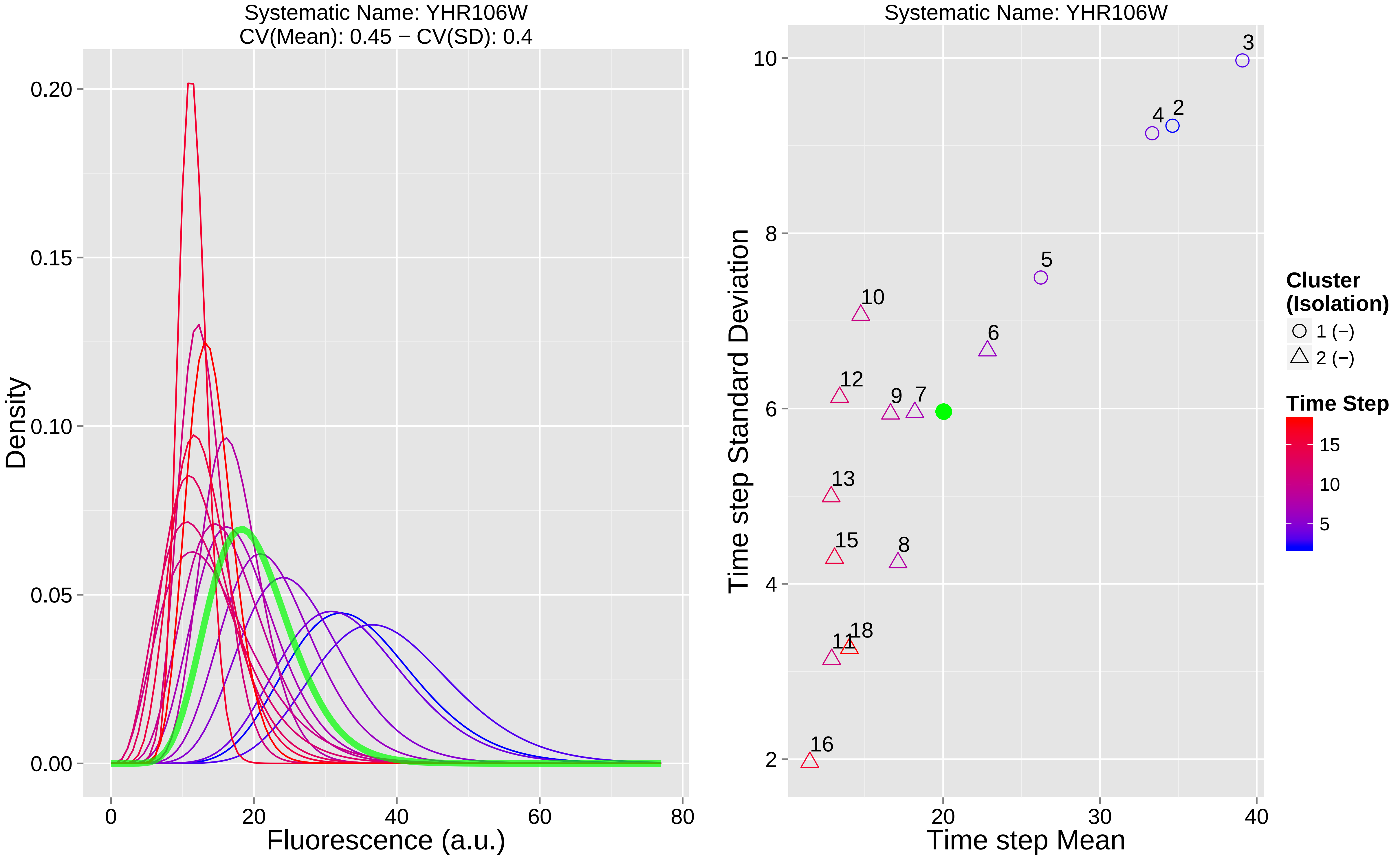

Systematic Name: YHR106W
CV(Mean): 0.45 − CV(SD): 0.4
Density
0.20
0.15
0.10
0.05
0.00
0
20
40
60
80
Fluorescence (a.u.)
Systematic Name: YHR106W
Time step Standard Deviation
10
8
6
4
2
20
30
40
Time step Mean
3
4
2
5
10
6
12
9
7
13
15
8
11
18
16
Cluster (Isolation)
1 (−)
2 (−)
Time Step
15
10
5

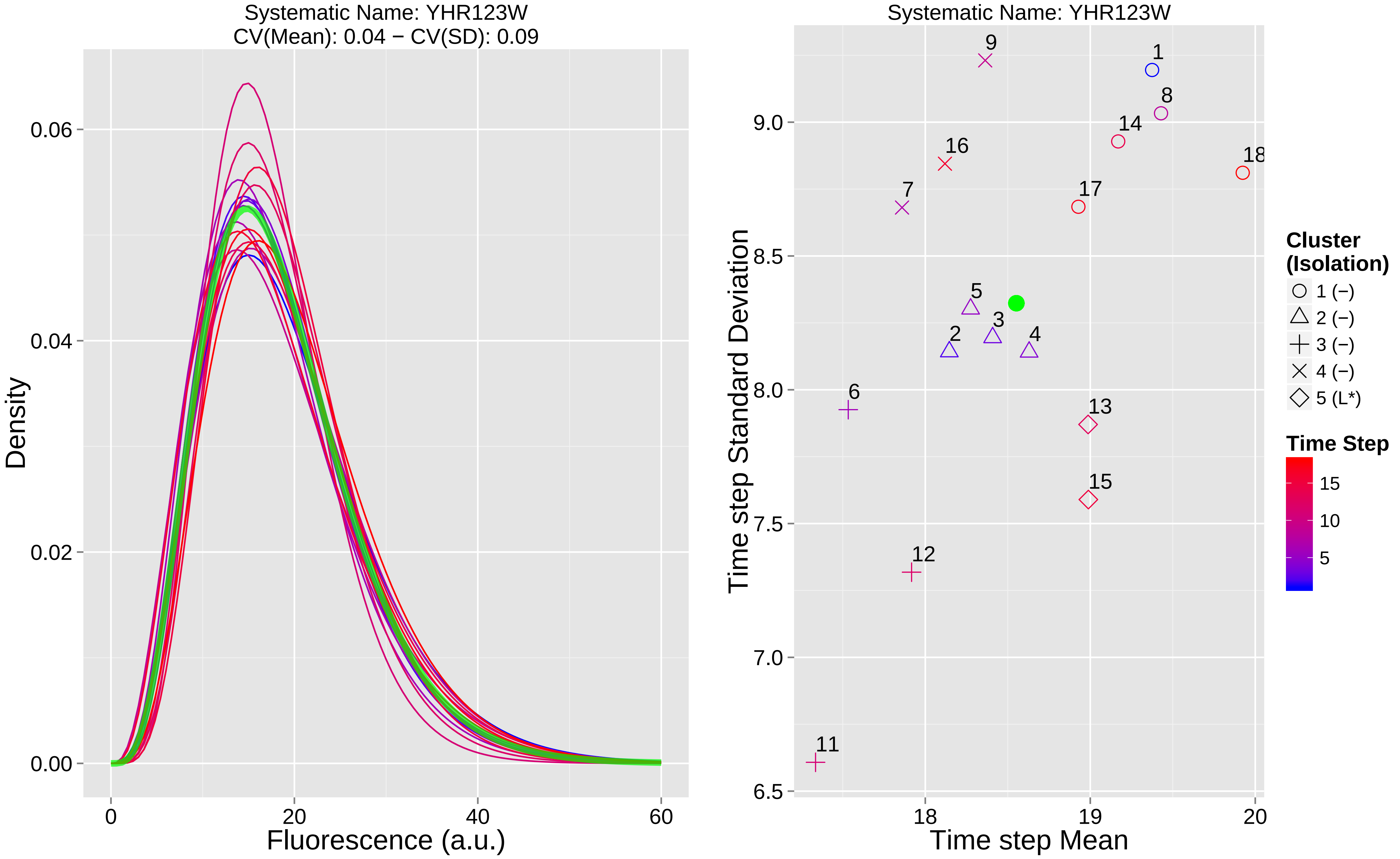

Systematic Name: YHR123W
CV(Mean): 0.04 − CV(SD): 0.09
Density
0.06
0.04
0.02
0.00
0
20
40
60
Fluorescence (a.u.)
Systematic Name: YHR123W
Time step Standard Deviation
9.0
8.5
8.0
7.5
7.0
6.5
18
19
20
Time step Mean
1
2
3
4
5
6
7
8
9
11
12
13
14
15
16
17
18
Cluster (Isolation)
1 (−)
2 (−)
3 (−)
4 (−)
5 (L*)
Time Step
15
10
5

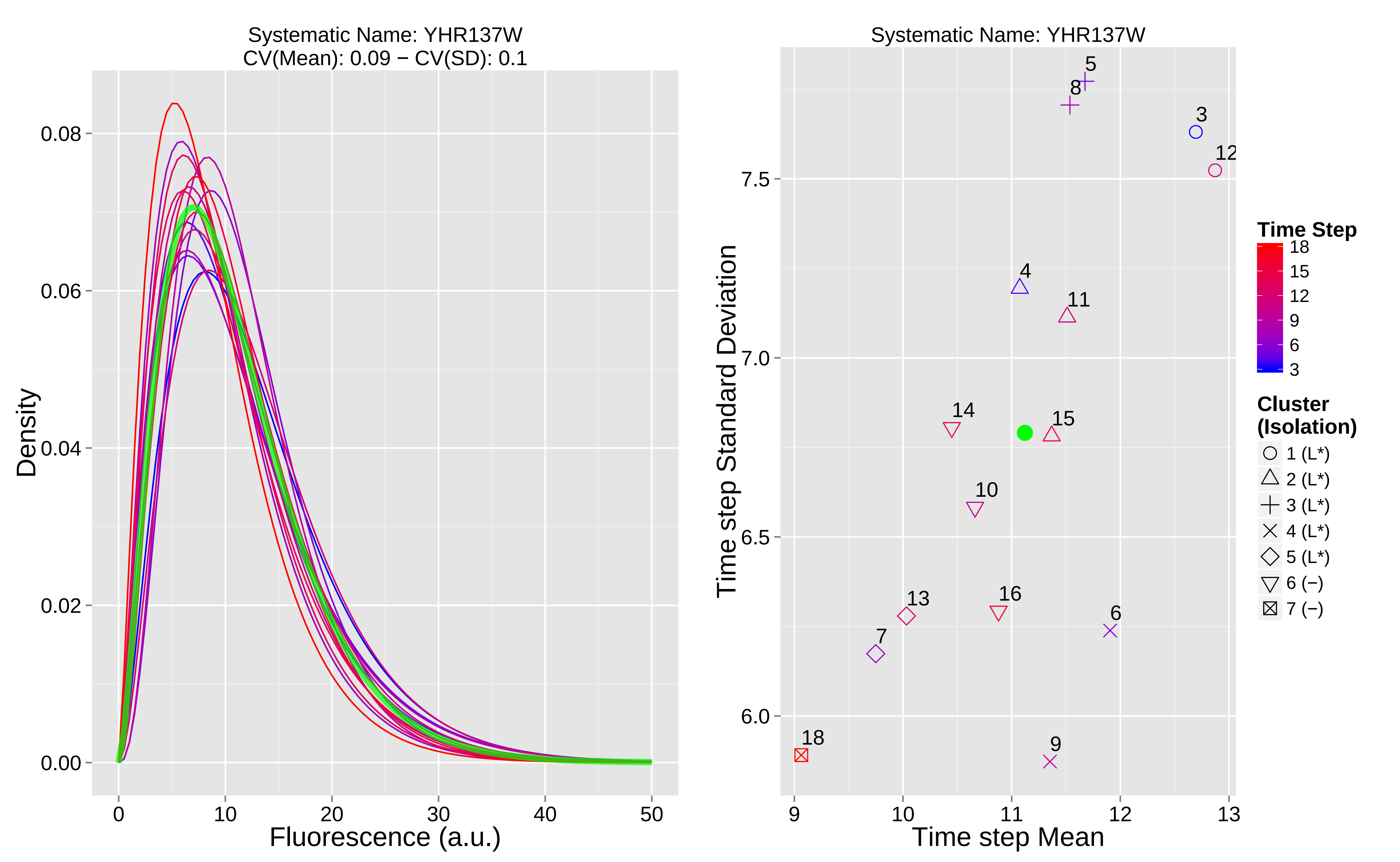
Systematic Name: YHR137W
CV(Mean): 0.09 − CV(SD): 0.1
Density
0.00
0.02
0.04
0.06
0.08
0
10
20
30
40
50
Fluorescence (a.u.)
Systematic Name: YHR137W
Time step Standard Deviation
6.0
6.5
7.0
7.5
9
10
11
12
13
Time step Mean
5
8
3
12
4
11
14
15
10
13
16
6
7
18
9
Time Step
18
15
12
9
6
3
Cluster (Isolation)
1 (L*)
2 (L*)
3 (L*)
4 (L*)
5 (L*)
6 (−)
7 (−)

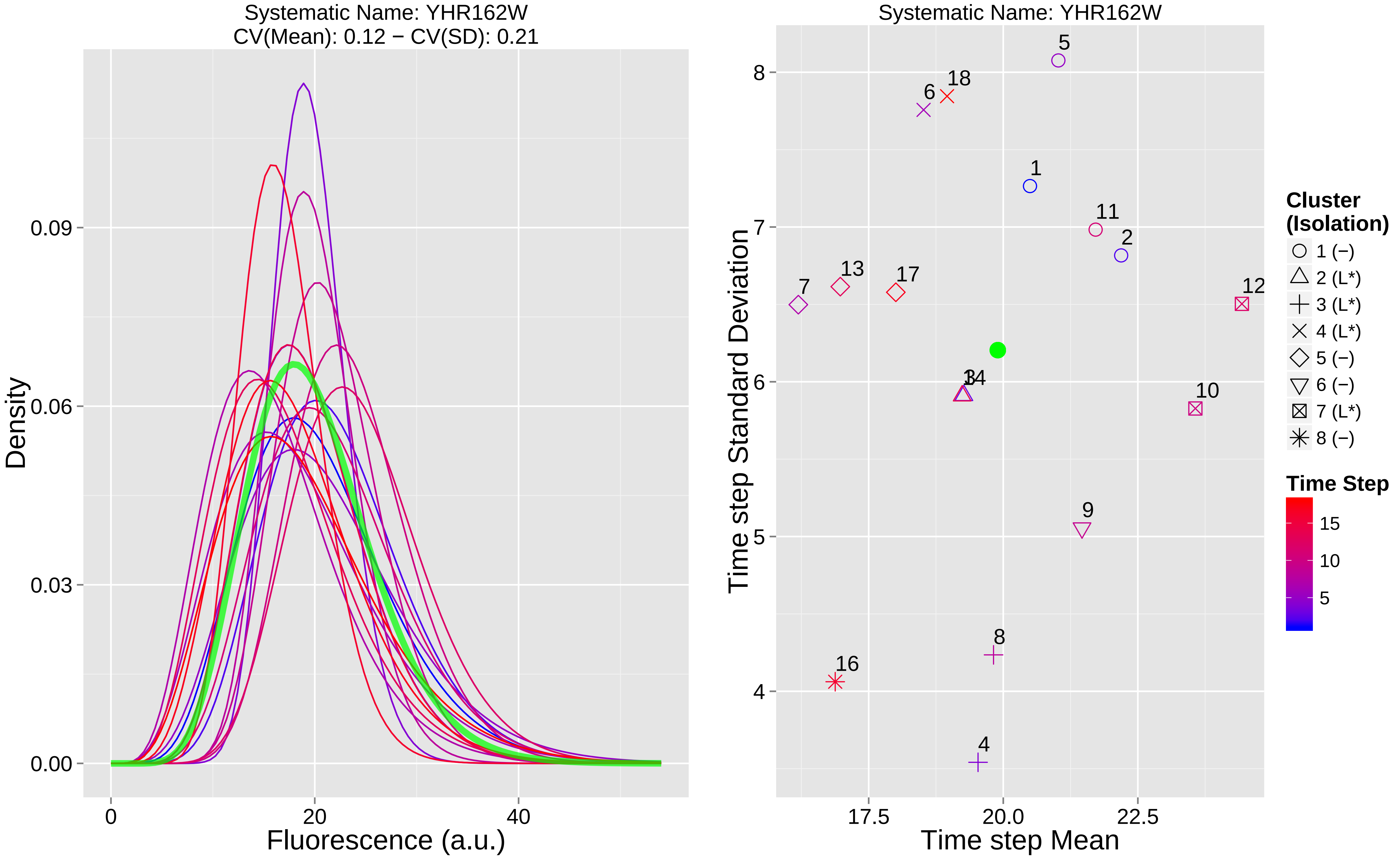
Systematic Name: YHR162W
CV(Mean): 0.12 − CV(SD): 0.21
Density
0.00
0.03
0.06
0.09
0
20
40
Fluorescence (a.u.)
Systematic Name: YHR162W
Time step Standard Deviation
Time step Mean
17.5
20.0
22.5
4
5
6
7
8
Cluster
(Isolation)
1 (−)
2 (L*)
3 (L*)
4 (L*)
5 (−)
6 (−)
7 (L*)
8 (−)
Time Step
5
10
15

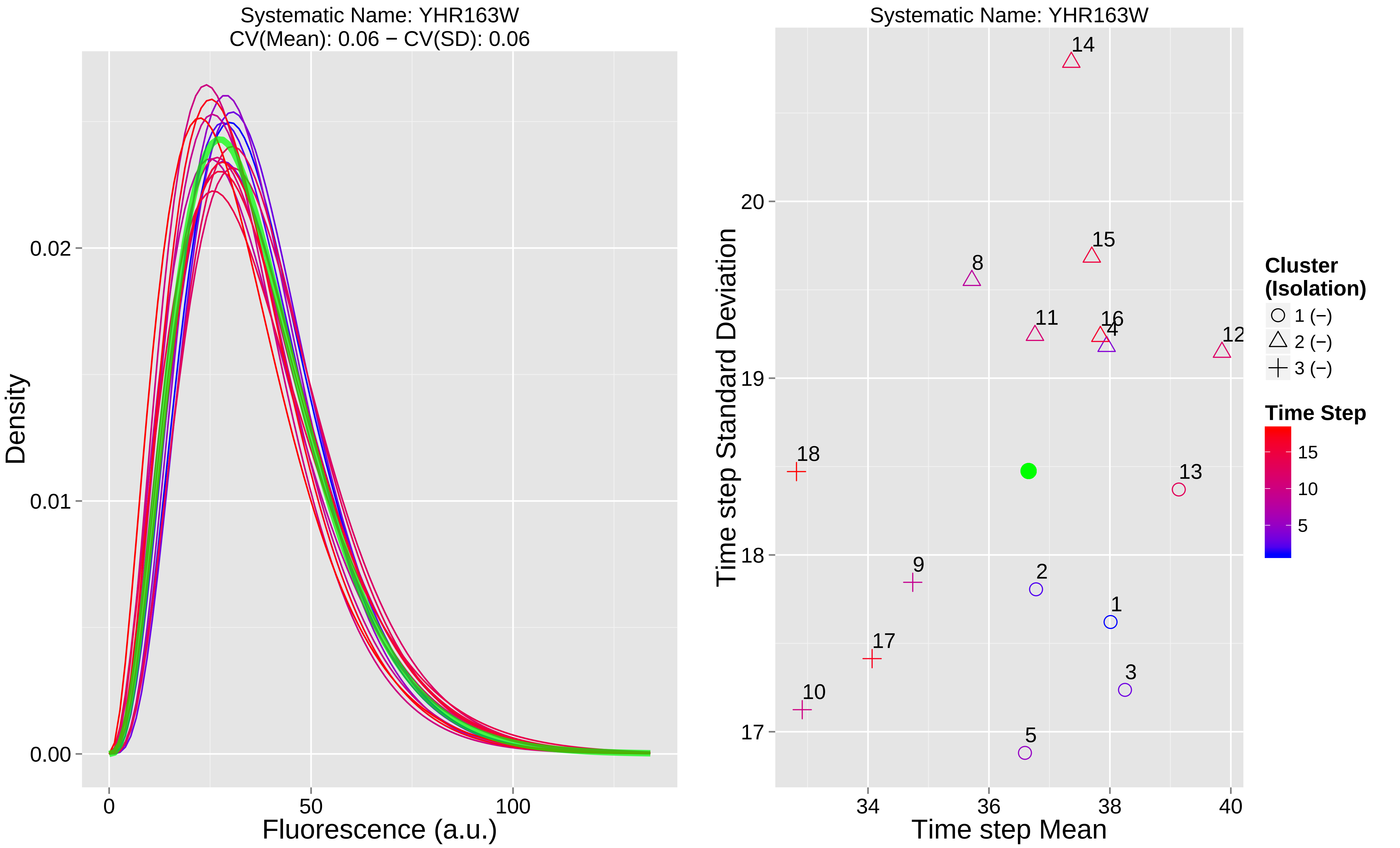

Systematic Name: YHR163W
CV(Mean): 0.06 − CV(SD): 0.06
Density
0.00
0.01
0.02
0
50
100
Fluorescence (a.u.)
Systematic Name: YHR163W
Time step Standard Deviation
17
18
19
20
34
36
38
40
Time step Mean
1
2
3
4
5
8
9
10
11
12
13
14
15
16
17
18
Cluster
(Isolation)
1 (−)
2 (−)
3 (−)
Time Step
5
10
15

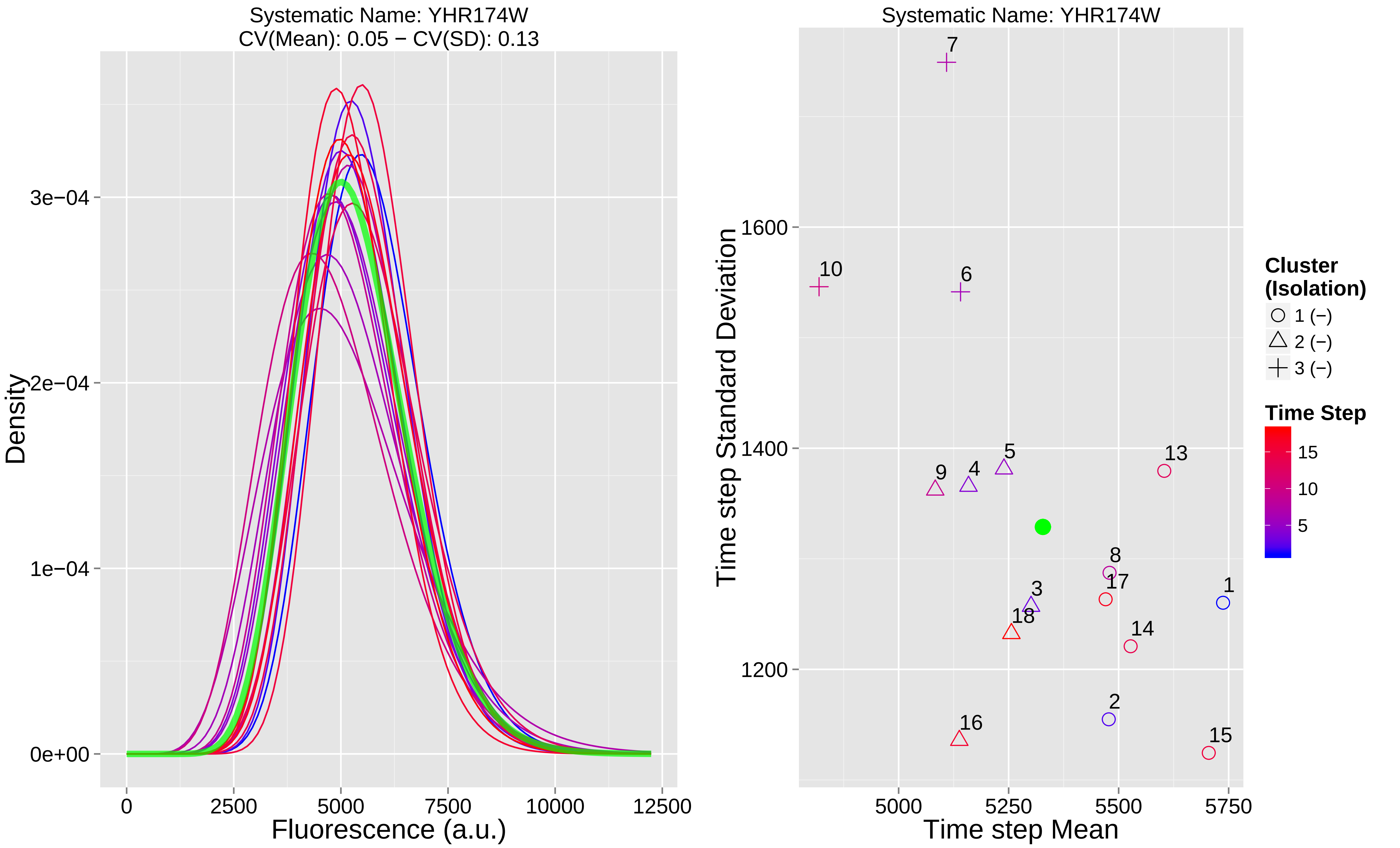

Systematic Name: YHR174W
CV(Mean): 0.05 − CV(SD): 0.13
Density
Fluorescence (a.u.)
0e+00
1e−04
2e−04
3e−04
0
2500
5000
7500
10000
12500
Systematic Name: YHR174W
Time step Standard Deviation
Time step Mean
1200
1400
1600
5000
5250
5500
5750
1
2
3
4
5
6
7
8
9
10
13
14
15
16
17
18
Cluster (Isolation)
1 (−)
2 (−)
3 (−)
Time Step
5
10
15

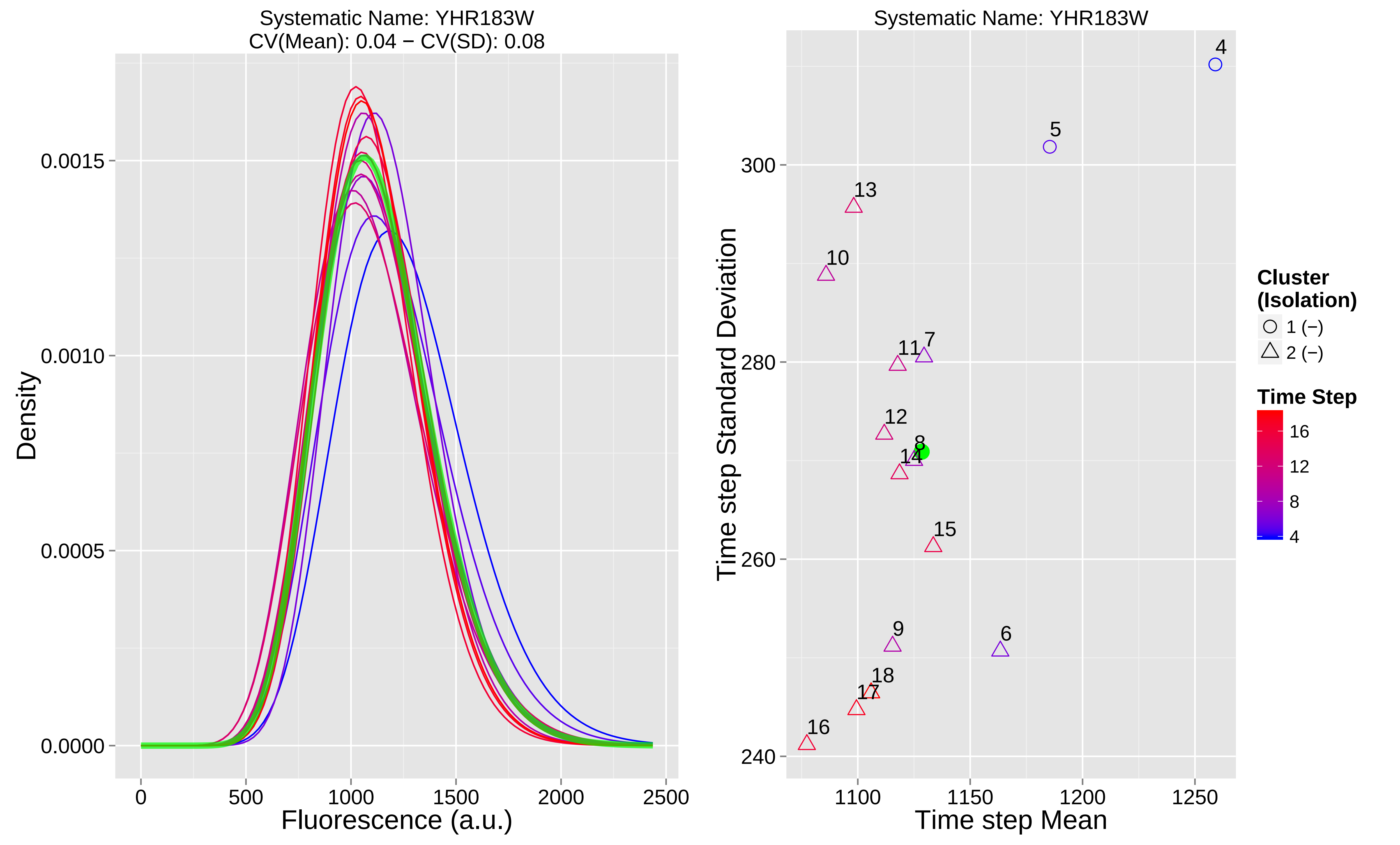
Systematic Name: YHR183W
CV(Mean): 0.04 − CV(SD): 0.08
Density
0.0015
0.0010
0.0005
0.0000
0
500
1000
1500
2000
2500
Fluorescence (a.u.)
Systematic Name: YHR183W
Time step Standard Deviation
300
280
260
240
1100
1150
1200
1250
Time step Mean
4
5
13
10
11
7
12
8
14
15
9
6
18
17
16
Cluster
(Isolation)
1 (−)
2 (−)
Time Step
16
12
8
4

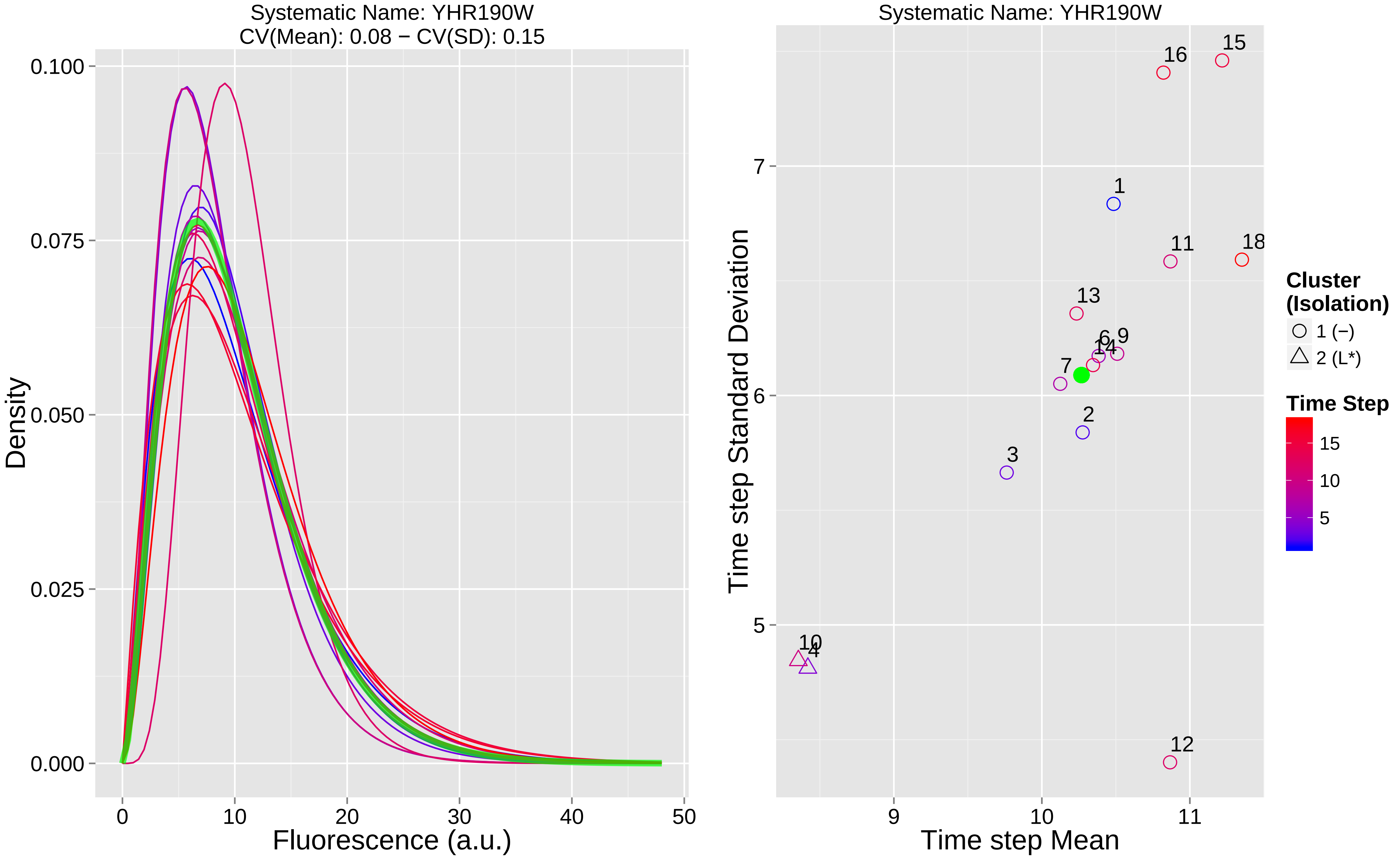
Systematic Name: YHR190W
CV(Mean): 0.08 − CV(SD): 0.15
Density
Fluorescence (a.u.)
0.100
0.075
0.050
0.025
0.000
0
10
20
30
40
50
Systematic Name: YHR190W
Time step Standard Deviation
Time step Mean
7
6
5
9
10
11
16
15
1
11
18
13
6
9
14
7
2
3
10
4
12
Cluster (Isolation)
1 (−)
2 (L*)
Time Step
15
10
5

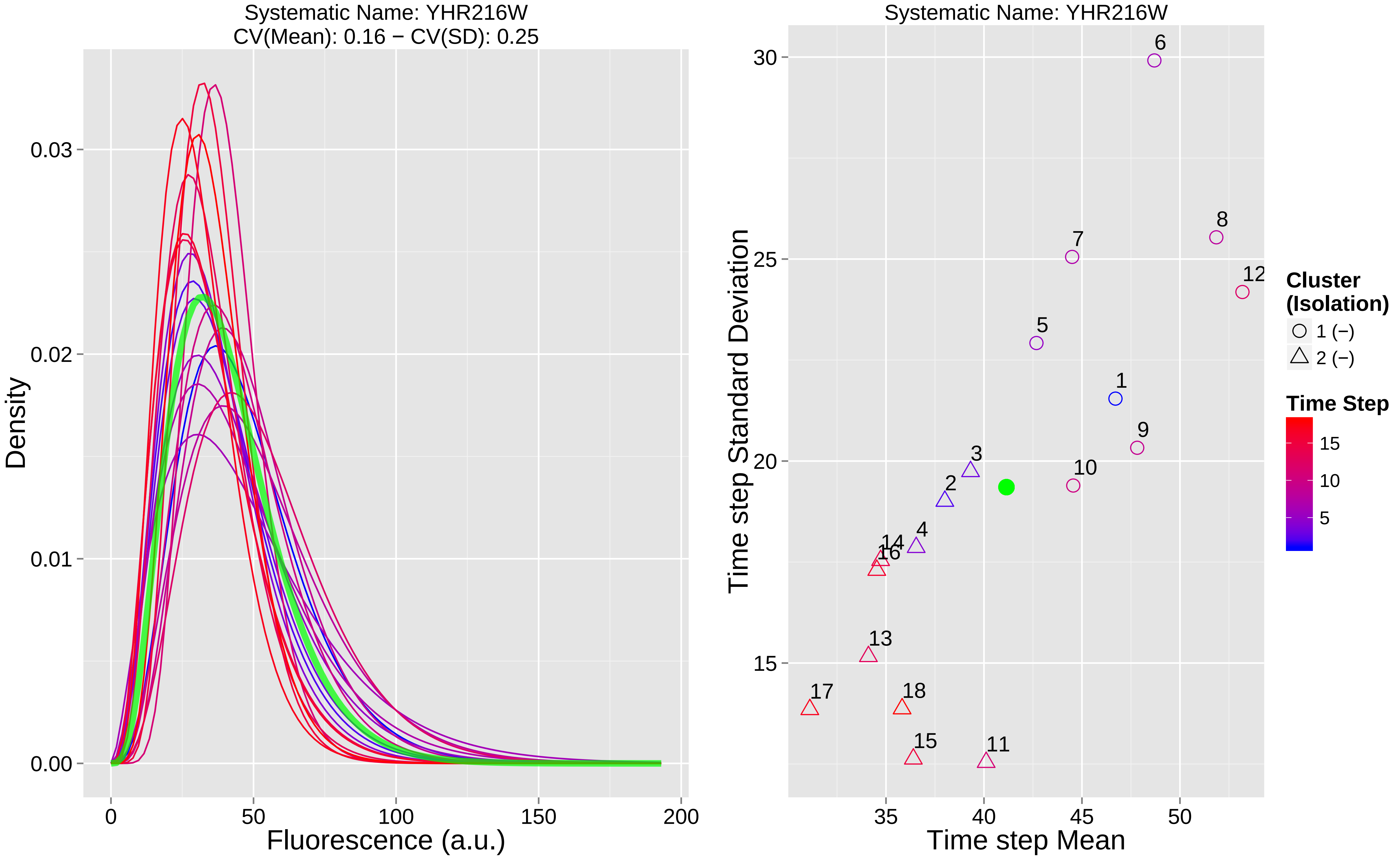

Systematic Name: YHR216W
CV(Mean): 0.16 − CV(SD): 0.25
Density
0.00
0.01
0.02
0.03
0
50
100
150
200
Fluorescence (a.u.)
Systematic Name: YHR216W
Time step Standard Deviation
15
20
25
30
35
40
45
50
Time step Mean
1
2
3
4
5
6
7
8
9
10
11
12
13
14
15
16
17
18
Cluster
(Isolation)
1 (−)
2 (−)
Time Step
15
10
5

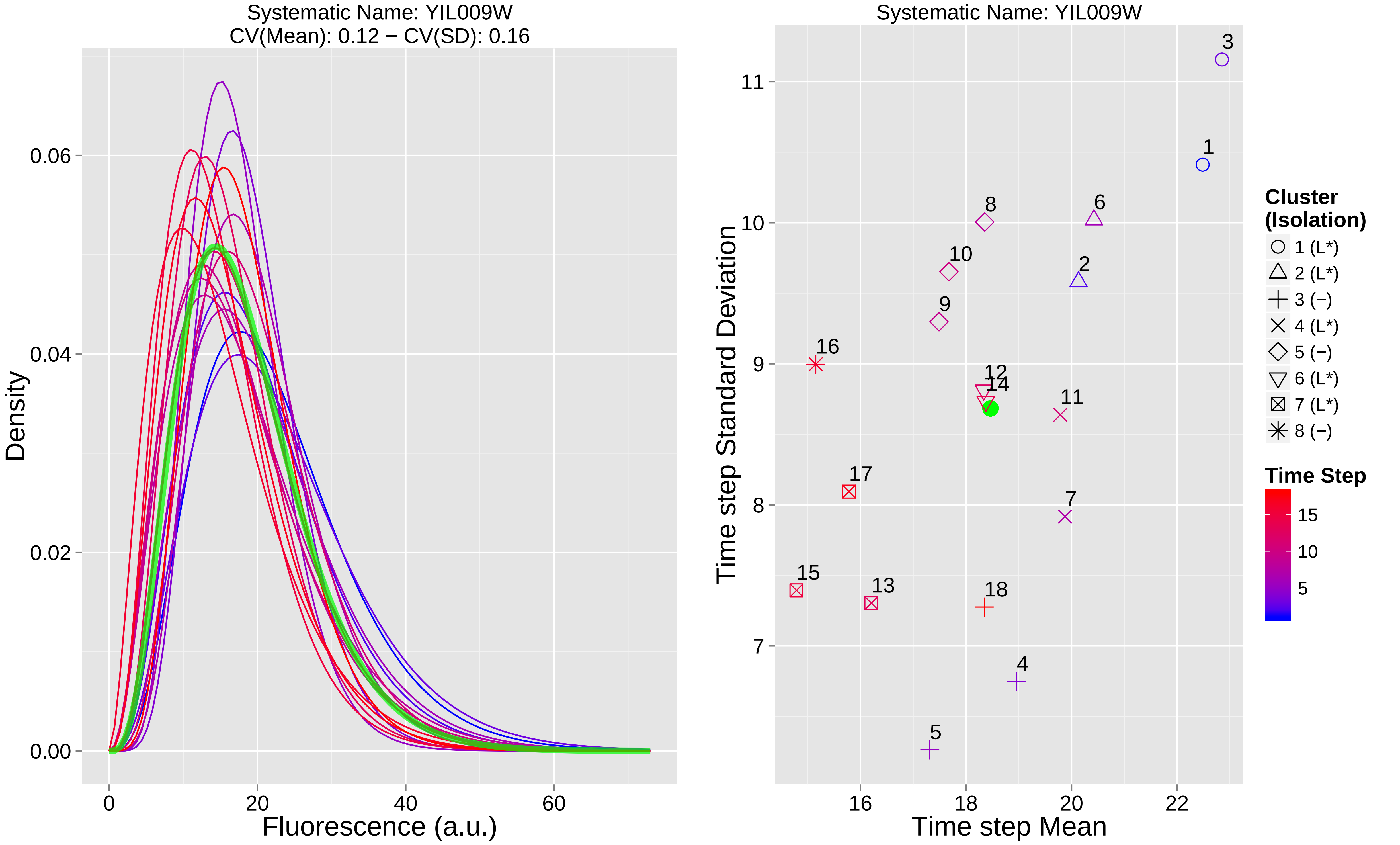

Systematic Name: YIL009W
CV(Mean): 0.12 − CV(SD): 0.16
Density
0.06
0.04
0.02
0.00
0
20
40
60
Fluorescence (a.u.)
Systematic Name: YIL009W
Time step Standard Deviation
11
10
9
8
7
16
18
20
22
Time step Mean
Cluster
(Isolation)
1 (L*)
2 (L*)
3 (−)
4 (L*)
5 (−)
6 (L*)
7 (L*)
8 (−)
Time Step
15
10
5

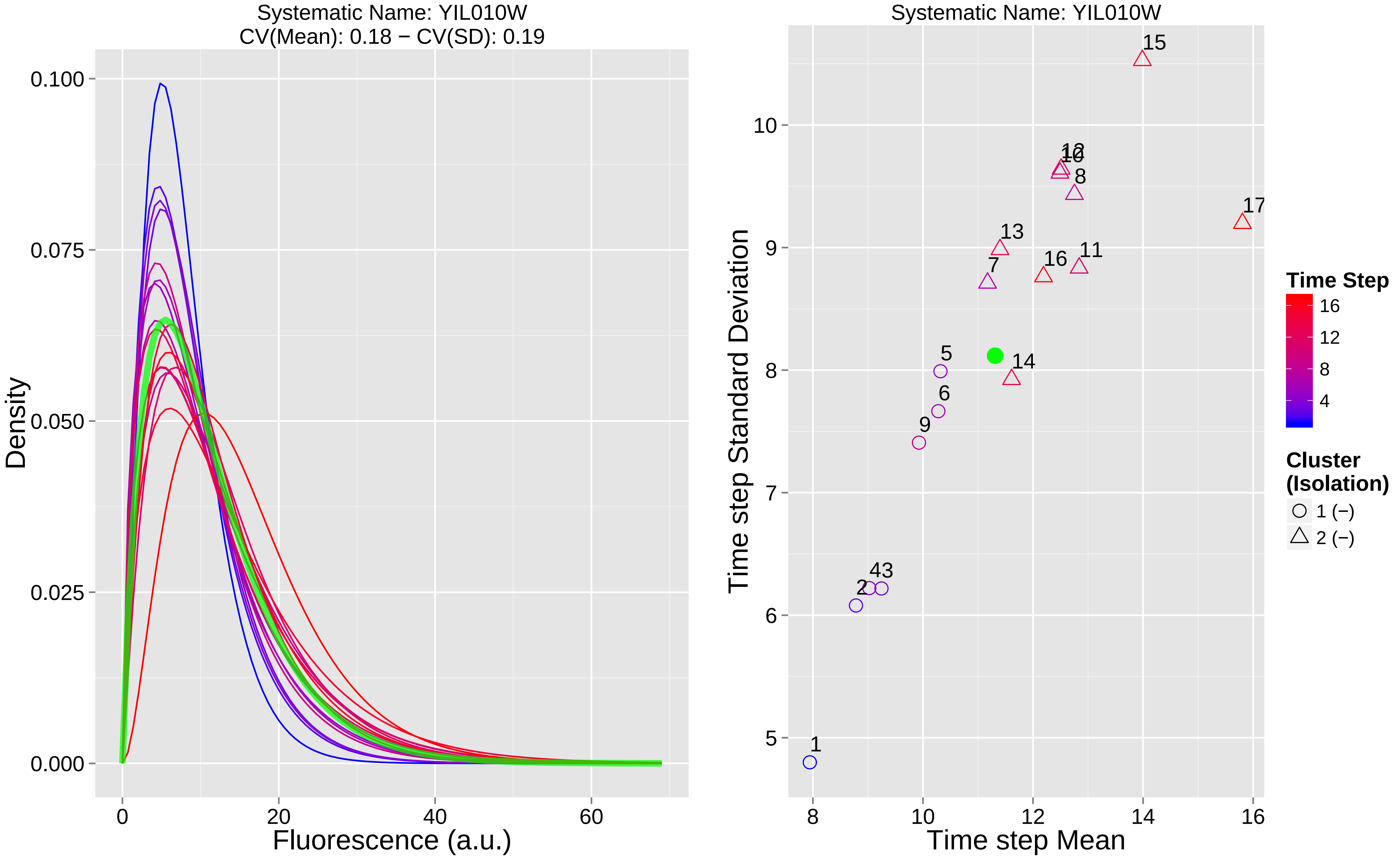

Systematic Name: YIL010W
CV(Mean): 0.18 − CV(SD): 0.19
Density
Fluorescence (a.u.)
Systematic Name: YIL010W
Time step Standard Deviation
Time step Mean
Time Step
Cluster (Isolation)
1 (−)
2 (−)

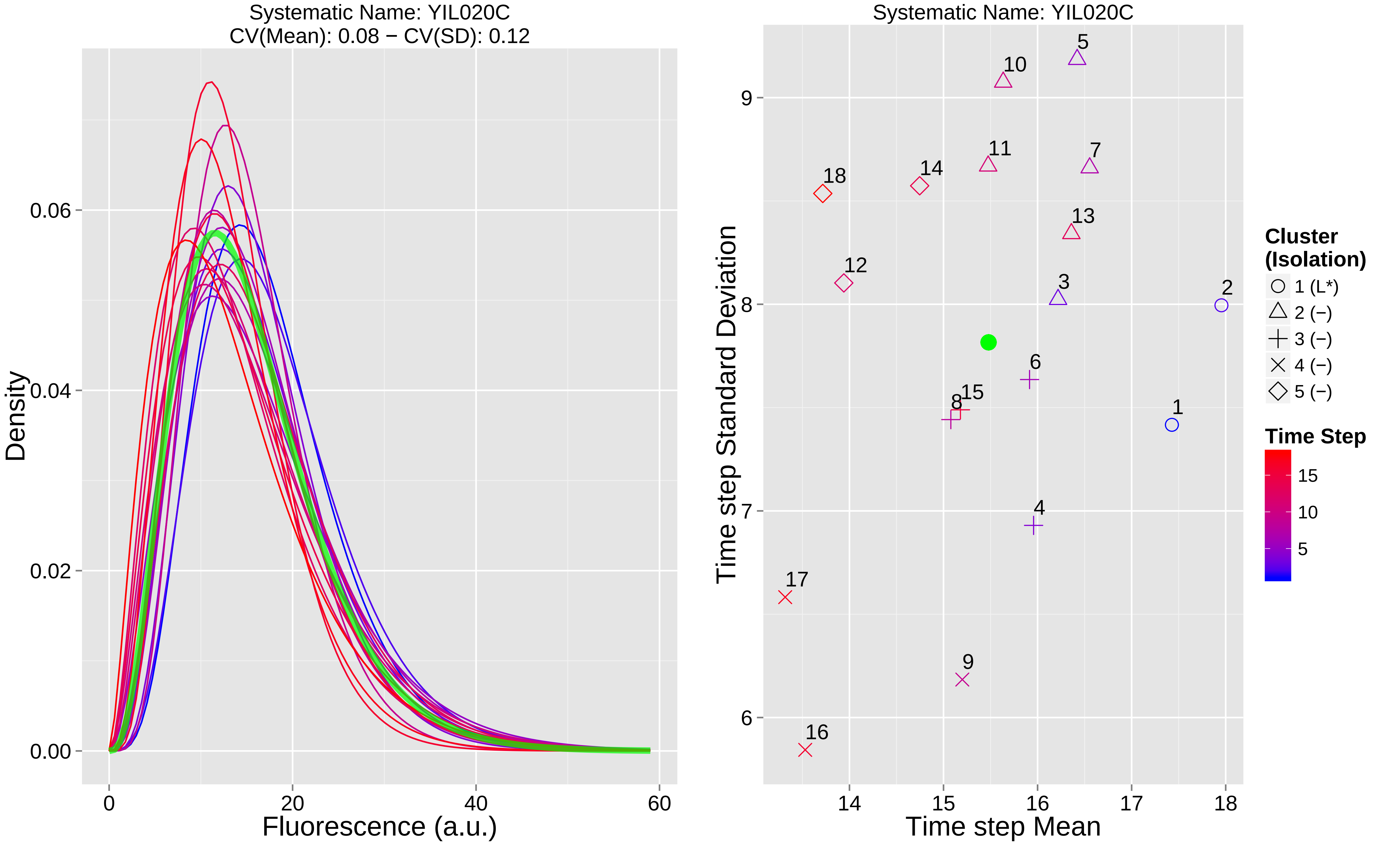

Systematic Name: YIL020C
CV(Mean): 0.08 − CV(SD): 0.12
Density
Fluorescence (a.u.)
0.00
0.02
0.04
0.06
0
20
40
60
Systematic Name: YIL020C
Time step Standard Deviation
Time step Mean
6
7
8
9
14
15
16
17
18
1
2
3
4
5
6
7
8
9
10
11
12
13
14
15
16
17
18
Cluster (Isolation)
1 (L*)
2 (−)
3 (−)
4 (−)
5 (−)
Time Step
5
10
15

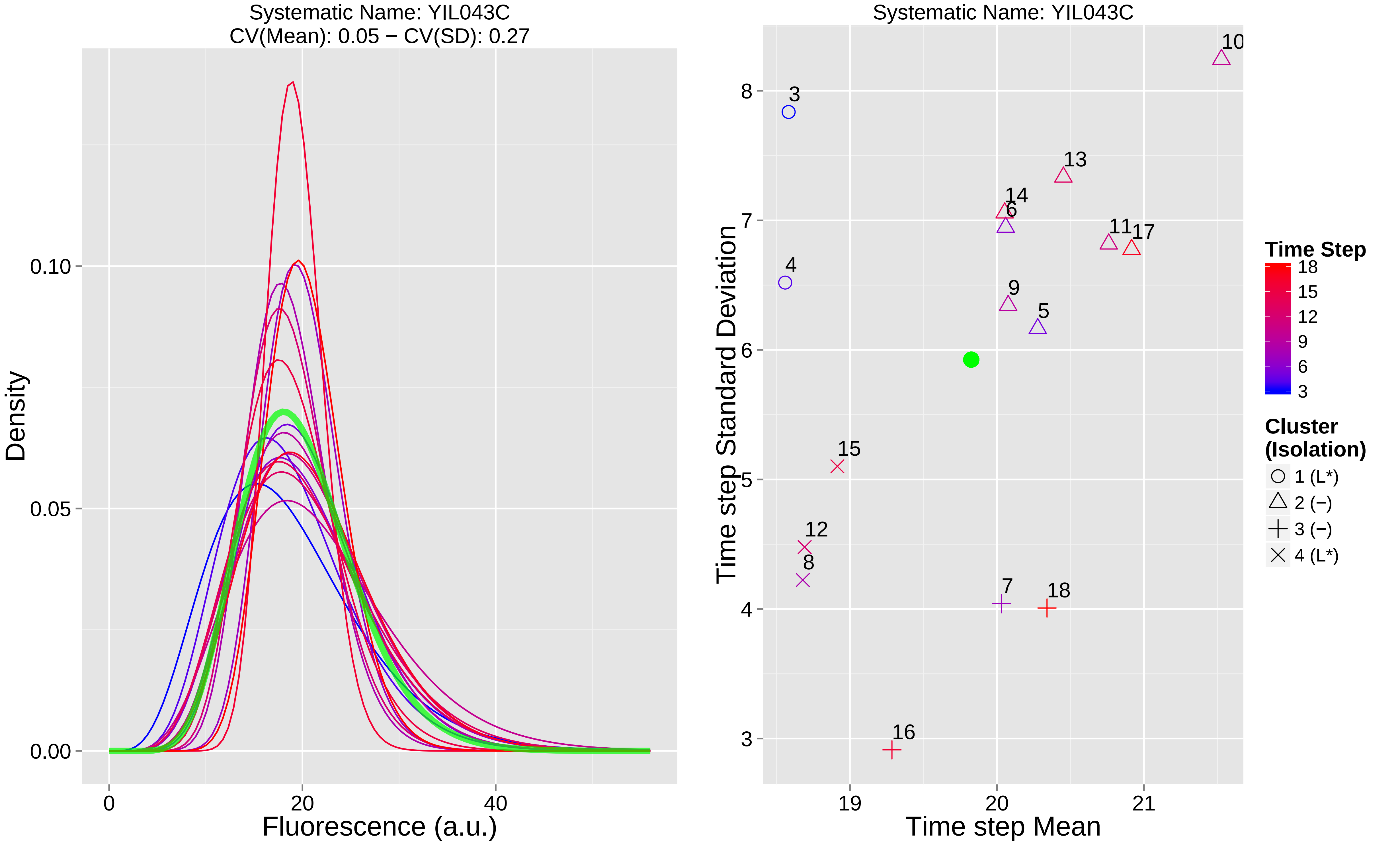

Systematic Name: YIL043C
CV(Mean): 0.05 − CV(SD): 0.27
Density
0.10
0.05
0.00
0
20
40
Fluorescence (a.u.)
Systematic Name: YIL043C
Time step Standard Deviation
8
7
6
5
4
3
19
20
21
Time step Mean
3
4
14
6
13
11
17
10
9
5
15
12
8
7
18
16
Time Step
18
15
12
9
6
3
Cluster
(Isolation)
1 (L*)
2 (−)
3 (−)
4 (L*)

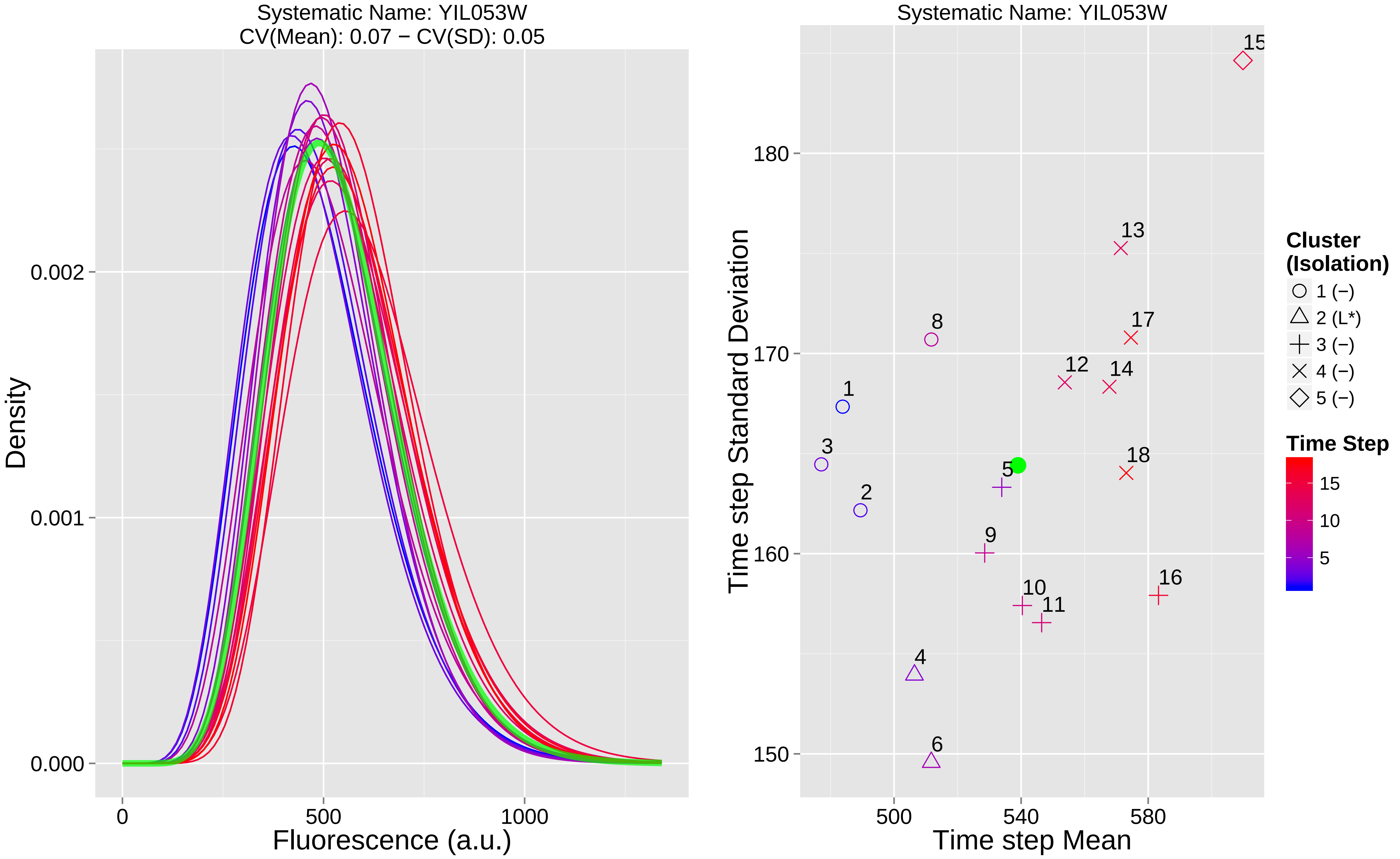

Systematic Name: YIL053W
CV(Mean): 0.07 − CV(SD): 0.05
Density
0.000
0.001
0.002
0
500
1000
Fluorescence (a.u.)
Systematic Name: YIL053W
Time step Standard Deviation
150
160
170
180
500
540
580
Time step Mean
1
2
3
4
5
6
8
9
10
11
12
13
14
15
16
17
18
Cluster (Isolation)
1 (−)
2 (L*)
3 (−)
4 (−)
5 (−)
Time Step
5
10
15

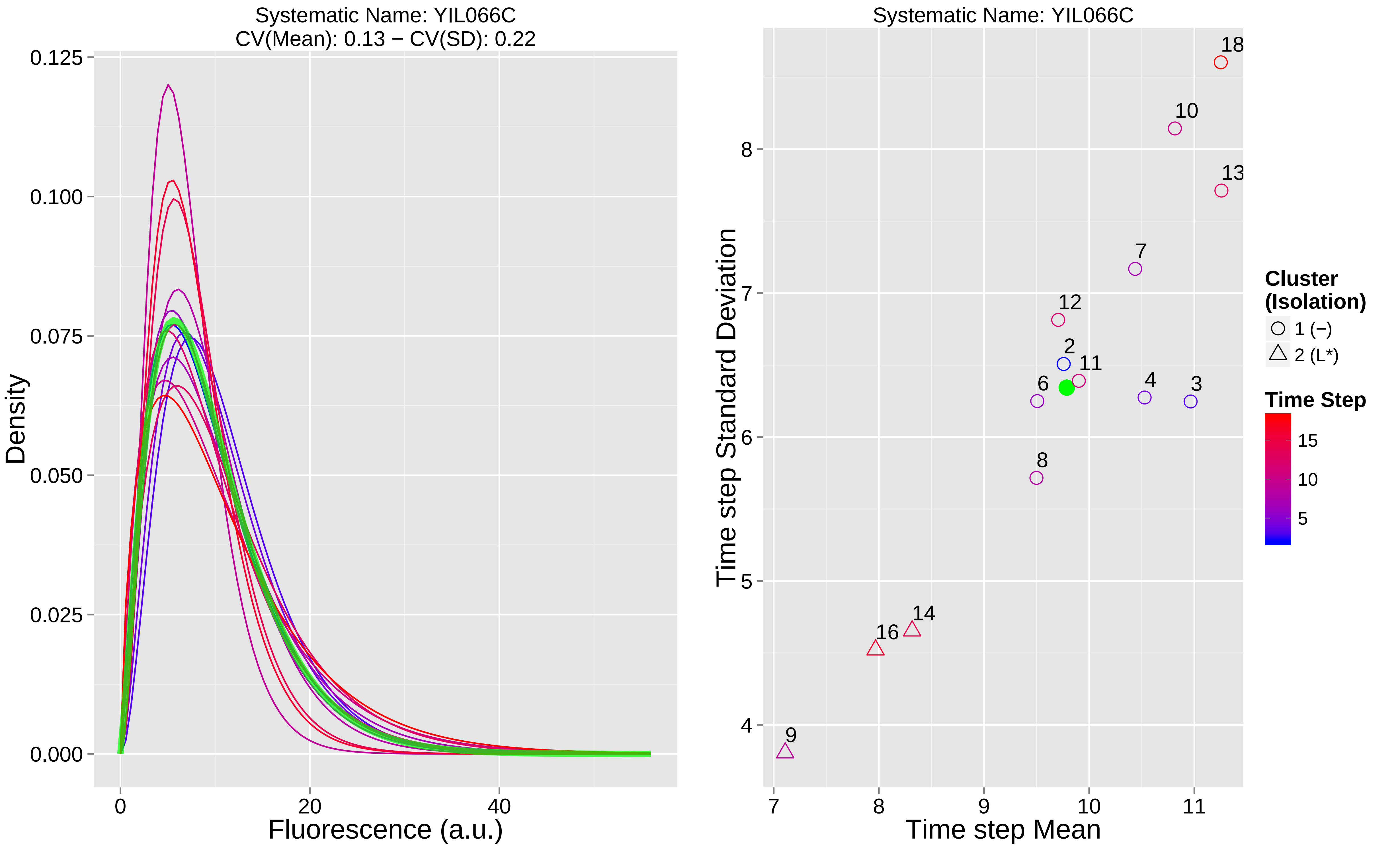

Systematic Name: YIL066C
CV(Mean): 0.13 − CV(SD): 0.22
Density
0.125
0.100
0.075
0.050
0.025
0.000
0
20
40
Fluorescence (a.u.)
Systematic Name: YIL066C
Time step Standard Deviation
Time step Mean
8
7
6
5
4
7
8
9
10
11
18
10
13
7
12
2
11
6
4
3
8
14
16
9
Cluster (Isolation)
1 (−)
2 (L*)
Time Step
15
10
5

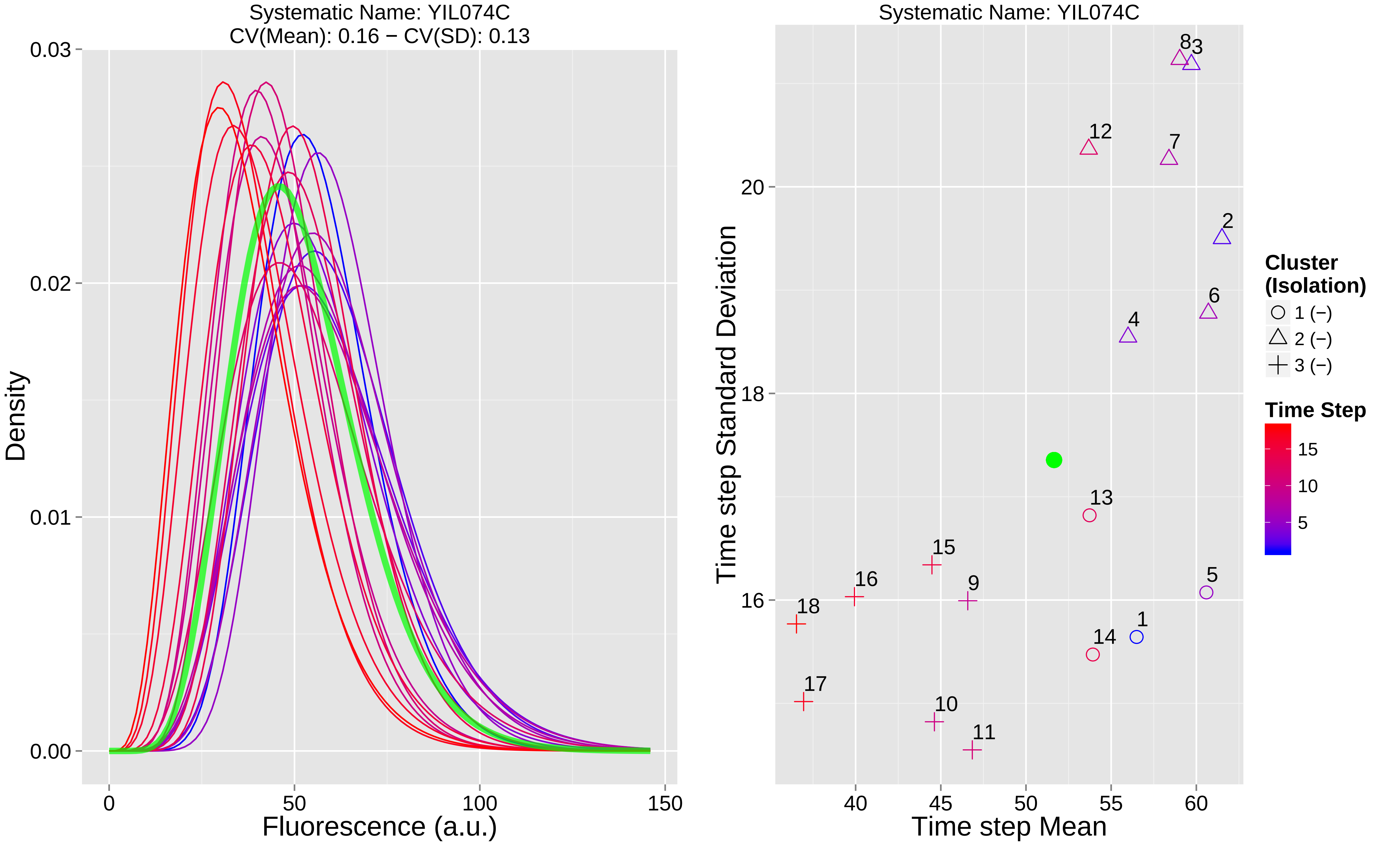
Systematic Name: YIL074C
CV(Mean): 0.16 − CV(SD): 0.13
Density
Fluorescence (a.u.)
0.03
0.02
0.01
0.00
0
50
100
150
Systematic Name: YIL074C
Time step Standard Deviation
Time step Mean
20
18
16
40
45
50
55
60
Cluster (Isolation)
1 (−)
2 (−)
3 (−)
Time Step
15
10
5
1
2
3
4
5
6
7
8
9
10
11
12
13
14
15
16
17
18

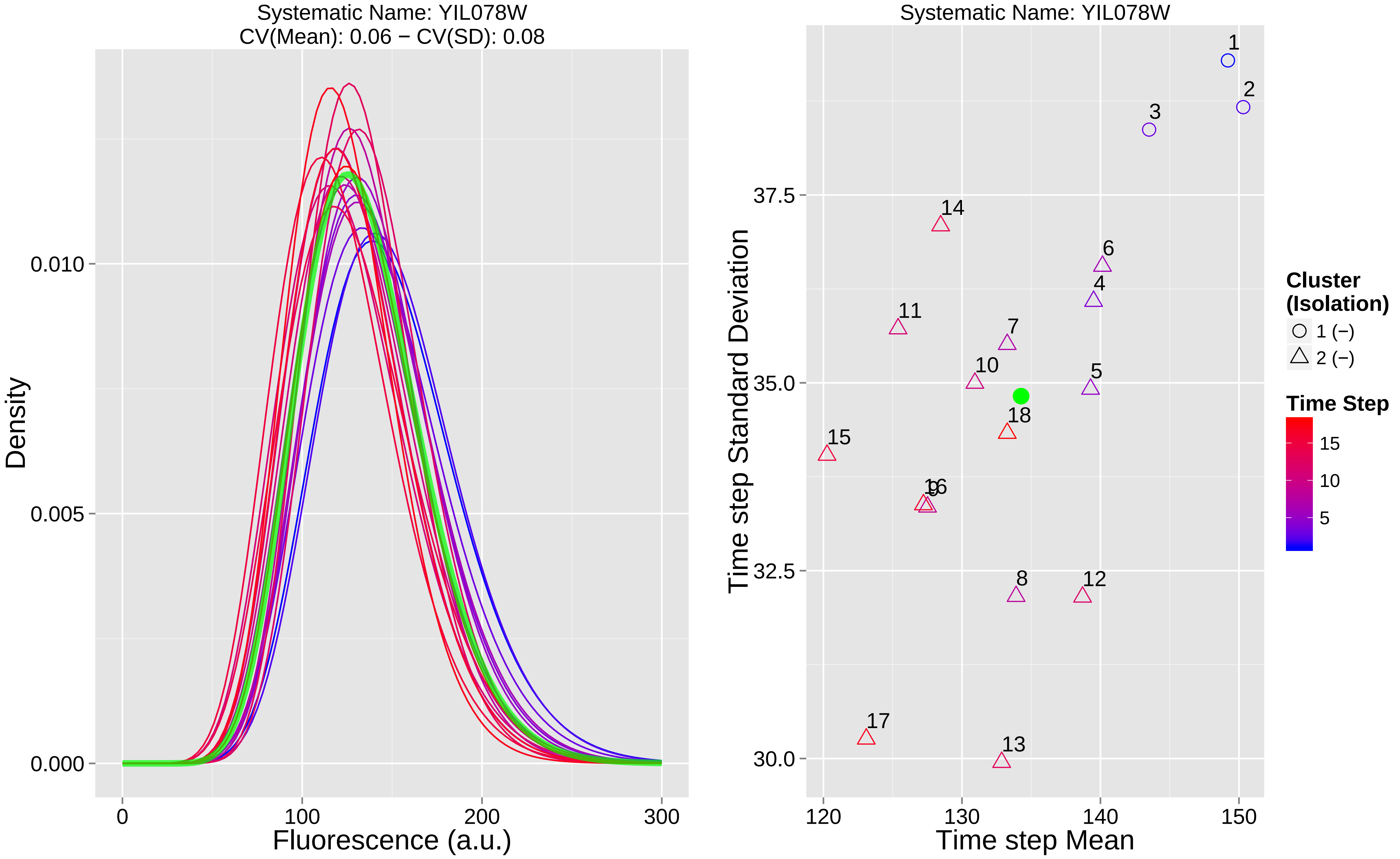

Systematic Name: YIL078W
CV(Mean): 0.06 − CV(SD): 0.08
Density
0.000
0.005
0.010
0
100
200
300
Fluorescence (a.u.)
Systematic Name: YIL078W
Time step Standard Deviation
30.0
32.5
35.0
37.5
120
130
140
150
Time step Mean
1
2
3
4
5
6
7
8
10
11
12
13
14
15
17
18
Cluster
(Isolation)
1 (−)
2 (−)
Time Step
5
10
15

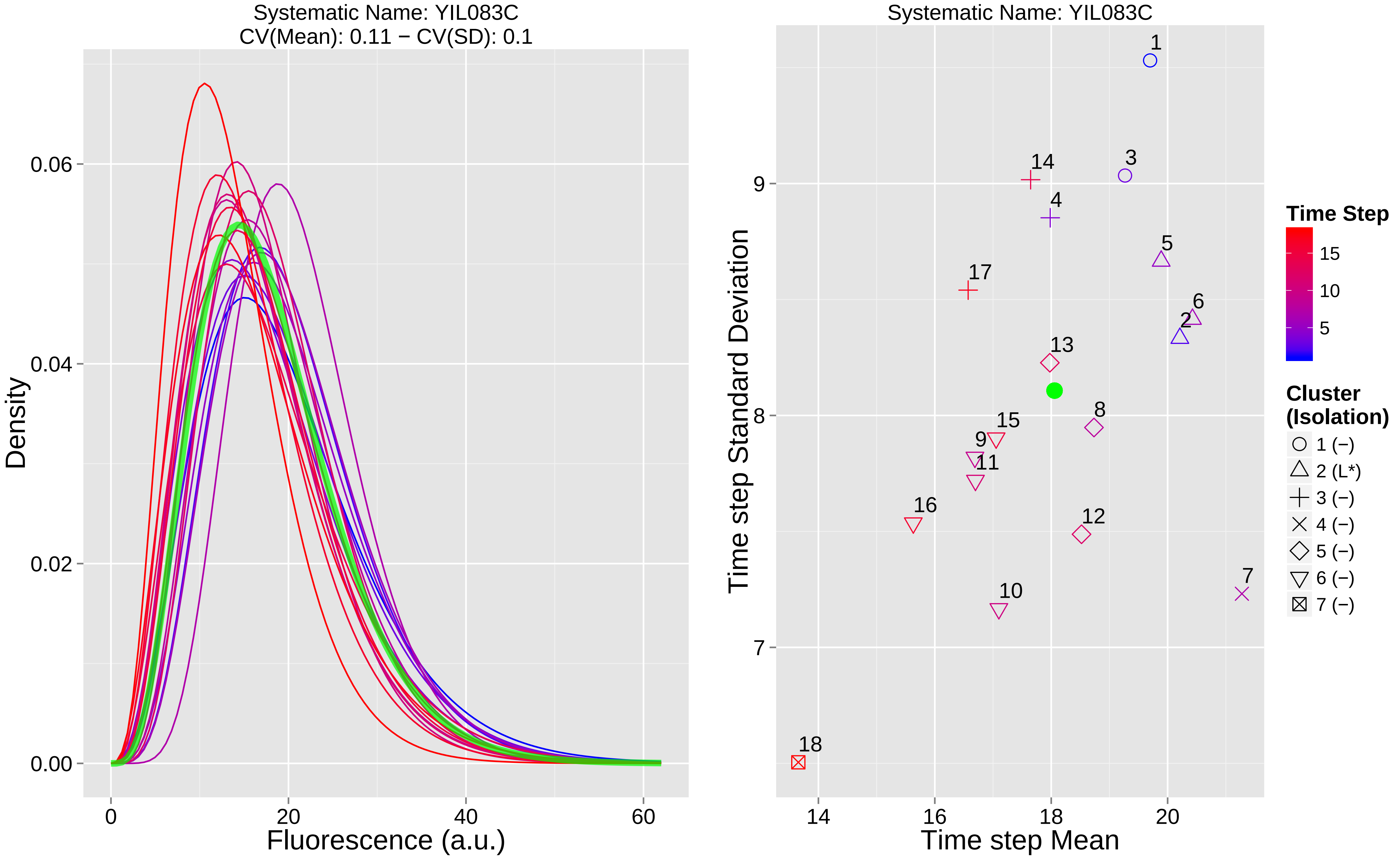
Systematic Name: YIL083C
CV(Mean): 0.11 − CV(SD): 0.1
Density
0.00
0.02
0.04
0.06
0
20
40
60
Fluorescence (a.u.)
Systematic Name: YIL083C
Time step Standard Deviation
Time step Mean
7
8
9
14
16
18
20
1
2
3
4
5
6
7
8
9
10
11
12
13
14
15
16
17
18
Time Step
15
10
5
Cluster (Isolation)
1 (−)
2 (L*)
3 (−)
4 (−)
5 (−)
6 (−)
7 (−)

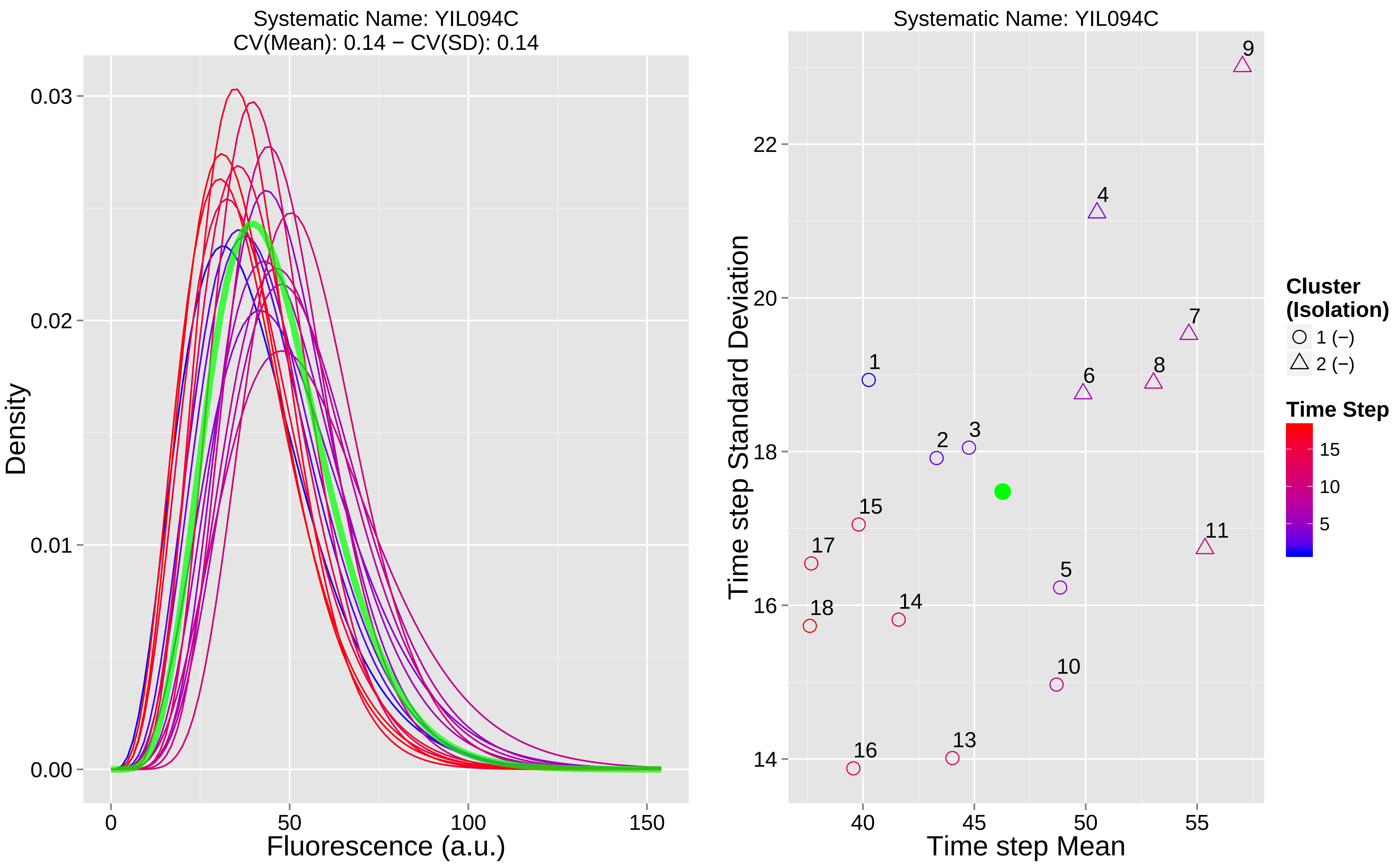

Systematic Name: YIL094C
CV(Mean): 0.14 − CV(SD): 0.14
Density
0.03
0.02
0.01
0.00
0
50
100
150
Fluorescence (a.u.)
Systematic Name: YIL094C
Time step Standard Deviation
22
20
18
16
14
40
45
50
55
Time step Mean
1
2
3
4
5
6
7
8
9
10
11
13
14
15
16
17
18
Cluster (Isolation)
1 (−)
2 (−)
Time Step
15
10
5

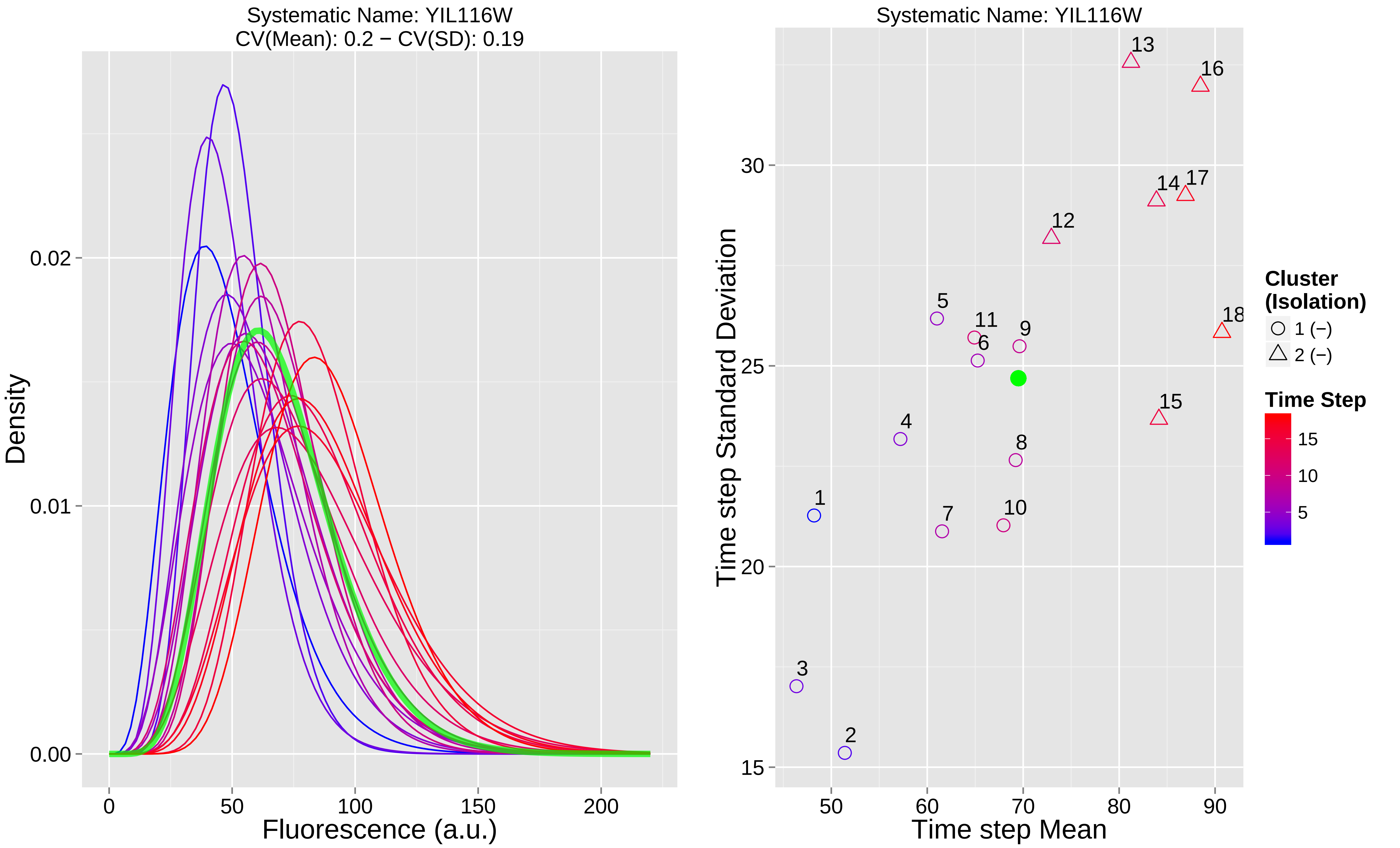
Systematic Name: YIL116W
CV(Mean): 0.2 − CV(SD): 0.19
Density
0.00
0.01
0.02
0
50
100
150
200
Fluorescence (a.u.)
Systematic Name: YIL116W
Time step Standard Deviation
15
20
25
30
50
60
70
80
90
Time step Mean
1
2
3
4
5
6
7
8
9
10
11
12
13
14
15
16
17
18
Cluster
(Isolation)
1 (−)
2 (−)
Time Step
5
10
15

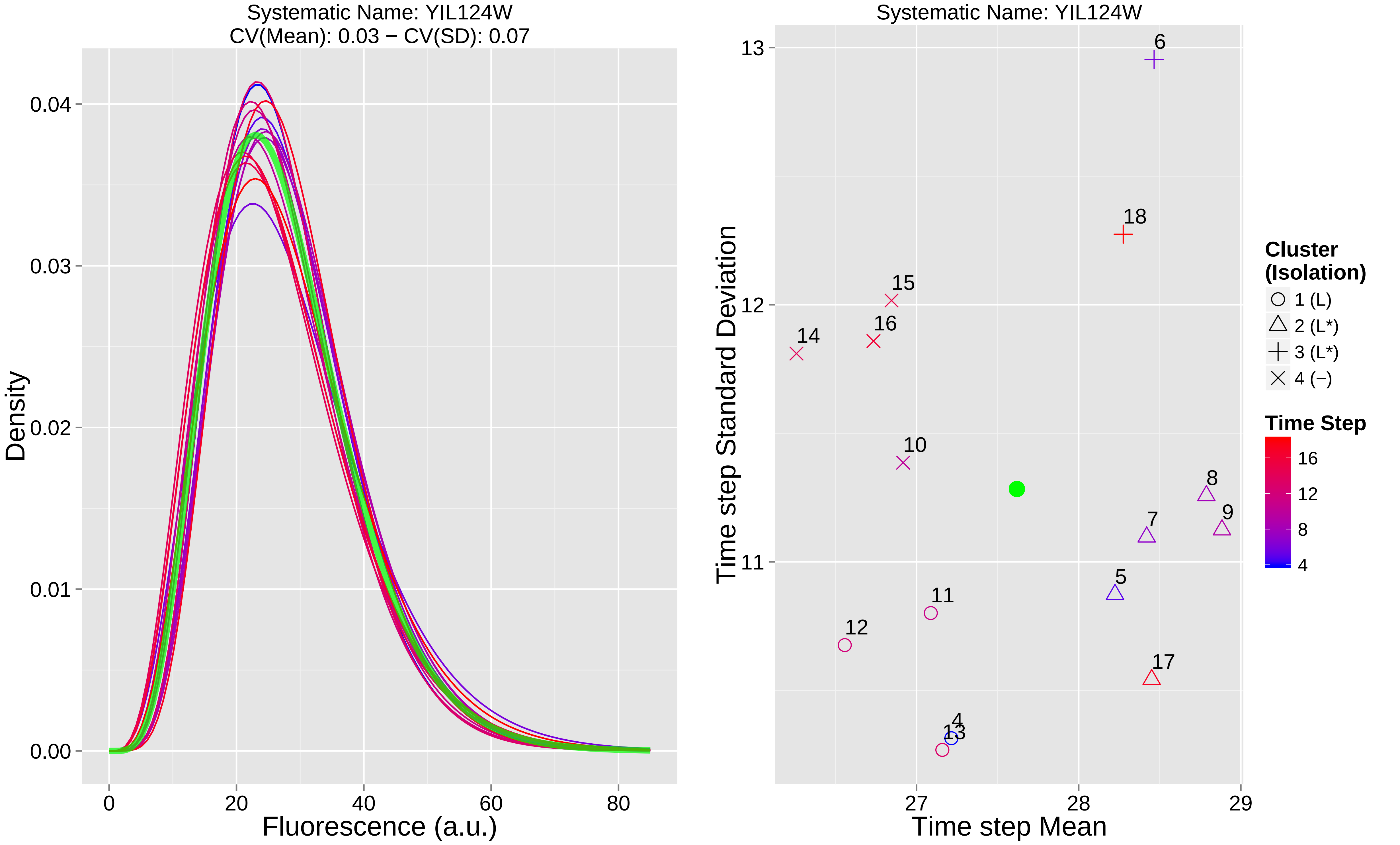
Systematic Name: YIL124W
CV(Mean): 0.03 − CV(SD): 0.07
Density
Fluorescence (a.u.)
0.00
0.01
0.02
0.03
0.04
0
20
40
60
80
Systematic Name: YIL124W
Time step Standard Deviation
Time step Mean
11
12
13
27
28
29
Cluster (Isolation)
1 (L)
2 (L*)
3 (L*)
4 (−)
Time Step
4
8
12
16

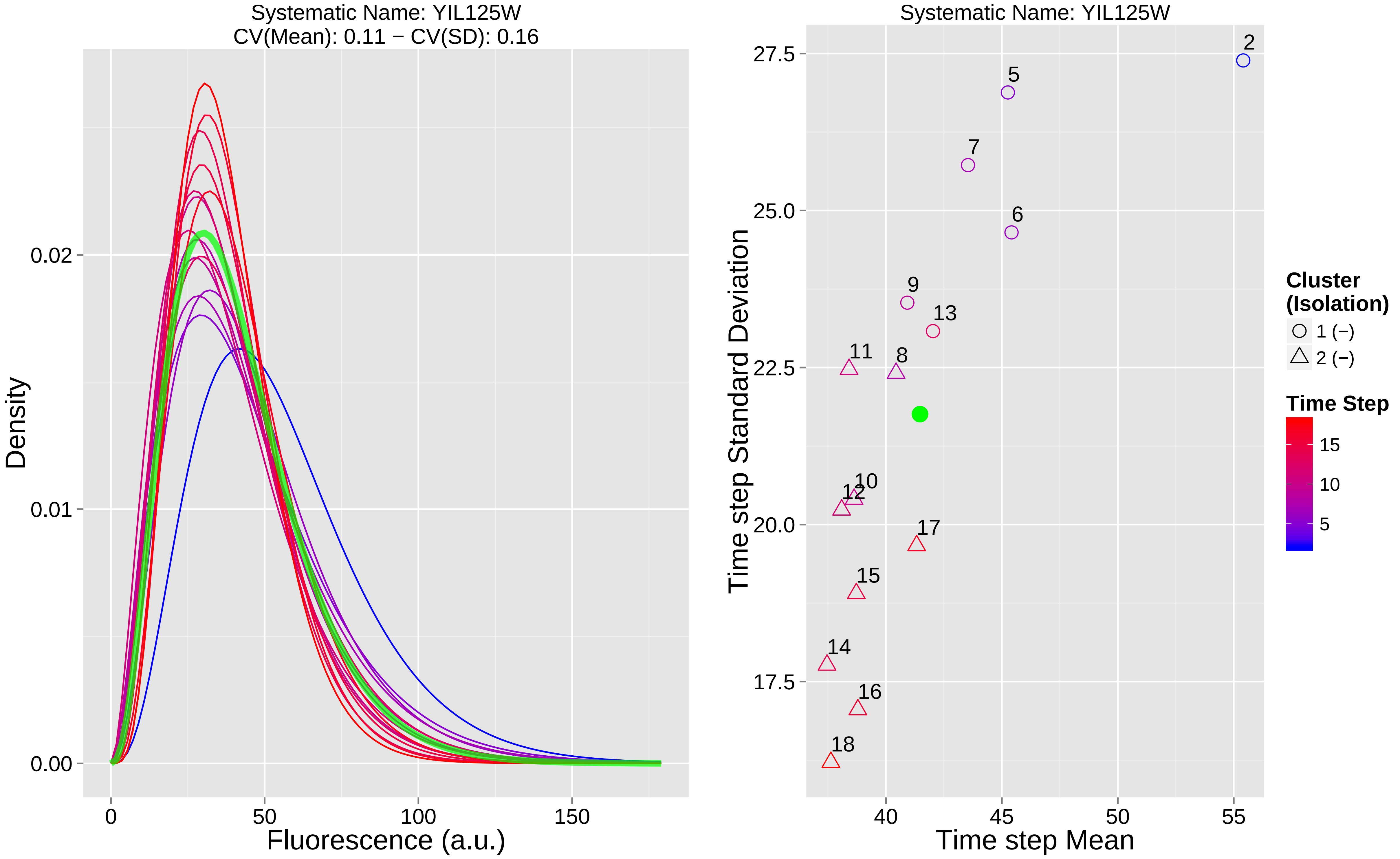

Systematic Name: YIL125W
CV(Mean): 0.11 − CV(SD): 0.16
Density
0.00
0.01
0.02
0
50
100
150
Fluorescence (a.u.)
Systematic Name: YIL125W
Time step Standard Deviation
17.5
20.0
22.5
25.0
27.5
40
45
50
55
Time step Mean
2
5
7
6
9
13
11
8
10
12
17
15
14
16
18
Cluster (Isolation)
1 (−)
2 (−)
Time Step
15
10
5

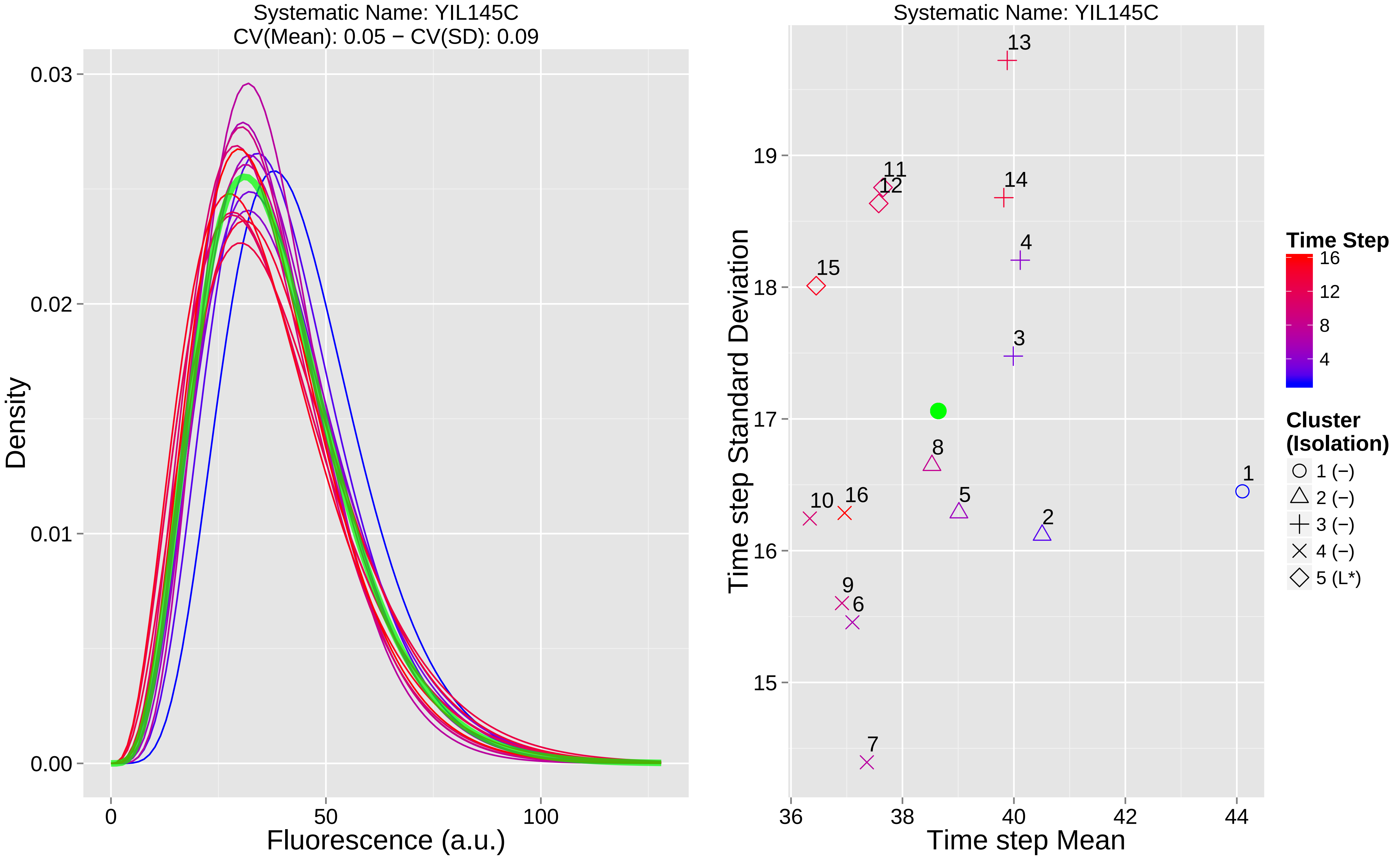

Systematic Name: YIL145C
CV(Mean): 0.05 − CV(SD): 0.09
Density
Fluorescence (a.u.)
Systematic Name: YIL145C
Time step Standard Deviation
Time step Mean
Time Step
Cluster (Isolation)
1 (−)
2 (−)
3 (−)
4 (−)
5 (L*)

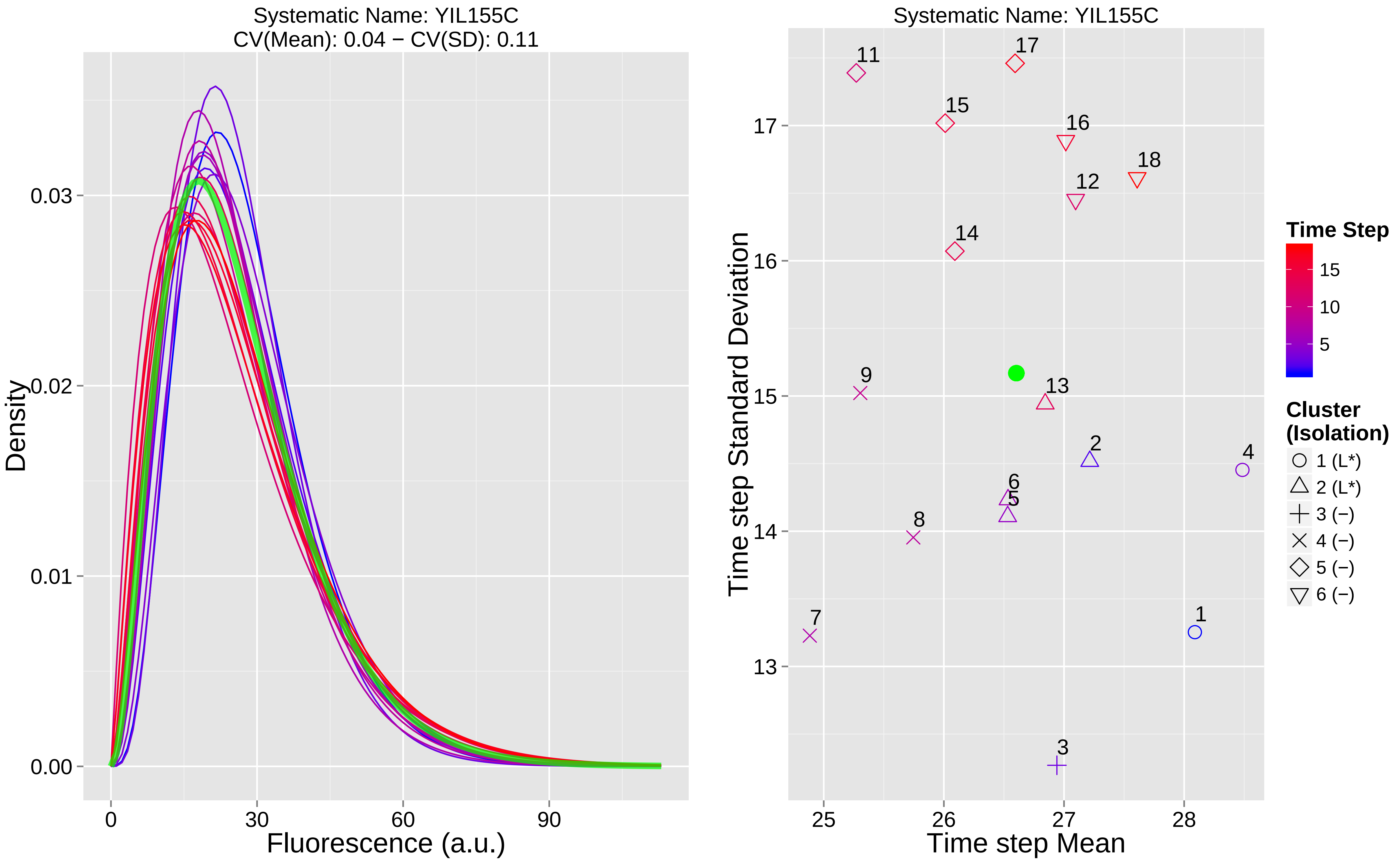
Systematic Name: YIL155C
CV(Mean): 0.04 − CV(SD): 0.11
Density
0.00
0.01
0.02
0.03
0
30
60
90
Fluorescence (a.u.)
Systematic Name: YIL155C
Time step Standard Deviation
13
14
15
16
17
25
26
27
28
Time step Mean
1
2
3
4
5
6
7
8
9
11
12
13
14
15
16
17
18
Time Step
15
10
5
Cluster
(Isolation)
1 (L*)
2 (L*)
3 (−)
4 (−)
5 (−)
6 (−)

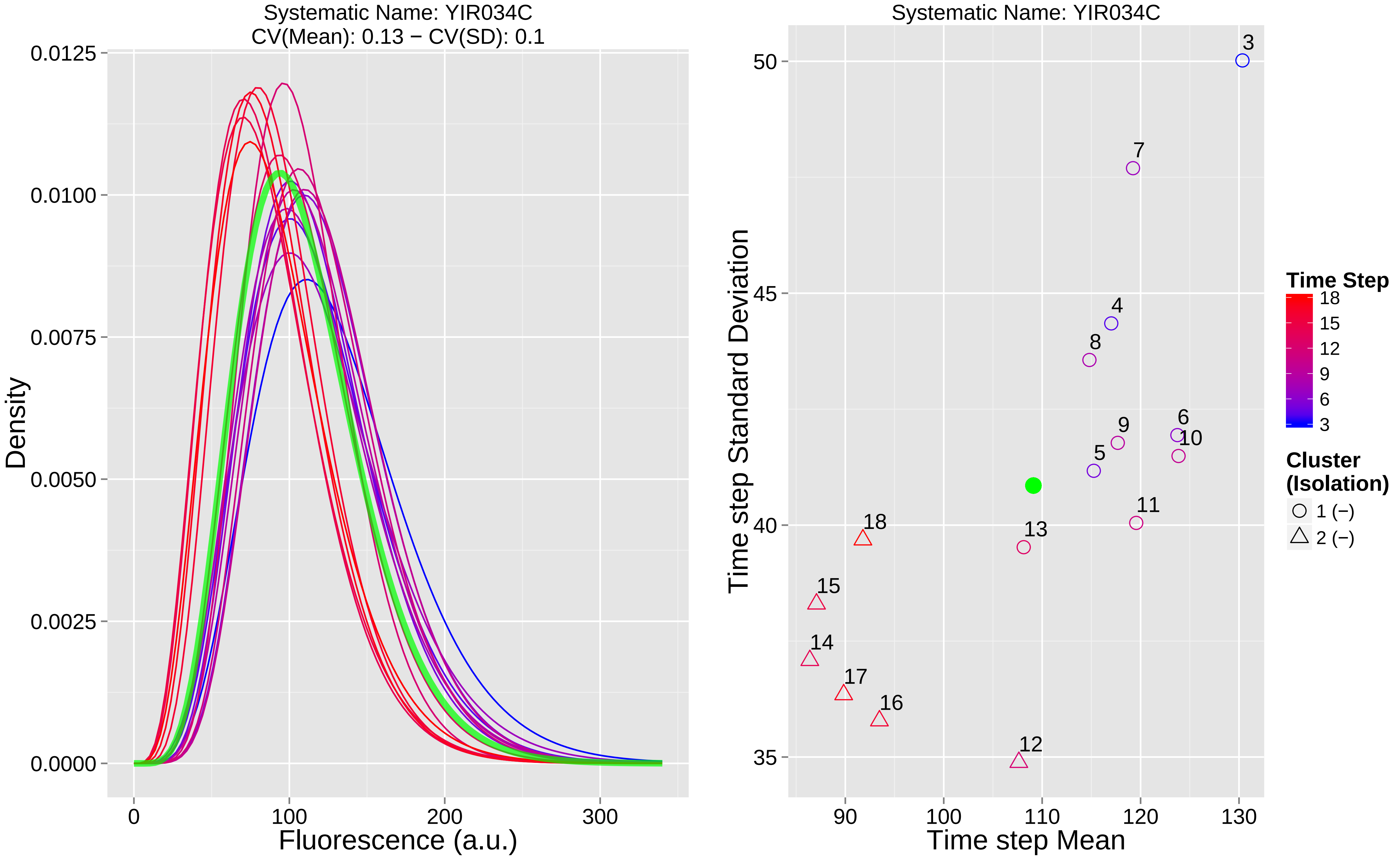
Systematic Name: YIR034C
CV(Mean): 0.13 − CV(SD): 0.1
Density
Fluorescence (a.u.)
0.0000
0.0025
0.0050
0.0075
0.0100
0.0125
0
100
200
300
Systematic Name: YIR034C
Time step Standard Deviation
Time step Mean
35
40
45
50
90
100
110
120
130
3
4
5
6
7
8
9
10
11
12
13
14
15
16
17
18
Time Step
18
15
12
9
6
3
Cluster
(Isolation)
1 (−)
2 (−)

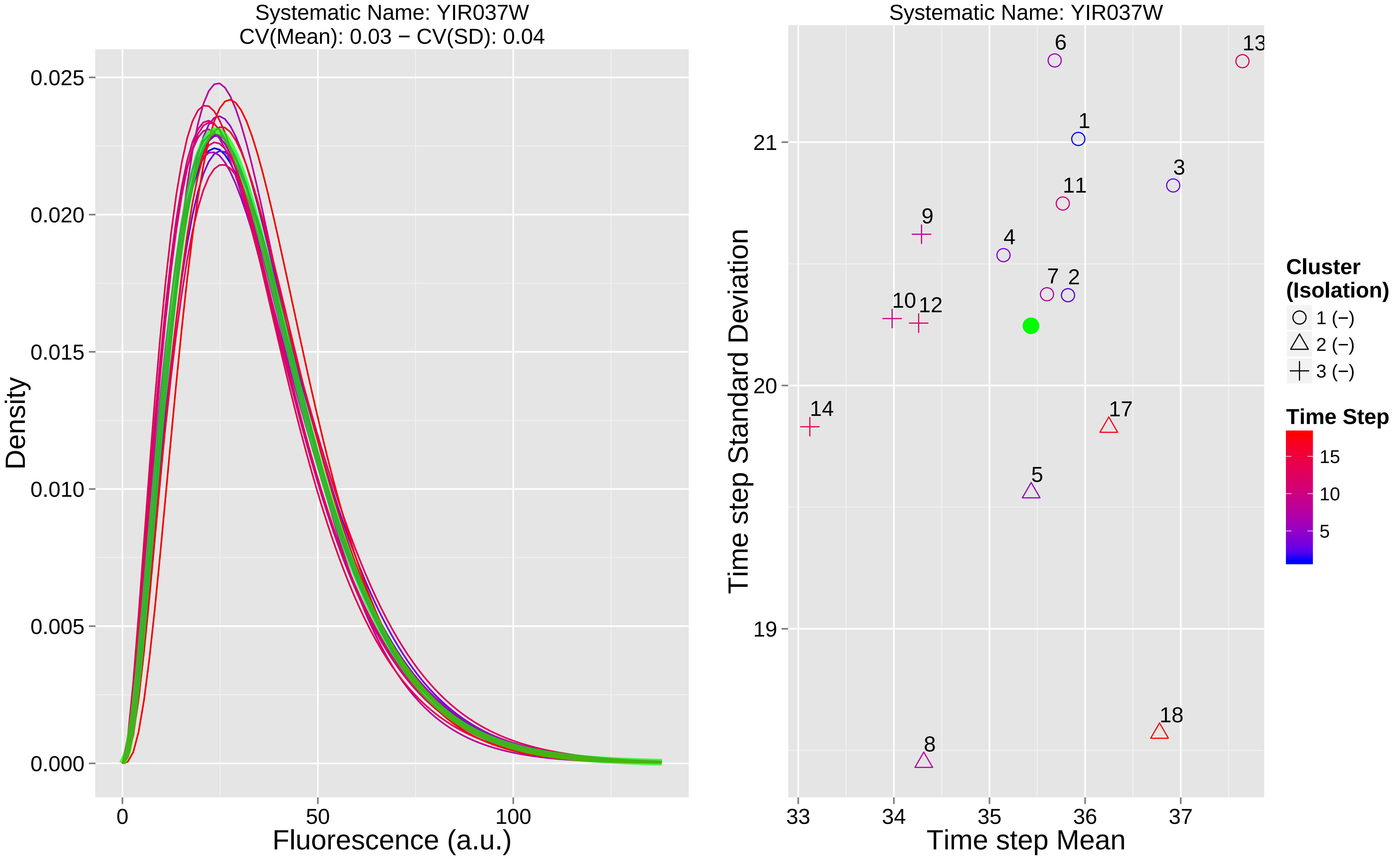

Systematic Name: YIR037W
CV(Mean): 0.03 − CV(SD): 0.04
Density
0.025
0.020
0.015
0.010
0.005
0.000
0
50
100
Fluorescence (a.u.)
Systematic Name: YIR037W
Time step Standard Deviation
21
20
19
33
34
35
36
37
Time step Mean
6
13
1
3
11
9
4
7
2
10
12
14
17
5
18
8
Cluster (Isolation)
1 (−)
2 (−)
3 (−)
Time Step
15
10
5

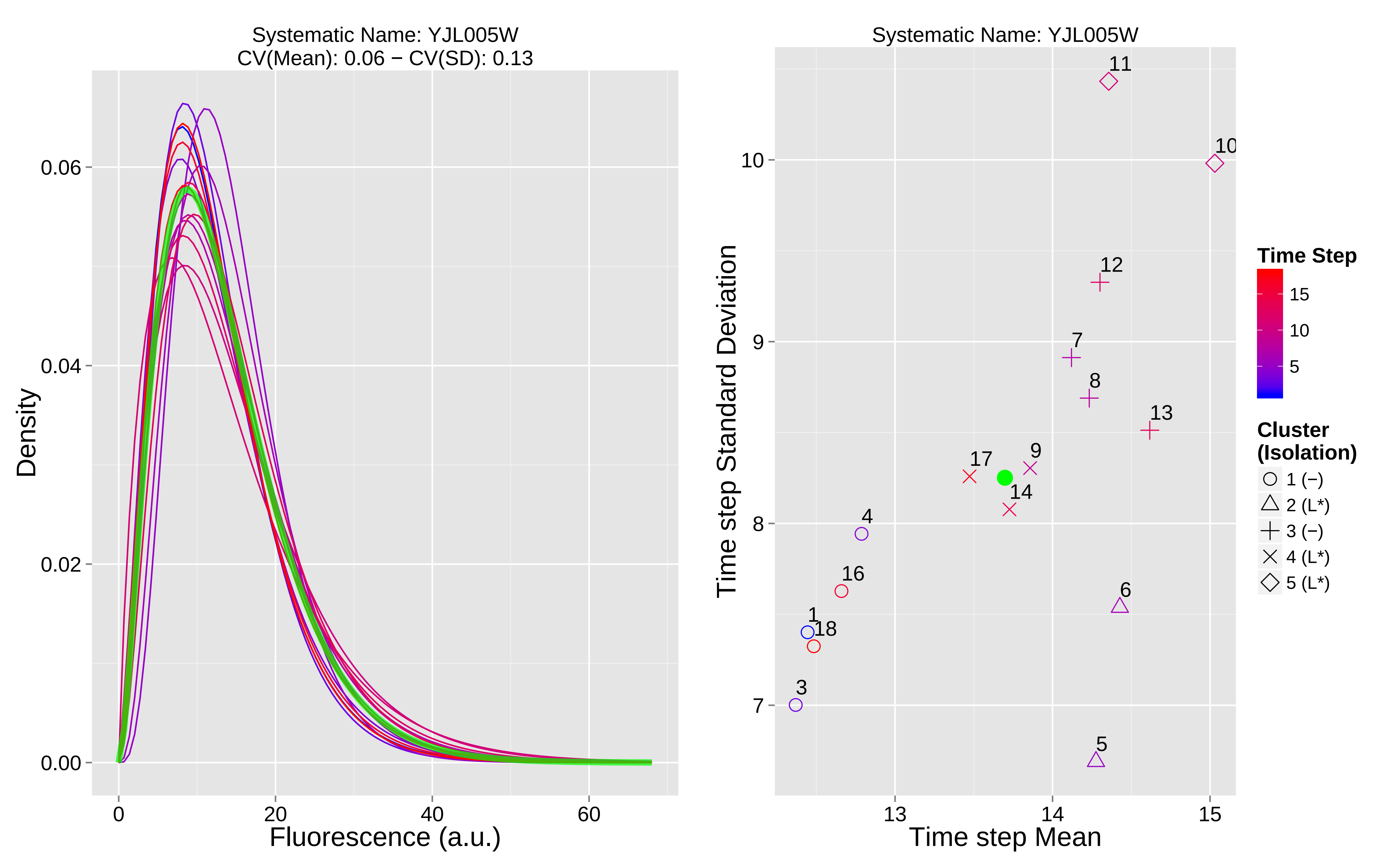

Systematic Name: YJL005W
CV(Mean): 0.06 − CV(SD): 0.13
Density
0.00
0.02
0.04
0.06
0
20
40
60
Fluorescence (a.u.)
Systematic Name: YJL005W
Time step Standard Deviation
Time step Mean
7
8
9
10
13
14
15
1
2
3
4
5
6
7
8
9
10
11
12
13
14
16
17
18
Time Step
15
10
5
Cluster (Isolation)
1 (−)
2 (L*)
3 (−)
4 (L*)
5 (L*)

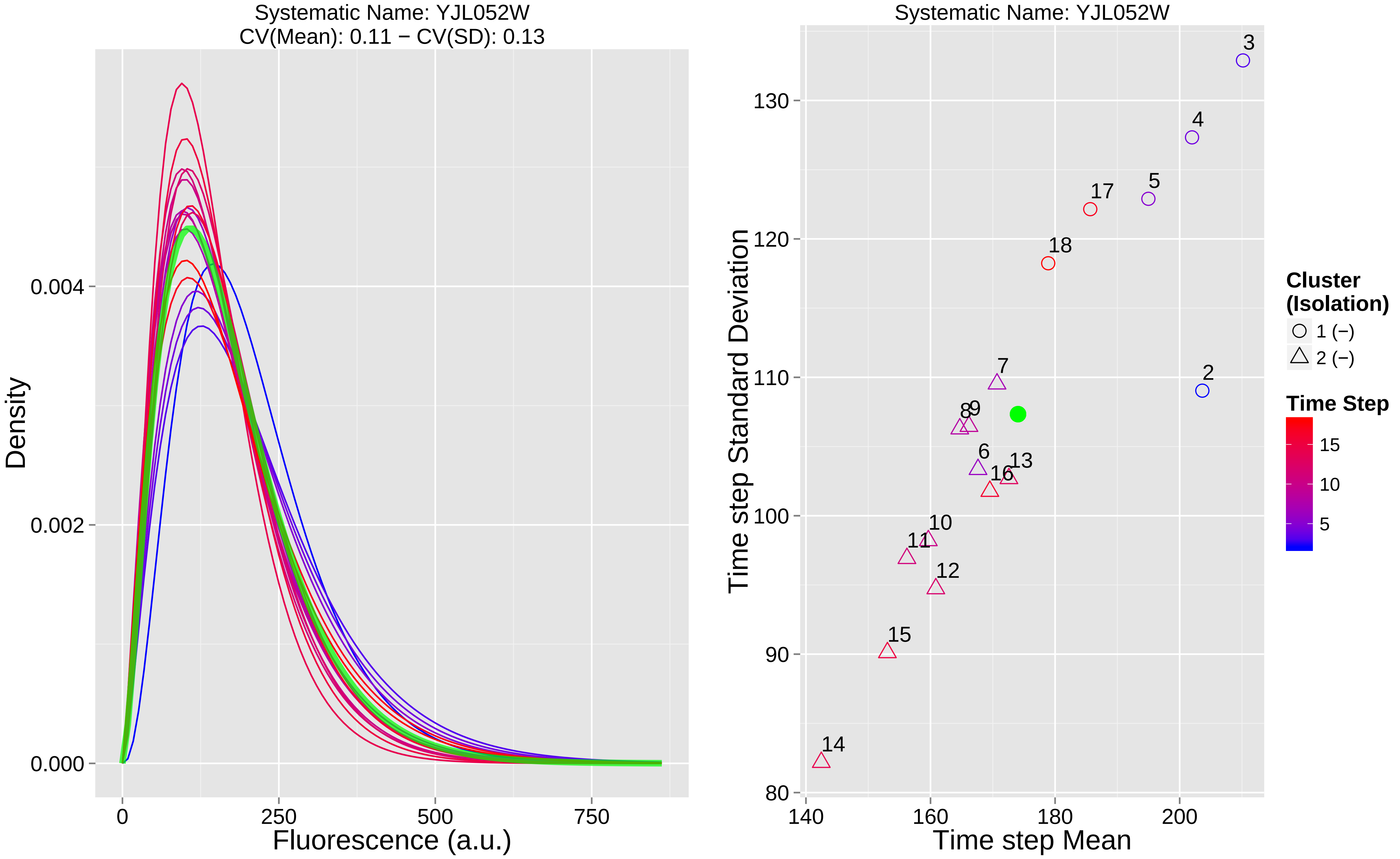

Systematic Name: YJL052W
CV(Mean): 0.11 − CV(SD): 0.13
Density
0.000
0.002
0.004
0
250
500
750
Fluorescence (a.u.)
Systematic Name: YJL052W
Time step Standard Deviation
80
90
100
110
120
130
140
160
180
200
Time step Mean
Cluster (Isolation)
1 (−)
2 (−)
Time Step
5
10
15

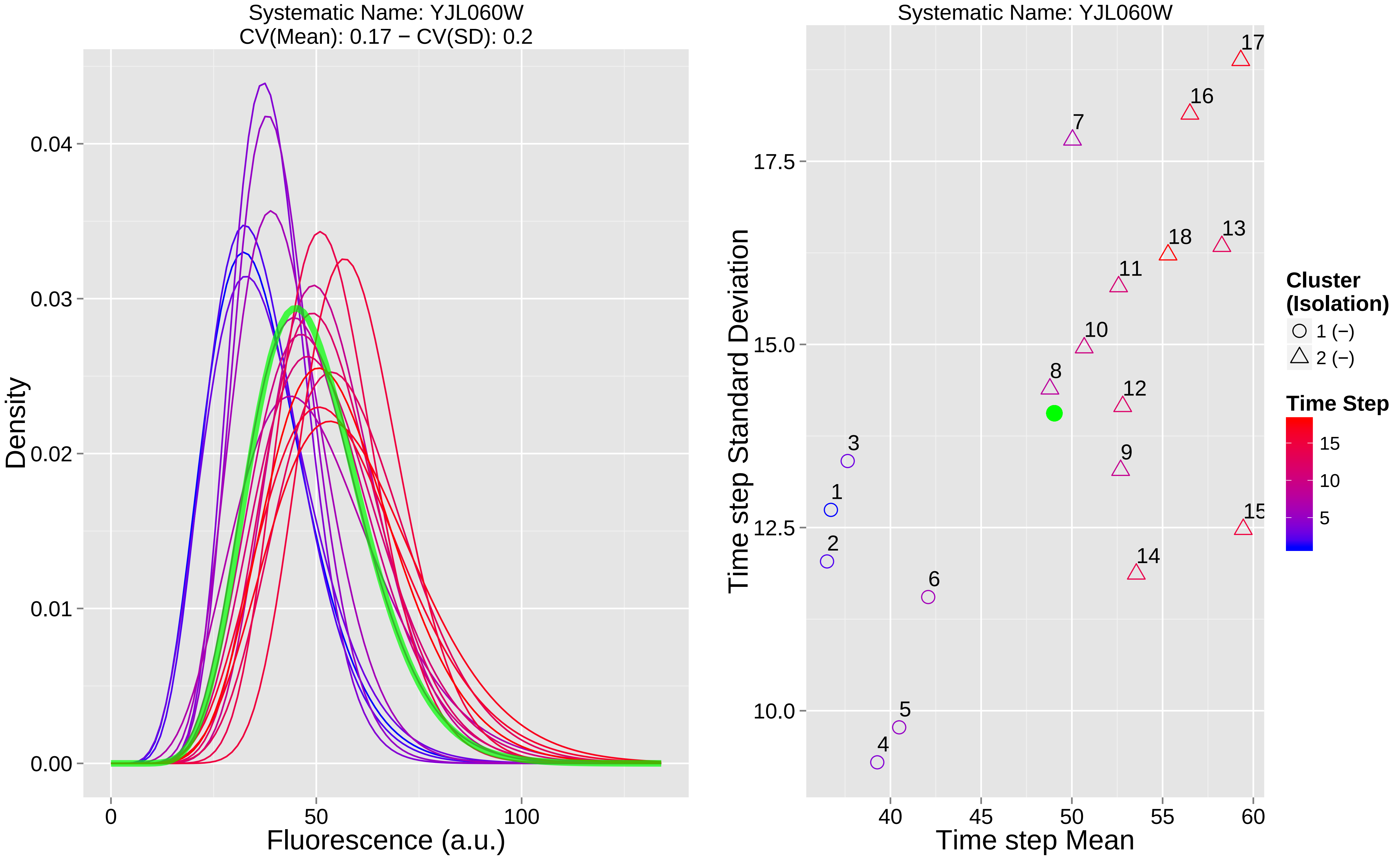
Systematic Name: YJL060W
CV(Mean): 0.17 − CV(SD): 0.2
Density
Fluorescence (a.u.)
0.00
0.01
0.02
0.03
0.04
0
50
100
Systematic Name: YJL060W
Time step Standard Deviation
Time step Mean
10.0
12.5
15.0
17.5
40
45
50
55
60
Cluster (Isolation)
1 (−)
2 (−)
Time Step
5
10
15

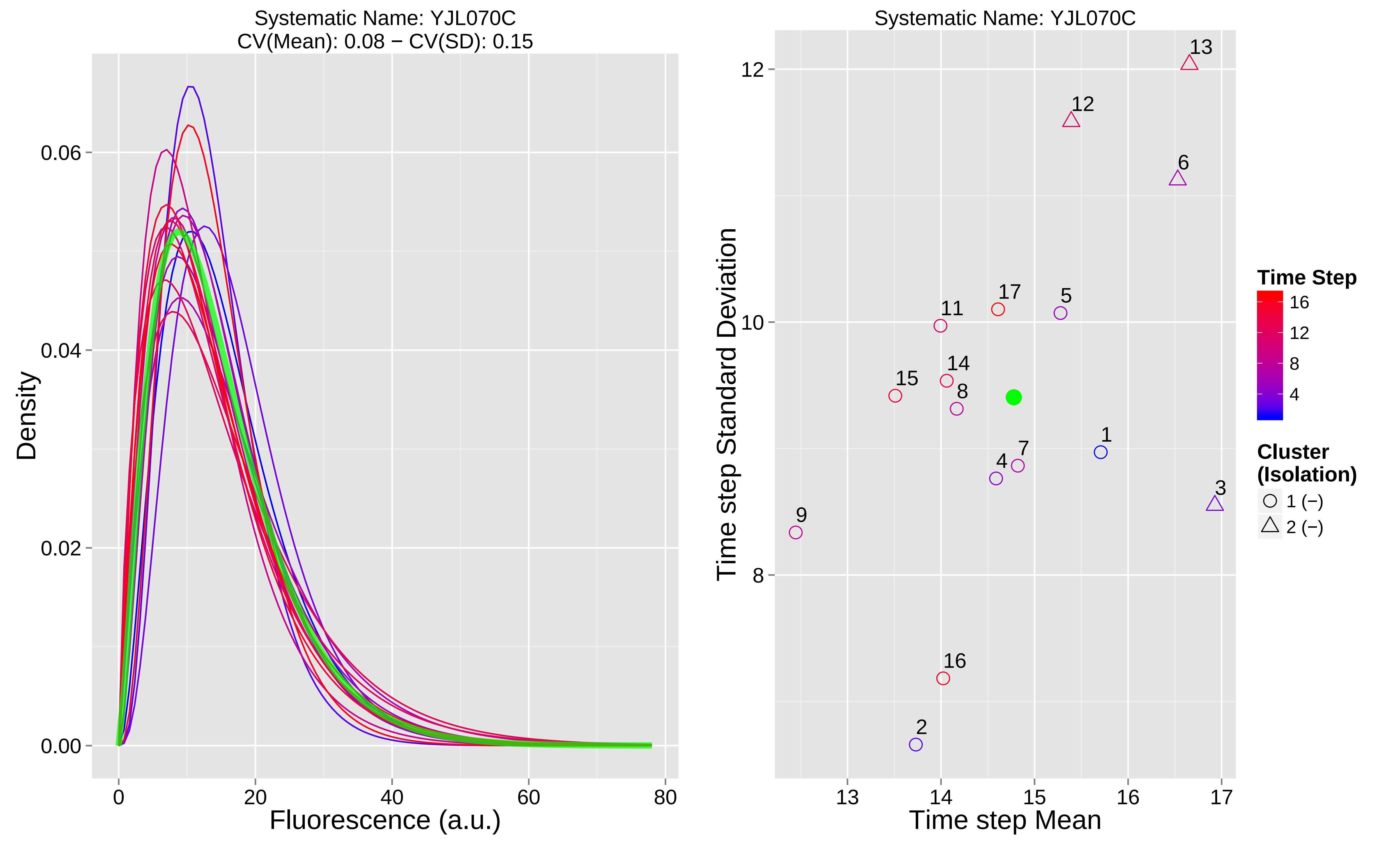

Systematic Name: YJL070C
CV(Mean): 0.08 − CV(SD): 0.15
Density
Fluorescence (a.u.)
Systematic Name: YJL070C
Time step Standard Deviation
Time step Mean
Time Step
Cluster
(Isolation)
1 (−)
2 (−)

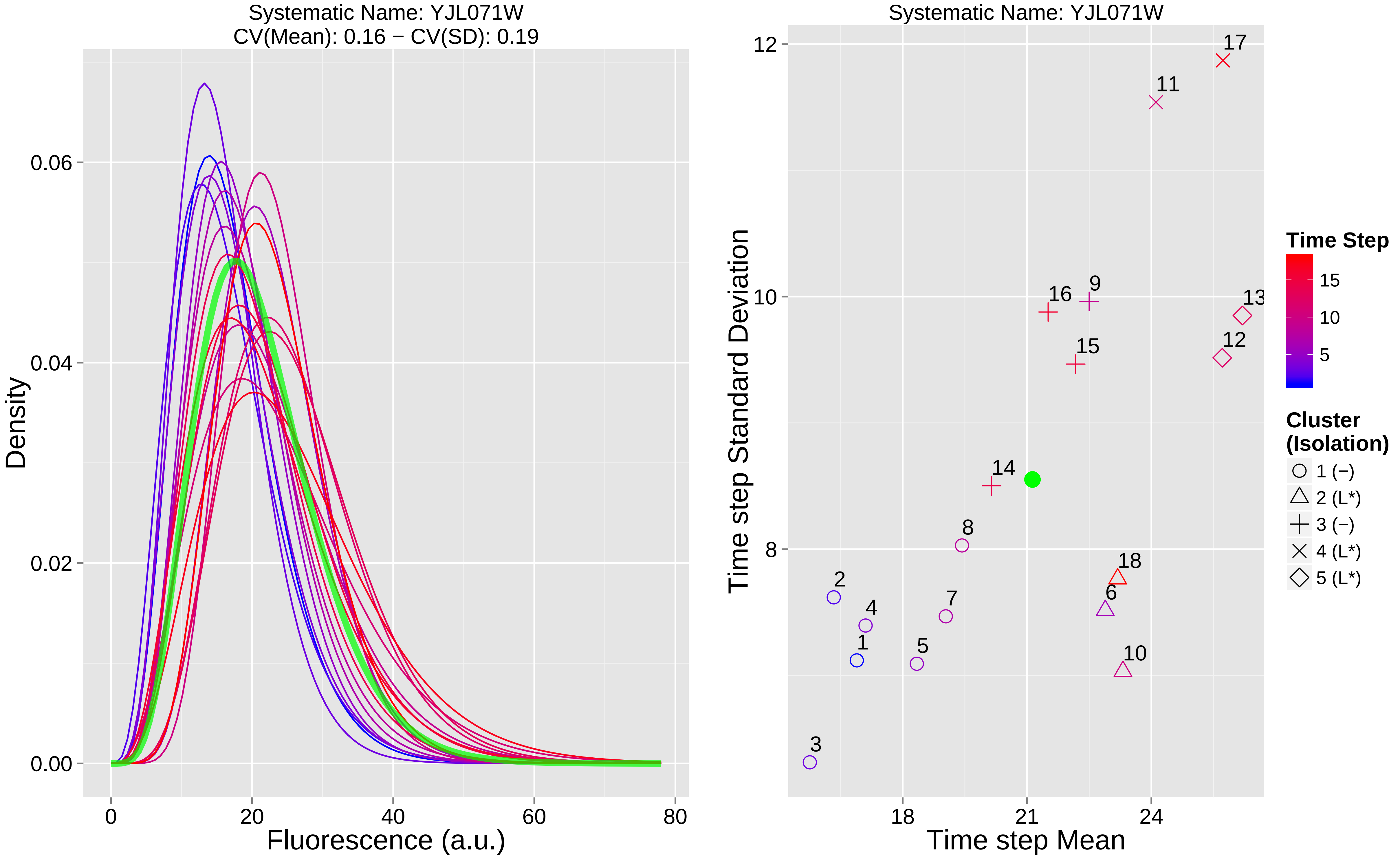
Systematic Name: YJL071W
CV(Mean): 0.16 − CV(SD): 0.19
Density
0.00
0.02
0.04
0.06
0
20
40
60
80
Fluorescence (a.u.)
Systematic Name: YJL071W
Time step Standard Deviation
12
10
8
18
21
24
Time step Mean
1
2
3
4
5
6
7
8
9
10
11
12
13
14
15
16
17
18
Time Step
15
10
5
Cluster
(Isolation)
1 (−)
2 (L*)
3 (−)
4 (L*)
5 (L*)

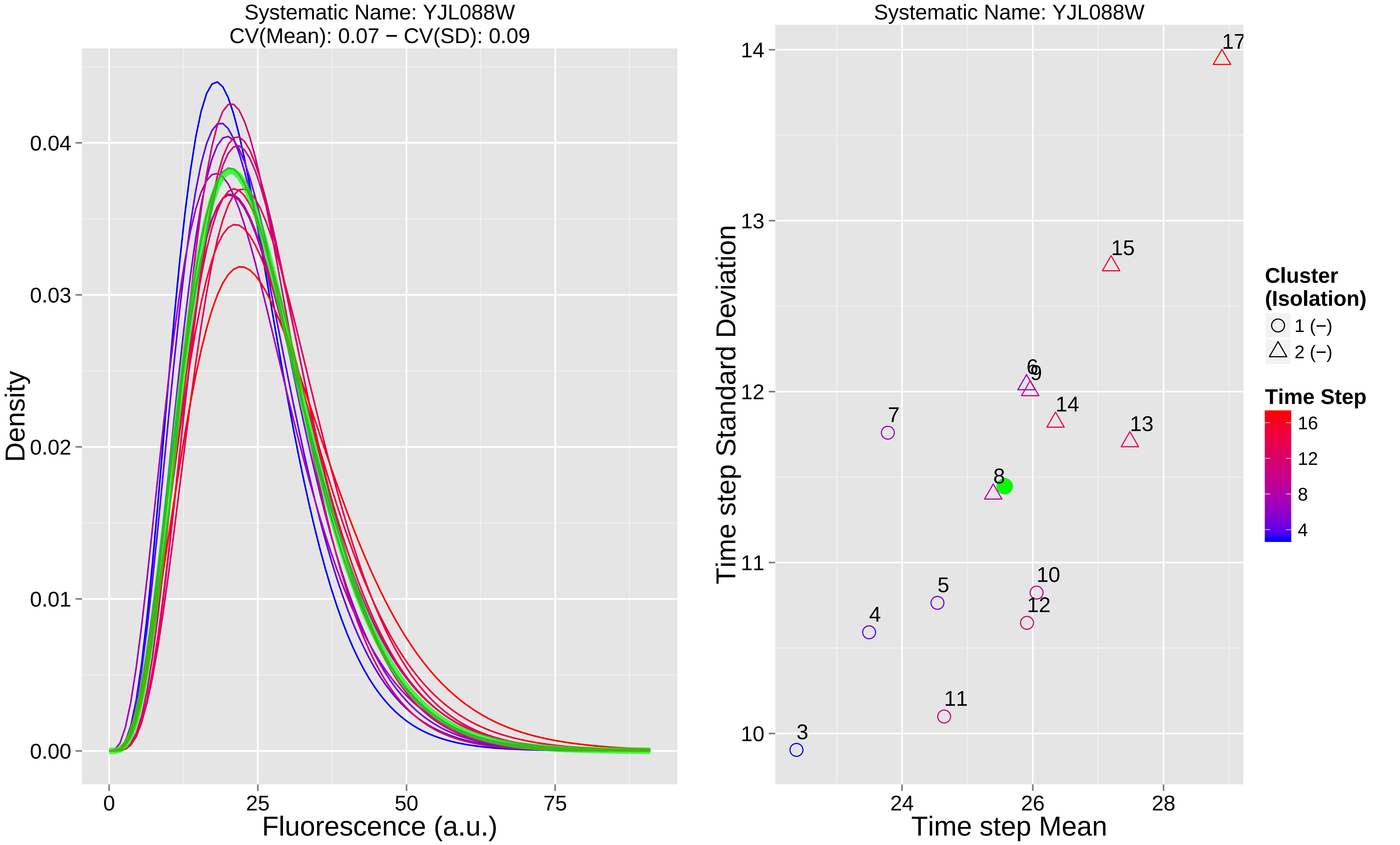
Systematic Name: YJL088W
CV(Mean): 0.07 − CV(SD): 0.09
Density
0.00
0.01
0.02
0.03
0.04
0
25
50
75
Fluorescence (a.u.)
Systematic Name: YJL088W
Time step Standard Deviation
10
11
12
13
14
24
26
28
Time step Mean
3
4
5
6
7
8
9
10
11
12
13
14
15
17
Cluster
(Isolation)
1 (−)
2 (−)
Time Step
16
12
8
4

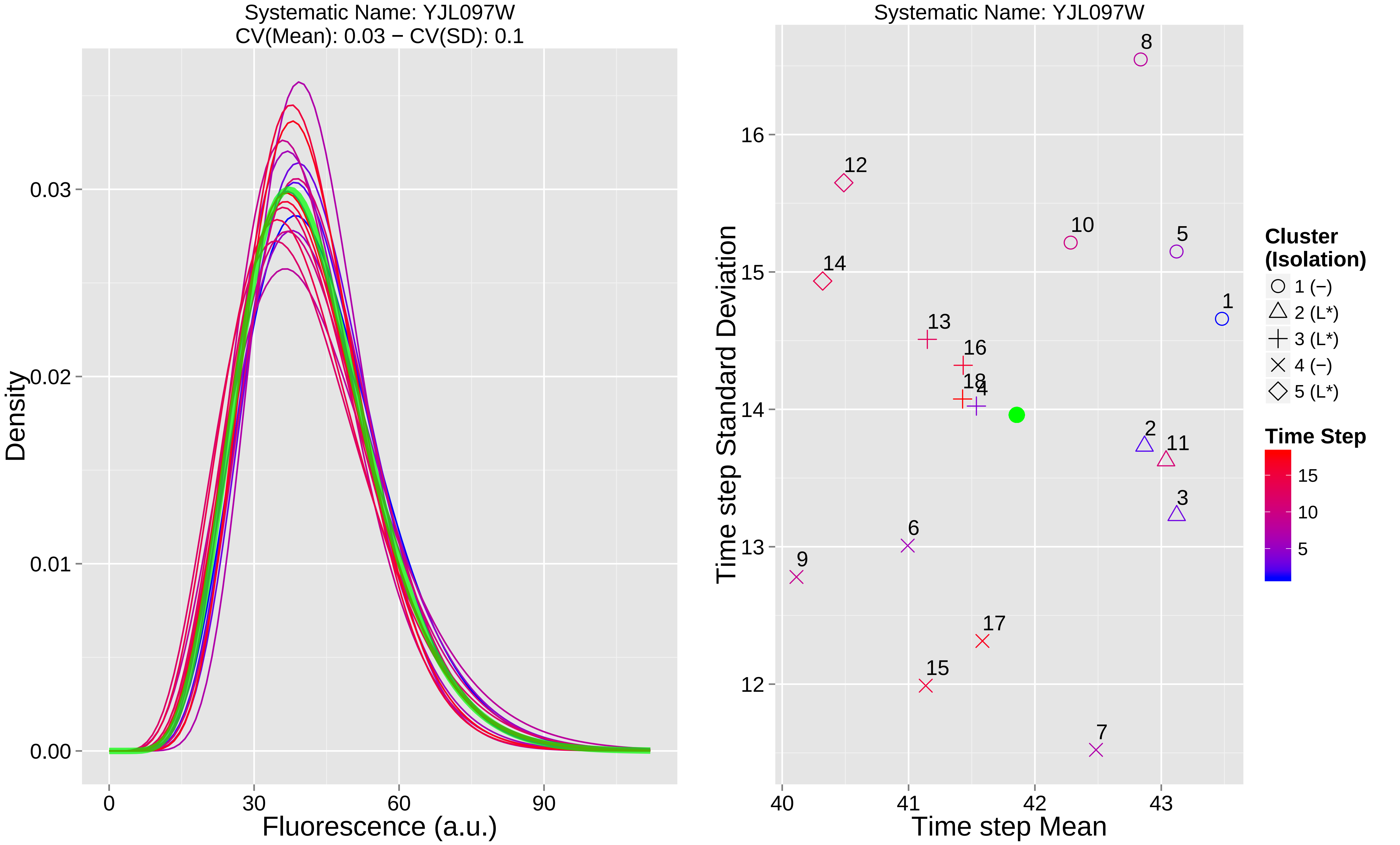
Systematic Name: YJL097W
CV(Mean): 0.03 − CV(SD): 0.1
Density
Fluorescence (a.u.)
0.00
0.01
0.02
0.03
0
30
60
90
Systematic Name: YJL097W
Time step Standard Deviation
Time step Mean
40
41
42
43
12
13
14
15
16
Cluster
(Isolation)
1 (−)
2 (L*)
3 (L*)
4 (−)
5 (L*)
Time Step
15
10
5

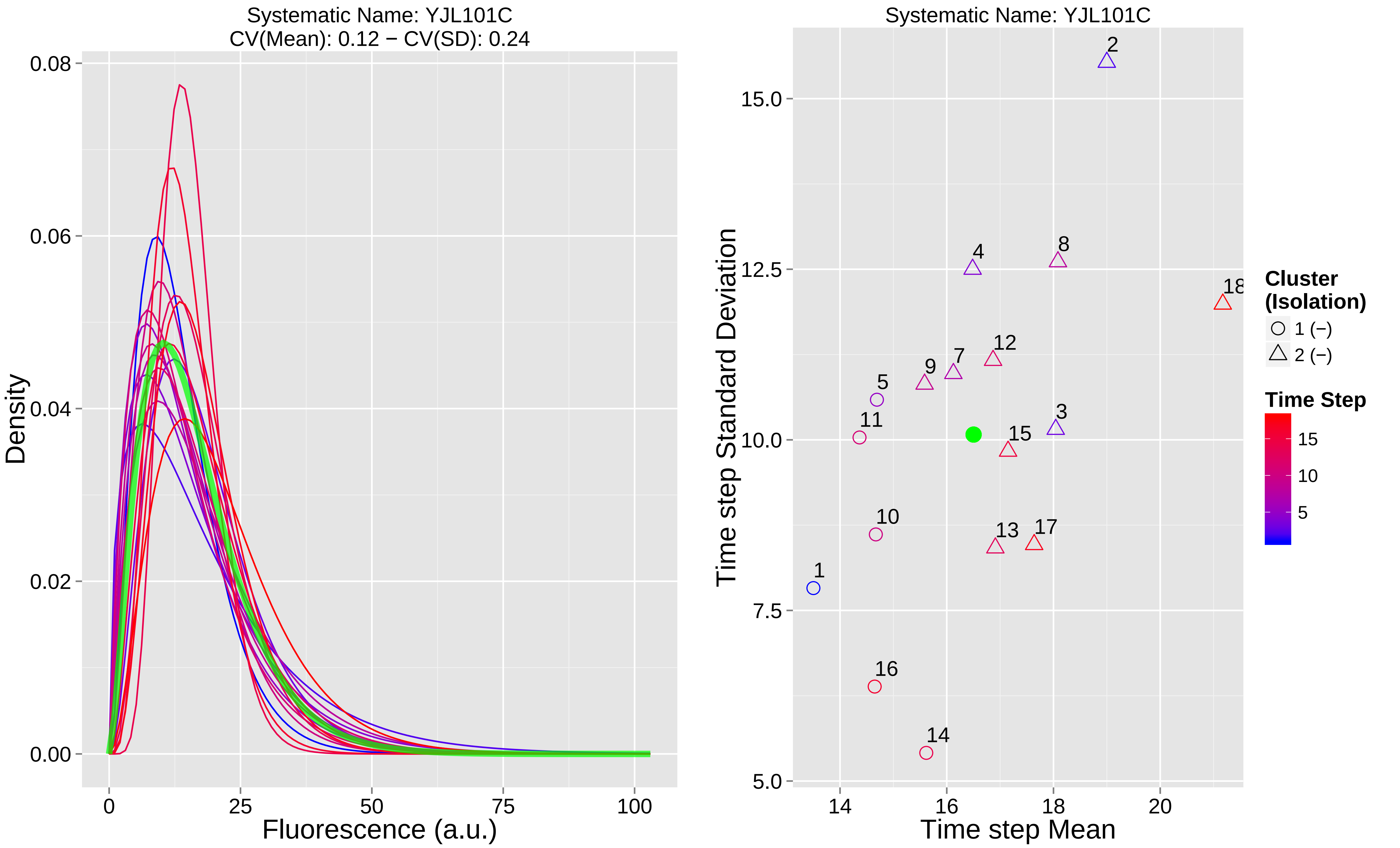

Systematic Name: YJL101C
CV(Mean): 0.12 − CV(SD): 0.24
Density
0.08
0.06
0.04
0.02
0.00
0
25
50
75
100
Fluorescence (a.u.)
Systematic Name: YJL101C
Time step Standard Deviation
15.0
12.5
10.0
7.5
5.0
14
16
18
20
Time step Mean
1
2
3
4
5
7
8
9
10
11
12
13
14
15
16
17
18
Cluster (Isolation)
1 (−)
2 (−)
Time Step
15
10
5

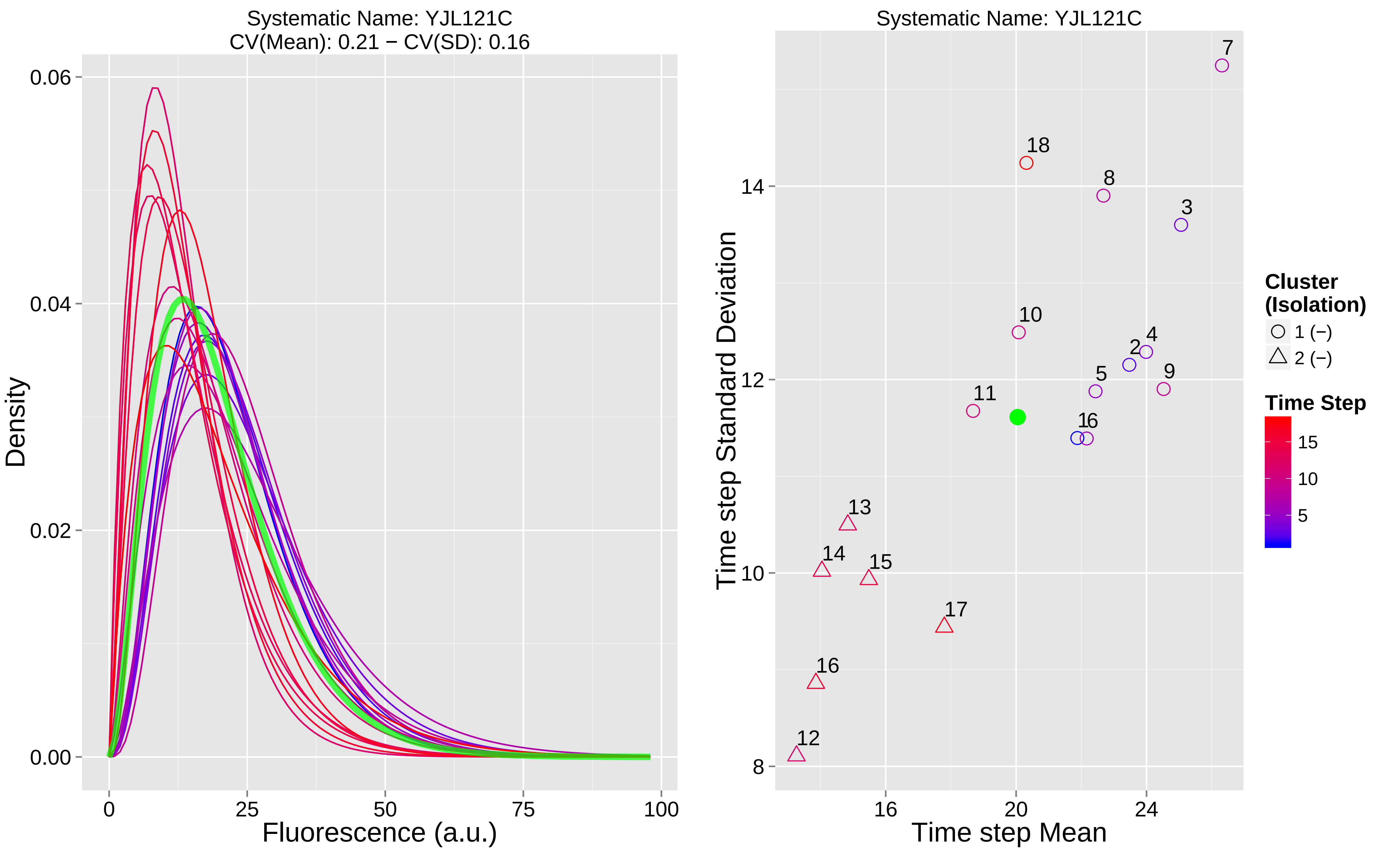
Systematic Name: YJL121C
CV(Mean): 0.21 − CV(SD): 0.16
Density
0.06
0.04
0.02
0.00
0
25
50
75
100
Fluorescence (a.u.)
Systematic Name: YJL121C
Time step Standard Deviation
14
12
10
8
16
20
24
Time step Mean
7
18
8
3
10
2
4
5
9
11
1
6
13
14
15
17
16
12
Cluster
(Isolation)
1 (−)
2 (−)
Time Step
15
10
5

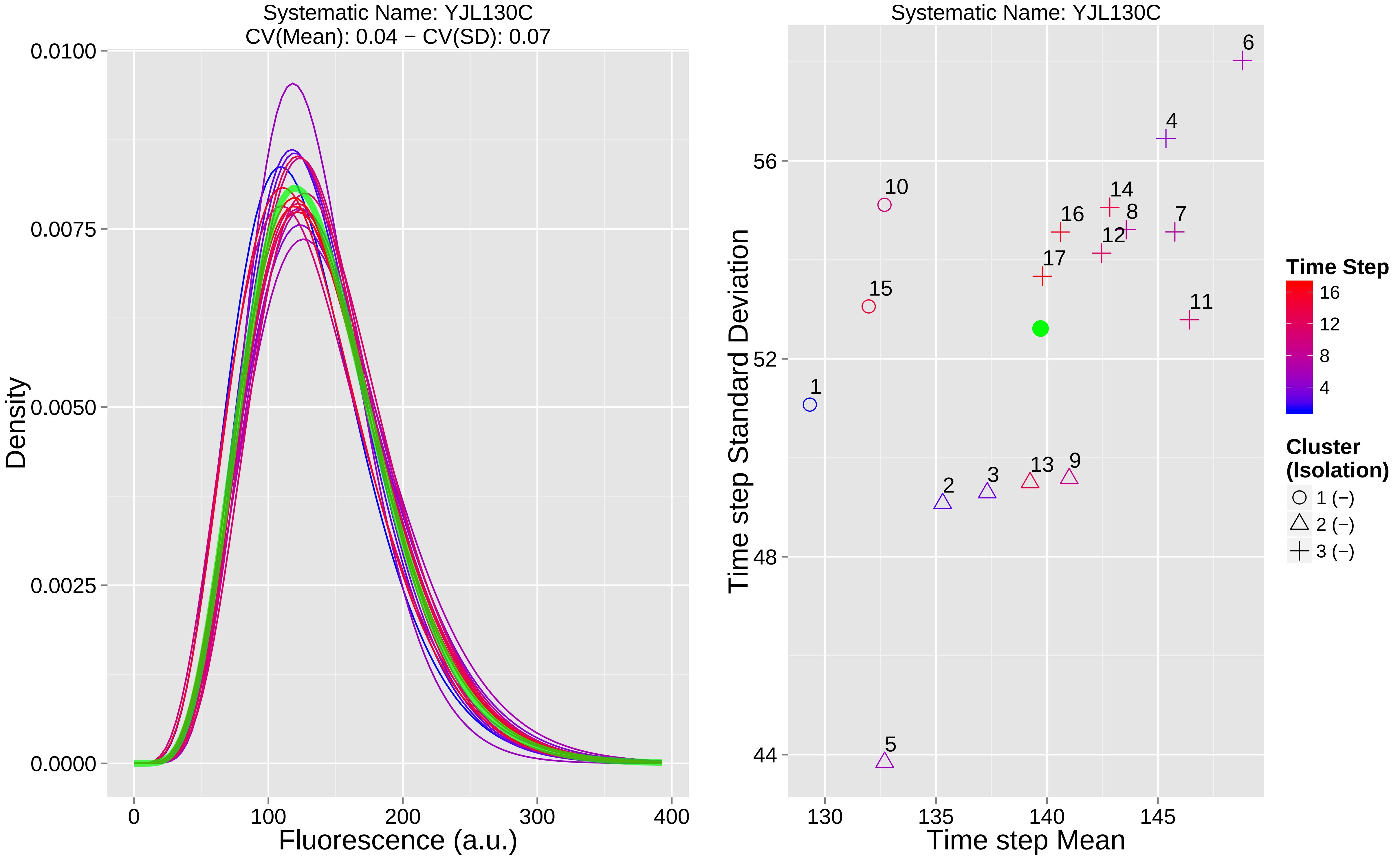
Systematic Name: YJL130C
CV(Mean): 0.04 − CV(SD): 0.07
Density
0.0100
0.0075
0.0050
0.0025
0.0000
0
100
200
300
400
Fluorescence (a.u.)
Systematic Name: YJL130C
Time step Standard Deviation
56
52
48
44
130
135
140
145
Time step Mean
1
2
3
4
5
6
7
8
9
10
11
12
13
14
15
16
17
Time Step
16
12
8
4
Cluster
(Isolation)
1 (−)
2 (−)
3 (−)

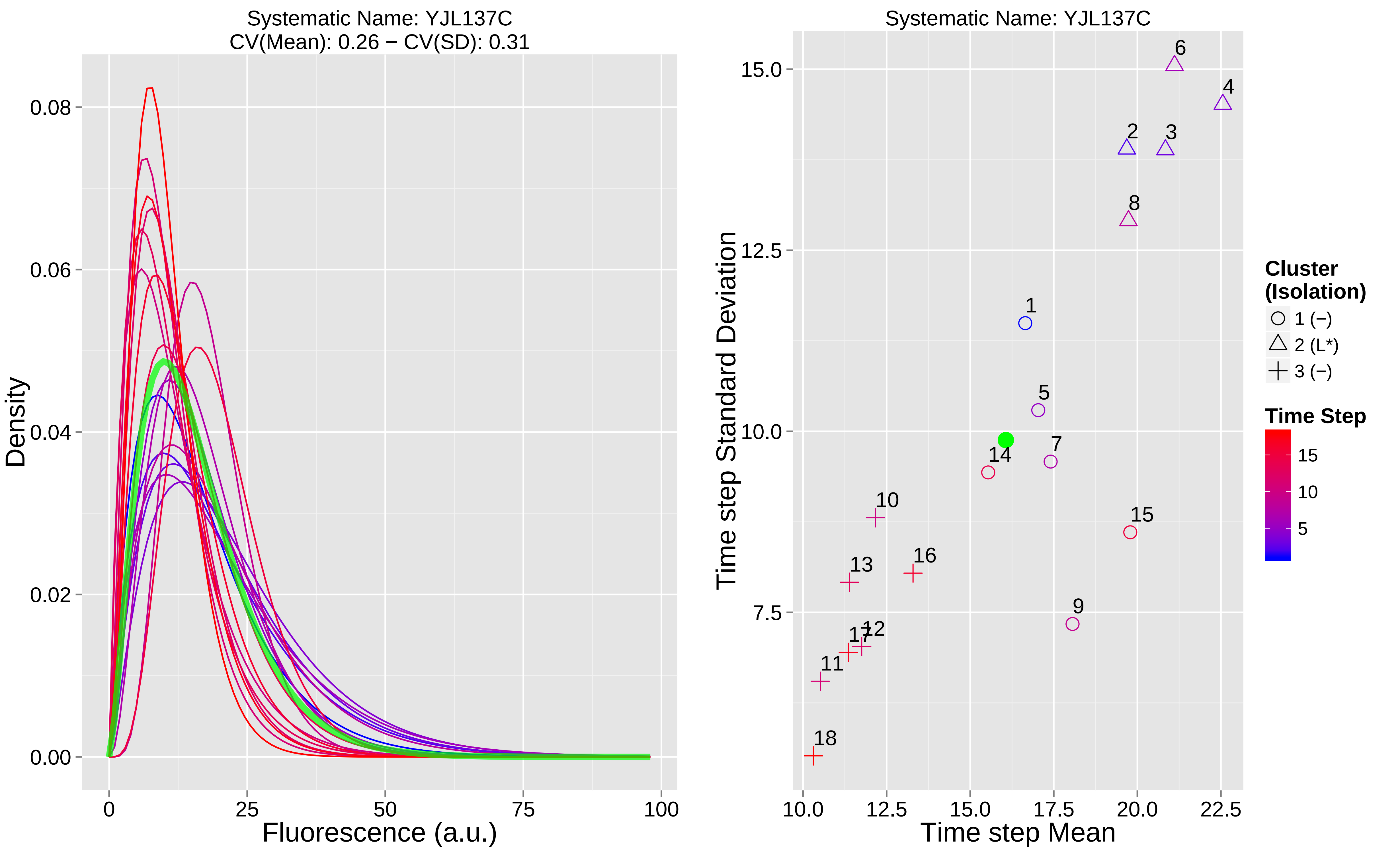

Systematic Name: YJL137C
CV(Mean): 0.26 − CV(SD): 0.31
Density
0.00
0.02
0.04
0.06
0.08
0
25
50
75
100
Fluorescence (a.u.)
Systematic Name: YJL137C
Time step Standard Deviation
7.5
10.0
12.5
15.0
10.0
12.5
15.0
17.5
20.0
22.5
Time step Mean
1
2
3
4
5
6
7
8
9
10
11
12
13
14
15
16
17
18
Cluster (Isolation)
1 (−)
2 (L*)
3 (−)
Time Step
15
10
5

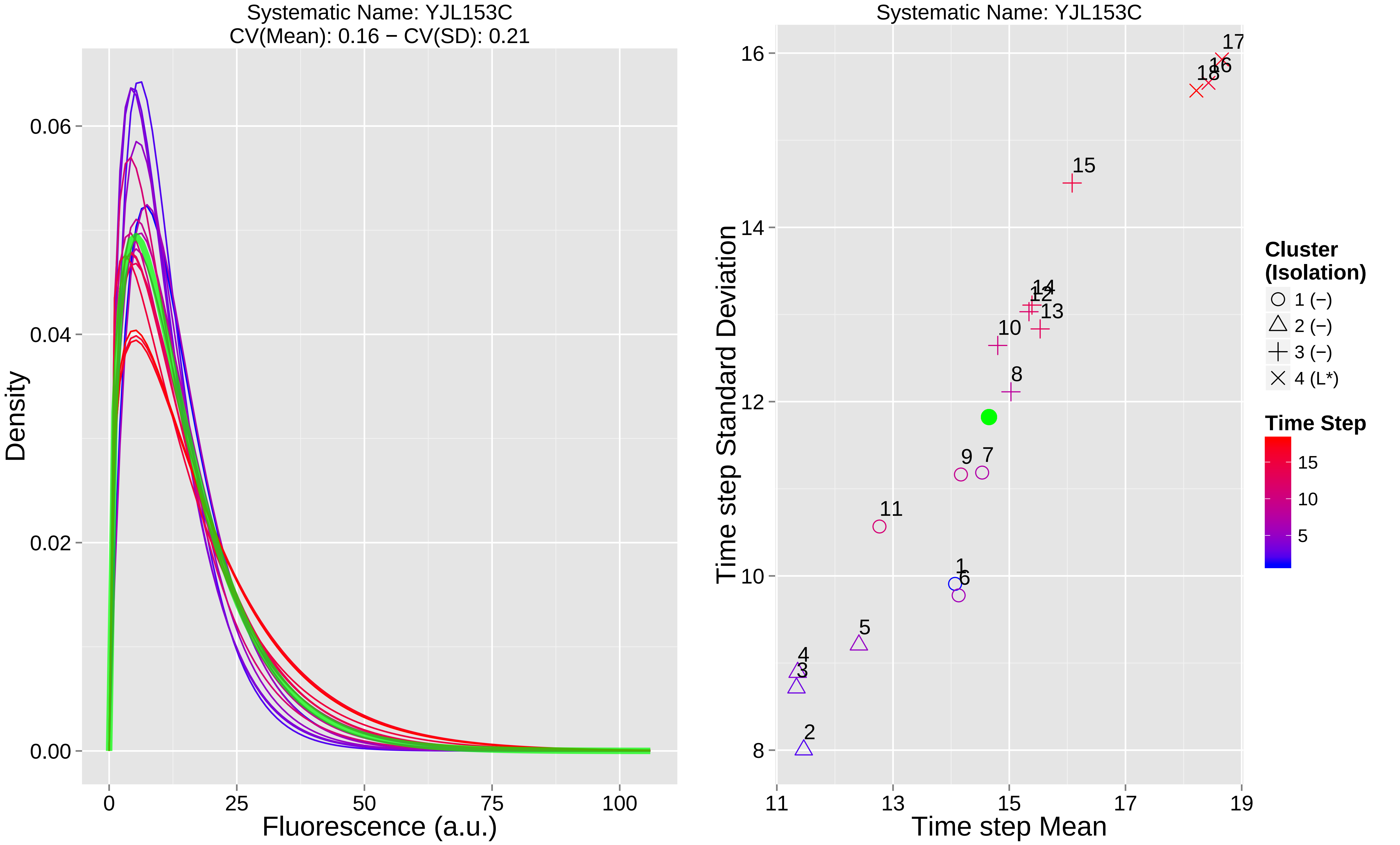

Systematic Name: YJL153C
CV(Mean): 0.16 − CV(SD): 0.21
Density
0.06
0.04
0.02
0.00
0
25
50
75
100
Fluorescence (a.u.)
Systematic Name: YJL153C
Time step Standard Deviation
16
14
12
10
8
11
13
15
17
19
Time step Mean
17
16
18
15
14
12
13
10
8
9
7
11
1
6
5
4
3
2
Cluster
(Isolation)
1 (−)
2 (−)
3 (−)
4 (L*)
Time Step
15
10
5

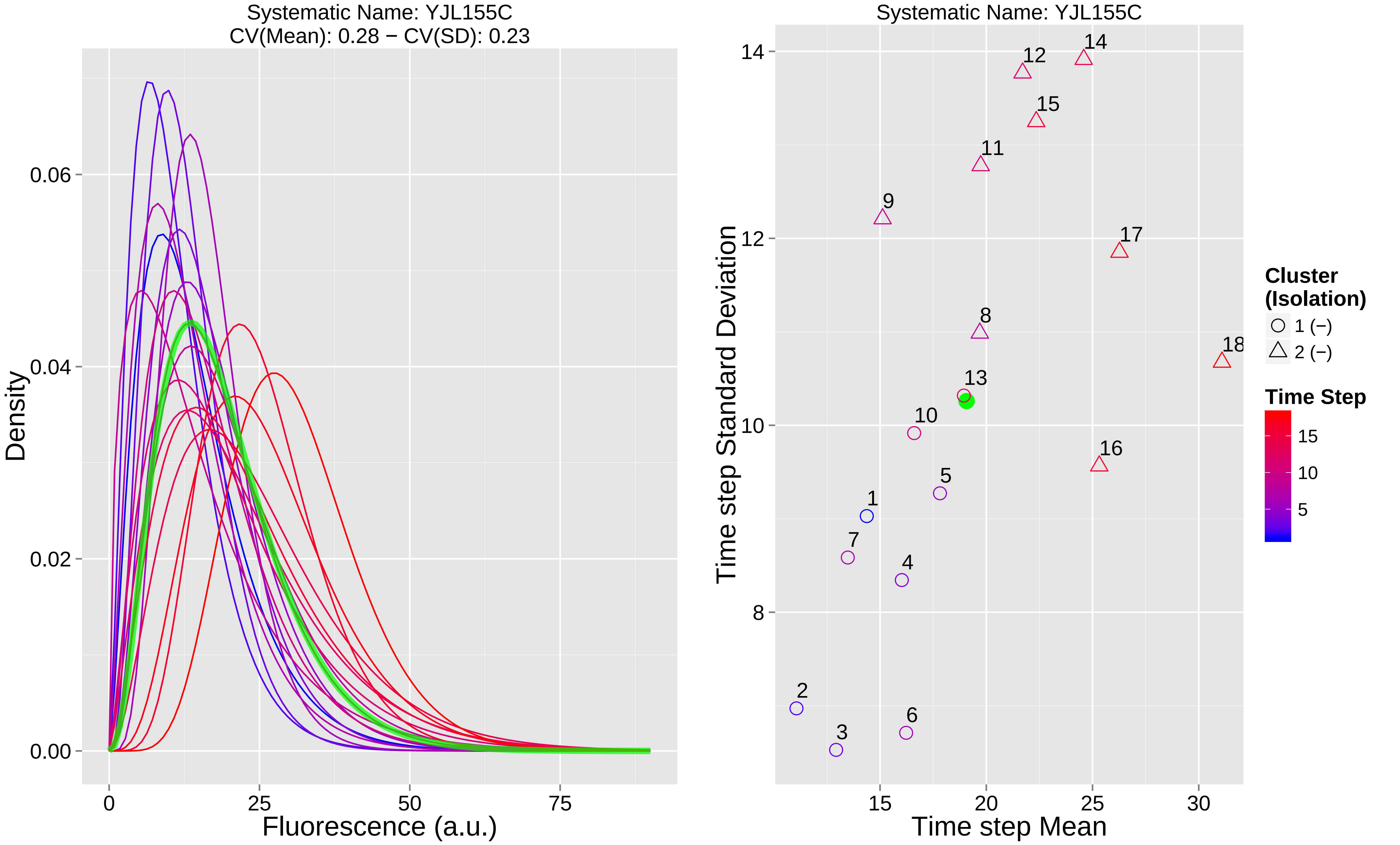
Systematic Name: YJL155C
CV(Mean): 0.28 − CV(SD): 0.23
Density
0.06
0.04
0.02
0.00
0
25
50
75
Fluorescence (a.u.)
Systematic Name: YJL155C
Time step Standard Deviation
14
12
10
8
15
20
25
30
Time step Mean
1
2
3
4
5
6
7
8
9
10
11
12
13
14
15
16
17
18
Cluster
(Isolation)
1 (−)
2 (−)
Time Step
15
10
5

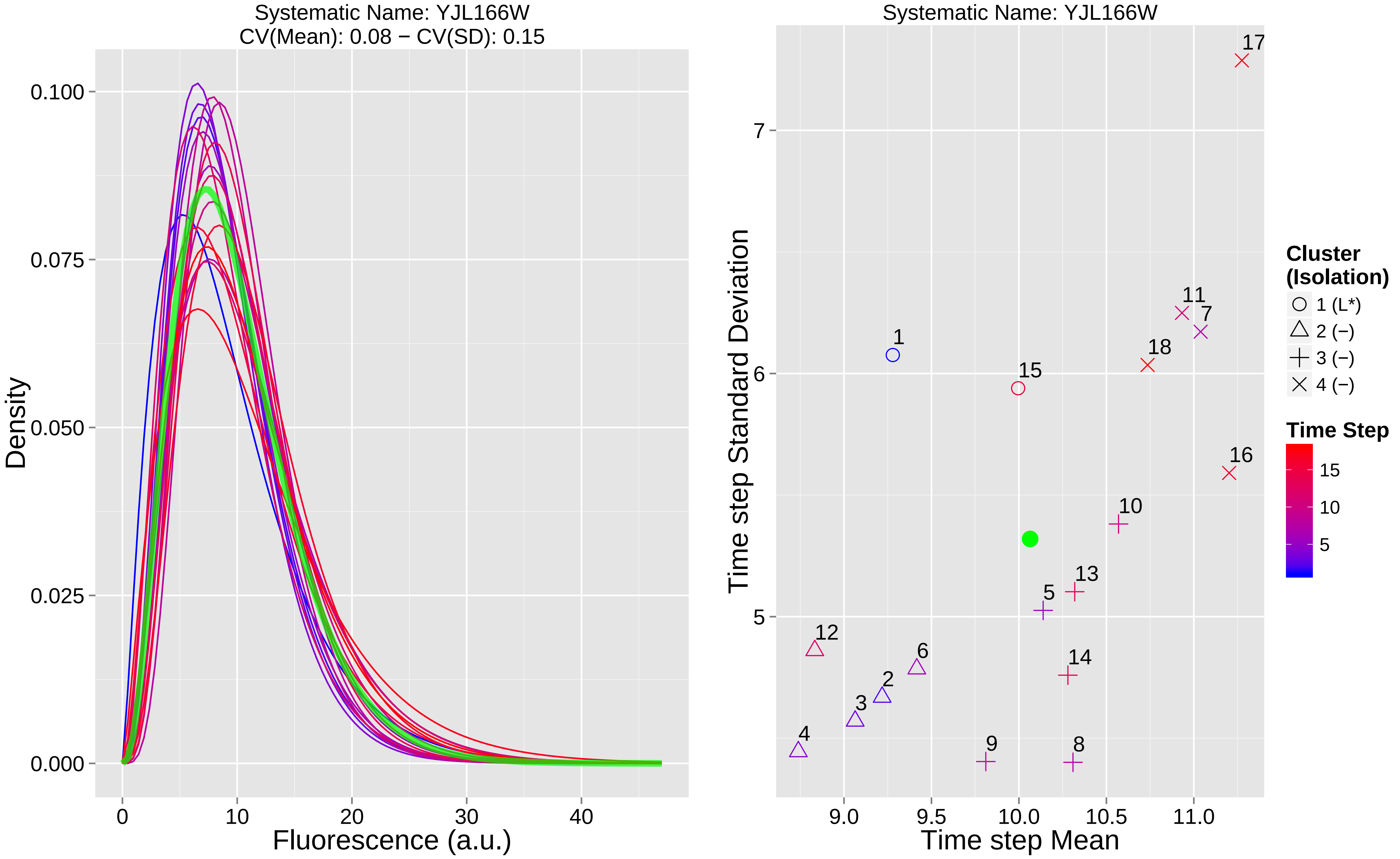

Systematic Name: YJL166W
CV(Mean): 0.08 − CV(SD): 0.15
Density
0.100
0.075
0.050
0.025
0.000
0
10
20
30
40
Fluorescence (a.u.)
Systematic Name: YJL166W
Time step Standard Deviation
7
6
5
9.0
9.5
10.0
10.5
11.0
Time step Mean
17
11
7
1
18
15
16
10
13
5
12
6
14
2
3
4
9
8
Cluster (Isolation)
1 (L*)
2 (−)
3 (−)
4 (−)
Time Step
15
10
5

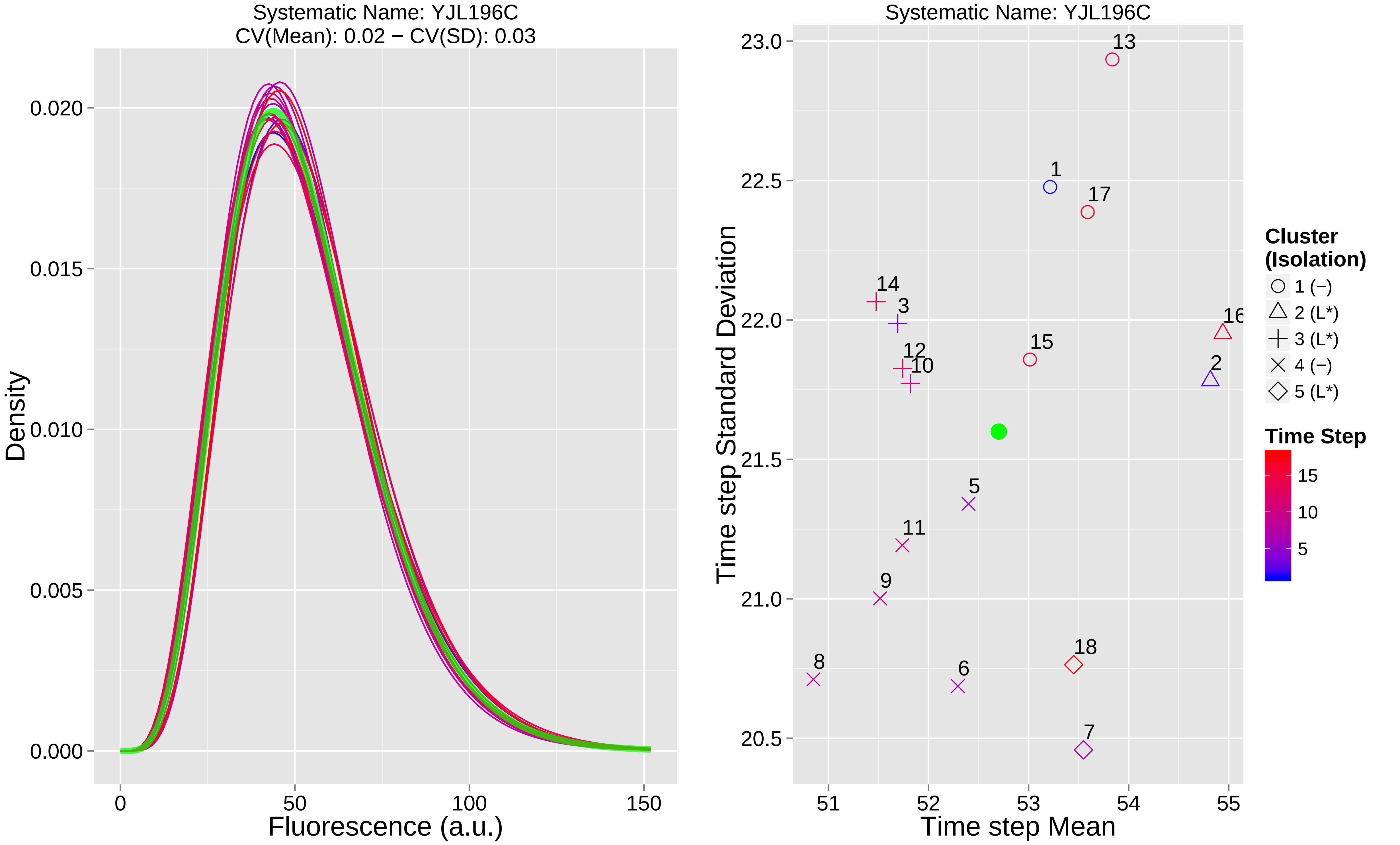

Systematic Name: YJL196C
CV(Mean): 0.02 − CV(SD): 0.03
Density
0.000
0.005
0.010
0.015
0.020
0
50
100
150
Fluorescence (a.u.)
Systematic Name: YJL196C
Time step Standard Deviation
20.5
21.0
21.5
22.0
22.5
23.0
51
52
53
54
55
Time step Mean
1
2
3
5
6
7
8
9
10
11
12
13
14
15
16
17
18
Cluster (Isolation)
1 (−)
2 (L*)
3 (L*)
4 (−)
5 (L*)
Time Step
5
10
15

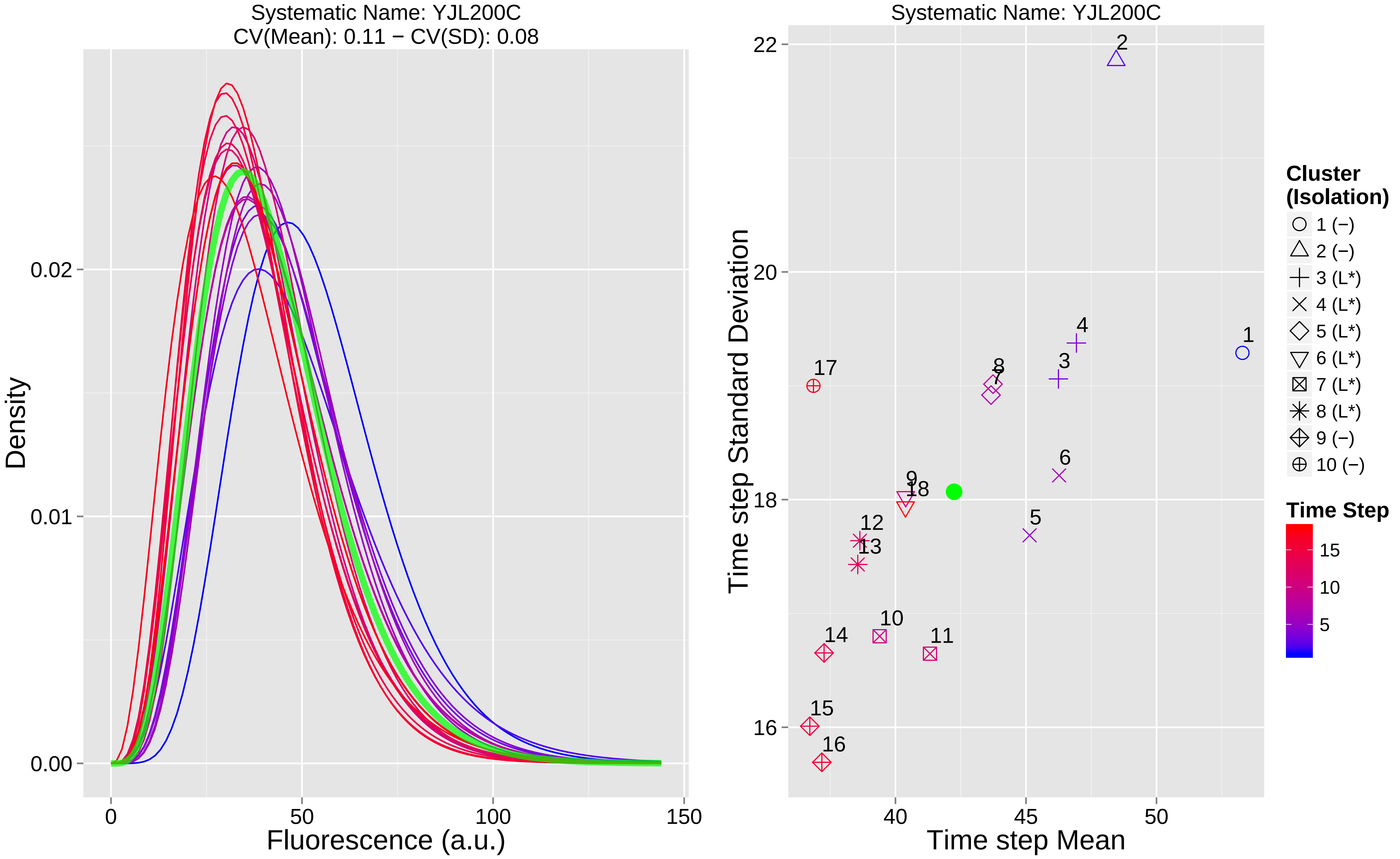

Systematic Name: YJL200C
CV(Mean): 0.11 − CV(SD): 0.08
Density
Fluorescence (a.u.)
0.00
0.01
0.02
0
50
100
150
Systematic Name: YJL200C
Time step Standard Deviation
Time step Mean
16
18
20
22
40
45
50
Cluster (Isolation)
1 (−)
2 (−)
3 (L*)
4 (L*)
5 (L*)
6 (L*)
7 (L*)
8 (L*)
9 (−)
10 (−)
Time Step
15
10
5

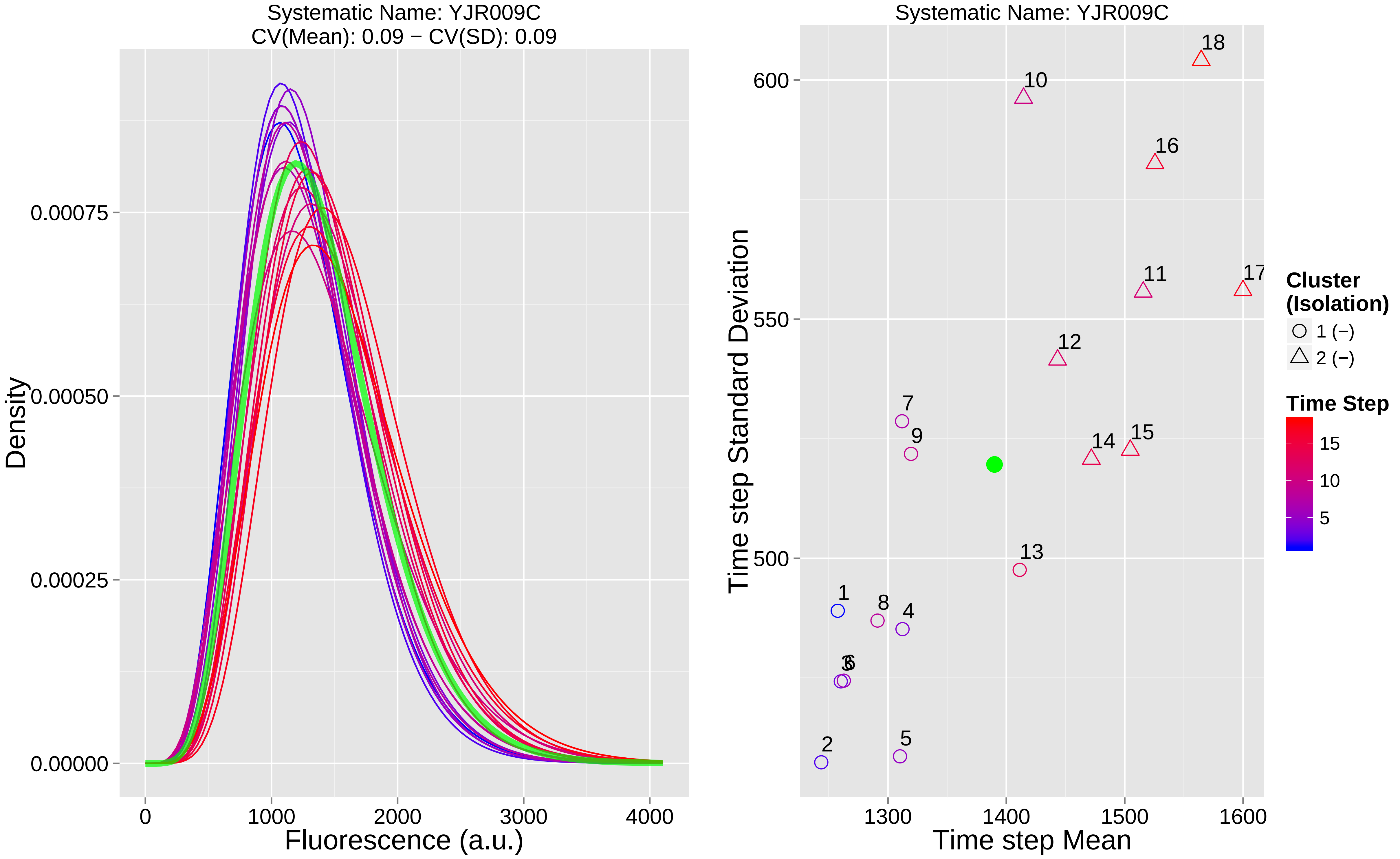
Systematic Name: YJR009C
CV(Mean): 0.09 − CV(SD): 0.09
Density
Fluorescence (a.u.)
0.00000
0.00025
0.00050
0.00075
0
1000
2000
3000
4000
Systematic Name: YJR009C
Time step Standard Deviation
Time step Mean
500
550
600
1300
1400
1500
1600
Cluster
(Isolation)
1 (−)
2 (−)
Time Step
5
10
15
1
2
3
4
5
6
7
8
9
10
11
12
13
14
15
16
17
18

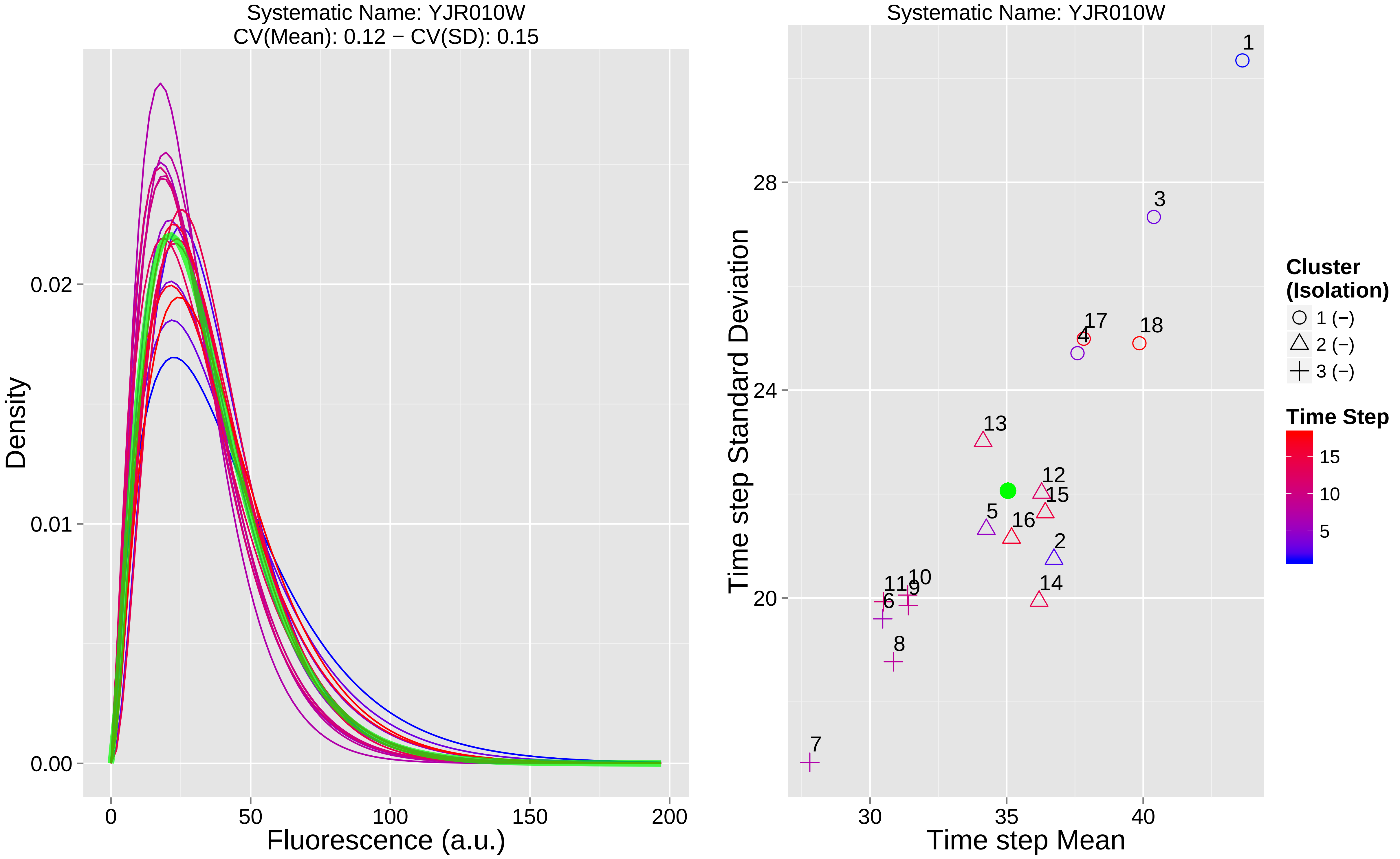

Systematic Name: YJR010W
CV(Mean): 0.12 − CV(SD): 0.15
Density
0.00
0.01
0.02
0
50
100
150
200
Fluorescence (a.u.)
Systematic Name: YJR010W
Time step Standard Deviation
20
24
28
30
35
40
Time step Mean
1
2
3
4
5
6
7
8
9
10
11
12
13
14
15
16
17
18
Cluster (Isolation)
1 (−)
2 (−)
3 (−)
Time Step
15
10
5

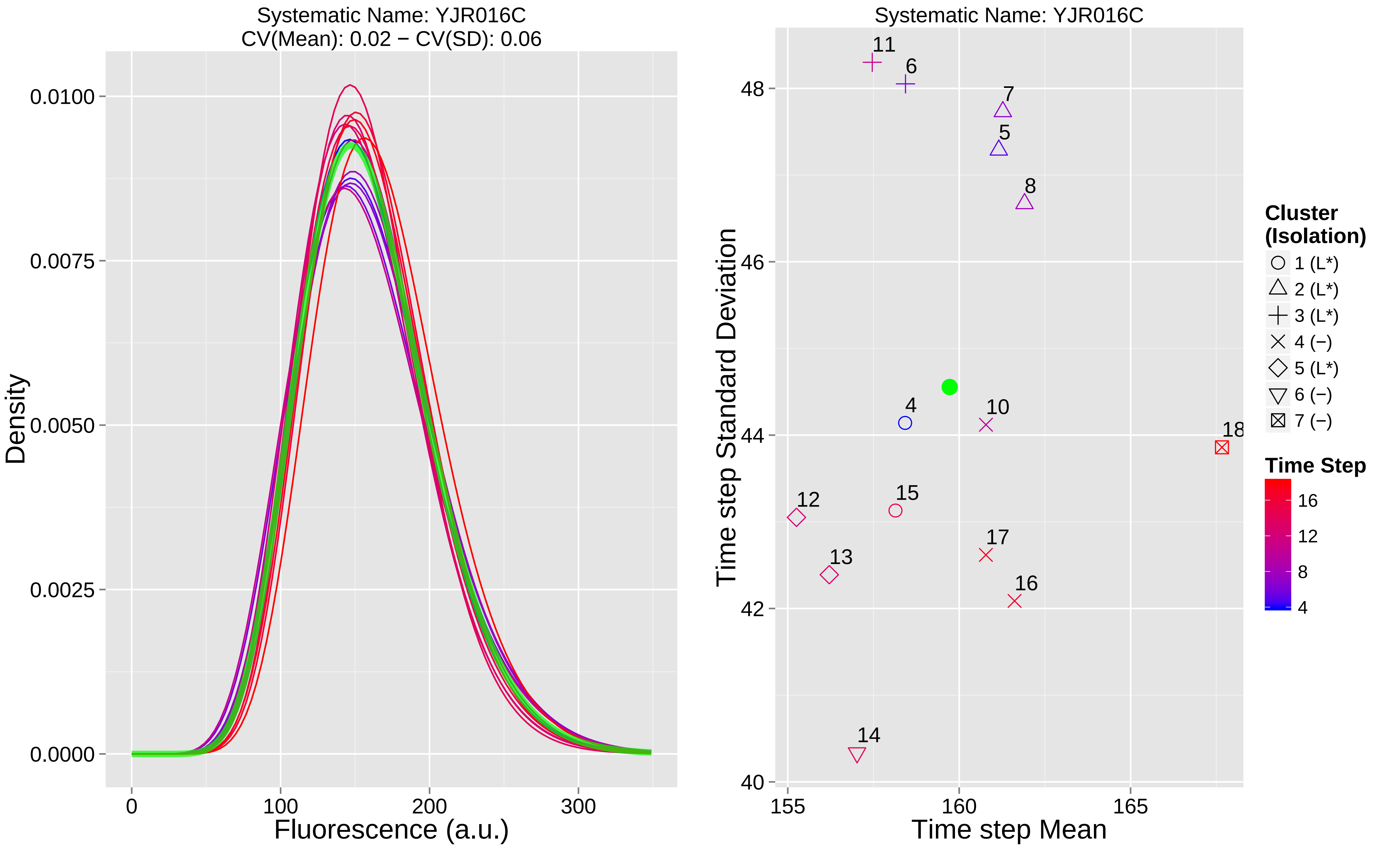

Systematic Name: YJR016C
CV(Mean): 0.02 − CV(SD): 0.06
Density
0.0100
0.0075
0.0050
0.0025
0.0000
0
100
200
300
Fluorescence (a.u.)
Systematic Name: YJR016C
Time step Standard Deviation
48
46
44
42
40
155
160
165
Time step Mean
11
6
7
5
8
4
10
18
12
15
17
13
16
14
Cluster (Isolation)
1 (L*)
2 (L*)
3 (L*)
4 (−)
5 (L*)
6 (−)
7 (−)
Time Step
16
12
8
4

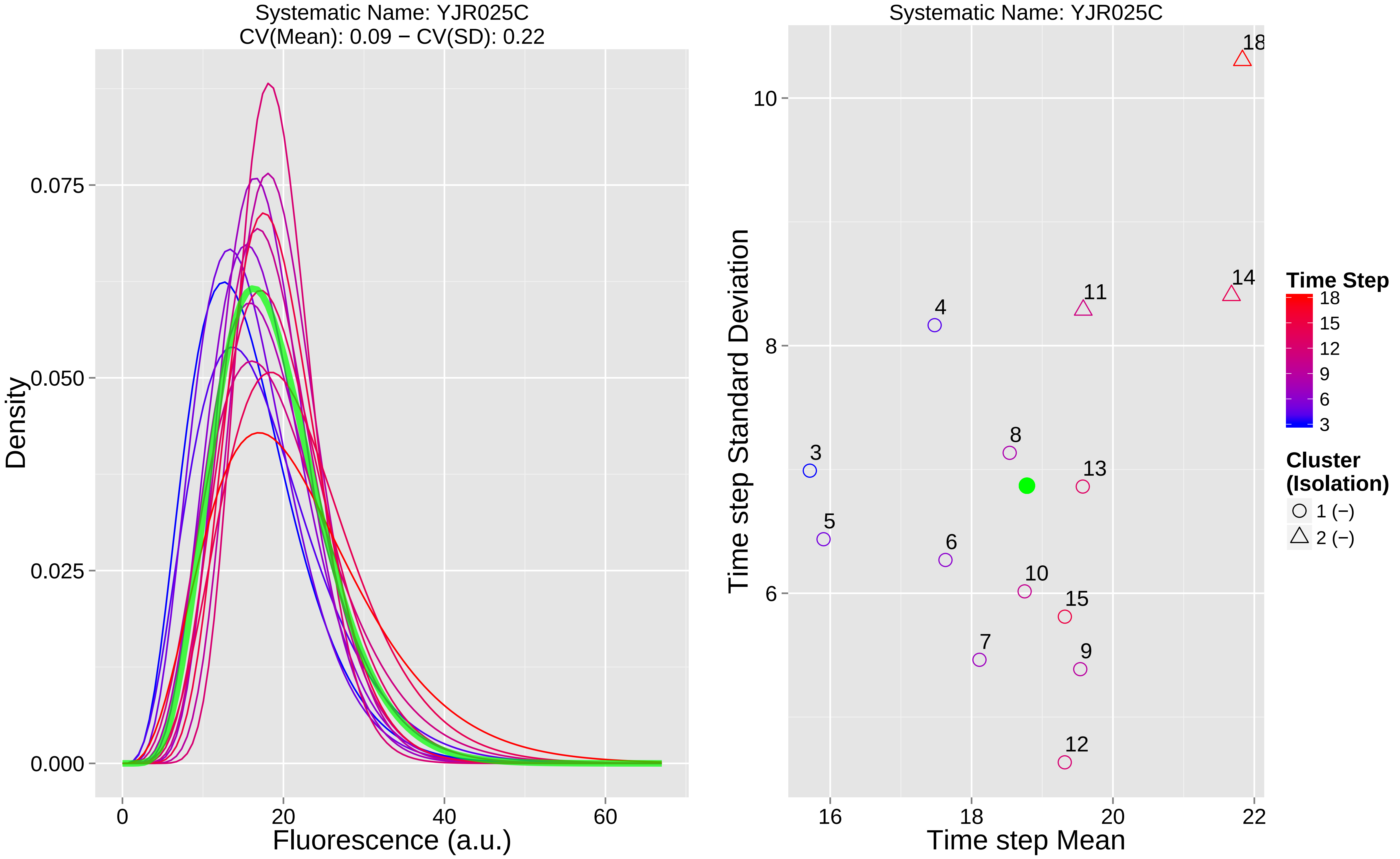

Systematic Name: YJR025C
CV(Mean): 0.09 − CV(SD): 0.22
Density
0.000
0.025
0.050
0.075
0
20
40
60
Fluorescence (a.u.)
Systematic Name: YJR025C
Time step Standard Deviation
Time step Mean
6
8
10
16
18
20
22
3
4
5
6
7
8
9
10
11
12
13
14
15
18
Time Step
18
15
12
9
6
3
Cluster
(Isolation)
1 (−)
2 (−)

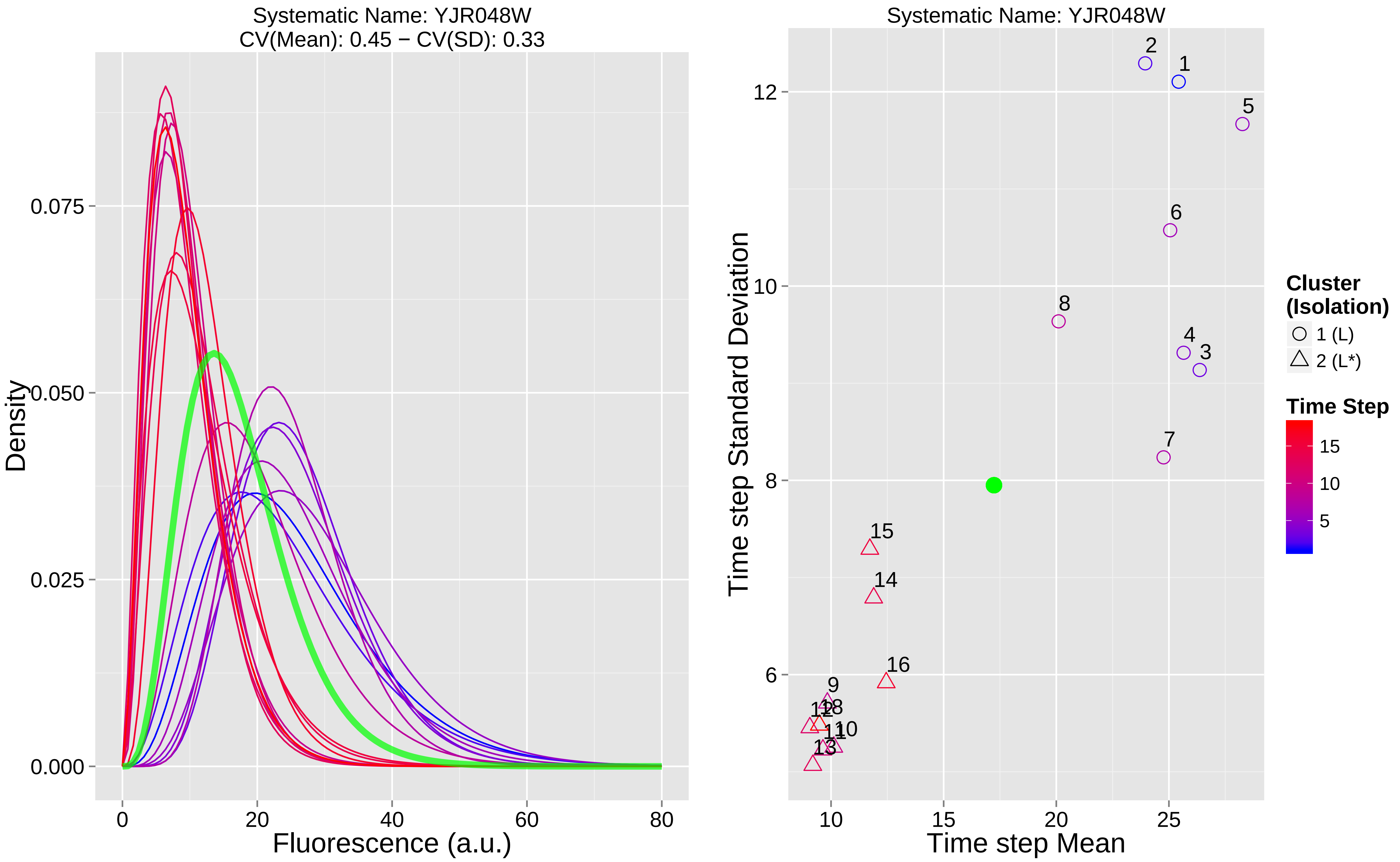
Systematic Name: YJR048W
CV(Mean): 0.45 − CV(SD): 0.33
Density
Fluorescence (a.u.)
0.000
0.025
0.050
0.075
0
20
40
60
80
Systematic Name: YJR048W
Time step Standard Deviation
Time step Mean
6
8
10
12
10
15
20
25
Cluster
(Isolation)
1 (L)
2 (L*)
Time Step
15
10
5

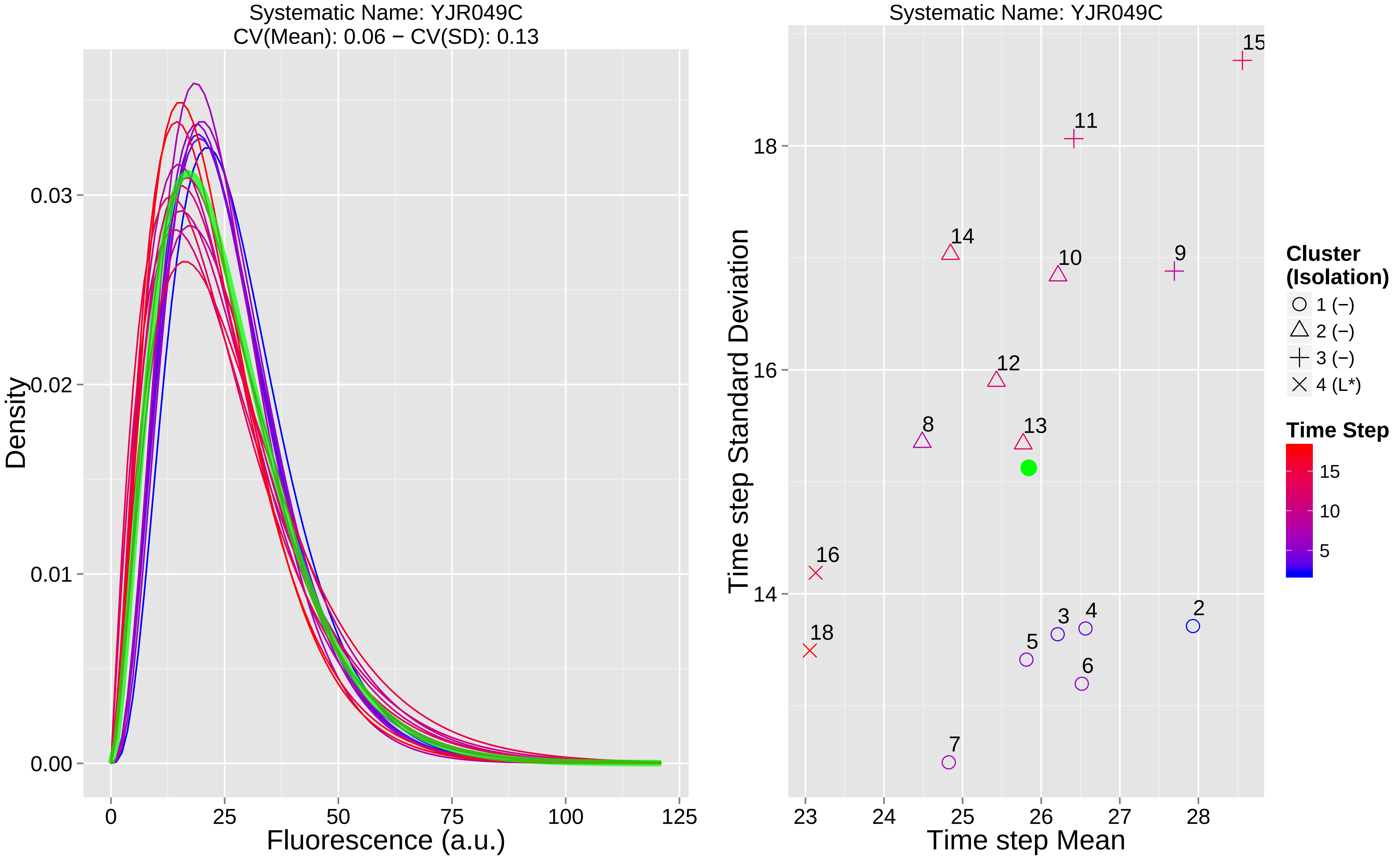

Systematic Name: YJR049C
CV(Mean): 0.06 − CV(SD): 0.13
Density
0.00
0.01
0.02
0.03
0
25
50
75
100
125
Fluorescence (a.u.)
Systematic Name: YJR049C
Time step Standard Deviation
14
16
18
23
24
25
26
27
28
Time step Mean
15
11
14
10
9
12
8
13
16
18
2
3
4
5
6
7
Cluster (Isolation)
1 (−)
2 (−)
3 (−)
4 (L*)
Time Step
15
10
5

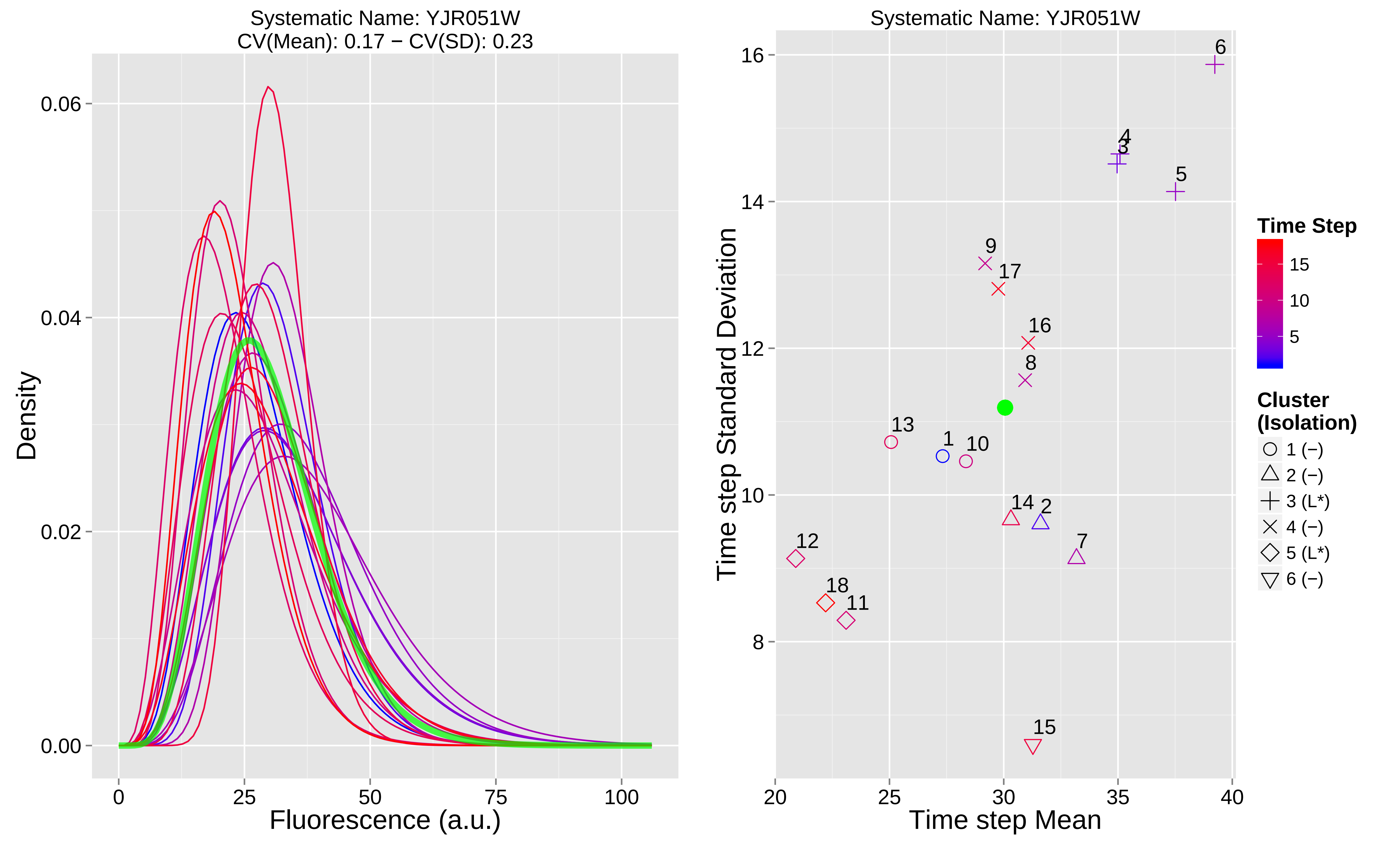

Systematic Name: YJR051W
CV(Mean): 0.17 − CV(SD): 0.23
Density
Fluorescence (a.u.)
Systematic Name: YJR051W
Time step Standard Deviation
Time step Mean
Time Step
Cluster (Isolation)
1 (−)
2 (−)
3 (L*)
4 (−)
5 (L*)
6 (−)

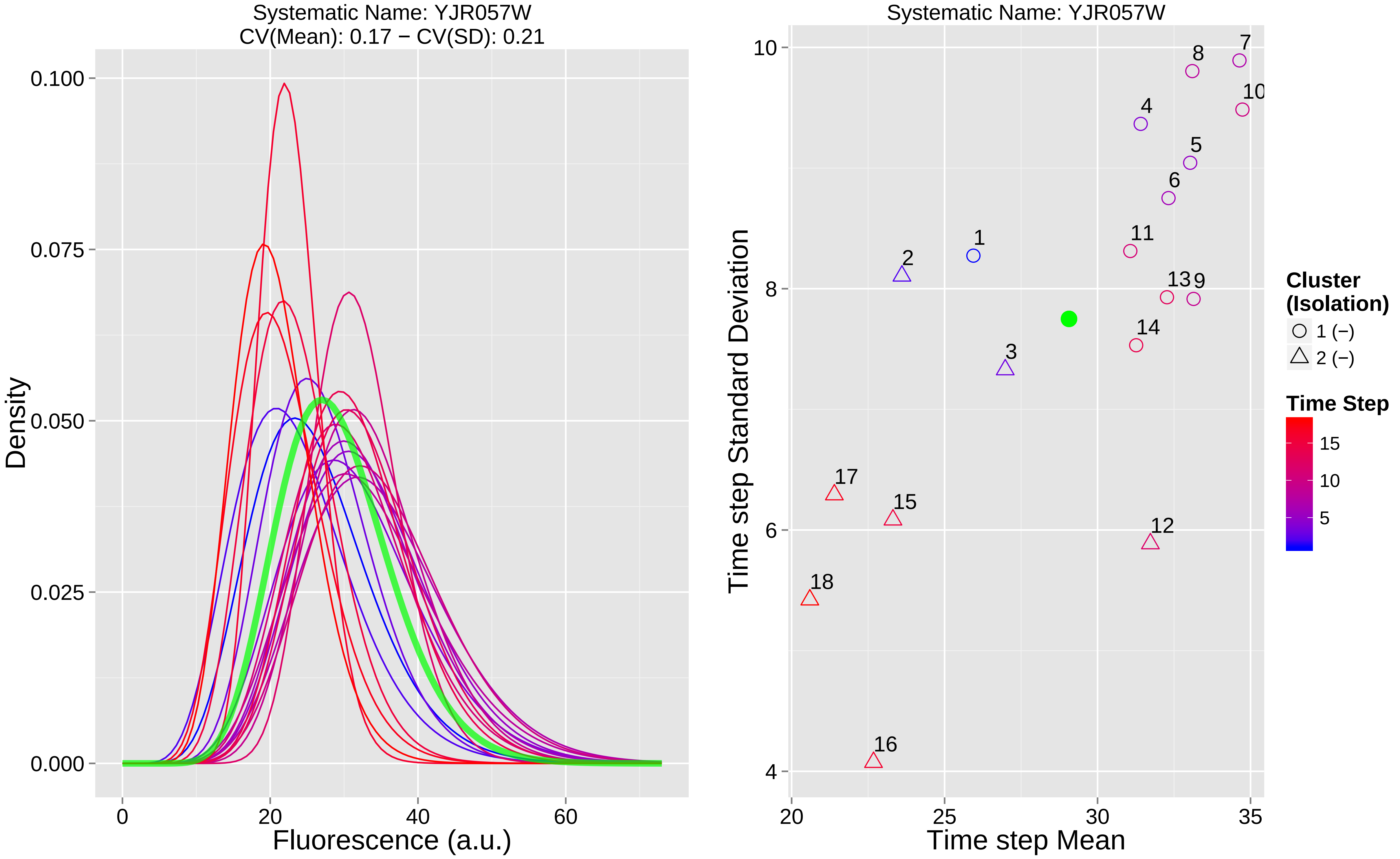

Systematic Name: YJR057W
CV(Mean): 0.17 − CV(SD): 0.21
Density
0.100
0.075
0.050
0.025
0.000
0
20
40
60
Fluorescence (a.u.)
Systematic Name: YJR057W
Time step Standard Deviation
10
8
6
4
20
25
30
35
Time step Mean
1
2
3
4
5
6
7
8
9
10
11
12
13
14
15
16
17
18
Cluster
(Isolation)
1 (−)
2 (−)
Time Step
15
10
5

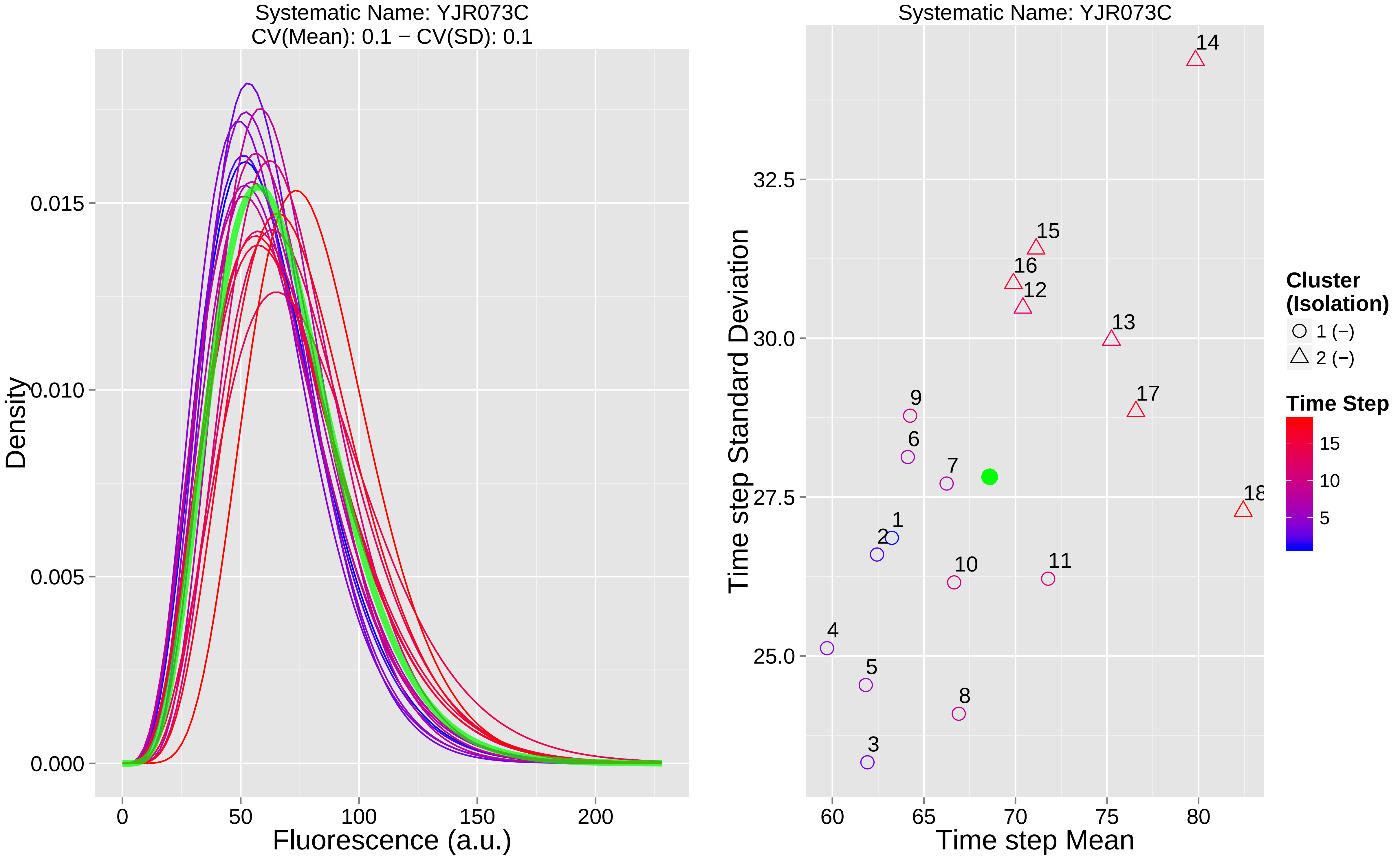

Systematic Name: YJR073C
CV(Mean): 0.1 − CV(SD): 0.1
Density
0.000
0.005
0.010
0.015
0
50
100
150
200
Fluorescence (a.u.)
Systematic Name: YJR073C
Time step Standard Deviation
25.0
27.5
30.0
32.5
60
65
70
75
80
Time step Mean
Cluster
(Isolation)
1 (−)
2 (−)
Time Step
15
10
5

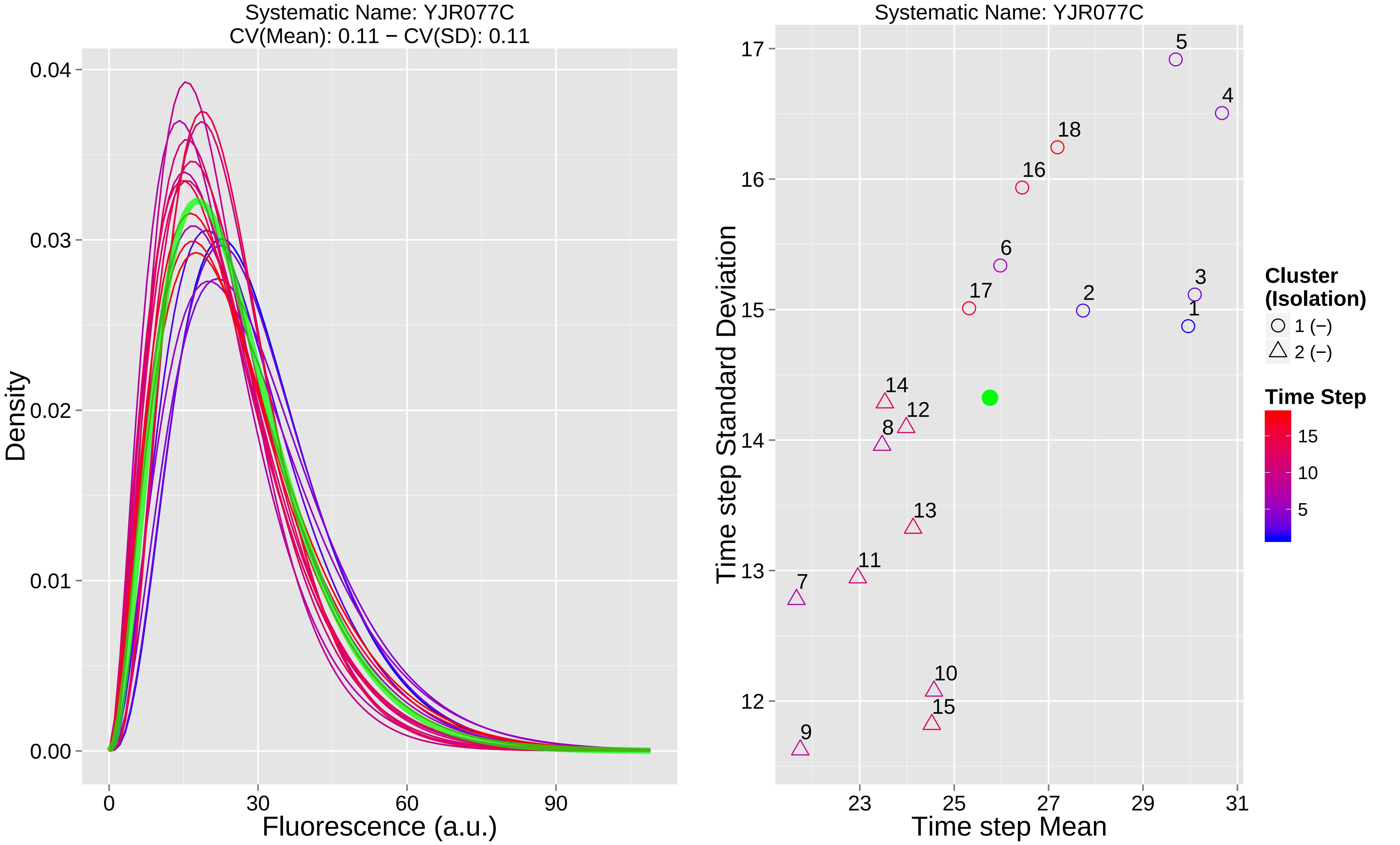

Systematic Name: YJR077C
CV(Mean): 0.11 − CV(SD): 0.11
Density
0.04
0.03
0.02
0.01
0.00
0
30
60
90
Fluorescence (a.u.)
Systematic Name: YJR077C
Time step Standard Deviation
17
16
15
14
13
12
23
25
27
29
31
Time step Mean
Cluster (Isolation)
1 (−)
2 (−)
Time Step
15
10
5

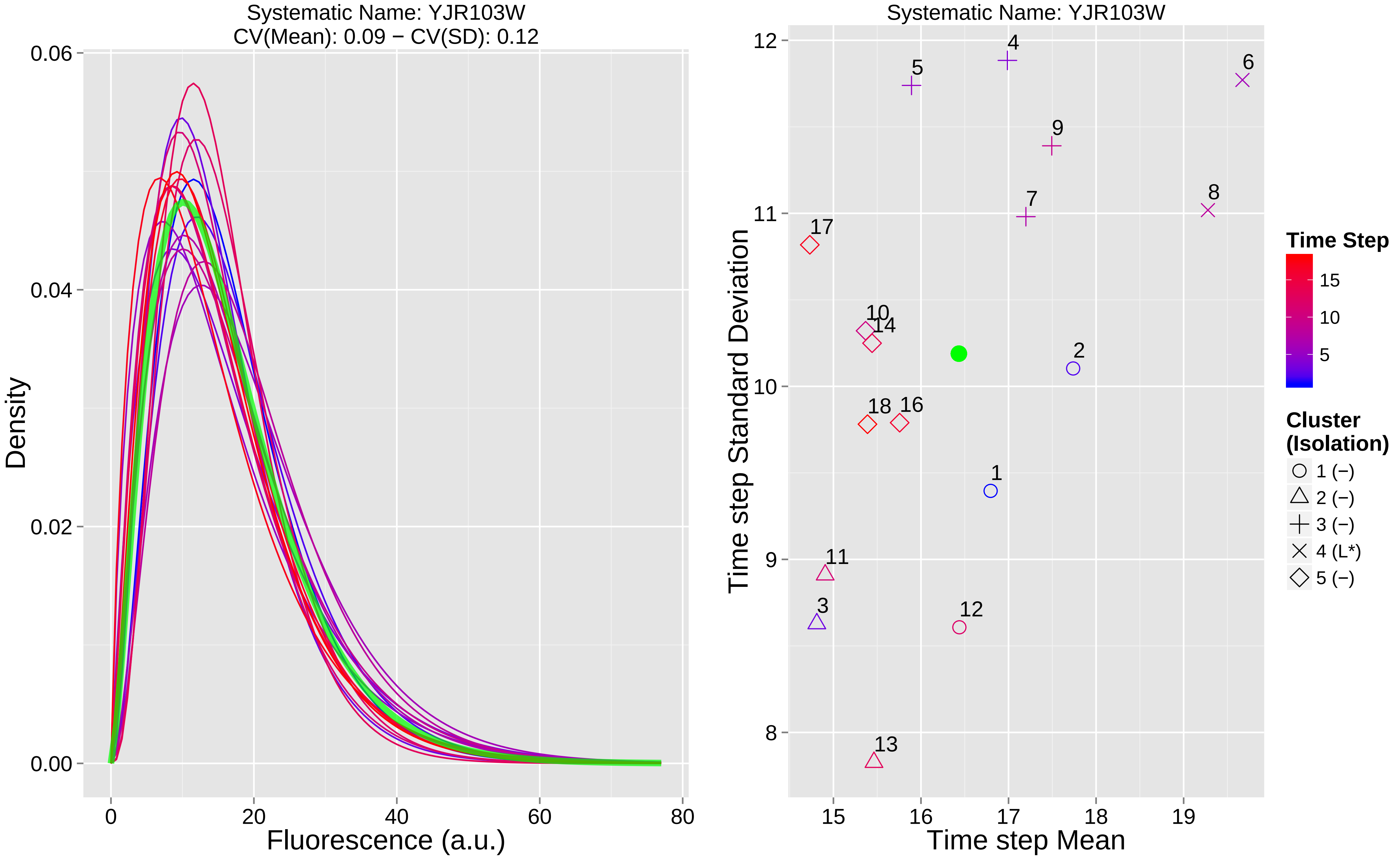

Systematic Name: YJR103W
CV(Mean): 0.09 − CV(SD): 0.12
Density
0.06
0.04
0.02
0.00
0
20
40
60
80
Fluorescence (a.u.)
Systematic Name: YJR103W
Time step Standard Deviation
12
11
10
9
8
15
16
17
18
19
Time step Mean
1
2
3
4
5
6
7
8
9
10
11
12
13
14
16
17
18
Time Step
15
10
5
Cluster
(Isolation)
1 (−)
2 (−)
3 (−)
4 (L*)
5 (−)

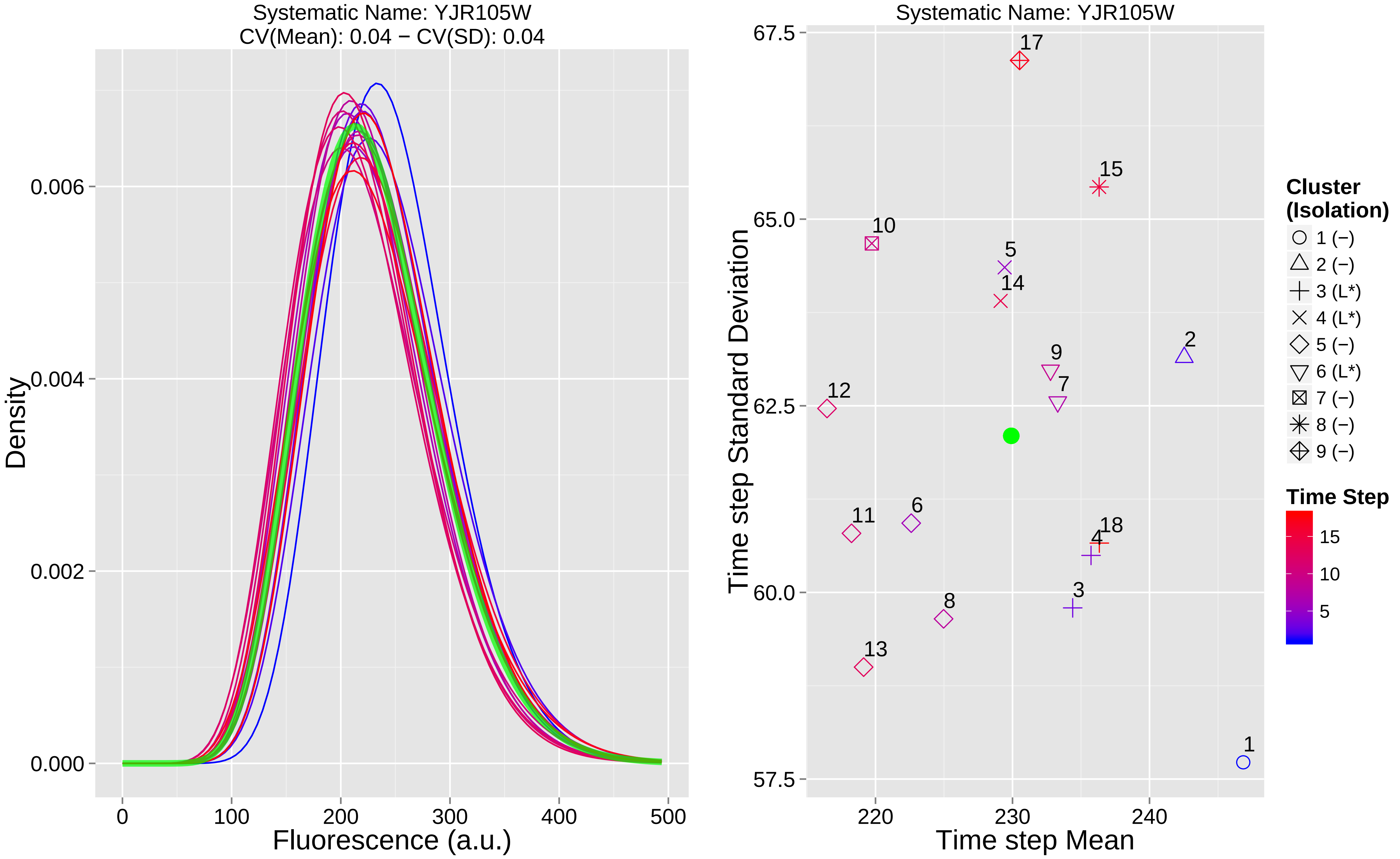

Systematic Name: YJR105W
CV(Mean): 0.04 − CV(SD): 0.04
Density
0.006
0.004
0.002
0.000
0
100
200
300
400
500
Fluorescence (a.u.)
Systematic Name: YJR105W
Time step Standard Deviation
67.5
65.0
62.5
60.0
57.5
220
230
240
Time step Mean
17
15
10
5
14
9
2
7
12
6
11
18
4
3
8
13
1
Cluster
(Isolation)
1 (−)
2 (−)
3 (L*)
4 (L*)
5 (−)
6 (L*)
7 (−)
8 (−)
9 (−)
Time Step
15
10
5

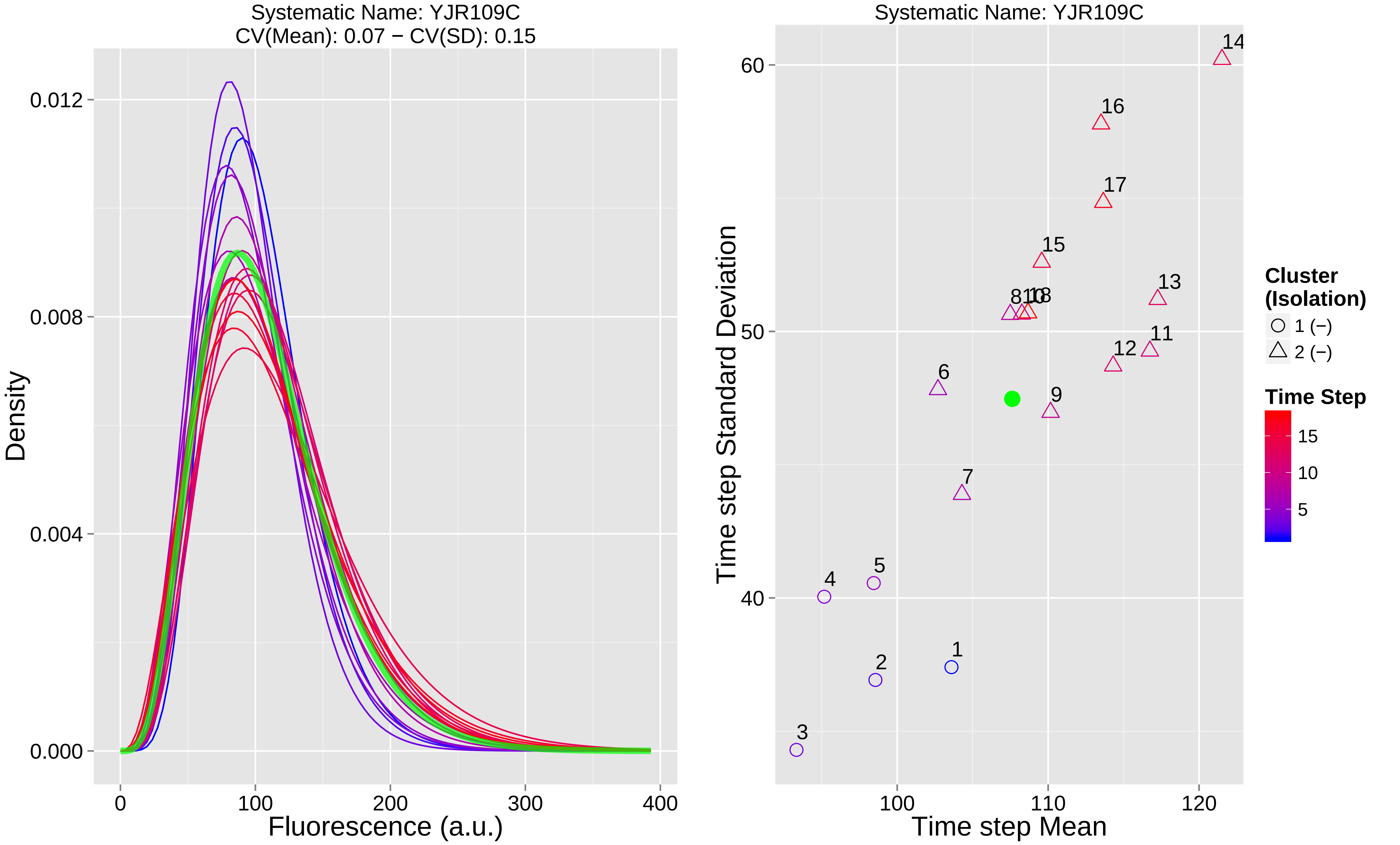

Systematic Name: YJR109C
CV(Mean): 0.07 − CV(SD): 0.15
Density
0.012
0.008
0.004
0.000
0
100
200
300
400
Fluorescence (a.u.)
Systematic Name: YJR109C
Time step Standard Deviation
60
50
40
100
110
120
Time step Mean
Cluster
(Isolation)
1 (−)
2 (−)
Time Step
15
10
5

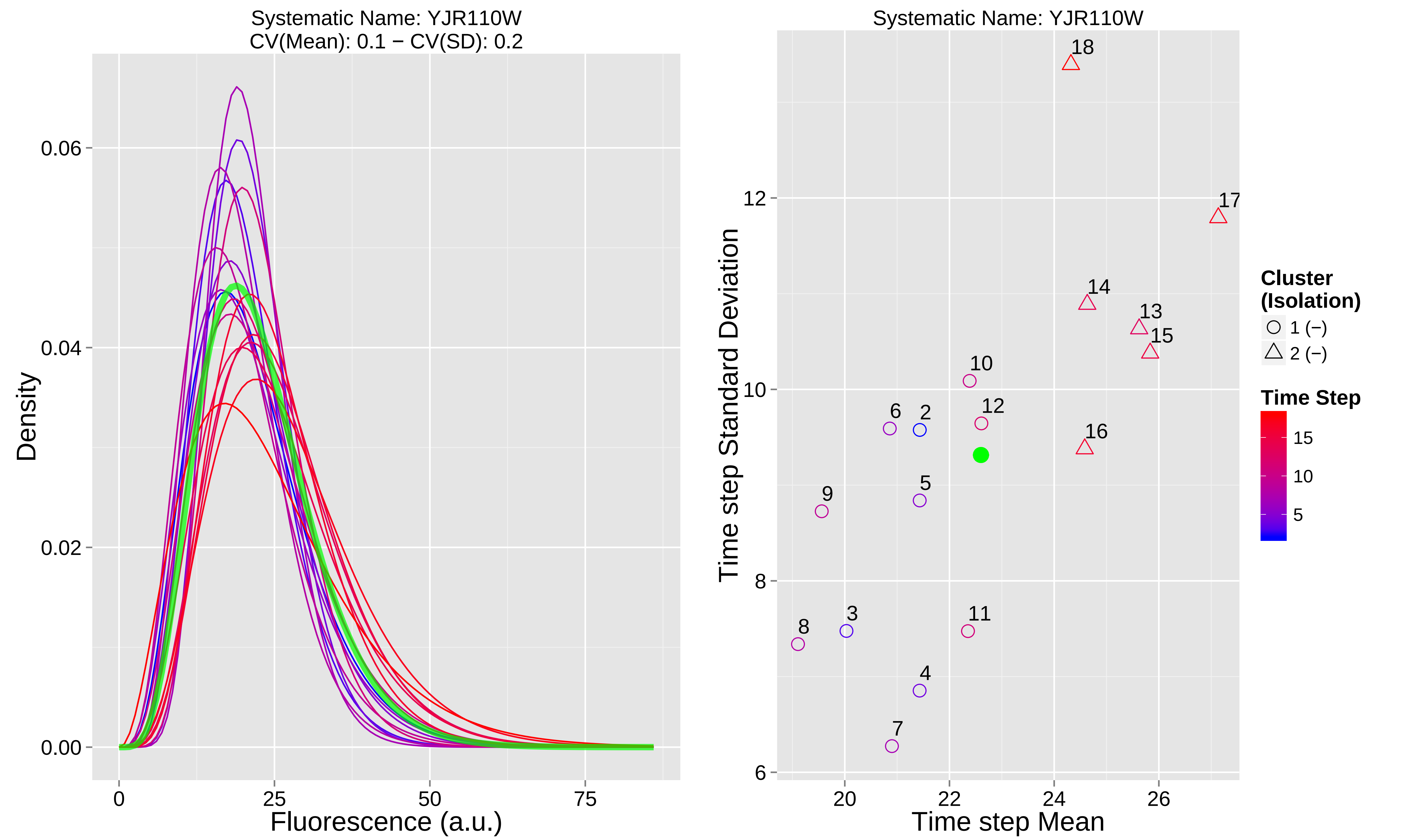
Systematic Name: YJR110W
CV(Mean): 0.1 − CV(SD): 0.2
Density
0.00
0.02
0.04
0.06
0
25
50
75
Fluorescence (a.u.)
Systematic Name: YJR110W
Time step Standard Deviation
Time step Mean
6
8
10
12
20
22
24
26
18
17
14
13
15
10
12
6
2
16
5
9
3
8
11
4
7
Cluster (Isolation)
1 (−)
2 (−)
Time Step
15
10
5

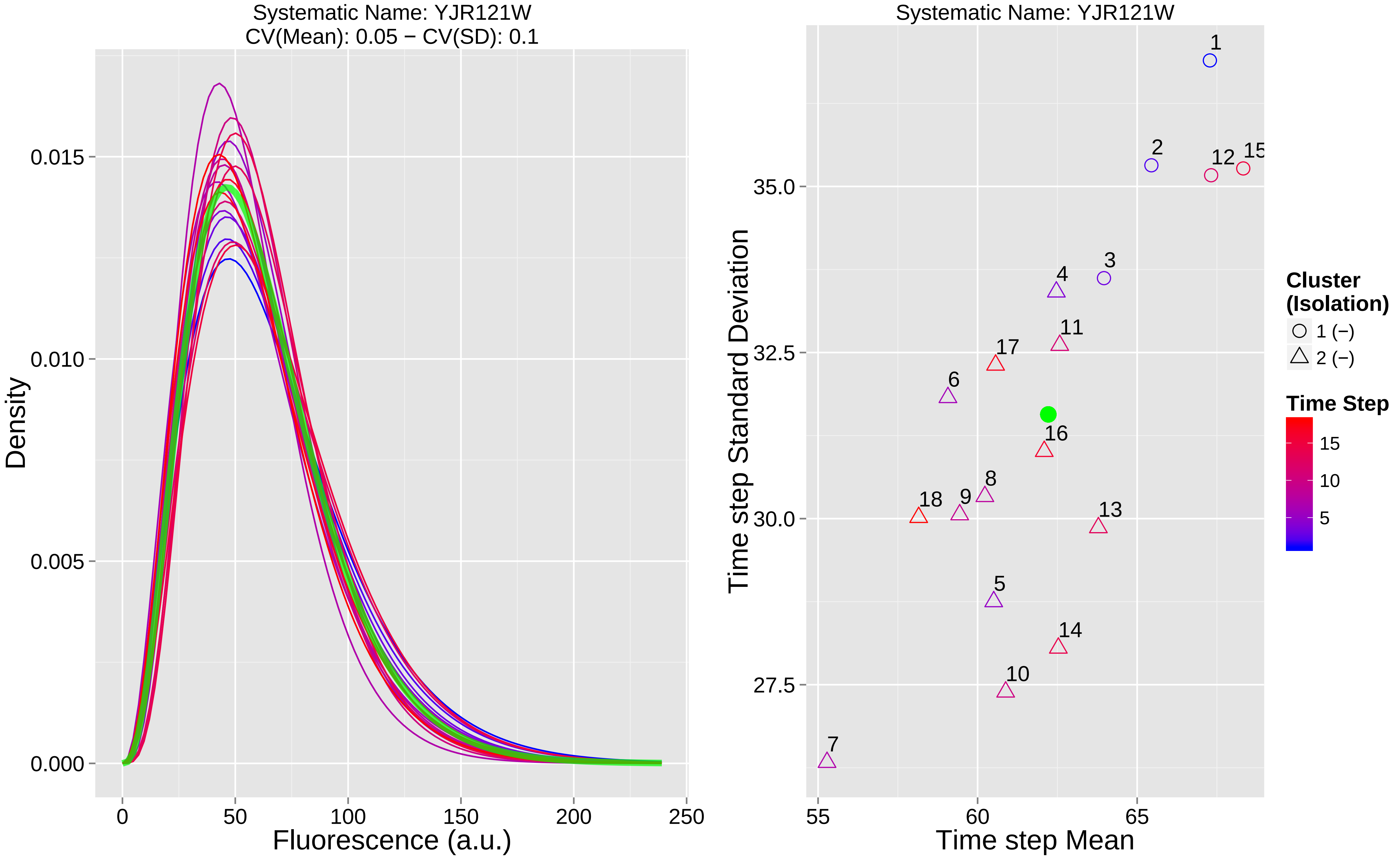
Systematic Name: YJR121W
CV(Mean): 0.05 − CV(SD): 0.1
Density
Fluorescence (a.u.)
0.000
0.005
0.010
0.015
0
50
100
150
200
250
Systematic Name: YJR121W
Time step Standard Deviation
Time step Mean
27.5
30.0
32.5
35.0
55
60
65
1
2
3
4
5
6
7
8
9
10
11
12
13
14
15
16
17
18
Cluster (Isolation)
1 (−)
2 (−)
Time Step
15
10
5

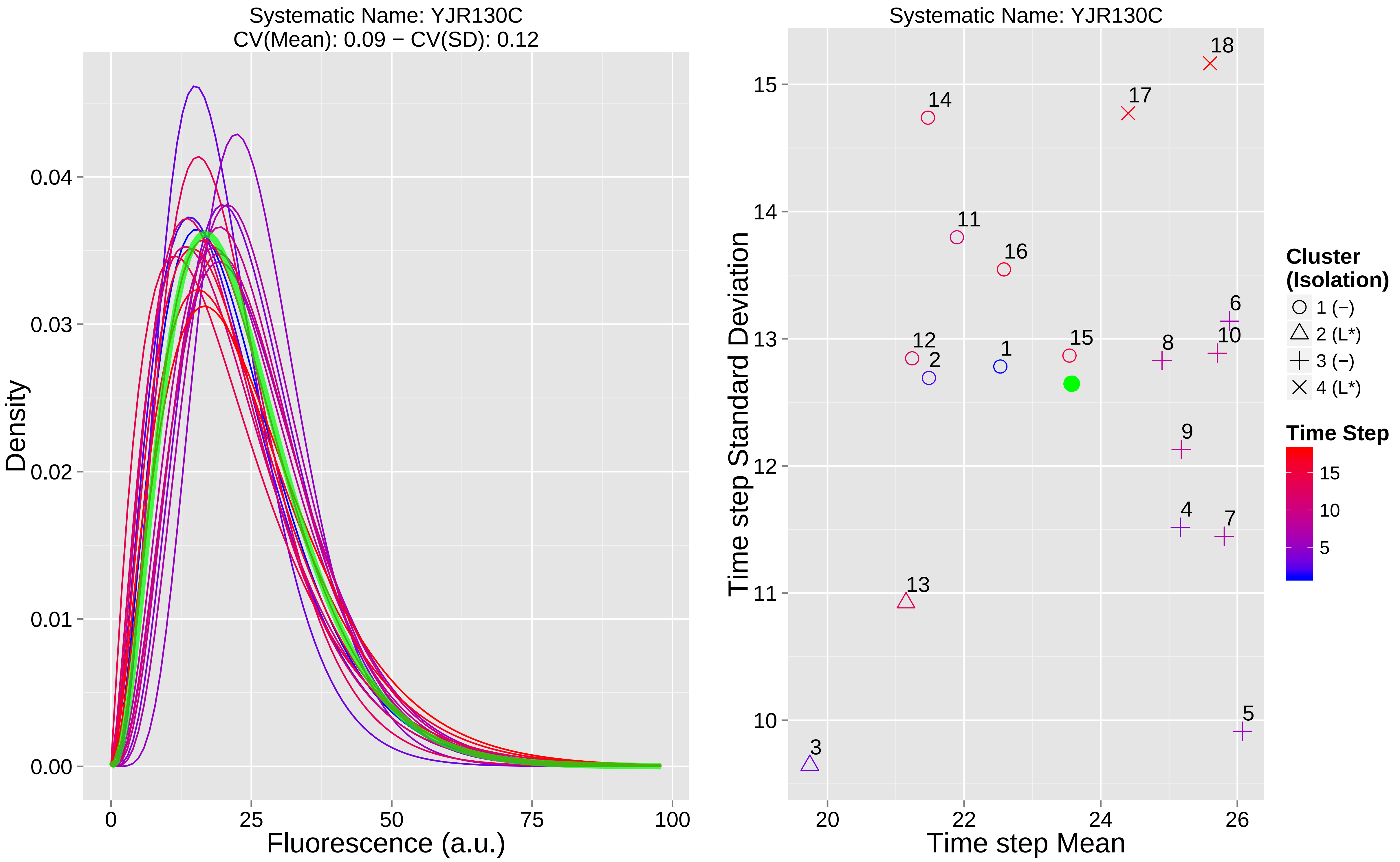
Systematic Name: YJR130C
CV(Mean): 0.09 − CV(SD): 0.12
Density
0.00
0.01
0.02
0.03
0.04
0
25
50
75
100
Fluorescence (a.u.)
Systematic Name: YJR130C
Time step Standard Deviation
10
11
12
13
14
15
20
22
24
26
Time step Mean
1
2
3
4
5
6
7
8
9
10
11
12
13
14
15
16
17
18
Cluster (Isolation)
1 (−)
2 (L*)
3 (−)
4 (L*)
Time Step
15
10
5

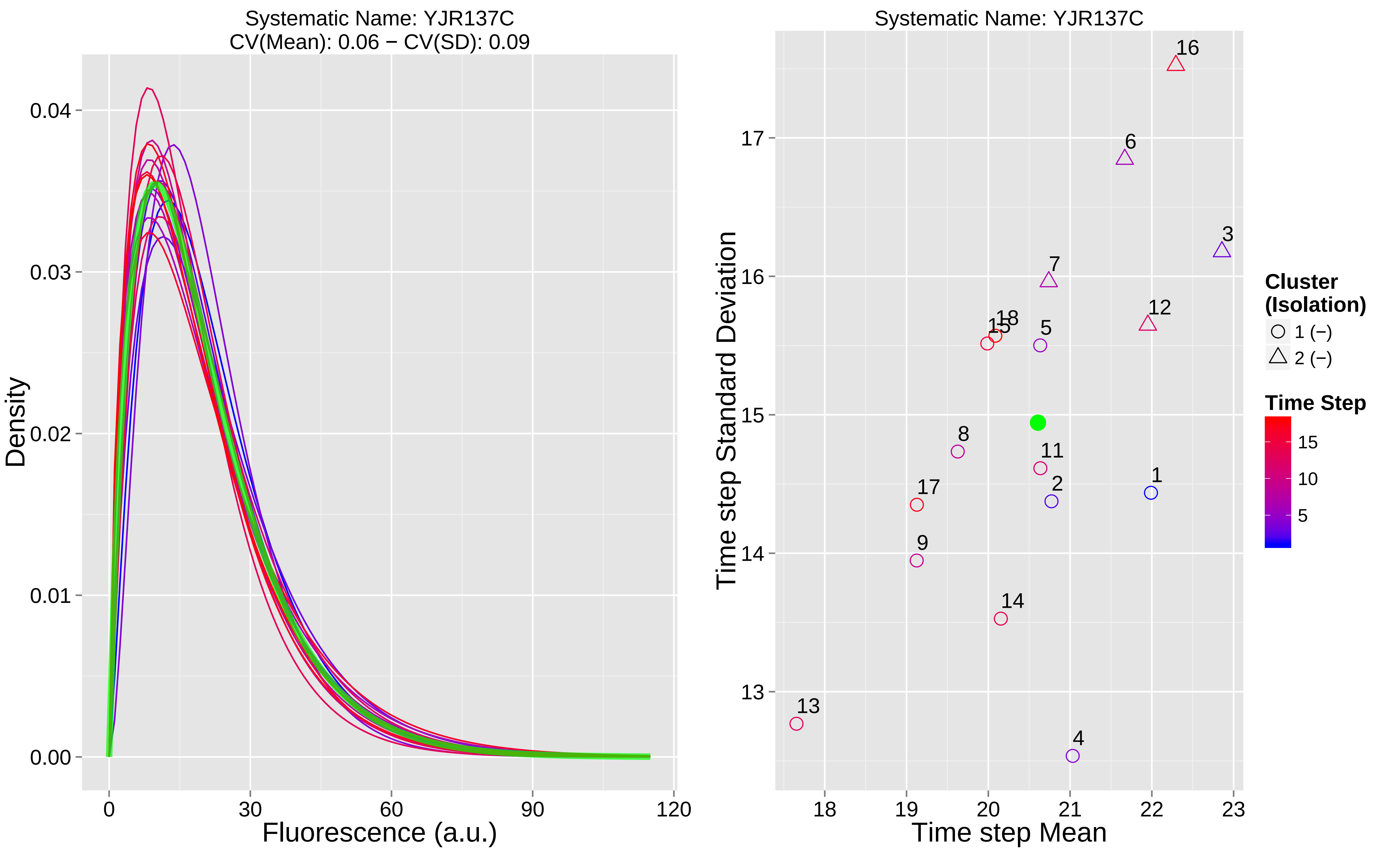

Systematic Name: YJR137C
CV(Mean): 0.06 − CV(SD): 0.09
Density
Fluorescence (a.u.)
0.00
0.01
0.02
0.03
0.04
0
30
60
90
120
Systematic Name: YJR137C
Time step Standard Deviation
Time step Mean
13
14
15
16
17
18
19
20
21
22
23
Cluster (Isolation)
1 (−)
2 (−)
Time Step
5
10
15

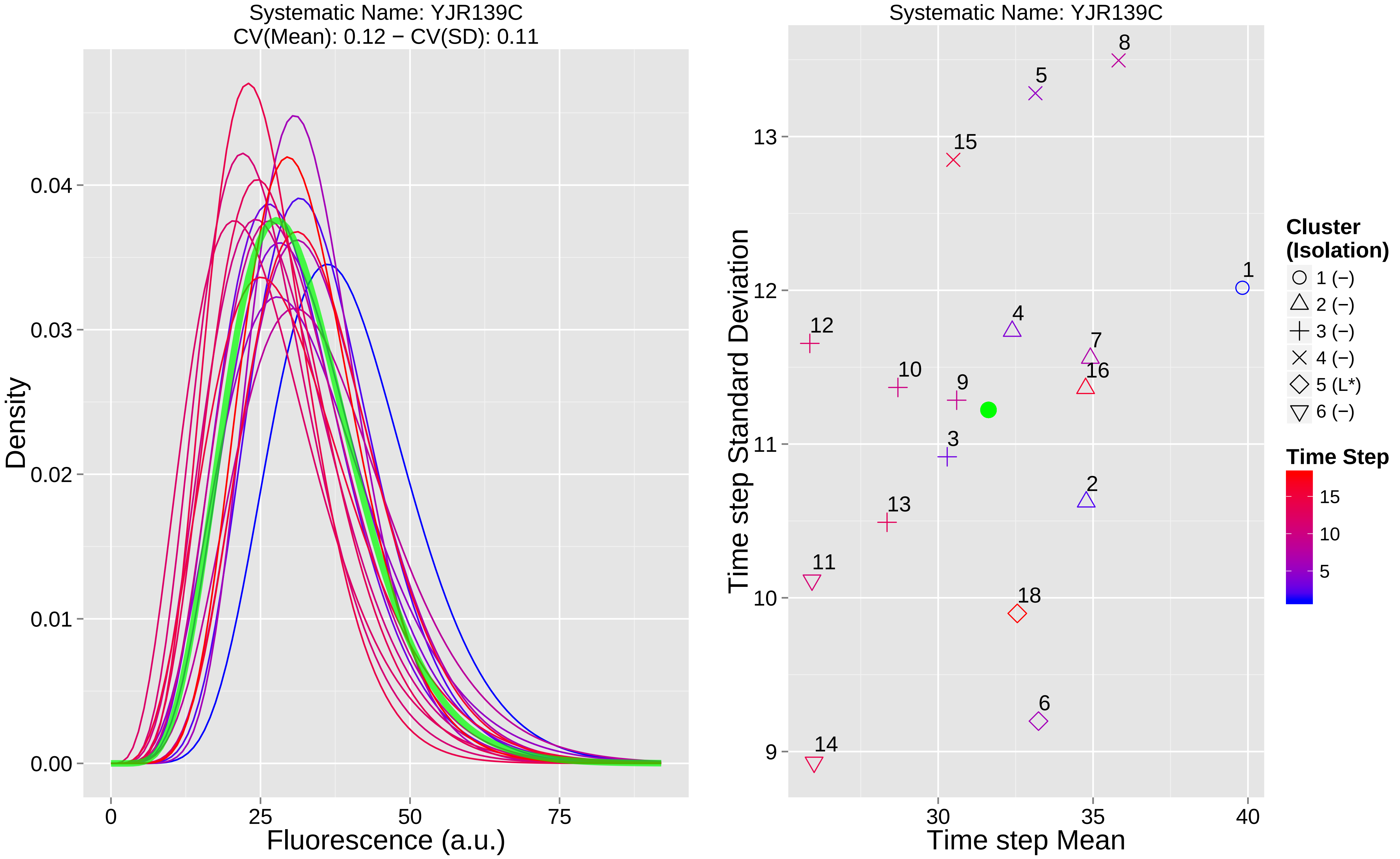

Systematic Name: YJR139C
CV(Mean): 0.12 − CV(SD): 0.11
Density
0.00
0.01
0.02
0.03
0.04
0
25
50
75
Fluorescence (a.u.)
Systematic Name: YJR139C
Time step Standard Deviation
9
10
11
12
13
30
35
40
Time step Mean
1
2
3
4
5
6
7
8
9
10
11
12
13
14
15
16
18
Cluster
(Isolation)
1 (−)
2 (−)
3 (−)
4 (−)
5 (L*)
6 (−)
Time Step
15
10
5

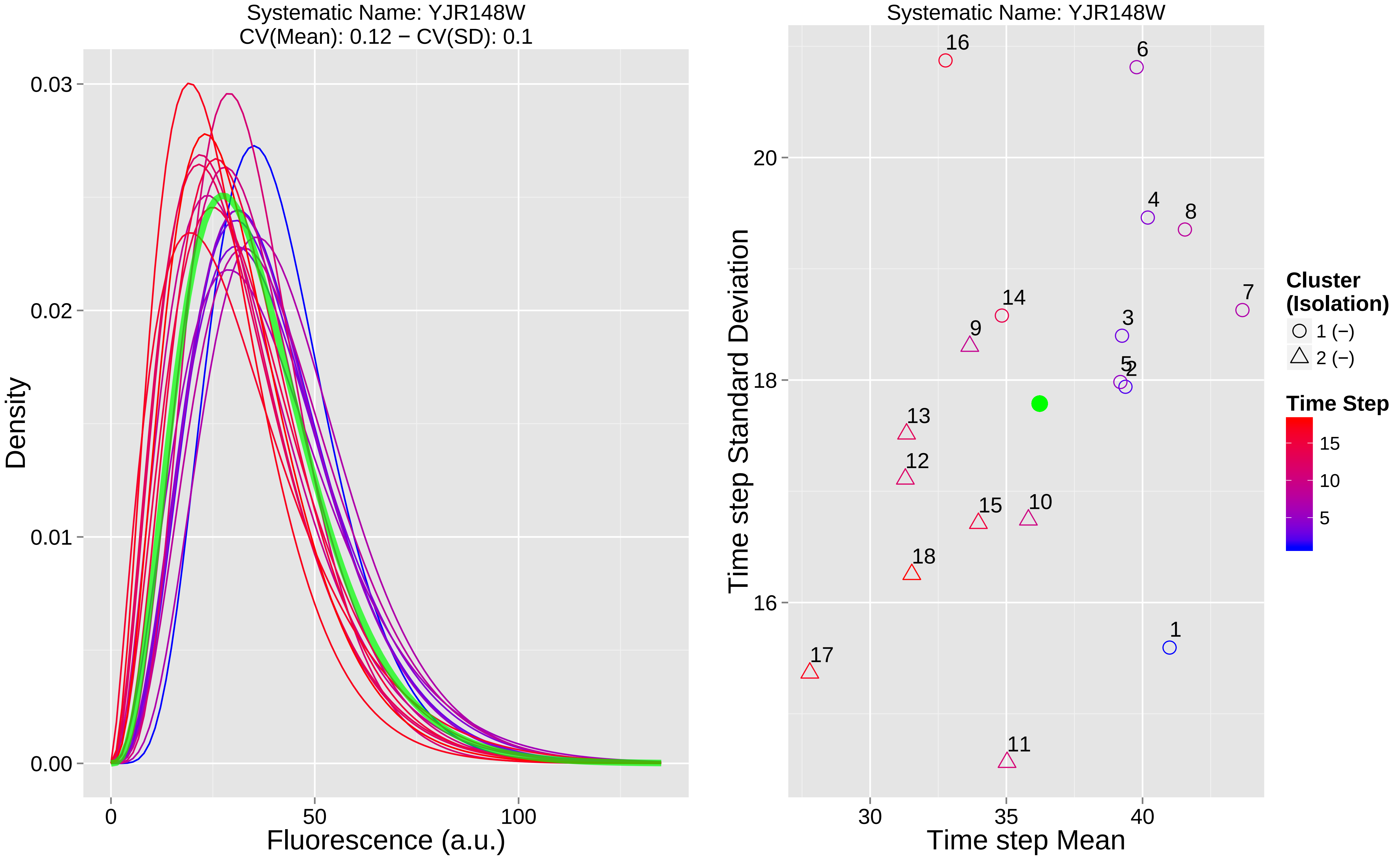

Systematic Name: YJR148W
CV(Mean): 0.12 − CV(SD): 0.1
Density
0.03
0.02
0.01
0.00
0
50
100
Fluorescence (a.u.)
Systematic Name: YJR148W
Time step Standard Deviation
20
18
16
30
35
40
Time step Mean
16
6
4
8
7
14
3
9
5
2
13
12
15
10
18
1
17
11
Cluster
(Isolation)
1 (−)
2 (−)
Time Step
15
10
5

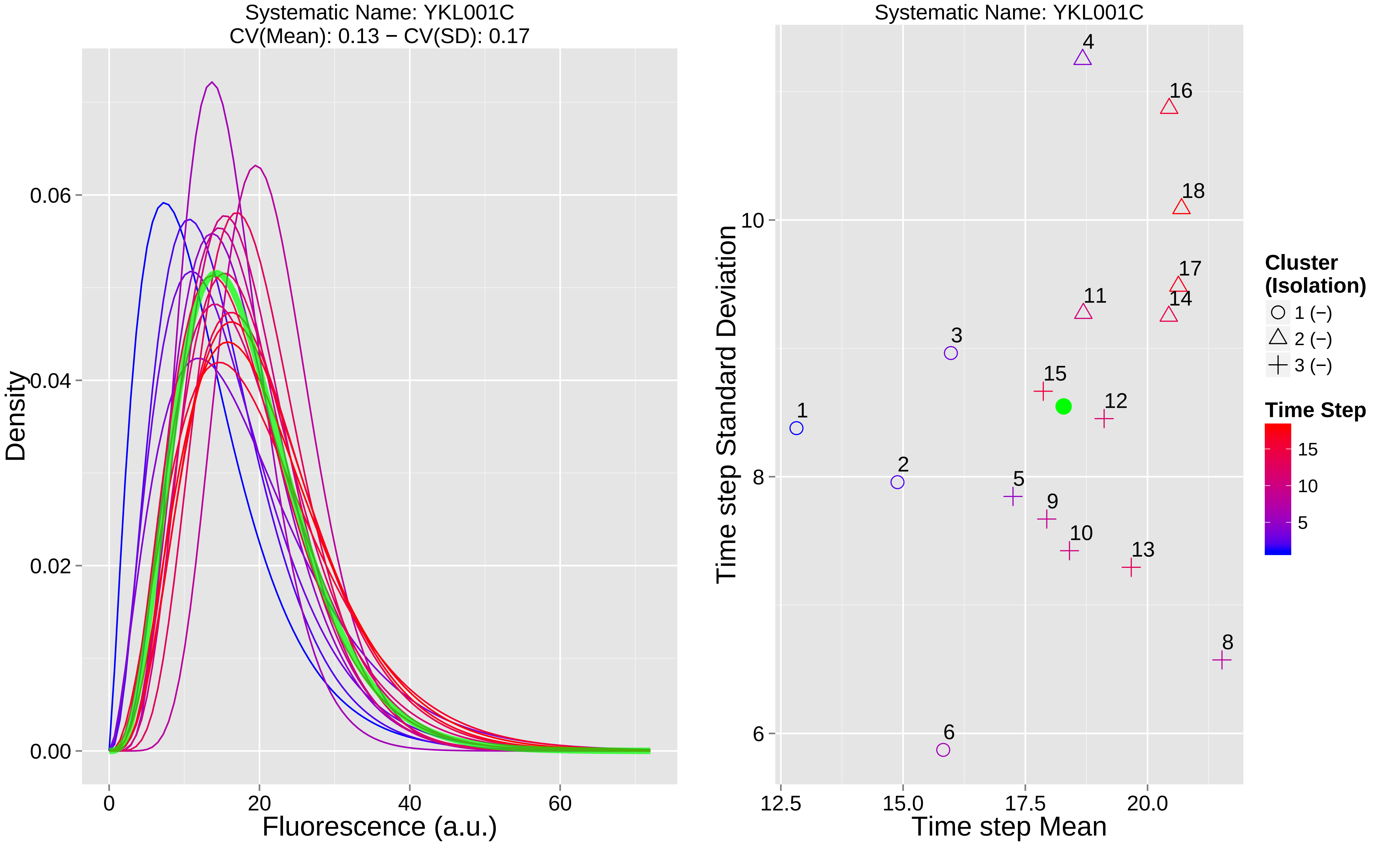

Systematic Name: YKL001C
CV(Mean): 0.13 − CV(SD): 0.17
Density
0.00
0.02
0.04
0.06
0
20
40
60
Fluorescence (a.u.)
Systematic Name: YKL001C
Time step Standard Deviation
6
8
10
12.5
15.0
17.5
20.0
Time step Mean
1
2
3
4
5
6
8
9
10
11
12
13
14
15
16
17
18
Cluster (Isolation)
1 (−)
2 (−)
3 (−)
Time Step
5
10
15

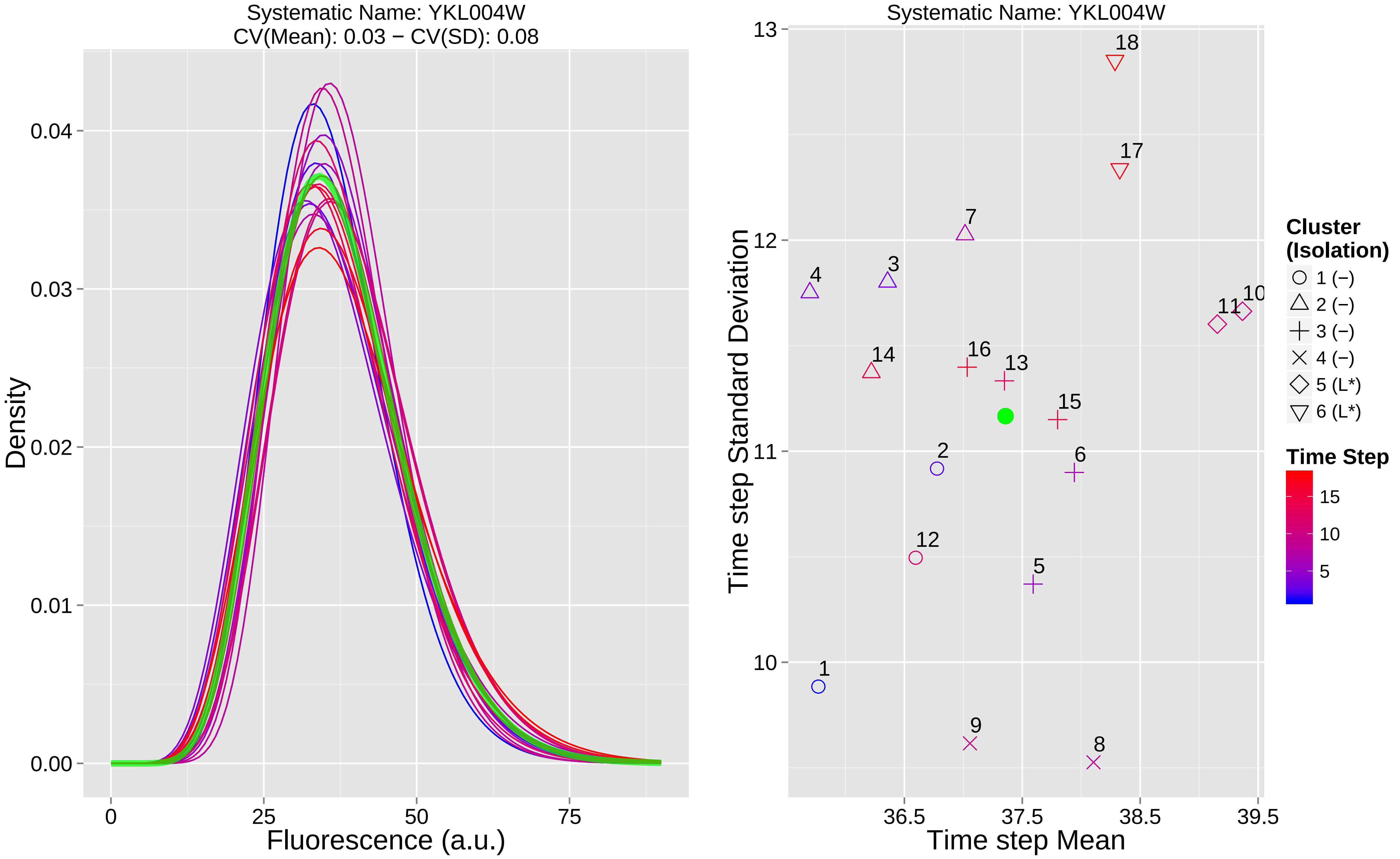

Systematic Name: YKL004W
CV(Mean): 0.03 − CV(SD): 0.08
Density
0.00
0.01
0.02
0.03
0.04
0
25
50
75
Fluorescence (a.u.)
Systematic Name: YKL004W
Time step Standard Deviation
Time step Mean
10
11
12
13
36.5
37.5
38.5
39.5
1
2
3
4
5
6
7
8
9
10
11
12
13
14
15
16
17
18
Cluster
(Isolation)
1 (−)
2 (−)
3 (−)
4 (−)
5 (L*)
6 (L*)
Time Step
15
10
5

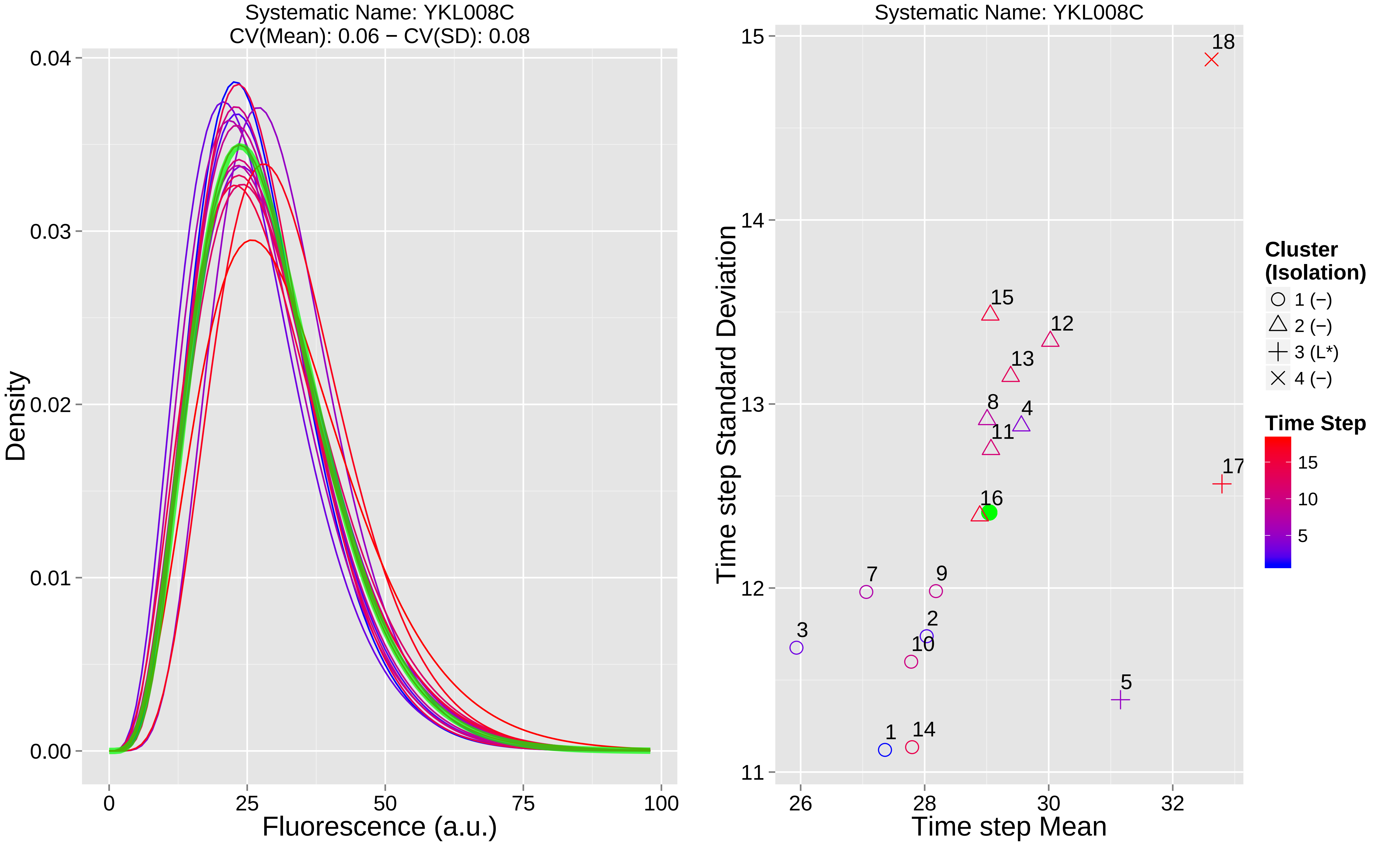

Systematic Name: YKL008C
CV(Mean): 0.06 − CV(SD): 0.08
Density
Fluorescence (a.u.)
0.00
0.01
0.02
0.03
0.04
0
25
50
75
100
Systematic Name: YKL008C
Time step Standard Deviation
Time step Mean
11
12
13
14
15
26
28
30
32
1
2
3
4
5
7
8
9
10
11
12
13
14
15
16
17
18
Cluster (Isolation)
1 (−)
2 (−)
3 (L*)
4 (−)
Time Step
5
10
15

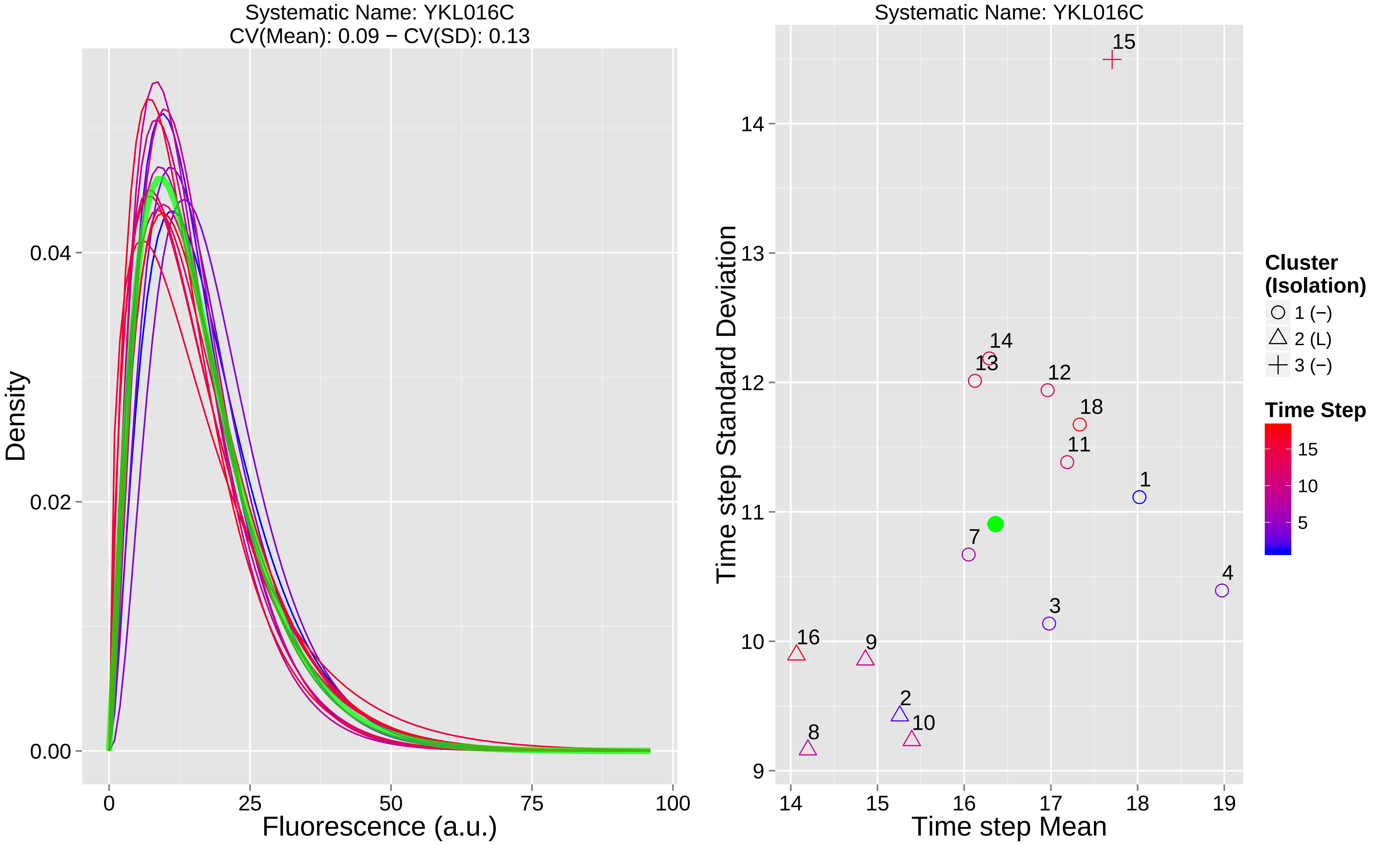

Systematic Name: YKL016C
CV(Mean): 0.09 − CV(SD): 0.13
Density
0.00
0.02
0.04
0
25
50
75
100
Fluorescence (a.u.)
Systematic Name: YKL016C
Time step Standard Deviation
9
10
11
12
13
14
14
15
16
17
18
19
Time step Mean
15
14
13
12
18
11
1
7
4
3
16
9
2
10
8
Cluster
(Isolation)
1 (−)
2 (L)
3 (−)
Time Step
15
10
5

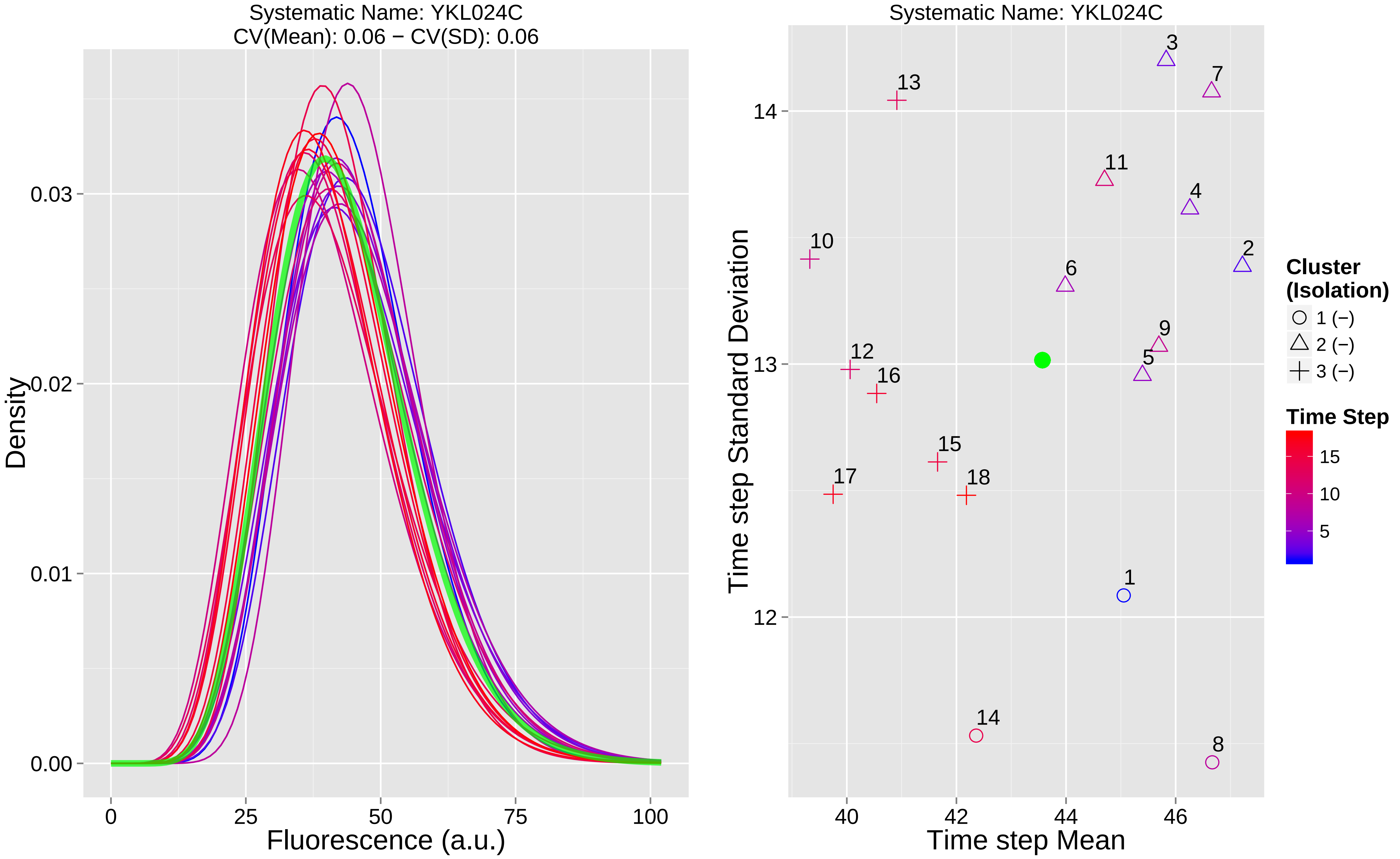

Systematic Name: YKL024C
CV(Mean): 0.06 − CV(SD): 0.06
Density
Fluorescence (a.u.)
0.00
0.01
0.02
0.03
0
25
50
75
100
Systematic Name: YKL024C
Time step Standard Deviation
Time step Mean
12
13
14
40
42
44
46
Cluster (Isolation)
1 (−)
2 (−)
3 (−)
Time Step
5
10
15

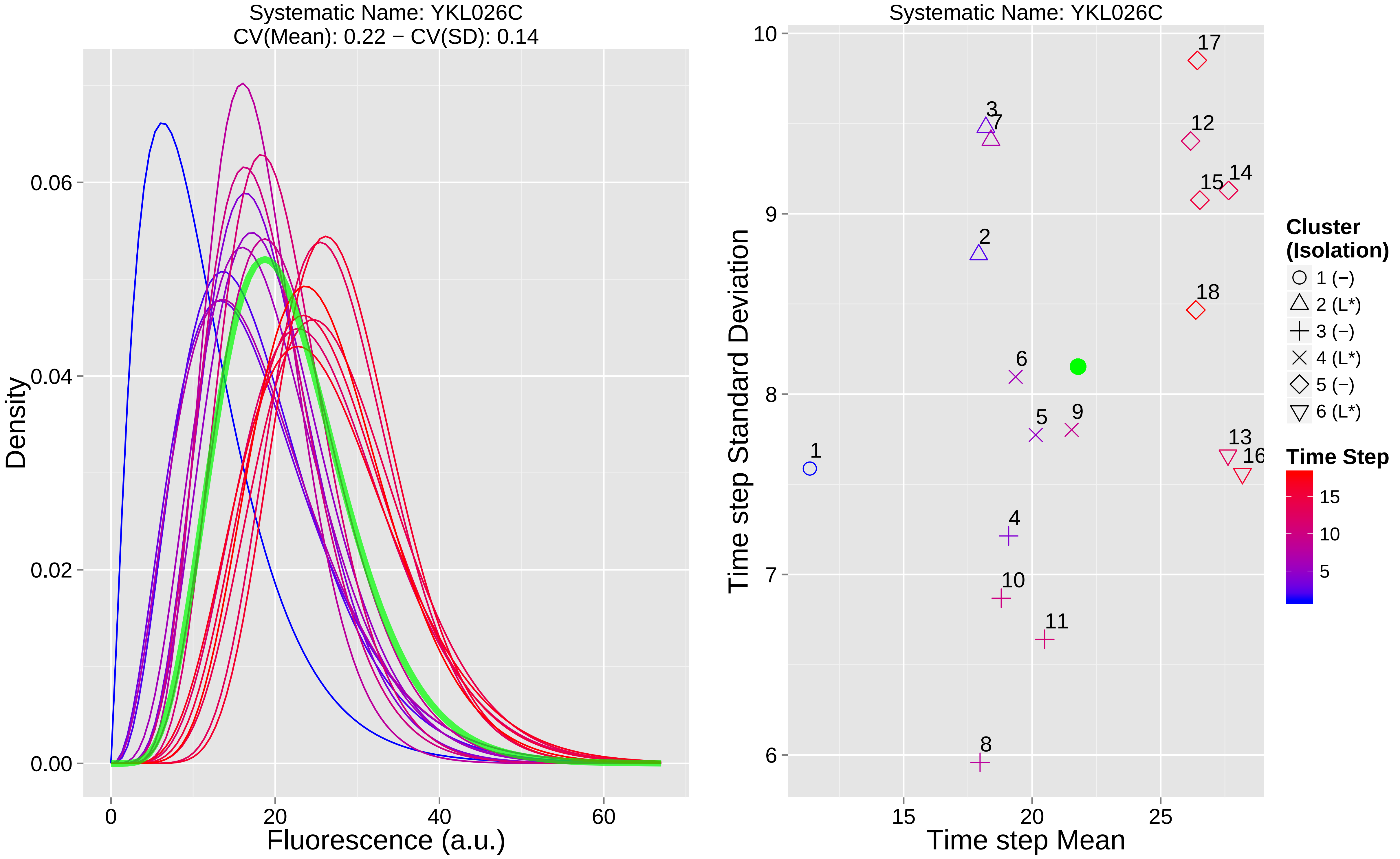

Systematic Name: YKL026C
CV(Mean): 0.22 − CV(SD): 0.14
Density
0.06
0.04
0.02
0.00
0
20
40
60
Fluorescence (a.u.)
Systematic Name: YKL026C
Time step Standard Deviation
10
9
8
7
6
15
20
25
Time step Mean
1
2
3
7
4
5
6
8
9
10
11
12
13
14
15
16
17
18
Cluster (Isolation)
1 (−)
2 (L*)
3 (−)
4 (L*)
5 (−)
6 (L*)
Time Step
15
10
5

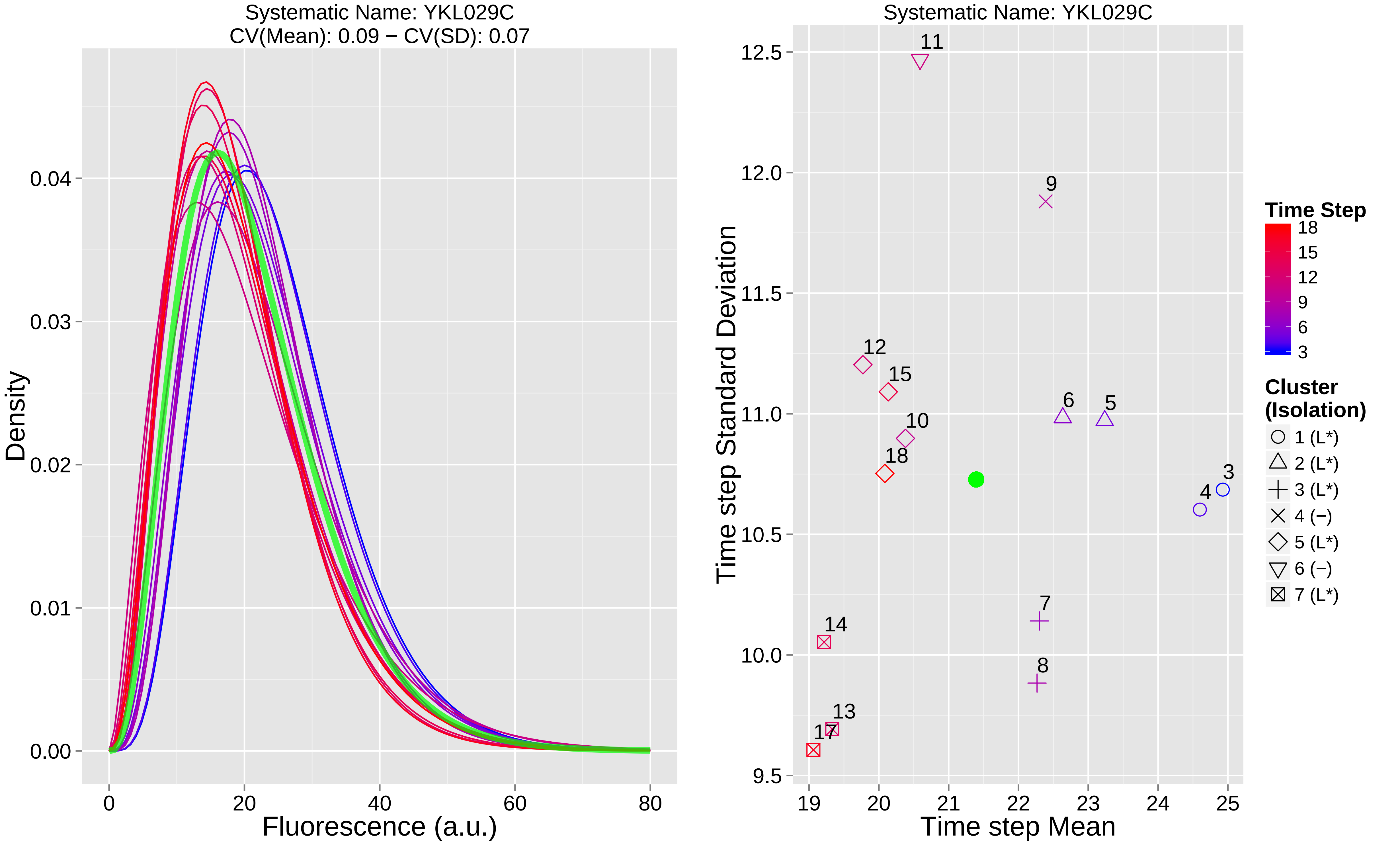

Systematic Name: YKL029C
CV(Mean): 0.09 − CV(SD): 0.07
Density
0.00
0.01
0.02
0.03
0.04
0
20
40
60
80
Fluorescence (a.u.)
Systematic Name: YKL029C
Time step Standard Deviation
9.5
10.0
10.5
11.0
11.5
12.0
12.5
19
20
21
22
23
24
25
Time step Mean
11
9
12
15
6
5
10
18
3
4
7
14
8
13
17
Time Step
18
15
12
9
6
3
Cluster (Isolation)
1 (L*)
2 (L*)
3 (L*)
4 (−)
5 (L*)
6 (−)
7 (L*)

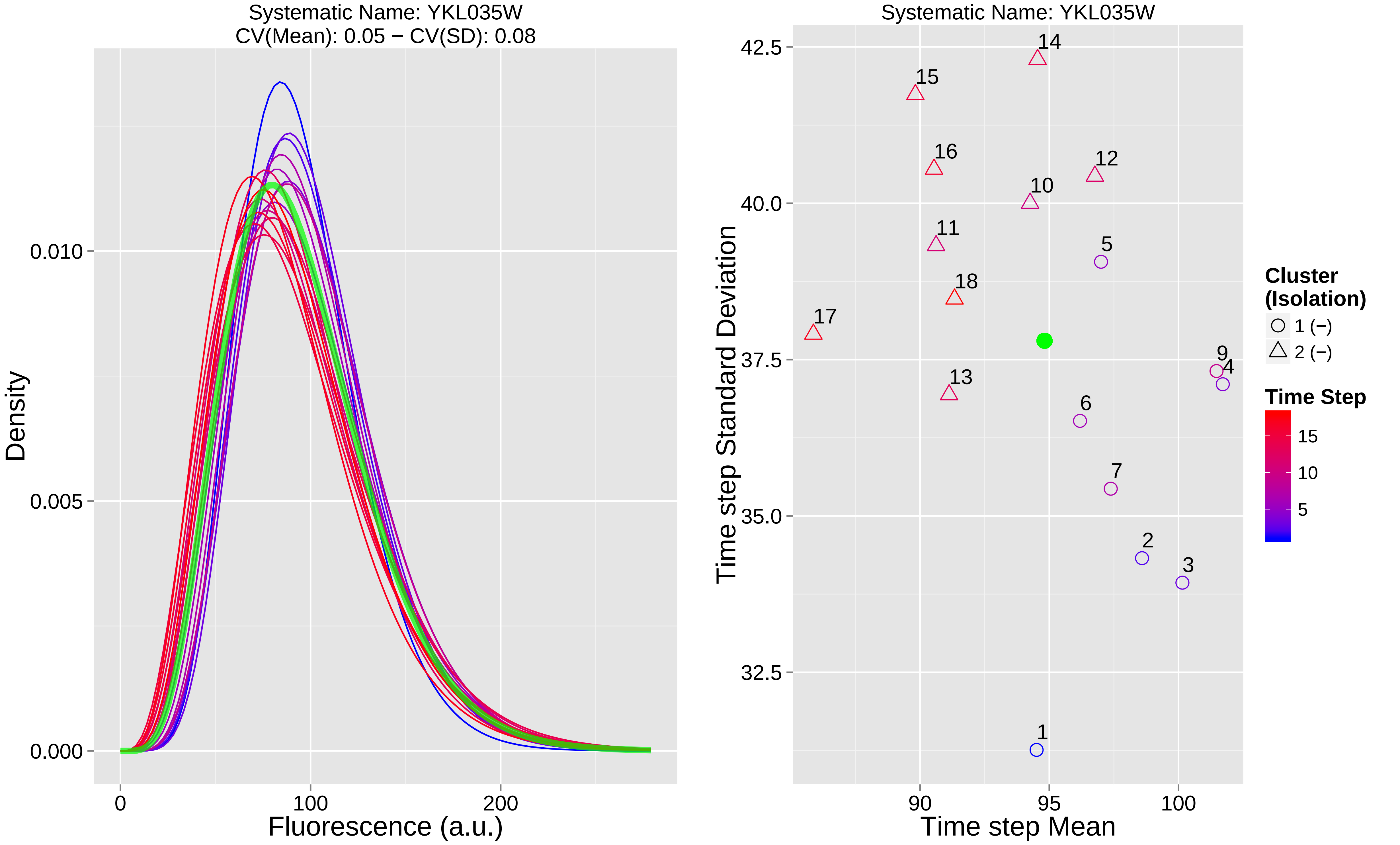

Systematic Name: YKL035W
CV(Mean): 0.05 − CV(SD): 0.08
Density
0.010
0.005
0.000
0
100
200
Fluorescence (a.u.)
Systematic Name: YKL035W
Time step Standard Deviation
42.5
40.0
37.5
35.0
32.5
90
95
100
Time step Mean
Cluster (Isolation)
1 (−)
2 (−)
Time Step
15
10
5

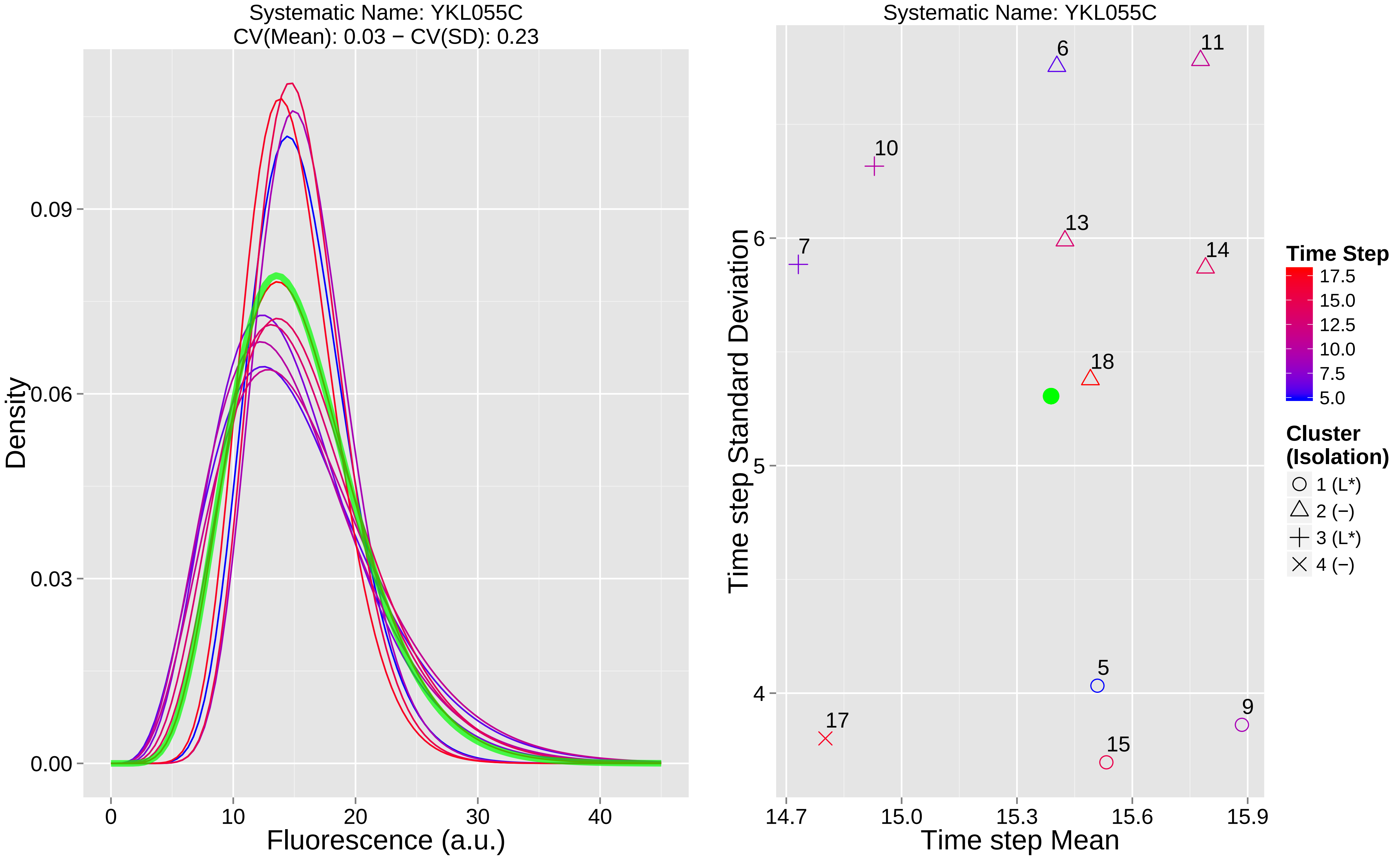

Systematic Name: YKL055C
CV(Mean): 0.03 − CV(SD): 0.23
Density
0.00
0.03
0.06
0.09
0
10
20
30
40
Fluorescence (a.u.)
Systematic Name: YKL055C
Time step Standard Deviation
4
5
6
14.7
15.0
15.3
15.6
15.9
Time step Mean
6
11
10
13
7
14
18
5
9
17
15
Time Step
17.5
15.0
12.5
10.0
7.5
5.0
Cluster
(Isolation)
1 (L*)
2 (−)
3 (L*)
4 (−)

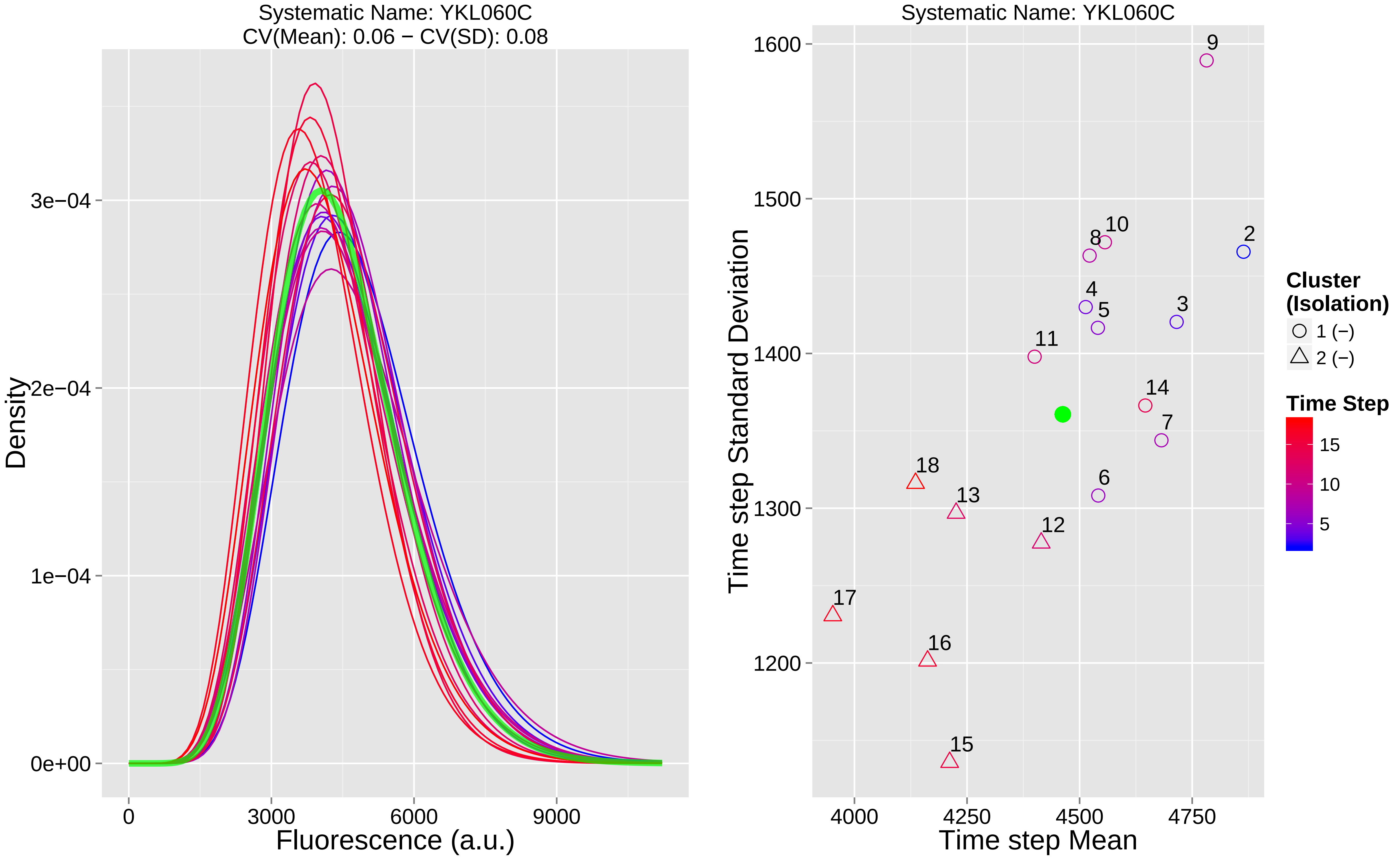

Systematic Name: YKL060C
CV(Mean): 0.06 − CV(SD): 0.08
Density
3e−04
2e−04
1e−04
0e+00
0
3000
6000
9000
Fluorescence (a.u.)
Systematic Name: YKL060C
Time step Standard Deviation
1600
1500
1400
1300
1200
4000
4250
4500
4750
Time step Mean
9
10
8
2
4
3
5
11
14
7
18
6
13
12
17
16
15
Cluster
(Isolation)
1 (−)
2 (−)
Time Step
15
10
5

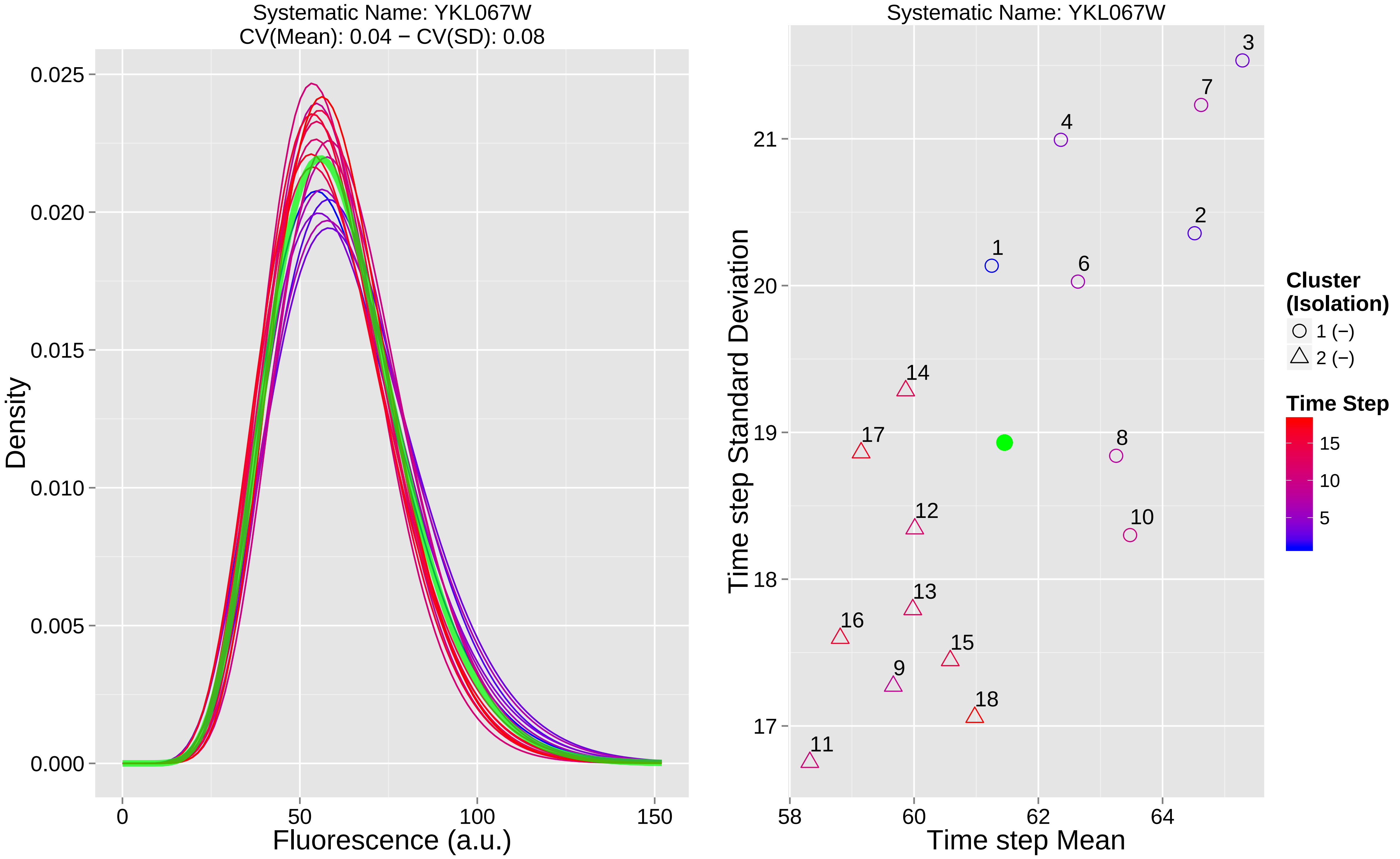

Systematic Name: YKL067W
CV(Mean): 0.04 − CV(SD): 0.08
Density
Fluorescence (a.u.)
0.025
0.020
0.015
0.010
0.005
0.000
0
50
100
150
Systematic Name: YKL067W
Time step Standard Deviation
Time step Mean
21
20
19
18
17
58
60
62
64
Cluster (Isolation)
1 (−)
2 (−)
Time Step
15
10
5

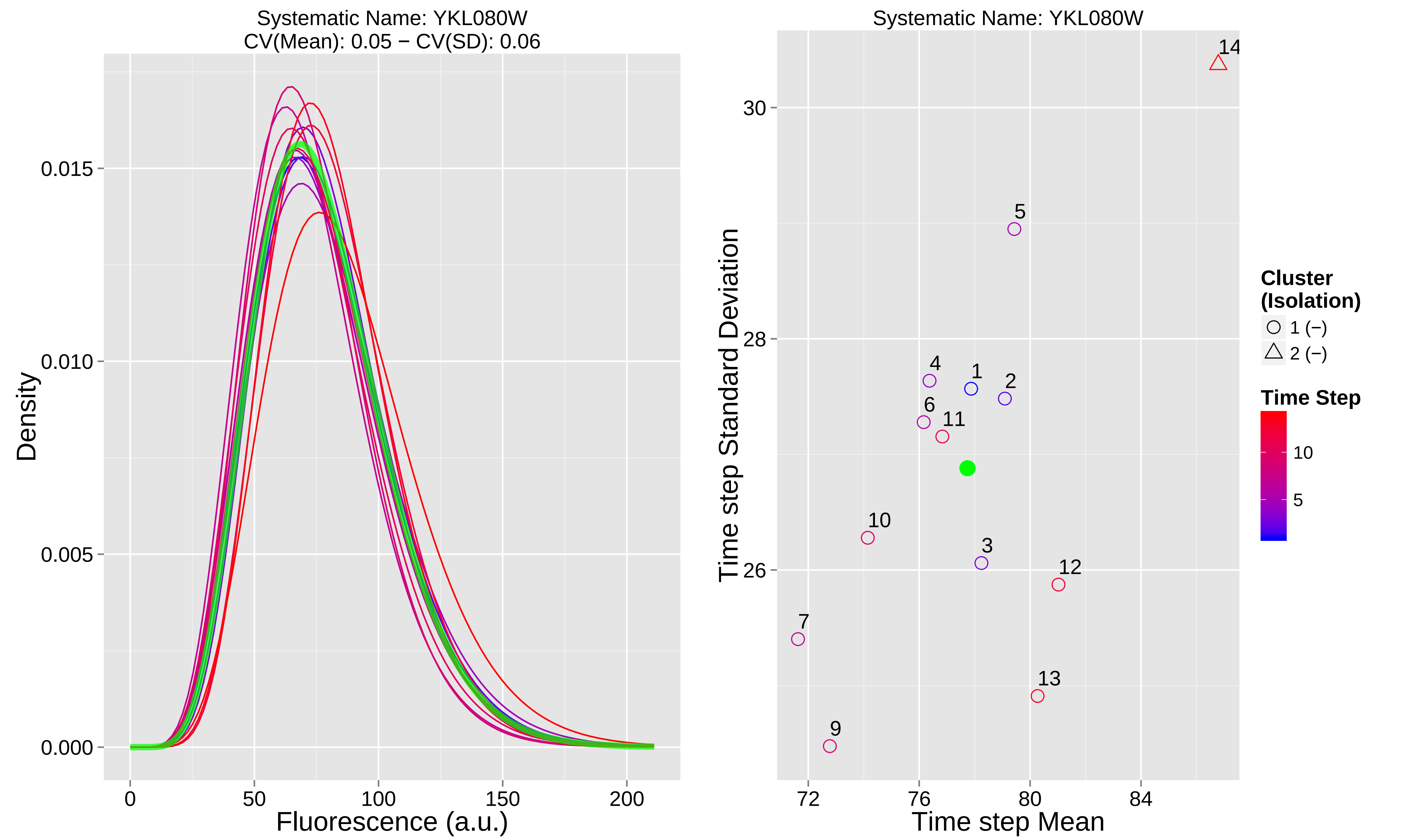

Systematic Name: YKL080W
CV(Mean): 0.05 − CV(SD): 0.06
Density
0.000
0.005
0.010
0.015
0
50
100
150
200
Fluorescence (a.u.)
Systematic Name: YKL080W
Time step Standard Deviation
26
28
30
72
76
80
84
Time step Mean
1
2
3
4
5
6
7
9
10
11
12
13
14
Cluster
(Isolation)
1 (−)
2 (−)
Time Step
5
10

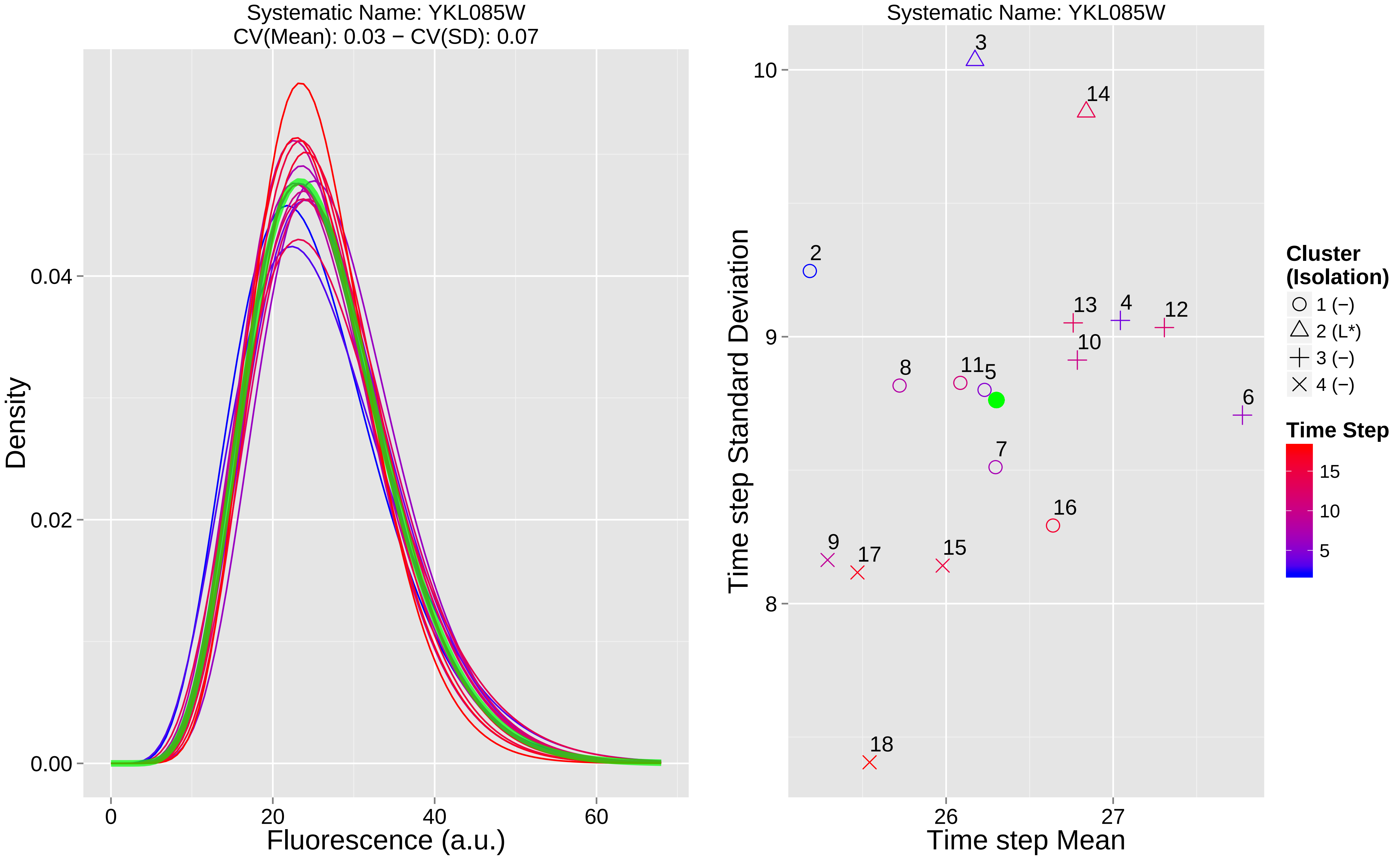
Systematic Name: YKL085W
CV(Mean): 0.03 − CV(SD): 0.07
Density
0.00
0.02
0.04
0
20
40
60
Fluorescence (a.u.)
Systematic Name: YKL085W
Time step Standard Deviation
8
9
10
26
27
Time step Mean
2
3
4
5
6
7
8
9
10
11
12
13
14
15
16
17
18
Cluster
(Isolation)
1 (−)
2 (L*)
3 (−)
4 (−)
Time Step
15
10
5

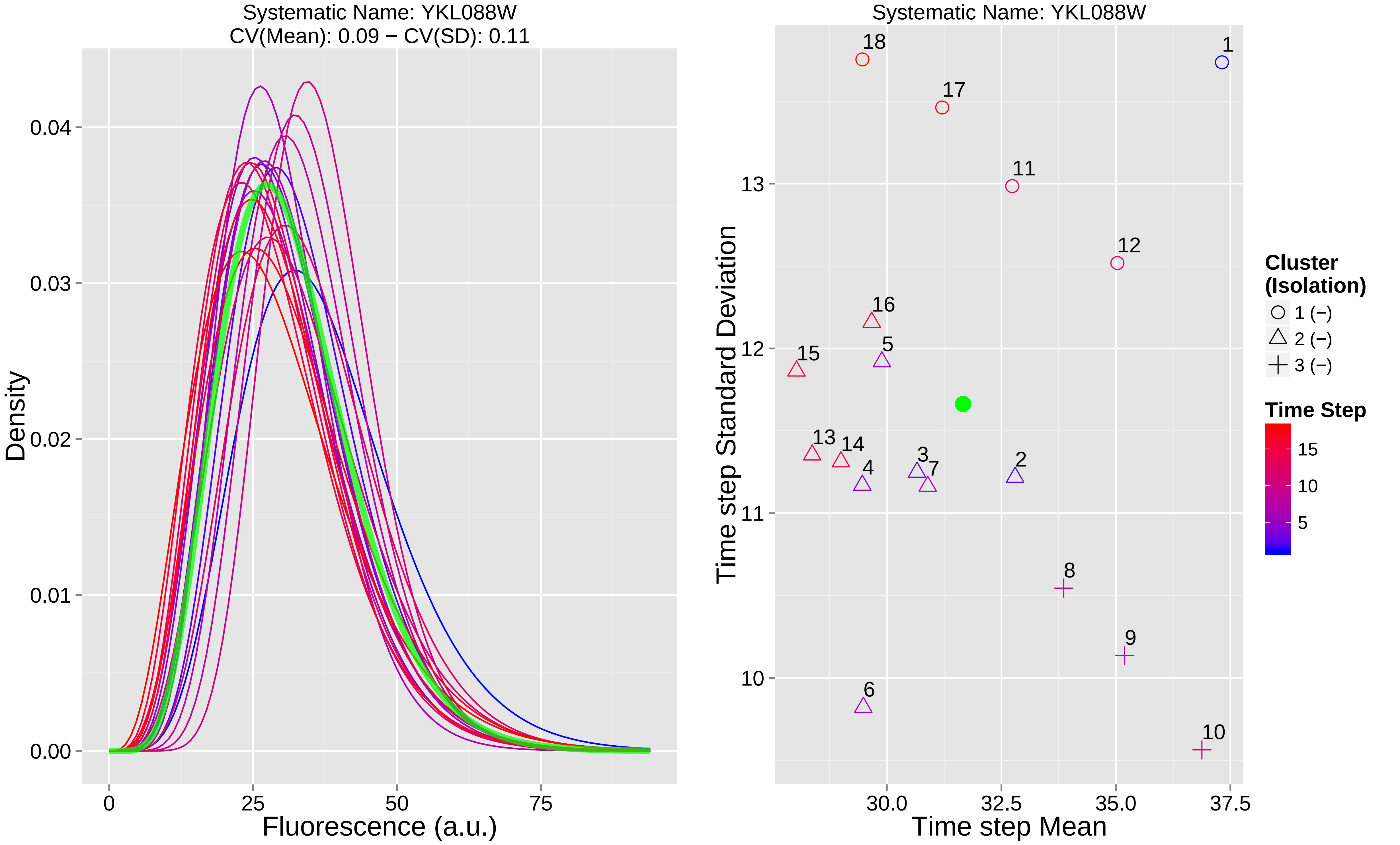

Systematic Name: YKL088W
CV(Mean): 0.09 − CV(SD): 0.11
Density
Fluorescence (a.u.)
0.00
0.01
0.02
0.03
0.04
0
25
50
75
Systematic Name: YKL088W
Time step Standard Deviation
Time step Mean
10
11
12
13
30.0
32.5
35.0
37.5
Cluster (Isolation)
1 (−)
2 (−)
3 (−)
Time Step
5
10
15

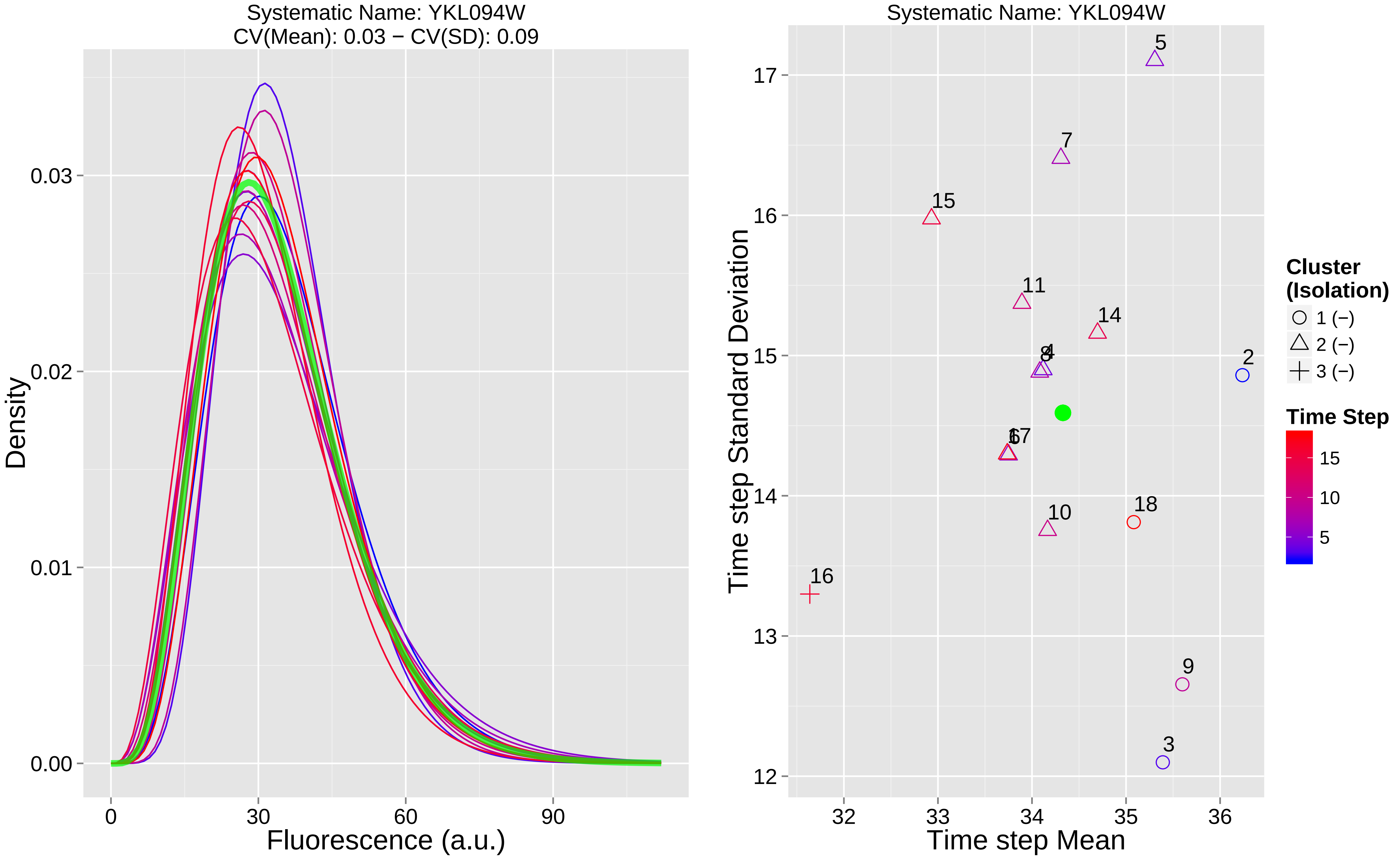

Systematic Name: YKL094W
CV(Mean): 0.03 − CV(SD): 0.09
Density
Fluorescence (a.u.)
Systematic Name: YKL094W
Time step Standard Deviation
Time step Mean
Cluster (Isolation)
1 (−)
2 (−)
3 (−)
Time Step

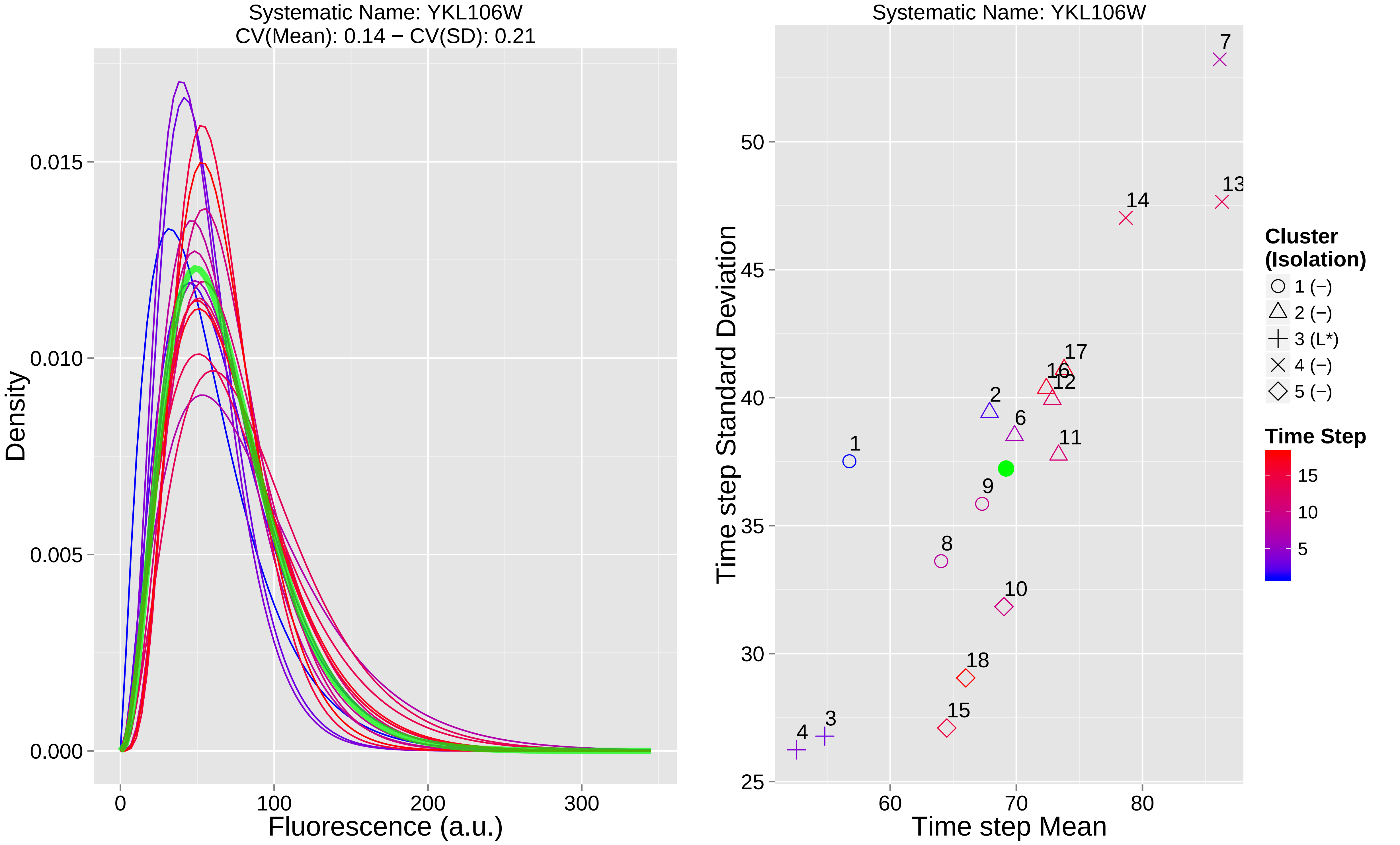

Systematic Name: YKL106W
CV(Mean): 0.14 − CV(SD): 0.21
Density
Fluorescence (a.u.)
0.000
0.005
0.010
0.015
0
100
200
300
Systematic Name: YKL106W
Time step Standard Deviation
Time step Mean
25
30
35
40
45
50
60
70
80
1
2
3
4
6
7
8
9
10
11
12
13
14
15
16
17
18
Cluster (Isolation)
1 (−)
2 (−)
3 (L*)
4 (−)
5 (−)
Time Step
5
10
15

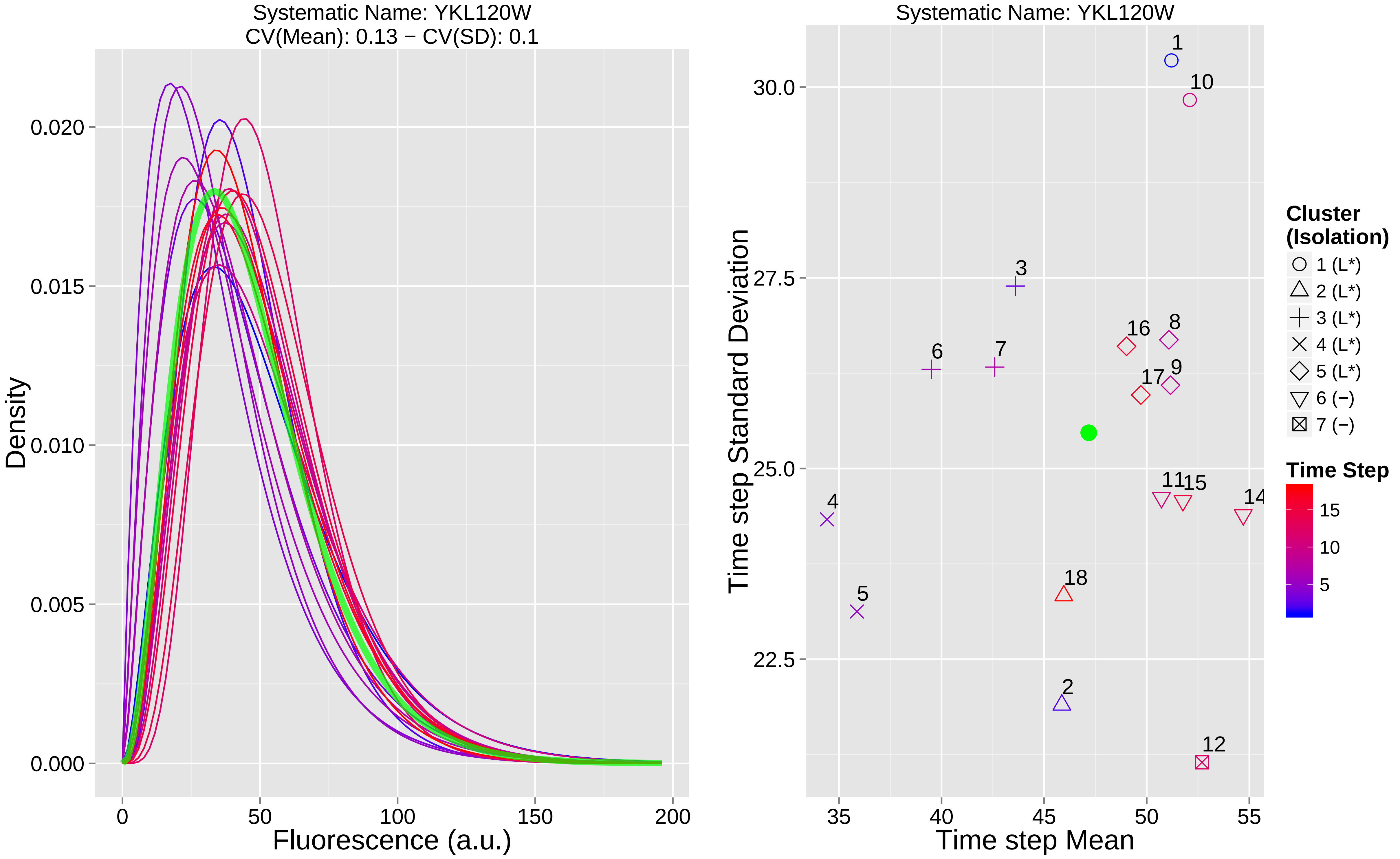

Systematic Name: YKL120W
CV(Mean): 0.13 − CV(SD): 0.1
Density
0.000
0.005
0.010
0.015
0.020
0
50
100
150
200
Fluorescence (a.u.)
Systematic Name: YKL120W
Time step Standard Deviation
22.5
25.0
27.5
30.0
35
40
45
50
55
Time step Mean
1
2
3
4
5
6
7
8
9
10
11
12
14
15
16
17
18
Cluster (Isolation)
1 (L*)
2 (L*)
3 (L*)
4 (L*)
5 (L*)
6 (−)
7 (−)
Time Step
5
10
15

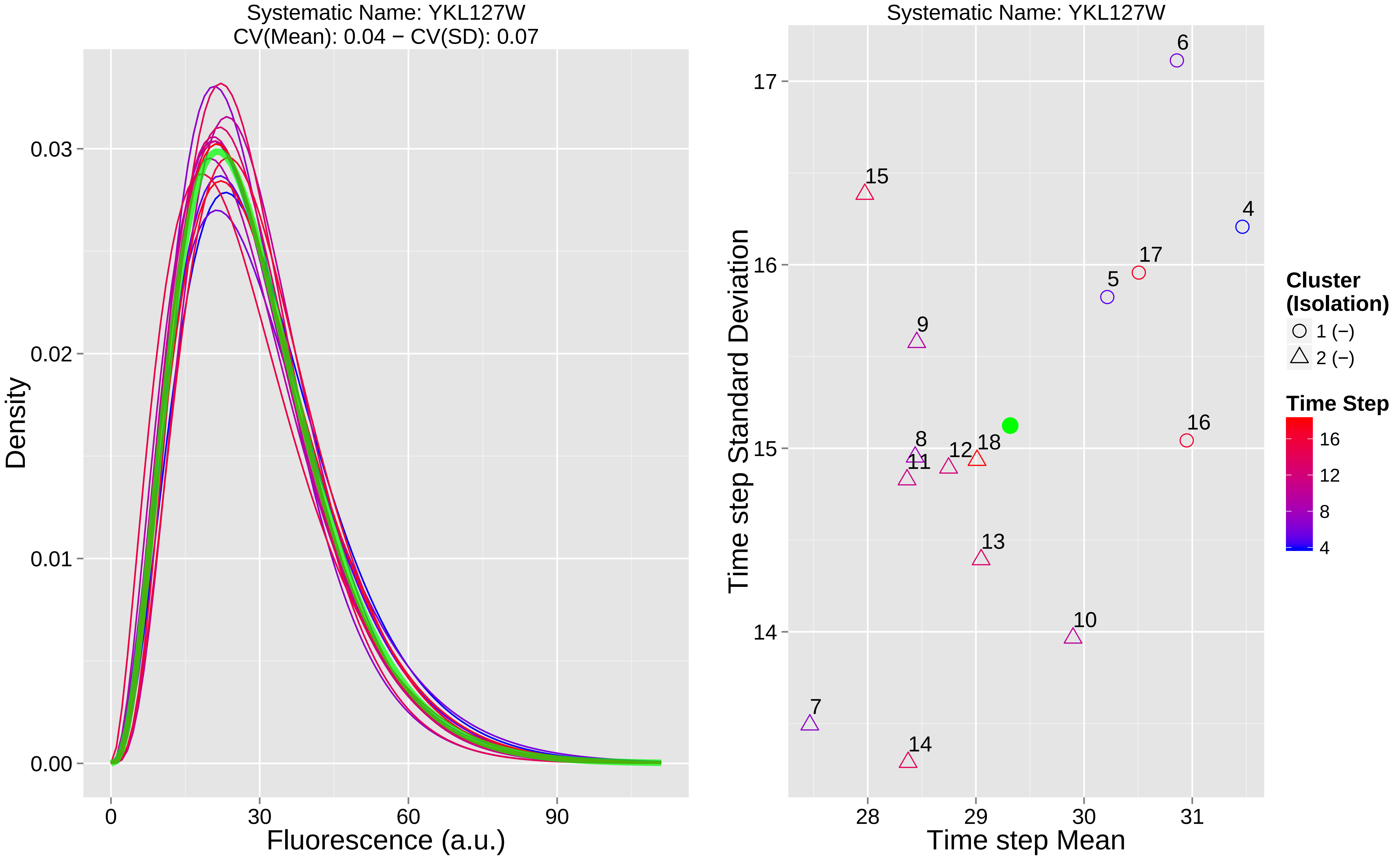
Systematic Name: YKL127W
CV(Mean): 0.04 − CV(SD): 0.07
Density
Fluorescence (a.u.)
0.00
0.01
0.02
0.03
0
30
60
90
Systematic Name: YKL127W
Time step Standard Deviation
Time step Mean
14
15
16
17
28
29
30
31
Cluster (Isolation)
1 (−)
2 (−)
Time Step
4
8
12
16

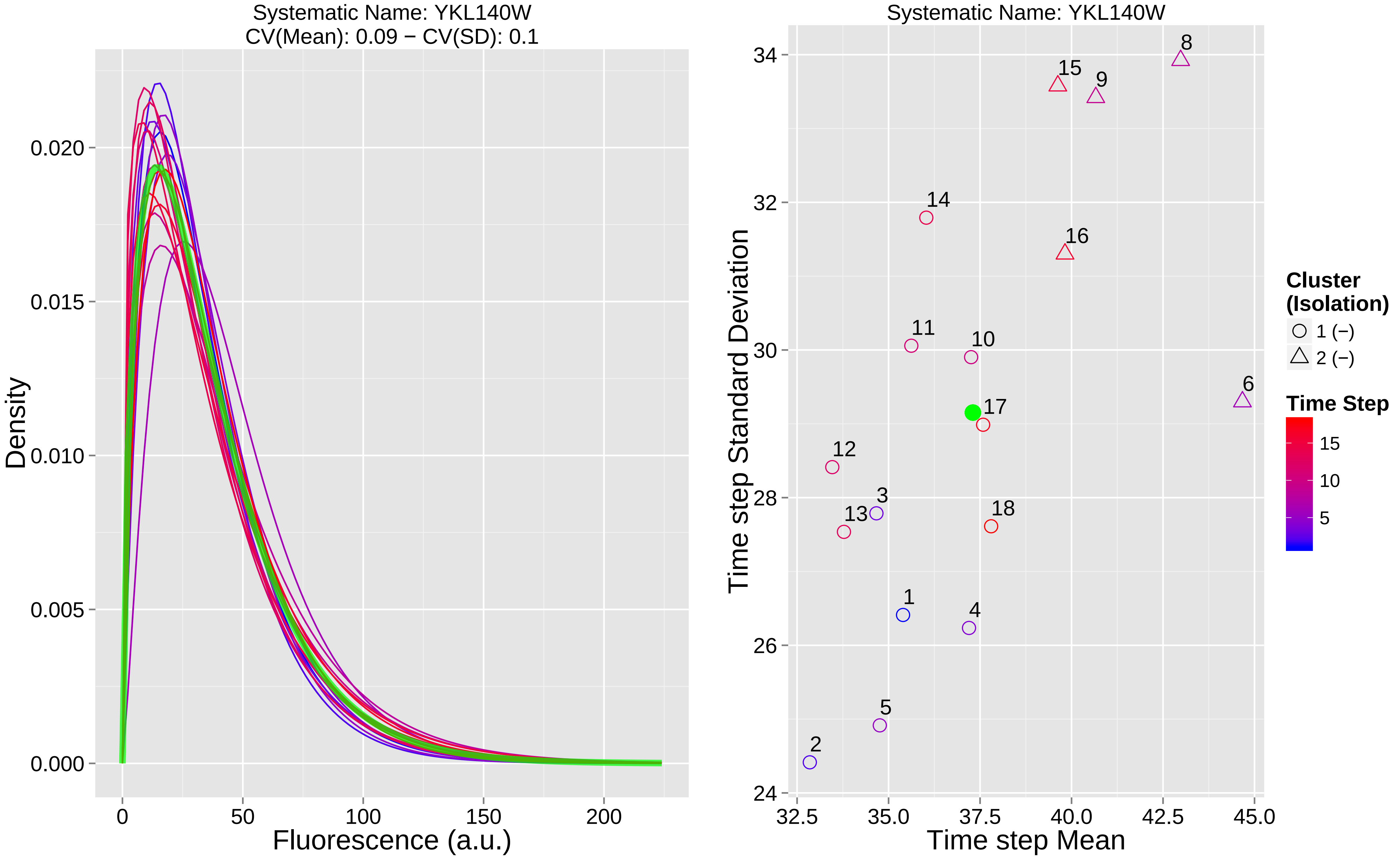
Systematic Name: YKL140W
CV(Mean): 0.09 − CV(SD): 0.1
Density
0.000
0.005
0.010
0.015
0.020
0
50
100
150
200
Fluorescence (a.u.)
Systematic Name: YKL140W
Time step Standard Deviation
24
26
28
30
32
34
32.5
35.0
37.5
40.0
42.5
45.0
Time step Mean
Cluster
(Isolation)
1 (−)
2 (−)
Time Step
5
10
15

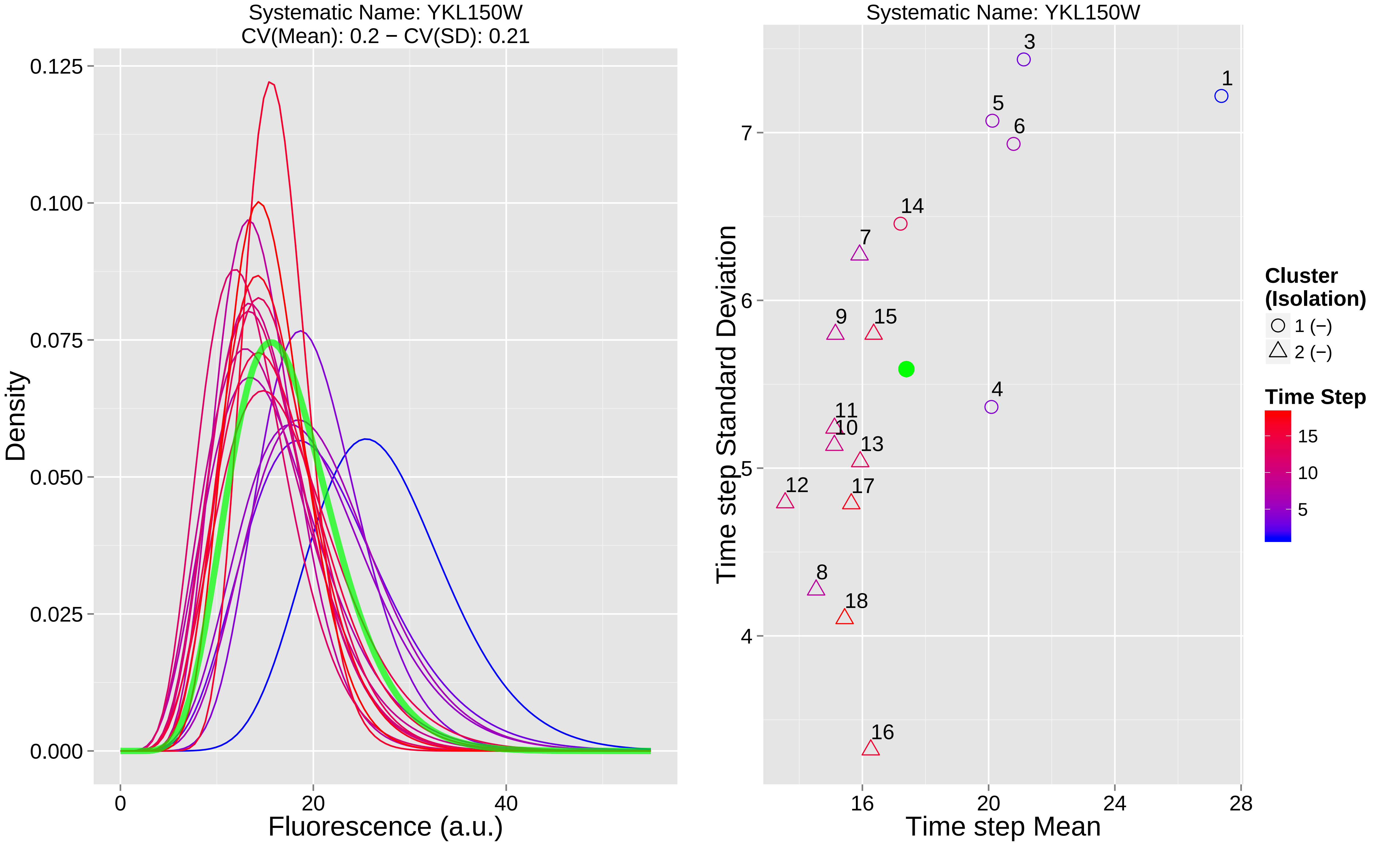

Systematic Name: YKL150W
CV(Mean): 0.2 − CV(SD): 0.21
Density
0.125
0.100
0.075
0.050
0.025
0.000
0
20
40
Fluorescence (a.u.)
Systematic Name: YKL150W
Time step Standard Deviation
7
6
5
4
16
20
24
28
Time step Mean
1
2
3
4
5
6
7
8
9
10
11
12
13
14
15
16
17
18
Cluster (Isolation)
1 (−)
2 (−)
Time Step
15
10
5

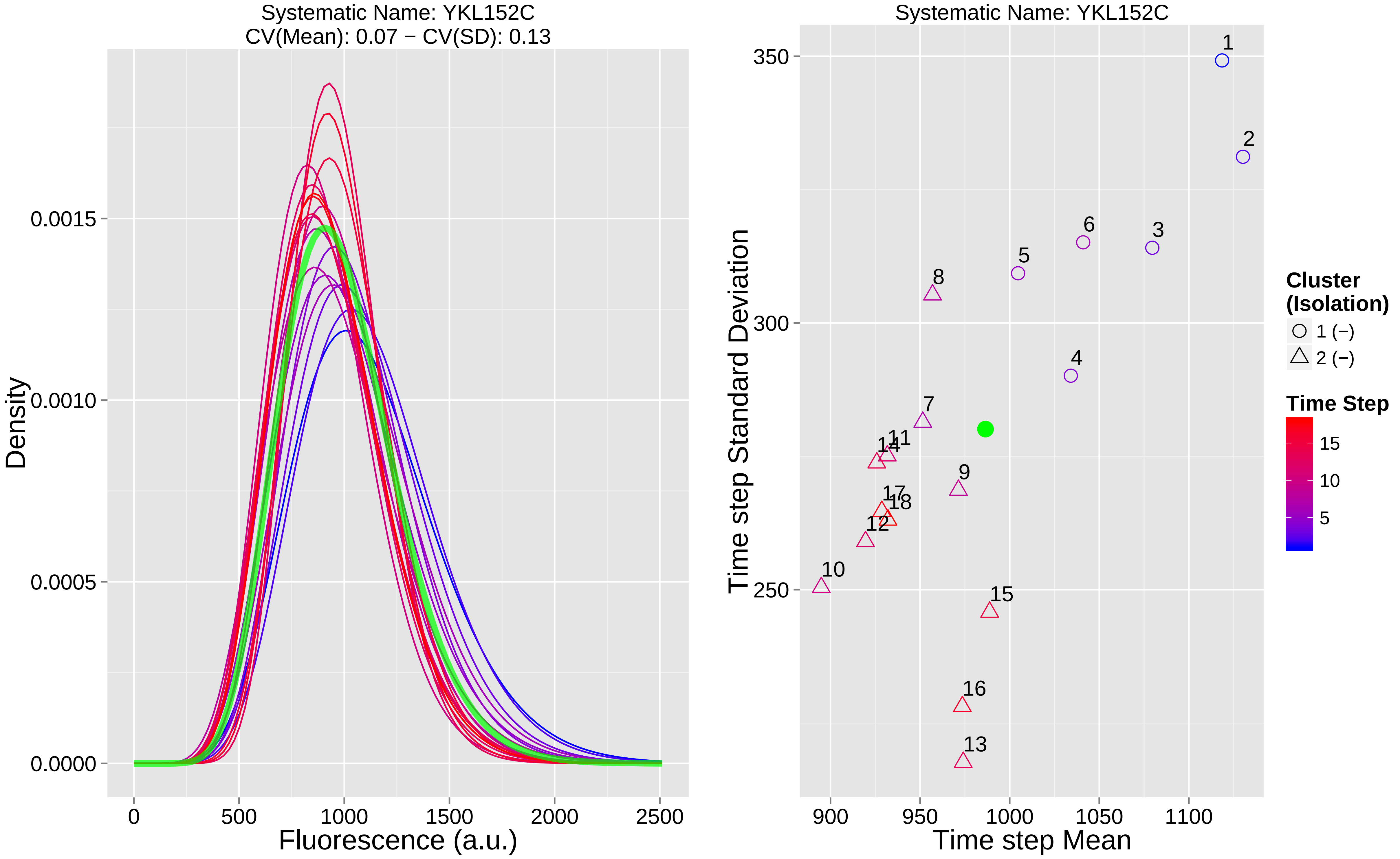
Systematic Name: YKL152C
CV(Mean): 0.07 − CV(SD): 0.13
Density
0.0015
0.0010
0.0005
0.0000
0
500
1000
1500
2000
2500
Fluorescence (a.u.)
Systematic Name: YKL152C
Time step Standard Deviation
350
300
250
900
950
1000
1050
1100
Time step Mean
1
2
3
4
5
6
7
8
9
10
11
12
13
14
15
16
17
18
Cluster
(Isolation)
1 (−)
2 (−)
Time Step
15
10
5

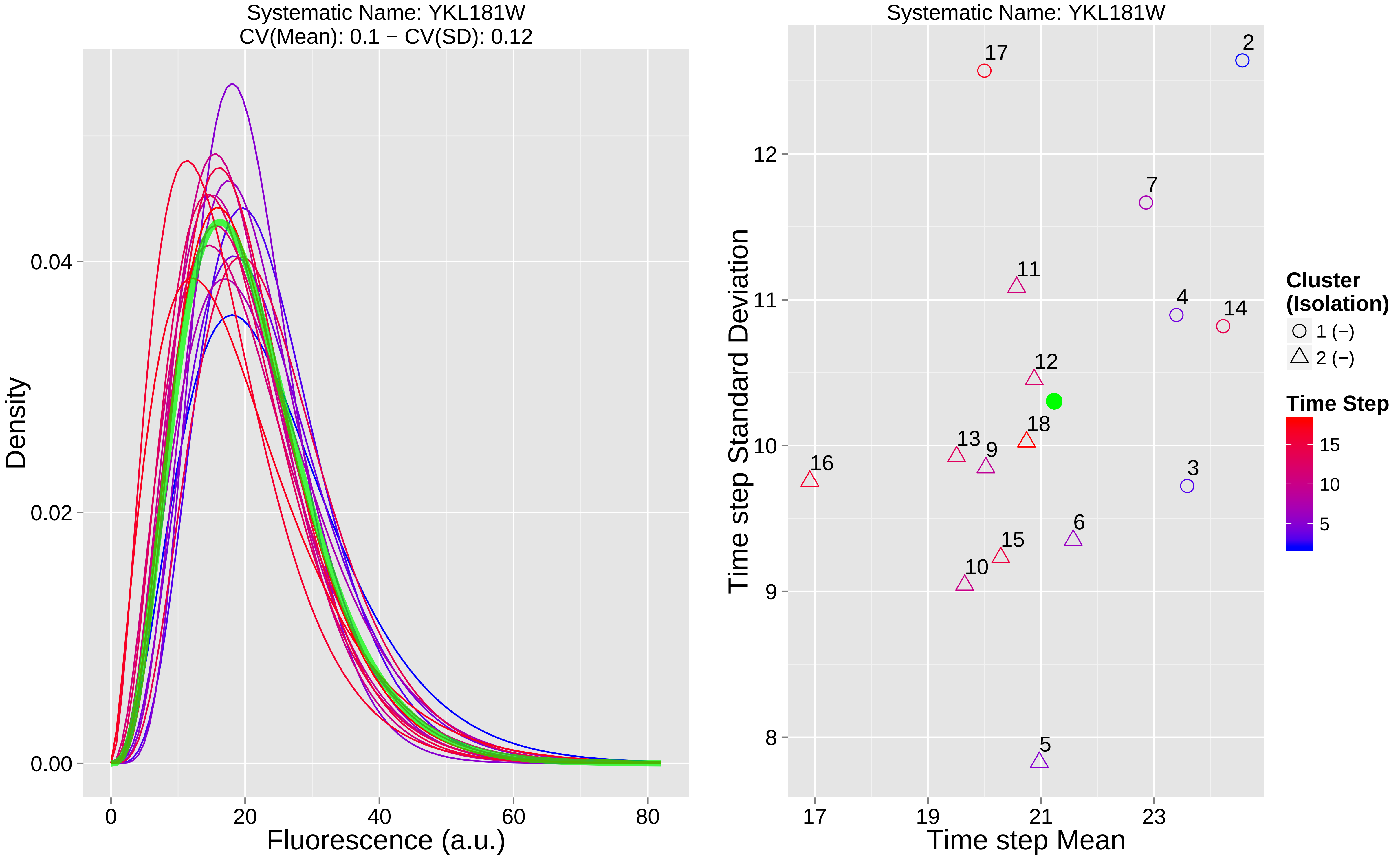

Systematic Name: YKL181W
CV(Mean): 0.1 − CV(SD): 0.12
Density
Fluorescence (a.u.)
0.00
0.02
0.04
0
20
40
60
80
Systematic Name: YKL181W
Time step Standard Deviation
Time step Mean
8
9
10
11
12
17
19
21
23
2
3
4
5
6
7
9
10
11
12
13
14
15
16
17
18
Cluster
(Isolation)
1 (−)
2 (−)
Time Step
5
10
15

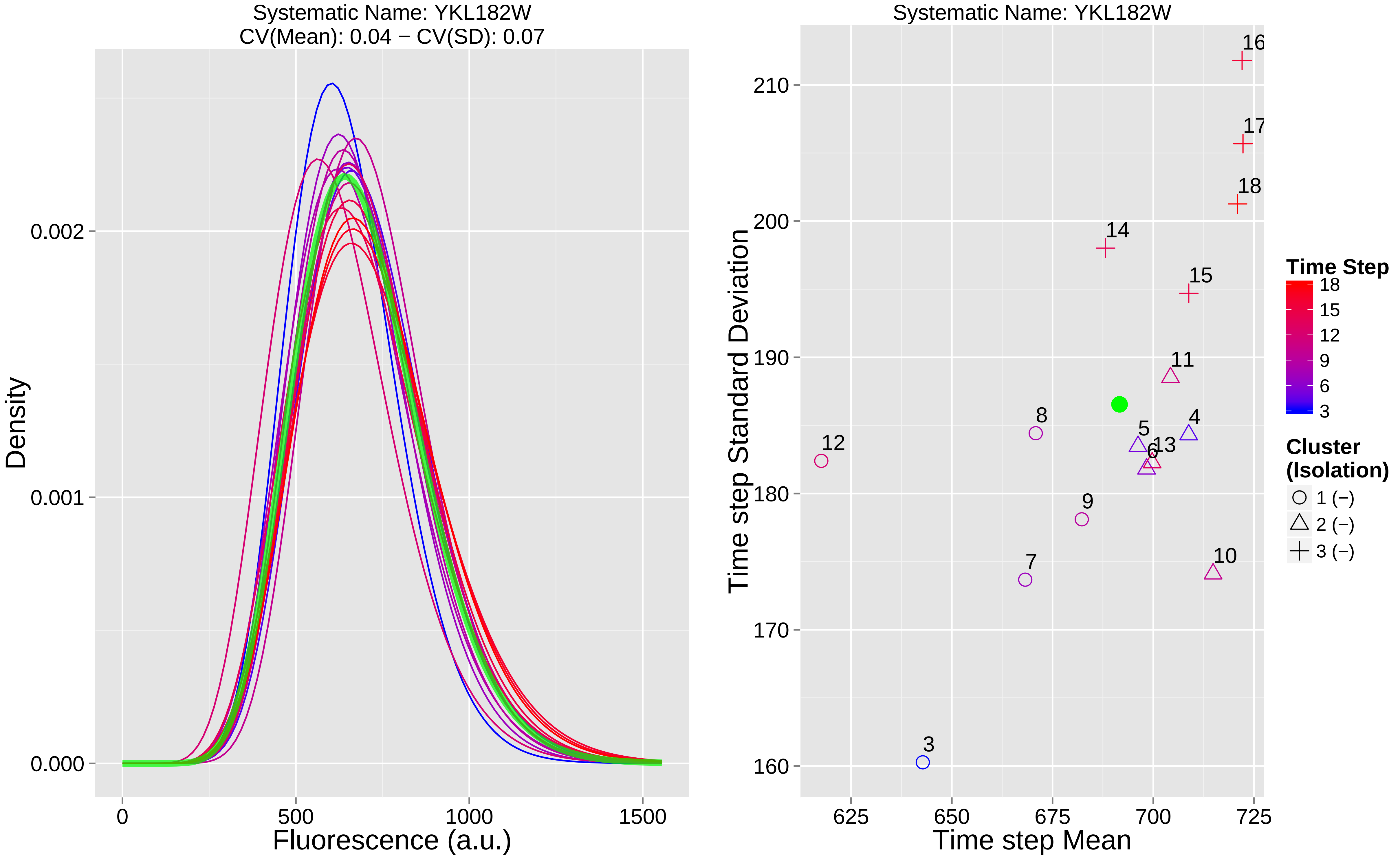
Systematic Name: YKL182W
CV(Mean): 0.04 − CV(SD): 0.07
Density
0.002
0.001
0.000
0
500
1000
1500
Fluorescence (a.u.)
Systematic Name: YKL182W
Time step Standard Deviation
210
200
190
180
170
160
625
650
675
700
725
Time step Mean
16
17
18
14
15
11
8
5
4
6
13
12
9
7
10
3
Time Step
18
15
12
9
6
3
Cluster
(Isolation)
1 (−)
2 (−)
3 (−)

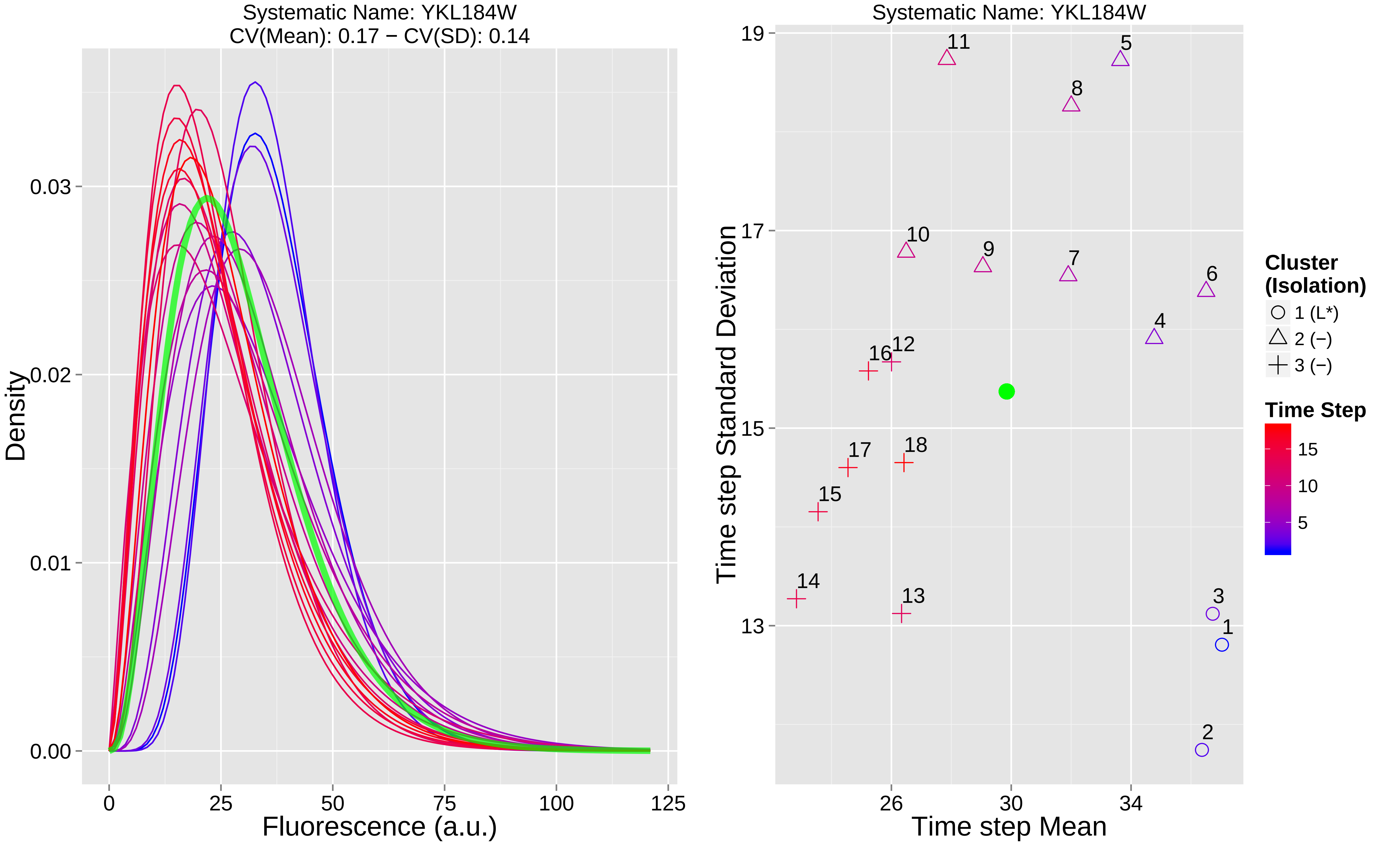

Systematic Name: YKL184W
CV(Mean): 0.17 − CV(SD): 0.14
Density
Fluorescence (a.u.)
0.00
0.01
0.02
0.03
0
25
50
75
100
125
Systematic Name: YKL184W
Time step Standard Deviation
Time step Mean
13
15
17
19
26
30
34
Cluster (Isolation)
1 (L*)
2 (−)
3 (−)
Time Step
5
10
15

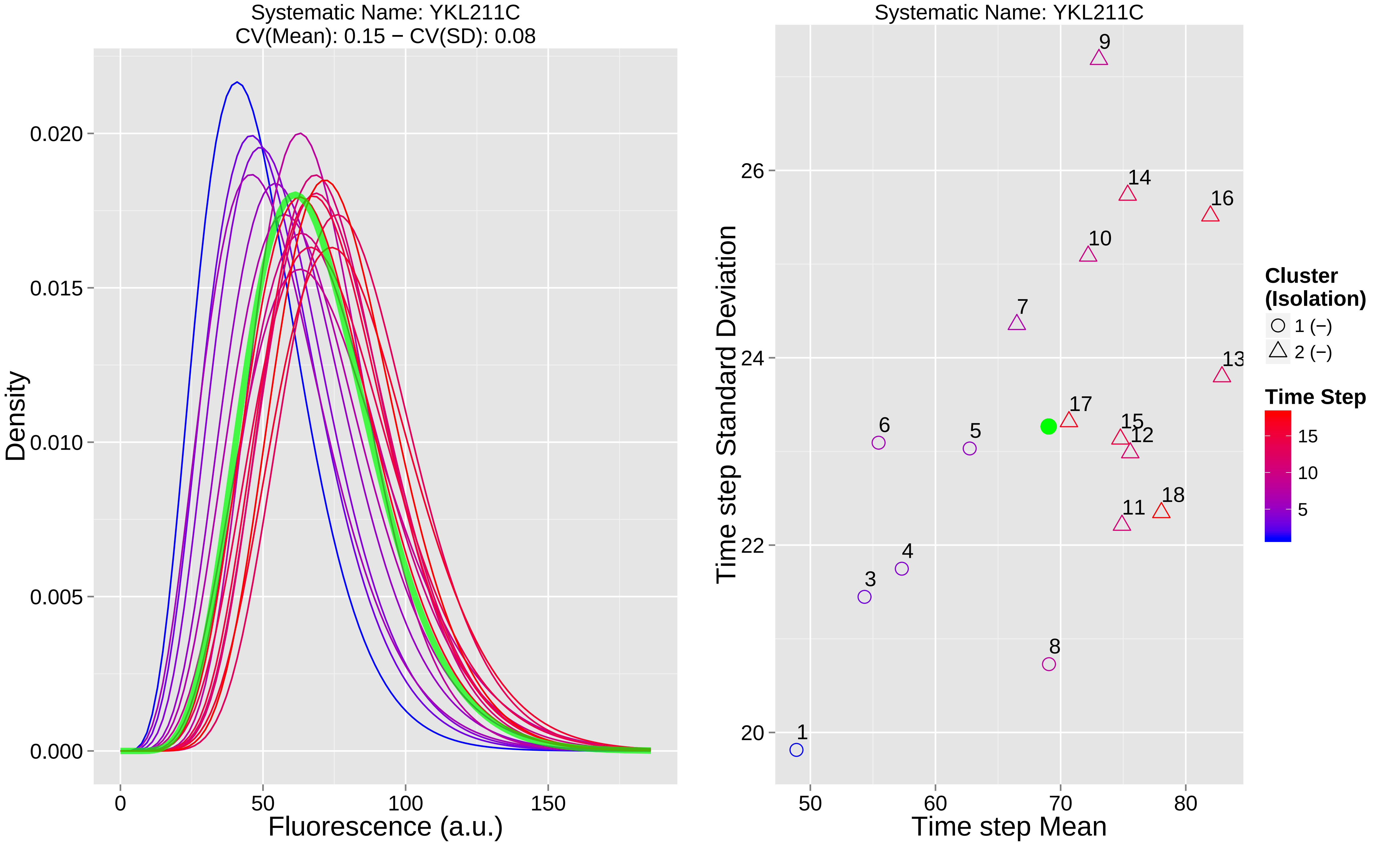

Systematic Name: YKL211C
CV(Mean): 0.15 − CV(SD): 0.08
Density
0.000
0.005
0.010
0.015
0.020
0
50
100
150
Fluorescence (a.u.)
Systematic Name: YKL211C
Time step Standard Deviation
20
22
24
26
50
60
70
80
Time step Mean
1
3
4
5
6
7
8
9
10
11
12
13
14
15
16
17
18
Cluster
(Isolation)
1 (−)
2 (−)
Time Step
5
10
15

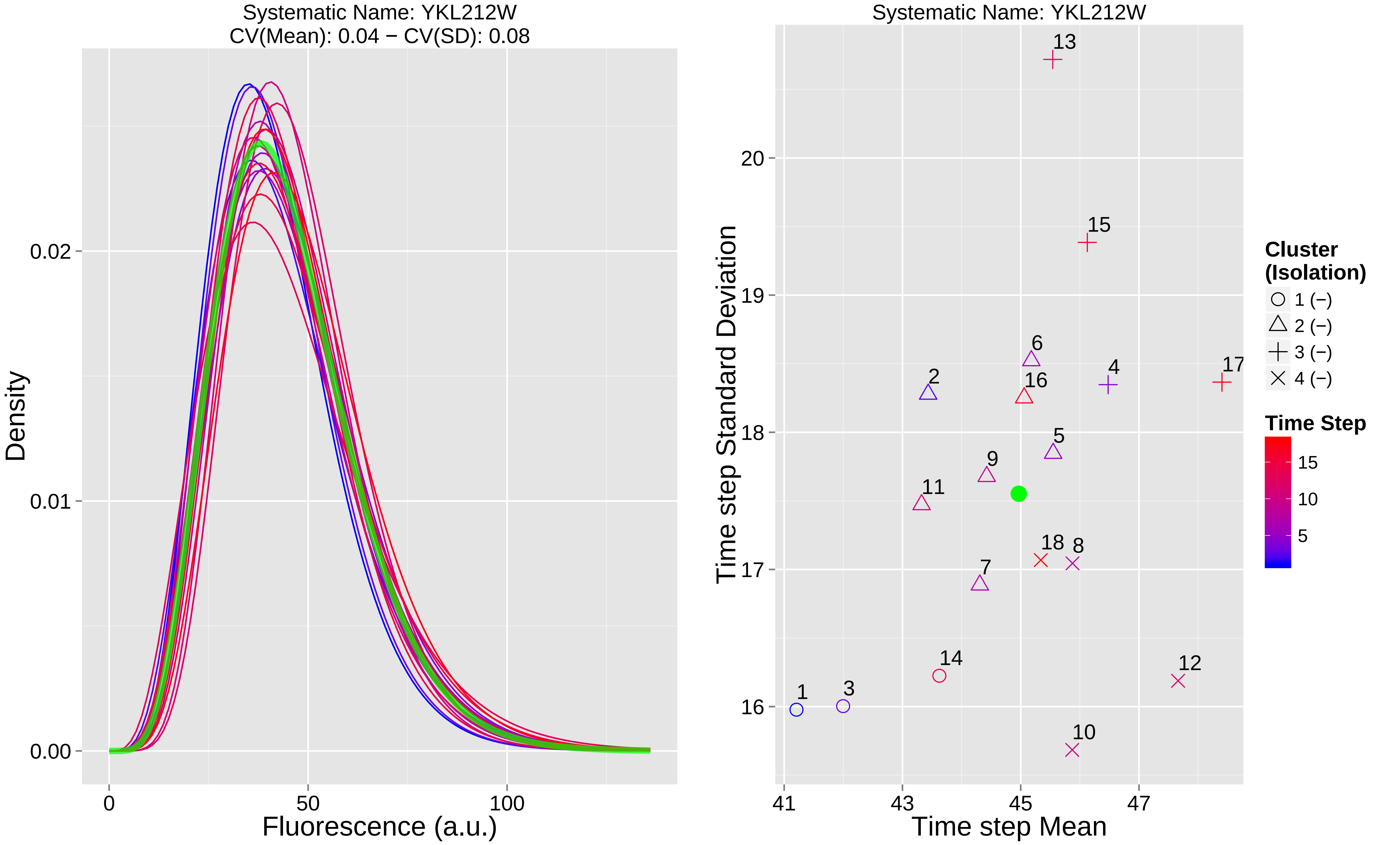

Systematic Name: YKL212W
CV(Mean): 0.04 − CV(SD): 0.08
Density
0.00
0.01
0.02
0
50
100
Fluorescence (a.u.)
Systematic Name: YKL212W
Time step Standard Deviation
Time step Mean
16
17
18
19
20
41
43
45
47
Cluster (Isolation)
1 (−)
2 (−)
3 (−)
4 (−)
Time Step
5
10
15

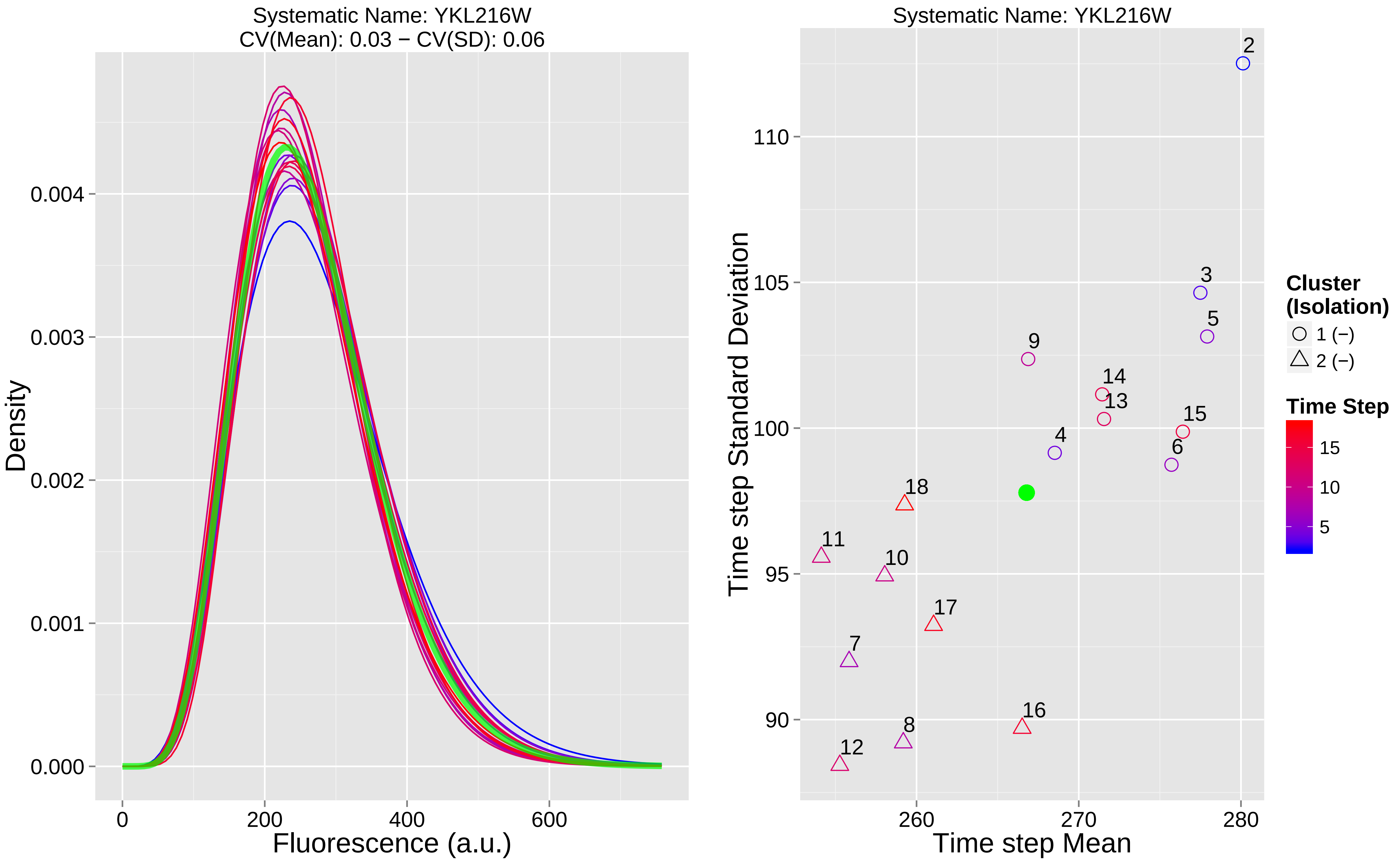
Systematic Name: YKL216W
CV(Mean): 0.03 − CV(SD): 0.06
Density
0.004
0.003
0.002
0.001
0.000
0
200
400
600
Fluorescence (a.u.)
Systematic Name: YKL216W
Time step Standard Deviation
110
105
100
95
90
260
270
280
Time step Mean
2
3
5
9
14
13
15
4
6
18
11
10
17
7
16
8
12
Cluster (Isolation)
1 (−)
2 (−)
Time Step
15
10
5

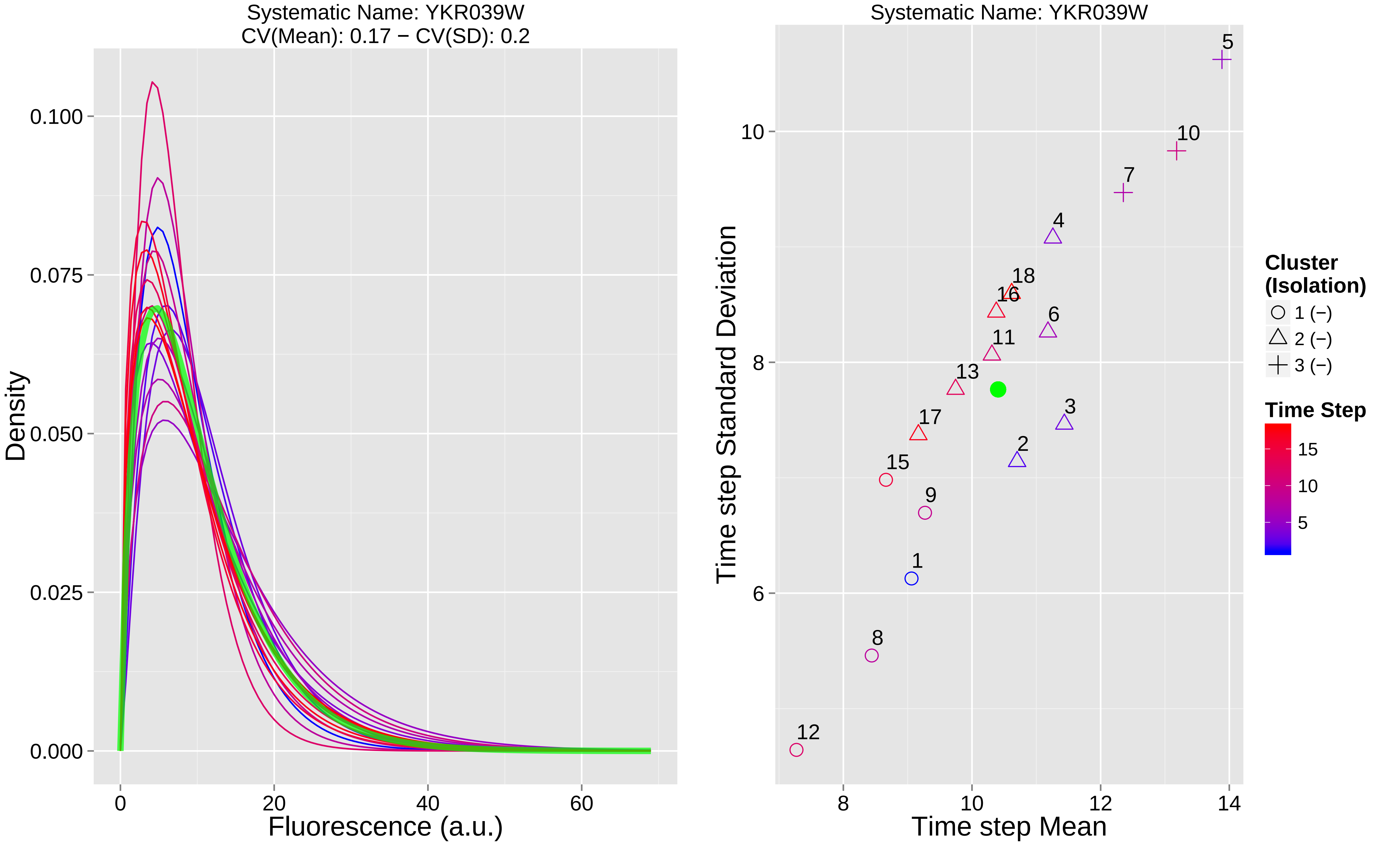
Systematic Name: YKR039W
CV(Mean): 0.17 − CV(SD): 0.2
Density
0.100
0.075
0.050
0.025
0.000
0
20
40
60
Fluorescence (a.u.)
Systematic Name: YKR039W
Time step Standard Deviation
10
8
6
8
10
12
14
Time step Mean
5
10
7
4
18
16
6
11
13
3
17
2
15
9
1
8
12
Cluster
(Isolation)
1 (−)
2 (−)
3 (−)
Time Step
15
10
5

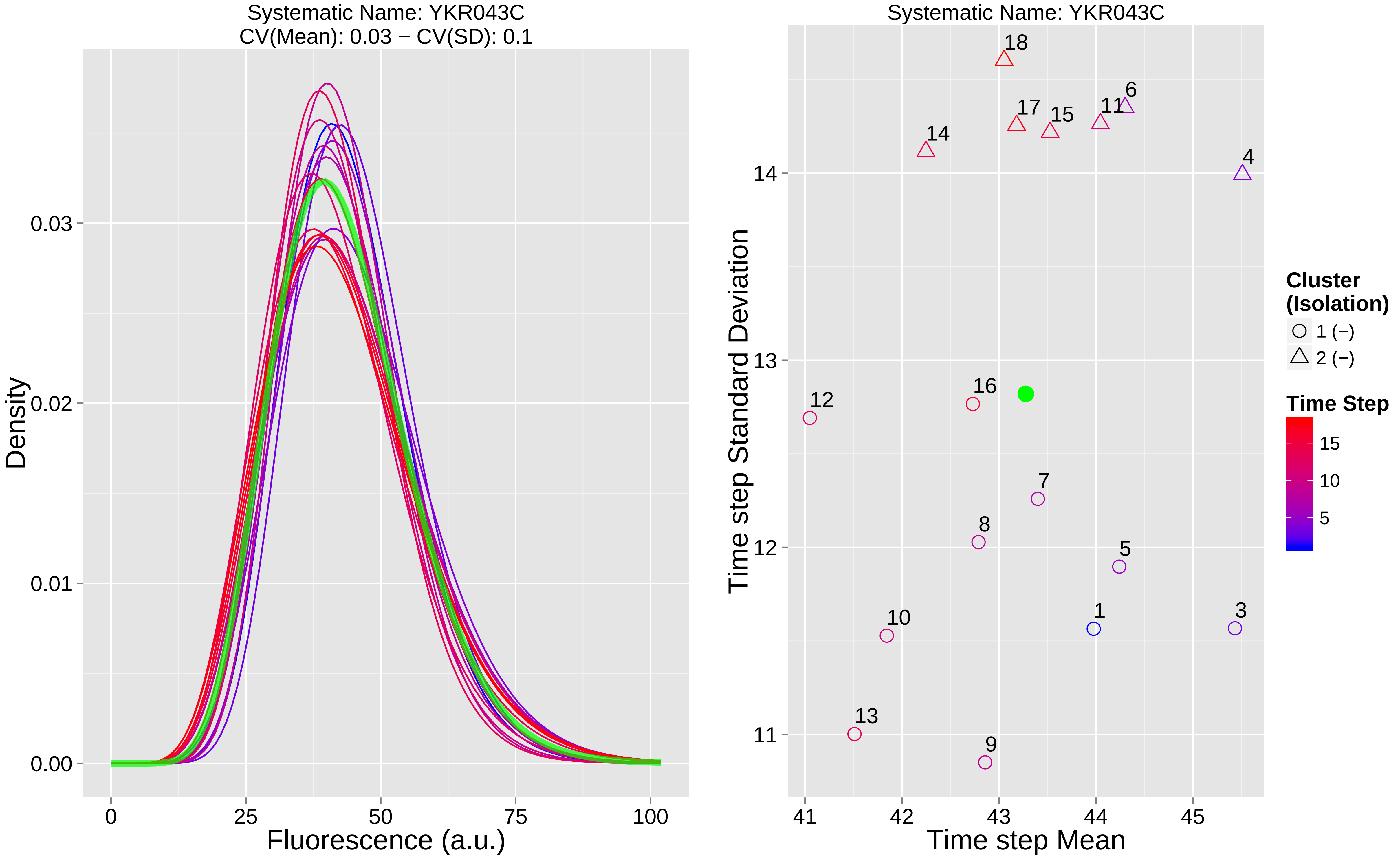
Systematic Name: YKR043C
CV(Mean): 0.03 − CV(SD): 0.1
Density
Fluorescence (a.u.)
0.00
0.01
0.02
0.03
0
25
50
75
100
Systematic Name: YKR043C
Time step Standard Deviation
Time step Mean
11
12
13
14
41
42
43
44
45
18
6
17
15
11
14
4
16
12
7
8
5
10
1
3
13
9
Cluster
(Isolation)
1 (−)
2 (−)
Time Step
15
10
5

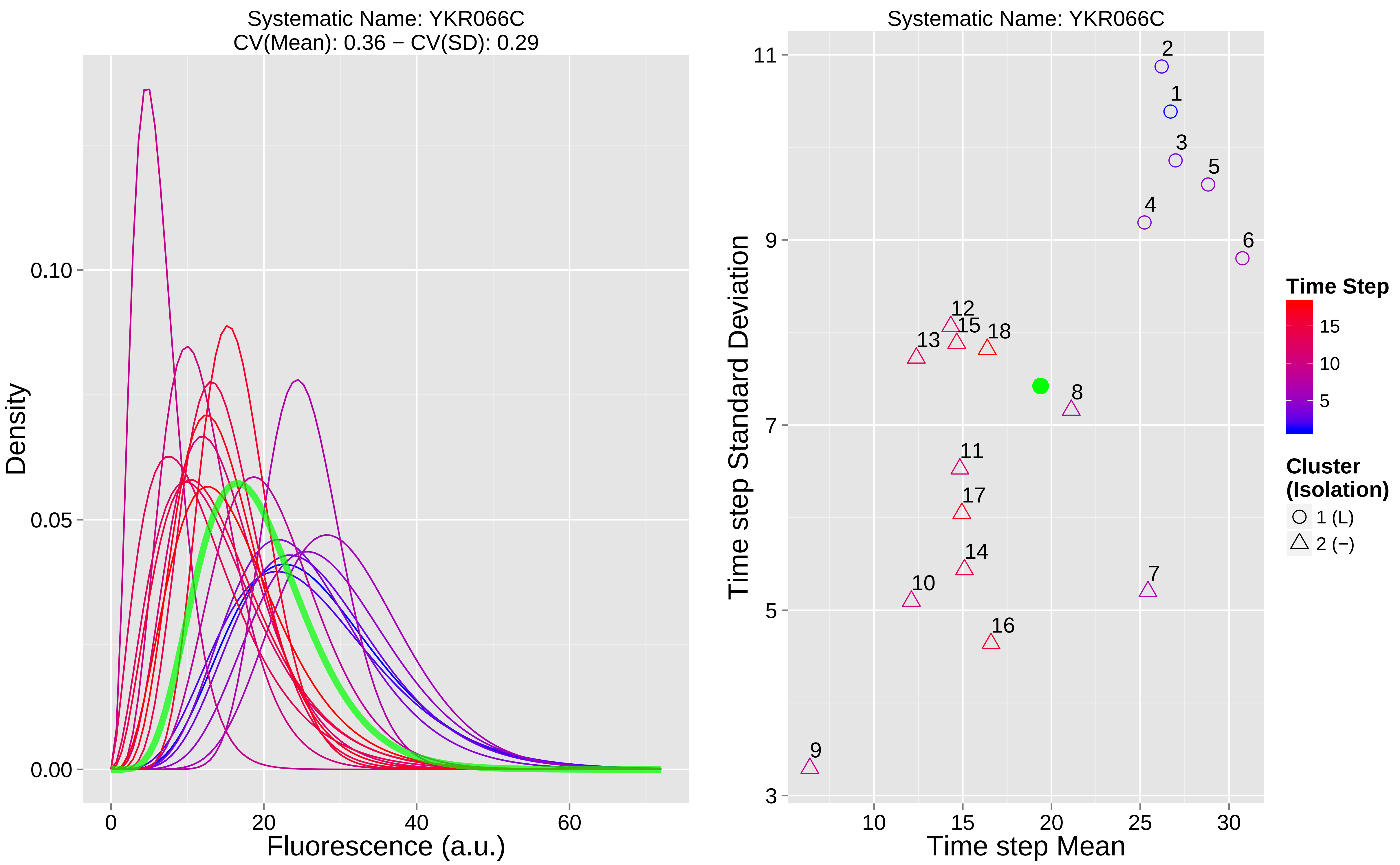
Systematic Name: YKR066C
CV(Mean): 0.36 − CV(SD): 0.29
Density
0.10
0.05
0.00
0
20
40
60
Fluorescence (a.u.)
Systematic Name: YKR066C
Time step Standard Deviation
11
9
7
5
3
10
15
20
25
30
Time step Mean
Time Step
15
10
5
Cluster (Isolation)
1 (L)
2 (−)

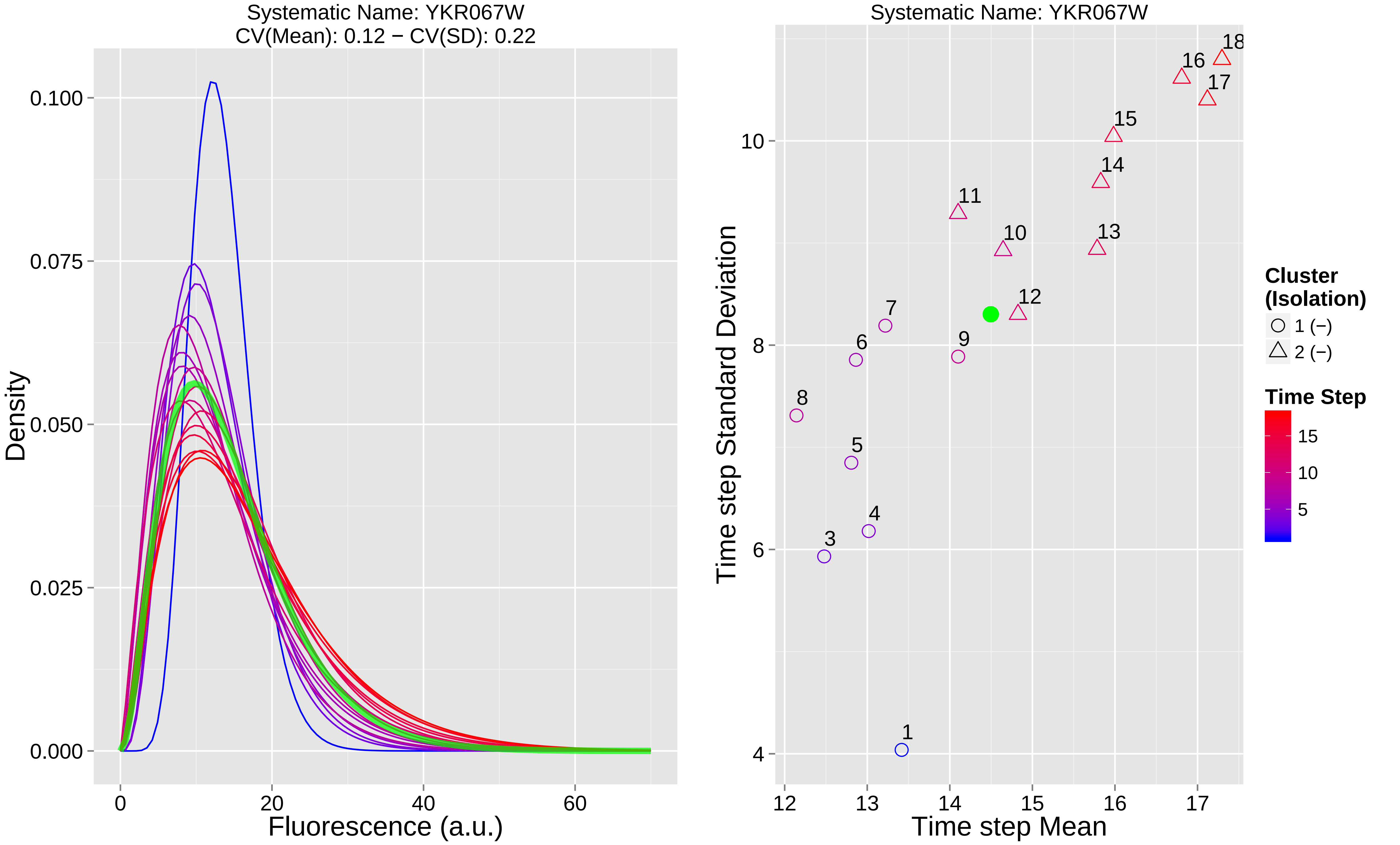
Systematic Name: YKR067W
CV(Mean): 0.12 − CV(SD): 0.22
Density
0.100
0.075
0.050
0.025
0.000
0
20
40
60
Fluorescence (a.u.)
Systematic Name: YKR067W
Time step Standard Deviation
10
8
6
4
12
13
14
15
16
17
Time step Mean
1
3
4
5
6
7
8
9
10
11
12
13
14
15
16
17
18
Cluster
(Isolation)
1 (−)
2 (−)
Time Step
15
10
5

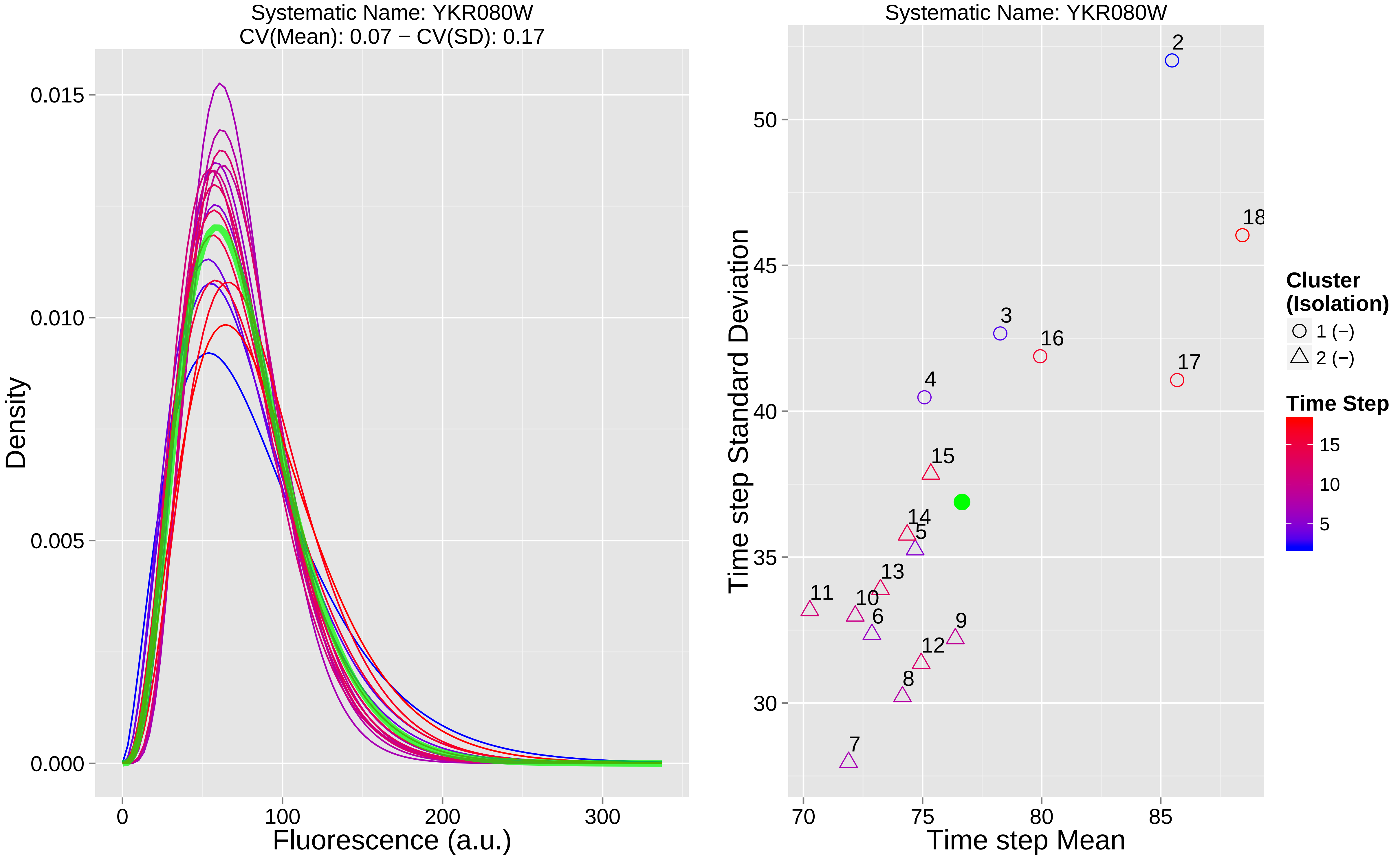

Systematic Name: YKR080W
CV(Mean): 0.07 − CV(SD): 0.17
Density
0.015
0.010
0.005
0.000
0
100
200
300
Fluorescence (a.u.)
Systematic Name: YKR080W
Time step Standard Deviation
50
45
40
35
30
70
75
80
85
Time step Mean
2
3
4
5
6
7
8
9
10
11
12
13
14
15
16
17
18
Cluster
(Isolation)
1 (−)
2 (−)
Time Step
15
10
5

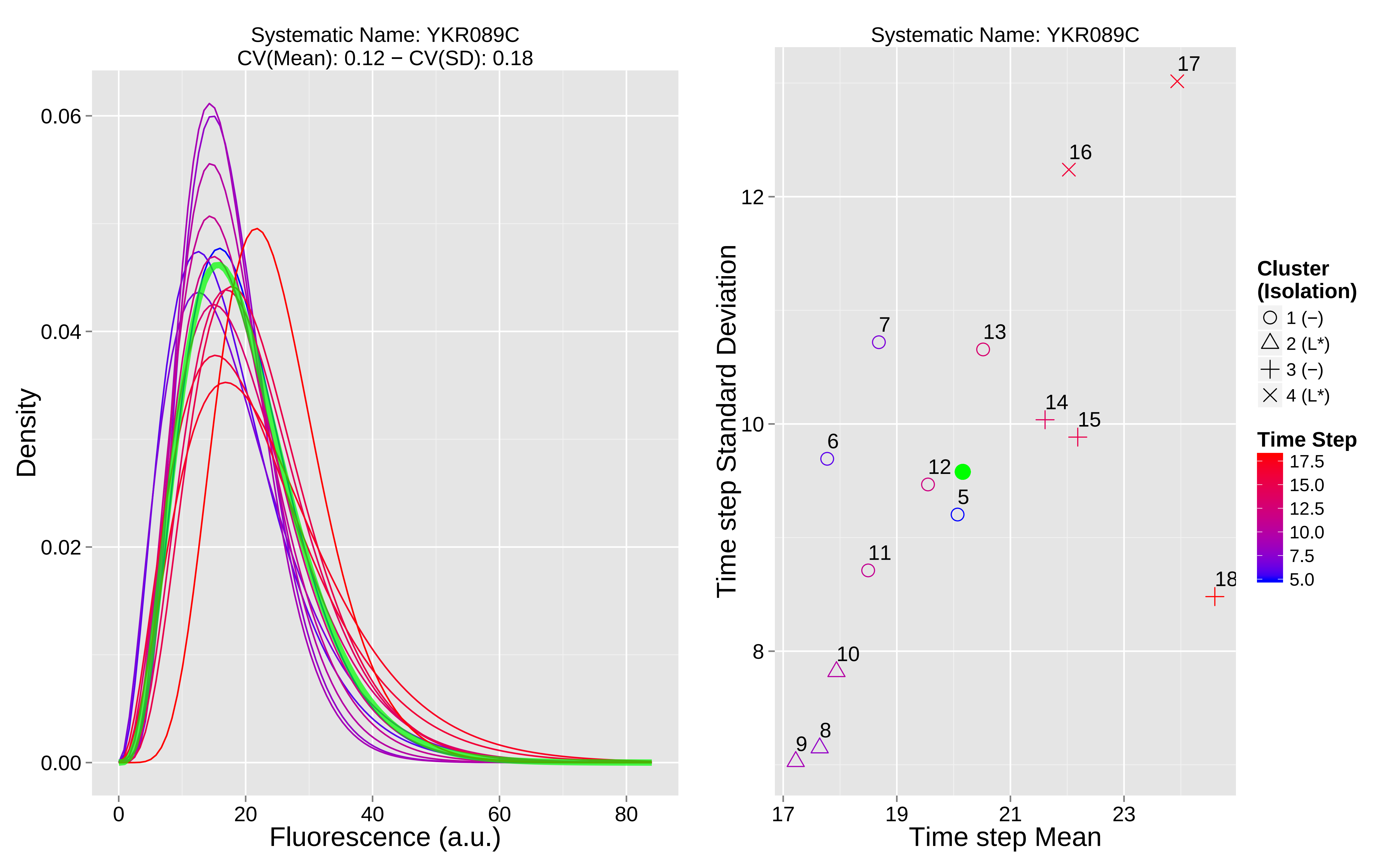
Systematic Name: YKR089C
CV(Mean): 0.12 − CV(SD): 0.18
Density
0.06
0.04
0.02
0.00
0
20
40
60
80
Fluorescence (a.u.)
Systematic Name: YKR089C
Time step Standard Deviation
12
10
8
17
19
21
23
Time step Mean
17
16
7
13
14
15
6
12
5
11
18
10
8
9
Cluster
(Isolation)
1 (−)
2 (L*)
3 (−)
4 (L*)
Time Step
17.5
15.0
12.5
10.0
7.5
5.0

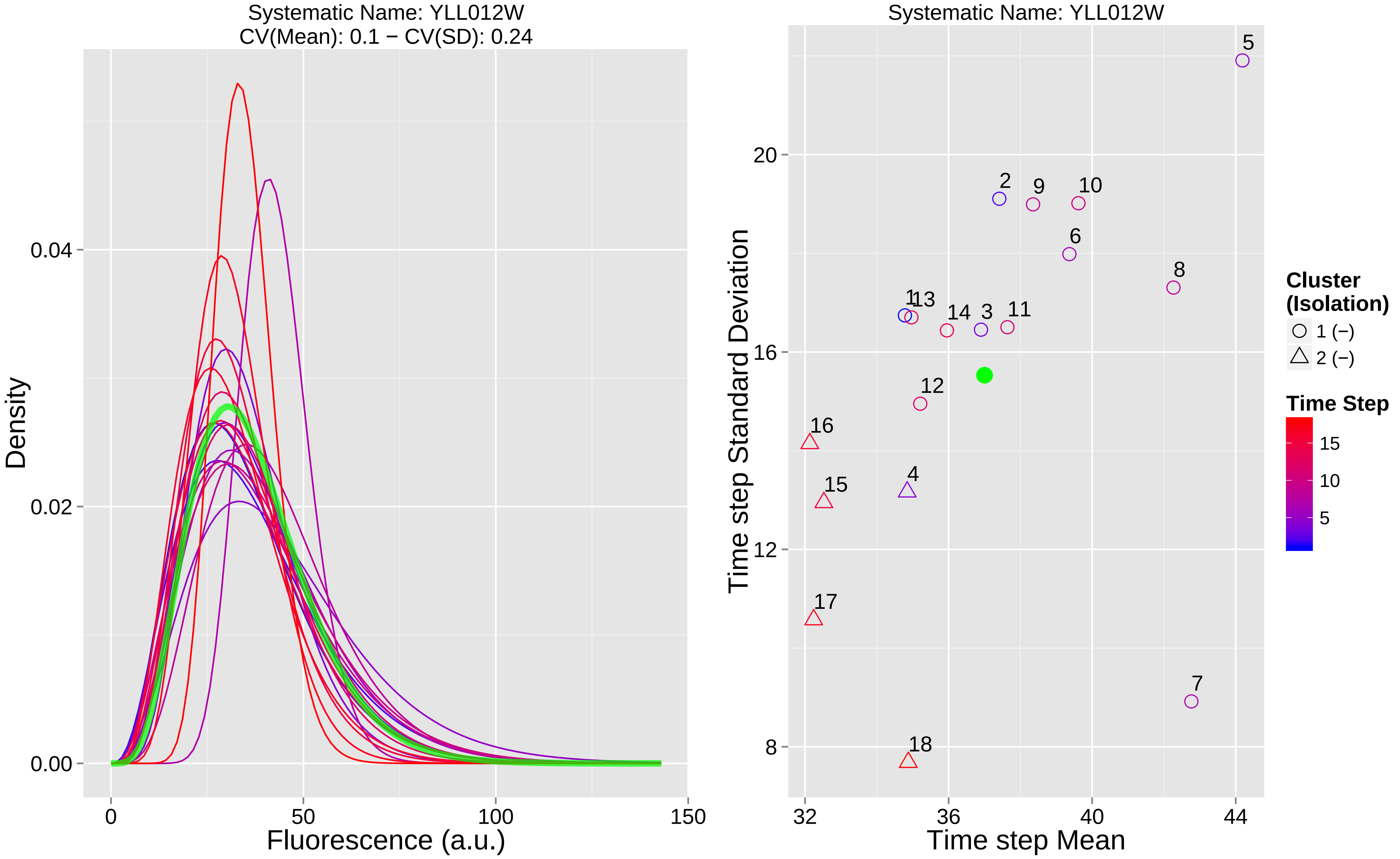

Systematic Name: YLL012W
CV(Mean): 0.1 − CV(SD): 0.24
Density
0.04
0.02
0.00
0
50
100
150
Fluorescence (a.u.)
Systematic Name: YLL012W
Time step Standard Deviation
20
16
12
8
32
36
40
44
Time step Mean
1
2
3
4
5
6
7
8
9
10
11
12
13
14
15
16
17
18
Cluster (Isolation)
1 (−)
2 (−)
Time Step
15
10
5

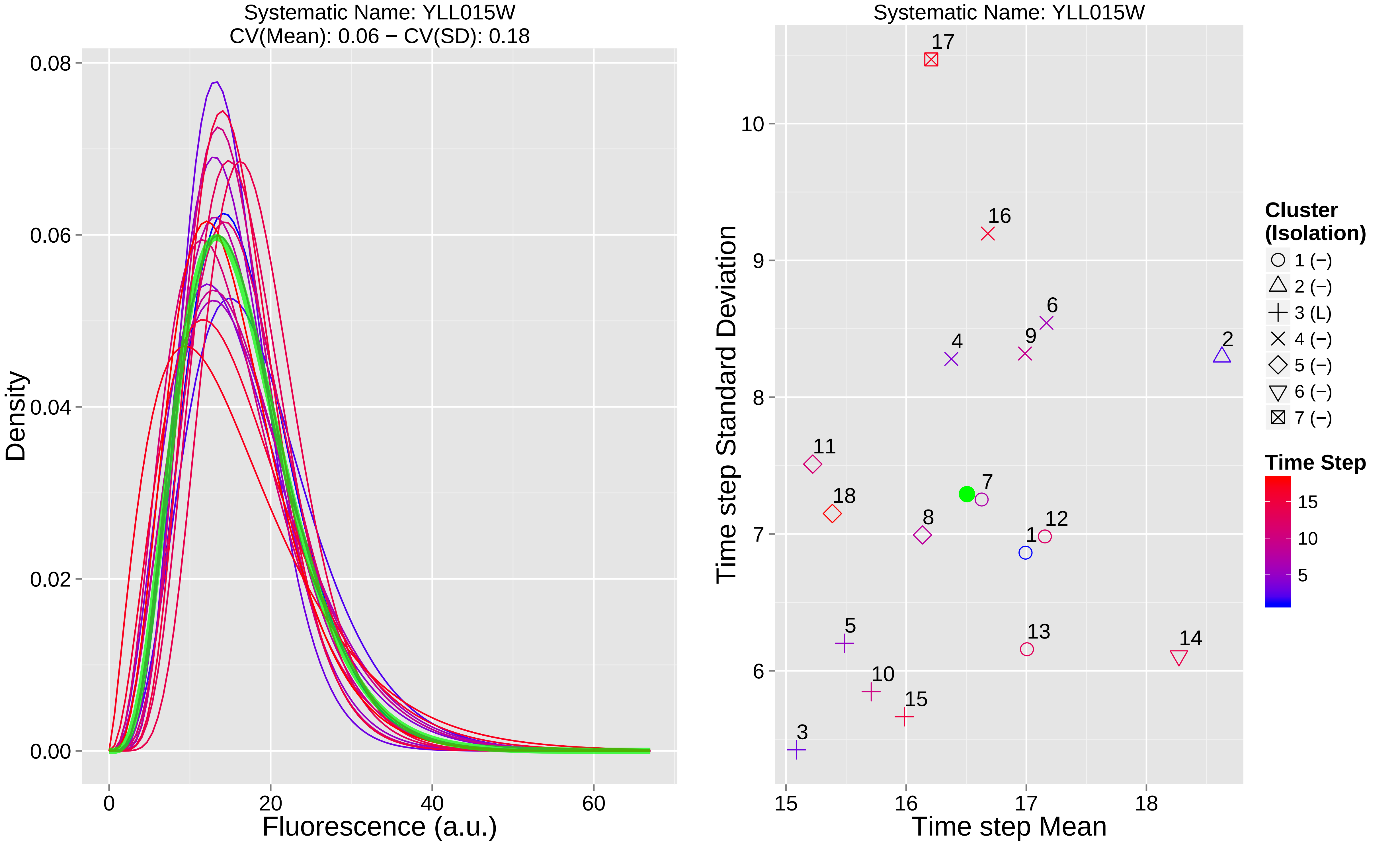
Systematic Name: YLL015W
CV(Mean): 0.06 − CV(SD): 0.18
Density
Fluorescence (a.u.)
0.00
0.02
0.04
0.06
0.08
0
20
40
60
Systematic Name: YLL015W
Time step Standard Deviation
Time step Mean
6
7
8
9
10
15
16
17
18
Cluster (Isolation)
1 (−)
2 (−)
3 (L)
4 (−)
5 (−)
6 (−)
7 (−)
Time Step
15
10
5

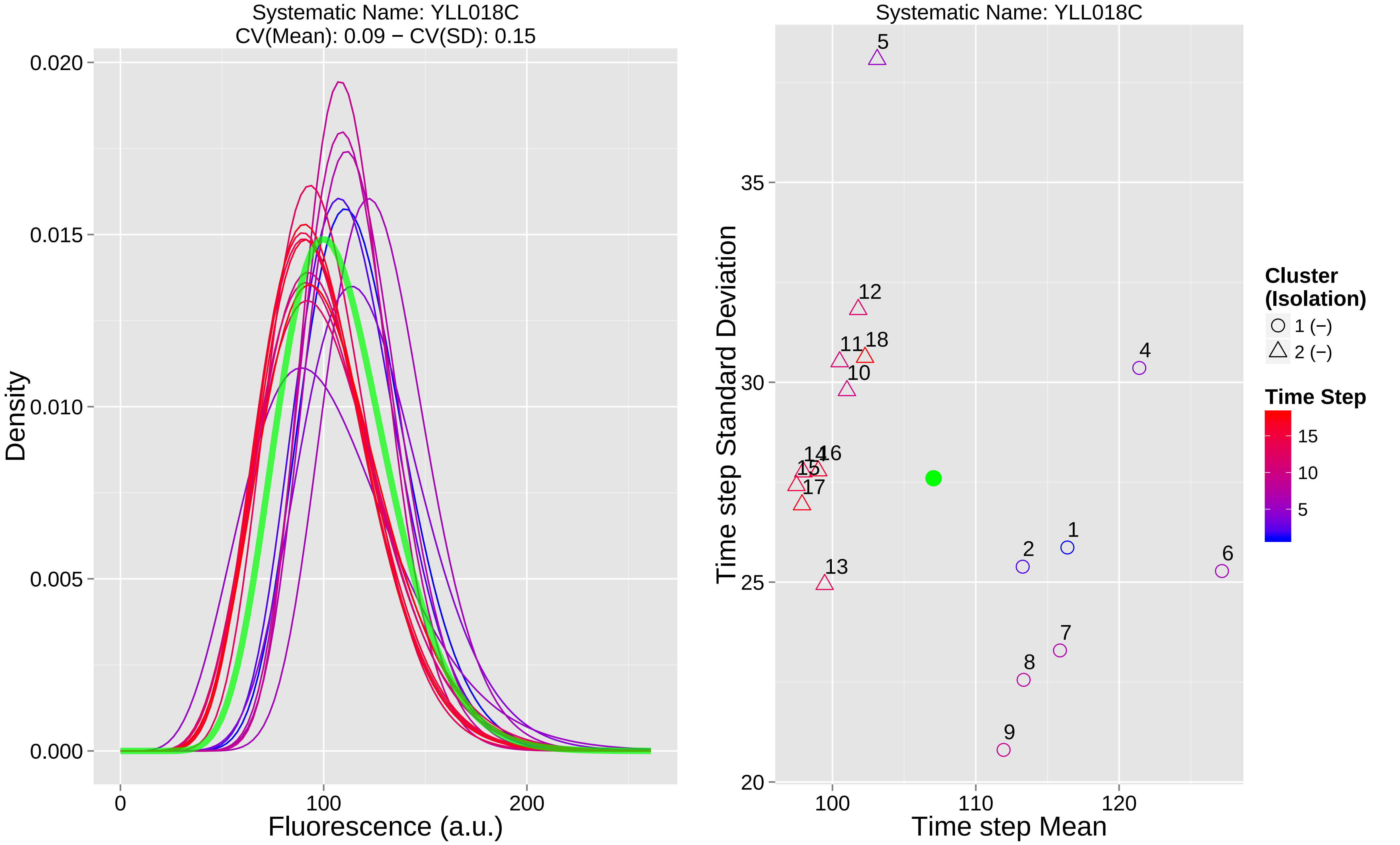
Systematic Name: YLL018C
CV(Mean): 0.09 − CV(SD): 0.15
Density
0.020
0.015
0.010
0.005
0.000
0
100
200
Fluorescence (a.u.)
Systematic Name: YLL018C
Time step Standard Deviation
35
30
25
20
100
110
120
Time step Mean
5
12
11
18
10
4
14
16
15
17
1
2
6
13
7
8
9
Cluster (Isolation)
1 (−)
2 (−)
Time Step
15
10
5

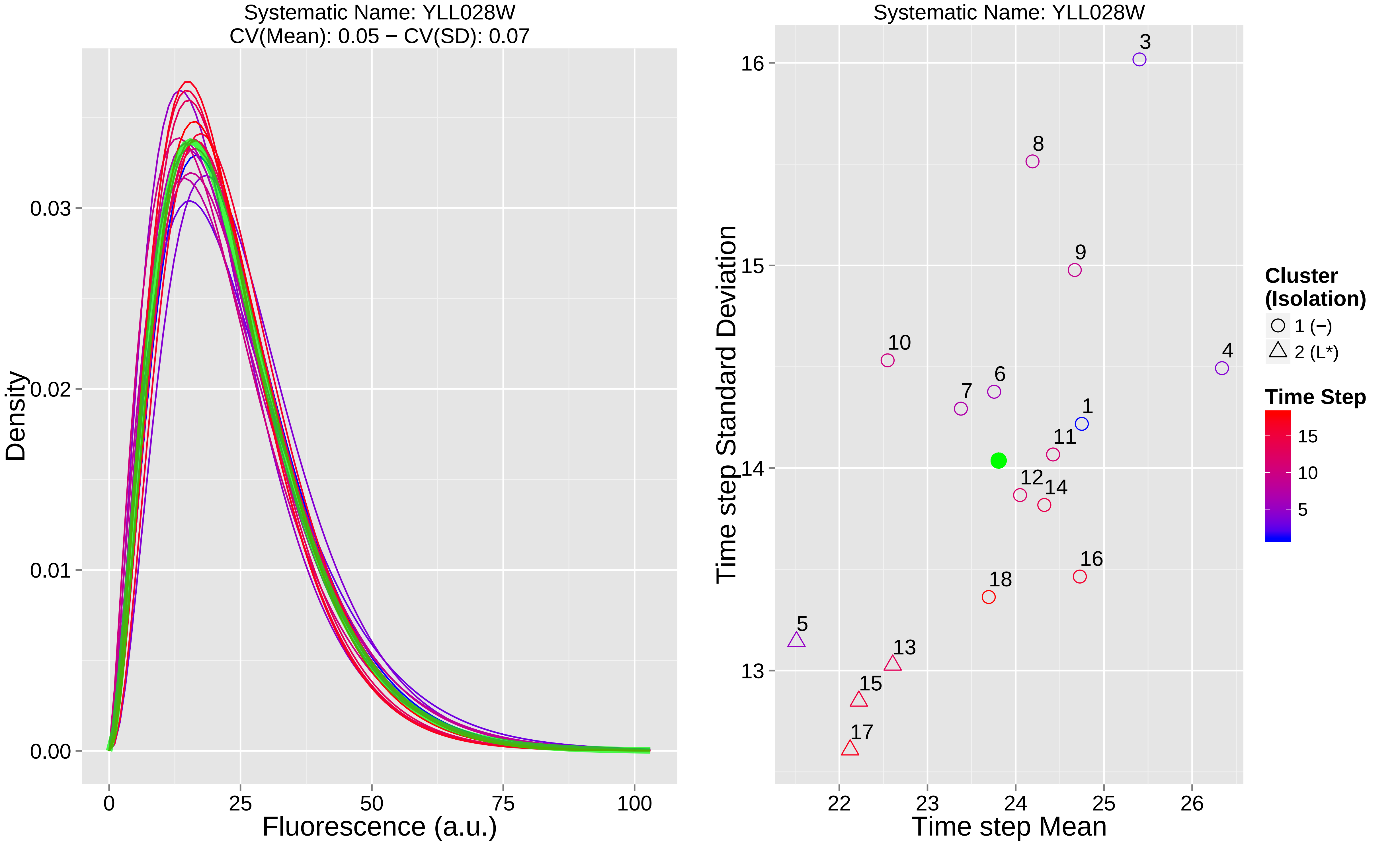
Systematic Name: YLL028W
CV(Mean): 0.05 − CV(SD): 0.07
Density
0.03
0.02
0.01
0.00
0
25
50
75
100
Fluorescence (a.u.)
Systematic Name: YLL028W
Time step Standard Deviation
16
15
14
13
22
23
24
25
26
Time step Mean
1
3
4
5
6
7
8
9
10
11
12
13
14
15
16
17
18
Cluster (Isolation)
1 (−)
2 (L*)
Time Step
15
10
5

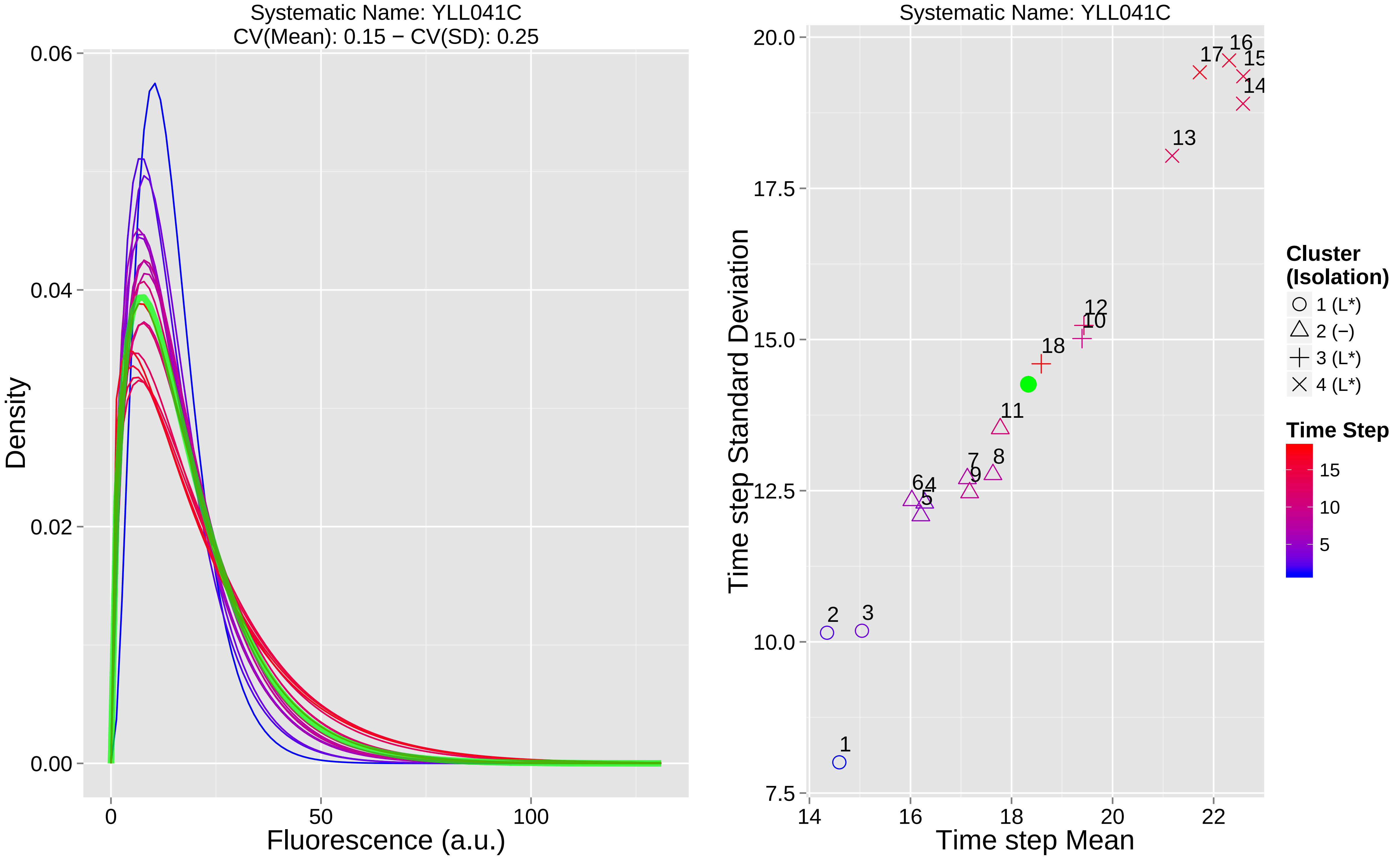
Systematic Name: YLL041C
CV(Mean): 0.15 − CV(SD): 0.25
Density
Fluorescence (a.u.)
0.06
0.04
0.02
0.00
0
50
100
Systematic Name: YLL041C
Time step Standard Deviation
Time step Mean
20.0
17.5
15.0
12.5
10.0
7.5
14
16
18
20
22
Cluster (Isolation)
1 (L*)
2 (−)
3 (L*)
4 (L*)
Time Step
15
10
5

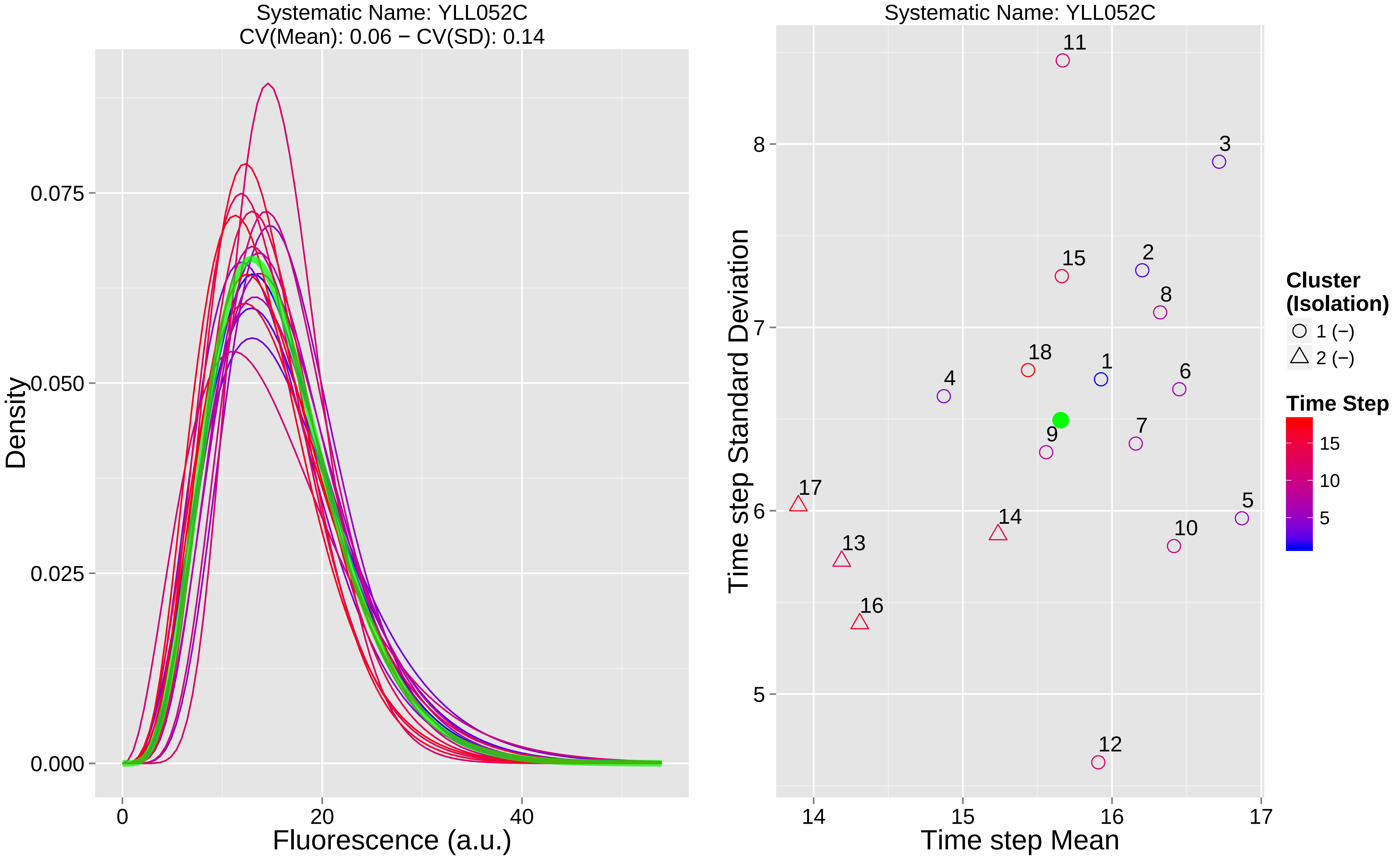

Systematic Name: YLL052C
CV(Mean): 0.06 − CV(SD): 0.14
Density
Fluorescence (a.u.)
Systematic Name: YLL052C
Time step Standard Deviation
Time step Mean
Cluster (Isolation)
1 (−)
2 (−)
Time Step

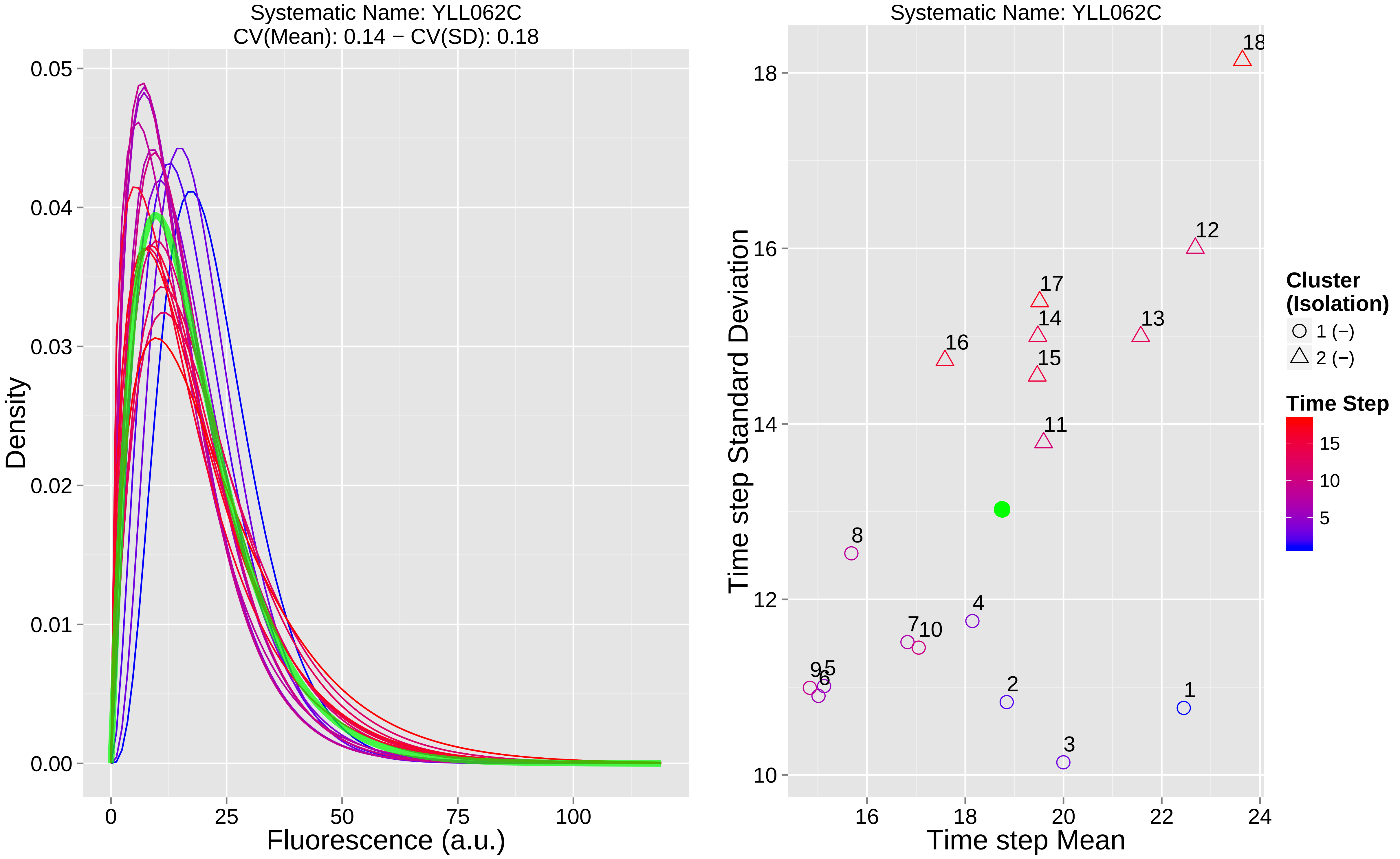

Systematic Name: YLL062C
CV(Mean): 0.14 − CV(SD): 0.18
Density
Fluorescence (a.u.)
0.00
0.01
0.02
0.03
0.04
0.05
0
25
50
75
100
Systematic Name: YLL062C
Time step Standard Deviation
Time step Mean
10
12
14
16
18
16
18
20
22
24
1
2
3
4
5
6
7
8
9
10
11
12
13
14
15
16
17
18
Cluster (Isolation)
1 (−)
2 (−)
Time Step
5
10
15

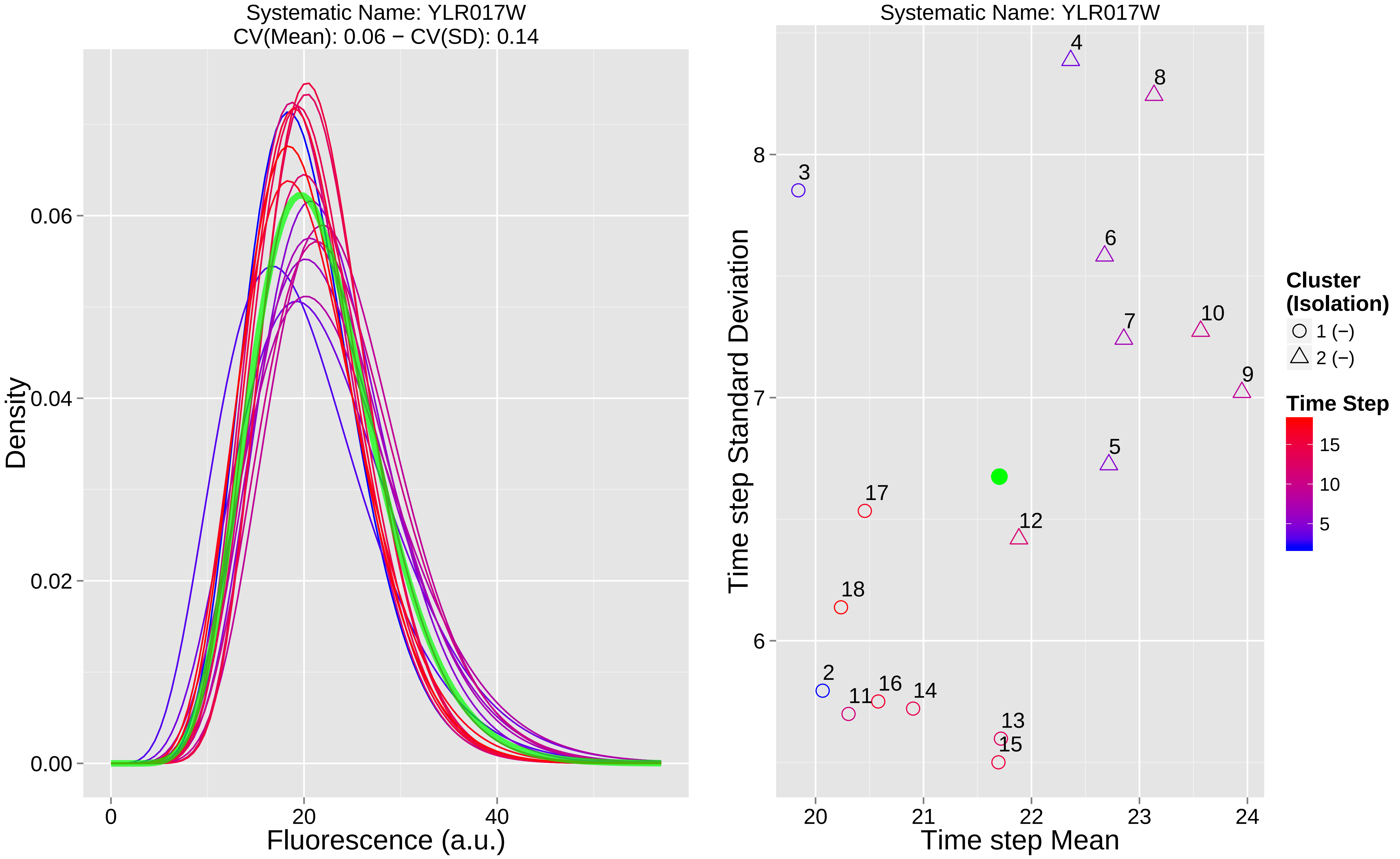
Systematic Name: YLR017W
CV(Mean): 0.06 − CV(SD): 0.14
Density
Fluorescence (a.u.)
0.00
0.02
0.04
0.06
0
20
40
Systematic Name: YLR017W
Time step Standard Deviation
Time step Mean
6
7
8
20
21
22
23
24
2
3
4
5
6
7
8
9
10
11
12
13
14
15
16
17
18
Cluster (Isolation)
1 (−)
2 (−)
Time Step
5
10
15

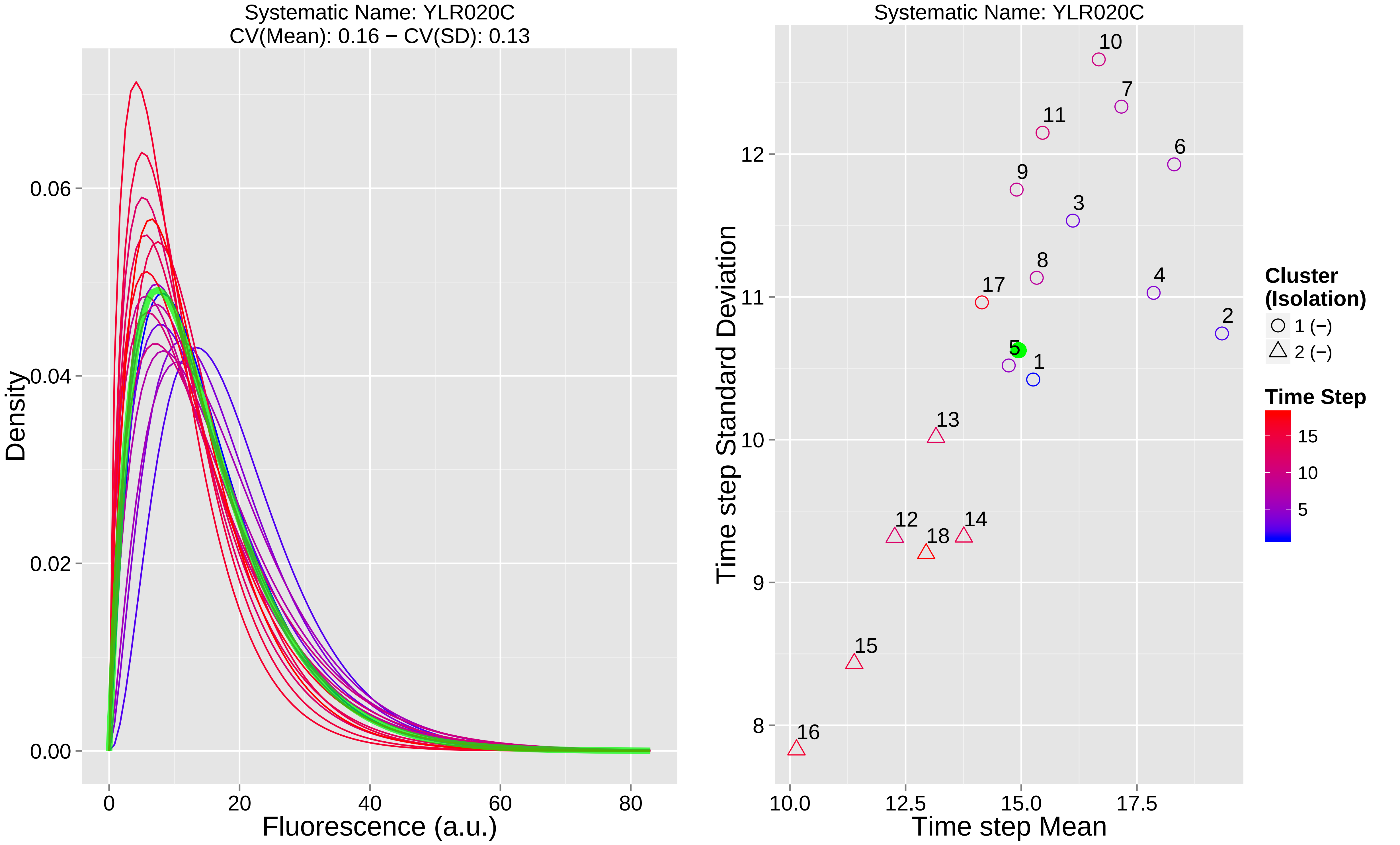
Systematic Name: YLR020C
CV(Mean): 0.16 − CV(SD): 0.13
Density
Fluorescence (a.u.)
0.00
0.02
0.04
0.06
0
20
40
60
80
Systematic Name: YLR020C
Time step Standard Deviation
Time step Mean
8
9
10
11
12
10.0
12.5
15.0
17.5
1
2
3
4
5
6
7
8
9
10
11
12
13
14
15
16
17
18
Cluster
(Isolation)
1 (−)
2 (−)
Time Step
15
10
5

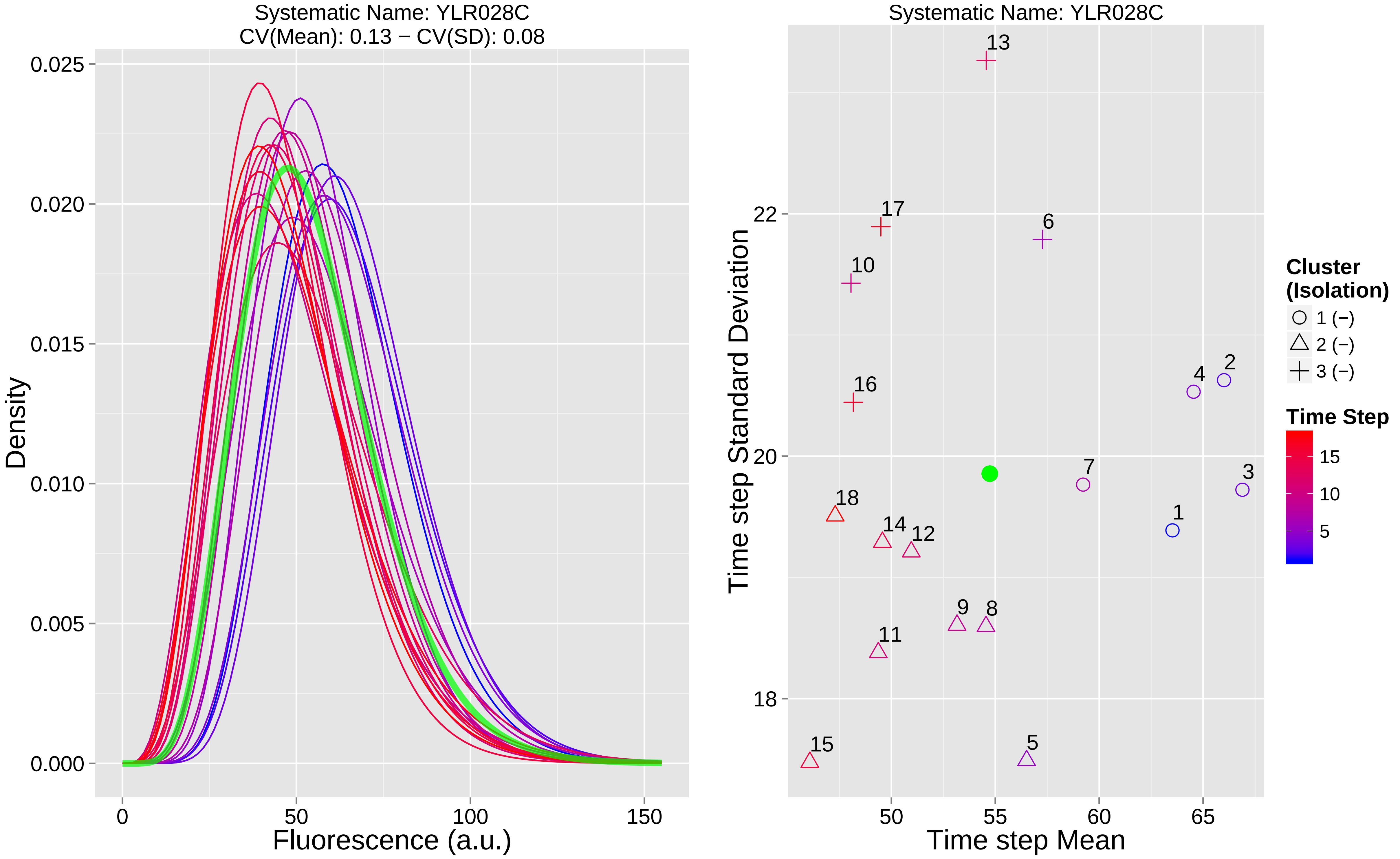
Systematic Name: YLR028C
CV(Mean): 0.13 − CV(SD): 0.08
Density
Fluorescence (a.u.)
0.025
0.020
0.015
0.010
0.005
0.000
0
50
100
150
Systematic Name: YLR028C
Time step Standard Deviation
Time step Mean
22
20
18
50
55
60
65
Cluster
(Isolation)
1 (−)
2 (−)
3 (−)
Time Step
15
10
5

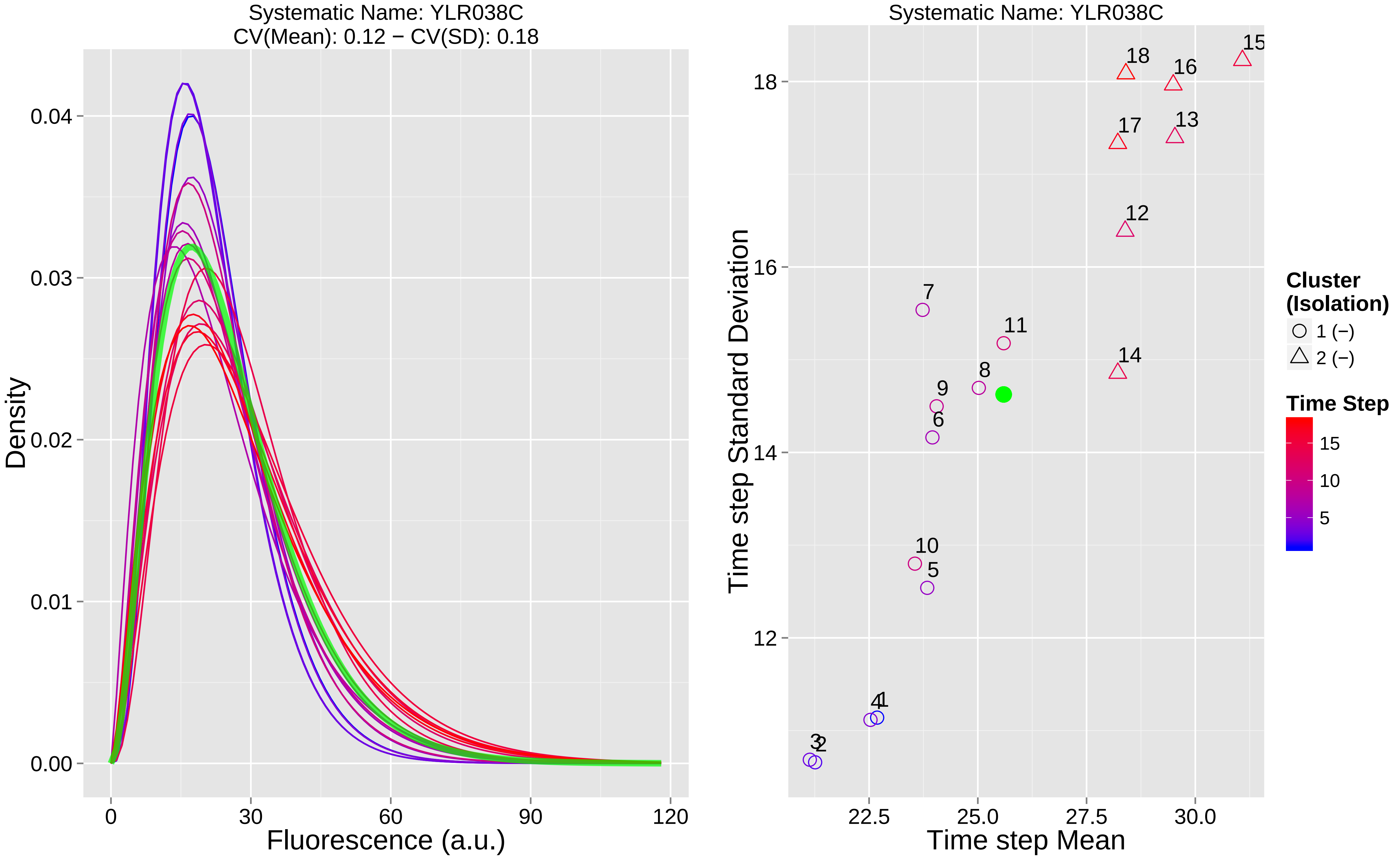

Systematic Name: YLR038C
CV(Mean): 0.12 − CV(SD): 0.18
Density
Fluorescence (a.u.)
0.00
0.01
0.02
0.03
0.04
0
30
60
90
120
Systematic Name: YLR038C
Time step Standard Deviation
Time step Mean
12
14
16
18
22.5
25.0
27.5
30.0
Cluster (Isolation)
1 (−)
2 (−)
Time Step
5
10
15

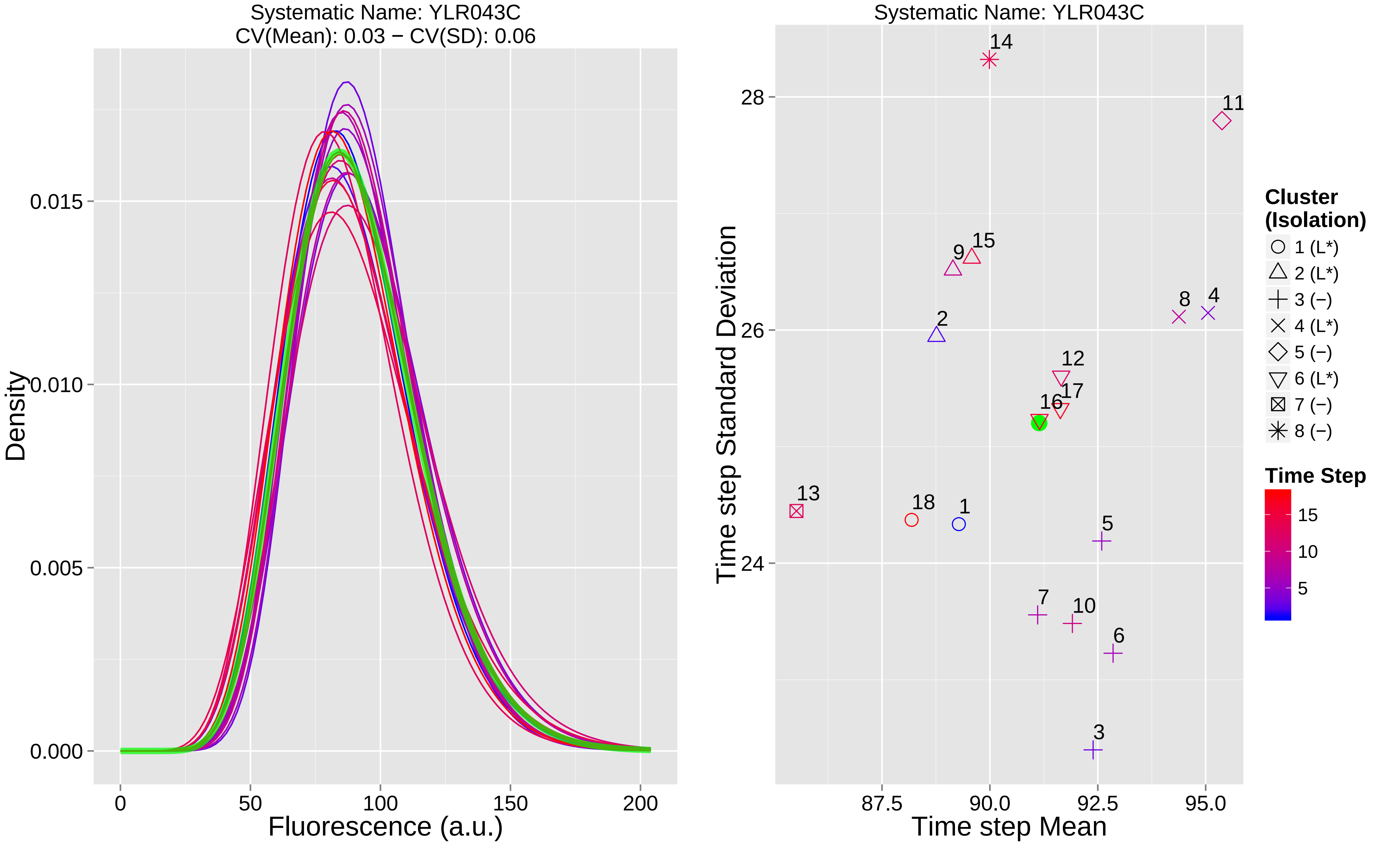

Systematic Name: YLR043C
CV(Mean): 0.03 − CV(SD): 0.06
Density
0.000
0.005
0.010
0.015
0
50
100
150
200
Fluorescence (a.u.)
Systematic Name: YLR043C
Time step Standard Deviation
24
26
28
87.5
90.0
92.5
95.0
Time step Mean
14
11
9
15
2
8
4
12
16
17
13
18
1
5
7
10
6
3
Cluster
(Isolation)
1 (L*)
2 (L*)
3 (−)
4 (L*)
5 (−)
6 (L*)
7 (−)
8 (−)
Time Step
15
10
5

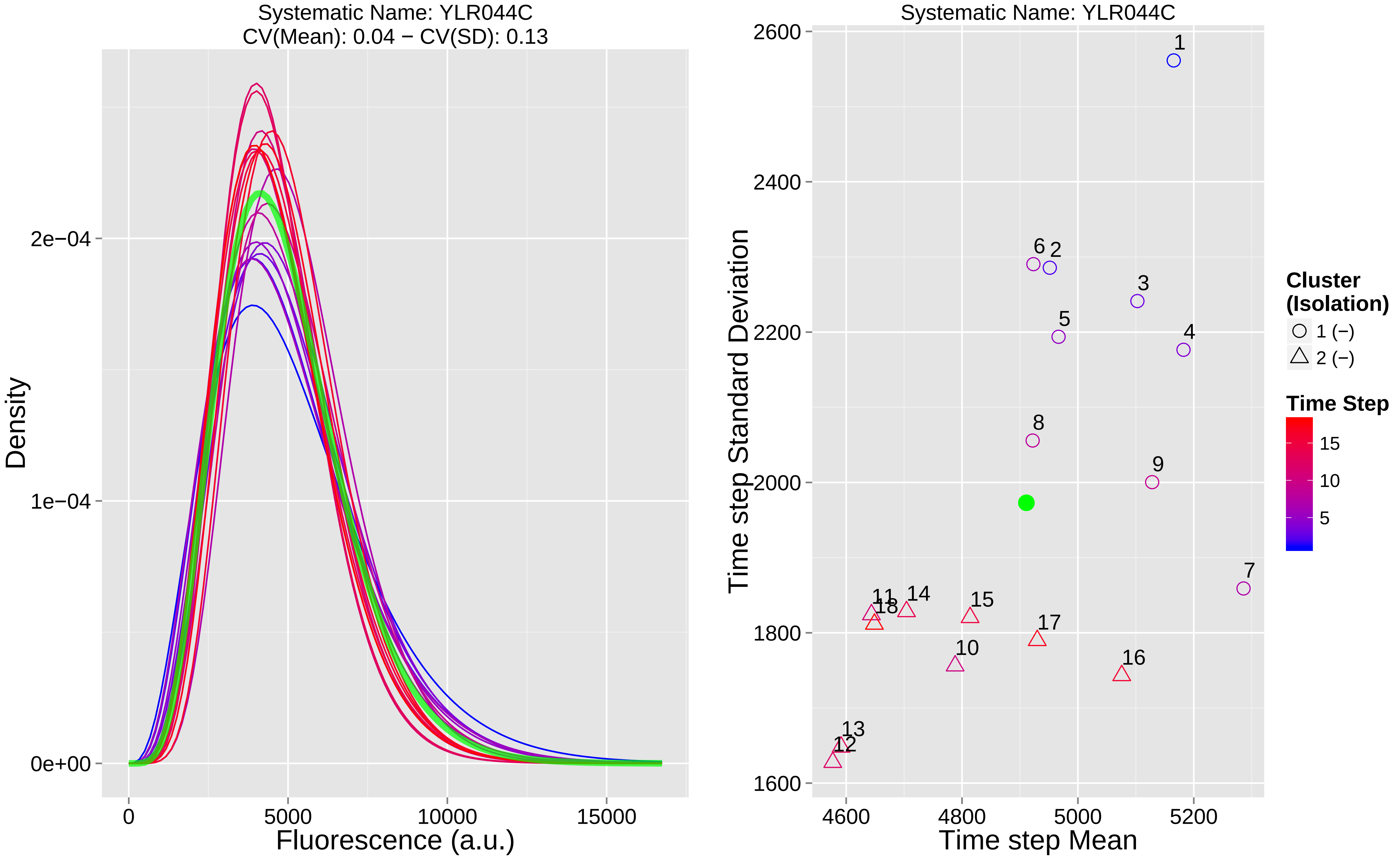
Systematic Name: YLR044C
CV(Mean): 0.04 − CV(SD): 0.13
Density
2e−04
1e−04
0e+00
0
5000
10000
15000
Fluorescence (a.u.)
Systematic Name: YLR044C
Time step Standard Deviation
2600
2400
2200
2000
1800
1600
4600
4800
5000
5200
Time step Mean
1
2
3
4
5
6
7
8
9
10
11
12
13
14
15
16
17
18
Cluster
(Isolation)
1 (−)
2 (−)
Time Step
15
10
5

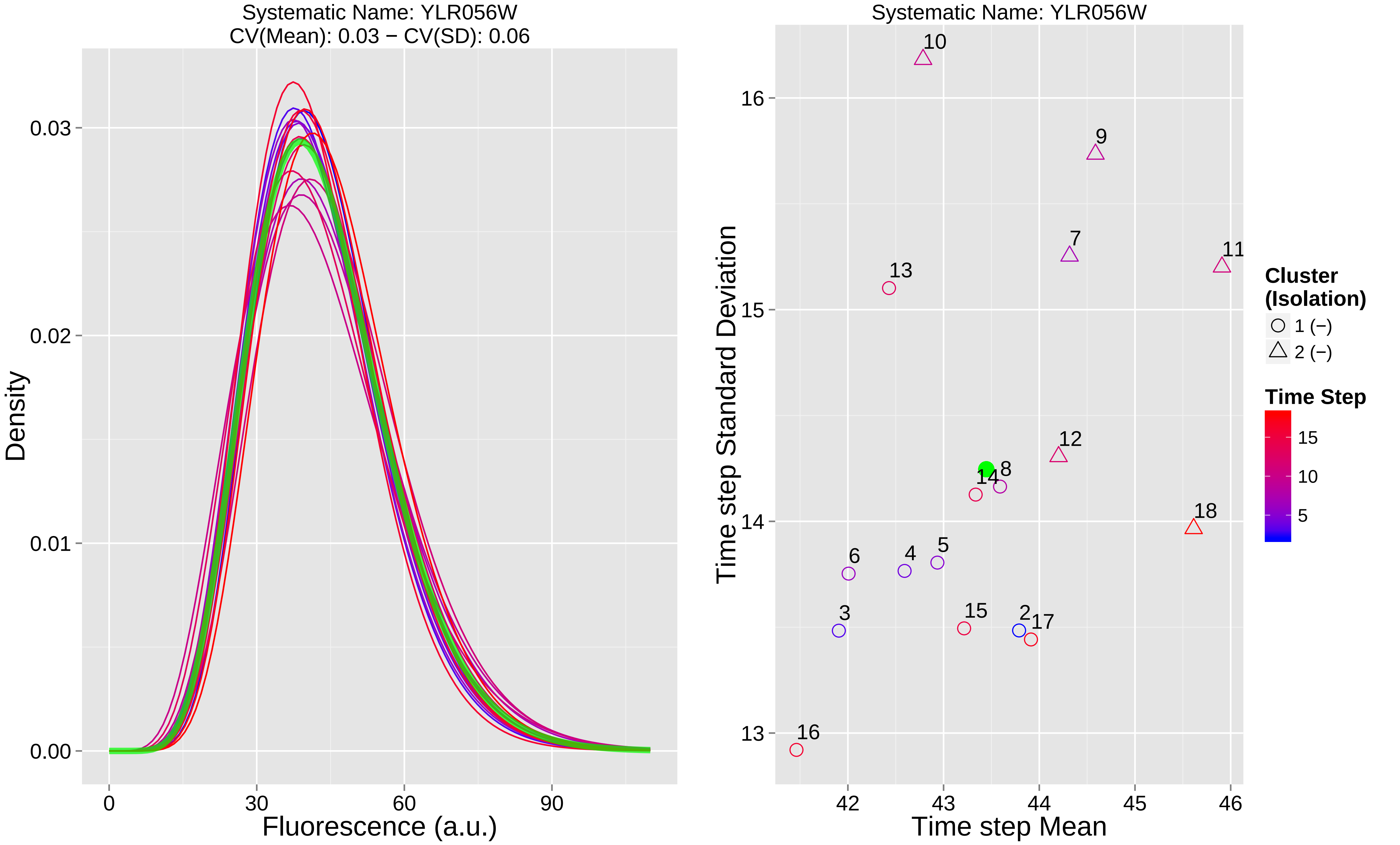

Systematic Name: YLR056W
CV(Mean): 0.03 − CV(SD): 0.06
Density
0.00
0.01
0.02
0.03
0
30
60
90
Fluorescence (a.u.)
Systematic Name: YLR056W
Time step Standard Deviation
13
14
15
16
42
43
44
45
46
Time step Mean
10
9
7
11
13
12
14
8
18
6
4
5
3
15
2
17
16
Cluster
(Isolation)
1 (−)
2 (−)
Time Step
15
10
5

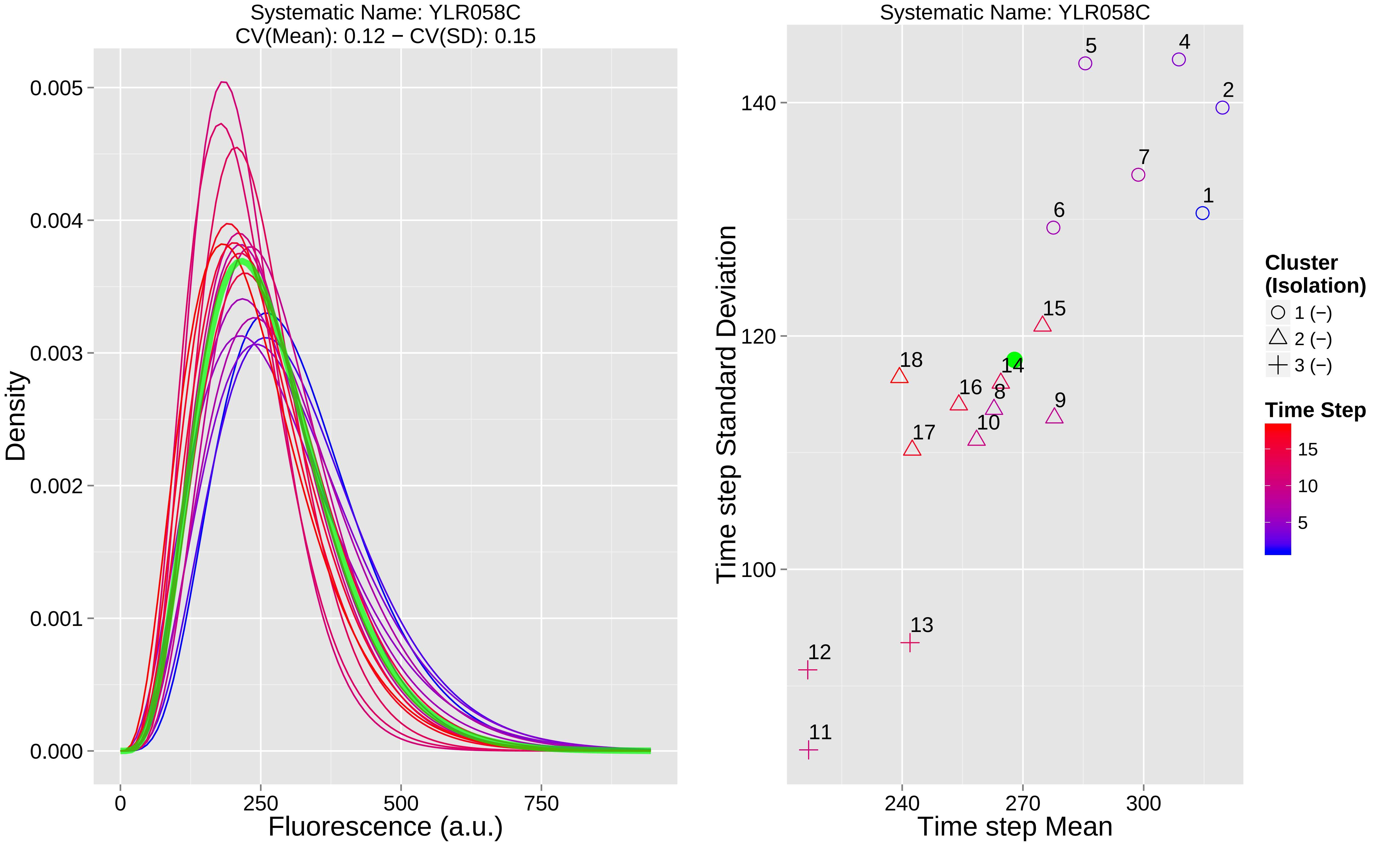

Systematic Name: YLR058C
CV(Mean): 0.12 − CV(SD): 0.15
Density
0.000
0.001
0.002
0.003
0.004
0.005
0
250
500
750
Fluorescence (a.u.)
Systematic Name: YLR058C
Time step Standard Deviation
100
120
140
240
270
300
Time step Mean
1
2
3
4
5
6
7
8
9
10
11
12
13
14
15
16
17
18
Cluster (Isolation)
1 (−)
2 (−)
3 (−)
Time Step
5
10
15

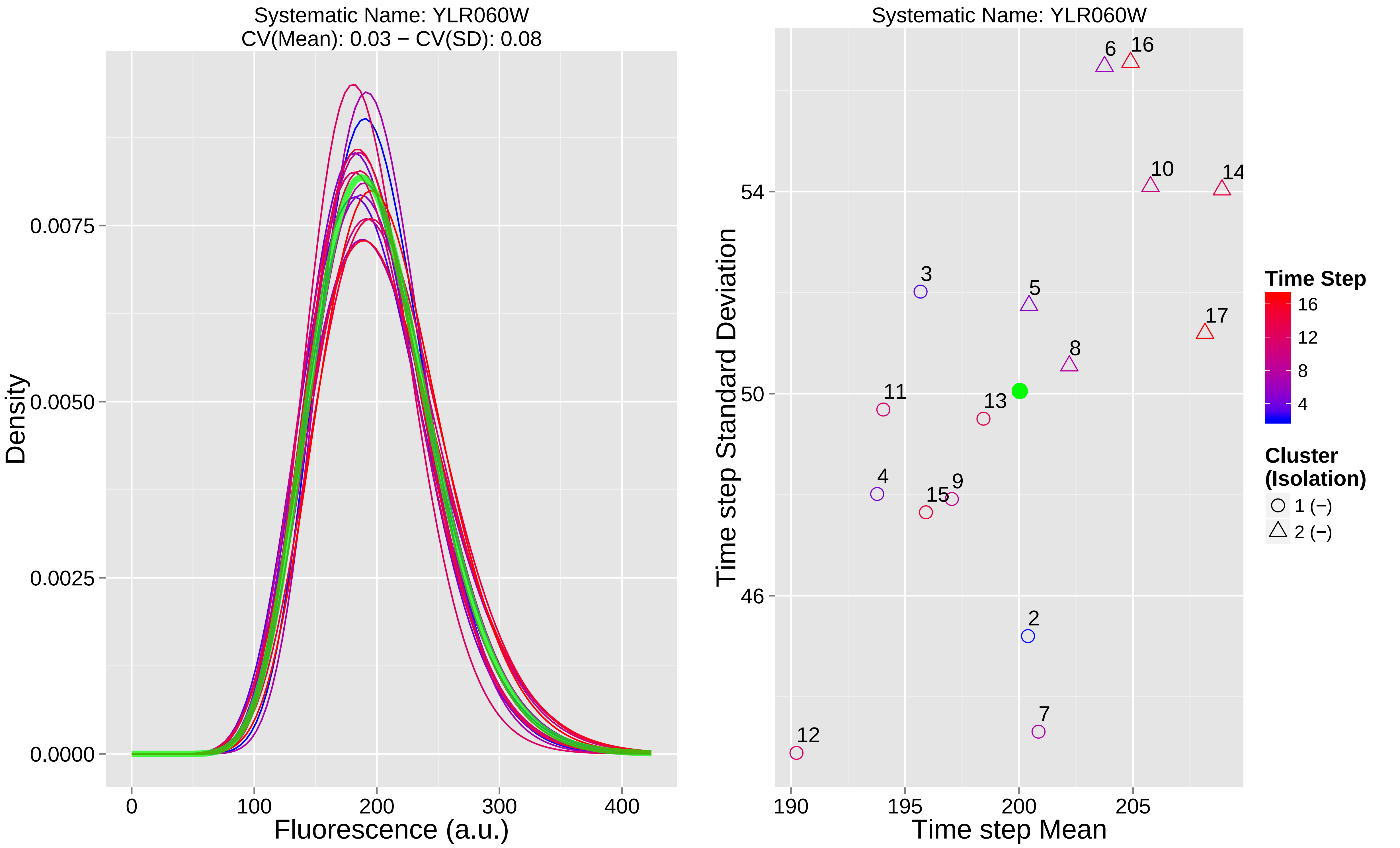

Systematic Name: YLR060W
CV(Mean): 0.03 − CV(SD): 0.08
Density
0.0075
0.0050
0.0025
0.0000
0
100
200
300
400
Fluorescence (a.u.)
Systematic Name: YLR060W
Time step Standard Deviation
54
50
46
190
195
200
205
Time step Mean
6
16
10
14
3
5
17
8
11
13
4
15
9
2
7
12
Time Step
16
12
8
4
Cluster
(Isolation)
1 (−)
2 (−)

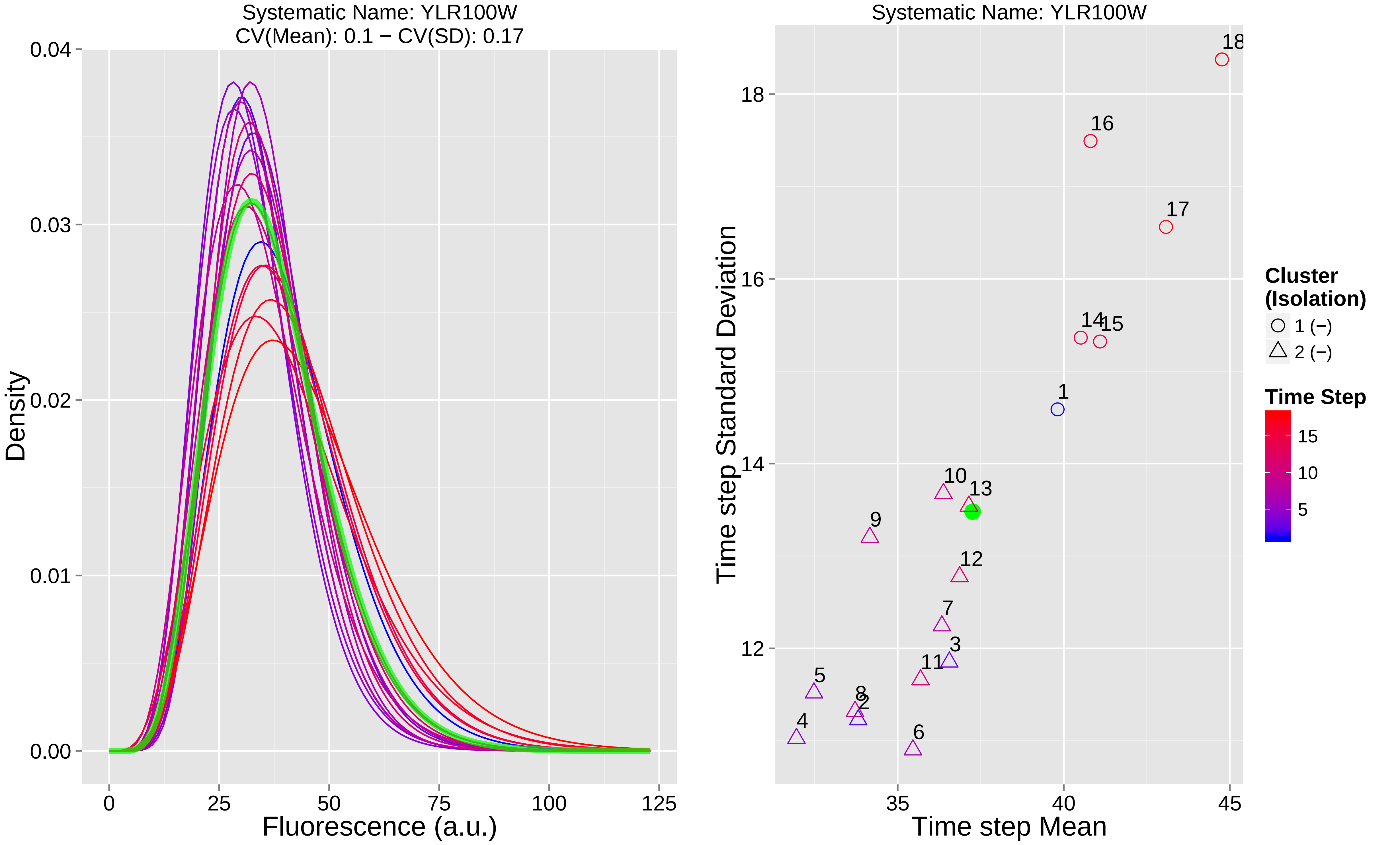

Systematic Name: YLR100W
CV(Mean): 0.1 − CV(SD): 0.17
Density
Fluorescence (a.u.)
0.00
0.01
0.02
0.03
0.04
0
25
50
75
100
125
Systematic Name: YLR100W
Time step Standard Deviation
Time step Mean
12
14
16
18
35
40
45
Cluster (Isolation)
1 (−)
2 (−)
Time Step
5
10
15

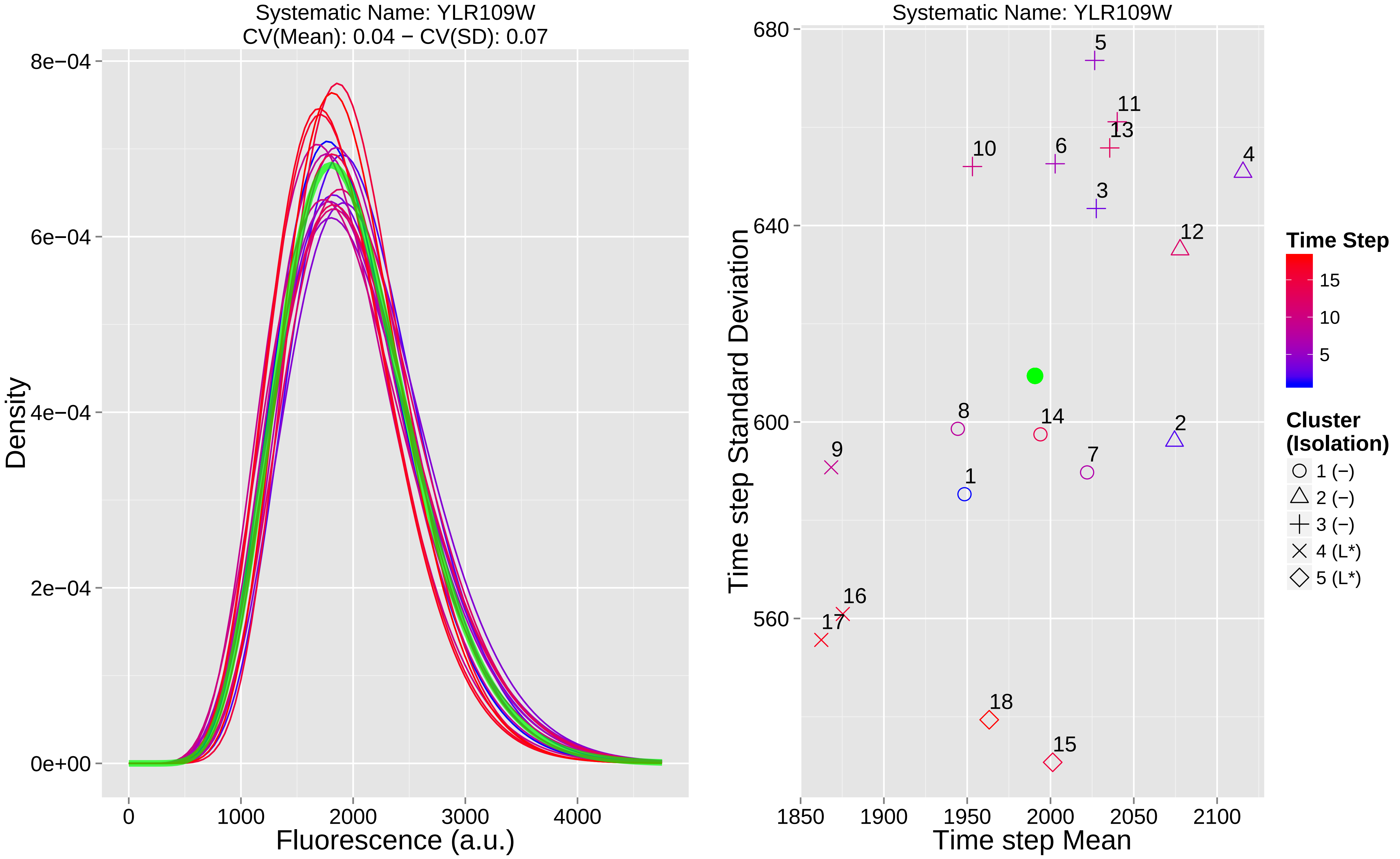
Systematic Name: YLR109W
CV(Mean): 0.04 − CV(SD): 0.07
Density
8e−04
6e−04
4e−04
2e−04
0e+00
0
1000
2000
3000
4000
Fluorescence (a.u.)
Systematic Name: YLR109W
Time step Standard Deviation
680
640
600
560
1850
1900
1950
2000
2050
2100
Time step Mean
Time Step
15
10
5
Cluster
(Isolation)
1 (−)
2 (−)
3 (−)
4 (L*)
5 (L*)

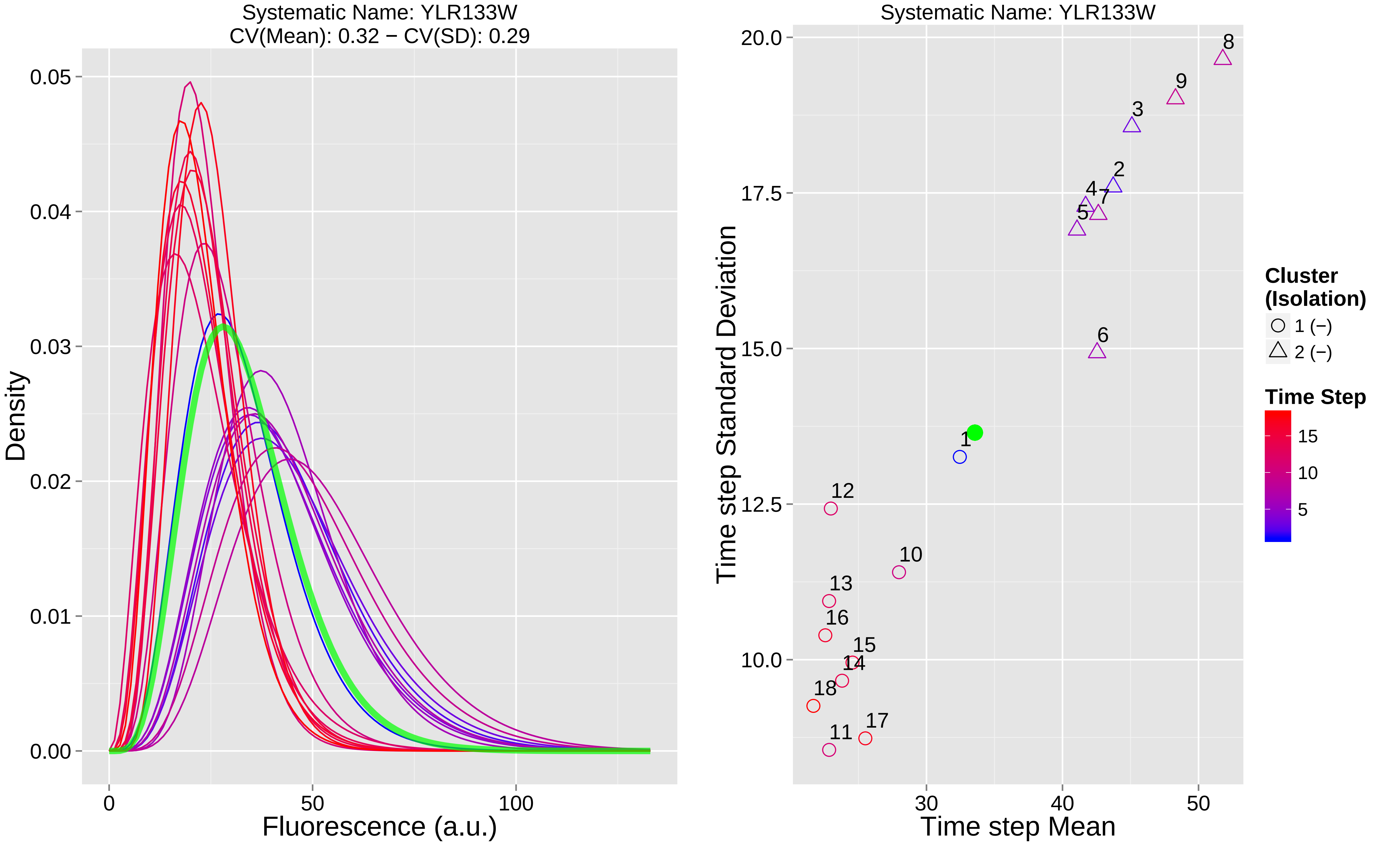
Systematic Name: YLR133W
CV(Mean): 0.32 − CV(SD): 0.29
Density
0.05
0.04
0.03
0.02
0.01
0.00
0
50
100
Fluorescence (a.u.)
Systematic Name: YLR133W
Time step Standard Deviation
20.0
17.5
15.0
12.5
10.0
30
40
50
Time step Mean
8
9
3
2
4
7
5
6
1
12
10
13
16
15
14
18
17
11
Cluster
(Isolation)
1 (−)
2 (−)
Time Step
15
10
5

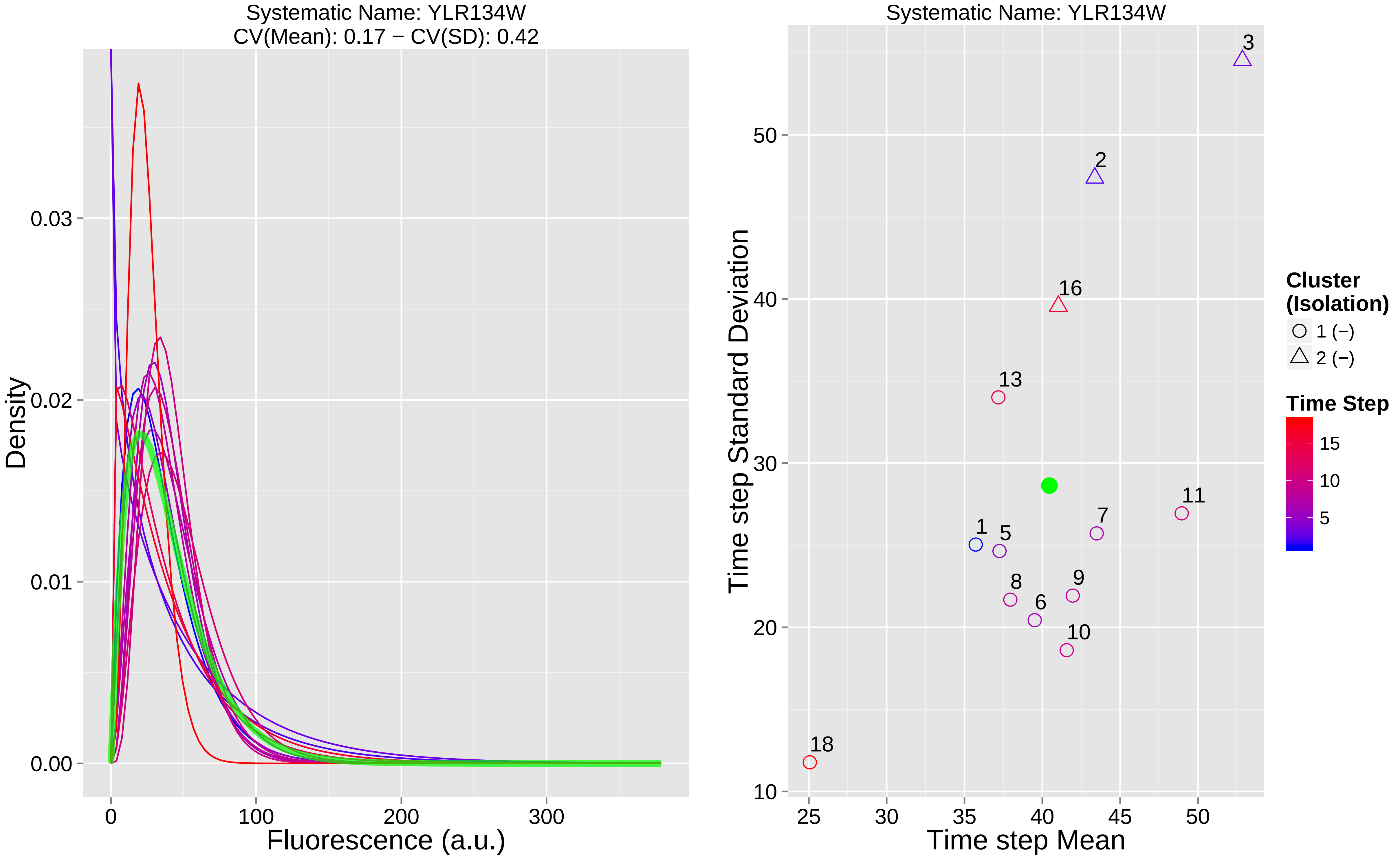
Systematic Name: YLR134W
CV(Mean): 0.17 − CV(SD): 0.42
Density
0.00
0.01
0.02
0.03
0
100
200
300
Fluorescence (a.u.)
Systematic Name: YLR134W
Time step Standard Deviation
10
20
30
40
50
25
30
35
40
45
50
Time step Mean
3
2
16
13
11
7
1
5
8
9
6
10
18
Cluster
(Isolation)
1 (−)
2 (−)
Time Step
15
10
5

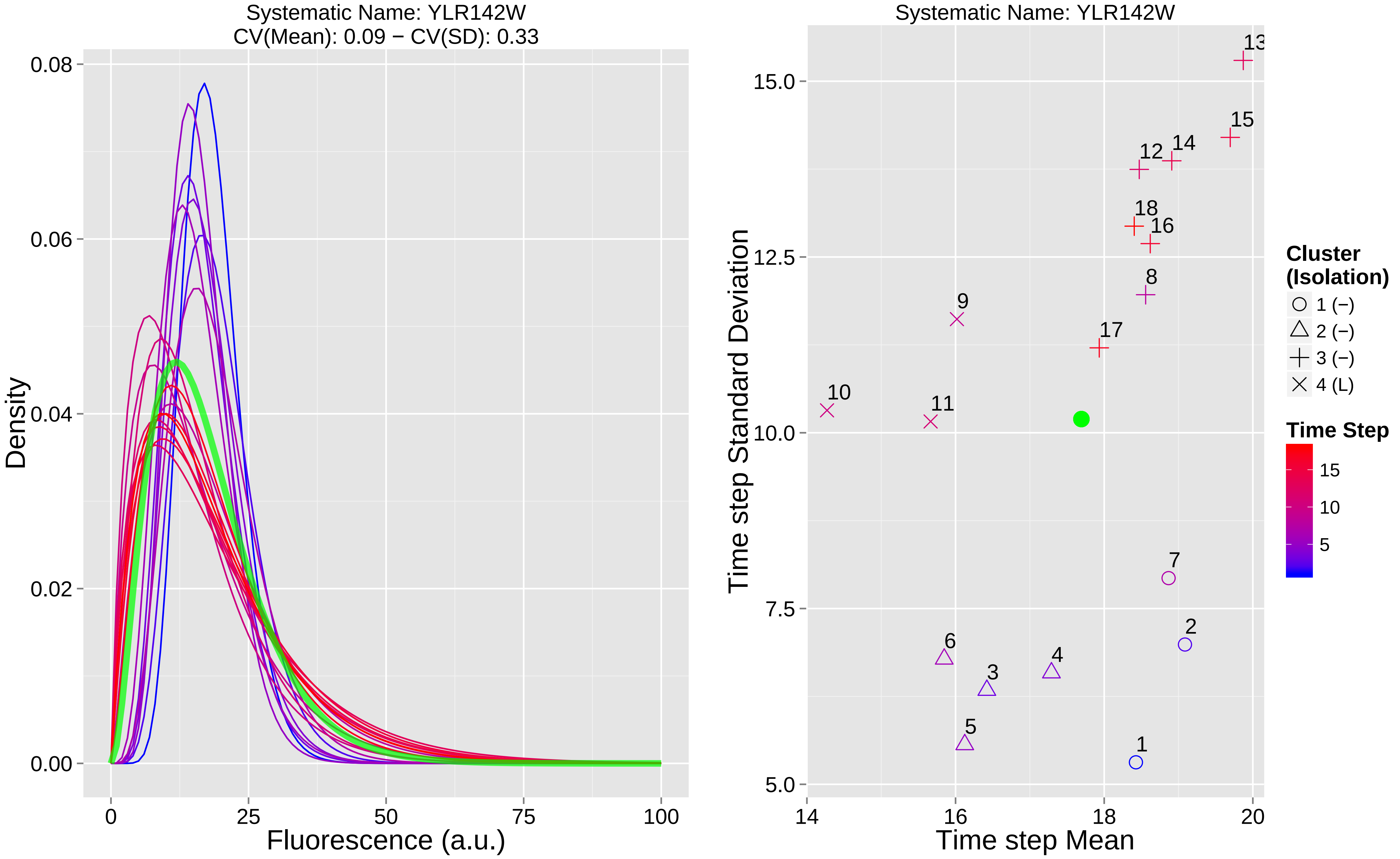

Systematic Name: YLR142W
CV(Mean): 0.09 − CV(SD): 0.33
Density
Fluorescence (a.u.)
0.00
0.02
0.04
0.06
0.08
0
25
50
75
100
Systematic Name: YLR142W
Time step Standard Deviation
Time step Mean
5.0
7.5
10.0
12.5
15.0
14
16
18
20
1
2
3
4
5
6
7
8
9
10
11
12
13
14
15
16
17
18
Cluster (Isolation)
1 (−)
2 (−)
3 (−)
4 (L)
Time Step
5
10
15

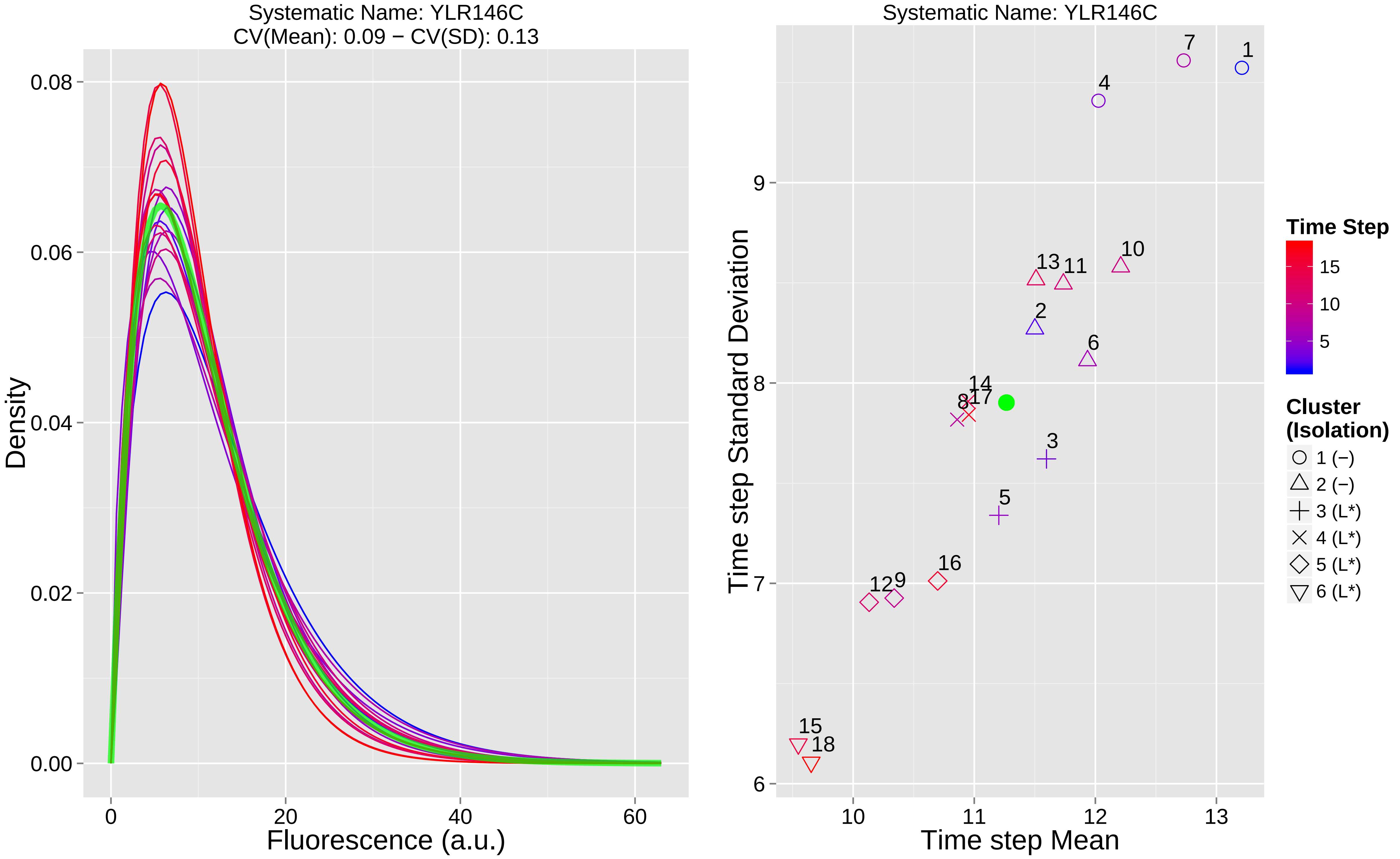

Systematic Name: YLR146C
CV(Mean): 0.09 − CV(SD): 0.13
Density
0.08
0.06
0.04
0.02
0.00
0
20
40
60
Fluorescence (a.u.)
Systematic Name: YLR146C
Time step Standard Deviation
9
8
7
6
10
11
12
13
Time step Mean
7
1
4
10
13
11
2
6
14
8
17
3
5
16
12
9
15
18
Time Step
15
10
5
Cluster
(Isolation)
1 (−)
2 (−)
3 (L*)
4 (L*)
5 (L*)
6 (L*)

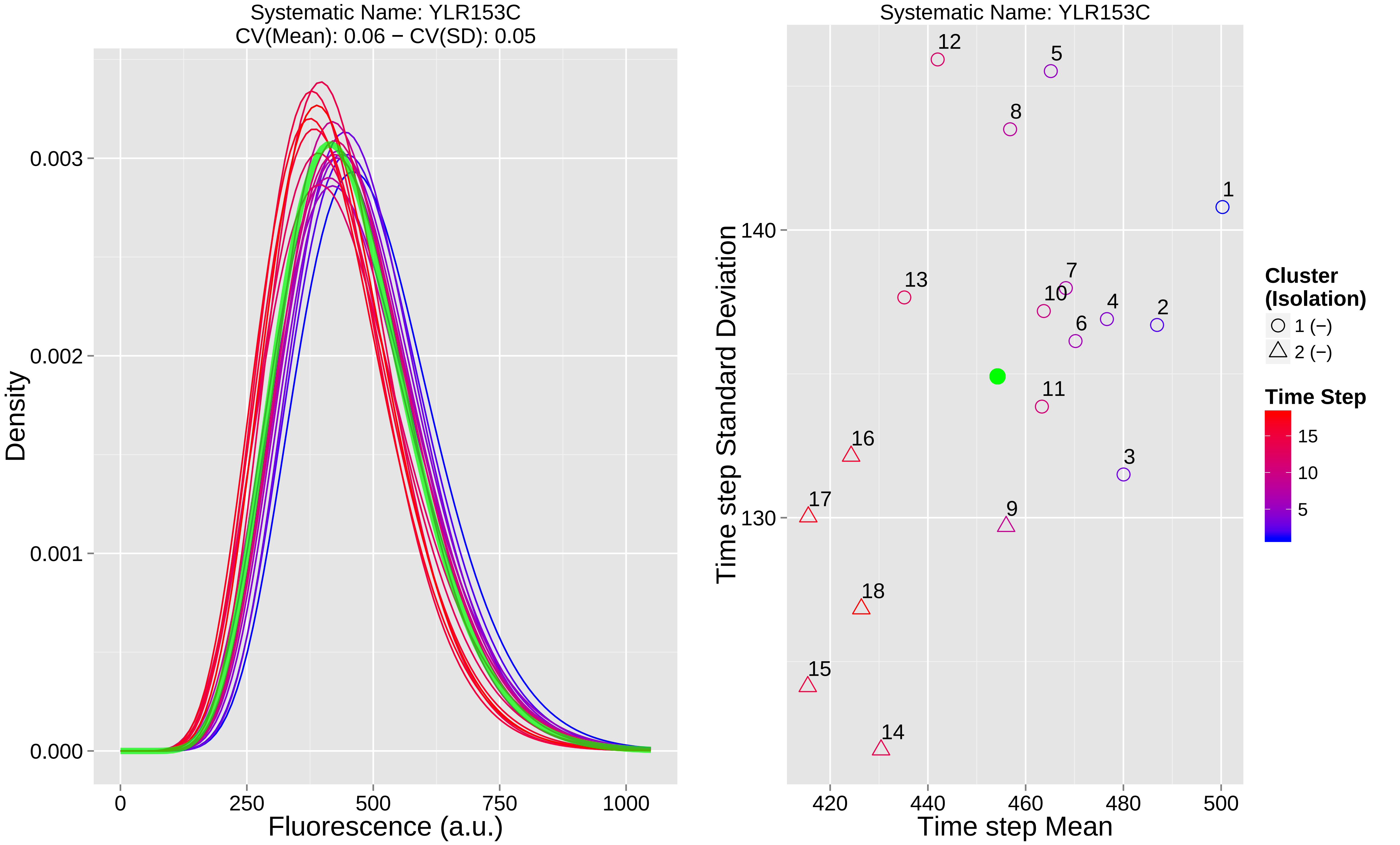

Systematic Name: YLR153C
CV(Mean): 0.06 − CV(SD): 0.05
Density
0.000
0.001
0.002
0.003
0
250
500
750
1000
Fluorescence (a.u.)
Systematic Name: YLR153C
Time step Standard Deviation
130
140
420
440
460
480
500
Time step Mean
1
2
3
4
5
6
7
8
9
10
11
12
13
14
15
16
17
18
Cluster (Isolation)
1 (−)
2 (−)
Time Step
15
10
5

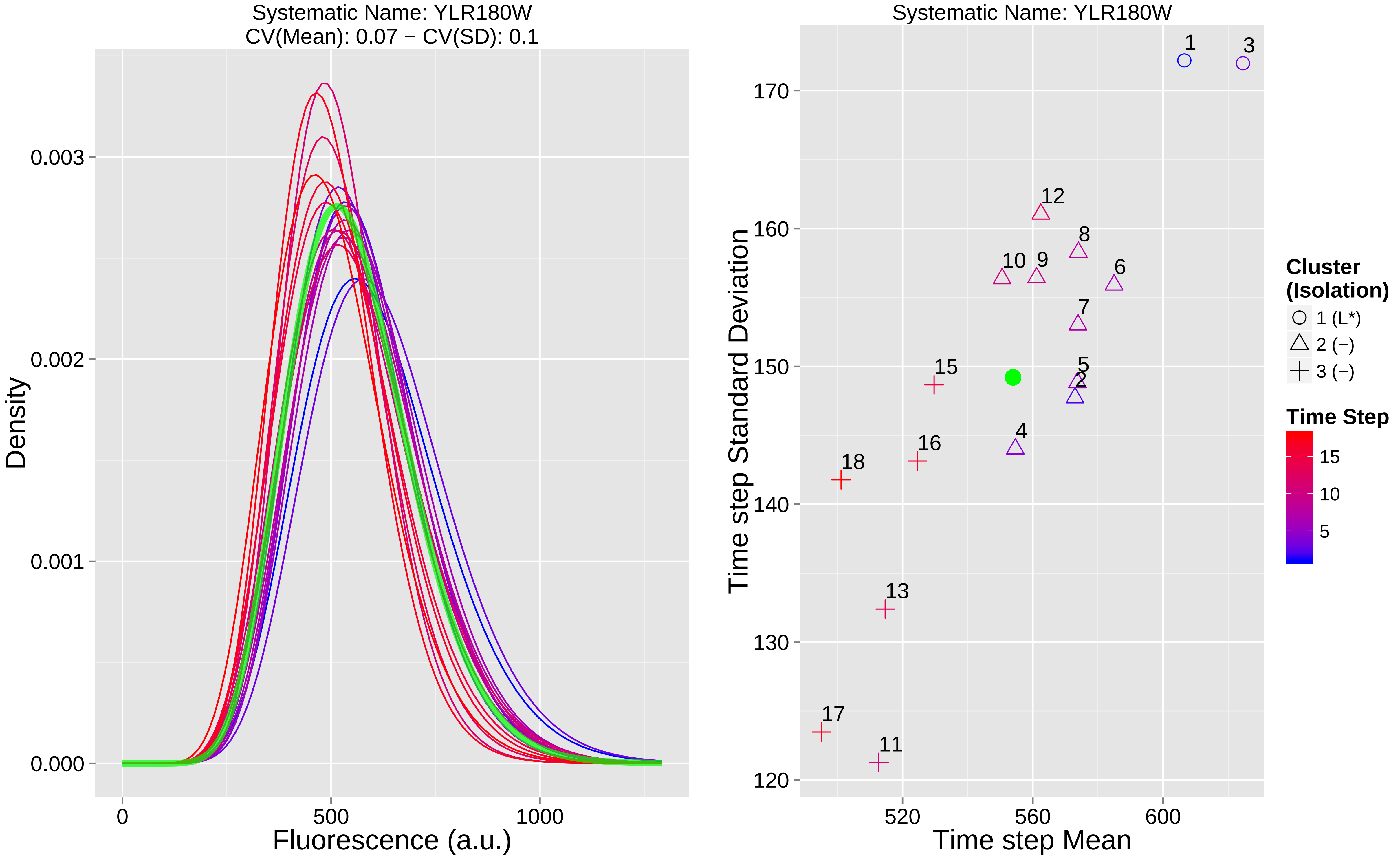
Systematic Name: YLR180W
CV(Mean): 0.07 − CV(SD): 0.1
Density
Fluorescence (a.u.)
0.000
0.001
0.002
0.003
0
500
1000
Systematic Name: YLR180W
Time step Standard Deviation
Time step Mean
120
130
140
150
160
170
520
560
600
1
2
3
4
5
6
7
8
9
10
11
12
13
15
16
17
18
Cluster (Isolation)
1 (L*)
2 (−)
3 (−)
Time Step
5
10
15

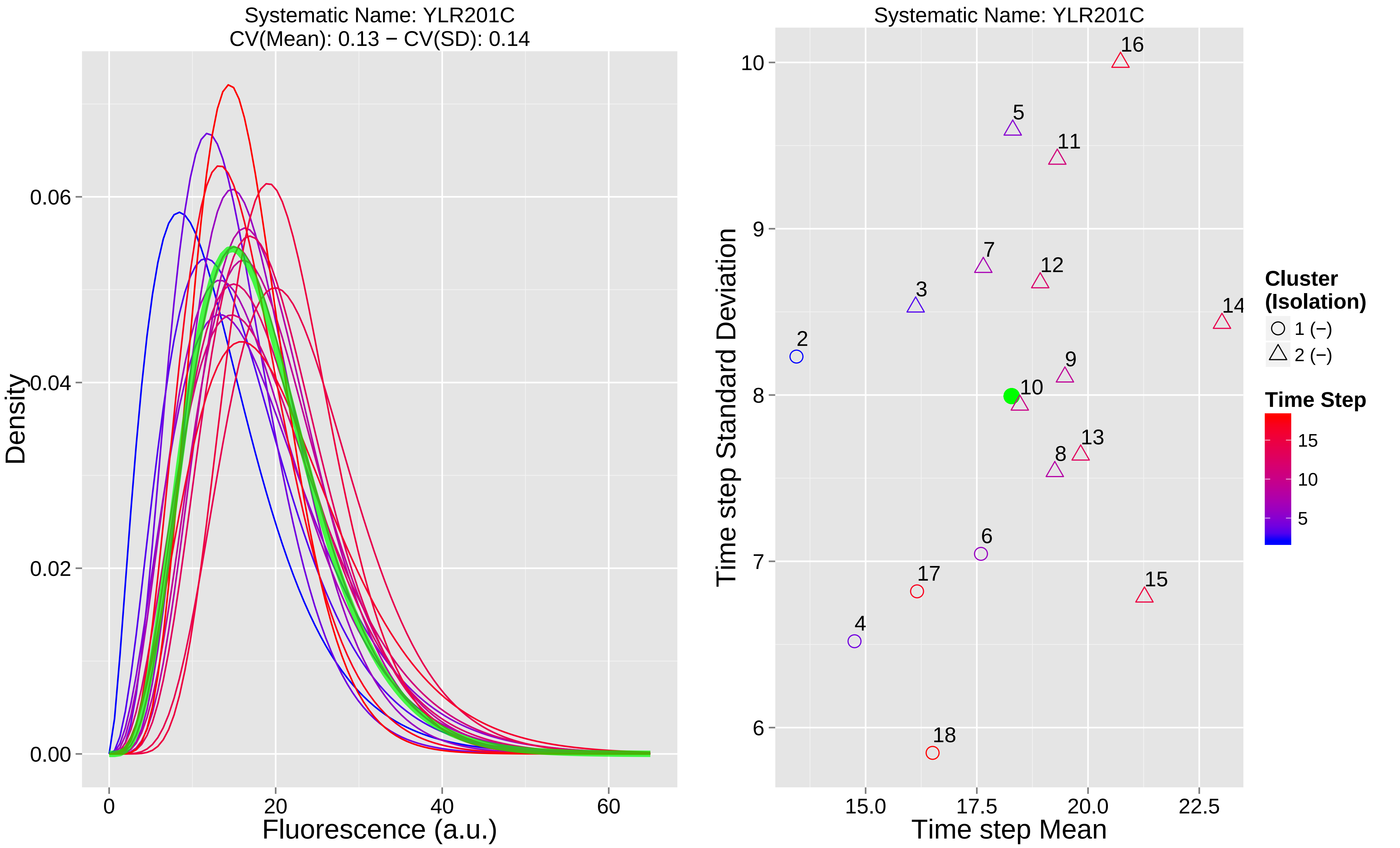

Systematic Name: YLR201C
CV(Mean): 0.13 − CV(SD): 0.14
Density
0.00
0.02
0.04
0.06
0
20
40
60
Fluorescence (a.u.)
Systematic Name: YLR201C
Time step Standard Deviation
6
7
8
9
10
15.0
17.5
20.0
22.5
Time step Mean
2
3
4
5
6
7
8
9
10
11
12
13
14
15
16
17
18
Cluster
(Isolation)
1 (−)
2 (−)
Time Step
15
10
5

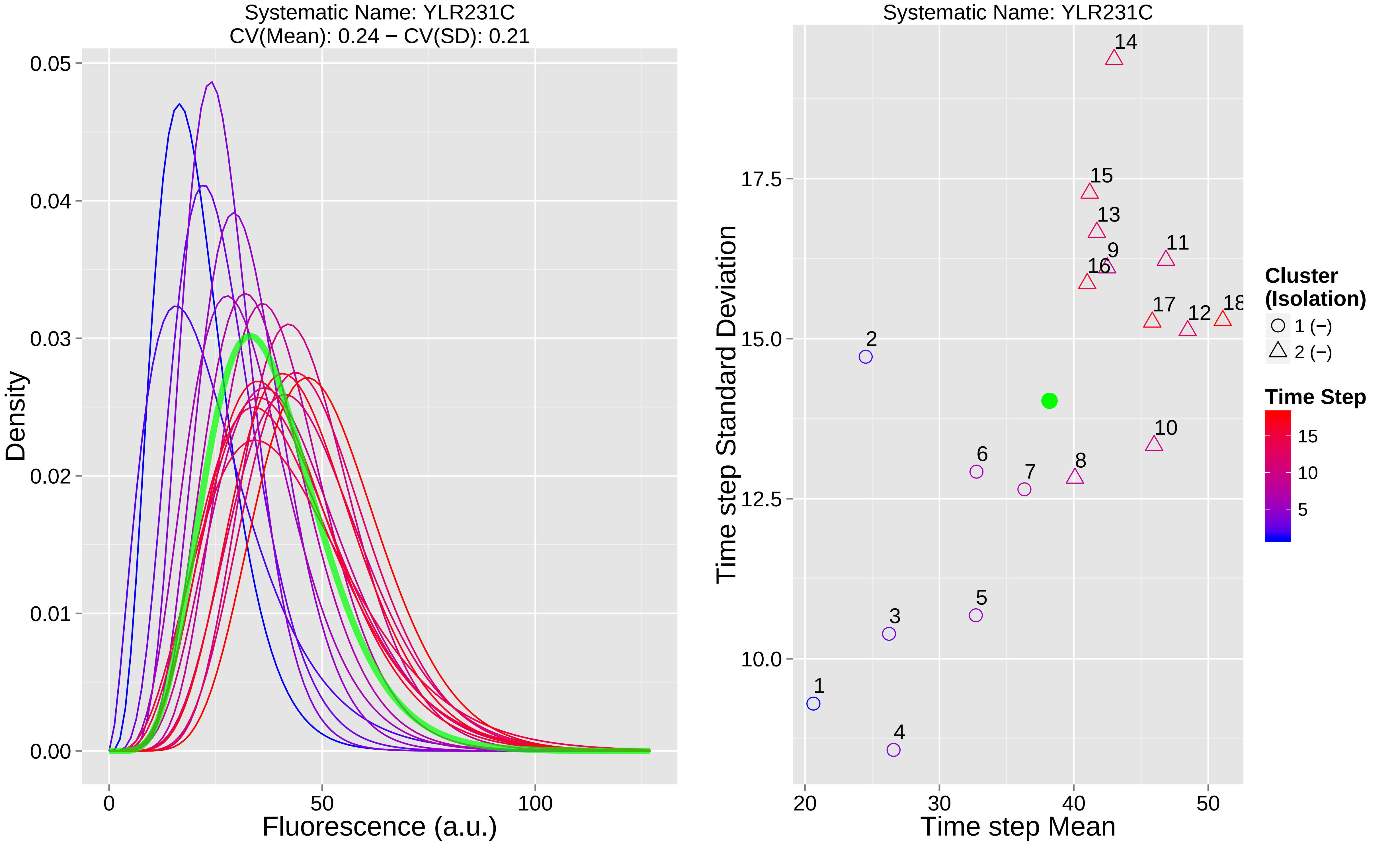
Systematic Name: YLR231C
CV(Mean): 0.24 − CV(SD): 0.21
Density
0.05
0.04
0.03
0.02
0.01
0.00
0
50
100
Fluorescence (a.u.)
Systematic Name: YLR231C
Time step Standard Deviation
17.5
15.0
12.5
10.0
20
30
40
50
Time step Mean
1
2
3
4
5
6
7
8
9
10
11
12
13
14
15
16
17
18
Cluster
(Isolation)
1 (−)
2 (−)
Time Step
15
10
5

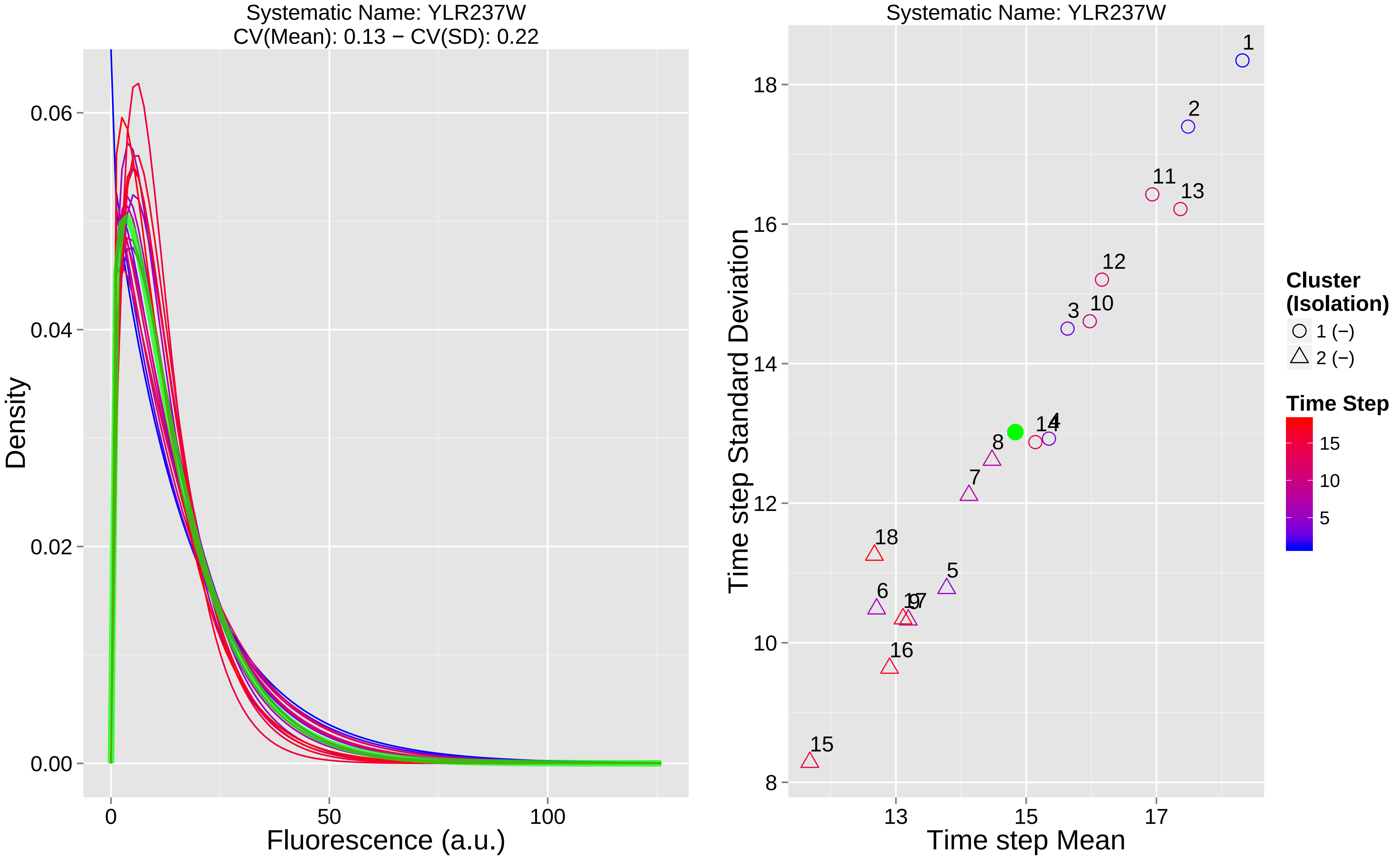

Systematic Name: YLR237W
CV(Mean): 0.13 − CV(SD): 0.22
Density
0.06
0.04
0.02
0.00
0
50
100
Fluorescence (a.u.)
Systematic Name: YLR237W
Time step Standard Deviation
18
16
14
12
10
8
13
15
17
Time step Mean
1
2
11
13
12
3
10
14
4
8
7
18
5
6
16
15
Cluster (Isolation)
1 (−)
2 (−)
Time Step
15
10
5

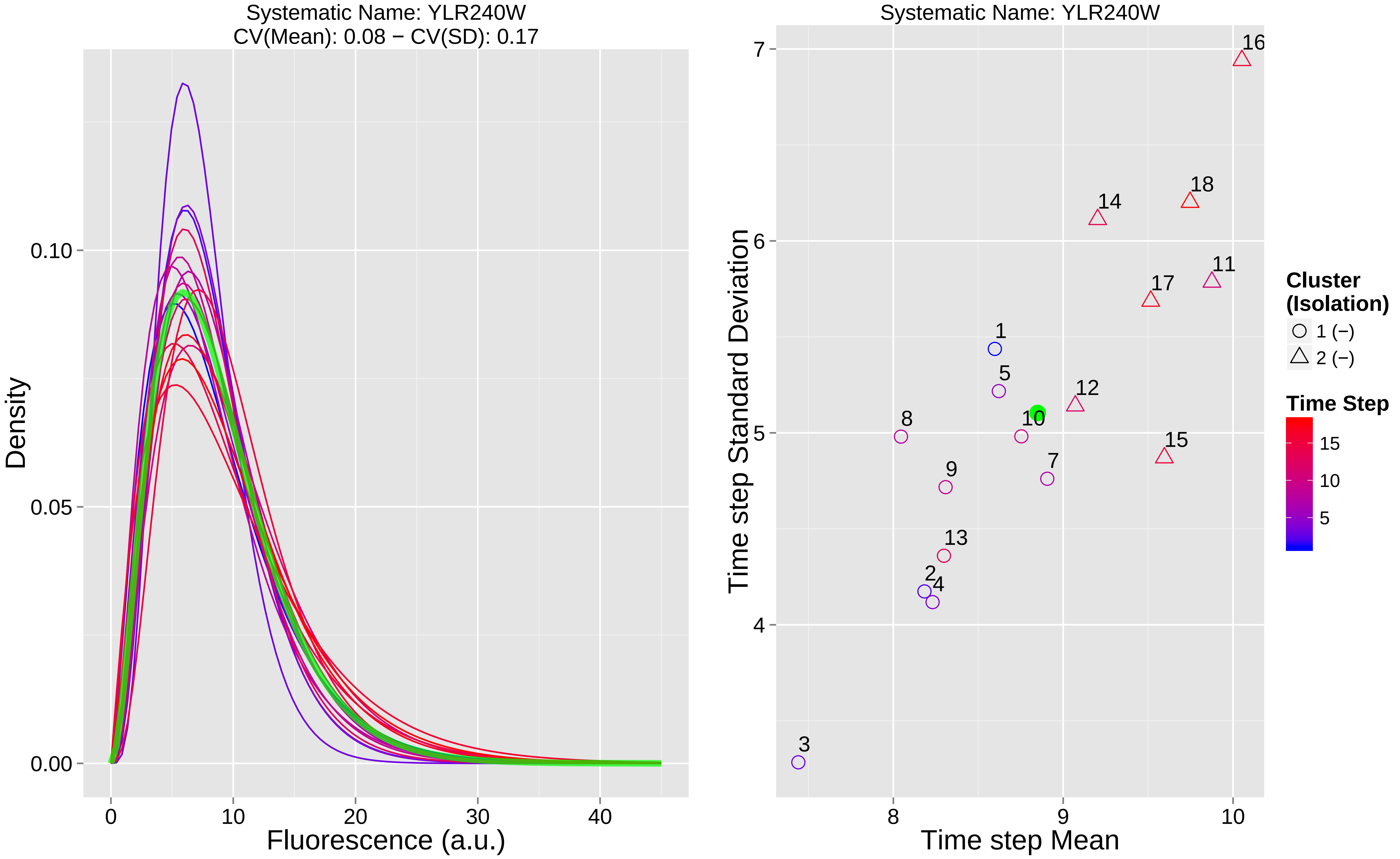
Systematic Name: YLR240W
CV(Mean): 0.08 − CV(SD): 0.17
Density
0.10
0.05
0.00
0
10
20
30
40
Fluorescence (a.u.)
Systematic Name: YLR240W
Time step Standard Deviation
7
6
5
4
8
9
10
Time step Mean
16
18
14
11
17
1
5
12
8
10
15
7
9
13
2
4
3
Cluster
(Isolation)
1 (−)
2 (−)
Time Step
15
10
5

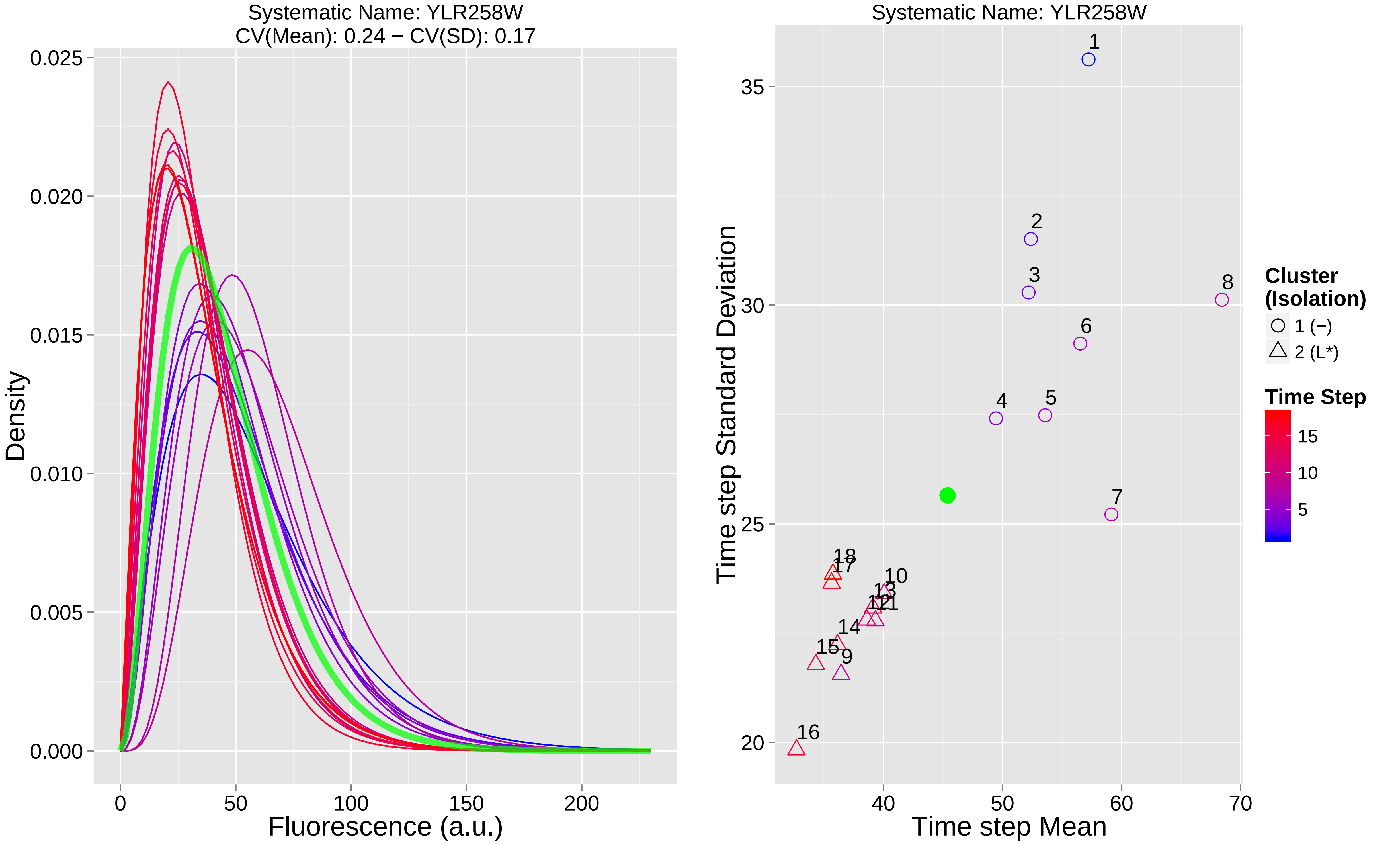

Systematic Name: YLR258W
CV(Mean): 0.24 − CV(SD): 0.17
Density
Fluorescence (a.u.)
0.000
0.005
0.010
0.015
0.020
0.025
0
50
100
150
200
Systematic Name: YLR258W
Time step Standard Deviation
Time step Mean
20
25
30
35
40
50
60
70
Cluster (Isolation)
1 (−)
2 (L*)
Time Step
5
10
15

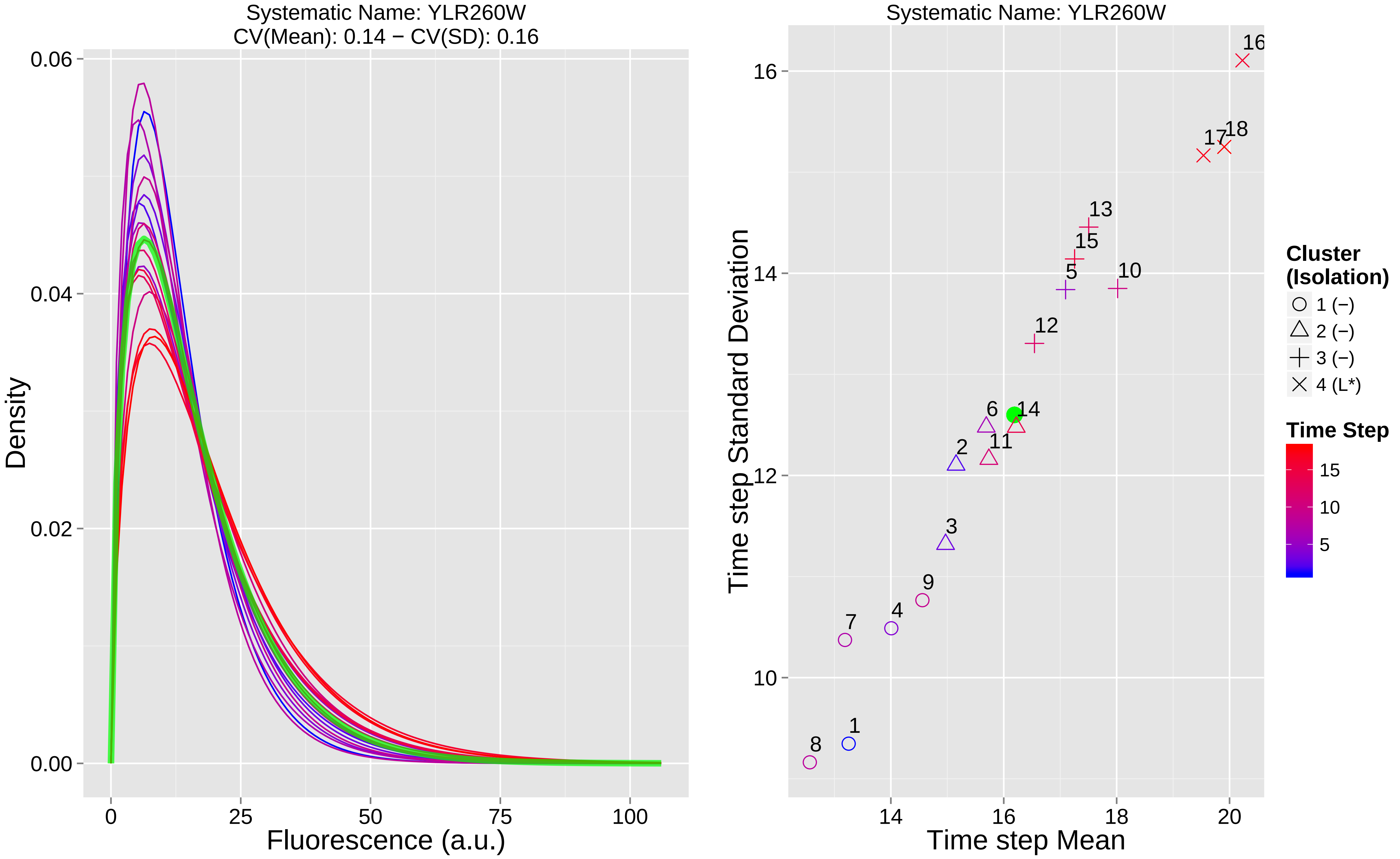

Systematic Name: YLR260W
CV(Mean): 0.14 − CV(SD): 0.16
Density
0.06
0.04
0.02
0.00
0
25
50
75
100
Fluorescence (a.u.)
Systematic Name: YLR260W
Time step Standard Deviation
16
14
12
10
14
16
18
20
Time step Mean
1
2
3
4
5
6
7
8
9
10
11
12
13
14
15
16
17
18
Cluster (Isolation)
1 (−)
2 (−)
3 (−)
4 (L*)
Time Step
15
10
5

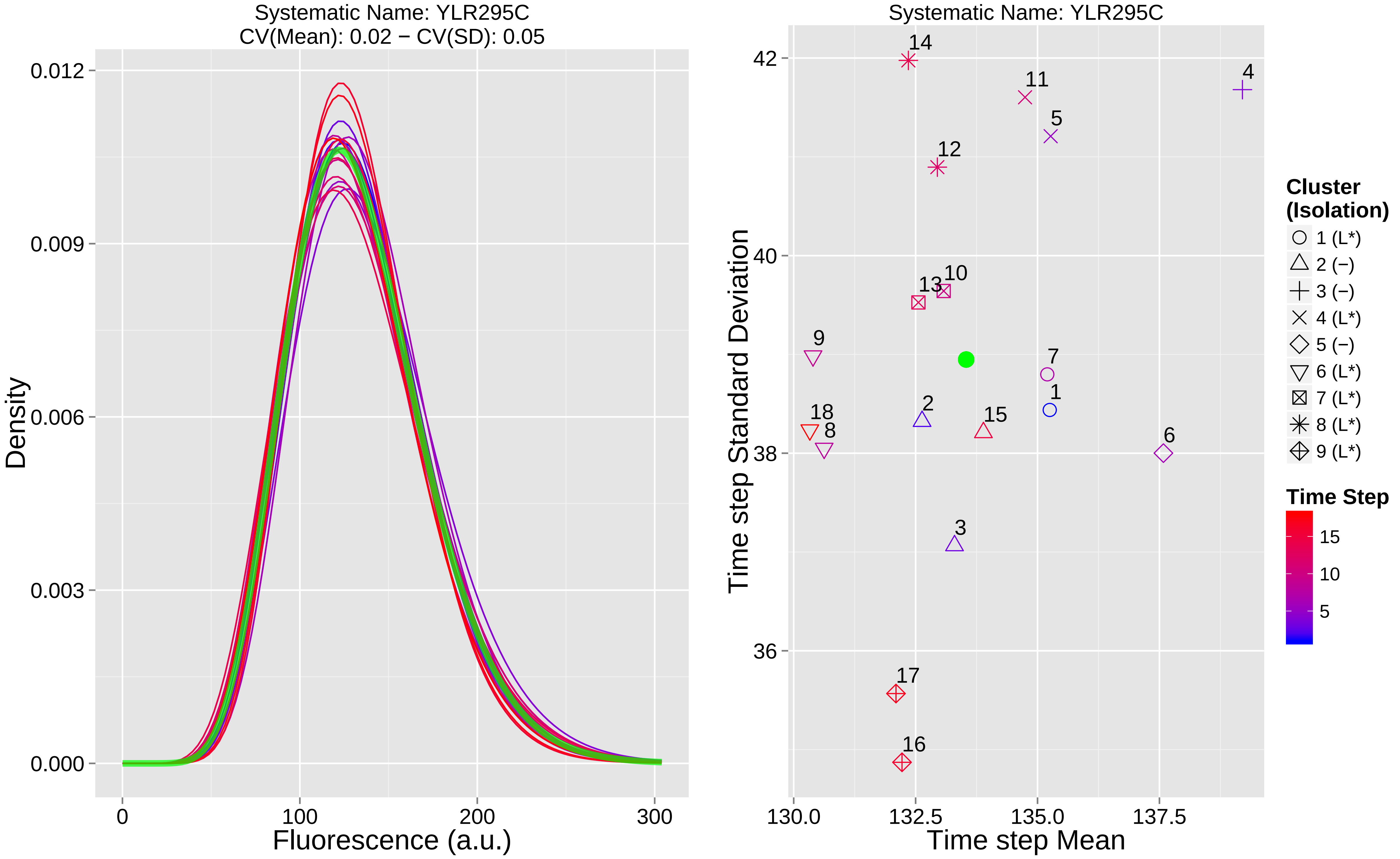

Systematic Name: YLR295C
CV(Mean): 0.02 − CV(SD): 0.05
Density
0.012
0.009
0.006
0.003
0.000
0
100
200
300
Fluorescence (a.u.)
Systematic Name: YLR295C
Time step Standard Deviation
42
40
38
36
130.0
132.5
135.0
137.5
Time step Mean
14
11
4
5
12
13
10
9
7
1
2
15
18
8
6
3
17
16
Cluster (Isolation)
1 (L*)
2 (−)
3 (−)
4 (L*)
5 (−)
6 (L*)
7 (L*)
8 (L*)
9 (L*)
Time Step
15
10
5

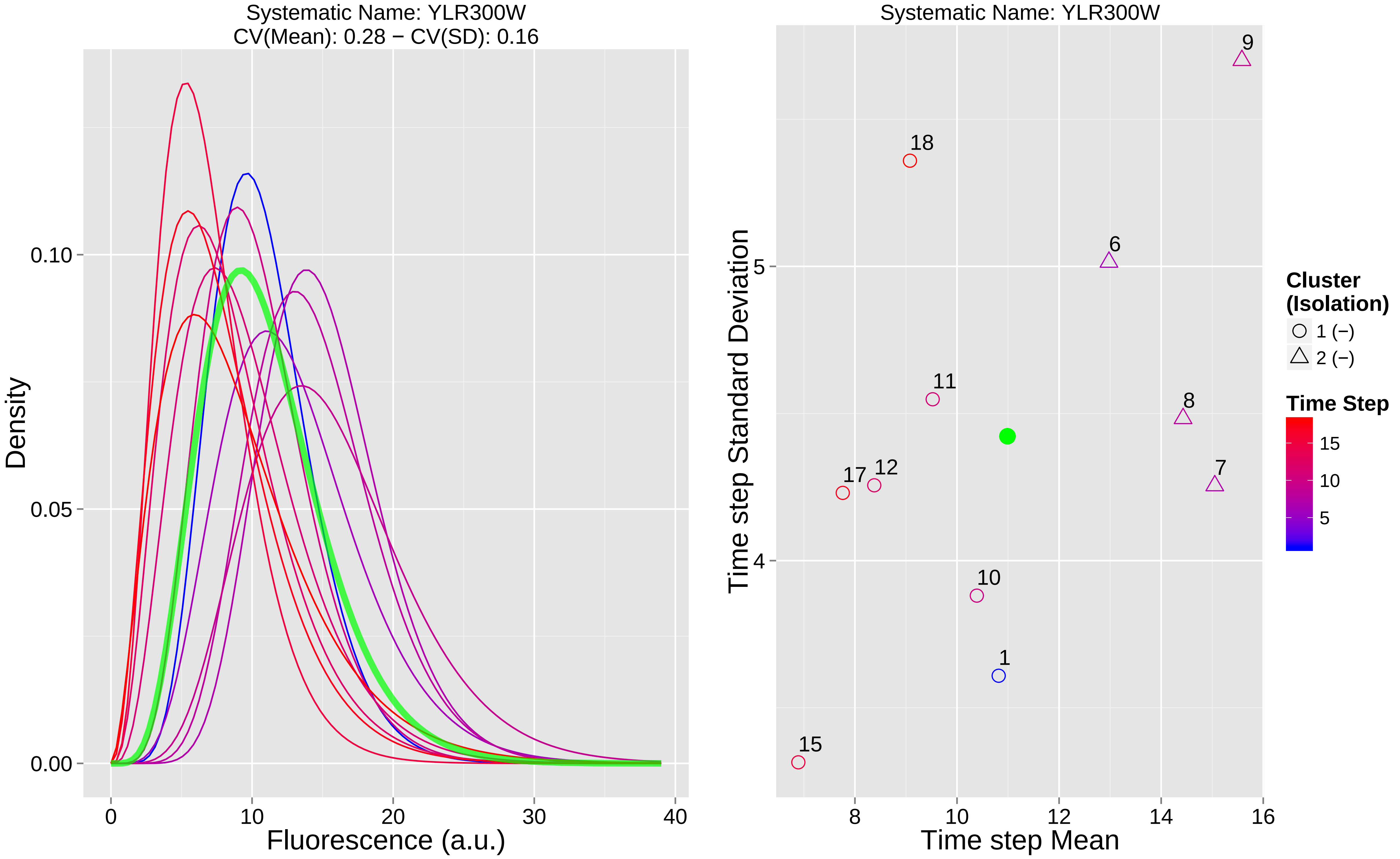
Systematic Name: YLR300W
CV(Mean): 0.28 − CV(SD): 0.16
Density
0.10
0.05
0.00
0
10
20
30
40
Fluorescence (a.u.)
Systematic Name: YLR300W
Time step Standard Deviation
Time step Mean
5
4
8
10
12
14
16
9
18
6
11
8
7
17
12
10
1
15
Cluster (Isolation)
1 (−)
2 (−)
Time Step
15
10
5

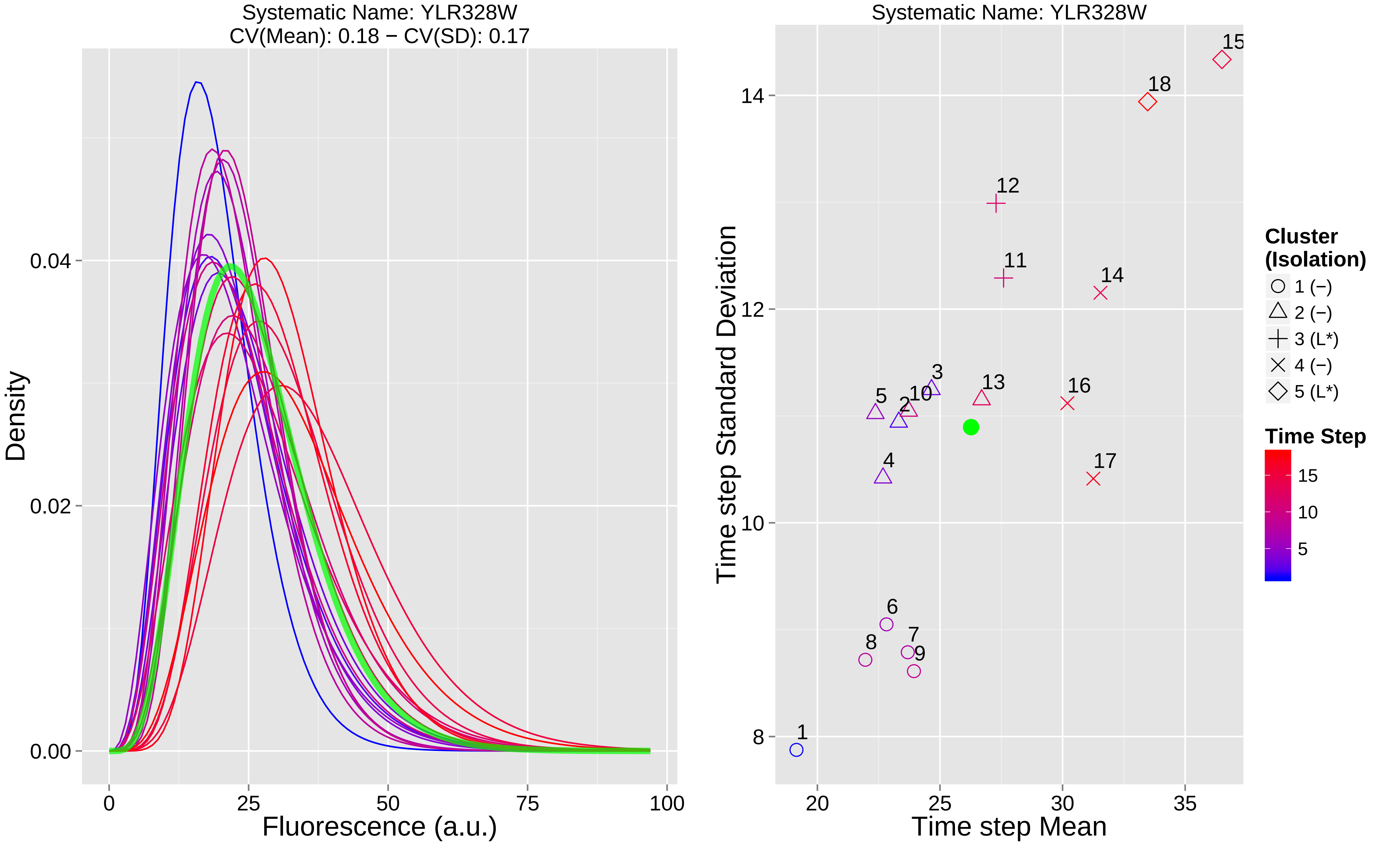

Systematic Name: YLR328W
CV(Mean): 0.18 − CV(SD): 0.17
Density
0.04
0.02
0.00
0
25
50
75
100
Fluorescence (a.u.)
Systematic Name: YLR328W
Time step Standard Deviation
14
12
10
8
20
25
30
35
Time step Mean
1
2
3
4
5
6
7
8
9
10
11
12
13
14
15
16
17
18
Cluster (Isolation)
1 (−)
2 (−)
3 (L*)
4 (−)
5 (L*)
Time Step
15
10
5

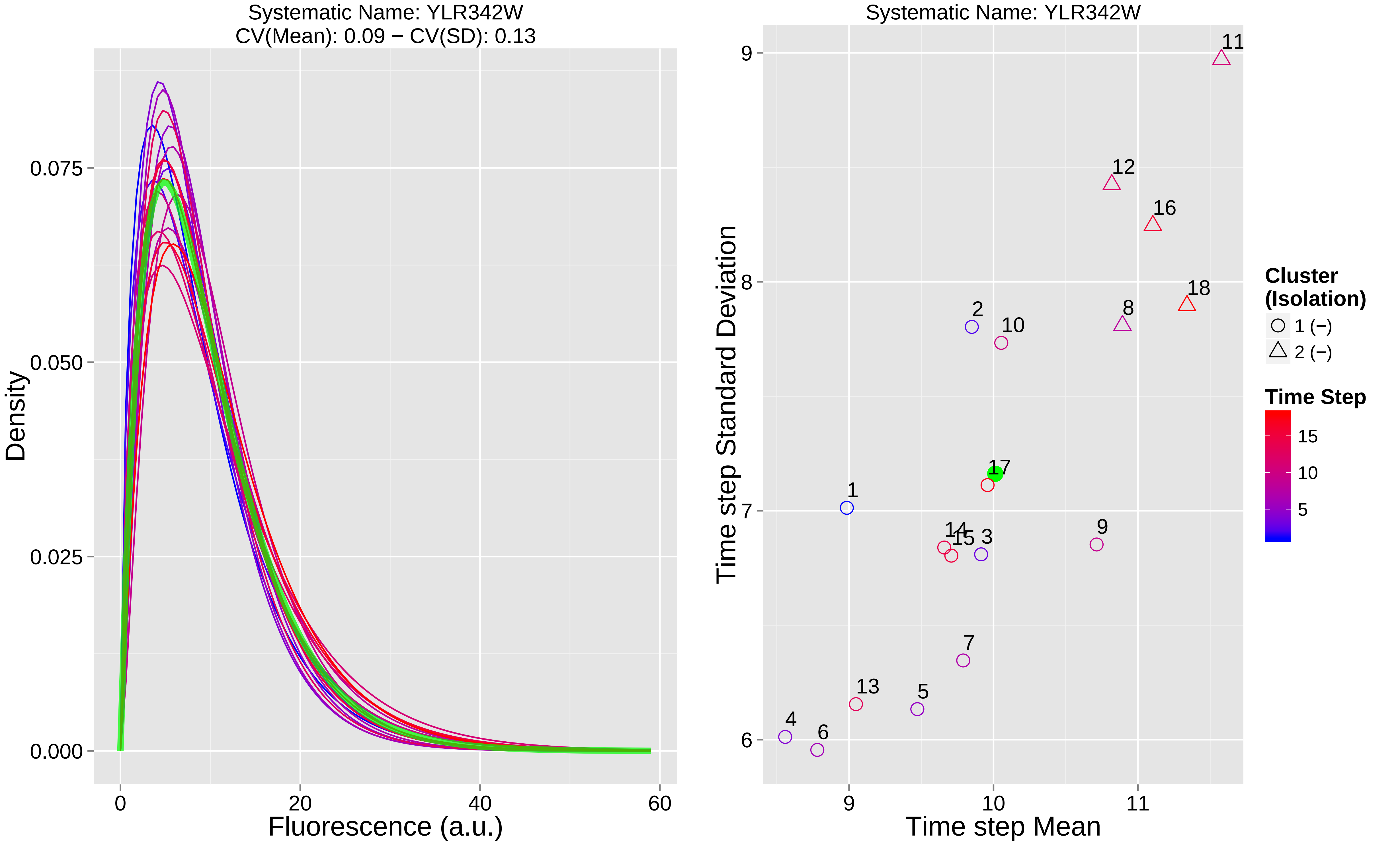

Systematic Name: YLR342W
CV(Mean): 0.09 − CV(SD): 0.13
Density
0.000
0.025
0.050
0.075
0
20
40
60
Fluorescence (a.u.)
Systematic Name: YLR342W
Time step Standard Deviation
Time step Mean
6
7
8
9
9
10
11
1
2
3
4
5
6
7
8
9
10
11
12
13
14
15
16
17
18
Cluster (Isolation)
1 (−)
2 (−)
Time Step
5
10
15

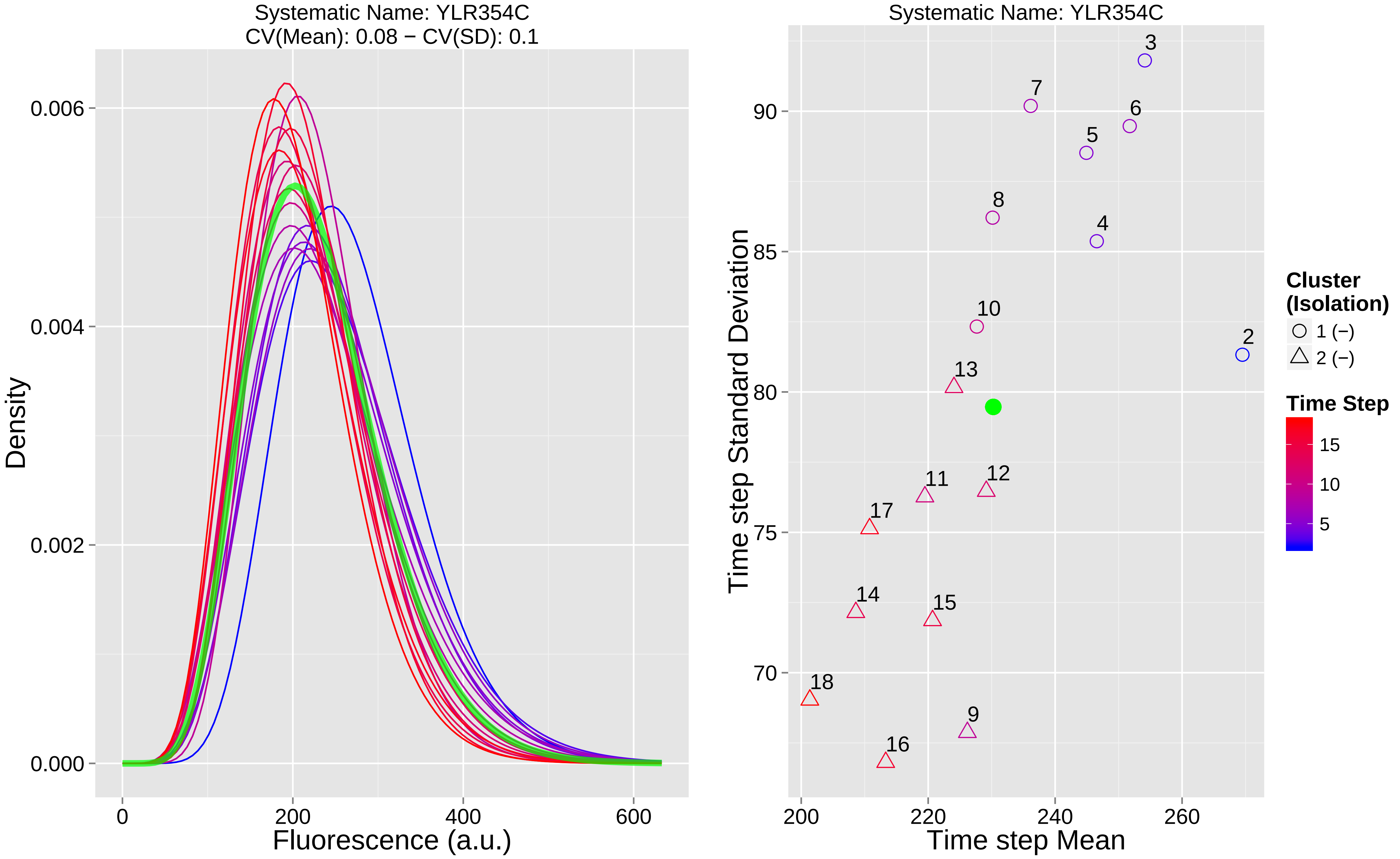
Systematic Name: YLR354C
CV(Mean): 0.08 − CV(SD): 0.1
Density
0.006
0.004
0.002
0.000
0
200
400
600
Fluorescence (a.u.)
Systematic Name: YLR354C
Time step Standard Deviation
90
85
80
75
70
200
220
240
260
Time step Mean
3
7
6
5
8
4
10
2
13
11
12
17
14
15
18
9
16
Cluster
(Isolation)
1 (−)
2 (−)
Time Step
15
10
5

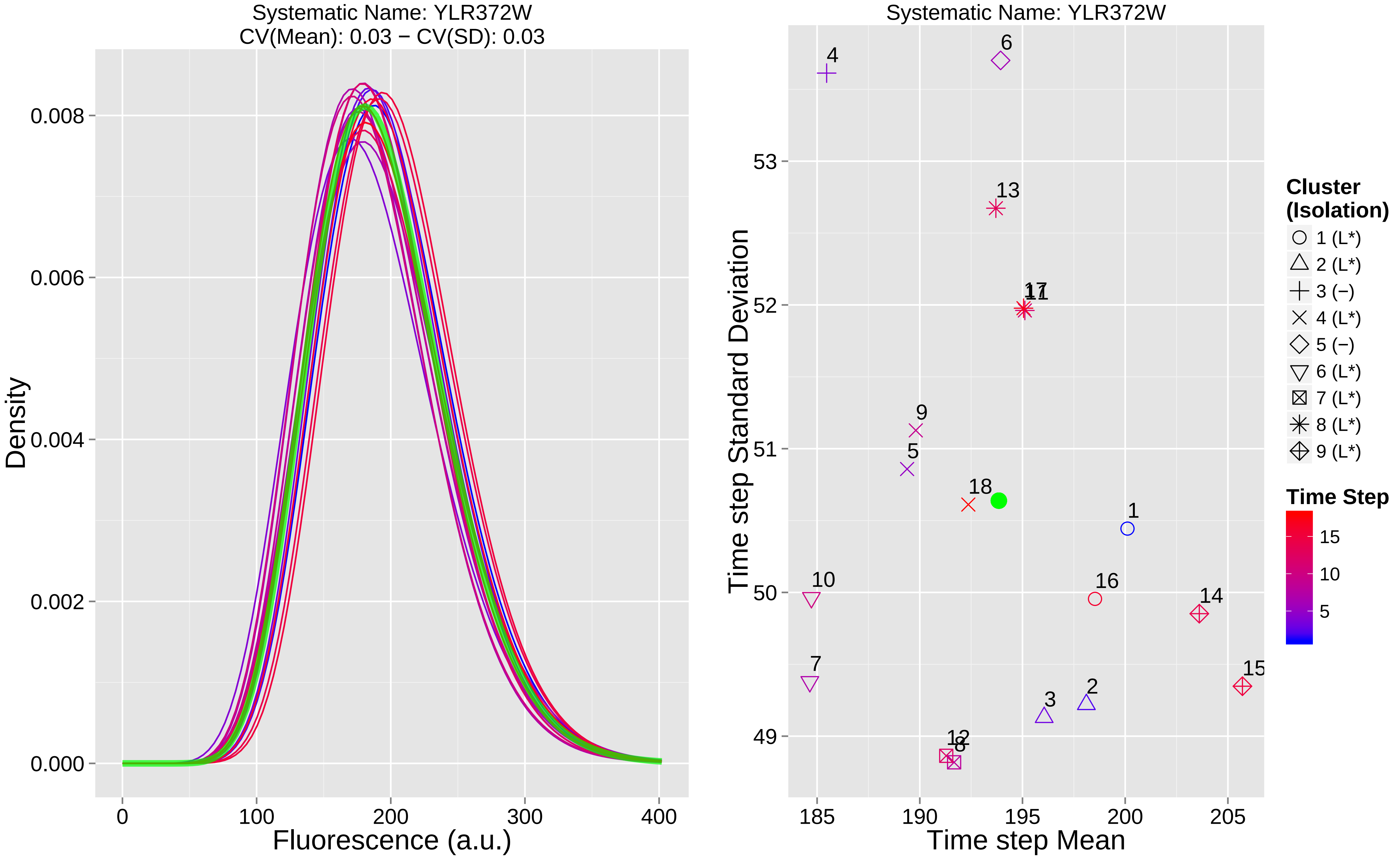

Systematic Name: YLR372W
CV(Mean): 0.03 − CV(SD): 0.03
Density
Fluorescence (a.u.)
0.000
0.002
0.004
0.006
0.008
0
100
200
300
400
Systematic Name: YLR372W
Time step Standard Deviation
Time step Mean
49
50
51
52
53
185
190
195
200
205
Cluster (Isolation)
1 (L*)
2 (L*)
3 (−)
4 (L*)
5 (−)
6 (L*)
7 (L*)
8 (L*)
9 (L*)
Time Step
5
10
15

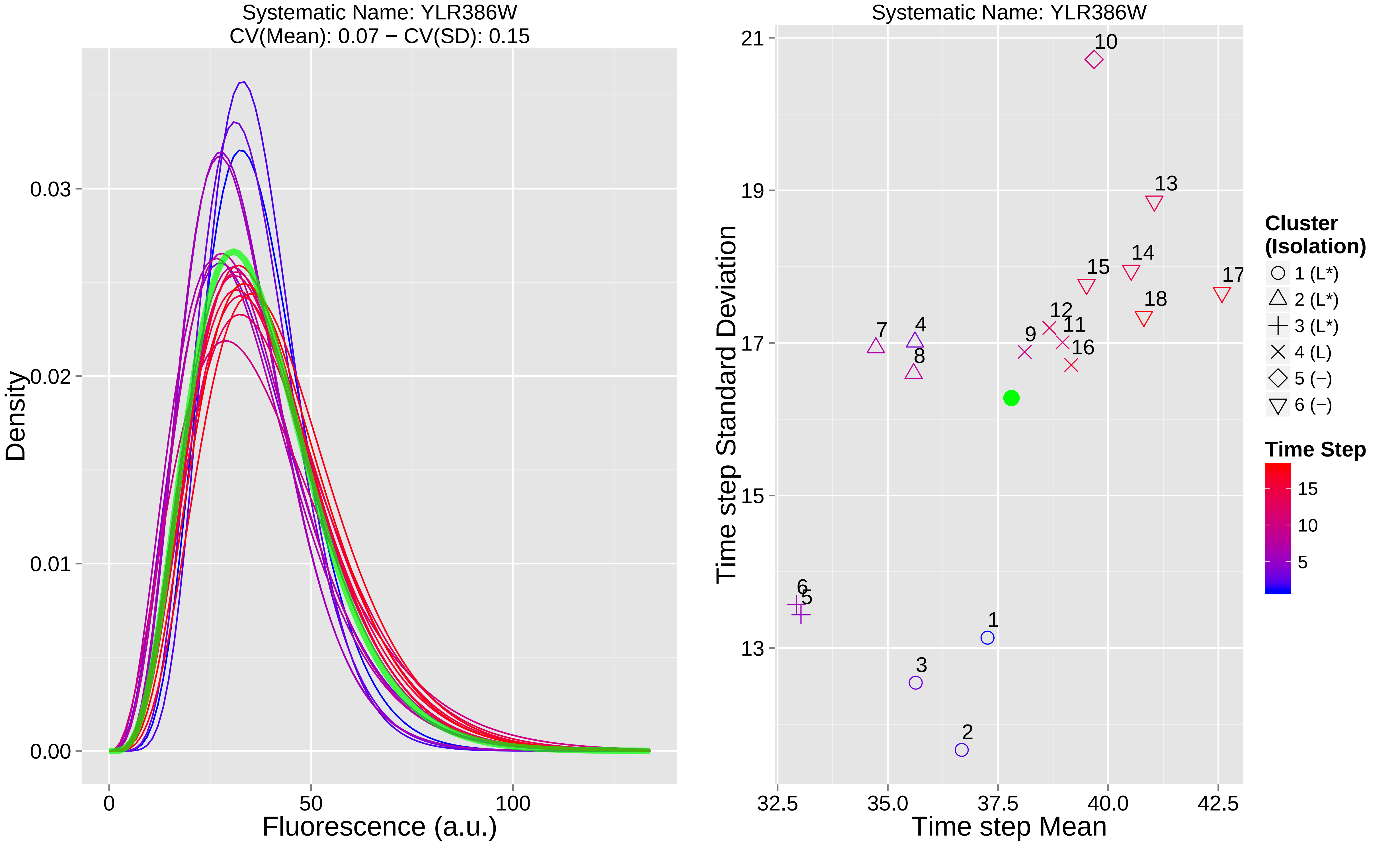

Systematic Name: YLR386W
CV(Mean): 0.07 − CV(SD): 0.15
Density
0.00
0.01
0.02
0.03
0
50
100
Fluorescence (a.u.)
Systematic Name: YLR386W
Time step Standard Deviation
13
15
17
19
21
32.5
35.0
37.5
40.0
42.5
Time step Mean
Cluster (Isolation)
1 (L*)
2 (L*)
3 (L*)
4 (L)
5 (−)
6 (−)
Time Step
5
10
15

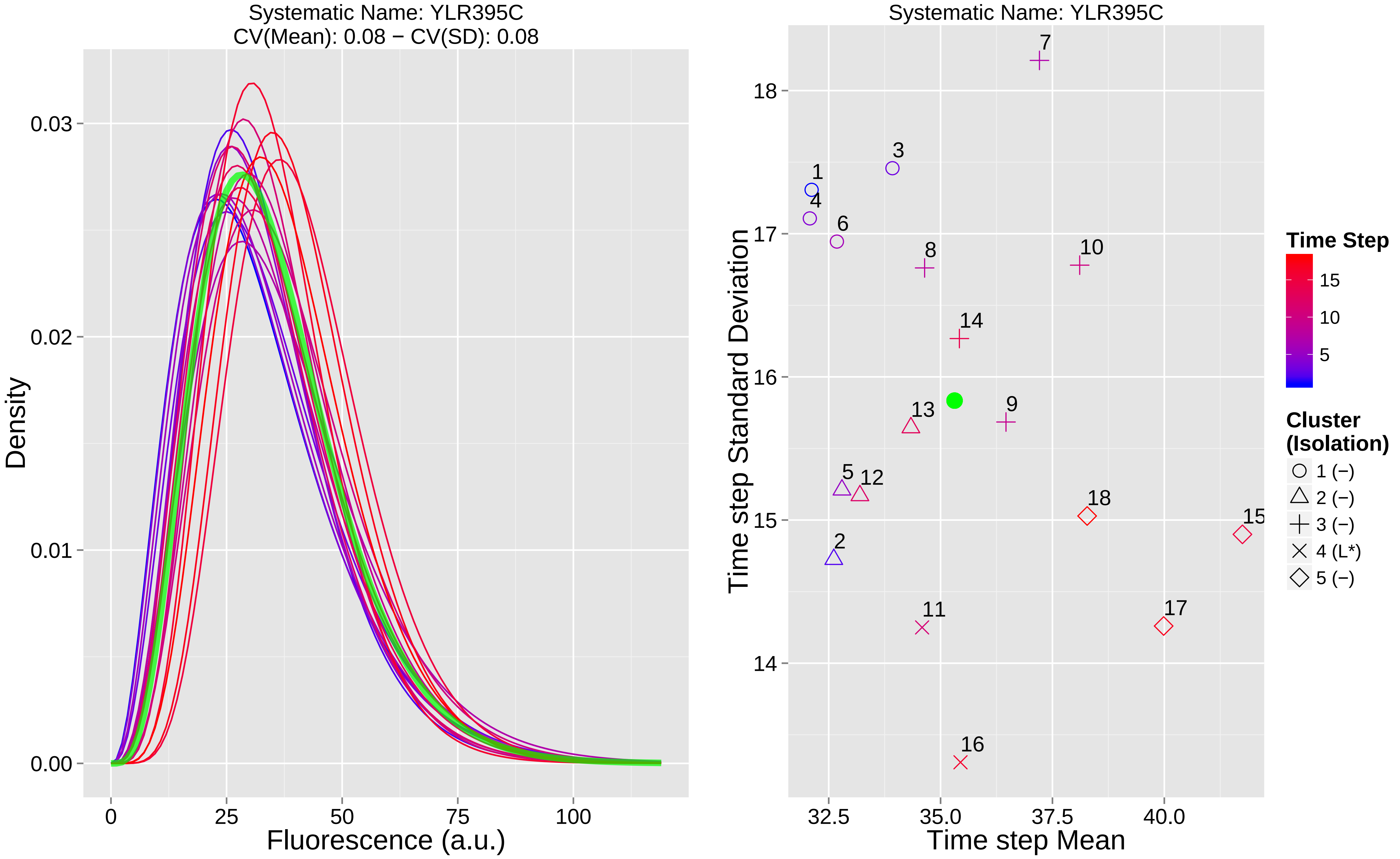

Systematic Name: YLR395C
CV(Mean): 0.08 − CV(SD): 0.08
Density
Fluorescence (a.u.)
0.00
0.01
0.02
0.03
0
25
50
75
100
Systematic Name: YLR395C
Time step Standard Deviation
Time step Mean
14
15
16
17
18
32.5
35.0
37.5
40.0
1
2
3
4
5
6
7
8
9
10
11
12
13
14
15
16
17
18
Time Step
15
10
5
Cluster
(Isolation)
1 (−)
2 (−)
3 (−)
4 (L*)
5 (−)

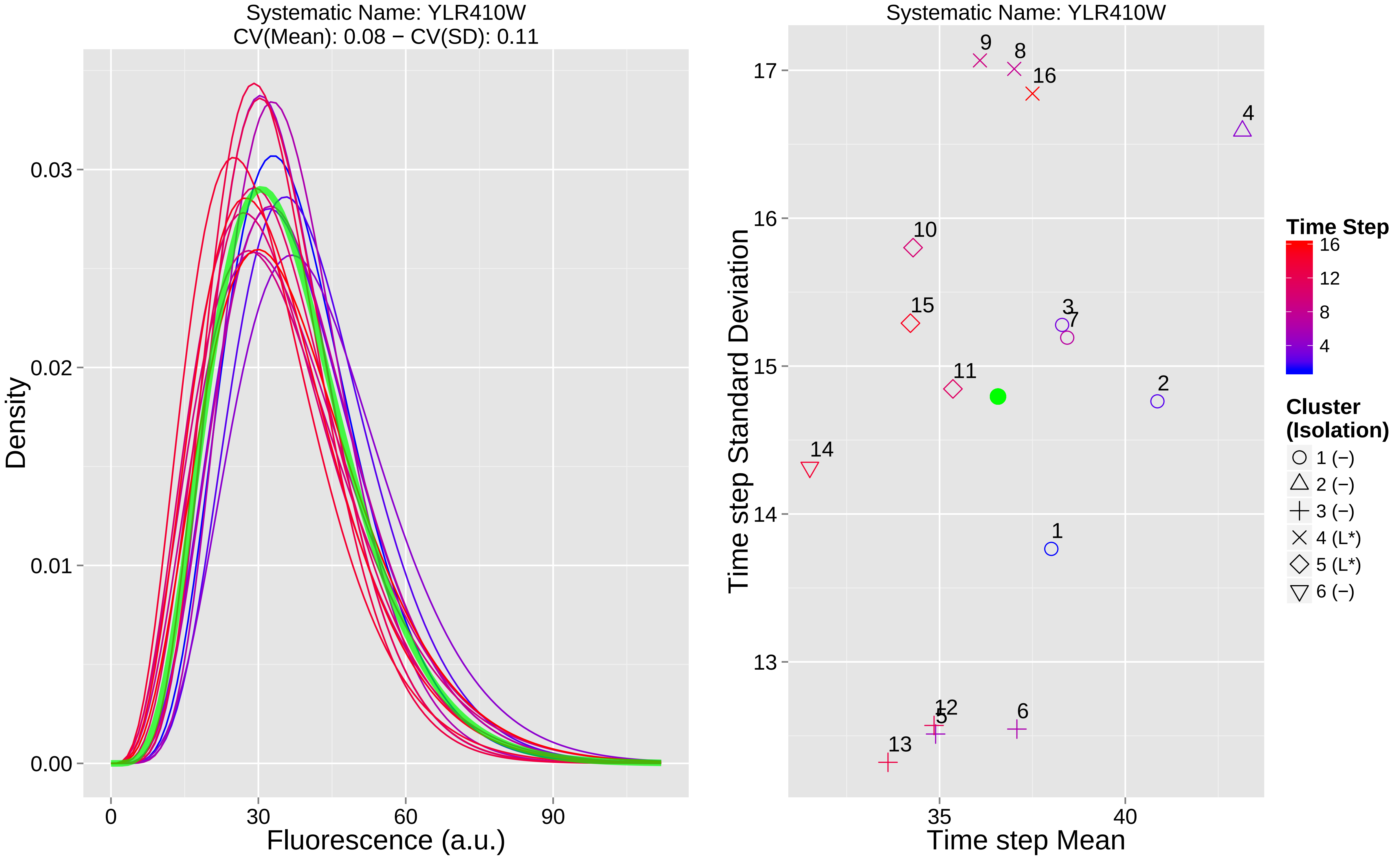

Systematic Name: YLR410W
CV(Mean): 0.08 − CV(SD): 0.11
Density
0.00
0.01
0.02
0.03
0
30
60
90
Fluorescence (a.u.)
Systematic Name: YLR410W
Time step Standard Deviation
13
14
15
16
17
35
40
Time step Mean
9
8
16
4
10
15
3
7
11
2
14
1
12
5
6
13
Time Step
16
12
8
4
Cluster
(Isolation)
1 (−)
2 (−)
3 (−)
4 (L*)
5 (L*)
6 (−)

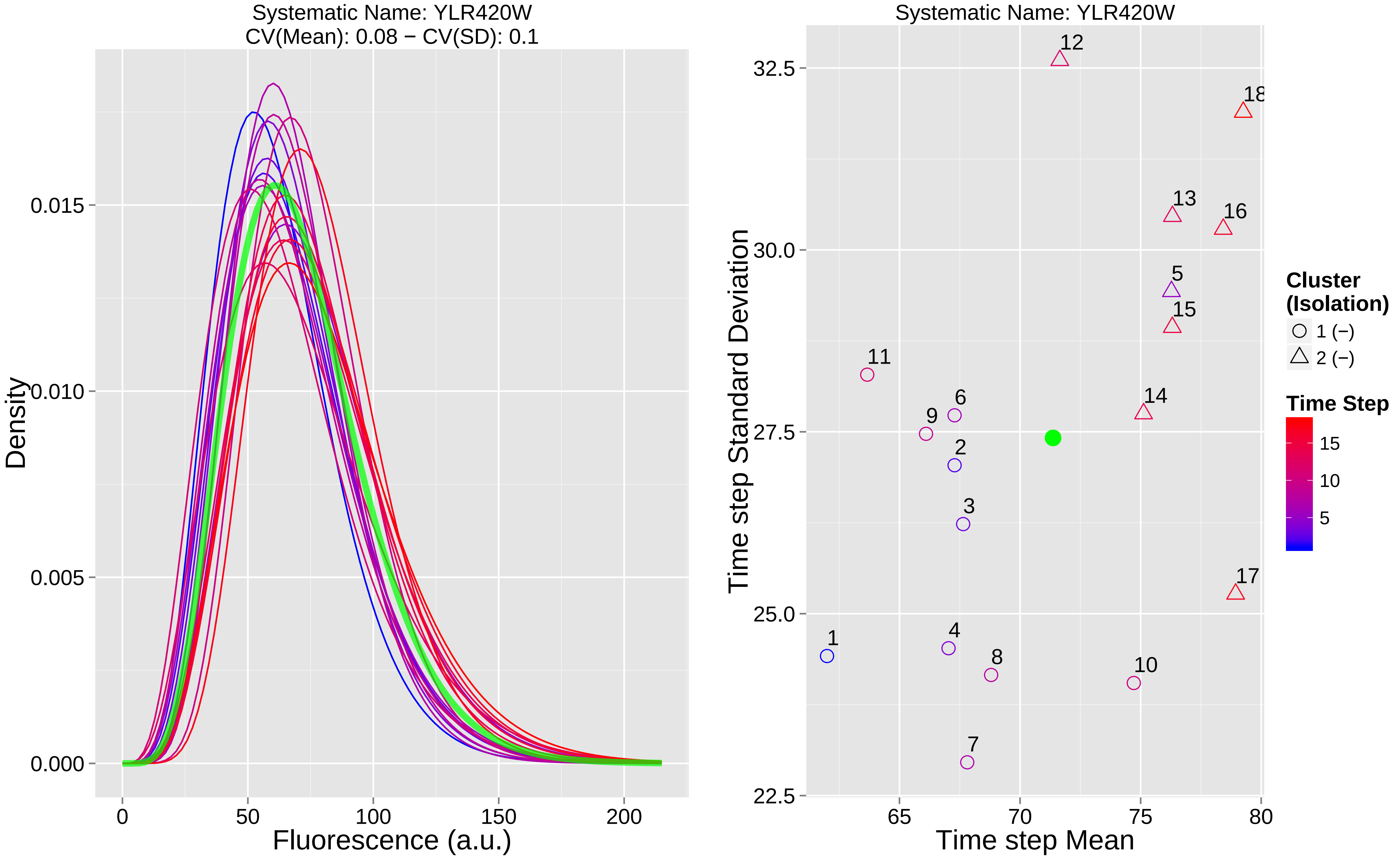
Systematic Name: YLR420W
CV(Mean): 0.08 − CV(SD): 0.1
Density
Fluorescence (a.u.)
0.000
0.005
0.010
0.015
0
50
100
150
200
Systematic Name: YLR420W
Time step Standard Deviation
Time step Mean
22.5
25.0
27.5
30.0
32.5
65
70
75
80
1
2
3
4
5
6
7
8
9
10
11
12
13
14
15
16
17
18
Cluster (Isolation)
1 (−)
2 (−)
Time Step
5
10
15

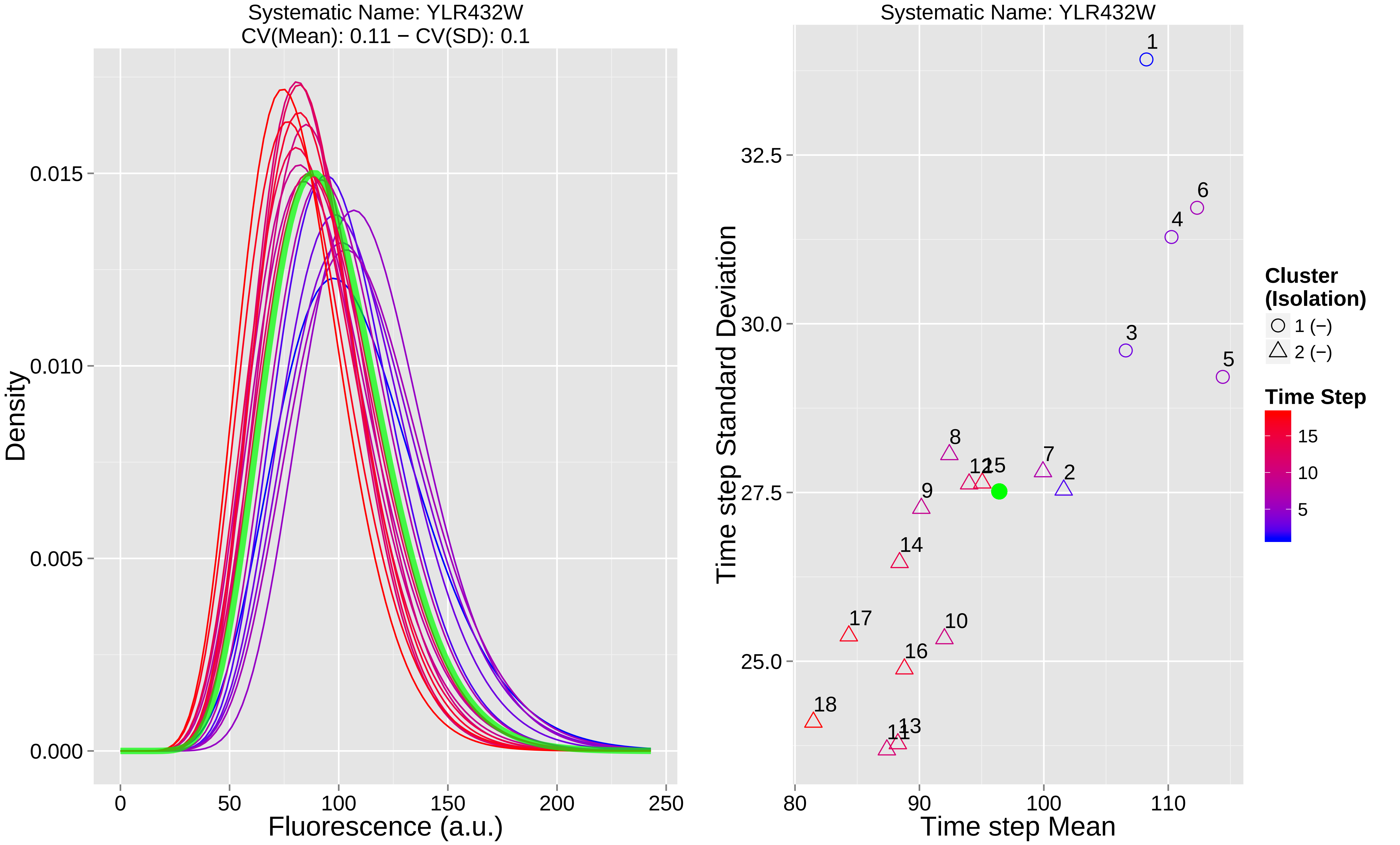
Systematic Name: YLR432W
CV(Mean): 0.11 − CV(SD): 0.1
Density
0.000
0.005
0.010
0.015
0
50
100
150
200
250
Fluorescence (a.u.)
Systematic Name: YLR432W
Time step Standard Deviation
25.0
27.5
30.0
32.5
80
90
100
110
Time step Mean
1
2
3
4
5
6
7
8
9
10
11
12
13
14
15
16
17
18
Cluster (Isolation)
1 (−)
2 (−)
Time Step
15
10
5

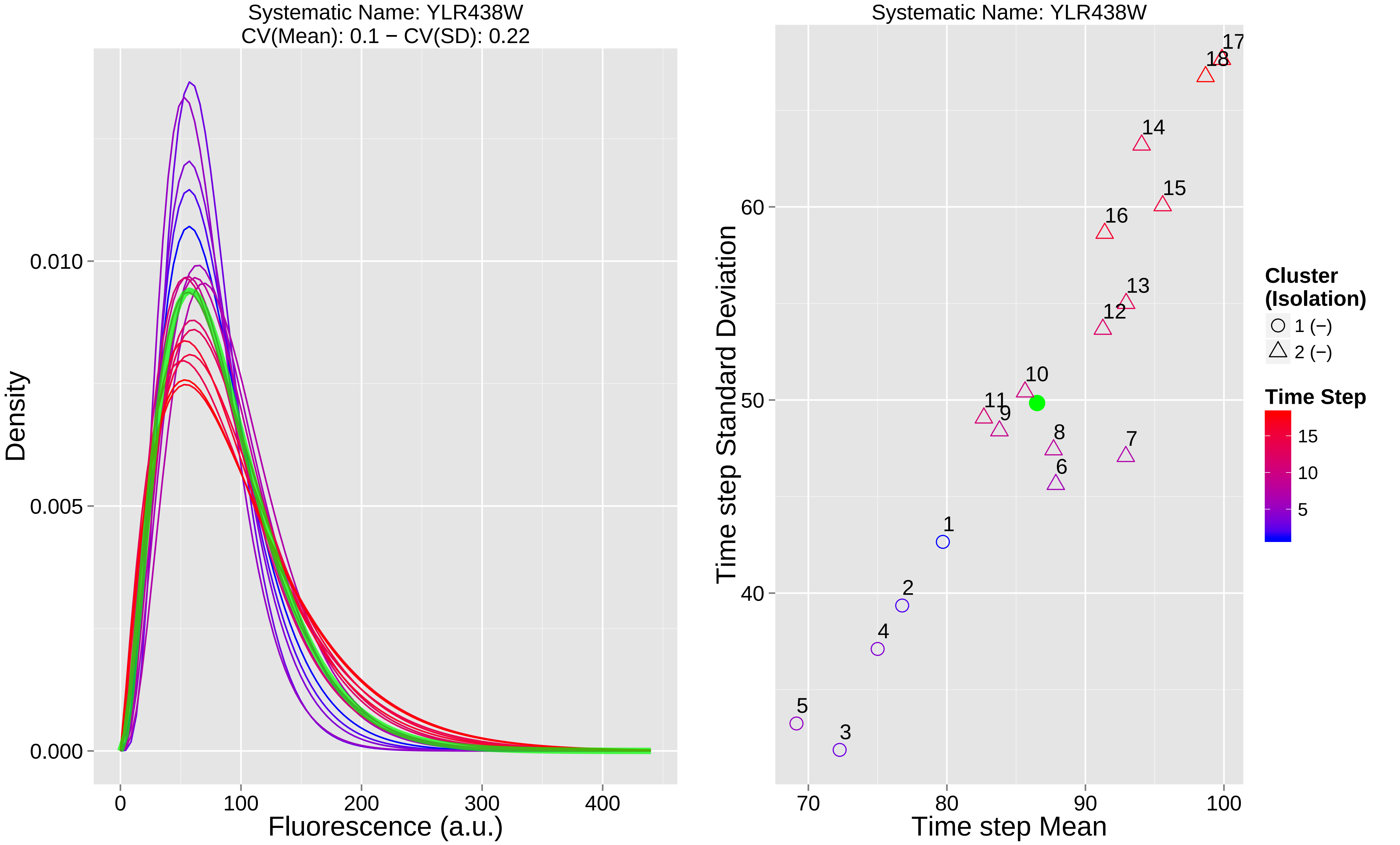

Systematic Name: YLR438W
CV(Mean): 0.1 − CV(SD): 0.22
Density
0.010
0.005
0.000
0
100
200
300
400
Fluorescence (a.u.)
Systematic Name: YLR438W
Time step Standard Deviation
60
50
40
70
80
90
100
Time step Mean
1
2
3
4
5
6
7
8
9
10
11
12
13
14
15
16
17
18
Cluster (Isolation)
1 (−)
2 (−)
Time Step
15
10
5

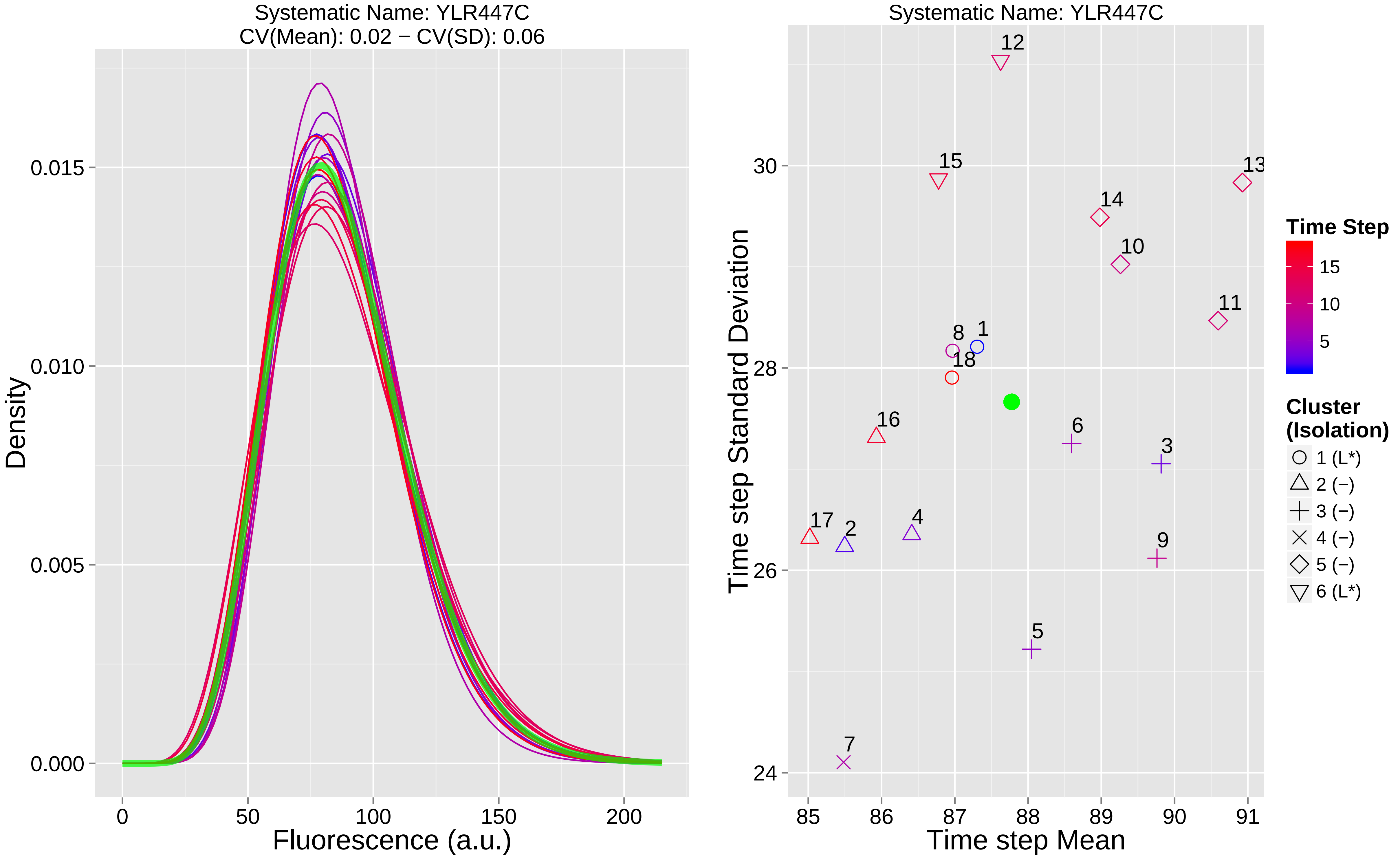

Systematic Name: YLR447C
CV(Mean): 0.02 − CV(SD): 0.06
Density
0.000
0.005
0.010
0.015
0
50
100
150
200
Fluorescence (a.u.)
Systematic Name: YLR447C
Time step Standard Deviation
24
26
28
30
85
86
87
88
89
90
91
Time step Mean
12
15
13
14
10
11
8
1
18
16
6
3
17
2
4
9
5
7
Time Step
15
10
5
Cluster
(Isolation)
1 (L*)
2 (−)
3 (−)
4 (−)
5 (−)
6 (L*)

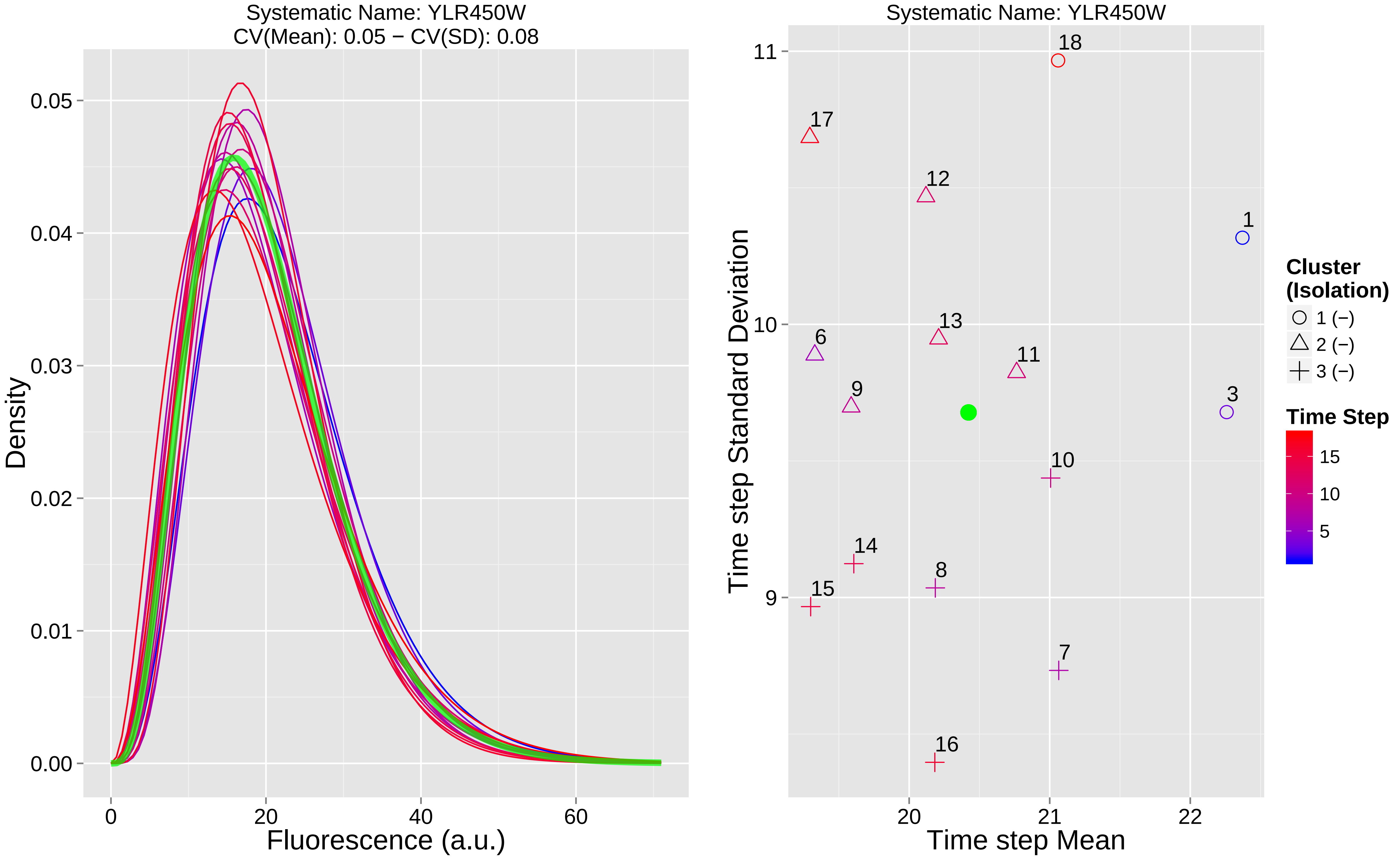

Systematic Name: YLR450W
CV(Mean): 0.05 − CV(SD): 0.08
Density
Fluorescence (a.u.)
0.00
0.01
0.02
0.03
0.04
0.05
0
20
40
60
Systematic Name: YLR450W
Time step Standard Deviation
Time step Mean
9
10
11
20
21
22
18
17
12
1
13
6
11
9
3
10
14
8
15
7
16
Cluster (Isolation)
1 (−)
2 (−)
3 (−)
Time Step
15
10
5

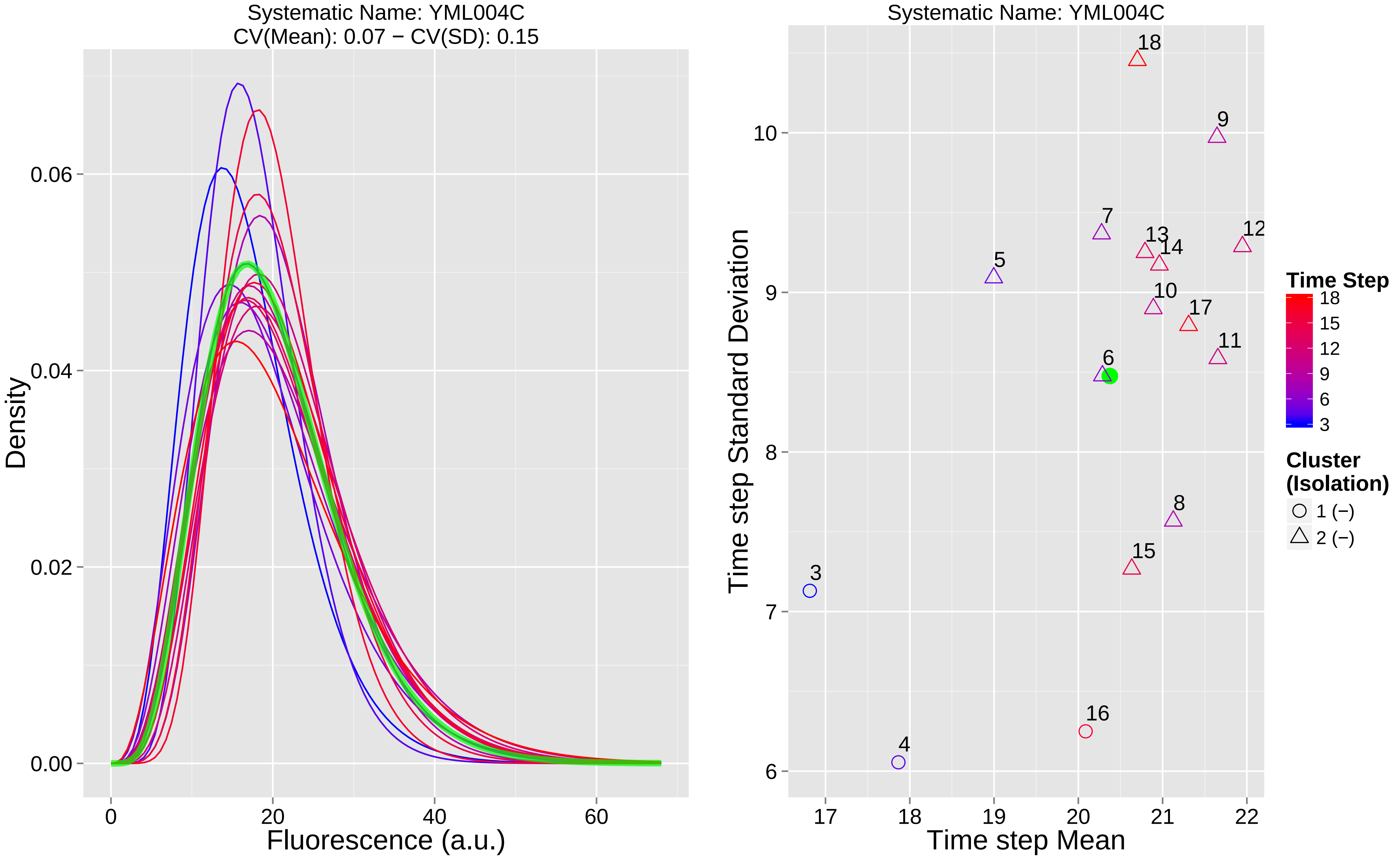
Systematic Name: YML004C
CV(Mean): 0.07 − CV(SD): 0.15
Density
Fluorescence (a.u.)
0.00
0.02
0.04
0.06
0
20
40
60
Systematic Name: YML004C
Time step Standard Deviation
Time step Mean
6
7
8
9
10
17
18
19
20
21
22
3
4
5
6
7
8
9
10
11
12
13
14
15
16
17
18
Time Step
18
15
12
9
6
3
Cluster
(Isolation)
1 (−)
2 (−)

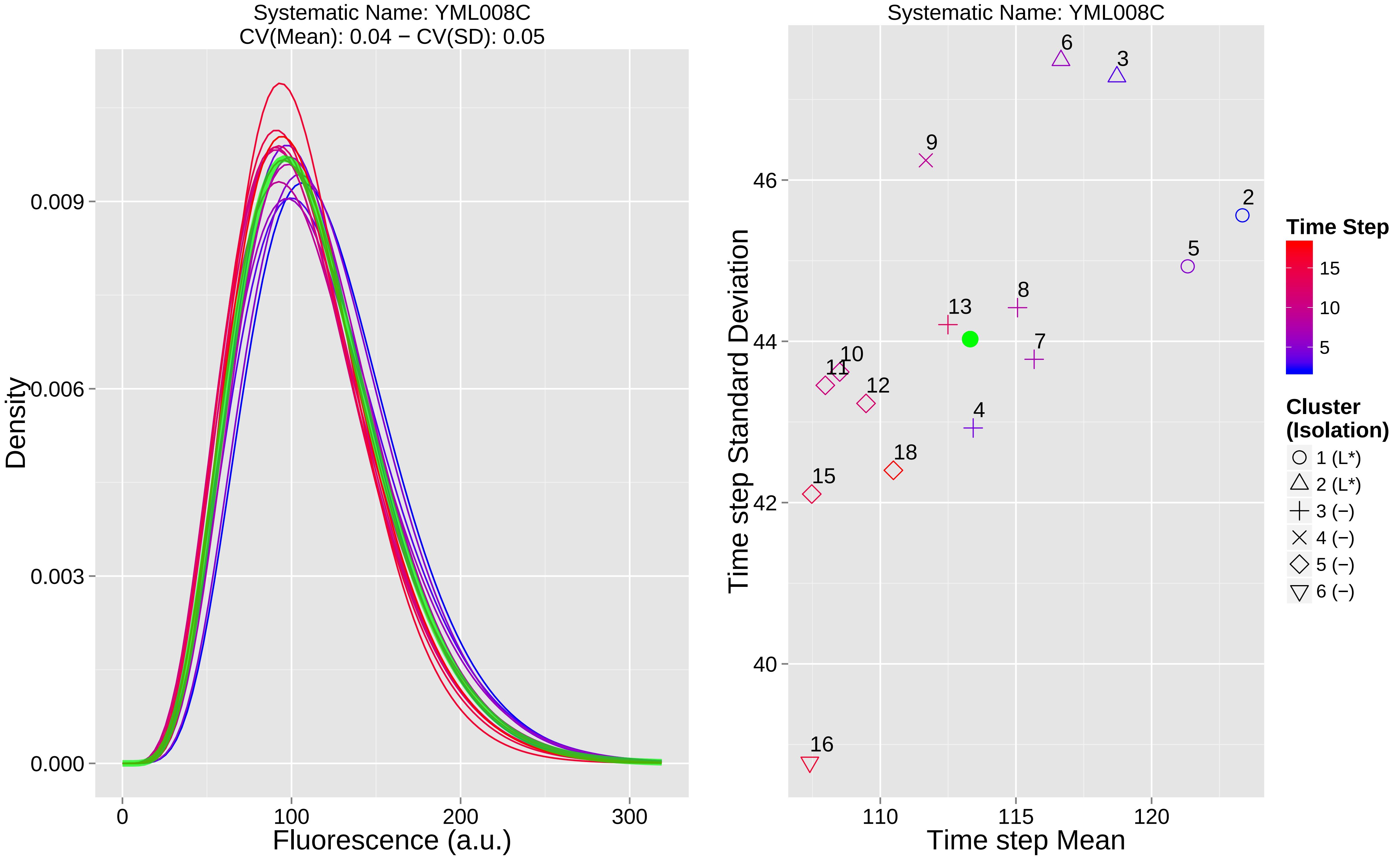

Systematic Name: YML008C
CV(Mean): 0.04 − CV(SD): 0.05
Density
0.009
0.006
0.003
0.000
0
100
200
300
Fluorescence (a.u.)
Systematic Name: YML008C
Time step Standard Deviation
46
44
42
40
110
115
120
Time step Mean
6
3
9
2
5
8
13
7
10
11
12
4
18
15
16
Time Step
15
10
5
Cluster
(Isolation)
1 (L*)
2 (L*)
3 (−)
4 (−)
5 (−)
6 (−)

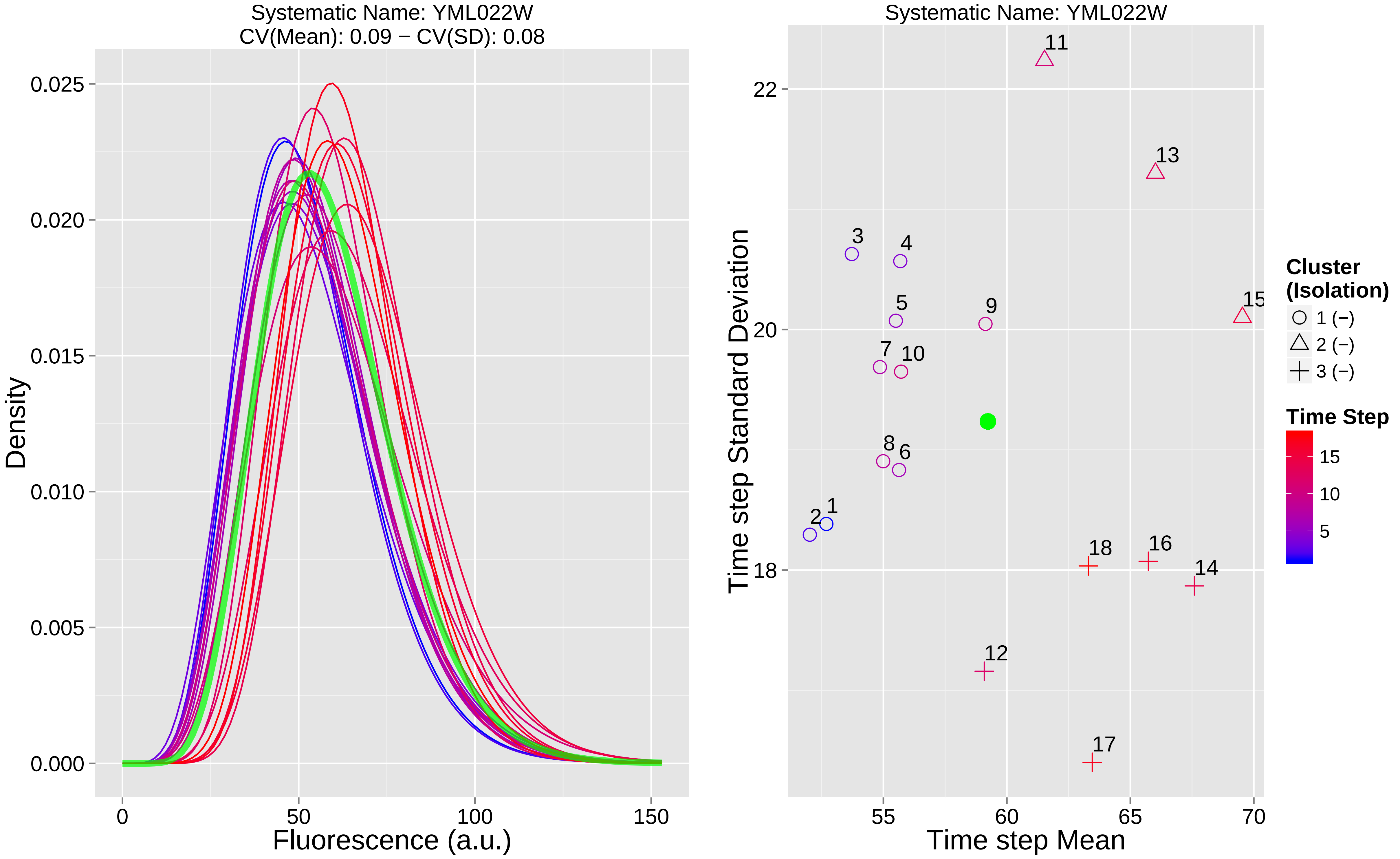

Systematic Name: YML022W
CV(Mean): 0.09 − CV(SD): 0.08
Density
0.025
0.020
0.015
0.010
0.005
0.000
0
50
100
150
Fluorescence (a.u.)
Systematic Name: YML022W
Time step Standard Deviation
22
20
18
55
60
65
70
Time step Mean
1
2
3
4
5
6
7
8
9
10
11
12
13
14
15
16
17
18
Cluster (Isolation)
1 (−)
2 (−)
3 (−)
Time Step
15
10
5

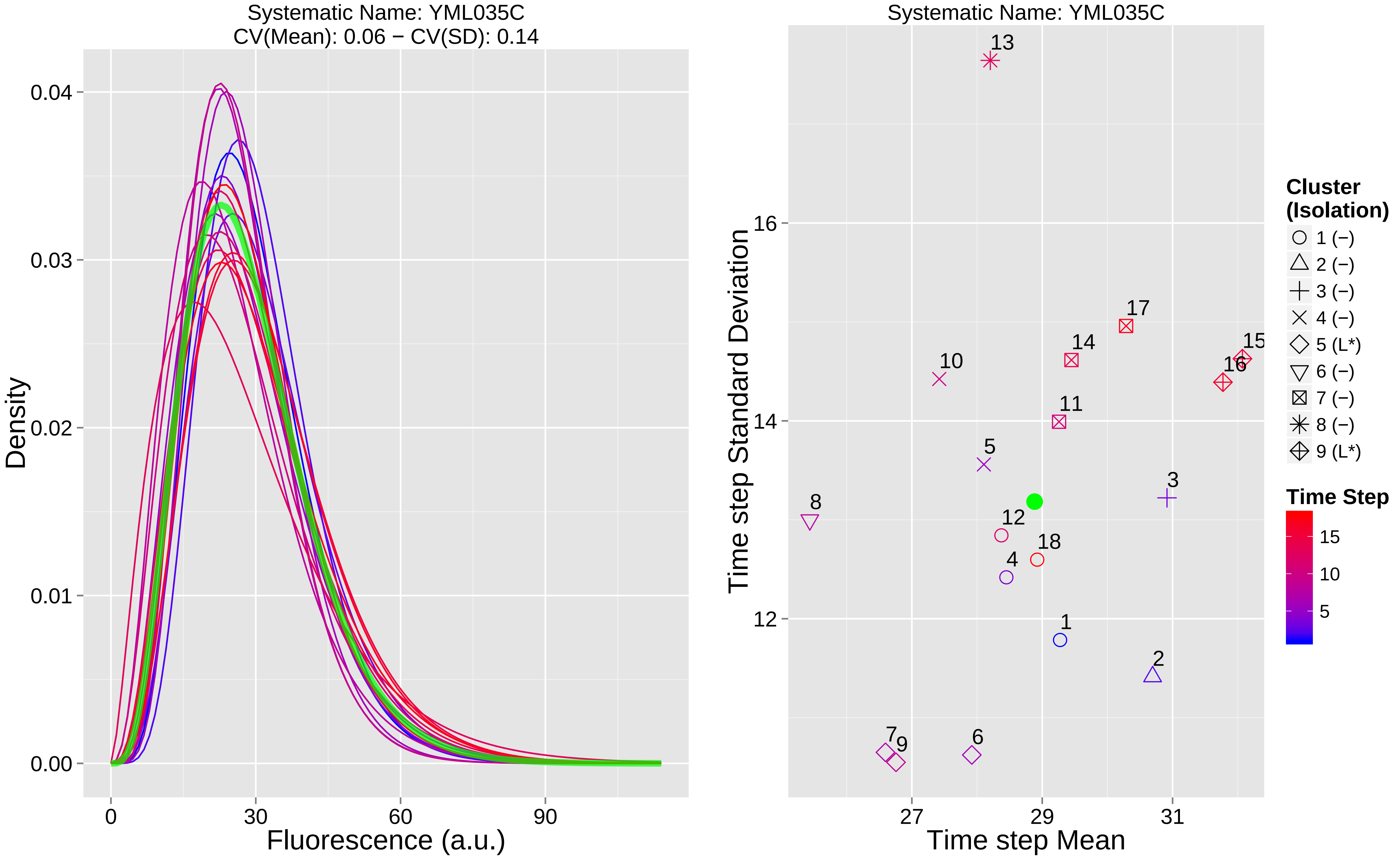

Systematic Name: YML035C
CV(Mean): 0.06 − CV(SD): 0.14
Density
0.00
0.01
0.02
0.03
0.04
0
30
60
90
Fluorescence (a.u.)
Systematic Name: YML035C
Time step Standard Deviation
12
14
16
27
29
31
Time step Mean
13
17
14
15
16
10
11
5
3
8
12
18
4
1
2
7
9
6
Cluster (Isolation)
1 (−)
2 (−)
3 (−)
4 (−)
5 (L*)
6 (−)
7 (−)
8 (−)
9 (L*)
Time Step
15
10
5

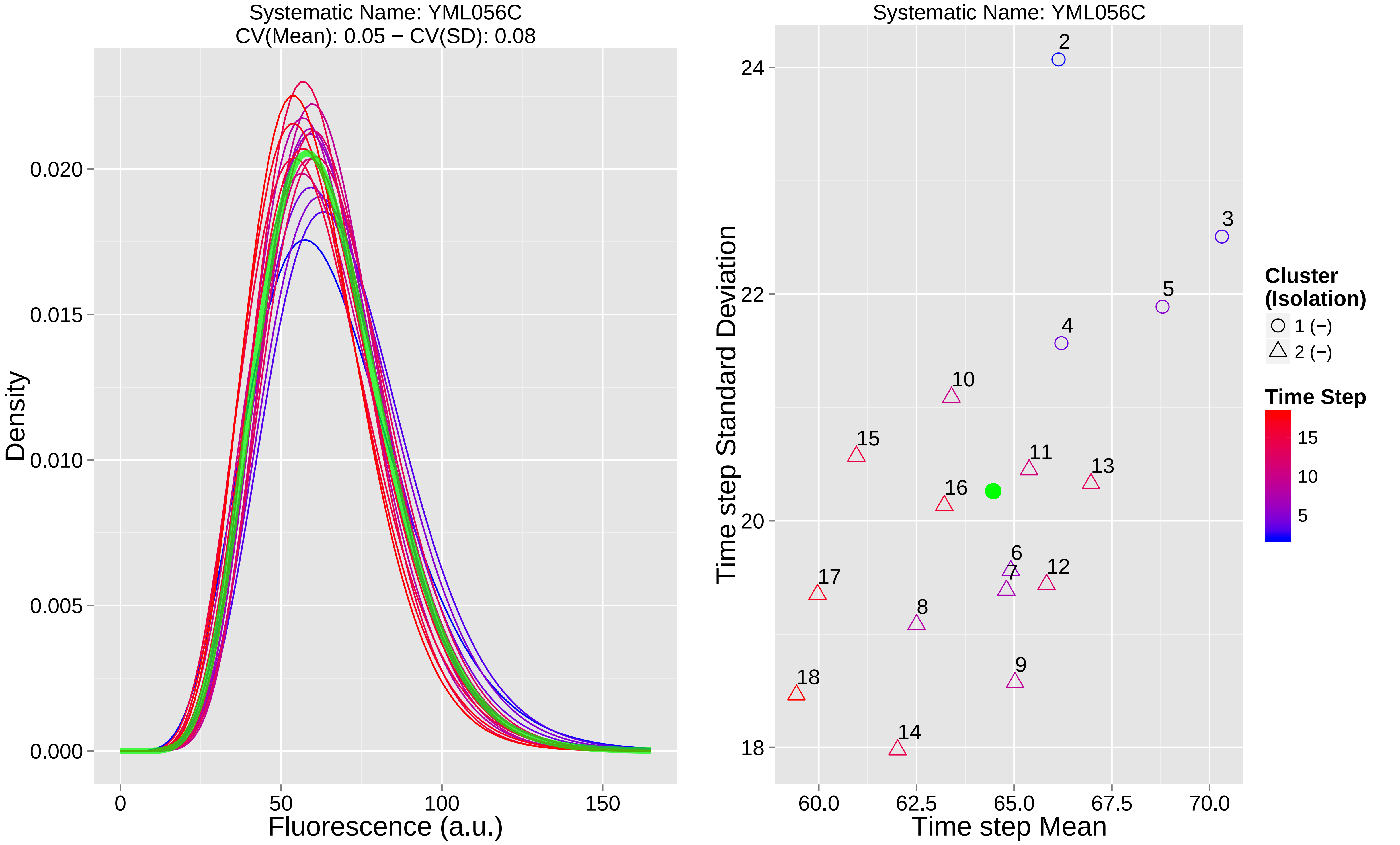
Systematic Name: YML056C
CV(Mean): 0.05 − CV(SD): 0.08
Density
0.000
0.005
0.010
0.015
0.020
0
50
100
150
Fluorescence (a.u.)
Systematic Name: YML056C
Time step Standard Deviation
18
20
22
24
60.0
62.5
65.0
67.5
70.0
Time step Mean
2
3
4
5
6
7
8
9
10
11
12
13
14
15
16
17
18
Cluster
(Isolation)
1 (−)
2 (−)
Time Step
15
10
5

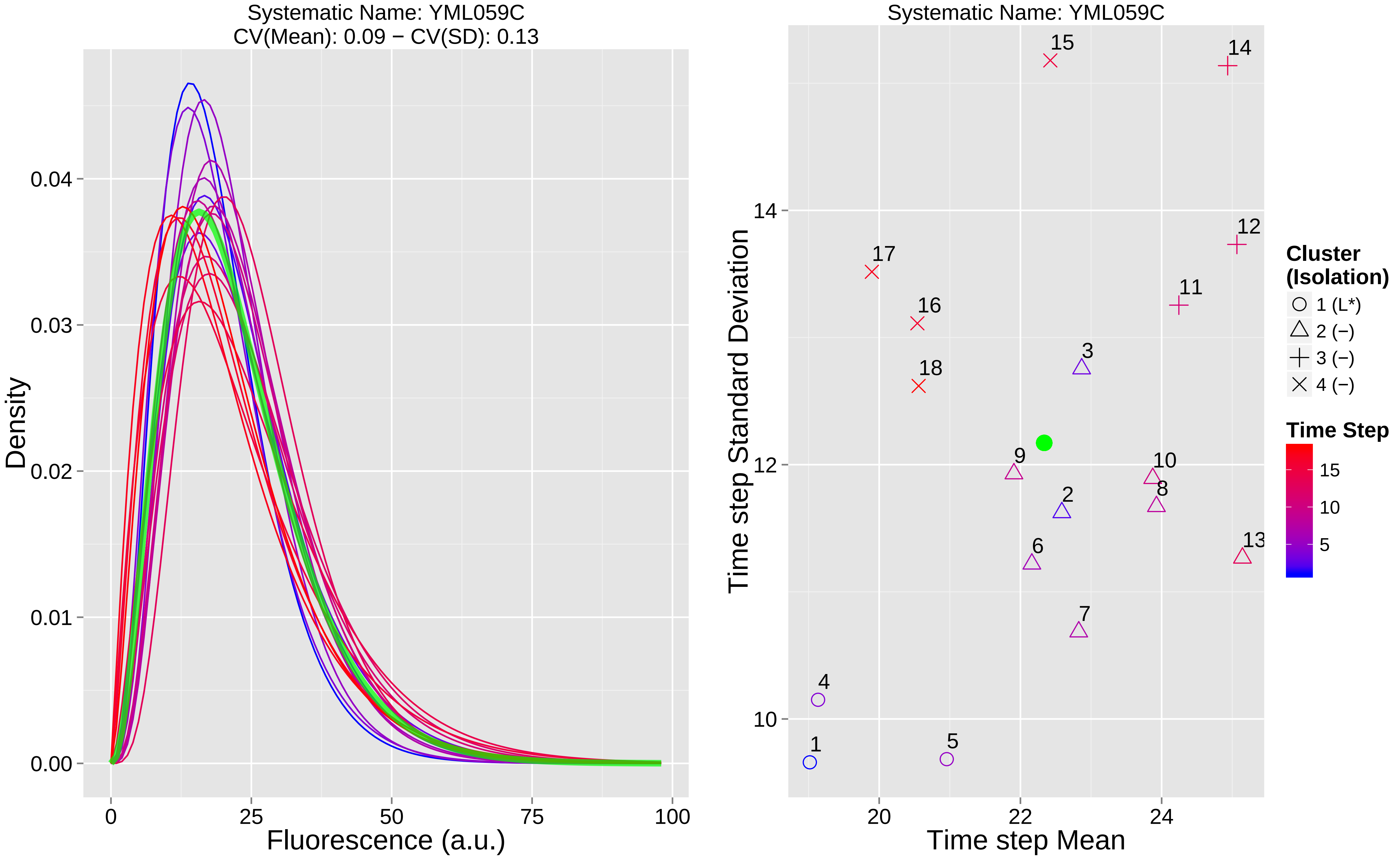

Systematic Name: YML059C
CV(Mean): 0.09 − CV(SD): 0.13
Density
0.00
0.01
0.02
0.03
0.04
0
25
50
75
100
Fluorescence (a.u.)
Systematic Name: YML059C
Time step Standard Deviation
10
12
14
20
22
24
Time step Mean
1
2
3
4
5
6
7
8
9
10
11
12
13
14
15
16
17
18
Cluster (Isolation)
1 (L*)
2 (−)
3 (−)
4 (−)
Time Step
5
10
15

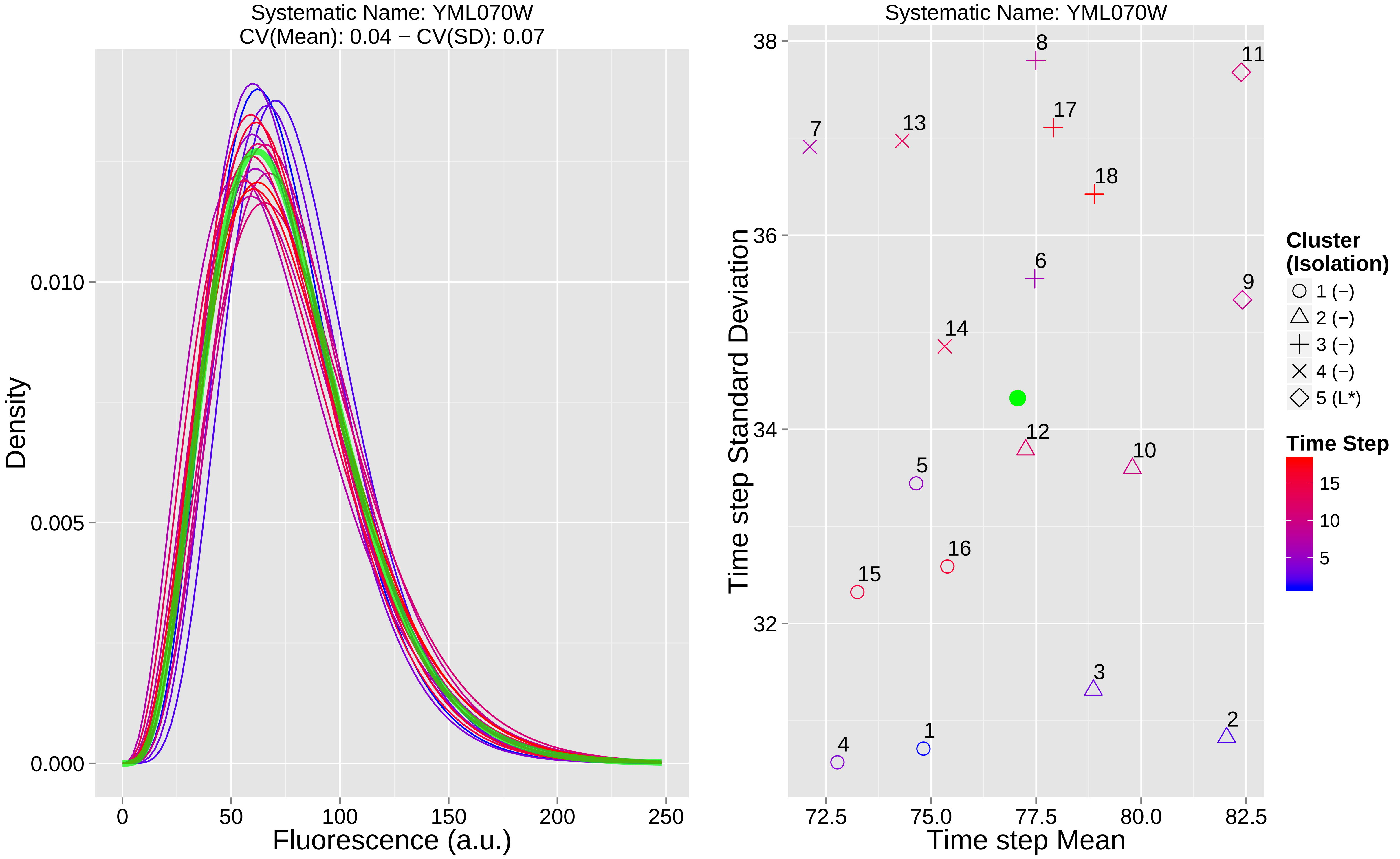
Systematic Name: YML070W
CV(Mean): 0.04 − CV(SD): 0.07
Density
Fluorescence (a.u.)
Systematic Name: YML070W
Time step Standard Deviation
Time step Mean
Cluster (Isolation)
1 (−)
2 (−)
3 (−)
4 (−)
5 (L*)
Time Step

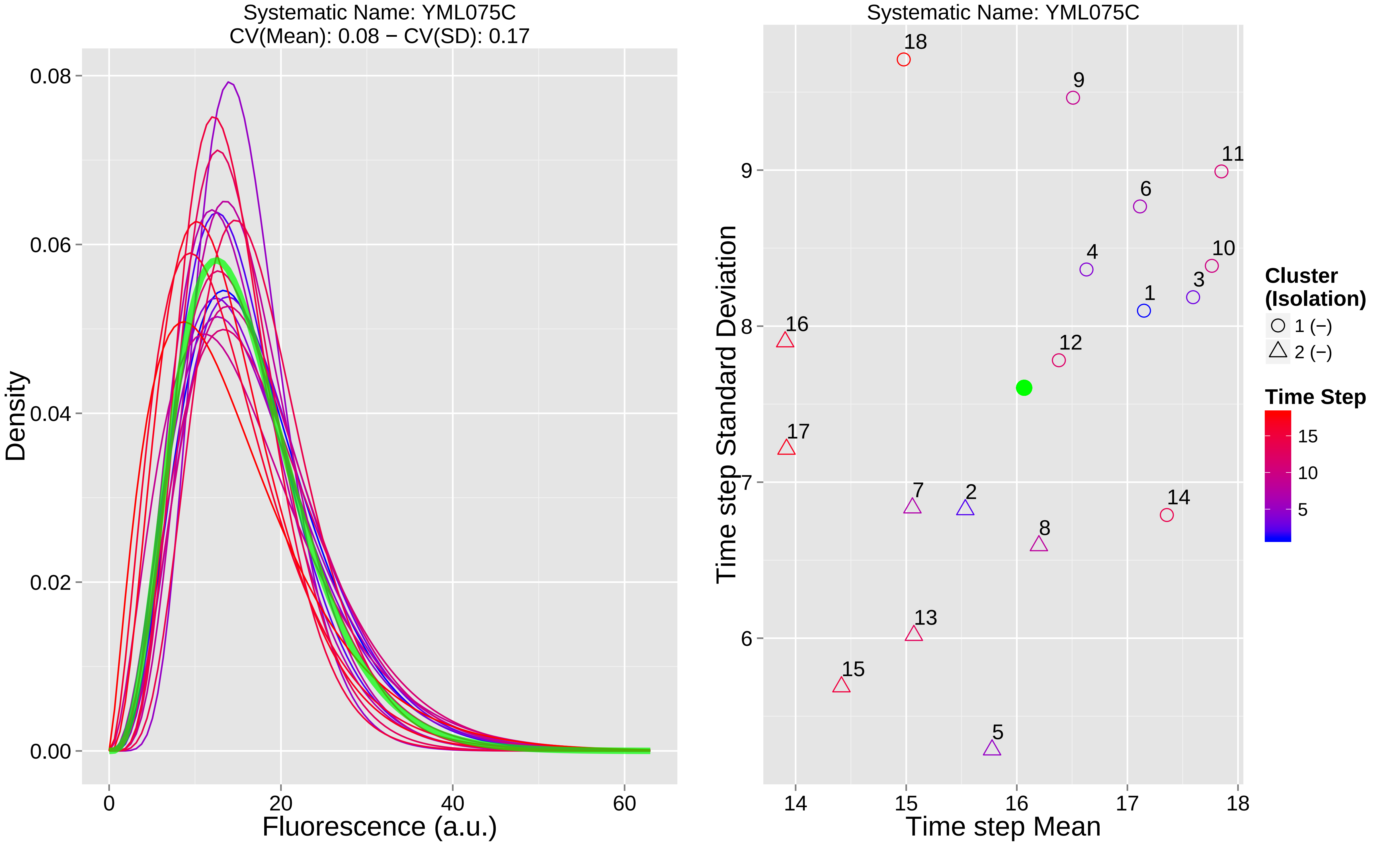

Systematic Name: YML075C
CV(Mean): 0.08 − CV(SD): 0.17
Density
0.00
0.02
0.04
0.06
0.08
0
20
40
60
Fluorescence (a.u.)
Systematic Name: YML075C
Time step Standard Deviation
Time step Mean
6
7
8
9
14
15
16
17
18
18
9
11
6
4
10
3
1
16
12
17
7
2
14
8
13
15
5
Cluster (Isolation)
1 (−)
2 (−)
Time Step
15
10
5

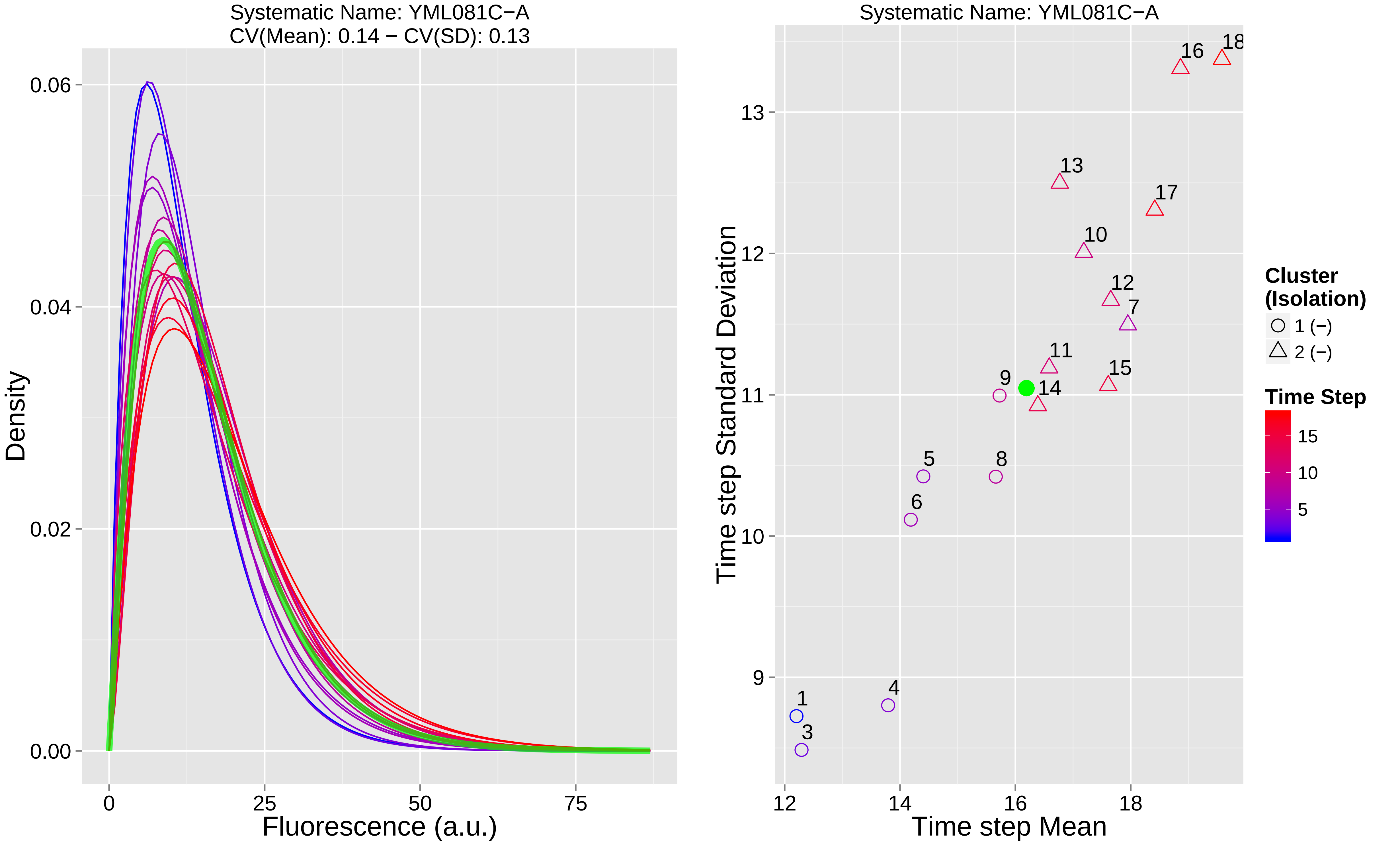

Systematic Name: YML081C−A
CV(Mean): 0.14 − CV(SD): 0.13
Density
0.06
0.04
0.02
0.00
0
25
50
75
Fluorescence (a.u.)
Systematic Name: YML081C−A
Time step Standard Deviation
13
12
11
10
9
12
14
16
18
Time step Mean
1
3
4
5
6
8
9
14
11
15
12
7
10
17
13
16
18
Cluster (Isolation)
1 (−)
2 (−)
Time Step
15
10
5

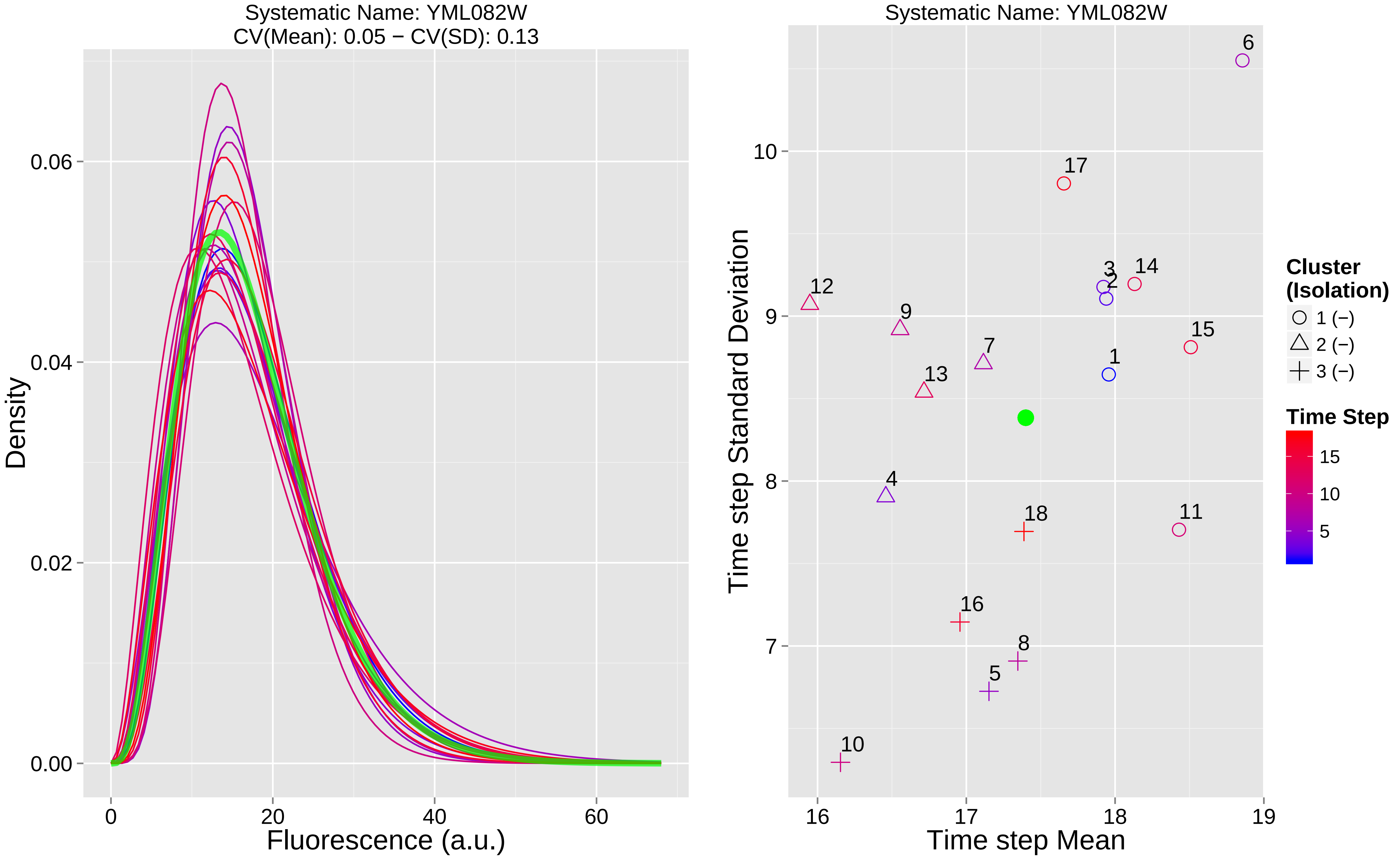

Systematic Name: YML082W
CV(Mean): 0.05 − CV(SD): 0.13
Density
Fluorescence (a.u.)
0.00
0.02
0.04
0.06
0
20
40
60
Systematic Name: YML082W
Time step Standard Deviation
Time step Mean
7
8
9
10
16
17
18
19
1
2
3
4
5
6
7
8
9
10
11
12
13
14
15
16
17
18
Cluster (Isolation)
1 (−)
2 (−)
3 (−)
Time Step
15
10
5

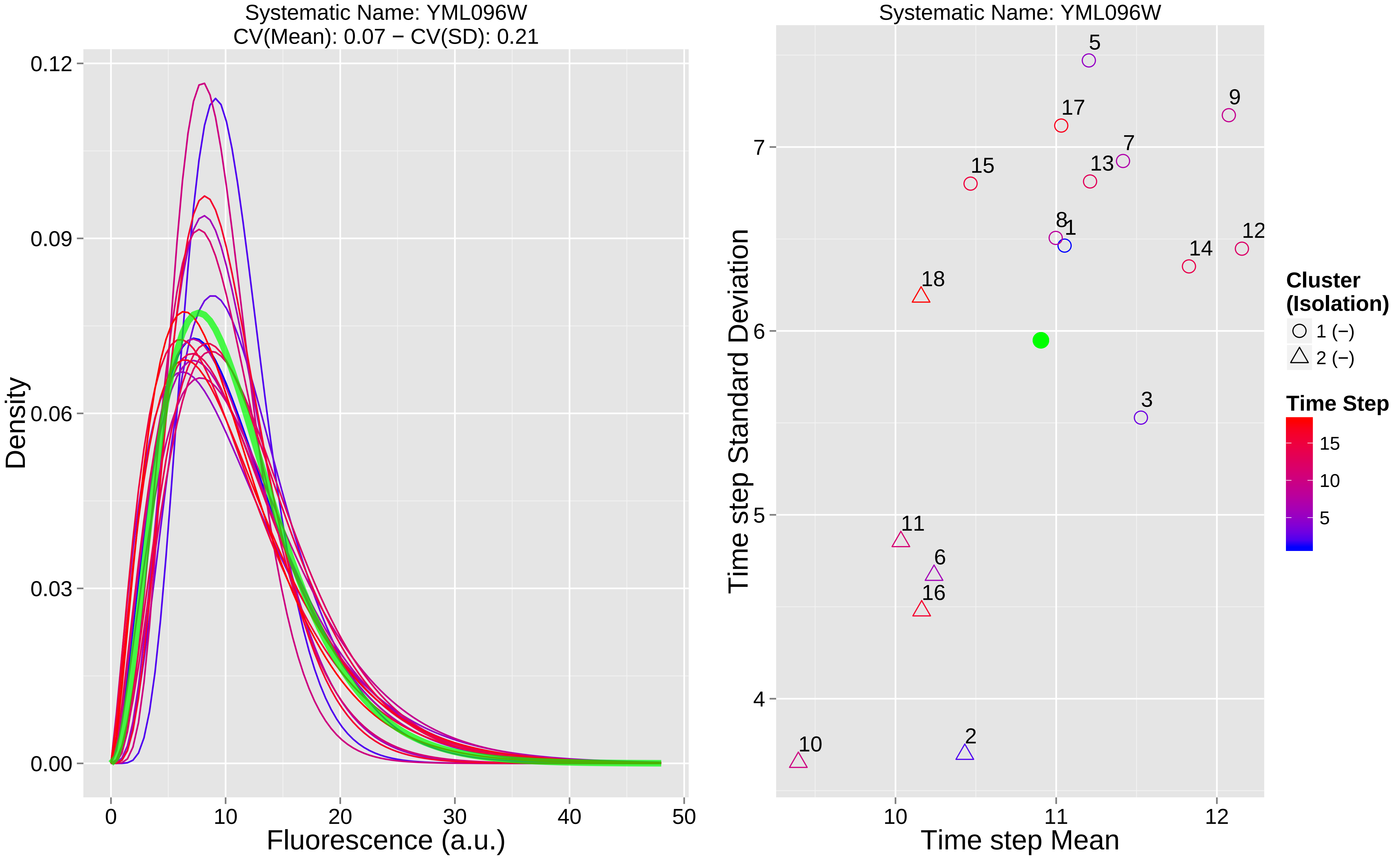
Systematic Name: YML096W
CV(Mean): 0.07 − CV(SD): 0.21
Density
Fluorescence (a.u.)
0.12
0.09
0.06
0.03
0.00
0
10
20
30
40
50
Systematic Name: YML096W
Time step Standard Deviation
Time step Mean
7
6
5
4
10
11
12
5
17
9
7
15
13
8
1
12
14
18
3
11
6
16
10
2
Cluster (Isolation)
1 (−)
2 (−)
Time Step
15
10
5

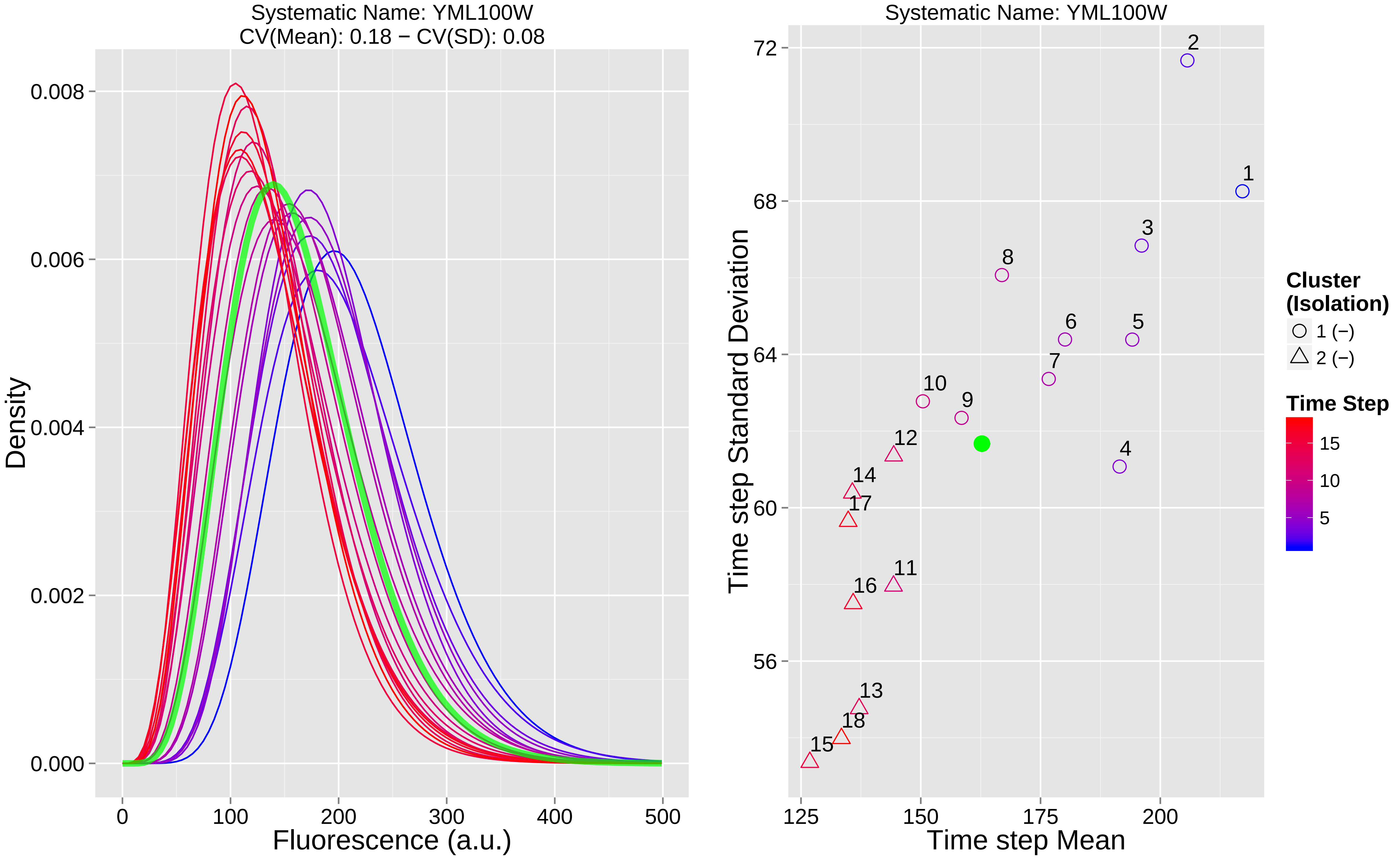

Systematic Name: YML100W
CV(Mean): 0.18 − CV(SD): 0.08
Density
0.000
0.002
0.004
0.006
0.008
0
100
200
300
400
500
Fluorescence (a.u.)
Systematic Name: YML100W
Time step Standard Deviation
56
60
64
68
72
125
150
175
200
Time step Mean
1
2
3
4
5
6
7
8
9
10
11
12
13
14
15
16
17
18
Cluster (Isolation)
1 (−)
2 (−)
Time Step
15
10
5

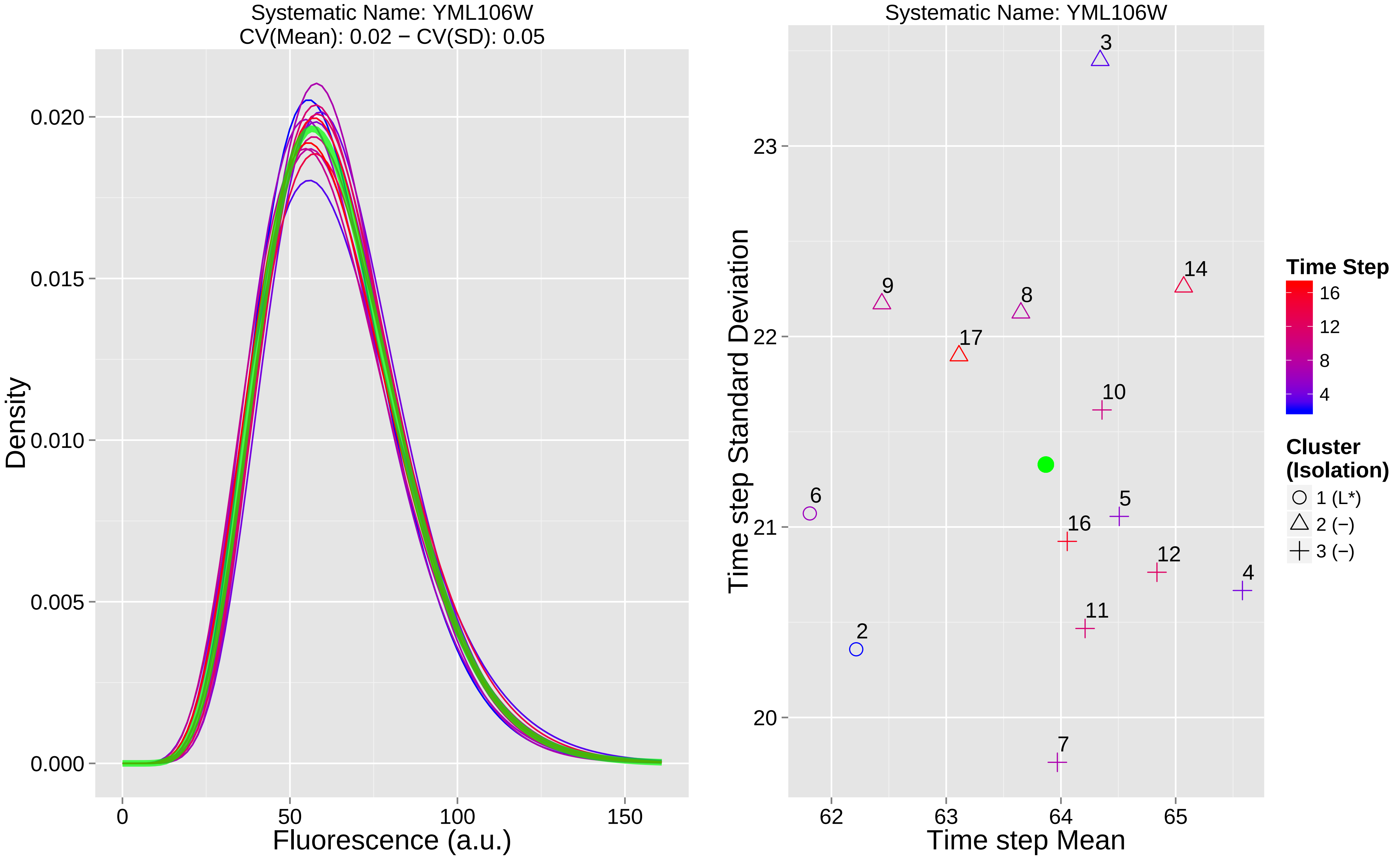
Systematic Name: YML106W
CV(Mean): 0.02 − CV(SD): 0.05
Density
0.000
0.005
0.010
0.015
0.020
0
50
100
150
Fluorescence (a.u.)
Systematic Name: YML106W
Time step Standard Deviation
20
21
22
23
62
63
64
65
Time step Mean
3
9
8
14
17
10
6
5
16
12
4
11
2
7
Time Step
16
12
8
4
Cluster
(Isolation)
1 (L*)
2 (−)
3 (−)

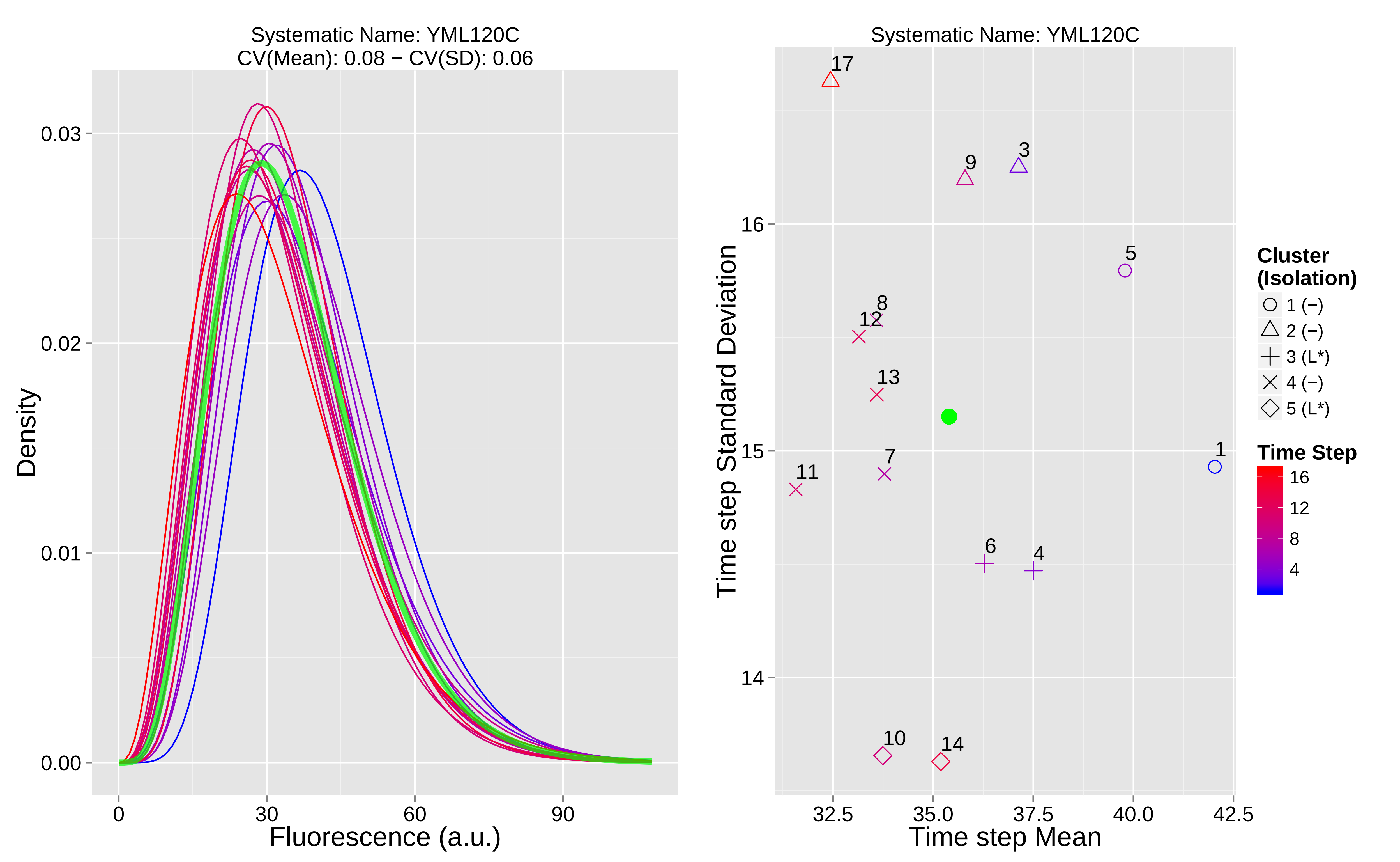
Systematic Name: YML120C
CV(Mean): 0.08 − CV(SD): 0.06
Density
Fluorescence (a.u.)
0.00
0.01
0.02
0.03
0
30
60
90
Systematic Name: YML120C
Time step Standard Deviation
Time step Mean
14
15
16
32.5
35.0
37.5
40.0
42.5
17
9
3
5
8
12
13
1
7
11
6
4
10
14
Cluster (Isolation)
1 (−)
2 (−)
3 (L*)
4 (−)
5 (L*)
Time Step
16
12
8
4

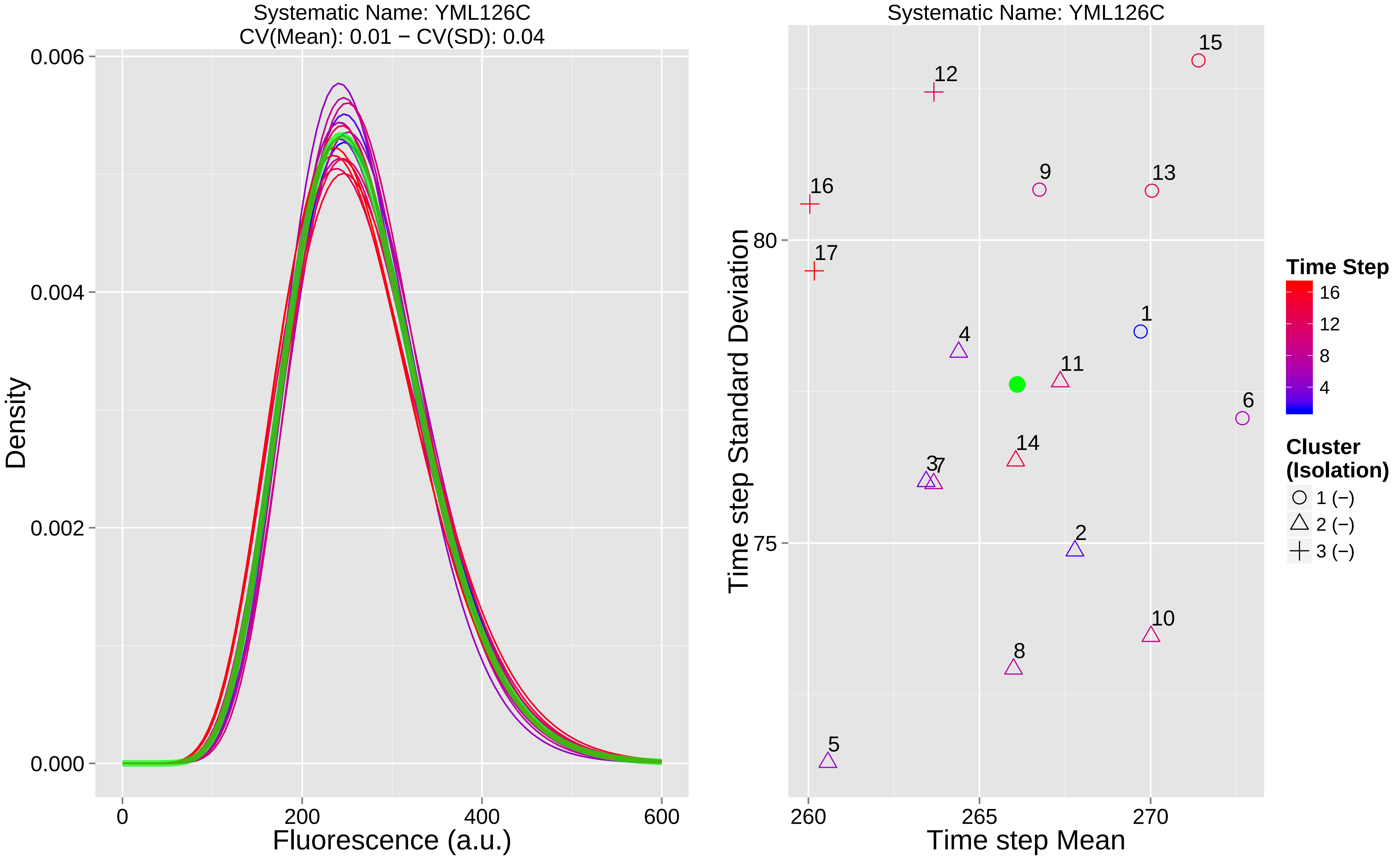

Systematic Name: YML126C
CV(Mean): 0.01 − CV(SD): 0.04
Density
0.006
0.004
0.002
0.000
0
200
400
600
Fluorescence (a.u.)
Systematic Name: YML126C
Time step Standard Deviation
80
75
260
265
270
Time step Mean
15
12
9
13
16
17
1
4
11
6
14
3
7
2
10
8
5
Time Step
16
12
8
4
Cluster (Isolation)
1 (−)
2 (−)
3 (−)

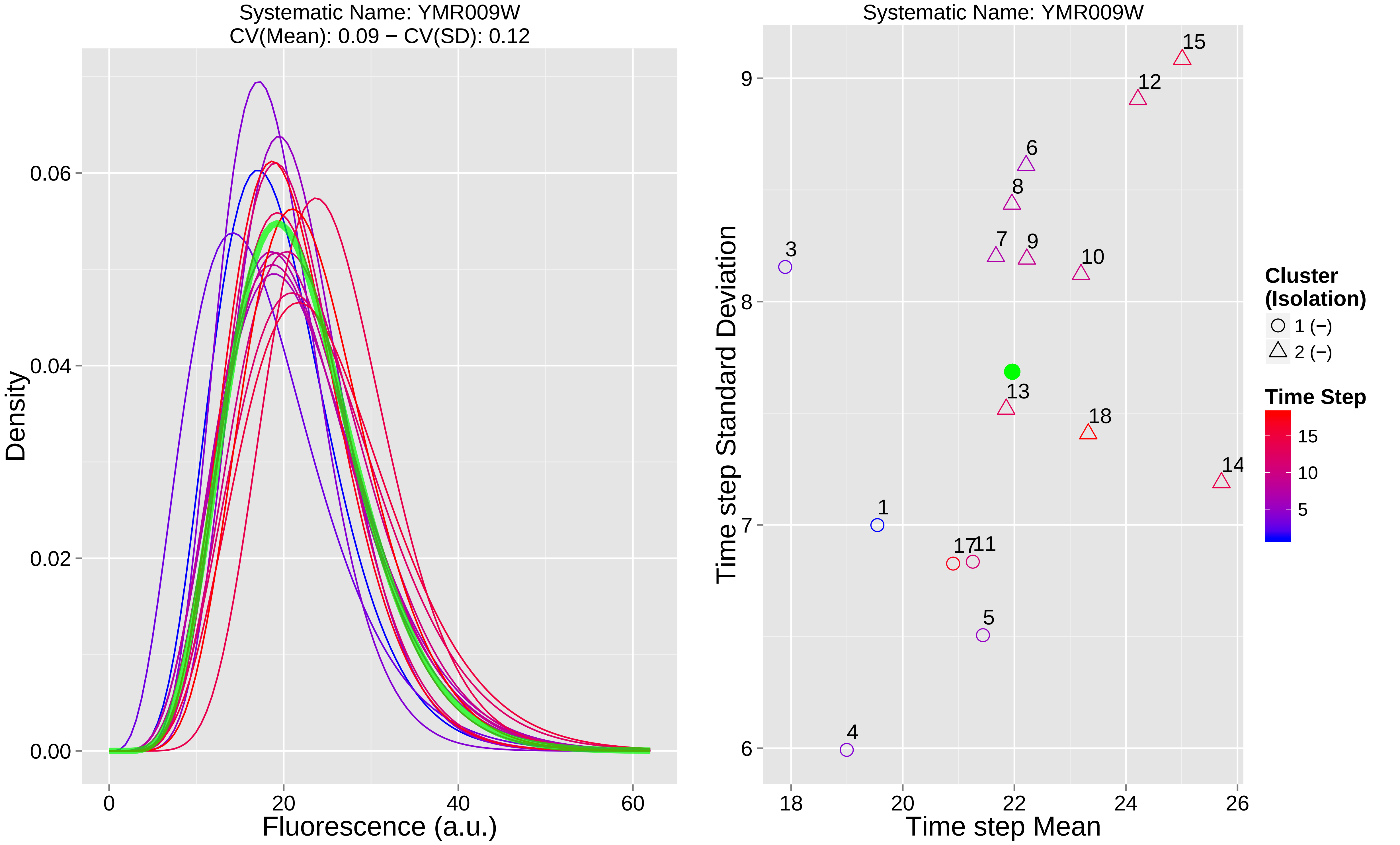
Systematic Name: YMR009W
CV(Mean): 0.09 − CV(SD): 0.12
Density
0.00
0.02
0.04
0.06
0
20
40
60
Fluorescence (a.u.)
Systematic Name: YMR009W
Time step Standard Deviation
6
7
8
9
18
20
22
24
26
Time step Mean
15
12
6
8
3
7
9
10
13
18
14
1
17
11
5
4
Cluster
(Isolation)
1 (−)
2 (−)
Time Step
15
10
5

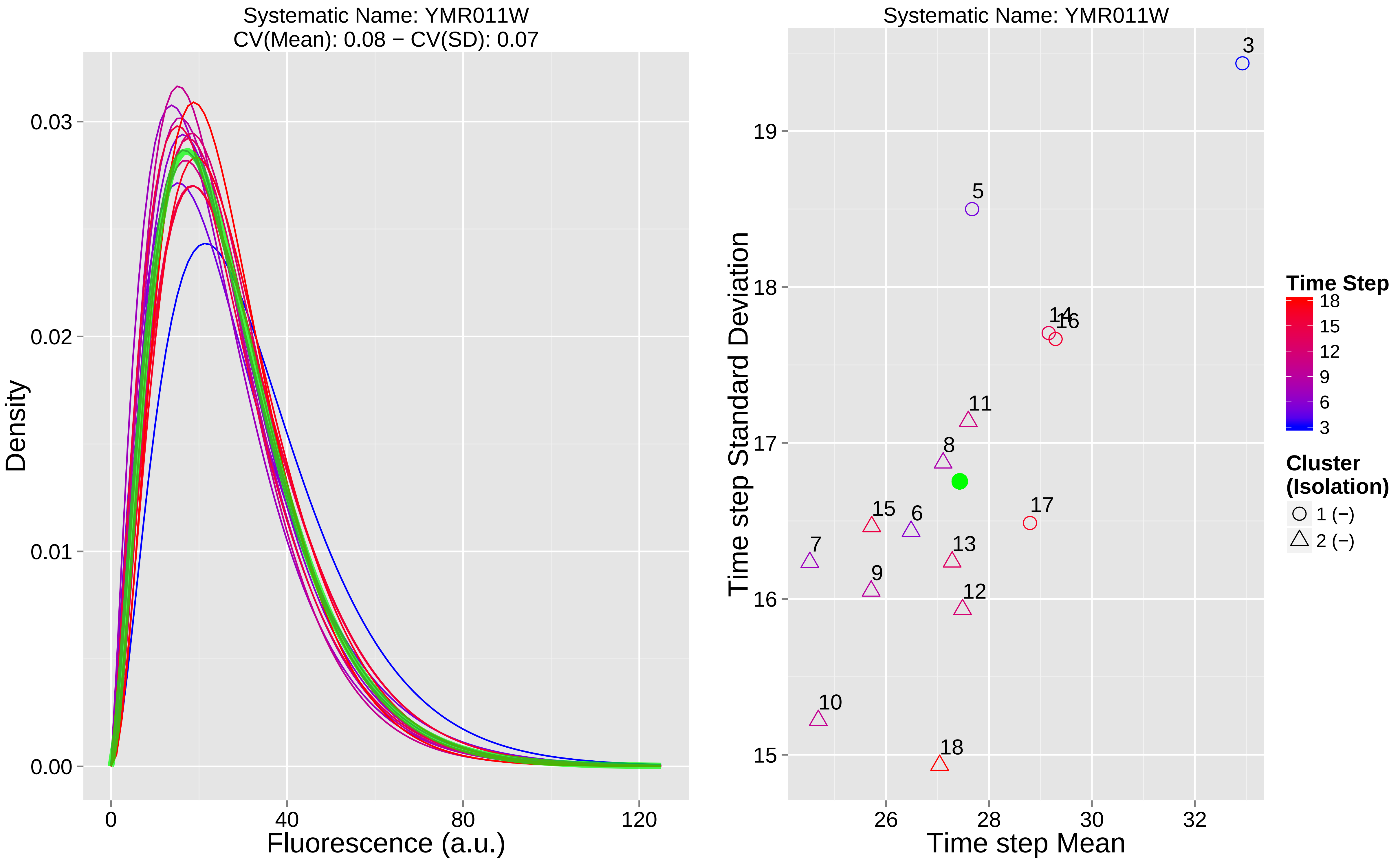
Systematic Name: YMR011W
CV(Mean): 0.08 − CV(SD): 0.07
Density
0.03
0.02
0.01
0.00
0
40
80
120
Fluorescence (a.u.)
Systematic Name: YMR011W
Time step Standard Deviation
19
18
17
16
15
26
28
30
32
Time step Mean
3
5
14
16
11
8
15
6
17
7
13
9
12
10
18
Time Step
18
15
12
9
6
3
Cluster
(Isolation)
1 (−)
2 (−)

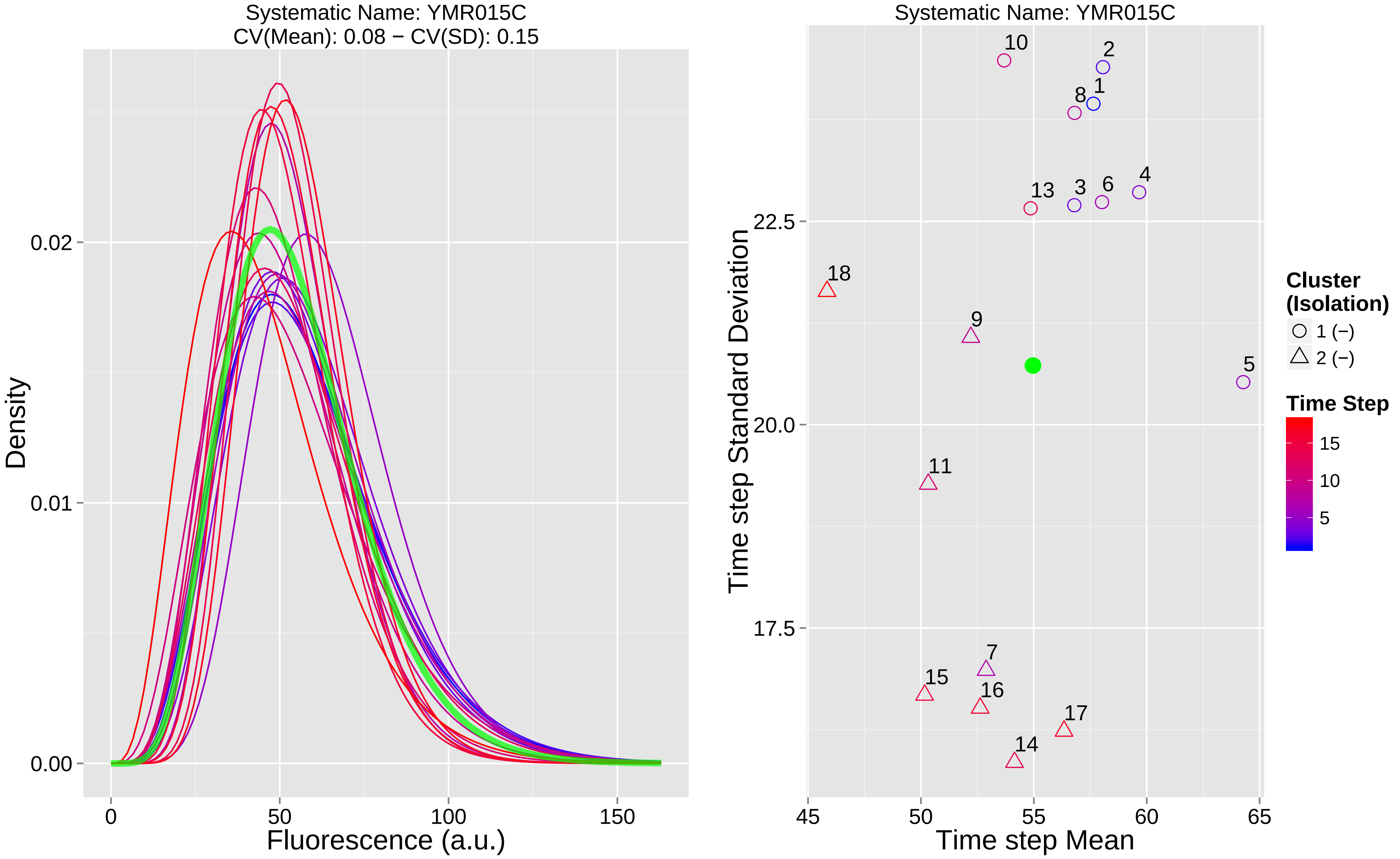

Systematic Name: YMR015C
CV(Mean): 0.08 − CV(SD): 0.15
Density
0.00
0.01
0.02
0
50
100
150
Fluorescence (a.u.)
Systematic Name: YMR015C
Time step Standard Deviation
17.5
20.0
22.5
45
50
55
60
65
Time step Mean
10
2
8
1
13
3
6
4
18
9
5
11
7
15
16
17
14
Cluster (Isolation)
1 (−)
2 (−)
Time Step
15
10
5

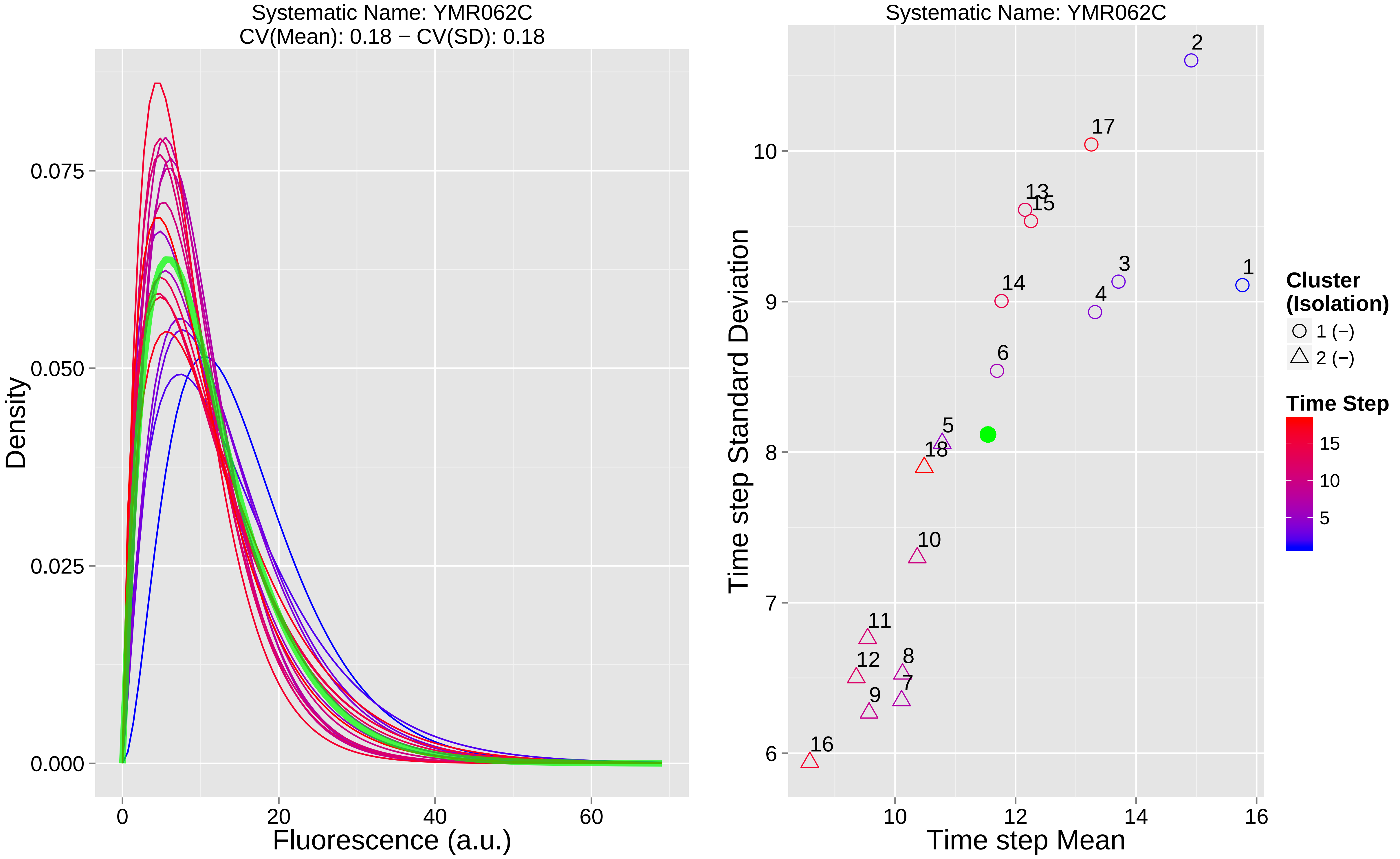

Systematic Name: YMR062C
CV(Mean): 0.18 − CV(SD): 0.18
Density
0.075
0.050
0.025
0.000
0
20
40
60
Fluorescence (a.u.)
Systematic Name: YMR062C
Time step Standard Deviation
10
9
8
7
6
Time step Mean
10
12
14
16
2
17
13
15
3
1
14
4
6
5
18
10
11
12
8
7
9
16
Cluster
(Isolation)
1 (−)
2 (−)
Time Step
15
10
5

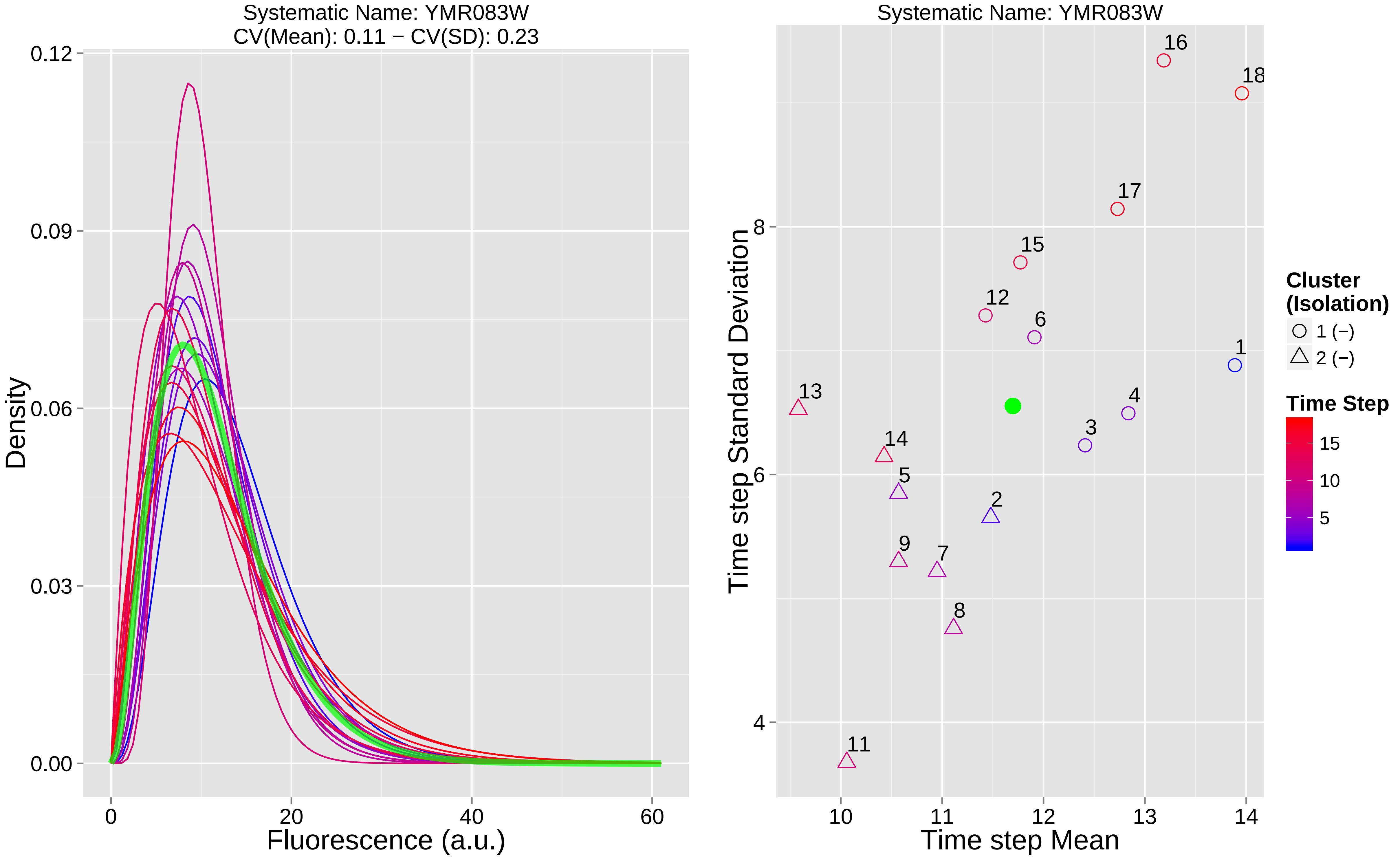

Systematic Name: YMR083W
CV(Mean): 0.11 − CV(SD): 0.23
Density
0.12
0.09
0.06
0.03
0.00
0
20
40
60
Fluorescence (a.u.)
Systematic Name: YMR083W
Time step Standard Deviation
8
6
4
10
11
12
13
14
Time step Mean
16
18
17
15
12
6
1
13
4
3
14
5
2
9
7
8
11
Cluster
(Isolation)
1 (−)
2 (−)
Time Step
15
10
5

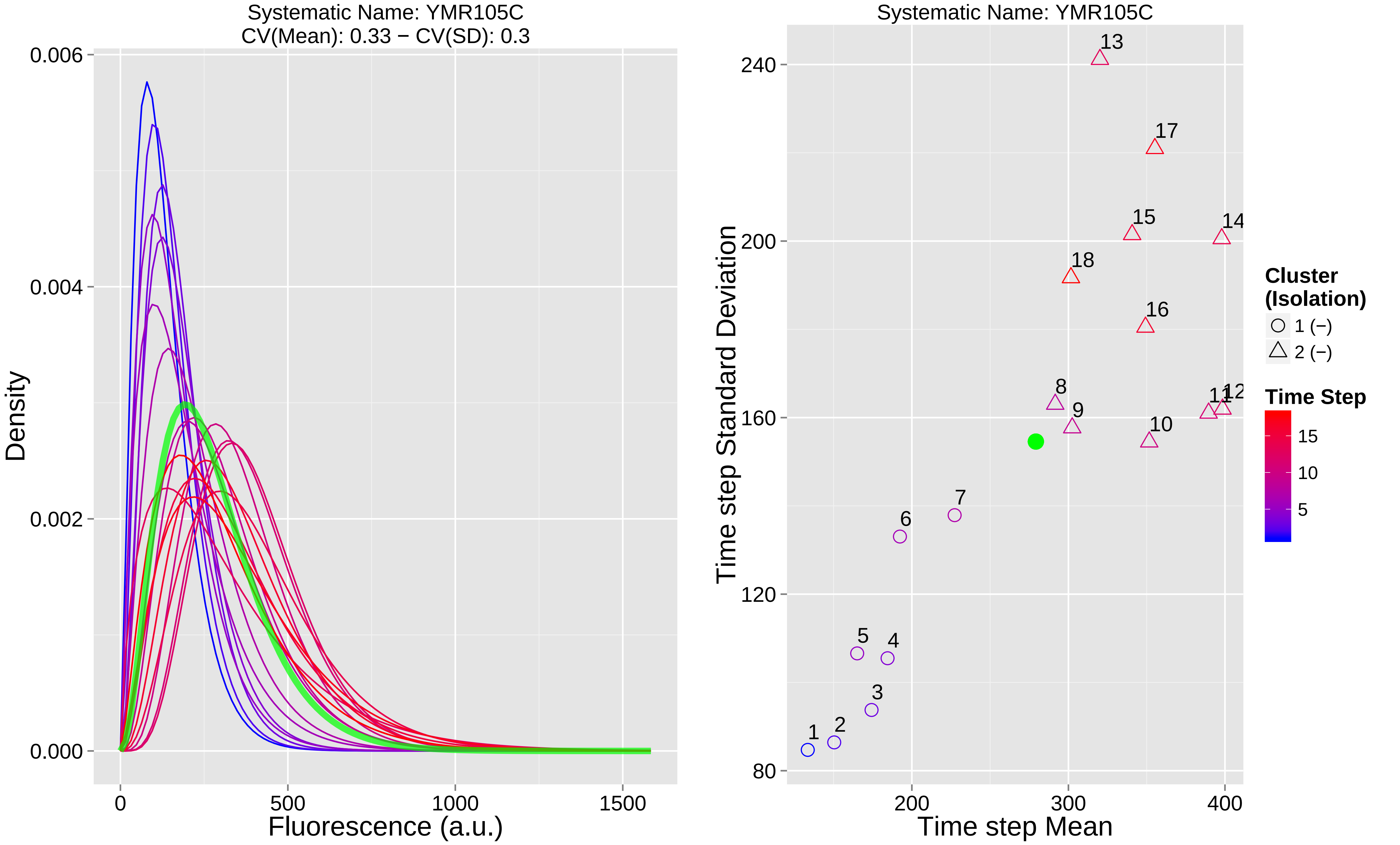

Systematic Name: YMR105C
CV(Mean): 0.33 − CV(SD): 0.3
Density
0.006
0.004
0.002
0.000
0
500
1000
1500
Fluorescence (a.u.)
Systematic Name: YMR105C
Time step Standard Deviation
240
200
160
120
80
200
300
400
Time step Mean
1
2
3
4
5
6
7
8
9
10
11
12
13
14
15
16
17
18
Cluster
(Isolation)
1 (−)
2 (−)
Time Step
15
10
5

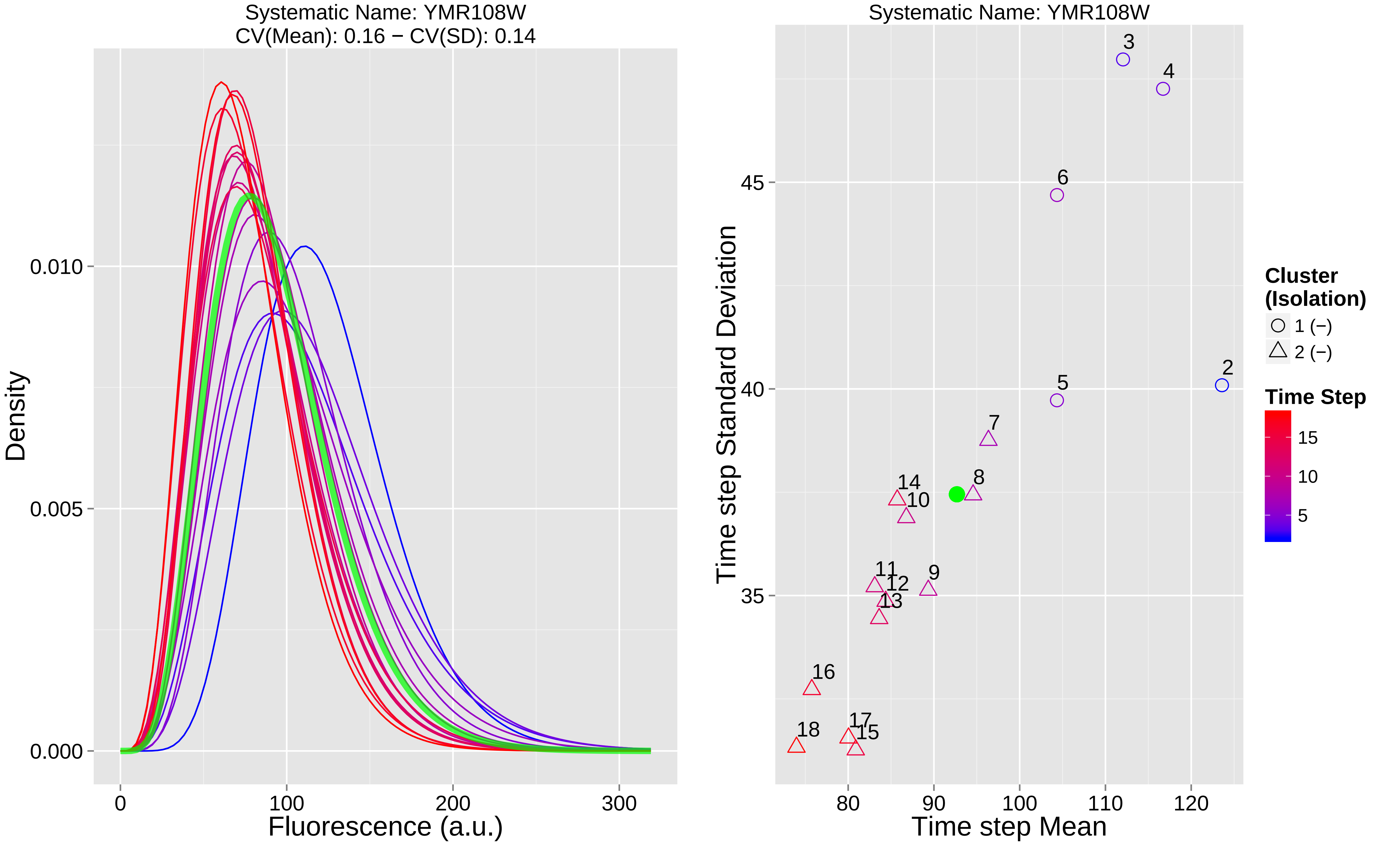
Systematic Name: YMR108W
CV(Mean): 0.16 − CV(SD): 0.14
Density
0.010
0.005
0.000
0
100
200
300
Fluorescence (a.u.)
Systematic Name: YMR108W
Time step Standard Deviation
45
40
35
80
90
100
110
120
Time step Mean
3
4
6
5
2
7
8
14
10
11
12
13
9
16
17
15
18
Cluster
(Isolation)
1 (−)
2 (−)
Time Step
15
10
5

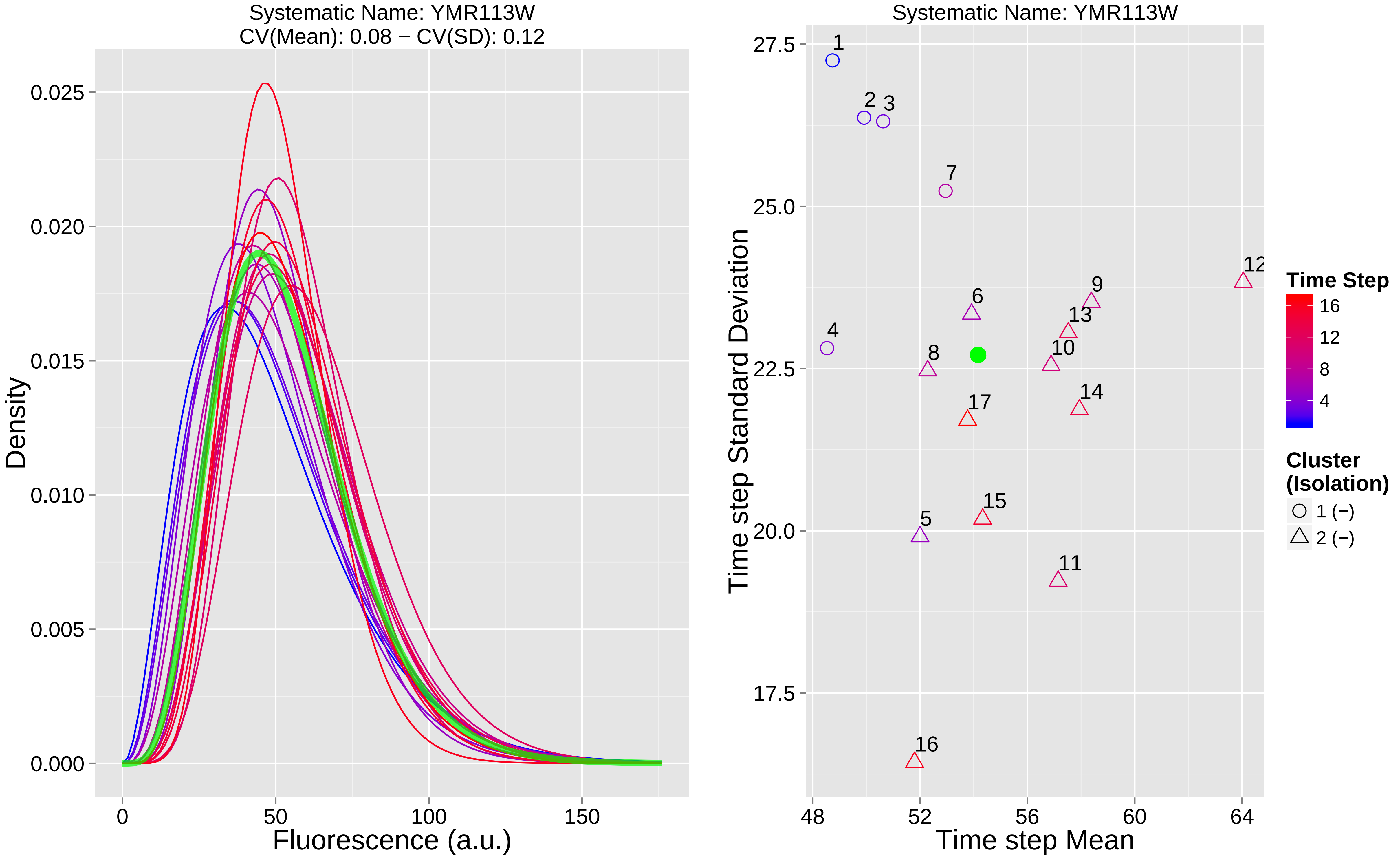

Systematic Name: YMR113W
CV(Mean): 0.08 − CV(SD): 0.12
Density
0.025
0.020
0.015
0.010
0.005
0.000
0
50
100
150
Fluorescence (a.u.)
Systematic Name: YMR113W
Time step Standard Deviation
27.5
25.0
22.5
20.0
17.5
48
52
56
60
64
Time step Mean
1
2
3
4
5
6
7
8
9
10
11
12
13
14
15
16
17
Time Step
16
12
8
4
Cluster
(Isolation)
1 (−)
2 (−)

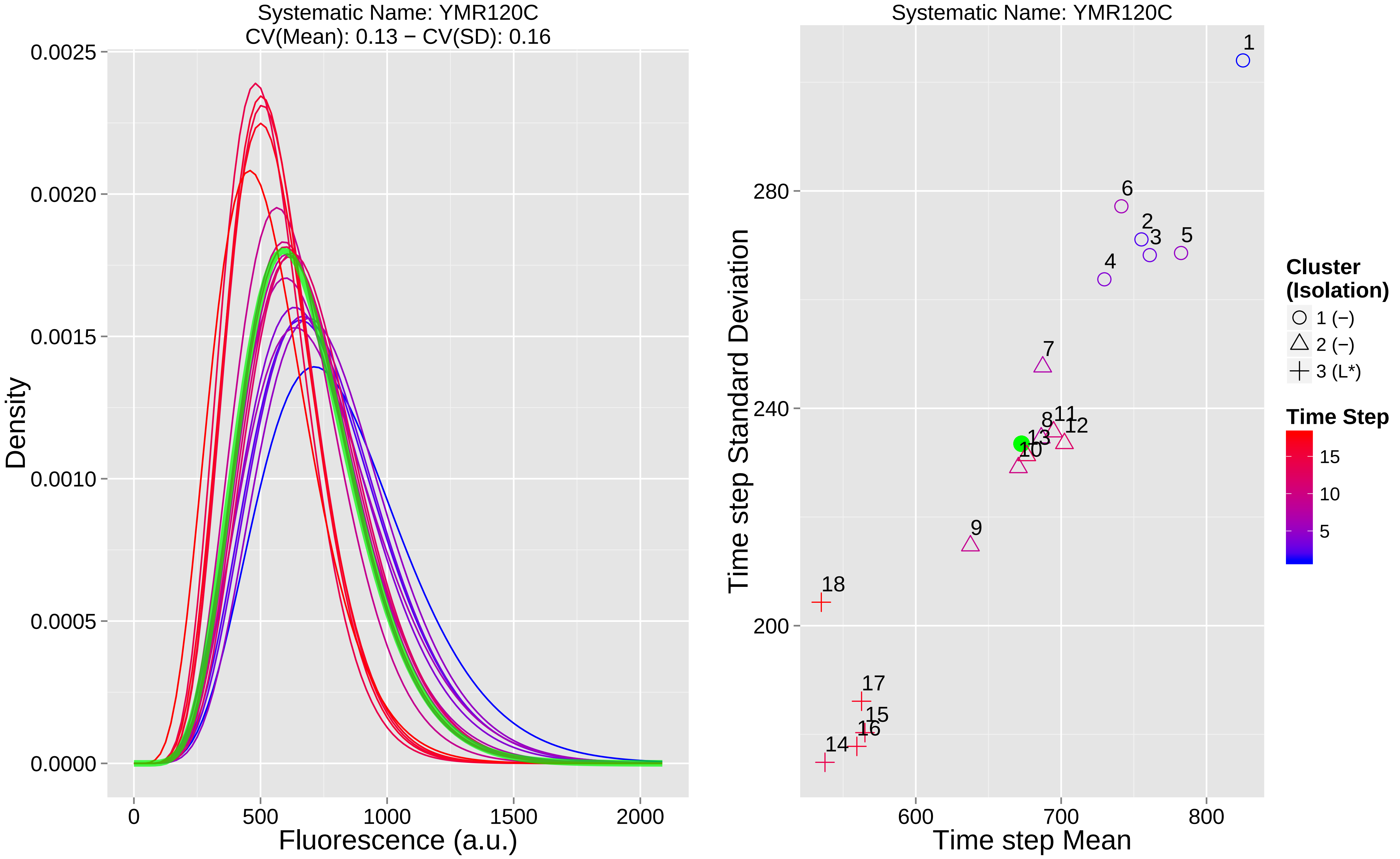

Systematic Name: YMR120C
CV(Mean): 0.13 − CV(SD): 0.16
Density
Fluorescence (a.u.)
0.0025
0.0020
0.0015
0.0010
0.0005
0.0000
0
500
1000
1500
2000
Systematic Name: YMR120C
Time step Standard Deviation
Time step Mean
280
240
200
600
700
800
1
2
3
4
5
6
7
8
9
10
11
12
13
14
15
16
17
18
Cluster (Isolation)
1 (−)
2 (−)
3 (L*)
Time Step
15
10
5

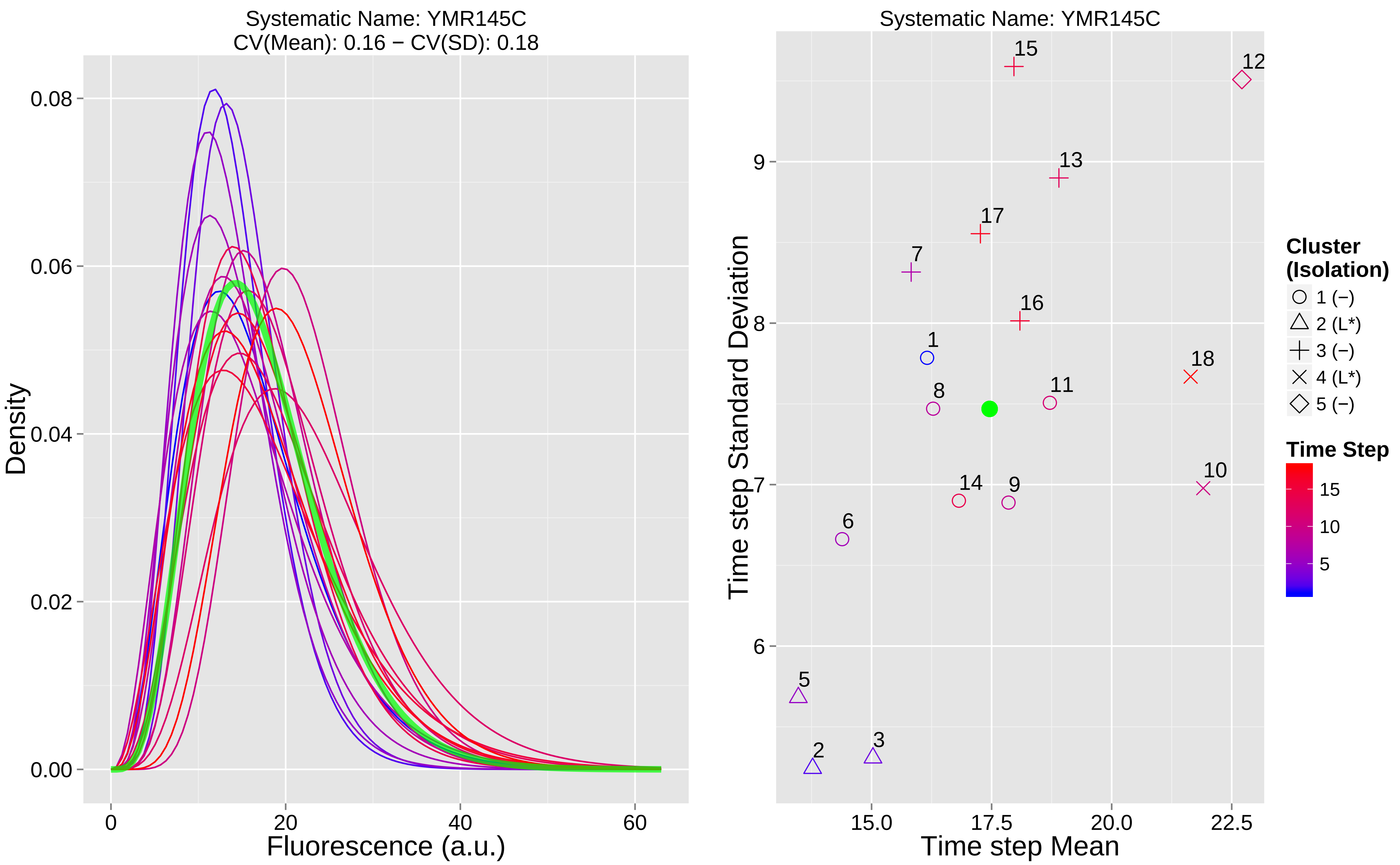

Systematic Name: YMR145C
CV(Mean): 0.16 − CV(SD): 0.18
Density
0.00
0.02
0.04
0.06
0.08
0
20
40
60
Fluorescence (a.u.)
Systematic Name: YMR145C
Time step Standard Deviation
6
7
8
9
15.0
17.5
20.0
22.5
Time step Mean
1
2
3
4
5
6
7
8
9
10
11
12
13
14
15
16
17
18
Cluster (Isolation)
1 (−)
2 (L*)
3 (−)
4 (L*)
5 (−)
Time Step
5
10
15

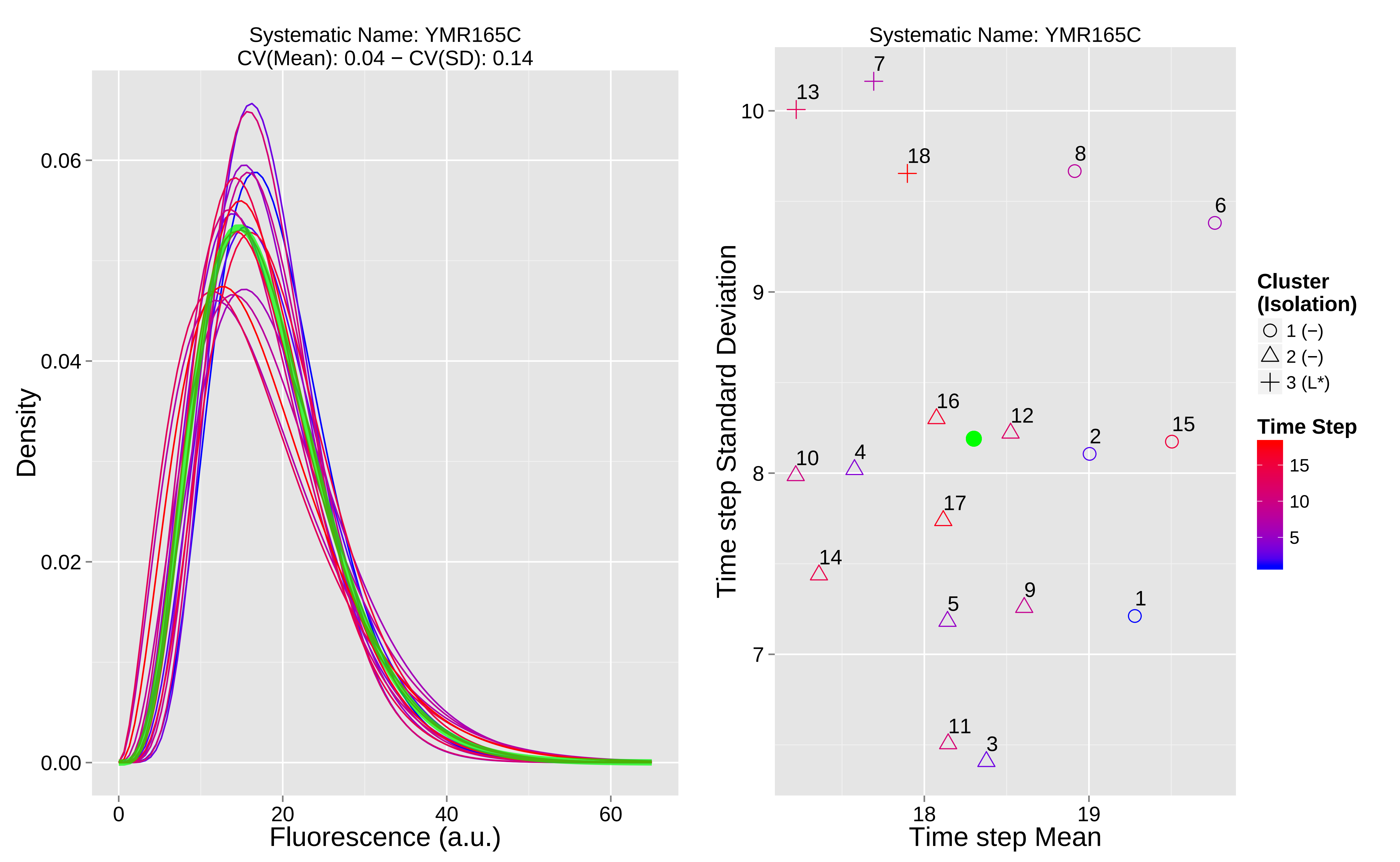

Systematic Name: YMR165C
CV(Mean): 0.04 − CV(SD): 0.14
Density
0.06
0.04
0.02
0.00
0
20
40
60
Fluorescence (a.u.)
Systematic Name: YMR165C
Time step Standard Deviation
10
9
8
7
18
19
Time step Mean
13
7
18
8
6
16
12
2
15
10
4
17
14
5
9
1
11
3
Cluster (Isolation)
1 (−)
2 (−)
3 (L*)
Time Step
15
10
5

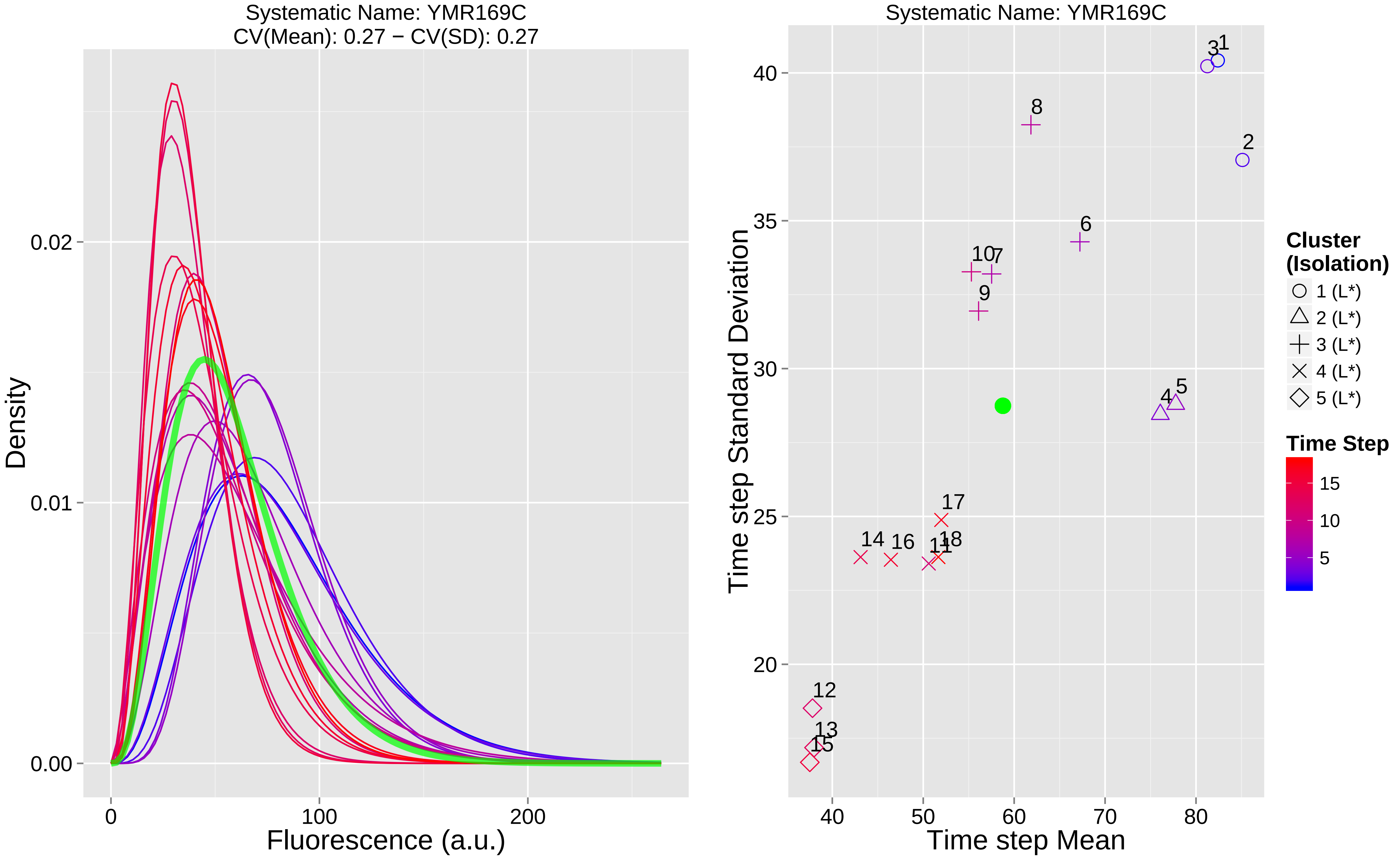
Systematic Name: YMR169C
CV(Mean): 0.27 − CV(SD): 0.27
Density
0.02
0.01
0.00
0
100
200
Fluorescence (a.u.)
Systematic Name: YMR169C
Time step Standard Deviation
40
35
30
25
20
Time step Mean
40
50
60
70
80
Cluster (Isolation)
1 (L*)
2 (L*)
3 (L*)
4 (L*)
5 (L*)
Time Step
15
10
5

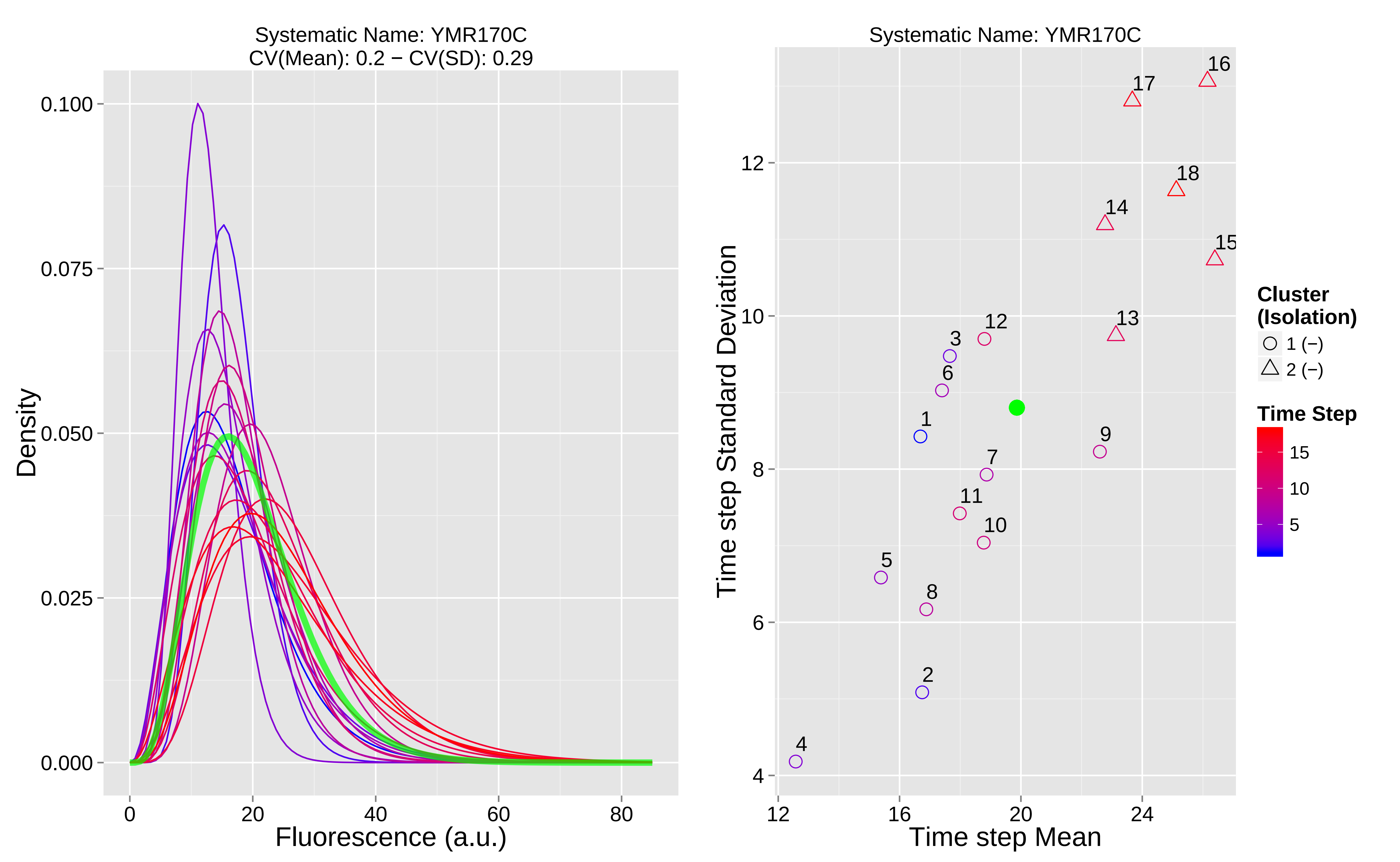

Systematic Name: YMR170C
CV(Mean): 0.2 − CV(SD): 0.29
Density
0.100
0.075
0.050
0.025
0.000
0
20
40
60
80
Fluorescence (a.u.)
Systematic Name: YMR170C
Time step Standard Deviation
Time step Mean
12
10
8
6
4
12
16
20
24
1
2
3
4
5
6
7
8
9
10
11
12
13
14
15
16
17
18
Cluster (Isolation)
1 (−)
2 (−)
Time Step
15
10
5

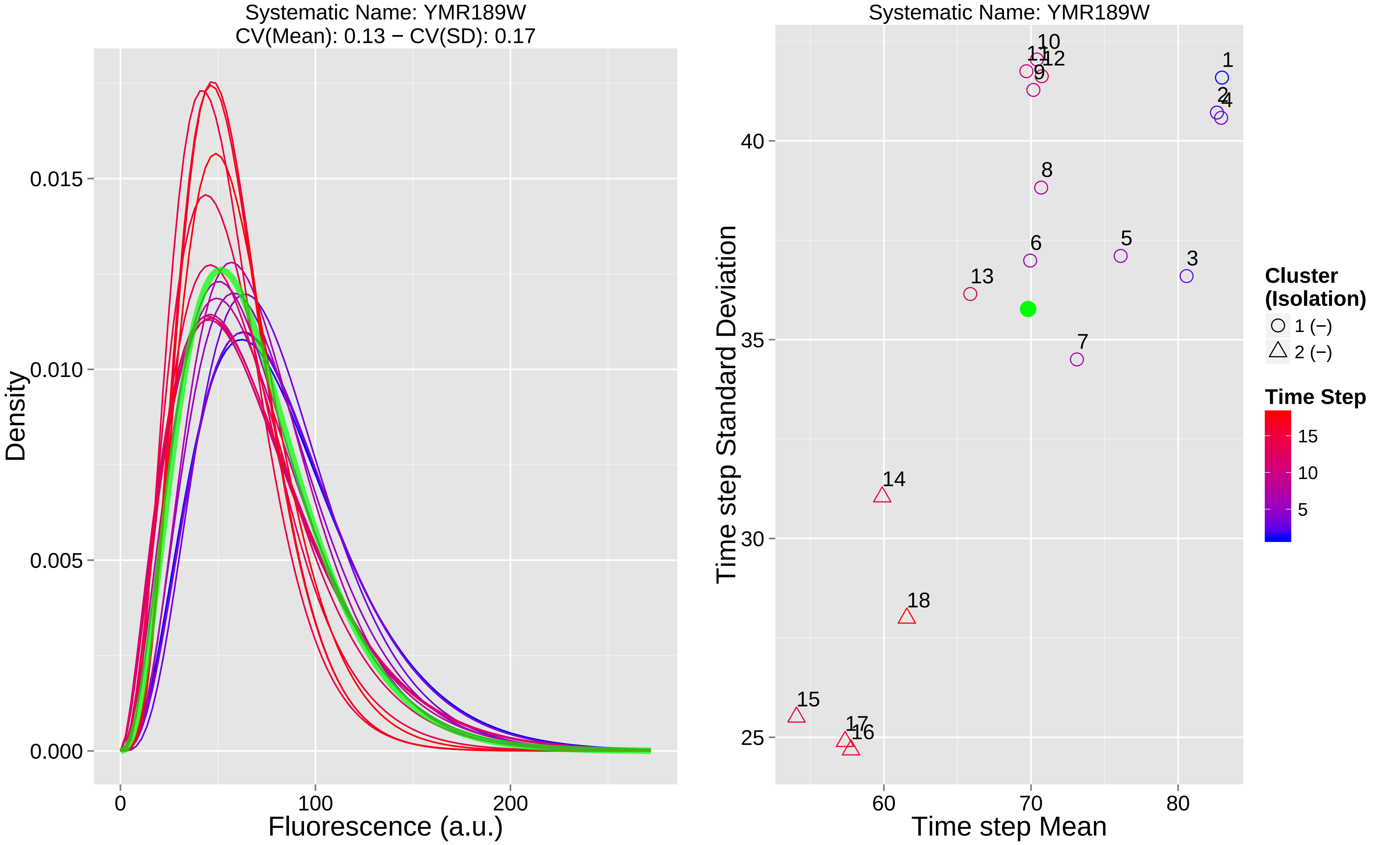

Systematic Name: YMR189W
CV(Mean): 0.13 − CV(SD): 0.17
Density
0.000
0.005
0.010
0.015
0
100
200
Fluorescence (a.u.)
Systematic Name: YMR189W
Time step Standard Deviation
25
30
35
40
60
70
80
Time step Mean
1
2
3
4
5
6
7
8
9
10
11
12
13
14
15
16
17
18
Cluster
(Isolation)
1 (−)
2 (−)
Time Step
5
10
15

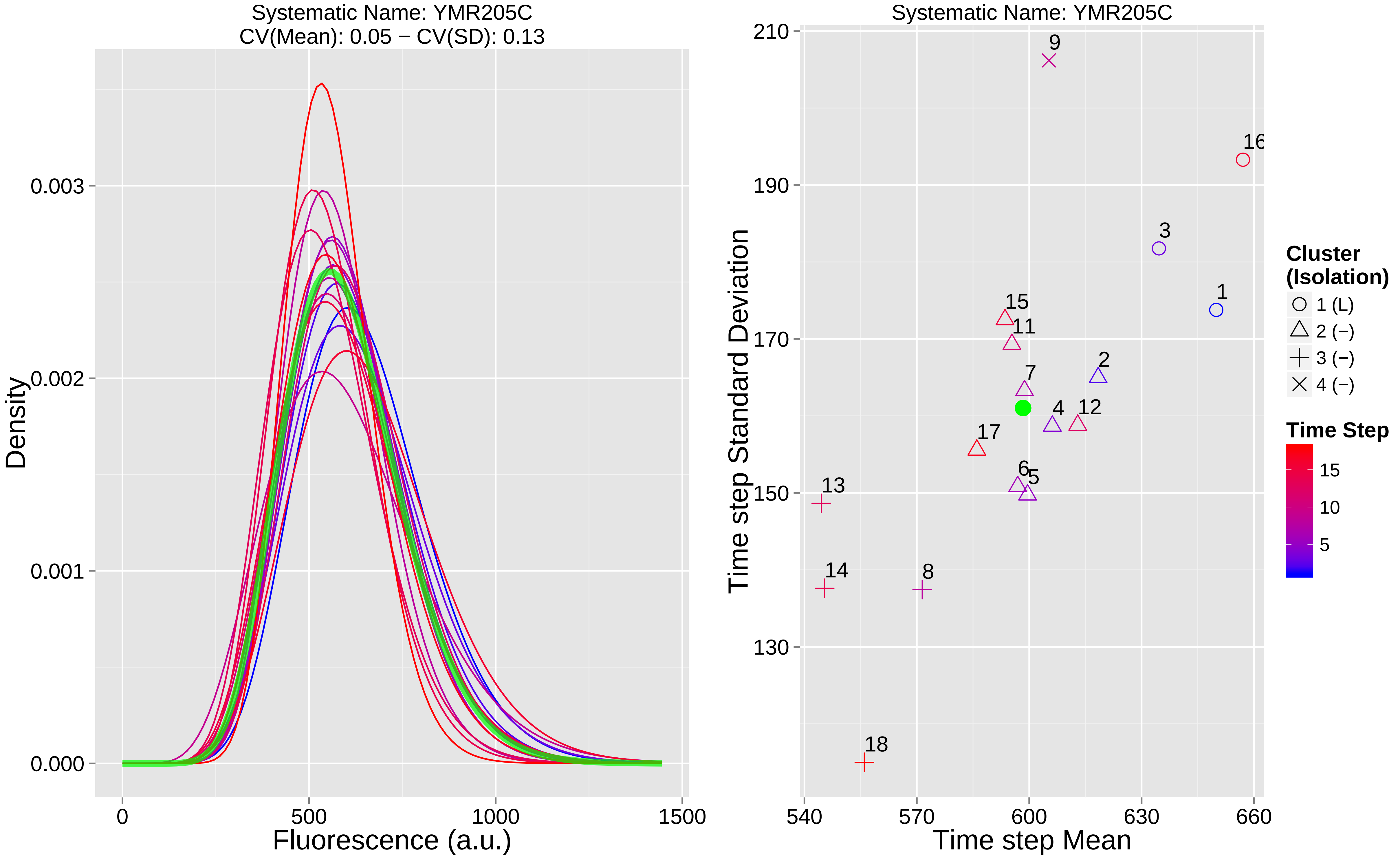

Systematic Name: YMR205C
CV(Mean): 0.05 − CV(SD): 0.13
0.003
0.002
0.001
0.000
Density
0
500
1000
1500
Fluorescence (a.u.)
Systematic Name: YMR205C
210
190
170
150
130
Time step Standard Deviation
540
570
600
630
660
Time step Mean
9
16
3
1
15
11
2
7
4
12
17
6
5
13
14
8
18
Cluster
(Isolation)
1 (L)
2 (−)
3 (−)
4 (−)
Time Step
15
10
5

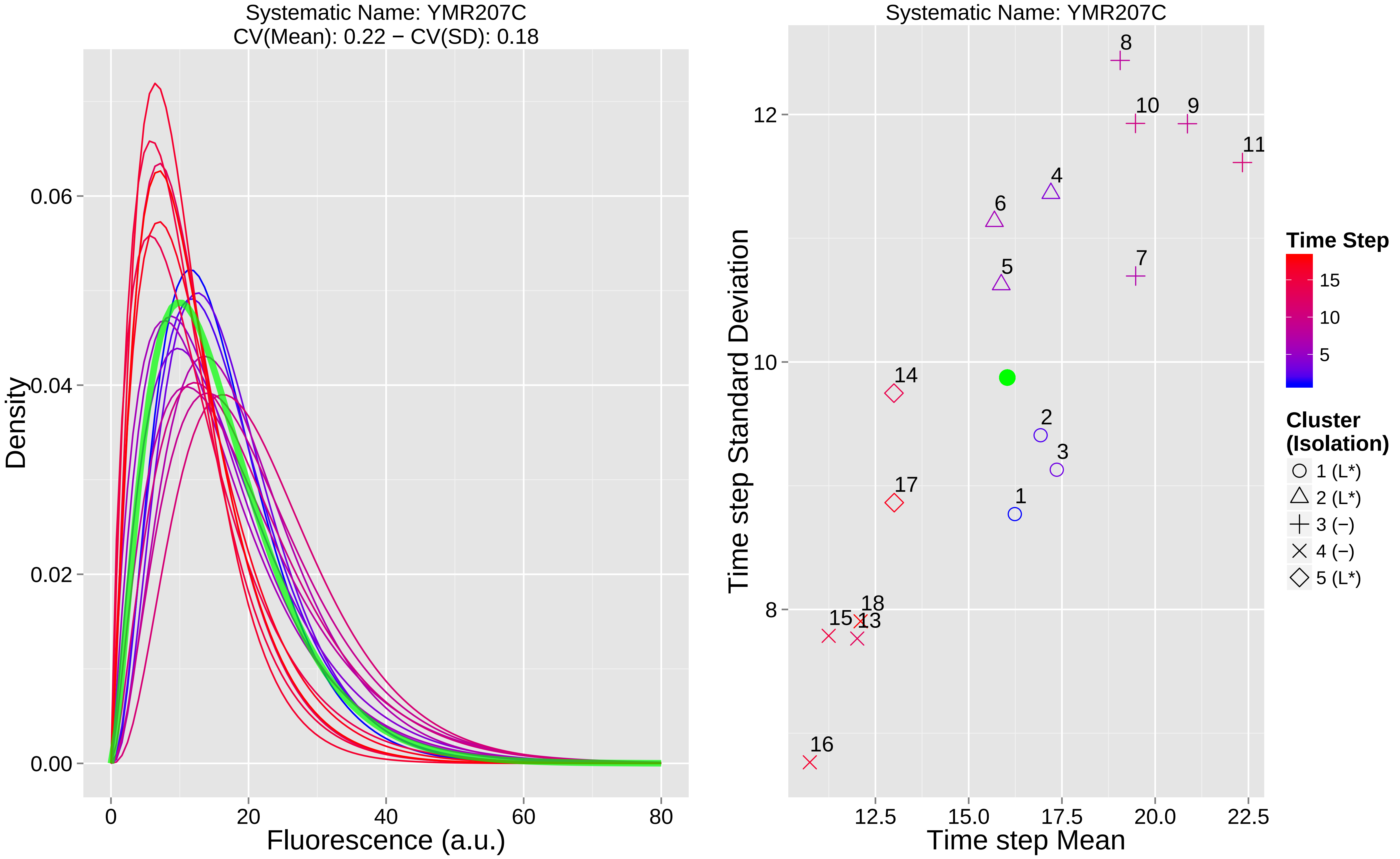

Systematic Name: YMR207C
CV(Mean): 0.22 − CV(SD): 0.18
Density
0.00
0.02
0.04
0.06
0
20
40
60
80
Fluorescence (a.u.)
Systematic Name: YMR207C
Time step Standard Deviation
8
10
12
12.5
15.0
17.5
20.0
22.5
Time step Mean
8
10
9
11
4
6
5
7
14
2
3
17
1
18
15
13
16
Time Step
15
10
5
Cluster (Isolation)
1 (L*)
2 (L*)
3 (−)
4 (−)
5 (L*)

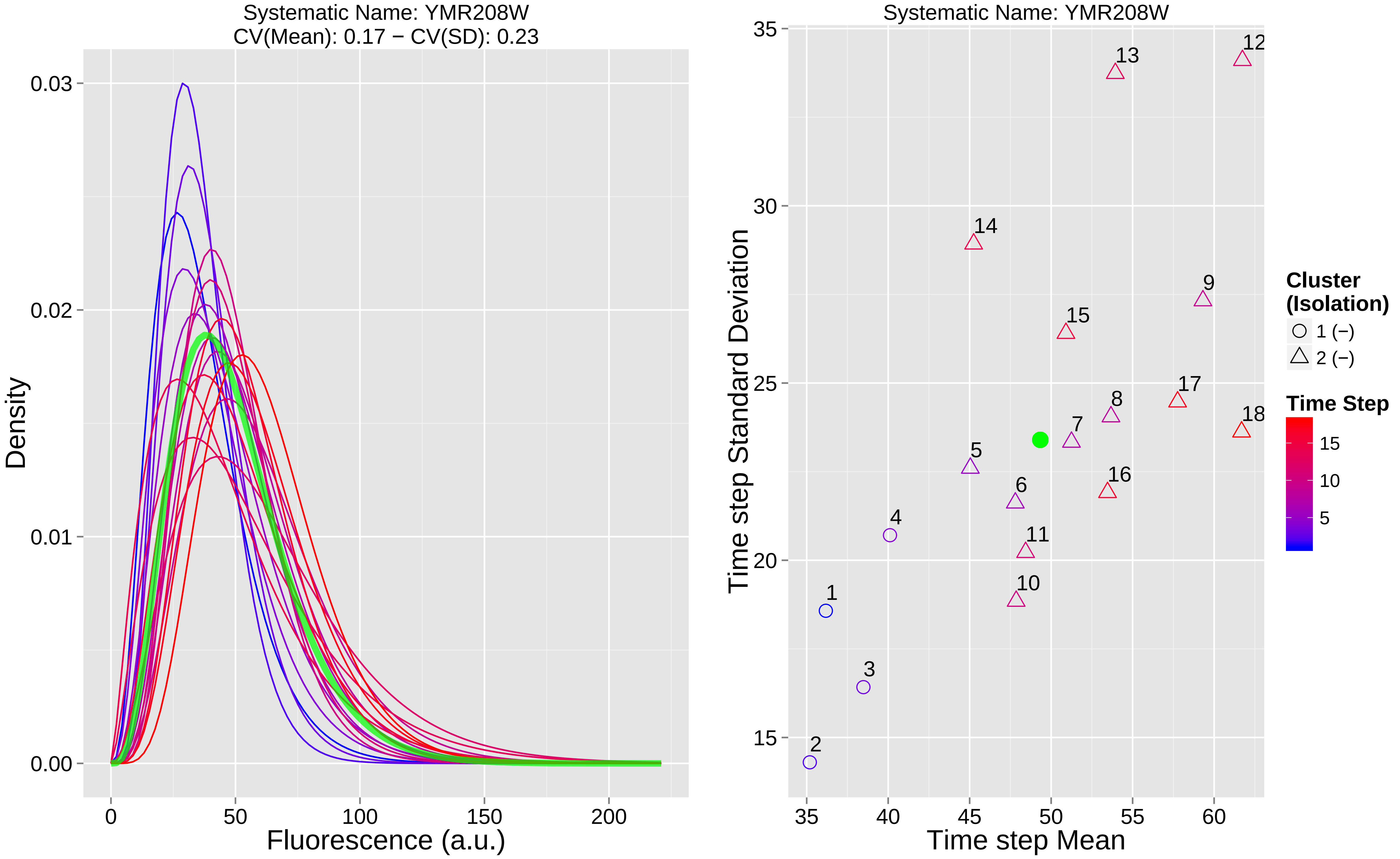

Systematic Name: YMR208W
CV(Mean): 0.17 − CV(SD): 0.23
Density
0.03
0.02
0.01
0.00
0
50
100
150
200
Fluorescence (a.u.)
Systematic Name: YMR208W
Time step Standard Deviation
35
30
25
20
15
35
40
45
50
55
60
Time step Mean
1
2
3
4
5
6
7
8
9
10
11
12
13
14
15
16
17
18
Cluster
(Isolation)
1 (−)
2 (−)
Time Step
15
10
5

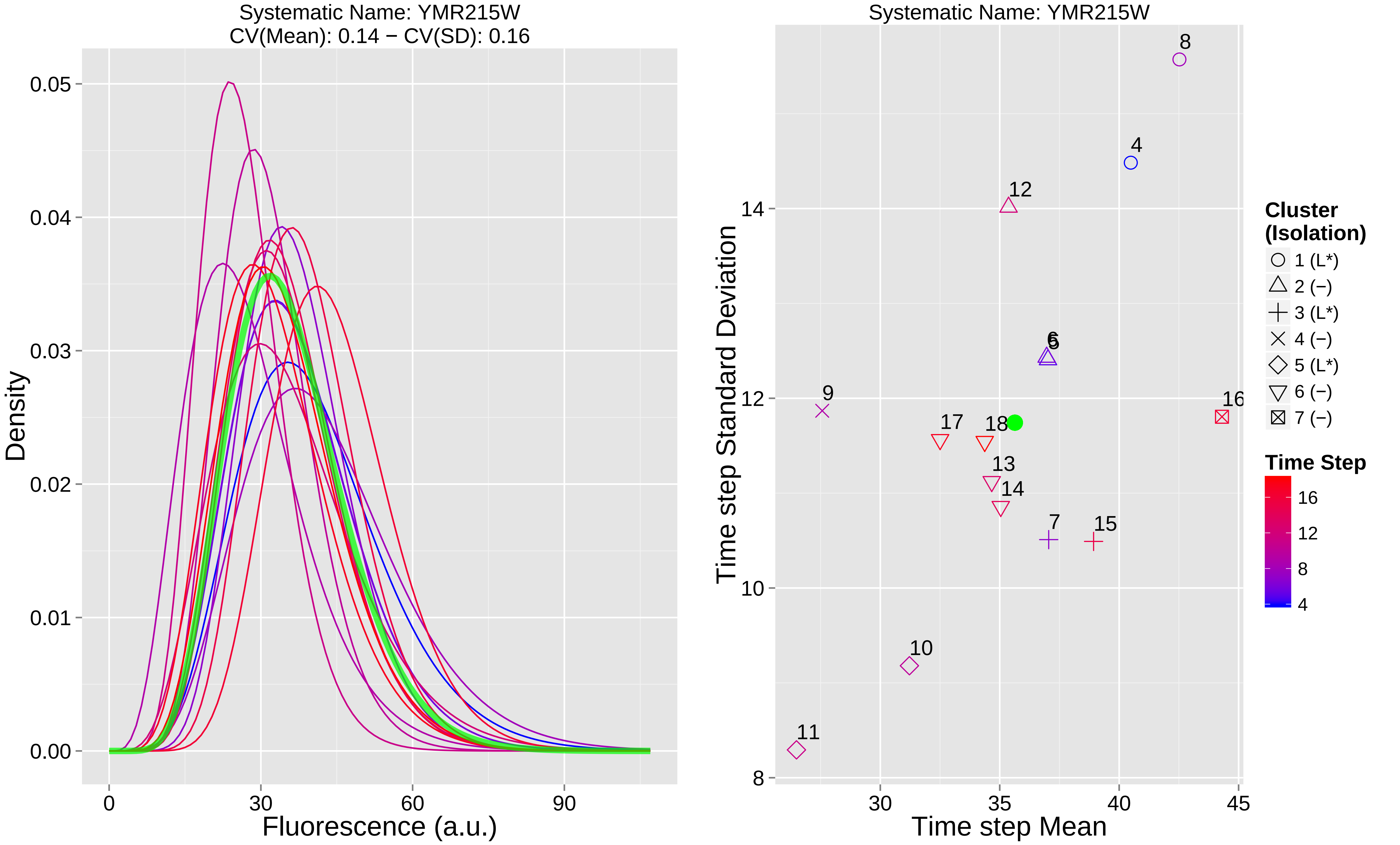
Systematic Name: YMR215W
CV(Mean): 0.14 − CV(SD): 0.16
Density
0.05
0.04
0.03
0.02
0.01
0.00
0
30
60
90
Fluorescence (a.u.)
Systematic Name: YMR215W
Time step Standard Deviation
14
12
10
8
30
35
40
45
Time step Mean
8
4
12
6
5
9
16
17
18
13
14
7
15
10
11
Cluster
(Isolation)
1 (L*)
2 (−)
3 (L*)
4 (−)
5 (L*)
6 (−)
7 (−)
Time Step
16
12
8
4

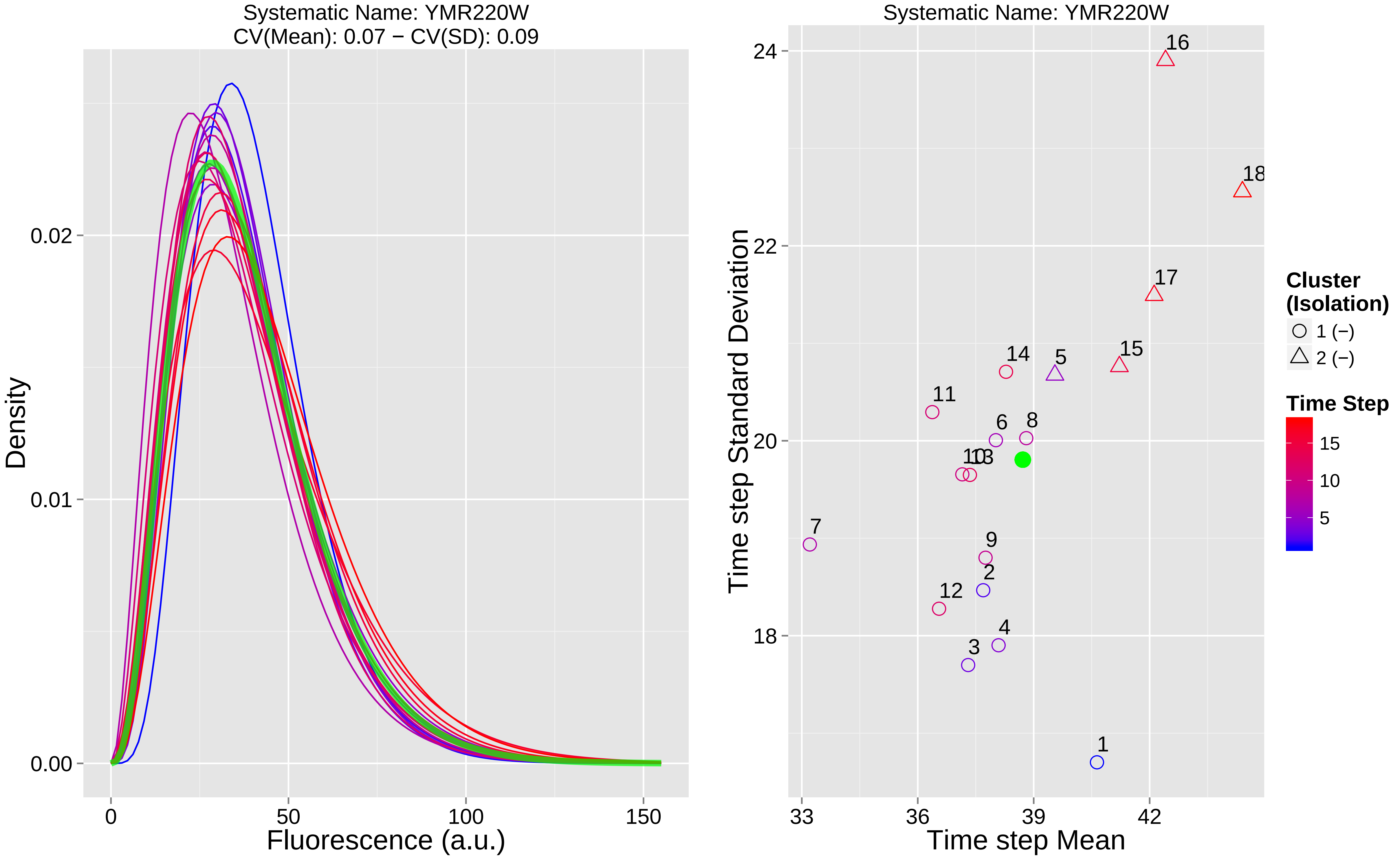

Systematic Name: YMR220W
CV(Mean): 0.07 − CV(SD): 0.09
Density
0.00
0.01
0.02
0
50
100
150
Fluorescence (a.u.)
Systematic Name: YMR220W
Time step Standard Deviation
18
20
22
24
33
36
39
42
Time step Mean
1
2
3
4
5
6
7
8
9
10
11
12
13
14
15
16
17
18
Cluster
(Isolation)
1 (−)
2 (−)
Time Step
5
10
15

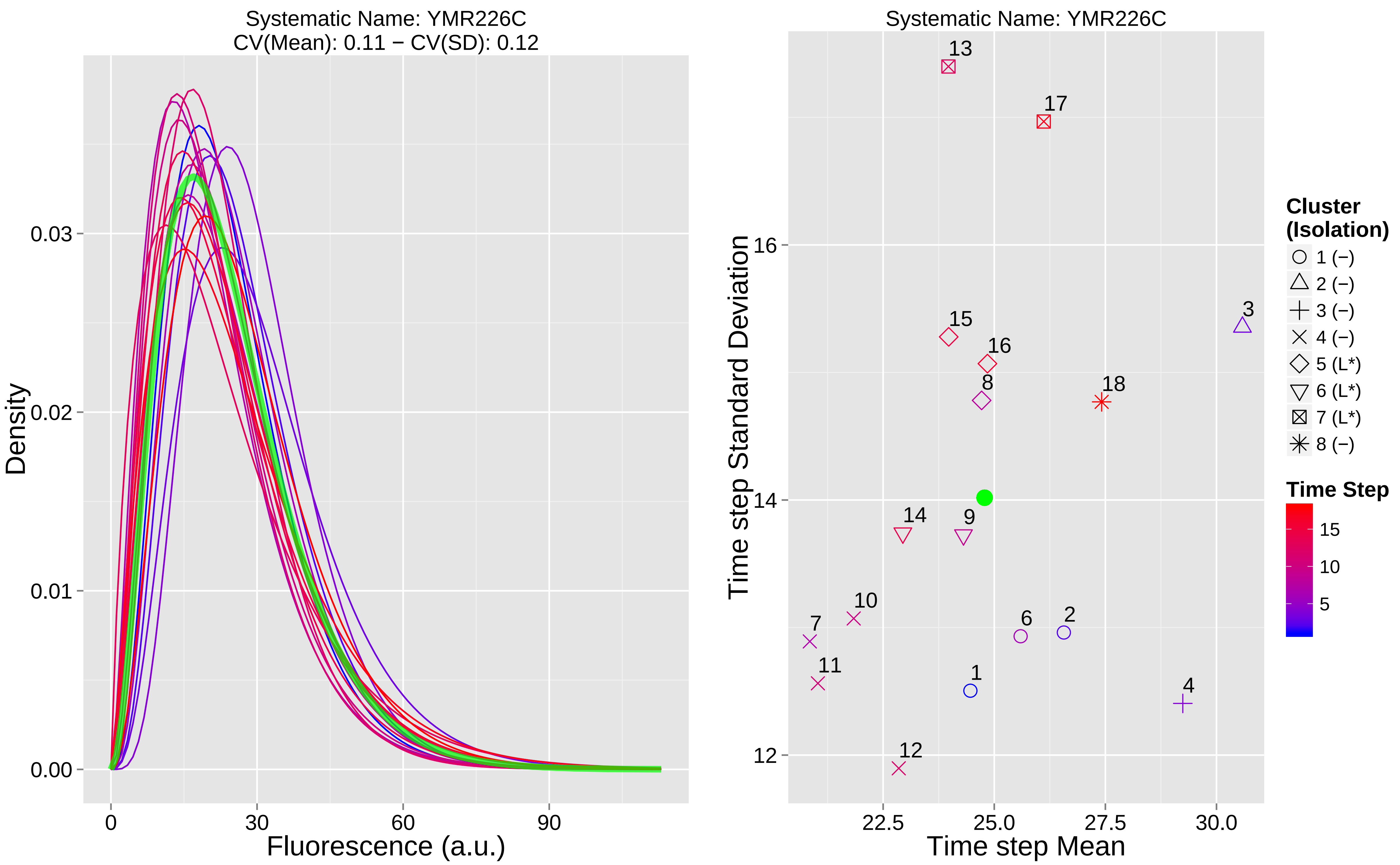

Systematic Name: YMR226C
CV(Mean): 0.11 − CV(SD): 0.12
Density
0.03
0.02
0.01
0.00
0
30
60
90
Fluorescence (a.u.)
Systematic Name: YMR226C
Time step Standard Deviation
16
14
12
22.5
25.0
27.5
30.0
Time step Mean
13
17
15
16
8
18
3
14
9
10
7
6
2
11
1
4
12
Cluster
(Isolation)
1 (−)
2 (−)
3 (−)
4 (−)
5 (L*)
6 (L*)
7 (L*)
8 (−)
Time Step
15
10
5

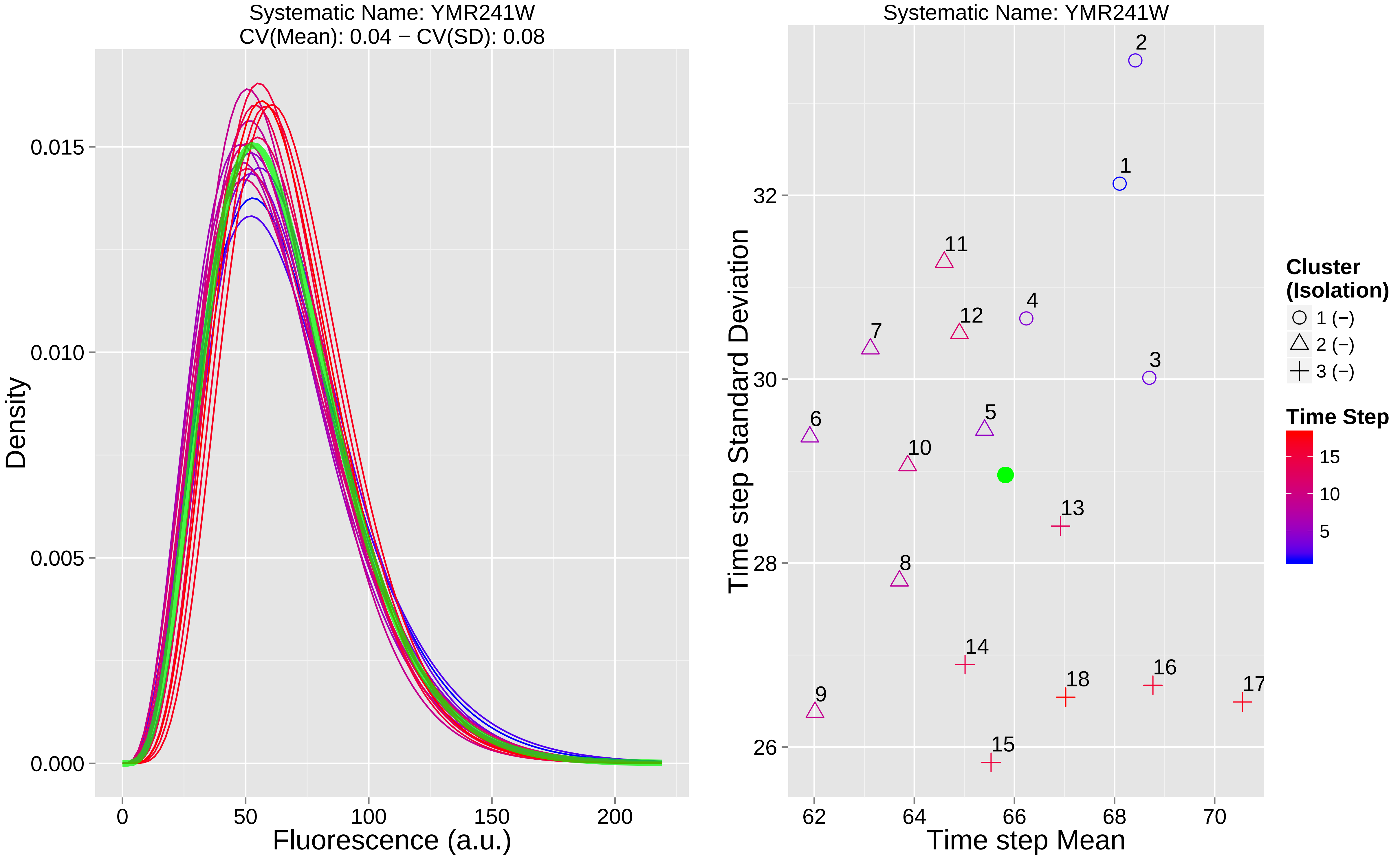

Systematic Name: YMR241W
CV(Mean): 0.04 − CV(SD): 0.08
Density
0.015
0.010
0.005
0.000
0
50
100
150
200
Fluorescence (a.u.)
Systematic Name: YMR241W
Time step Standard Deviation
32
30
28
26
62
64
66
68
70
Time step Mean
1
2
3
4
5
6
7
8
9
10
11
12
13
14
15
16
17
18
Cluster (Isolation)
1 (−)
2 (−)
3 (−)
Time Step
15
10
5

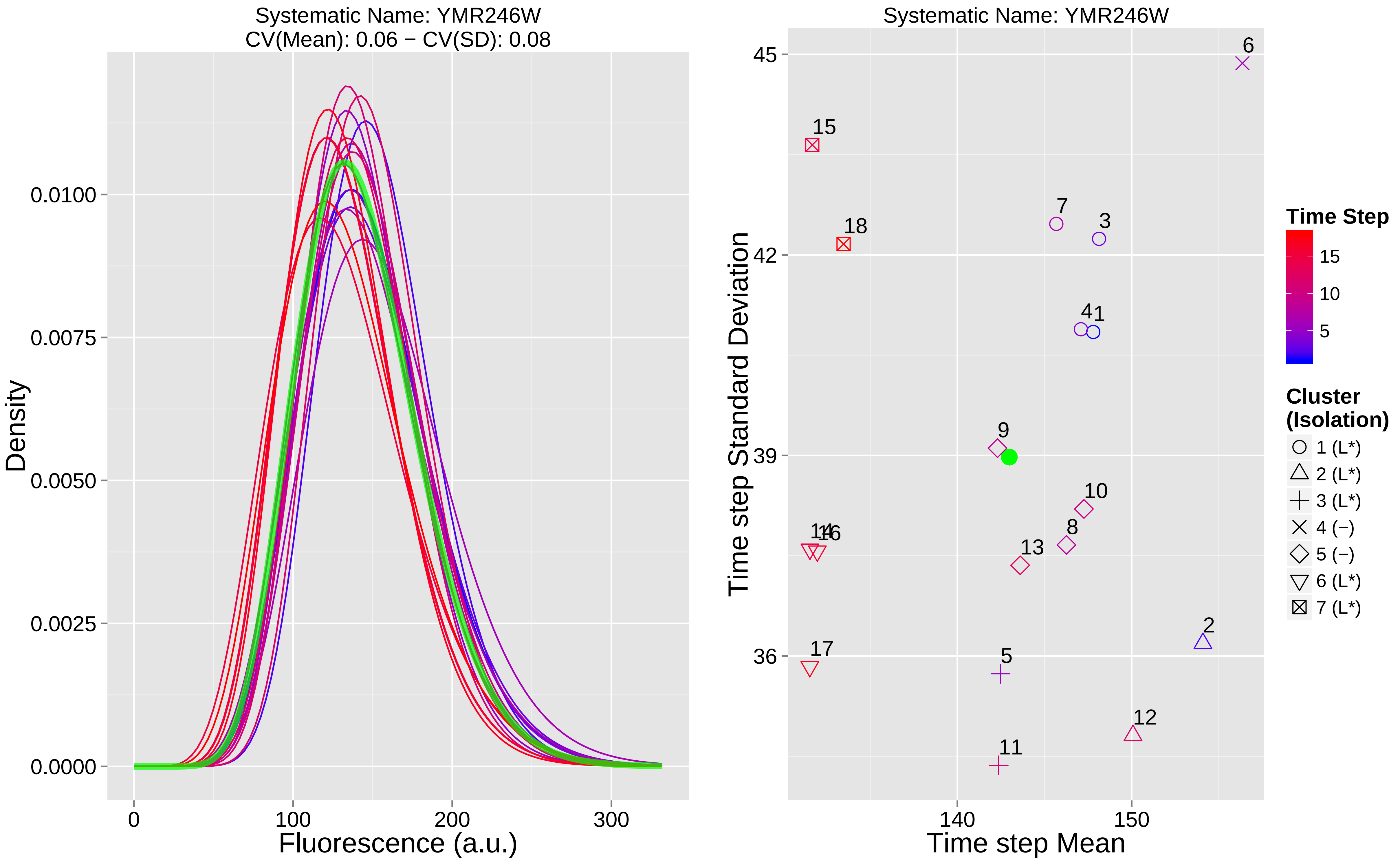

Systematic Name: YMR246W
CV(Mean): 0.06 − CV(SD): 0.08
Density
0.0000
0.0025
0.0050
0.0075
0.0100
0
100
200
300
Fluorescence (a.u.)
Systematic Name: YMR246W
Time step Standard Deviation
Time step Mean
45
42
39
36
140
150
6
15
18
7
3
41
9
10
8
13
14
16
17
5
2
12
11
Time Step
15
10
5
Cluster
(Isolation)
1 (L*)
2 (L*)
3 (L*)
4 (−)
5 (−)
6 (L*)
7 (L*)

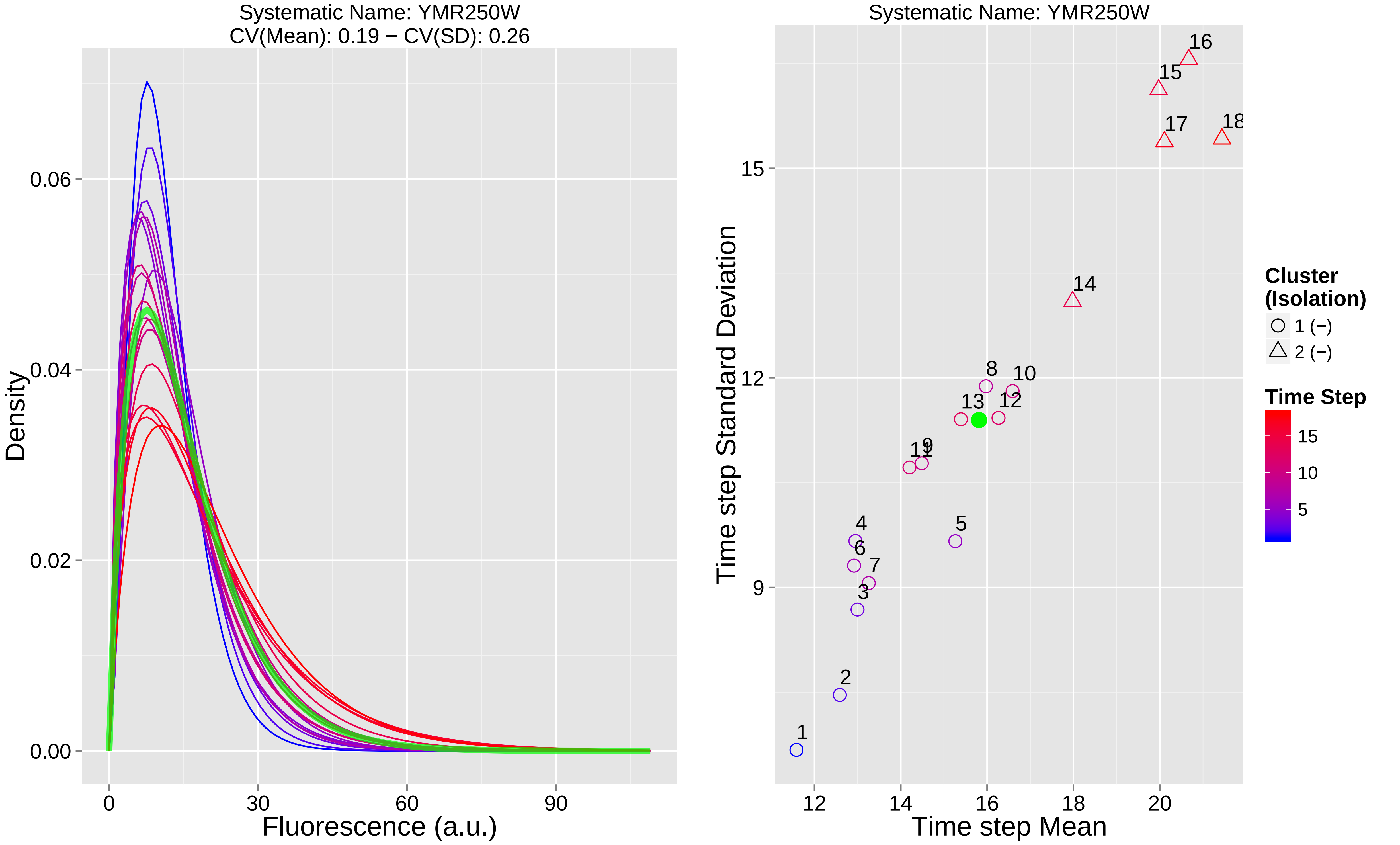

Systematic Name: YMR250W
CV(Mean): 0.19 − CV(SD): 0.26
Density
0.06
0.04
0.02
0.00
0
30
60
90
Fluorescence (a.u.)
Systematic Name: YMR250W
Time step Standard Deviation
15
12
9
12
14
16
18
20
Time step Mean
Cluster (Isolation)
1 (−)
2 (−)
Time Step
15
10
5

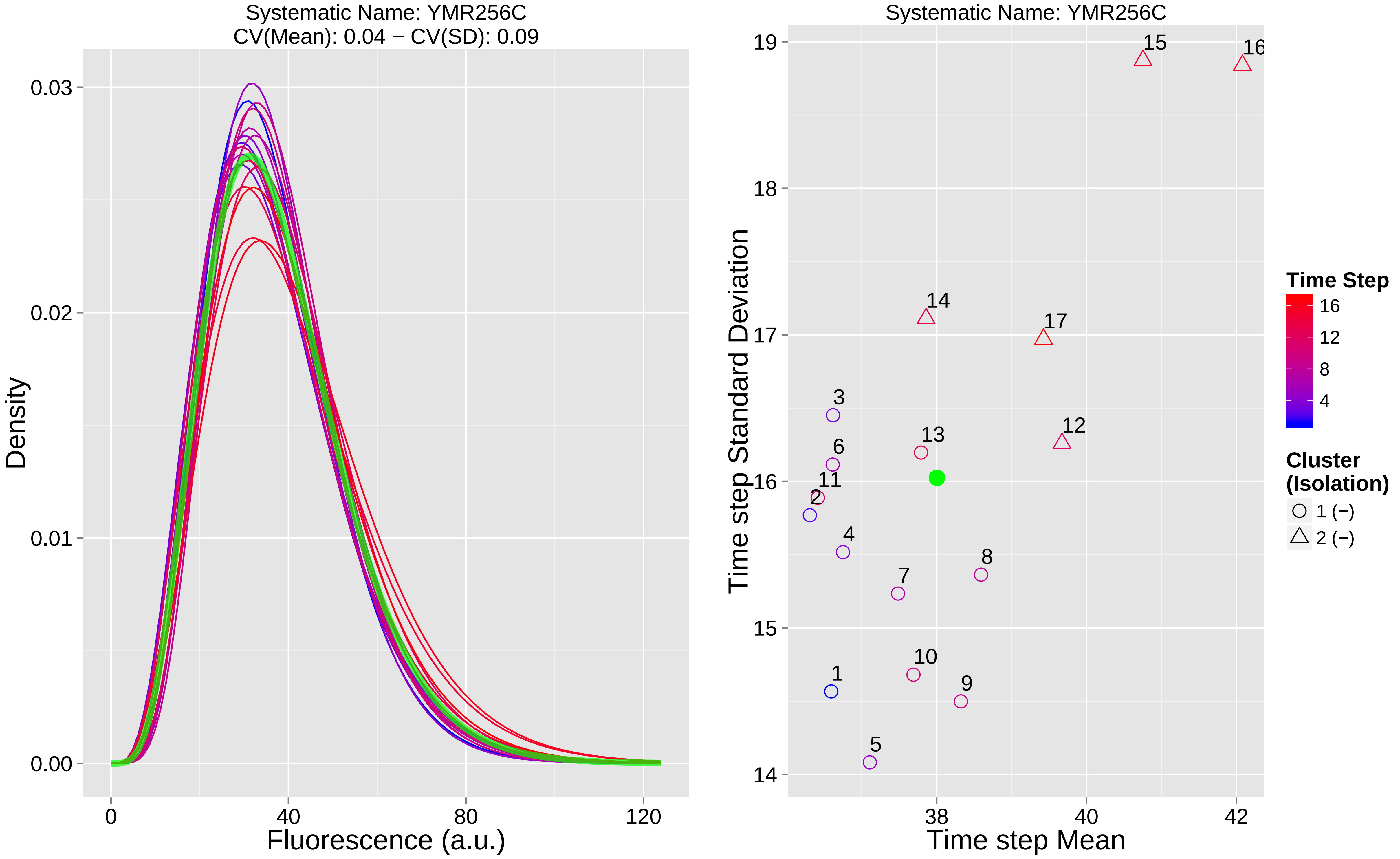

Systematic Name: YMR256C
CV(Mean): 0.04 − CV(SD): 0.09
0.03
0.02
0.01
0.00
Density
0
40
80
120
Fluorescence (a.u.)
Systematic Name: YMR256C
19
18
17
16
15
14
Time step Standard Deviation
38
40
42
Time step Mean
1
2
3
4
5
6
7
8
9
10
11
12
13
14
15
16
17
Time Step
16
12
8
4
Cluster (Isolation)
1 (−)
2 (−)

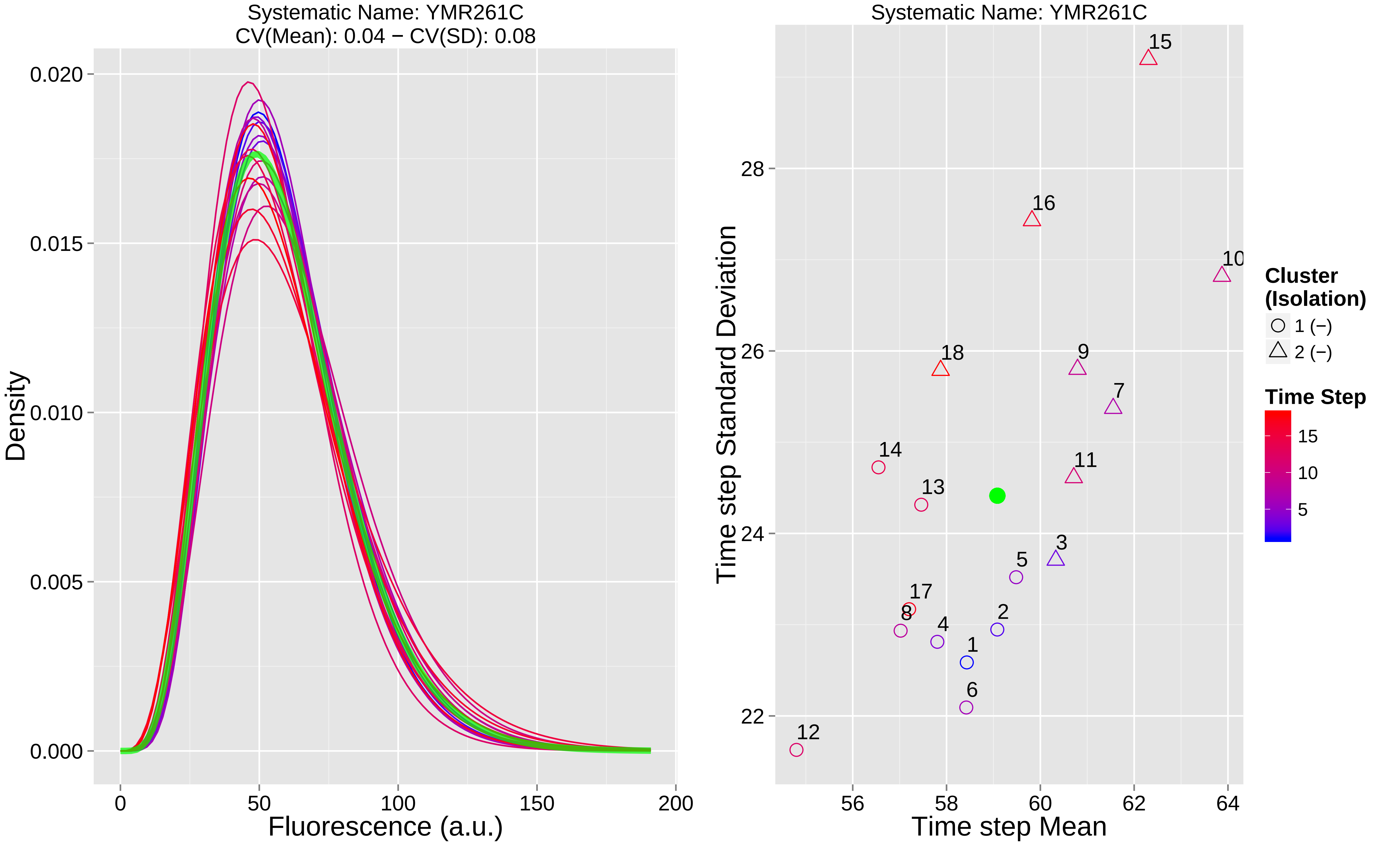

Systematic Name: YMR261C
CV(Mean): 0.04 − CV(SD): 0.08
Density
Fluorescence (a.u.)
0.000
0.005
0.010
0.015
0.020
0
50
100
150
200
Systematic Name: YMR261C
Time step Standard Deviation
Time step Mean
22
24
26
28
56
58
60
62
64
1
2
3
4
5
6
7
8
9
10
11
12
13
14
15
16
17
18
Cluster (Isolation)
1 (−)
2 (−)
Time Step
5
10
15

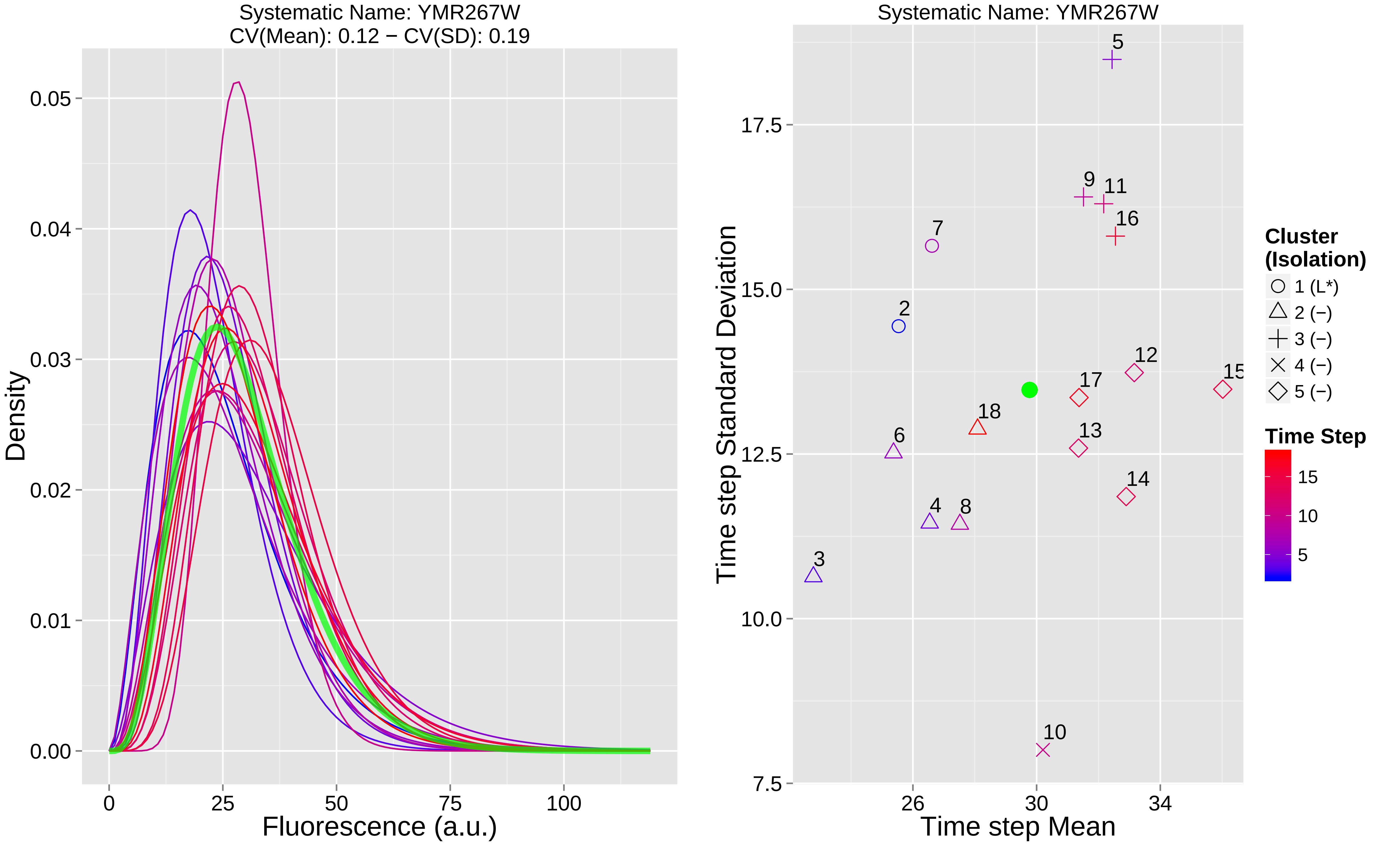

Systematic Name: YMR267W
CV(Mean): 0.12 − CV(SD): 0.19
Density
Fluorescence (a.u.)
0.05
0.04
0.03
0.02
0.01
0.00
0
25
50
75
100
Systematic Name: YMR267W
Time step Standard Deviation
Time step Mean
17.5
15.0
12.5
10.0
7.5
26
30
34
Cluster (Isolation)
1 (L*)
2 (−)
3 (−)
4 (−)
5 (−)
Time Step
15
10
5

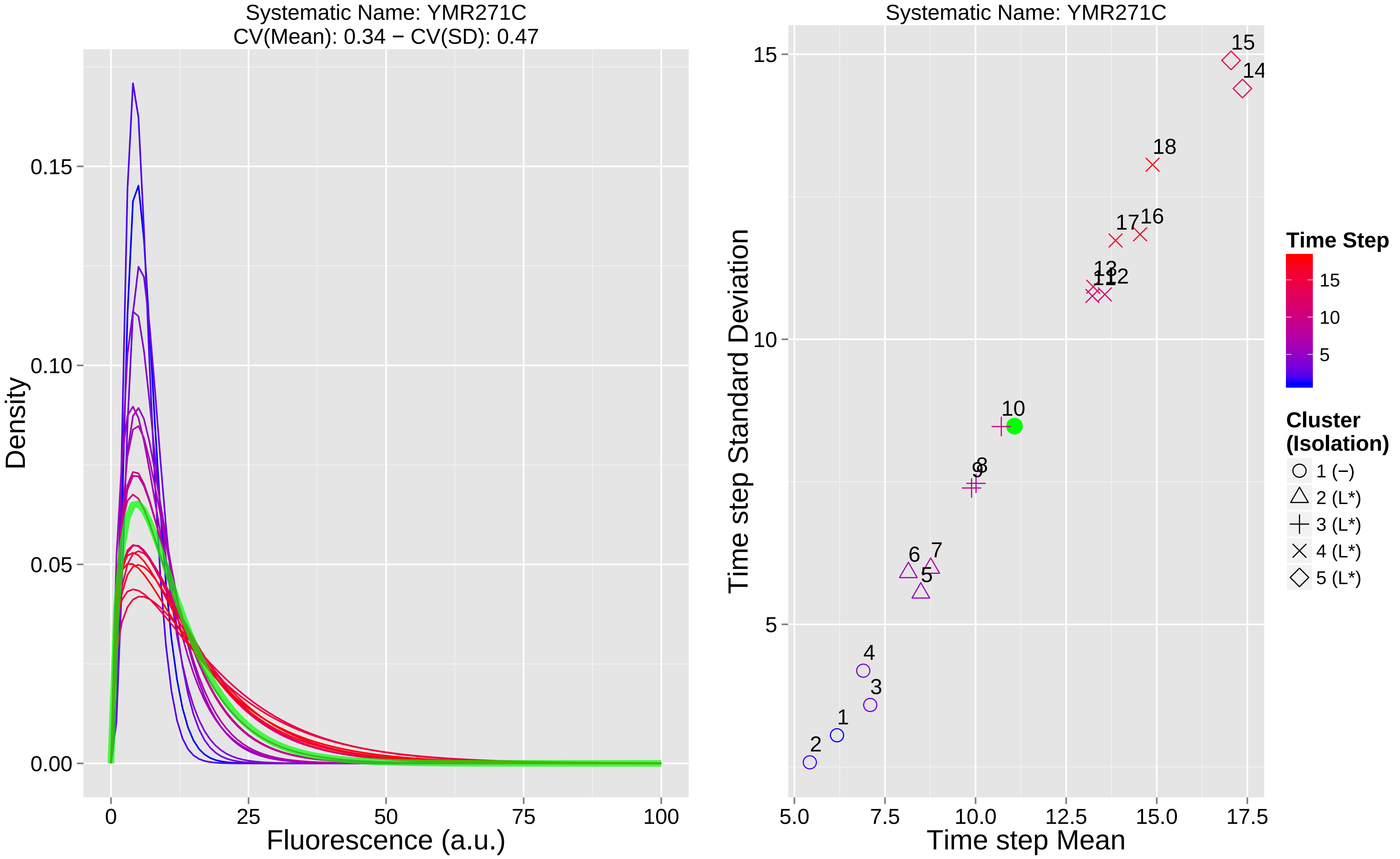
Systematic Name: YMR271C
CV(Mean): 0.34 − CV(SD): 0.47
Density
Fluorescence (a.u.)
0.00
0.05
0.10
0.15
0
25
50
75
100
Systematic Name: YMR271C
Time step Standard Deviation
Time step Mean
5.0
7.5
10.0
12.5
15.0
17.5
5
10
15
Time Step
15
10
5
Cluster (Isolation)
1 (−)
2 (L*)
3 (L*)
4 (L*)
5 (L*)

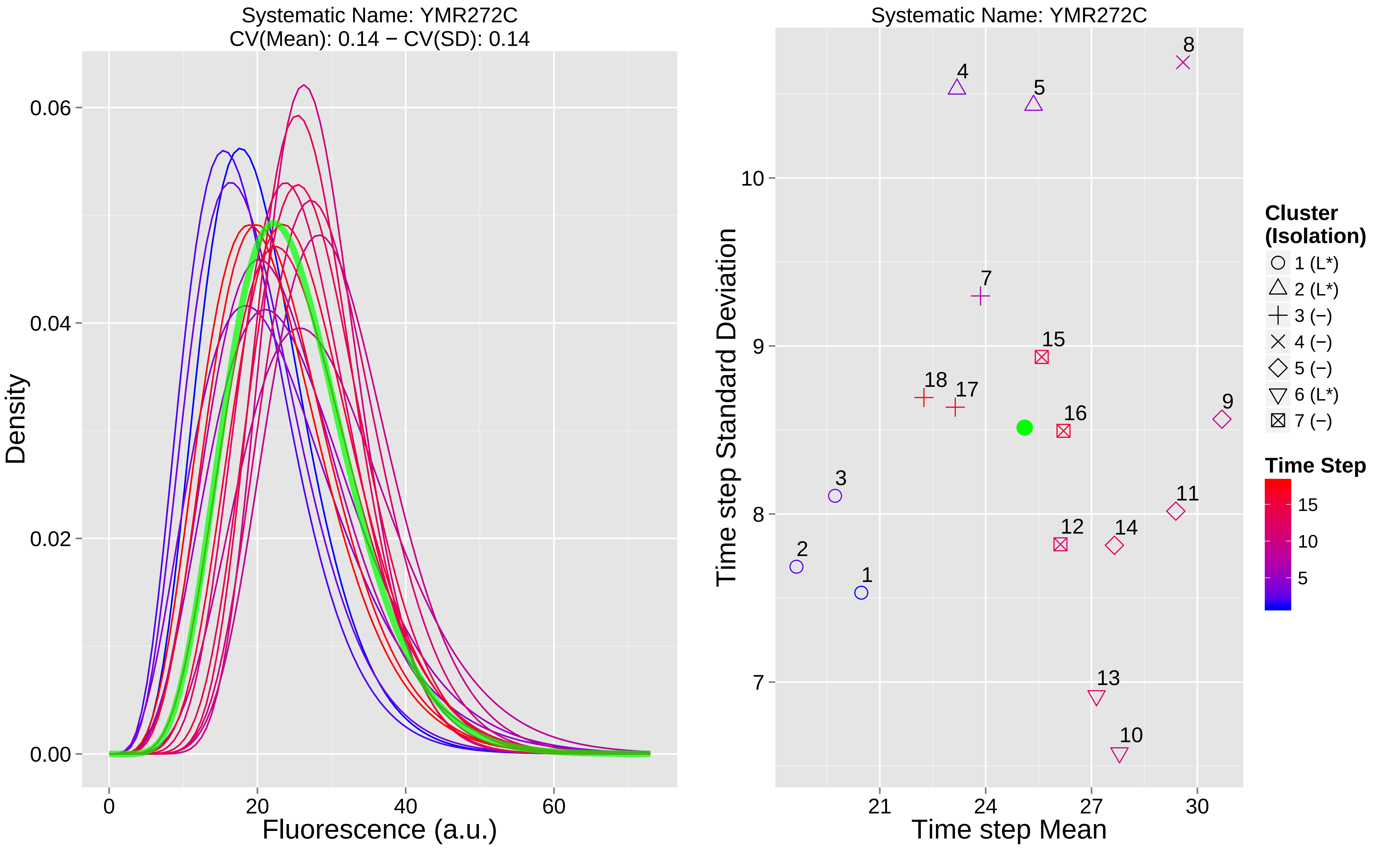

Systematic Name: YMR272C
CV(Mean): 0.14 − CV(SD): 0.14
Density
0.06
0.04
0.02
0.00
0
20
40
60
Fluorescence (a.u.)
Systematic Name: YMR272C
Time step Standard Deviation
10
9
8
7
21
24
27
30
Time step Mean
1
2
3
4
5
7
8
9
10
11
12
13
14
15
16
17
18
Cluster
(Isolation)
1 (L*)
2 (L*)
3 (−)
4 (−)
5 (−)
6 (L*)
7 (−)
Time Step
15
10
5

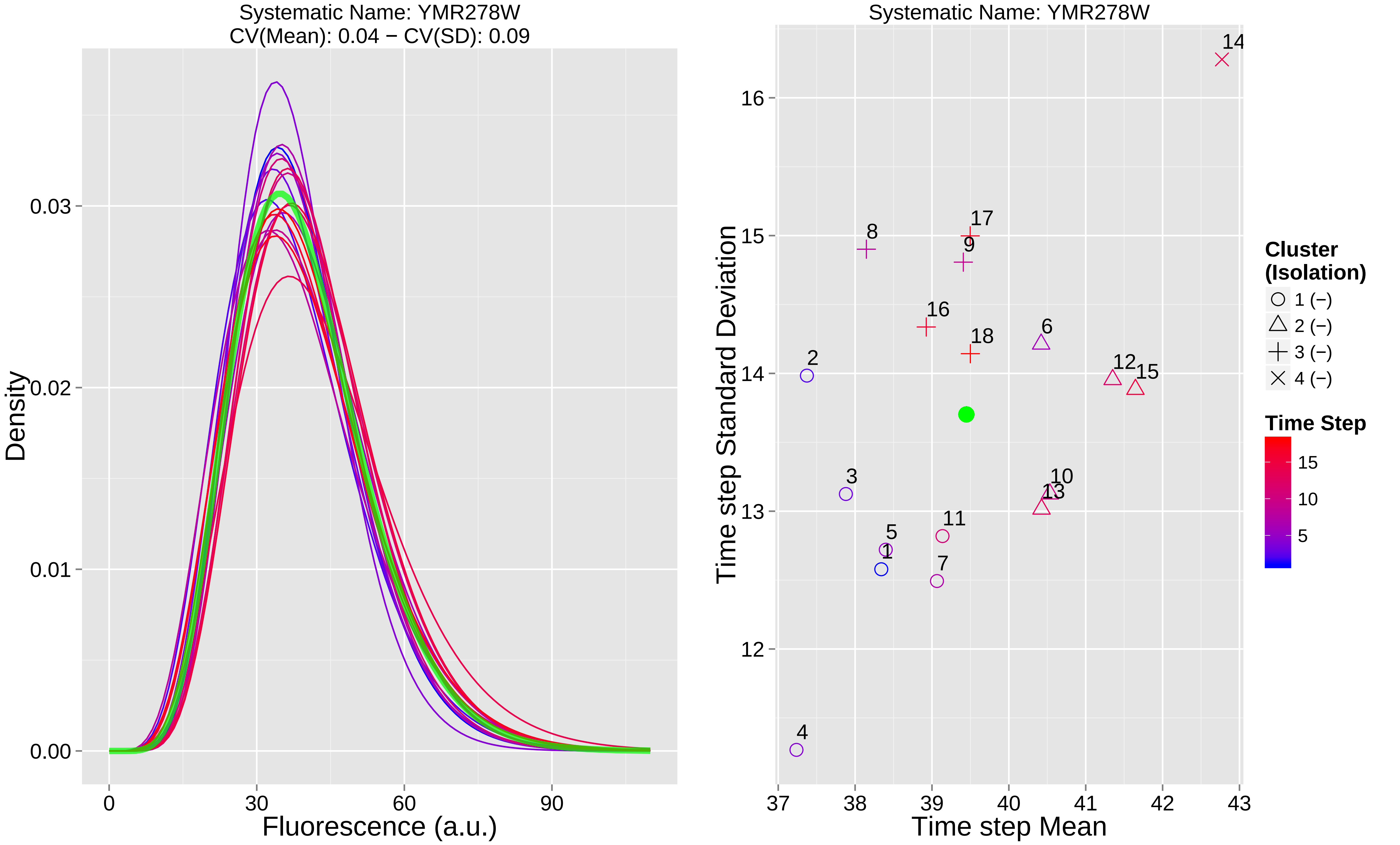

Systematic Name: YMR278W
CV(Mean): 0.04 − CV(SD): 0.09
Density
Fluorescence (a.u.)
0.00
0.01
0.02
0.03
0
30
60
90
Systematic Name: YMR278W
Time step Standard Deviation
Time step Mean
12
13
14
15
16
37
38
39
40
41
42
43
1
2
3
4
5
6
7
8
9
10
11
12
13
14
15
16
17
18
Cluster (Isolation)
1 (−)
2 (−)
3 (−)
4 (−)
Time Step
15
10
5

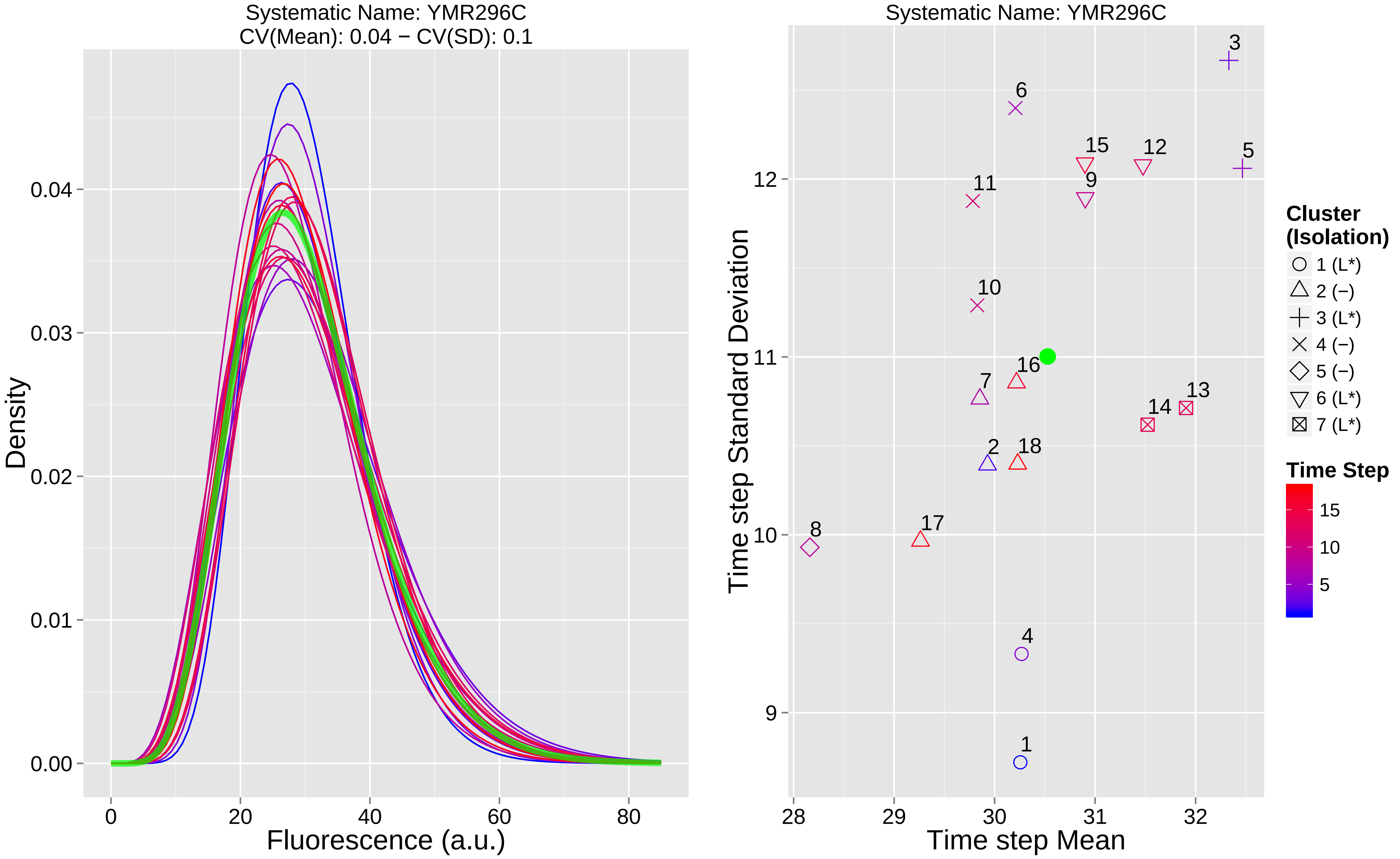

Systematic Name: YMR296C
CV(Mean): 0.04 − CV(SD): 0.1
Density
Fluorescence (a.u.)
0.00
0.01
0.02
0.03
0.04
0
20
40
60
80
Systematic Name: YMR296C
Time step Standard Deviation
Time step Mean
9
10
11
12
28
29
30
31
32
1
2
3
4
5
6
7
8
9
10
11
12
13
14
15
16
17
18
Cluster (Isolation)
1 (L*)
2 (−)
3 (L*)
4 (−)
5 (−)
6 (L*)
7 (L*)
Time Step
15
10
5

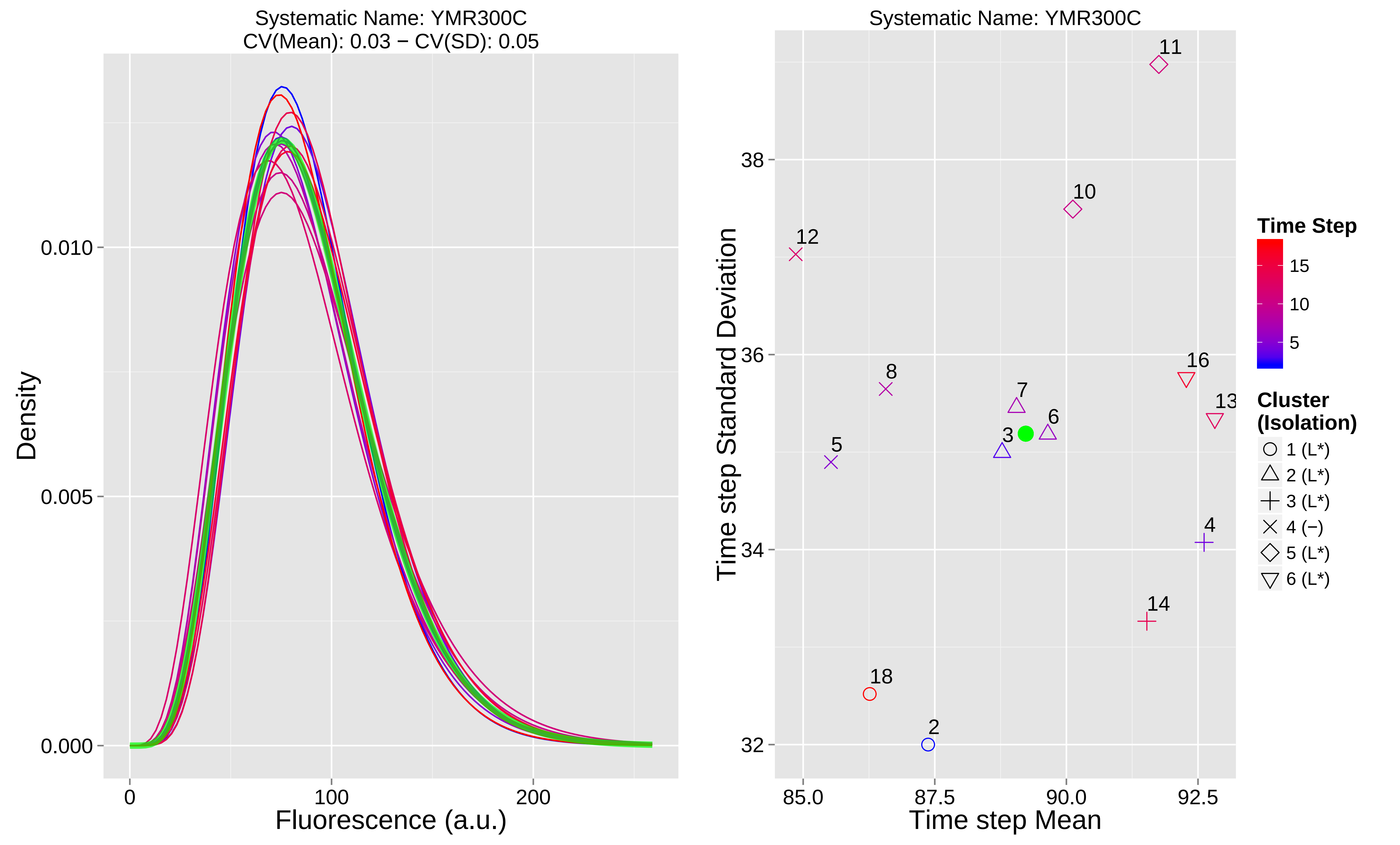

Systematic Name: YMR300C
CV(Mean): 0.03 − CV(SD): 0.05
Density
0.010
0.005
0.000
0
100
200
Fluorescence (a.u.)
Systematic Name: YMR300C
Time step Standard Deviation
38
36
34
32
85.0
87.5
90.0
92.5
Time step Mean
11
10
12
16
13
8
7
6
3
5
4
14
18
2
Time Step
15
10
5
Cluster
(Isolation)
1 (L*)
2 (L*)
3 (L*)
4 (−)
5 (L*)
6 (L*)

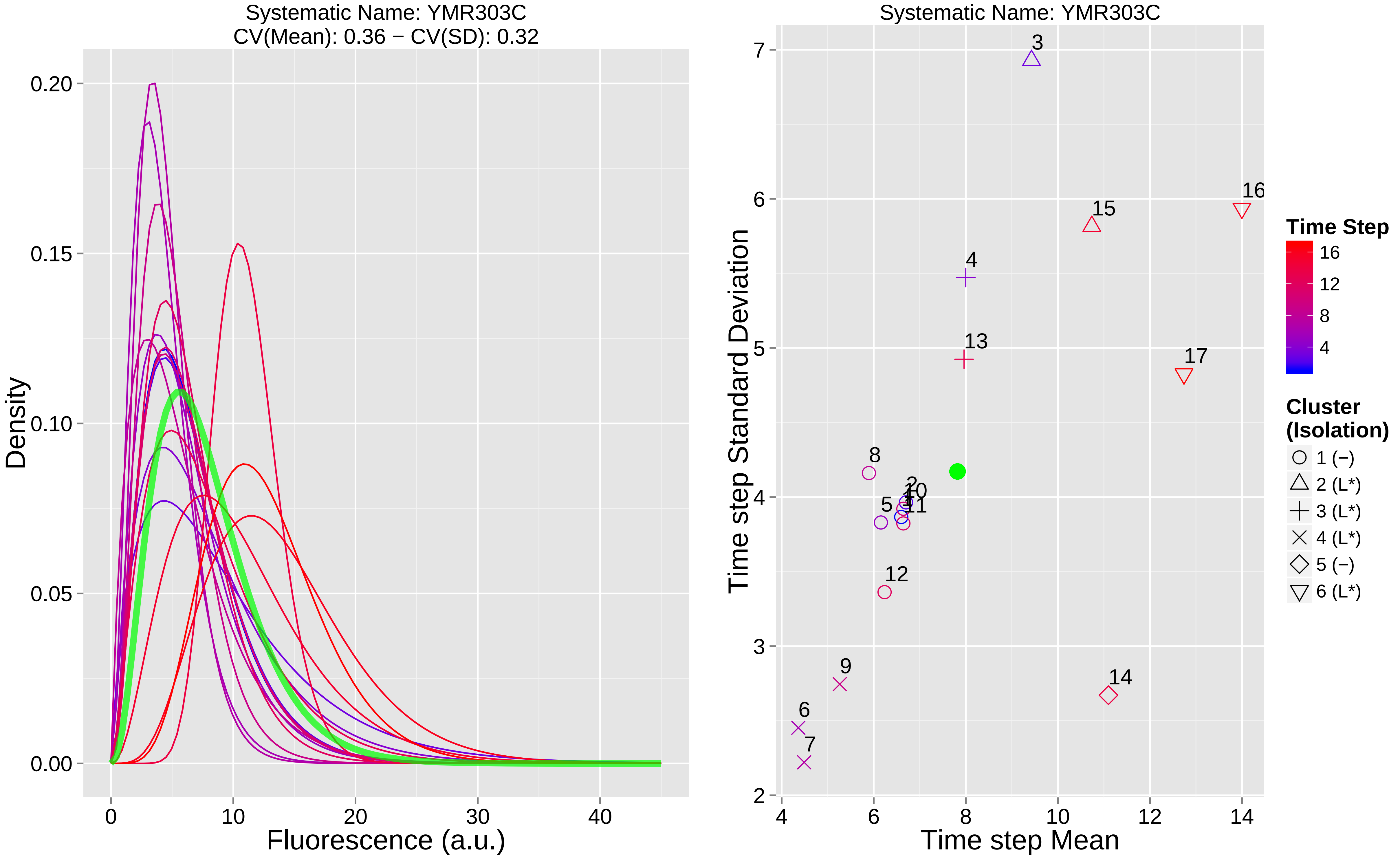

Systematic Name: YMR303C
CV(Mean): 0.36 − CV(SD): 0.32
Density
Fluorescence (a.u.)
0.00
0.05
0.10
0.15
0.20
0
10
20
30
40
Systematic Name: YMR303C
Time step Standard Deviation
Time step Mean
Time Step
16
12
8
4
Cluster
(Isolation)
1 (−)
2 (L*)
3 (L*)
4 (L*)
5 (−)
6 (L*)

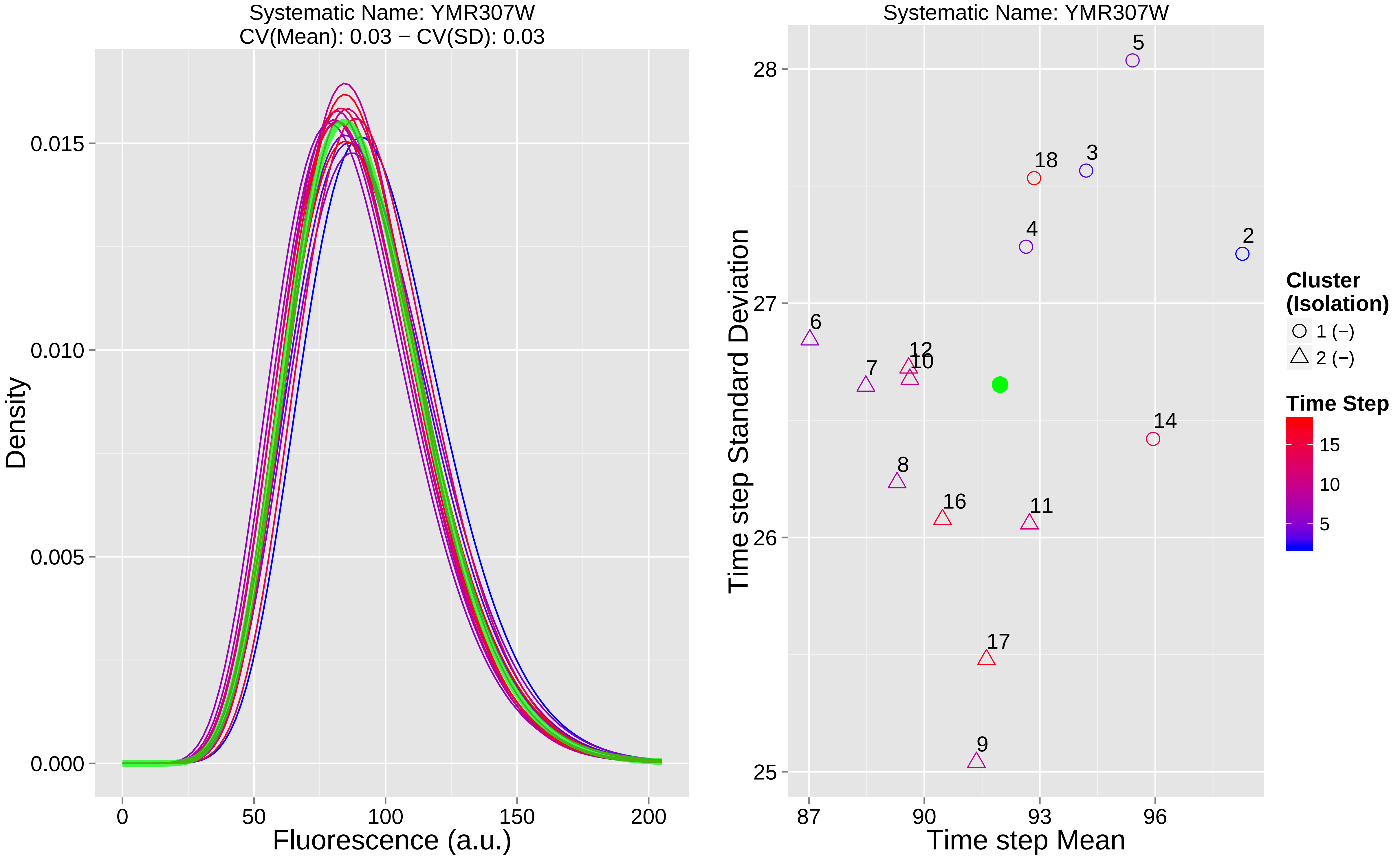

Systematic Name: YMR307W
CV(Mean): 0.03 − CV(SD): 0.03
Density
0.015
0.010
0.005
0.000
0
50
100
150
200
Fluorescence (a.u.)
Systematic Name: YMR307W
Time step Standard Deviation
28
27
26
25
87
90
93
96
Time step Mean
Cluster (Isolation)
1 (−)
2 (−)
Time Step
15
10
5

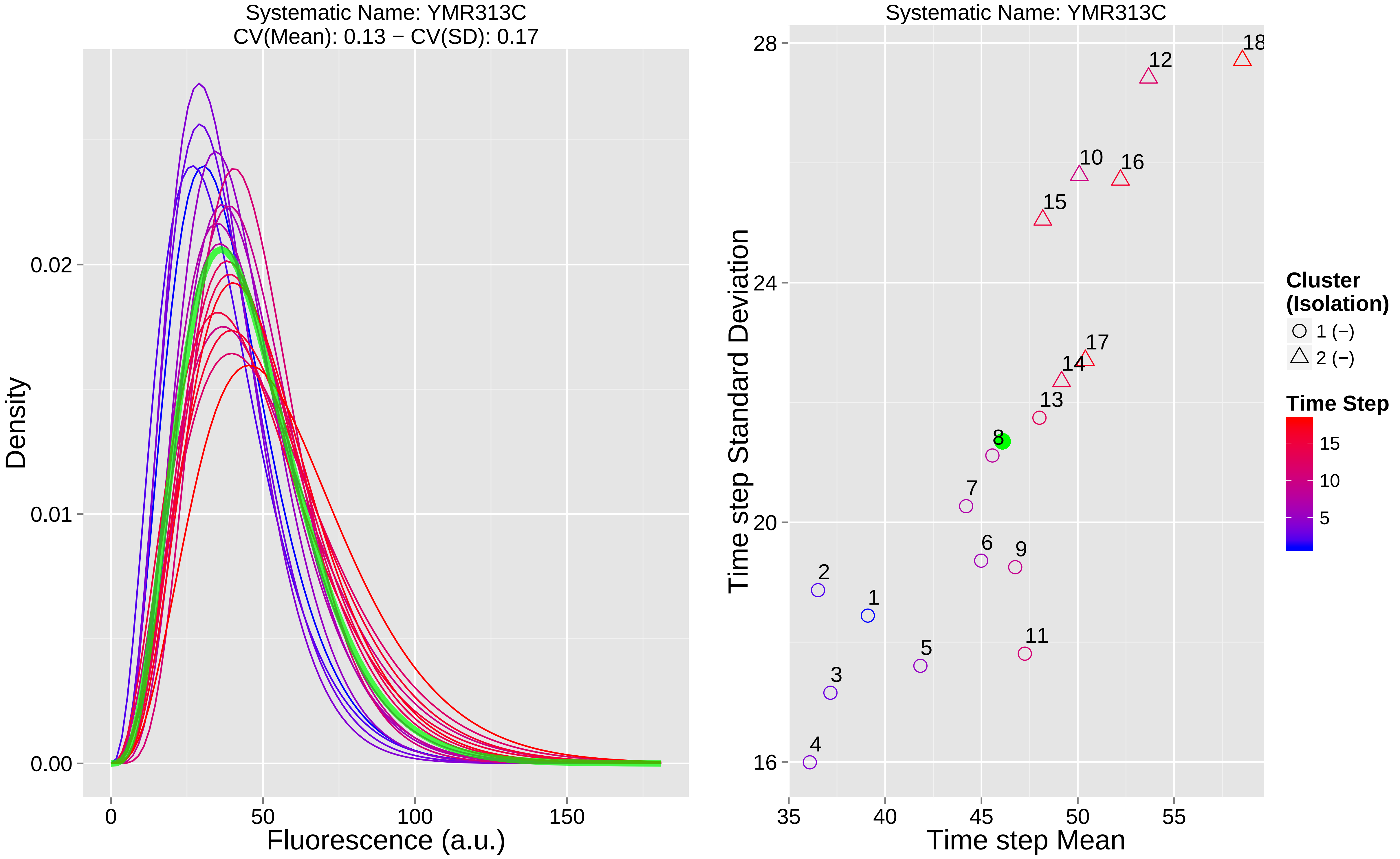
Systematic Name: YMR313C
CV(Mean): 0.13 − CV(SD): 0.17
Density
0.00
0.01
0.02
0
50
100
150
Fluorescence (a.u.)
Systematic Name: YMR313C
Time step Standard Deviation
16
20
24
28
35
40
45
50
55
Time step Mean
1
2
3
4
5
6
7
8
9
10
11
12
13
14
15
16
17
18
Cluster
(Isolation)
1 (−)
2 (−)
Time Step
5
10
15

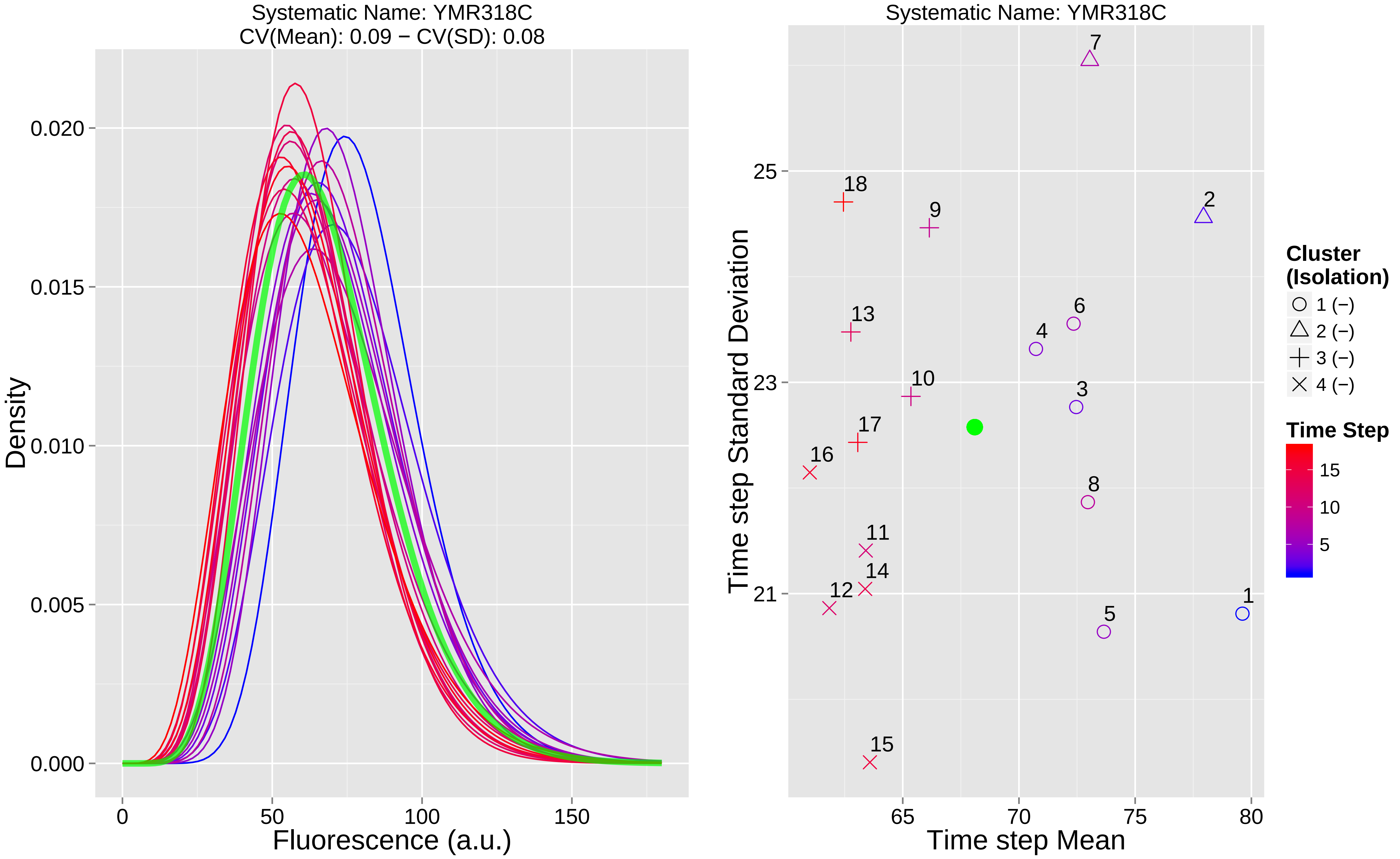
Systematic Name: YMR318C
CV(Mean): 0.09 − CV(SD): 0.08
Density
0.000
0.005
0.010
0.015
0.020
0
50
100
150
Fluorescence (a.u.)
Systematic Name: YMR318C
Time step Standard Deviation
21
23
25
65
70
75
80
Time step Mean
1
2
3
4
5
6
7
8
9
10
11
12
13
14
15
16
17
18
Cluster
(Isolation)
1 (−)
2 (−)
3 (−)
4 (−)
Time Step
15
10
5

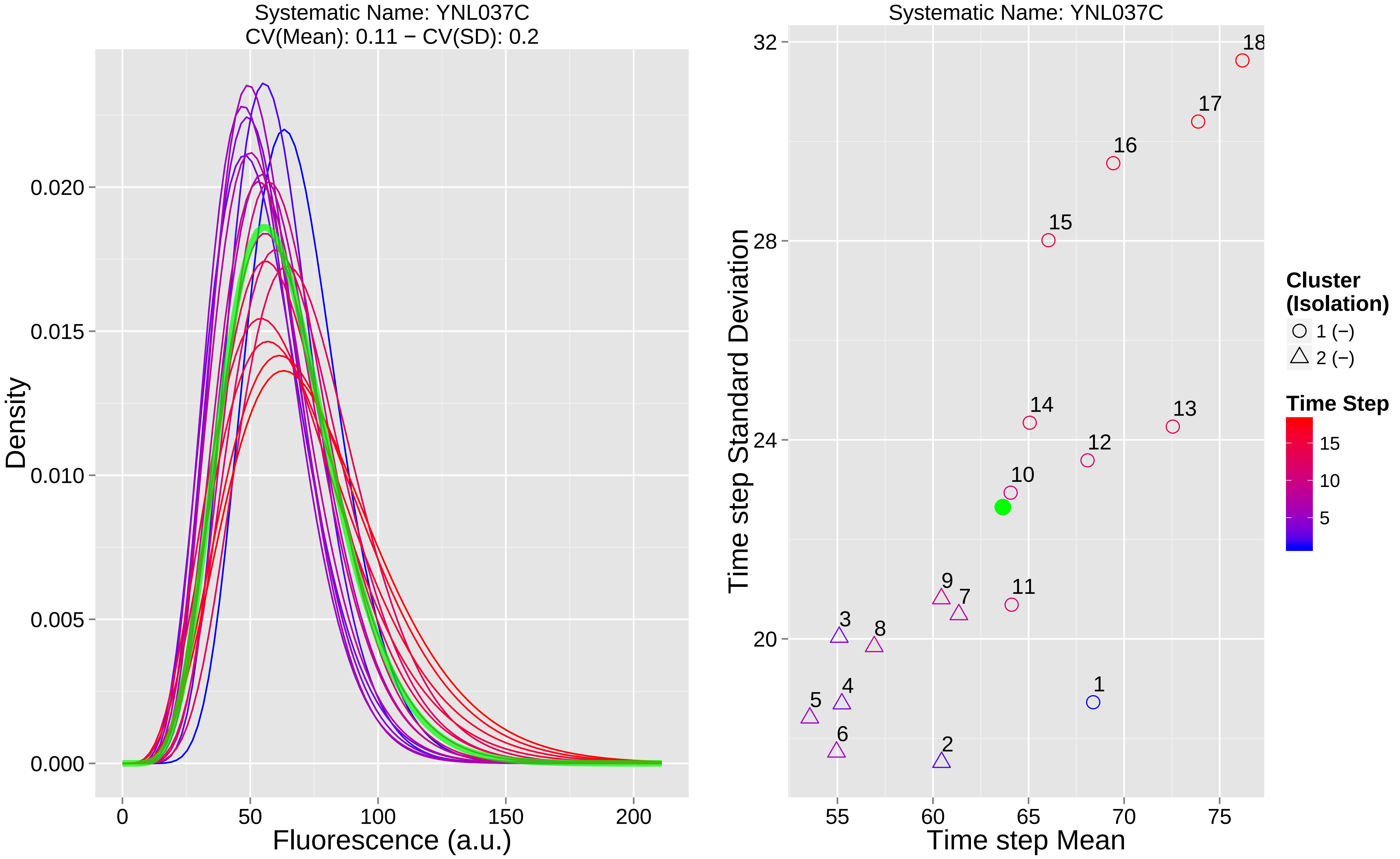
Systematic Name: YNL037C
CV(Mean): 0.11 − CV(SD): 0.2
Density
Fluorescence (a.u.)
0.000
0.005
0.010
0.015
0.020
0
50
100
150
200
Systematic Name: YNL037C
Time step Standard Deviation
Time step Mean
20
24
28
32
55
60
65
70
75
1
2
3
4
5
6
7
8
9
10
11
12
13
14
15
16
17
18
Cluster
(Isolation)
1 (−)
2 (−)
Time Step
5
10
15

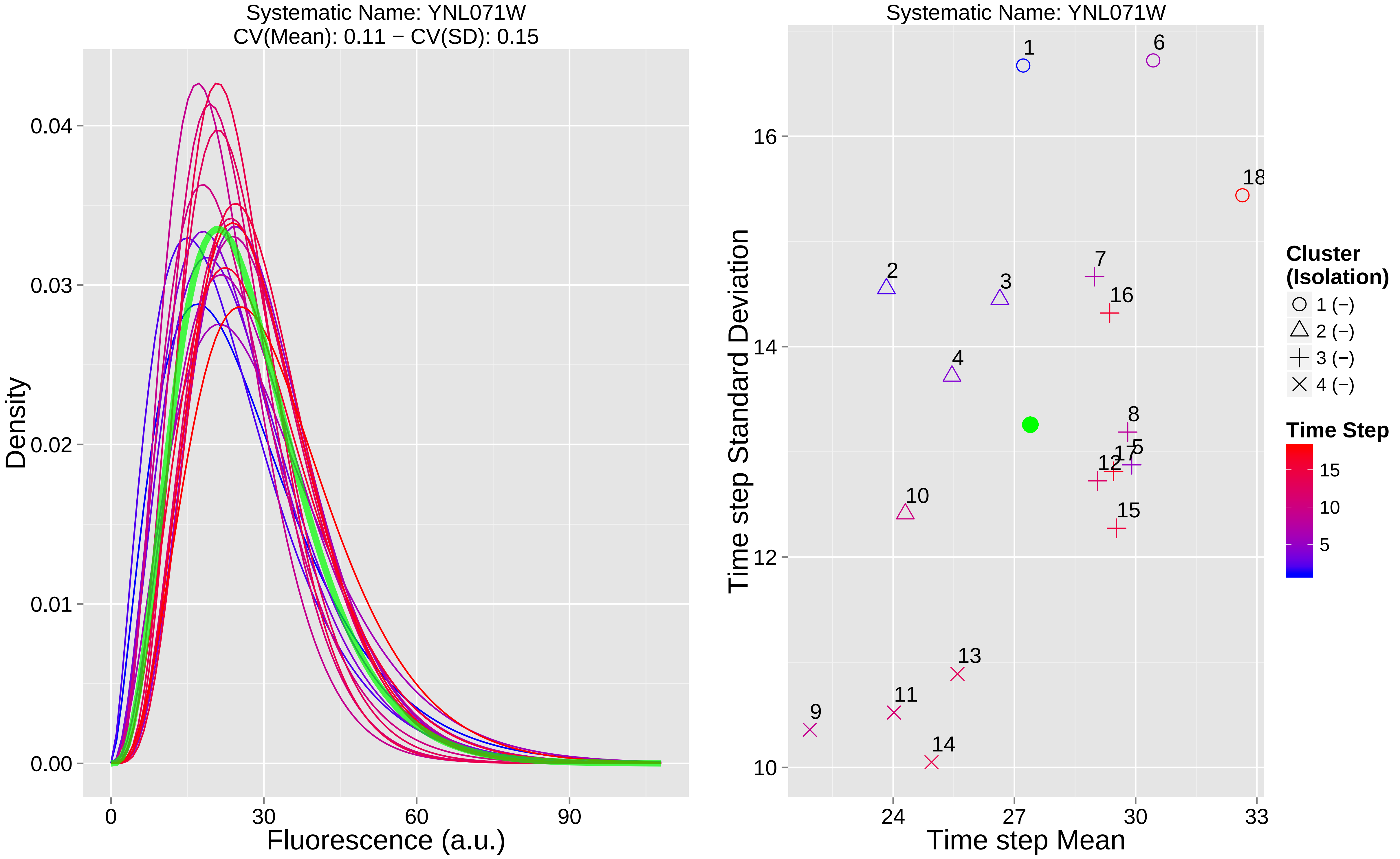

Systematic Name: YNL071W
CV(Mean): 0.11 − CV(SD): 0.15
Density
Fluorescence (a.u.)
0.00
0.01
0.02
0.03
0.04
0
30
60
90
Systematic Name: YNL071W
Time step Standard Deviation
Time step Mean
10
12
14
16
24
27
30
33
1
2
3
4
5
6
7
8
9
10
11
12
13
14
15
16
17
18
Cluster
(Isolation)
1 (−)
2 (−)
3 (−)
4 (−)
Time Step
15
10
5

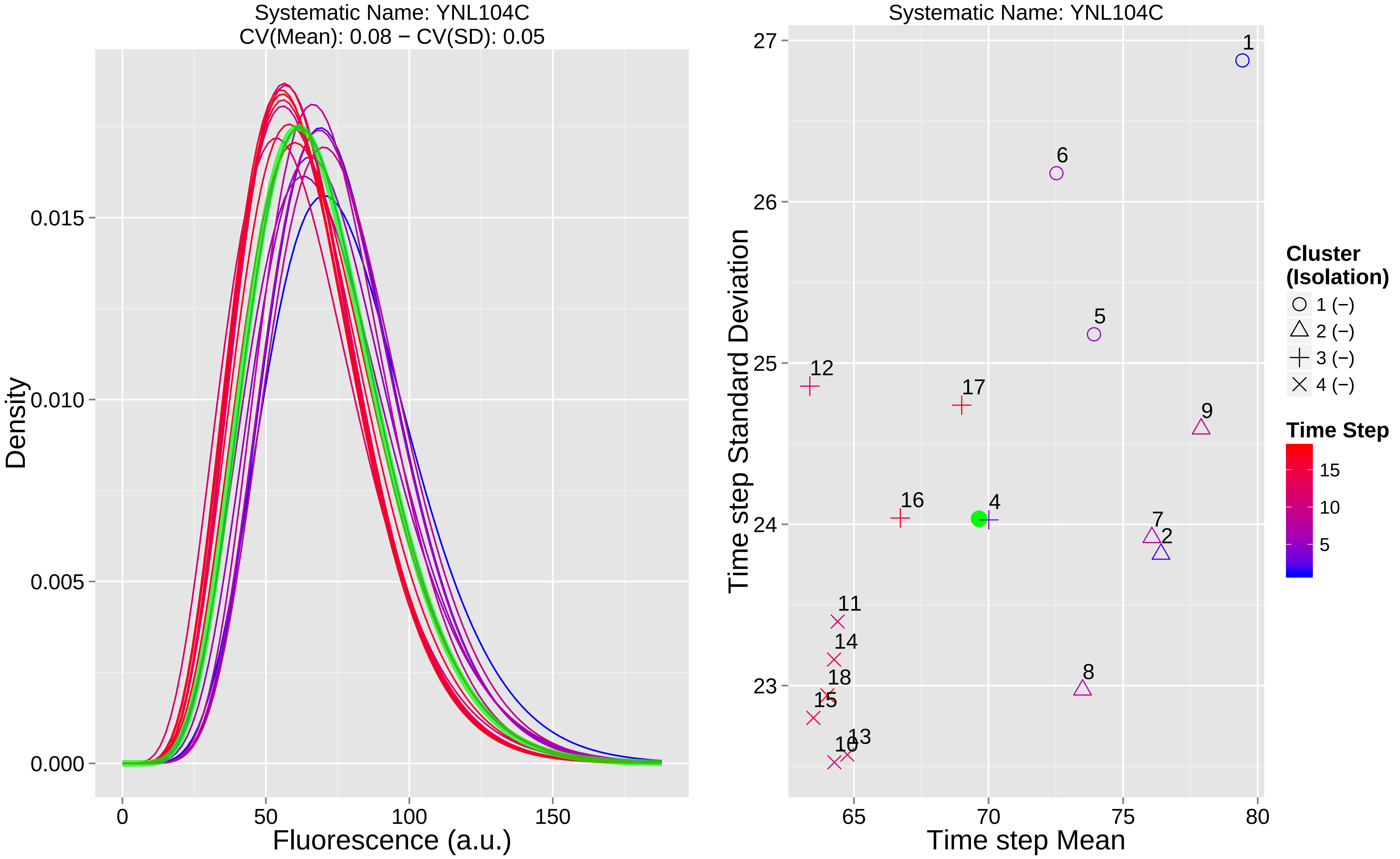
Systematic Name: YNL104C
CV(Mean): 0.08 − CV(SD): 0.05
Density
0.000
0.005
0.010
0.015
0
50
100
150
Fluorescence (a.u.)
Systematic Name: YNL104C
Time step Standard Deviation
23
24
25
26
27
65
70
75
80
Time step Mean
1
2
4
5
6
7
8
9
10
11
12
13
14
15
16
17
18
Cluster (Isolation)
1 (−)
2 (−)
3 (−)
4 (−)
Time Step
5
10
15

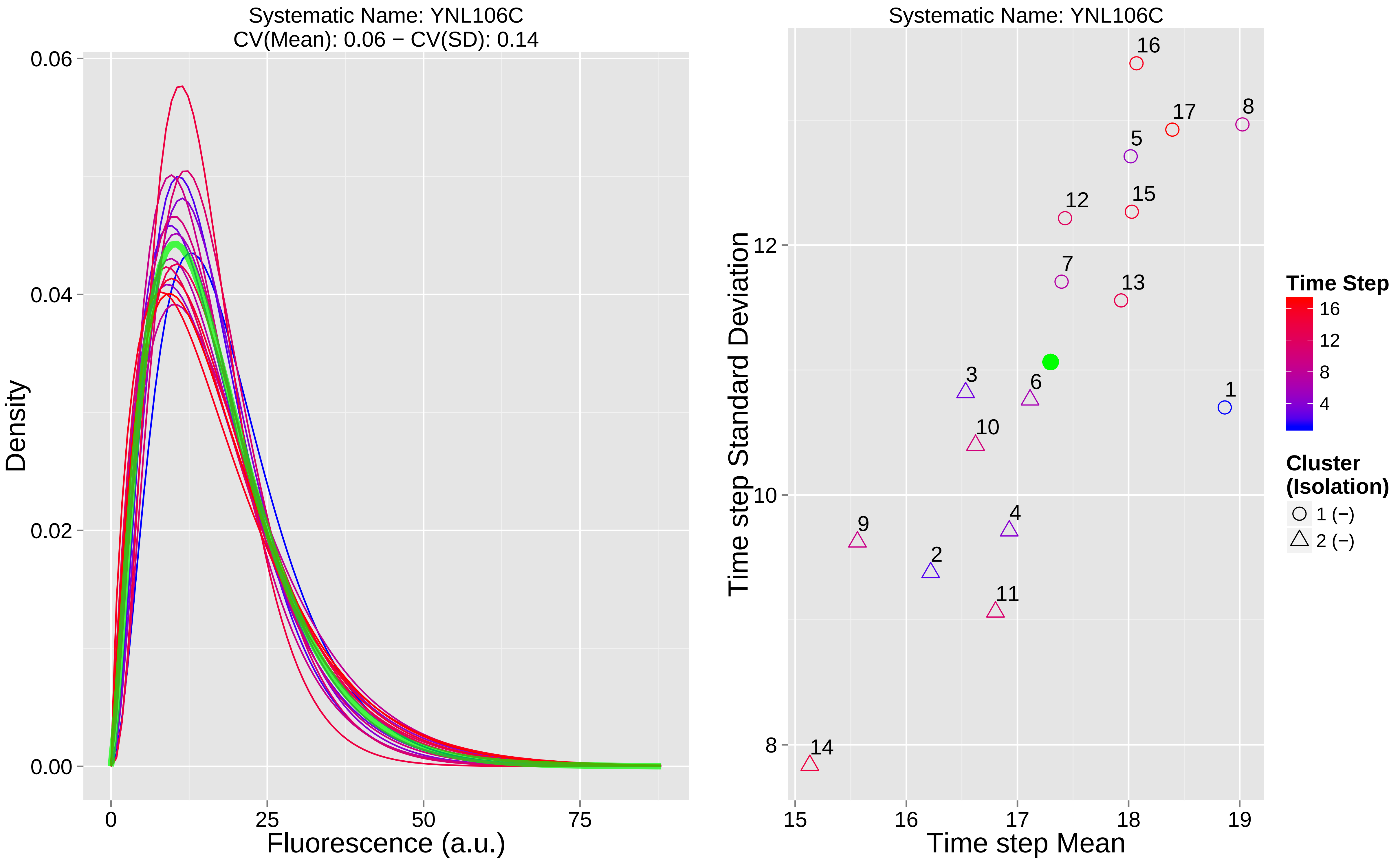

Systematic Name: YNL106C
CV(Mean): 0.06 − CV(SD): 0.14
Density
0.06
0.04
0.02
0.00
0
25
50
75
Fluorescence (a.u.)
Systematic Name: YNL106C
Time step Standard Deviation
12
10
8
15
16
17
18
19
Time step Mean
16
17
8
5
12
15
7
13
3
6
1
10
9
4
2
11
14
Time Step
16
12
8
4
Cluster
(Isolation)
1 (−)
2 (−)

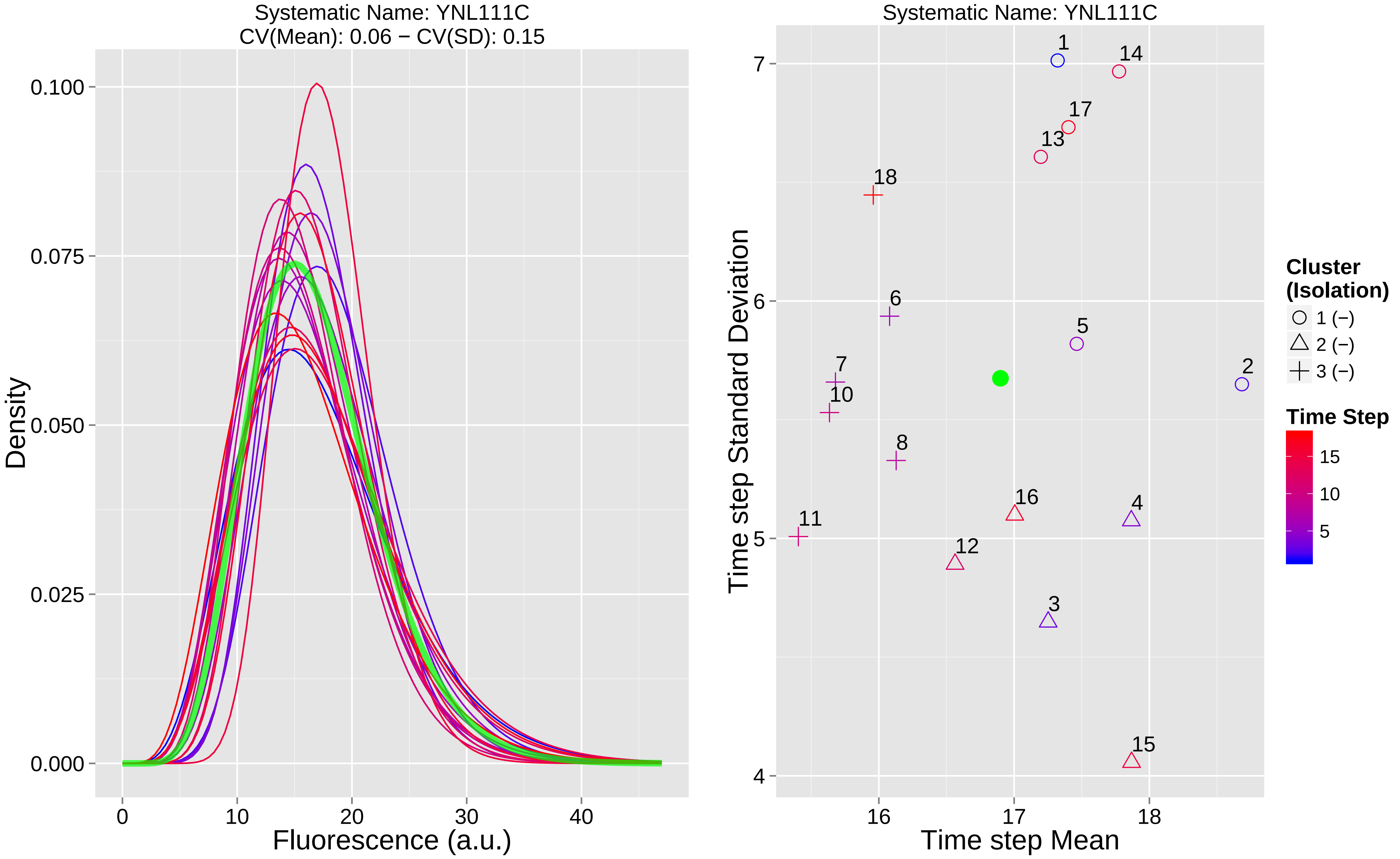
Systematic Name: YNL111C
CV(Mean): 0.06 − CV(SD): 0.15
Density
0.100
0.075
0.050
0.025
0.000
0
10
20
30
40
Fluorescence (a.u.)
Systematic Name: YNL111C
Time step Standard Deviation
7
6
5
4
16
17
18
Time step Mean
1
2
3
4
5
6
7
8
10
11
12
13
14
15
16
17
18
Cluster
(Isolation)
1 (−)
2 (−)
3 (−)
Time Step
15
10
5

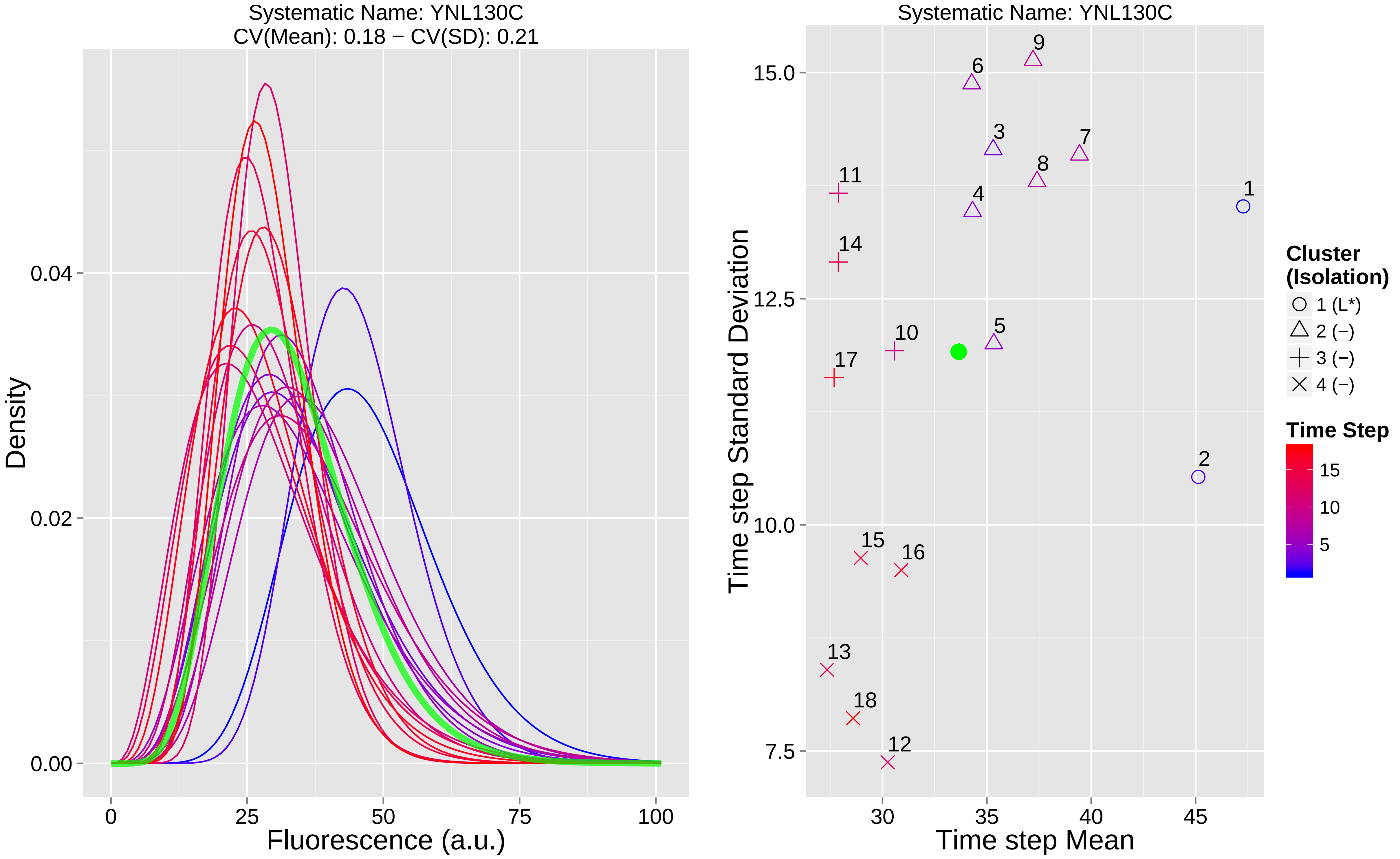
Systematic Name: YNL130C
CV(Mean): 0.18 − CV(SD): 0.21
Density
0.04
0.02
0.00
0
25
50
75
100
Fluorescence (a.u.)
Systematic Name: YNL130C
Time step Standard Deviation
15.0
12.5
10.0
7.5
30
35
40
45
Time step Mean
Cluster (Isolation)
1 (L*)
2 (−)
3 (−)
4 (−)
Time Step
15
10
5

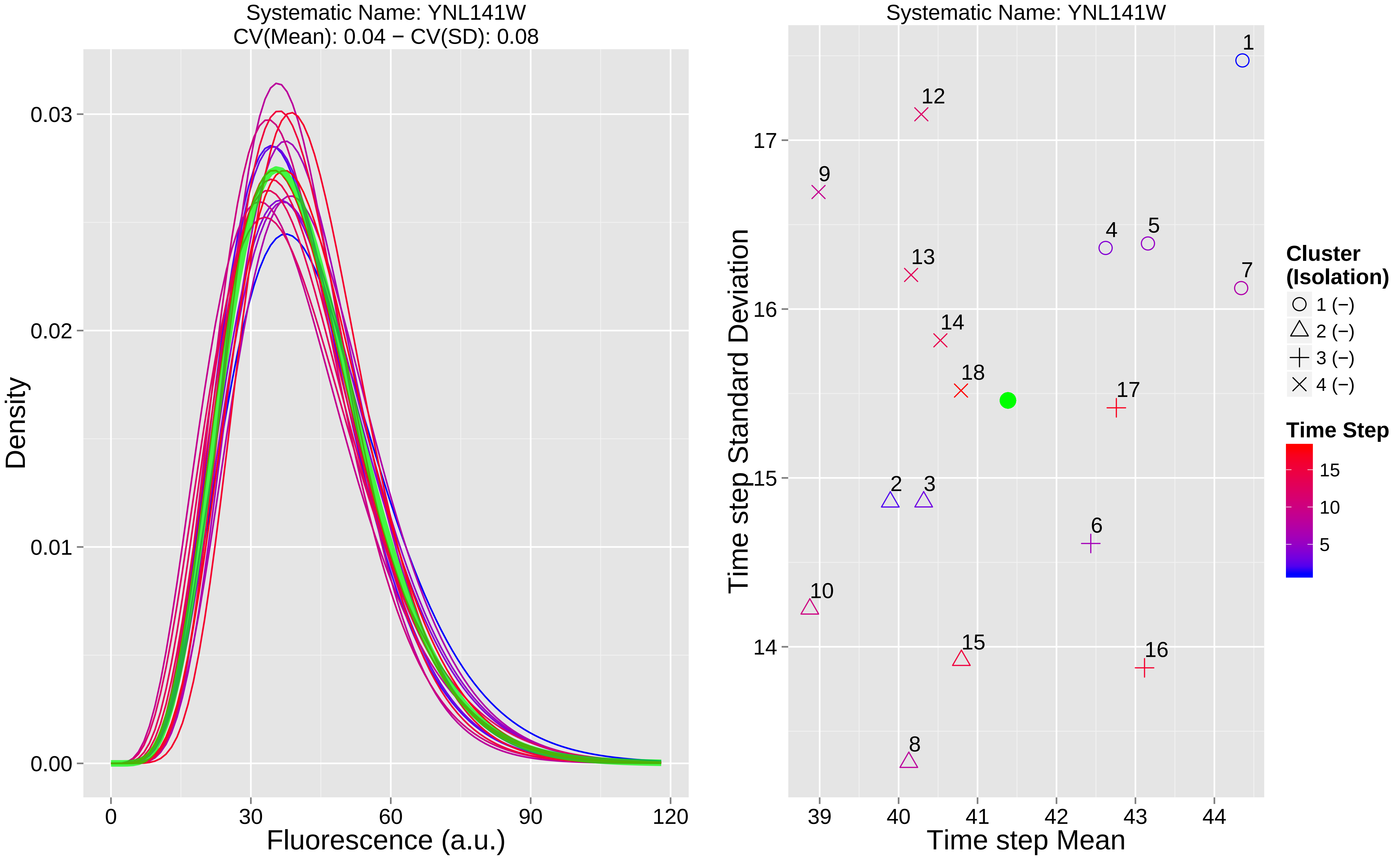
Systematic Name: YNL141W
CV(Mean): 0.04 − CV(SD): 0.08
Density
Fluorescence (a.u.)
0.00
0.01
0.02
0.03
0
30
60
90
120
Systematic Name: YNL141W
Time step Standard Deviation
Time step Mean
14
15
16
17
39
40
41
42
43
44
1
2
3
4
5
6
7
8
9
10
12
13
14
15
16
17
18
Cluster (Isolation)
1 (−)
2 (−)
3 (−)
4 (−)
Time Step
15
10
5

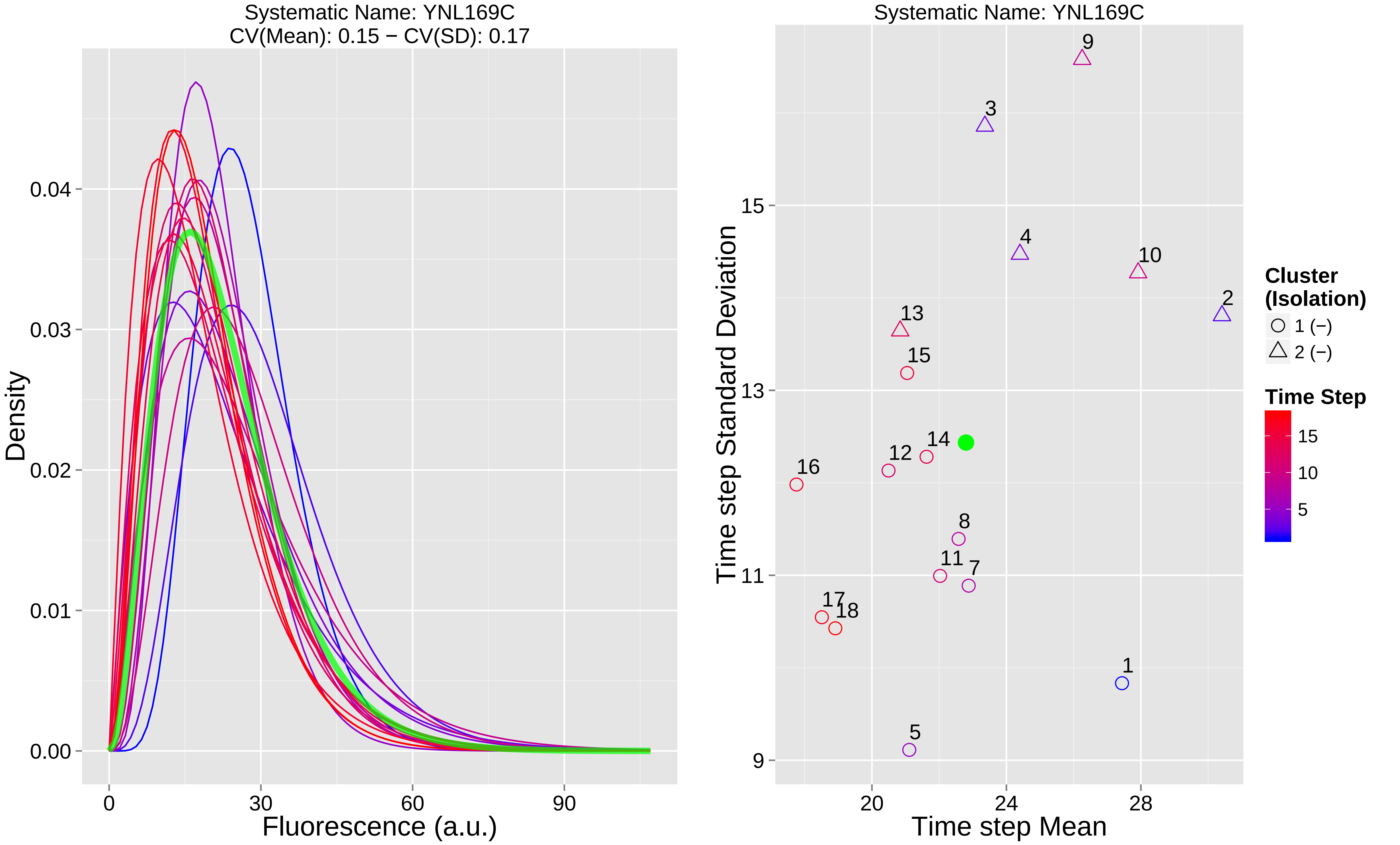

Systematic Name: YNL169C
CV(Mean): 0.15 − CV(SD): 0.17
Density
0.00
0.01
0.02
0.03
0.04
0
30
60
90
Fluorescence (a.u.)
Systematic Name: YNL169C
Time step Standard Deviation
Time step Mean
9
11
13
15
20
24
28
1
2
3
4
5
7
8
9
10
11
12
13
14
15
16
17
18
Cluster (Isolation)
1 (−)
2 (−)
Time Step
5
10
15

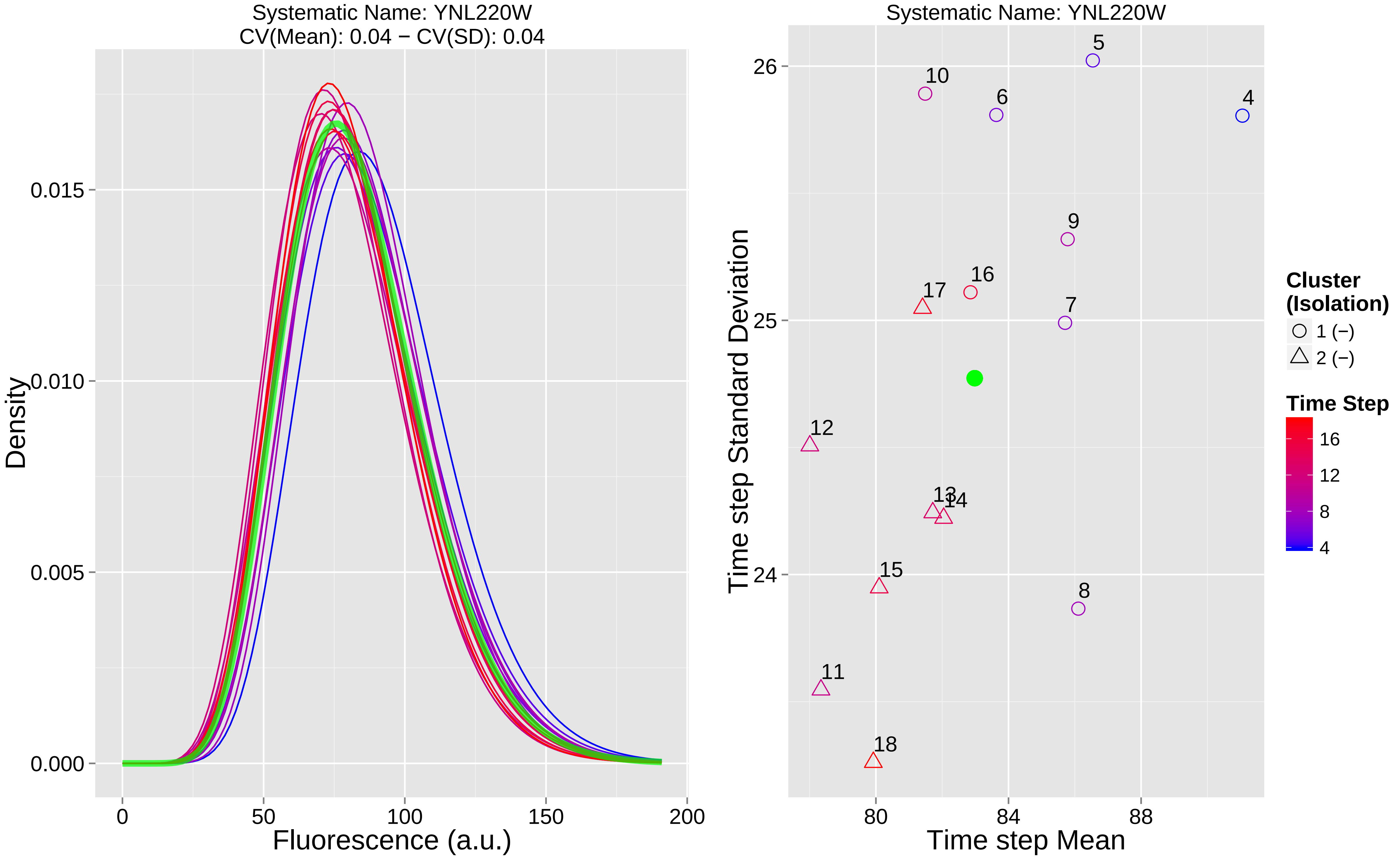

Systematic Name: YNL220W
CV(Mean): 0.04 − CV(SD): 0.04
Density
0.015
0.010
0.005
0.000
0
50
100
150
200
Fluorescence (a.u.)
Systematic Name: YNL220W
Time step Standard Deviation
26
25
24
80
84
88
Time step Mean
5
10
6
4
9
16
17
7
12
13
14
15
8
11
18
Cluster (Isolation)
1 (−)
2 (−)
Time Step
16
12
8
4

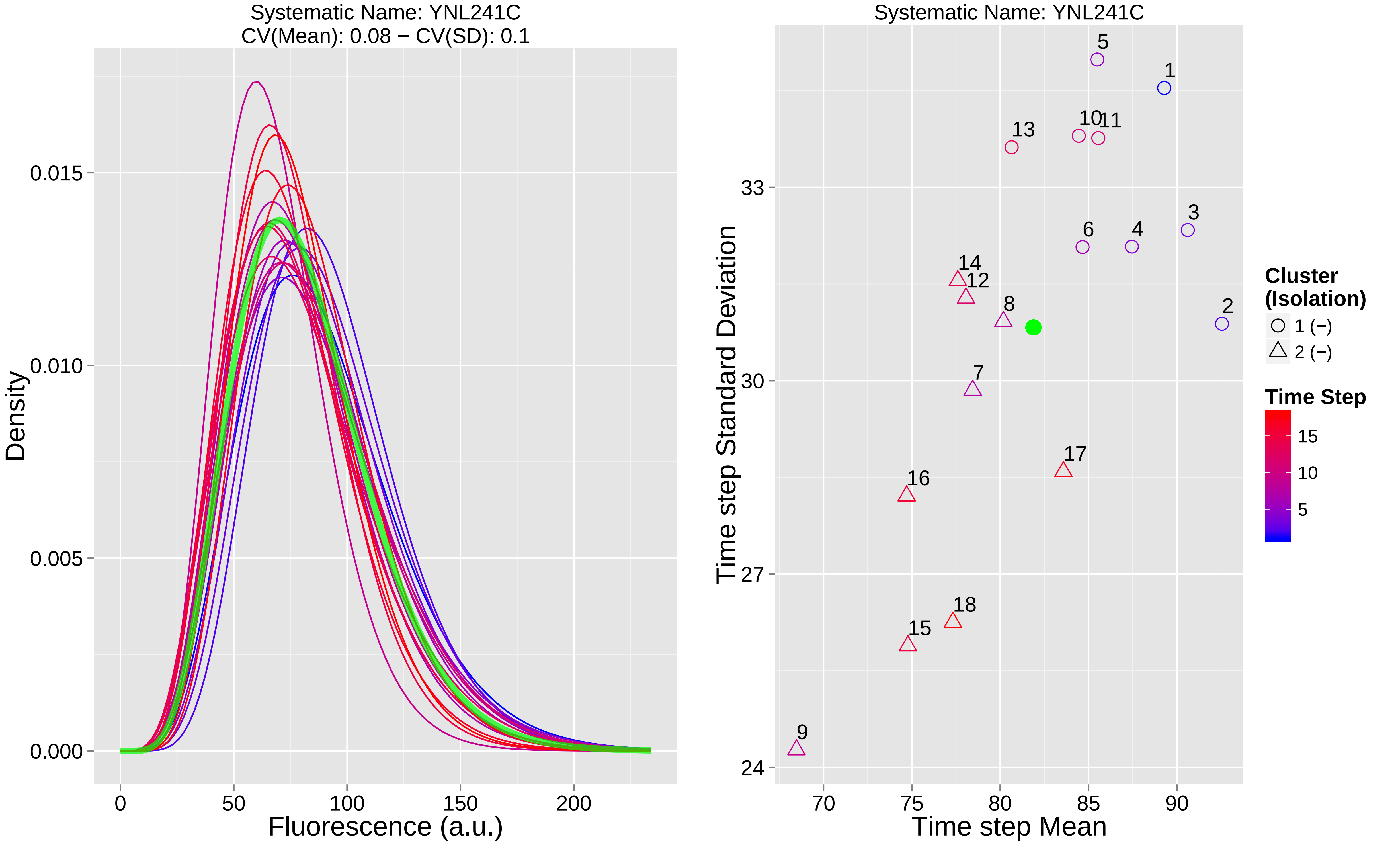

Systematic Name: YNL241C
CV(Mean): 0.08 − CV(SD): 0.1
Density
0.015
0.010
0.005
0.000
0
50
100
150
200
Fluorescence (a.u.)
Systematic Name: YNL241C
Time step Standard Deviation
33
30
27
24
70
75
80
85
90
Time step Mean
1
2
3
4
5
6
7
8
9
10
11
12
13
14
15
16
17
18
Cluster
(Isolation)
1 (−)
2 (−)
Time Step
15
10
5

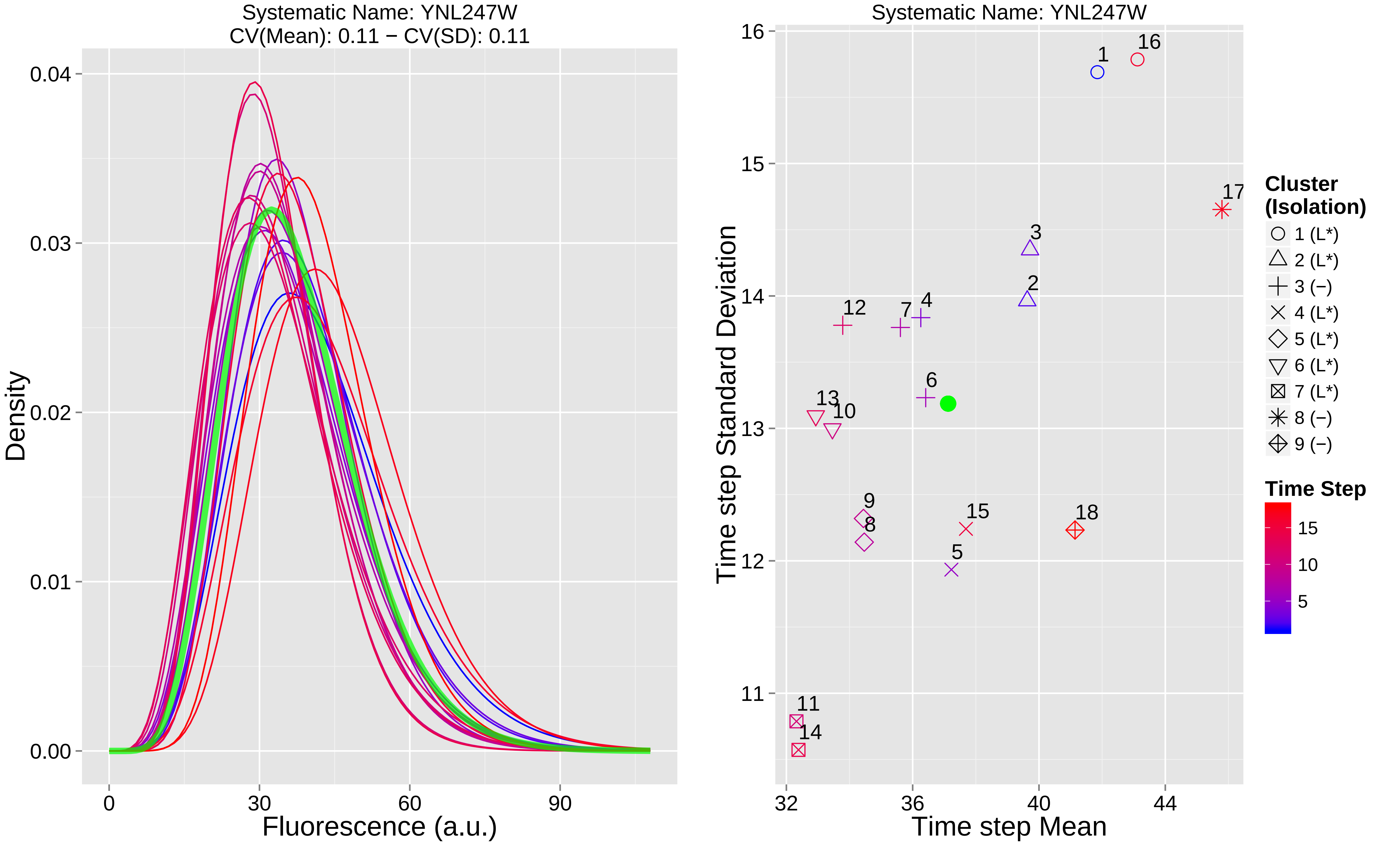

Systematic Name: YNL247W
CV(Mean): 0.11 − CV(SD): 0.11
Density
0.04
0.03
0.02
0.01
0.00
0
30
60
90
Fluorescence (a.u.)
Systematic Name: YNL247W
Time step Standard Deviation
16
15
14
13
12
11
32
36
40
44
Time step Mean
1
2
3
4
5
6
7
8
9
10
11
12
13
14
15
16
17
18
Cluster (Isolation)
1 (L*)
2 (L*)
3 (−)
4 (L*)
5 (L*)
6 (L*)
7 (L*)
8 (−)
9 (−)
Time Step
15
10
5

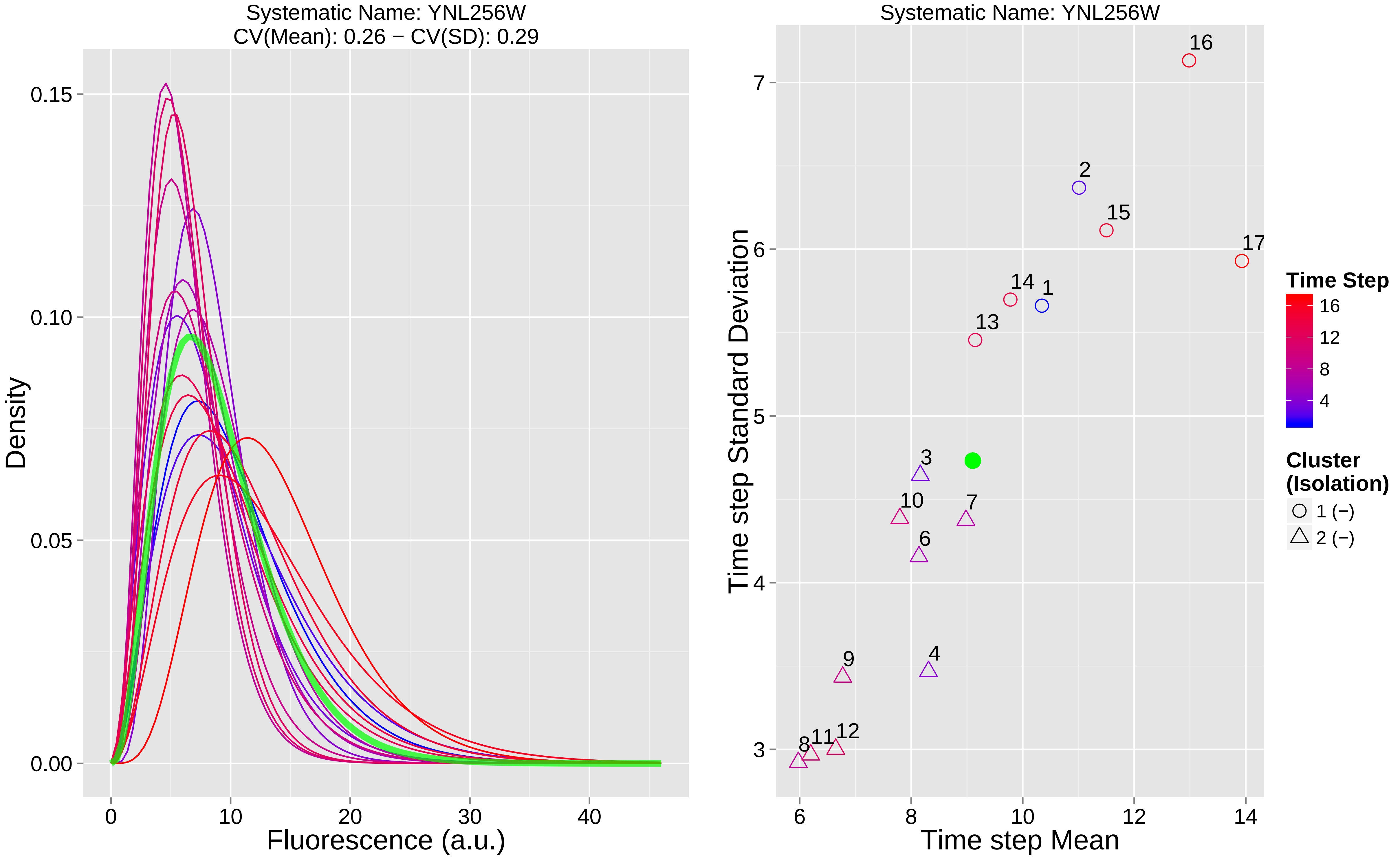

Systematic Name: YNL256W
CV(Mean): 0.26 − CV(SD): 0.29
Density
Fluorescence (a.u.)
0.00
0.05
0.10
0.15
0
10
20
30
40
Systematic Name: YNL256W
Time step Standard Deviation
Time step Mean
3
4
5
6
7
6
8
10
12
14
1
2
3
4
6
7
8
9
10
11
12
13
14
15
16
17
Time Step
16
12
8
4
Cluster
(Isolation)
1 (−)
2 (−)

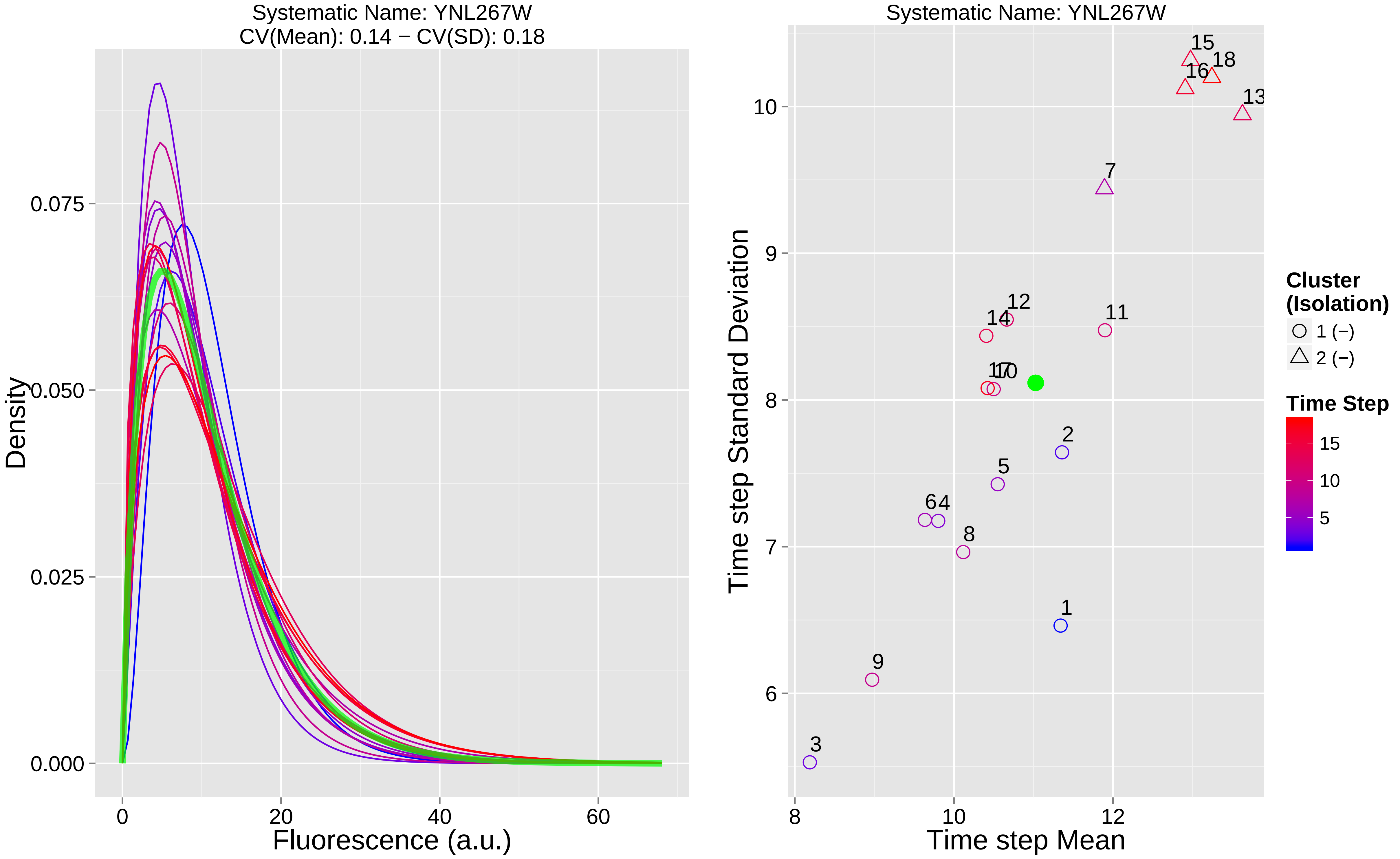

Systematic Name: YNL267W
CV(Mean): 0.14 − CV(SD): 0.18
Density
Fluorescence (a.u.)
0.000
0.025
0.050
0.075
0
20
40
60
Systematic Name: YNL267W
Time step Standard Deviation
Time step Mean
6
7
8
9
10
8
10
12
Cluster
(Isolation)
1 (−)
2 (−)
Time Step
15
10
5

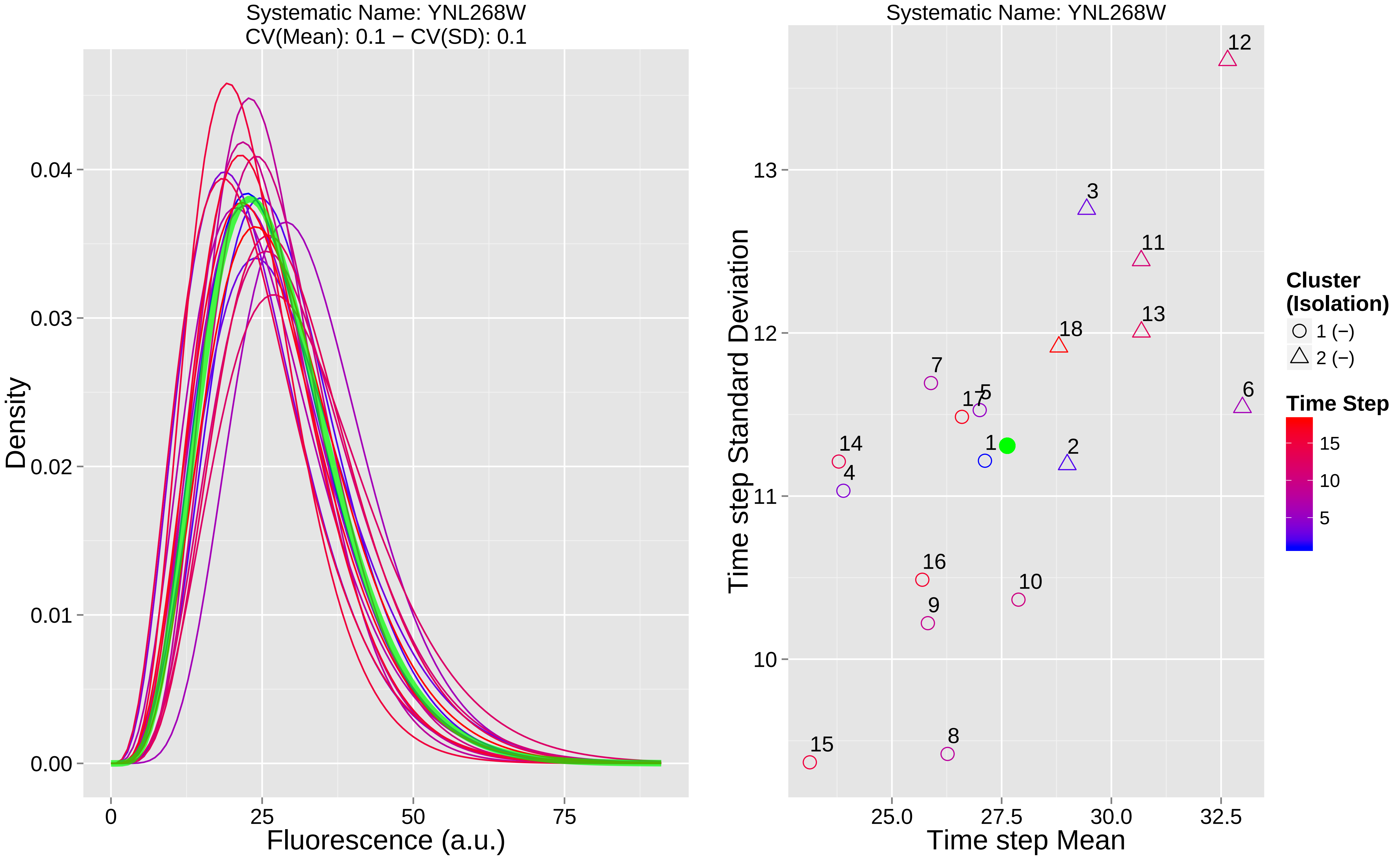
Systematic Name: YNL268W
CV(Mean): 0.1 − CV(SD): 0.1
Density
0.00
0.01
0.02
0.03
0.04
0
25
50
75
Fluorescence (a.u.)
Systematic Name: YNL268W
Time step Standard Deviation
10
11
12
13
25.0
27.5
30.0
32.5
Time step Mean
1
2
3
4
5
6
7
8
9
10
11
12
13
14
15
16
17
18
Cluster
(Isolation)
1 (−)
2 (−)
Time Step
15
10
5

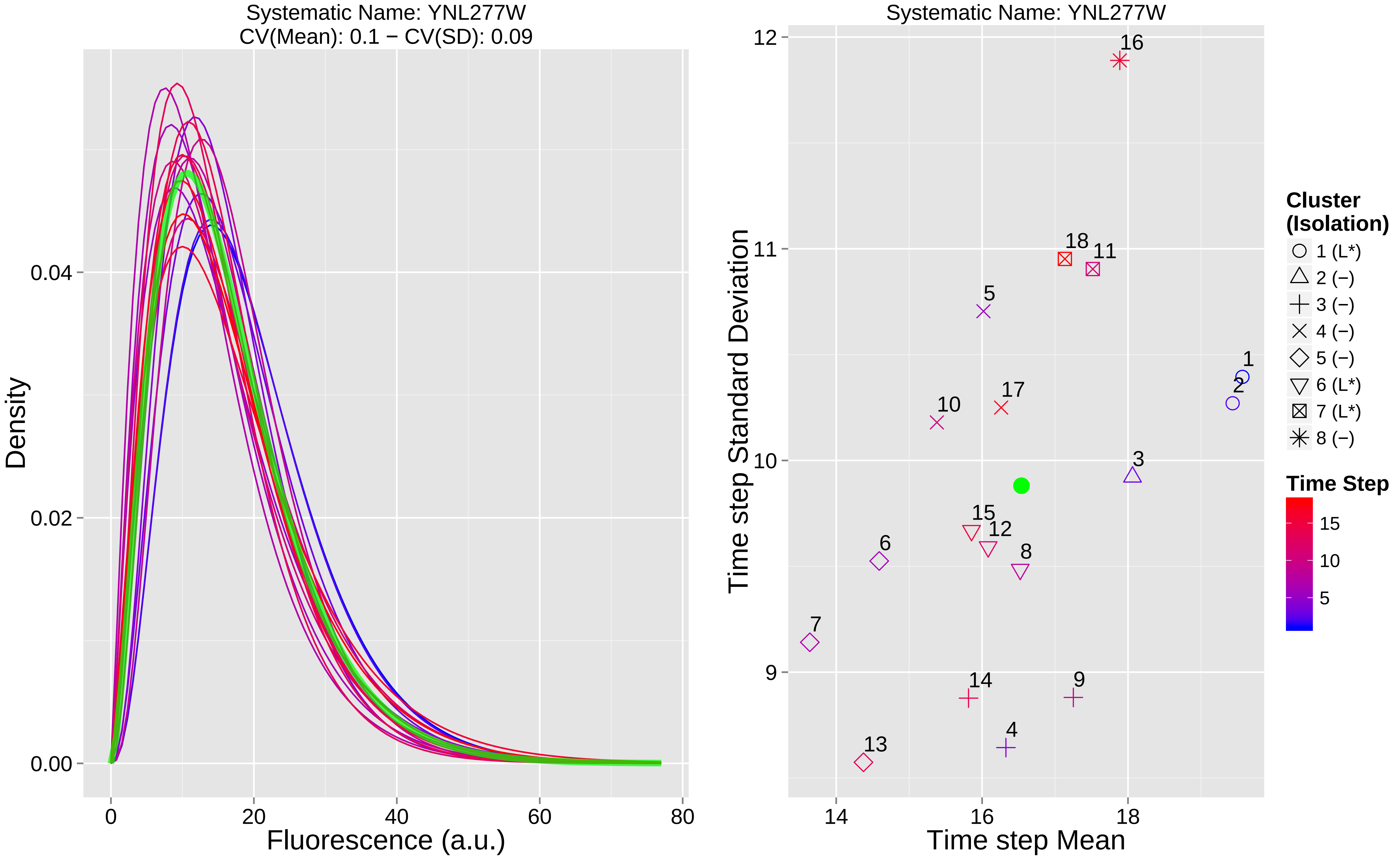

Systematic Name: YNL277W
CV(Mean): 0.1 − CV(SD): 0.09
Density
0.04
0.02
0.00
0
20
40
60
80
Fluorescence (a.u.)
Systematic Name: YNL277W
Time step Standard Deviation
12
11
10
9
14
16
18
Time step Mean
1
2
3
4
5
6
7
8
9
10
11
12
13
14
15
16
17
18
Cluster
(Isolation)
1 (L*)
2 (−)
3 (−)
4 (−)
5 (−)
6 (L*)
7 (L*)
8 (−)
Time Step
15
10
5

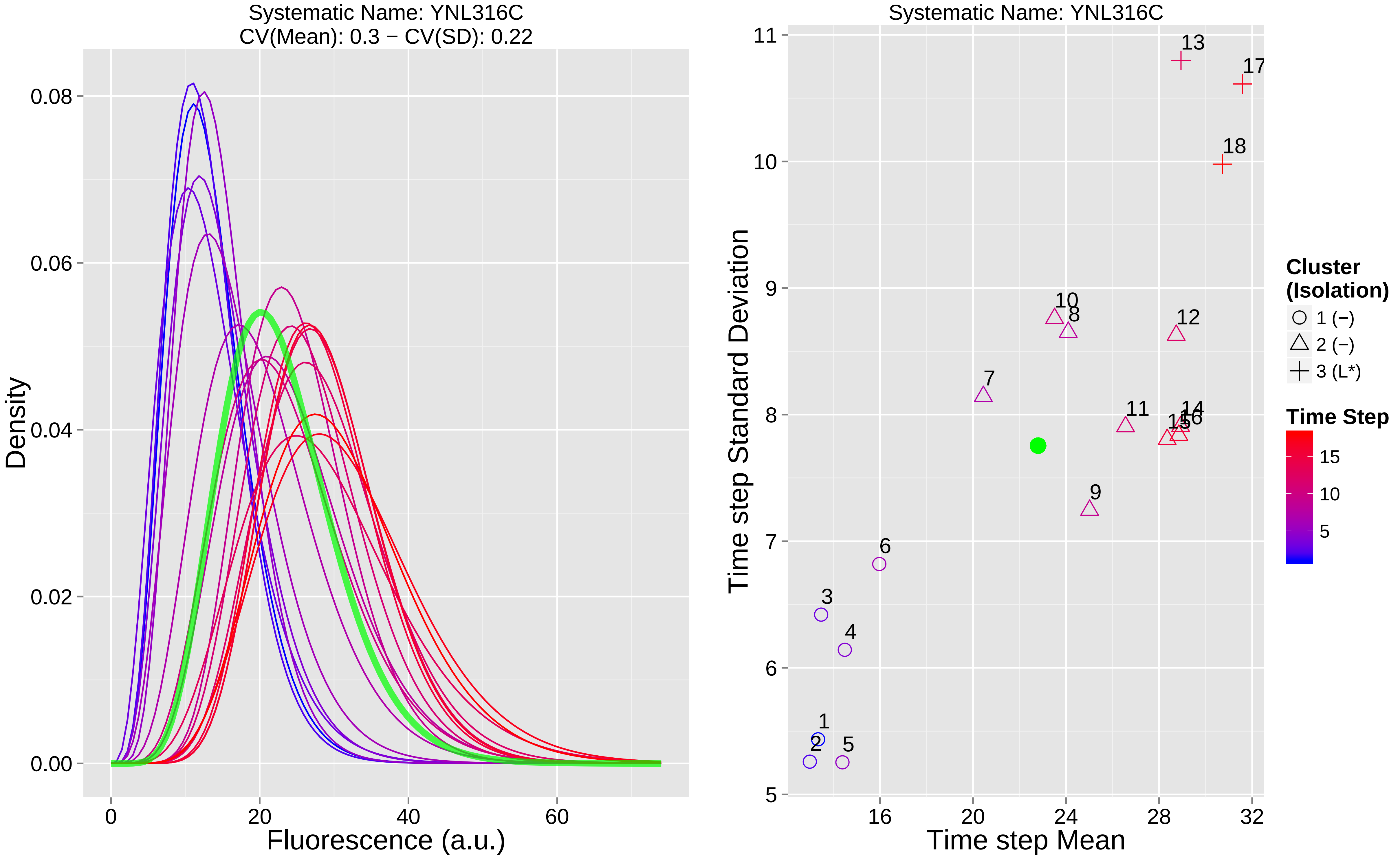

Systematic Name: YNL316C
CV(Mean): 0.3 − CV(SD): 0.22
Density
Fluorescence (a.u.)
0.00
0.02
0.04
0.06
0.08
0
20
40
60
Systematic Name: YNL316C
Time step Standard Deviation
Time step Mean
Cluster (Isolation)
1 (−)
2 (−)
3 (L*)
Time Step
15
10
5

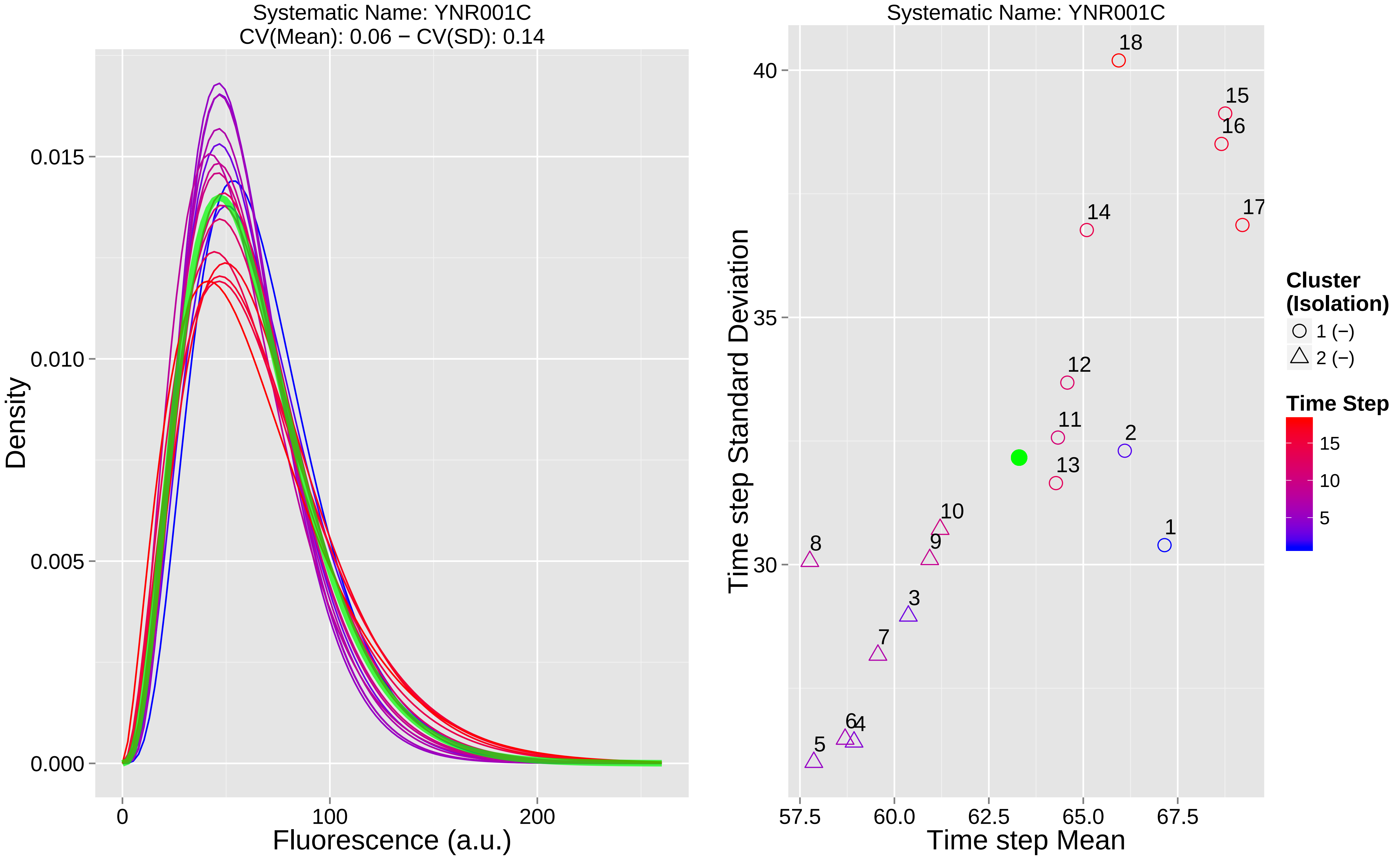
Systematic Name: YNR001C
CV(Mean): 0.06 − CV(SD): 0.14
Density
0.000
0.005
0.010
0.015
0
100
200
Fluorescence (a.u.)
Systematic Name: YNR001C
Time step Standard Deviation
30
35
40
57.5
60.0
62.5
65.0
67.5
Time step Mean
1
2
3
4
5
6
7
8
9
10
11
12
13
14
15
16
17
18
Cluster (Isolation)
1 (−)
2 (−)
Time Step
5
10
15

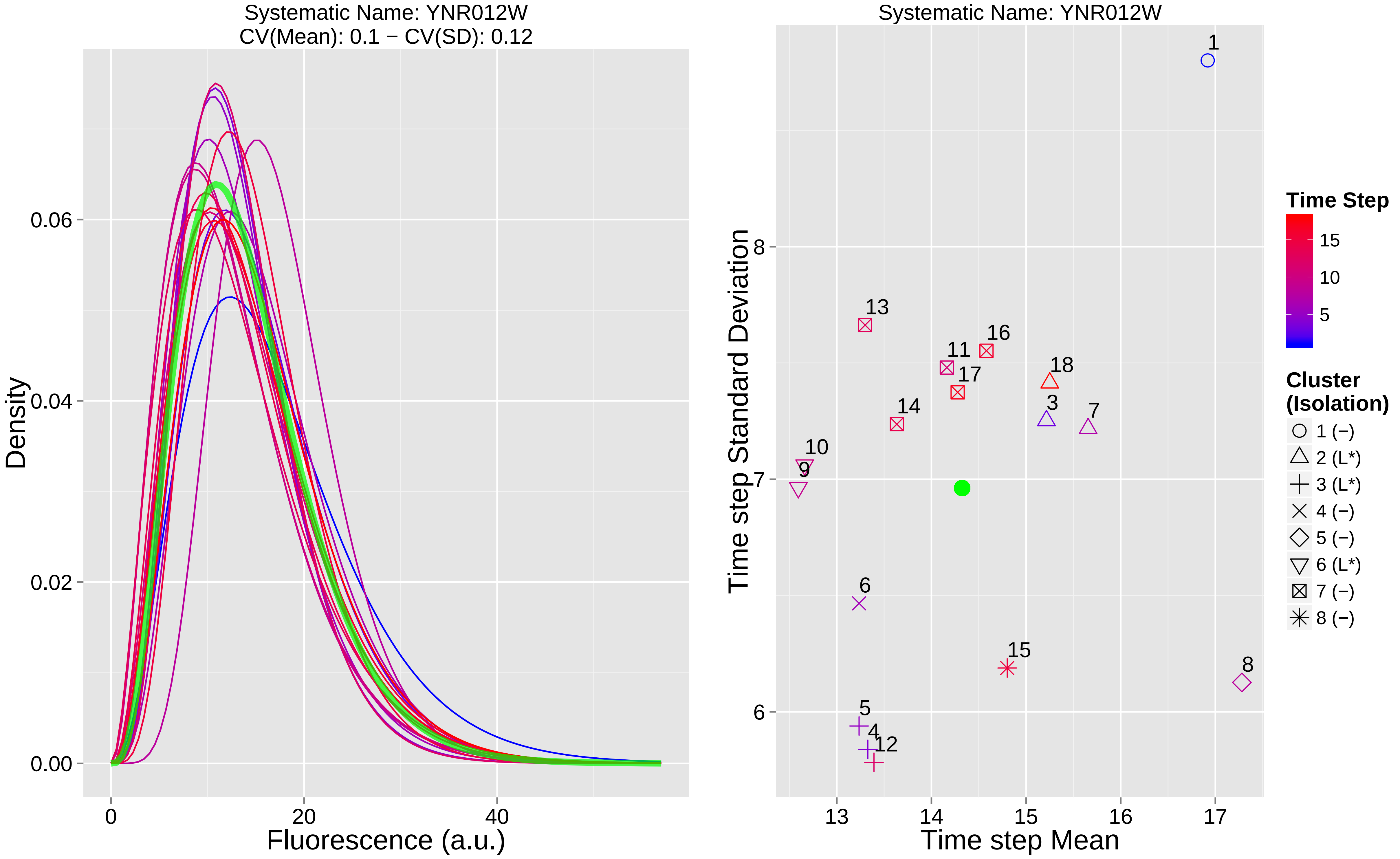

Systematic Name: YNR012W
CV(Mean): 0.1 − CV(SD): 0.12
Density
0.00
0.02
0.04
0.06
0
20
40
Fluorescence (a.u.)
Systematic Name: YNR012W
Time step Standard Deviation
6
7
8
13
14
15
16
17
Time step Mean
1
13
16
11
17
18
14
3
7
10
9
6
15
8
5
4
12
Time Step
15
10
5
Cluster (Isolation)
1 (−)
2 (L*)
3 (L*)
4 (−)
5 (−)
6 (L*)
7 (−)
8 (−)

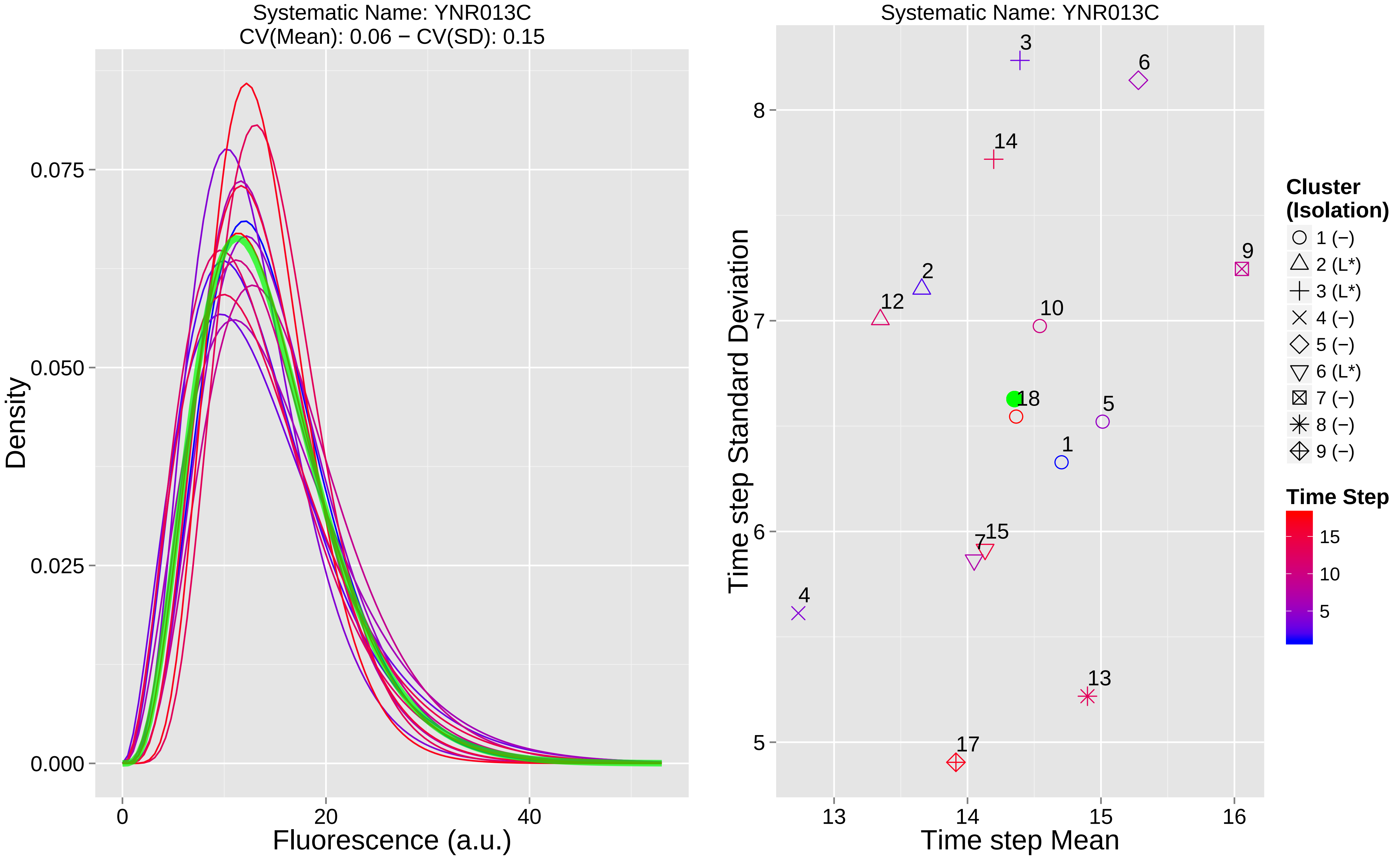
Systematic Name: YNR013C
CV(Mean): 0.06 − CV(SD): 0.15
Density
0.000
0.025
0.050
0.075
0
20
40
Fluorescence (a.u.)
Systematic Name: YNR013C
Time step Standard Deviation
5
6
7
8
13
14
15
16
Time step Mean
3
6
14
9
2
12
10
18
5
1
7
15
4
13
17
Cluster (Isolation)
1 (−)
2 (L*)
3 (L*)
4 (−)
5 (−)
6 (L*)
7 (−)
8 (−)
9 (−)
Time Step
15
10
5

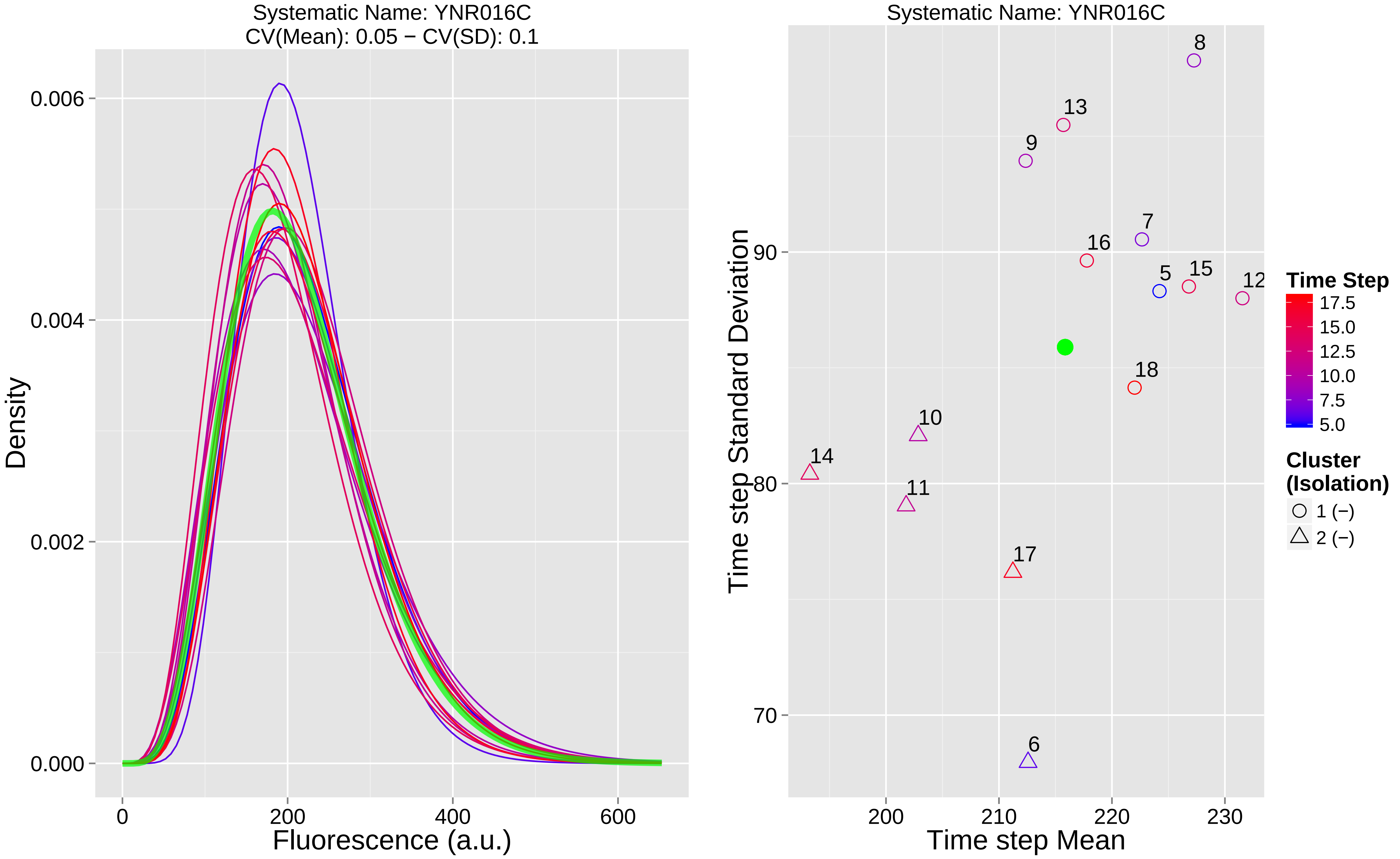

Systematic Name: YNR016C
CV(Mean): 0.05 − CV(SD): 0.1
Density
0.006
0.004
0.002
0.000
0
200
400
600
Fluorescence (a.u.)
Systematic Name: YNR016C
Time step Standard Deviation
90
80
70
200
210
220
230
Time step Mean
8
13
9
7
16
5
15
12
18
10
14
11
17
6
Time Step
17.5
15.0
12.5
10.0
7.5
5.0
Cluster
(Isolation)
1 (−)
2 (−)

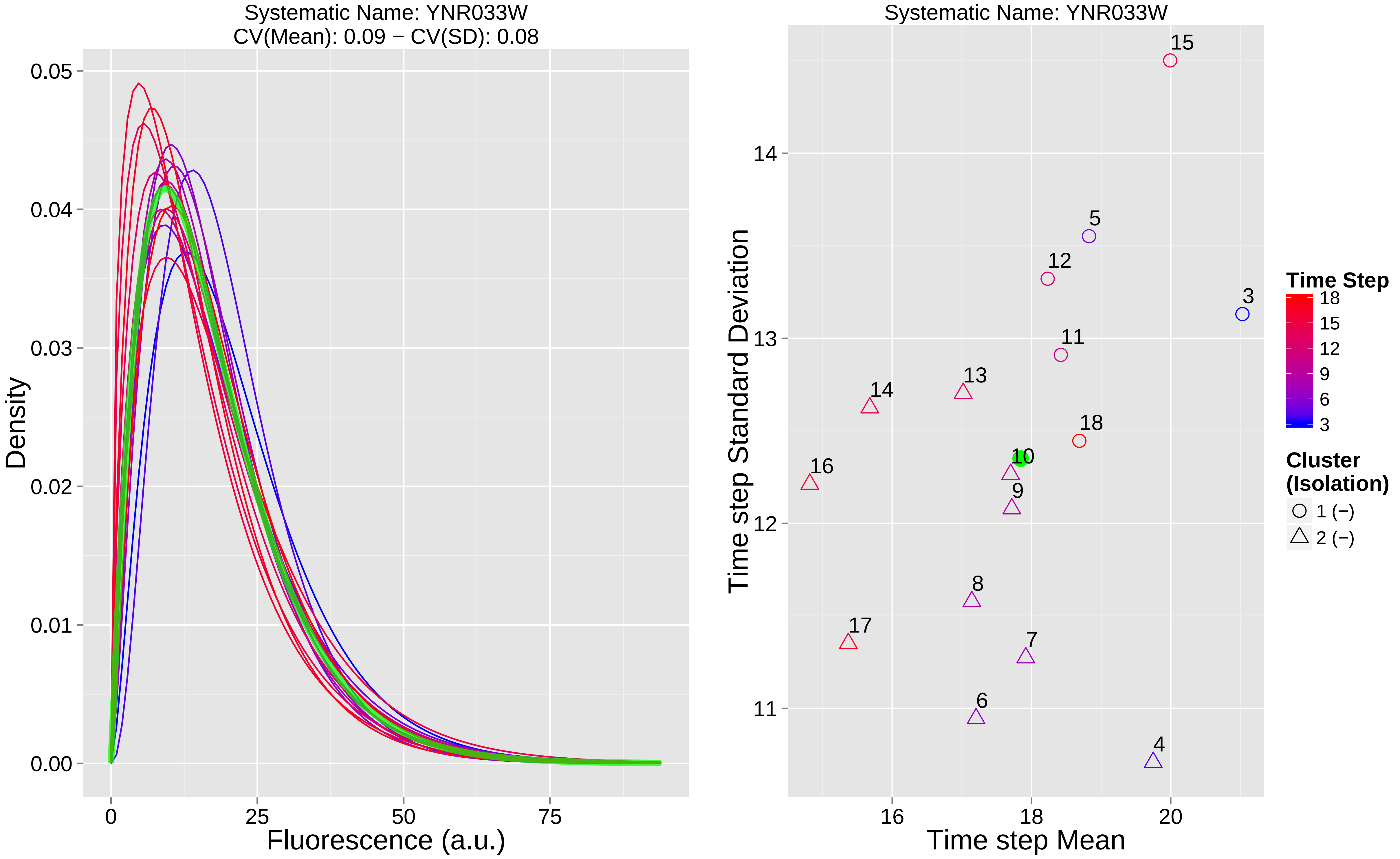

Systematic Name: YNR033W
CV(Mean): 0.09 − CV(SD): 0.08
Density
Fluorescence (a.u.)
0.00
0.01
0.02
0.03
0.04
0.05
0
25
50
75
Systematic Name: YNR033W
Time step Standard Deviation
Time step Mean
11
12
13
14
16
18
20
15
5
12
3
11
13
14
18
10
16
9
8
17
7
6
4
Time Step
18
15
12
9
6
3
Cluster
(Isolation)
1 (−)
2 (−)

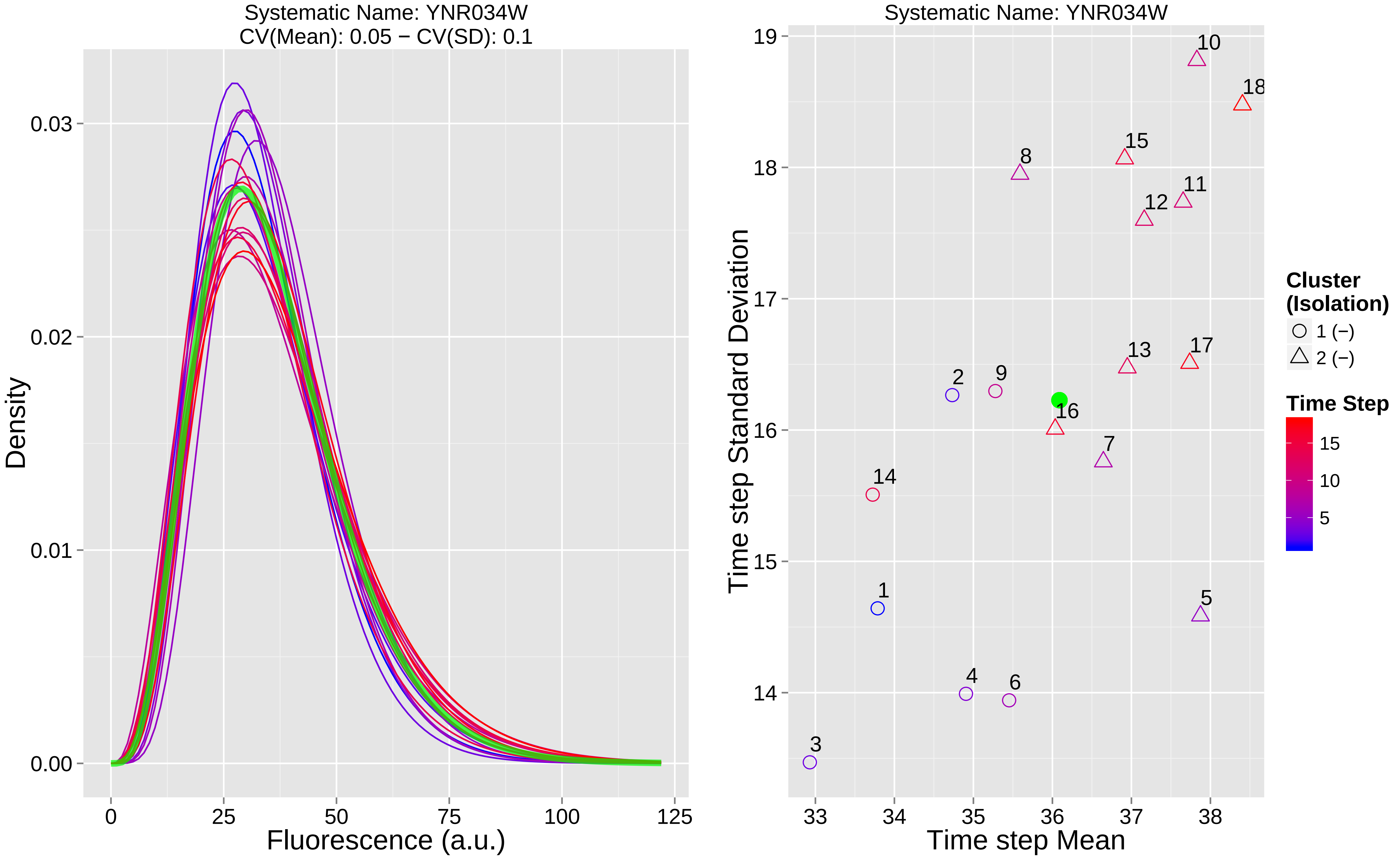

Systematic Name: YNR034W
CV(Mean): 0.05 − CV(SD): 0.1
Density
0.03
0.02
0.01
0.00
0
25
50
75
100
125
Fluorescence (a.u.)
Systematic Name: YNR034W
Time step Standard Deviation
19
18
17
16
15
14
33
34
35
36
37
38
Time step Mean
Cluster
(Isolation)
1 (−)
2 (−)
Time Step
15
10
5

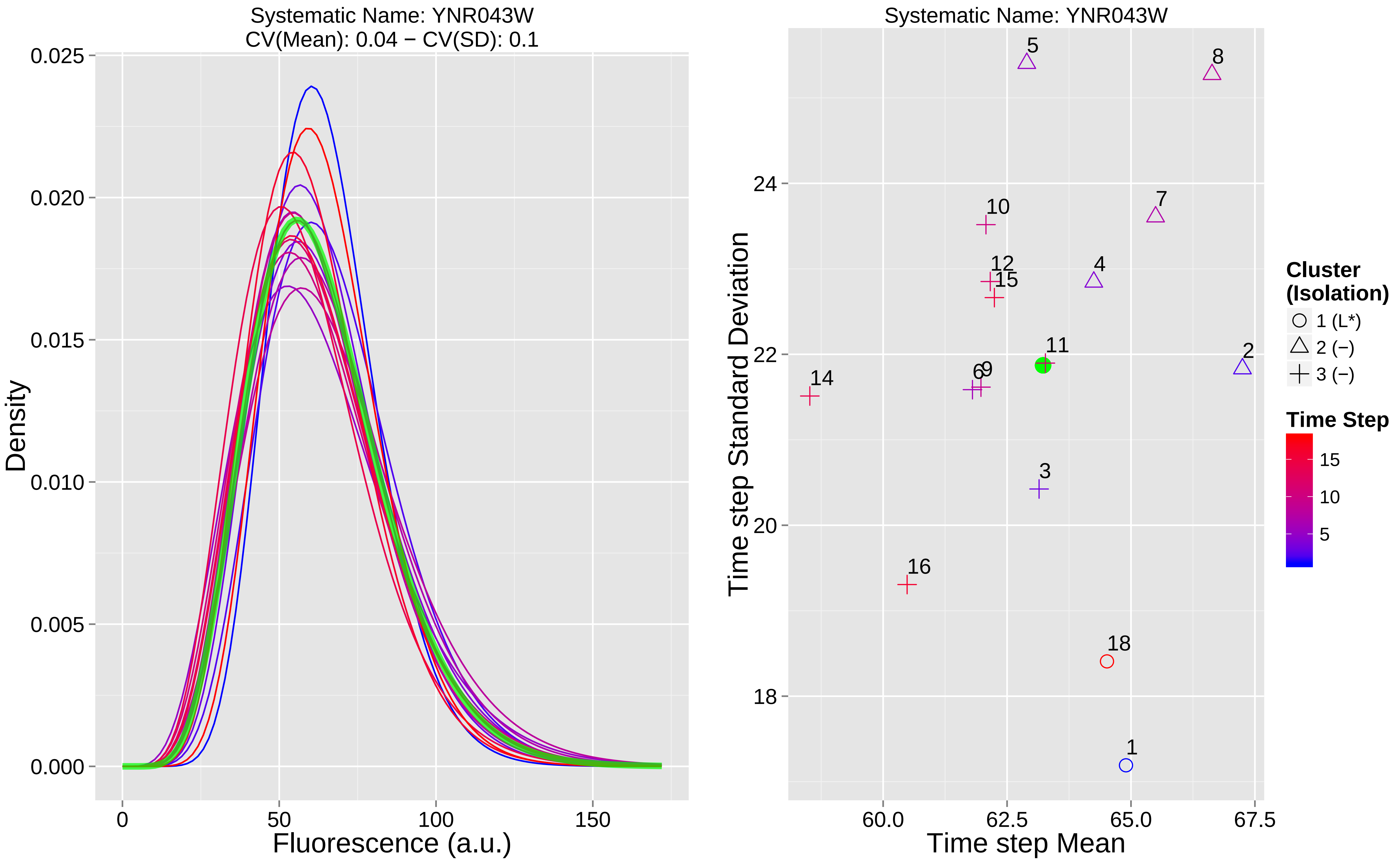

Systematic Name: YNR043W
CV(Mean): 0.04 − CV(SD): 0.1
Density
Fluorescence (a.u.)
0.000
0.005
0.010
0.015
0.020
0.025
0
50
100
150
Systematic Name: YNR043W
Time step Standard Deviation
Time step Mean
18
20
22
24
60.0
62.5
65.0
67.5
Cluster (Isolation)
1 (L*)
2 (−)
3 (−)
Time Step
5
10
15

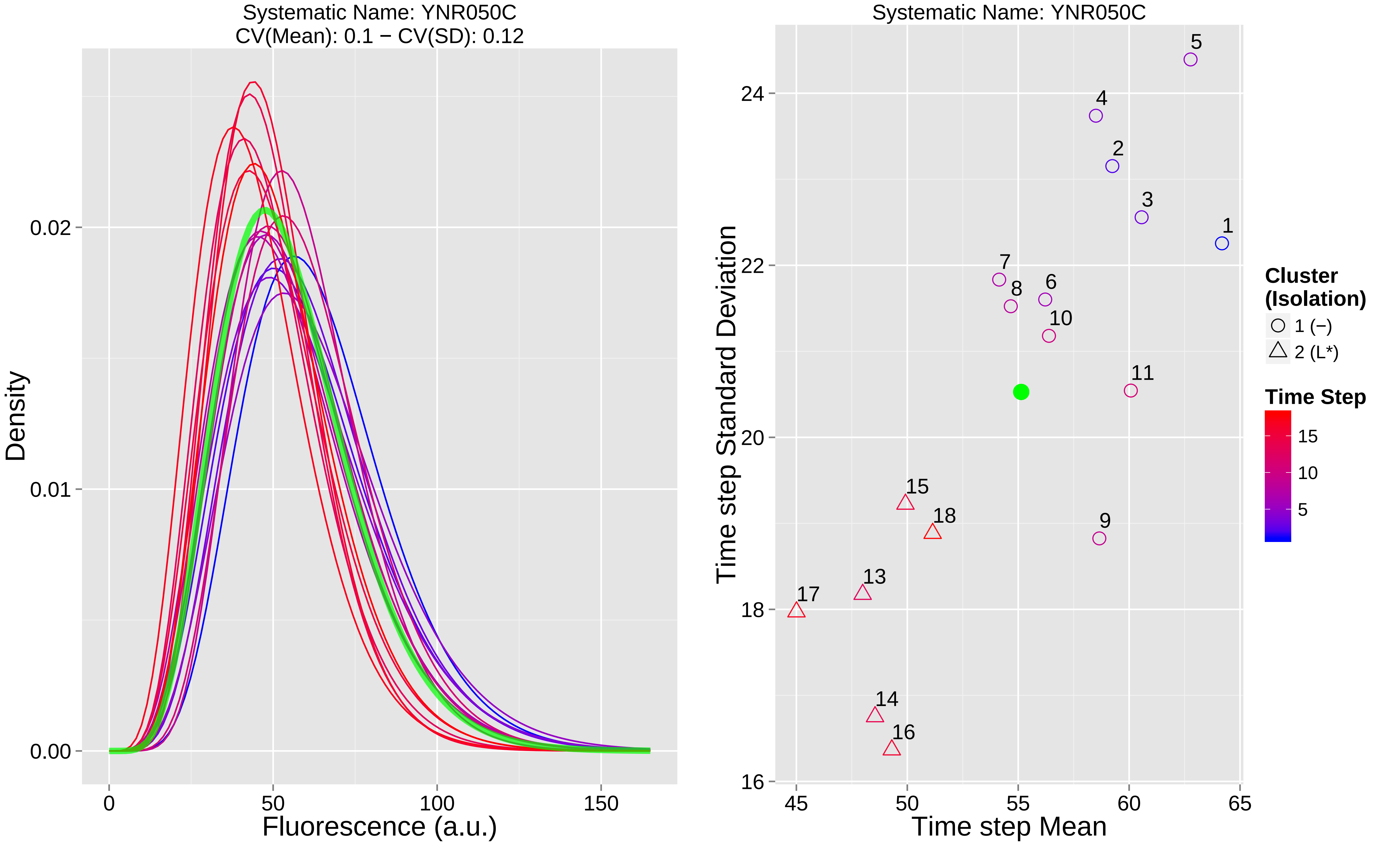

Systematic Name: YNR050C
CV(Mean): 0.1 − CV(SD): 0.12
Density
0.00
0.01
0.02
0
50
100
150
Fluorescence (a.u.)
Systematic Name: YNR050C
Time step Standard Deviation
16
18
20
22
24
45
50
55
60
65
Time step Mean
1
2
3
4
5
6
7
8
9
10
11
13
14
15
16
17
18
Cluster (Isolation)
1 (−)
2 (L*)
Time Step
15
10
5

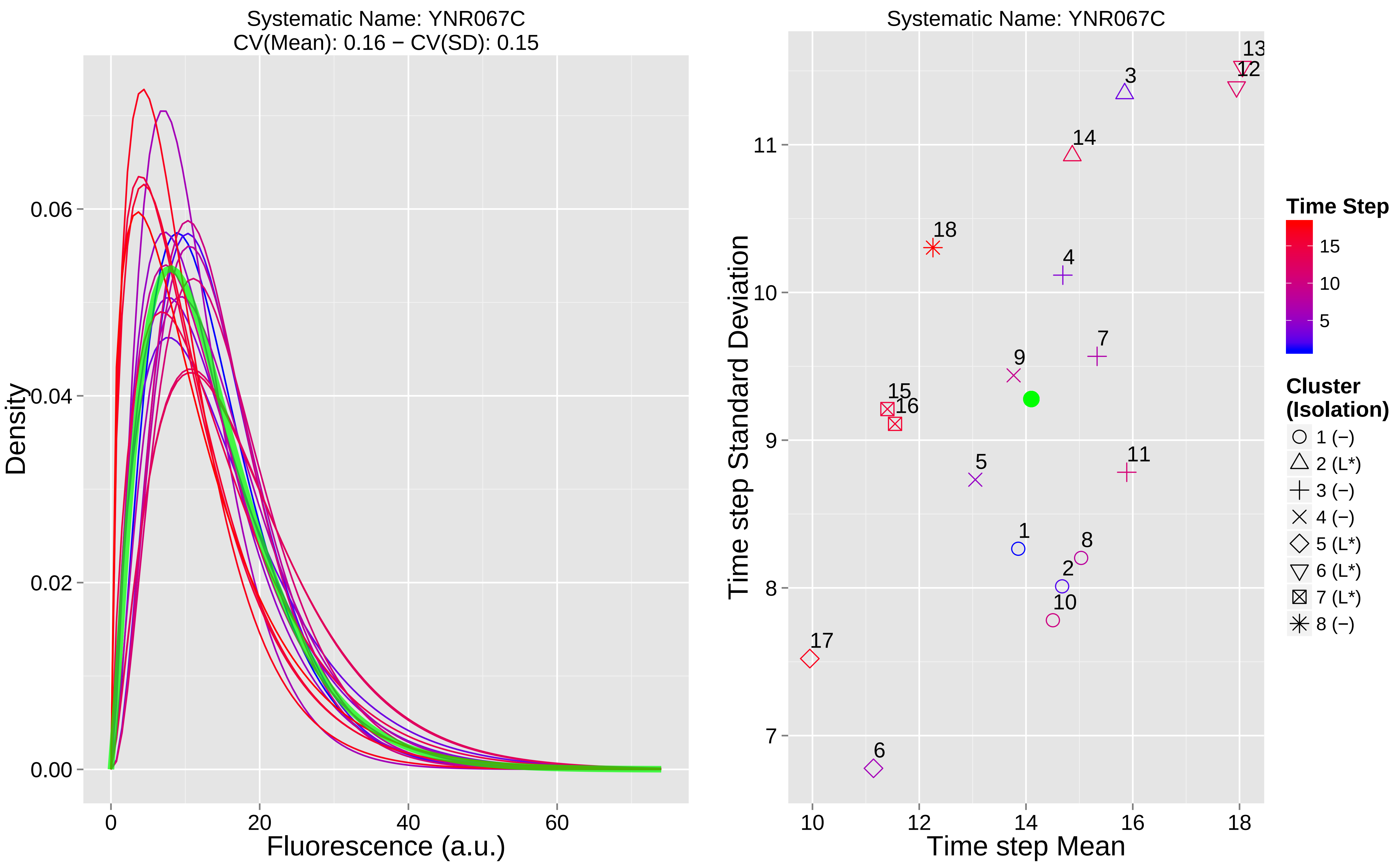

Systematic Name: YNR067C
CV(Mean): 0.16 − CV(SD): 0.15
Density
Fluorescence (a.u.)
0.00
0.02
0.04
0.06
0
20
40
60
Systematic Name: YNR067C
Time step Standard Deviation
Time step Mean
Time Step
15
10
5
Cluster (Isolation)
1 (−)
2 (L*)
3 (−)
4 (−)
5 (L*)
6 (L*)
7 (L*)
8 (−)

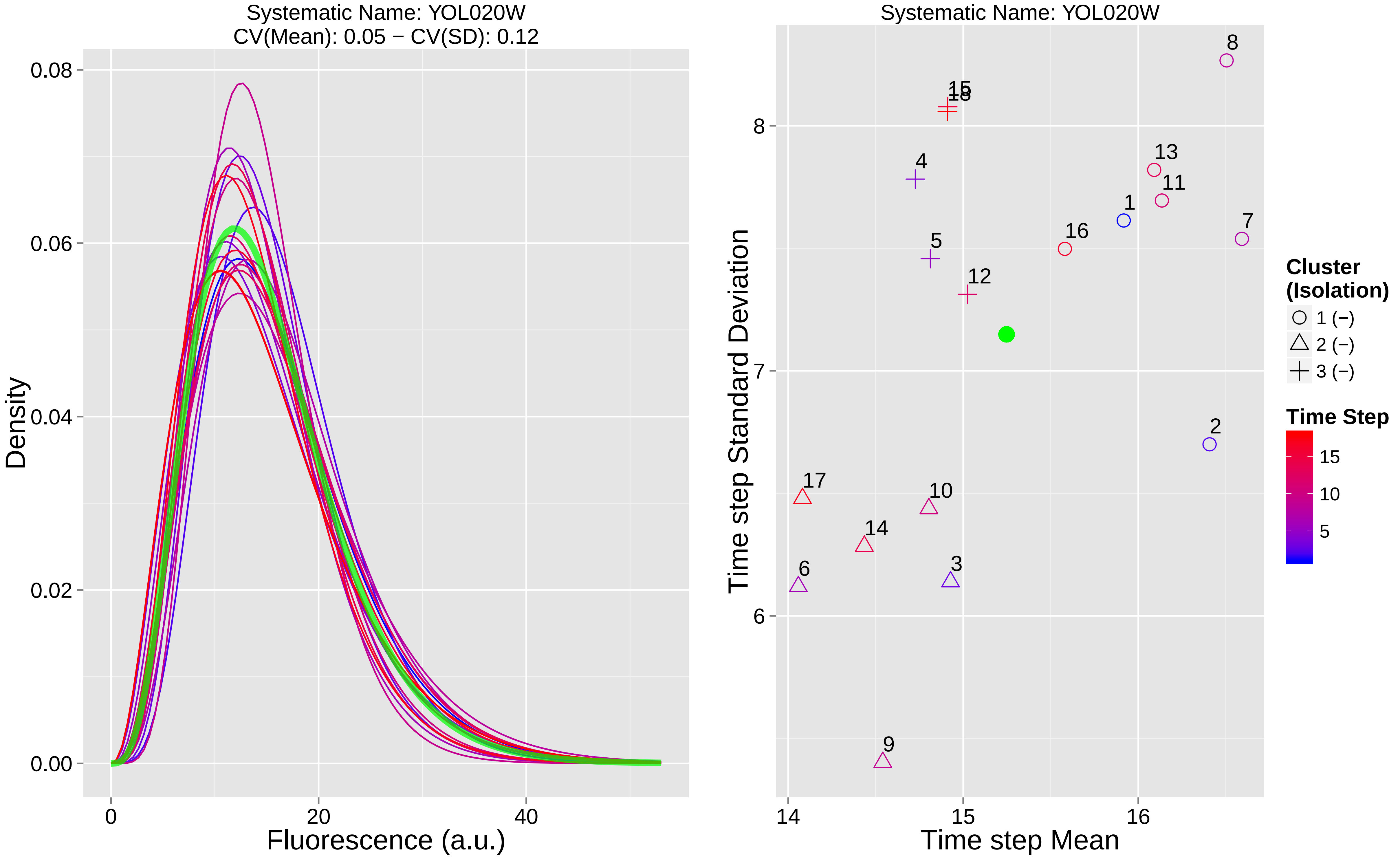
Systematic Name: YOL020W
CV(Mean): 0.05 − CV(SD): 0.12
Density
Fluorescence (a.u.)
0.00
0.02
0.04
0.06
0.08
0
20
40
Systematic Name: YOL020W
Time step Standard Deviation
Time step Mean
6
7
8
14
15
16
1
2
3
4
5
6
7
8
9
10
11
12
13
14
15
16
17
18
Cluster
(Isolation)
1 (−)
2 (−)
3 (−)
Time Step
5
10
15

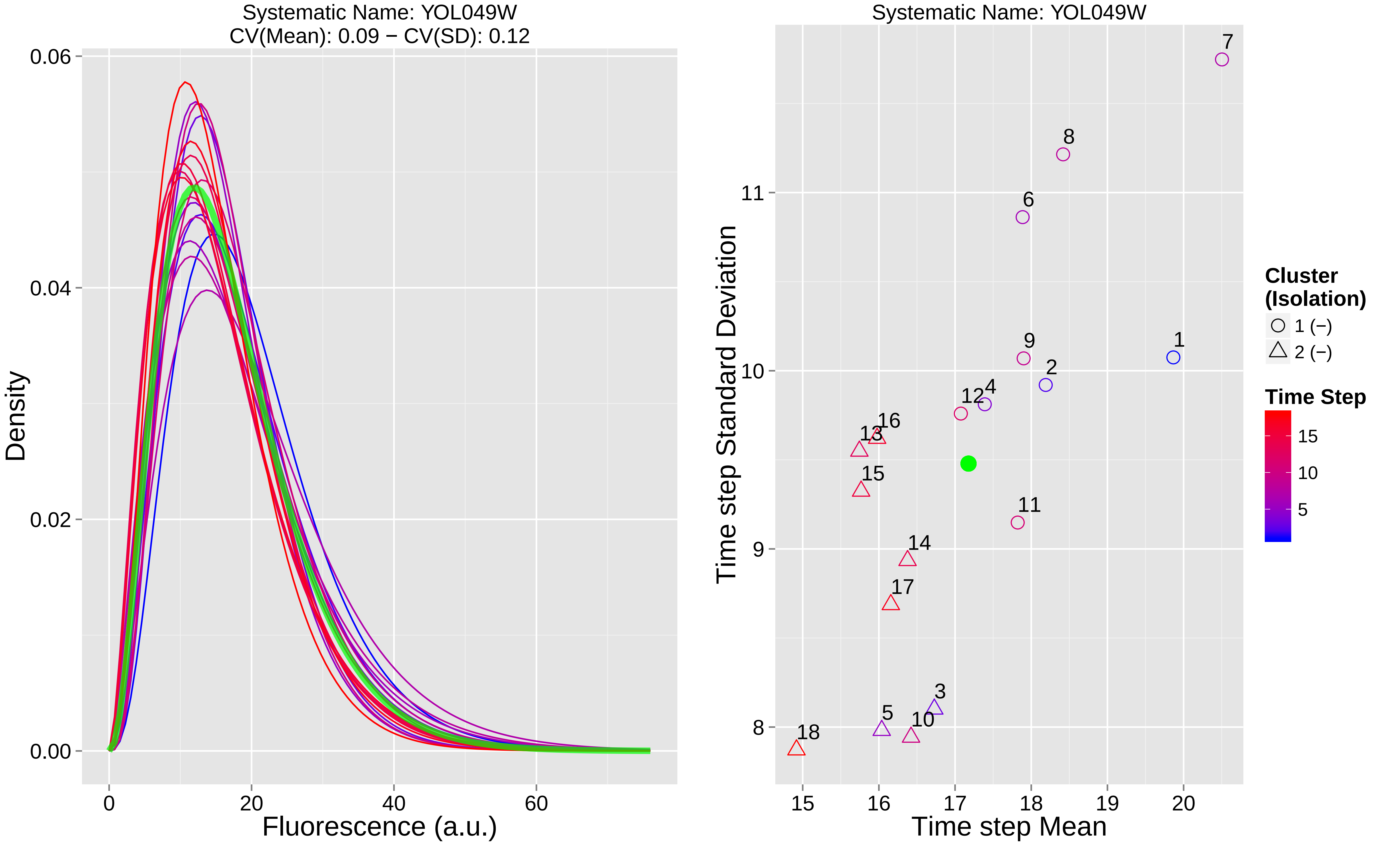
Systematic Name: YOL049W
CV(Mean): 0.09 − CV(SD): 0.12
Density
Fluorescence (a.u.)
0.06
0.04
0.02
0.00
0
20
40
60
Systematic Name: YOL049W
Time step Standard Deviation
Time step Mean
Cluster (Isolation)
1 (−)
2 (−)
Time Step
15
10
5

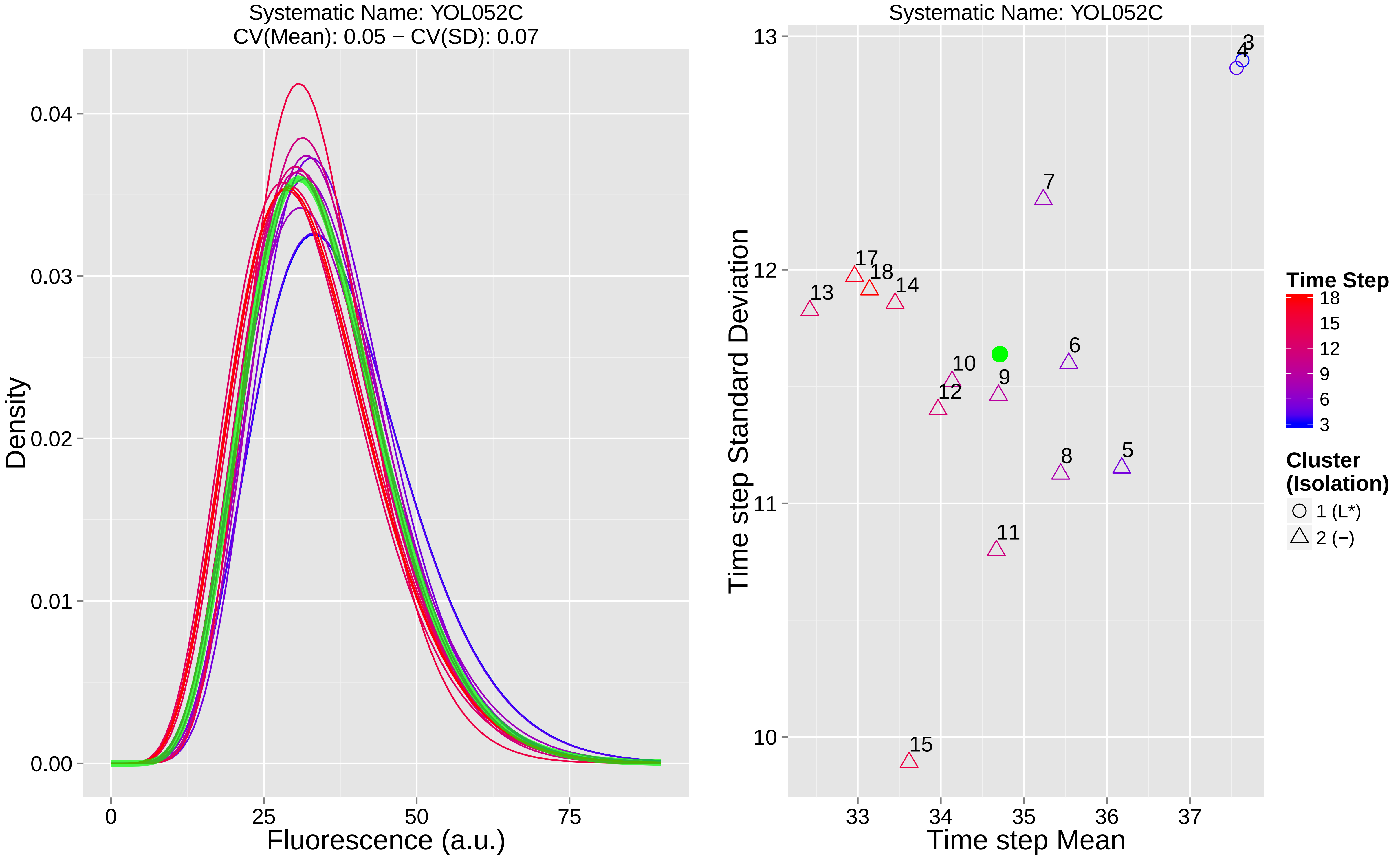

Systematic Name: YOL052C
CV(Mean): 0.05 − CV(SD): 0.07
Density
0.00
0.01
0.02
0.03
0.04
0
25
50
75
Fluorescence (a.u.)
Systematic Name: YOL052C
Time step Standard Deviation
10
11
12
13
33
34
35
36
37
Time step Mean
3
4
5
6
7
8
9
10
11
12
13
14
15
17
18
Time Step
18
15
12
9
6
3
Cluster
(Isolation)
1 (L*)
2 (−)

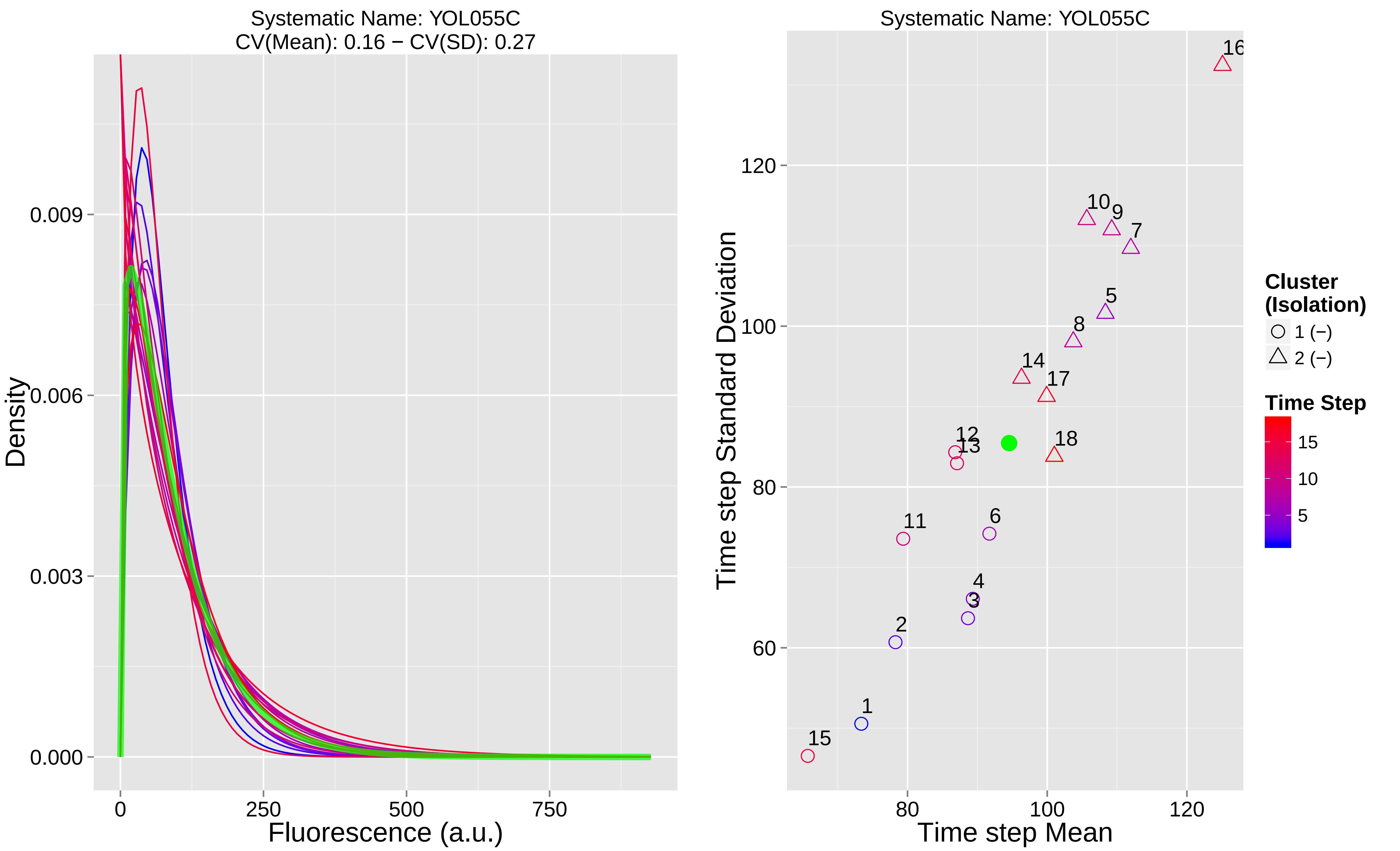

Systematic Name: YOL055C
CV(Mean): 0.16 − CV(SD): 0.27
Density
0.009
0.006
0.003
0.000
0
250
500
750
Fluorescence (a.u.)
Systematic Name: YOL055C
Time step Standard Deviation
120
100
80
60
Time step Mean
80
100
120
16
10
9
7
5
8
14
17
12
13
18
11
6
4
3
2
1
15
Cluster (Isolation)
1 (−)
2 (−)
Time Step
15
10
5

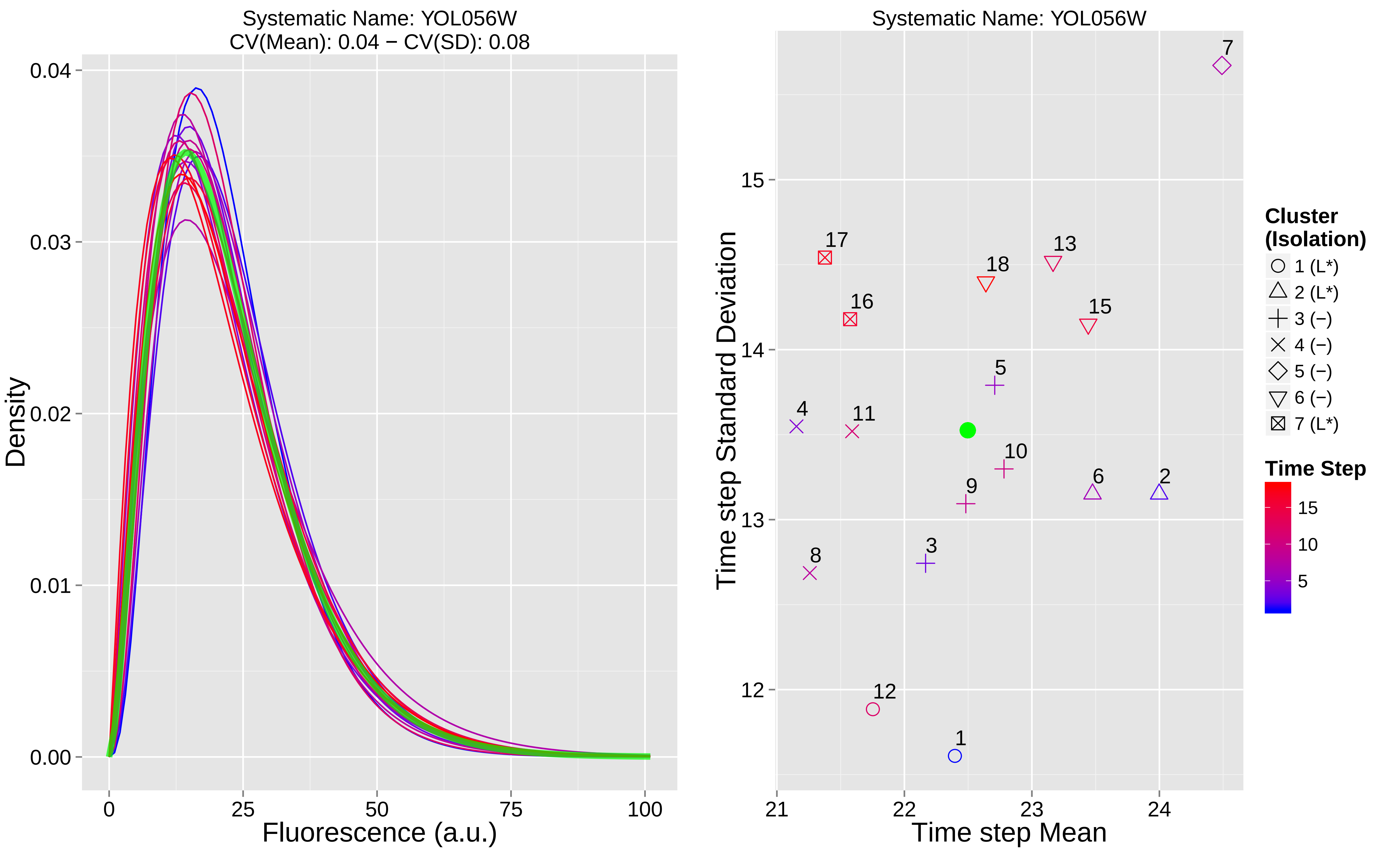

Systematic Name: YOL056W
CV(Mean): 0.04 − CV(SD): 0.08
Density
0.04
0.03
0.02
0.01
0.00
0
25
50
75
100
Fluorescence (a.u.)
Systematic Name: YOL056W
Time step Standard Deviation
15
14
13
12
21
22
23
24
Time step Mean
7
17
13
18
16
15
5
4
11
10
9
6
2
3
8
12
1
Cluster (Isolation)
1 (L*)
2 (L*)
3 (−)
4 (−)
5 (−)
6 (−)
7 (L*)
Time Step
15
10
5

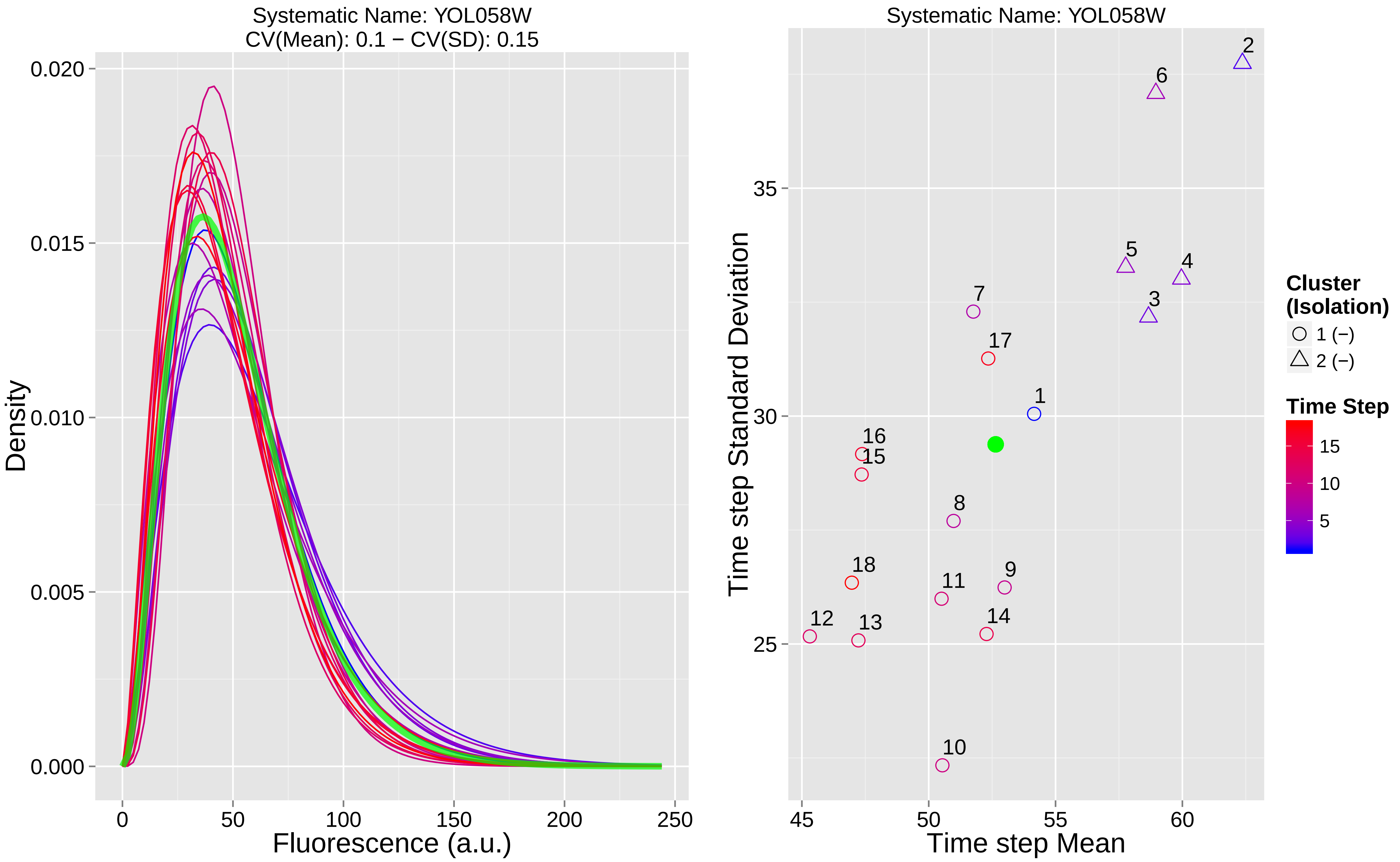

Systematic Name: YOL058W
CV(Mean): 0.1 − CV(SD): 0.15
Density
Fluorescence (a.u.)
0.020
0.015
0.010
0.005
0.000
0
50
100
150
200
250
Systematic Name: YOL058W
Time step Standard Deviation
Time step Mean
35
30
25
45
50
55
60
Cluster (Isolation)
1 (−)
2 (−)
Time Step
15
10
5

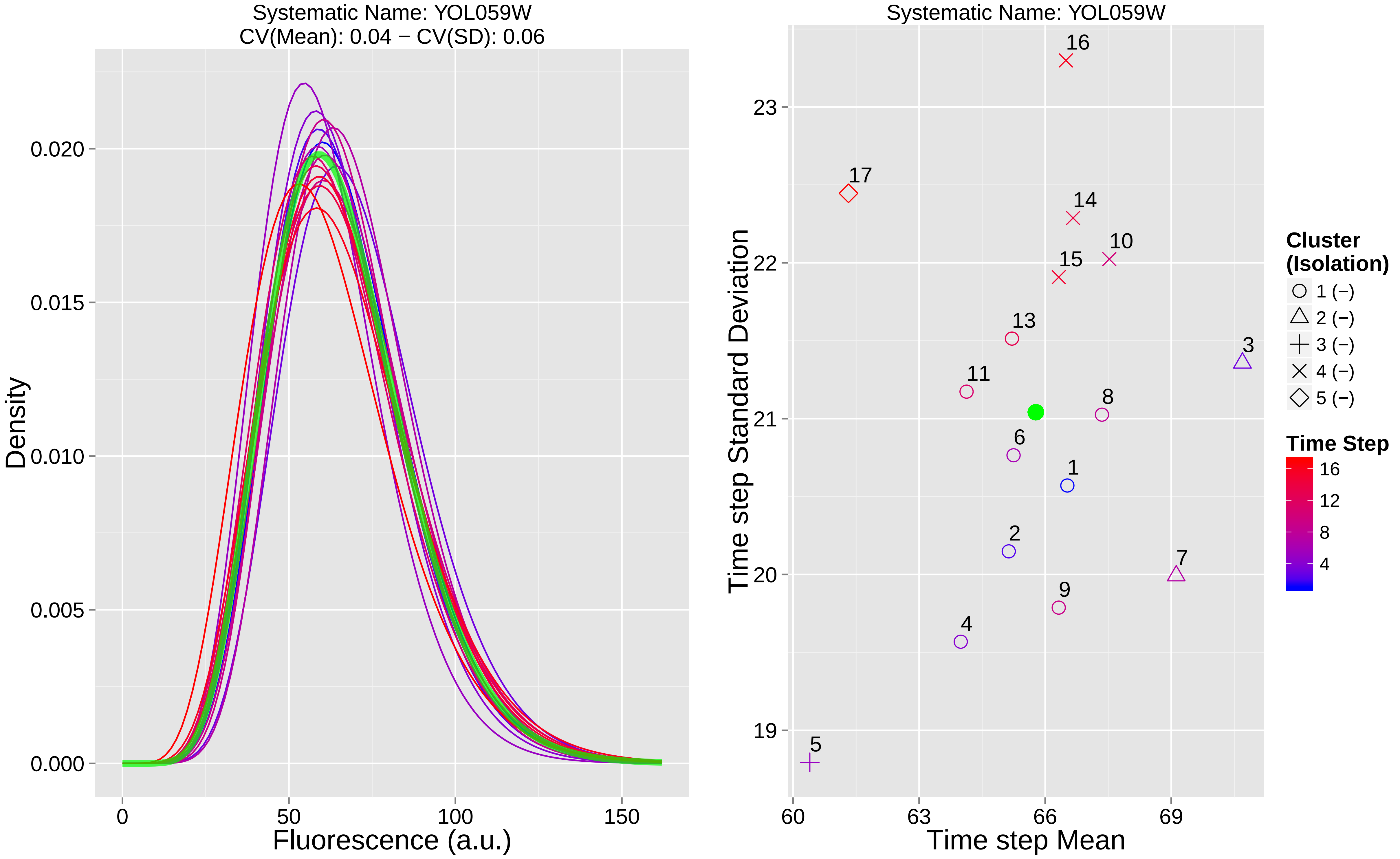

Systematic Name: YOL059W
CV(Mean): 0.04 − CV(SD): 0.06
Density
0.020
0.015
0.010
0.005
0.000
0
50
100
150
Fluorescence (a.u.)
Systematic Name: YOL059W
Time step Standard Deviation
23
22
21
20
19
60
63
66
69
Time step Mean
16
17
14
10
15
13
3
11
8
6
1
2
7
9
4
5
Cluster (Isolation)
1 (−)
2 (−)
3 (−)
4 (−)
5 (−)
Time Step
16
12
8
4

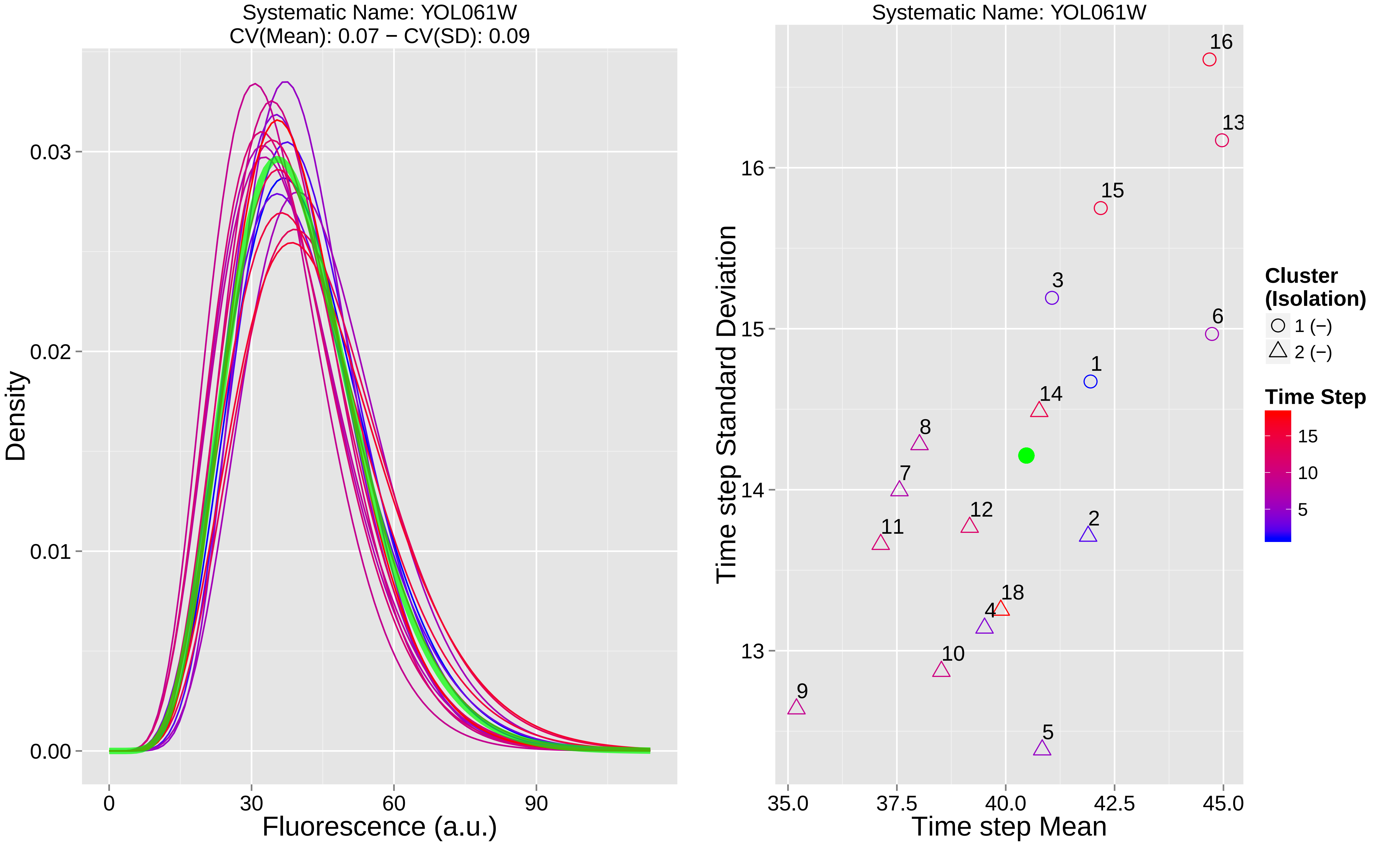
Systematic Name: YOL061W
CV(Mean): 0.07 − CV(SD): 0.09
Density
0.03
0.02
0.01
0.00
0
30
60
90
Fluorescence (a.u.)
Systematic Name: YOL061W
Time step Standard Deviation
Time step Mean
16
15
14
13
35.0
37.5
40.0
42.5
45.0
Cluster
(Isolation)
1 (−)
2 (−)
Time Step
15
10
5

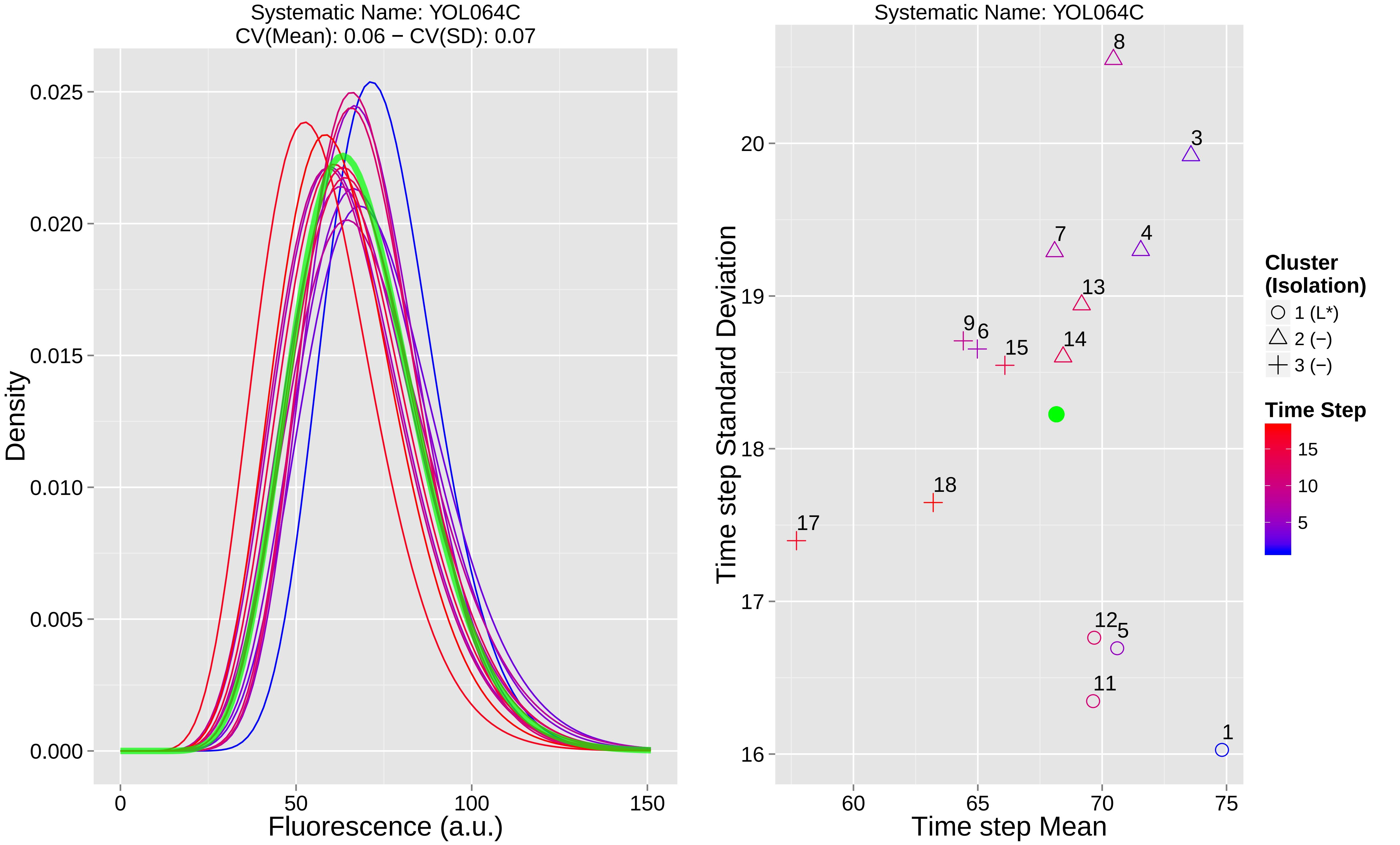

Systematic Name: YOL064C
CV(Mean): 0.06 − CV(SD): 0.07
Density
0.025
0.020
0.015
0.010
0.005
0.000
0
50
100
150
Fluorescence (a.u.)
Systematic Name: YOL064C
Time step Standard Deviation
20
19
18
17
16
60
65
70
75
Time step Mean
8
3
7
4
13
9
6
15
14
18
17
12
5
11
1
Cluster (Isolation)
1 (L*)
2 (−)
3 (−)
Time Step
15
10
5

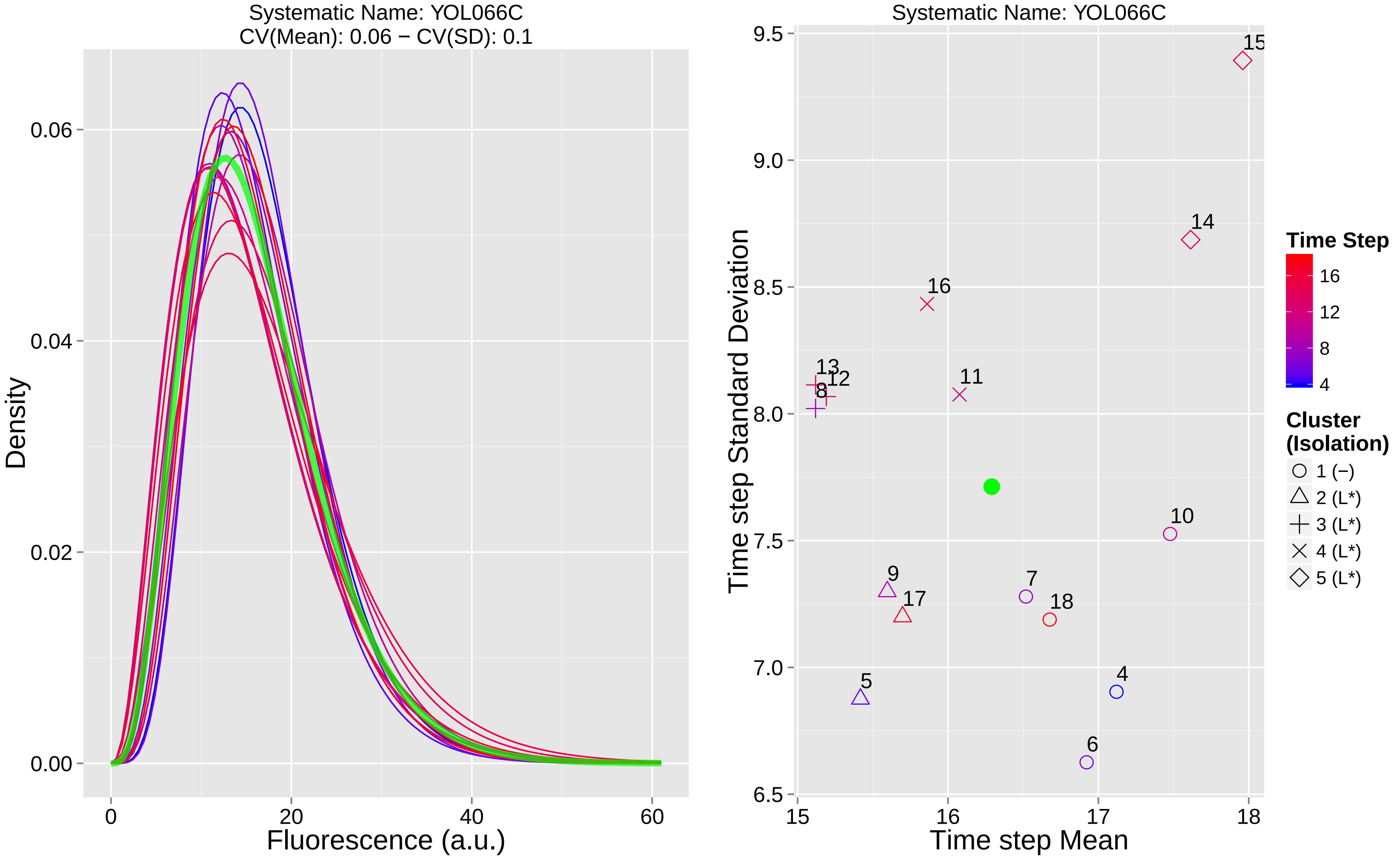

Systematic Name: YOL066C
CV(Mean): 0.06 − CV(SD): 0.1
Density
0.00
0.02
0.04
0.06
0
20
40
60
Fluorescence (a.u.)
Systematic Name: YOL066C
Time step Standard Deviation
6.5
7.0
7.5
8.0
8.5
9.0
9.5
15
16
17
18
Time step Mean
15
14
16
13
12
8
11
10
9
7
17
18
5
4
6
Time Step
16
12
8
4
Cluster
(Isolation)
1 (−)
2 (L*)
3 (L*)
4 (L*)
5 (L*)

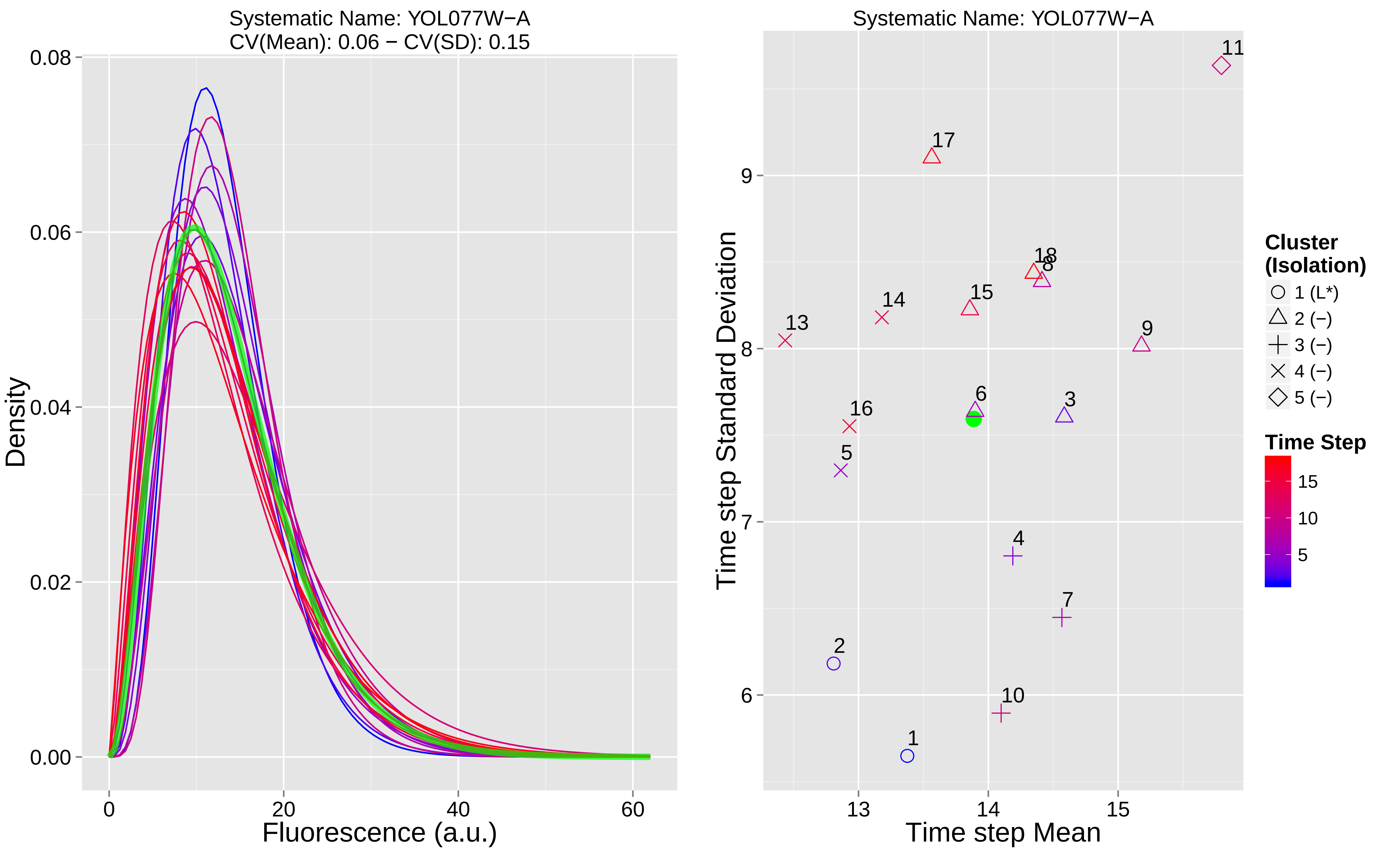

Systematic Name: YOL077W−A
CV(Mean): 0.06 − CV(SD): 0.15
Density
Fluorescence (a.u.)
0.00
0.02
0.04
0.06
0.08
0
20
40
60
Systematic Name: YOL077W−A
Time step Standard Deviation
Time step Mean
6
7
8
9
13
14
15
1
2
3
4
5
6
7
8
9
10
11
13
14
15
16
17
18
Cluster (Isolation)
1 (L*)
2 (−)
3 (−)
4 (−)
5 (−)
Time Step
5
10
15

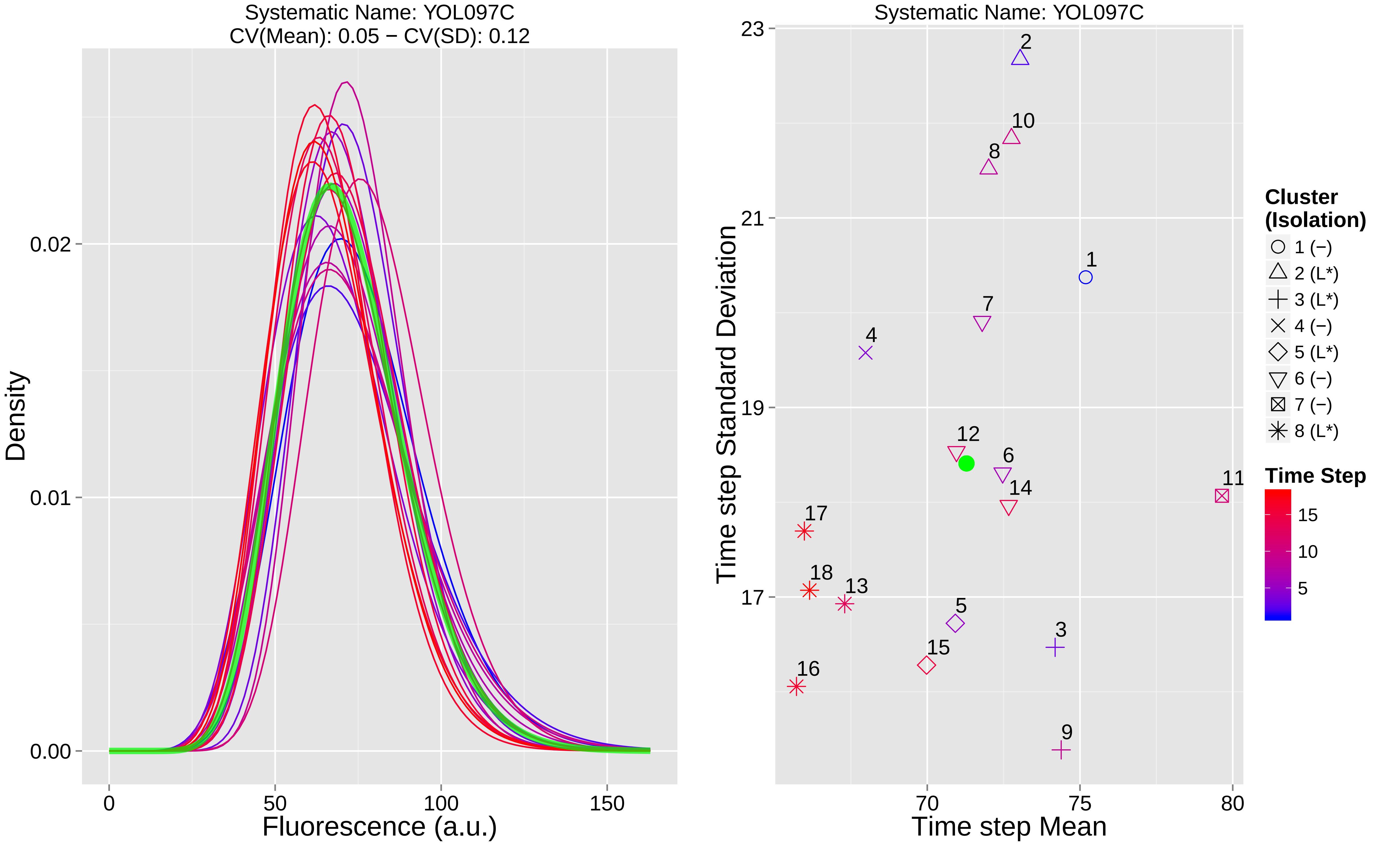

Systematic Name: YOL097C
CV(Mean): 0.05 − CV(SD): 0.12
Density
0.00
0.01
0.02
0
50
100
150
Fluorescence (a.u.)
Systematic Name: YOL097C
Time step Standard Deviation
17
19
21
23
70
75
80
Time step Mean
1
2
3
4
5
6
7
8
9
10
11
12
13
14
15
16
17
18
Cluster (Isolation)
1 (−)
2 (L*)
3 (L*)
4 (−)
5 (L*)
6 (−)
7 (−)
8 (L*)
Time Step
15
10
5

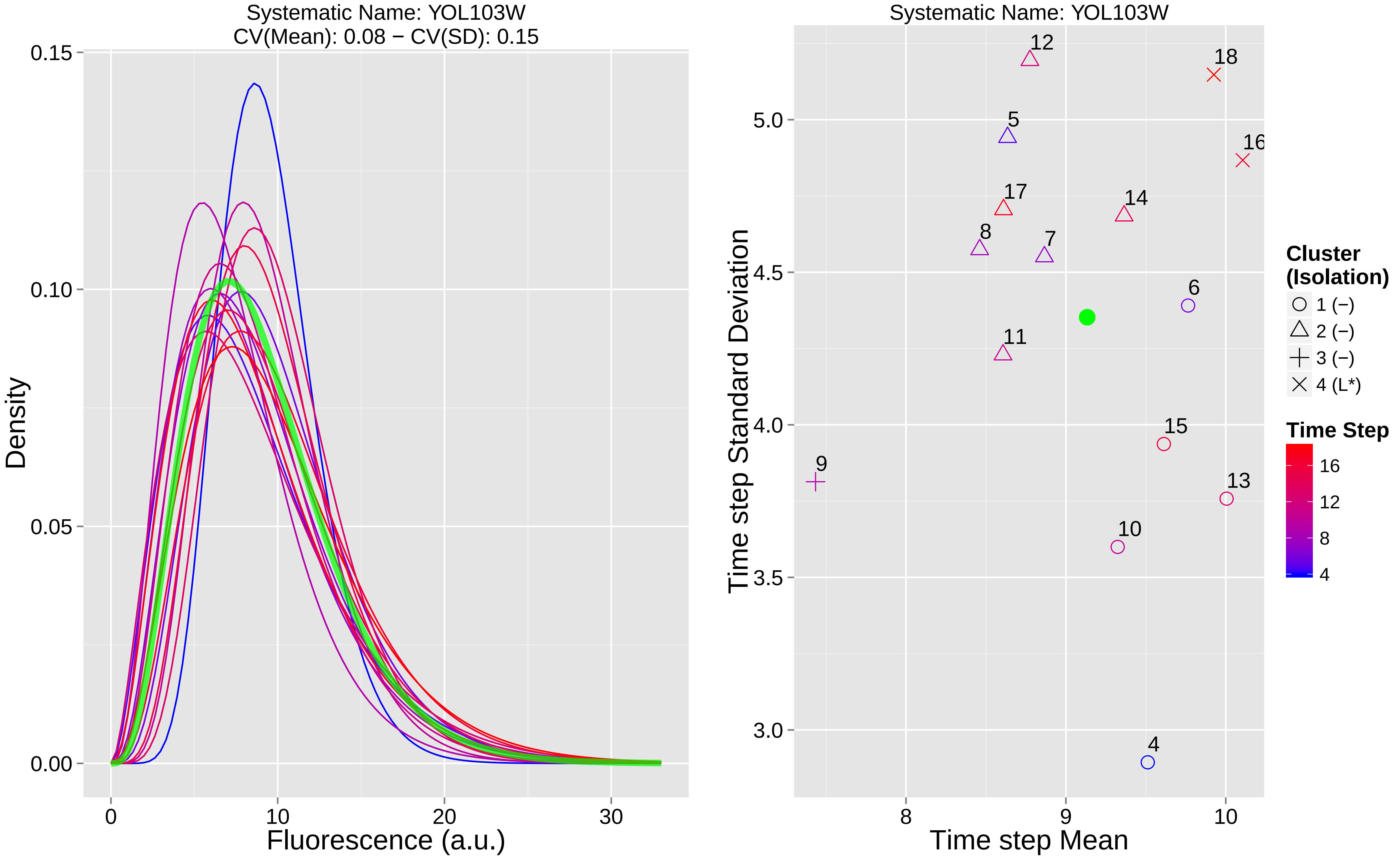
Systematic Name: YOL103W
CV(Mean): 0.08 − CV(SD): 0.15
Density
Fluorescence (a.u.)
0.15
0.10
0.05
0.00
0
10
20
30
Systematic Name: YOL103W
Time step Standard Deviation
Time step Mean
5.0
4.5
4.0
3.5
3.0
8
9
10
12
18
5
16
17
14
8
7
6
11
15
9
13
10
4
Cluster
(Isolation)
1 (−)
2 (−)
3 (−)
4 (L*)
Time Step
16
12
8
4

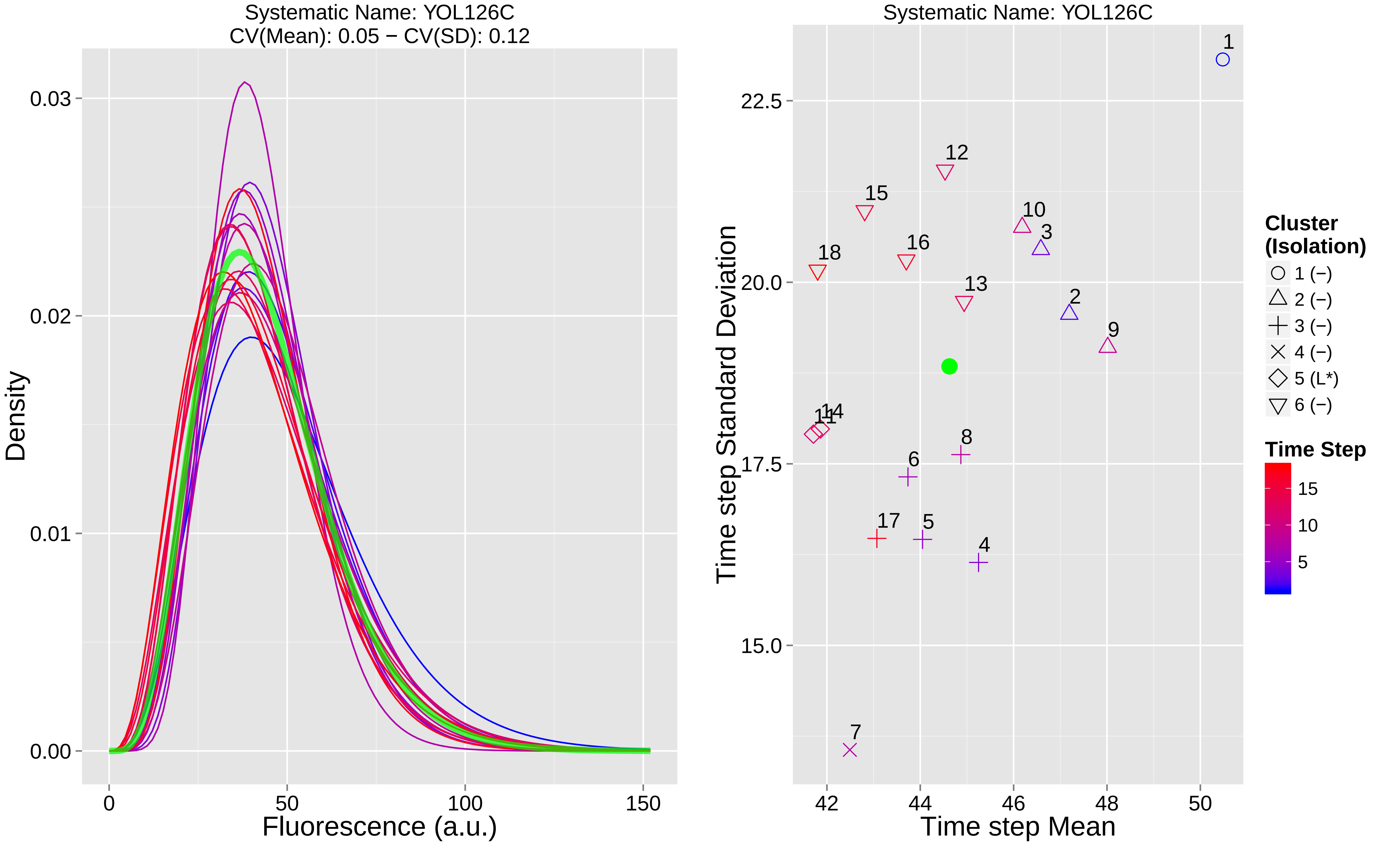
Systematic Name: YOL126C
CV(Mean): 0.05 − CV(SD): 0.12
Density
Fluorescence (a.u.)
0.00
0.01
0.02
0.03
0
50
100
150
Systematic Name: YOL126C
Time step Standard Deviation
Time step Mean
15.0
17.5
20.0
22.5
42
44
46
48
50
Cluster (Isolation)
1 (−)
2 (−)
3 (−)
4 (−)
5 (L*)
6 (−)
Time Step
5
10
15

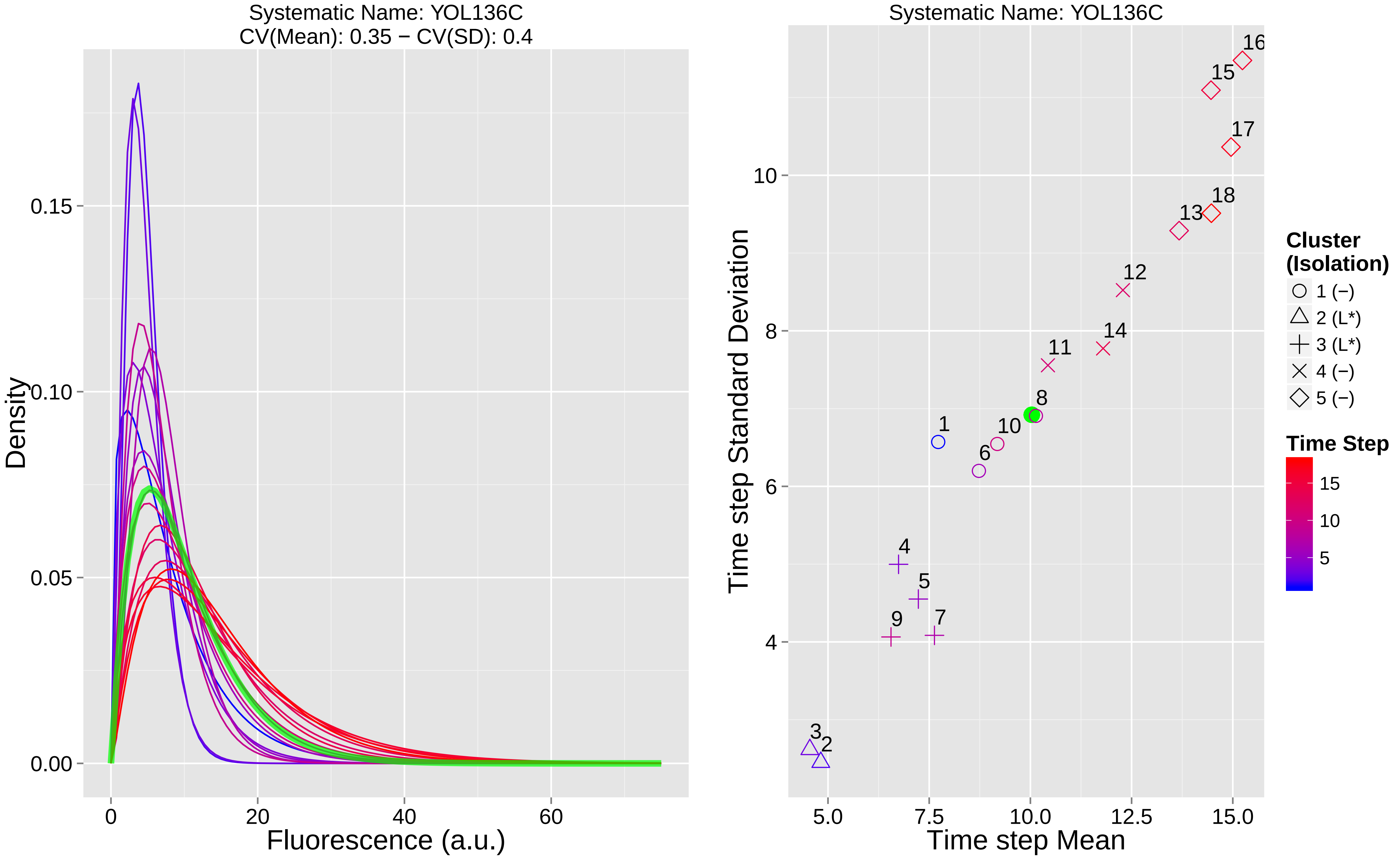

Systematic Name: YOL136C
CV(Mean): 0.35 − CV(SD): 0.4
Density
0.00
0.05
0.10
0.15
0
20
40
60
Fluorescence (a.u.)
Systematic Name: YOL136C
Time step Standard Deviation
4
6
8
10
5.0
7.5
10.0
12.5
15.0
Time step Mean
Cluster
(Isolation)
1 (−)
2 (L*)
3 (L*)
4 (−)
5 (−)
Time Step
5
10
15

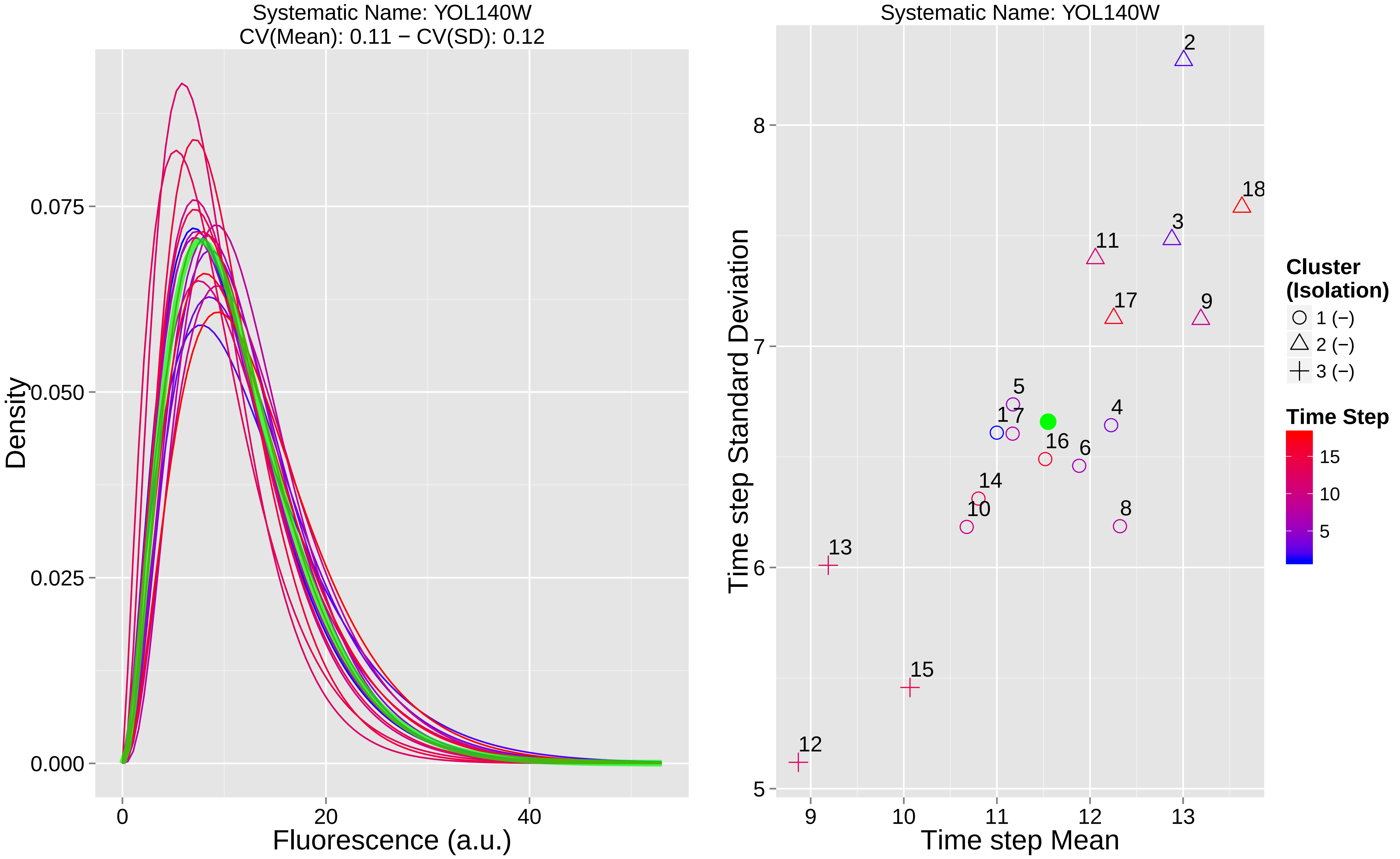

Systematic Name: YOL140W
CV(Mean): 0.11 − CV(SD): 0.12
Density
0.000
0.025
0.050
0.075
0
20
40
Fluorescence (a.u.)
Systematic Name: YOL140W
Time step Standard Deviation
5
6
7
8
9
10
11
12
13
Time step Mean
2
18
3
11
17
9
5
17
4
16
6
14
10
8
13
15
12
Cluster (Isolation)
1 (−)
2 (−)
3 (−)
Time Step
15
10
5

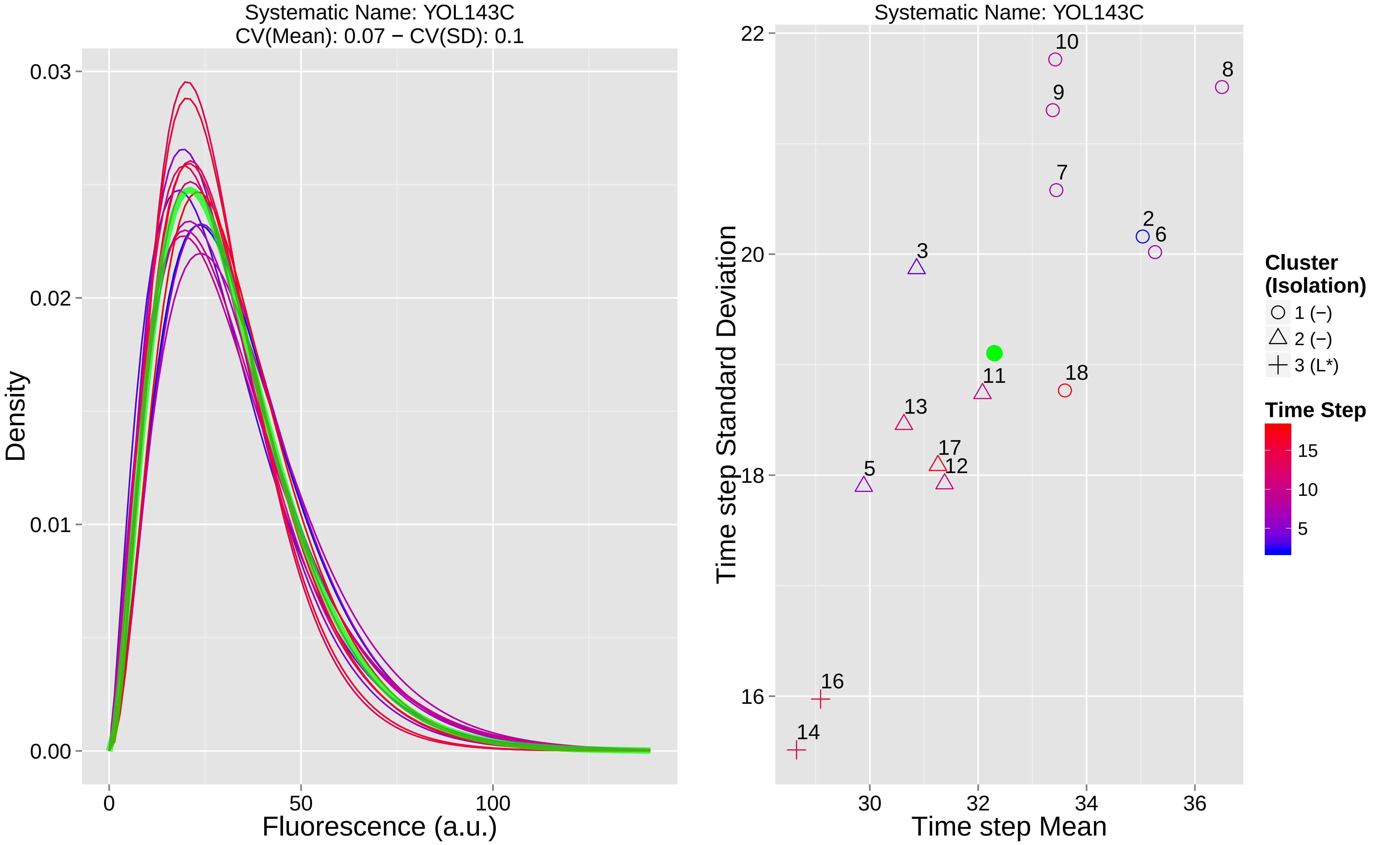

Systematic Name: YOL143C
CV(Mean): 0.07 − CV(SD): 0.1
Density
0.03
0.02
0.01
0.00
0
50
100
Fluorescence (a.u.)
Systematic Name: YOL143C
Time step Standard Deviation
22
20
18
16
30
32
34
36
Time step Mean
10
8
9
7
2
6
3
11
18
13
17
12
5
16
14
Cluster (Isolation)
1 (−)
2 (−)
3 (L*)
Time Step
15
10
5

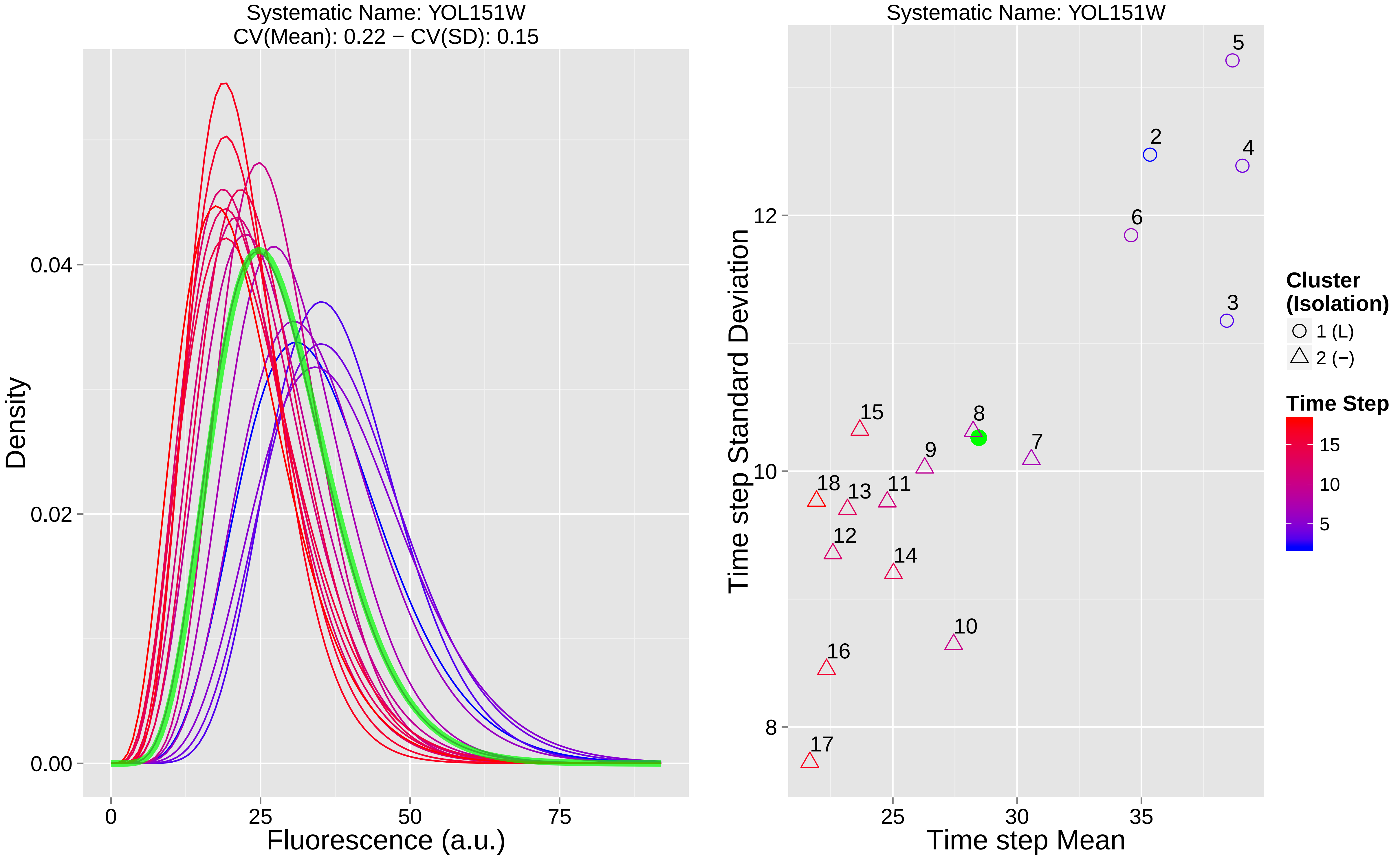

Systematic Name: YOL151W
CV(Mean): 0.22 − CV(SD): 0.15
Density
0.00
0.02
0.04
0
25
50
75
Fluorescence (a.u.)
Systematic Name: YOL151W
Time step Standard Deviation
8
10
12
25
30
35
Time step Mean
1
2
3
4
5
6
7
8
9
10
11
12
13
14
15
16
17
18
Cluster
(Isolation)
1 (L)
2 (−)
Time Step
15
10
5

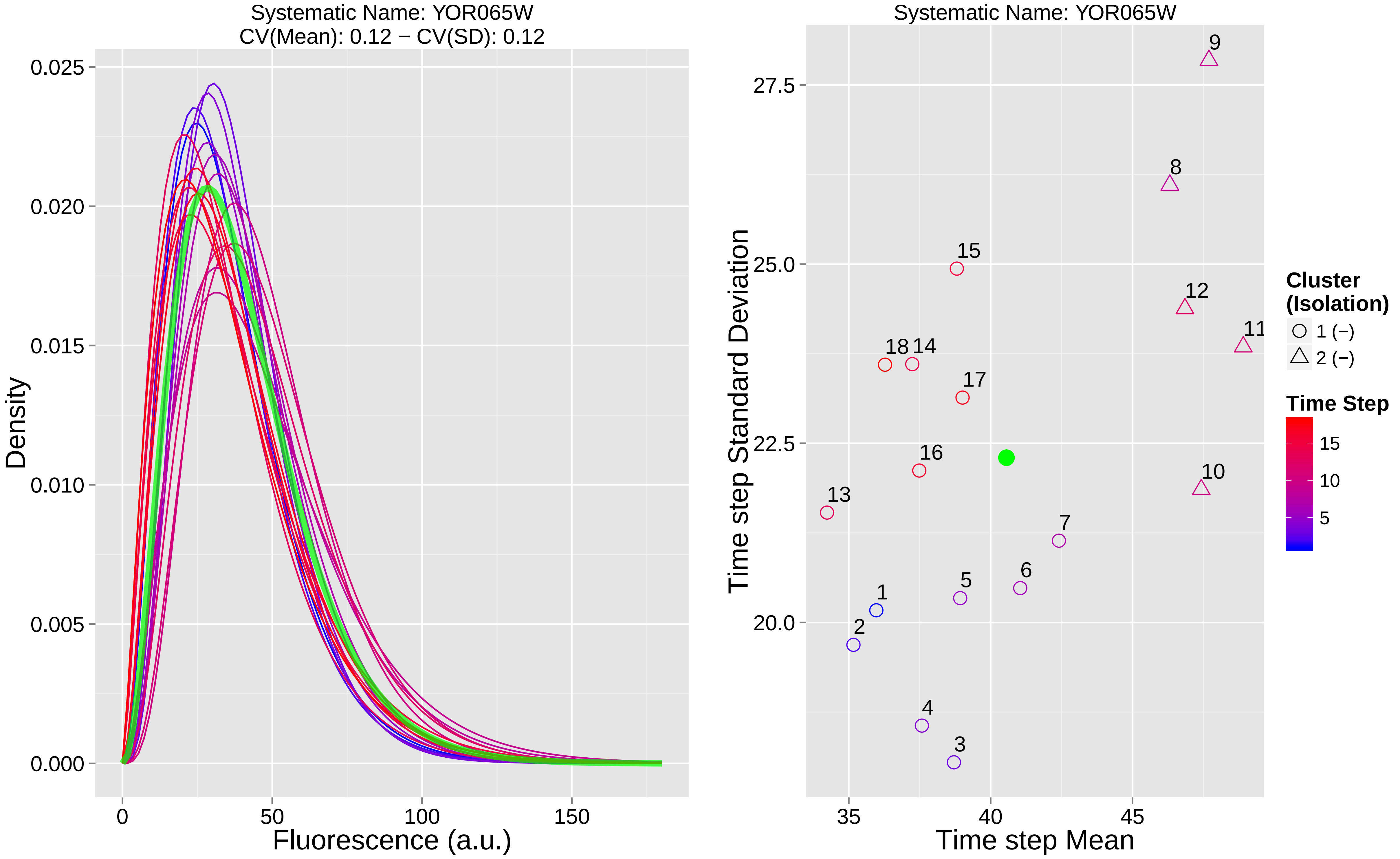

Systematic Name: YOR065W
CV(Mean): 0.12 − CV(SD): 0.12
Density
0.025
0.020
0.015
0.010
0.005
0.000
0
50
100
150
Fluorescence (a.u.)
Systematic Name: YOR065W
Time step Standard Deviation
27.5
25.0
22.5
20.0
35
40
45
Time step Mean
1
2
3
4
5
6
7
8
9
10
11
12
13
14
15
16
17
18
Cluster
(Isolation)
1 (−)
2 (−)
Time Step
15
10
5

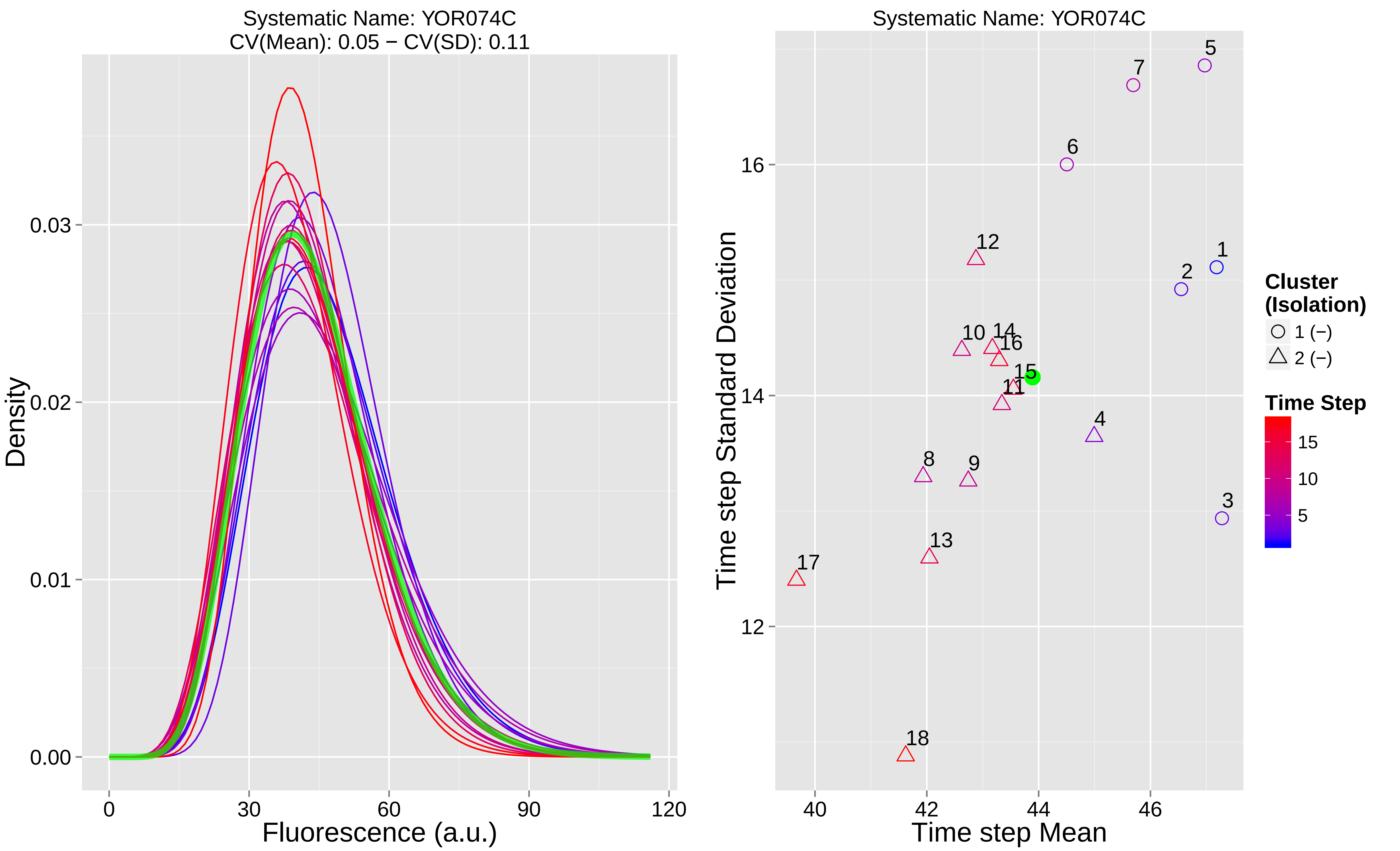
Systematic Name: YOR074C
CV(Mean): 0.05 − CV(SD): 0.11
Density
0.03
0.02
0.01
0.00
0
30
60
90
120
Fluorescence (a.u.)
Systematic Name: YOR074C
Time step Standard Deviation
16
14
12
40
42
44
46
Time step Mean
1
2
3
4
5
6
7
8
9
10
11
12
13
14
15
16
17
18
Cluster
(Isolation)
1 (−)
2 (−)
Time Step
15
10
5

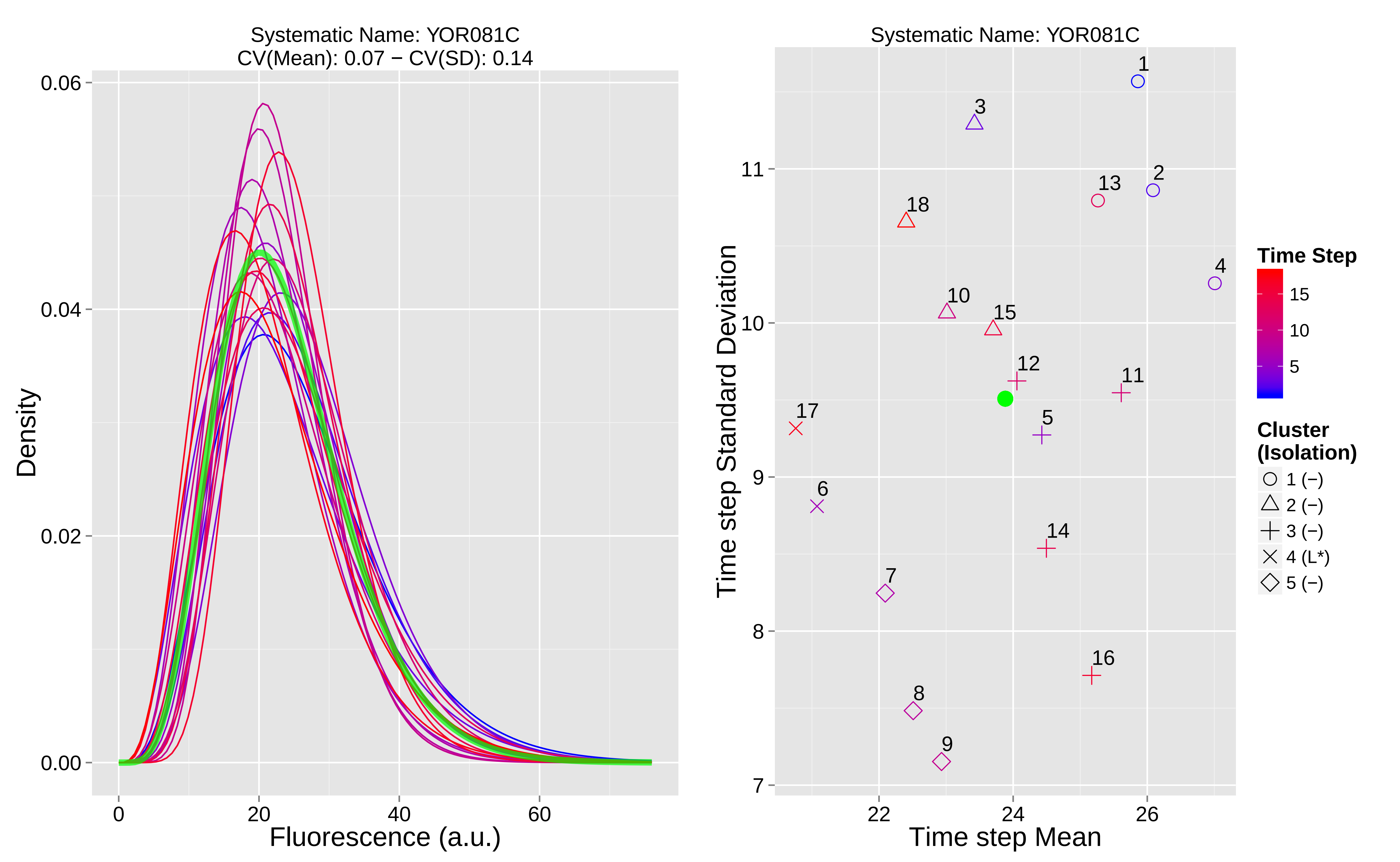
Systematic Name: YOR081C
CV(Mean): 0.07 − CV(SD): 0.14
Density
Fluorescence (a.u.)
0.06
0.04
0.02
0.00
0
20
40
60
Systematic Name: YOR081C
Time step Standard Deviation
Time step Mean
11
10
9
8
7
22
24
26
1
2
3
4
5
6
7
8
9
10
11
12
13
14
15
16
17
18
Time Step
15
10
5
Cluster (Isolation)
1 (−)
2 (−)
3 (−)
4 (L*)
5 (−)

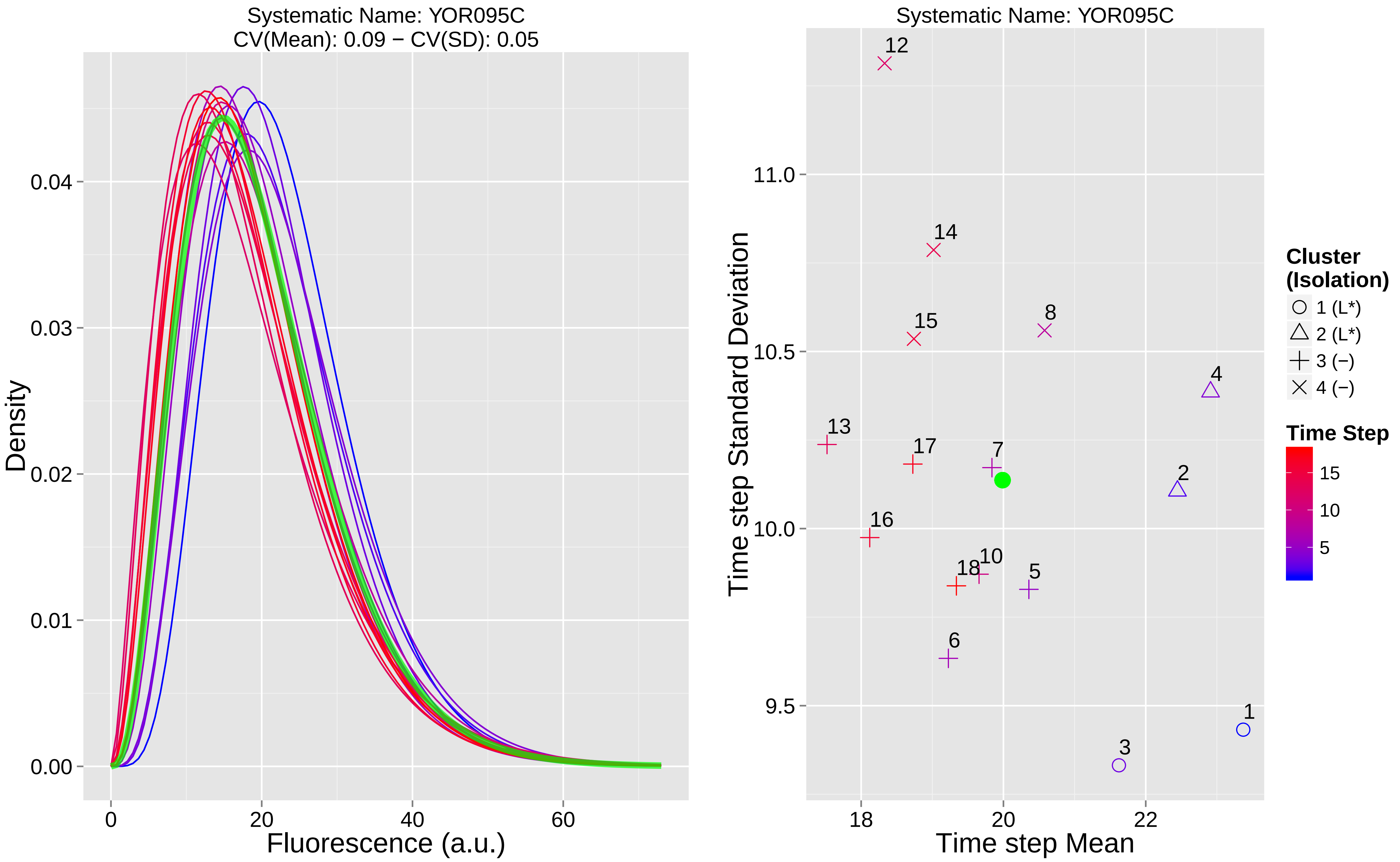

Systematic Name: YOR095C
CV(Mean): 0.09 − CV(SD): 0.05
Density
0.00
0.01
0.02
0.03
0.04
0
20
40
60
Fluorescence (a.u.)
Systematic Name: YOR095C
Time step Standard Deviation
9.5
10.0
10.5
11.0
18
20
22
Time step Mean
12
14
15
8
4
13
17
7
2
16
18
10
5
6
1
3
Cluster (Isolation)
1 (L*)
2 (L*)
3 (−)
4 (−)
Time Step
15
10
5

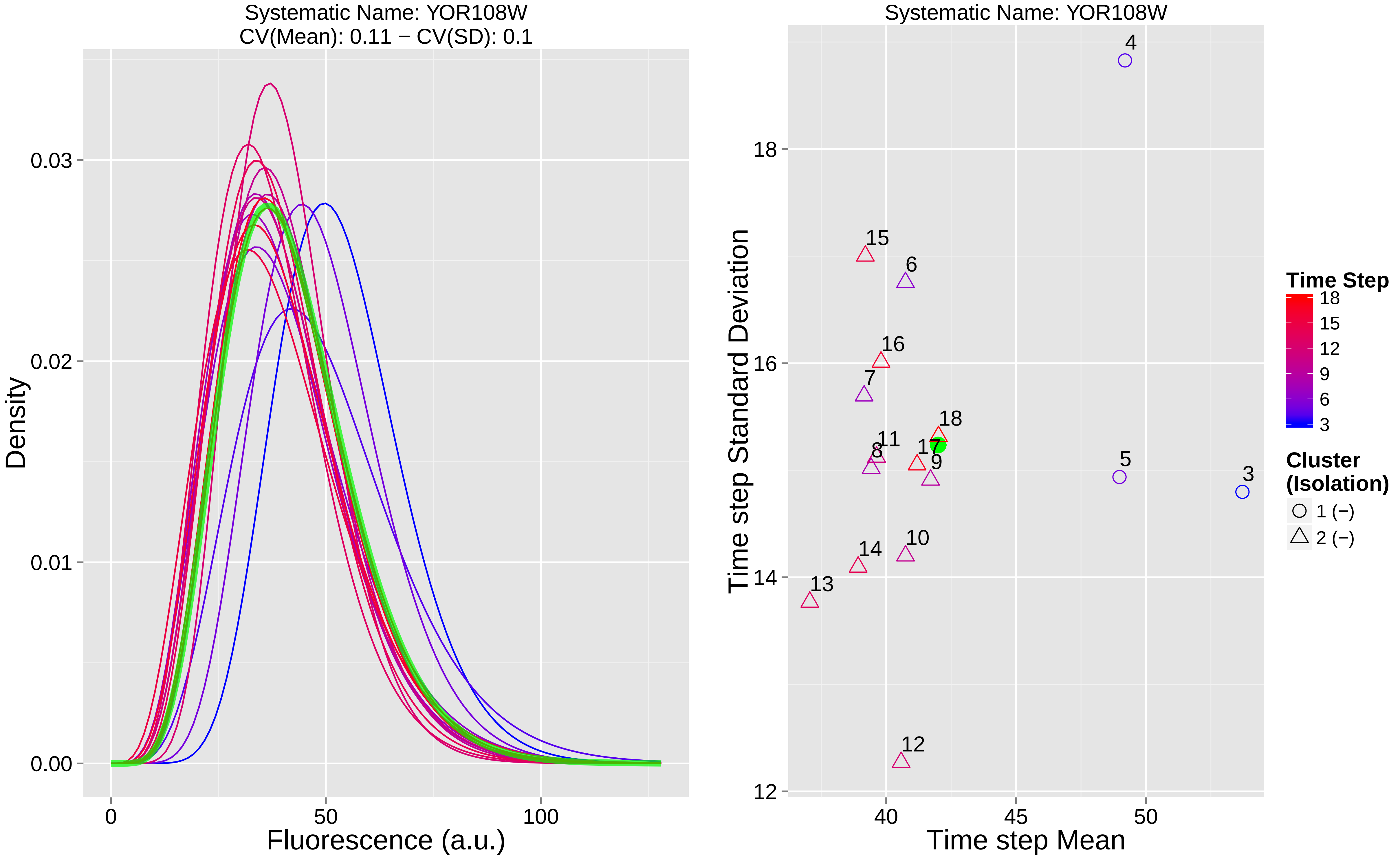

Systematic Name: YOR108W
CV(Mean): 0.11 − CV(SD): 0.1
Density
0.03
0.02
0.01
0.00
0
50
100
Fluorescence (a.u.)
Systematic Name: YOR108W
Time step Standard Deviation
18
16
14
12
40
45
50
Time step Mean
4
15
6
16
7
18
11
8
17
9
5
3
10
14
13
12
Time Step
18
15
12
9
6
3
Cluster (Isolation)
1 (−)
2 (−)

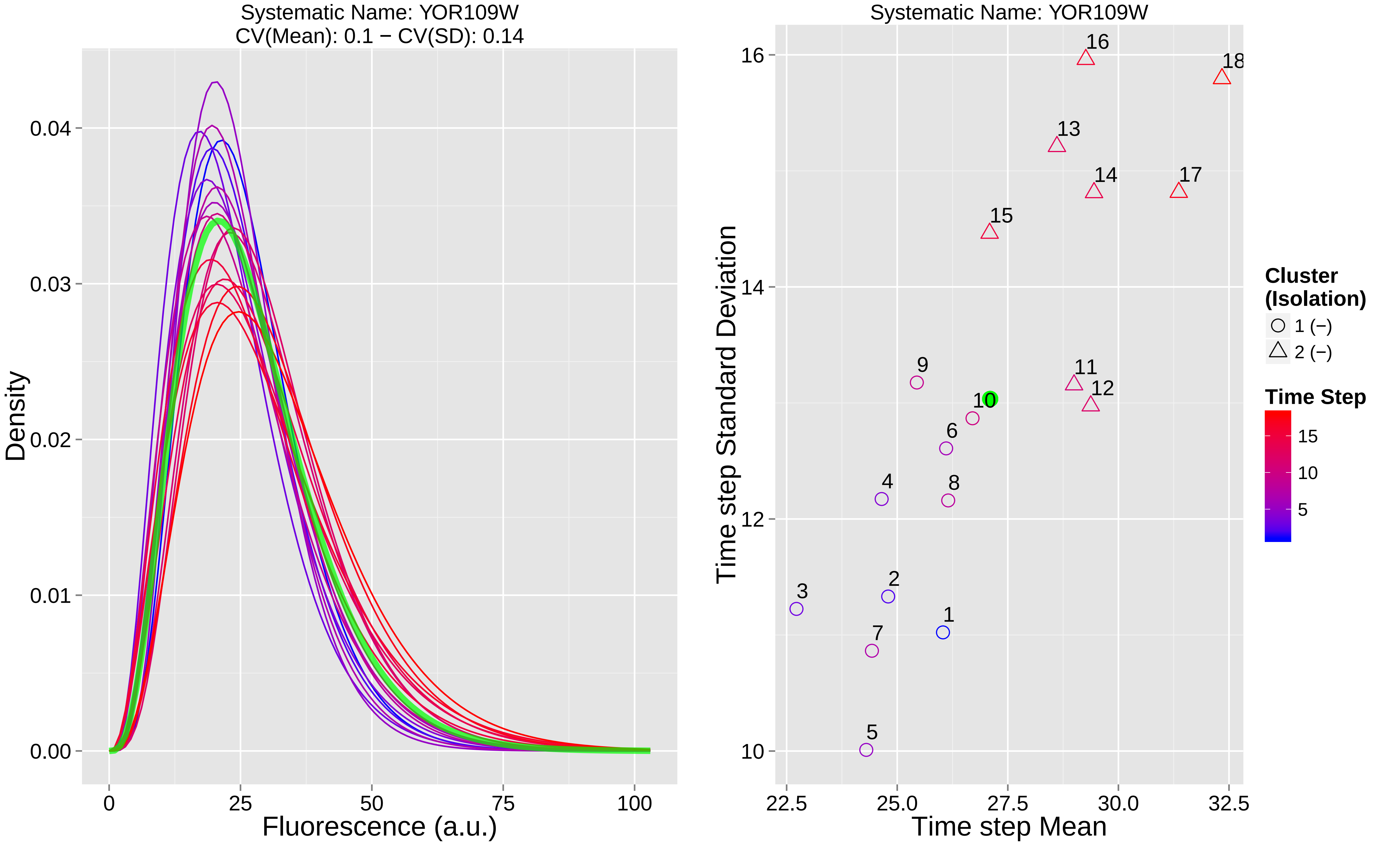

Systematic Name: YOR109W
CV(Mean): 0.1 − CV(SD): 0.14
Density
0.00
0.01
0.02
0.03
0.04
0
25
50
75
100
Fluorescence (a.u.)
Systematic Name: YOR109W
Time step Standard Deviation
10
12
14
16
22.5
25.0
27.5
30.0
32.5
Time step Mean
Cluster
(Isolation)
1 (−)
2 (−)
Time Step
5
10
15

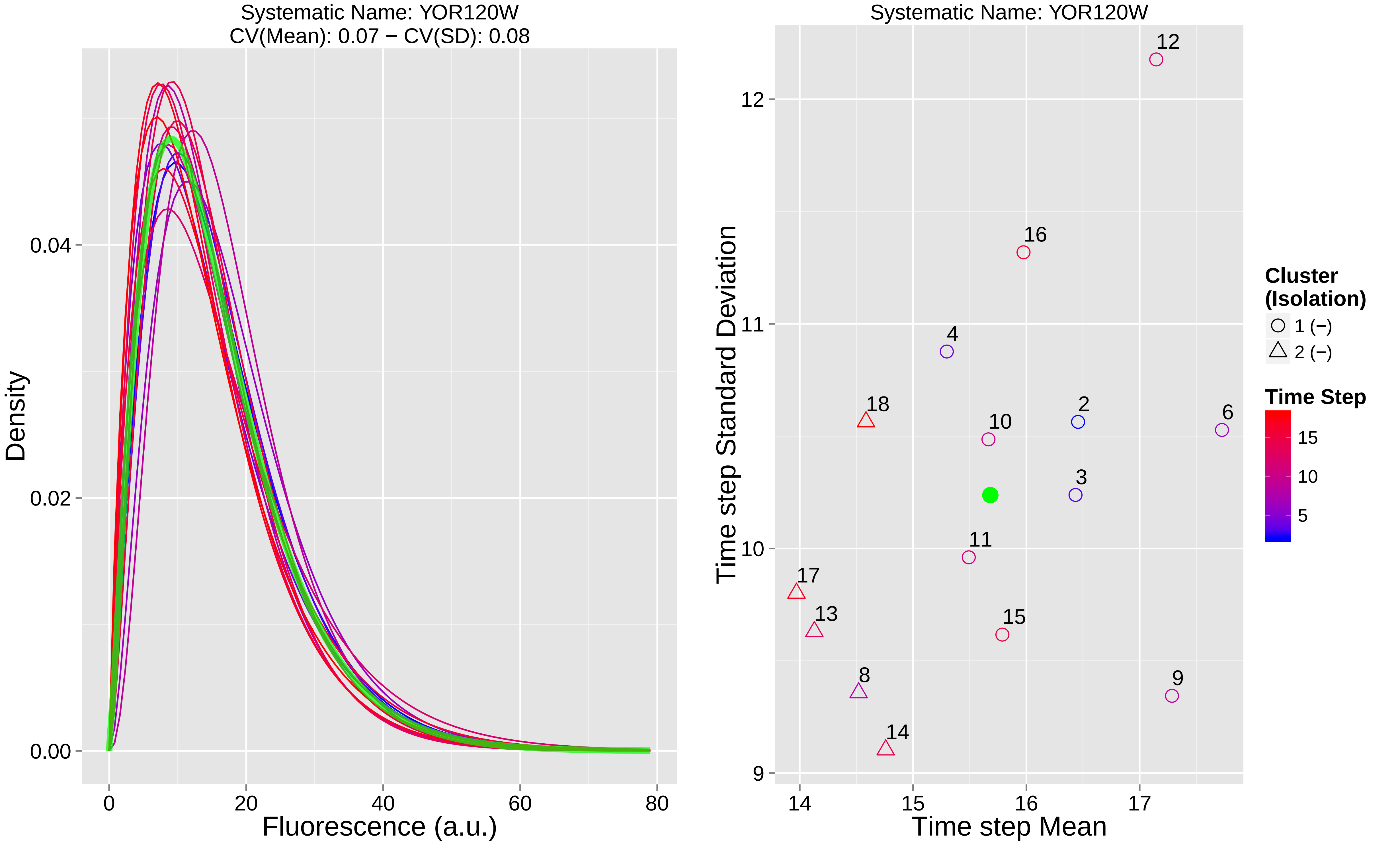
Systematic Name: YOR120W
CV(Mean): 0.07 − CV(SD): 0.08
Density
0.00
0.02
0.04
0
20
40
60
80
Fluorescence (a.u.)
Systematic Name: YOR120W
Time step Standard Deviation
9
10
11
12
14
15
16
17
Time step Mean
12
16
4
18
10
2
6
3
11
17
13
15
8
9
14
Cluster
(Isolation)
1 (−)
2 (−)
Time Step
15
10
5

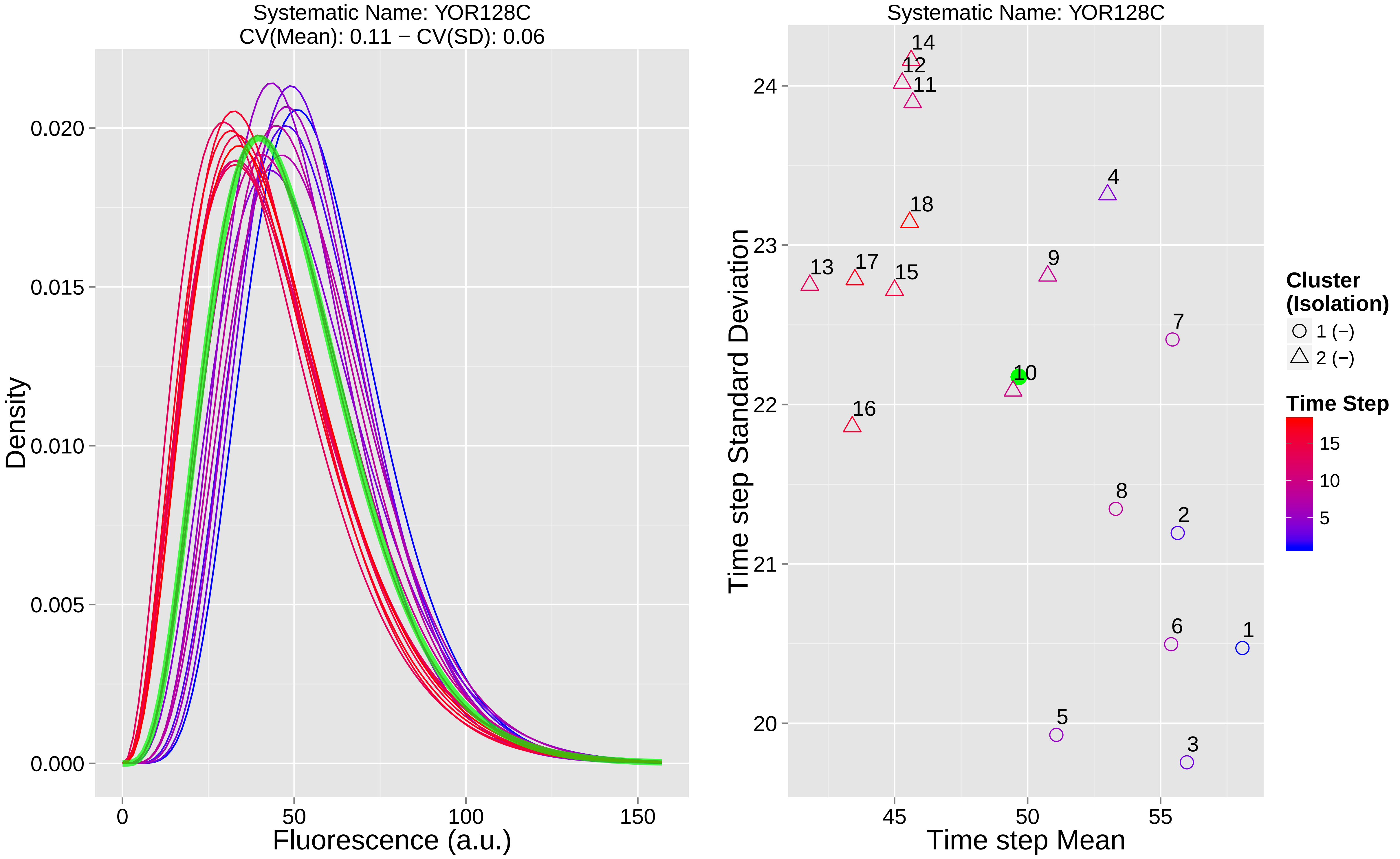

Systematic Name: YOR128C
CV(Mean): 0.11 − CV(SD): 0.06
Density
Fluorescence (a.u.)
0.000
0.005
0.010
0.015
0.020
0
50
100
150
Systematic Name: YOR128C
Time step Standard Deviation
Time step Mean
20
21
22
23
24
45
50
55
Cluster
(Isolation)
1 (−)
2 (−)
Time Step
15
10
5

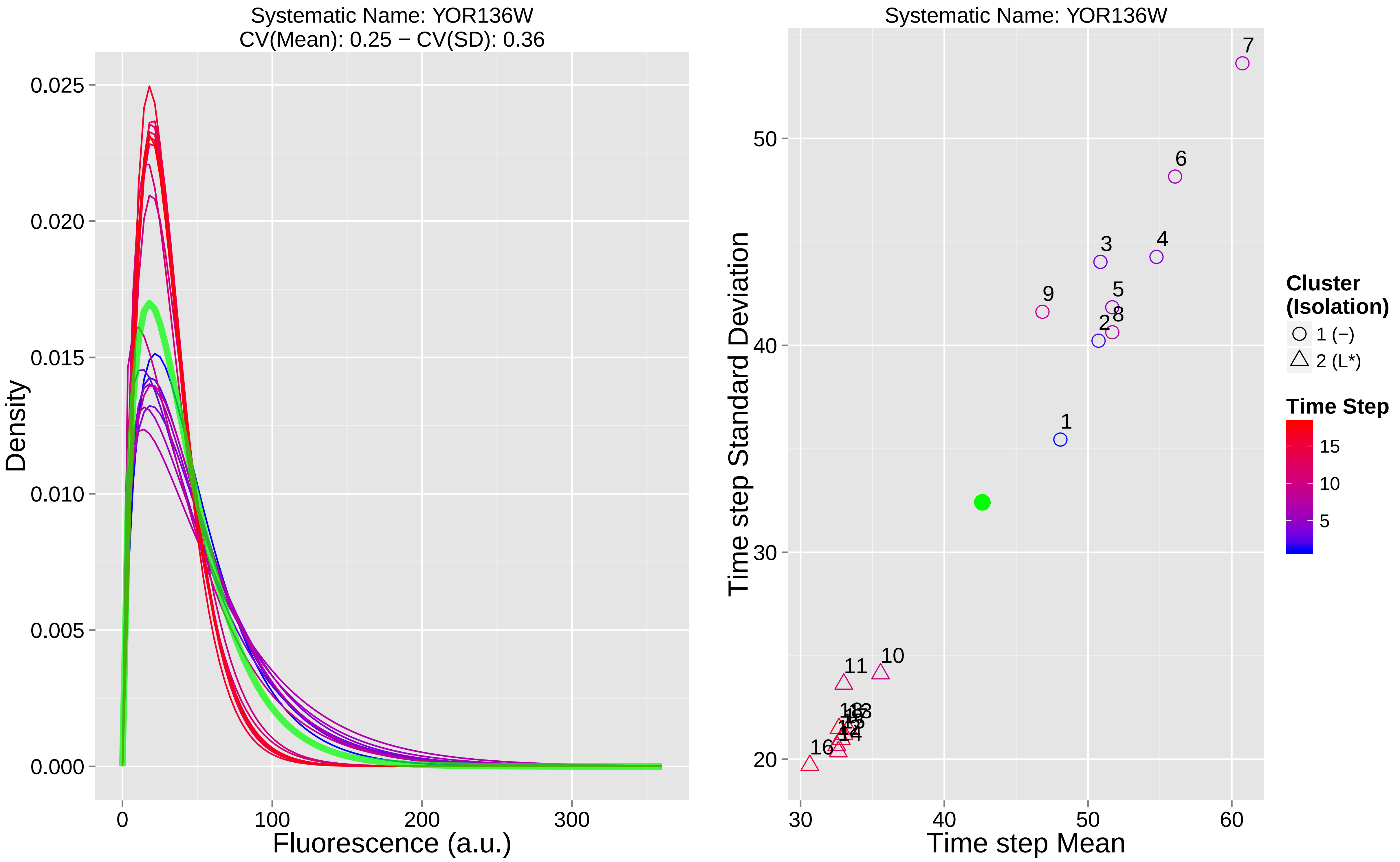

Systematic Name: YOR136W
CV(Mean): 0.25 − CV(SD): 0.36
Density
0.025
0.020
0.015
0.010
0.005
0.000
0
100
200
300
Fluorescence (a.u.)
Systematic Name: YOR136W
Time step Standard Deviation
50
40
30
20
Time step Mean
30
40
50
60
7
6
3
4
9
5
2
8
1
10
11
16
Cluster
(Isolation)
1 (−)
2 (L*)
Time Step
15
10
5

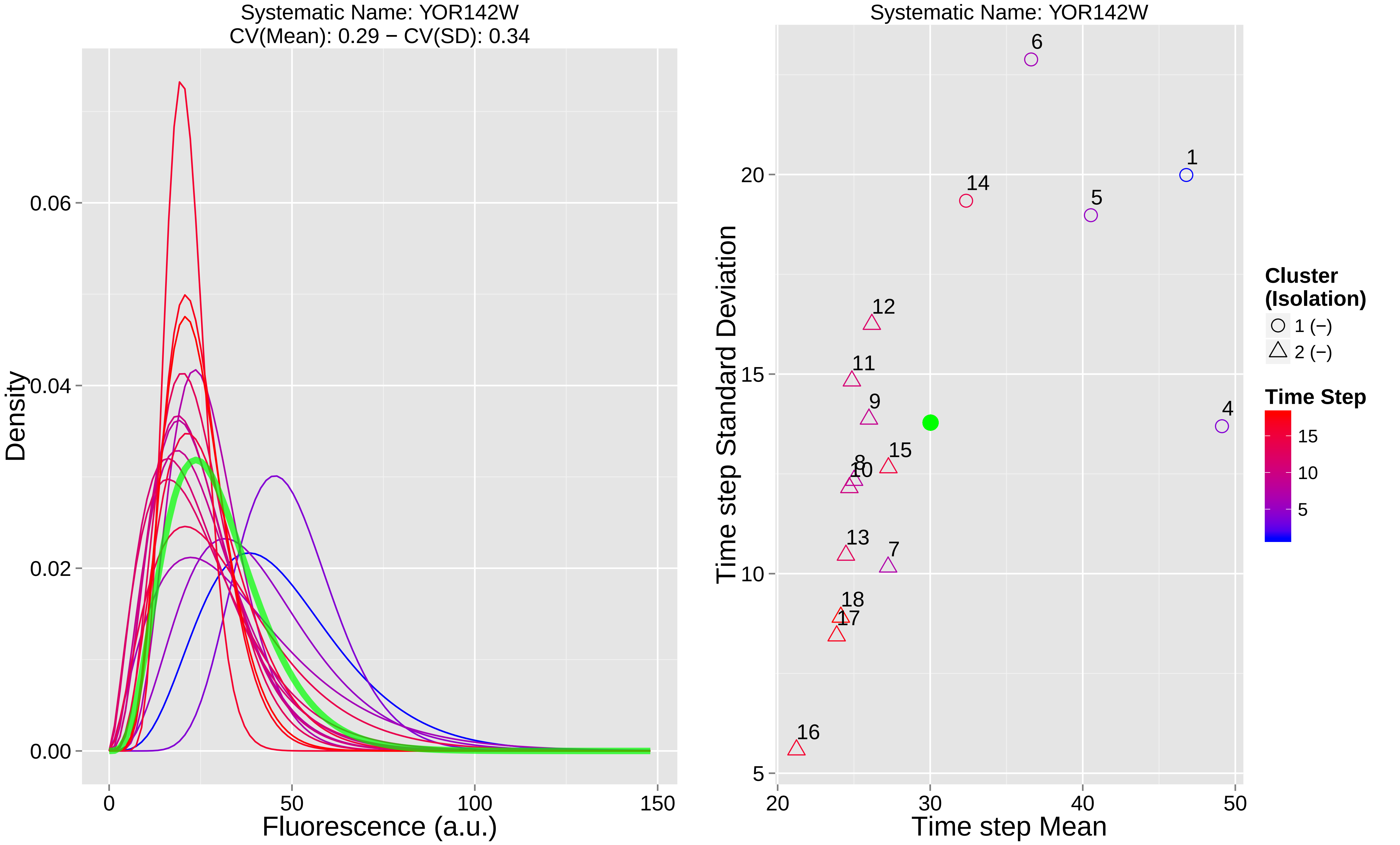

Systematic Name: YOR142W
CV(Mean): 0.29 − CV(SD): 0.34
Density
0.06
0.04
0.02
0.00
0
50
100
150
Fluorescence (a.u.)
Systematic Name: YOR142W
Time step Standard Deviation
20
15
10
5
20
30
40
50
Time step Mean
6
1
14
5
12
11
9
4
15
8
10
13
7
18
17
16
Cluster
(Isolation)
1 (−)
2 (−)
Time Step
15
10
5

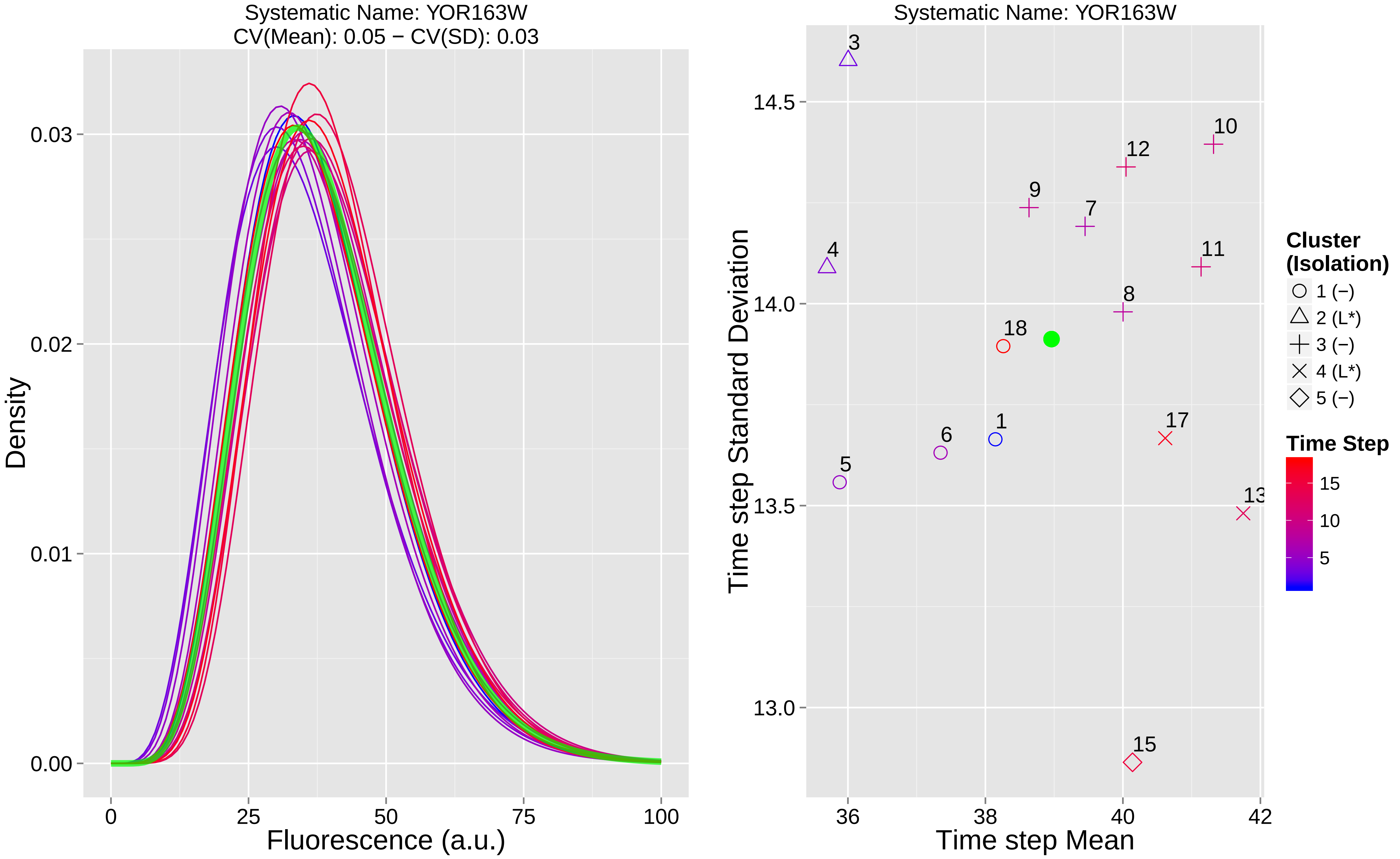

Systematic Name: YOR163W
CV(Mean): 0.05 − CV(SD): 0.03
Density
Fluorescence (a.u.)
0.00
0.01
0.02
0.03
0
25
50
75
100
Systematic Name: YOR163W
Time step Standard Deviation
Time step Mean
13.0
13.5
14.0
14.5
36
38
40
42
1
3
4
5
6
7
8
9
10
11
12
13
15
17
18
Cluster (Isolation)
1 (−)
2 (L*)
3 (−)
4 (L*)
5 (−)
Time Step
5
10
15

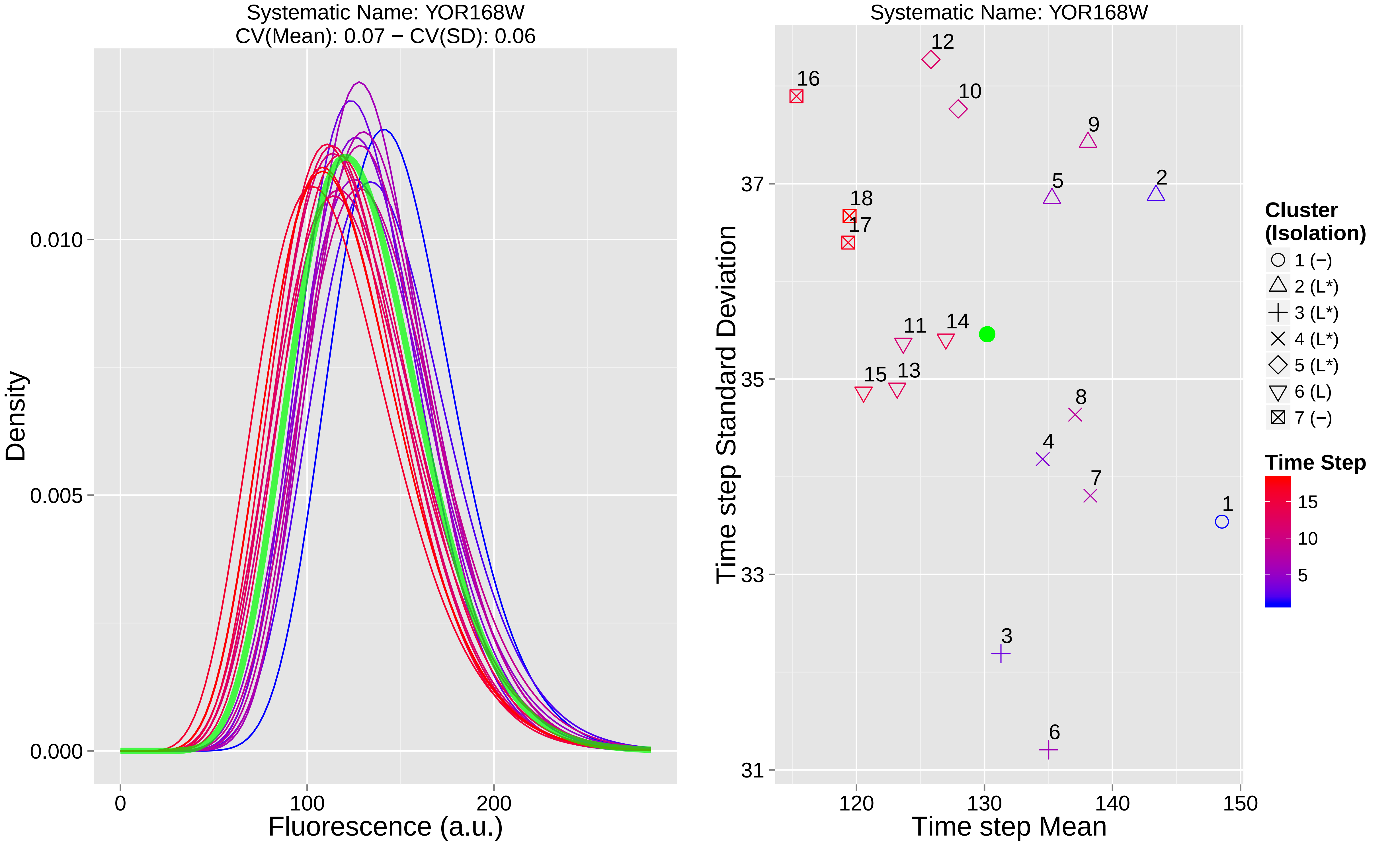

Systematic Name: YOR168W
CV(Mean): 0.07 − CV(SD): 0.06
Density
0.000
0.005
0.010
0
100
200
Fluorescence (a.u.)
Systematic Name: YOR168W
Time step Standard Deviation
Time step Mean
31
33
35
37
120
130
140
150
Cluster (Isolation)
1 (−)
2 (L*)
3 (L*)
4 (L*)
5 (L*)
6 (L)
7 (−)
Time Step
5
10
15

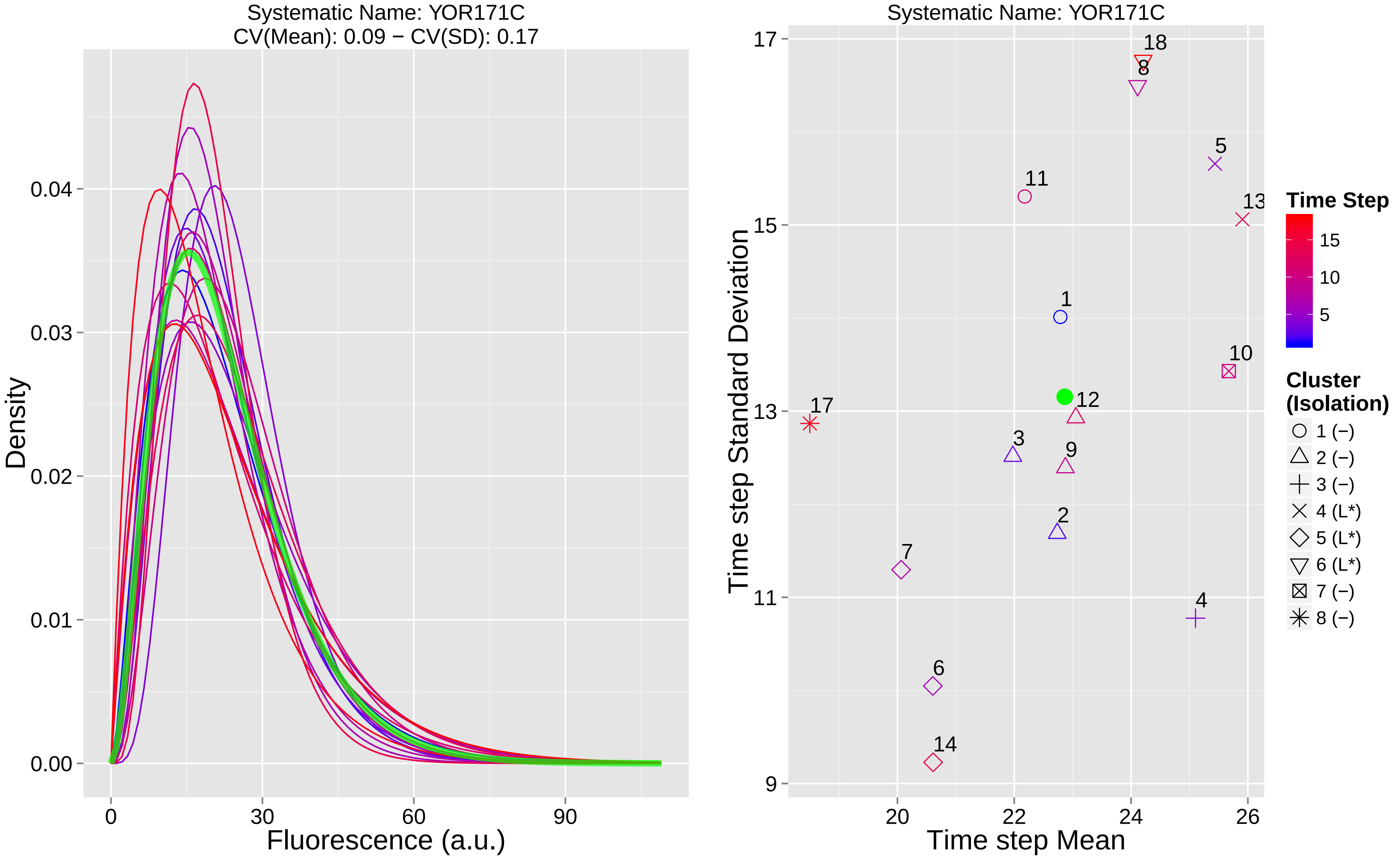
Systematic Name: YOR171C
CV(Mean): 0.09 − CV(SD): 0.17
Density
Fluorescence (a.u.)
0.00
0.01
0.02
0.03
0.04
0
30
60
90
Systematic Name: YOR171C
Time step Standard Deviation
Time step Mean
9
11
13
15
17
20
22
24
26
1
2
3
4
5
6
7
8
9
10
11
12
13
14
17
18
Time Step
15
10
5
Cluster (Isolation)
1 (−)
2 (−)
3 (−)
4 (L*)
5 (L*)
6 (L*)
7 (−)
8 (−)

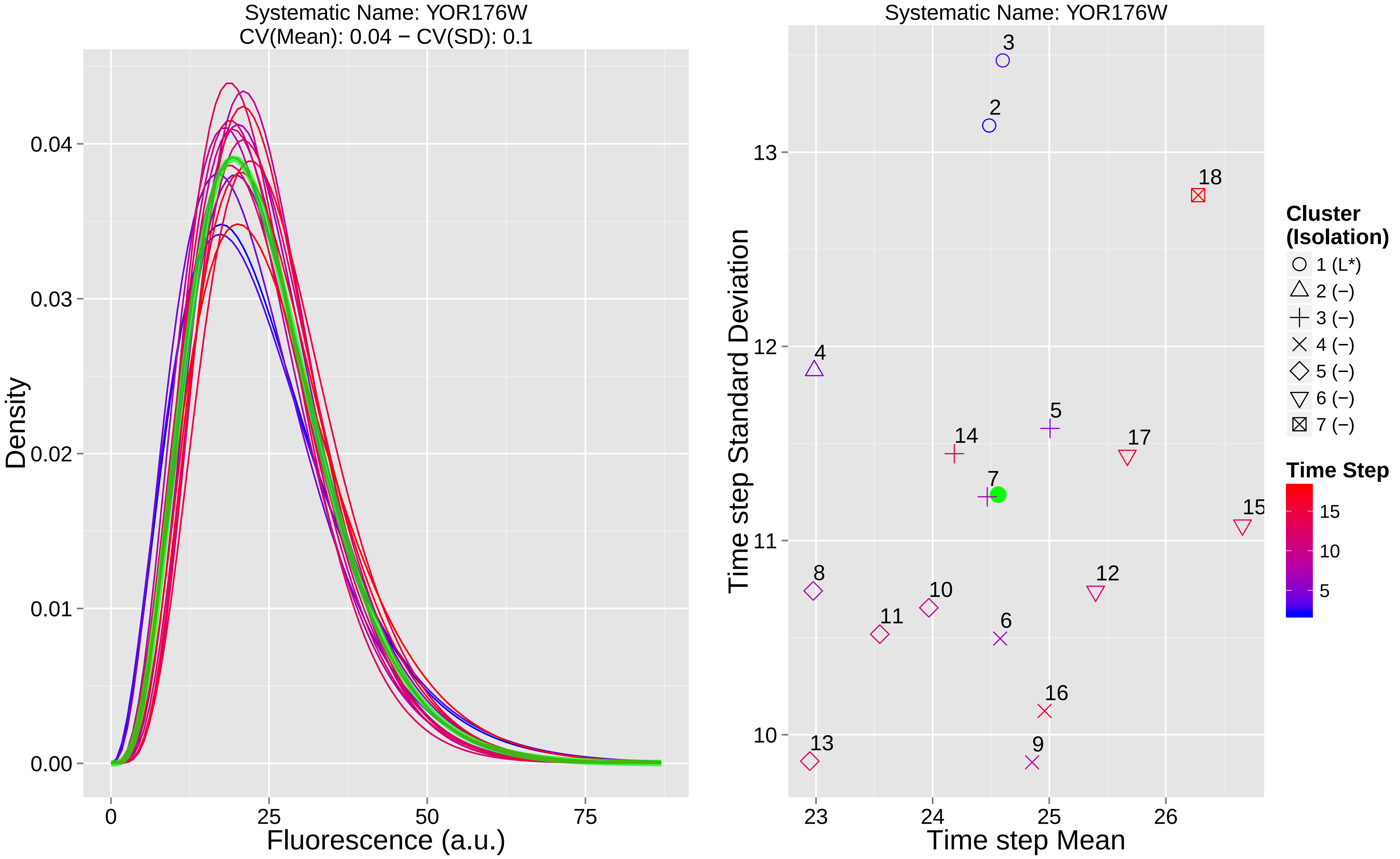

Systematic Name: YOR176W
CV(Mean): 0.04 − CV(SD): 0.1
Density
Fluorescence (a.u.)
0.00
0.01
0.02
0.03
0.04
0
25
50
75
Systematic Name: YOR176W
Time step Standard Deviation
Time step Mean
10
11
12
13
23
24
25
26
Cluster (Isolation)
1 (L*)
2 (−)
3 (−)
4 (−)
5 (−)
6 (−)
7 (−)
Time Step
5
10
15

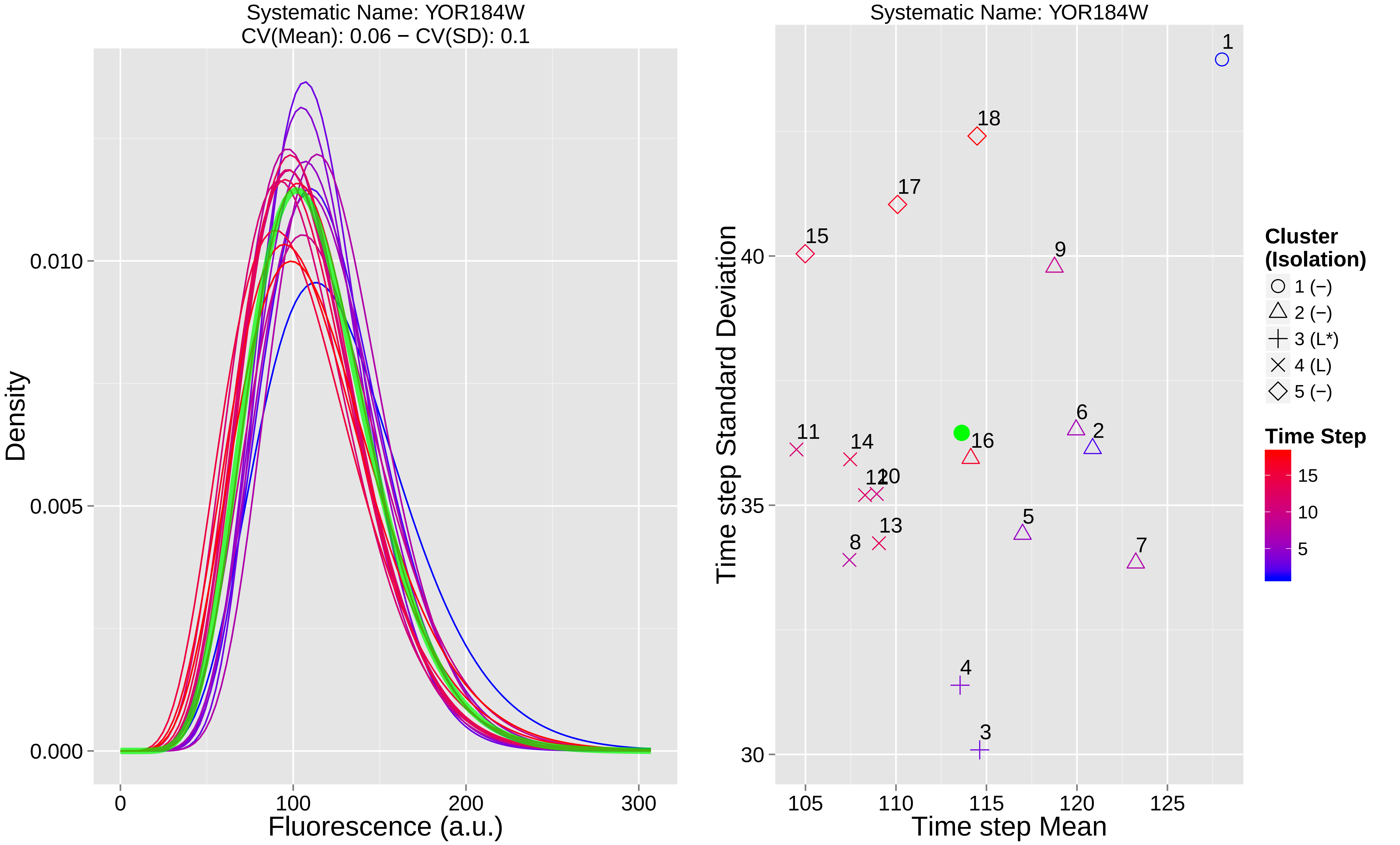
Systematic Name: YOR184W
CV(Mean): 0.06 − CV(SD): 0.1
Density
Fluorescence (a.u.)
0.000
0.005
0.010
0
100
200
300
Systematic Name: YOR184W
Time step Standard Deviation
Time step Mean
30
35
40
105
110
115
120
125
Cluster (Isolation)
1 (−)
2 (−)
3 (L*)
4 (L)
5 (−)
Time Step
5
10
15

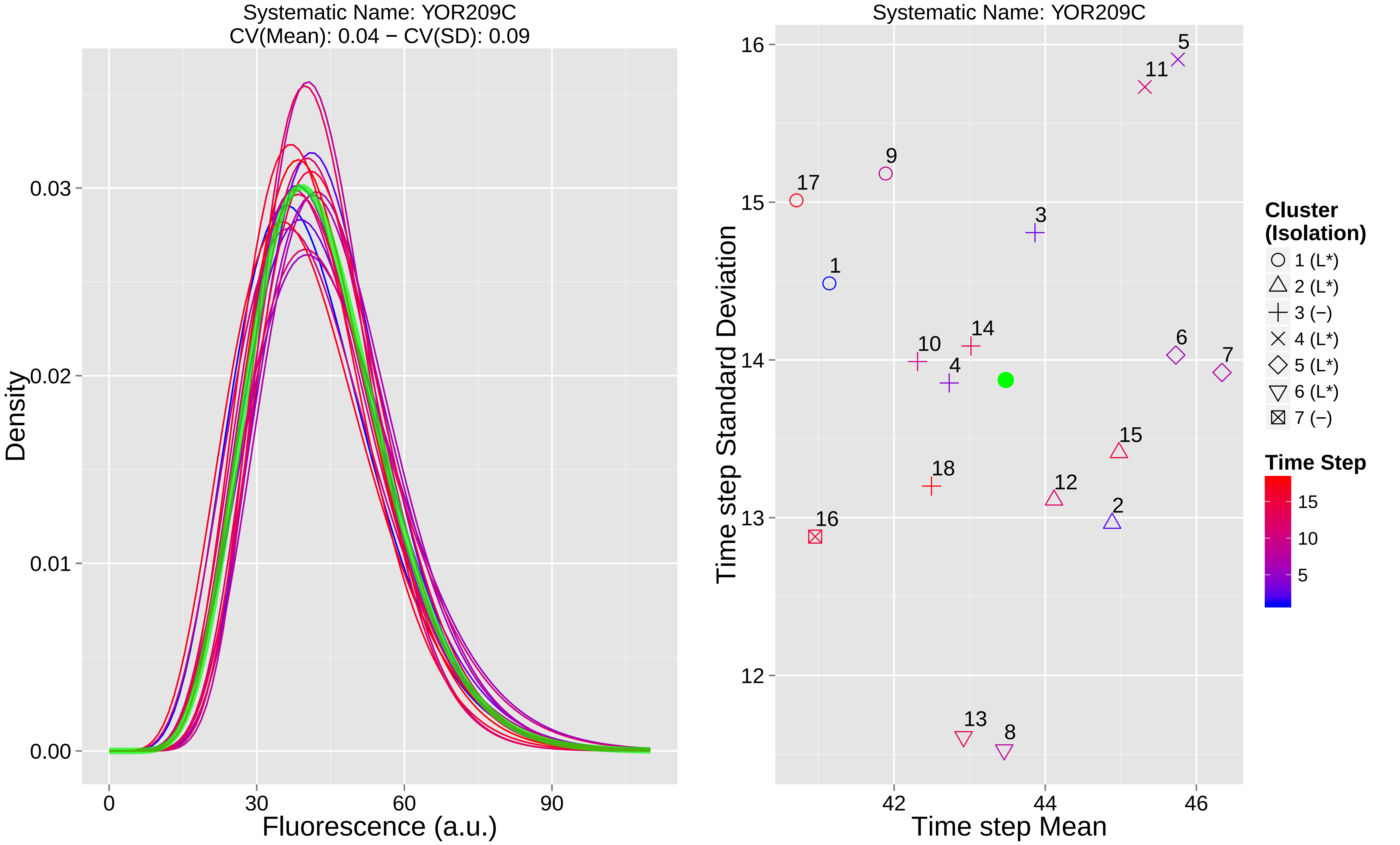

Systematic Name: YOR209C
CV(Mean): 0.04 − CV(SD): 0.09
Density
0.00
0.01
0.02
0.03
0
30
60
90
Fluorescence (a.u.)
Systematic Name: YOR209C
Time step Standard Deviation
12
13
14
15
16
42
44
46
Time step Mean
1
2
3
4
5
6
7
8
9
10
11
12
13
14
15
16
17
18
Cluster (Isolation)
1 (L*)
2 (L*)
3 (−)
4 (L*)
5 (L*)
6 (L*)
7 (−)
Time Step
5
10
15

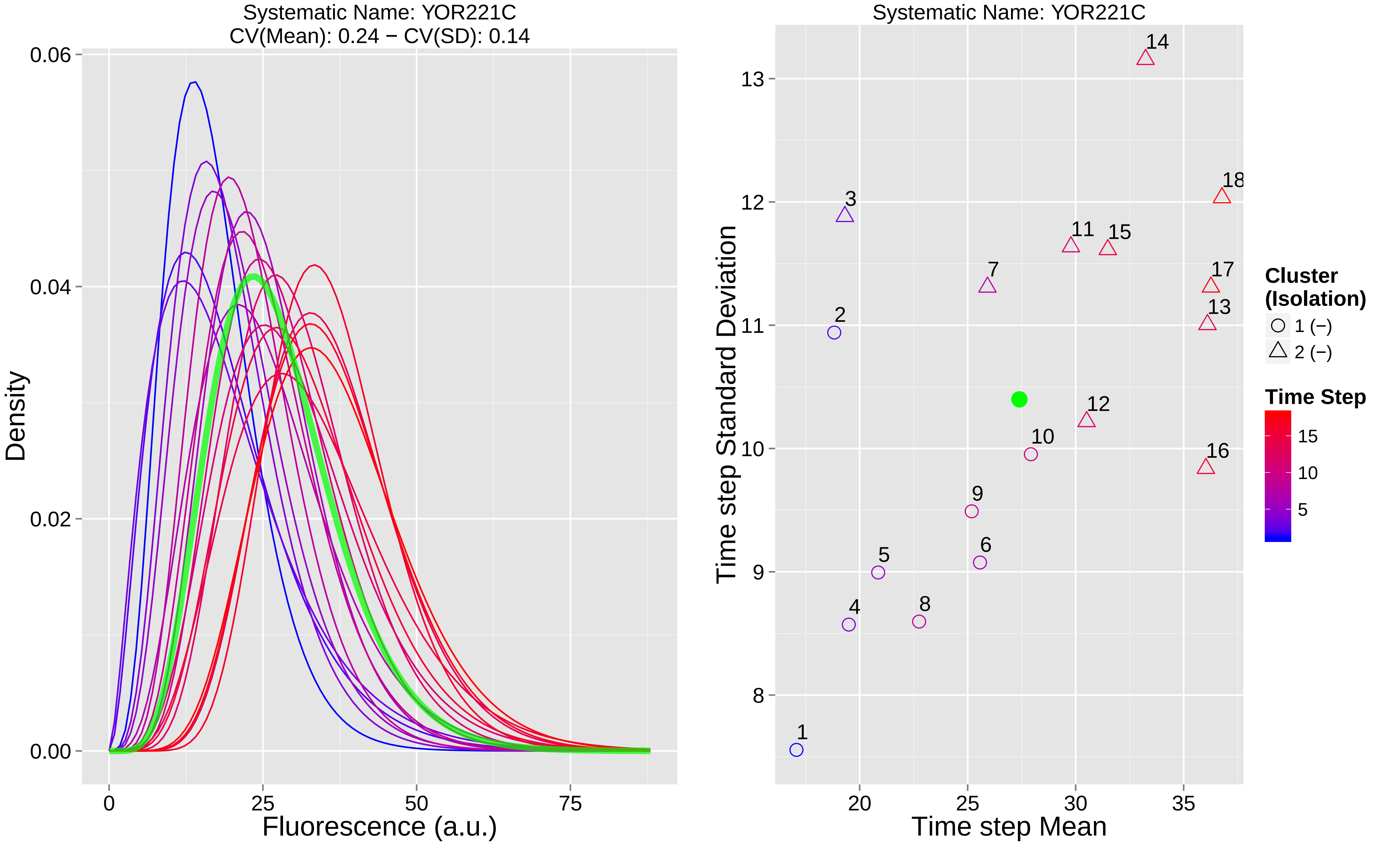

Systematic Name: YOR221C
CV(Mean): 0.24 − CV(SD): 0.14
Density
Fluorescence (a.u.)
0.06
0.04
0.02
0.00
0
25
50
75
Systematic Name: YOR221C
Time step Standard Deviation
Time step Mean
13
12
11
10
9
8
20
25
30
35
Cluster (Isolation)
1 (−)
2 (−)
Time Step
15
10
5

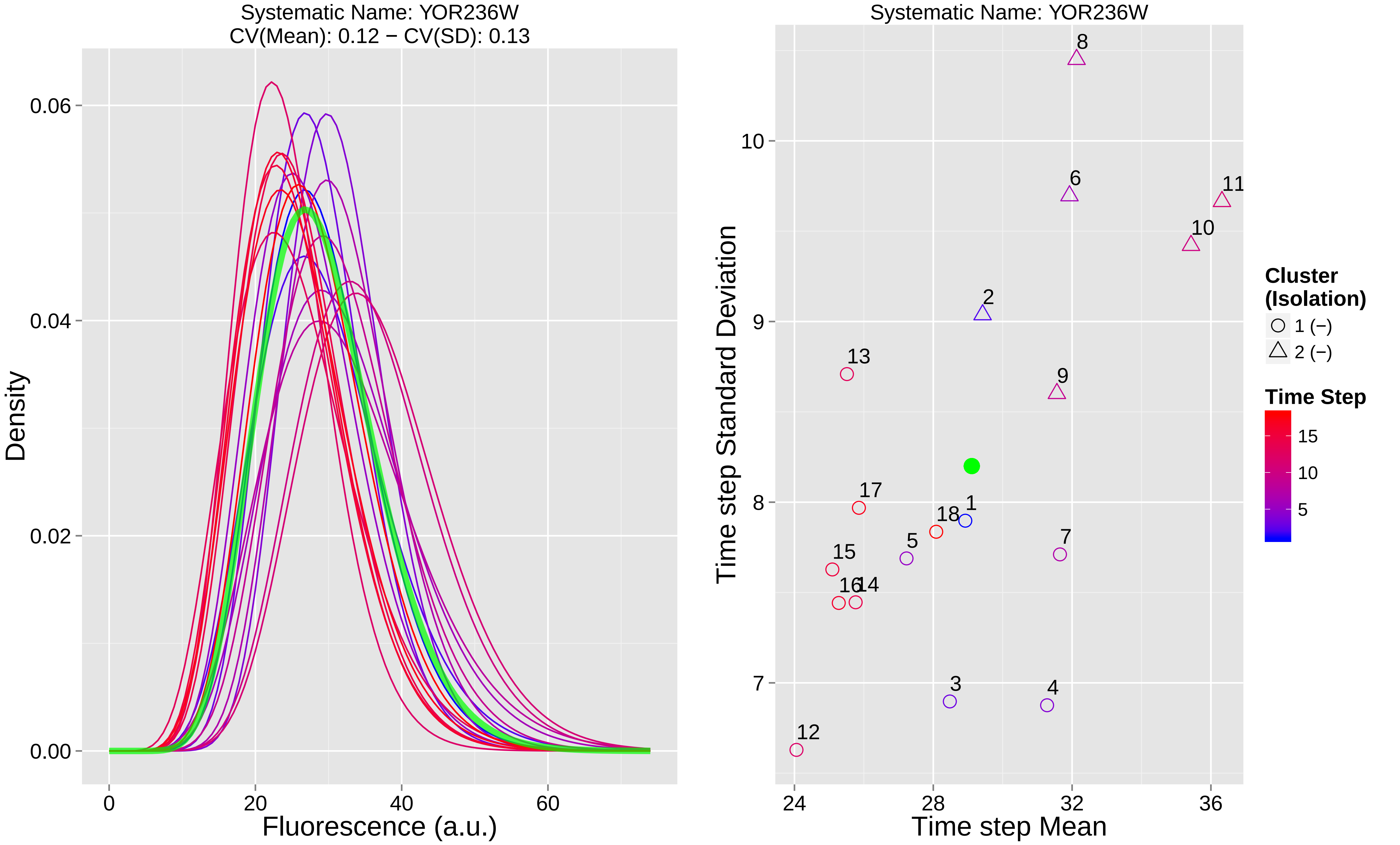
Systematic Name: YOR236W
CV(Mean): 0.12 − CV(SD): 0.13
Density
0.06
0.04
0.02
0.00
0
20
40
60
Fluorescence (a.u.)
Systematic Name: YOR236W
Time step Standard Deviation
10
9
8
7
24
28
32
36
Time step Mean
8
6
11
10
2
13
9
17
18
1
5
7
15
16
14
3
4
12
Cluster
(Isolation)
1 (−)
2 (−)
Time Step
15
10
5

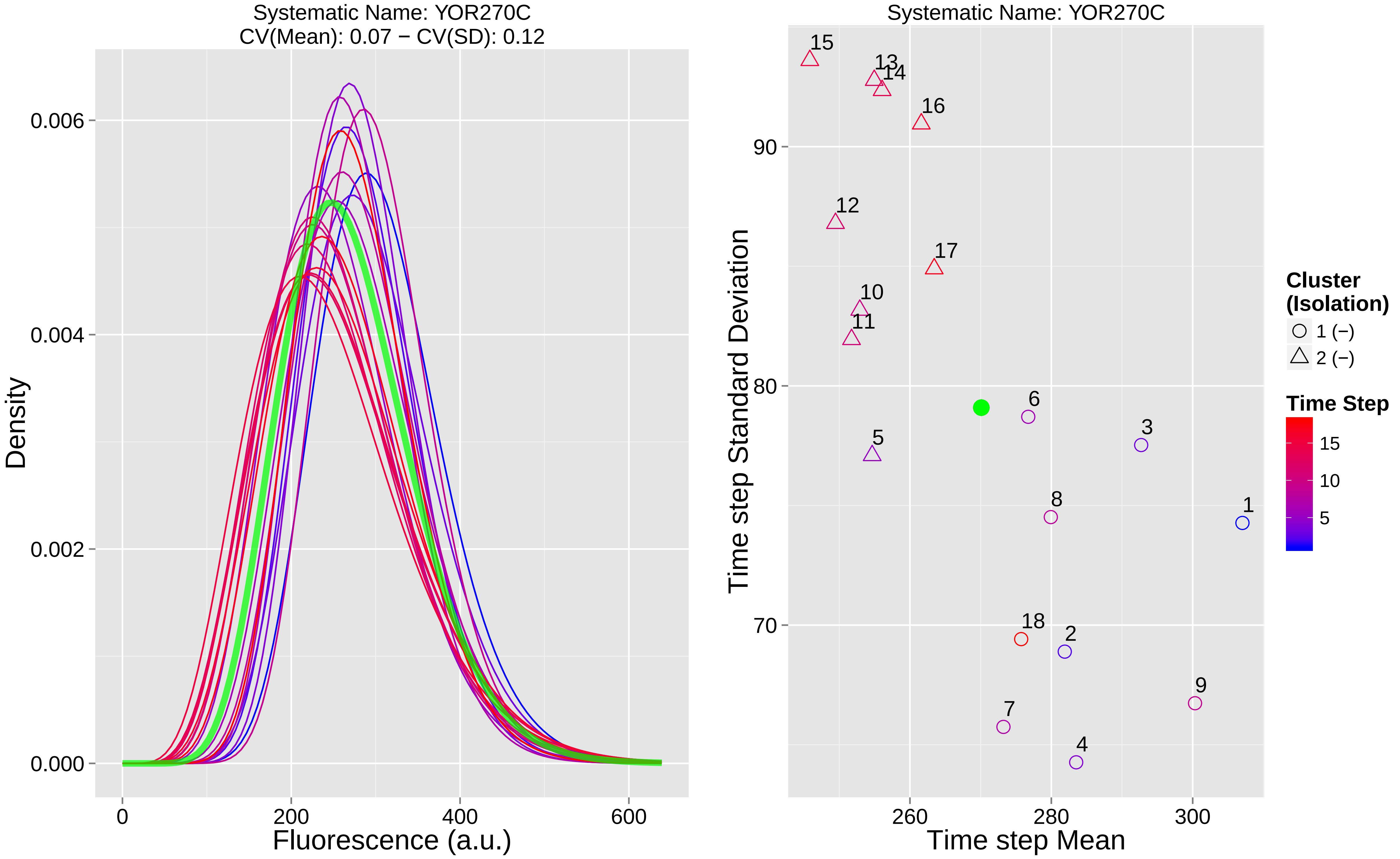
Systematic Name: YOR270C
CV(Mean): 0.07 − CV(SD): 0.12
Density
0.006
0.004
0.002
0.000
0
200
400
600
Fluorescence (a.u.)
Systematic Name: YOR270C
Time step Standard Deviation
90
80
70
260
280
300
Time step Mean
15
13
14
16
12
17
10
11
6
5
3
8
1
18
2
9
7
4
Cluster
(Isolation)
1 (−)
2 (−)
Time Step
15
10
5

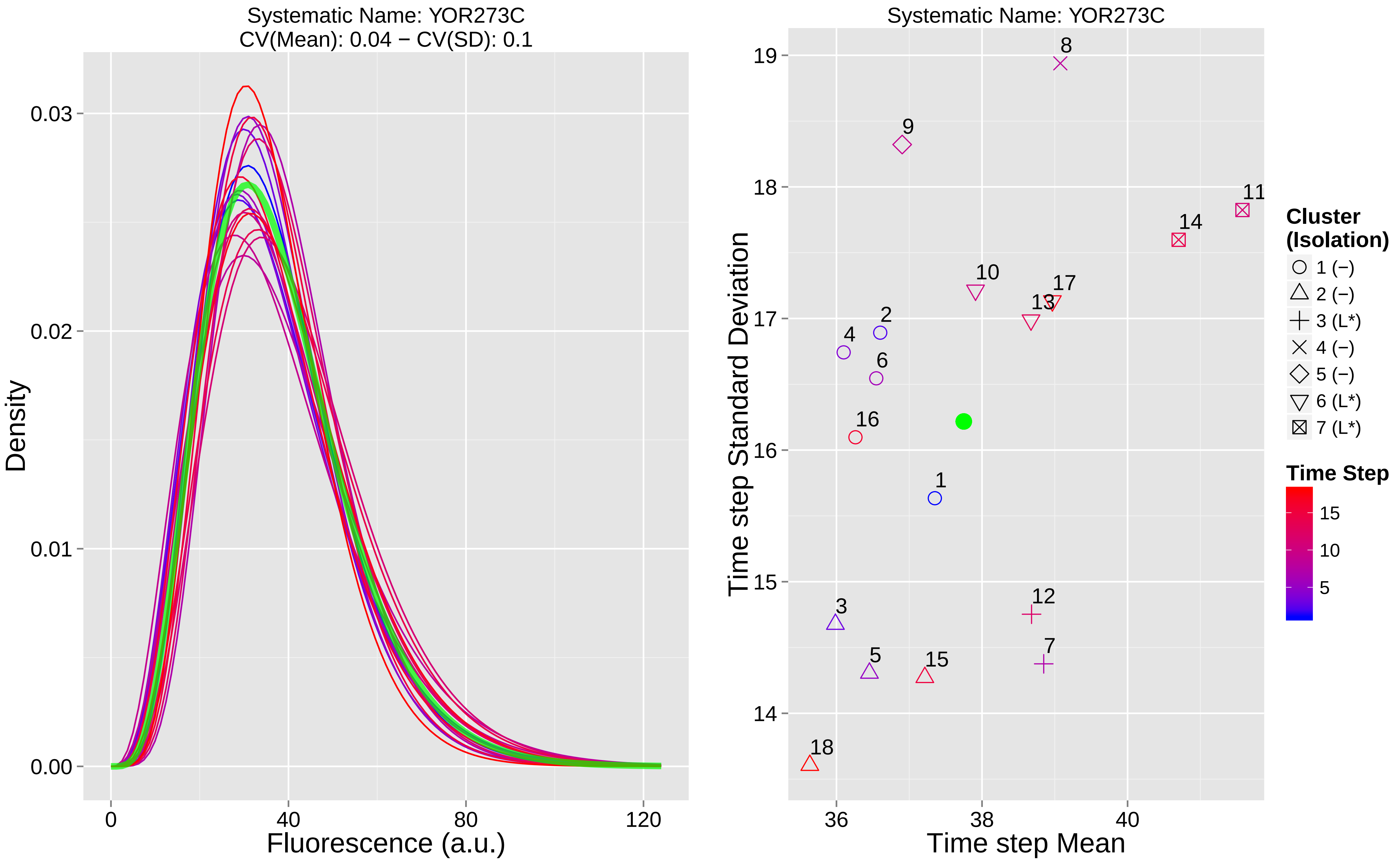

Systematic Name: YOR273C
CV(Mean): 0.04 − CV(SD): 0.1
Density
0.03
0.02
0.01
0.00
0
40
80
120
Fluorescence (a.u.)
Systematic Name: YOR273C
Time step Standard Deviation
19
18
17
16
15
14
36
38
40
Time step Mean
Cluster
(Isolation)
1 (−)
2 (−)
3 (L*)
4 (−)
5 (−)
6 (L*)
7 (L*)
Time Step
15
10
5

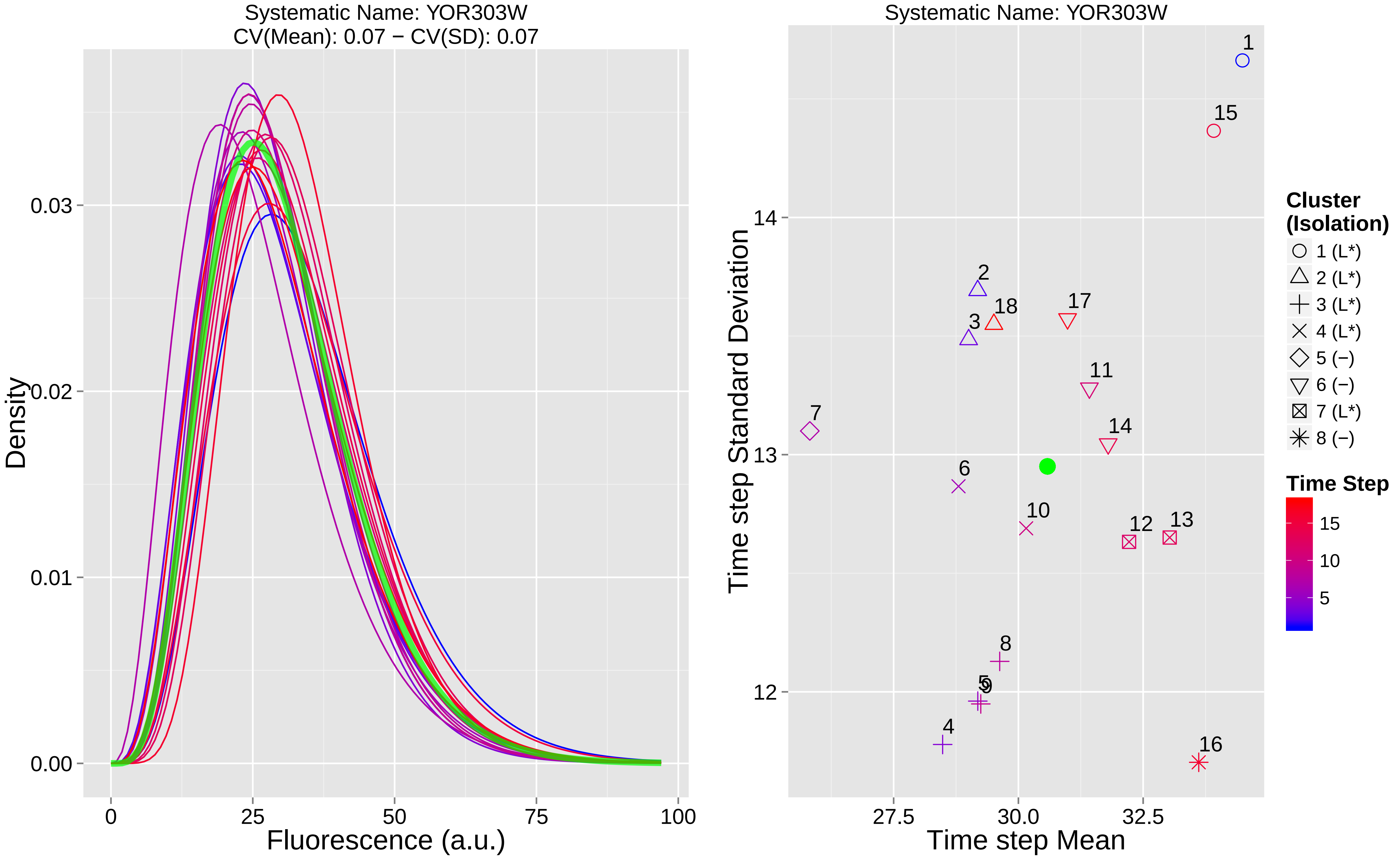
Systematic Name: YOR303W
CV(Mean): 0.07 − CV(SD): 0.07
Density
0.03
0.02
0.01
0.00
0
25
50
75
100
Fluorescence (a.u.)
Systematic Name: YOR303W
Time step Standard Deviation
14
13
12
27.5
30.0
32.5
Time step Mean
1
2
3
4
5
6
7
8
9
10
11
12
13
14
15
16
17
18
Cluster (Isolation)
1 (L*)
2 (L*)
3 (L*)
4 (L*)
5 (−)
6 (−)
7 (L*)
8 (−)
Time Step
15
10
5

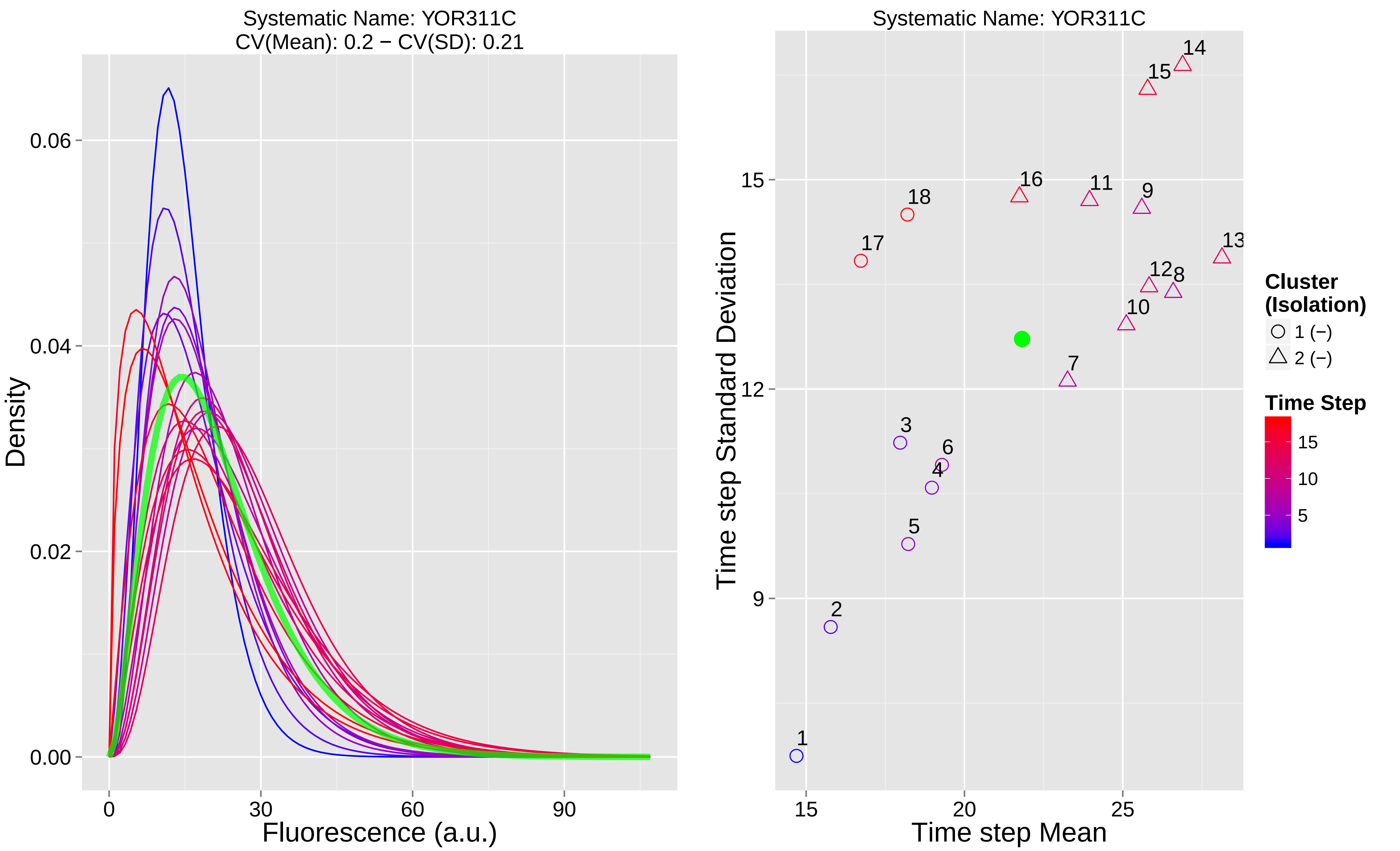
Systematic Name: YOR311C
CV(Mean): 0.2 − CV(SD): 0.21
Density
0.06
0.04
0.02
0.00
0
30
60
90
Fluorescence (a.u.)
Systematic Name: YOR311C
Time step Standard Deviation
15
12
9
15
20
25
Time step Mean
1
2
3
4
5
6
7
8
9
10
11
12
13
14
15
16
17
18
Cluster (Isolation)
1 (−)
2 (−)
Time Step
15
10
5

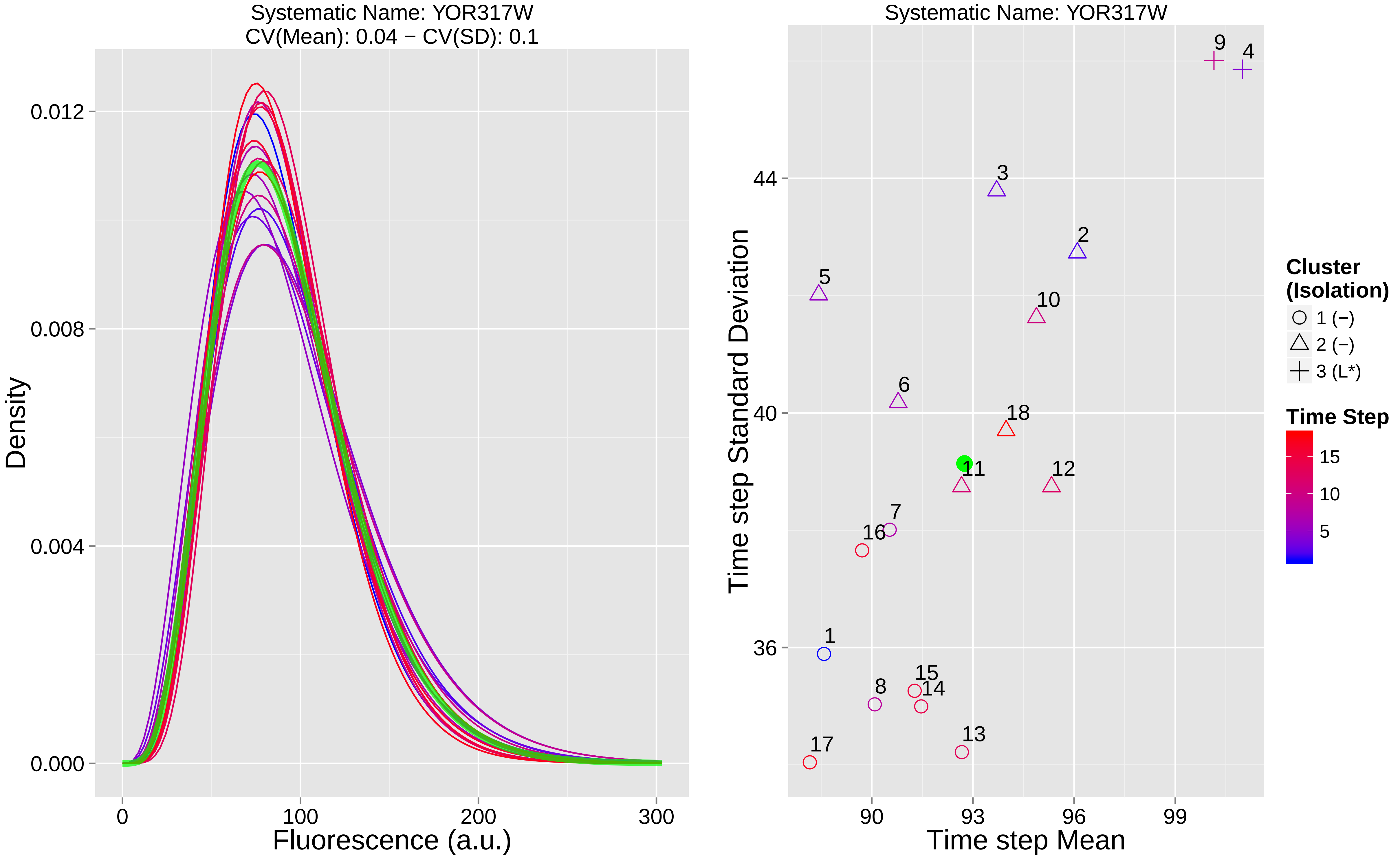

Systematic Name: YOR317W
CV(Mean): 0.04 − CV(SD): 0.1
Density
0.012
0.008
0.004
0.000
0
100
200
300
Fluorescence (a.u.)
Systematic Name: YOR317W
Time step Standard Deviation
44
40
36
90
93
96
99
Time step Mean
1
2
3
4
5
6
7
8
9
10
11
12
13
14
15
16
17
18
Cluster (Isolation)
1 (−)
2 (−)
3 (L*)
Time Step
15
10
5

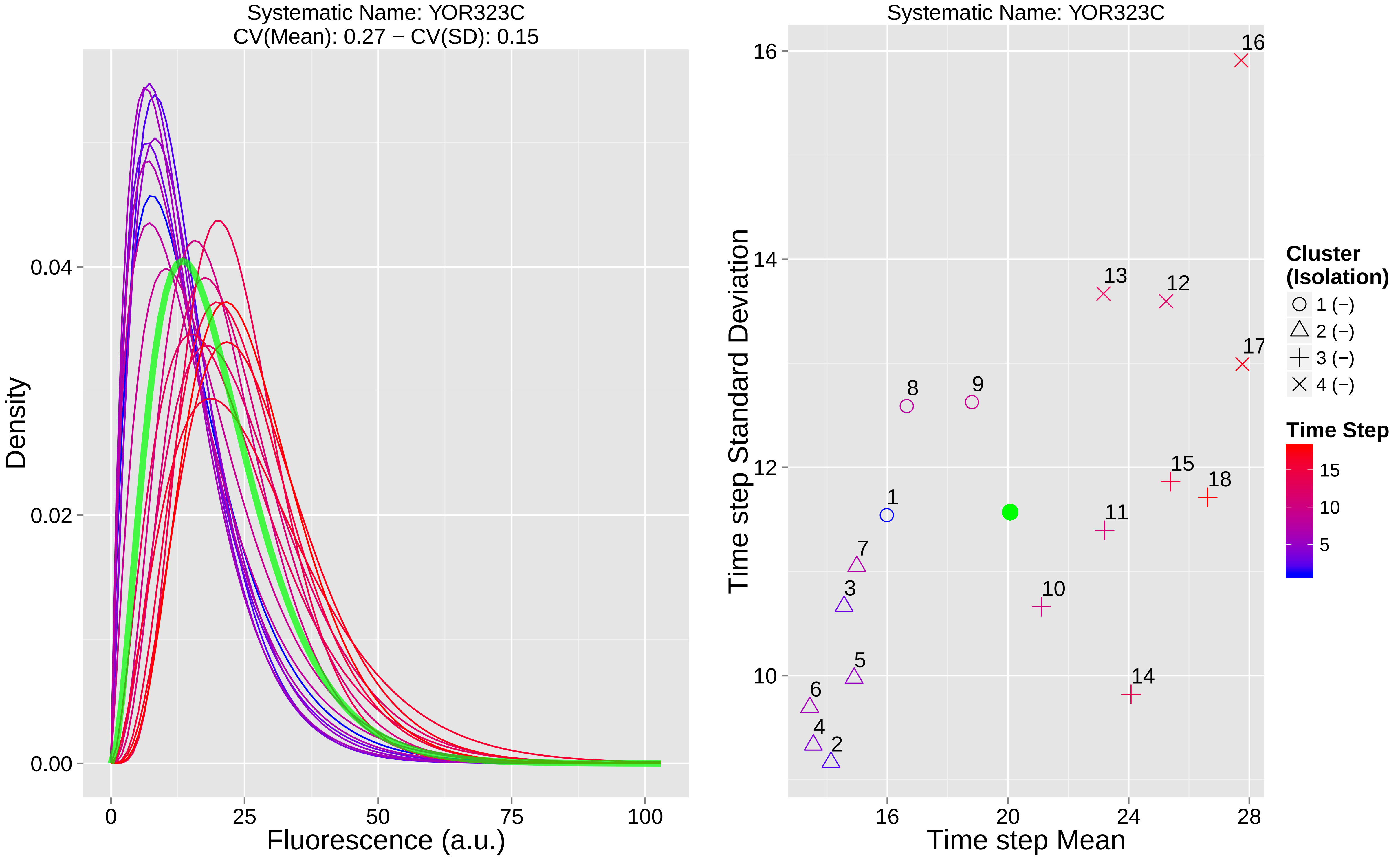

Systematic Name: YOR323C
CV(Mean): 0.27 − CV(SD): 0.15
Density
0.00
0.02
0.04
0
25
50
75
100
Fluorescence (a.u.)
Systematic Name: YOR323C
Time step Standard Deviation
10
12
14
16
16
20
24
28
Time step Mean
1
2
3
4
5
6
7
8
9
10
11
12
13
14
15
16
17
18
Cluster
(Isolation)
1 (−)
2 (−)
3 (−)
4 (−)
Time Step
15
10
5

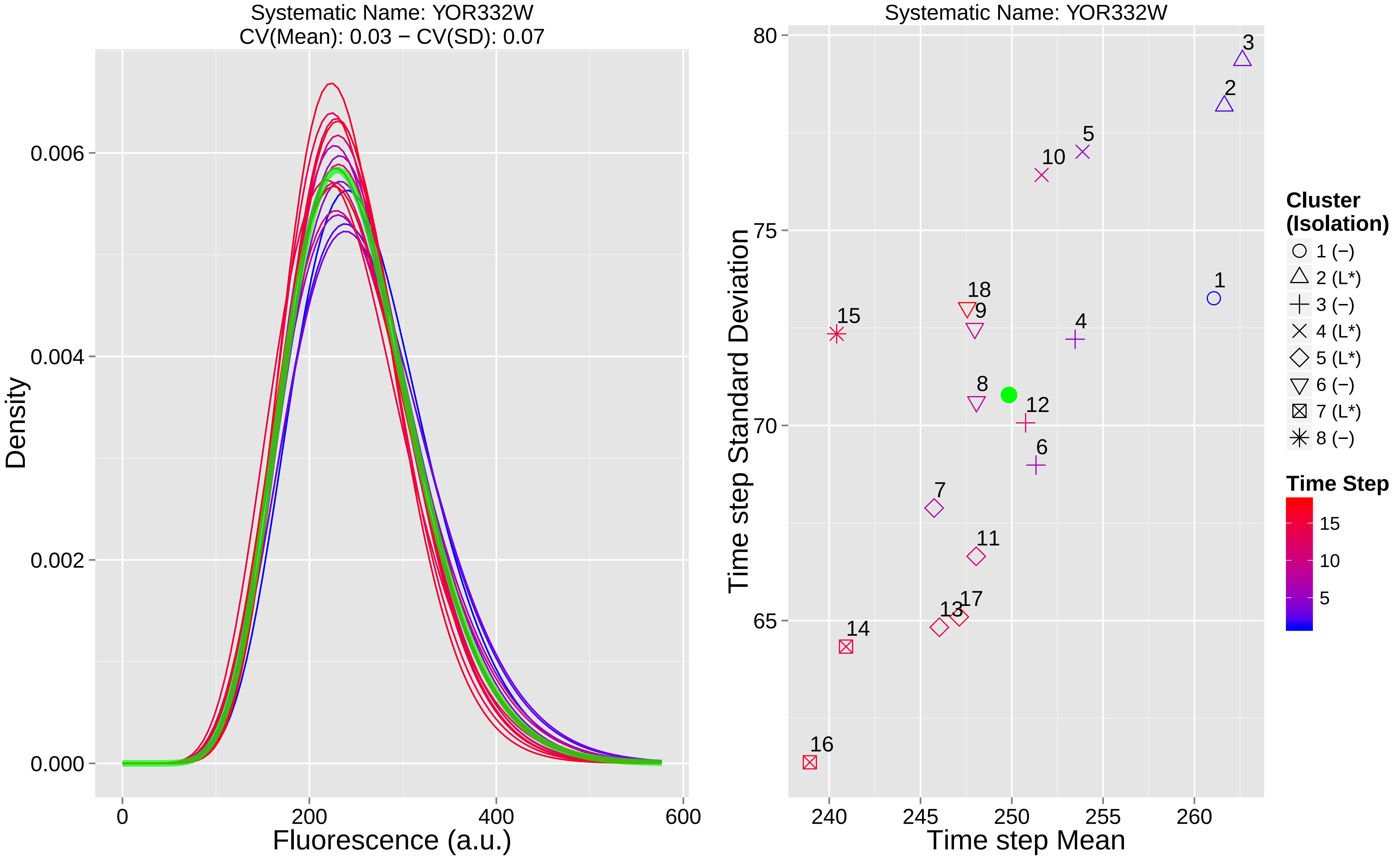

Systematic Name: YOR332W
CV(Mean): 0.03 − CV(SD): 0.07
Density
0.006
0.004
0.002
0.000
0
200
400
600
Fluorescence (a.u.)
Systematic Name: YOR332W
Time step Standard Deviation
80
75
70
65
240
245
250
255
260
Time step Mean
1
2
3
4
5
6
7
8
9
10
11
12
13
14
15
16
17
18
Cluster (Isolation)
1 (−)
2 (L*)
3 (−)
4 (L*)
5 (L*)
6 (−)
7 (L*)
8 (−)
Time Step
15
10
5

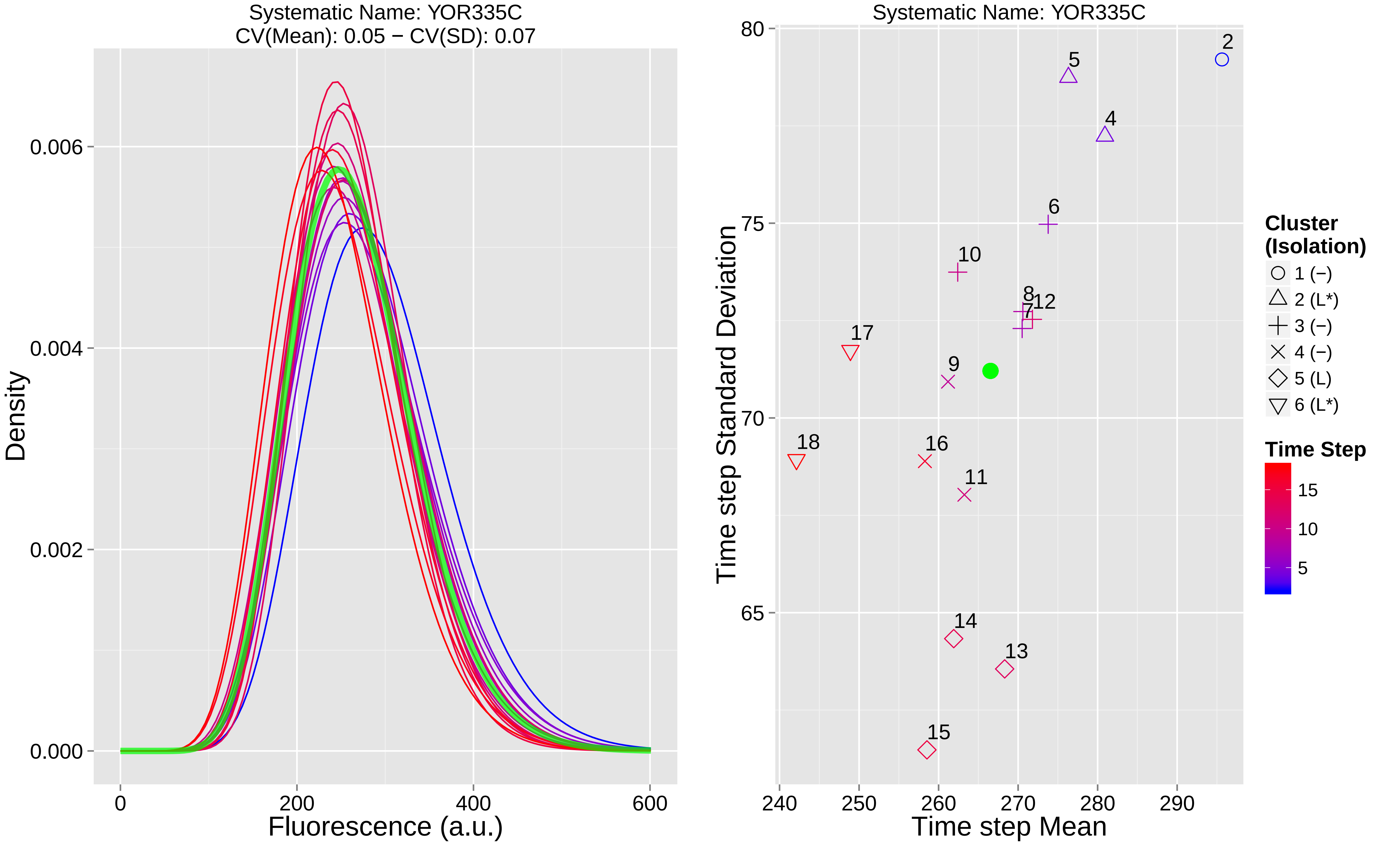
Systematic Name: YOR335C
CV(Mean): 0.05 − CV(SD): 0.07
Density
0.006
0.004
0.002
0.000
0
200
400
600
Fluorescence (a.u.)
Systematic Name: YOR335C
Time step Standard Deviation
80
75
70
65
240
250
260
270
280
290
Time step Mean
2
5
4
6
10
8
7
12
17
9
18
16
11
14
13
15
Cluster
(Isolation)
1 (−)
2 (L*)
3 (−)
4 (−)
5 (L)
6 (L*)
Time Step
15
10
5

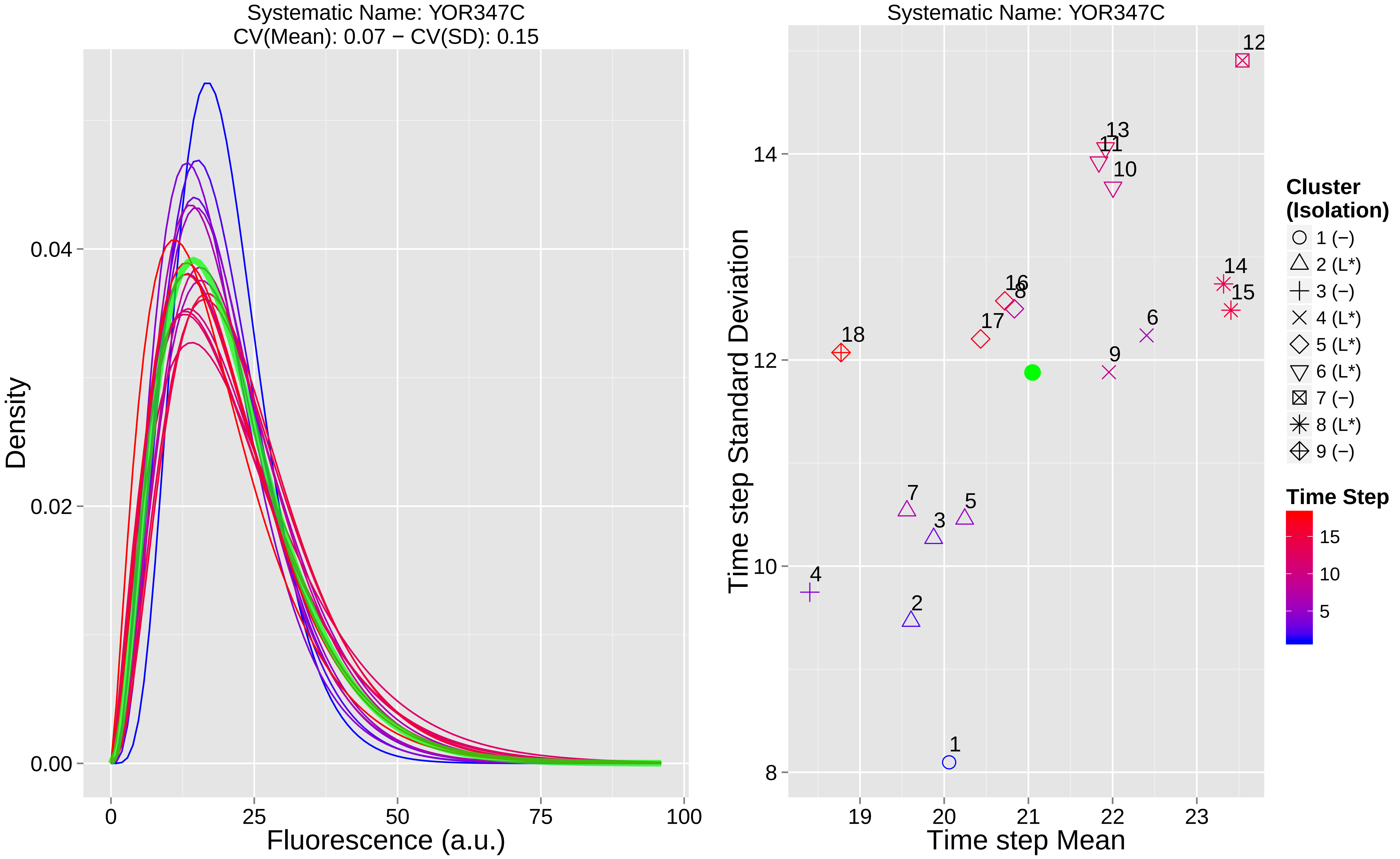
Systematic Name: YOR347C
CV(Mean): 0.07 − CV(SD): 0.15
Density
0.00
0.02
0.04
0
25
50
75
100
Fluorescence (a.u.)
Systematic Name: YOR347C
Time step Standard Deviation
8
10
12
14
19
20
21
22
23
Time step Mean
Cluster
(Isolation)
1 (−)
2 (L*)
3 (−)
4 (L*)
5 (L*)
6 (L*)
7 (−)
8 (L*)
9 (−)
Time Step
15
10
5

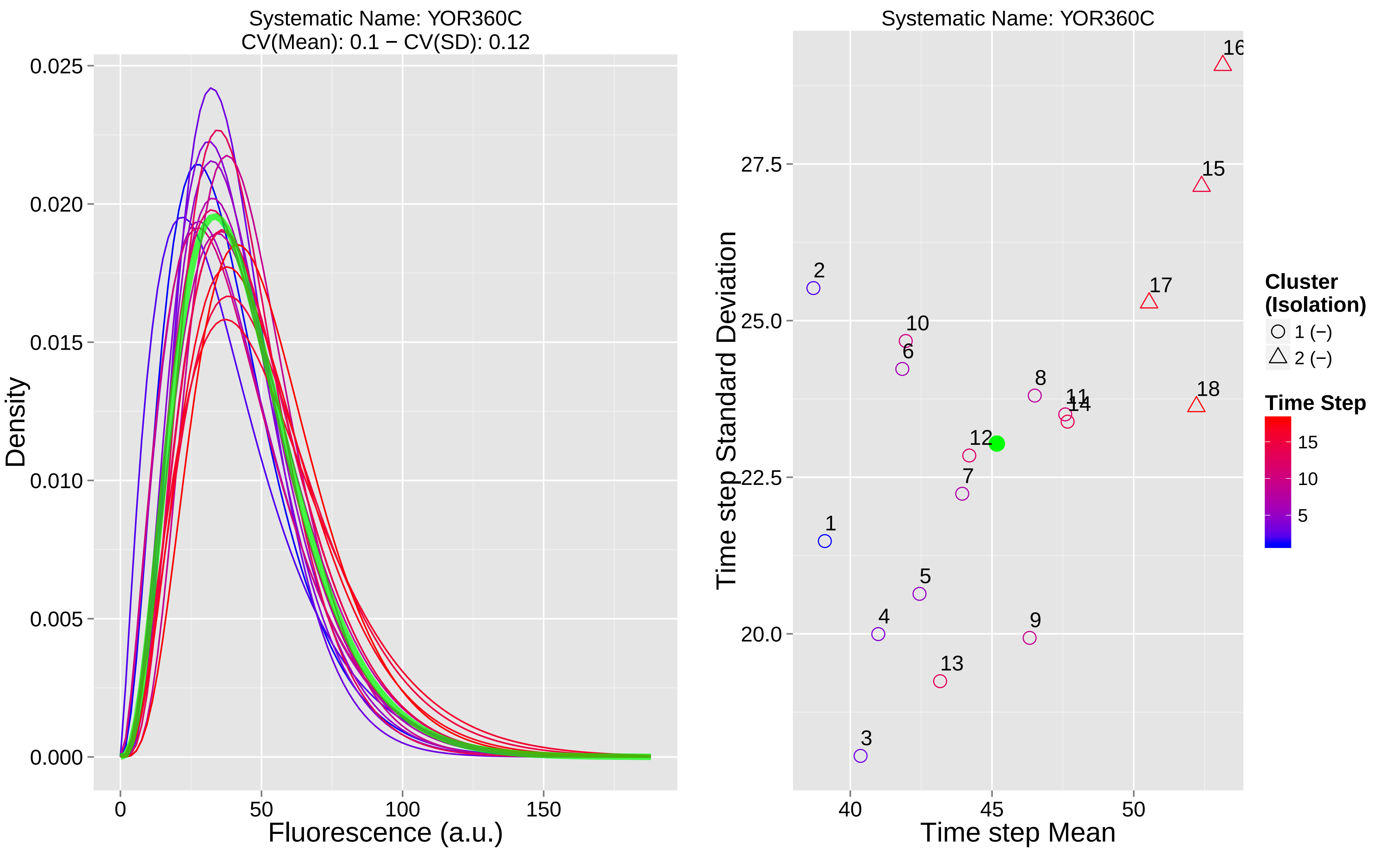

Systematic Name: YOR360C
CV(Mean): 0.1 − CV(SD): 0.12
Density
0.025
0.020
0.015
0.010
0.005
0.000
0
50
100
150
Fluorescence (a.u.)
Systematic Name: YOR360C
Time step Standard Deviation
27.5
25.0
22.5
20.0
40
45
50
Time step Mean
1
2
3
4
5
6
7
8
9
10
11
12
13
14
15
16
17
18
Cluster
(Isolation)
1 (−)
2 (−)
Time Step
15
10
5

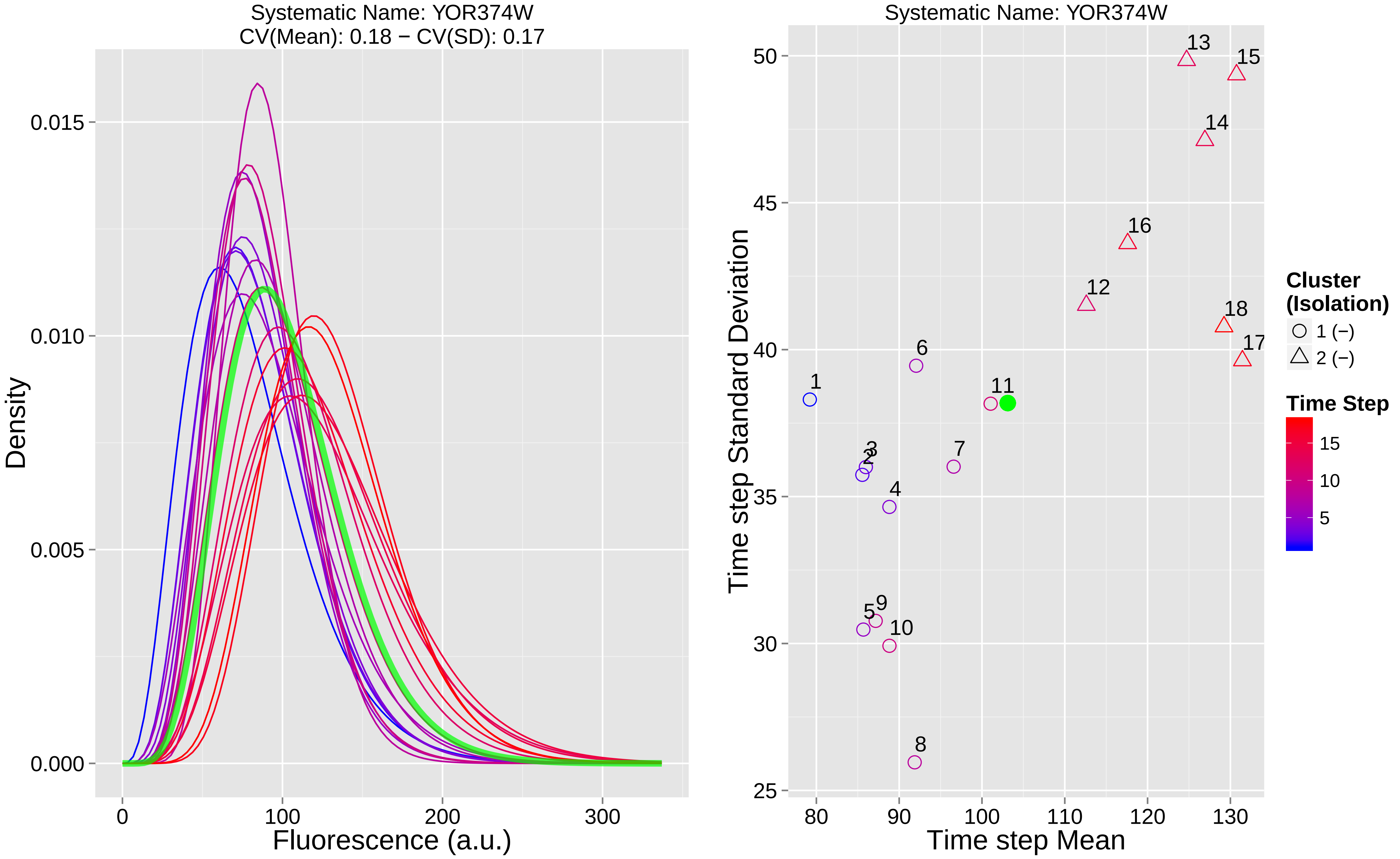
Systematic Name: YOR374W
CV(Mean): 0.18 − CV(SD): 0.17
Density
0.015
0.010
0.005
0.000
0
100
200
300
Fluorescence (a.u.)
Systematic Name: YOR374W
Time step Standard Deviation
50
45
40
35
30
25
80
90
100
110
120
130
Time step Mean
1
2
3
4
5
6
7
8
9
10
11
12
13
14
15
16
17
18
Cluster (Isolation)
1 (−)
2 (−)
Time Step
15
10
5

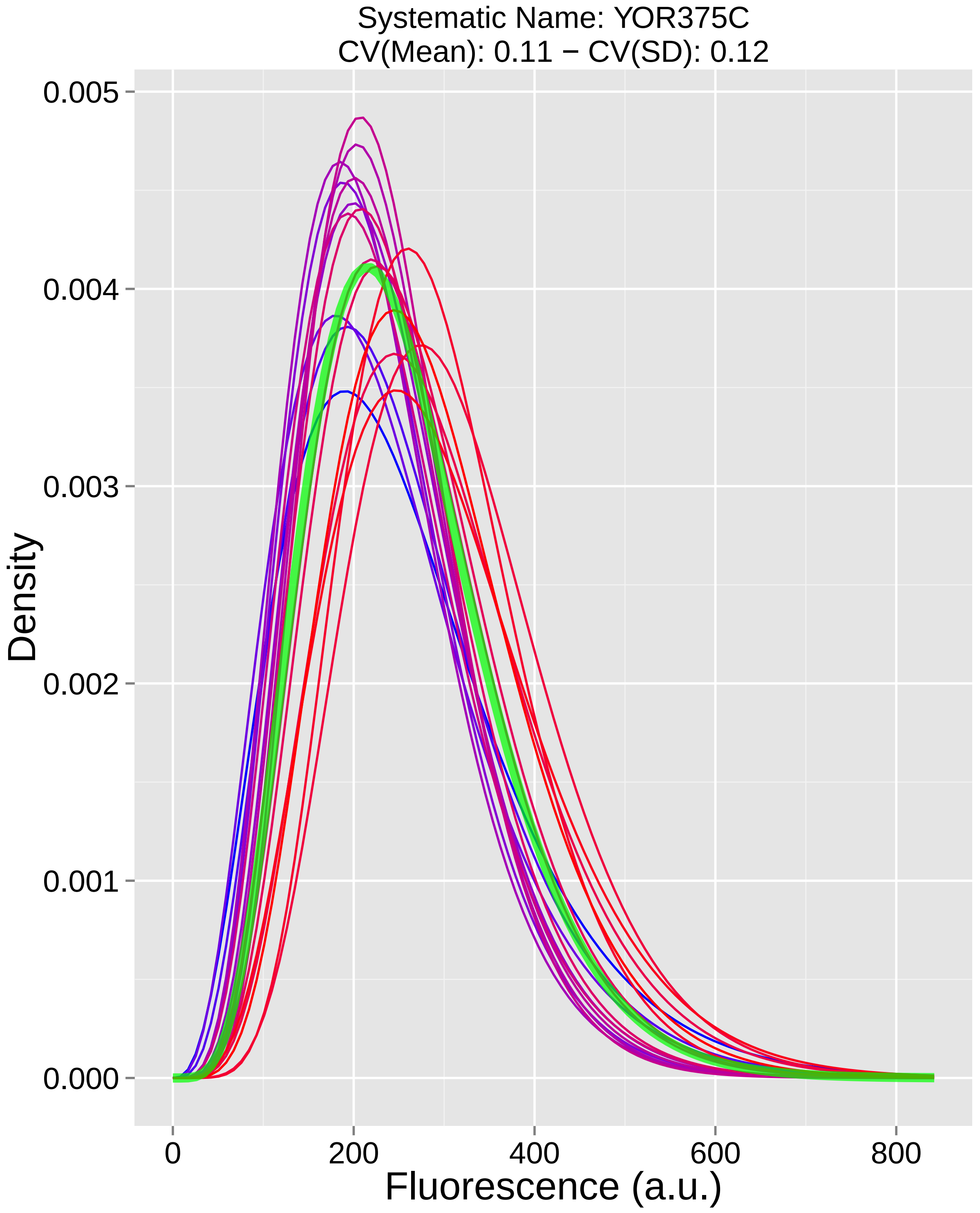

Systematic Name: YOR375C
CV(Mean): 0.11 − CV(SD): 0.12
Density
Fluorescence (a.u.)
0.005
0.004
0.003
0.002
0.001
0.000
0
200
400
600
800

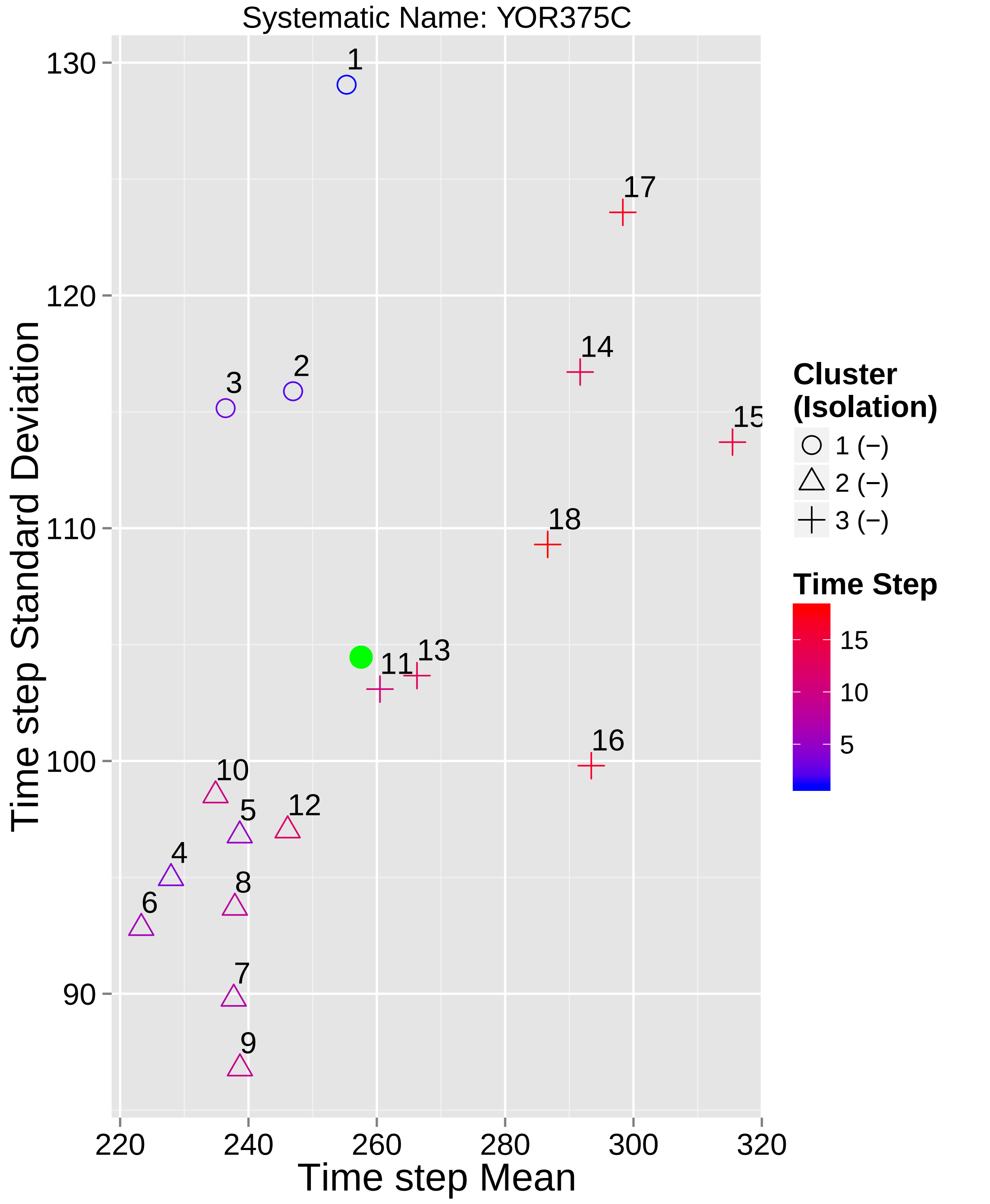

Systematic Name: YOR375C
Time step Standard Deviation
Time step Mean
130
120
110
100
90
220
240
260
280
300
320
1
2
3
4
5
6
7
8
9
10
11
12
13
14
15
16
17
18
Cluster (Isolation)
1 (−)
2 (−)
3 (−)
Time Step
15
10
5

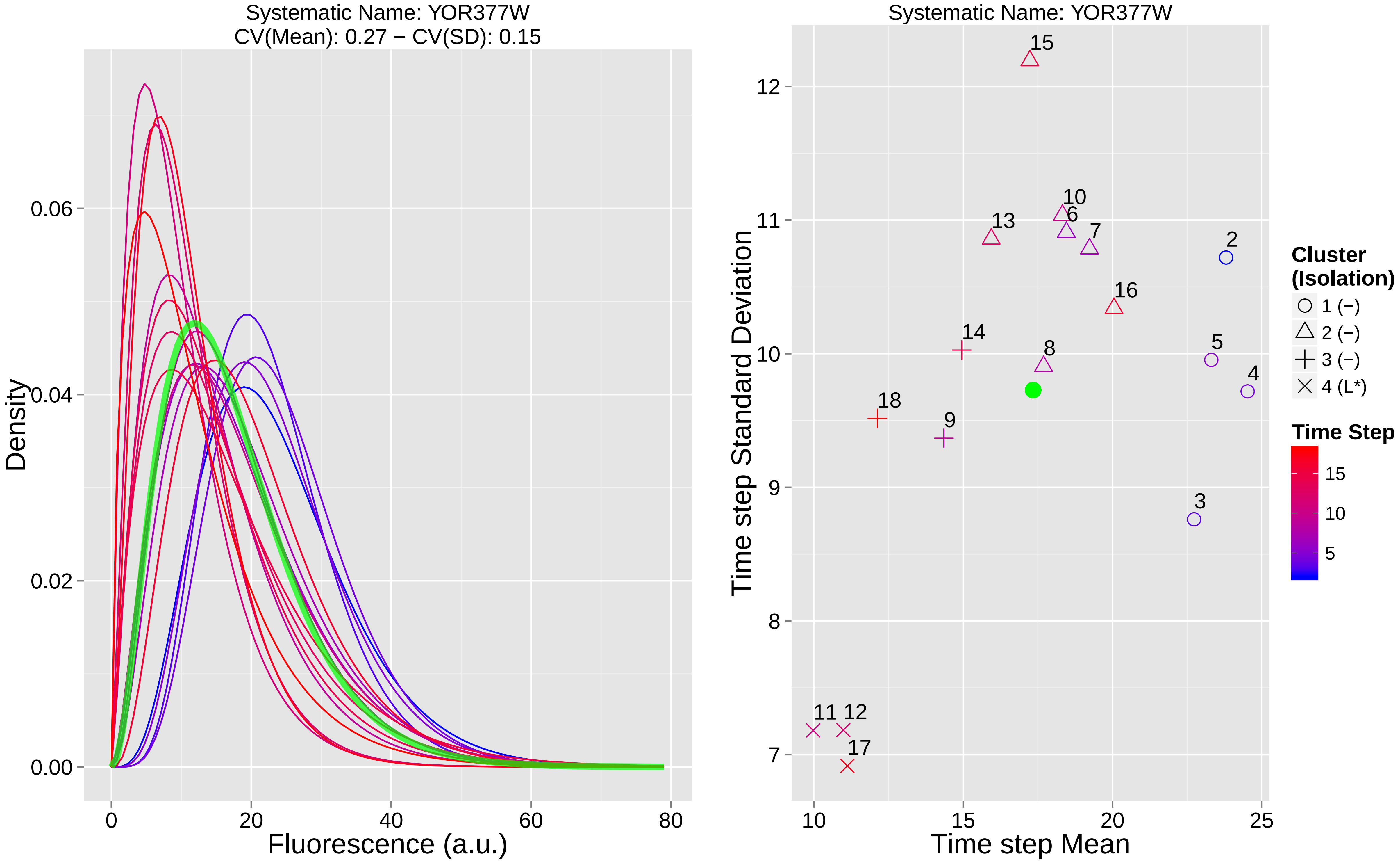
Systematic Name: YOR377W
CV(Mean): 0.27 − CV(SD): 0.15
Density
0.00
0.02
0.04
0.06
0
20
40
60
80
Fluorescence (a.u.)
Systematic Name: YOR377W
Time step Standard Deviation
7
8
9
10
11
12
10
15
20
25
Time step Mean
15
10
6
13
7
2
16
14
5
8
4
18
9
3
11
12
17
Cluster
(Isolation)
1 (−)
2 (−)
3 (−)
4 (L*)
Time Step
15
10
5

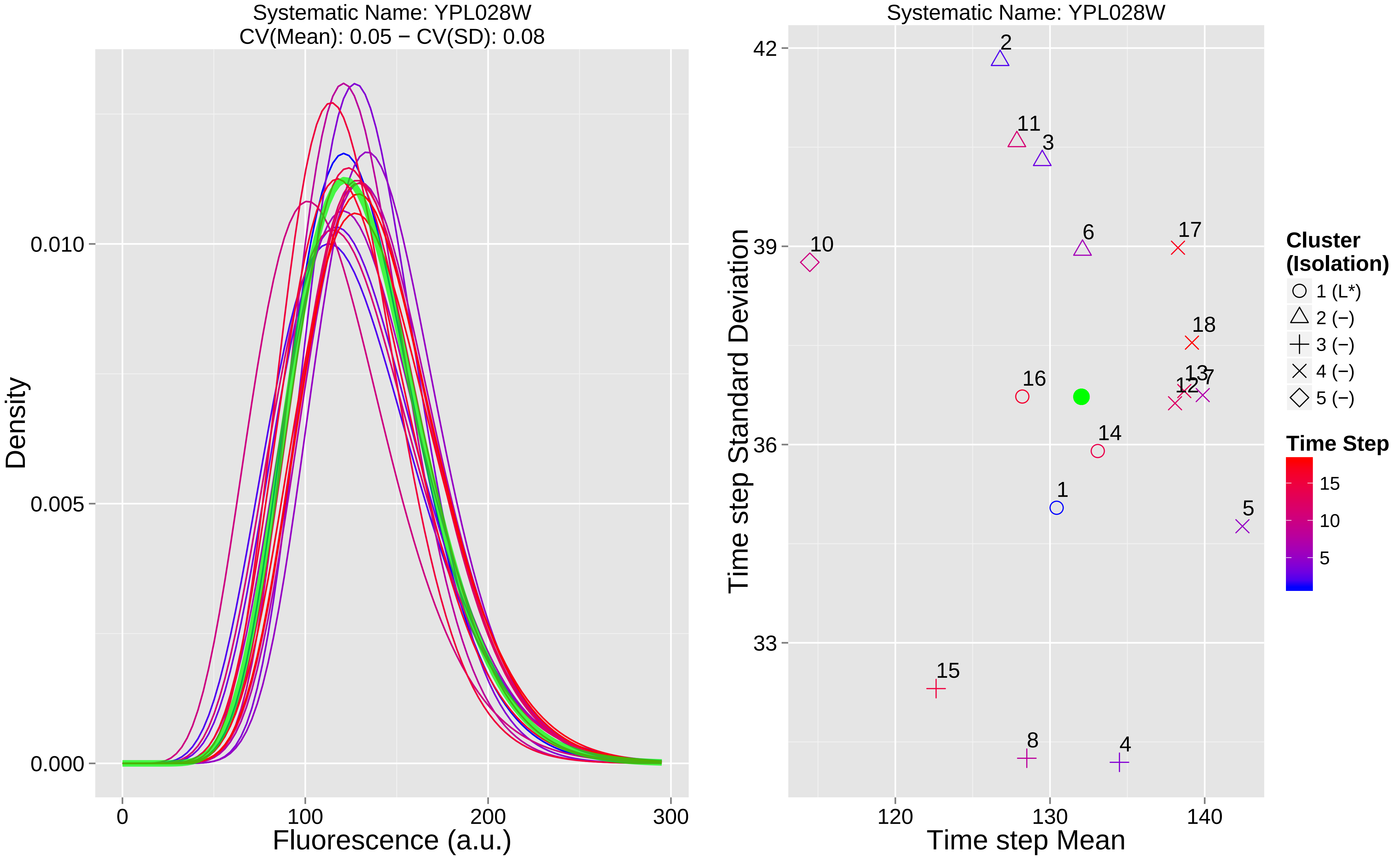

Systematic Name: YPL028W
CV(Mean): 0.05 − CV(SD): 0.08
Density
0.010
0.005
0.000
0
100
200
300
Fluorescence (a.u.)
Systematic Name: YPL028W
Time step Standard Deviation
42
39
36
33
120
130
140
Time step Mean
Cluster (Isolation)
1 (L*)
2 (−)
3 (−)
4 (−)
5 (−)
Time Step
15
10
5

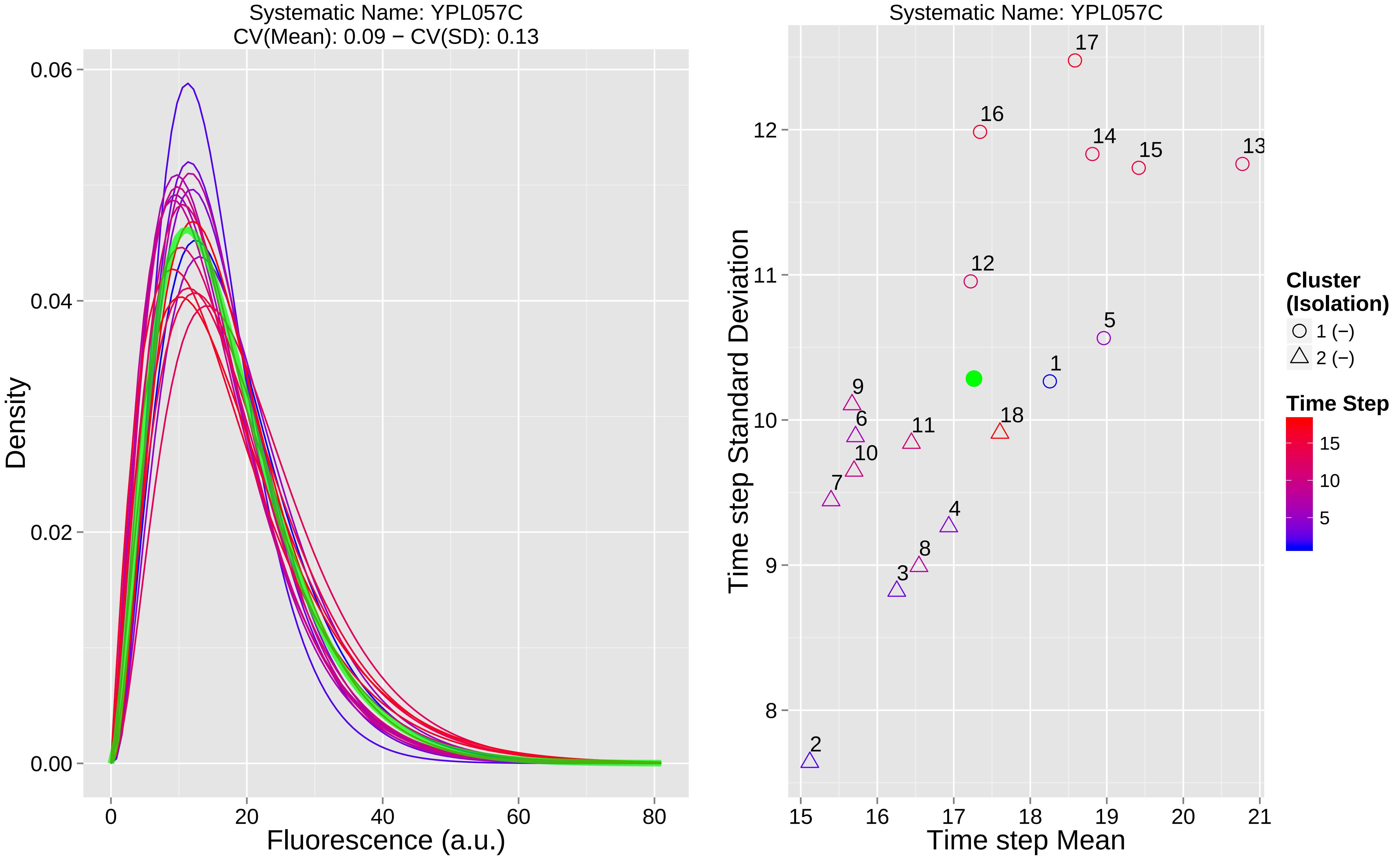
Systematic Name: YPL057C
CV(Mean): 0.09 − CV(SD): 0.13
Density
Fluorescence (a.u.)
0.06
0.04
0.02
0.00
0
20
40
60
80
Systematic Name: YPL057C
Time step Standard Deviation
Time step Mean
12
11
10
9
8
15
16
17
18
19
20
21
17
16
14
15
13
12
5
1
9
6
11
18
10
7
4
8
3
2
Cluster
(Isolation)
1 (−)
2 (−)
Time Step
15
10
5

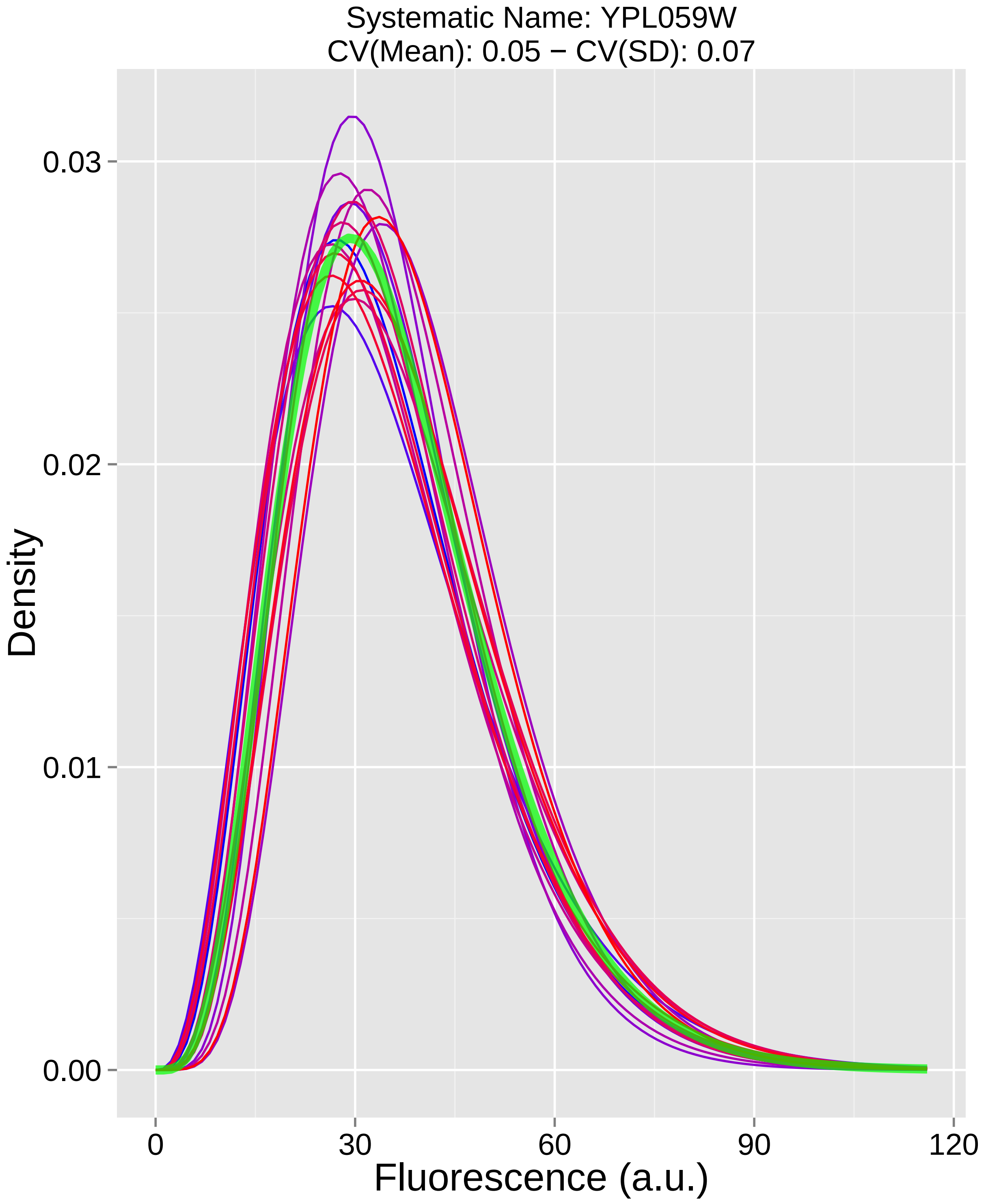

Systematic Name: YPL059W
CV(Mean): 0.05 − CV(SD): 0.07
Density
0.03
0.02
0.01
0.00
0
30
60
90
120
Fluorescence (a.u.)

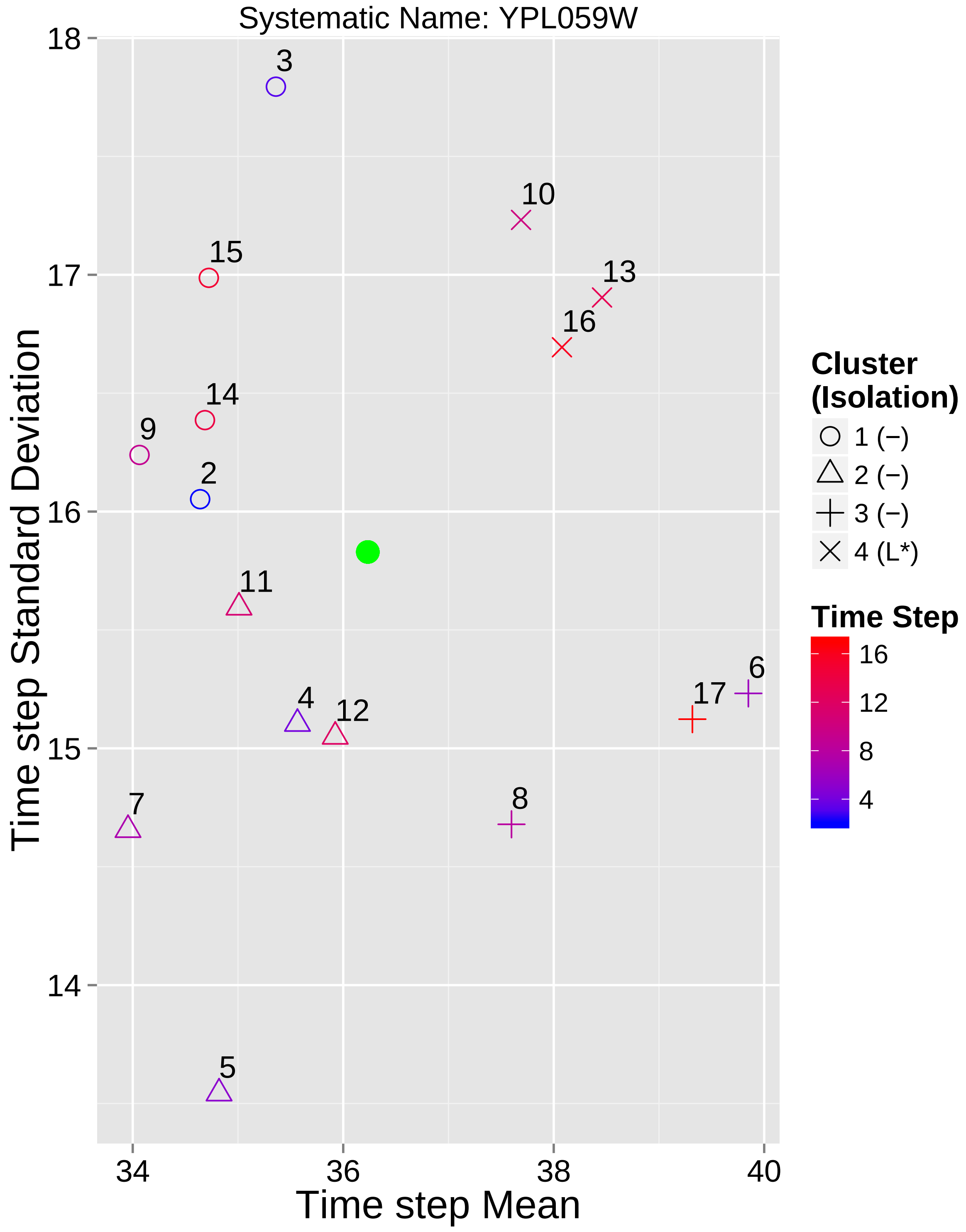

Systematic Name: YPL059W
Time step Standard Deviation
18
17
16
15
14
34
36
38
40
Time step Mean
3
10
15
13
16
14
9
2
11
6
17
4
12
7
8
5
Cluster (Isolation)
1 (−)
2 (−)
3 (−)
4 (L*)
Time Step
16
12
8
4

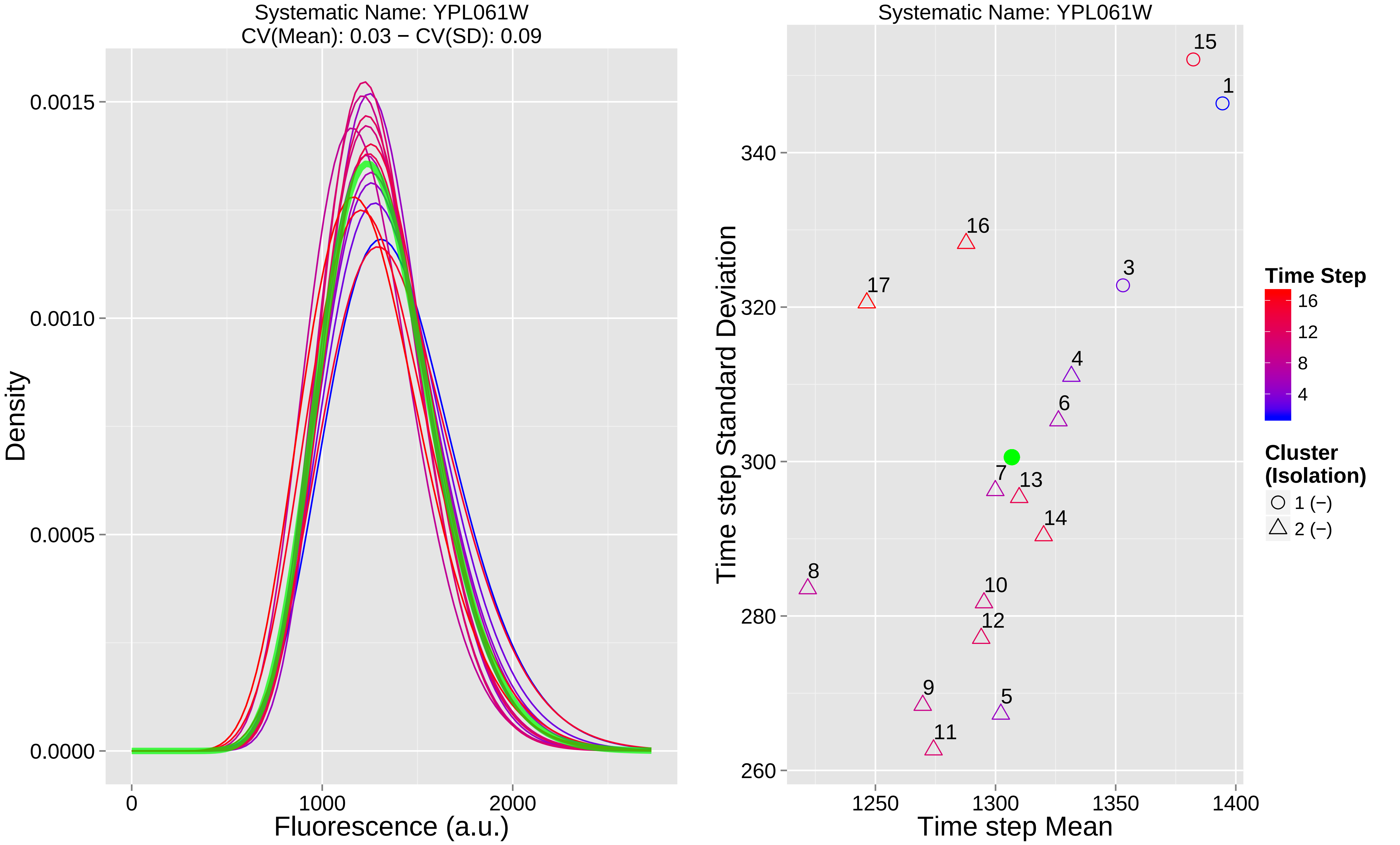

Systematic Name: YPL061W
CV(Mean): 0.03 − CV(SD): 0.09
Density
0.0015
0.0010
0.0005
0.0000
0
1000
2000
Fluorescence (a.u.)
Systematic Name: YPL061W
Time step Standard Deviation
340
320
300
280
260
1250
1300
1350
1400
Time step Mean
15
1
16
3
17
4
6
7
13
14
8
10
12
9
5
11
Time Step
16
12
8
4
Cluster
(Isolation)
1 (−)
2 (−)

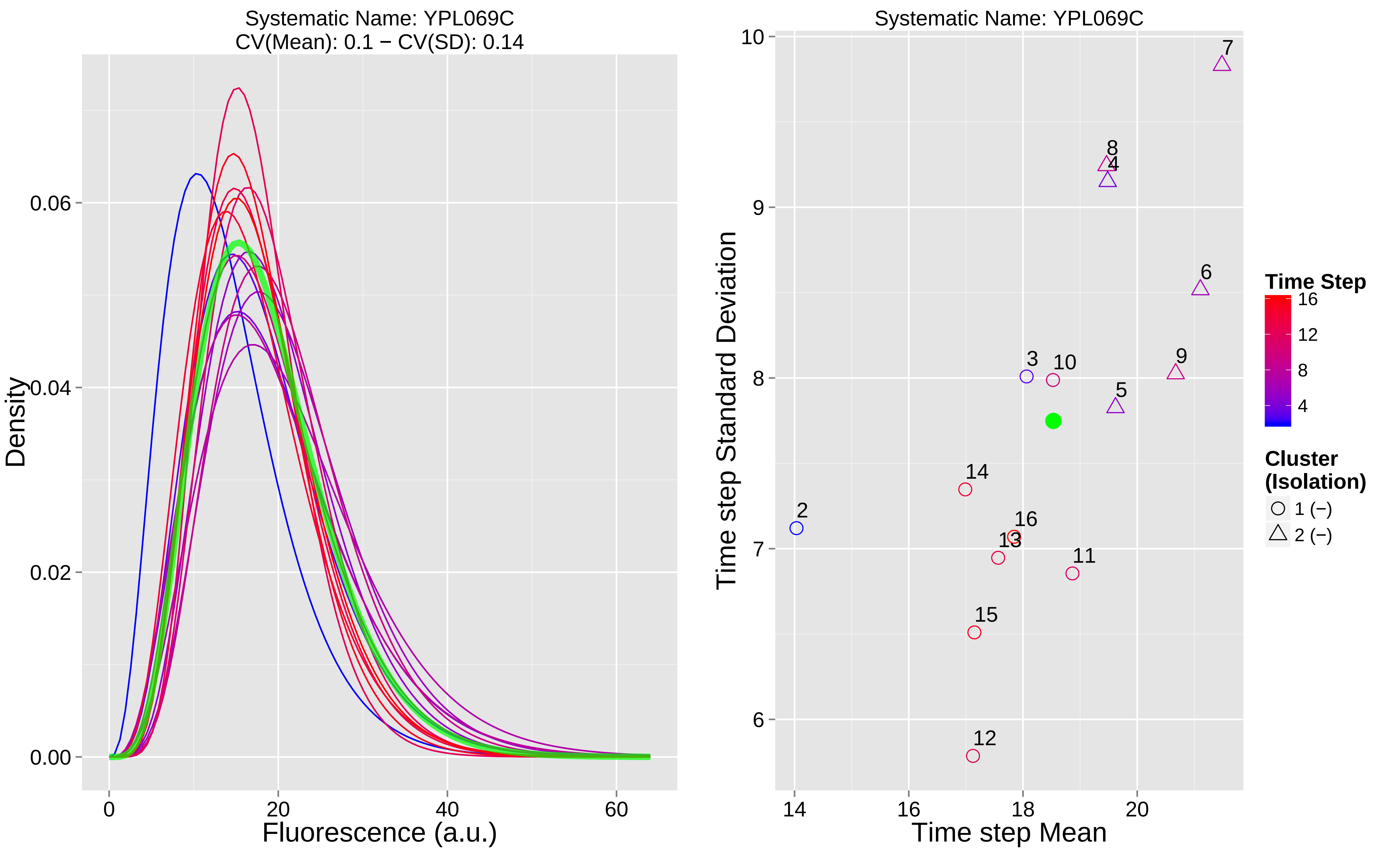

Systematic Name: YPL069C
CV(Mean): 0.1 − CV(SD): 0.14
Density
0.06
0.04
0.02
0.00
0
20
40
60
Fluorescence (a.u.)
Systematic Name: YPL069C
Time step Standard Deviation
10
9
8
7
6
14
16
18
20
Time step Mean
7
8
4
6
3
10
9
5
14
2
16
13
11
15
12
Time Step
16
12
8
4
Cluster
(Isolation)
1 (−)
2 (−)

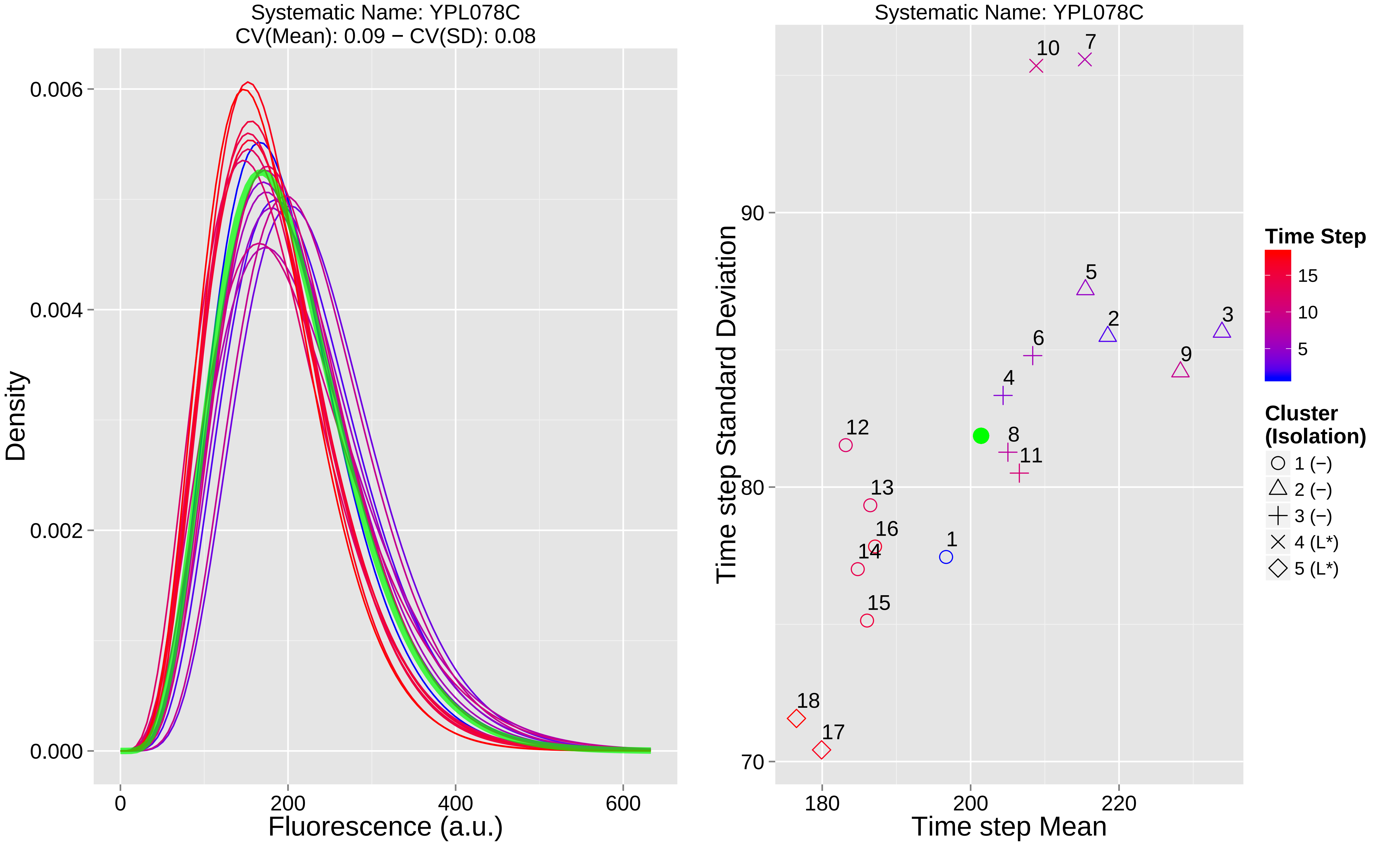
Systematic Name: YPL078C
CV(Mean): 0.09 − CV(SD): 0.08
Density
0.006
0.004
0.002
0.000
0
200
400
600
Fluorescence (a.u.)
Systematic Name: YPL078C
Time step Standard Deviation
90
80
70
180
200
220
Time step Mean
10
7
5
2
3
6
9
4
12
8
11
13
16
1
14
15
18
17
Time Step
15
10
5
Cluster
(Isolation)
1 (−)
2 (−)
3 (−)
4 (L*)
5 (L*)

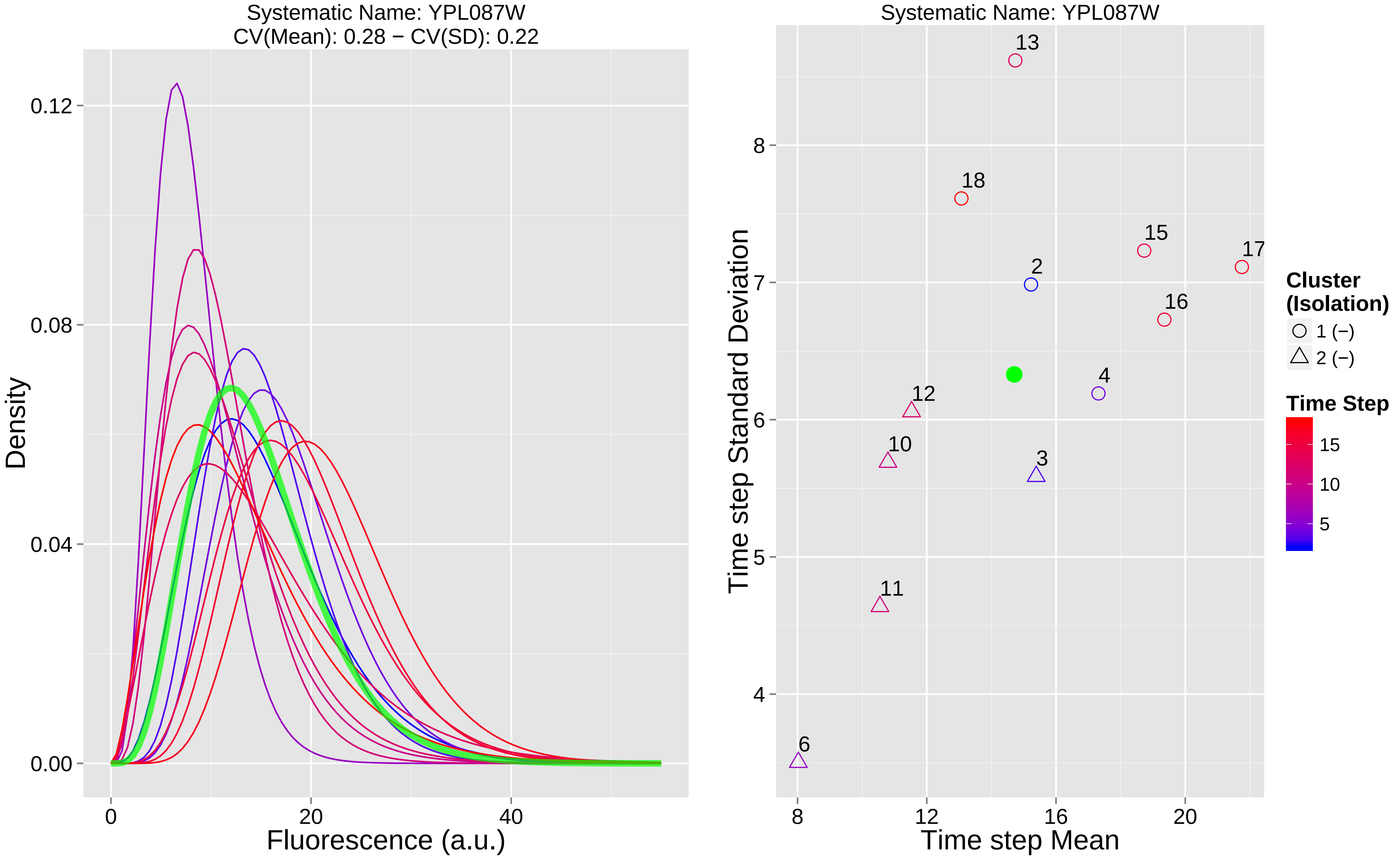
Systematic Name: YPL087W
CV(Mean): 0.28 − CV(SD): 0.22
Density
0.12
0.08
0.04
0.00
0
20
40
Fluorescence (a.u.)
Systematic Name: YPL087W
Time step Standard Deviation
8
7
6
5
4
8
12
16
20
Time step Mean
13
18
15
17
2
16
4
12
10
3
11
6
Cluster (Isolation)
1 (−)
2 (−)
Time Step
15
10
5

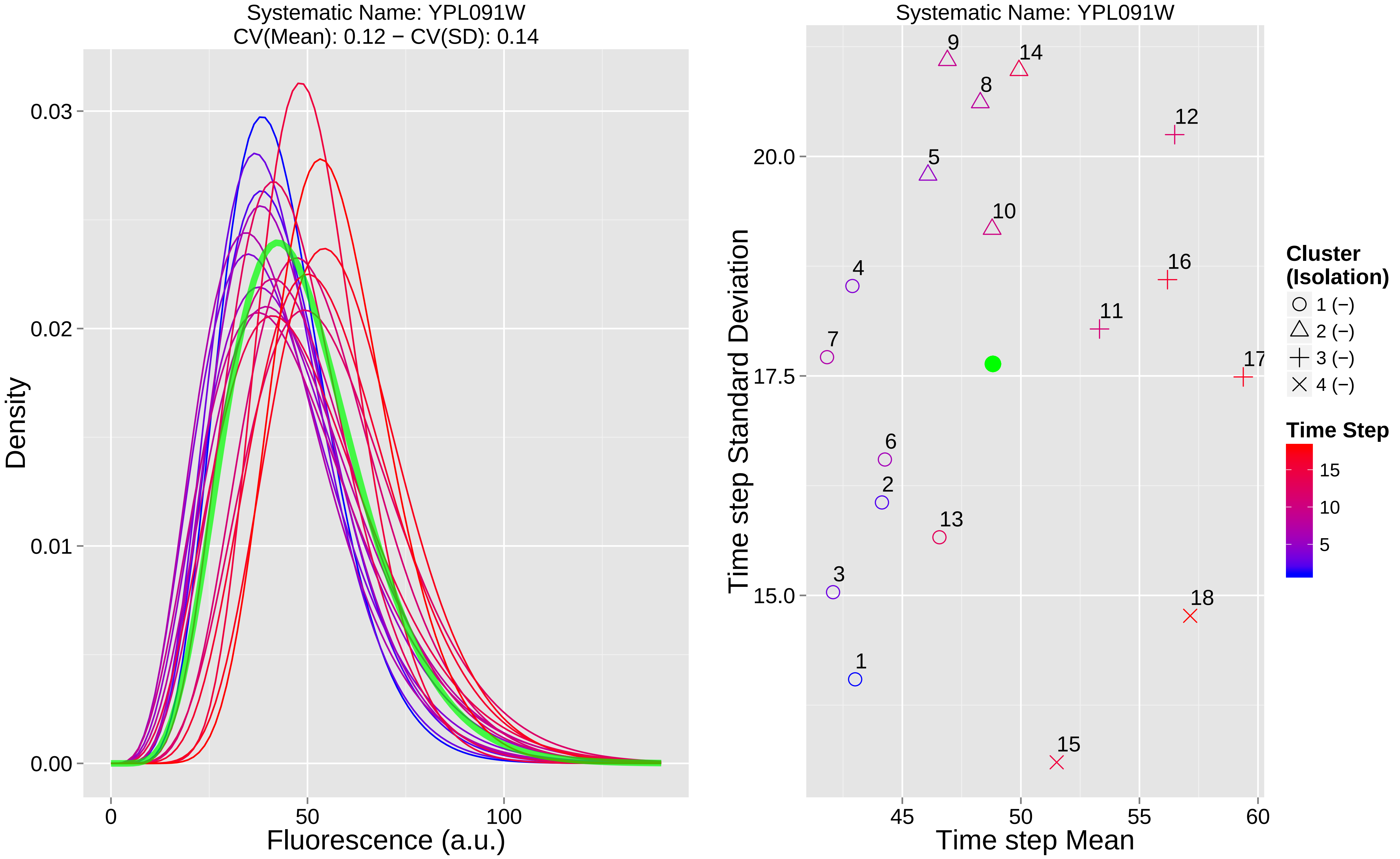

Systematic Name: YPL091W
CV(Mean): 0.12 − CV(SD): 0.14
Density
Fluorescence (a.u.)
0.00
0.01
0.02
0.03
0
50
100
Systematic Name: YPL091W
Time step Standard Deviation
Time step Mean
15.0
17.5
20.0
45
50
55
60
1
2
3
4
5
6
7
8
9
10
11
12
13
14
15
16
17
18
Cluster
(Isolation)
1 (−)
2 (−)
3 (−)
4 (−)
Time Step
5
10
15

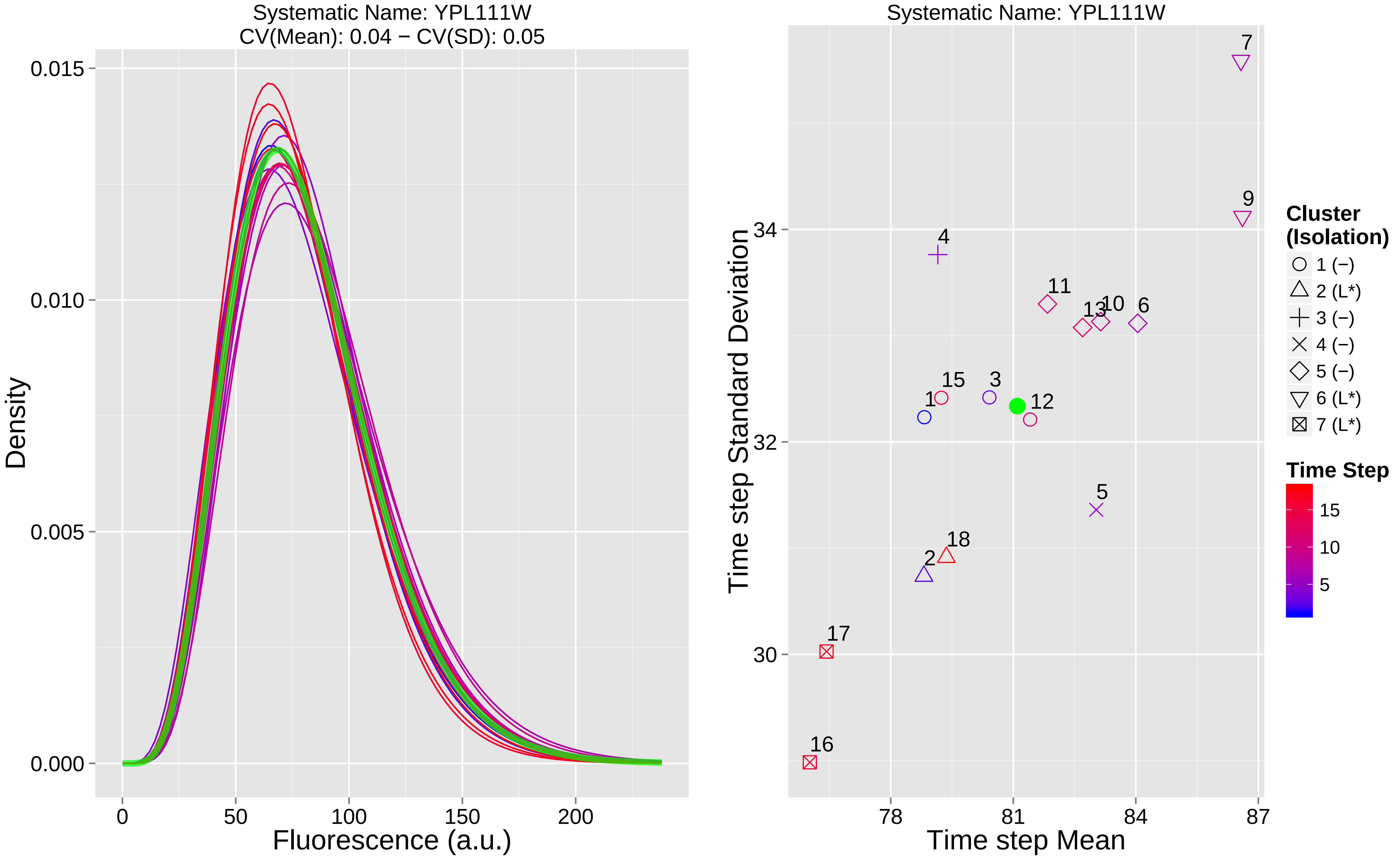

Systematic Name: YPL111W
CV(Mean): 0.04 − CV(SD): 0.05
Density
0.015
0.010
0.005
0.000
0
50
100
150
200
Fluorescence (a.u.)
Systematic Name: YPL111W
Time step Standard Deviation
34
32
30
78
81
84
87
Time step Mean
Cluster
(Isolation)
1 (−)
2 (L*)
3 (−)
4 (−)
5 (−)
6 (L*)
7 (L*)
Time Step
15
10
5

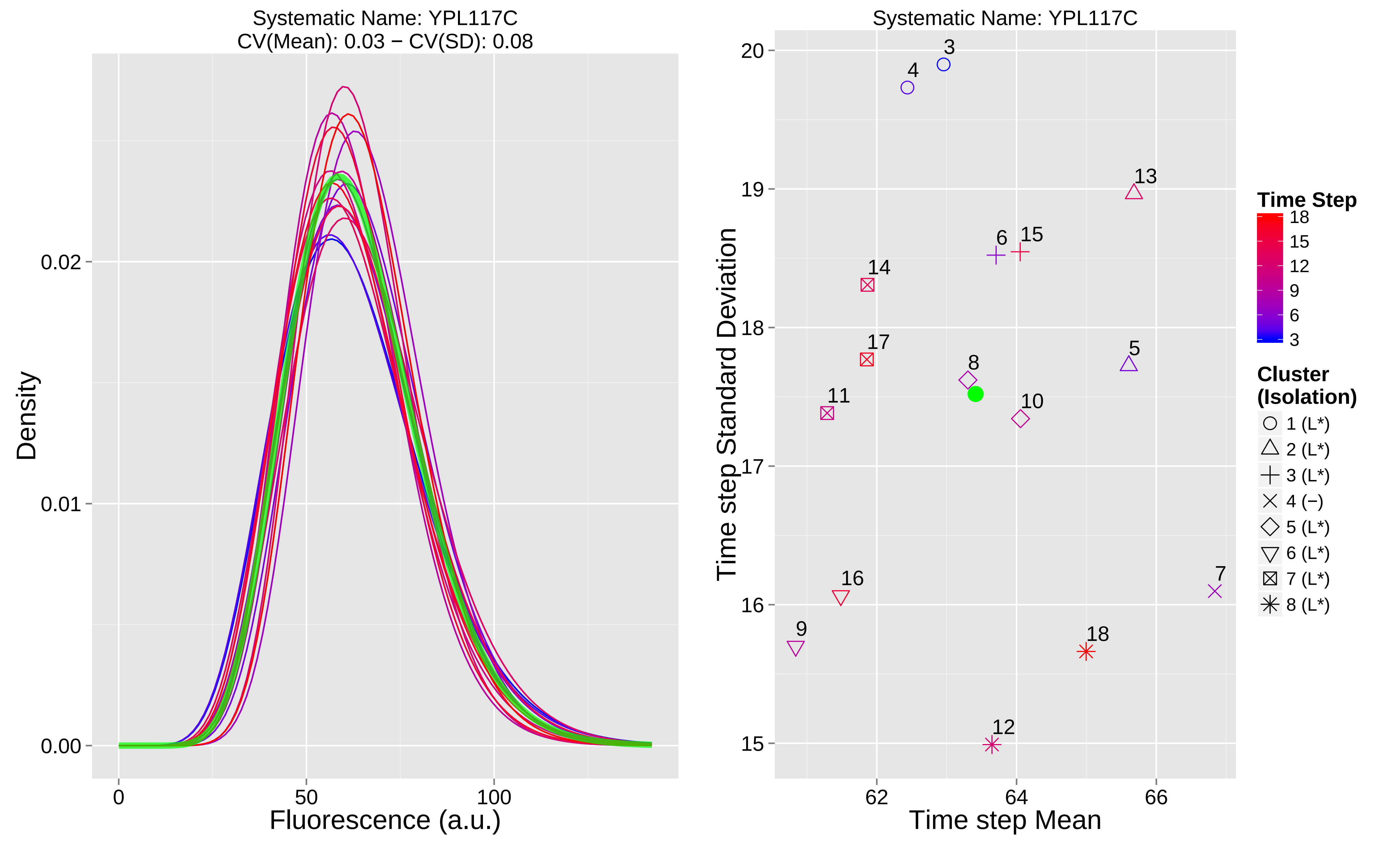
Systematic Name: YPL117C
CV(Mean): 0.03 − CV(SD): 0.08
Density
0.00
0.01
0.02
0
50
100
Fluorescence (a.u.)
Systematic Name: YPL117C
Time step Standard Deviation
15
16
17
18
19
20
62
64
66
Time step Mean
3
4
13
6
15
14
17
8
5
11
10
16
7
9
18
12
Time Step
18
15
12
9
6
3
Cluster
(Isolation)
1 (L*)
2 (L*)
3 (L*)
4 (−)
5 (L*)
6 (L*)
7 (L*)
8 (L*)

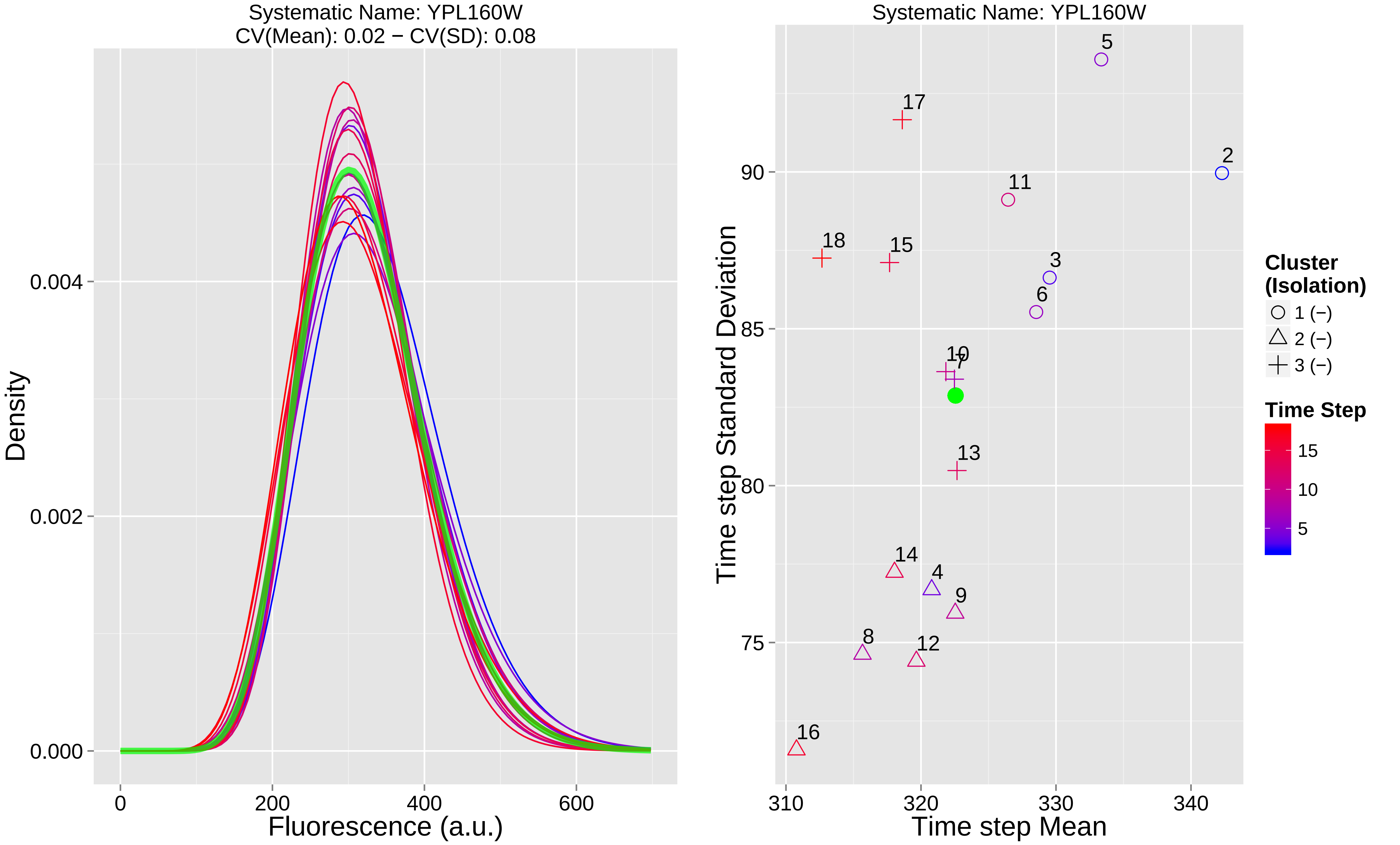

Systematic Name: YPL160W
CV(Mean): 0.02 − CV(SD): 0.08
Density
0.004
0.002
0.000
0
200
400
600
Fluorescence (a.u.)
Systematic Name: YPL160W
Time step Standard Deviation
90
85
80
75
310
320
330
340
Time step Mean
5
17
2
11
18
15
3
6
10
7
13
14
4
9
8
12
16
Cluster (Isolation)
1 (−)
2 (−)
3 (−)
Time Step
15
10
5

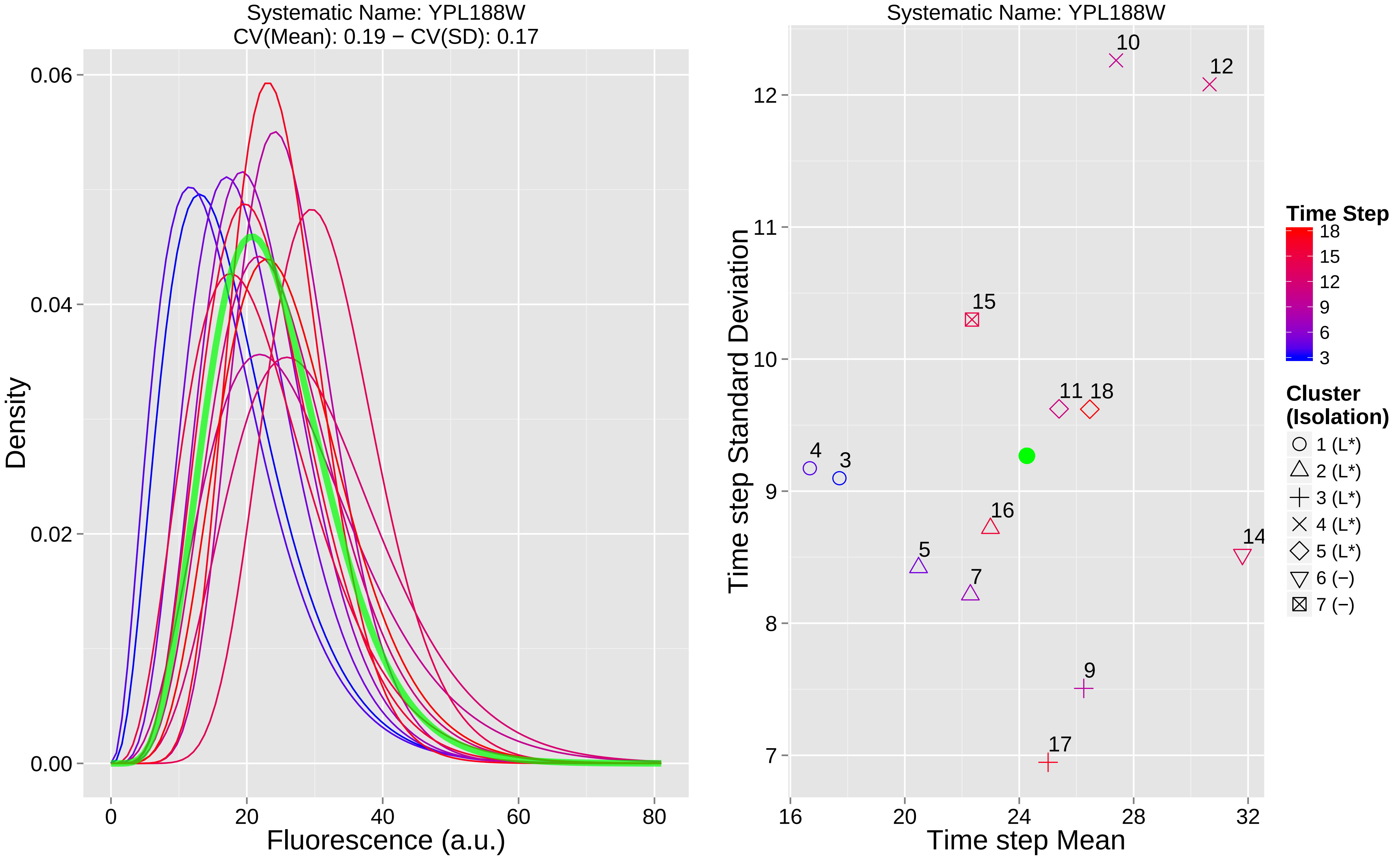

Systematic Name: YPL188W
CV(Mean): 0.19 − CV(SD): 0.17
Density
0.00
0.02
0.04
0.06
0
20
40
60
80
Fluorescence (a.u.)
Systematic Name: YPL188W
Time step Standard Deviation
Time step Mean
7
8
9
10
11
12
16
20
24
28
32
10
12
15
11 18
4
3
16
5
7
14
9
17
Time Step
18
15
12
9
6
3
Cluster (Isolation)
1 (L*)
2 (L*)
3 (L*)
4 (L*)
5 (L*)
6 (−)
7 (−)

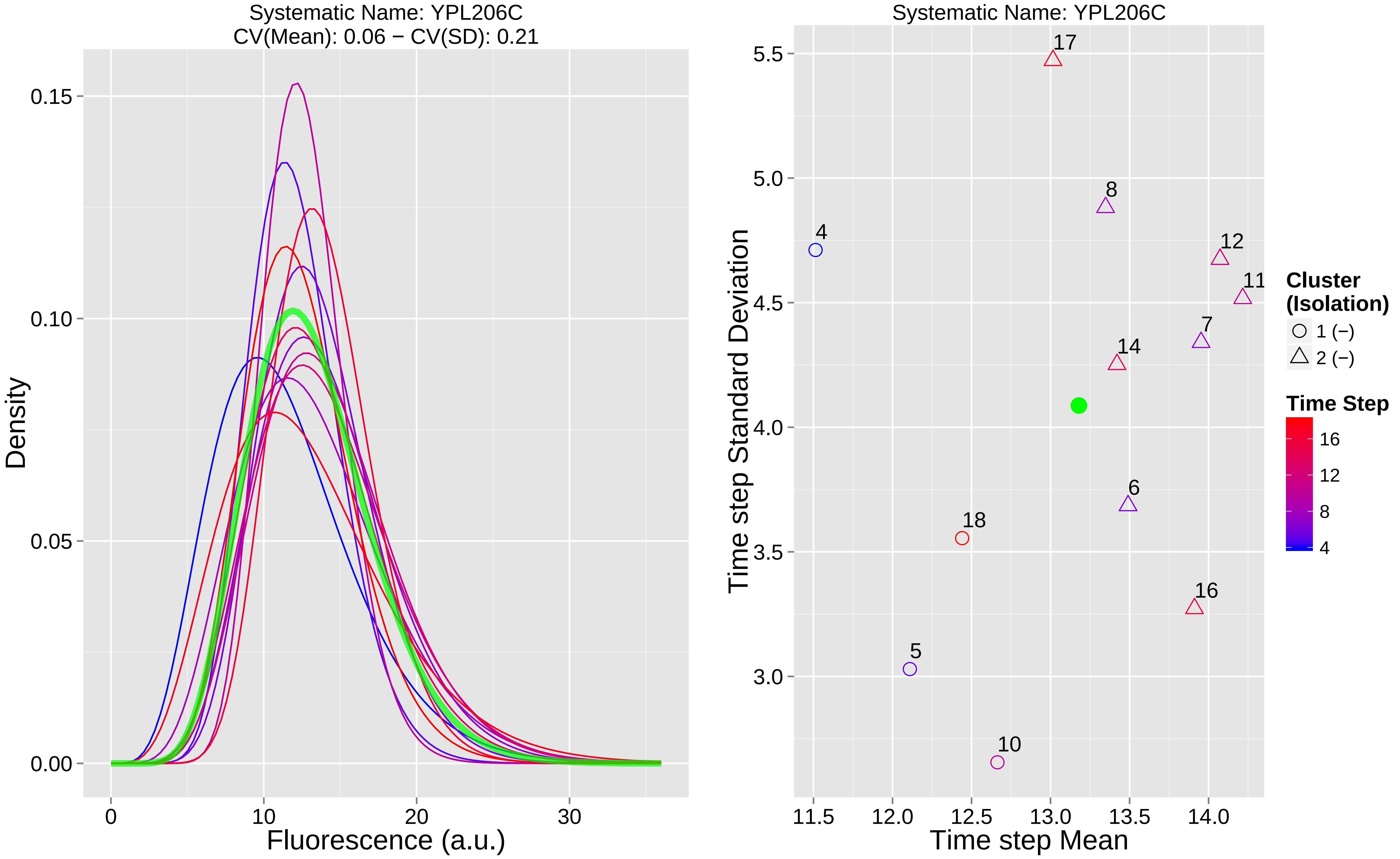
Systematic Name: YPL206C
CV(Mean): 0.06 − CV(SD): 0.21
Density
Fluorescence (a.u.)
0.15
0.10
0.05
0.00
0
10
20
30
Systematic Name: YPL206C
Time step Standard Deviation
Time step Mean
5.5
5.0
4.5
4.0
3.5
3.0
11.5
12.0
12.5
13.0
13.5
14.0
17
8
4
12
11
7
14
6
18
16
5
10
Cluster
(Isolation)
1 (−)
2 (−)
Time Step
16
12
8
4

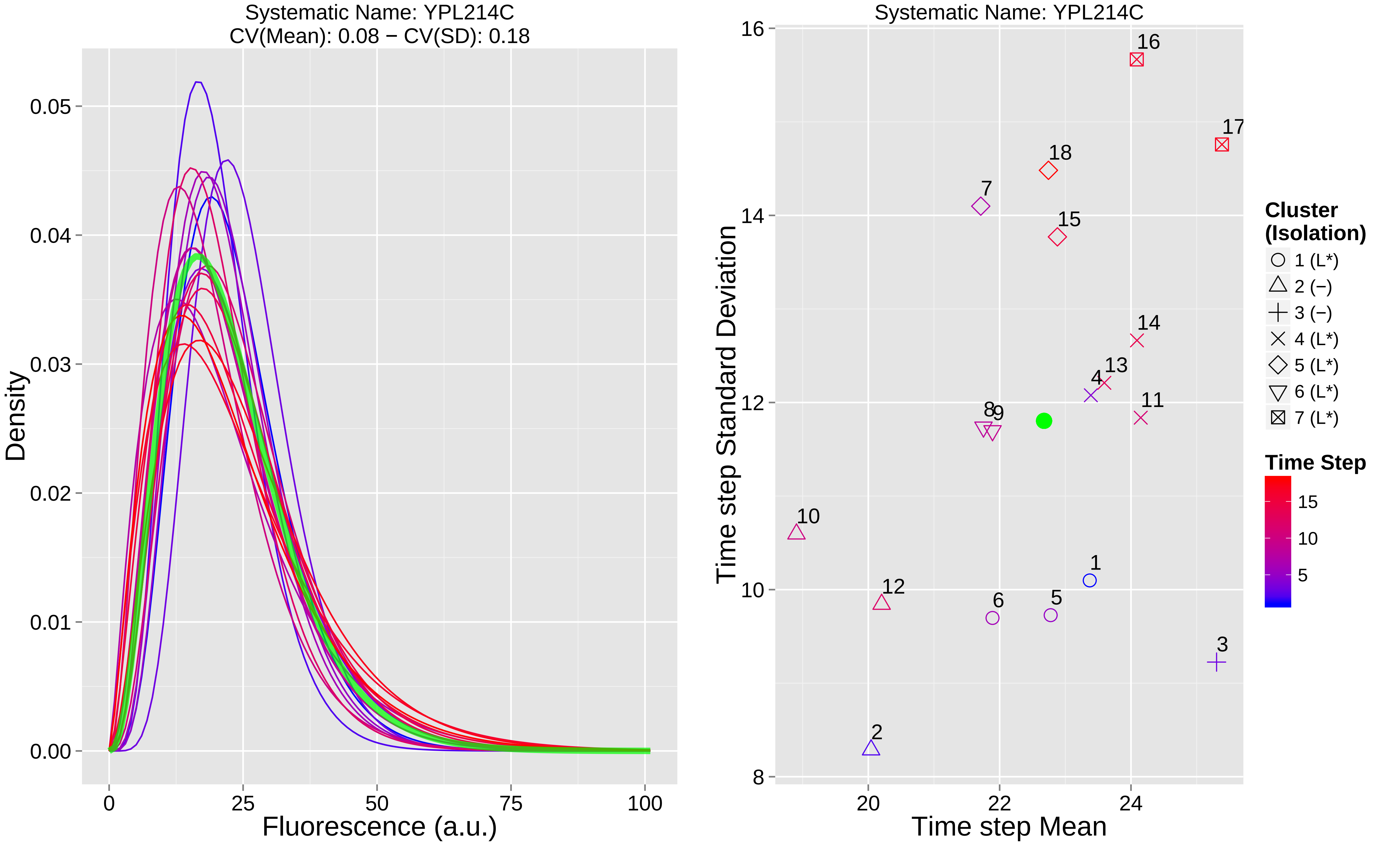

Systematic Name: YPL214C
CV(Mean): 0.08 − CV(SD): 0.18
Density
Fluorescence (a.u.)
0.00
0.01
0.02
0.03
0.04
0.05
0
25
50
75
100
Systematic Name: YPL214C
Time step Standard Deviation
Time step Mean
8
10
12
14
16
20
22
24
Cluster
(Isolation)
1 (L*)
2 (−)
3 (−)
4 (L*)
5 (L*)
6 (L*)
7 (L*)
Time Step
15
10
5

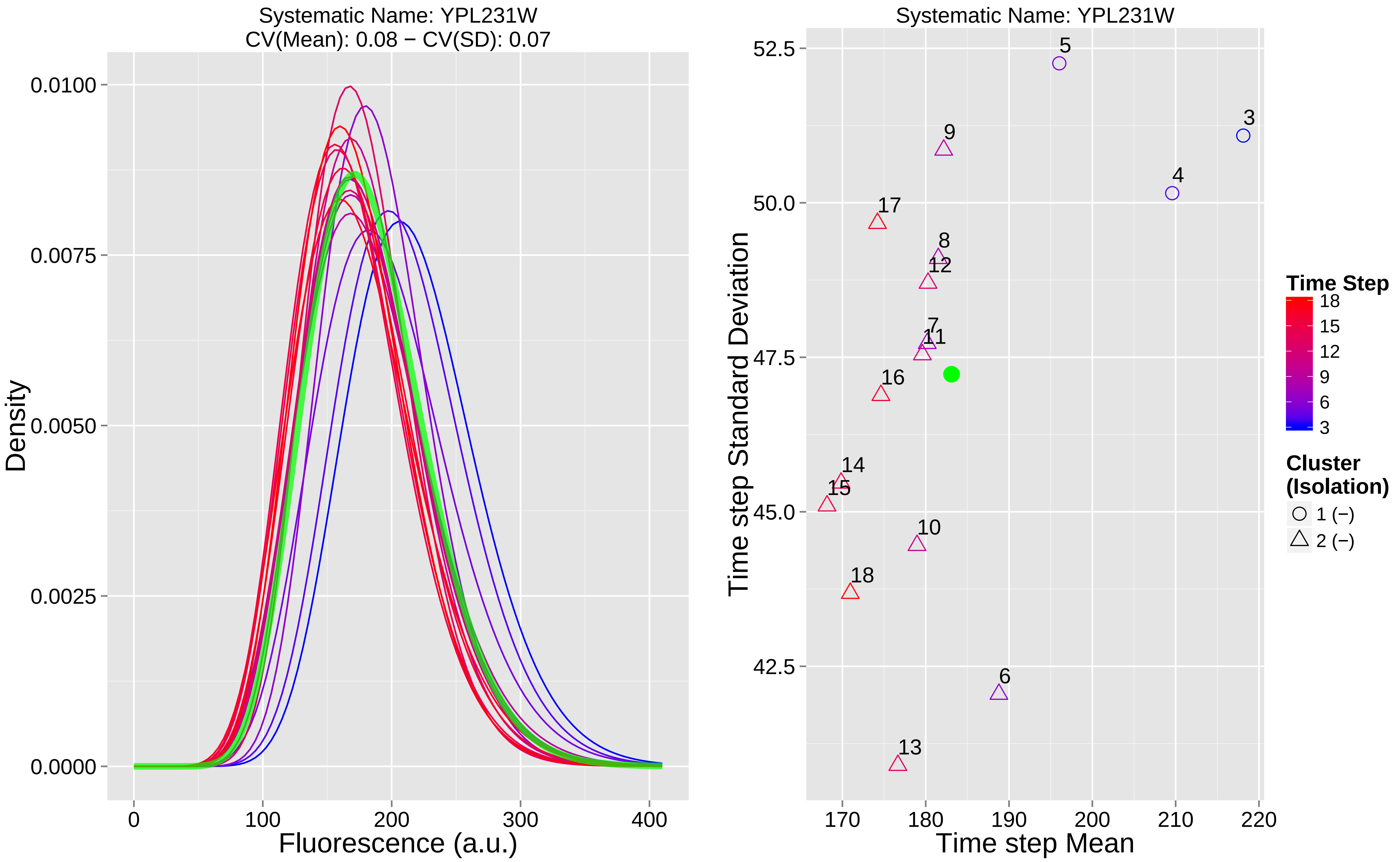

Systematic Name: YPL231W
CV(Mean): 0.08 − CV(SD): 0.07
Density
0.0100
0.0075
0.0050
0.0025
0.0000
0
100
200
300
400
Fluorescence (a.u.)
Systematic Name: YPL231W
Time step Standard Deviation
52.5
50.0
47.5
45.0
42.5
170
180
190
200
210
220
Time step Mean
5
3
9
4
17
8
12
7
11
16
14
15
10
18
6
13
Time Step
18
15
12
9
6
3
Cluster
(Isolation)
1 (−)
2 (−)

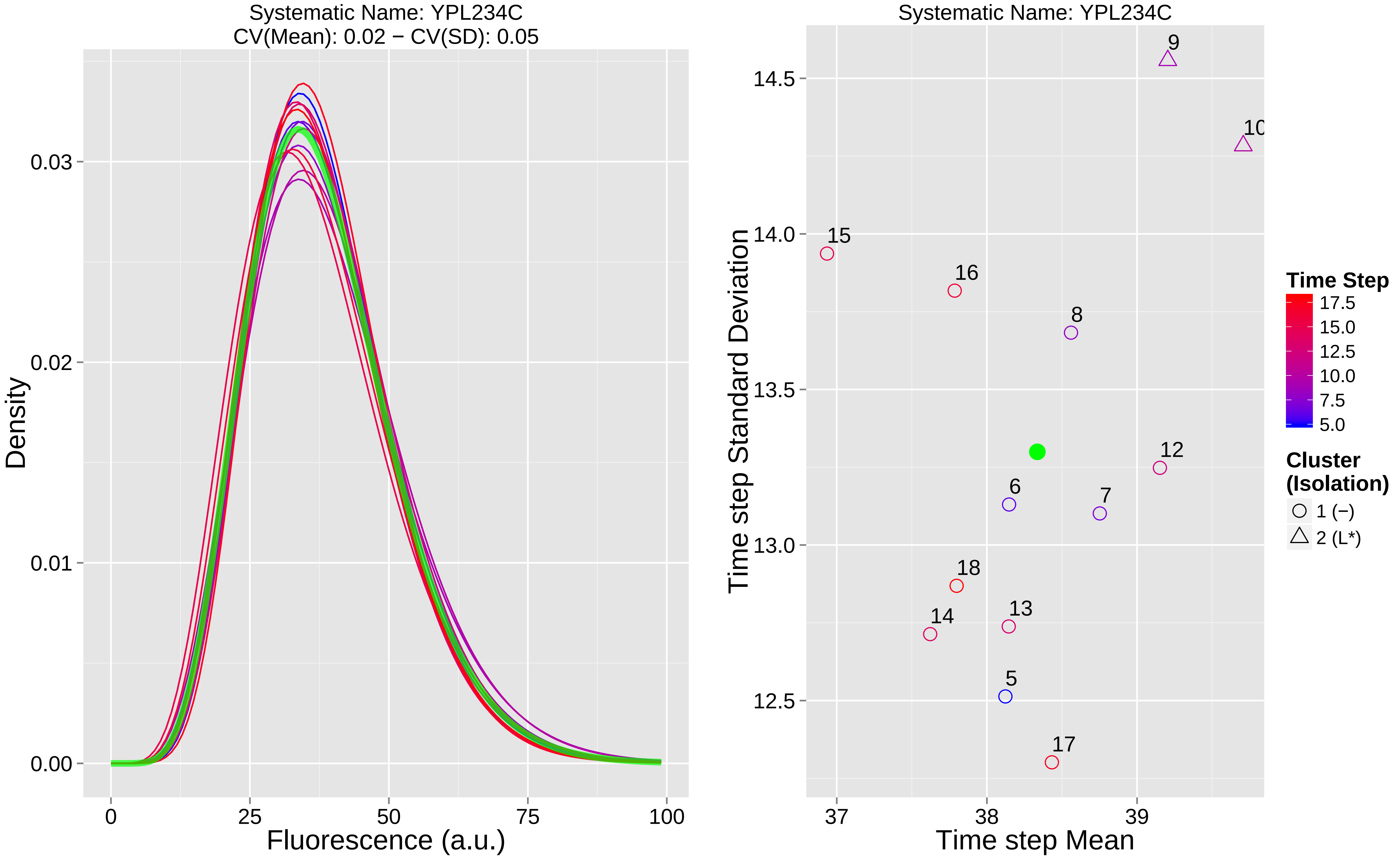

Systematic Name: YPL234C
CV(Mean): 0.02 − CV(SD): 0.05
Density
0.03
0.02
0.01
0.00
0
25
50
75
100
Fluorescence (a.u.)
Systematic Name: YPL234C
Time step Standard Deviation
14.5
14.0
13.5
13.0
12.5
37
38
39
Time step Mean
9
10
15
16
8
12
6
7
18
14
13
5
17
Time Step
17.5
15.0
12.5
10.0
7.5
5.0
Cluster
(Isolation)
1 (−)
2 (L*)

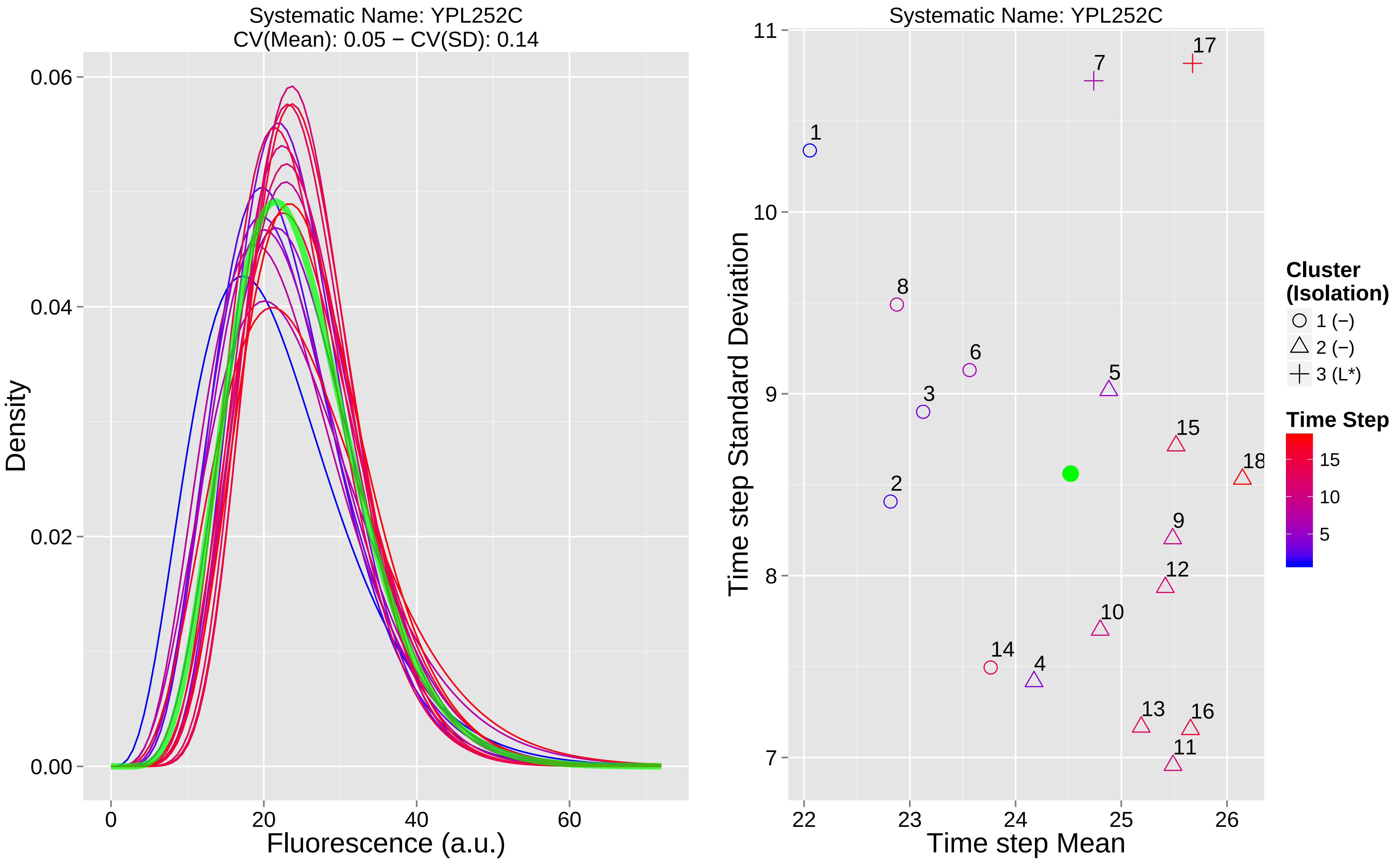

Systematic Name: YPL252C
CV(Mean): 0.05 − CV(SD): 0.14
Density
0.00
0.02
0.04
0.06
0
20
40
60
Fluorescence (a.u.)
Systematic Name: YPL252C
Time step Standard Deviation
Time step Mean
7
8
9
10
11
22
23
24
25
26
1
2
3
4
5
6
7
8
9
10
11
12
13
14
15
16
17
18
Cluster (Isolation)
1 (−)
2 (−)
3 (L*)
Time Step
5
10
15

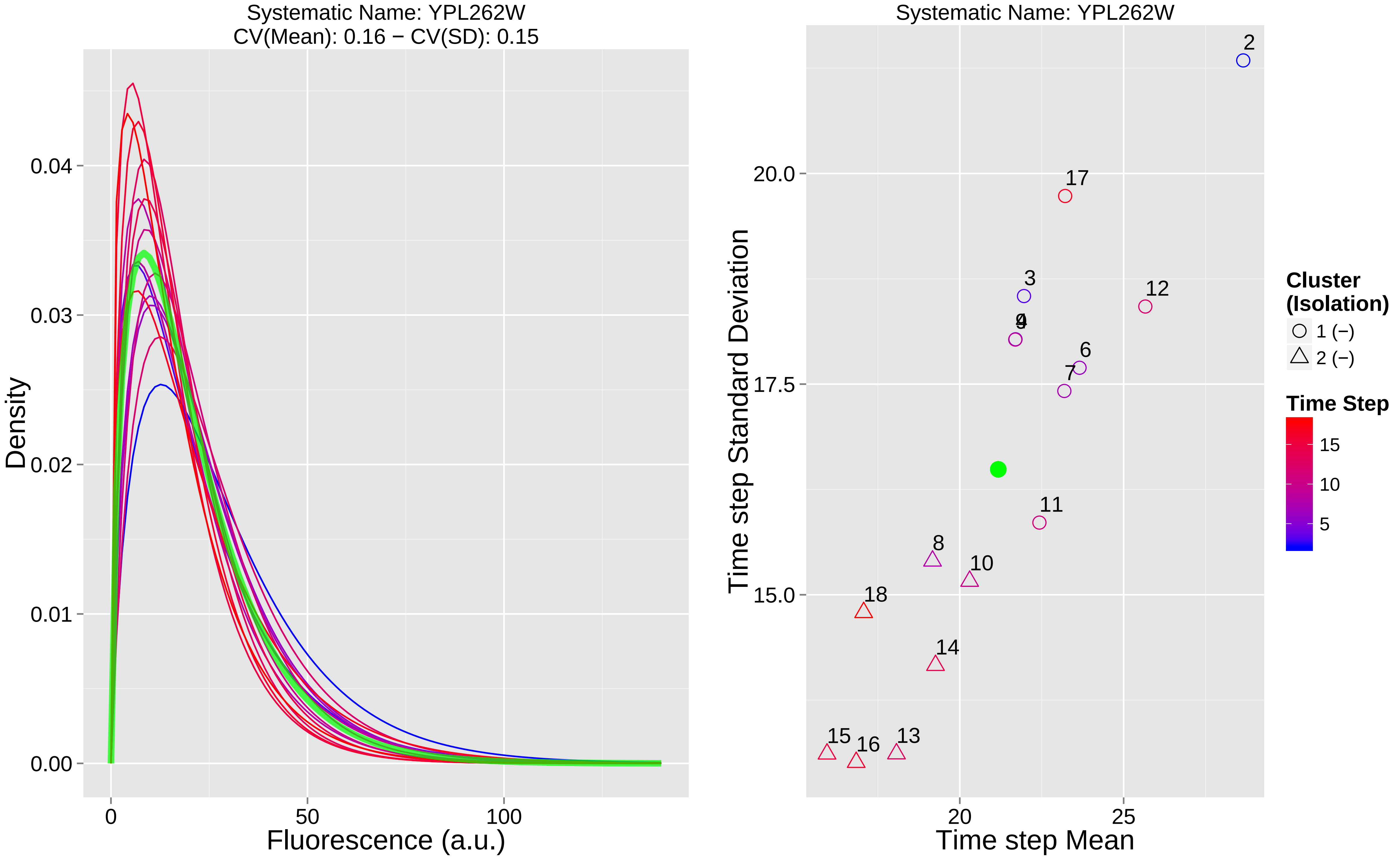

Systematic Name: YPL262W
CV(Mean): 0.16 − CV(SD): 0.15
Density
0.00
0.01
0.02
0.03
0.04
0
50
100
Fluorescence (a.u.)
Systematic Name: YPL262W
Time step Standard Deviation
15.0
17.5
20.0
20
25
Time step Mean
2
17
3
12
4
6
7
11
8
10
18
14
15
16
13
Cluster
(Isolation)
1 (−)
2 (−)
Time Step
15
10
5

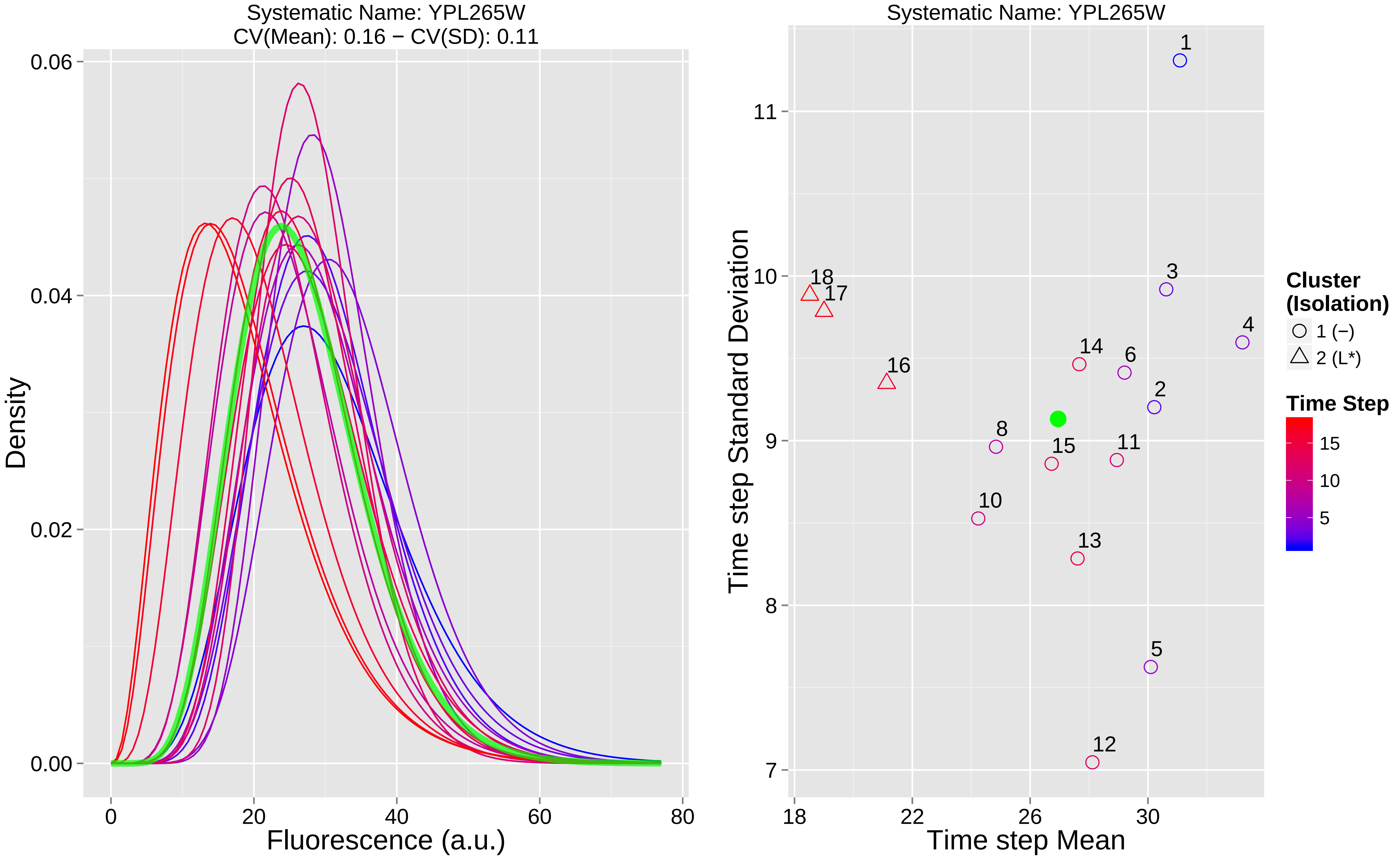

Systematic Name: YPL265W
CV(Mean): 0.16 − CV(SD): 0.11
Density
Fluorescence (a.u.)
0.06
0.04
0.02
0.00
0
20
40
60
80
Systematic Name: YPL265W
Time step Standard Deviation
Time step Mean
11
10
9
8
7
18
22
26
30
Cluster
(Isolation)
1 (−)
2 (L*)
Time Step
15
10
5

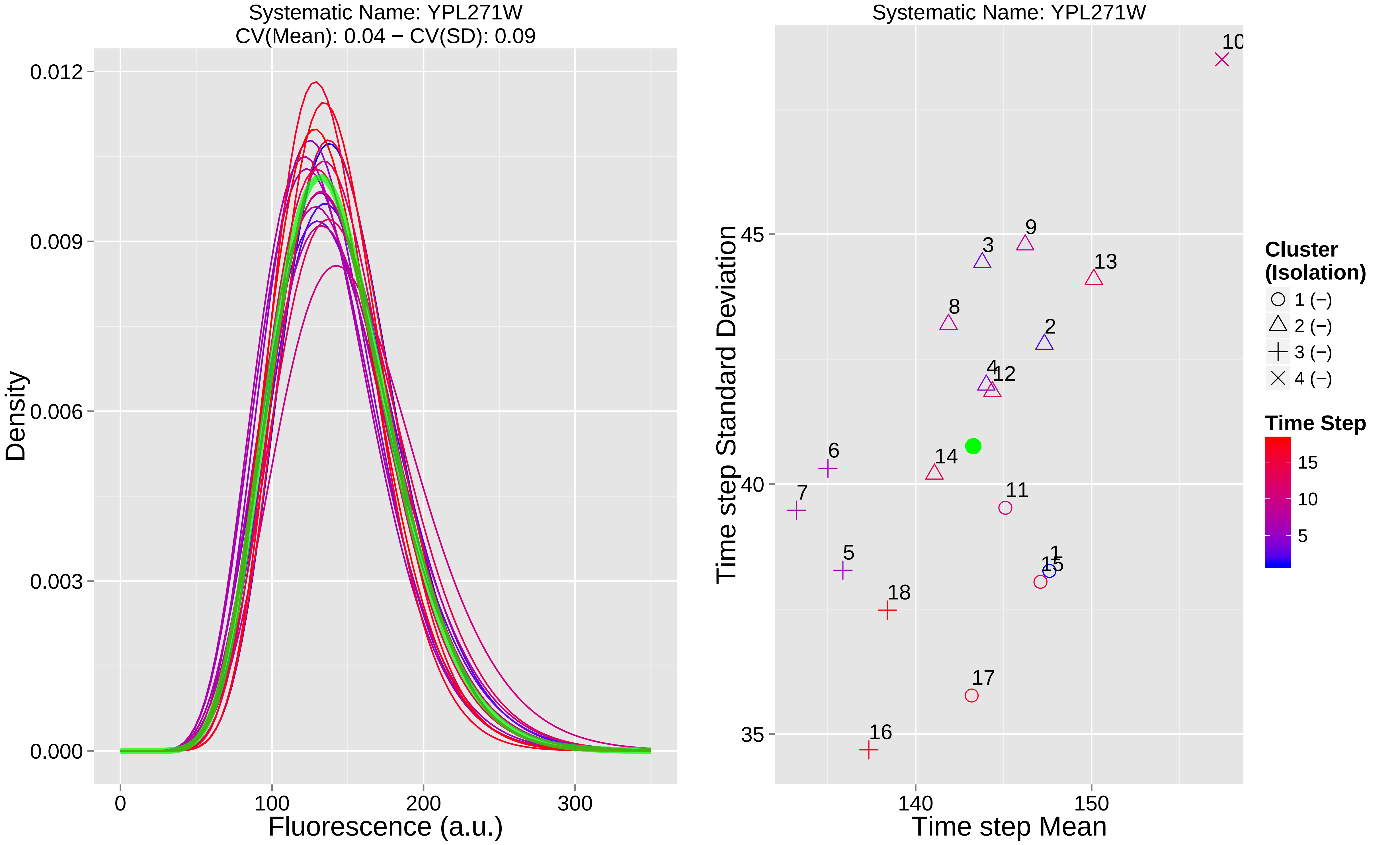
Systematic Name: YPL271W
CV(Mean): 0.04 − CV(SD): 0.09
Density
0.012
0.009
0.006
0.003
0.000
0
100
200
300
Fluorescence (a.u.)
Systematic Name: YPL271W
Time step Standard Deviation
45
40
35
140
150
Time step Mean
1
2
3
4
5
6
7
8
9
10
11
12
13
14
15
16
17
18
Cluster (Isolation)
1 (−)
2 (−)
3 (−)
4 (−)
Time Step
15
10
5

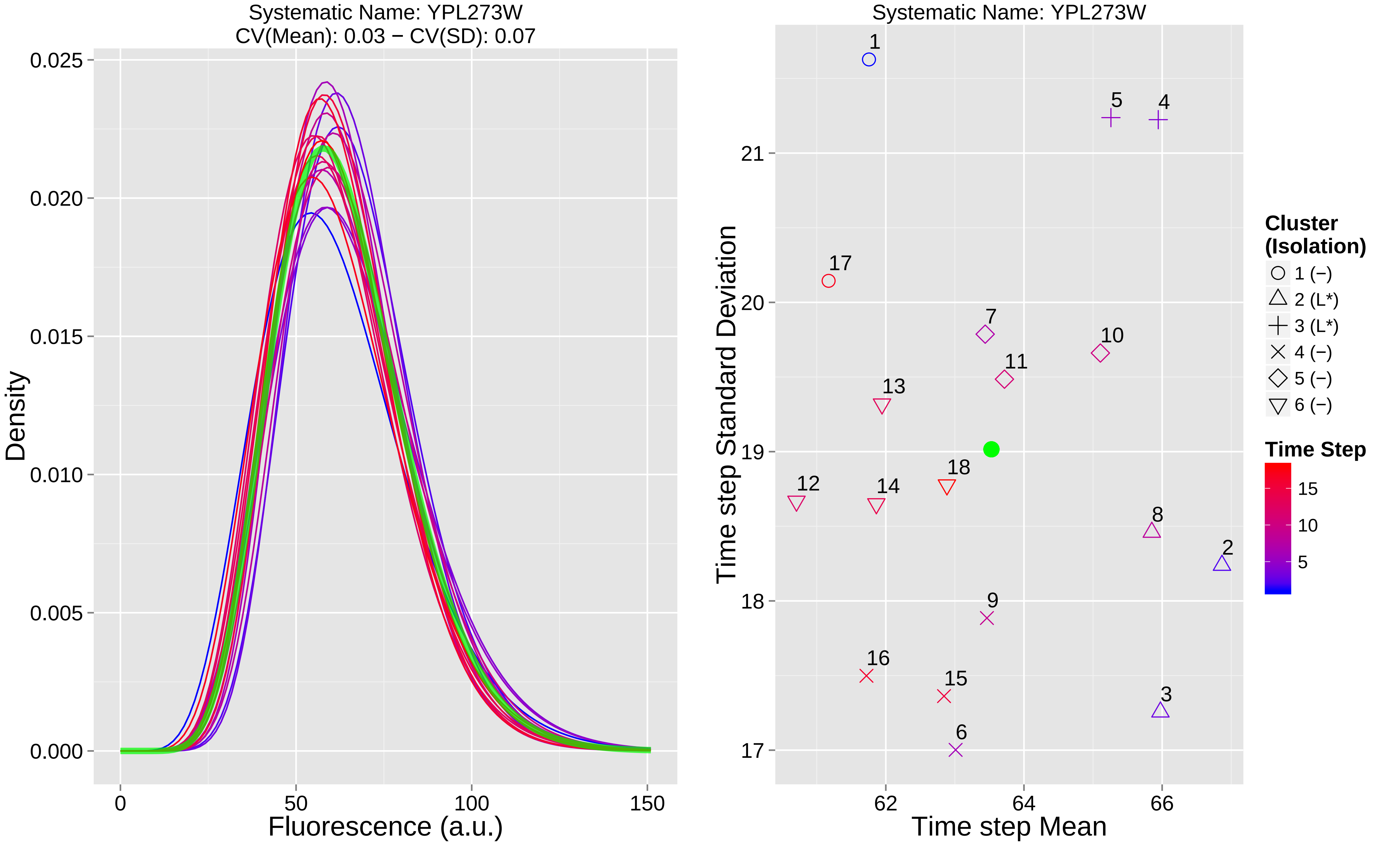

Systematic Name: YPL273W
CV(Mean): 0.03 − CV(SD): 0.07
Density
0.025
0.020
0.015
0.010
0.005
0.000
0
50
100
150
Fluorescence (a.u.)
Systematic Name: YPL273W
Time step Standard Deviation
21
20
19
18
17
62
64
66
Time step Mean
1
2
3
4
5
6
7
8
9
10
11
12
13
14
15
16
17
18
Cluster (Isolation)
1 (−)
2 (L*)
3 (L*)
4 (−)
5 (−)
6 (−)
Time Step
15
10
5

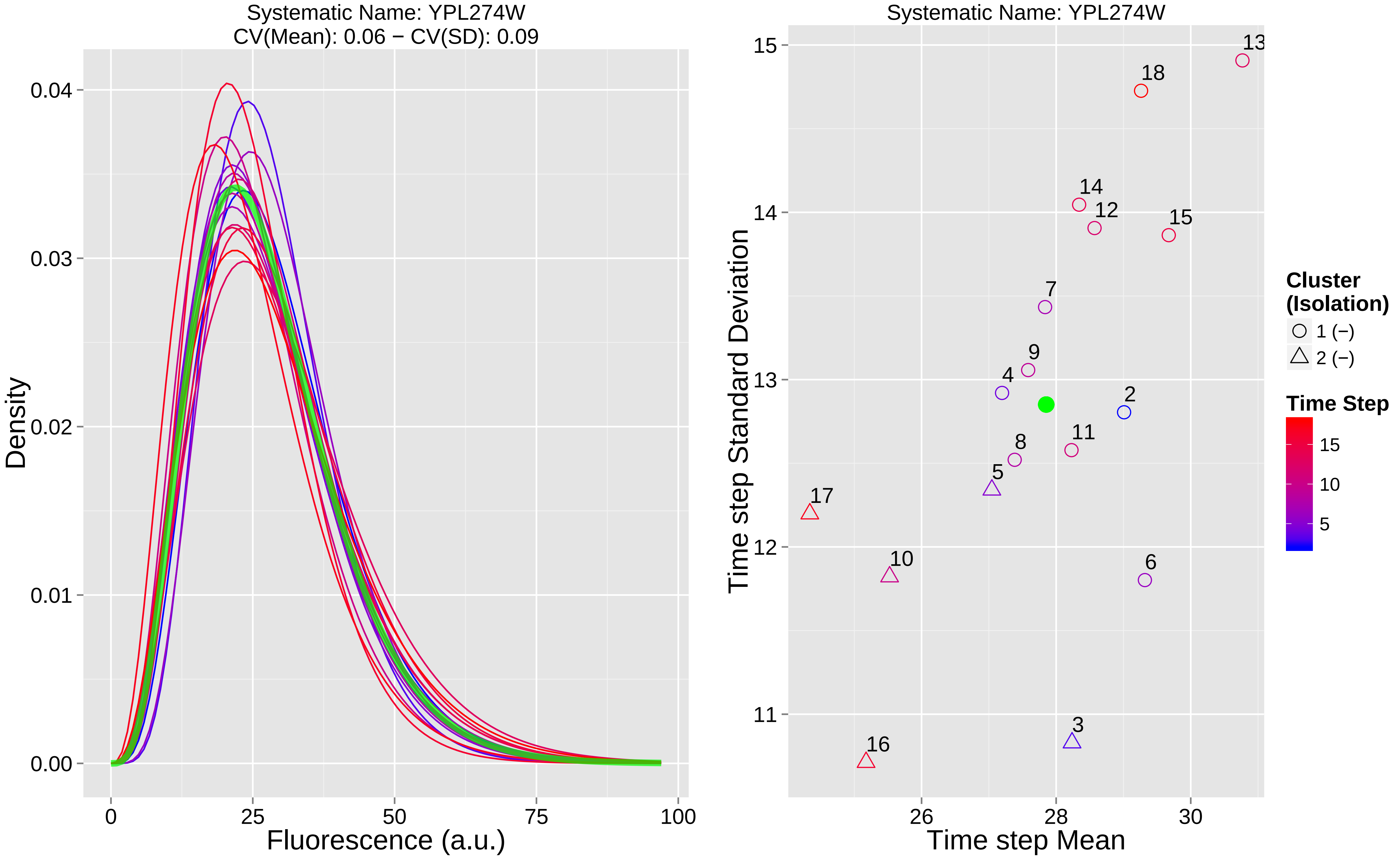

Systematic Name: YPL274W
CV(Mean): 0.06 − CV(SD): 0.09
Density
0.04
0.03
0.02
0.01
0.00
0
25
50
75
100
Fluorescence (a.u.)
Systematic Name: YPL274W
Time step Standard Deviation
15
14
13
12
11
26
28
30
Time step Mean
13
18
14
12
15
7
9
4
2
11
8
5
17
10
6
16
3
Cluster (Isolation)
1 (−)
2 (−)
Time Step
15
10
5

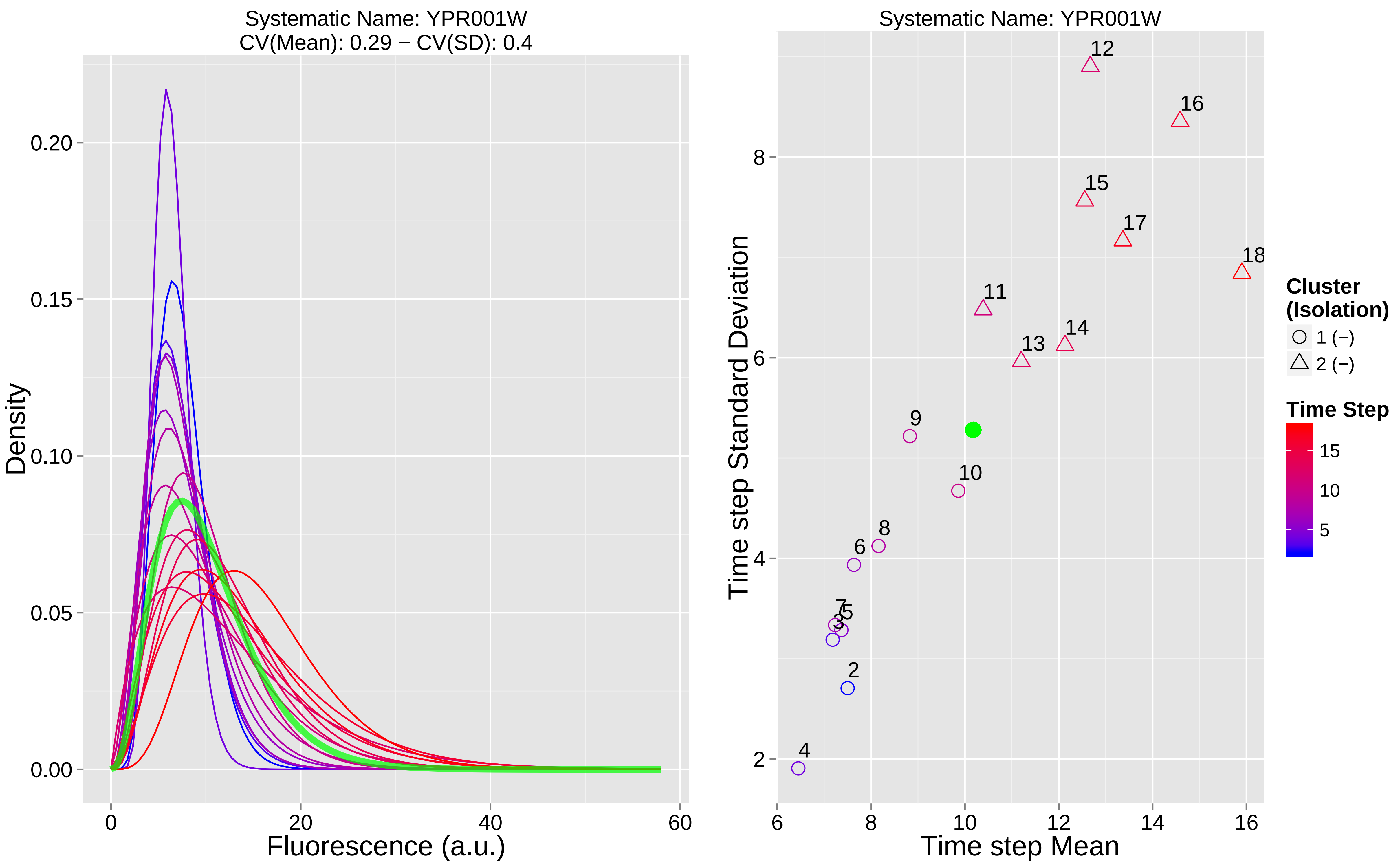

Systematic Name: YPR001W
CV(Mean): 0.29 − CV(SD): 0.4
Density
Fluorescence (a.u.)
Systematic Name: YPR001W
Time step Standard Deviation
Time step Mean
Cluster (Isolation)
1 (−)
2 (−)
Time Step

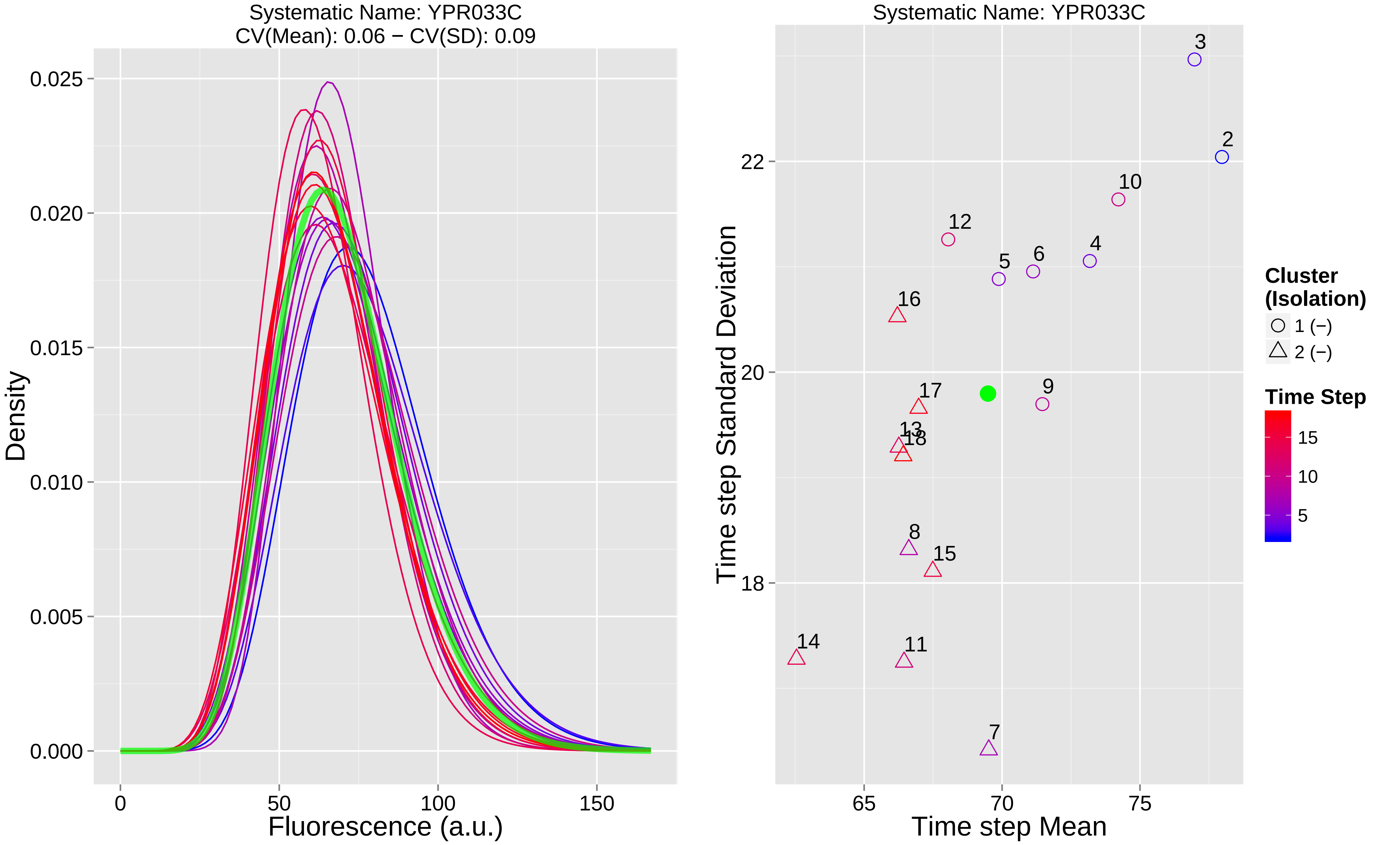

Systematic Name: YPR033C
CV(Mean): 0.06 − CV(SD): 0.09
Density
Fluorescence (a.u.)
0.000
0.005
0.010
0.015
0.020
0.025
0
50
100
150
Systematic Name: YPR033C
Time step Standard Deviation
Time step Mean
18
20
22
65
70
75
3
2
10
12
4
6
5
16
17
9
13
18
8
15
14
11
7
Cluster (Isolation)
1 (−)
2 (−)
Time Step
15
10
5

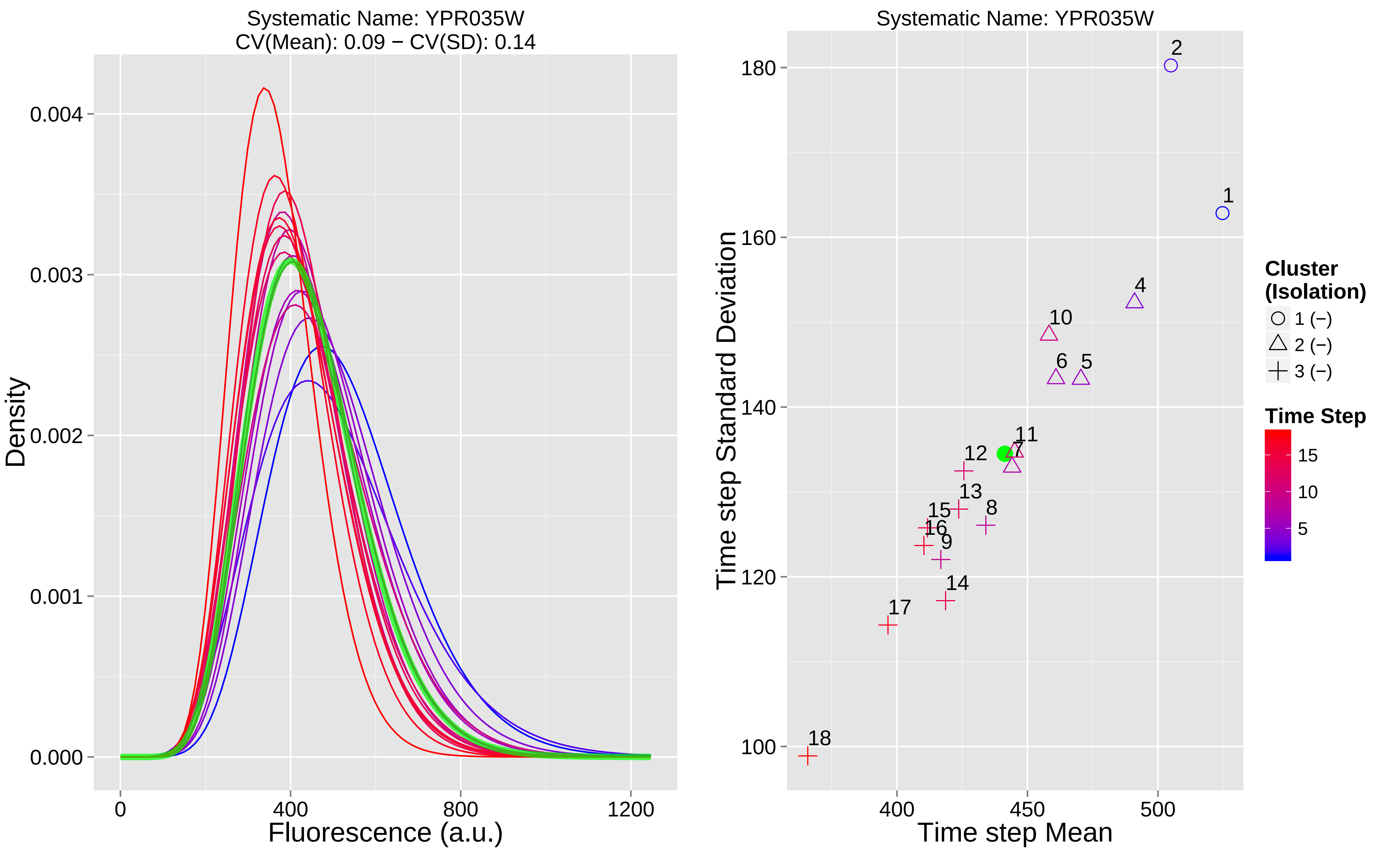

Systematic Name: YPR035W
CV(Mean): 0.09 − CV(SD): 0.14
Density
Fluorescence (a.u.)
0.000
0.001
0.002
0.003
0.004
0
400
800
1200
Systematic Name: YPR035W
Time step Standard Deviation
Time step Mean
100
120
140
160
180
400
450
500
Cluster
(Isolation)
1 (−)
2 (−)
3 (−)
Time Step
15
10
5

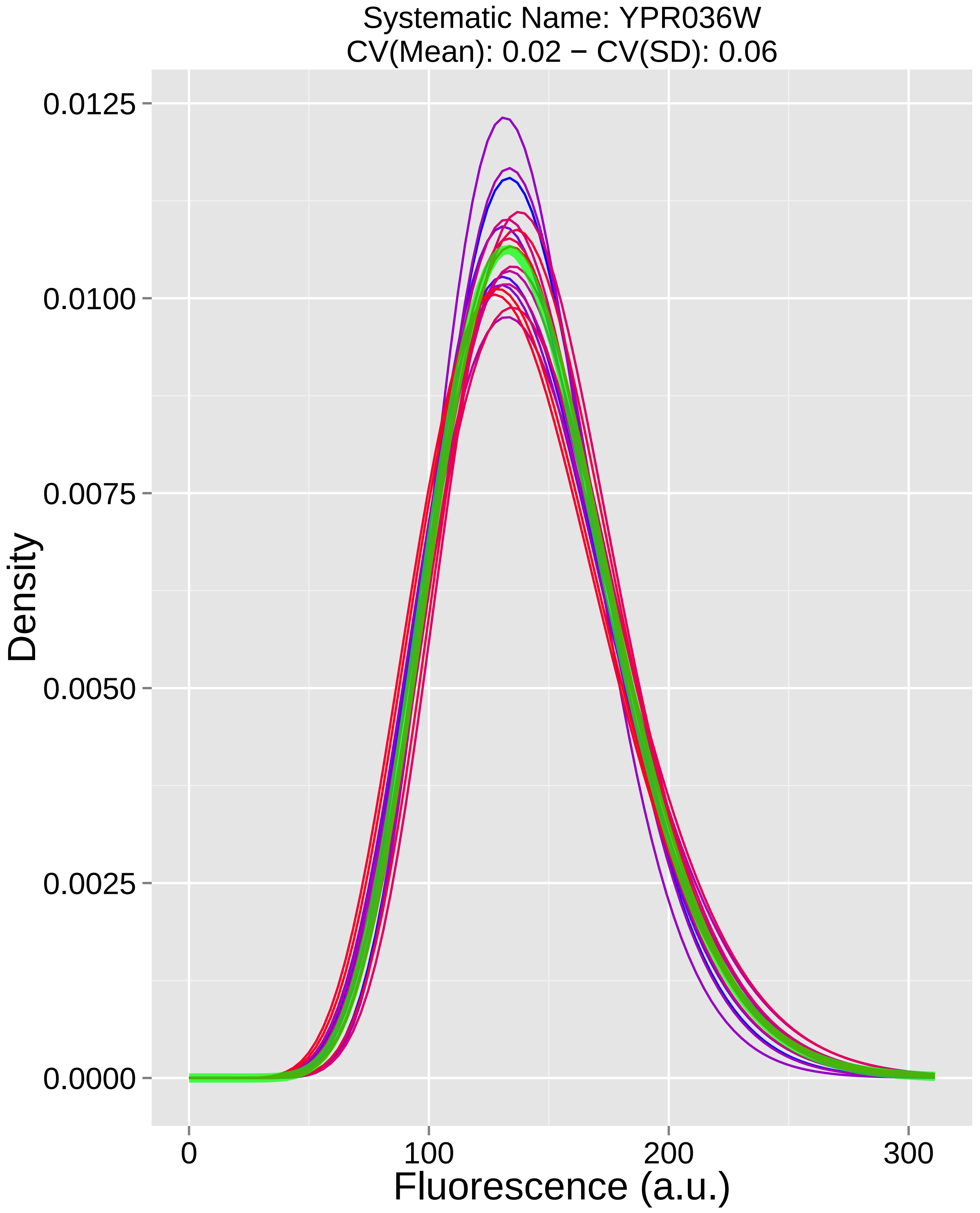
Systematic Name: YPR036W
CV(Mean): 0.02 − CV(SD): 0.06
Density
0.0125
0.0100
0.0075
0.0050
0.0025
0.0000
0
100
200
300
Fluorescence (a.u.)

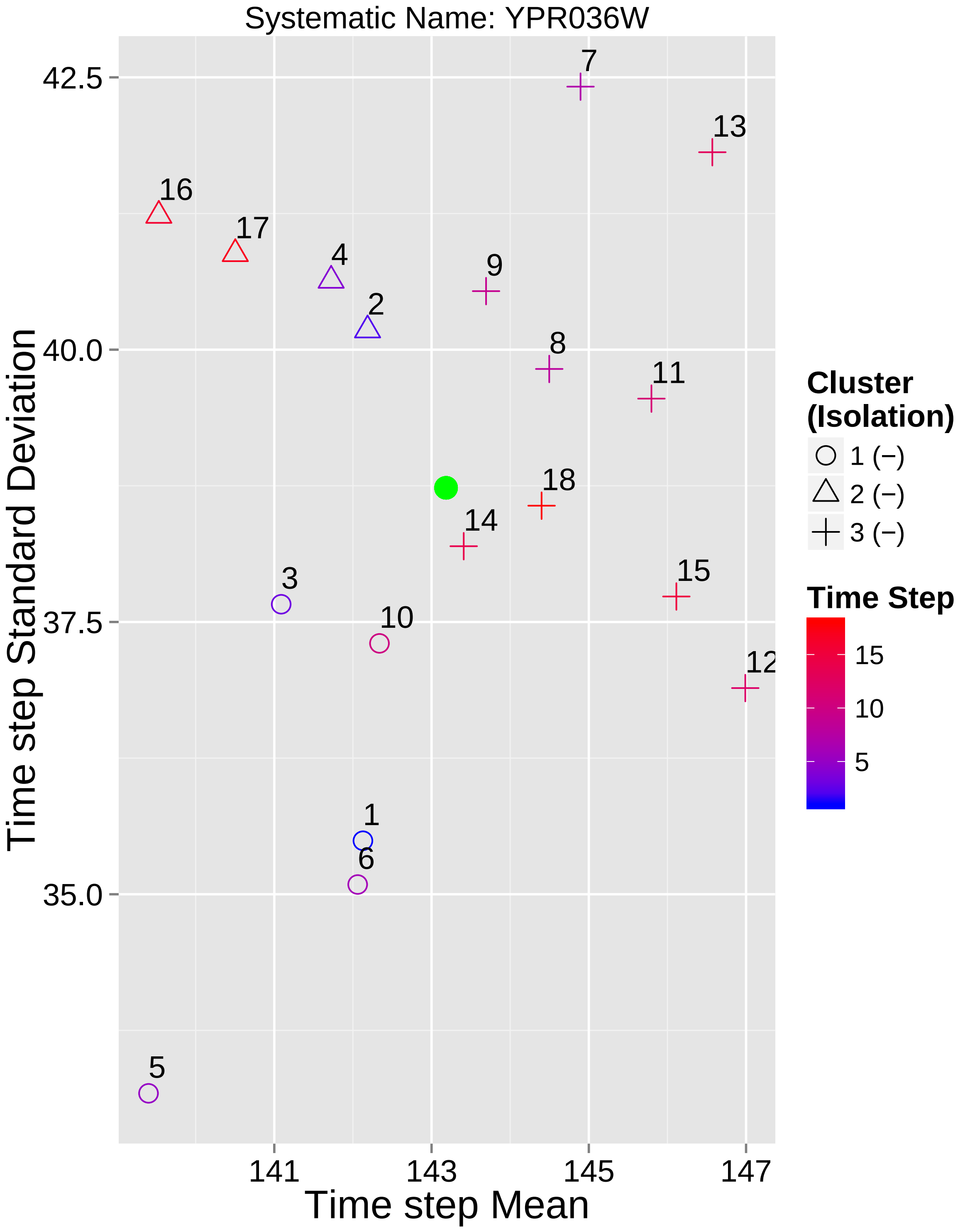
Systematic Name: YPR036W
Time step Standard Deviation
42.5
40.0
37.5
35.0
141
143
145
147
Time step Mean
16
17
4
2
9
7
13
8
11
18
14
3
10
15
12
1
6
5
Cluster (Isolation)
1 (−)
2 (−)
3 (−)
Time Step
15
10
5

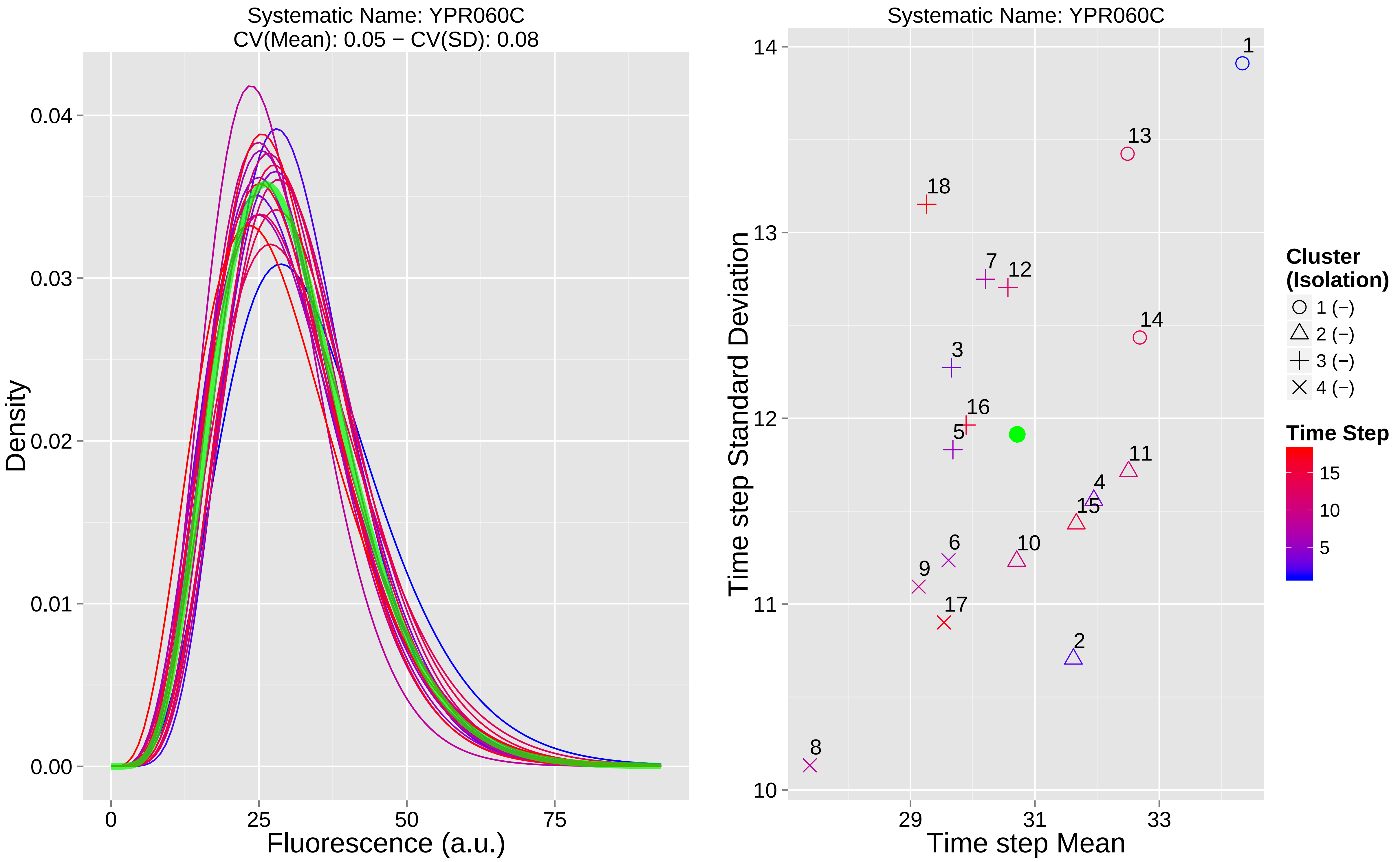

Systematic Name: YPR060C
CV(Mean): 0.05 − CV(SD): 0.08
Density
Fluorescence (a.u.)
0.00
0.01
0.02
0.03
0.04
0
25
50
75
Systematic Name: YPR060C
Time step Standard Deviation
Time step Mean
10
11
12
13
14
29
31
33
1
2
3
4
5
6
7
8
9
10
11
12
13
14
15
16
17
18
Cluster (Isolation)
1 (−)
2 (−)
3 (−)
4 (−)
Time Step
15
10
5

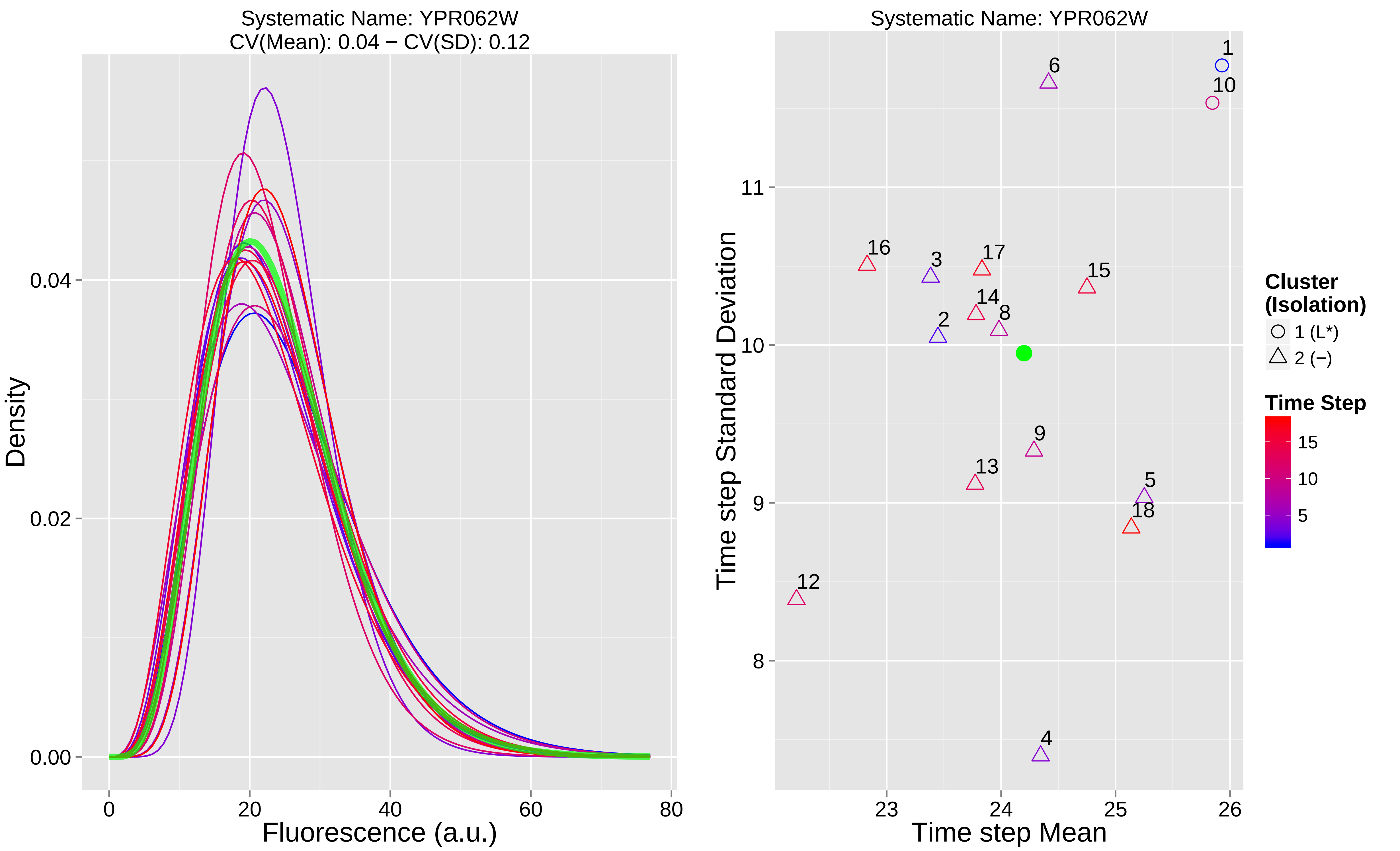
Systematic Name: YPR062W
CV(Mean): 0.04 − CV(SD): 0.12
Density
Fluorescence (a.u.)
0.00
0.02
0.04
0
20
40
60
80
Systematic Name: YPR062W
Time step Standard Deviation
Time step Mean
8
9
10
11
23
24
25
26
1
2
3
4
5
6
8
9
10
12
13
14
15
16
17
18
Cluster
(Isolation)
1 (L*)
2 (−)
Time Step
15
10
5

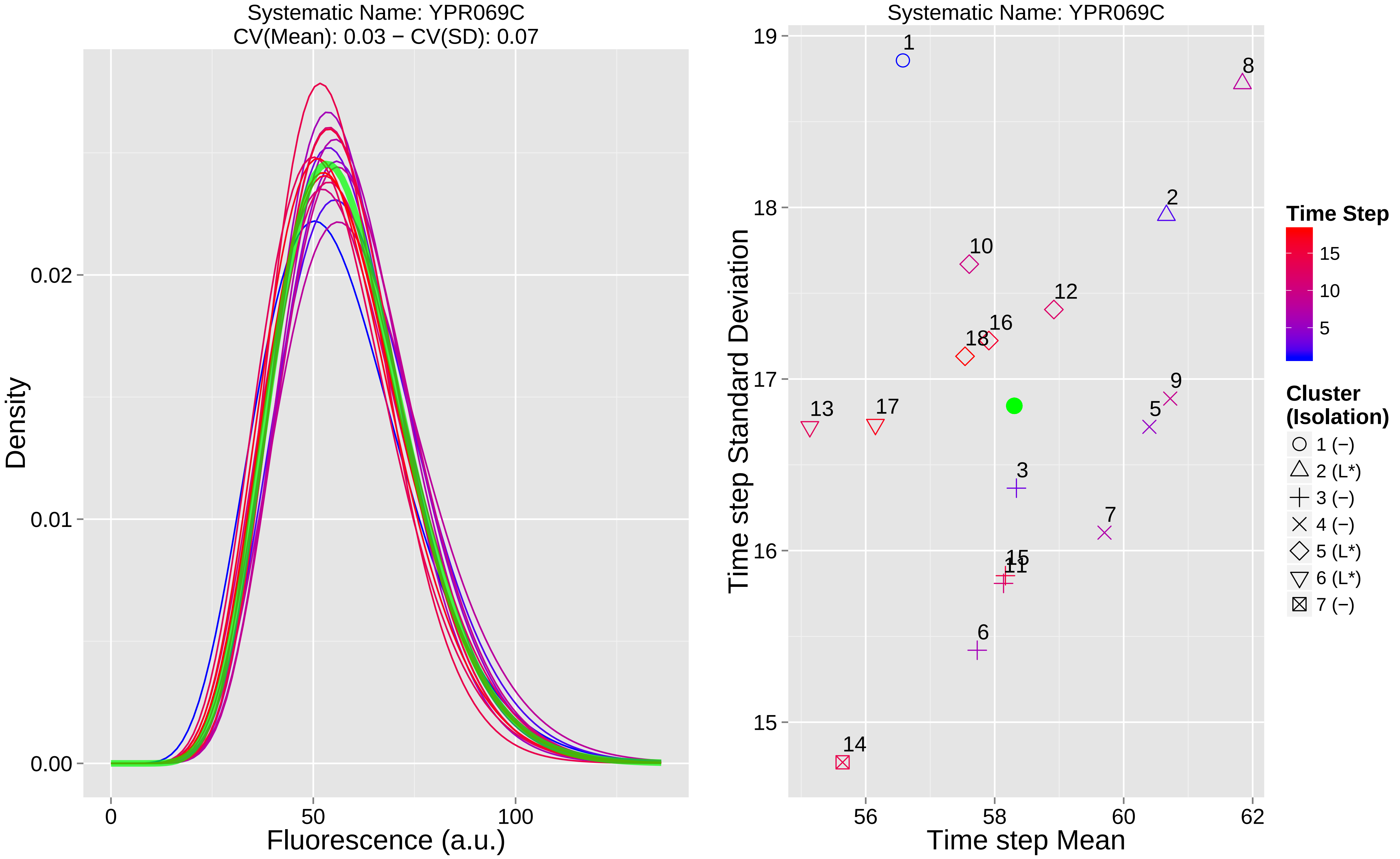

Systematic Name: YPR069C
CV(Mean): 0.03 − CV(SD): 0.07
Density
0.00
0.01
0.02
0
50
100
Fluorescence (a.u.)
Systematic Name: YPR069C
Time step Standard Deviation
Time step Mean
15
16
17
18
19
56
58
60
62
1
2
3
5
6
7
8
9
10
11
12
13
14
15
16
17
18
Time Step
5
10
15
Cluster
(Isolation)
1 (−)
2 (L*)
3 (−)
4 (−)
5 (L*)
6 (L*)
7 (−)

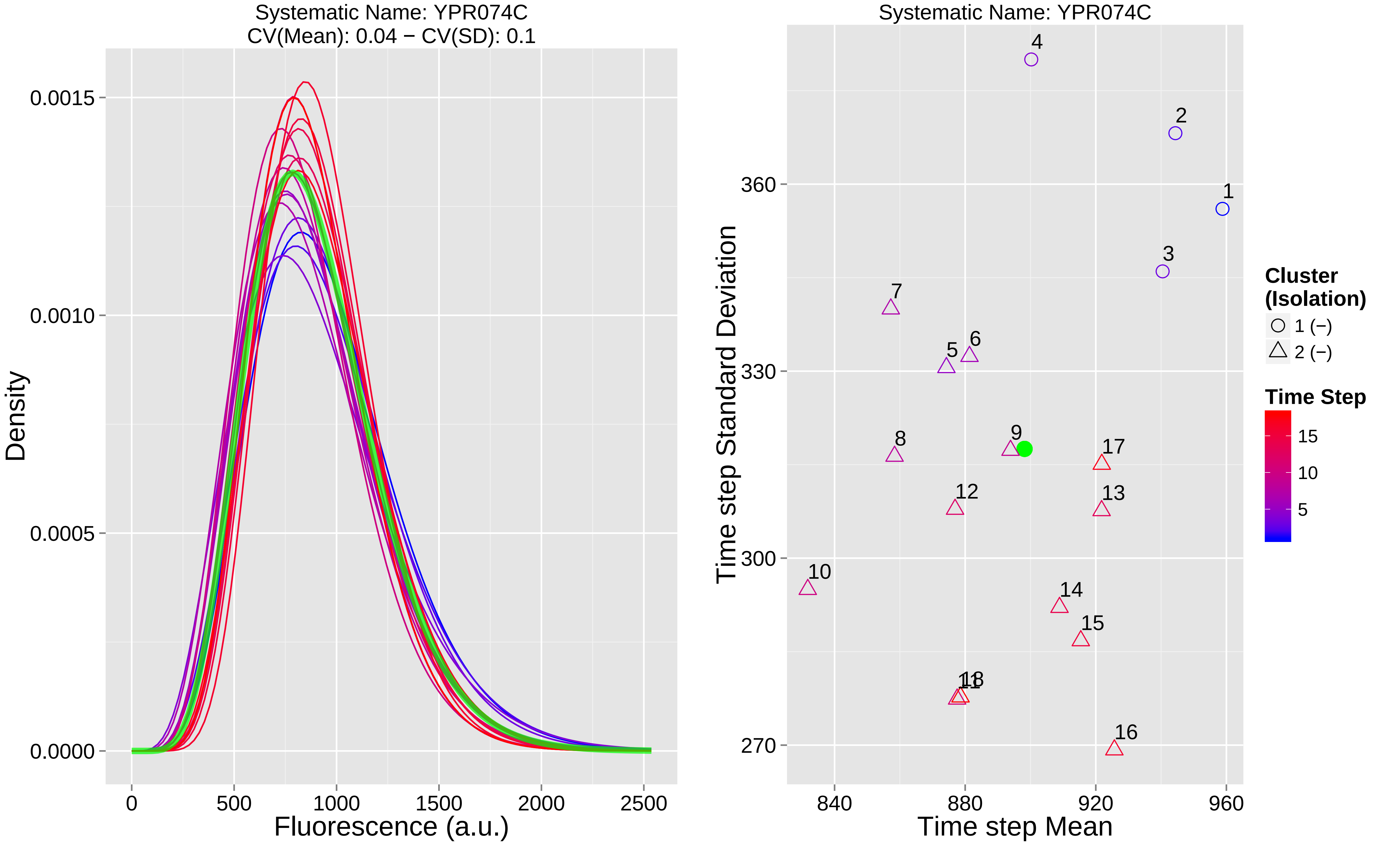
Systematic Name: YPR074C
CV(Mean): 0.04 − CV(SD): 0.1
Density
0.0015
0.0010
0.0005
0.0000
0
500
1000
1500
2000
2500
Fluorescence (a.u.)
Systematic Name: YPR074C
Time step Standard Deviation
360
330
300
270
840
880
920
960
Time step Mean
1
2
3
4
5
6
7
8
9
10
12
13
14
15
16
17
Cluster
(Isolation)
1 (−)
2 (−)
Time Step
15
10
5

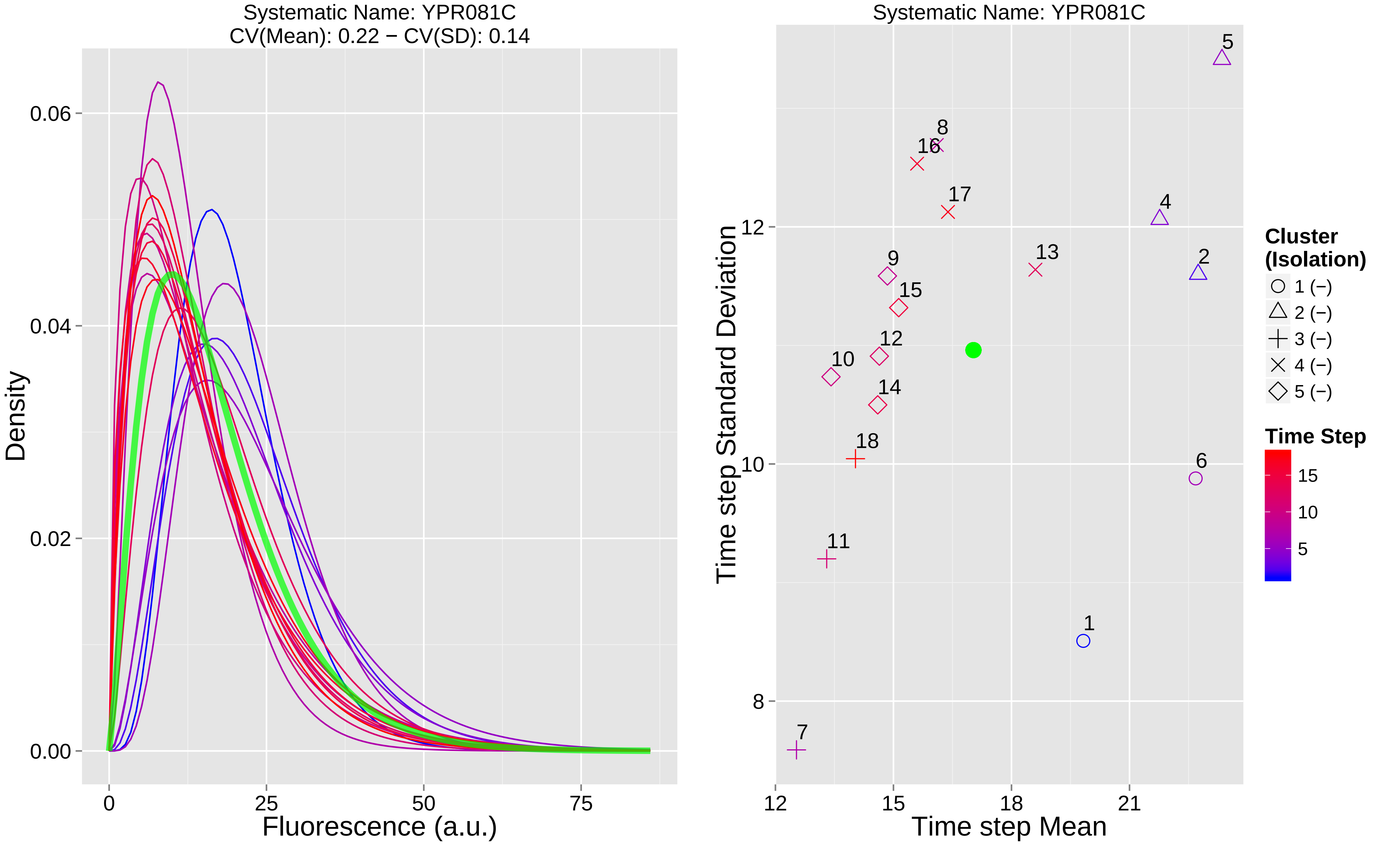
Systematic Name: YPR081C
CV(Mean): 0.22 − CV(SD): 0.14
Density
Fluorescence (a.u.)
0.00
0.02
0.04
0.06
0
25
50
75
Systematic Name: YPR081C
Time step Standard Deviation
Time step Mean
8
10
12
12
15
18
21
Cluster (Isolation)
1 (−)
2 (−)
3 (−)
4 (−)
5 (−)
Time Step
5
10
15

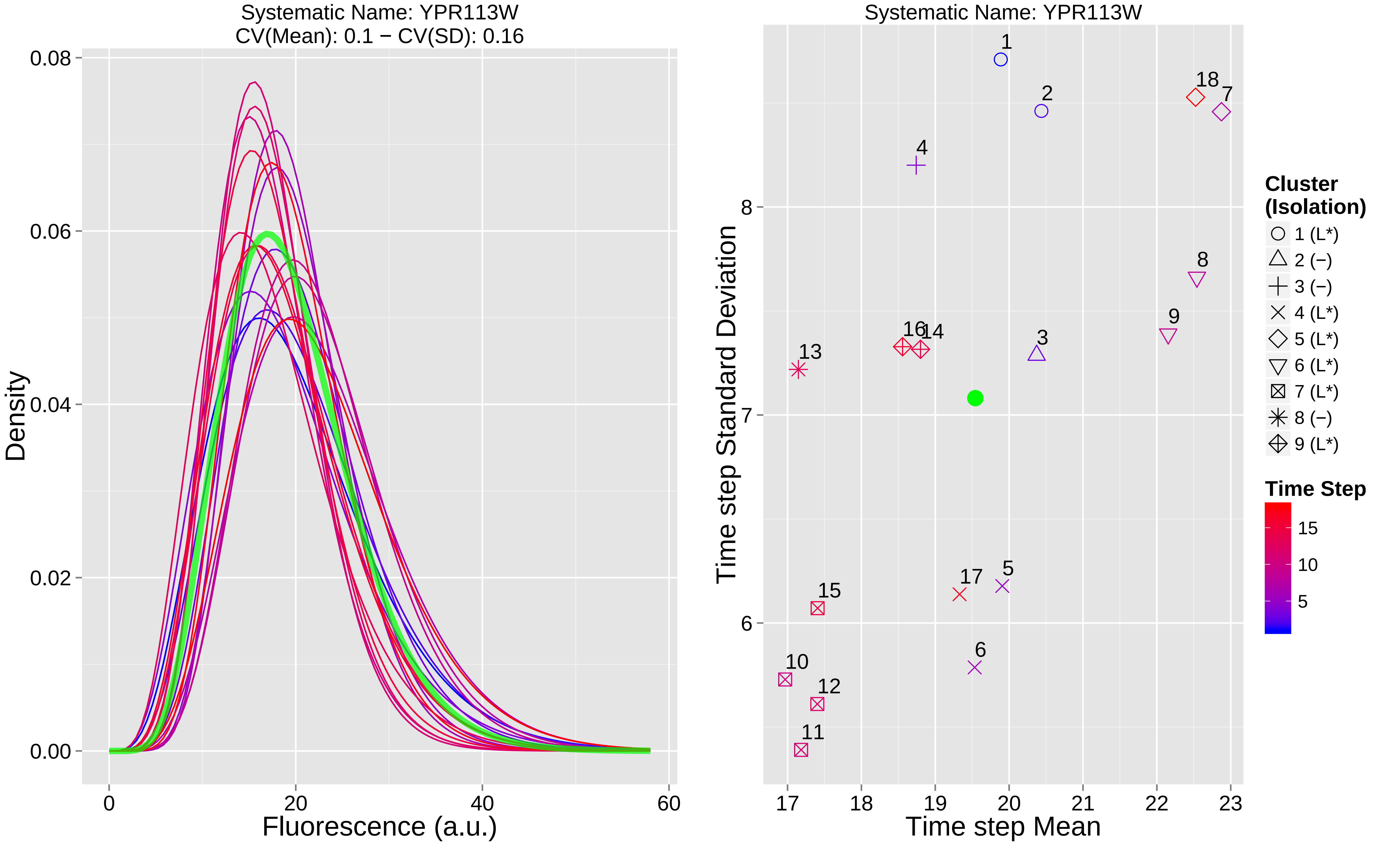

Systematic Name: YPR113W
CV(Mean): 0.1 − CV(SD): 0.16
Density
Fluorescence (a.u.)
0.00
0.02
0.04
0.06
0.08
0
20
40
60
Systematic Name: YPR113W
Time step Standard Deviation
Time step Mean
Cluster (Isolation)
1 (L*)
2 (−)
3 (−)
4 (L*)
5 (L*)
6 (L*)
7 (L*)
8 (−)
9 (L*)
Time Step
15
10
5

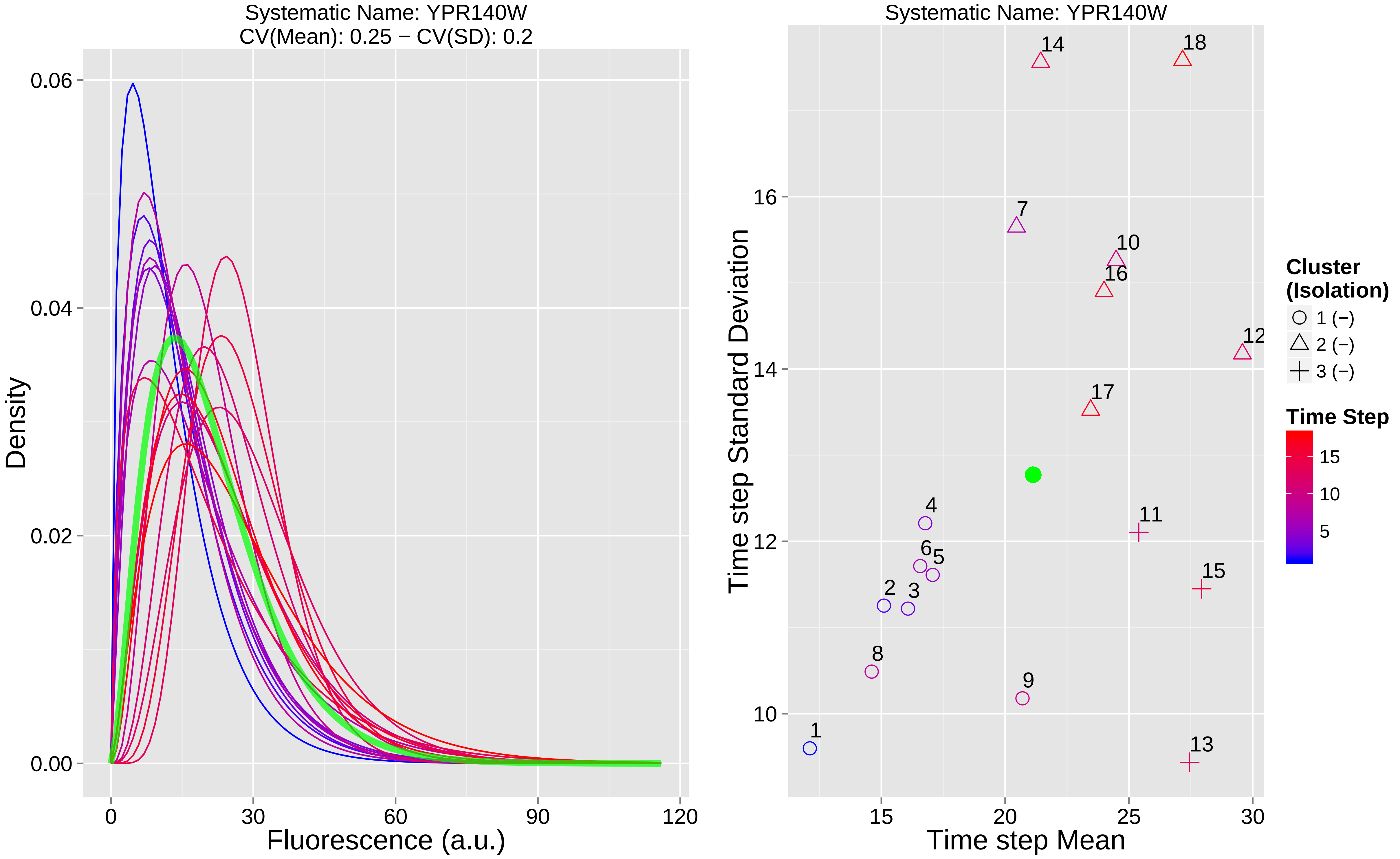
Systematic Name: YPR140W
CV(Mean): 0.25 − CV(SD): 0.2
Density
Fluorescence (a.u.)
0.00
0.02
0.04
0.06
0
30
60
90
120
Systematic Name: YPR140W
Time step Standard Deviation
Time step Mean
10
12
14
16
15
20
25
30
1
2
3
4
65
7
8
9
10
11
12
13
14
15
16
17
18
Cluster (Isolation)
1 (−)
2 (−)
3 (−)
Time Step
5
10
15

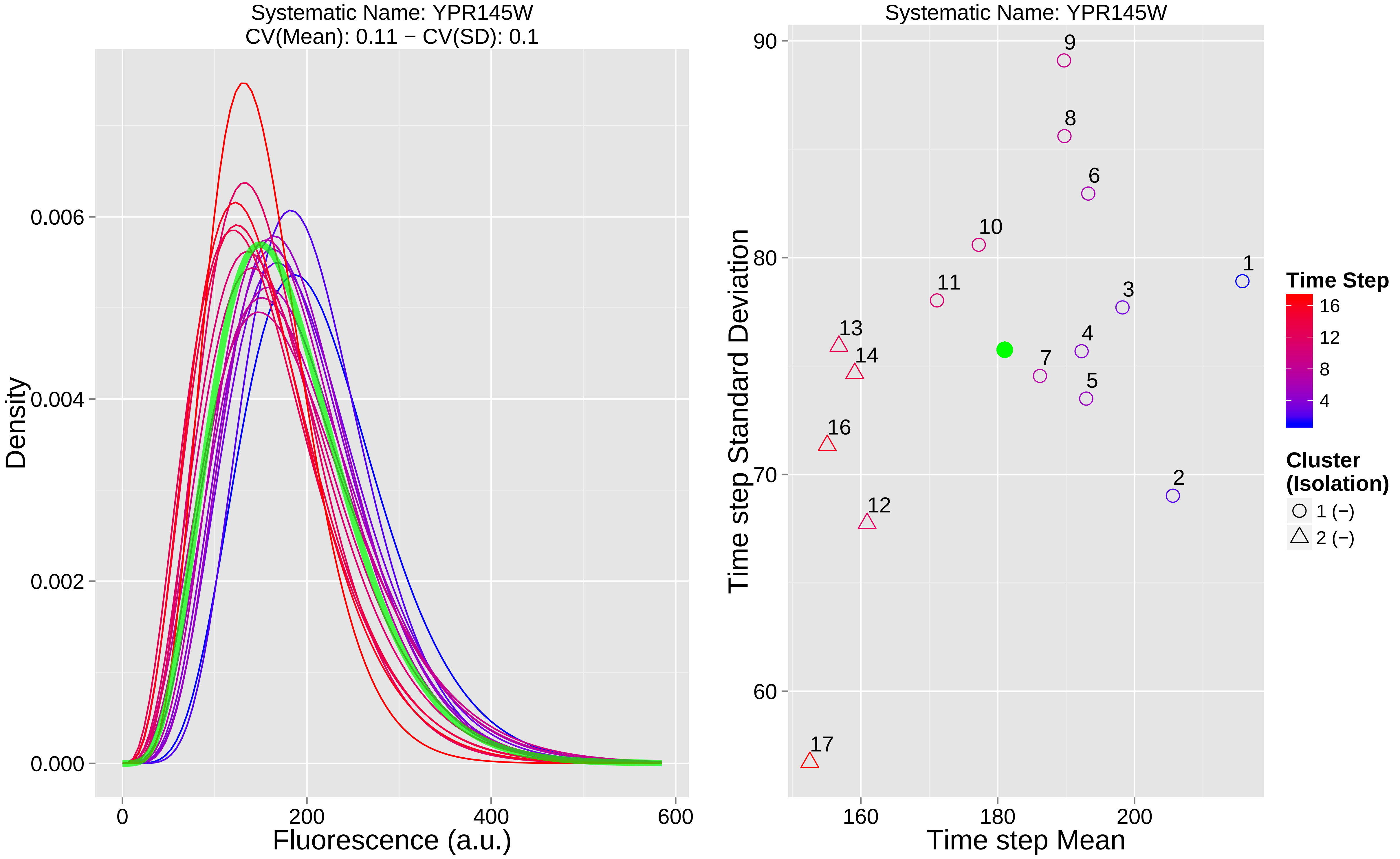
Systematic Name: YPR145W
CV(Mean): 0.11 − CV(SD): 0.1
Density
0.000
0.002
0.004
0.006
0
200
400
600
Fluorescence (a.u.)
Systematic Name: YPR145W
Time step Standard Deviation
60
70
80
90
160
180
200
Time step Mean
1
2
3
4
5
6
7
8
9
10
11
12
13
14
16
17
Time Step
16
12
8
4
Cluster
(Isolation)
1 (−)
2 (−)

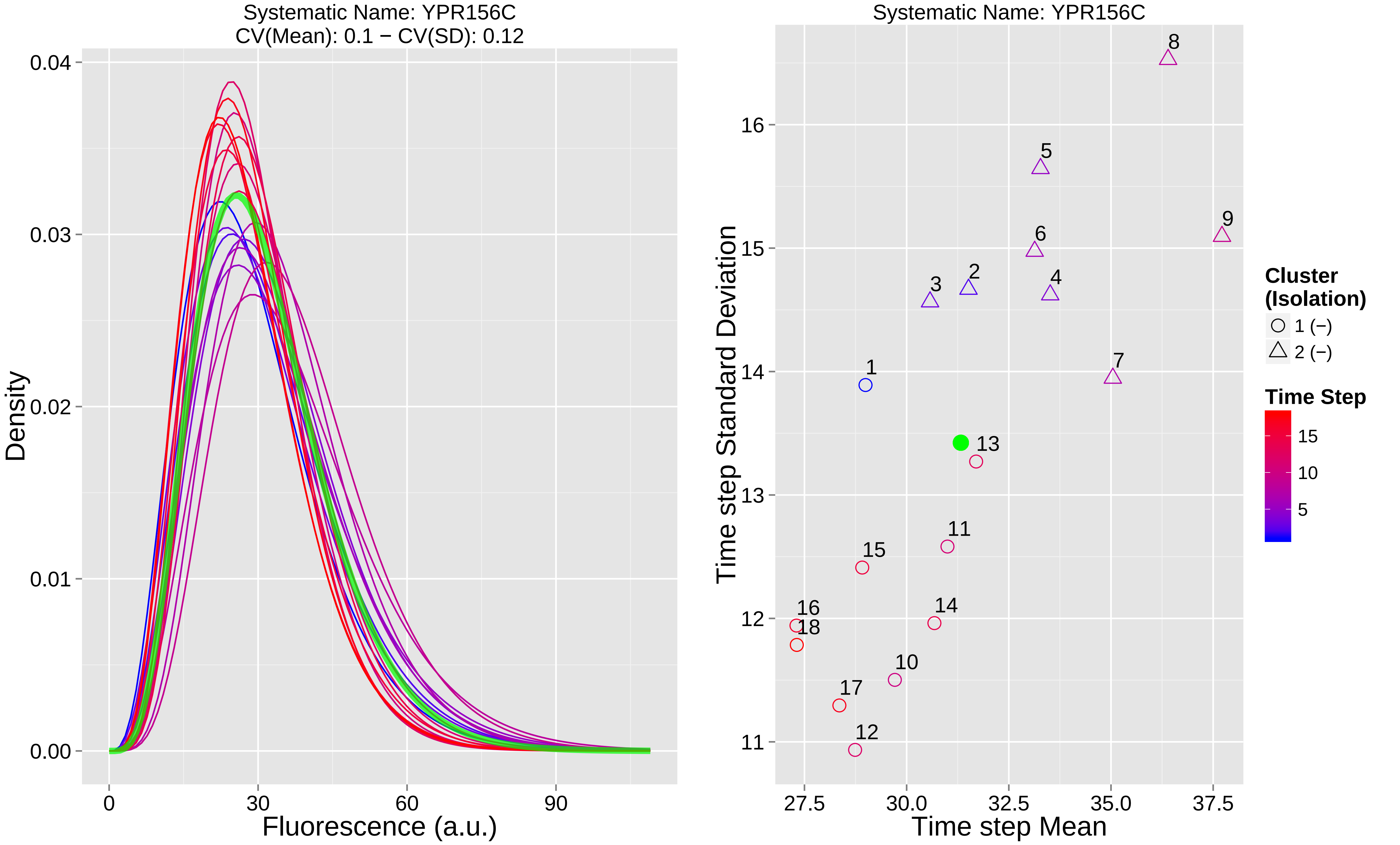

Systematic Name: YPR156C
CV(Mean): 0.1 − CV(SD): 0.12
Density
0.04
0.03
0.02
0.01
0.00
0
30
60
90
Fluorescence (a.u.)
Systematic Name: YPR156C
Time step Standard Deviation
16
15
14
13
12
11
27.5
30.0
32.5
35.0
37.5
Time step Mean
1
2
3
4
5
6
7
8
9
10
11
12
13
14
15
16
17
18
Cluster (Isolation)
1 (−)
2 (−)
Time Step
15
10
5

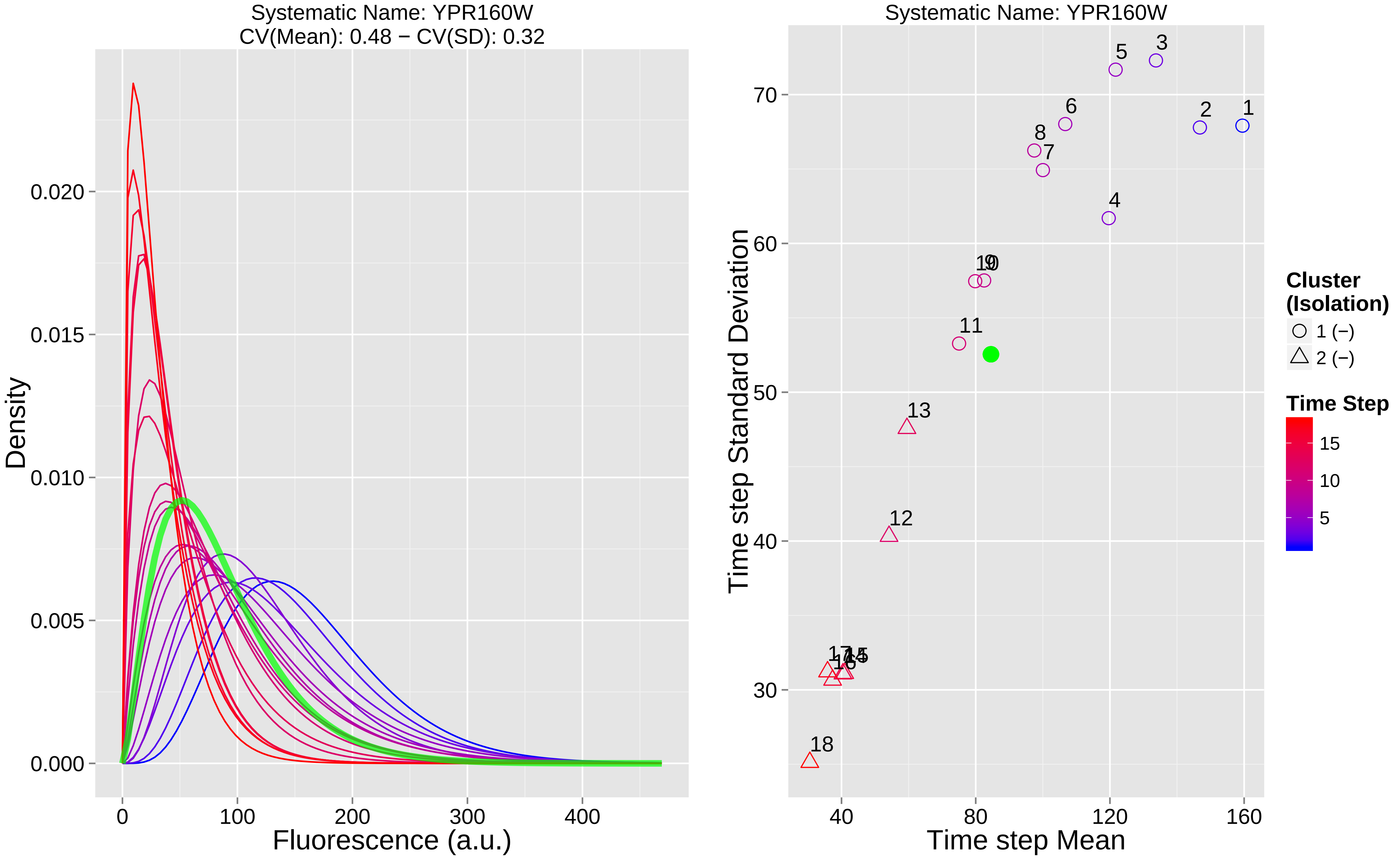
Systematic Name: YPR160W
CV(Mean): 0.48 − CV(SD): 0.32
Density
0.000
0.005
0.010
0.015
0.020
0
100
200
300
400
Fluorescence (a.u.)
Systematic Name: YPR160W
Time step Standard Deviation
30
40
50
60
70
40
80
120
160
Time step Mean
1
2
3
4
5
6
7
8
11
12
13
18
Cluster (Isolation)
1 (−)
2 (−)
Time Step
15
10
5

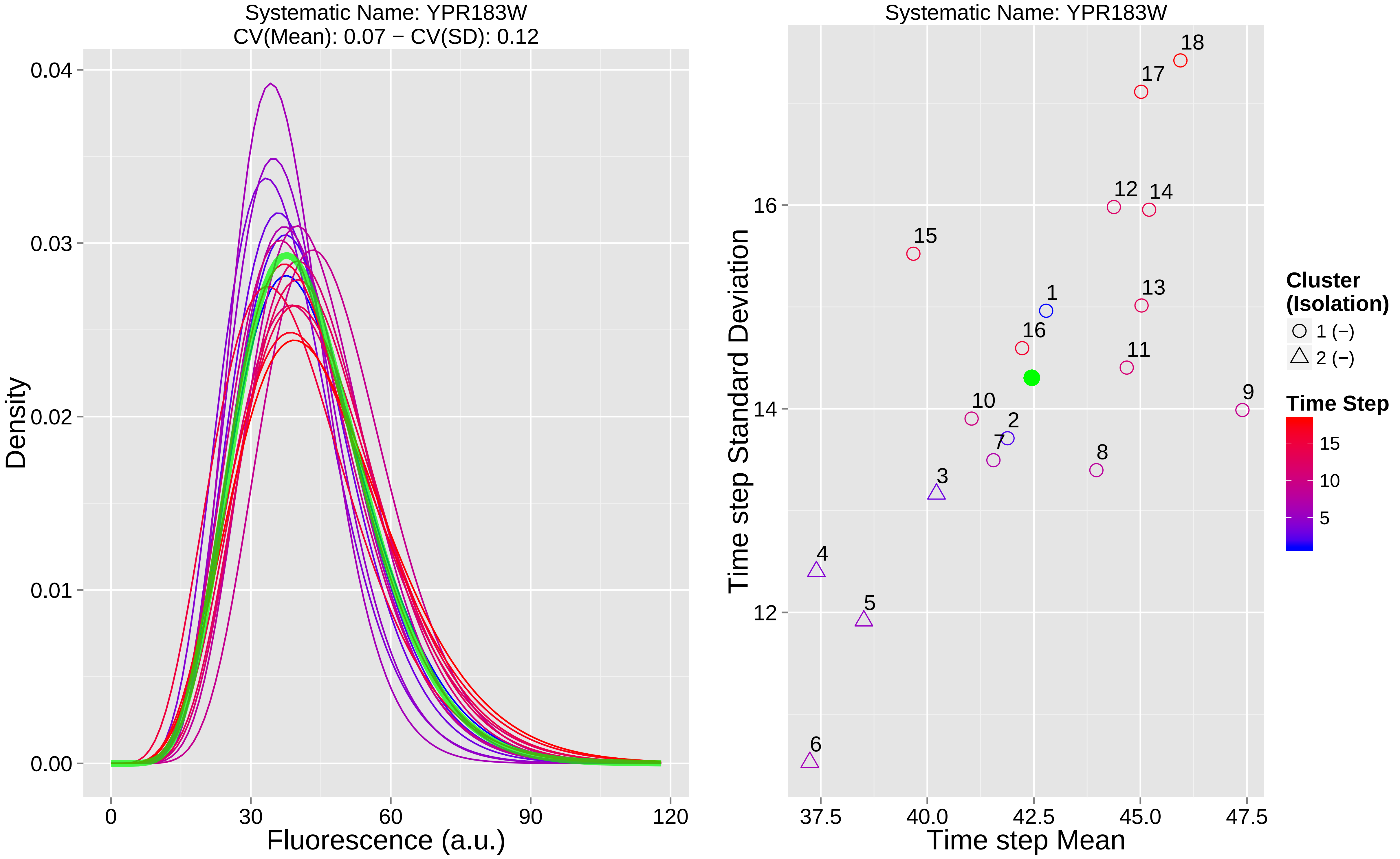
Systematic Name: YPR183W
CV(Mean): 0.07 − CV(SD): 0.12
Density
0.04
0.03
0.02
0.01
0.00
0
30
60
90
120
Fluorescence (a.u.)
Systematic Name: YPR183W
Time step Standard Deviation
16
14
12
37.5
40.0
42.5
45.0
47.5
Time step Mean
1
2
3
4
5
6
7
8
9
10
11
12
13
14
15
16
17
18
Cluster
(Isolation)
1 (−)
2 (−)
Time Step
15
10
5

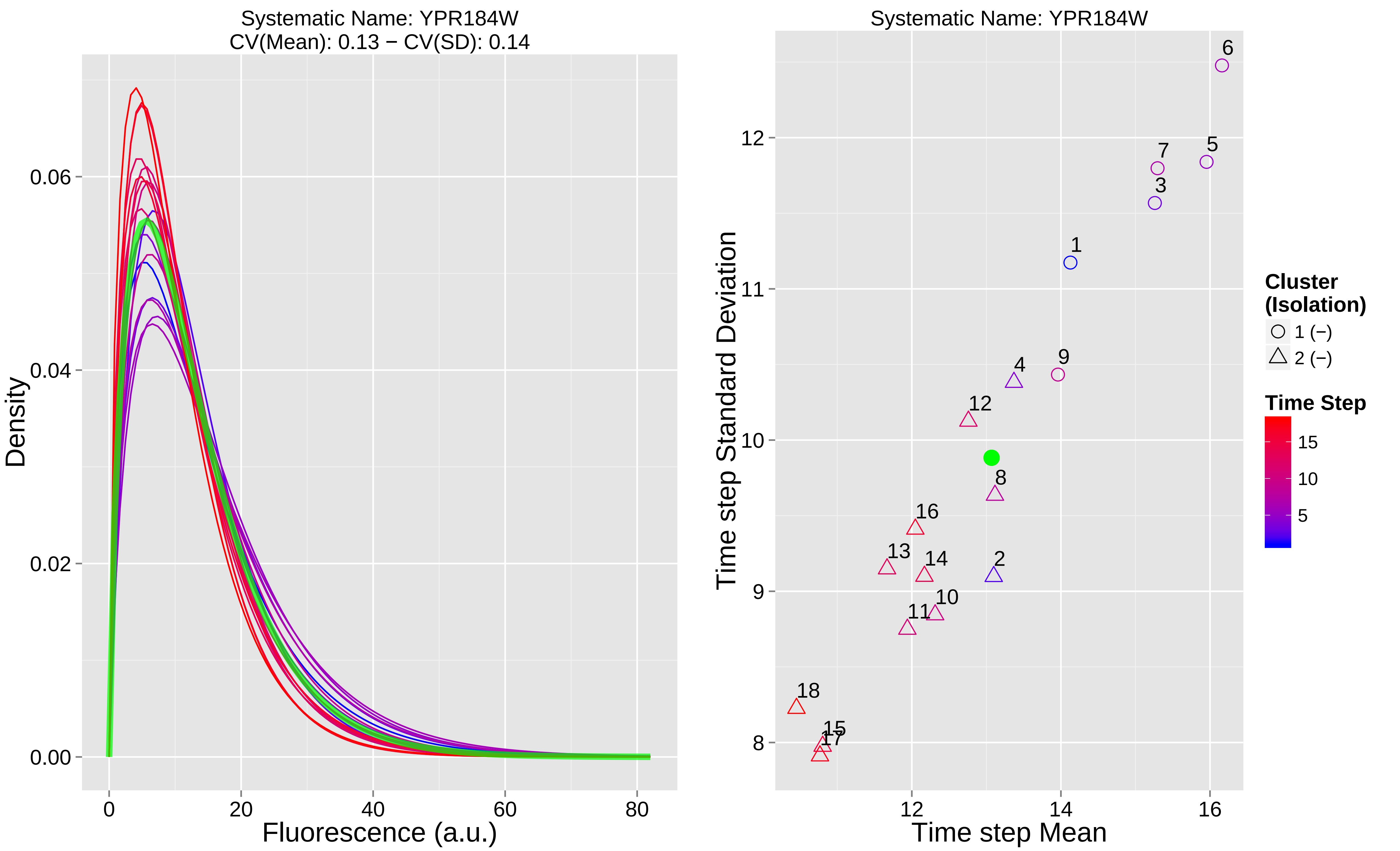

Systematic Name: YPR184W
CV(Mean): 0.13 − CV(SD): 0.14
Density
Fluorescence (a.u.)
0.00
0.02
0.04
0.06
0
20
40
60
80
Systematic Name: YPR184W
Time step Standard Deviation
Time step Mean
8
9
10
11
12
14
16
1
2
3
4
5
6
7
8
9
10
11
12
13
14
15
16
17
18
Cluster (Isolation)
1 (−)
2 (−)
Time Step
5
10
15
